# Epigenetic regulation of the PGE_2_ pathway modulates macrophage phenotype in normal and pathologic wound repair

**DOI:** 10.1172/jci.insight.138443

**Published:** 2020-09-03

**Authors:** Frank M. Davis, Lam C. Tsoi, Rachael Wasikowski, Aaron denDekker, Amrita Joshi, Carol Wilke, Hongping Deng, Sonya Wolf, Andrea Obi, Steven Huang, Allison C. Billi, Scott Robinson, Jay Lipinski, William J. Melvin, Christopher O. Audu, Stephan Weidinger, Steven L. Kunkel, Andrew Smith, Johann E. Gudjonsson, Bethany B. Moore, Katherine A. Gallagher

**Affiliations:** 1 Section of Vascular Surgery, Department of Surgery,; 2 Department of Microbiology and Immunology,; 3 Department of Dermatology, and; 4 Department of Internal Medicine, University of Michigan Medical School, Ann Arbor, Michigan, USA.; 5 Department of Bioengineering, University of Illinois, Champaign, Illinois, USA.; 6 Department of Dermatology, Venereology and Allergy, University Hospital Schleswig-Holstein, Campus Kiel, Kiel, Germany.; 7 Department of Pathology, University of Michigan Medical School, Ann Arbor, Michigan, USA.

**Keywords:** Endocrinology, Inflammation, Diabetes, Epigenetics, Macrophages

## Abstract

Macrophages are a primary immune cell involved in inflammation, and their cell plasticity allows for transition from an inflammatory to a reparative phenotype and is critical for normal tissue repair following injury. Evidence suggests that epigenetic alterations play a critical role in establishing macrophage phenotype and function during normal and pathologic wound repair. Here, we find in human and murine wound macrophages that cyclooxygenase 2/prostaglandin E_2_ (COX-2/PGE_2_) is elevated in diabetes and regulates downstream macrophage-mediated inflammation and host defense. Using single-cell RNA sequencing of human wound tissue, we identify increased NF-κB–mediated inflammation in diabetic wounds and show increased COX-2/PGE_2_ in diabetic macrophages. Further, we identify that COX-2/PGE_2_ production in wound macrophages requires epigenetic regulation of 2 key enzymes in the cytosolic phospholipase A_2_/COX-2/PGE_2_ (cPLA_2_/COX-2/PGE_2_) pathway. We demonstrate that TGF-β–induced miRNA29b increases COX-2/PGE_2_ production via inhibition of DNA methyltransferase 3b–mediated hypermethylation of the *Cox-2* promoter. Further, we find mixed-lineage leukemia 1 (MLL1) upregulates cPLA_2_ expression and drives COX-2/PGE_2_. Inhibition of the COX-2/PGE_2_ pathway genetically (*Cox2^fl/fl^ Lyz2^Cre+^*) or with a macrophage-specific nanotherapy targeting COX-2 in tissue macrophages reverses the inflammatory macrophage phenotype and improves diabetic tissue repair. Our results indicate the epigenetically regulated PGE_2_ pathway controls wound macrophage function, and cell-targeted manipulation of this pathway is feasible to improve diabetic wound repair.

## Abstract 2

Epigenetic modification of the COX-2/PGE2 pathway modulates diabetic macrophage phenotype, and cell-targeted manipulation improves diabetic tissue repair.

## Graphical abstract

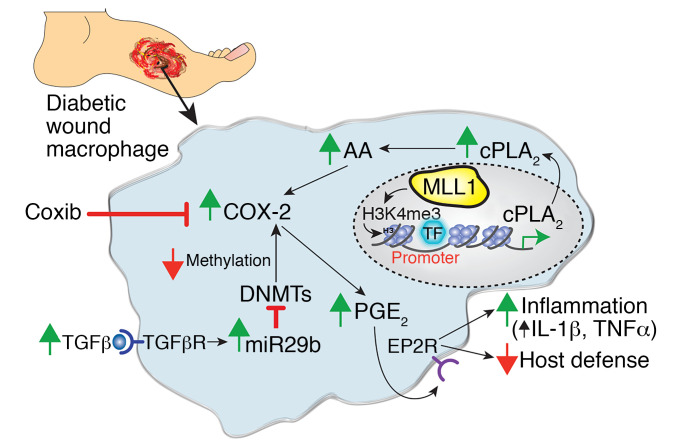

## Introduction

Wound repair is a complex process that occurs in overlapping stages of coagulation, inflammation, proliferation, and remodeling (1). During the inflammatory phase, macrophage plasticity is essential for wound remodeling. The inflammatory phase of healing can be divided into an “early” phase demonstrated by proinflammatory macrophages that promote inflammation and tissue destruction and a “late” phase where antiinflammatory macrophages promote tissue repair and allow for transition to the proliferative phase of healing. Early inflammatory phase macrophages predominately derive from infiltrating monocytes and demonstrate increased production of inflammatory cytokines (IL-1β, IL-12, and TNF-α) with enhanced pathogen killing capacity (2), while late inflammatory phase macrophages produce IL-10 and other mediators important in the transition to the proliferative phase of wound healing (3, 4). The predominance of these phenotypically distinct macrophages at specific times following injury facilitates healing through this tailored innate immune response. In pathologic, nonhealing type 2 diabetic (T2D) wounds, macrophages remain in an inflammatory state and fail to transition to a reparative phenotype. Although it is well established that macrophage plasticity in wound tissue is essential for repair, the molecular mechanisms that program and sustain macrophage phenotypes in wounds have not been identified.

The prostaglandin E_2_ (PGE_2_) pathway has been associated with chronic inflammation and impaired host defense in several pathologic conditions (5–8); however, its role in normal and diabetic wound healing and particularly the macrophage inflammatory response remains poorly defined. PGE_2_ is a lipid mediator derived from membrane phospholipids via the actions of the enzyme cytosolic phospholipase A_2_ (cPLA_2_), which generates arachidonic acid (AA). Free AA can then be metabolized via the enzymatic activity of cyclooxygenase 1 or 2 (COX-1 or COX-2) and PGE synthase 1. Constitutively expressed, COX-1 is important for mediating homeostatic effects, while COX-2 is upregulated by inflammatory signals (9). Ultimately, PGE_2_ exerts its downstream effects via binding to G protein–coupled 7 transmembrane spanning E prostanoid (EP) receptors (EP1–4) that signal distinct intracellular pathways regulating NF-κB–mediated inflammation and host defense (10–13).

Accumulating evidence suggests that epigenetic regulation of gene expression, via mechanisms such as histone modification or DNA methylation, plays a critical role in influencing immune cell phenotypes in both normal and pathologic conditions by controlling downstream protein expression patterns (14). In mammalian cells, DNA methylation is performed by 3 members of the DNA methyltransferase (DNMT) family: DNMT1, DNMT3a, and DNMT3b. DNA methylation primarily occurs at specific dinucleotide sites (CpG islands). Generally, DNA methylation is a repressive modification that mediates gene silencing by inhibiting the binding of transcription complexes to target gene promoters. Epigenetic histone modifications and DNA methylation changes have been shown by us and others to regulate inflammatory gene expression in macrophages (6, 15–17). We found that the chromatin-modifying enzyme mixed-lineage leukemia 1 (MLL1), a histone methyltransferase with site specificity for lysine 4 on histone H3 (H3K4) (18, 19), drives macrophage phenotype in wound repair (15). Additional studies have identified the COX-2/PGE_2_ pathway may be epigenetically regulated in lung and other tissues (20, 21). Based on this work, we examined whether epigenetic modification of the PGE_2_ pathway regulates the persistent macrophage inflammation and enhanced susceptibility to pathogens in diabetic wounds.

Here, using human single-cell transcriptome profiling of diabetic wounds and diabetic murine models, we demonstrate differential expression of the COX-2/PGE_2_ pathway in diabetic wound macrophages. Mechanistically, experiments in multiple myeloid-specific murine models (*Mll1^fl/fl^ Lyz2^Cre+^*, *Cox2^fl/fl^ Lyz2^Cre+^*, and *Alk5^fl/fl^ Lyz2^Cre+^*) reveal that the COX-2/PGE_2_ pathway is epigenetically regulated in wound macrophages via MLL1-mediated histone methylation on the *cPLA_2_* gene and TGF-β–mediated miRNA 29b (miR29b) regulation of DNMT 3a/3b on the *Cox-2* gene promoter. These epigenetic regulatory mechanisms converge to increase COX-2/PGE_2_ production in diabetic wound macrophages, resulting in a persistent proinflammatory phenotype and impaired macrophage function driving inadequate tissue repair and host defense. Importantly, macrophage targeting of the PGE_2_ pathway through genetic deletion (*Cox2^fl/fl^ Lyz2^Cre+^*) or pharmacologic inhibition with localized delivery of macrophage-specific polysaccharide nanocarriers containing a selective COX-2 inhibitor facilitated the transition of wound macrophages from an inflammatory to a reparative phenotype and improved diabetic wound repair. This work mechanistically identifies practical therapeutic targets for abrogating dysregulated macrophage function in nonhealing wounds.

## Results

### COX-2/PGE_2_ pathway is increased in murine and human diabetic monocyte/macrophages.

**Figure 1:**
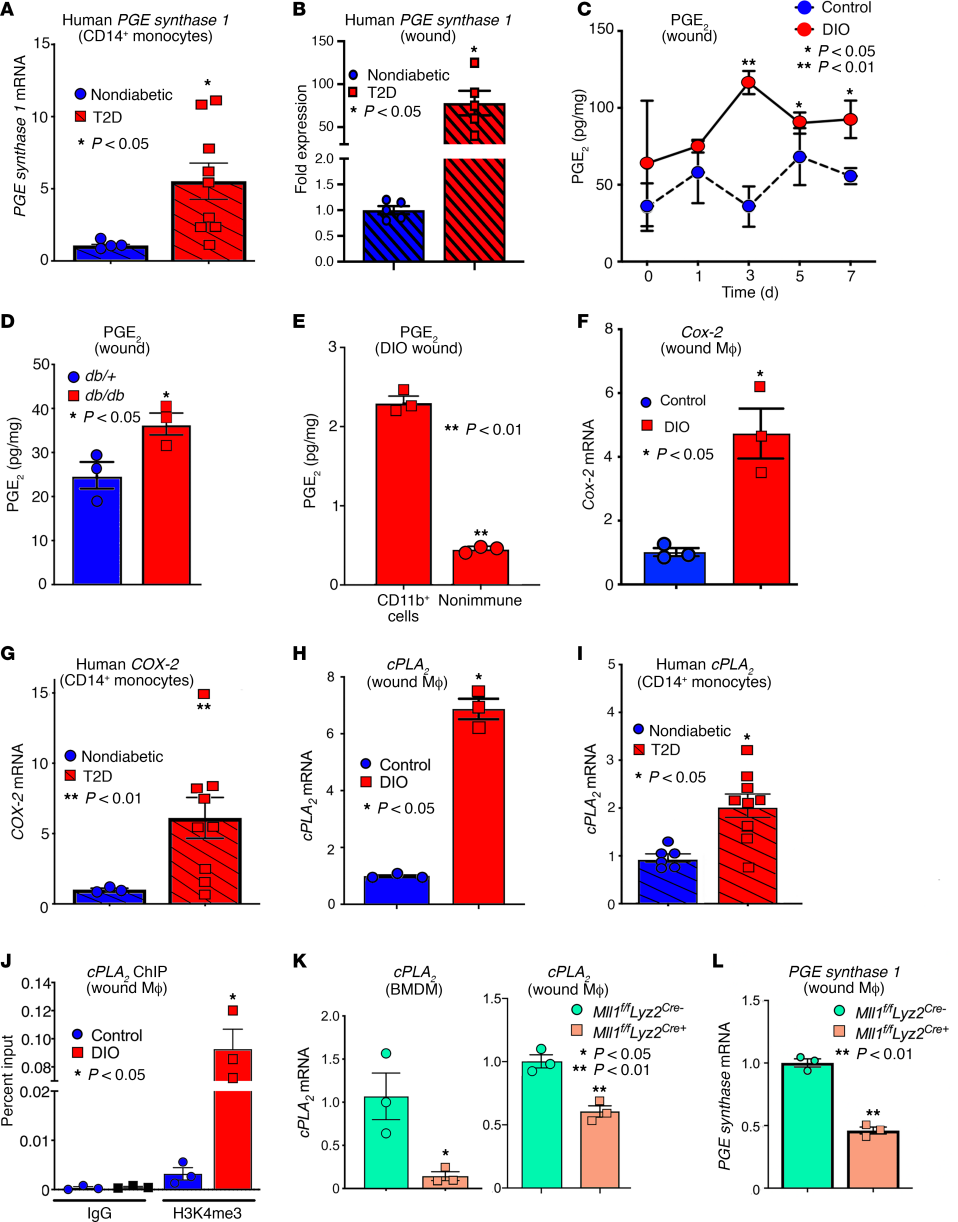
COX-2/PGE_2_ pathway is increased in murine and human diabetic monocytes and wound macrophages. (**A**) Peripheral blood (30 mL) was collected from patients with T2D and control subjects without diabetes and underwent red blood cell lysis followed by Ficoll separation. CD14^+^ monocytes were then positively selected by magnetic activated cell sorting (MACS), and *PGE Synthase 1* gene expression was measured (*n* = 9 T2D and 4 nondiabetic, repeated 2 times in triplicate). (**B**) Human wound macrophages (CD68^+^) were sorted via MACS from patients with T2D and nonischemic controls, and *PGE Synthase 1* expression was measured (*n* = 5, repeated 3 times in triplicate). (**C**) Wound monocyte/macrophages (MФs) (CD11b^+^[CD3^–^CD19^–^NK1.1^–^Ly6G^–^CD11b^+^]) were isolated on days 0, 1, 3, 5, and 7 postwounding and analyzed for secretion of PGE_2_ in DIO and control mice (*n* = 5/group, repeated 2 times). (**D**) Wound monocyte/macrophages (MФs) (CD11b^+^[CD3^–^CD19^–^NK1.1^–^Ly6G^–^CD11b^+^]) were isolated on day 5 postwounding and analyzed for secretion of PGE_2_ in *db/db* and control (*db/+*) mice (*n* = 3/group, repeated 2 times). (**E**) Wound cells were sorted from DIO mice and PGE_2_ levels were analyzed in myeloid cells (CD11b^+^[CD3^–^CD19^–^NK1.1^–^Ly6G^–^CD11b^+^]) and nonimmune cells (*n* = 3/group, repeated 2 times). (**F**) Wound monocyte/macrophages (MФs) (CD11b^+^[CD3^–^CD19^–^NK1.1^–^Ly6G^–^CD11b^+^]) from DIO and controls were isolated on day 5 postwounding and analyzed for *Cox-2* expression. (*n* = 3/group, repeated in triplicate.) (**G**) Peripheral blood was collected from patients with T2D and control subjects without diabetes and underwent red blood cell lysis followed by Ficoll separation. CD14^+^ monocytes were then positively selected by MACS, and *COX-2* gene expression was measured (*n* = 9 T2D and 4 nondiabetic, repeated 2 times in triplicate). (**H**) Wound monocyte/macrophages (MФs) (CD11b^+^[CD3^–^CD19^–^NK1.1^–^Ly6G^–^CD11b^+^]) from DIO and controls were isolated on day 5 postwounding and analyzed for *cPLA_2_* expression. (*n* = 3/group, repeated in triplicate.) (**I**) Peripheral blood was collected from patients with T2D and control subjects without diabetes and underwent red blood cell lysis followed by Ficoll separation. CD14^+^ monocytes were then positively selected by MACs, and *cPLA_2_* gene expression was measured (*n* = 9 T2D and 4 nondiabetic, repeated 2 times in triplicate). (**J**) ChIP analysis was performed for H3K4me3 at the NF-κB binding site on the *cPLA_2_* promoter in DIO and control wound monocyte/macrophages (MФs) (CD11b^+^[CD3^–^CD19^–^NK1.1^–^Ly6G^–^CD11b^+^]) isolated on day 5 (*n* = 3/group, repeated 2 times in triplicate). (**K**) BMDMs and wound monocyte/macrophages (MФs) (CD11b^+^[CD3^–^CD19^–^NK1.1^–^Ly6G^–^CD11b^+^]) were isolated from *Mll1^fl/fl^ Lyz2^Cre+^* mice and littermate controls on day 5 postwounding. *cPLA_2_* gene expression was quantified by quantitative PCR (qPCR; *n* = 3/group, repeated 2 times in triplicate). (**L**) Wound monocyte/macrophages (MФs) (CD11b^+^[CD3^–^CD19^–^NK1.1^–^Ly6G^–^CD11b^+^]) were isolated from *Mll1^fl/fl^ Lyz2^Cre+^* mice and littermate controls on day 5 postwounding. *PGE* gene expression was quantified by qPCR (*n* = 3/group, repeated 2 times in triplicate). *P < 0.05, **P < 0.01. Data are presented as the mean ± SEM. Data were first analyzed for normal distribution, and if data passed normality test, 2-tailed Student’s *t* test was used.

Increasing evidence suggests that proper wound healing requires the establishment of a regulated inflammatory response mediated by infiltrating monocyte/macrophages (4, 22, 23). Although the PGE_2_ pathway has been well studied in chronic inflammatory diseases (i.e., rheumatoid arthritis and Crohn disease) (7, 8) as well as infection (5, 6); little is known about the COX-2/PGE_2_ pathway in regulation of wound monocyte/macrophage function in normal and diabetic tissue repair. It is well established that in inflammatory disease states, such as in T2D, monocyte/macrophages remain persistently in an inflammatory phenotype and fail to transition to the reparative state. To determine if the PGE_2_ pathway is altered in inflammatory monocytes from patients with T2D, we isolated peripheral blood monocytes (CD14^+^) from patients with T2D and healthy volunteers. We found expression of *PGE Synthase 1* was markedly increased in T2D blood monocytes compared with nondiabetic control monocytes (Figure 1A). This increased expression of *PGE Synthase 1* in blood monocytes is highly relevant as the majority of wound macrophages following injury are recruited monocytes from the blood that transition to macrophages in the tissues. Next, to analyze the PGE_2_ pathway in human wound healing, wound macrophages were isolated from chronic nonhealing wounds from T2D and nondiabetic patients. Human wound-derived macrophages (CD68^+^) isolated from patients with T2D showed a significant upregulation of *PGE Synthase 1* compared with wound macrophages from nondiabetic patients (Figure 1B). Taken together, these results indicate that the PGE_2_ pathway in monocyte/macrophages is increased in human T2D patients and may influence wound tissue monocyte/macrophage phenotype.

Inflammation is both necessary for progression of normal wound healing and pathologic if it occurs in excess or at inappropriate times. Given that the PGE_2_ pathway was upregulated in myeloid cells from patients with T2D, we examined PGE_2_ levels in a murine diet-induced obesity (DIO) T2D model that has been shown to display delayed wound healing (4). The DIO mouse most closely mirrors human physiology in the diet-induced weight gain and the development of insulin resistance and glucose intolerance (24). Following acute injury, wound monocyte/macrophages (CD11b^+^[CD3^–^CD19^–^NK1.1^–^Ly6G^–^CD11b^+^]) were isolated from DIO and control (normoglycemic) murine wounds. PGE_2_ levels were significantly increased in diabetic wound monocyte/macrophages during the inflammatory phase of wound repair (days 3–7) compared with controls (Figure 1C). Notably, there were no significant changes in expression of leukotrienes (Supplemental Figure 1, A and B; supplemental material available online with this article; https://doi.org/10.1172/jci.insight.138443DS1). Additionally, a second diabetic model, *db/db* mice, was wounded, and wound monocyte/macrophages demonstrated upregulation of both *PGE Synthase 1* and PGE_2_ on day 5 after injury (Figure 1D and Supplemental Figure 1C) compared with *db/+* controls. Further, to confirm the upregulation of the PGE_2_ pathway was specific to wound monocyte/macrophages, and not other structural cells, PGE_2_ levels were analyzed in both myeloid cells (CD11b^+^[CD3^–^CD19^–^NK1.1^–^Ly6G^–^CD11b^+^]) and nonimmune cell fractions in both the DIO and *db/db* models. This demonstrated significant upregulation of PGE_2_ in myeloid cells in both models compared with nonimmune wound cells (Figure 1E and Supplemental Figure 1D). Last, although the murine full-thickness punch biopsy model can provide adequate assessment of acute injury and tissue response, one limitation is that contraction plays a predominant role in wound closure. PGE_2_ has previously been shown to limit fibroblast contraction (25, 26). Thus, to confirm that upregulation of the PGE_2_ pathway results in an altered monocyte/macrophage phenotype and impaired healing that is not secondary to alteration in wound contraction, a well-established model of splinted chronic wound healing was used (27). Wounds harvested from this chronic murine model also demonstrated sustained overproduction of *PGE Synthase 1*, consistent with the DIO and *db/db* models (Supplemental Figure 2A), suggesting that the PGE_2_ pathway is increased in wound monocyte/macrophages in multiple models of diabetes and wound healing.

PGE_2_ synthesis involves the enzymatic conversion of AA into PGE_2_ via the actions of the COX-1 or COX-2 enzyme (28). The COX-1 enzyme is constitutively expressed while the COX-2 enzyme is upregulated during inflammation (9). Given that COX-2 is the rate-limiting enzyme for PGE_2_ production, we examined *Cox-2* expression during diabetic wound healing. Wound monocyte/macrophages (CD11b^+^[CD3^–^CD19^–^NK1.1^–^Ly6G^–^CD11b^+^]) were sorted from DIO and control mice, and *Cox-2* expression was found to be significantly elevated in murine diabetic wound monocyte/macrophages on day 5 (Figure 1F). *Cox-2* was also elevated in wound monocyte/macrophages isolated from the chronic splinted model (Supplemental Figure 2B). As a translational corollary, we isolated peripheral blood monocytes (CD14^+^) from matched patients with T2D and healthy volunteers and found expression of *COX-2* was markedly increased in T2D blood monocytes compared with nondiabetic control monocytes (Figure 1G). Additionally, within the PGE_2_ synthesis pathway, production of the substrate for COX-2 is mediated by the enzyme cPLA_2_ which regulates conversion of membrane phospholipids to AA. We also found that cPLA_2_ expression was markedly elevated in T2D blood monocytes and wound macrophages compared with nondiabetic controls (Figure 1, H and I) and in the splinted wound murine model (Supplemental Figure 2C). *cPLA_2_* expression has previously been shown to be regulated at the transcriptional level (29), and epigenetics-based histone modifications regulate macrophage phenotypes by controlling downstream gene expression (30–32). To examine epigenetic transcriptional regulation of cPLA_2_, we isolated wound monocyte/macrophages from DIO and controls on day 5 postinjury and performed ChIP analysis. We found that the histone methylation mark H3K4 trimethylation (H3K4me3) was significantly increased on the *cPLA_2_* promoter in DIO wound monocyte/macrophages (Figure 1J). The H3K4me3 methylation mark maintains chromatin in a conformation that promotes active transcription. In mammals, H3K4me3 is controlled by the SET1-MLL family of enzymes and the epigenetic enzyme, MLL1, which is a histone methyltransferase with site specificity for H3K4. Because we previously found that MLL1 can influence wound repair, we examined whether MLL1 regulates increased H3K4me3 on the cPLA_2_ promoter. We generated mice deficient in *Mll1* in cells of the myeloid lineage (monocytes, macrophages, granulocytes) by floxing out the MLL1 gene using a lysozyme Cre driver (*Mll1^fl/fl^ Lyz2^Cre+^*). Myeloid-specific depletion of *Mll1* was confirmed by examining splenic and wound monocyte/macrophages from *Mll1^fl/fl^ Lyz2^Cre+^* mice and littermate controls (*Mll1^fl/fl^ Lyz2^Cre–^*) (15). No differences in MLL1 were detected in nonmyeloid cells (15). We found *cPLA_2_* and *PGE* expression was significantly decreased in both bone marrow–derived macrophages (BMDMs) and wound monocyte/macrophages isolated from *Mll1*-deficient mice (Figure 1, K and L), suggesting MLL1-derived H3K4me3 methylation may regulate *cPLA_2_* expression in tissue monocyte/macrophages. These data indicate that elevated PGE_2_ production in human and murine wound monocyte/macrophages may be partially driven by epigenetic upregulation of the upstream prostaglandin pathway enzyme, cPLA_2_, via MLL-mediated H3K4 methylation. Taken together, these results indicate that the COX-2/PGE_2_ pathway is elevated in diabetic human and murine wound monocyte/macrophages and this pathway may control downstream macrophage-mediated inflammation.

### TGF-β–induced mir29b increases COX-2 production via inhibition of DNMT3-mediated methylation in wound macrophages.

**Figure 2:**
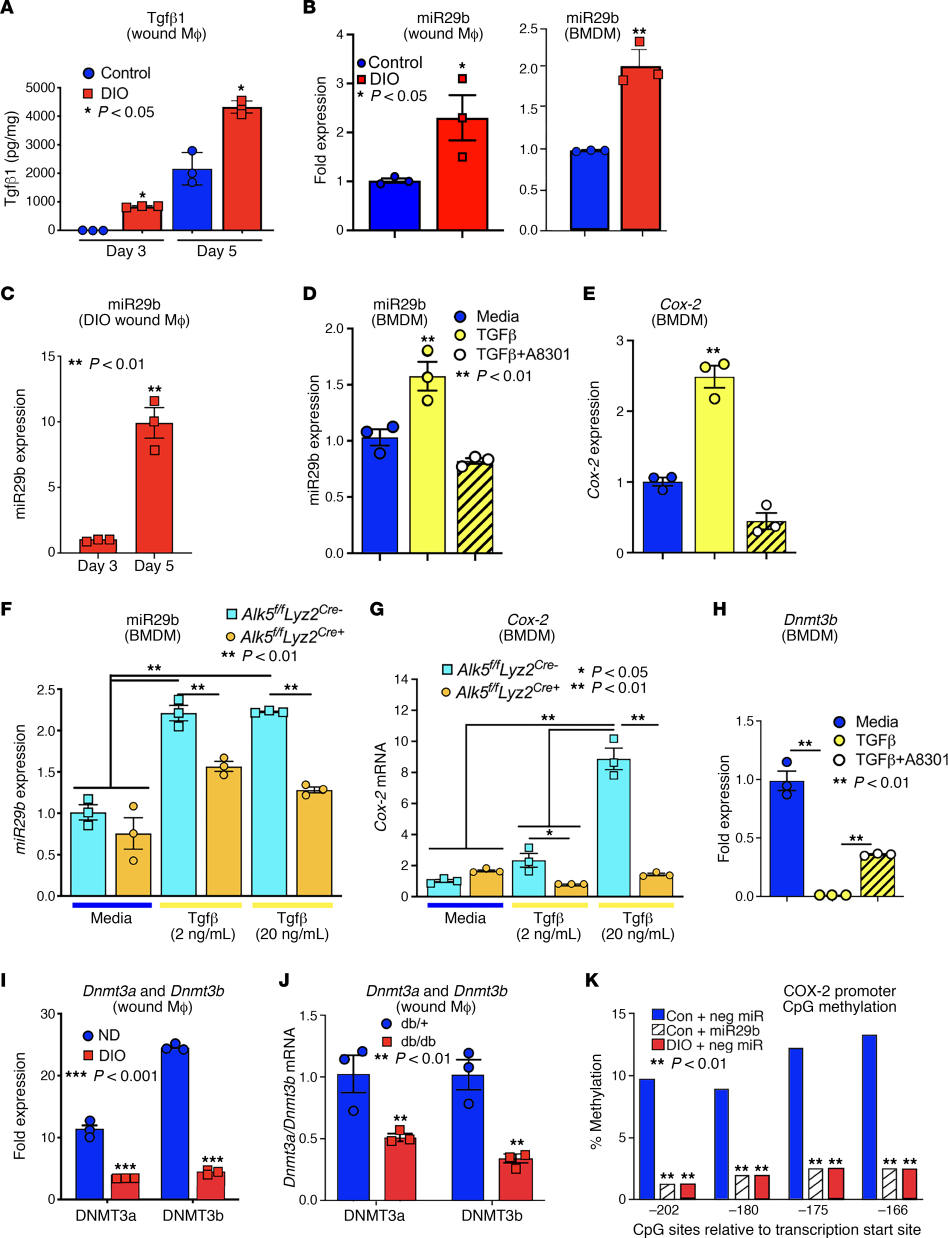
TGF-β–induced mir29b increases COX-2 production via inhibition of DNMT3-mediated methylation in wound macrophages. (**A**) Whole wounds from DIO and control mice were isolated on days 3 and 7 postwounding and analyzed for TGF-β1 protein levels by Bioplex. (*n* = 3/group, repeated once.) (**B**) Wound monocyte/macrophages and BMDMs were isolated on day 5 postwounding and analyzed for miR29b expression. (*n* = 3/group, repeated in triplicate.) (**C**) Wound monocyte/macrophages from DIO mice were sorted on days 3 and 5 postwounding via MACS and miR29b levels were analyzed. (**D** and **E**) BMDMs were isolated and stimulated with media, TGF-β (2 μg/mL), or TGF-β (2 μg/mL)+A8301 (0.5 μM) for 4 hours and analyzed for miR29b (**D**) and *Cox-2* (**E**) expression. (*n* = 3/group, repeated in triplicate.) (**F** and **G**) BMDMs were isolated from *Alk5^fl/fl^ Lyz2^Cre–^* and *Alk5^fl/fl^ Lyz2^Cre+^* and stimulated with TGF-β (2 or 20 μg/mL) for 4 hours and analyzed for miR29b (**F**) and *Cox-2* (**G**) expression. (*n* = 3/group, repeated in triplicate.) (**H**) BMDMs were isolated and stimulated with media, TGF-β (2 μg/mL), or TGF-β (2 μg/mL)+A8301 (0.5 μM) for 4 hours and analyzed for *Dnmt3b* expression. (*n* = 3/group, repeated in triplicate.) (**I** and **J**) Wound monocyte/macrophages from DIO and *db/db* mice and their respective controls were isolated on day 3 and analyzed for *DNMT3a* and *DNMT3b* expression (*n* = 3/group, repeated twice). (**K**) Bisulfite sequencing was performed on DIO and control wound monocyte/macrophages treated with miR29b or a negative miR control (30 nM) and the *Cox-2* promoter was analyzed for DNA methylation (*n* = 3/group). *P < 0.05, **P < 0.01. Data are presented as the mean ± SEM. Data were first analyzed for normal distribution, and if data passed normality test, 2-tailed Student’s *t* test for 2 groups and 2-way ANOVA for multiple groups was used.

Alterations in COX-2 expression have been previously shown to be epigenetically regulated (33, 34). TGF-β1 has been shown to influence the regulation of the PGE_2_ pathway in diabetic macrophages and tissue repair (35). As such, we isolated diabetic wound monocyte/macrophages at early and late time points postinjury and found TGF-β1 was significantly increased in diabetic wounds compared with controls and remained upregulated even at late reparative time points (Figure 2A). In parallel, we found miR29b was significantly upregulated in BMDMs from DIO mice and in DIO wound monocyte/macrophages during the transition of tissue macrophages from the inflammatory to the reparative phenotype (Figure 2, B and C). To further examine the relationship between TGF-β1, miR29b, and COX-2 in monocyte/macrophages, BMDMs were stimulated with TGF-β1 (2 μg/mL) with or without a pharmacologic TGF-β receptor inhibitor (A8301, 0.5 M). TGF-β1 stimulation increased miR29b and *Cox-2* expression, and these changes were reversed with TGF-β receptor inhibitor administration (Figure 2, D and E). We then sought to translate these in vitro findings using a myeloid-specific genetic model, and as such we generated mice deficient in the TGF-β1 receptor (Alk5) in cells of the myeloid lineage (monocytes, macrophages, granulocytes) by using the Cre-lox system. Myeloid-specific depletion of Alk5 was confirmed by examining sorted monocyte/macrophages from *Alk5^fl/fl^ Lyz2^Cre+^* mice and littermate controls (*Alk5^fl/fl^ Lyz2^Cre–^*) (data not shown). BMDMs from *Alk5^fl/fl^ Lyz2^Cre+^* and littermate controls were isolated and stimulated with TGF-β1 (2 μg/mL or 20 μg/mL). TGF-β1 stimulation resulted in significant upregulation of miR29b and *Cox-2* expression in a dose-dependent manner in *Alk5^fl/fl^ Lyz2^Cre–^* BMDMs, but this was negated with genetic depletion of Alk5 (Figure 2, F and G).

miR29b has been shown to influence *Cox-2* expression by regulating the actions of DNMTs (33). DNMTs are responsible for the transfer of methyl groups to CpG dinucleotides located in gene promoters, resulting in the silencing of gene expression. DNMT3a and DNMT3b deposit de novo methylation marks, while DNMT1 is responsible for maintaining these marks. It is unknown if DNMTs play a role in the regulation of the PGE_2_ pathway in diabetic macrophages and tissue repair. As such, we analyzed *Dnmt3b* expression in BMDMs stimulated with TGF-β1 (2 μg/mL) with or without a TGF-β receptor inhibitor (A8301, 0.5 μM). TGF-β1 stimulation reduced *Dnmt3b* expression, which was reversed with administration of a TGF-β receptor inhibitor (Figure 2H). Next, we verified these findings in our in vivo murine model; *Dnmt3a* and *Dnmt3b* expression were reduced in DIO and *db/db* wound monocyte/macrophages compared with controls (Figure 2, I and J). Given the reduction in DNMT3a/b we sought to determine whether this has functional impact on *Cox-2* gene promoter methylation in diabetic wound monocyte/macrophages. We performed bisulfite conversion and pyrosequencing, which identified a significant reduction in *Cox-2* promoter from sites -202 to -166, important for *Cox-2* transcription, in DIO wound monocyte/macrophages compared with controls. Further, the transfection of miR29b into control wound monocyte/macrophages reduced monocyte/macrophage methylation on the *Cox-2* promoter to levels seen in DIO wound monocyte/macrophages (Figure 2K). Taken together, these results suggest that elevated *Cox-2* in diabetic wound monocyte/macrophages is due, at least partially, to TGF-β1–regulated miR29b-induced destabilization of DNMT3a/3b and subsequent hypomethylation of the *Cox-2* promoter.

### Diabetic wound macrophages exhibit increased EP2 and EP4, resulting in increased inflammatory cytokines and impaired phagocytosis.

**Figure 3:**
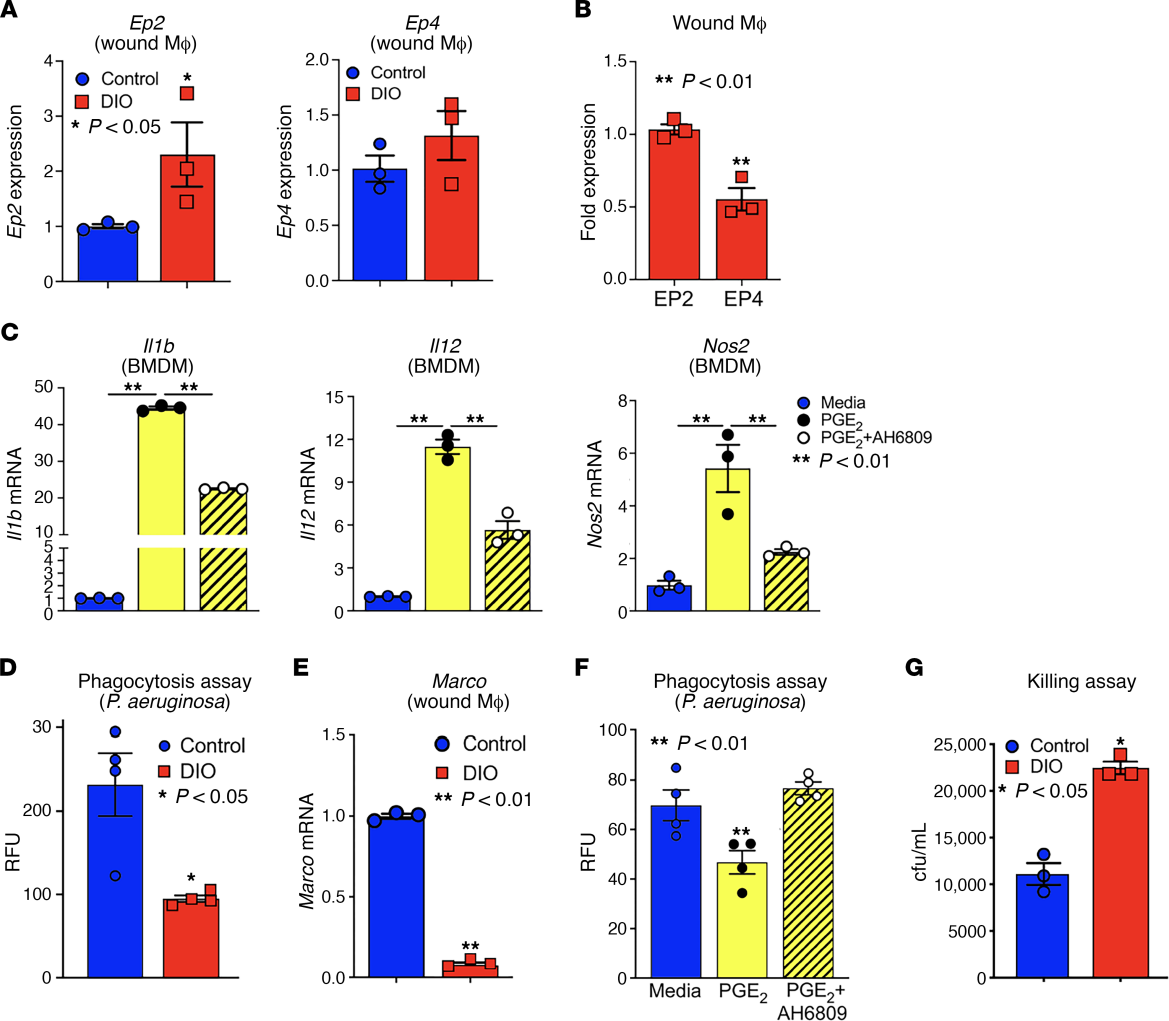
Diabetic wound macrophages exhibit increased EP2 and EP4, resulting in increased inflammation and impaired phagocytosis. (**A**) Wound monocyte/macrophages from DIO and control mice were sorted on day 5 postwounding and analyzed for *Ep2* and *Ep4* receptor expression (*n* = 3/group, repeated in triplicate). (**B**) Relative expression of *Ep2* versus *Ep4* in day 5 wound monocyte/macrophages (MФs) (*n* = 3/group, repeated in triplicate). (**C**) BMDMs from controls were stimulated with media, PGE_2_ (1000 nM), or PGE_2_ (1000 nM) + AH6809 (10 μM), and expression of *Il1b*, *Il12*, and *Nos2* was analyzed by qPCR (*n* = 3/group, run in triplicate). (**D**) Wound monocyte/macrophages from DIO mice or controls were incubated with fluorescently labeled *P*. *aeruginosa* for 2 hours, and the percentage of cells ingesting bacteria was determined in relative fluorescence units (RFU) (*n* = 4/group, repeated twice). (**E**) Wound monocyte/macrophages from DIO and control mice were isolated on day 5 postwounding and analyzed for *Marco* receptor expression (*n* = 3/group, run in triplicate). (**F**) Wound monocyte/macrophages from controls were incubated with media, PGE_2_ (1000 nM), or PGE_2_ (1000 nM) + AH6809 (10 μM) for 6 hours and then were exposed to fluorescently labeled *P*. *aeruginosa* for 2 hours, and the percentage of cells ingesting bacteria was determined in RFU (*n* = 3/group, repeated twice). (**G**) BMDMs from DIO mice or controls were infected with *P*. *aeruginosa* for 30 minutes before extracellular bacteria were removed. Cells were allowed to kill ingested bacteria for 2 hours before cells were lysed and CFU determined. Higher CFU/mL indicate impaired intracellular killing (*n* = 3/group, repeated twice). Data are presented as the mean ± SEM. Data were first analyzed for normal distribution, and if data passed normality test, 2-tailed Student’s *t* test for 2 groups and 2-way ANOVA for multiple groups was used.

Chronic elevation of PGE_2_ can have a lasting impact on myeloid cells, resulting in altered inflammatory signaling and impaired host defense to common diabetic pathogens (e.g., *Staphylococcus aureus*, *Escherichia coli*, and *Pseudomonas aeruginosa*) (5). For diabetic wounds this is uniquely and clinically important because an increased bacterial burden, particularly of *Pseudomonas aeruginosa*, plays a pivotal role in the risk for amputation (36). Despite the known susceptibility of diabetic wounds to infection, little is known about the molecular mechanisms that allow for increased infections in diabetic tissues. PGE_2_ exerts its effects on myeloid cells via binding to EP receptors (EP1–4) and stimulation of cAMP downstream signaling. Given the predisposition of diabetic wounds to infection, we evaluated *Ep2* and *Ep4* expression in diabetic wound monocyte/macrophages. Diabetic and control wound monocyte/macrophages (CD11b^+^[CD3^–^CD19^–^NK1.1^–^Ly6G^–^CD11b^+^]) were isolated from DIO mice and normal-diet controls; on day 5 and *Ep2* and *Ep4* were found to be significantly upregulated in DIO wound monocyte/macrophages, with *Ep2* expression representing the predominant receptor (Figure 3, A and B). To support that PGE_2_ signaling through the EP2 receptor contributes to aberrant inflammatory mediator production, BMDMs were stimulated with PGE_2_ and/or EP2 receptor antagonist (AH6809). PGE_2_ stimulation significantly increased inflammatory gene expression (*Il1b*, *Il12*, and *Nos2*), but this upregulation was prevented with EP2 receptor inhibitor treatment (Figure 3C). Taken together, these data suggest that PGE_2_ stimulation through the EP2 receptor may contribute to the chronic inflammatory phenotype seen in diabetic monocyte/macrophages.

Further, to determine if the altered *Ep2* and *Ep4* expression found in diabetic wound monocyte/macrophages resulted in a functional impact on the ability of macrophages to clear infection we analyzed the ability of control and DIO wound monocyte/macrophages to phagocytize common bacteria in diabetic wounds, including unopsonized *Pseudomonas aeruginosa* (*P*. *aeruginosa*), *Escherichia coli* (*E*. *coli*), and methicillin-resistant *Staphylococcus aureus*. At 2 hours after infection, the phagocytic ability of monocyte/macrophages from DIO mice to all 3 common pathogens was significantly reduced compared with control (Figure 3D and Supplemental Figure 3). This was also associated with reduced levels of macrophage receptor with collagenous structure (*Marco*), a scavenger receptor important for in vivo host response and phagocytosis against nonopsonized bacteria (37), in DIO wound monocyte/macrophages (Figure 3E). To determine if altered PGE_2_ levels contributed to the impairment in phagocytosis, control macrophages were treated ex vivo with PGE_2_ with or without the EP2 receptor inhibitor (AH6809). Stimulation with PGE_2_ reduced monocyte/macrophage phagocytosis, but this was rescued in the monocyte/macrophages with blockage of the EP2 receptor (Figure 3F). Last, monocyte/macrophage bacterial clearance relies on both the phagocytosis of specific pathogens and intracellular killing of the organism. Although a defect in phagocytosis, as shown above, increases susceptibility of diabetic wounds to infection, we also found that DIO monocyte/macrophages exhibit impairment in bacterial killing (Figure 3G). These findings demonstrate that the increased PGE_2_ in diabetic wound monocyte/macrophages results in increased inflammatory mediator production and impaired host defense via an EP2-mediated mechanism.

### Macrophage-specific genetic depletion of Cox-2 or administration of nanocarriers conjugated with a selective COX-2 inhibitor improves diabetic wound healing.

**Figure 4:**
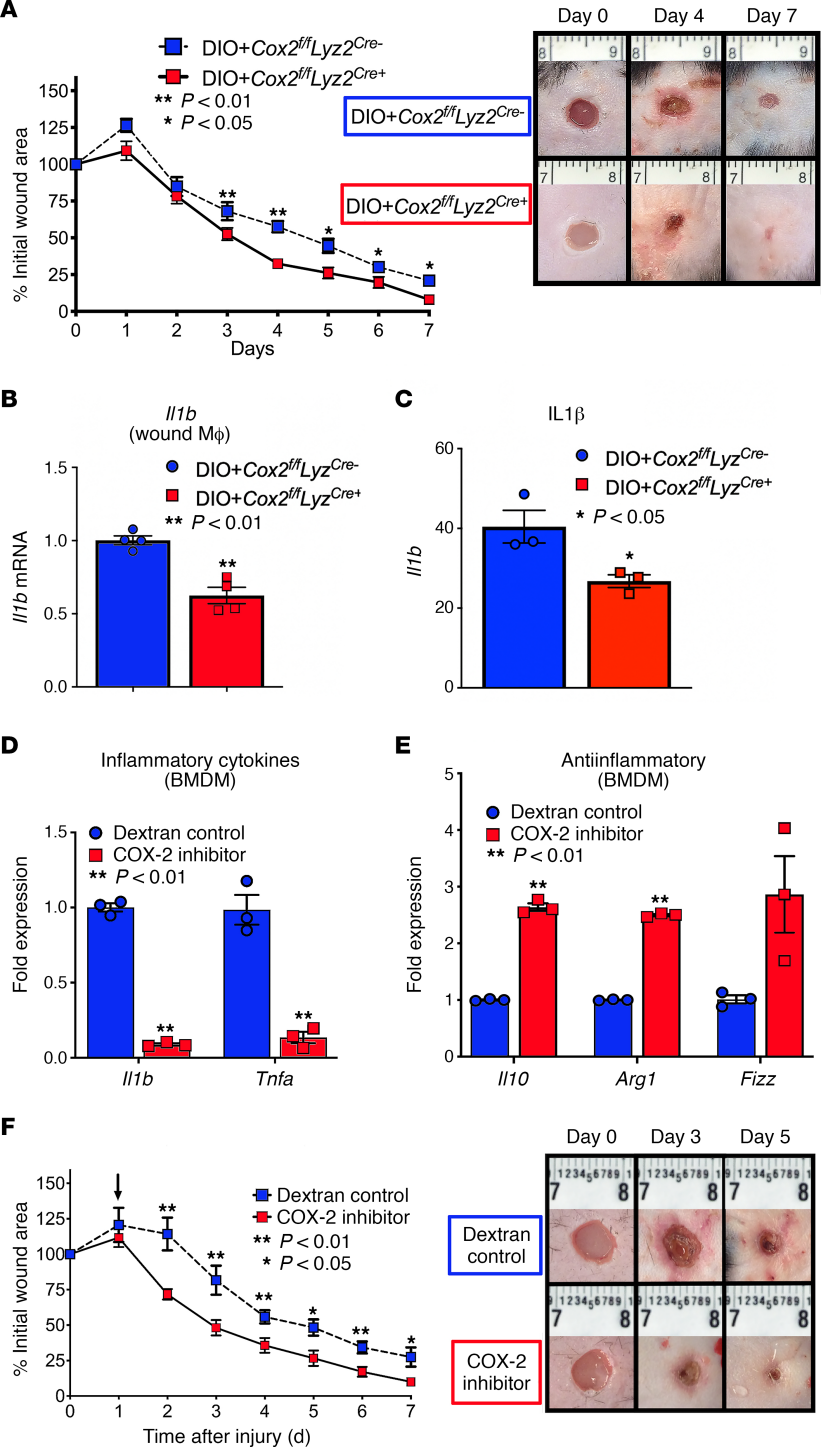
Macrophage-specific genetic depletion of Cox-2 or administration of nanocarriers conjugated with a selective COX-2 inhibitor improves diabetic wound healing. (**A**) DIO+*Cox2^fl/fl^ Lyz2^Cre+^* and littermate controls (DIO+*Cox2^fl/fl^ Lyz2*^Cre–^) were wounded with a 4 mm punch biopsy. Wound area was measured daily with ImageJ software throughout wound healing course (*n* = 5/group, repeated once). Representative images at days 0, 4, and 7 are shown. (**B** and **C**) Wound monocyte/macrophages from DIO+*Cox2^fl/fl^ Lyz2*^Cre+^ and littermate controls (DIO+*Cox2^fl/fl^ Lyz2*^Cre–^) were isolated and analyzed for *Il1b* gene expression by qPCR and IL-1β protein expression by ELISA (*n* = 3/group, repeated in triplicate). (**D** and **E**) BMDMs harvested from DIO mice were treated with PS nanocarriers conjugated to either a Cox-2 inhibitor (10 μM) or dextrose control (10 μM) and analyzed for *Il1b*, *Tnfa*, *Il10*, *Arg1*, and *Fizz* expression (*n* = 3/group, repeated in triplicate). (**F**) DIO mice were wounded with a 4 mm punch biopsy, and wounds were injected daily starting on day 1 postinjury with PS nanoparticles containing either selective COX-2 inhibitor (16 mg/kg) or dextrose control. Arrow indicates start of injections. Wound area was measured daily with ImageJ software throughout wound healing course (*n* = 5/group, repeated once). Representative images at days 0, 3, and 5 are shown. *P < 0.05, **P < 0.01. Data are presented as the mean ± SEM. Data were first analyzed for normal distribution and if data passed normality test, 2-tailed Student’s *t* test was used.

Given the increase in the PGE_2_ pathway in T2D monocyte/macrophages during the critical period where monocyte/macrophages transition from an inflammatory to a reparative phenotype, we sought to investigate whether interruption of this pathway may alter diabetic inflammation and wound healing. We created a myeloid-specific Cox-2–deficient mouse using a Cox-2–floxed mouse crossed with a Lyz-M–Cre mouse (*Cox2^fl/fl^ Lyz2^Cre+^*). *Cox2^fl/fl^ Lyz2^Cre+^* mice and their littermate controls were placed on a high-fat diet and after confirmation of hyperglycemia were wounded with a 4 mm punch biopsy, with wound healing rates monitored over time. We identified that mice lacking Cox-2 in their monocytes/macrophages (*Cox2^fl/fl^ Lyz2^Cre+^*) had significantly improved wound healing compared with littermate controls (Figure 4A). Wound monocyte/macrophages isolated from DIO^+^ *Cox2^fl/fl^ Lyz2^Cre+^* mice showed lower levels of proinflammatory cytokine (*Il1b* and *Tnfa*) gene expression and protein production (Figure 4, B and C; and Supplemental Figure 4A) and showed a functional improvement in the ability of *Cox2^fl/fl^ Lyz2^Cre+^* monocyte/macrophages to phagocytize pathologic bacteria in comparison with their littermate controls (Supplemental Figure 4B).

Given the upregulated PGE_2_/COX-2 pathway in diabetic wounds and its regulation of monocyte/macrophage phenotype, we assessed the ability of a pharmacologic COX-2–specific inhibitor to decrease monocyte/macrophage inflammatory mediator production and improve wound healing in DIO mice. For our drug delivery method, since the PGE_2_ pathway is likely important in multiple cell types, and to avoid systemic pharmaceutical administration, we chose to directly target tissue monocyte/macrophages with a nanomedicine drug delivery platform based on an FDA-approved polysaccharide (PS) nanocarrier composed of dextran that has been shown by our group to be selective for monocyte/macrophages (38). Dextran, like many other PSs, is known for efficient and selective uptake by monocyte/macrophages in a variety of tissues with uptake dependent on monocyte/macrophage phenotypic polarization (M1 like **→** M2 like) (38, 39). A 500 kDa dextran derived from commercial biopolymers was conjugated to the COX-2 inhibitor through ester bonds, which are hydrolyzed by monocyte/macrophage intracellular esterases. To first assess the capacity of this conjugate drug delivery system to modulate macrophage-mediated inflammation, DIO BMDMs were exposed to the nanocarrier containing either the COX-2 inhibitor or no drug (control) (10 μM) and cytokine production was determined. DIO BMDMs treated with the nanocarrier containing COX-2 inhibitor demonstrated a significant reduction in proinflammatory cytokines (*Il1b* and *Tnfa*) and a reciprocal increase in antiinflammatory gene expression (*Il10*, *Arg1*, and *Fizz*) (Figure 4, D and E). To translate these findings in vivo, DIO mice were wounded, and the nanocarriers containing either the COX-2 inhibitor or control were administered daily via local subcutaneous injection to DIO wounds starting on day 1 postwounding. DIO mice treated with the nanocarrier containing the COX-2 inhibitor demonstrated a significant improvement in wound healing (Figure 4F). These results suggest that local macrophage-specific targeting of the PGE_2_ pathway with nanocarriers may represent a novel cell-specific therapeutic strategy for improving healing and controlling macrophage inflammatory phenotype in diabetic wounds.

### Transcription profiling demonstrates upregulation of inflammatory pathways in human diabetic wound monocyte/macrophages.

**Figure 5:**
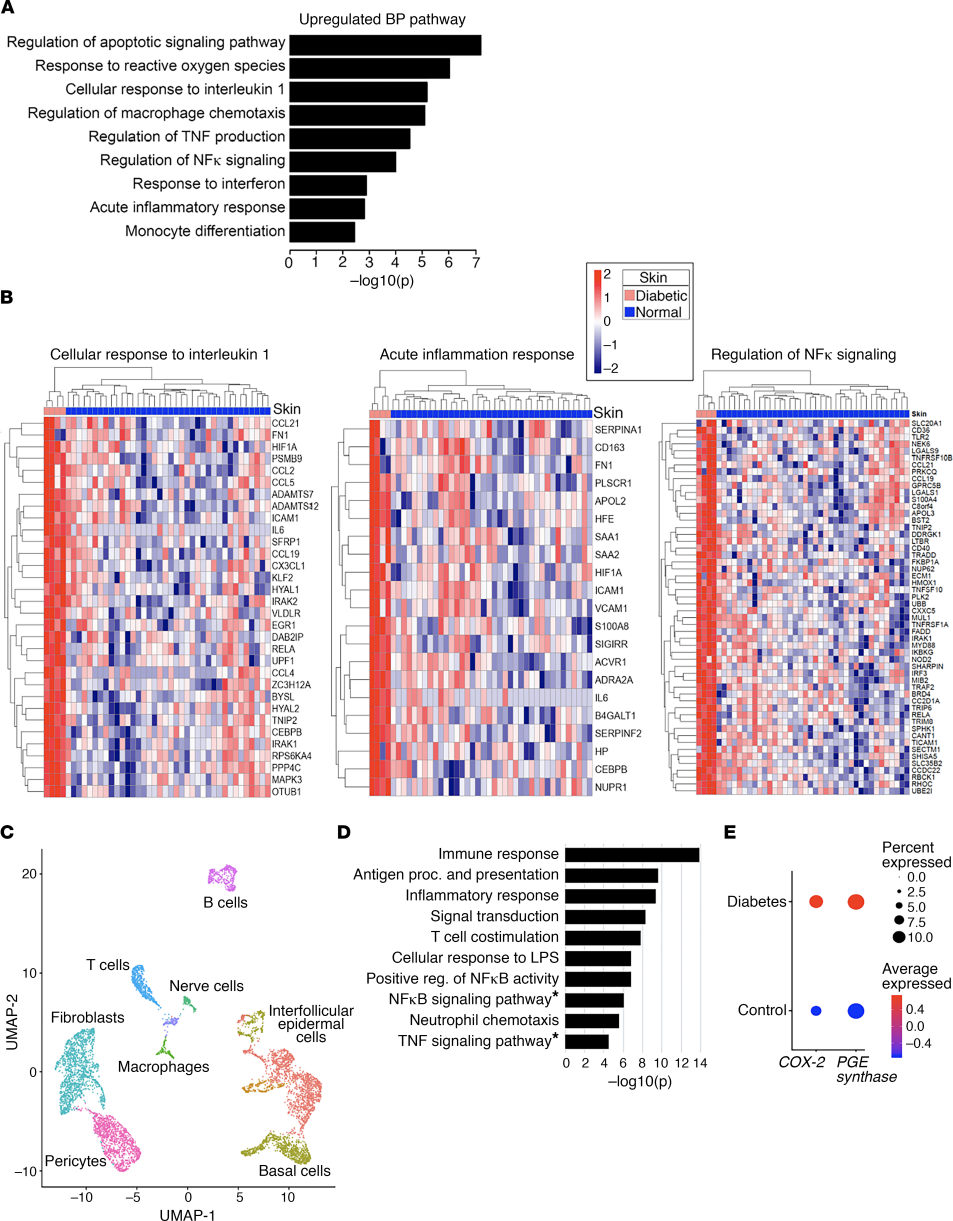
Transcription profiling demonstrates upregulation of inflammatory pathways in human diabetic wound macrophages. (**A**) Graphs demonstrate the highly enriched Gene Ontology (GO) terms in the bulk RNA transcriptomes of diabetic wound tissue (*n* = 4) compared with healthy controls (*n* = 38). (**B**) Heatmap illustrating the expression profiles for selective genes (rows) across different samples (columns; stratified by different skin types) from cellular response to IL-1β, acute inflammatory response, and regulation of NF-κB signaling GO pathway analysis with upregulation in diabetic (*n* = 4) compared with healthy controls (*n* = 38). (**C**) Cluster analysis using the UMAP technique of single-cell sequencing from human T2D and nondiabetic wound samples revealed 10 distinct cell clusters (representative, performed in triplicate). (**D**) GO biological process enrichment and KEGG pathway analysis of macrophage DEGs in T2D samples. KEGG pathways are denoted with *. The combined score metric corresponds to the *P* value (2-sided Fisher exact test) multiplied by the *Z* score of the deviation from the expected rank, and *q* values were determined by Benjamini-Hochberg correction. (**E**) Dot plot demonstrating *COX-2* and *PGE Synthase 1* expression within macrophage population in human T2D and nondiabetic control samples. Dot size corresponds to proportion of cells within the group expressing each transcript, and dot color corresponds to expression level.

Excess COX-2/PGE_2_ in diabetic wound macrophages results in altered macrophage function related to inflammation and host defense. To more fully translate these findings into human disease, we first performed bulk RNA sequencing to compare the biopsies from human diabetic wounds (*n* = 4) versus normal skin samples (*n* = 38), enabling us to conduct a robust analysis of differences in diabetic tissue inflammation and repair. We obtained more than 40 million read pairs per sample and detected 31,777 genes. Principal components analysis demonstrated a complete separation between diabetic and nondiabetic control samples, indicating a dramatic difference in transcriptome between the 2 skin types (Supplemental Figure 5A). We identified 6989 differentially expressed genes (DEGs), including 3476 upregulated genes and 3513 downregulated genes, in skin lesions of diabetic wounds compared with that in healthy control skin, after controlling for family-wise error rate at *P* ≤ 0.05 (i.e., *P* ≤ 1.57 × 10^–6^) and using |log_2_ fold change| ≥ 1 (Supplemental Table 1). Pathway analysis demonstrated diabetic wounds had enrichment for biological functions involved in multiple aspects of the inflammatory cascade and host defense functions, including IL-1β response, monocyte differentiation, macrophage chemotaxis, regulation of inflammatory response, response to reactive oxygen species, regulation of NF-κB transcription factor activity, and apoptotic signaling (selected pathways illustrated in Figure 5, A and B; and Supplemental Figure 5, B–G).

From the bulk RNA sequencing, inflammatory and host defense pathways regulated by monocyte/macrophages were key pathways identified. To further investigate the inflammatory macrophage phenotype and regulatory cellular pathways in diabetic wounds, we performed single-cell RNA sequencing (scRNA-Seq) analysis on a second cohort of biopsies of wounds from human T2D and non-T2D control patients. Cluster analysis using the uniform manifold approximation and projection (UMAP) technique identified 10 cell clusters present in the area of tissue injury. We attributed clusters to their putative identities using gene signatures distinctive to the corresponding clusters (Figure 5C), with several clusters mapped to immune cells. GO and Kyoto Encyclopedia of Genes and Genomes (KEGG) enrichment analysis of the T2D macrophage cluster in comparison with non-T2D control macrophage cluster revealed that diabetic wound macrophages upregulated the expression of genes with roles in immune response, inflammatory response, NF-κB signaling, and TNF signaling pathways (Figure 5D). Specific analysis of human scRNA-Seq data demonstrated that *COX-2* and *PGE Synthase 1* were markedly upregulated in human T2D wound macrophages in comparison with non-T2D controls (Figure 5E). Together, these data highlight a key role of the COX-2/PGE_2_ pathway in human diabetic wound macrophage inflammation.

## Discussion

**Figure 6:**
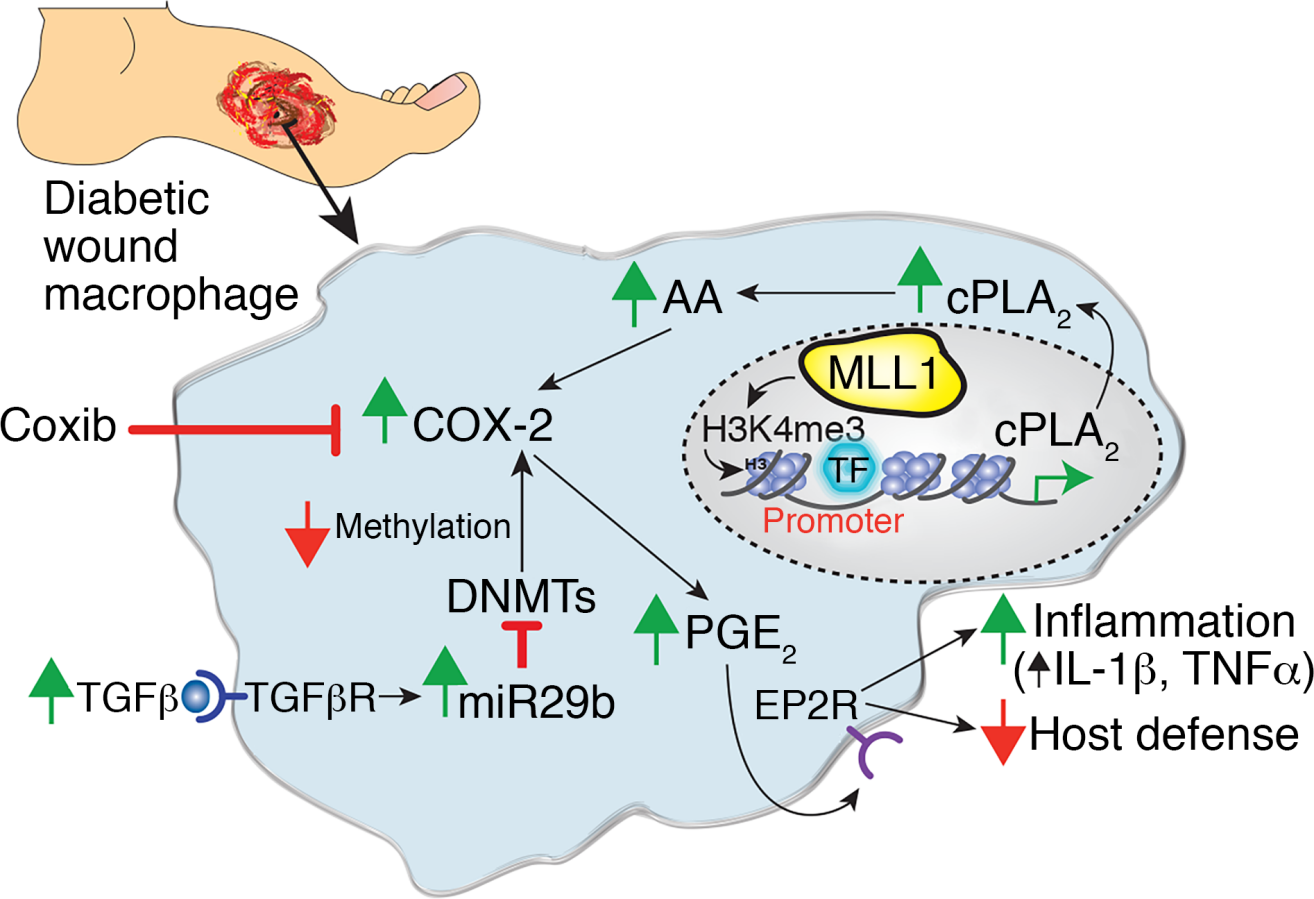
Schematic of PGE_2_ pathway regulation in normal and diabetic wound macrophage.

In this study, we identified that COX-2/PGE_2_–regulated macrophage function plays a central role in normal and diabetic wound repair. Here we find that COX-2/PGE_2_ levels in wound macrophages resulted from epigenetic regulation of essential enzymes in the upstream pathways involved in PGE_2_ synthesis. Specifically, there was increased MLL1-mediated H3K4 trimethylation of the *cPLA_2_* promoter, upregulating *cPLA_2_* gene expression, in monocytes from T2D patients and in murine diabetic wound macrophages. Further, we demonstrate that elevated levels of TGF-β1 in diabetic wounds augmented miR29b, which destabilized DNMT3a/b, resulting in hypomethylation of the *Cox-2* gene promoter and increasing COX-2 levels. This in turn drove PGE_2_ synthesis in diabetic wound macrophages, which through the EP2 receptor spurred wound macrophage inflammatory cytokine production and impaired phagocytosis and killing of common diabetic wound bacterial pathogens. Importantly, macrophage targeting of the COX-2/PGE_2_ pathway through genetic deletion (*Cox2^fl/fl^ Lyz2^Cre+^*), or pharmacologic inhibition with a macrophage-specific PS nanocarrier containing an FDA-approved selective COX-2 inhibitor, improved diabetic wound healing and altered macrophage phenotype (Figure 6). Thus, manipulation of the COX-2/PGE_2_ pathway in wound macrophages offers promise as a cell-specific, local, translational therapy for diabetic wound repair.

It is well established that diabetic wounds fail to heal secondary to an impaired resolution of tissue inflammation driven by wound monocyte/macrophages (40–42). Recent literature has identified a transition between macrophage phenotypes in wounds, with patients with diabetes having a predisposition to harbor a resilient proinflammatory macrophage phenotype beyond the early inflammatory phase of healing (30, 41, 43). The etiology behind this resilient proinflammatory macrophage population has been unclear. Overproduction of PGE_2_ has been demonstrated in a wide range of chronic inflammatory disease processes, including allergic rhinitis, inflammatory bowel disease, as well as multiple cancers (44, 45). The PGE_2_ pathway has been sparingly investigated in diabetes, with limited reports demonstrating reduced PGE_2_ synthesis (46, 47) while other studies exhibited elevated COX-2/PGE_2_ levels in diabetic tissues (48–51). However, there is a paucity of literature regarding the role of COX-2/PGE_2_ in dysfunctional diabetic wound healing and what exists has discordant conclusions (50, 51). In this study, we demonstrate PGE_2_ levels were increased in diabetic macrophages at the end of the inflammatory phase of wound healing during a period when diabetic macrophages fail to transition to an antiinflammatory state. The persistent elevation of PGE_2_ increased macrophage inflammatory cytokine production that was negated with administration of an EP2 receptor antagonist. Further, the adverse effects of elevated PGE_2_ in diabetic macrophages were not limited to cytokine production, but diabetic macrophages also demonstrated impaired macrophage function. In patients with diabetes, nonhealing wounds are at significant risk of infection secondary to impaired diabetic host defense (52, 53); however, the mechanisms underlying this innate immune impairment remain unknown. We found that PGE_2_ inhibited phagocytosis and bacterial killing capabilities of wound macrophages to multiple bacterial pathogens that commonly affect diabetic wounds. Given the upregulated PGE_2_ levels seen in human diabetic tissues and multiple diabetic murine models, it is likely that aberrant PGE_2_ impedes the regulated wound macrophage response that is vital to control infections in diabetic wounds.

Accumulating evidence suggests that epigenetic regulation of gene expression plays a major role in influencing immune cell phenotypes in both normal and pathologic conditions (14, 54). We have previously demonstrated that the histone methyltransferase MLL1 is elevated in human and murine diabetic wound macrophages, resulting in increased H3K4me3 at inflammatory gene promoters (15). Here, we demonstrate MLL1-mediated H3K4me3 increased upstream cPLA_2_ levels in macrophages. The role of cPLA_2_ in diabetes has been investigated in relation to diabetic retinopathy, where increased glucose concentrations contribute to elevated inflammation and aberrant angiogenesis; however, the impact of epigenetic modifications on cPLA_2_ levels, and subsequent COX-2/PGE_2_ production, has not been previously demonstrated to our knowledge (55). Prior investigations in alveolar macrophages following bone marrow transplant have demonstrated that the *Cox-2* promoter can be subjected to changes in DNA methylation (33). Here we demonstrate that TGF-β1 upregulated miR29b in diabetic wound macrophages, resulting in reciprocal downregulation of the inducible DNMTs, specifically DNMT3a and DNMT3b, thereby leading to hypomethylation of the *Cox-2* promoter. TGF-β1 has been characterized to have dual effects on COX-2 (56, 57). One small study in human non–small cell lung cancer A549 cells showed that TGF-β downregulates COX-2 (57); however, the bulk of the literature has shown induction of COX-2 in response to TGF-β in models using mammary epithelial cells and human mesangial cells (35). Overall, our results suggest CpG methylation is an important epigenetic mechanism that regulates COX-2 in macrophages that leads to increased PGE_2_ production.

Because macrophages exhibit different functional phenotypes as tissue repair progresses, the ability to modulate macrophage phenotype in diabetic wounds at a particular time after injury is an attractive therapeutic strategy (58). Antiinflammatory drugs, such as glucocorticoids (e.g., dexamethasone), salicylic acid, and celecoxib, have been clinically tested in a non–cell-specific fashion in patients with diabetes patients (59). However, serious side effects in off-target cells and tissues, including Cushing-like syndromes and gastrointestinal bleeding, have resulted from the systemic use of these therapeutics. Further, the role of COX-2/PGE_2_ can be variable in different cells, and thus, global inhibition of COX-2 may negatively affect the function of other cells in the local milieu involved in the healing process. Nanomedicine offers a potential solution to the systemic, non–cell-specific effects of these drugs by enabling tissue-specific drug delivery through molecular and cellular targeting that can reduce off-target effects (60, 61). An assortment of macrophage-targeted, nanomedicine-based strategies have been contrived using antibodies against macrophage receptors, monosaccharide-based targeting, and PS-mediated delivery (39, 62). To increase cell targeting specificity, certain PSs of glucose, such as dextran, are known to be efficiently and selectively internalized by macrophages due to their expression of dextran-binding C-type lectins and scavenger receptors (63). In the current study, we demonstrate that diabetic macrophages treated with dextran conjugated to a COX-2 inhibitor expressed fewer inflammatory mRNA transcripts. Further, when applied in vivo, via local subcutaneous injection, dextran-conjugated COX-2 inhibitor improved diabetic wound healing without apparent off-target effects in other cells. Therefore, dextran-conjugated COX-2 inhibitors designed to target tissue macrophages, administered at the appropriate time postinjury, may be an ideal therapy to specifically target PGE_2_-mediated chronic inflammation in diabetic wounds. It may be particularly important to target COX-2 inhibitors to tissue macrophages uniquely, as systemic inhibition of COX-2 could also affect prostacyclin and thromboxane levels that are needed for proper healing. Additionally, previous reports have suggested that prostacyclin D_2_ production (mediated by COX-1 activity) in structural cells around wounds is beneficial for tissue repair (64), and thereby cell-specific therapy is likely to be crucial.

Although this study provides valuable insight into the mechanism(s) behind dysregulated inflammation in diabetic wound healing, some limitations must be addressed. First, our finding of overexpression of PGE_2_ in human and murine wound macrophages associated with increased late inflammation is likely due to multiple factors. Although improved wound healing with inhibition of the PGE_2_ pathway by macrophage-targeted PS nanoparticles suggests one potential mechanism, it is likely that altered PGE_2_ production is not the sole contributor to the diabetic macrophage inflammatory phenotype. Furthermore, although MLL1, miR29b, and DNMTs appear to influence the cPLA_2_/COX-2/PGE_2_ pathway in wound macrophages, we recognize that other epigenetic enzymes may regulate aberrant macrophage function in pathologic states as well (4).

In summary, altered COX-2/PGE_2_ levels in wound macrophages result in defective macrophage function, including dysregulated inflammation and impaired phagocytosis and bacterial killing. These findings suggest that the PGE_2_ pathway plays a significant role in dictating wound macrophage function; and it may have significant relevance to macrophage-mediated inflammation in other secondary complications of diabetes.

## Methods

### Study design.

For our murine models, we used 2 well-described diabetic models, DIO and *db/db*, which were randomized to undergo either an acute wound model or a chronic splinted wound model. For these experiments, hyperglycemia was confirmed with glucose tolerance testing before enrollment in the study, and only male mice were used because female mice do not develop DIO. All mice were weighed before the beginning of the experiments, and mice were randomized to different treatment groups. Studies in the genetically engineered murine models of *Mll1^fl/fl^ Lyz2^Cre+^*, *ALk5^fl/fl^ Lyz2^Cre+^*, and *Cox2^fl/fl^ Lyz2^Cre+^* enrolled a minimum of 5 to 7 animals per group and were repeated at least once. Wound healing experiments with coxib containing nanocarriers enrolled 8 to 10 mice per group. The number of animals per group in all the experiments was determined on the basis of prior literature, power calculation, and experience from our previous studies to ensure sufficient sample sizes to allow the detection of statistically significant differences. No animals were excluded from analysis. The number of replicates for each experiment is labeled in all figures or legends. Animal studies were approved by the ethics committees of the Institutional Animal Care and Use Committee (IACUC).

### Mice.

All mice were maintained at the University of Michigan Biomedical Science Research Building in the Unit for Laboratory and Animal Medicine (ULAM). Mouse experiments were conducted with approval from our IACUC, and all regulatory and safety standards were strictly adhered to. C57BL/6, *db/db*, *db/+*, and *Cox-2^fl/fl^* mice were obtained at 6–7 weeks age from The Jackson Laboratory and were maintained in breeding pairs in the ULAM facilities. *Alk5^fl/fl^* mice on a C57BL/6 background were obtained from B. Levi (University of Michigan). Mice with the Alk5 or Cox-2 gene deleted in myeloid cells were generated by mating Cox2*^fl/fl^* or *Alk5^fl/fl^* mice with *Lyz2-Cre* mice (The Jackson Laboratory) (65). Animals were housed in a barrier facility on a 14-hour light/10-hour dark cycle (ambient temperature of 22°C) with free access to water, food (Lab Supply LabDiet Rodent 5001), and bedding (Andersons Lab Bedding Bed-o’Cobs combo).

To induce a “prediabetic” state, male C57BL/6 mice were maintained on a standard normal rodent diet (13.5% kcal saturated fat, 28.5% protein, 58% carbohydrate; LabDiet) or standard high-fat diet (60% kcal saturated fat, 20% protein, 20% carbohydrate; Research Diets, Inc) for 12–18 weeks to induce the DIO model of T2D as previously described (66, 67). After the appropriate period, high-fat diet–fed (DIO) mice developed obesity and insulin resistance with fasting blood sugars in the mid-200s and elevated insulin levels. Of note 2 separate diabetic models were used (DIO murine model and *db/db* murine model) to confirm findings were diabetic disease specific and not secondary to the impact of leptin receptor deficiency on immune cell function. All animals underwent procedures at 20–32 weeks of age with IACUC approval. For these experiments only male mice were used because female mice do not develop DIO. For all surgical procedures, mice were anesthetized with 80 mg/kg i.p. injection of ketamine (Hospira, Inc) and 20 mg/kg xylazine (Lloyd, Inc). Number of mice used per experiment can be found in the figure legend of each corresponding experiment.

### Wound healing assessment and tissue isolation.

For the acute wound model, peripheral wounds were generated by anesthetizing the mice with ketamine, removing the dorsal fur with hair removal cream (Veet), and creating 2 full-thickness skin wounds in the midback with a 4 mm punch biopsy. For the chronic splinted wound model, a splinted full-thickness excisional model of murine wound healing was used to minimize wound contracture (68). Briefly, a 4 mm full-thickness excisional wound was created in duplicate on the shaved dorsum of anesthetized mice. A donut-shaped silicone splint with a 10 mm diameter was centered on the wound and fixed to the skin using an adhesive (Krazy Glue) and interrupted 6-0 nylon sutures (Ethicon). A transparent dressing (Tegaderm) was applied to the wound. Gross images were taken every other day beginning on day 4.

In nanocarrier wound experiments, PS nanocarriers containing either dextran or dextran-conjugated coxib were synthesized in-house. Briefly, in a 50 mL reaction flask, sodium azide (1.3 g, 20 mmol) was added into a solution of 2-bromoethanol (1.25 g, 10 mmol) in 8 mL deionization water. Under nitrogen protection, the mixture was stirred at 80°C overnight. After cooling to room temperature, the water solution was extracted with dichloromethane and ethyl acetate 3 times each. The organic solvents were combined, dried over sodium sulfate, and concentrated to get an oil product as 2-azidoethanol. Meanwhile, lumiracoxib (59 mg, 0.2 mmol, Toronto Research Chemicals) and 2-azidoethanol (43.6 mg, 0.5 mmol) were dissolved in dichloromethane. Then, the mixture was added with 4-Dimethylaminopyridine (4.9 mg, 0.04 mmol) and dicyclohexylcarbodiimide (52 mg, 0.25 mmol). After one night, the mixture was concentrated and purified via a silica gel column using hexanes and dichloromethane as the eluents to obtain azido lumiracoxib. In a 100 mL reaction flask, amino dextran (75 mg) was dissolved in 2.5 mL anhydrous DMSO. Triethylamine (26.2 μL, 0.186 μmol) and cyclooctyne succinimidyl carbonate (2 mg) were added. After one night, the mixture was precipitated into cold ethanol to get a solid product as dextran-COT. Finally, in a 50 mL reaction flask, dextran-COT (50 mg) was dissolved in 2.5 mL anhydrous DMSO. Then, azido lumiracoxib (2.2 mg) was added and reacted overnight. Then, the mixture was precipitated into cold ethanol to obtain a white solid. The final drug loading ratio was 2.1 wt%. Dextran or dextran-conjugated coxib (16 mg/kg) PS nanoparticle injection was performed subcutaneously at 4 points along the wound edge as described previously by our group (69). An 8-megapixel iPad camera with an internal scale was used to record wound size. Wound photographs were then obtained each day postinjury, and wound closure was calculated each day as a percentage of initial wound area. All images were evaluated by 2 independent blinded observers. Wound area was calculated by using ImageJ software (NIH). Wound tissue was harvested postinjury by 6 mm punch biopsy. Wounds were digested at 37°C for 30 minutes with Liberase (50 mg/mL; Roche) and DNase I (20 units/mL; MilliporeSigma). Samples were filtered over a 100 mm cell strainer to produce a single-cell suspension and used in cellular experiments as described below.

### Human wound isolation.

All experiments using human samples were approved by the IRB at the University of Michigan and were conducted in accordance with the principles in the Declaration of Helsinki. For the bulk RNA sequencing cohort, biopsies from human diabetic wounds (*n* = 4) versus normal skin samples (*n* = 38) were collected. For a separate scRNA-Seq cohort, biopsies from human diabetic wounds versus normal skin samples were collected. Within the diabetic patient samples for scRNA-Seq, the average age was 60 years, with all patients having diabetes, hypertension, hyperlipidemia, and coronary artery disease. Within the nondiabetic patient samples, the average age was 70 years, with 50% of patients having hypertension, hyperlipidemia, and coronary artery disease. Wounds were obtained from the specimens using an 8 mm punch biopsy tool and processed for reverse transcription PCR (RT-PCR) as described for the murine wounds. RNA with RNA integrity number scores of greater than 8 were used, and all values were done with comparison to 28S/18S ratios and other housekeeping genes.

### MACS of murine wound and human monocyte cell isolates.

MACS of wound cell isolates was performed as described (70). Briefly, wound cell isolates were incubated with fluorescein isothiocyanate–labeled (FITC-labeled) anti-mouse anti-CD3, anti-NK1.1, anti-CD19, and anti-Ly6G (BioLegend) monoclonal antibodies. Wound isolates were then washed and incubated with anti-FITC microbeads (Miltenyi Biotec, Inc, catalog 130-049-601) and passed through a MACS column (Miltenyi Biotec, Inc). The resultant eluent was then incubated with anti-mouse anti-CD11b microbeads (Miltenyi Biotec, Inc, catalog 130-049-601). The remaining cell population was analyzed by flow cytometry and found to be 97% macrophages, consistent with previous literature (4, 70). For human monocyte isolation, peripheral blood was collected and subjected to RBC lysis and Ficoll-Paque separation (GE Healthcare). Cell suspensions were then treated with anti-human CD14 microbeads. Magnetic separation yielded 95% purity by flow cytometry.

### RNA isolation.

Total RNA extraction was performed with TRIzol (Invitrogen, Thermo Fisher Scientific) using the manufacturer’s directions. RNA was extracted using chloroform, isopropanol, and ethanol. iScript (Bio-Rad) or Superscript III Reverse Transcriptase (Thermo Fisher Scientific) kits were used to synthesize cDNA from extracted RNA. We used cDNA primers for *IL1b* (Mm00434228_m1), *Tnfa* (Mm00443258_m1), *Il12* (Mm01288992_m1), *Nos2* (Mm00440502_m1), *Il10* (Mm01288386_m1), *Arg1* (Mm00475988_m1), *Fizz* (forward TCCAGCTAACTATCCCTCCACT and reverse AGCCACAAGCACACCCAGTAG), *Cpla_2_* (forward GGAGACACGTGAAGAGAGGC and reverse GGATGAGCATGACCCTGAGTAGTT), *Cox-2* (forward TGACCCCCAAGGCTCAAAT and reverse GAACCCAGGTCCGCGCTTATG), *PGE* *Synthase 1* (forward AACCTGGGCGAGTGGATCT and reverse CTGTAAGTGGCTCCAAATGGG), *miR29b* (hsa-miR-29b), *Tgfb1* (Mm00441724_m1), *Ep2* (forward TGCGCTCAGTCCTCTGTTGT and reverse TGGCACTGGACTGGGTAGAAC), *Dnmt3a* (forward GGTGCTTCCTCTAGCAGG and reverse CCGAGATTGCAGGTCAG), *Dnmt3b* (forward TGTGGCTAGTCCTCACGA and reverse CCTTCGTATCCCTCACAC), *Marco* (forward CCTGGACGAGTCGGTCAG and reverse CTTCAGCTCGGCCTCTGTT), and human *cPLA_2_* (forward GGTGGGATTCTCTCTGGTGT and reverse TACCAGGTGGAGCCAGAA). RT-PCR was performed and run on a 7500 Real-Time PCR System (Applied Biosciences, Thermo Fisher Scientific). Data were then reviewed in a relative quantification analysis to the 18S ribosomal RNA (2^–ΔΔCt^). All samples were assayed in triplicate.

### Bulk RNA sequencing and scRNA-Seq analyses.

Generation of single-cell suspensions for scRNA-Seq was performed as follows: Skin was harvested via punch biopsy from diabetic and nondiabetic control patient wounds. Samples were incubated overnight in 0.4% Dispase (Life Technologies, Thermo Fisher Scientific) in HBSS (Gibco, Thermo Fisher Scientific) at 4°C. Epidermis and dermis were separated. Epidermis was digested in 0.25% Trypsin-EDTA (Gibco, Thermo Fisher Scientific) with 10 units/mL DNase I (Thermo Fisher Scientific) for 1 hour at 37°C, quenched with FBS (Atlanta Biologicals), and strained through a 70 μM mesh. Dermis was minced, digested in 0.2% Collagenase II (Life Technologies, Thermo Fisher Scientific) and 0.2% Collagenase V (MilliporeSigma) in plain medium for 1.5 hours at 37°C, and strained through a 70 μM mesh. Epidermal and dermal cells were combined in a 1:1 ratio for scRNA-Seq by the University of Michigan Advanced Genomics Core on the 10x Genomics Chromium System. Libraries were sequencing on the Illumina NovaSeq 6000 sequencer. NovaSeq was used as the sequencing platform to generate 151 bp paired-end reads. We then conducted adapter trimming and quality control procedures as described previously (71, 72). The reads were then mapped using STAR (73) to build human GRCh37, and gene expression levels were quantified and normalized by HTSeq (74) and DESeq2 (75), respectively. Negative binomial models in DESeq2 were used to conduct differential expression analysis. To increase the sample size of the control samples, we used the skin biopsies obtained from our previous study (76). For bulk RNA sequencing and scRNA-Seq data accession, numbers include GSE154556 and GSE154557 (Gene Expression Omnibus). For scRNA-Seq data, data processing, including quality control, read alignment, and gene quantification, was conducted using the 10X Genomics Cell Ranger software. Seurat was then used for normalization, data integration, and clustering analysis (77). Clustered cells were mapped to corresponding cell types by matching cell cluster gene signatures with putative cell type–specific markers.

### Prostaglandin and leukotriene pathway ELISAs.

Wound monocyte/macrophages were MACS isolated and cultured for 1 hour in serum-free RPMI. After stimulation, cell-free supernatants were collected and analyzed by specific enzyme immunoassay kits for cysteinyl leukotriene and leukotriene B_4_ (all ELISAs from Cayman Chemical) according to the manufacturer’s instructions.

### BMDM culture.

Femurs and tibias of mice were flushed with RPMI, and BMDMs were cultured as described previously (30). After initial cell counting and plating in RPMI, FBS, L-cell supernatant, glutamine, and penicillin/streptomycin, cells were cultured for 7 days. Briefly, on day 7, cells were replated in triplicate (3 × 10^5^ cells/well). When indicated, BMDMs were stimulated with/without LPS (100 ng/mL). For nanoparticle in vitro experiments, BMDMs were stimulated with PS nanoparticles containing coxib (10 μM) for 4 hours. For human monocyte-derived macrophages, CD14^+^ monocytes were cultured in complete media supplemented with 50 ng/mL of M-CSF (R&D Systems, Bio-Techne) for 1 week. Adherent cells were washed and harvested with trypsin/EDTA (Lonza).

### Bisulfite conversion and pyrosequencing.

Pyrosequencing was performed as previously described (33). Briefly, DNA was isolated from 5 × 10^5^ to 1 × 10^6^ cells using the DNeasy kit (QIAGEN). The Zymo Research EZ DNA Methylation Kit was used according to the manufacturer’s instructions. In brief, samples were treated with the bisulfite reagent overnight in a thermocycler set to cycle between 95°C for 30 seconds followed by 50°C for 60 minutes for 16 cycles. After bisulfite conversion, the Ptgs2 (COX-2) promoter was PCR amplified using primer set ADS2001 from EpigenDx. The biotinylated PCR product was isolated using sepharose beads, denatured, and sequenced using the ADS2001 sequencing primer (EpigenDx). The PCR conditions for determining the methylation profile of the DNA using ADS2001 are as follows: 95°C 15 minutes; (95°C 30 seconds; 54°C 30 seconds; 72°C 30 seconds); 72°C 5 minutes; 4°C hold. PCR conditions using ADS2002 were 95°C 15 minutes; (95°C 30 seconds; 60°C 30 seconds; 72°C 30 seconds); 72°C 5 minutes; 4°C hold.

### In vitro phagocytosis assays.

*P*. *aeruginosa* (PAO1) stock were grown in nutrient broth (Difco; BD). The culture concentration was determined via absorbance measurements, as previously described (78). For FITC labeling, a culture was centrifuged and washed 2 times by resuspending the pellet in 1 mL sterile PBS and transferring it into a sterile tube. The bacteria were heat killed by autoclaving for 20 minutes and resuspended at 10^9^–10^10^ CFU/mL in 0.1 M NaHCO_3_; 0.2 mg/mL FITC (MilliporeSigma) in DMSO was added to heat-killed *P*. *aeruginosa* and allowed to incubate in the dark for 1 hour at room temperature. Following FITC labeling, heat-killed *P*. *aeruginosa* were washed and resuspended in sterile PBS at 6 × 10^9^ CFU/mL.

BMDMs were harvested, as described above, and the ability of the BMDMs from control and DIO mice to phagocytize via opsonin-independent pathways was examined using a 300:1 ratio of FITC-labeled *P*. *aeruginosa* to BMDMs. Briefly, 2 × 10^5^ BMDMs were plated on a half-area black 96-well plate and incubated overnight at 37°C. The medium was changed to serum-free medium, and 10 mL FITC-labeled *P*. *aeruginosa* was added. After 2 hours at 37°C, trypan blue was added to quench extracellular fluorescence, and phagocytosis was quantified (10).

### Bacterial killing assay.

The ability of BMDMs from control and DIO mice to kill *P*. *aeruginosa* was quantified, as described elsewhere (79). Briefly, BMDMs from WT and DIO mice were aliquoted in RPMI + 10% FBS into a 24-well plate at 5 × 10^5^ cells per well. *E*. *coli* (ATCC 700973 serotype 018ac:k1:H7) at MOI of 0.3 or 3 were placed onto macrophages. Cells and bacteria were then spun at 400*g*/5 minutes at 4°C. Mixture was then resuspended in RPMI + 10% FBS and incubated at 37°C for 30 minutes. Following this, the monolayer of cells was incubated in RPMI + 10% FBS + gentamicin (50 μg/mL) to kill extracellular bacteria. At 3 hours following resuspension, 1 mL of 0.1% Triton X-100 was applied. Serial dilutions of cell lysates were then applied on trypticase soy agar plates and colony-forming units counted.

### In vitro miRNA transfection.

Primary wound monocyte/macrophages were obtained from control and DIO mice on day 3 postwounding. Wound monocyte/macrophages (2 × 10^6^) were transfected with corresponding miRNA (control miR or miR29b) as outlined by the manufacturer (Thermo Fisher Scientific). Briefly, 30 nM of miRNA was diluted and gently mixed in Opti-MEM I Reduced Serum Medium (Life Technologies, Thermo Fisher Scientific). Lipofectamine RNAiMAX Reagent (Life Technologies, Thermo Fisher Scientific) was mixed with Opti-MEM/miRNA and rocked at room temperature for 20 minutes before transfection. For real-time RT-PCR or bisulfite conversion studies, transfections were performed for 24 hours for DNMT and miR29b analysis and 48 hours for bisulfite conversion studies of the COX-2 promoter. For bisulfite conversion and pyrosequencing, a total of 2 × 10^5^ primary wound monocyte/macrophages were transfected with control miRNA or miR29b (30 nM), as described above.

### Statistics.

GraphPad Prism software version 6.0 was used to analyze the data. Data were analyzed for normal distribution and equal variance with either the D’Agostino’s K-squared test or Shapiro-Wilk test; then statistical significance between multiple groups was determined using a 1-way analysis of variance test followed by Newman-Keuls post hoc test. For all single-group comparisons, if data passed normality test, we used a 2-tailed Student’s *t* test. Otherwise data were analyzed using the Mann-Whitney *U* test. All data are representative of at least 2 independent experiments as detailed in the figure legends. A *P* value of less than or equal to 0.05 was significant.

## Author contributions

FMD, LCT, JEG, SLK, BBM, and KAG designed the experiments. FMD, AD, AJ, CW, HP, AS, AO, SH, SR, JL, WJM, and JF performed experiments. FMD, AD, AJ, CW, HD, AS, AO, SH, ACB, COA, S Wolf, S Weidinger, LCT, RW, and JEG analyzed data. FMD, LCT, JEG, AD, AJ, CW, HD, AS, AO, SH, SR, JL, WJM, BBM, and KAG prepared the manuscript.

## Supplementary Material

Supplemental data

## Version 1 09/03/2020

Electronic publication
