# Supplementary material for: Epigenetic regulation of the PGE2 pathway modulates macrophage phenotype in normal and pathologic wound repair
Source: JCI Insight. 2020 Sep 3;5(17):e138443. doi: 10.1172/jci.insight.138443 (PMC7526451; doi:10.1172/jci.insight.138443)
Supplement: Supplemental data [file jciinsight-5-138443-s064.pdf]

Supplemental Figure 1

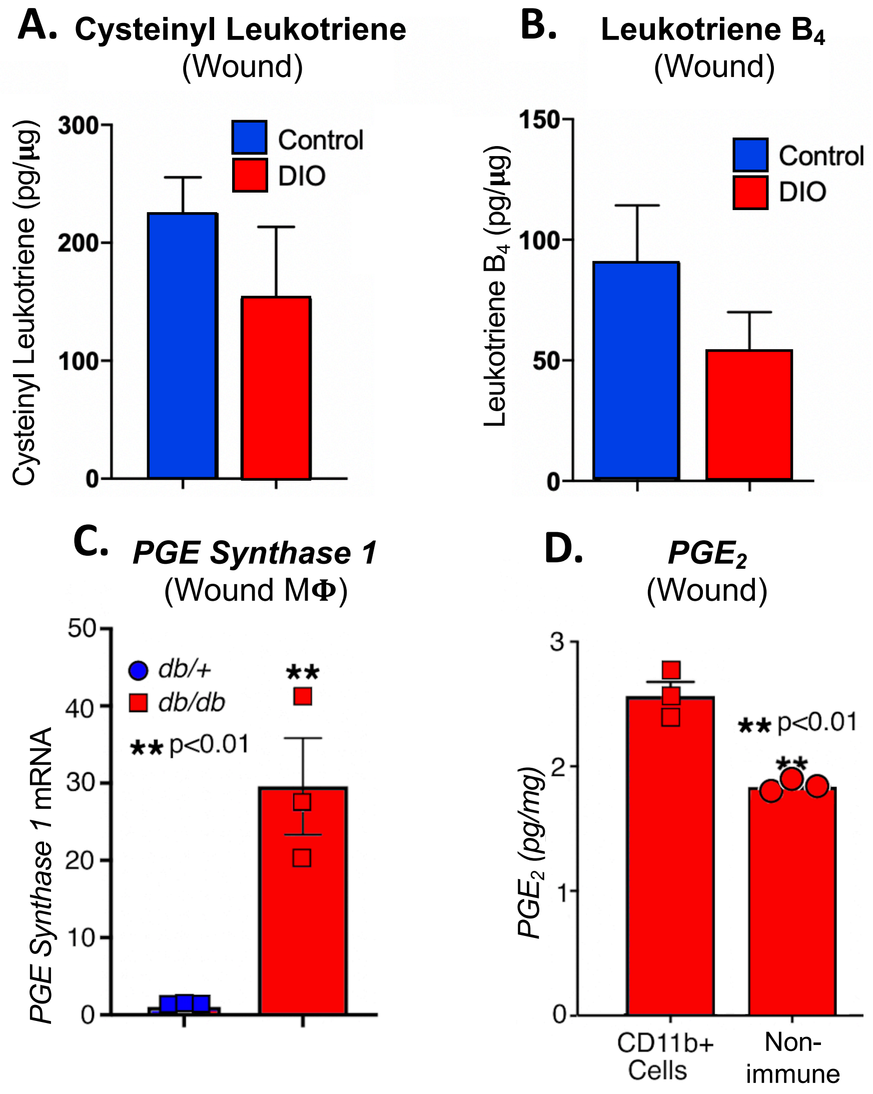

Supplemental Figure 1. Leukotriene and lipid mediators similar in diabetic and control wounds. (A, B) Wound monocyte/macrophages (MΦs) ($CD11b^+[CD3^-/CD19^-/NK1.1^-/Ly6G^-/CD11b+]$) were isolated on day 3 post-wounding and analyzed for secretion of Cysteinyl Leukotriene (A) and Leukotriene B4 (B) in DIO and control mice (N=3/group, repeated two times). (C) Wound monocyte/macrophages (MΦs) ($CD11b^+[CD3^-/CD19^-/NK1.1^-/Ly6G^-/CD11b^+]$)) were isolated from *db/db* mice and controls and analyzed for *PGE synthase 1* (n=3/group, repeated two times in triplicate). (D) Wound cells were sorted from *db/db* and PGE_2 levels were analyzed in myeloid cells ($CD11b^+[CD3^-/CD19^-/NK1.1^-/Ly6G^-/CD11b^+]$) and non-immune cells ($CD11b^-[CD3^-/CD19^-/NK1.1^-/Ly6G^-/CD11b^-]$) (n=3/group, repeated two times). Data are presented as the mean±SEM. Data were first analyzed for normal distribution and if data passed normality test, 2-tailed Student's t test was used.

Supplemental Figure 2

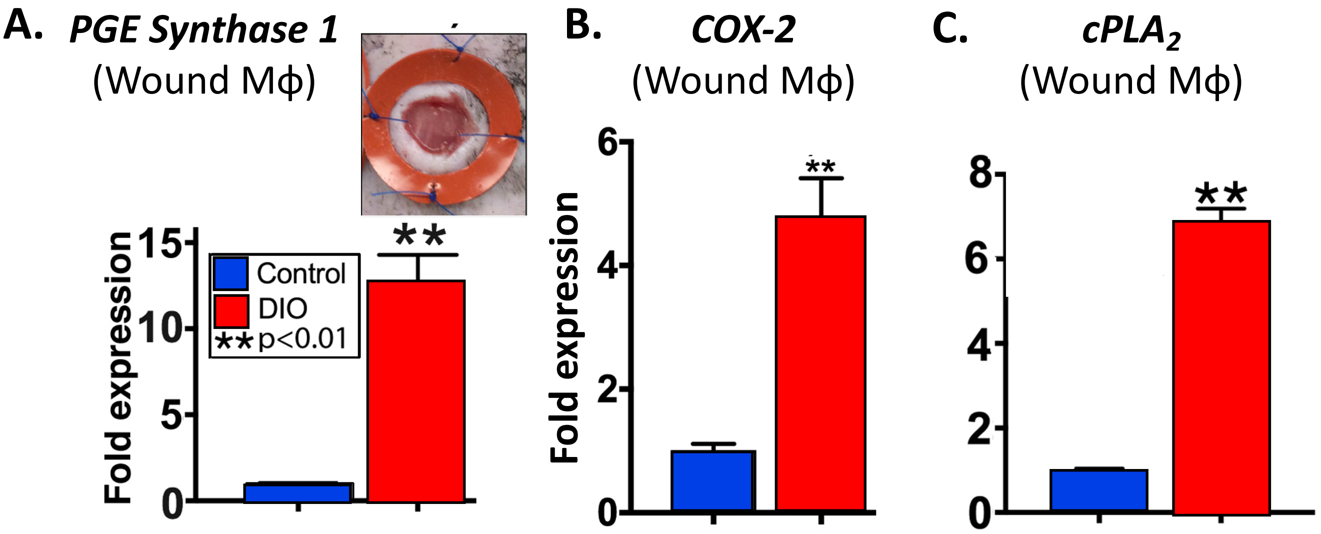

Supplemental Figure 2. Increased expression of the COX-2/PGE$_2$ pathway in DIO mice in chronic splinted wound model. (A-C) Wound monocyte/macrophages (MΦs) (CD11b$^+$[CD3$^-$/CD19$^-$/NK1.1$^-$/Ly6G$^-$/CD11b$^+$]) were isolated on day 5 from DIO and control mice subjected to a chronic wound model (splinted wound; inset) and analyzed for *PGE synthase 1, Cox-2,* and *cpla$_2$* (n=3/group, repeated two times in triplicate). Data are presented as the mean±SEM. Data were first analyzed for normal distribution and if data passed normality test, 2-tailed Student's t test was used.

Supplemental Figure 3

A.

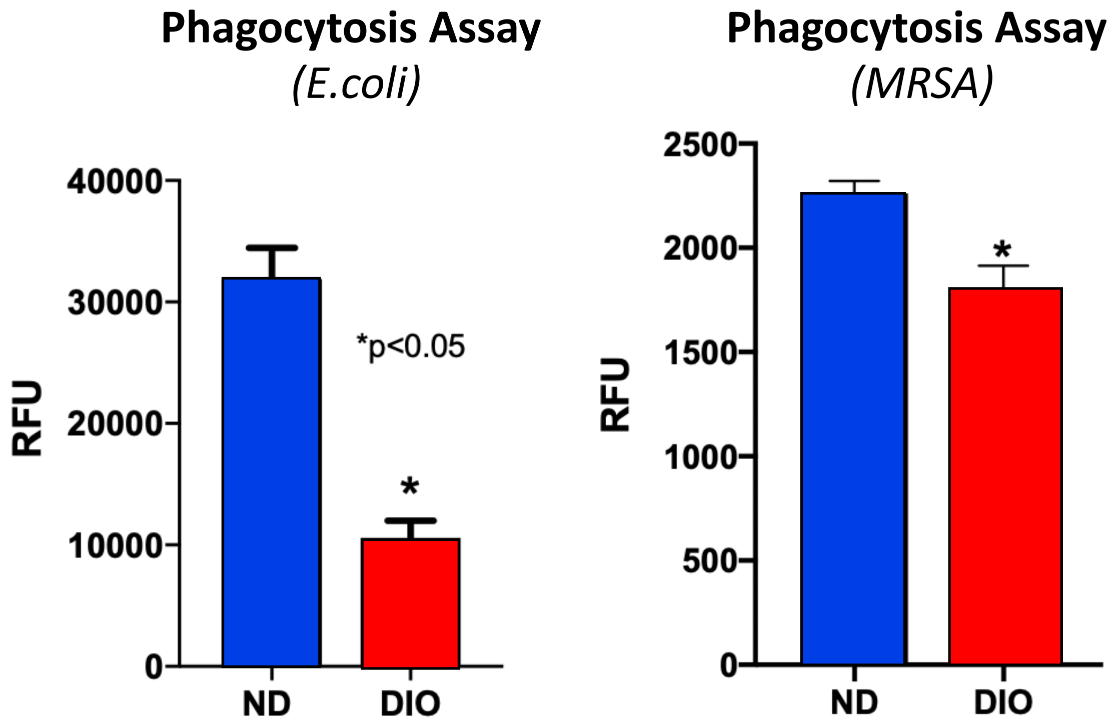

Supplemental Figure 3. Diabetic wound Mϕs exhibit impaired phagocytosis. (A,B). In vivo monocyte/macrophages (MΦs) (CD11b$^+$[CD3$^-$/CD19$^-$/NK1.1$^-$/Ly6G$^-$/CD11b$^+$]) from DIO mice or controls were incubated with fluorescently labeled *E.coli* (A) or *MRSA* (B) for 2h and the percent of cells ingesting bacteria were determined in relative fluorescence units (RFU) (n=4/group, repeated twice). Data are presented as the mean±SEM. *P < 0.05. Data were first analyzed for normal distribution and if data passed normality test, 2-tailed Student’s t test was used.

Supplemental Figure 4

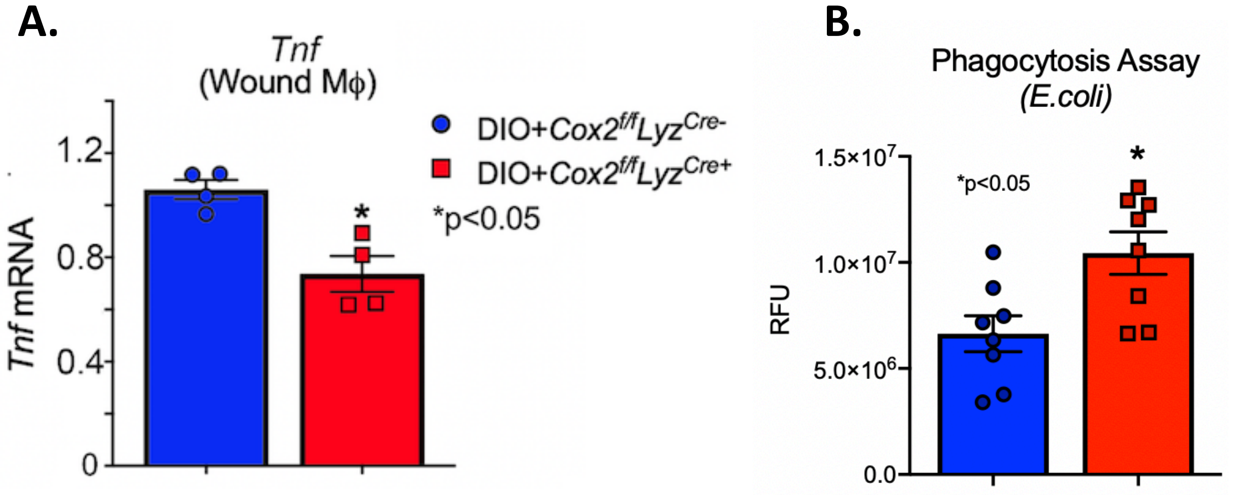

Supplemental Figure 4. Macrophage-specific Genetic Depletion of Cox-2 Decrease Inflammation and Improve Phagocytosis. (A) Wound monocyte/macrophages (MΦs) ($CD11b^+$[$CD3^-$/$CD19^-$/$NK1.1^-$/$Ly6G^-$/$CD11b^+$]) from DIO+*Cox2*$^{f/f}$*Lyz2*$^{Cre+}$ and littermate controls (DIO+*Cox2*$^{f/f}$*Lyz2*$^{Cre-}$) were isolated and analyzed for *Tnf* gene expression by qPCR (n=3/group, repeated twice). (B) BMDM from DIO+*Cox2*$^{f/f}$*Lyz2*$^{Cre+}$ and littermate controls (DIO+*Cox2*$^{f/f}$*Lyz2*$^{Cre-}$) were incubated with fluorescently labeled *E.coli* for 2h and the percent of cells ingesting bacteria were determined in relative fluorescence units (RFU) (n=8/group, repeated once). $^*P < 0.05$. Data were first analyzed for normal distribution and if data passed normality test, 2-tailed Student's t test with Welch's correction was used.

Supplemental Figure 5

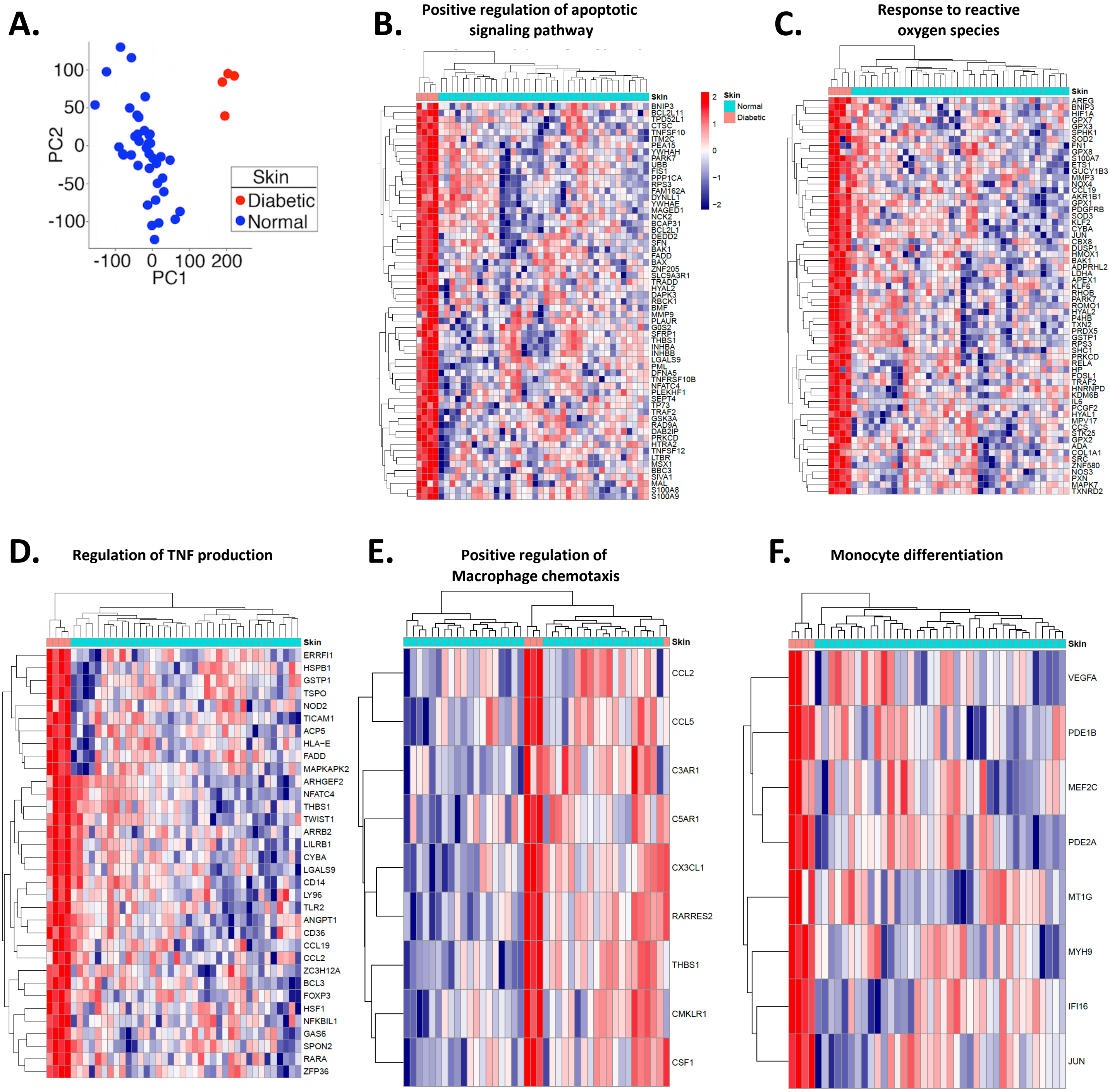

Supplemental Figure 5

G.

Response to interferon β

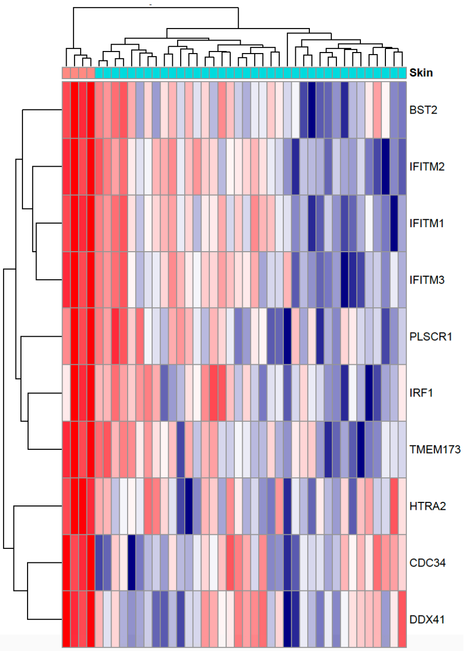

Supplemental Figure 5. Gene expression within GO pathway analysis of diabetic and control. (A) Principal components analysis of microarray data in wound tissue from diabetic (n=4) and healthy controls (n=38). (B-G) Heatmap illustrating the expression profiles for selective genes from GO pathway analysis with progressive pattern of upregulation from diabetic (n=4) compared to healthy controls (n=38).

Supplemental Table 1

	log2FoldChange	pvalue	padj
**RMRP**	-11.57	0.00E+00	0.00E+00
**RN7SK**	-11.78	0.00E+00	0.00E+00
**RPPH1**	-12.60	0.00E+00	0.00E+00
**RN7SL1**	-8.70	5.92E-291	4.71E-287
**ZNF460**	-6.10	4.02E-290	2.56E-286
**SCARNA2**	-8.59	1.71E-270	9.07E-267
**RN7SL2**	-7.92	1.36E-226	6.19E-223
**HIST1H1E**	-9.71	2.31E-200	9.19E-197
**POMK**	-4.19	2.61E-176	9.21E-173
**SCARNA7**	-9.92	1.03E-174	3.29E-171
**SNORA73B**	-10.33	1.61E-160	4.66E-157
**SNORD3A**	-11.79	1.10E-151	2.93E-148
**GADD45B**	4.06	5.48E-151	1.34E-147
**HIST2H2AC**	-7.56	4.01E-148	9.11E-145
**KLHL11**	-3.47	6.96E-121	1.47E-117
**ZNF121**	-2.47	2.78E-116	5.52E-113
**GDAP2**	-2.80	1.68E-112	3.14E-109
**ZNF805**	-2.71	6.38E-112	1.07E-108
**HIST1H4C**	-7.77	6.41E-112	1.07E-108
**ZBED6**	-2.76	7.26E-112	1.15E-108
**LNPEP**	-2.72	2.34E-108	3.55E-105
**TOR1AIP2**	-2.14	2.88E-104	4.16E-101
**PWAR5**	-4.34	6.54E-103	9.03E-100
**ALMS1**	-2.20	9.21E-103	1.22E-99
**PTMS**	3.42	6.46E-99	8.21E-96
**SNORD17**	-11.44	9.71E-99	1.19E-95
**TMPPE**	-3.53	1.33E-97	1.57E-94
**JUN**	5.00	1.05E-95	1.20E-92
**HIST1H1D**	-9.22	1.67E-95	1.83E-92
**CTC-444N24.7**	-6.85	1.18E-94	1.25E-91
**SCARNA9**	-7.42	7.96E-94	8.16E-91
**RBM15**	-2.03	3.38E-93	3.36E-90
**BAX**	3.20	5.73E-93	5.52E-90
**ZBTB37**	-2.55	2.09E-92	1.95E-89
**AKT1S1**	2.27	8.91E-90	8.09E-87
**XLOC_013174**	-4.23	3.72E-89	3.29E-86
**HIST1H2BH**	-5.78	6.21E-89	5.33E-86
**HIST2H2BF**	-4.91	5.23E-88	4.37E-85
**FLOT1**	3.43	2.78E-87	2.27E-84
**HIST1H4E**	-9.24	3.62E-85	2.87E-82
**RIF1**	-2.16	1.16E-84	8.97E-82
**TULP4**	-2.55	1.41E-84	1.07E-81

**HIST1H1C**	-4.13	2.16E-84	1.59E-81
**EXPH5**	-3.63	3.59E-84	2.60E-81
**HIST1H2AE**	-6.05	6.59E-84	4.66E-81
**HIST1H2AG**	-6.23	2.10E-83	1.45E-80
**MANBAL**	1.64	8.63E-83	5.83E-80
**RC3H1**	-2.33	1.48E-82	9.77E-80
**SNHG3**	-5.49	1.14E-80	7.38E-78
**HIST1H4H**	-5.25	4.57E-80	2.90E-77
**VWA1**	3.57	4.76E-80	2.96E-77
**ANP32B**	2.40	9.19E-80	5.61E-77
**FBLIM1**	3.33	1.34E-79	8.06E-77
**RPL28**	2.60	1.11E-78	6.56E-76
**ZNF451**	-1.79	5.35E-78	3.09E-75
**GSK3A**	1.94	1.42E-77	8.04E-75
**CCDC124**	3.03	2.11E-77	1.18E-74
**SYNE1**	-3.33	3.01E-77	1.65E-74
**CEP192**	-1.75	5.03E-77	2.71E-74
**FAAP20**	2.72	4.16E-76	2.20E-73
**HIST1H2BN**	-4.74	1.19E-75	6.18E-73
**MT2A**	3.71	1.99E-75	1.02E-72
**ZNF850**	-2.44	2.64E-75	1.33E-72
**PTAR1**	-1.85	7.78E-75	3.86E-72
**FLOT2**	2.75	4.77E-74	2.33E-71
**NUFIP2**	-1.57	9.37E-74	4.51E-71
**CDC42EP5**	4.53	5.73E-73	2.72E-70
**U2AF2**	1.50	1.01E-72	4.72E-70
**CAPZB**	1.78	1.27E-72	5.85E-70
**MMP24-AS1**	2.57	1.31E-72	5.93E-70
**CTC-444N24.11**	-2.38	4.74E-72	2.12E-69
**COPS6**	1.87	1.70E-71	7.50E-69
**HIST1H2BC**	-4.50	1.87E-71	8.15E-69
**MYO9A**	-1.67	2.58E-71	1.11E-68
**KNTC1**	-1.93	2.68E-71	1.14E-68
**SYNE2**	-2.70	3.08E-71	1.29E-68
**USP34**	-1.92	9.48E-71	3.91E-68
**SCARNA21**	-10.25	2.73E-70	1.11E-67
**C10orf12**	-2.89	3.31E-70	1.33E-67
**INAFM1**	3.15	6.86E-70	2.72E-67
**MKLN1**	-1.83	1.12E-69	4.40E-67
**TNIP1**	1.56	2.46E-69	9.53E-67
**CEBPD**	2.59	8.77E-69	3.36E-66
**RNF169**	-1.53	9.84E-69	3.72E-66
**KANSL1L**	-2.18	2.24E-68	8.36E-66
**NDUFA4L2**	4.69	2.49E-68	9.22E-66
**BANF1**	2.84	3.52E-68	1.29E-65

**RAB13**	2.99	8.29E-68	2.99E-65
**PRR14L**	-1.73	1.88E-67	6.71E-65
**BARD1**	-2.71	2.23E-67	7.89E-65
**RN7SL3**	-8.29	2.55E-67	8.85E-65
**HIST1H2BG**	-4.68	2.56E-67	8.85E-65
**INO80D**	-2.44	3.47E-67	1.18E-64
**GAN**	-4.03	4.99E-67	1.69E-64
**FKBP2**	1.90	1.89E-66	6.32E-64
**HIST1H3G**	-7.42	2.85E-66	9.42E-64
**C11orf96**	5.51	3.69E-66	1.21E-63
**MDM2**	-1.52	7.39E-66	2.40E-63
**HIST1H3H**	-5.37	8.59E-66	2.76E-63
**PARD6B**	-2.83	1.07E-65	3.40E-63
**UBALD2**	3.26	1.74E-65	5.46E-63
**CBX5**	-1.85	1.94E-65	6.05E-63
**UBE2J2**	1.28	2.10E-65	6.47E-63
**NUP155**	-1.69	2.69E-65	8.22E-63
**EVI5**	-2.02	2.97E-65	8.99E-63
**PRCC**	1.73	3.51E-65	1.05E-62
**UBN2**	-2.43	4.79E-65	1.42E-62
**NR2C2**	-1.38	5.45E-65	1.61E-62
**SSBP3**	1.54	5.62E-65	1.64E-62
**ZNF283**	-2.07	1.75E-64	5.06E-62
**CCM2**	2.35	1.99E-64	5.68E-62
**SBNO2**	2.21	2.22E-64	6.31E-62
**TSR3**	1.87	3.26E-64	9.17E-62
**PHC3**	-1.80	4.67E-64	1.30E-61
**C11orf31**	2.12	1.13E-63	3.10E-61
**OAZ2**	1.79	1.13E-63	3.10E-61
**XRN1**	-1.90	1.60E-63	4.34E-61
**CEP350**	-1.91	1.76E-63	4.74E-61
**ACOX1**	-1.99	2.17E-63	5.80E-61
**ATF5**	2.55	2.96E-63	7.84E-61
**STRA13**	2.13	6.46E-63	1.70E-60
**PAF1**	1.98	1.22E-62	3.17E-60
**BIRC6**	-1.92	2.18E-62	5.64E-60
**NIN**	-1.41	4.05E-62	1.04E-59
**FKBP8**	2.16	1.29E-61	3.28E-59
**ZNF790**	-1.62	2.17E-61	5.48E-59
**USF3**	-1.84	5.07E-61	1.27E-58
**SLC8A1**	-4.33	5.15E-61	1.28E-58
**RALY**	1.26	6.12E-61	1.51E-58
**SMARCB1**	1.84	8.42E-61	2.06E-58
**HIST1H4F**	-9.14	1.16E-60	2.81E-58
**IKZF2**	-3.50	1.23E-60	2.97E-58

**C11orf68**	2.08	1.94E-60	4.65E-58
**USE1**	1.90	2.11E-60	4.99E-58
**CTB-50L17.10**	1.83	3.37E-60	7.94E-58
**FBXL15**	1.96	3.48E-60	8.14E-58
**ZNF417**	-2.10	4.65E-60	1.08E-57
**COL4A2**	5.58	1.43E-59	3.29E-57
**AQP9**	-3.41	1.59E-59	3.64E-57
**NAA60**	1.85	1.63E-59	3.69E-57
**RNU4-2**	-12.74	2.85E-59	6.41E-57
**USF2**	1.67	3.20E-59	7.16E-57
**GADD45A**	2.62	7.72E-59	1.72E-56
**TNFRSF1A**	1.68	9.97E-59	2.20E-56
**NHLRC2**	-1.99	1.59E-58	3.48E-56
**PHPT1**	2.20	1.62E-58	3.52E-56
**BASP1**	3.78	2.01E-58	4.35E-56
**ZFP30**	-1.79	2.22E-58	4.76E-56
**ZNF124**	-3.29	2.94E-58	6.28E-56
**TRAPPC1**	2.06	4.13E-58	8.75E-56
**PITPNB**	2.39	4.79E-58	1.01E-55
**QSER1**	-1.82	8.05E-58	1.68E-55
**HIST1H1B**	-10.92	9.53E-58	1.98E-55
**SNORA12**	-8.74	1.19E-57	2.46E-55
**BTC**	-5.49	1.33E-57	2.72E-55
**CDK6**	-2.62	1.51E-57	3.07E-55
**HNRNPM**	1.37	2.18E-57	4.42E-55
**PEX14**	1.62	2.73E-57	5.50E-55
**CDC37**	1.70	3.21E-57	6.42E-55
**NOTCH2NL**	-2.95	3.24E-57	6.43E-55
**IRGQ**	-1.51	3.62E-57	7.14E-55
**CORO1B**	1.78	4.79E-57	9.39E-55
**TBX2**	5.72	8.32E-57	1.62E-54
**HIST1H2BE**	-7.13	1.47E-56	2.84E-54
**STX5**	1.30	1.98E-56	3.82E-54
**SENP5**	-1.27	3.11E-56	5.96E-54
**ZNF579**	2.31	3.22E-56	6.13E-54
**FAM213B**	2.36	3.34E-56	6.31E-54
**SLC9A3R2**	4.34	4.22E-56	7.93E-54
**TMEM256-PLSCR3**	2.48	5.86E-56	1.10E-53
**RP11-255H23.2**	-3.67	6.12E-56	1.14E-53
**ZNF414**	2.90	6.31E-56	1.17E-53
**YPEL3**	1.64	6.97E-56	1.28E-53
**ABHD4**	2.02	1.06E-55	1.94E-53
**POLR2E**	1.79	1.11E-55	2.01E-53
**CCDC9**	1.99	1.67E-55	3.02E-53
**FER**	-1.73	1.94E-55	3.48E-53

**MAF1**	1.62	4.22E-55	7.53E-53
**VPS28**	1.67	6.36E-55	1.13E-52
**FAM127B**	2.75	6.47E-55	1.14E-52
**SHANK3**	3.58	7.34E-55	1.29E-52
**HIST4H4**	-3.96	7.94E-55	1.39E-52
**CTD-3099C6.9**	-3.43	9.10E-55	1.58E-52
**OTUD5**	1.26	1.05E-54	1.81E-52
**HIST1H2BJ**	-5.23	1.07E-54	1.83E-52
**STAG1**	-1.17	1.32E-54	2.26E-52
**PFDN2**	2.32	1.84E-54	3.13E-52
**KIAA1109**	-1.91	3.46E-54	5.85E-52
**NOTCH2**	-1.62	3.57E-54	6.00E-52
**ZFP36**	3.35	4.95E-54	8.29E-52
**SSH3**	2.09	8.99E-54	1.49E-51
**CCDC12**	1.86	1.15E-53	1.90E-51
**LSM7**	2.24	1.21E-53	2.00E-51
**GPX1**	2.99	1.36E-53	2.23E-51
**CSK**	2.01	1.42E-53	2.31E-51
**ZNF787**	2.08	3.01E-53	4.88E-51
**COL4A1**	5.83	4.03E-53	6.51E-51
**ZNF264**	-1.90	5.33E-53	8.56E-51
**NBEAL1**	-2.51	5.42E-53	8.66E-51
**JOSD2**	2.60	8.25E-53	1.31E-50
**USP37**	-1.74	8.41E-53	1.33E-50
**ARHGDIA**	2.39	8.61E-53	1.35E-50
**DOPEY1**	-1.85	1.65E-52	2.58E-50
**RALGAPA1**	-2.06	1.65E-52	2.58E-50
**DENND4C**	-1.83	2.71E-52	4.20E-50
**PLEKHM3**	-2.18	2.73E-52	4.21E-50
**ESAM**	4.90	3.01E-52	4.62E-50
**TMSB10**	3.25	3.06E-52	4.68E-50
**GNB4**	-2.50	3.75E-52	5.70E-50
**ARID2**	-1.57	5.71E-52	8.64E-50
**BRWD3**	-2.14	5.92E-52	8.91E-50
**NFAT5**	-2.47	6.45E-52	9.66E-50
**MARCKSL1**	3.52	7.53E-52	1.12E-49
**TNFRSF19**	-2.17	1.15E-51	1.71E-49
**LCOR**	-1.63	1.19E-51	1.77E-49
**WIF1**	-7.62	1.34E-51	1.97E-49
**HM13**	1.53	1.73E-51	2.53E-49
**ALG10**	-1.95	2.17E-51	3.17E-49
**MIR17HG**	-5.01	2.20E-51	3.19E-49
**ZNF81**	-2.01	2.22E-51	3.21E-49
**LTN1**	-1.66	2.45E-51	3.53E-49
**MED13**	-1.68	3.16E-51	4.52E-49

**ZNF808**	-2.01	3.32E-51	4.73E-49
**CUEDC2**	1.89	4.88E-51	6.93E-49
**FAM50A**	2.09	5.65E-51	7.98E-49
**PIKFYVE**	-1.40	6.06E-51	8.51E-49
**BRWD1**	-1.90	7.43E-51	1.04E-48
**ENG**	4.05	8.58E-51	1.20E-48
**FBXW5**	1.48	8.97E-51	1.24E-48
**RAB7A**	1.57	1.03E-50	1.43E-48
**SCAND1**	2.38	1.04E-50	1.43E-48
**SDF4**	1.74	1.26E-50	1.72E-48
**RP11-498C9.15**	-3.08	1.36E-50	1.86E-48
**ZNF33A**	-1.44	1.38E-50	1.88E-48
**PFN1**	2.03	2.07E-50	2.80E-48
**ZNF573**	-2.58	2.59E-50	3.49E-48
**ATP5D**	1.93	2.87E-50	3.85E-48
**CDC25B**	3.07	3.00E-50	4.00E-48
**ZNF557**	-1.44	3.72E-50	4.95E-48
**ZNF506**	-1.69	5.44E-50	7.21E-48
**HIST1H2AI**	-8.75	6.61E-50	8.72E-48
**CTSB**	3.21	8.82E-50	1.16E-47
**NRSN2**	3.26	1.16E-49	1.52E-47
**ATL3**	-1.75	1.25E-49	1.63E-47
**AMY2B**	-2.58	1.27E-49	1.64E-47
**DYRK1A**	-1.07	1.82E-49	2.35E-47
**TERC**	-8.19	1.85E-49	2.38E-47
**SHISA5**	2.05	2.03E-49	2.61E-47
**TFE3**	1.26	2.46E-49	3.14E-47
**TCEA2**	2.59	2.75E-49	3.49E-47
**MDK**	4.87	2.86E-49	3.62E-47
**AKIRIN2**	1.60	4.38E-49	5.52E-47
**AP2M1**	1.65	4.76E-49	5.98E-47
**RN7SKP80**	-6.00	4.88E-49	6.10E-47
**WDFY3**	-1.53	6.75E-49	8.42E-47
**EPS15**	-1.31	7.33E-49	9.10E-47
**SF3B4**	1.57	7.48E-49	9.24E-47
**C10orf10**	5.02	8.57E-49	1.06E-46
**CCDC92**	3.00	9.12E-49	1.12E-46
**NOL9**	-1.04	1.02E-48	1.25E-46
**PRKCSH**	1.73	1.08E-48	1.32E-46
**MIR3609**	-10.35	1.32E-48	1.60E-46
**SOCS3**	5.19	1.40E-48	1.69E-46
**AHNAK**	-1.98	1.45E-48	1.75E-46
**DAPK3**	2.01	1.76E-48	2.11E-46
**ANAPC11**	2.10	2.16E-48	2.58E-46
**MDM4**	-2.09	2.18E-48	2.58E-46

**UTRN**	-2.03	2.18E-48	2.58E-46
**SFT2D2**	-2.01	2.26E-48	2.67E-46
**CLASP2**	-1.29	2.31E-48	2.72E-46
**SGTA**	1.49	2.83E-48	3.32E-46
**CCNT1**	-1.27	3.08E-48	3.59E-46
**WRN**	-1.52	4.03E-48	4.69E-46
**PIN1**	1.61	4.10E-48	4.75E-46
**TBL1XR1**	-1.95	4.11E-48	4.75E-46
**URM1**	1.81	4.87E-48	5.61E-46
**ARMC5**	1.95	5.01E-48	5.74E-46
**DMXL1**	-1.76	6.09E-48	6.96E-46
**FAM173A**	2.35	6.62E-48	7.54E-46
**UBE2M**	1.82	7.18E-48	8.14E-46
**PGF**	5.06	7.27E-48	8.23E-46
**CDON**	-2.64	1.06E-47	1.19E-45
**TMPRSS11F**	-3.54	1.18E-47	1.32E-45
**LGI4**	4.06	1.31E-47	1.47E-45
**STUB1**	1.89	1.34E-47	1.49E-45
**FKBP1A**	1.83	1.49E-47	1.65E-45
**CNN2**	1.93	1.97E-47	2.18E-45
**FUT10**	-1.65	3.44E-47	3.79E-45
**ZNF26**	2.61	3.80E-47	4.18E-45
**ARHGEF26**	-2.80	4.17E-47	4.57E-45
**SRM**	2.07	4.60E-47	5.03E-45
**INTU**	-2.14	5.53E-47	6.02E-45
**SERPINE1**	6.02	5.82E-47	6.31E-45
**EML4**	-1.28	6.07E-47	6.56E-45
**SMCHD1**	-1.48	6.90E-47	7.44E-45
**TNIP2**	1.99	9.04E-47	9.70E-45
**SPTAN1**	-1.40	9.13E-47	9.77E-45
**SHC1**	2.08	9.16E-47	9.77E-45
**C19orf68**	3.11	9.42E-47	1.00E-44
**GRN**	2.13	9.89E-47	1.05E-44
**MORF4L1P1**	2.47	1.03E-46	1.08E-44
**NUBP2**	1.72	1.05E-46	1.11E-44
**PPP4C**	1.69	1.22E-46	1.28E-44
**STX4**	1.82	1.51E-46	1.58E-44
**GADD45GIP1**	1.92	1.93E-46	2.01E-44
**PLEKHJ1**	1.66	2.04E-46	2.12E-44
**SPATA5**	-1.69	2.10E-46	2.18E-44
**DPF2**	1.30	2.15E-46	2.22E-44
**TMEM222**	1.68	2.79E-46	2.87E-44
**ENO1**	2.13	2.92E-46	2.99E-44
**SCARNA10**	-14.60	3.39E-46	3.47E-44
**SYNPO**	3.80	3.52E-46	3.58E-44

**NUDT16L1**	1.60	4.04E-46	4.10E-44
**NBAS**	-1.18	4.34E-46	4.39E-44
**ARHGEF15**	3.79	4.36E-46	4.40E-44
**TRIP6**	1.45	4.53E-46	4.55E-44
**PNMA1**	2.00	5.04E-46	5.05E-44
**MED29**	1.20	6.80E-46	6.79E-44
**CDKN2A**	4.47	6.89E-46	6.87E-44
**CCS**	1.62	7.74E-46	7.69E-44
**RORA**	-3.12	8.44E-46	8.35E-44
**ELK4**	-1.55	8.56E-46	8.44E-44
**ATXN3**	-1.72	9.03E-46	8.88E-44
**ZNF891**	-2.44	9.94E-46	9.75E-44
**LONP2**	-1.11	1.07E-45	1.04E-43
**RELL1**	-2.50	1.47E-45	1.44E-43
**ALDOA**	2.11	1.48E-45	1.44E-43
**CLEC14A**	4.34	1.85E-45	1.80E-43
**TROVE2**	-2.12	1.92E-45	1.86E-43
**NR2F2**	4.11	1.97E-45	1.90E-43
**PPP1R14A**	5.74	2.51E-45	2.41E-43
**SUPT5H**	1.09	2.68E-45	2.56E-43
**FAM3A**	1.57	2.90E-45	2.76E-43
**C3orf52**	-2.94	2.95E-45	2.81E-43
**MRPL41**	2.20	3.08E-45	2.93E-43
**MALAT1**	-4.18	3.39E-45	3.21E-43
**SHPRH**	-2.16	3.59E-45	3.38E-43
**MLF2**	1.84	4.13E-45	3.88E-43
**RBM42**	1.64	4.47E-45	4.18E-43
**SOX17**	4.46	4.47E-45	4.18E-43
**MBNL3**	-2.18	5.24E-45	4.89E-43
**HIST1H3B**	-10.58	5.51E-45	5.12E-43
**NUCB1**	1.92	7.84E-45	7.26E-43
**NKIRAS2**	1.35	8.16E-45	7.54E-43
**TARBP2**	1.41	9.89E-45	9.09E-43
**TNRC6B**	-1.82	9.89E-45	9.09E-43
**HERC1**	-1.54	1.09E-44	9.98E-43
**MOSPD3**	2.56	1.48E-44	1.35E-42
**FRYL**	-1.56	1.64E-44	1.49E-42
**BCAR1**	2.73	1.83E-44	1.66E-42
**PRKCB**	-2.76	1.91E-44	1.73E-42
**HIPK3**	-1.44	2.26E-44	2.04E-42
**BCL2**	-2.25	2.27E-44	2.04E-42
**TSC22D4**	1.79	2.38E-44	2.14E-42
**GNAI2**	2.71	2.46E-44	2.21E-42
**ZFAND3**	1.56	2.49E-44	2.23E-42
**ZNF736**	-2.04	2.76E-44	2.46E-42

**AGO1**	-1.19	2.81E-44	2.49E-42
**MAP3K11**	2.02	3.24E-44	2.87E-42
**PDLIM7**	3.67	3.30E-44	2.92E-42
**IRF4**	-2.91	3.36E-44	2.96E-42
**RP11-158K1.3**	-2.26	3.56E-44	3.12E-42
**GPR68**	3.27	3.56E-44	3.12E-42
**SETX**	-1.36	4.06E-44	3.54E-42
**SAT2**	1.83	4.12E-44	3.59E-42
**RGS3**	2.89	4.76E-44	4.13E-42
**ADCY2**	-2.56	5.37E-44	4.65E-42
**VPS13C**	-1.80	5.84E-44	5.05E-42
**MDN1**	-2.13	6.07E-44	5.23E-42
**PLVAP**	5.08	6.15E-44	5.28E-42
**HNRNPA0**	1.49	9.19E-44	7.87E-42
**ZNF106**	-1.23	1.14E-43	9.76E-42
**ZNF107**	-2.49	1.21E-43	1.03E-41
**GJA4**	5.17	1.25E-43	1.06E-41
**PRLR**	-3.78	1.29E-43	1.10E-41
**TAF15**	2.05	1.31E-43	1.11E-41
**MAPK3**	1.96	1.39E-43	1.17E-41
**ATR**	-1.55	1.45E-43	1.22E-41
**CERS6**	-2.38	1.74E-43	1.46E-41
**CYR61**	5.47	1.87E-43	1.56E-41
**KDELR1**	1.53	2.05E-43	1.71E-41
**PUS7L**	-1.79	2.34E-43	1.94E-41
**SRF**	1.35	2.67E-43	2.21E-41
**STK11**	1.41	2.74E-43	2.27E-41
**BRAF**	-1.36	2.78E-43	2.29E-41
**SHARPIN**	1.46	4.12E-43	3.39E-41
**SCARNA13**	-7.79	4.75E-43	3.90E-41
**TBCB**	1.94	5.06E-43	4.14E-41
**TGFB1**	3.56	5.36E-43	4.38E-41
**AGO2**	-1.69	5.87E-43	4.78E-41
**ZBTB25**	-1.56	6.49E-43	5.27E-41
**C1orf132**	-4.23	6.56E-43	5.32E-41
**RIC8A**	1.10	6.59E-43	5.33E-41
**ZNF503**	2.84	6.91E-43	5.57E-41
**ARHGAP12**	-1.38	7.23E-43	5.82E-41
**MAPK7**	1.76	7.67E-43	6.15E-41
**ASH1L**	-1.33	8.19E-43	6.56E-41
**RASGEF1B**	-1.72	9.55E-43	7.62E-41
**DAXX**	1.27	9.92E-43	7.90E-41
**COMMD4**	1.55	1.09E-42	8.70E-41
**FSCN1**	3.53	1.15E-42	9.13E-41
**MID1IP1**	1.44	1.19E-42	9.44E-41

**VPS13B**	-1.87	1.24E-42	9.77E-41
**CNTROB**	1.34	1.25E-42	9.81E-41
**CEBPB**	1.91	1.58E-42	1.24E-40
**TXN2**	1.75	1.70E-42	1.33E-40
**LENG1**	1.89	1.73E-42	1.35E-40
**LOXL2**	5.07	1.91E-42	1.48E-40
**CDK11B**	1.66	2.02E-42	1.57E-40
**SLC1A5**	3.27	2.21E-42	1.71E-40
**GPATCH2L**	-1.10	2.94E-42	2.27E-40
**USP3**	-1.31	3.28E-42	2.53E-40
**ZNF8**	-2.01	4.77E-42	3.67E-40
**TAB1**	1.42	5.26E-42	4.04E-40
**FAM126B**	-1.66	5.36E-42	4.10E-40
**PRKAR1B**	2.24	8.39E-42	6.41E-40
**CLDN5**	4.41	8.51E-42	6.49E-40
**BRD2**	1.14	9.21E-42	7.00E-40
**RPL41**	1.95	9.28E-42	7.04E-40
**FAM127A**	2.02	9.96E-42	7.54E-40
**RABAC1**	2.17	1.08E-41	8.17E-40
**ERBB2IP**	-1.87	1.09E-41	8.17E-40
**HIST1H2BL**	-7.21	1.15E-41	8.63E-40
**OPHN1**	-1.65	1.22E-41	9.14E-40
**SOX12**	2.49	1.31E-41	9.80E-40
**C7orf50**	2.07	1.38E-41	1.03E-39
**TGIF1**	1.32	1.66E-41	1.23E-39
**UBE2I**	1.12	1.73E-41	1.28E-39
**CARHSP1**	2.46	2.34E-41	1.73E-39
**DPM3**	2.69	2.52E-41	1.87E-39
**CHMP6**	1.60	2.59E-41	1.91E-39
**FKTN**	-1.32	2.64E-41	1.94E-39
**SOX18**	5.25	3.15E-41	2.31E-39
**ASAP2**	-1.64	3.27E-41	2.40E-39
**PKM**	2.23	3.44E-41	2.51E-39
**RP11-444D3.1**	-5.18	3.79E-41	2.76E-39
**ZNF543**	-1.45	3.84E-41	2.79E-39
**SLC35F5**	-2.03	4.26E-41	3.09E-39
**RNF187**	1.75	4.41E-41	3.19E-39
**SSNA1**	1.86	4.41E-41	3.19E-39
**ZNF525**	-2.15	4.45E-41	3.21E-39
**CLCN5**	-1.75	4.50E-41	3.24E-39
**MVP**	1.89	5.09E-41	3.65E-39
**LZTS2**	1.67	5.22E-41	3.74E-39
**GTF2F1**	1.27	5.52E-41	3.94E-39
**DDIT4**	4.40	5.81E-41	4.14E-39
**RP11-598P20.3**	-2.85	6.40E-41	4.55E-39

**GDI1**	1.54	6.94E-41	4.92E-39
**TPI1**	2.15	7.90E-41	5.59E-39
**GCSAM**	-3.22	9.35E-41	6.60E-39
**CITED4**	2.63	9.48E-41	6.68E-39
**RAB3B**	-5.22	1.01E-40	7.08E-39
**VCPIP1**	-1.67	1.07E-40	7.49E-39
**TTN**	-4.28	1.15E-40	8.03E-39
**HIST1H2BK**	-3.01	1.17E-40	8.20E-39
**GJB2**	4.61	1.23E-40	8.58E-39
**CD2BP2**	1.16	1.24E-40	8.61E-39
**SLC2A4RG**	1.81	1.25E-40	8.64E-39
**PRDX5**	2.40	1.27E-40	8.80E-39
**PRPF31**	1.45	1.52E-40	1.05E-38
**CDK9**	1.33	1.57E-40	1.08E-38
**COL18A1**	2.95	1.92E-40	1.32E-38
**SMAD2**	-1.36	2.12E-40	1.46E-38
**KCNE4**	4.36	2.33E-40	1.59E-38
**PRKCDBP**	3.38	2.98E-40	2.03E-38
**JMJD1C**	-1.27	2.98E-40	2.03E-38
**MXD4**	1.66	3.02E-40	2.05E-38
**TMUB1**	2.06	3.12E-40	2.12E-38
**IGFBP4**	4.64	3.33E-40	2.26E-38
**TWF2**	2.04	4.22E-40	2.85E-38
**B4GALT7**	1.56	7.50E-40	5.06E-38
**KIAA2022**	-2.57	7.91E-40	5.33E-38
**EPN1**	1.86	8.10E-40	5.44E-38
**CCDC137**	1.66	1.34E-39	8.96E-38
**NSRP1**	1.70	1.62E-39	1.09E-37
**COPS7A**	1.35	2.00E-39	1.33E-37
**KIAA1919**	-2.01	2.17E-39	1.45E-37
**RING1**	1.33	3.02E-39	2.01E-37
**PIM3**	1.91	3.04E-39	2.01E-37
**ZNF587B**	-1.71	3.21E-39	2.13E-37
**MACC1**	-2.60	3.26E-39	2.16E-37
**CCSAP**	-2.36	3.32E-39	2.19E-37
**ERCC6L2**	-1.63	3.47E-39	2.28E-37
**RP11-156E6.1**	-2.03	3.65E-39	2.40E-37
**RGS16**	5.31	3.91E-39	2.56E-37
**MAP3K2**	-1.57	4.15E-39	2.71E-37
**ZNF141**	-2.32	4.36E-39	2.85E-37
**ARL4C**	2.13	5.67E-39	3.69E-37
**SH2D3C**	3.92	5.69E-39	3.70E-37
**TEAD1**	-1.84	5.89E-39	3.82E-37
**GNAS**	1.41	5.91E-39	3.83E-37
**FPGS**	2.34	6.38E-39	4.12E-37

**CRTC2**	2.13	6.44E-39	4.15E-37
**DYNLT1**	1.58	6.61E-39	4.25E-37
**BCL2L1**	1.61	6.63E-39	4.26E-37
**RAMP3**	3.71	8.48E-39	5.44E-37
**AIP**	2.31	8.58E-39	5.49E-37
**BCL7B**	1.43	9.04E-39	5.77E-37
**CDK18**	3.50	9.75E-39	6.21E-37
**RLIM**	-1.63	1.07E-38	6.77E-37
**MED23**	-1.15	1.12E-38	7.09E-37
**MAPK11**	2.99	1.15E-38	7.28E-37
**HIST2H3D**	-6.26	1.29E-38	8.18E-37
**CCSER1**	-3.41	1.33E-38	8.39E-37
**ARID5A**	2.77	1.44E-38	9.08E-37
**IGF2**	6.15	1.58E-38	9.95E-37
**FAM234B**	-1.97	1.60E-38	1.00E-36
**TMEM9**	2.07	1.69E-38	1.06E-36
**MDFI**	4.12	1.89E-38	1.18E-36
**EML3**	1.65	1.99E-38	1.24E-36
**DOCK1**	-1.41	2.14E-38	1.33E-36
**CCDC51**	1.55	2.14E-38	1.33E-36
**RBL2**	-0.87	2.20E-38	1.36E-36
**ZNF428**	2.51	2.35E-38	1.45E-36
**PAK4**	2.00	2.61E-38	1.61E-36
**RBM12B**	-1.41	2.64E-38	1.63E-36
**LIMK1**	1.95	2.73E-38	1.68E-36
**PTPN14**	-1.96	2.82E-38	1.73E-36
**CCDC106**	1.89	3.31E-38	2.03E-36
**CARD19**	1.65	3.38E-38	2.06E-36
**NME4**	2.96	3.69E-38	2.25E-36
**RPS19BP1**	1.30	3.83E-38	2.33E-36
**4-Sep**	3.05	4.07E-38	2.48E-36
**POLA1**	-1.45	4.97E-38	3.01E-36
**GPR137**	1.89	5.41E-38	3.27E-36
**ZNHIT1**	1.61	5.96E-38	3.60E-36
**TMEM173**	3.74	6.12E-38	3.69E-36
**HS6ST1**	1.79	6.38E-38	3.84E-36
**CEP97**	-1.56	6.90E-38	4.15E-36
**FBXO46**	1.70	7.35E-38	4.41E-36
**FAM195B**	3.13	9.33E-38	5.58E-36
**CLPP**	1.76	9.34E-38	5.58E-36
**CANT1**	1.42	9.49E-38	5.66E-36
**LRBA**	-1.84	1.08E-37	6.42E-36
**SPHK1**	3.05	1.18E-37	7.00E-36
**PSMF1**	1.01	1.28E-37	7.58E-36
**PTPRN2**	3.46	1.42E-37	8.37E-36

**SEMA4B**	2.57	1.42E-37	8.41E-36
**TMEM204**	3.51	1.56E-37	9.22E-36
**ZMYM2**	-1.25	1.61E-37	9.46E-36
**SIPA1**	2.44	1.66E-37	9.72E-36
**FABP5**	4.05	1.71E-37	1.00E-35
**SMTN**	2.58	1.90E-37	1.11E-35
**KMT2C**	-1.80	1.94E-37	1.13E-35
**NXN**	2.10	2.24E-37	1.31E-35
**UBXN6**	1.55	2.25E-37	1.31E-35
**SMAD4**	-1.15	2.39E-37	1.39E-35
**CRYBG3**	-2.19	2.43E-37	1.41E-35
**RGMB**	-1.60	2.59E-37	1.50E-35
**NCR3LG1**	-3.30	2.65E-37	1.53E-35
**TSTA3**	1.76	2.79E-37	1.61E-35
**TCF3**	1.39	2.81E-37	1.62E-35
**SZRD1**	1.24	2.94E-37	1.69E-35
**HNRNPA2B1**	1.06	3.37E-37	1.93E-35
**ADRA2A**	5.29	3.41E-37	1.95E-35
**DLL4**	4.89	3.70E-37	2.11E-35
**PGAP1**	-1.95	3.76E-37	2.15E-35
**CNPPD1**	1.26	3.78E-37	2.15E-35
**LYPLA2**	1.60	3.86E-37	2.20E-35
**SHKBP1**	1.67	4.13E-37	2.34E-35
**GMPPA**	1.42	4.20E-37	2.38E-35
**SORBS3**	2.51	4.84E-37	2.74E-35
**PKN3**	2.17	5.01E-37	2.83E-35
**MAP2K2**	1.50	5.11E-37	2.88E-35
**UBE4A**	-1.29	5.17E-37	2.91E-35
**DDX41**	1.61	5.46E-37	3.07E-35
**SLC35A4**	1.66	5.81E-37	3.25E-35
**SNORD15B**	-8.28	6.08E-37	3.40E-35
**MAP3K10**	2.08	6.30E-37	3.52E-35
**ZNF724P**	-2.77	6.92E-37	3.86E-35
**ARHGEF17**	3.38	8.54E-37	4.75E-35
**ZNF91**	-2.55	8.54E-37	4.75E-35
**TACO1**	1.26	8.81E-37	4.89E-35
**RASA2**	-1.45	8.92E-37	4.94E-35
**SCARNA5**	-13.22	1.10E-36	6.09E-35
**METRN**	3.14	1.15E-36	6.37E-35
**SOCS4**	-1.66	1.37E-36	7.57E-35
**CHMP4B**	1.46	1.49E-36	8.21E-35
**PQLC1**	1.41	1.53E-36	8.40E-35
**PTPN13**	-1.94	1.70E-36	9.33E-35
**6-Mar**	-1.17	1.76E-36	9.61E-35
**RGS5**	3.39	1.77E-36	9.66E-35

**H1FX**	2.40	1.98E-36	1.08E-34
**NOL3**	1.61	2.15E-36	1.17E-34
**MFGE8**	2.66	2.23E-36	1.21E-34
**EXOC3L2**	4.51	2.54E-36	1.38E-34
**RBCK1**	1.17	2.68E-36	1.45E-34
**UPF3B**	2.05	3.24E-36	1.75E-34
**C5orf38**	2.68	3.35E-36	1.81E-34
**IFITM3**	4.29	3.73E-36	2.01E-34
**PTK7**	1.81	3.80E-36	2.05E-34
**SF3B2**	1.15	4.21E-36	2.26E-34
**ZNF644**	-1.54	4.45E-36	2.38E-34
**KIAA1328**	-1.44	4.47E-36	2.39E-34
**PXDN**	4.97	4.49E-36	2.40E-34
**EGFL7**	3.91	4.59E-36	2.45E-34
**MBOAT7**	1.38	5.14E-36	2.74E-34
**HRNR**	7.81	5.16E-36	2.74E-34
**PARK7**	1.90	5.36E-36	2.84E-34
**CEP295**	-1.32	5.93E-36	3.14E-34
**MRFAP1**	1.48	6.06E-36	3.20E-34
**XPO4**	-1.42	6.47E-36	3.41E-34
**NLGN4X**	4.69	6.60E-36	3.48E-34
**UGT3A2**	-7.89	6.76E-36	3.55E-34
**APLNR**	5.12	7.14E-36	3.75E-34
**H2AFJ**	2.03	7.57E-36	3.97E-34
**CHD9**	-1.40	7.75E-36	4.05E-34
**GPAA1**	1.35	8.37E-36	4.38E-34
**BCAM**	2.32	8.60E-36	4.49E-34
**CNP**	1.22	9.31E-36	4.85E-34
**ZNF408**	1.59	9.38E-36	4.88E-34
**TPBG**	2.27	9.40E-36	4.88E-34
**NPAT**	-1.39	1.11E-35	5.77E-34
**SCAF8**	-0.99	1.13E-35	5.84E-34
**VAMP5**	4.03	1.13E-35	5.86E-34
**MT-ND6**	5.86	1.21E-35	6.22E-34
**CHMP2A**	1.37	1.21E-35	6.22E-34
**PFKL**	1.43	1.29E-35	6.61E-34
**AGO3**	-1.54	1.38E-35	7.10E-34
**CTD-2270P14.1**	-3.30	1.43E-35	7.32E-34
**WBP2**	1.44	1.43E-35	7.32E-34
**AKAP11**	-1.36	1.56E-35	7.96E-34
**ZGRF1**	-1.98	1.77E-35	9.03E-34
**VMA21**	-1.79	2.02E-35	1.03E-33
**PHC2**	2.02	2.02E-35	1.03E-33
**SAFB2**	1.66	2.56E-35	1.30E-33
**KDM7A**	-1.57	2.56E-35	1.30E-33

**SUGP1**	1.03	2.68E-35	1.35E-33
**BTBD2**	1.28	2.71E-35	1.37E-33
**GTPBP2**	1.47	2.97E-35	1.50E-33
**LYST**	-1.79	2.97E-35	1.50E-33
**DMD**	-1.98	3.03E-35	1.52E-33
**DNAJC13**	-0.96	3.21E-35	1.61E-33
**ARRDC2**	2.11	3.22E-35	1.61E-33
**BCL3**	3.49	3.37E-35	1.69E-33
**ITPA**	1.54	3.42E-35	1.71E-33
**WHSC1L1**	-0.93	3.58E-35	1.79E-33
**SFN**	2.60	3.58E-35	1.79E-33
**APOBEC3C**	2.04	3.61E-35	1.79E-33
**MED16**	1.53	3.73E-35	1.85E-33
**UTP20**	-1.39	3.79E-35	1.88E-33
**TMPRSS11D**	6.27	3.90E-35	1.93E-33
**GPANK1**	1.45	4.06E-35	2.00E-33
**UCK1**	1.16	4.16E-35	2.05E-33
**ZBTB45**	1.72	4.46E-35	2.20E-33
**LL0XNC01-131B10.2**	-3.29	4.47E-35	2.20E-33
**TIE1**	3.93	4.59E-35	2.25E-33
**ACD**	1.99	5.25E-35	2.58E-33
**CSNK1G2**	1.29	5.52E-35	2.70E-33
**EPPK1**	-3.82	6.13E-35	3.00E-33
**AP3D1**	1.06	6.25E-35	3.05E-33
**BDP1**	-1.60	6.83E-35	3.33E-33
**CDH3**	2.72	7.48E-35	3.64E-33
**HELLS**	-1.98	8.15E-35	3.96E-33
**SNORA54**	-14.51	8.63E-35	4.19E-33
**SNTA1**	2.23	8.90E-35	4.31E-33
**ST14**	1.77	9.83E-35	4.75E-33
**ZNF510**	-1.19	1.00E-34	4.85E-33
**GPIHBP1**	4.33	1.08E-34	5.19E-33
**APC**	-1.54	1.21E-34	5.81E-33
**SH3BGRL3**	2.14	1.23E-34	5.89E-33
**C2CD2**	-2.03	1.25E-34	5.98E-33
**ZC3H12C**	-2.02	1.32E-34	6.35E-33
**HMGN5**	2.97	1.52E-34	7.26E-33
**LRP6**	-1.25	1.55E-34	7.40E-33
**NFKBIL1**	1.74	1.56E-34	7.44E-33
**PIH1D1**	1.55	1.58E-34	7.55E-33
**AP2S1**	2.03	1.59E-34	7.56E-33
**EYS**	-4.18	1.70E-34	8.08E-33
**C19orf43**	1.66	1.72E-34	8.16E-33
**USP33**	-1.00	1.76E-34	8.31E-33
**BAG3**	1.70	1.82E-34	8.62E-33

**ZNF490**	-2.19	1.97E-34	9.29E-33
**RP11-53O19.1**	-2.84	1.99E-34	9.37E-33
**USP9X**	-1.33	2.12E-34	9.98E-33
**BLOC1S4**	1.58	2.13E-34	1.00E-32
**SBF2**	-1.09	2.21E-34	1.04E-32
**TMEM214**	1.41	2.21E-34	1.04E-32
**GALK1**	2.35	2.26E-34	1.06E-32
**NLRX1**	2.08	2.31E-34	1.08E-32
**C19orf53**	1.68	2.47E-34	1.15E-32
**SLC35C1**	2.11	2.66E-34	1.24E-32
**PDGFB**	2.87	2.81E-34	1.31E-32
**HIST1H4D**	-12.74	2.88E-34	1.34E-32
**DAPP1**	-1.60	2.89E-34	1.34E-32
**VPS54**	-1.23	3.12E-34	1.44E-32
**MAK16**	-1.55	3.14E-34	1.45E-32
**RFNG**	1.59	3.19E-34	1.47E-32
**SNORA79**	-9.77	3.25E-34	1.50E-32
**ROBO4**	3.95	3.35E-34	1.54E-32
**AP000648.5**	-2.72	3.39E-34	1.56E-32
**SLC1A2**	-5.04	3.58E-34	1.64E-32
**ZNF529**	-1.36	3.74E-34	1.72E-32
**RPL26**	-2.66	3.77E-34	1.72E-32
**SART1**	1.50	3.77E-34	1.73E-32
**RNF19B**	1.27	3.78E-34	1.73E-32
**SNORA34**	-7.81	3.91E-34	1.78E-32
**TMEM120A**	1.58	4.26E-34	1.94E-32
**MFSD10**	1.94	4.48E-34	2.04E-32
**RABL6**	1.46	4.57E-34	2.08E-32
**CD151**	2.04	4.69E-34	2.13E-32
**RICTOR**	-1.72	4.73E-34	2.14E-32
**ZNF711**	-2.70	4.95E-34	2.24E-32
**ANKFN1**	-3.88	5.16E-34	2.33E-32
**CPNE2**	2.13	5.65E-34	2.55E-32
**GPATCH2**	-1.22	5.76E-34	2.59E-32
**GGT5**	5.60	6.02E-34	2.70E-32
**ATP6V0A2**	-1.37	6.19E-34	2.78E-32
**TMBIM1**	1.26	6.23E-34	2.79E-32
**C8orf82**	1.86	7.00E-34	3.13E-32
**ZNF84**	2.26	7.47E-34	3.34E-32
**PLEKHA4**	3.33	7.57E-34	3.38E-32
**BRCA2**	-2.00	7.68E-34	3.42E-32
**ZNF780A**	-1.88	7.82E-34	3.48E-32
**MED10**	1.63	7.86E-34	3.49E-32
**NCSTN**	0.94	8.04E-34	3.57E-32
**ARHGAP5**	-1.64	8.09E-34	3.58E-32

**FAM189B**	2.52	8.21E-34	3.63E-32
**NF1**	-1.32	8.43E-34	3.73E-32
**ATM**	-1.46	8.85E-34	3.91E-32
**ZNF765**	-1.54	9.23E-34	4.07E-32
**HIST1H2BI**	-9.10	1.02E-33	4.49E-32
**PRR14**	2.07	1.04E-33	4.57E-32
**MRPL28**	1.73	1.11E-33	4.86E-32
**SPG11**	-1.11	1.12E-33	4.91E-32
**TCP11L2**	-2.12	1.15E-33	5.01E-32
**MCAM**	4.86	1.20E-33	5.25E-32
**PTP4A3**	3.79	1.25E-33	5.46E-32
**P3H4**	2.24	1.30E-33	5.67E-32
**PTPN4**	-1.80	1.35E-33	5.87E-32
**ARAP3**	3.14	1.41E-33	6.11E-32
**PRKDC**	-1.42	1.46E-33	6.34E-32
**SF3A2**	2.15	1.51E-33	6.55E-32
**C7orf26**	1.42	1.55E-33	6.70E-32
**CHST1**	6.97	1.56E-33	6.76E-32
**REV3L**	-1.78	1.57E-33	6.79E-32
**P4HB**	1.30	1.59E-33	6.87E-32
**ARHGEF12**	-1.42	1.63E-33	7.01E-32
**ZNF138**	-1.66	1.79E-33	7.70E-32
**SNORA22**	-6.31	1.84E-33	7.92E-32
**MON2**	-1.41	1.93E-33	8.28E-32
**CTSD**	1.86	2.03E-33	8.71E-32
**MESDC1**	2.19	2.15E-33	9.18E-32
**ANGPTL5**	-5.62	2.16E-33	9.24E-32
**UAP1L1**	2.81	2.27E-33	9.67E-32
**ZZZ3**	-1.19	2.36E-33	1.01E-31
**STRN4**	1.41	2.43E-33	1.03E-31
**POLE4**	1.97	2.53E-33	1.08E-31
**PIK3C2A**	-1.23	2.56E-33	1.09E-31
**R3HDM4**	1.91	2.57E-33	1.09E-31
**ARPC4**	1.45	2.69E-33	1.14E-31
**NFKBIB**	1.51	2.72E-33	1.15E-31
**SYT8**	-3.25	2.86E-33	1.21E-31
**TRAF4**	1.96	2.87E-33	1.21E-31
**CSRP2**	3.14	2.90E-33	1.22E-31
**VPS9D1**	2.19	3.04E-33	1.28E-31
**SLC31A2**	-2.47	3.18E-33	1.33E-31
**CACFD1**	2.21	3.24E-33	1.36E-31
**SRC**	1.39	3.40E-33	1.43E-31
**DPM2**	1.64	3.46E-33	1.45E-31
**TADA3**	1.34	3.62E-33	1.51E-31
**PTPN21**	-1.98	3.99E-33	1.66E-31

**GPR108**	1.27	4.63E-33	1.93E-31
**TCF25**	1.12	4.70E-33	1.95E-31
**RP11-379F4.4**	-3.90	5.04E-33	2.09E-31
**SAT1**	2.49	5.16E-33	2.14E-31
**SCN4B**	2.81	5.62E-33	2.33E-31
**SIRT3**	1.39	5.70E-33	2.36E-31
**EPB41**	-1.64	5.74E-33	2.37E-31
**CAPNS1**	2.59	5.79E-33	2.39E-31
**USP31**	-1.74	5.90E-33	2.43E-31
**TIMM13**	1.82	6.00E-33	2.47E-31
**NXPH4**	3.25	6.02E-33	2.47E-31
**PES1**	1.44	6.30E-33	2.59E-31
**RASL11A**	2.53	6.68E-33	2.74E-31
**CHPF**	2.24	7.09E-33	2.90E-31
**HELZ**	-1.37	7.25E-33	2.97E-31
**LONP1**	1.38	7.27E-33	2.97E-31
**KRT14**	2.64	7.44E-33	3.03E-31
**SEMA6B**	5.58	8.49E-33	3.46E-31
**GIPC2**	-2.26	8.87E-33	3.61E-31
**RELA**	1.20	9.06E-33	3.68E-31
**CCDC7**	-2.52	9.64E-33	3.91E-31
**TMEM238**	2.71	9.70E-33	3.93E-31
**GRK5**	2.69	9.92E-33	4.01E-31
**ATP13A2**	1.43	1.06E-32	4.28E-31
**JAK2**	-1.40	1.19E-32	4.79E-31
**LDOC1**	2.24	1.24E-32	4.98E-31
**DUSP23**	1.83	1.28E-32	5.15E-31
**SLC16A7**	-2.64	1.29E-32	5.17E-31
**PDRG1**	1.75	1.29E-32	5.18E-31
**TRMT112**	1.73	1.30E-32	5.23E-31
**ASL**	1.63	1.31E-32	5.26E-31
**HAPLN3**	3.28	1.32E-32	5.28E-31
**PDAP1**	1.56	1.33E-32	5.32E-31
**SPRR1B**	5.26	1.34E-32	5.35E-31
**TMEM134**	1.85	1.38E-32	5.52E-31
**SKIDA1**	-2.35	1.42E-32	5.64E-31
**C19orf24**	2.06	1.43E-32	5.69E-31
**RRAS**	2.49	1.50E-32	5.96E-31
**TMEM33**	-1.49	1.50E-32	5.96E-31
**PPM1F**	1.67	1.58E-32	6.28E-31
**TAGLN**	4.12	1.60E-32	6.35E-31
**ZFX**	-1.22	1.64E-32	6.50E-31
**N4BP2**	-1.78	1.70E-32	6.73E-31
**PELP1**	1.48	1.72E-32	6.78E-31
**TAF10**	1.43	1.79E-32	7.06E-31

**ATP5SL**	1.31	1.80E-32	7.08E-31
**MLST8**	1.57	1.84E-32	7.21E-31
**GIPC1**	1.83	1.87E-32	7.33E-31
**NR1H2**	1.40	1.98E-32	7.75E-31
**ZMYM1**	-1.51	2.06E-32	8.07E-31
**ZNF282**	1.35	2.15E-32	8.41E-31
**BSG**	1.77	2.16E-32	8.42E-31
**ZBTB11**	-1.16	2.24E-32	8.72E-31
**ZNF398**	-1.02	2.35E-32	9.15E-31
**INTS2**	-1.16	2.55E-32	9.91E-31
**STARD10**	1.65	2.89E-32	1.12E-30
**TRPM7**	-1.25	2.95E-32	1.14E-30
**CCDC85B**	2.62	2.99E-32	1.16E-30
**HIST1H2BF**	-7.34	3.10E-32	1.20E-30
**CNDP2**	1.58	3.11E-32	1.20E-30
**AES**	1.28	3.19E-32	1.23E-30
**ZBTB3**	-1.53	3.30E-32	1.27E-30
**SLC38A10**	1.69	3.36E-32	1.29E-30
**PMPCA**	0.93	3.54E-32	1.36E-30
**CES2**	1.14	4.03E-32	1.55E-30
**UBR3**	-1.33	4.18E-32	1.60E-30
**NFATC2**	-1.54	4.21E-32	1.61E-30
**TPGS1**	2.05	4.23E-32	1.62E-30
**RLF**	-1.57	4.32E-32	1.65E-30
**RALB**	1.53	4.67E-32	1.78E-30
**GRASP**	2.84	4.70E-32	1.79E-30
**UBR2**	-1.13	5.18E-32	1.97E-30
**ATAD5**	-1.70	5.24E-32	2.00E-30
**ATP7A**	-2.11	5.25E-32	2.00E-30
**HIST1H4B**	-10.19	5.26E-32	2.00E-30
**CDK2AP1**	1.55	5.36E-32	2.03E-30
**MGLL**	1.90	5.46E-32	2.07E-30
**SPRY1**	1.89	5.57E-32	2.11E-30
**C17orf62**	2.00	5.93E-32	2.24E-30
**NMT1**	0.73	6.32E-32	2.38E-30
**CASP14**	3.14	6.70E-32	2.53E-30
**HYAL2**	1.98	7.01E-32	2.64E-30
**CSNK1D**	1.89	7.22E-32	2.71E-30
**NOSIP**	1.46	7.75E-32	2.91E-30
**KAT6A**	-1.43	7.93E-32	2.98E-30
**HRAS**	1.60	8.10E-32	3.04E-30
**KCNJ13**	-3.28	8.18E-32	3.06E-30
**SCARNA6**	-12.22	8.86E-32	3.31E-30
**PLEKHO1**	3.26	8.89E-32	3.32E-30
**RCAN3**	-1.51	9.10E-32	3.39E-30

**TMEM132A**	4.37	9.93E-32	3.70E-30
**ZNF319**	1.69	9.94E-32	3.70E-30
**EPHB4**	1.50	1.03E-31	3.82E-30
**ZNF652**	-1.97	1.04E-31	3.88E-30
**CCNJL**	3.58	1.06E-31	3.93E-30
**RAB11B**	2.77	1.09E-31	4.02E-30
**FAM83A**	3.57	1.10E-31	4.06E-30
**PPP1R9B**	1.59	1.14E-31	4.21E-30
**RP1-225E12.2**	-5.23	1.17E-31	4.30E-30
**MTPAP**	-1.42	1.17E-31	4.32E-30
**MAP7D1**	1.64	1.18E-31	4.35E-30
**DENND4A**	-1.16	1.19E-31	4.39E-30
**KRT9**	9.95	1.20E-31	4.39E-30
**TNFAIP1**	1.04	1.23E-31	4.51E-30
**SIGIRR**	2.62	1.23E-31	4.52E-30
**SIRT6**	1.99	1.28E-31	4.69E-30
**PLIN3**	1.88	1.28E-31	4.69E-30
**RP11-632K20.7**	-2.34	1.28E-31	4.69E-30
**ICE1**	-1.00	1.29E-31	4.70E-30
**SLC39A7**	2.02	1.31E-31	4.78E-30
**MMP15**	2.34	1.32E-31	4.81E-30
**ZNF431**	-1.62	1.33E-31	4.82E-30
**IL4R**	2.63	1.67E-31	6.05E-30
**RXRB**	1.42	1.72E-31	6.24E-30
**ATG2B**	-1.70	1.79E-31	6.49E-30
**TMED9**	1.71	1.85E-31	6.70E-30
**ALG10B**	-1.77	1.87E-31	6.76E-30
**STARD3**	1.50	2.10E-31	7.58E-30
**ARHGEF1**	1.53	2.41E-31	8.68E-30
**RABGAP1L**	-1.78	2.45E-31	8.81E-30
**PVRL2**	1.64	2.67E-31	9.59E-30
**PIK3CA**	-1.12	2.67E-31	9.59E-30
**MEGF11**	-4.26	2.80E-31	1.01E-29
**ZYX**	2.74	2.83E-31	1.02E-29
**ZNF615**	-1.49	2.87E-31	1.03E-29
**TRABD**	1.89	2.90E-31	1.04E-29
**LHPP**	2.00	2.93E-31	1.05E-29
**ADPRHL2**	1.25	2.94E-31	1.05E-29
**HEATR1**	-1.27	3.08E-31	1.10E-29
**RN7SL4P**	-12.07	3.18E-31	1.13E-29
**PPP1R26**	1.90	3.18E-31	1.13E-29
**YBX1**	1.54	3.20E-31	1.14E-29
**AFF4**	-1.10	3.22E-31	1.14E-29
**HDAC1**	1.18	3.27E-31	1.16E-29
**DGKZ**	1.49	3.48E-31	1.23E-29

**MOB1B**	-1.60	3.65E-31	1.29E-29
**ATAD2B**	-1.66	3.79E-31	1.34E-29
**RNY1**	-13.45	3.89E-31	1.37E-29
**MSN**	1.47	3.95E-31	1.39E-29
**MRPS30**	-1.33	4.03E-31	1.42E-29
**CASP9**	2.33	4.35E-31	1.53E-29
**ZNF780B**	-2.03	4.42E-31	1.55E-29
**MMP11**	7.01	4.48E-31	1.57E-29
**SOX5**	-3.38	4.54E-31	1.59E-29
**TRADD**	1.78	4.60E-31	1.61E-29
**FAM160A1**	-1.99	5.08E-31	1.78E-29
**ZNF619**	-1.67	5.15E-31	1.80E-29
**TET2**	-1.50	6.25E-31	2.18E-29
**RABGGTA**	1.50	6.52E-31	2.27E-29
**PHLDA3**	1.65	7.48E-31	2.61E-29
**MOB2**	1.24	7.81E-31	2.72E-29
**CD63**	2.89	8.16E-31	2.84E-29
**TMEM259**	1.93	8.88E-31	3.08E-29
**PCM1**	-1.16	8.90E-31	3.09E-29
**BCL2L12**	2.24	9.43E-31	3.27E-29
**NMRAL1**	1.16	9.62E-31	3.33E-29
**IER2**	2.58	9.69E-31	3.35E-29
**LIN7C**	-2.03	9.73E-31	3.36E-29
**HMBOX1**	-1.40	9.78E-31	3.37E-29
**DCP2**	-1.36	1.04E-30	3.58E-29
**CDKN1C**	3.27	1.09E-30	3.74E-29
**LINC01254**	-5.05	1.10E-30	3.77E-29
**ZCCHC11**	-1.27	1.13E-30	3.89E-29
**SNORA49**	-12.48	1.16E-30	3.97E-29
**PLOD1**	2.55	1.19E-30	4.07E-29
**TFPT**	2.38	1.25E-30	4.29E-29
**HOMER3**	2.50	1.28E-30	4.39E-29
**PLPPR2**	2.15	1.28E-30	4.39E-29
**TMEM132B**	-3.59	1.32E-30	4.50E-29
**PML**	1.87	1.37E-30	4.68E-29
**FTSJ3**	1.11	1.38E-30	4.69E-29
**UPP1**	3.32	1.54E-30	5.24E-29
**CDKAL1**	-1.32	1.54E-30	5.24E-29
**CEP44**	-1.51	1.62E-30	5.50E-29
**RN7SKP203**	-12.17	1.69E-30	5.73E-29
**CDH12**	-5.26	1.71E-30	5.78E-29
**RNASEH2C**	1.75	1.72E-30	5.80E-29
**HOOK3**	-1.44	1.89E-30	6.38E-29
**SNORA7B**	-7.08	1.97E-30	6.66E-29
**GNPTG**	1.33	2.02E-30	6.81E-29

**ST5**	2.21	2.07E-30	6.96E-29
**STXBP2**	1.91	2.09E-30	7.03E-29
**NAB1**	-1.84	2.10E-30	7.07E-29
**SNORD89**	-7.54	2.11E-30	7.08E-29
**TOPBP1**	-0.98	2.11E-30	7.09E-29
**C12orf10**	1.28	2.12E-30	7.11E-29
**CCND3**	1.35	2.27E-30	7.61E-29
**SRSF9**	1.33	2.29E-30	7.65E-29
**RP11-757F18.5**	-3.85	2.33E-30	7.79E-29
**MAPK1**	-1.24	2.42E-30	8.07E-29
**PAIP2B**	-2.23	2.48E-30	8.27E-29
**TTC21B**	-1.68	2.49E-30	8.30E-29
**CCDC97**	1.58	2.54E-30	8.46E-29
**CALM3**	1.50	2.56E-30	8.52E-29
**FHL3**	2.35	2.64E-30	8.77E-29
**SRSF10**	1.80	2.67E-30	8.86E-29
**C10orf99**	4.78	2.83E-30	9.38E-29
**MACF1**	-1.86	2.94E-30	9.73E-29
**CENPC**	-1.55	3.13E-30	1.04E-28
**TMEM39B**	1.77	3.25E-30	1.07E-28
**MPZL2**	2.47	3.30E-30	1.09E-28
**ZFYVE9**	-1.23	3.45E-30	1.14E-28
**MYO1C**	1.26	3.56E-30	1.17E-28
**ZC3H11A**	-0.83	3.59E-30	1.18E-28
**ACP5**	2.60	3.62E-30	1.19E-28
**MYL6B**	1.82	3.67E-30	1.20E-28
**NDUFA9**	-1.24	4.37E-30	1.43E-28
**SCAF1**	2.07	4.42E-30	1.45E-28
**WDR7**	-1.12	4.66E-30	1.52E-28
**HIST1H3F**	-9.88	4.79E-30	1.57E-28
**HEYL**	4.04	5.02E-30	1.64E-28
**GRAMD1A**	2.77	5.10E-30	1.66E-28
**PRKACA**	1.88	5.15E-30	1.68E-28
**CYGB**	3.76	5.30E-30	1.73E-28
**WDR1**	1.14	5.36E-30	1.74E-28
**ARFRP1**	1.72	5.40E-30	1.75E-28
**SCNM1**	1.20	5.52E-30	1.79E-28
**ISG20L2**	1.10	5.55E-30	1.80E-28
**MVB12A**	1.51	5.60E-30	1.81E-28
**EHBP1L1**	1.88	5.73E-30	1.85E-28
**USP24**	-1.27	5.94E-30	1.92E-28
**VAV2**	1.70	5.96E-30	1.92E-28
**SCYL1**	1.16	6.08E-30	1.96E-28
**PPDPF**	2.66	6.23E-30	2.01E-28
**ZNF347**	-1.25	6.36E-30	2.05E-28

**EGFLAM**	4.08	6.56E-30	2.11E-28
**RAB35**	1.04	6.94E-30	2.23E-28
**EHD2**	2.52	7.08E-30	2.27E-28
**NELFB**	1.34	7.14E-30	2.29E-28
**RPUSD1**	1.61	7.33E-30	2.35E-28
**ZNF704**	-1.55	7.37E-30	2.36E-28
**AURKAIP1**	1.66	7.62E-30	2.44E-28
**NUP62**	1.14	8.01E-30	2.56E-28
**UBALD1**	2.29	8.03E-30	2.56E-28
**RIN3**	2.47	8.11E-30	2.58E-28
**CIB1**	2.08	8.14E-30	2.59E-28
**SCEL**	-3.05	8.17E-30	2.60E-28
**RNPS1**	0.95	8.62E-30	2.74E-28
**PRMT1**	1.57	8.65E-30	2.75E-28
**KXD1**	1.19	9.40E-30	2.98E-28
**MYPOP**	1.93	9.45E-30	2.99E-28
**FTH1**	3.82	9.54E-30	3.02E-28
**SPTBN1**	-1.39	1.03E-29	3.24E-28
**ZNF503-AS2**	2.32	1.10E-29	3.48E-28
**SWT1**	-1.20	1.27E-29	4.00E-28
**MT1M**	3.27	1.29E-29	4.05E-28
**NDUFB8**	-1.79	1.34E-29	4.24E-28
**LCORL**	-1.65	1.36E-29	4.28E-28
**DDRGK1**	1.27	1.42E-29	4.47E-28
**DAZAP1**	1.23	1.45E-29	4.55E-28
**SAFB**	1.18	1.45E-29	4.55E-28
**C5orf42**	-1.49	1.63E-29	5.10E-28
**ADAM12**	5.01	1.74E-29	5.44E-28
**RIC1**	-0.90	1.78E-29	5.57E-28
**RHOT1**	-1.62	1.90E-29	5.95E-28
**TIMM44**	1.07	1.93E-29	6.02E-28
**RARRES2**	6.60	1.94E-29	6.04E-28
**RASSF9**	-2.04	1.95E-29	6.09E-28
**ARFGEF1**	-1.18	2.42E-29	7.54E-28
**B4GALT1**	1.96	2.49E-29	7.74E-28
**PPIP5K2**	-1.15	2.69E-29	8.37E-28
**FURIN**	1.61	2.76E-29	8.57E-28
**FANCM**	-1.51	2.79E-29	8.65E-28
**DPP7**	1.70	2.79E-29	8.65E-28
**USB1**	2.69	2.92E-29	9.05E-28
**TRIP10**	2.01	3.07E-29	9.50E-28
**ZNF184**	-1.48	3.10E-29	9.56E-28
**VPS13A**	-2.04	3.23E-29	9.96E-28
**ILF3-AS1**	1.68	3.39E-29	1.04E-27
**SPAG9**	-0.88	3.46E-29	1.07E-27

**RIN2**	1.38	3.54E-29	1.09E-27
**FBL**	1.74	3.78E-29	1.16E-27
**NEXN**	2.65	3.83E-29	1.18E-27
**GMDS**	2.23	3.90E-29	1.20E-27
**EIF5B**	1.49	3.94E-29	1.21E-27
**NCAPD3**	-1.31	4.02E-29	1.23E-27
**RAB24**	2.29	4.07E-29	1.24E-27
**AP5M1**	-1.24	4.21E-29	1.29E-27
**TRAPPC2L**	1.30	4.25E-29	1.30E-27
**RORA-AS1**	-4.77	4.28E-29	1.31E-27
**DYSF**	4.04	4.29E-29	1.31E-27
**C19orf60**	1.44	4.32E-29	1.31E-27
**MIS18BP1**	-1.39	4.37E-29	1.33E-27
**GNB2**	1.84	4.46E-29	1.35E-27
**CST3**	2.86	4.54E-29	1.38E-27
**CXXC1**	1.39	4.58E-29	1.39E-27
**PLEKHA5**	-1.19	5.02E-29	1.52E-27
**ADCK4**	1.32	5.04E-29	1.52E-27
**SNORD94**	-6.19	5.16E-29	1.56E-27
**CHCHD5**	2.12	5.40E-29	1.63E-27
**GAPDH**	2.05	5.83E-29	1.76E-27
**TGM2**	5.71	5.85E-29	1.76E-27
**QDPR**	1.66	5.97E-29	1.80E-27
**FAM83F**	-1.80	6.06E-29	1.83E-27
**C12orf75**	2.05	6.09E-29	1.83E-27
**DICER1**	-1.21	6.38E-29	1.92E-27
**SAP30BP**	0.95	6.45E-29	1.93E-27
**HPCAL1**	1.91	6.67E-29	2.00E-27
**UQCRC1**	1.31	6.76E-29	2.02E-27
**KIF27**	-1.76	7.44E-29	2.23E-27
**OCEL1**	2.20	7.73E-29	2.31E-27
**NCOA2**	-1.33	8.08E-29	2.41E-27
**CCDC102A**	1.84	8.40E-29	2.51E-27
**SPRY4**	4.55	8.47E-29	2.52E-27
**AFF1**	-1.34	8.59E-29	2.56E-27
**RHBDD3**	1.93	8.63E-29	2.57E-27
**PIK3C2G**	-2.11	8.81E-29	2.62E-27
**DHRS7**	1.64	9.04E-29	2.69E-27
**CRABP2**	2.99	1.00E-28	2.97E-27
**ZNF688**	1.79	1.02E-28	3.03E-27
**COTL1**	3.09	1.02E-28	3.03E-27
**FGD4**	-1.66	1.03E-28	3.06E-27
**PODNL1**	2.93	1.04E-28	3.07E-27
**SOX4**	1.99	1.11E-28	3.28E-27
**KLF2**	3.30	1.15E-28	3.39E-27

**AKR1B10**	5.33	1.19E-28	3.50E-27
**NAT14**	2.70	1.19E-28	3.50E-27
**MAP2K7**	1.37	1.21E-28	3.56E-27
**ST6GALNAC6**	1.44	1.25E-28	3.67E-27
**RAB1B**	1.40	1.27E-28	3.72E-27
**HMG20B**	1.50	1.32E-28	3.86E-27
**SERPINB6**	1.85	1.32E-28	3.86E-27
**G29816**	-5.58	1.34E-28	3.91E-27
**OGFR**	1.25	1.37E-28	4.01E-27
**CTC-559E9.5**	-2.35	1.40E-28	4.08E-27
**LCMT1-AS2**	-4.13	1.45E-28	4.25E-27
**GYS1**	1.47	1.56E-28	4.55E-27
**PCGF2**	3.36	1.57E-28	4.59E-27
**SOX7**	2.48	1.59E-28	4.64E-27
**ZNF467**	1.86	1.74E-28	5.07E-27
**JUNB**	2.76	1.89E-28	5.50E-27
**NSMF**	2.09	2.02E-28	5.88E-27
**NUBP1**	1.49	2.10E-28	6.08E-27
**MYL9**	3.85	2.24E-28	6.48E-27
**MPP5**	-1.17	2.29E-28	6.64E-27
**ARFGEF2**	-1.23	2.47E-28	7.15E-27
**RAB11FIP3**	1.54	2.52E-28	7.29E-27
**UPF3A**	1.34	2.57E-28	7.43E-27
**CALD1**	2.07	2.64E-28	7.62E-27
**STK32C**	2.28	2.73E-28	7.87E-27
**TMEM129**	1.24	2.79E-28	8.02E-27
**MMP14**	2.73	2.81E-28	8.08E-27
**XPNPEP3**	-1.41	3.05E-28	8.78E-27
**C12orf43**	1.20	3.08E-28	8.86E-27
**OGFOD3**	1.44	3.09E-28	8.87E-27
**USP45**	-1.60	3.26E-28	9.35E-27
**TIMM22**	1.07	3.30E-28	9.46E-27
**PAMR1**	-2.97	3.32E-28	9.51E-27
**PRPF38B**	1.51	3.34E-28	9.55E-27
**ADGRG6**	-1.65	3.39E-28	9.70E-27
**QSOX1**	1.81	3.53E-28	1.01E-26
**ATF6B**	1.36	3.80E-28	1.08E-26
**FABP5P7**	4.46	3.85E-28	1.10E-26
**ZNF623**	-1.03	4.43E-28	1.26E-26
**AGRN**	1.73	4.59E-28	1.31E-26
**SCARF2**	4.43	4.59E-28	1.31E-26
**ATP1A1**	1.86	4.77E-28	1.36E-26
**C11orf84**	2.40	4.80E-28	1.36E-26
**UBA1**	0.90	5.01E-28	1.42E-26
**CA13**	-2.00	5.08E-28	1.44E-26

**MYDGF**	1.92	5.11E-28	1.45E-26
**EIF4EBP1**	2.78	5.88E-28	1.66E-26
**CHRM4**	-4.10	5.90E-28	1.67E-26
**SYDE2**	-2.60	6.17E-28	1.74E-26
**CCDC61**	1.92	6.18E-28	1.74E-26
**NCKIPSD**	1.38	6.22E-28	1.75E-26
**NMB**	3.14	6.28E-28	1.77E-26
**CTGF**	4.23	6.43E-28	1.81E-26
**CRELD2**	1.72	6.45E-28	1.81E-26
**ZNF484**	-1.64	6.59E-28	1.85E-26
**SLC35F6**	-1.41	6.63E-28	1.86E-26
**HBB**	-5.98	6.70E-28	1.88E-26
**GNL1**	1.04	6.95E-28	1.95E-26
**GJB6**	3.62	7.00E-28	1.96E-26
**EIF1**	1.32	7.22E-28	2.02E-26
**ISYNA1**	2.88	7.44E-28	2.08E-26
**CLTA**	1.20	7.55E-28	2.11E-26
**ADAMTSL3**	-2.99	7.63E-28	2.13E-26
**HSPBP1**	1.76	7.65E-28	2.13E-26
**RCE1**	1.49	7.75E-28	2.16E-26
**EFHD2**	1.86	7.82E-28	2.18E-26
**MCOLN1**	1.69	8.10E-28	2.25E-26
**KLHDC3**	1.21	8.20E-28	2.28E-26
**MRPL2**	1.36	8.26E-28	2.29E-26
**NUPR1**	1.63	8.49E-28	2.35E-26
**TRAF6**	-1.17	8.53E-28	2.36E-26
**ATXN7L3**	1.14	8.60E-28	2.38E-26
**ZNF732**	-3.18	8.66E-28	2.39E-26
**ZNF700**	-1.18	9.03E-28	2.49E-26
**CDH5**	3.71	9.18E-28	2.53E-26
**RFXANK**	1.26	9.29E-28	2.56E-26
**CNPY3**	1.70	9.44E-28	2.60E-26
**KLHL28**	-1.47	9.54E-28	2.62E-26
**ARIH1**	-1.26	9.58E-28	2.63E-26
**MED13L**	-1.13	9.69E-28	2.66E-26
**SMAD7**	1.69	1.03E-27	2.82E-26
**LPCAT1**	2.31	1.04E-27	2.84E-26
**ZNF17**	-1.28	1.07E-27	2.92E-26
**CAMK2G**	0.91	1.10E-27	3.01E-26
**UBXN1**	1.49	1.11E-27	3.05E-26
**IFITM2**	4.32	1.13E-27	3.09E-26
**TESK1**	1.55	1.15E-27	3.14E-26
**FAM208B**	-1.32	1.16E-27	3.17E-26
**FZR1**	1.25	1.19E-27	3.23E-26
**OIP5-AS1**	-1.50	1.19E-27	3.23E-26

**NCLN**	1.60	1.20E-27	3.26E-26
**NCK2**	1.32	1.20E-27	3.27E-26
**NLGN2**	3.31	1.22E-27	3.32E-26
**BCAP31**	1.49	1.25E-27	3.39E-26
**CHID1**	1.24	1.26E-27	3.42E-26
**CYBA**	3.54	1.27E-27	3.43E-26
**PREB**	1.19	1.28E-27	3.47E-26
**SYDE1**	3.21	1.29E-27	3.48E-26
**PANK3**	-1.80	1.30E-27	3.52E-26
**CEMIP**	4.84	1.34E-27	3.63E-26
**YDJC**	1.51	1.36E-27	3.67E-26
**TMEM237**	-1.34	1.38E-27	3.73E-26
**ZNF598**	1.73	1.40E-27	3.77E-26
**CCDC71**	1.25	1.44E-27	3.88E-26
**N4BP2L2-IT2**	-3.80	1.46E-27	3.93E-26
**HIST1H3E**	-3.40	1.49E-27	3.99E-26
**MYL6**	1.43	1.49E-27	3.99E-26
**PEAR1**	3.90	1.56E-27	4.19E-26
**ADGRF5**	3.58	1.57E-27	4.20E-26
**FMN1**	-1.85	1.69E-27	4.52E-26
**EPAS1**	1.75	1.75E-27	4.69E-26
**DNAH11**	-5.01	1.98E-27	5.29E-26
**RP11-15H20.6**	-3.40	2.01E-27	5.37E-26
**CLEC11A**	3.24	2.03E-27	5.43E-26
**SEC14L1**	1.41	2.15E-27	5.72E-26
**TMC6**	2.36	2.36E-27	6.27E-26
**GPR4**	6.31	2.46E-27	6.55E-26
**LTBR**	1.25	2.52E-27	6.71E-26
**GRIN3A**	-2.72	2.56E-27	6.81E-26
**EMILIN1**	4.80	2.57E-27	6.81E-26
**WBSCR16**	1.21	2.73E-27	7.23E-26
**ACVR2A**	-1.53	2.76E-27	7.32E-26
**CHTOP**	0.84	2.81E-27	7.43E-26
**GXYLT1**	-1.20	2.93E-27	7.75E-26
**PACS1**	2.21	2.93E-27	7.75E-26
**CRYM-AS1**	-3.21	2.94E-27	7.78E-26
**KCNJ15**	2.83	3.13E-27	8.26E-26
**AKAP5**	-3.34	3.19E-27	8.41E-26
**PKHD1L1**	-3.92	3.23E-27	8.50E-26
**HIST1H2AH**	-11.33	3.34E-27	8.79E-26
**FAM219A**	1.37	4.12E-27	1.08E-25
**FMO3**	6.91	4.26E-27	1.12E-25
**CCNT2**	-1.10	4.28E-27	1.13E-25
**HIST2H2AB**	-8.07	4.45E-27	1.17E-25
**PSD3**	-1.63	4.61E-27	1.21E-25

**CUL7**	1.13	4.75E-27	1.24E-25
**STMN2**	7.41	4.79E-27	1.25E-25
**NIPBL**	-1.25	4.79E-27	1.25E-25
**CPXM1**	7.91	4.86E-27	1.27E-25
**NDUFB10**	1.20	4.86E-27	1.27E-25
**FBXL4**	-1.46	4.88E-27	1.27E-25
**GTF3C5**	0.89	4.96E-27	1.29E-25
**G32779**	3.83	5.06E-27	1.32E-25
**MIR4435-2HG**	4.32	5.27E-27	1.37E-25
**LAMB4**	-2.52	5.42E-27	1.41E-25
**GAR1**	1.52	5.64E-27	1.47E-25
**REPIN1**	1.27	6.03E-27	1.57E-25
**LRIG2**	-1.40	6.10E-27	1.58E-25
**MPV17L2**	1.55	6.21E-27	1.61E-25
**KIAA0586**	-1.02	6.31E-27	1.63E-25
**CTD-2328D6.1**	9.46	6.50E-27	1.68E-25
**RAMP2**	3.18	6.55E-27	1.69E-25
**CRYL1**	2.14	6.64E-27	1.71E-25
**AGTPBP1**	-1.18	7.12E-27	1.84E-25
**LARP4**	-1.34	7.29E-27	1.88E-25
**GNA12**	1.91	7.44E-27	1.92E-25
**ZNF772**	-1.89	7.54E-27	1.94E-25
**GPATCH3**	1.16	7.64E-27	1.96E-25
**RP11-437B10.1**	-1.36	7.95E-27	2.04E-25
**BGN**	4.22	8.28E-27	2.13E-25
**TBKBP1**	2.50	8.28E-27	2.13E-25
**YIF1A**	1.57	8.32E-27	2.13E-25
**SBNO1**	-1.08	8.38E-27	2.15E-25
**APOL1**	3.96	8.45E-27	2.16E-25
**TP53INP2**	2.62	8.46E-27	2.16E-25
**ZBTB21**	-1.78	8.52E-27	2.18E-25
**ERF**	1.74	8.61E-27	2.20E-25
**NAA25**	-1.20	8.67E-27	2.21E-25
**EDF1**	1.27	8.91E-27	2.27E-25
**RPL8**	1.75	9.03E-27	2.30E-25
**ZNF737**	-2.09	9.63E-27	2.45E-25
**TMEM55B**	1.15	9.92E-27	2.52E-25
**EGFR-AS1**	-5.34	1.03E-26	2.63E-25
**FAM208A**	-1.11	1.08E-26	2.74E-25
**PPP2R4**	1.33	1.18E-26	2.99E-25
**ID1**	2.45	1.20E-26	3.05E-25
**VEGFB**	1.56	1.20E-26	3.05E-25
**PRKRIP1**	1.12	1.35E-26	3.42E-25
**ADAMTSL2**	4.92	1.35E-26	3.43E-25
**GRINA**	1.85	1.39E-26	3.51E-25

**ITGA7**	3.04	1.39E-26	3.51E-25
**POLR2H**	1.64	1.41E-26	3.57E-25
**PLXDC2**	-1.50	1.44E-26	3.63E-25
**GMFG**	3.78	1.50E-26	3.79E-25
**SUGT1**	-1.22	1.60E-26	4.03E-25
**A2M**	3.92	1.64E-26	4.14E-25
**DYNC1H1**	-1.46	1.65E-26	4.14E-25
**CYB5R3**	1.67	1.66E-26	4.18E-25
**ZNF580**	1.65	1.67E-26	4.19E-25
**ZBTB34**	-1.52	1.71E-26	4.30E-25
**BAG6**	1.13	1.74E-26	4.36E-25
**CREB1**	-1.26	1.80E-26	4.50E-25
**HIST1H3I**	-11.16	1.85E-26	4.62E-25
**DNPH1**	1.82	1.85E-26	4.62E-25
**SCRN2**	1.63	1.91E-26	4.78E-25
**APLN**	5.49	1.95E-26	4.88E-25
**ZFAND4**	-1.25	1.99E-26	4.95E-25
**ZBTB33**	-1.58	2.03E-26	5.05E-25
**TOM1**	1.31	2.03E-26	5.05E-25
**RAPH1**	-2.01	2.08E-26	5.17E-25
**KRI1**	1.50	2.33E-26	5.80E-25
**TMEM160**	2.15	2.47E-26	6.13E-25
**FAM179B**	-1.06	2.48E-26	6.15E-25
**SEPSECS**	-1.07	2.50E-26	6.21E-25
**H1F0**	1.63	2.51E-26	6.23E-25
**MANF**	1.41	2.61E-26	6.47E-25
**MZT2A**	1.84	2.73E-26	6.76E-25
**NME3**	1.81	2.82E-26	6.97E-25
**PABPC1**	1.45	2.84E-26	7.03E-25
**EMD**	1.27	3.02E-26	7.44E-25
**TP53I3**	2.25	3.03E-26	7.48E-25
**ITPKC**	1.26	3.09E-26	7.61E-25
**HES5**	-3.84	3.10E-26	7.65E-25
**PPP1R12C**	1.63	3.16E-26	7.77E-25
**PTX3**	6.82	3.25E-26	7.99E-25
**MAGED1**	1.74	3.28E-26	8.06E-25
**SLC7A8**	2.44	3.49E-26	8.56E-25
**TIMP1**	4.73	3.69E-26	9.06E-25
**OCIAD2**	1.69	3.86E-26	9.47E-25
**RHOT2**	2.05	3.97E-26	9.72E-25
**SORCS1**	-2.65	4.10E-26	1.00E-24
**PGBD5**	4.04	4.18E-26	1.02E-24
**RPS28**	1.74	4.25E-26	1.04E-24
**KLHL15**	-1.38	4.26E-26	1.04E-24
**SEZ6L2**	3.43	4.50E-26	1.10E-24

**ARRB2**	2.42	4.57E-26	1.11E-24
**GIMAP7**	3.22	4.71E-26	1.15E-24
**MRPL55**	1.08	4.74E-26	1.15E-24
**PSMD6-AS2**	-3.76	4.99E-26	1.21E-24
**OTULIN**	-1.18	5.06E-26	1.23E-24
**DEAF1**	1.32	5.23E-26	1.27E-24
**EFHC2**	-2.17	5.26E-26	1.28E-24
**KCNIP4**	-2.26	5.29E-26	1.28E-24
**RFX3**	-1.85	5.41E-26	1.31E-24
**ECE1**	1.82	5.66E-26	1.37E-24
**MRPS34**	1.29	5.82E-26	1.41E-24
**GPLD1**	-2.22	5.84E-26	1.41E-24
**TICAM1**	2.34	5.94E-26	1.44E-24
**RNH1**	1.25	6.06E-26	1.46E-24
**ZYG11B**	-1.37	6.07E-26	1.46E-24
**RAB5C**	1.33	6.83E-26	1.65E-24
**POLR2J**	1.28	6.89E-26	1.66E-24
**TEAD2**	3.06	7.25E-26	1.74E-24
**TBC1D13**	1.21	7.41E-26	1.78E-24
**FASTK**	1.33	7.53E-26	1.81E-24
**RASIP1**	2.43	8.12E-26	1.95E-24
**KHSRP**	1.34	8.25E-26	1.98E-24
**SLC18A2**	-2.73	8.63E-26	2.07E-24
**PROB1**	2.85	8.66E-26	2.08E-24
**HIST1H3J**	-7.94	8.79E-26	2.10E-24
**DCTN3**	1.25	8.88E-26	2.12E-24
**SEPN1**	2.21	9.12E-26	2.18E-24
**GJA5**	5.92	9.22E-26	2.20E-24
**TPM4**	1.55	9.25E-26	2.21E-24
**ZNF37A**	-1.14	9.32E-26	2.22E-24
**DMPK**	2.46	9.36E-26	2.23E-24
**MTMR14**	0.84	9.39E-26	2.24E-24
**FAM20C**	2.60	9.59E-26	2.28E-24
**ASB13**	1.46	9.85E-26	2.34E-24
**HAUS3**	-0.94	9.85E-26	2.34E-24
**RP11-121C2.2**	-2.04	9.86E-26	2.34E-24
**PGAM1**	1.71	1.01E-25	2.39E-24
**MINK1**	1.43	1.02E-25	2.41E-24
**ANKRD33B**	-3.39	1.03E-25	2.44E-24
**LMBRD2**	-1.57	1.04E-25	2.47E-24
**GOLGA3**	-0.89	1.05E-25	2.48E-24
**PLEKHA1**	-1.56	1.09E-25	2.58E-24
**SLC27A3**	1.53	1.10E-25	2.60E-24
**SUMF2**	1.01	1.11E-25	2.61E-24
**AGPAT2**	1.73	1.14E-25	2.69E-24

**THADA**	-1.18	1.15E-25	2.71E-24
**TMEM116**	-1.78	1.19E-25	2.79E-24
**ZNF444**	2.12	1.28E-25	3.00E-24
**ANKRD11**	1.23	1.29E-25	3.05E-24
**DDA1**	1.35	1.31E-25	3.08E-24
**FGD3**	2.55	1.36E-25	3.19E-24
**S100A11**	1.80	1.38E-25	3.23E-24
**IGFBP7**	3.29	1.39E-25	3.27E-24
**VMP1**	1.05	1.42E-25	3.34E-24
**HTRA2**	1.45	1.43E-25	3.36E-24
**PLEKHG2**	3.46	1.46E-25	3.42E-24
**9-Mar**	1.72	1.54E-25	3.60E-24
**TRDMT1**	-1.36	1.55E-25	3.62E-24
**BRI3**	1.65	1.59E-25	3.71E-24
**RP11-517B11.7**	-1.81	1.64E-25	3.82E-24
**LASP1**	1.43	1.79E-25	4.17E-24
**PHF1**	2.11	1.85E-25	4.31E-24
**AKIP1**	1.84	1.87E-25	4.35E-24
**G2E3**	-1.44	2.05E-25	4.76E-24
**NFIB**	-1.29	2.10E-25	4.87E-24
**CASP8AP2**	-1.11	2.10E-25	4.89E-24
**YIPF3**	1.11	2.14E-25	4.96E-24
**PDE6D**	1.01	2.20E-25	5.09E-24
**GOLGB1**	-1.18	2.33E-25	5.39E-24
**MATN4**	-5.24	2.35E-25	5.44E-24
**FXYD5**	2.72	2.57E-25	5.94E-24
**VPS13D**	-1.50	2.63E-25	6.08E-24
**HOXD9**	3.44	2.73E-25	6.32E-24
**ZBTB17**	1.37	2.85E-25	6.59E-24
**SUMO3**	1.46	2.88E-25	6.64E-24
**ATP13A3**	-1.33	2.90E-25	6.70E-24
**CFL1**	1.58	2.95E-25	6.80E-24
**AOC3**	3.61	3.08E-25	7.10E-24
**SLC12A4**	1.73	3.21E-25	7.38E-24
**PAK3**	-2.46	3.21E-25	7.39E-24
**PRPSAP1**	1.03	3.23E-25	7.43E-24
**IRF2BP1**	1.16	3.58E-25	8.23E-24
**TP53I13**	1.77	3.73E-25	8.56E-24
**ANGPT2**	3.71	3.78E-25	8.67E-24
**RHOJ**	3.12	3.89E-25	8.90E-24
**LEMD2**	1.02	3.95E-25	9.05E-24
**VAMP2**	2.06	3.98E-25	9.12E-24
**BRE**	1.05	4.24E-25	9.69E-24
**SOX6**	-2.11	4.26E-25	9.74E-24
**CLEC2A**	-2.50	4.28E-25	9.77E-24

**SPRYD3**	1.21	4.41E-25	1.01E-23
**DAB2IP**	1.48	4.46E-25	1.02E-23
**LRRC20**	3.28	4.51E-25	1.03E-23
**UBXN7**	-1.11	4.51E-25	1.03E-23
**ZNF384**	0.99	4.56E-25	1.04E-23
**TNFRSF21**	3.40	4.57E-25	1.04E-23
**PPP1R15A**	1.35	4.66E-25	1.06E-23
**RNU5A-1**	-11.22	4.66E-25	1.06E-23
**TMPRSS4**	5.46	5.05E-25	1.15E-23
**SLC9C1**	-4.94	5.16E-25	1.17E-23
**KCNK3**	4.79	5.27E-25	1.19E-23
**NOTCH4**	3.27	5.29E-25	1.20E-23
**ARAF**	1.02	5.37E-25	1.21E-23
**NEBL**	-1.94	5.43E-25	1.23E-23
**BFAR**	1.12	5.47E-25	1.24E-23
**PRELID1**	1.84	5.53E-25	1.25E-23
**SMAD5**	-1.35	5.56E-25	1.25E-23
**STXBP5**	-1.59	5.57E-25	1.25E-23
**NDUFB7**	1.50	5.64E-25	1.27E-23
**LAMTOR2**	1.37	5.90E-25	1.33E-23
**TTC37**	-1.43	6.46E-25	1.45E-23
**NID2**	3.98	6.73E-25	1.51E-23
**BTBD6**	0.91	7.09E-25	1.59E-23
**ARFGAP1**	1.64	7.28E-25	1.63E-23
**CYC1**	1.29	7.36E-25	1.65E-23
**FAM63B**	-1.43	7.50E-25	1.68E-23
**CDC42EP1**	1.97	7.51E-25	1.68E-23
**ECSIT**	1.29	7.62E-25	1.70E-23
**MTR**	-1.31	7.92E-25	1.77E-23
**IL32**	4.26	7.97E-25	1.78E-23
**MGA**	-1.74	8.20E-25	1.83E-23
**UBAP1**	0.97	8.46E-25	1.89E-23
**MSRB2**	1.60	8.63E-25	1.92E-23
**MRGBP**	1.45	8.88E-25	1.98E-23
**ATP1B2**	4.39	8.89E-25	1.98E-23
**OBSL1**	2.22	9.09E-25	2.02E-23
**COLGALT1**	1.38	9.25E-25	2.06E-23
**TUSC1**	1.69	9.35E-25	2.08E-23
**FAM129B**	1.40	9.36E-25	2.08E-23
**SHB**	1.80	9.46E-25	2.10E-23
**PPP1R11**	1.00	9.53E-25	2.11E-23
**TRMT1**	1.61	1.00E-24	2.23E-23
**LINC01515**	-3.54	1.04E-24	2.31E-23
**GADD45G**	3.46	1.05E-24	2.32E-23
**FANCB**	-2.80	1.05E-24	2.33E-23

**RNF40**	0.91	1.12E-24	2.48E-23
**HEATR5B**	-1.12	1.14E-24	2.51E-23
**RNF10**	0.90	1.20E-24	2.64E-23
**KY**	-3.23	1.20E-24	2.65E-23
**POGK**	-1.01	1.21E-24	2.66E-23
**RP11-159G9.5**	-1.47	1.23E-24	2.70E-23
**RNU4-1**	-11.17	1.24E-24	2.74E-23
**TENM1**	-3.14	1.25E-24	2.75E-23
**ZNF845**	-1.48	1.28E-24	2.81E-23
**CCDC134**	-1.67	1.29E-24	2.83E-23
**PLAC4**	-6.18	1.29E-24	2.83E-23
**NECAP2**	0.94	1.36E-24	2.98E-23
**PRSS23**	3.52	1.36E-24	2.99E-23
**PLD4**	-3.09	1.43E-24	3.13E-23
**DCT**	-2.89	1.50E-24	3.28E-23
**ABCA5**	-1.98	1.51E-24	3.31E-23
**NUDT22**	1.67	1.52E-24	3.32E-23
**ORAI2**	2.80	1.58E-24	3.45E-23
**TPD52L2**	1.18	1.61E-24	3.50E-23
**FBXO10**	2.21	1.65E-24	3.60E-23
**TANC1**	-1.60	1.66E-24	3.62E-23
**ALKBH7**	1.96	1.73E-24	3.78E-23
**DST**	-1.67	1.87E-24	4.07E-23
**LINC00624**	-4.93	1.93E-24	4.19E-23
**KIAA0825**	-1.87	1.93E-24	4.19E-23
**CCDC171**	-1.74	2.04E-24	4.43E-23
**MAD2L2**	2.13	2.13E-24	4.63E-23
**UBR5**	-1.01	2.16E-24	4.69E-23
**CX3CL1**	2.88	2.19E-24	4.75E-23
**RNF139-AS1**	-3.23	2.22E-24	4.81E-23
**RNF208**	2.57	2.22E-24	4.82E-23
**ENDOG**	2.48	2.29E-24	4.96E-23
**DGAT1**	1.47	2.32E-24	5.02E-23
**JUND**	2.03	2.35E-24	5.07E-23
**ZNF655**	-1.23	2.35E-24	5.07E-23
**CARM1**	1.27	2.37E-24	5.10E-23
**HEY1**	2.91	2.39E-24	5.16E-23
**MED25**	1.70	2.42E-24	5.21E-23
**ZC3HAV1**	-0.99	2.45E-24	5.27E-23
**RNF180**	-2.02	2.47E-24	5.31E-23
**PRSS27**	3.68	2.50E-24	5.37E-23
**IFI16**	1.56	2.65E-24	5.69E-23
**CTD-2031P19.4**	3.37	2.72E-24	5.85E-23
**ZNF518A**	-1.66	2.79E-24	5.98E-23
**DTX1**	1.69	2.84E-24	6.09E-23

**SMARCD1**	0.81	2.84E-24	6.09E-23
**HIRIP3**	1.73	3.04E-24	6.51E-23
**MTMR4**	-0.87	3.17E-24	6.78E-23
**L3MBTL4**	-2.13	3.19E-24	6.81E-23
**MMRN2**	2.11	3.19E-24	6.81E-23
**SPAG7**	1.37	3.20E-24	6.84E-23
**WDR36**	-1.38	3.29E-24	7.03E-23
**NGFRAP1**	2.15	3.30E-24	7.03E-23
**PDZK1IP1**	1.80	3.34E-24	7.12E-23
**HUWE1**	-1.20	3.50E-24	7.46E-23
**SMC5**	-1.07	3.53E-24	7.52E-23
**BLVRA**	1.49	3.58E-24	7.62E-23
**ZNF493**	-2.09	3.62E-24	7.69E-23
**C1QTNF6**	3.89	3.62E-24	7.69E-23
**ZNF12**	-1.14	3.63E-24	7.70E-23
**PTGDS**	5.39	3.67E-24	7.78E-23
**PABPC4**	1.14	3.71E-24	7.86E-23
**DCUN1D1**	-1.46	3.71E-24	7.86E-23
**GUCY1B3**	3.03	3.72E-24	7.88E-23
**CSTB**	2.88	3.73E-24	7.90E-23
**NABP2**	1.55	3.87E-24	8.18E-23
**TOPORS-AS1**	-3.70	3.90E-24	8.24E-23
**PNP**	2.12	3.98E-24	8.40E-23
**FRK**	-1.52	4.04E-24	8.53E-23
**TET1**	-2.35	4.20E-24	8.86E-23
**TPR**	-1.09	4.25E-24	8.95E-23
**PITPNM1**	2.52	4.45E-24	9.37E-23
**RPS9**	1.66	4.51E-24	9.49E-23
**MORF4L1**	1.17	4.59E-24	9.64E-23
**FCGR3A**	5.09	4.59E-24	9.65E-23
**HIST1H2BB**	-10.67	4.60E-24	9.67E-23
**CNOT6L**	-1.33	4.71E-24	9.89E-23
**PFKP**	1.85	4.76E-24	9.99E-23
**BRIP1**	-1.59	4.82E-24	1.01E-22
**DDX54**	1.07	4.99E-24	1.04E-22
**COMMD7**	1.20	5.10E-24	1.07E-22
**C1orf35**	1.41	5.20E-24	1.09E-22
**MIR3687**	-7.19	5.32E-24	1.11E-22
**ODF3B**	2.22	5.39E-24	1.13E-22
**UBE2Z**	0.82	5.48E-24	1.14E-22
**RILPL2**	2.15	5.75E-24	1.20E-22
**G34162**	4.62	5.83E-24	1.21E-22
**GSS**	1.14	5.85E-24	1.22E-22
**PLD2**	1.46	5.89E-24	1.23E-22
**FAM135A**	-1.75	5.94E-24	1.24E-22

**HIST1H2BM**	-10.48	5.96E-24	1.24E-22
**FKRP**	2.03	6.16E-24	1.28E-22
**SLC16A3**	4.29	6.26E-24	1.30E-22
**ZNF253**	-1.45	6.29E-24	1.31E-22
**NUP205**	-0.91	6.72E-24	1.39E-22
**SMARCD3**	2.47	6.74E-24	1.40E-22
**LAMTOR5-AS1**	-3.07	6.92E-24	1.43E-22
**ABHD17A**	1.48	7.37E-24	1.53E-22
**SGCG**	-3.29	7.47E-24	1.54E-22
**HNRNPUL1**	0.92	7.68E-24	1.59E-22
**TNFRSF1B**	3.18	7.87E-24	1.63E-22
**ABHD12B**	-2.08	7.90E-24	1.63E-22
**CTSL**	3.35	8.09E-24	1.67E-22
**ELOF1**	1.65	8.17E-24	1.69E-22
**PBRM1**	-1.15	8.21E-24	1.69E-22
**SSBP4**	2.55	8.56E-24	1.76E-22
**B3GNT9**	1.69	8.60E-24	1.77E-22
**SWI5**	1.52	8.68E-24	1.79E-22
**POR**	1.44	8.81E-24	1.81E-22
**LMF2**	1.78	8.83E-24	1.81E-22
**G38513**	-3.71	9.34E-24	1.92E-22
**CTU1**	1.77	9.35E-24	1.92E-22
**RN7SL471P**	-8.58	9.62E-24	1.97E-22
**PKIG**	2.38	9.89E-24	2.03E-22
**G38514**	-2.31	1.01E-23	2.06E-22
**SNORA37**	-8.09	1.01E-23	2.07E-22
**HMOX1**	2.37	1.02E-23	2.08E-22
**TCF7L1**	3.37	1.08E-23	2.20E-22
**SULF1**	3.96	1.08E-23	2.21E-22
**AC008074.4**	-3.10	1.10E-23	2.24E-22
**RP11-547D13.1**	-2.57	1.10E-23	2.25E-22
**CNKSR2**	-4.00	1.11E-23	2.25E-22
**AGTRAP**	2.06	1.11E-23	2.26E-22
**B3GALT6**	1.33	1.13E-23	2.31E-22
**CDPF1**	1.67	1.14E-23	2.32E-22
**NRXN2**	5.37	1.17E-23	2.37E-22
**ZCCHC3**	1.03	1.17E-23	2.38E-22
**OS9**	1.21	1.17E-23	2.38E-22
**TBC1D10B**	1.30	1.22E-23	2.48E-22
**PTGES2**	1.41	1.27E-23	2.57E-22
**ZNF540**	-2.71	1.28E-23	2.59E-22
**STX10**	1.46	1.28E-23	2.60E-22
**TEAD3**	1.28	1.33E-23	2.70E-22
**OSBPL8**	-1.75	1.34E-23	2.72E-22
**ERI3**	0.99	1.37E-23	2.76E-22

**C18orf25**	-1.02	1.37E-23	2.76E-22
**DYNC2H1**	-1.58	1.40E-23	2.82E-22
**MAPKAPK2**	1.13	1.41E-23	2.85E-22
**HIST1H2AL**	-10.40	1.42E-23	2.86E-22
**PCBP1**	1.61	1.45E-23	2.92E-22
**IGFBP2**	4.03	1.51E-23	3.05E-22
**RPL18A**	1.64	1.55E-23	3.11E-22
**ITCH**	-1.20	1.56E-23	3.13E-22
**RPIA**	1.15	1.59E-23	3.20E-22
**TRIP11**	-1.32	1.60E-23	3.22E-22
**NIPAL1**	-2.12	1.60E-23	3.22E-22
**ZNF654**	-1.34	1.61E-23	3.23E-22
**ANGEL2**	-0.91	1.63E-23	3.26E-22
**TUSC2**	1.28	1.63E-23	3.26E-22
**SENP6**	-0.97	1.67E-23	3.34E-22
**RAB30-AS1**	-2.02	1.70E-23	3.40E-22
**SLC3A2**	1.18	1.70E-23	3.40E-22
**SEC61A1**	1.04	1.70E-23	3.41E-22
**FAM20A**	3.51	1.77E-23	3.53E-22
**CHST3**	1.44	1.88E-23	3.75E-22
**AC006262.11**	-5.60	2.01E-23	4.02E-22
**NARF**	1.08	2.02E-23	4.02E-22
**RHOB**	2.24	2.03E-23	4.04E-22
**OGFRL1**	-1.63	2.03E-23	4.05E-22
**PDE4D**	-1.32	2.05E-23	4.08E-22
**C6orf48**	1.52	2.07E-23	4.11E-22
**EDN2**	4.97	2.21E-23	4.38E-22
**WDR13**	0.99	2.27E-23	4.50E-22
**ECT2L**	-3.66	2.35E-23	4.67E-22
**SPRR1A**	5.80	2.36E-23	4.67E-22
**KIF21A**	-2.45	2.36E-23	4.68E-22
**MED15**	1.83	2.42E-23	4.80E-22
**ZNF814**	-1.50	2.71E-23	5.37E-22
**RPS14**	1.60	2.74E-23	5.42E-22
**PNPLA6**	1.37	2.77E-23	5.48E-22
**KIAA1551**	-1.01	2.77E-23	5.48E-22
**TLDC1**	1.49	2.80E-23	5.54E-22
**TMEM176B**	4.66	2.84E-23	5.61E-22
**PARL**	1.26	2.89E-23	5.69E-22
**ZNF784**	1.38	2.94E-23	5.79E-22
**EEA1**	-1.35	3.10E-23	6.10E-22
**ATP6AP1**	1.07	3.12E-23	6.15E-22
**KRT5**	1.84	3.29E-23	6.47E-22
**VPS18**	1.14	3.35E-23	6.58E-22
**POU2F1**	-1.22	3.38E-23	6.64E-22

**TGFB1I1**	2.81	3.40E-23	6.68E-22
**SGMS2**	-1.12	3.42E-23	6.71E-22
**HNRNPU-AS1**	-3.61	3.45E-23	6.76E-22
**USP54**	-1.41	3.72E-23	7.29E-22
**MKL1**	1.23	3.72E-23	7.30E-22
**CMYA5**	-2.77	3.75E-23	7.34E-22
**FHOD1**	2.09	3.75E-23	7.35E-22
**AP1M1**	0.99	3.84E-23	7.52E-22
**ADAM23**	4.85	3.86E-23	7.55E-22
**VPS50**	-1.21	3.95E-23	7.71E-22
**YWHAH**	1.42	4.08E-23	7.96E-22
**ZNF420**	-1.42	4.10E-23	7.99E-22
**CTC-343N3.1**	-5.28	4.15E-23	8.09E-22
**CD93**	3.65	4.19E-23	8.17E-22
**FAM153B**	-5.05	4.24E-23	8.26E-22
**N4BP2L2**	-1.36	4.36E-23	8.48E-22
**PSKH1**	1.31	4.37E-23	8.50E-22
**PPM1G**	1.06	4.38E-23	8.52E-22
**SLC39A1**	1.47	4.41E-23	8.57E-22
**CDK2AP2**	1.95	4.42E-23	8.58E-22
**ZNF260**	-1.41	4.52E-23	8.76E-22
**CSNK2B**	-1.40	4.60E-23	8.92E-22
**SPP1**	8.63	4.72E-23	9.15E-22
**XLOC_013866**	-3.84	4.82E-23	9.33E-22
**FAM43A**	2.32	4.85E-23	9.38E-22
**ZBTB44**	-1.19	4.92E-23	9.51E-22
**UBE2R2**	0.98	4.93E-23	9.54E-22
**SULF2**	3.21	5.24E-23	1.01E-21
**CNOT3**	1.41	5.32E-23	1.03E-21
**NGF**	4.91	5.35E-23	1.03E-21
**NES**	3.19	5.51E-23	1.06E-21
**CYB5D2**	1.46	5.53E-23	1.06E-21
**HIST1H3C**	-9.99	5.73E-23	1.10E-21
**ANKIB1**	-0.84	5.98E-23	1.15E-21
**SNORA23**	-10.90	6.02E-23	1.16E-21
**ARPC1B**	2.35	6.04E-23	1.16E-21
**PTGIR**	4.45	6.05E-23	1.16E-21
**PPP1R35**	1.68	6.06E-23	1.16E-21
**HIST3H2BB**	-2.55	6.11E-23	1.17E-21
**CDCA7L**	-1.34	6.52E-23	1.25E-21
**RNU5B-1**	-12.33	6.54E-23	1.25E-21
**BCL11B**	-1.67	6.70E-23	1.28E-21
**CCDC34**	1.78	6.73E-23	1.29E-21
**SLC47A1**	-2.84	6.86E-23	1.31E-21
**ACADS**	1.97	6.91E-23	1.32E-21

**SLF2**	-1.18	6.98E-23	1.33E-21
**HIST1H2AJ**	-10.32	7.31E-23	1.40E-21
**RHBDD2**	1.46	7.46E-23	1.42E-21
**ILVBL**	1.52	7.56E-23	1.44E-21
**MAN1B1**	1.10	7.66E-23	1.46E-21
**CXorf36**	2.96	7.68E-23	1.46E-21
**PITPNC1**	2.88	8.45E-23	1.61E-21
**ZNF775**	2.22	8.47E-23	1.61E-21
**ZBTB41**	-1.50	8.58E-23	1.63E-21
**ZRSR2**	1.45	8.60E-23	1.63E-21
**RNF126**	1.29	8.70E-23	1.65E-21
**ARMC7**	1.38	8.88E-23	1.69E-21
**AHCTF1**	-1.03	9.00E-23	1.71E-21
**MAP3K1**	-1.68	9.17E-23	1.74E-21
**COASY**	1.30	9.85E-23	1.87E-21
**SMOC2**	3.22	9.88E-23	1.87E-21
**HIST1H2BO**	-10.25	9.94E-23	1.88E-21
**TTF1**	1.03	1.01E-22	1.90E-21
**XCR1**	-2.58	1.02E-22	1.92E-21
**CDK5R1**	2.25	1.02E-22	1.93E-21
**MEN1**	1.30	1.04E-22	1.96E-21
**EMC9**	1.33	1.05E-22	1.99E-21
**G6PC3**	2.12	1.10E-22	2.07E-21
**TSPAN9**	1.41	1.10E-22	2.07E-21
**CHIC1**	-1.60	1.11E-22	2.08E-21
**TINF2**	0.99	1.11E-22	2.10E-21
**BCL2L11**	1.69	1.12E-22	2.11E-21
**RASSF1**	1.51	1.13E-22	2.12E-21
**HIST1H4A**	-10.32	1.14E-22	2.15E-21
**SIKE1**	-1.24	1.19E-22	2.24E-21
**FAM96B**	1.34	1.21E-22	2.27E-21
**REST**	-1.31	1.25E-22	2.35E-21
**RAB31**	2.57	1.29E-22	2.42E-21
**COL15A1**	3.92	1.29E-22	2.43E-21
**TINAGL1**	2.95	1.32E-22	2.48E-21
**ABCB11**	-3.53	1.36E-22	2.55E-21
**SHROOM1**	3.62	1.37E-22	2.56E-21
**FGD6**	-1.27	1.43E-22	2.67E-21
**RP11-690D19.3**	-1.72	1.45E-22	2.70E-21
**G3383**	-2.05	1.45E-22	2.71E-21
**MRPL10**	1.13	1.45E-22	2.71E-21
**MIB1**	-1.14	1.48E-22	2.76E-21
**ESRRA**	1.58	1.54E-22	2.86E-21
**PDE1B**	3.18	1.54E-22	2.87E-21
**TMEM176A**	4.90	1.55E-22	2.88E-21

**IFT43**	1.01	1.59E-22	2.95E-21
**C19orf70**	1.21	1.60E-22	2.97E-21
**TMPRSS6**	-5.17	1.65E-22	3.07E-21
**CTF1**	3.60	1.66E-22	3.08E-21
**RHOC**	1.47	1.66E-22	3.09E-21
**LRFN3**	1.78	1.69E-22	3.13E-21
**SCRG1**	-3.83	1.79E-22	3.33E-21
**UBE2S**	2.23	1.82E-22	3.37E-21
**APEX1**	1.57	1.87E-22	3.46E-21
**C3orf58**	-1.59	1.87E-22	3.47E-21
**APOA1BP**	1.26	1.96E-22	3.63E-21
**KEAP1**	1.17	2.02E-22	3.74E-21
**TSPAN15**	3.27	2.04E-22	3.76E-21
**HMOX2**	1.22	2.06E-22	3.80E-21
**ACAP2**	-1.38	2.10E-22	3.88E-21
**PFN2**	1.94	2.12E-22	3.91E-21
**ERCC6**	-1.22	2.13E-22	3.92E-21
**MCM8**	-1.06	2.14E-22	3.94E-21
**ADM**	3.29	2.23E-22	4.10E-21
**POF1B**	-2.42	2.26E-22	4.15E-21
**ZBTB7A**	1.67	2.26E-22	4.16E-21
**S100A2**	3.10	2.29E-22	4.21E-21
**PSMA7**	1.21	2.34E-22	4.29E-21
**NUDT14**	2.18	2.37E-22	4.35E-21
**RUVBL2**	1.18	2.39E-22	4.39E-21
**SQSTM1**	1.33	2.41E-22	4.41E-21
**FAM110D**	3.46	2.45E-22	4.48E-21
**STOML1**	1.21	2.46E-22	4.51E-21
**HBA1**	-5.41	2.48E-22	4.55E-21
**CELF1**	-0.63	2.55E-22	4.66E-21
**NSUN3**	-1.16	2.55E-22	4.66E-21
**RAB20**	3.43	2.59E-22	4.73E-21
**PPP6R3**	-0.86	2.63E-22	4.80E-21
**HIPK1**	-1.29	2.63E-22	4.80E-21
**KLK13**	2.76	2.64E-22	4.82E-21
**TMUB2**	1.39	2.67E-22	4.87E-21
**PPP1R14B**	1.32	2.71E-22	4.94E-21
**GSK3B**	-1.04	2.72E-22	4.95E-21
**FRRS1**	-1.36	2.76E-22	5.02E-21
**SLC38A5**	2.08	2.79E-22	5.08E-21
**ZNF571**	-1.58	2.79E-22	5.08E-21
**TRIM13**	-1.16	2.83E-22	5.14E-21
**PMEPA1**	2.48	2.84E-22	5.16E-21
**ZNF638-IT1**	-3.75	2.85E-22	5.16E-21
**PPP1CA**	1.20	2.88E-22	5.22E-21

**MRPS5**	0.99	2.91E-22	5.27E-21
**IRF2BPL**	1.11	2.91E-22	5.28E-21
**DCAF16**	-1.13	3.11E-22	5.63E-21
**UNC119**	1.08	3.24E-22	5.86E-21
**COG5**	-0.89	3.31E-22	5.99E-21
**MMS22L**	-1.38	3.43E-22	6.20E-21
**ZNF682**	-1.61	3.49E-22	6.31E-21
**RAC1**	1.03	3.54E-22	6.39E-21
**NDRG4**	3.00	3.55E-22	6.41E-21
**SDF2**	1.09	3.58E-22	6.46E-21
**NDUFV1**	0.99	3.62E-22	6.53E-21
**DDX6**	-1.11	3.71E-22	6.67E-21
**CUL5**	-1.07	3.75E-22	6.75E-21
**B3GAT3**	1.32	3.83E-22	6.89E-21
**SMG9**	1.71	3.98E-22	7.17E-21
**RP11-1008C21.1**	-5.58	4.02E-22	7.22E-21
**EEF2K**	-2.14	4.08E-22	7.33E-21
**MRE11A**	-0.96	4.09E-22	7.34E-21
**TTYH3**	2.75	4.15E-22	7.44E-21
**TYMP**	3.12	4.21E-22	7.55E-21
**KIAA1958**	-1.70	4.24E-22	7.61E-21
**GORASP2**	1.01	4.39E-22	7.87E-21
**ZNF507**	-1.05	4.41E-22	7.90E-21
**NKAIN1**	2.55	4.42E-22	7.91E-21
**ZNF638**	-1.02	4.44E-22	7.93E-21
**LINGO1**	3.62	4.44E-22	7.93E-21
**LIMD1**	-0.89	4.58E-22	8.19E-21
**RP11-295P9.3**	-2.12	4.66E-22	8.31E-21
**PDLIM1**	1.52	4.69E-22	8.37E-21
**FAM101A**	5.30	4.70E-22	8.38E-21
**FARSA**	1.16	4.72E-22	8.42E-21
**LBH**	2.65	4.77E-22	8.50E-21
**SNX17**	1.09	4.78E-22	8.51E-21
**B3GNT8**	1.93	5.17E-22	9.20E-21
**ZNF76**	1.32	5.37E-22	9.55E-21
**DECR2**	1.79	5.47E-22	9.72E-21
**HIST1H3A**	-9.98	5.75E-22	1.02E-20
**SCARA5**	-4.42	5.78E-22	1.03E-20
**PHF23**	1.28	6.02E-22	1.07E-20
**RALGAPB**	-1.01	6.13E-22	1.09E-20
**RNU2-59P**	-11.08	6.19E-22	1.10E-20
**TIMM10**	1.72	6.20E-22	1.10E-20
**PCGF5**	-1.75	6.22E-22	1.10E-20
**PLEKHH3**	1.61	6.25E-22	1.11E-20
**FAM234A**	1.40	6.28E-22	1.11E-20

**EFCC1**	2.00	6.29E-22	1.11E-20
**PCNX**	-0.92	6.30E-22	1.11E-20
**NSFL1C**	1.04	6.39E-22	1.13E-20
**YIF1B**	1.63	6.54E-22	1.15E-20
**MIR99AHG**	-2.04	6.58E-22	1.16E-20
**LGR4**	-1.36	6.60E-22	1.16E-20
**RANBP3**	0.85	6.65E-22	1.17E-20
**NAGK**	1.57	6.69E-22	1.18E-20
**PARP3**	1.88	6.75E-22	1.19E-20
**PBX2**	0.77	6.83E-22	1.20E-20
**SNORA71A**	-8.73	7.13E-22	1.25E-20
**VMO1**	5.14	7.14E-22	1.25E-20
**RBAK**	-0.98	7.25E-22	1.27E-20
**RNF5**	1.45	7.29E-22	1.28E-20
**TPP2**	-1.10	7.44E-22	1.30E-20
**KIAA0895**	-2.25	7.47E-22	1.31E-20
**SIX5**	2.49	7.51E-22	1.32E-20
**HUNK**	-1.74	7.60E-22	1.33E-20
**ADAMTS4**	4.92	7.62E-22	1.33E-20
**GRB10**	2.47	7.65E-22	1.34E-20
**COMP**	6.47	7.75E-22	1.36E-20
**TMEM161A**	1.51	7.91E-22	1.38E-20
**CPLX1**	4.84	7.92E-22	1.38E-20
**LRPAP1**	1.12	8.15E-22	1.42E-20
**SLC39A13**	1.59	8.33E-22	1.45E-20
**DSC2**	2.84	8.41E-22	1.47E-20
**ZNF782**	-1.53	8.49E-22	1.48E-20
**CTSZ**	2.71	8.51E-22	1.48E-20
**RC3H2**	-0.93	8.62E-22	1.50E-20
**PPP1R18**	1.78	8.69E-22	1.51E-20
**DVL3**	1.34	9.13E-22	1.59E-20
**GUSB**	1.72	9.18E-22	1.59E-20
**KANK3**	3.39	9.51E-22	1.65E-20
**THAP4**	0.92	9.75E-22	1.69E-20
**BMP1**	2.76	9.80E-22	1.70E-20
**RP11-557H15.3**	3.65	9.94E-22	1.72E-20
**ZNF708**	-1.76	9.96E-22	1.73E-20
**ERG**	2.98	1.01E-21	1.76E-20
**PLEKHM2**	1.23	1.03E-21	1.78E-20
**KCTD10**	1.10	1.11E-21	1.91E-20
**SAMD8**	-0.95	1.11E-21	1.92E-20
**ANKRD36BP2**	-3.39	1.11E-21	1.92E-20
**FBXO2**	2.13	1.14E-21	1.97E-20
**ZNF681**	-2.14	1.15E-21	1.99E-20
**APLP2**	1.15	1.16E-21	2.00E-20

**CAPN1**	1.22	1.16E-21	2.01E-20
**RP11-61K9.3**	1.54	1.19E-21	2.06E-20
**LTB4R**	1.73	1.20E-21	2.06E-20
**FBRS**	1.66	1.21E-21	2.08E-20
**G21034**	-6.53	1.21E-21	2.09E-20
**MYSM1**	-1.26	1.25E-21	2.15E-20
**DLST**	0.99	1.26E-21	2.16E-20
**EDEM2**	1.06	1.28E-21	2.19E-20
**VPS37B**	1.24	1.28E-21	2.19E-20
**AHNAK2**	-2.02	1.29E-21	2.22E-20
**AC072062.1**	-2.86	1.31E-21	2.25E-20
**FLYWCH2**	1.61	1.32E-21	2.26E-20
**RP3-453C12.14**	-4.76	1.33E-21	2.28E-20
**SYNGR2**	1.22	1.35E-21	2.31E-20
**PCF11**	-1.18	1.40E-21	2.40E-20
**PGLS**	1.04	1.45E-21	2.48E-20
**ARL8A**	1.43	1.46E-21	2.50E-20
**TAF3**	1.10	1.47E-21	2.51E-20
**ZNF841**	-1.51	1.49E-21	2.55E-20
**SORCS2**	3.38	1.50E-21	2.56E-20
**LAGE3**	1.56	1.51E-21	2.58E-20
**GRB2**	0.87	1.53E-21	2.60E-20
**COPE**	1.22	1.59E-21	2.71E-20
**RASD1**	3.39	1.61E-21	2.74E-20
**ATP2B1**	-1.70	1.62E-21	2.75E-20
**DAP**	1.58	1.63E-21	2.77E-20
**RP4-635A23.6**	-2.68	1.63E-21	2.77E-20
**HIST1H2AB**	-9.97	1.65E-21	2.80E-20
**AC007038.7**	-4.06	1.70E-21	2.88E-20
**FAH**	1.67	1.72E-21	2.92E-20
**HIST1H4L**	-10.38	1.77E-21	3.00E-20
**ITPK1**	1.26	1.78E-21	3.03E-20
**ZC3H3**	1.68	1.79E-21	3.03E-20
**ZNF483**	-2.70	1.79E-21	3.04E-20
**TNFSF9**	3.49	1.82E-21	3.08E-20
**RNF215**	1.96	1.82E-21	3.08E-20
**HMGN2**	1.64	1.86E-21	3.15E-20
**RUSC1**	1.45	1.91E-21	3.22E-20
**CXCL16**	1.71	1.96E-21	3.31E-20
**ZNF236**	-1.04	2.03E-21	3.43E-20
**PNRC2**	2.32	2.09E-21	3.53E-20
**RANBP2**	-1.45	2.09E-21	3.53E-20
**TNFSF12**	2.94	2.10E-21	3.55E-20
**PITX1**	4.76	2.13E-21	3.58E-20
**SLC4A2**	1.33	2.15E-21	3.62E-20

**TSFM**	1.10	2.15E-21	3.62E-20
**NUP58**	-1.04	2.15E-21	3.62E-20
**EDNRA**	2.84	2.16E-21	3.64E-20
**TAOK1**	-1.28	2.25E-21	3.78E-20
**TMEM67**	-1.39	2.26E-21	3.79E-20
**TTC7B**	1.79	2.31E-21	3.88E-20
**GSDMD**	2.65	2.35E-21	3.93E-20
**SELM**	3.06	2.36E-21	3.96E-20
**SGSH**	1.47	2.37E-21	3.97E-20
**NCBP1**	-1.11	2.51E-21	4.20E-20
**UNC93B1**	1.53	2.52E-21	4.22E-20
**NAGLU**	2.02	2.53E-21	4.24E-20
**MRPL9**	1.21	2.56E-21	4.28E-20
**PKN1**	3.21	2.61E-21	4.36E-20
**COX4I1**	1.23	2.61E-21	4.36E-20
**MFSD8**	-1.26	2.62E-21	4.38E-20
**CAB39L**	-1.49	2.66E-21	4.43E-20
**RNMTL1**	1.20	2.69E-21	4.48E-20
**LRRC45**	1.47	3.09E-21	5.15E-20
**MPP7**	-1.96	3.17E-21	5.28E-20
**MYH9**	1.10	3.19E-21	5.30E-20
**COMMD5**	1.15	3.20E-21	5.33E-20
**NEO1**	-1.16	3.21E-21	5.33E-20
**TELO2**	1.88	3.21E-21	5.33E-20
**CDR2L**	2.07	3.23E-21	5.37E-20
**SP3**	-1.06	3.25E-21	5.40E-20
**SRRM3**	2.13	3.26E-21	5.41E-20
**HECA**	-1.24	3.26E-21	5.41E-20
**EPHX1**	1.93	3.29E-21	5.46E-20
**CXCR4**	4.36	3.30E-21	5.46E-20
**ARAP2**	-1.72	3.33E-21	5.52E-20
**RPS6KA3**	-1.11	3.34E-21	5.53E-20
**FHL2**	1.48	3.37E-21	5.58E-20
**FBXW2**	-1.07	3.39E-21	5.61E-20
**HEBP1**	1.33	3.45E-21	5.71E-20
**GNG12-AS1**	-2.22	3.51E-21	5.81E-20
**ABCA1**	-1.64	3.67E-21	6.05E-20
**C5orf46**	-2.84	3.68E-21	6.07E-20
**PLAUR**	3.51	3.83E-21	6.32E-20
**EGFL6**	6.79	3.97E-21	6.54E-20
**BCKDK**	1.42	3.98E-21	6.57E-20
**CYTH2**	1.23	4.04E-21	6.66E-20
**ZNF777**	1.34	4.15E-21	6.83E-20
**G18244**	3.69	4.19E-21	6.90E-20
**GAPVD1**	-1.28	4.44E-21	7.30E-20

**CHST14**	2.20	4.46E-21	7.33E-20
**EPB41L1**	1.77	4.53E-21	7.44E-20
**CIB2**	2.40	4.61E-21	7.58E-20
**CTSC**	1.95	4.69E-21	7.70E-20
**C4A**	4.87	4.69E-21	7.70E-20
**HES4**	2.38	4.86E-21	7.96E-20
**OXA1L**	0.80	5.08E-21	8.32E-20
**MBD6**	2.79	5.42E-21	8.88E-20
**SNORA71D**	-10.66	5.48E-21	8.97E-20
**TRIB2**	2.35	5.56E-21	9.10E-20
**FAM65C**	3.00	5.74E-21	9.39E-20
**ARL3**	1.26	5.90E-21	9.64E-20
**ZNF92**	-1.66	6.06E-21	9.90E-20
**MARVELD1**	1.93	6.23E-21	1.02E-19
**KRIT1**	-0.75	6.36E-21	1.04E-19
**NCBP2-AS2**	1.40	6.45E-21	1.05E-19
**SPATA18**	-1.08	6.47E-21	1.05E-19
**S1PR1**	3.06	6.48E-21	1.06E-19
**TRAPPC8**	-1.29	6.53E-21	1.06E-19
**IDH2**	1.85	6.60E-21	1.07E-19
**HECTD1**	-1.29	6.66E-21	1.08E-19
**BHLHE40**	2.01	6.69E-21	1.09E-19
**PNKD**	1.30	7.00E-21	1.14E-19
**ZFP36L1**	1.76	7.14E-21	1.16E-19
**RNF26**	1.66	7.24E-21	1.18E-19
**NFE2L1**	0.81	7.27E-21	1.18E-19
**SP2**	2.17	7.48E-21	1.21E-19
**ZFP14**	-1.45	7.53E-21	1.22E-19
**NDRG1**	1.95	7.64E-21	1.24E-19
**TBC1D22A**	0.95	7.65E-21	1.24E-19
**KLHDC10**	-0.85	7.67E-21	1.24E-19
**PHF3**	-1.15	7.78E-21	1.26E-19
**PRRX2**	1.75	7.98E-21	1.29E-19
**SUCO**	-1.08	8.03E-21	1.30E-19
**DPP9**	1.14	8.17E-21	1.32E-19
**NID1**	3.43	8.31E-21	1.34E-19
**PTDSS2**	1.26	8.43E-21	1.36E-19
**WDR60**	1.54	8.66E-21	1.40E-19
**FAM109A**	1.58	8.91E-21	1.44E-19
**YIPF2**	1.46	8.92E-21	1.44E-19
**CTD-2647L4.4**	-2.06	9.28E-21	1.49E-19
**TNKS**	-1.04	9.48E-21	1.53E-19
**WDR45**	1.09	9.52E-21	1.53E-19
**CHN1**	3.60	9.55E-21	1.54E-19
**TMEM54**	1.77	9.62E-21	1.55E-19

**RHOG**	1.43	9.74E-21	1.56E-19
**VPS41**	-0.98	1.00E-20	1.61E-19
**RP11-362K14.5**	-4.14	1.02E-20	1.64E-19
**PHF6**	-1.24	1.03E-20	1.65E-19
**SEC24B**	-0.93	1.04E-20	1.66E-19
**LMCD1**	3.04	1.04E-20	1.66E-19
**PCED1B**	2.55	1.05E-20	1.68E-19
**UROD**	1.27	1.05E-20	1.68E-19
**HSPB1**	2.10	1.05E-20	1.69E-19
**TMEM8A**	2.56	1.07E-20	1.71E-19
**EXOC6B**	-1.59	1.07E-20	1.71E-19
**LCE5A**	-3.37	1.08E-20	1.72E-19
**NOVA2**	3.91	1.09E-20	1.74E-19
**HBA2**	-4.97	1.12E-20	1.79E-19
**NRBP1**	0.84	1.16E-20	1.84E-19
**DOCK7**	-0.90	1.16E-20	1.85E-19
**AK7**	-2.19	1.18E-20	1.87E-19
**TPM2**	3.78	1.18E-20	1.88E-19
**RP11-701H24.7**	-4.06	1.18E-20	1.88E-19
**SCARF1**	2.64	1.19E-20	1.89E-19
**MCHR1**	3.92	1.19E-20	1.90E-19
**SNTB1**	-1.54	1.19E-20	1.90E-19
**EDRF1**	-1.33	1.21E-20	1.92E-19
**TMEM161B**	-1.28	1.22E-20	1.94E-19
**SLC25A39**	1.33	1.23E-20	1.94E-19
**TIMM17B**	1.46	1.24E-20	1.96E-19
**ZNF721**	-1.44	1.25E-20	1.98E-19
**CDK13**	-0.64	1.25E-20	1.98E-19
**SGK1**	2.08	1.25E-20	1.99E-19
**BYSL**	1.39	1.26E-20	1.99E-19
**ATF4**	1.15	1.26E-20	2.00E-19
**DHX15**	-0.76	1.28E-20	2.03E-19
**DNAJB14**	-1.33	1.30E-20	2.05E-19
**ZNF362**	1.28	1.32E-20	2.08E-19
**RNA5-8SP6**	7.45	1.34E-20	2.11E-19
**SHMT2**	1.09	1.35E-20	2.13E-19
**SLC25A28**	1.67	1.36E-20	2.14E-19
**THY1**	5.45	1.36E-20	2.15E-19
**CXCL2**	5.44	1.41E-20	2.23E-19
**COMT**	1.24	1.52E-20	2.39E-19
**GJC1**	3.35	1.53E-20	2.41E-19
**TMEM88B**	5.52	1.53E-20	2.41E-19
**COL9A3**	1.73	1.53E-20	2.41E-19
**ANAPC1**	-1.00	1.55E-20	2.43E-19
**SNORA5C**	-4.04	1.55E-20	2.44E-19

**LSM10**	1.82	1.56E-20	2.45E-19
**ALDH16A1**	1.48	1.57E-20	2.46E-19
**HGS**	0.99	1.59E-20	2.49E-19
**ASPM**	-2.10	1.59E-20	2.49E-19
**C19orf25**	1.44	1.59E-20	2.49E-19
**KCNJ8**	4.51	1.61E-20	2.52E-19
**IL20RB-AS1**	-3.31	1.61E-20	2.52E-19
**RP11-101E13.5**	-1.69	1.61E-20	2.52E-19
**SDCBP2-AS1**	-2.41	1.64E-20	2.56E-19
**ATG101**	1.32	1.65E-20	2.58E-19
**METRNL**	1.40	1.68E-20	2.62E-19
**ASPA**	-2.19	1.69E-20	2.64E-19
**ZNF397**	-1.08	1.74E-20	2.71E-19
**SPAG1**	-2.02	1.77E-20	2.76E-19
**GALE**	1.52	1.80E-20	2.81E-19
**NDUFV2**	-3.26	1.80E-20	2.81E-19
**BMPR1A**	-1.05	1.82E-20	2.84E-19
**GLIS2**	3.62	1.83E-20	2.85E-19
**MAP4K4**	1.31	1.84E-20	2.86E-19
**CD320**	2.54	1.85E-20	2.88E-19
**WDR5**	0.79	1.85E-20	2.88E-19
**BST2**	4.15	1.87E-20	2.90E-19
**LRFN4**	2.12	1.92E-20	2.98E-19
**SLC9A3R1**	1.53	1.96E-20	3.05E-19
**MGMT**	1.67	2.05E-20	3.18E-19
**PAXIP1-AS1**	1.97	2.07E-20	3.22E-19
**P3H1**	2.71	2.13E-20	3.30E-19
**MIR222HG**	-3.48	2.19E-20	3.39E-19
**FNIP1**	-1.25	2.19E-20	3.39E-19
**PIP5K1C**	1.59	2.19E-20	3.40E-19
**KLF3**	-1.25	2.20E-20	3.40E-19
**SLC35B2**	1.44	2.27E-20	3.51E-19
**ARHGEF28**	-1.66	2.33E-20	3.61E-19
**WDR34**	1.14	2.42E-20	3.74E-19
**MCAT**	1.09	2.42E-20	3.74E-19
**OXR1**	-1.42	2.53E-20	3.91E-19
**PALD1**	1.96	2.56E-20	3.95E-19
**IRF2**	0.97	2.57E-20	3.96E-19
**TET3**	-1.49	2.58E-20	3.98E-19
**CAPN5**	2.79	2.63E-20	4.05E-19
**LAYN**	3.52	2.64E-20	4.07E-19
**HIST1H1A**	-9.71	2.70E-20	4.16E-19
**AC058791.1**	-4.11	2.74E-20	4.22E-19
**IBTK**	-0.99	2.81E-20	4.32E-19
**SNX13**	-1.19	2.81E-20	4.32E-19

**SNORA74A**	-10.41	2.81E-20	4.32E-19
**RP6-109B7.4**	-4.11	2.82E-20	4.33E-19
**TRAF3IP2**	0.82	2.82E-20	4.33E-19
**UBR1**	-0.89	2.92E-20	4.48E-19
**SNORA53**	-8.62	2.93E-20	4.50E-19
**TNFRSF10B**	1.74	2.95E-20	4.52E-19
**RNU11**	-6.49	3.08E-20	4.72E-19
**RP11-218F10.3**	-4.50	3.13E-20	4.79E-19
**ETS1**	1.80	3.14E-20	4.81E-19
**KRT16**	7.16	3.16E-20	4.83E-19
**CTD-2104P17.2**	-6.32	3.22E-20	4.93E-19
**RNU2-6P**	-10.70	3.24E-20	4.95E-19
**TMEM106B**	-1.54	3.25E-20	4.97E-19
**LTBP3**	2.12	3.27E-20	4.99E-19
**P4HA3**	4.65	3.28E-20	5.01E-19
**SEC11A**	1.07	3.30E-20	5.04E-19
**FAM181B**	3.84	3.32E-20	5.06E-19
**ASCC3**	-1.32	3.36E-20	5.12E-19
**ILF3**	0.89	3.38E-20	5.15E-19
**TBC1D17**	1.39	3.44E-20	5.24E-19
**ATP8A1**	-2.07	3.45E-20	5.25E-19
**CREB3**	1.33	3.46E-20	5.27E-19
**ZNF800**	-1.35	3.48E-20	5.30E-19
**AP1G1**	-0.85	3.50E-20	5.32E-19
**FRS2**	-1.10	3.57E-20	5.42E-19
**APP**	1.05	3.64E-20	5.52E-19
**AKT1**	1.09	3.73E-20	5.66E-19
**LAMTOR1**	1.11	3.83E-20	5.81E-19
**CDKN2D**	1.85	3.86E-20	5.85E-19
**R3HCC1**	1.17	3.92E-20	5.94E-19
**HSF1**	1.27	3.93E-20	5.95E-19
**PHB**	1.33	3.94E-20	5.97E-19
**MLH3**	-0.95	4.01E-20	6.07E-19
**TTC39C**	-1.09	4.02E-20	6.09E-19
**ZDBF2**	-1.45	4.14E-20	6.27E-19
**PPP2R1A**	1.00	4.15E-20	6.27E-19
**RNU2-61P**	-11.38	4.28E-20	6.47E-19
**ZNF205**	1.51	4.49E-20	6.77E-19
**GPR18**	-4.67	4.53E-20	6.83E-19
**PTPN11**	-1.10	4.54E-20	6.85E-19
**OCLN**	-1.72	4.62E-20	6.96E-19
**NELFE**	1.01	4.64E-20	6.99E-19
**CTSA**	1.28	4.69E-20	7.06E-19
**HIST1H2BD**	-2.96	4.73E-20	7.12E-19
**MCOLN3**	-2.40	4.76E-20	7.16E-19

**SERPINB1**	2.01	4.76E-20	7.16E-19
**TSEN34**	1.16	4.82E-20	7.24E-19
**ANKAR**	-2.02	4.86E-20	7.31E-19
**MED9**	0.79	4.90E-20	7.36E-19
**EXOC3L1**	2.03	5.01E-20	7.53E-19
**BRK1**	1.22	5.07E-20	7.61E-19
**VASP**	1.23	5.11E-20	7.67E-19
**SND1**	0.75	5.13E-20	7.68E-19
**SMG1**	-1.50	5.21E-20	7.80E-19
**FAM214B**	1.37	5.29E-20	7.92E-19
**SERPINH1**	2.70	5.35E-20	8.00E-19
**PPARD**	1.96	5.37E-20	8.03E-19
**EFHD1**	3.50	5.37E-20	8.03E-19
**MCPH1**	-0.81	5.53E-20	8.27E-19
**RAB11FIP1**	-1.67	5.58E-20	8.34E-19
**PLEKHO2**	2.46	5.65E-20	8.43E-19
**GALK2**	-0.88	5.79E-20	8.64E-19
**IVL**	2.41	5.81E-20	8.67E-19
**MANEA-AS1**	-2.73	5.87E-20	8.76E-19
**NEFL**	4.94	6.04E-20	9.01E-19
**COMTD1**	1.88	6.13E-20	9.14E-19
**LYNX1**	2.26	6.16E-20	9.18E-19
**ABCD3**	-1.36	6.17E-20	9.18E-19
**ATP6V0E1**	1.19	6.26E-20	9.32E-19
**SSX2IP**	-1.32	6.27E-20	9.33E-19
**SH3TC1**	2.92	6.30E-20	9.37E-19
**ZNF628**	2.06	6.45E-20	9.58E-19
**CUEDC1**	2.11	6.46E-20	9.59E-19
**TNXB**	-3.91	6.50E-20	9.64E-19
**RP11-778O17.4**	-6.85	6.61E-20	9.81E-19
**TPRXL**	2.54	6.64E-20	9.85E-19
**C16orf52**	-1.08	6.74E-20	9.99E-19
**SIVA1**	1.21	6.94E-20	1.03E-18
**CCDC22**	1.13	7.11E-20	1.05E-18
**MAP4K3**	-1.15	7.16E-20	1.06E-18
**G31356**	-3.38	7.35E-20	1.09E-18
**ROGDI**	2.31	7.88E-20	1.16E-18
**FADD**	1.39	7.90E-20	1.17E-18
**RP11-456K23.1**	2.81	7.91E-20	1.17E-18
**ZCCHC2**	-1.17	7.91E-20	1.17E-18
**MLLT1**	1.28	7.99E-20	1.18E-18
**RP11-566E18.1**	-2.02	8.01E-20	1.18E-18
**TAX1BP3**	-1.87	8.09E-20	1.19E-18
**HMCES**	1.03	8.44E-20	1.24E-18
**PGK1**	1.58	8.63E-20	1.27E-18

**SCAI**	-1.18	8.67E-20	1.28E-18
**GHDC**	2.22	8.83E-20	1.30E-18
**STC1**	4.37	8.83E-20	1.30E-18
**MYO7B**	-2.85	9.11E-20	1.34E-18
**SNTB2**	-1.06	9.27E-20	1.36E-18
**TMEM119**	3.86	9.48E-20	1.39E-18
**TCIRG1**	2.56	9.67E-20	1.42E-18
**CD81**	1.24	9.69E-20	1.42E-18
**ZBTB42**	1.55	9.94E-20	1.46E-18
**PPWD1**	-0.78	1.00E-19	1.47E-18
**TMEM115**	0.81	1.01E-19	1.48E-18
**DRAP1**	1.59	1.01E-19	1.48E-18
**ZNF675**	-1.73	1.02E-19	1.49E-18
**RP11-832A4.7**	3.97	1.03E-19	1.50E-18
**CHRNG**	-3.62	1.04E-19	1.52E-18
**FAM83B**	-1.60	1.05E-19	1.54E-18
**GSTK1**	0.78	1.06E-19	1.55E-18
**HYMAI**	-4.91	1.07E-19	1.56E-18
**NOC3L**	-1.38	1.07E-19	1.56E-18
**DCBLD1**	1.64	1.08E-19	1.58E-18
**FAM84A**	1.66	1.09E-19	1.59E-18
**HOXB7**	3.44	1.09E-19	1.59E-18
**SGPL1**	-1.10	1.10E-19	1.60E-18
**TRIOBP**	1.36	1.10E-19	1.61E-18
**PTPN3**	-1.63	1.11E-19	1.61E-18
**MRVI1**	2.25	1.11E-19	1.62E-18
**MPHOSPH8**	0.99	1.12E-19	1.63E-18
**AC006262.4**	-2.87	1.12E-19	1.64E-18
**CNFN**	2.27	1.13E-19	1.64E-18
**XLOC_008878**	5.81	1.17E-19	1.69E-18
**ITIH3**	4.31	1.19E-19	1.73E-18
**FTL**	2.59	1.22E-19	1.77E-18
**STAT6**	0.89	1.23E-19	1.79E-18
**AGAP3**	1.01	1.26E-19	1.82E-18
**FAAP100**	1.32	1.27E-19	1.85E-18
**XLOC_007801**	-1.89	1.30E-19	1.88E-18
**KDM6A**	-0.87	1.33E-19	1.93E-18
**SERTAD3**	1.19	1.33E-19	1.93E-18
**CLASRP**	1.84	1.36E-19	1.96E-18
**RP11-665G4.1**	-3.51	1.37E-19	1.98E-18
**LGALS9**	2.76	1.39E-19	2.01E-18
**FBXL12**	1.08	1.41E-19	2.04E-18
**ZNF407**	-1.10	1.42E-19	2.06E-18
**PDPN**	2.94	1.43E-19	2.06E-18
**BOD1**	0.91	1.44E-19	2.08E-18

**RP11-447D11.3**	-1.88	1.45E-19	2.09E-18
**TNFAIP8L1**	2.36	1.48E-19	2.14E-18
**LRRC32**	4.00	1.54E-19	2.22E-18
**CIZ1**	0.89	1.54E-19	2.23E-18
**APPL1**	-1.28	1.56E-19	2.25E-18
**CCDC102B**	3.77	1.62E-19	2.33E-18
**INADL**	-1.49	1.66E-19	2.39E-18
**NNMT**	4.20	1.72E-19	2.48E-18
**TBC1D2**	1.10	1.75E-19	2.51E-18
**COX4I2**	5.31	1.76E-19	2.52E-18
**LMTK2**	-1.11	1.76E-19	2.53E-18
**LIG3**	-1.11	1.78E-19	2.55E-18
**ADCK5**	1.68	1.80E-19	2.58E-18
**G3BP1**	-1.26	1.83E-19	2.62E-18
**ATF7IP**	-1.24	1.84E-19	2.64E-18
**B3GALNT2**	-1.21	1.86E-19	2.66E-18
**PEA15**	1.08	1.87E-19	2.67E-18
**AC000123.2**	-2.48	1.88E-19	2.69E-18
**AC020951.1**	-3.69	1.90E-19	2.72E-18
**CSGALNACT1**	2.51	1.92E-19	2.75E-18
**CLDN1**	-1.90	1.95E-19	2.79E-18
**GTF2A1**	-1.21	2.00E-19	2.86E-18
**REL**	-1.28	2.05E-19	2.92E-18
**PLXND1**	2.92	2.07E-19	2.96E-18
**PLK2**	1.20	2.09E-19	2.98E-18
**ELP6**	1.27	2.10E-19	2.99E-18
**COL5A3**	1.98	2.11E-19	3.01E-18
**KCNA2**	-3.03	2.12E-19	3.02E-18
**STK25**	1.04	2.16E-19	3.08E-18
**NAPEPLD**	-1.18	2.20E-19	3.13E-18
**CDK4**	1.18	2.22E-19	3.16E-18
**ZNF148**	-1.25	2.23E-19	3.17E-18
**LINC00630**	-1.87	2.23E-19	3.17E-18
**RP11-102L12.2**	-4.55	2.26E-19	3.21E-18
**NMI**	1.26	2.35E-19	3.33E-18
**SNX30**	-0.94	2.36E-19	3.36E-18
**GUK1**	1.06	2.43E-19	3.45E-18
**WAPL**	-1.19	2.49E-19	3.54E-18
**PTTG1IP**	1.19	2.58E-19	3.66E-18
**LINC01235**	4.87	2.62E-19	3.71E-18
**TXNRD2**	1.64	2.68E-19	3.80E-18
**C14orf132**	-2.07	2.73E-19	3.87E-18
**ATP6V0D1**	1.15	2.84E-19	4.02E-18
**CLSTN1**	1.07	2.87E-19	4.07E-18
**P3H3**	2.91	2.89E-19	4.08E-18

**NEU1**	1.20	2.89E-19	4.09E-18
**AHR**	-1.03	2.91E-19	4.12E-18
**ZDHHC8**	1.62	2.95E-19	4.16E-18
**ATAD2**	-1.21	2.97E-19	4.19E-18
**KIF1B**	-1.16	3.02E-19	4.26E-18
**NSD1**	-0.81	3.03E-19	4.28E-18
**9-Sep**	1.13	3.07E-19	4.33E-18
**SLC43A2**	2.85	3.08E-19	4.33E-18
**RP11-178H8.7**	-4.07	3.12E-19	4.40E-18
**RAB34**	1.70	3.13E-19	4.41E-18
**ELK1**	1.02	3.16E-19	4.45E-18
**FBRSL1**	1.83	3.25E-19	4.57E-18
**HCFC2**	-1.00	3.25E-19	4.57E-18
**SPCS2**	1.13	3.25E-19	4.57E-18
**RP5-1112D6.7**	-3.68	3.40E-19	4.78E-18
**C20orf27**	1.60	3.42E-19	4.80E-18
**IFI35**	2.03	3.44E-19	4.83E-18
**CMAHP**	-2.05	3.51E-19	4.92E-18
**RP11-872J21.5**	-4.79	3.61E-19	5.07E-18
**XLOC_012579**	-2.21	3.71E-19	5.19E-18
**DTX3**	2.02	3.79E-19	5.32E-18
**ZNF865**	2.54	3.80E-19	5.33E-18
**ST3GAL6**	-1.37	3.81E-19	5.33E-18
**TP53I11**	2.39	3.84E-19	5.37E-18
**MAP1S**	2.08	3.97E-19	5.55E-18
**SURF4**	1.13	3.97E-19	5.55E-18
**FIZ1**	1.17	3.98E-19	5.56E-18
**RP6-109B7.2**	-4.85	3.98E-19	5.56E-18
**TBC1D32**	-1.80	4.01E-19	5.60E-18
**ATG4B**	0.69	4.02E-19	5.62E-18
**F3**	-1.91	4.03E-19	5.63E-18
**CBX8**	1.59	4.20E-19	5.85E-18
**PEX1**	-1.15	4.23E-19	5.89E-18
**SP1**	-0.89	4.24E-19	5.90E-18
**RNF167**	1.10	4.24E-19	5.90E-18
**TRAF2**	1.27	4.26E-19	5.93E-18
**CD164L2**	3.12	4.40E-19	6.12E-18
**ASCC2**	0.87	4.43E-19	6.17E-18
**DPP3**	1.11	4.57E-19	6.36E-18
**AC072062.3**	-4.37	4.61E-19	6.40E-18
**ELP5**	1.17	4.69E-19	6.51E-18
**LYPD3**	1.81	4.70E-19	6.52E-18
**MAMDC2-AS1**	-4.64	4.70E-19	6.53E-18
**CKAP4**	1.43	4.73E-19	6.56E-18
**GNA13**	-1.09	4.75E-19	6.59E-18

**GORASP1**	0.83	4.76E-19	6.60E-18
**CMTM3**	2.64	4.77E-19	6.60E-18
**NOC4L**	1.60	4.89E-19	6.77E-18
**STC2**	5.16	4.93E-19	6.83E-18
**TMED8**	-0.78	4.95E-19	6.85E-18
**DNAL4**	1.20	5.01E-19	6.92E-18
**MAFF**	1.22	5.08E-19	7.02E-18
**SNORA80**	-10.45	5.24E-19	7.24E-18
**NUDT8**	1.90	5.30E-19	7.32E-18
**TREX2**	2.65	5.32E-19	7.35E-18
**FTX**	-2.97	5.33E-19	7.35E-18
**LAMB1**	1.77	5.41E-19	7.46E-18
**ESCO1**	-0.94	5.44E-19	7.49E-18
**SGK223**	1.82	5.52E-19	7.61E-18
**CEP131**	1.63	5.55E-19	7.64E-18
**TEKT4P2**	4.60	5.64E-19	7.76E-18
**ALPL**	4.53	5.66E-19	7.79E-18
**TMEM154**	-1.79	5.69E-19	7.83E-18
**ID3**	2.00	5.71E-19	7.86E-18
**SNAPC2**	1.67	5.74E-19	7.89E-18
**MIDN**	1.87	5.77E-19	7.93E-18
**IRAK1**	1.43	5.79E-19	7.95E-18
**EPB41L5**	-0.90	5.91E-19	8.11E-18
**UCHL3**	-1.66	5.93E-19	8.13E-18
**PLCD1**	1.11	6.03E-19	8.26E-18
**PATZ1**	1.47	6.09E-19	8.35E-18
**PTPN18**	1.73	6.25E-19	8.57E-18
**FAIM2**	6.19	6.26E-19	8.58E-18
**LINC01126**	-3.74	6.31E-19	8.64E-18
**ORC4**	-1.01	6.39E-19	8.74E-18
**SHFM1**	1.42	6.45E-19	8.82E-18
**CFLAR-AS1**	-2.83	6.49E-19	8.87E-18
**KLF7**	1.80	6.54E-19	8.94E-18
**AC009120.6**	-2.50	6.70E-19	9.15E-18
**C1orf123**	1.31	6.75E-19	9.21E-18
**STX17**	-1.11	6.77E-19	9.24E-18
**SAC3D1**	1.48	6.88E-19	9.38E-18
**SRP68**	0.96	7.11E-19	9.69E-18
**ADGRF2**	-2.02	7.48E-19	1.02E-17
**CD276**	2.11	7.73E-19	1.05E-17
**TMEM255B**	3.23	7.76E-19	1.06E-17
**SLC39A3**	1.51	7.92E-19	1.08E-17
**SLC6A8**	1.65	7.97E-19	1.08E-17
**C3orf38**	-0.99	8.34E-19	1.13E-17
**NCDN**	1.55	8.54E-19	1.16E-17

**C4orf48**	3.32	8.64E-19	1.17E-17
**METTL25**	-1.30	8.67E-19	1.18E-17
**POLK**	-1.16	8.76E-19	1.19E-17
**KLF6**	1.10	8.81E-19	1.20E-17
**MRPS21**	1.13	8.83E-19	1.20E-17
**STXBP4**	-1.41	8.86E-19	1.20E-17
**PCBP4**	1.20	9.07E-19	1.23E-17
**GAA**	2.13	9.47E-19	1.28E-17
**RNU5E-1**	-10.30	9.61E-19	1.30E-17
**SNHG25**	-4.67	9.65E-19	1.31E-17
**SIRT7**	1.44	9.69E-19	1.31E-17
**EPT1**	-1.52	9.78E-19	1.32E-17
**ZNF626**	-2.17	9.80E-19	1.33E-17
**SMIM13**	-1.15	9.88E-19	1.33E-17
**PLA2G4D**	2.66	1.01E-18	1.36E-17
**UPF1**	1.01	1.03E-18	1.39E-17
**CHPF2**	1.45	1.06E-18	1.42E-17
**SPARCL1**	2.91	1.07E-18	1.45E-17
**IFITM1**	4.03	1.08E-18	1.45E-17
**USHBP1**	2.93	1.08E-18	1.46E-17
**USF1**	1.02	1.09E-18	1.47E-17
**IFRD2**	1.68	1.10E-18	1.48E-17
**USH2A**	-4.28	1.10E-18	1.48E-17
**EEFSEC**	1.38	1.13E-18	1.52E-17
**TRIP12**	-0.86	1.16E-18	1.56E-17
**C3orf49**	-3.54	1.16E-18	1.56E-17
**GPS1**	0.96	1.16E-18	1.57E-17
**EIF2AK3**	-0.87	1.18E-18	1.59E-17
**PDCD7**	0.82	1.19E-18	1.60E-17
**FBXL6**	1.76	1.20E-18	1.62E-17
**JAK3**	2.84	1.21E-18	1.63E-17
**SNN**	1.48	1.23E-18	1.65E-17
**MTG2**	1.10	1.23E-18	1.65E-17
**DTYMK**	1.24	1.23E-18	1.65E-17
**FHL5**	4.19	1.27E-18	1.70E-17
**GPX2**	3.10	1.27E-18	1.70E-17
**SLC38A7**	1.74	1.28E-18	1.71E-17
**CXXC5**	1.23	1.28E-18	1.72E-17
**ARF5**	1.17	1.39E-18	1.85E-17
**LILRB2**	4.59	1.40E-18	1.87E-17
**RP9**	1.13	1.41E-18	1.88E-17
**LGI3**	-2.88	1.41E-18	1.88E-17
**ZNF836**	-1.02	1.43E-18	1.91E-17
**APLF**	-1.62	1.44E-18	1.92E-17
**EIF5A**	1.47	1.44E-18	1.93E-17

**STXBP6**	-1.98	1.47E-18	1.96E-17
**FEM1B**	-1.19	1.48E-18	1.97E-17
**DMWD**	1.24	1.52E-18	2.03E-17
**CAPN7**	-0.81	1.57E-18	2.09E-17
**SNRPA**	0.99	1.58E-18	2.11E-17
**MARK4**	1.31	1.59E-18	2.12E-17
**PANK1**	-1.55	1.59E-18	2.12E-17
**CAMTA1**	1.07	1.61E-18	2.14E-17
**ETFB**	1.34	1.66E-18	2.21E-17
**SETD2**	-0.92	1.67E-18	2.22E-17
**PRR5**	1.70	1.69E-18	2.25E-17
**REEP4**	1.35	1.73E-18	2.29E-17
**TNFSF10**	1.51	1.73E-18	2.30E-17
**FAM13A-AS1**	-3.04	1.73E-18	2.30E-17
**RP1-140J1.1**	-4.23	1.75E-18	2.32E-17
**LATS1**	-0.89	1.79E-18	2.38E-17
**CTB-152G17.6**	-2.99	1.83E-18	2.43E-17
**FCGBP**	-2.21	1.87E-18	2.48E-17
**ZNF212**	1.10	1.89E-18	2.50E-17
**RSAD1**	1.07	1.91E-18	2.53E-17
**WNT2B**	-2.22	1.92E-18	2.54E-17
**RNF122**	1.69	1.94E-18	2.56E-17
**AF131216.6**	-4.07	1.96E-18	2.59E-17
**DIP2B**	-1.12	1.97E-18	2.60E-17
**EPS8**	1.59	2.04E-18	2.69E-17
**GIMAP1**	2.75	2.08E-18	2.74E-17
**SNX27**	-1.02	2.09E-18	2.76E-17
**SPOPL**	-0.98	2.13E-18	2.81E-17
**DVL1**	1.05	2.13E-18	2.81E-17
**ADGRL4**	3.18	2.17E-18	2.86E-17
**APOL2**	1.79	2.20E-18	2.89E-17
**UNC45A**	0.94	2.20E-18	2.90E-17
**ADCY4**	2.41	2.22E-18	2.92E-17
**TXNDC5**	3.01	2.36E-18	3.11E-17
**TAGLN2**	1.50	2.37E-18	3.12E-17
**RP4-742J24.2**	-4.89	2.39E-18	3.14E-17
**WDR18**	1.51	2.39E-18	3.14E-17
**YWHAE**	1.05	2.41E-18	3.17E-17
**LAMB3**	1.40	2.43E-18	3.19E-17
**MPG**	1.59	2.43E-18	3.19E-17
**CTC-321K16.1**	-3.28	2.44E-18	3.20E-17
**ZFAND5**	0.87	2.47E-18	3.24E-17
**EMC4**	1.18	2.49E-18	3.26E-17
**DNAJC16**	-0.84	2.53E-18	3.32E-17
**PCED1A**	1.85	2.55E-18	3.34E-17

**FBXW7**	-1.29	2.63E-18	3.45E-17
**AADACL3**	-12.37	2.69E-18	3.52E-17
**TUBA1C**	1.78	2.70E-18	3.53E-17
**CTB-36H16.2**	-1.83	2.86E-18	3.74E-17
**PRPS1**	1.26	2.91E-18	3.80E-17
**CDH22**	-2.87	2.91E-18	3.80E-17
**DDX39A**	1.36	2.93E-18	3.83E-17
**OST4**	1.13	2.99E-18	3.90E-17
**PGD**	1.40	2.99E-18	3.91E-17
**STMN3**	2.02	3.00E-18	3.92E-17
**FRAS1**	-2.84	3.01E-18	3.93E-17
**UBB**	1.57	3.04E-18	3.96E-17
**PDE1A**	4.07	3.04E-18	3.96E-17
**DPCD**	1.37	3.16E-18	4.11E-17
**GPR162**	3.34	3.18E-18	4.14E-17
**TRIM47**	2.00	3.19E-18	4.15E-17
**SELO**	1.57	3.23E-18	4.20E-17
**MT-ND2**	5.12	3.24E-18	4.22E-17
**PLCG1**	1.01	3.29E-18	4.27E-17
**NUDT9**	0.96	3.46E-18	4.50E-17
**DDX25**	-3.41	3.57E-18	4.63E-17
**SPON2**	2.06	3.73E-18	4.84E-17
**MRPL37**	0.94	3.73E-18	4.84E-17
**GANAB**	0.91	3.79E-18	4.91E-17
**ARL4D**	2.26	3.80E-18	4.92E-17
**UGGT1**	-0.92	3.82E-18	4.94E-17
**KLC4**	1.02	3.87E-18	5.01E-17
**DOHH**	1.44	3.94E-18	5.10E-17
**ANKRD29**	2.68	3.94E-18	5.10E-17
**PARP10**	1.59	4.00E-18	5.18E-17
**RSBN1**	-1.03	4.04E-18	5.22E-17
**CTDNEP1**	1.78	4.10E-18	5.29E-17
**SNX8**	1.52	4.28E-18	5.53E-17
**KPNA5**	-1.18	4.41E-18	5.69E-17
**PLAG1**	-2.14	4.52E-18	5.83E-17
**CTD-2267D19.3**	1.53	4.54E-18	5.86E-17
**PPP6R1**	1.18	4.63E-18	5.97E-17
**FOXS1**	5.53	4.67E-18	6.02E-17
**PPM1A**	-1.03	4.67E-18	6.02E-17
**BNC1**	1.52	4.74E-18	6.10E-17
**UBAC2**	0.88	4.77E-18	6.14E-17
**SURF1**	1.20	4.78E-18	6.15E-17
**TCEB2**	1.39	4.88E-18	6.28E-17
**CHD1**	-1.02	4.98E-18	6.41E-17
**DEDD2**	1.09	5.01E-18	6.44E-17

**SFMBT1**	-1.02	5.03E-18	6.47E-17
**NEMP2**	-1.79	5.09E-18	6.54E-17
**CYHR1**	1.27	5.14E-18	6.59E-17
**MAGED2**	1.33	5.26E-18	6.75E-17
**SNORA14B**	-6.51	5.29E-18	6.78E-17
**INA**	3.05	5.32E-18	6.82E-17
**ALDH7A1**	1.14	5.40E-18	6.92E-17
**LINC01355**	-3.27	5.46E-18	7.00E-17
**RUNX2**	2.60	5.50E-18	7.04E-17
**CTD-2651B20.6**	-9.47	5.52E-18	7.07E-17
**SAR1B**	-1.19	5.53E-18	7.08E-17
**SMDT1**	1.52	5.61E-18	7.18E-17
**RP11-4K16.2**	-4.02	5.65E-18	7.23E-17
**TCHHL1**	-22.12	5.70E-18	7.28E-17
**ZDHHC24**	1.01	5.70E-18	7.28E-17
**ZNF551**	-1.27	5.80E-18	7.41E-17
**HRH2**	4.26	5.80E-18	7.41E-17
**RASGRP2**	3.45	5.93E-18	7.56E-17
**BCL6B**	3.20	5.96E-18	7.61E-17
**LOXL1**	3.25	5.97E-18	7.61E-17
**TMEM168**	-1.05	6.03E-18	7.68E-17
**ERCC8**	-1.08	6.15E-18	7.84E-17
**GSTP1**	1.47	6.28E-18	8.00E-17
**YKT6**	1.10	6.33E-18	8.06E-17
**SLC41A3**	0.93	6.43E-18	8.18E-17
**RP4-530I15.9**	-3.92	6.44E-18	8.19E-17
**COCH**	-4.72	6.45E-18	8.20E-17
**TRIM33**	-1.06	6.58E-18	8.36E-17
**SLC39A4**	1.79	6.62E-18	8.41E-17
**DTNBP1**	1.85	6.68E-18	8.49E-17
**FRMD8**	1.36	6.69E-18	8.49E-17
**ATP11B**	-1.22	6.78E-18	8.61E-17
**CRELD1**	1.70	6.83E-18	8.66E-17
**SRGAP2B**	-1.32	6.85E-18	8.69E-17
**KHDRBS3**	2.74	6.89E-18	8.74E-17
**SLC2A5**	3.36	6.99E-18	8.86E-17
**SNHG18**	1.65	7.07E-18	8.95E-17
**GUF1**	-0.93	7.18E-18	9.09E-17
**GLUL**	1.61	7.25E-18	9.17E-17
**OLFML2B**	4.06	7.26E-18	9.19E-17
**HOXC9**	1.75	7.37E-18	9.32E-17
**TMEM109**	1.15	7.40E-18	9.35E-17
**ZNF496**	1.21	7.49E-18	9.47E-17
**RTN2**	1.72	7.50E-18	9.47E-17
**RBL1**	-1.37	7.61E-18	9.60E-17

**STK16**	1.11	7.72E-18	9.75E-17
**FLT4**	3.43	7.77E-18	9.80E-17
**TMSB4X**	1.87	7.82E-18	9.86E-17
**ELK3**	1.74	7.82E-18	9.86E-17
**EMC10**	1.13	7.86E-18	9.90E-17
**IFFO1**	2.57	7.93E-18	9.99E-17
**RP11-768B22.2**	-5.83	7.95E-18	1.00E-16
**HAGH**	1.01	7.96E-18	1.00E-16
**APBA3**	1.52	7.97E-18	1.00E-16
**STAT3**	0.92	7.99E-18	1.01E-16
**PURB**	-1.08	8.16E-18	1.03E-16
**PPIH**	0.99	8.21E-18	1.03E-16
**CTNNBL1**	0.82	8.23E-18	1.03E-16
**FAM65A**	2.48	8.29E-18	1.04E-16
**CPTP**	1.27	8.53E-18	1.07E-16
**ZNF440**	-1.54	8.63E-18	1.08E-16
**ZNF280C**	-1.46	8.98E-18	1.13E-16
**STK19**	1.01	9.03E-18	1.13E-16
**COL8A1**	4.19	9.05E-18	1.13E-16
**RP11-544M22.8**	-6.03	9.24E-18	1.16E-16
**TRIM26**	0.84	9.27E-18	1.16E-16
**RP11-55K13.1**	-4.77	9.29E-18	1.16E-16
**RAPGEF4**	-1.69	9.40E-18	1.18E-16
**CHCHD10**	3.00	9.59E-18	1.20E-16
**C3orf35**	-2.76	9.72E-18	1.21E-16
**HOXA11-AS**	4.09	9.81E-18	1.23E-16
**ZNF549**	-1.10	9.93E-18	1.24E-16
**G21027**	-4.22	1.00E-17	1.25E-16
**GIMAP4**	2.66	1.00E-17	1.25E-16
**COL6A1**	3.31	1.01E-17	1.26E-16
**ZNF596**	-1.26	1.01E-17	1.27E-16
**RAD54B**	-2.08	1.02E-17	1.27E-16
**KLRF2**	-4.16	1.04E-17	1.29E-16
**USP12**	-1.07	1.05E-17	1.31E-16
**SOS2**	-0.78	1.05E-17	1.31E-16
**MAP7D3**	1.26	1.06E-17	1.32E-16
**TUBG2**	2.16	1.07E-17	1.33E-16
**AC139100.3**	-4.08	1.07E-17	1.33E-16
**PYGL**	1.77	1.08E-17	1.34E-16
**EIF3G**	1.05	1.08E-17	1.34E-16
**GFER**	1.71	1.08E-17	1.34E-16
**ERVFRD-1**	-4.21	1.08E-17	1.34E-16
**ERRFI1**	1.41	1.09E-17	1.36E-16
**FUS**	2.22	1.12E-17	1.39E-16
**RP5-1085F17.3**	-1.39	1.13E-17	1.40E-16

**MTCL1**	2.24	1.19E-17	1.47E-16
**SNX18P3**	-4.22	1.20E-17	1.49E-16
**BFSP1**	2.08	1.22E-17	1.51E-16
**ZNF234**	-1.20	1.22E-17	1.52E-16
**RP11-70L8.4**	-3.55	1.23E-17	1.52E-16
**AAAS**	1.09	1.23E-17	1.53E-16
**NUDT3**	-0.78	1.25E-17	1.55E-16
**PMM1**	1.48	1.28E-17	1.59E-16
**QTRT1**	1.62	1.30E-17	1.60E-16
**MPV17**	1.32	1.30E-17	1.60E-16
**CLOCK**	-1.07	1.30E-17	1.61E-16
**POLRMT**	1.13	1.32E-17	1.63E-16
**MT-ATP6**	3.79	1.32E-17	1.63E-16
**ARMC8**	-0.67	1.34E-17	1.65E-16
**FIS1**	1.17	1.34E-17	1.66E-16
**C10orf54**	1.38	1.35E-17	1.67E-16
**RP11-286N22.8**	-3.90	1.37E-17	1.68E-16
**CIR1**	1.26	1.37E-17	1.69E-16
**ORC2**	-0.70	1.43E-17	1.76E-16
**IDH3G**	0.81	1.45E-17	1.79E-16
**RNF121**	0.81	1.49E-17	1.83E-16
**C11orf95**	1.86	1.49E-17	1.83E-16
**STXBP1**	1.57	1.50E-17	1.84E-16
**SDF2L1**	2.31	1.51E-17	1.86E-16
**PLBD2**	1.88	1.54E-17	1.89E-16
**TMEM187**	1.28	1.54E-17	1.90E-16
**LONRF1**	-1.91	1.55E-17	1.90E-16
**AQR**	-0.89	1.58E-17	1.94E-16
**FANCD2**	-1.25	1.62E-17	1.98E-16
**FMR1**	-0.87	1.63E-17	1.99E-16
**BBIP1**	-1.58	1.63E-17	2.00E-16
**C16orf13**	1.21	1.64E-17	2.01E-16
**PRPF6**	0.89	1.67E-17	2.04E-16
**RP11-849H4.2**	-2.25	1.68E-17	2.05E-16
**PPIB**	1.65	1.70E-17	2.08E-16
**VWF**	3.23	1.73E-17	2.11E-16
**SDHAF1**	1.27	1.75E-17	2.14E-16
**THOP1**	1.08	1.76E-17	2.15E-16
**ZNF674**	-1.07	1.76E-17	2.15E-16
**TXNDC17**	1.55	1.82E-17	2.22E-16
**C16orf72**	-0.68	1.83E-17	2.23E-16
**GPR75**	-2.39	1.84E-17	2.25E-16
**TNFRSF4**	5.40	1.90E-17	2.32E-16
**PFKFB3**	1.49	1.91E-17	2.33E-16
**PLCE1**	-1.29	1.91E-17	2.33E-16

**HRC**	3.50	1.92E-17	2.34E-16
**CTD-2017D11.1**	-4.06	1.93E-17	2.35E-16
**BTAF1**	-1.52	2.05E-17	2.49E-16
**CNGB1**	7.89	2.05E-17	2.50E-16
**LDLRAP1**	0.99	2.12E-17	2.58E-16
**NDUFA8**	1.37	2.18E-17	2.65E-16
**MRPL4**	1.29	2.18E-17	2.66E-16
**NFXL1**	-1.08	2.23E-17	2.71E-16
**NDUFS3**	0.96	2.23E-17	2.71E-16
**TMEM165**	1.37	2.23E-17	2.71E-16
**RAB29**	1.75	2.27E-17	2.75E-16
**TTBK2**	-1.03	2.29E-17	2.78E-16
**SLC15A3**	3.18	2.30E-17	2.79E-16
**ZCCHC17**	1.06	2.32E-17	2.81E-16
**CD55**	-1.13	2.32E-17	2.81E-16
**MAEA**	0.80	2.34E-17	2.84E-16
**MT-ND1**	4.86	2.44E-17	2.95E-16
**ANAPC4**	-0.90	2.44E-17	2.95E-16
**TAPT1**	-0.76	2.49E-17	3.01E-16
**MET**	-1.56	2.50E-17	3.02E-16
**PHLPP2**	-1.17	2.52E-17	3.05E-16
**WDR46**	0.93	2.53E-17	3.06E-16
**ZNF571-AS1**	-2.31	2.76E-17	3.34E-16
**ANGPTL2**	3.23	2.78E-17	3.35E-16
**SAMD4B**	1.37	2.90E-17	3.50E-16
**TNC**	3.40	2.92E-17	3.52E-16
**UNC13C**	-3.68	2.92E-17	3.52E-16
**RFESD**	-2.30	2.93E-17	3.53E-16
**ACER2**	-1.78	3.02E-17	3.64E-16
**AMDHD2**	1.48	3.02E-17	3.64E-16
**XRCC1**	1.12	3.02E-17	3.64E-16
**DUS1L**	1.41	3.05E-17	3.67E-16
**BMP2**	-1.54	3.07E-17	3.69E-16
**FSIP2**	-4.25	3.08E-17	3.71E-16
**RP11-264I13.2**	-1.71	3.14E-17	3.78E-16
**AMER1**	-1.06	3.21E-17	3.86E-16
**EVI5L**	1.48	3.27E-17	3.93E-16
**MAGOH**	1.39	3.27E-17	3.93E-16
**KRR1**	-1.03	3.28E-17	3.94E-16
**ZNF513**	1.37	3.30E-17	3.96E-16
**TBC1D5**	-0.81	3.34E-17	4.01E-16
**SMAD6**	2.24	3.39E-17	4.07E-16
**PSME3**	1.07	3.41E-17	4.09E-16
**DYRK1B**	1.35	3.43E-17	4.11E-16
**CCDC71L**	2.37	3.45E-17	4.13E-16

**RIMKLBP2**	-5.12	3.50E-17	4.20E-16
**SERTAD1**	1.48	3.56E-17	4.27E-16
**ZDHHC1**	1.64	3.63E-17	4.35E-16
**SERPINE2**	3.24	3.68E-17	4.40E-16
**ADAMTS7**	4.54	3.75E-17	4.49E-16
**CNIH3**	1.91	3.87E-17	4.62E-16
**MUL1**	1.03	4.01E-17	4.80E-16
**PDZD8**	-0.94	4.04E-17	4.83E-16
**POLDIP2**	0.88	4.09E-17	4.89E-16
**DACT3**	2.88	4.10E-17	4.89E-16
**RP11-408H20.3**	-3.26	4.15E-17	4.96E-16
**AC015971.2**	-3.55	4.18E-17	4.99E-16
**LRRFIP1**	1.18	4.18E-17	4.99E-16
**CXorf40B**	1.21	4.20E-17	5.00E-16
**BPTF**	-0.92	4.20E-17	5.00E-16
**GALNT18**	1.69	4.26E-17	5.08E-16
**PELI2**	-1.79	4.32E-17	5.15E-16
**GTF2A2**	1.08	4.46E-17	5.30E-16
**KIAA1715**	-1.22	4.54E-17	5.40E-16
**RP11-571I18.5**	-6.51	4.54E-17	5.40E-16
**MECOM**	2.77	4.56E-17	5.42E-16
**FOXN3**	-1.33	4.69E-17	5.58E-16
**EYA2**	2.84	4.86E-17	5.77E-16
**PA2G4**	1.19	4.87E-17	5.78E-16
**GAS6**	1.49	4.90E-17	5.81E-16
**KLHDC8B**	1.70	4.94E-17	5.86E-16
**MT1F**	2.82	4.97E-17	5.89E-16
**TECPR1**	1.25	4.99E-17	5.91E-16
**LYN**	2.07	4.99E-17	5.91E-16
**TARDBP**	0.80	5.04E-17	5.97E-16
**CHML**	-1.47	5.06E-17	5.99E-16
**PIAS2**	-1.15	5.08E-17	6.02E-16
**ATG9A**	1.03	5.12E-17	6.05E-16
**MXRA8**	3.52	5.14E-17	6.09E-16
**CTIF**	1.09	5.17E-17	6.11E-16
**MMP9**	5.87	5.23E-17	6.18E-16
**CALR**	1.55	5.25E-17	6.20E-16
**PDE3A**	3.45	5.27E-17	6.22E-16
**LINC00842**	-2.17	5.27E-17	6.22E-16
**STOML2**	1.13	5.30E-17	6.25E-16
**CENPB**	1.33	5.31E-17	6.27E-16
**WDR81**	1.19	5.36E-17	6.32E-16
**RBPMS2**	3.59	5.38E-17	6.35E-16
**PDIA5**	2.19	5.43E-17	6.40E-16
**IER5**	1.17	5.46E-17	6.44E-16

**MAP3K4**	-0.94	5.70E-17	6.72E-16
**SLC2A3**	2.88	5.75E-17	6.76E-16
**RP11-126H7.4**	-4.61	5.75E-17	6.77E-16
**LRP2BP**	-2.17	5.76E-17	6.78E-16
**MOSPD2**	-1.38	5.78E-17	6.80E-16
**SCIN**	-2.03	5.84E-17	6.86E-16
**SDR42E1**	-1.53	5.89E-17	6.92E-16
**CTDSPL**	-0.82	5.89E-17	6.92E-16
**C19orf47**	0.95	5.97E-17	7.01E-16
**DNAJC4**	1.20	5.98E-17	7.02E-16
**RP11-380B4.3**	-4.21	6.00E-17	7.04E-16
**SENP2**	-0.75	6.15E-17	7.21E-16
**PCDH11X**	-3.52	6.18E-17	7.24E-16
**NAGS**	1.58	6.18E-17	7.25E-16
**INTS6**	-1.12	6.24E-17	7.31E-16
**DUS3L**	1.11	6.25E-17	7.32E-16
**RP11-21L23.2**	-2.44	6.30E-17	7.37E-16
**ITGB8**	-1.75	6.33E-17	7.41E-16
**MT-TY**	-5.99	6.35E-17	7.43E-16
**SLC10A1**	-6.31	6.39E-17	7.47E-16
**ATP2B4**	-0.89	6.40E-17	7.48E-16
**UBE3A**	-0.84	6.46E-17	7.55E-16
**RP11-347P5.1**	-4.54	6.92E-17	8.08E-16
**TARS2**	0.93	6.95E-17	8.11E-16
**SPINT2**	1.24	7.07E-17	8.26E-16
**LY6E**	2.27	7.23E-17	8.43E-16
**CDKN2B**	1.93	7.25E-17	8.46E-16
**PACSIN3**	1.33	7.27E-17	8.48E-16
**SCAMP3**	0.96	7.30E-17	8.51E-16
**ARHGAP22**	2.48	7.32E-17	8.53E-16
**ADRBK1**	1.00	7.35E-17	8.56E-16
**ZFYVE16**	-0.96	7.36E-17	8.57E-16
**VSNL1**	1.77	7.42E-17	8.64E-16
**SNHG22**	-4.36	7.56E-17	8.80E-16
**MIR133A1HG**	-5.34	7.58E-17	8.82E-16
**CLIP4**	-1.38	7.82E-17	9.09E-16
**BOK**	1.21	7.92E-17	9.20E-16
**KRT26**	-20.47	7.92E-17	9.20E-16
**SH3GL1**	1.33	7.94E-17	9.22E-16
**LCE3E**	5.89	7.95E-17	9.22E-16
**TBC1D25**	1.03	7.97E-17	9.25E-16
**RP11-452L6.5**	-1.49	7.98E-17	9.26E-16
**SH3RF3**	1.61	8.02E-17	9.30E-16
**CAMSAP2**	-1.06	8.04E-17	9.32E-16
**DOCK6**	1.65	8.06E-17	9.34E-16

**ZNF770**	-1.45	8.11E-17	9.40E-16
**LINC01473**	-3.16	8.13E-17	9.41E-16
**UTP15**	-1.05	8.15E-17	9.44E-16
**ATP6V1F**	1.14	8.34E-17	9.66E-16
**KIAA0232**	-0.89	8.37E-17	9.68E-16
**SAMD1**	1.20	8.48E-17	9.81E-16
**RPL35**	1.31	8.58E-17	9.91E-16
**CLEC7A**	2.52	8.69E-17	1.00E-15
**ZNF280D**	-0.97	8.70E-17	1.00E-15
**F2RL3**	4.67	8.74E-17	1.01E-15
**ADD1**	0.59	8.80E-17	1.02E-15
**ARID3A**	1.66	8.81E-17	1.02E-15
**S100P**	3.94	8.87E-17	1.02E-15
**EYA3**	-0.84	8.88E-17	1.02E-15
**TPPP**	-3.41	8.91E-17	1.03E-15
**LRRC58**	-1.12	8.93E-17	1.03E-15
**RAD23A**	1.04	9.02E-17	1.04E-15
**LINC01197**	3.57	9.44E-17	1.09E-15
**IER5L**	2.69	9.65E-17	1.11E-15
**PXN**	1.65	9.76E-17	1.12E-15
**CCL21**	3.89	9.79E-17	1.13E-15
**AKT2**	0.79	9.80E-17	1.13E-15
**MYC**	2.16	9.87E-17	1.13E-15
**NCOA3**	-0.94	9.90E-17	1.14E-15
**PCAT6**	2.70	9.94E-17	1.14E-15
**TMEM121**	2.72	9.99E-17	1.15E-15
**GPR176**	3.07	1.00E-16	1.15E-15
**RP11-49K24.8**	-5.41	1.01E-16	1.16E-15
**SLC44A2**	0.88	1.03E-16	1.18E-15
**TSSC4**	1.22	1.03E-16	1.18E-15
**AC009299.2**	-4.51	1.03E-16	1.18E-15
**DLG1**	-0.92	1.04E-16	1.19E-15
**MRPL19**	-1.09	1.04E-16	1.19E-15
**LGALS1**	2.89	1.05E-16	1.20E-15
**PTPN9**	1.05	1.06E-16	1.21E-15
**SCRIB**	1.42	1.06E-16	1.21E-15
**SNORA84**	-9.58	1.06E-16	1.21E-15
**DENND2C**	-1.42	1.07E-16	1.22E-15
**FAM109B**	1.65	1.08E-16	1.23E-15
**NDUFS2**	0.84	1.08E-16	1.23E-15
**CCDC186**	-1.48	1.12E-16	1.28E-15
**RPA1**	0.93	1.12E-16	1.28E-15
**PLXNA2**	-1.17	1.12E-16	1.28E-15
**OBFC1**	0.91	1.12E-16	1.28E-15
**ATP5S**	-0.87	1.13E-16	1.29E-15

**SRP14**	1.24	1.13E-16	1.29E-15
**AVPR2**	3.12	1.13E-16	1.29E-15
**PLAU**	2.45	1.15E-16	1.31E-15
**WIZ**	1.46	1.16E-16	1.32E-15
**HTR7**	5.00	1.18E-16	1.34E-15
**SNORA71B**	-5.63	1.19E-16	1.35E-15
**OAF**	3.17	1.19E-16	1.36E-15
**TSPO**	1.44	1.20E-16	1.36E-15
**GAS5-AS1**	-2.62	1.20E-16	1.37E-15
**TMEM255A**	-2.34	1.21E-16	1.37E-15
**NFIA**	-1.24	1.21E-16	1.37E-15
**NARFL**	1.15	1.21E-16	1.38E-15
**ZNF581**	1.05	1.24E-16	1.40E-15
**C1R**	3.11	1.26E-16	1.43E-15
**STARD13-AS**	-5.71	1.26E-16	1.43E-15
**FAM199X**	-1.08	1.27E-16	1.43E-15
**DLG4**	2.45	1.28E-16	1.45E-15
**RP11-173A16.2**	-5.41	1.28E-16	1.45E-15
**SNRNP25**	1.57	1.29E-16	1.46E-15
**LINC01023**	1.93	1.29E-16	1.46E-15
**TUFM**	1.00	1.31E-16	1.48E-15
**IL10RB**	0.95	1.31E-16	1.48E-15
**ZFAND2A**	1.50	1.31E-16	1.48E-15
**MEOX1**	2.42	1.36E-16	1.53E-15
**ABCF3**	0.80	1.37E-16	1.54E-15
**GPALPP1**	-0.83	1.37E-16	1.54E-15
**SGTB**	-1.24	1.37E-16	1.55E-15
**MAD1L1**	0.88	1.39E-16	1.57E-15
**TJAP1**	1.47	1.40E-16	1.58E-15
**SNHG19**	1.74	1.41E-16	1.59E-15
**NUDT1**	1.36	1.46E-16	1.64E-15
**RNF145**	1.16	1.46E-16	1.65E-15
**STK4**	-0.81	1.46E-16	1.65E-15
**RP11-109J4.1**	3.99	1.46E-16	1.65E-15
**PCMTD1**	-1.19	1.48E-16	1.67E-15
**BACH1-IT1**	-2.84	1.49E-16	1.67E-15
**TMEM74B**	2.81	1.49E-16	1.67E-15
**LRIG3**	-0.77	1.50E-16	1.69E-15
**ATN1**	2.85	1.53E-16	1.72E-15
**PERM1**	2.21	1.53E-16	1.72E-15
**TAF6**	1.17	1.55E-16	1.75E-15
**JHDM1D-AS1**	1.57	1.59E-16	1.79E-15
**MB21D1**	2.02	1.61E-16	1.81E-15
**RP11-840I19.3**	-3.76	1.61E-16	1.81E-15
**EPN2-AS1**	-4.52	1.62E-16	1.81E-15

**AC083884.8**	-4.90	1.62E-16	1.82E-15
**RP5-1158E12.3**	-3.79	1.63E-16	1.83E-15
**AP1S1**	1.54	1.64E-16	1.83E-15
**SNORA28**	-8.70	1.64E-16	1.84E-15
**LMAN2**	1.07	1.65E-16	1.85E-15
**PLD3**	1.52	1.66E-16	1.86E-15
**RP3-368A4.6**	-3.30	1.69E-16	1.89E-15
**MGAT4B**	0.85	1.71E-16	1.91E-15
**CDIPT**	0.92	1.72E-16	1.93E-15
**ZNF66**	-1.78	1.73E-16	1.93E-15
**B3GALT2**	-3.93	1.73E-16	1.94E-15
**GMPS**	-0.81	1.74E-16	1.94E-15
**ABTB1**	2.17	1.78E-16	1.99E-15
**GLTPD2**	1.93	1.79E-16	2.00E-15
**RPS15**	1.27	1.80E-16	2.01E-15
**C20orf96**	2.66	1.82E-16	2.03E-15
**FAM98C**	1.29	1.82E-16	2.03E-15
**DDX60**	-2.09	1.83E-16	2.05E-15
**SASS6**	-1.17	1.86E-16	2.08E-15
**AK1**	2.24	1.88E-16	2.09E-15
**RALGAPA1P**	-2.32	1.89E-16	2.11E-15
**RP4-724E16.2**	-3.01	1.90E-16	2.12E-15
**PAQR3**	-1.67	1.92E-16	2.14E-15
**NRIP3**	2.26	1.93E-16	2.15E-15
**TAF1**	-0.78	1.94E-16	2.15E-15
**ACVR1**	1.22	1.94E-16	2.16E-15
**TRPT1**	1.10	1.94E-16	2.16E-15
**GALNT6**	2.41	1.95E-16	2.16E-15
**UHRF1BP1L**	-1.05	1.96E-16	2.17E-15
**BCYRN1**	3.48	1.96E-16	2.17E-15
**POU2F2**	3.46	1.99E-16	2.21E-15
**PNPLA2**	1.80	1.99E-16	2.21E-15
**SETDB2**	-1.05	2.00E-16	2.21E-15
**CLPTM1**	0.84	2.05E-16	2.27E-15
**RPL14**	1.19	2.05E-16	2.28E-15
**MORC3**	-1.02	2.07E-16	2.29E-15
**ALDH4A1**	1.60	2.11E-16	2.34E-15
**ERAP1**	-1.22	2.12E-16	2.34E-15
**C1S**	3.35	2.13E-16	2.36E-15
**KYNU**	2.79	2.13E-16	2.36E-15
**ITGA3**	1.12	2.14E-16	2.37E-15
**MIER3**	-1.04	2.17E-16	2.40E-15
**ZNF410**	-2.80	2.22E-16	2.45E-15
**ABCD1**	1.75	2.25E-16	2.49E-15
**ZBTB24**	-1.05	2.26E-16	2.49E-15

**NFKB2**	1.61	2.29E-16	2.53E-15
**ATP13A1**	1.15	2.33E-16	2.57E-15
**PAFAH1B3**	1.34	2.34E-16	2.58E-15
**RAB1A**	1.08	2.36E-16	2.60E-15
**ACP2**	1.18	2.36E-16	2.61E-15
**SLC16A10**	-2.27	2.36E-16	2.61E-15
**HECTD3**	1.04	2.37E-16	2.61E-15
**RTTN**	-1.33	2.39E-16	2.63E-15
**IFNGR2**	0.91	2.40E-16	2.64E-15
**NUP133**	-0.86	2.44E-16	2.68E-15
**SLC35D2**	1.11	2.48E-16	2.73E-15
**SIPA1L1**	-0.73	2.56E-16	2.81E-15
**LRRC4B**	3.02	2.59E-16	2.85E-15
**DNPEP**	1.05	2.60E-16	2.86E-15
**FBXL20**	-1.19	2.62E-16	2.88E-15
**DLX5**	1.66	2.66E-16	2.92E-15
**SCAMP5**	3.72	2.68E-16	2.94E-15
**ARHGAP1**	1.27	2.75E-16	3.02E-15
**RP4-714D9.5**	-2.49	2.75E-16	3.02E-15
**SYT9**	-4.55	2.75E-16	3.02E-15
**ASNA1**	1.23	2.79E-16	3.06E-15
**PSAP**	1.38	2.80E-16	3.07E-15
**IPMK**	-1.69	2.84E-16	3.11E-15
**LHX6**	3.08	2.87E-16	3.14E-15
**NDUFB9**	1.00	2.92E-16	3.20E-15
**RPS6KA4**	1.29	2.96E-16	3.24E-15
**CSNK1E**	1.09	3.00E-16	3.28E-15
**WRAP53**	1.36	3.00E-16	3.28E-15
**FLI1**	2.21	3.02E-16	3.30E-15
**VLDLR**	1.63	3.03E-16	3.31E-15
**ITGB1BP1**	0.86	3.05E-16	3.33E-15
**ZNF254**	-1.55	3.11E-16	3.39E-15
**MT1E**	2.46	3.11E-16	3.40E-15
**ZNF519**	-1.77	3.13E-16	3.42E-15
**SUGT1P3**	-3.03	3.13E-16	3.42E-15
**AC083843.1**	-2.38	3.14E-16	3.42E-15
**G12890**	-2.76	3.28E-16	3.58E-15
**MCC**	-1.25	3.28E-16	3.58E-15
**RAB11FIP5**	1.10	3.28E-16	3.58E-15
**NSUN5**	1.20	3.31E-16	3.61E-15
**NEB**	-2.44	3.36E-16	3.66E-15
**PRRT1**	2.12	3.40E-16	3.70E-15
**G32166**	-1.52	3.44E-16	3.74E-15
**SPCS3**	-1.19	3.47E-16	3.78E-15
**THOC6**	1.19	3.48E-16	3.78E-15

**CXorf40A**	0.87	3.50E-16	3.80E-15
**SIRPA**	1.17	3.53E-16	3.83E-15
**PDZD2**	-1.52	3.53E-16	3.83E-15
**AC069547.2**	-8.97	3.54E-16	3.84E-15
**WASH3P**	1.55	3.55E-16	3.86E-15
**CTDSP1**	0.90	3.56E-16	3.87E-15
**GDF7**	-1.40	3.62E-16	3.93E-15
**FAM50B**	1.12	3.66E-16	3.96E-15
**NTHL1**	1.44	3.70E-16	4.02E-15
**GIPC3**	3.72	3.74E-16	4.06E-15
**PORCN**	1.38	3.78E-16	4.09E-15
**NPC2**	1.37	3.78E-16	4.10E-15
**C22orf46**	1.42	3.82E-16	4.14E-15
**LIMA1**	1.14	3.86E-16	4.18E-15
**TOP2B**	-0.92	3.87E-16	4.18E-15
**SNORA38**	-9.07	3.88E-16	4.19E-15
**P4HA2**	1.11	3.90E-16	4.21E-15
**NPRL3**	1.28	3.92E-16	4.24E-15
**LILRB1**	5.31	3.96E-16	4.28E-15
**RP11-282K24.3**	-4.19	3.98E-16	4.30E-15
**LRP10**	0.96	4.05E-16	4.37E-15
**RPS6KA2**	1.34	4.18E-16	4.51E-15
**DHRS1**	1.40	4.21E-16	4.54E-15
**INPP5K**	0.96	4.23E-16	4.56E-15
**LINC01290**	-3.59	4.29E-16	4.62E-15
**MFNG**	2.63	4.36E-16	4.69E-15
**MTX3**	-1.09	4.41E-16	4.76E-15
**RP11-425M5.7**	-4.99	4.43E-16	4.77E-15
**TBX1**	2.79	4.43E-16	4.77E-15
**TMEM170A**	-0.96	4.46E-16	4.80E-15
**AC079807.2**	2.00	4.48E-16	4.82E-15
**DFNA5**	2.81	4.51E-16	4.86E-15
**ALKBH4**	1.01	4.52E-16	4.86E-15
**CPNE5**	2.83	4.53E-16	4.86E-15
**EGFR**	-1.15	4.53E-16	4.87E-15
**APOL3**	2.41	4.62E-16	4.97E-15
**C8orf4**	3.56	4.73E-16	5.08E-15
**RP11-448A19.1**	-2.14	4.82E-16	5.17E-15
**SEPHS2**	1.62	4.86E-16	5.22E-15
**RPL29**	1.26	4.91E-16	5.26E-15
**MPZL1**	1.01	4.92E-16	5.27E-15
**RASAL2**	-1.35	4.93E-16	5.28E-15
**SUCNR1**	-2.95	4.98E-16	5.34E-15
**AEBP1**	3.40	4.98E-16	5.34E-15
**RRP9**	1.37	5.02E-16	5.38E-15

**SLC25A25**	1.26	5.04E-16	5.39E-15
**HIST1H4I**	-2.03	5.04E-16	5.39E-15
**GSTM4**	1.55	5.09E-16	5.45E-15
**EFCAB13**	-2.30	5.20E-16	5.56E-15
**ZUFSP**	-1.00	5.37E-16	5.74E-15
**NIPA1**	-0.94	5.37E-16	5.74E-15
**RASA1**	-0.83	5.51E-16	5.89E-15
**TRRAP**	-1.08	5.53E-16	5.90E-15
**EEF1A1P4**	-5.34	5.60E-16	5.98E-15
**SERINC2**	2.35	5.64E-16	6.03E-15
**WBSCR22**	1.05	5.65E-16	6.03E-15
**G20736**	-1.81	5.69E-16	6.07E-15
**ZNF586**	-1.04	5.69E-16	6.07E-15
**KMT2A**	-1.14	5.73E-16	6.10E-15
**ST6GALNAC1**	4.28	5.73E-16	6.10E-15
**ACAA1**	1.66	5.80E-16	6.18E-15
**SEMA4C**	0.95	5.82E-16	6.19E-15
**PDE2A**	2.57	5.98E-16	6.37E-15
**SF1**	1.26	5.99E-16	6.38E-15
**RRBP1**	1.51	6.02E-16	6.41E-15
**VGLL4**	0.90	6.02E-16	6.41E-15
**NR2F1**	4.00	6.05E-16	6.44E-15
**RP11-767N6.2**	-3.09	6.11E-16	6.50E-15
**HNRNPD**	1.31	6.18E-16	6.56E-15
**FUOM**	2.05	6.25E-16	6.64E-15
**ZNF726**	-2.55	6.31E-16	6.70E-15
**TTN-AS1**	-2.38	6.31E-16	6.70E-15
**CTA-363E19.2**	-3.09	6.33E-16	6.71E-15
**RP11-73M18.6**	-4.18	6.33E-16	6.72E-15
**ANKRD18A**	-3.13	6.36E-16	6.75E-15
**TBRG4**	0.83	6.47E-16	6.86E-15
**ERAL1**	0.91	6.73E-16	7.13E-15
**MYCBP2-AS1**	-4.74	6.86E-16	7.27E-15
**DHRS13**	1.76	7.02E-16	7.44E-15
**KDR**	2.85	7.25E-16	7.67E-15
**AC084082.3**	-5.34	7.26E-16	7.68E-15
**PTBP3**	-1.23	7.40E-16	7.83E-15
**RP11-9E17.1**	-1.78	7.45E-16	7.88E-15
**GAMT**	1.94	7.47E-16	7.90E-15
**TRIM60P18**	-2.25	7.48E-16	7.91E-15
**MYD88**	1.65	7.52E-16	7.95E-15
**ATP13A4**	-1.80	7.54E-16	7.96E-15
**CD14**	4.29	7.61E-16	8.04E-15
**RPS6KA6**	-1.87	7.62E-16	8.05E-15
**RGS14**	1.26	7.67E-16	8.10E-15

**PODXL**	2.35	7.70E-16	8.12E-15
**ELFN1**	2.27	7.73E-16	8.15E-15
**EVA1B**	2.82	7.76E-16	8.18E-15
**CTXN1**	2.99	7.78E-16	8.20E-15
**TYROBP**	3.43	7.82E-16	8.23E-15
**SEMA3D**	-3.17	8.09E-16	8.52E-15
**ZNF358**	1.90	8.31E-16	8.74E-15
**SCN1B**	3.50	8.45E-16	8.89E-15
**PURA**	-1.13	8.54E-16	8.98E-15
**UBE2H**	0.78	8.75E-16	9.20E-15
**KIAA1456**	-1.84	8.84E-16	9.30E-15
**CBFA2T3**	1.89	8.86E-16	9.31E-15
**PI4KB**	0.64	9.08E-16	9.54E-15
**EGR1**	3.51	9.22E-16	9.68E-15
**SMURF2P1**	2.28	9.26E-16	9.72E-15
**RELB**	1.59	9.32E-16	9.78E-15
**IQGAP1**	-0.85	9.48E-16	9.95E-15
**MGAT1**	1.30	9.81E-16	1.03E-14
**G41074**	-3.83	9.81E-16	1.03E-14
**ANKDD1B**	-3.74	9.82E-16	1.03E-14
**GPSM3**	2.61	9.83E-16	1.03E-14
**ISY1**	1.06	9.91E-16	1.04E-14
**SMAD5-AS1**	-3.12	9.91E-16	1.04E-14
**G6313**	-2.93	9.97E-16	1.04E-14
**ERGIC2**	-0.93	1.00E-15	1.05E-14
**CD82**	1.26	1.01E-15	1.05E-14
**PSMD13**	0.92	1.03E-15	1.07E-14
**USP15**	-1.04	1.03E-15	1.07E-14
**GCDH**	1.13	1.04E-15	1.08E-14
**RP11-164P12.4**	-1.81	1.05E-15	1.10E-14
**MBTD1**	-1.16	1.06E-15	1.10E-14
**PPFIBP1**	-0.86	1.07E-15	1.12E-14
**ZNF852**	-1.74	1.08E-15	1.13E-14
**LCE3D**	5.45	1.09E-15	1.13E-14
**ACADL**	-2.24	1.09E-15	1.14E-14
**FRMD3**	2.17	1.09E-15	1.14E-14
**SRA1**	1.06	1.10E-15	1.15E-14
**ZNF680**	-1.41	1.10E-15	1.15E-14
**KIAA0319L**	0.84	1.11E-15	1.15E-14
**SMG1P3**	-2.46	1.11E-15	1.16E-14
**SLC8B1**	1.13	1.12E-15	1.17E-14
**ZNRF1**	1.07	1.12E-15	1.17E-14
**ZNF678**	-1.63	1.13E-15	1.17E-14
**NUDC**	1.12	1.13E-15	1.17E-14
**EPHB2**	3.08	1.13E-15	1.17E-14

**HDAC4**	-0.98	1.13E-15	1.18E-14
**BAHCC1**	1.68	1.14E-15	1.19E-14
**NDUFS6**	1.37	1.15E-15	1.19E-14
**SAA1**	6.06	1.15E-15	1.20E-14
**INHBB**	3.54	1.16E-15	1.21E-14
**SACS**	-1.42	1.18E-15	1.22E-14
**GNA11**	0.87	1.19E-15	1.23E-14
**ALYREF**	1.05	1.19E-15	1.23E-14
**ZNF658**	-1.51	1.20E-15	1.25E-14
**KPNA4**	-0.96	1.21E-15	1.25E-14
**AC006126.4**	4.51	1.22E-15	1.26E-14
**CFI**	2.79	1.23E-15	1.28E-14
**ZNF714**	-2.09	1.26E-15	1.30E-14
**DPYSL4**	2.90	1.26E-15	1.30E-14
**PPP5C**	0.81	1.27E-15	1.32E-14
**ACKR1**	2.62	1.29E-15	1.34E-14
**ZNF445**	-0.82	1.31E-15	1.35E-14
**ZBTB22**	0.99	1.33E-15	1.38E-14
**MGAT3**	2.16	1.36E-15	1.40E-14
**ZNF470**	-1.46	1.36E-15	1.40E-14
**UBE2W**	-1.08	1.37E-15	1.41E-14
**ZNF250**	-0.90	1.37E-15	1.41E-14
**MT-CO2**	2.16	1.37E-15	1.42E-14
**AC124789.1**	2.12	1.39E-15	1.43E-14
**KATNAL1**	-1.09	1.40E-15	1.45E-14
**TMEM208**	1.31	1.41E-15	1.46E-14
**UBQLN4**	1.13	1.42E-15	1.47E-14
**ROMO1**	1.44	1.43E-15	1.47E-14
**CTD-3035K23.3**	-4.43	1.44E-15	1.48E-14
**ZNF181**	-0.97	1.46E-15	1.50E-14
**GNG11**	2.18	1.46E-15	1.51E-14
**RP11-64D22.1**	-2.25	1.47E-15	1.51E-14
**PIGQ**	1.23	1.48E-15	1.52E-14
**SCG2**	5.47	1.48E-15	1.53E-14
**RCHY1**	-1.08	1.49E-15	1.54E-14
**SEC14L2**	1.86	1.50E-15	1.54E-14
**TM4SF18**	3.42	1.54E-15	1.58E-14
**PRKCD**	1.08	1.54E-15	1.59E-14
**MAFG**	0.90	1.55E-15	1.60E-14
**CFAP44**	-1.57	1.60E-15	1.64E-14
**SEC61G**	1.45	1.60E-15	1.64E-14
**RP11-727A23.5**	-2.60	1.60E-15	1.64E-14
**NREP**	2.00	1.61E-15	1.65E-14
**RN7SKP255**	-8.32	1.62E-15	1.66E-14
**PXDC1**	1.28	1.62E-15	1.66E-14

**ANGPTL1**	-3.03	1.69E-15	1.73E-14
**RP11-191L9.4**	-2.53	1.73E-15	1.77E-14
**LSR**	1.26	1.74E-15	1.78E-14
**ZNF570**	-1.06	1.77E-15	1.81E-14
**UNK**	0.77	1.80E-15	1.84E-14
**MRPL46**	-1.17	1.81E-15	1.85E-14
**RP11-637N19.1**	-8.68	1.82E-15	1.86E-14
**P3H2**	-1.85	1.82E-15	1.86E-14
**ATF7IP2**	-2.33	1.84E-15	1.88E-14
**TTC3**	-0.72	1.85E-15	1.88E-14
**RFTN1**	2.00	1.85E-15	1.89E-14
**BORA**	-1.37	1.85E-15	1.89E-14
**KRT17**	4.72	1.85E-15	1.89E-14
**IL13RA1**	1.74	1.89E-15	1.92E-14
**VSIG8**	-2.44	1.89E-15	1.93E-14
**RP11-357N13.6**	-5.28	1.91E-15	1.94E-14
**RCN3**	3.59	1.93E-15	1.96E-14
**LRRC8A**	0.97	1.94E-15	1.97E-14
**PP2D1**	-2.41	1.99E-15	2.03E-14
**C1QC**	4.38	1.99E-15	2.03E-14
**ACIN1**	0.80	1.99E-15	2.03E-14
**RPL26L1**	1.37	2.01E-15	2.04E-14
**SLC4A3**	3.26	2.01E-15	2.05E-14
**ACOT7**	1.21	2.01E-15	2.05E-14
**PGBD4**	-1.28	2.04E-15	2.08E-14
**PA2G4P4**	-4.09	2.05E-15	2.08E-14
**CCDC167**	1.59	2.08E-15	2.11E-14
**SPPL2A**	-0.99	2.09E-15	2.12E-14
**SUFU**	1.31	2.11E-15	2.14E-14
**GSTO1**	1.35	2.14E-15	2.17E-14
**PDE4A**	2.18	2.14E-15	2.17E-14
**PPP1R15B**	-0.88	2.14E-15	2.17E-14
**RP11-420L9.5**	2.49	2.15E-15	2.18E-14
**SCARNA1**	-9.49	2.17E-15	2.20E-14
**SNORD3B-2**	-5.09	2.17E-15	2.20E-14
**IMPA2**	1.39	2.19E-15	2.22E-14
**SERPINI1**	1.99	2.26E-15	2.29E-14
**RP11-46D6.1**	3.01	2.28E-15	2.31E-14
**BRAT1**	1.14	2.31E-15	2.34E-14
**STK11IP**	0.98	2.33E-15	2.35E-14
**LHX4**	-2.77	2.35E-15	2.38E-14
**CDKN1A**	1.43	2.37E-15	2.40E-14
**snoU13**	-2.09	2.39E-15	2.41E-14
**CRTC1**	1.88	2.39E-15	2.42E-14
**MPP1**	1.26	2.43E-15	2.45E-14

**PLOD3**	1.79	2.45E-15	2.48E-14
**CPSF3L**	0.93	2.46E-15	2.49E-14
**KIF24**	-1.27	2.46E-15	2.49E-14
**G3793**	-8.56	2.57E-15	2.60E-14
**HHLA3**	1.78	2.58E-15	2.60E-14
**TRIM28**	0.85	2.62E-15	2.64E-14
**PCGF1**	1.05	2.66E-15	2.68E-14
**GEM**	3.18	2.67E-15	2.69E-14
**SUV39H2**	-1.19	2.69E-15	2.71E-14
**SNORA50**	-9.45	2.73E-15	2.74E-14
**ITPR2**	-1.68	2.74E-15	2.75E-14
**BLOC1S3**	1.56	2.74E-15	2.76E-14
**TMC8**	2.50	2.78E-15	2.80E-14
**VAT1**	1.04	2.80E-15	2.82E-14
**THAP3**	0.98	2.88E-15	2.90E-14
**TPM3**	1.01	2.89E-15	2.90E-14
**RN7SL5P**	-8.25	2.90E-15	2.92E-14
**NRGN**	3.90	2.93E-15	2.94E-14
**TOP1**	0.98	2.96E-15	2.97E-14
**CDK16**	0.78	2.96E-15	2.97E-14
**FAM133CP**	6.01	2.97E-15	2.97E-14
**THOC3**	1.70	2.99E-15	2.99E-14
**EXOSC5**	1.49	3.00E-15	3.01E-14
**SPOCD1**	5.39	3.00E-15	3.01E-14
**ZNF566**	-1.18	3.02E-15	3.02E-14
**SLC25A51**	-0.82	3.02E-15	3.03E-14
**GCFC2**	-1.03	3.03E-15	3.03E-14
**ZNF337-AS1**	-2.16	3.08E-15	3.08E-14
**LPP**	-1.06	3.10E-15	3.10E-14
**PCBD1**	1.49	3.10E-15	3.10E-14
**TOR4A**	2.82	3.14E-15	3.14E-14
**P2RY4**	-3.88	3.17E-15	3.17E-14
**PAPOLG**	-0.93	3.17E-15	3.17E-14
**EPHA2**	1.19	3.17E-15	3.17E-14
**MAOB**	2.11	3.19E-15	3.18E-14
**EBF4**	2.16	3.19E-15	3.19E-14
**TMEM41B**	-1.22	3.19E-15	3.19E-14
**MALT1**	-1.07	3.27E-15	3.26E-14
**BET1L**	1.20	3.28E-15	3.27E-14
**DALRD3**	0.99	3.29E-15	3.28E-14
**EIF2B2**	0.82	3.29E-15	3.28E-14
**ZNF749**	-1.39	3.33E-15	3.31E-14
**EFR3A**	-1.08	3.33E-15	3.32E-14
**DSG3**	1.50	3.39E-15	3.37E-14
**LRP4**	-1.59	3.49E-15	3.47E-14

**MRPS2**	0.89	3.52E-15	3.50E-14
**ARHGDIB**	1.92	3.56E-15	3.54E-14
**CNTRL**	-1.08	3.57E-15	3.55E-14
**TMTC3**	-1.38	3.59E-15	3.57E-14
**MAN2A1**	-1.08	3.60E-15	3.58E-14
**AC092835.2**	-2.34	3.60E-15	3.58E-14
**SAMD10**	1.34	3.62E-15	3.60E-14
**RAMP1**	1.97	3.63E-15	3.61E-14
**DNAJC24**	-0.79	3.66E-15	3.64E-14
**RPL10**	1.04	3.69E-15	3.66E-14
**FAM221A**	-2.51	3.69E-15	3.66E-14
**ZFP62**	-0.98	3.71E-15	3.68E-14
**CCDC66**	-1.24	3.71E-15	3.68E-14
**RPLP0P2**	4.86	3.71E-15	3.68E-14
**NT5C3B**	1.48	3.75E-15	3.72E-14
**C2orf81**	2.55	3.76E-15	3.72E-14
**LINC00346**	3.25	3.80E-15	3.76E-14
**TGM1**	1.88	3.80E-15	3.77E-14
**SPATA2L**	1.23	3.81E-15	3.77E-14
**GJB4**	-2.20	3.81E-15	3.77E-14
**SNHG16**	-0.86	3.85E-15	3.80E-14
**COL13A1**	2.28	3.88E-15	3.84E-14
**PTS**	0.84	3.92E-15	3.87E-14
**DGCR14**	0.93	4.00E-15	3.96E-14
**ZNF197**	-0.70	4.03E-15	3.98E-14
**MT1G**	3.15	4.04E-15	3.99E-14
**ALCAM**	-1.86	4.06E-15	4.01E-14
**ABI3**	1.95	4.09E-15	4.03E-14
**AP2A2**	0.91	4.10E-15	4.04E-14
**ZNF273**	-2.07	4.20E-15	4.14E-14
**PKN2**	-0.78	4.23E-15	4.17E-14
**GCSH**	1.78	4.23E-15	4.17E-14
**BAIAP2-AS1**	1.76	4.29E-15	4.22E-14
**G12854**	5.04	4.29E-15	4.23E-14
**LRP5**	0.98	4.36E-15	4.29E-14
**ZDHHC12**	1.21	4.39E-15	4.32E-14
**TPRG1**	-1.49	4.40E-15	4.33E-14
**PINK1**	0.84	4.41E-15	4.34E-14
**IQCJ-SCHIP1**	2.50	4.44E-15	4.36E-14
**NCOA5**	0.96	4.47E-15	4.40E-14
**TRAF7**	0.91	4.51E-15	4.44E-14
**FBXW9**	1.30	4.52E-15	4.45E-14
**APEX2**	0.81	4.57E-15	4.49E-14
**SDC3**	2.12	4.57E-15	4.49E-14
**CECR5**	0.95	4.60E-15	4.52E-14

**SNAP47**	0.74	4.63E-15	4.55E-14
**FAM167B**	4.94	4.63E-15	4.55E-14
**NCKAP1**	-0.99	4.65E-15	4.56E-14
**DLEU2**	-2.18	4.72E-15	4.63E-14
**C10orf128**	2.28	4.74E-15	4.64E-14
**BAG4**	-0.89	4.83E-15	4.73E-14
**TMEM203**	0.90	4.84E-15	4.74E-14
**C1QTNF7**	-3.00	4.85E-15	4.75E-14
**RNU6ATAC**	-8.14	4.85E-15	4.75E-14
**TDRD6**	-2.46	4.90E-15	4.80E-14
**RP5-1085F17.4**	-4.07	4.92E-15	4.81E-14
**AC005154.6**	-1.85	4.93E-15	4.83E-14
**ZKSCAN1**	-0.74	5.03E-15	4.92E-14
**CLIP3**	3.01	5.09E-15	4.97E-14
**USP25**	-0.74	5.13E-15	5.01E-14
**EIF4E2**	0.84	5.16E-15	5.04E-14
**MRPL16**	0.98	5.18E-15	5.06E-14
**SPI1**	2.36	5.27E-15	5.14E-14
**PEX5**	0.77	5.27E-15	5.14E-14
**HIVEP1**	-1.20	5.29E-15	5.17E-14
**HSPB6**	4.07	5.31E-15	5.18E-14
**TSPYL2**	1.30	5.32E-15	5.19E-14
**TMEM184B**	1.41	5.38E-15	5.24E-14
**TMEM186**	1.04	5.46E-15	5.32E-14
**SNORA38B**	-8.06	5.48E-15	5.33E-14
**AP2A1**	0.94	5.52E-15	5.37E-14
**TAC1**	-5.03	5.54E-15	5.39E-14
**MSRA**	1.23	5.63E-15	5.48E-14
**LINC01605**	3.45	5.63E-15	5.48E-14
**SNRPN**	-2.00	5.64E-15	5.48E-14
**RP11-504P24.9**	-3.56	5.66E-15	5.51E-14
**FSTL3**	2.26	5.68E-15	5.53E-14
**KRT6B**	5.71	5.71E-15	5.55E-14
**DNTTIP1**	1.14	5.72E-15	5.56E-14
**REXO4**	1.05	5.75E-15	5.58E-14
**RARA**	1.41	5.75E-15	5.58E-14
**XKR8**	1.34	5.77E-15	5.60E-14
**ZNF268**	-1.24	5.83E-15	5.65E-14
**WARS**	0.96	5.83E-15	5.66E-14
**NPM3**	1.51	5.84E-15	5.67E-14
**DNAJC8**	0.98	5.89E-15	5.71E-14
**DNAJC17**	0.89	5.97E-15	5.79E-14
**MTMR9**	-0.62	5.99E-15	5.80E-14
**GREB1L**	-3.36	6.02E-15	5.83E-14
**LAIR1**	4.01	6.04E-15	5.85E-14

**KDM5A**	-0.97	6.10E-15	5.91E-14
**GIGYF2**	-0.77	6.11E-15	5.91E-14
**RILPL1**	1.28	6.12E-15	5.92E-14
**CHMP1A**	1.17	6.12E-15	5.92E-14
**XAB2**	1.15	6.14E-15	5.94E-14
**RSPO4**	4.49	6.14E-15	5.94E-14
**PCBP3**	3.23	6.18E-15	5.97E-14
**ATXN7**	-0.96	6.19E-15	5.98E-14
**PIK3C3**	-0.85	6.26E-15	6.04E-14
**ZNF180**	-1.10	6.27E-15	6.05E-14
**THEMIS2**	2.63	6.29E-15	6.07E-14
**PRR7-AS1**	-3.01	6.31E-15	6.09E-14
**TRIM8**	1.37	6.33E-15	6.11E-14
**RBSN**	-0.84	6.44E-15	6.21E-14
**TBXA2R**	4.98	6.50E-15	6.26E-14
**ST7-OT4**	-5.80	6.52E-15	6.29E-14
**BRCA1**	-1.19	6.55E-15	6.31E-14
**NPTX1**	-5.77	6.57E-15	6.33E-14
**ZNF292**	-1.23	6.66E-15	6.41E-14
**NELFA**	0.87	6.77E-15	6.52E-14
**GABPB2**	-1.08	6.84E-15	6.58E-14
**PLTP**	2.00	6.86E-15	6.60E-14
**SURF6**	1.06	6.87E-15	6.60E-14
**DVL2**	0.78	6.90E-15	6.63E-14
**CH17-360D5.3**	-2.31	7.01E-15	6.74E-14
**RIN1**	1.13	7.09E-15	6.81E-14
**CHERP**	1.41	7.17E-15	6.89E-14
**RAC3**	1.68	7.26E-15	6.97E-14
**HEY2**	2.74	7.39E-15	7.09E-14
**FAM210B**	1.06	7.40E-15	7.10E-14
**SNRPD2**	1.31	7.44E-15	7.13E-14
**RP11-206L10.8**	-2.72	7.50E-15	7.19E-14
**THBS1**	3.55	7.57E-15	7.25E-14
**ECI1**	1.29	7.59E-15	7.27E-14
**RP11-182N22.9**	-4.21	7.61E-15	7.29E-14
**KRT6A**	5.68	7.69E-15	7.37E-14
**RP11-701H24.4**	-3.66	7.74E-15	7.41E-14
**SETD1A**	1.68	7.80E-15	7.47E-14
**SH2B1**	1.45	7.83E-15	7.50E-14
**PYCR1**	2.52	7.87E-15	7.53E-14
**OTUB1**	1.14	7.94E-15	7.59E-14
**ITPR3**	-1.38	8.02E-15	7.67E-14
**ERBB4**	-3.55	8.19E-15	7.83E-14
**MEA1**	1.23	8.23E-15	7.86E-14
**RPS3AP34**	-4.63	8.37E-15	7.99E-14

**PNKP**	1.36	8.51E-15	8.13E-14
**SPAST**	-1.06	8.53E-15	8.14E-14
**MBNL1**	-0.91	8.60E-15	8.21E-14
**WDR54**	1.85	8.62E-15	8.22E-14
**EPG5**	-1.06	8.66E-15	8.26E-14
**EHMT2**	1.13	8.69E-15	8.29E-14
**MRPL22**	0.85	8.70E-15	8.29E-14
**GTF3C4**	-1.02	8.82E-15	8.41E-14
**LIPK**	-1.92	8.93E-15	8.50E-14
**DGCR12**	-3.22	9.01E-15	8.58E-14
**HOXC8**	2.25	9.09E-15	8.65E-14
**SOD3**	3.07	9.14E-15	8.70E-14
**NKD1**	-1.67	9.15E-15	8.71E-14
**TTF2**	-1.14	9.17E-15	8.72E-14
**RASL12**	2.70	9.22E-15	8.76E-14
**C12orf66**	-1.30	9.26E-15	8.80E-14
**GNPTAB**	-0.89	9.26E-15	8.80E-14
**OTUD3**	-0.97	9.39E-15	8.92E-14
**MON1B**	0.56	9.42E-15	8.95E-14
**ARHGEF2**	1.73	9.64E-15	9.16E-14
**FCER1A**	-2.21	9.79E-15	9.29E-14
**RP5-894A10.6**	-2.21	9.79E-15	9.29E-14
**PXMP2**	-2.40	9.82E-15	9.32E-14
**NRXN3**	-2.04	9.87E-15	9.36E-14
**COL5A2**	3.06	9.93E-15	9.41E-14
**FAM91A1**	-0.85	1.01E-14	9.57E-14
**RILP**	1.63	1.01E-14	9.61E-14
**CSRP1**	1.47	1.04E-14	9.84E-14
**ZNF585A**	-1.08	1.04E-14	9.88E-14
**ZBTB38**	-0.89	1.05E-14	9.94E-14
**CDH26**	2.51	1.05E-14	9.95E-14
**RHBDF2**	1.34	1.05E-14	9.95E-14
**ITM2C**	1.98	1.07E-14	1.02E-13
**TALDO1**	1.07	1.08E-14	1.02E-13
**ISCU**	0.79	1.08E-14	1.02E-13
**HLA-E**	1.28	1.10E-14	1.04E-13
**GBP6**	3.01	1.10E-14	1.04E-13
**RP5-1074L1.4**	-2.45	1.12E-14	1.06E-13
**ZNF611**	-1.01	1.13E-14	1.06E-13
**LINC00235**	-2.18	1.15E-14	1.08E-13
**SLC29A3**	2.16	1.16E-14	1.09E-13
**RP11-288K12.1**	-4.10	1.16E-14	1.10E-13
**PGM1**	1.27	1.18E-14	1.11E-13
**PCOLCE**	3.68	1.19E-14	1.12E-13
**SKIL**	-1.28	1.19E-14	1.12E-13

**FAM110C**	1.37	1.19E-14	1.12E-13
**LL22NC03-2H8.5**	5.39	1.19E-14	1.12E-13
**RP11-71H17.8**	-4.68	1.20E-14	1.13E-13
**SNRPGP4**	-4.91	1.21E-14	1.14E-13
**FN3K**	2.42	1.21E-14	1.14E-13
**RP11-135N5.3**	-6.22	1.21E-14	1.14E-13
**CADPS**	-3.36	1.22E-14	1.15E-13
**ELF2**	-0.58	1.23E-14	1.15E-13
**SERPINB3**	3.42	1.23E-14	1.15E-13
**FAM196A**	-3.31	1.25E-14	1.17E-13
**ARF3**	0.77	1.26E-14	1.18E-13
**ZNF383**	-0.77	1.26E-14	1.19E-13
**ATP6V0B**	1.17	1.27E-14	1.19E-13
**ERVK3-1**	-2.02	1.29E-14	1.21E-13
**MPHOSPH9**	-1.05	1.31E-14	1.23E-13
**RP11-395A13.2**	1.29	1.34E-14	1.25E-13
**RP11-481C4.2**	-2.11	1.34E-14	1.26E-13
**CISD3**	1.42	1.35E-14	1.26E-13
**RP11-11N7.5**	-2.89	1.35E-14	1.26E-13
**NDUFC2**	-1.37	1.36E-14	1.28E-13
**EXOC5**	-0.98	1.38E-14	1.29E-13
**RP11-474P2.6**	-4.21	1.39E-14	1.31E-13
**EXOSC3**	-0.95	1.41E-14	1.32E-13
**PRRC2A**	1.11	1.41E-14	1.32E-13
**MIR22HG**	1.27	1.44E-14	1.34E-13
**CSF1**	2.05	1.44E-14	1.34E-13
**ASB11**	-4.16	1.47E-14	1.37E-13
**SLC19A2**	-1.38	1.50E-14	1.40E-13
**CDH24**	1.67	1.50E-14	1.40E-13
**APCDD1L-AS1**	-3.02	1.51E-14	1.41E-13
**CTBP1-AS2**	1.10	1.53E-14	1.43E-13
**AKAP9**	-1.06	1.54E-14	1.43E-13
**KLF16**	1.65	1.54E-14	1.44E-13
**BTBD18**	-4.00	1.55E-14	1.44E-13
**LINC01091**	-3.14	1.55E-14	1.44E-13
**CHSY3**	2.66	1.56E-14	1.45E-13
**DNAJB5**	2.21	1.57E-14	1.46E-13
**APOLD1**	2.03	1.57E-14	1.47E-13
**C6orf15**	3.21	1.63E-14	1.52E-13
**CTC-321K16.4**	-7.32	1.70E-14	1.58E-13
**LGALSL**	-1.39	1.72E-14	1.60E-13
**RP11-107E5.3**	-5.03	1.72E-14	1.60E-13
**TXNDC16**	-1.28	1.72E-14	1.60E-13
**AWAT2**	-10.92	1.74E-14	1.62E-13
**SNHG20**	-3.29	1.74E-14	1.62E-13

**LRCH1**	-1.02	1.75E-14	1.63E-13
**GNG12**	-1.26	1.76E-14	1.64E-13
**CNTNAP1**	2.59	1.79E-14	1.66E-13
**CASC5**	-1.73	1.79E-14	1.66E-13
**RGS12**	1.05	1.79E-14	1.66E-13
**UBE2FP3**	-2.81	1.79E-14	1.66E-13
**IGF1**	4.30	1.79E-14	1.66E-13
**ANKRD17**	-0.64	1.79E-14	1.66E-13
**TIMM50**	0.87	1.79E-14	1.66E-13
**DCAF15**	1.20	1.80E-14	1.67E-13
**AC010976.2**	-2.77	1.81E-14	1.68E-13
**TMEM51**	1.45	1.84E-14	1.70E-13
**PSMD4**	1.01	1.84E-14	1.70E-13
**NUDT18**	1.44	1.85E-14	1.71E-13
**CXCL3**	5.61	1.86E-14	1.72E-13
**ANGPTL4**	3.20	1.86E-14	1.72E-13
**SSU72**	1.02	1.87E-14	1.73E-13
**DIEXF**	-0.84	1.89E-14	1.75E-13
**ACE**	2.97	1.92E-14	1.77E-13
**IQCA1**	4.38	1.93E-14	1.79E-13
**NXF1**	1.32	1.95E-14	1.80E-13
**GCNT7**	-4.01	1.97E-14	1.82E-13
**SLC25A1**	1.41	2.01E-14	1.85E-13
**BLOC1S1**	-1.47	2.05E-14	1.89E-13
**TRAPPC10**	-0.68	2.06E-14	1.90E-13
**NT5C**	1.07	2.07E-14	1.91E-13
**PSMC3**	1.08	2.07E-14	1.91E-13
**FOXP4**	2.12	2.07E-14	1.91E-13
**NF2**	-0.78	2.08E-14	1.92E-13
**RPL41P1**	1.89	2.11E-14	1.95E-13
**HEPH**	2.42	2.12E-14	1.95E-13
**ZNF672**	0.74	2.13E-14	1.96E-13
**ZNF432**	-0.88	2.13E-14	1.96E-13
**SECISBP2L**	-0.98	2.16E-14	1.99E-13
**SPN**	-1.52	2.18E-14	2.01E-13
**BMPR2**	-0.88	2.24E-14	2.06E-13
**MRPS11**	0.69	2.25E-14	2.07E-13
**CTD-2260A17.1**	-3.10	2.25E-14	2.07E-13
**RNU12**	-5.52	2.27E-14	2.09E-13
**LMOD1**	2.80	2.27E-14	2.09E-13
**FCGR1A**	5.31	2.32E-14	2.13E-13
**NAIF1**	0.90	2.32E-14	2.13E-13
**SRRT**	1.41	2.33E-14	2.14E-13
**MRPL17**	0.96	2.41E-14	2.21E-13
**MTHFD1P1**	-5.56	2.45E-14	2.25E-13

**PCDHGB8P**	-3.37	2.46E-14	2.26E-13
**FCGRT**	1.63	2.47E-14	2.26E-13
**WBP5**	1.75	2.49E-14	2.28E-13
**G32150**	-3.61	2.51E-14	2.30E-13
**FANCC**	-1.47	2.53E-14	2.32E-13
**ZC3H18**	1.17	2.58E-14	2.36E-13
**NRP2**	2.63	2.59E-14	2.37E-13
**NOS3**	2.49	2.61E-14	2.39E-13
**TBC1D31**	-1.12	2.69E-14	2.46E-13
**SNORA40**	-2.93	2.69E-14	2.46E-13
**KLK14**	2.68	2.70E-14	2.47E-13
**OGN**	-3.69	2.72E-14	2.48E-13
**NUP153**	-0.75	2.73E-14	2.50E-13
**LRRC8C**	-1.45	2.74E-14	2.51E-13
**GPD2**	-1.04	2.76E-14	2.52E-13
**LMBR1**	-0.78	2.77E-14	2.53E-13
**G26344**	-3.76	2.77E-14	2.53E-13
**ZNF492**	-2.63	2.78E-14	2.54E-13
**LURAP1L**	1.30	2.79E-14	2.54E-13
**NDUFAF2**	1.20	2.83E-14	2.58E-13
**PBXIP1**	0.84	2.86E-14	2.61E-13
**EXOSC4**	1.13	2.95E-14	2.69E-13
**PAG1**	-1.04	2.96E-14	2.70E-13
**USP11**	1.11	2.97E-14	2.71E-13
**RENBP**	2.45	3.01E-14	2.74E-13
**RP11-180M15.7**	-2.44	3.01E-14	2.74E-13
**FLG2**	-2.41	3.02E-14	2.75E-13
**FGL2**	-2.97	3.04E-14	2.77E-13
**S100A10**	1.32	3.09E-14	2.81E-13
**SDSL**	3.11	3.14E-14	2.86E-13
**PSMA1**	-1.21	3.14E-14	2.86E-13
**RP11-823P9.3**	-3.76	3.15E-14	2.87E-13
**MRPS26**	1.05	3.18E-14	2.89E-13
**AGO4**	-0.87	3.20E-14	2.91E-13
**KB-1410C5.3**	-4.63	3.23E-14	2.93E-13
**SOCS6**	-0.94	3.28E-14	2.98E-13
**SLC39A10**	-1.31	3.32E-14	3.02E-13
**PYCRL**	1.19	3.34E-14	3.03E-13
**RNF217**	-1.28	3.37E-14	3.06E-13
**CIC**	1.43	3.39E-14	3.07E-13
**GTF3C3**	-0.84	3.39E-14	3.08E-13
**CREBRF**	-1.14	3.40E-14	3.08E-13
**RP11-423P10.2**	-2.48	3.42E-14	3.10E-13
**GINS3**	1.24	3.47E-14	3.14E-13
**MEGF9**	-1.18	3.51E-14	3.18E-13

**BORCS6**	1.29	3.52E-14	3.19E-13
**LSM4**	1.00	3.52E-14	3.19E-13
**DTD1**	1.04	3.52E-14	3.19E-13
**TPST2**	1.06	3.54E-14	3.21E-13
**VPS8**	-1.00	3.64E-14	3.30E-13
**PUF60**	0.80	3.67E-14	3.32E-13
**PM20D1**	-7.83	3.67E-14	3.32E-13
**G31357**	-4.89	3.71E-14	3.35E-13
**CDH4**	-3.22	3.76E-14	3.40E-13
**MINOS1**	-1.05	3.78E-14	3.42E-13
**MAGI1-IT1**	-3.49	3.81E-14	3.44E-13
**ITGA5**	2.43	3.83E-14	3.46E-13
**ITGB4**	1.09	3.94E-14	3.56E-13
**SERPINF2**	1.56	3.96E-14	3.57E-13
**VPS25**	0.96	3.97E-14	3.58E-13
**WWP2**	0.72	3.97E-14	3.58E-13
**RPL29P14**	-3.94	4.09E-14	3.69E-13
**ZNF768**	1.21	4.13E-14	3.72E-13
**CTAGE7P**	-3.04	4.15E-14	3.74E-13
**ARSA**	1.57	4.16E-14	3.74E-13
**SNORA76**	-8.25	4.16E-14	3.74E-13
**HACD2**	-1.20	4.17E-14	3.75E-13
**POLR2L**	1.36	4.22E-14	3.80E-13
**RABEP2**	1.19	4.24E-14	3.82E-13
**BRD9**	1.00	4.31E-14	3.88E-13
**SPR**	1.69	4.32E-14	3.89E-13
**GPX4**	1.57	4.33E-14	3.90E-13
**VOPP1**	1.68	4.38E-14	3.94E-13
**TERF2IP**	0.78	4.39E-14	3.95E-13
**G25746**	-3.95	4.43E-14	3.98E-13
**HPS1**	0.89	4.49E-14	4.03E-13
**HIC1**	3.22	4.51E-14	4.05E-13
**LGALS3BP**	2.17	4.56E-14	4.09E-13
**TECR**	1.21	4.56E-14	4.09E-13
**ZNF771**	1.43	4.59E-14	4.12E-13
**ARL2**	1.16	4.63E-14	4.15E-13
**LINC00519**	3.18	4.66E-14	4.18E-13
**FAM53B**	0.74	4.74E-14	4.24E-13
**RP5-1112D6.4**	-1.96	4.76E-14	4.27E-13
**FTH1P20**	4.80	4.79E-14	4.29E-13
**ALDH3A2**	-1.69	4.80E-14	4.30E-13
**SLC10A3**	1.03	4.87E-14	4.36E-13
**RP11-53O19.3**	-1.21	4.89E-14	4.38E-13
**EFNA1**	1.15	4.94E-14	4.42E-13
**ZNF112**	-1.19	4.97E-14	4.44E-13

**CNOT1**	-0.82	5.02E-14	4.49E-13
**TGFB3**	2.75	5.11E-14	4.57E-13
**MTX1**	1.06	5.14E-14	4.59E-13
**RNU6-2**	-5.95	5.16E-14	4.61E-13
**MPP6**	-1.35	5.16E-14	4.61E-13
**GJD3**	2.61	5.16E-14	4.61E-13
**FOXL1**	4.62	5.17E-14	4.62E-13
**CCSER2**	-1.02	5.18E-14	4.63E-13
**IGSF11**	-2.09	5.20E-14	4.64E-13
**MMP1**	10.16	5.25E-14	4.69E-13
**SNORA74B**	-8.25	5.31E-14	4.74E-13
**MIB2**	1.25	5.32E-14	4.74E-13
**PYGO2**	0.81	5.39E-14	4.80E-13
**SNORD8**	-8.59	5.47E-14	4.87E-13
**EIF4A3**	1.10	5.50E-14	4.90E-13
**CBX4**	1.16	5.50E-14	4.90E-13
**REM1**	3.35	5.51E-14	4.91E-13
**DNAJC2**	0.78	5.53E-14	4.92E-13
**AIFM1**	0.85	5.55E-14	4.94E-13
**BMP6**	2.93	5.57E-14	4.95E-13
**SERPINA12**	-2.40	5.63E-14	5.01E-13
**GEN1**	-1.13	5.66E-14	5.03E-13
**CD109**	-0.99	5.67E-14	5.04E-13
**SESN2**	1.37	5.68E-14	5.05E-13
**Y_RNA**	-2.03	5.72E-14	5.08E-13
**BNIP2**	-1.08	5.73E-14	5.09E-13
**RP11-474L11.5**	-8.06	5.74E-14	5.10E-13
**ARPC5L**	1.08	5.81E-14	5.15E-13
**VPS37C**	0.92	5.86E-14	5.20E-13
**WDR35**	-1.02	5.93E-14	5.26E-13
**HLA-DMA**	2.25	5.94E-14	5.26E-13
**AC087294.2**	-3.95	5.96E-14	5.28E-13
**DUSP6**	1.74	5.98E-14	5.30E-13
**WDFY2**	-1.28	6.05E-14	5.36E-13
**ZNF668**	1.14	6.09E-14	5.39E-13
**C5**	-1.53	6.15E-14	5.45E-13
**DCTD**	0.95	6.22E-14	5.50E-13
**ABCA12**	-1.57	6.28E-14	5.56E-13
**RP11-666F17.1**	-4.16	6.42E-14	5.68E-13
**C4orf3**	1.31	6.42E-14	5.68E-13
**CTD-2547E10.3**	-3.46	6.46E-14	5.71E-13
**FSD2**	-3.58	6.47E-14	5.72E-13
**CTD-2587M2.1**	-4.03	6.56E-14	5.80E-13
**HMHA1**	1.61	6.60E-14	5.84E-13
**SLC30A7**	-0.92	6.64E-14	5.86E-13

**LGALS8**	-0.75	6.64E-14	5.86E-13
**RBBP5**	-0.83	6.71E-14	5.92E-13
**MYNN**	-0.89	6.77E-14	5.98E-13
**OSBPL3**	-0.86	6.83E-14	6.03E-13
**LMO2**	2.04	6.88E-14	6.07E-13
**LDHA**	1.30	6.88E-14	6.07E-13
**NFATC3**	-0.61	6.99E-14	6.16E-13
**MIA3**	-0.77	7.03E-14	6.19E-13
**ANKRD44**	-1.20	7.06E-14	6.22E-13
**AKR1B15**	4.74	7.20E-14	6.34E-13
**ZBTB39**	-0.82	7.21E-14	6.35E-13
**FAM127C**	0.98	7.21E-14	6.35E-13
**MTMR10**	-0.78	7.29E-14	6.42E-13
**PRKD2**	1.20	7.40E-14	6.52E-13
**CIRBP**	0.98	7.41E-14	6.52E-13
**RP11-245J9.5**	-3.27	7.49E-14	6.59E-13
**SUGCT**	2.82	7.49E-14	6.59E-13
**CCDC8**	2.69	7.65E-14	6.73E-13
**FASTKD2**	-0.86	7.68E-14	6.75E-13
**CDIP1**	0.81	7.69E-14	6.76E-13
**HAUS6**	-0.75	7.76E-14	6.81E-13
**ZNF649**	-1.02	7.79E-14	6.84E-13
**MAP4K5**	-0.81	7.83E-14	6.88E-13
**AKAP8**	0.66	7.86E-14	6.90E-13
**GPRC5C**	1.66	7.98E-14	7.00E-13
**LYL1**	2.87	8.01E-14	7.03E-13
**GNRH1**	-2.33	8.04E-14	7.05E-13
**CCM2L**	3.33	8.04E-14	7.05E-13
**LTBP2**	2.56	8.13E-14	7.12E-13
**BRMS1**	0.98	8.16E-14	7.15E-13
**TMEM50A**	1.01	8.19E-14	7.18E-13
**ETNK1**	-1.08	8.22E-14	7.19E-13
**OXLD1**	1.26	8.23E-14	7.21E-13
**ZMYND15**	2.29	8.25E-14	7.22E-13
**SLC3A1**	-4.01	8.29E-14	7.25E-13
**TTLL12**	1.30	8.33E-14	7.28E-13
**NT5DC2**	1.17	8.38E-14	7.32E-13
**RP11-363N22.3**	-3.99	8.65E-14	7.56E-13
**SNX11**	0.91	8.65E-14	7.56E-13
**RP11-734K2.4**	-2.01	8.80E-14	7.69E-13
**PLPP7**	3.40	8.86E-14	7.74E-13
**RP13-39P12.3**	-3.39	8.90E-14	7.77E-13
**UIMC1**	0.64	8.94E-14	7.80E-13
**CYP4Z2P**	-2.99	9.12E-14	7.96E-13
**CACNA2D1**	-1.87	9.14E-14	7.97E-13

**MAMDC2**	-2.62	9.18E-14	8.01E-13
**TMCO3**	0.84	9.42E-14	8.21E-13
**RP11-47A8.5**	1.48	9.45E-14	8.24E-13
**C9orf72**	-1.04	9.50E-14	8.28E-13
**SLC2A8**	1.17	9.52E-14	8.30E-13
**ARHGAP31**	-0.79	9.53E-14	8.30E-13
**CD24P4**	1.93	9.68E-14	8.43E-13
**CC2D1A**	1.15	9.89E-14	8.61E-13
**MASP2**	-2.76	9.90E-14	8.62E-13
**PRMT9**	-0.80	9.93E-14	8.64E-13
**EMB**	-1.86	9.95E-14	8.65E-13
**DHX58**	1.27	9.96E-14	8.66E-13
**GRK6**	1.00	1.00E-13	8.70E-13
**ADAMTS12**	3.83	1.00E-13	8.72E-13
**TCF20**	-0.70	1.01E-13	8.75E-13
**TPD52L1**	1.22	1.01E-13	8.79E-13
**SPINK6**	6.49	1.02E-13	8.89E-13
**IMPDH1**	1.46	1.03E-13	8.93E-13
**GLMN**	-0.91	1.04E-13	9.04E-13
**EMID1**	3.06	1.05E-13	9.07E-13
**CACNA1A**	3.10	1.06E-13	9.16E-13
**RP1-68D18.2**	-3.27	1.06E-13	9.17E-13
**AP000350.5**	-9.28	1.06E-13	9.20E-13
**TIAM1**	-1.32	1.07E-13	9.25E-13
**TRIM36**	-2.17	1.07E-13	9.25E-13
**RP11-65I12.1**	-4.51	1.07E-13	9.28E-13
**KIAA0368**	-0.54	1.09E-13	9.43E-13
**AL109761.5**	-3.92	1.09E-13	9.43E-13
**TNS2**	2.06	1.09E-13	9.44E-13
**SNORA5A**	-5.07	1.09E-13	9.45E-13
**FAAP24**	1.68	1.09E-13	9.45E-13
**AC010468.1**	-2.82	1.10E-13	9.48E-13
**RP11-526D8.11**	-2.17	1.10E-13	9.48E-13
**ZDHHC11B**	-3.45	1.11E-13	9.61E-13
**CBLL1**	-0.90	1.12E-13	9.64E-13
**GMIP**	1.13	1.13E-13	9.76E-13
**RPP25L**	1.39	1.14E-13	9.84E-13
**EHD4**	1.28	1.14E-13	9.86E-13
**NTMT1**	1.10	1.14E-13	9.87E-13
**GIMAP6**	2.34	1.14E-13	9.88E-13
**CNTF**	-3.06	1.15E-13	9.96E-13
**HLX**	2.41	1.17E-13	1.01E-12
**B4GALNT2**	-4.58	1.17E-13	1.01E-12
**PDE12**	-0.86	1.17E-13	1.01E-12
**SLC25A22**	1.32	1.18E-13	1.02E-12

**RP11-539I5.1**	-1.55	1.20E-13	1.03E-12
**G30492**	-2.25	1.20E-13	1.03E-12
**METTL15P1**	-3.22	1.20E-13	1.03E-12
**RP11-685B24.1**	-8.02	1.22E-13	1.05E-12
**ZBTB1**	-0.77	1.22E-13	1.05E-12
**RPL3**	1.06	1.22E-13	1.05E-12
**ZMYND19**	1.02	1.25E-13	1.07E-12
**SLC38A9**	-1.04	1.25E-13	1.08E-12
**RP11-56B16.2**	-2.74	1.26E-13	1.08E-12
**HAGLR**	2.97	1.26E-13	1.08E-12
**PIAS1**	-0.78	1.27E-13	1.09E-12
**SRPR**	0.85	1.27E-13	1.09E-12
**AC073052.1**	-3.17	1.30E-13	1.12E-12
**C6orf226**	1.50	1.32E-13	1.13E-12
**RP1-197B17.3**	-2.58	1.32E-13	1.13E-12
**IKBKG**	1.22	1.34E-13	1.15E-12
**CTD-2336O2.1**	1.28	1.34E-13	1.15E-12
**PPP4R3B**	-0.85	1.35E-13	1.16E-12
**FNDC1**	5.27	1.36E-13	1.16E-12
**ANKRD13D**	1.44	1.36E-13	1.16E-12
**GPR22**	-4.83	1.37E-13	1.18E-12
**RCC1**	1.36	1.37E-13	1.18E-12
**MCM7**	0.80	1.37E-13	1.18E-12
**NAA38**	1.09	1.38E-13	1.18E-12
**FAM162B**	4.09	1.39E-13	1.19E-12
**TBC1D4**	-1.01	1.40E-13	1.19E-12
**RP9P**	1.15	1.40E-13	1.20E-12
**RNF25**	0.90	1.40E-13	1.20E-12
**ROM1**	1.51	1.41E-13	1.21E-12
**SP110**	1.23	1.41E-13	1.21E-12
**ZNF175**	-0.84	1.42E-13	1.21E-12
**RP11-175K6.2**	3.31	1.44E-13	1.23E-12
**RANBP10**	1.06	1.46E-13	1.25E-12
**ECSCR**	2.60	1.46E-13	1.25E-12
**RP11-630A13.4**	-2.28	1.46E-13	1.25E-12
**ACBD5**	-1.10	1.47E-13	1.26E-12
**RP11-707A18.1**	-4.75	1.48E-13	1.27E-12
**RP11-342K2.1**	-2.17	1.49E-13	1.27E-12
**SOX13**	2.33	1.49E-13	1.27E-12
**ELMO3**	1.30	1.51E-13	1.29E-12
**CLASP1**	-0.80	1.52E-13	1.30E-12
**IDUA**	2.13	1.53E-13	1.30E-12
**ACBD6**	0.66	1.54E-13	1.31E-12
**MFSD3**	1.58	1.54E-13	1.31E-12
**SNORA47**	-8.03	1.56E-13	1.33E-12

**CYLD**	-0.81	1.57E-13	1.33E-12
**FKBP10**	3.17	1.59E-13	1.35E-12
**EN1**	1.85	1.61E-13	1.37E-12
**KB-318B8.7**	-3.39	1.63E-13	1.39E-12
**RPS2**	1.16	1.65E-13	1.40E-12
**ERGIC3**	0.97	1.66E-13	1.41E-12
**POU5F2**	-4.14	1.66E-13	1.41E-12
**WDR24**	1.12	1.68E-13	1.43E-12
**PLEKHG3**	1.13	1.68E-13	1.43E-12
**MESDC2**	0.70	1.69E-13	1.43E-12
**TRPC4**	4.63	1.70E-13	1.44E-12
**MLLT6**	0.81	1.70E-13	1.44E-12
**SNORD46**	-5.60	1.70E-13	1.44E-12
**FMNL3**	2.04	1.71E-13	1.45E-12
**ZNF622**	1.03	1.72E-13	1.46E-12
**MTERF2**	-1.62	1.72E-13	1.46E-12
**KIAA2013**	0.97	1.73E-13	1.47E-12
**SMIM12**	0.80	1.74E-13	1.48E-12
**CRHR1-IT1**	-1.42	1.75E-13	1.49E-12
**KIAA0391**	-0.99	1.75E-13	1.49E-12
**CD7**	3.61	1.77E-13	1.50E-12
**RN7SKP116**	-3.17	1.78E-13	1.51E-12
**FES**	1.97	1.79E-13	1.51E-12
**NFATC4**	2.38	1.79E-13	1.51E-12
**CTU2**	1.24	1.80E-13	1.52E-12
**PSD**	1.90	1.80E-13	1.52E-12
**NPDC1**	1.72	1.80E-13	1.52E-12
**CLCA4**	-2.47	1.82E-13	1.54E-12
**METTL10**	-1.18	1.85E-13	1.56E-12
**VPS72**	0.92	1.85E-13	1.56E-12
**ANKS1B**	-2.06	1.86E-13	1.57E-12
**CFAP70**	-2.24	1.87E-13	1.58E-12
**SMOX**	2.42	1.87E-13	1.58E-12
**CEPT1**	-0.85	1.88E-13	1.58E-12
**EVA1A**	2.76	1.88E-13	1.59E-12
**AC002543.2**	-4.34	1.89E-13	1.60E-12
**PITPNA**	0.95	1.90E-13	1.60E-12
**LYPD6B**	-1.84	1.91E-13	1.61E-12
**C15orf48**	-2.82	1.93E-13	1.63E-12
**TMEM150A**	1.21	1.95E-13	1.64E-12
**RNF219**	-0.99	1.96E-13	1.65E-12
**PRX**	1.23	1.97E-13	1.66E-12
**GLMP**	1.13	1.97E-13	1.66E-12
**PRR29**	4.91	1.98E-13	1.67E-12
**RAD54L2**	-0.81	1.99E-13	1.67E-12

**FAM172A**	-0.88	2.00E-13	1.68E-12
**FAM8A1**	-0.93	2.00E-13	1.68E-12
**SCML2**	-1.64	2.00E-13	1.68E-12
**ENKD1**	1.43	2.01E-13	1.69E-12
**SYNGAP1**	1.80	2.02E-13	1.70E-12
**SCAF11**	-0.74	2.03E-13	1.70E-12
**UBE2L3**	0.81	2.03E-13	1.71E-12
**HCLS1**	2.60	2.06E-13	1.73E-12
**ECHS1**	0.96	2.08E-13	1.75E-12
**TBC1D10A**	1.27	2.09E-13	1.75E-12
**PKIB**	-2.25	2.09E-13	1.75E-12
**CNEP1R1**	-0.70	2.13E-13	1.79E-12
**LANCL1-AS1**	-3.09	2.15E-13	1.80E-12
**S100A16**	1.31	2.15E-13	1.80E-12
**ITGB5**	1.11	2.15E-13	1.80E-12
**DAZAP2**	1.00	2.15E-13	1.80E-12
**ESCO2**	-1.61	2.17E-13	1.82E-12
**DPP6**	-2.51	2.18E-13	1.82E-12
**HES6**	2.26	2.18E-13	1.83E-12
**ZNF248**	-1.48	2.18E-13	1.83E-12
**GRIPAP1**	0.75	2.23E-13	1.87E-12
**IFITM10**	1.82	2.25E-13	1.88E-12
**RP11-294J22.7**	-5.30	2.29E-13	1.91E-12
**PAN3**	-0.78	2.30E-13	1.92E-12
**UFD1L**	0.70	2.31E-13	1.93E-12
**ATG4D**	1.27	2.32E-13	1.94E-12
**ANKRD9**	1.28	2.32E-13	1.94E-12
**HDHD3**	1.08	2.34E-13	1.95E-12
**TOR3A**	0.95	2.34E-13	1.95E-12
**STARD8**	2.74	2.34E-13	1.95E-12
**CCDC141**	-4.19	2.36E-13	1.97E-12
**RPS17**	-2.28	2.37E-13	1.98E-12
**ALDH3B2**	1.68	2.37E-13	1.98E-12
**RPS23P8**	-2.11	2.39E-13	1.99E-12
**USP53**	-0.90	2.40E-13	2.00E-12
**IDH3B**	0.79	2.43E-13	2.02E-12
**AL161626.1**	4.87	2.46E-13	2.05E-12
**FAM20B**	-0.78	2.49E-13	2.07E-12
**PAPSS2**	2.46	2.53E-13	2.11E-12
**RP11-157K17.5**	-4.51	2.55E-13	2.12E-12
**RP11-888D10.4**	-2.69	2.55E-13	2.13E-12
**ADSSL1**	1.59	2.56E-13	2.13E-12
**NCOA7**	1.30	2.61E-13	2.17E-12
**RP5-1021I20.5**	-4.41	2.61E-13	2.17E-12
**TPTE2P5**	-2.51	2.62E-13	2.18E-12

**LENG9**	1.73	2.62E-13	2.18E-12
**MAFB**	1.58	2.64E-13	2.19E-12
**KRT18P7**	-4.05	2.65E-13	2.20E-12
**OTUD4**	-0.87	2.66E-13	2.21E-12
**ZNF567**	-1.14	2.66E-13	2.21E-12
**SIN3B**	1.44	2.67E-13	2.21E-12
**TGFBR3**	-1.50	2.71E-13	2.25E-12
**CD36**	3.29	2.77E-13	2.30E-12
**HIST1H4K**	-5.04	2.81E-13	2.33E-12
**CACUL1**	-0.70	2.83E-13	2.35E-12
**MAN1A2**	-0.92	2.83E-13	2.35E-12
**KCNK6**	1.26	2.86E-13	2.37E-12
**ULK1**	1.34	2.87E-13	2.37E-12
**CTA-941F9.10**	-2.69	2.87E-13	2.38E-12
**ALKBH2**	1.08	2.87E-13	2.38E-12
**RP11-434P11.1**	-4.20	2.89E-13	2.40E-12
**RGMA**	2.25	2.90E-13	2.40E-12
**VANGL1**	-0.66	2.92E-13	2.42E-12
**COL5A1**	3.23	2.93E-13	2.43E-12
**CYYR1**	2.33	2.94E-13	2.43E-12
**TWIST1**	2.89	2.96E-13	2.45E-12
**NBN**	-0.94	2.97E-13	2.45E-12
**ISOC2**	1.47	3.03E-13	2.51E-12
**EVL**	1.78	3.13E-13	2.58E-12
**RAB36**	1.52	3.13E-13	2.58E-12
**PEX16**	1.33	3.15E-13	2.60E-12
**GALNT10**	1.25	3.15E-13	2.60E-12
**ALPK3**	3.38	3.16E-13	2.61E-12
**GFM2**	-0.92	3.20E-13	2.64E-12
**CDC34**	1.04	3.22E-13	2.65E-12
**KAT7**	-0.73	3.25E-13	2.68E-12
**APH1A**	0.87	3.27E-13	2.70E-12
**MLLT4**	-0.88	3.27E-13	2.70E-12
**FBXO3**	-0.90	3.28E-13	2.70E-12
**TOMM40**	1.06	3.29E-13	2.71E-12
**SBSN**	1.72	3.30E-13	2.72E-12
**EXD3**	1.55	3.31E-13	2.72E-12
**SERPING1**	2.89	3.32E-13	2.73E-12
**SF3B5**	1.11	3.34E-13	2.75E-12
**C7orf49**	0.95	3.34E-13	2.75E-12
**EBPL**	1.40	3.35E-13	2.76E-12
**CCP110**	-0.89	3.37E-13	2.77E-12
**HERC3**	-0.99	3.41E-13	2.80E-12
**AZIN2**	2.09	3.49E-13	2.87E-12
**RP1-78B3.1**	-3.45	3.50E-13	2.87E-12

**ZC4H2**	1.32	3.50E-13	2.88E-12
**MIEN1**	0.87	3.51E-13	2.88E-12
**P2RX4**	1.72	3.54E-13	2.91E-12
**TUBB6**	1.28	3.54E-13	2.91E-12
**C9orf142**	1.06	3.56E-13	2.92E-12
**MAN1B1-AS1**	1.76	3.57E-13	2.93E-12
**RPS3**	1.39	3.57E-13	2.93E-12
**SVIP**	-1.42	3.61E-13	2.96E-12
**STARD3NL**	1.37	3.63E-13	2.98E-12
**COA3**	1.18	3.63E-13	2.98E-12
**TIMMDC1**	0.93	3.64E-13	2.98E-12
**RP1-34H18.1**	-3.15	3.65E-13	2.99E-12
**CEP57L1**	-1.13	3.70E-13	3.03E-12
**PIM1**	1.35	3.71E-13	3.04E-12
**G36217**	-4.46	3.72E-13	3.04E-12
**SNORA20**	-9.01	3.72E-13	3.05E-12
**GAS2L3**	-1.49	3.73E-13	3.05E-12
**SCYL2**	-0.86	3.80E-13	3.11E-12
**EFEMP2**	2.65	3.82E-13	3.12E-12
**IFI27L2**	1.83	3.82E-13	3.13E-12
**PRICKLE3**	1.22	3.85E-13	3.15E-12
**SPEG**	2.22	3.87E-13	3.16E-12
**CYP2W1**	-2.95	3.89E-13	3.18E-12
**TRIM11**	0.95	3.90E-13	3.18E-12
**ANK3**	-1.31	3.94E-13	3.22E-12
**CAMK2N1**	3.11	3.97E-13	3.24E-12
**RP11-405O10.2**	-5.26	3.97E-13	3.24E-12
**LEMD1**	4.12	3.97E-13	3.24E-12
**RAB3GAP2**	-0.71	4.00E-13	3.26E-12
**GNB1**	0.68	4.02E-13	3.28E-12
**H3F3A**	1.01	4.03E-13	3.29E-12
**ICK**	-0.80	4.05E-13	3.30E-12
**SCAMP4**	1.42	4.06E-13	3.31E-12
**LINC01422**	-3.10	4.07E-13	3.32E-12
**SRGN**	2.66	4.14E-13	3.37E-12
**CEP128**	-1.38	4.14E-13	3.37E-12
**TPCN1**	1.36	4.16E-13	3.38E-12
**PSMD10P1**	-4.97	4.16E-13	3.39E-12
**CDH20**	-2.14	4.18E-13	3.40E-12
**MOB3C**	1.06	4.20E-13	3.41E-12
**SDCBP2**	1.46	4.28E-13	3.48E-12
**IRF7**	1.70	4.29E-13	3.48E-12
**AC007383.3**	1.30	4.29E-13	3.48E-12
**SSTR2**	3.01	4.30E-13	3.49E-12
**EXOC8**	-1.27	4.34E-13	3.53E-12

**RP11-475O6.1**	-2.36	4.34E-13	3.53E-12
**NETO2**	2.70	4.34E-13	3.53E-12
**MEGF8**	1.00	4.37E-13	3.55E-12
**WDR47**	-1.18	4.39E-13	3.57E-12
**RAB3IL1**	3.17	4.44E-13	3.60E-12
**PSMB9**	1.73	4.49E-13	3.64E-12
**ENAH**	1.15	4.54E-13	3.68E-12
**DIAPH2**	-1.01	4.57E-13	3.71E-12
**STRN**	-1.07	4.59E-13	3.72E-12
**TBCA**	1.07	4.62E-13	3.74E-12
**SSFA2**	-1.27	4.63E-13	3.75E-12
**TUBB1**	-3.91	4.63E-13	3.75E-12
**BTBD7**	-0.75	4.68E-13	3.79E-12
**NEIL3**	-1.56	4.72E-13	3.82E-12
**PTGR1**	1.37	4.73E-13	3.83E-12
**AP001442.2**	-3.82	4.74E-13	3.83E-12
**AGL**	-0.89	4.76E-13	3.85E-12
**RP11-439E19.10**	-2.88	4.77E-13	3.86E-12
**GOLGA7B**	1.40	4.77E-13	3.86E-12
**MFAP2**	3.56	4.78E-13	3.86E-12
**AXIN1**	0.81	5.01E-13	4.05E-12
**EPM2AIP1**	-0.78	5.01E-13	4.05E-12
**MEIS3**	2.94	5.07E-13	4.09E-12
**ESR2**	-3.20	5.11E-13	4.12E-12
**CD300E**	4.57	5.12E-13	4.13E-12
**RNF113A**	1.07	5.13E-13	4.14E-12
**C2**	3.37	5.14E-13	4.15E-12
**BMF**	1.18	5.18E-13	4.18E-12
**MYO1B**	1.82	5.19E-13	4.19E-12
**MRGPRF**	2.44	5.21E-13	4.20E-12
**THAP5**	-1.14	5.25E-13	4.23E-12
**ZNF720**	-0.97	5.37E-13	4.33E-12
**C5AR1**	3.60	5.40E-13	4.35E-12
**GBP2**	1.22	5.50E-13	4.43E-12
**CD248**	2.79	5.51E-13	4.44E-12
**GRB7**	1.31	5.53E-13	4.45E-12
**FAM188A**	-1.03	5.59E-13	4.50E-12
**RP3-394A18.1**	-2.34	5.63E-13	4.53E-12
**ACPT**	-3.86	5.64E-13	4.54E-12
**RP11-133K1.8**	-7.37	5.65E-13	4.54E-12
**G35531**	-4.42	5.74E-13	4.62E-12
**RP11-373D23.2**	-3.30	5.81E-13	4.67E-12
**ZBTB6**	-1.24	5.81E-13	4.67E-12
**DHX30**	0.66	5.84E-13	4.69E-12
**CALML3**	2.68	5.85E-13	4.69E-12

**HIVEP2**	-1.23	5.90E-13	4.74E-12
**RP11-1336O20.2**	-8.06	5.91E-13	4.74E-12
**G35510**	-1.75	5.97E-13	4.79E-12
**GNA15**	1.21	5.97E-13	4.79E-12
**CTDSPL2**	-0.92	5.98E-13	4.79E-12
**TRPC4AP**	0.60	6.01E-13	4.82E-12
**RP4-673D20.3**	-3.14	6.01E-13	4.82E-12
**SNAI2**	1.11	6.07E-13	4.86E-12
**G40907**	-4.09	6.08E-13	4.87E-12
**RP11-305O6.4**	-4.39	6.11E-13	4.90E-12
**RP11-594N15.3**	-2.61	6.12E-13	4.90E-12
**SLC11A1**	3.23	6.13E-13	4.91E-12
**UHMK1**	-0.83	6.16E-13	4.93E-12
**BACH1**	-1.02	6.19E-13	4.95E-12
**PPM1L**	-1.08	6.25E-13	5.00E-12
**HSPA5**	1.23	6.32E-13	5.06E-12
**PRPF4B**	-0.70	6.36E-13	5.09E-12
**ZNF653**	1.27	6.44E-13	5.14E-12
**ZNF418**	-1.65	6.54E-13	5.23E-12
**EPOR**	1.86	6.56E-13	5.24E-12
**CHD8**	-0.77	6.56E-13	5.24E-12
**PRKCQ**	3.39	6.57E-13	5.24E-12
**NUP210L**	-3.97	6.65E-13	5.31E-12
**TMEM191A**	1.59	6.66E-13	5.31E-12
**ENPEP**	3.84	6.80E-13	5.43E-12
**RASGRF2**	2.24	6.82E-13	5.44E-12
**PIK3R4**	-0.77	6.88E-13	5.49E-12
**TKT**	1.36	6.97E-13	5.56E-12
**KRTCAP3**	1.56	6.99E-13	5.57E-12
**RP11-391L3.4**	-5.46	7.02E-13	5.60E-12
**ALG3**	1.07	7.14E-13	5.69E-12
**ENO2**	1.89	7.17E-13	5.71E-12
**MED28**	-0.57	7.19E-13	5.73E-12
**ZC3H14**	-0.64	7.23E-13	5.76E-12
**ZNF160**	-0.75	7.26E-13	5.78E-12
**OPA1**	-0.94	7.27E-13	5.78E-12
**FAM171A2**	1.69	7.29E-13	5.80E-12
**LIPE**	2.57	7.36E-13	5.85E-12
**DCTN1**	0.61	7.44E-13	5.91E-12
**HSPD1P11**	-5.62	7.45E-13	5.92E-12
**AP001469.5**	-3.47	7.54E-13	6.00E-12
**ZNF252P**	-1.04	7.55E-13	6.00E-12
**AFAP1L2**	1.69	7.55E-13	6.00E-12
**GSN-AS1**	-2.90	7.61E-13	6.04E-12
**RPS16**	2.10	7.70E-13	6.11E-12

**G19270**	-7.75	7.73E-13	6.14E-12
**ATIC**	1.32	7.79E-13	6.18E-12
**UCKL1**	0.80	7.92E-13	6.28E-12
**ZSWIM4**	1.25	7.97E-13	6.32E-12
**SIL1**	0.93	7.99E-13	6.34E-12
**HES2**	1.18	8.01E-13	6.35E-12
**BROX**	-1.10	8.05E-13	6.38E-12
**RPAP2**	-0.82	8.09E-13	6.41E-12
**LGMN**	1.74	8.18E-13	6.48E-12
**SHROOM2**	1.17	8.25E-13	6.54E-12
**RP11-337C18.8**	-2.41	8.27E-13	6.55E-12
**MIPOL1**	-1.28	8.27E-13	6.55E-12
**COL6A2**	2.87	8.28E-13	6.55E-12
**PANK4**	1.01	8.32E-13	6.59E-12
**RGL2**	0.93	8.34E-13	6.60E-12
**RP11-307C19.2**	-3.29	8.41E-13	6.65E-12
**KIRREL**	2.00	8.41E-13	6.65E-12
**DNAAF5**	0.79	8.49E-13	6.71E-12
**RUNX1**	2.60	8.54E-13	6.75E-12
**TBL2**	0.69	8.54E-13	6.75E-12
**RP11-511P7.5**	1.42	8.54E-13	6.75E-12
**PHYHIP**	-2.52	8.56E-13	6.76E-12
**NFKBIE**	1.32	8.56E-13	6.76E-12
**LNX1-AS1**	-5.22	8.69E-13	6.86E-12
**ARL6**	-1.13	8.74E-13	6.89E-12
**DNAJB2**	0.87	8.75E-13	6.90E-12
**SPATA24**	1.80	8.83E-13	6.96E-12
**USP3-AS1**	-3.61	8.84E-13	6.97E-12
**CHRDL2**	7.74	8.86E-13	6.99E-12
**AFAP1L1**	2.15	9.02E-13	7.11E-12
**MED7**	-0.93	9.09E-13	7.16E-12
**SLC26A11**	1.13	9.11E-13	7.17E-12
**E2F5**	-1.27	9.36E-13	7.37E-12
**ICAM2**	3.15	9.38E-13	7.39E-12
**SSR2**	0.98	9.43E-13	7.42E-12
**PBDC1**	0.95	9.45E-13	7.44E-12
**WTIP**	2.01	9.50E-13	7.47E-12
**NSMAF**	-0.66	9.62E-13	7.57E-12
**SENP3**	0.92	9.67E-13	7.61E-12
**GPATCH4**	1.26	9.78E-13	7.69E-12
**SSSCA1**	1.06	9.92E-13	7.80E-12
**PPP1R16B**	2.12	9.96E-13	7.83E-12
**TMEM14EP**	-5.65	9.99E-13	7.85E-12
**PAFAH1B1**	-0.62	1.00E-12	7.87E-12
**GLIS1**	2.15	1.01E-12	7.97E-12

**BNIP3**	1.82	1.02E-12	8.00E-12
**XPO1**	-0.78	1.03E-12	8.05E-12
**ZNRF3**	-1.12	1.03E-12	8.05E-12
**SAA2**	5.80	1.05E-12	8.22E-12
**OMA1**	-1.54	1.06E-12	8.29E-12
**C2orf49**	-0.78	1.06E-12	8.34E-12
**EPS8L2**	1.00	1.07E-12	8.40E-12
**NHP2**	1.15	1.08E-12	8.48E-12
**CA1**	-6.73	1.08E-12	8.48E-12
**DOCK5**	-1.05	1.08E-12	8.49E-12
**HSPA4L**	-1.42	1.09E-12	8.56E-12
**TMEM198**	2.62	1.10E-12	8.61E-12
**BTN2A1**	0.99	1.10E-12	8.61E-12
**ALS2**	-0.64	1.10E-12	8.63E-12
**AD000684.2**	-3.42	1.11E-12	8.65E-12
**RP1-179N16.3**	-3.48	1.11E-12	8.70E-12
**RP4-758J18.2**	1.09	1.12E-12	8.74E-12
**TRAPPC11**	-0.67	1.12E-12	8.78E-12
**GPR153**	1.63	1.12E-12	8.78E-12
**G35821**	-1.47	1.13E-12	8.79E-12
**FBXO25**	-0.83	1.13E-12	8.79E-12
**IL17RC**	0.91	1.13E-12	8.82E-12
**EXOC1**	-0.71	1.13E-12	8.84E-12
**MTERF1**	-0.70	1.13E-12	8.86E-12
**ESM1**	8.17	1.16E-12	9.03E-12
**SNORD60**	-8.65	1.17E-12	9.16E-12
**DGKH**	-1.15	1.18E-12	9.22E-12
**TMCO4**	1.21	1.19E-12	9.30E-12
**CTD-2349P21.3**	-4.99	1.19E-12	9.30E-12
**SLC35C2**	0.66	1.20E-12	9.39E-12
**SLC10A6**	-1.14	1.21E-12	9.42E-12
**ATP5O**	-0.97	1.22E-12	9.50E-12
**VWA8**	-0.89	1.22E-12	9.52E-12
**ZNF544**	-0.97	1.25E-12	9.72E-12
**CPXM2**	2.21	1.26E-12	9.78E-12
**SNORD3C**	-9.00	1.26E-12	9.81E-12
**FLYWCH1**	1.36	1.26E-12	9.82E-12
**H2AFX**	1.25	1.27E-12	9.85E-12
**RP11-819C21.1**	-1.63	1.30E-12	1.01E-11
**C1RL**	1.48	1.31E-12	1.02E-11
**SGSM3**	0.96	1.34E-12	1.04E-11
**TMEM144**	-1.02	1.34E-12	1.04E-11
**C10orf67**	-4.59	1.35E-12	1.05E-11
**HNRNPUL2**	0.81	1.35E-12	1.05E-11
**GALNT2**	1.12	1.35E-12	1.05E-11

**PHLDA2**	1.81	1.37E-12	1.06E-11
**KDM5B**	-0.76	1.37E-12	1.06E-11
**ZNF304**	-0.82	1.38E-12	1.07E-11
**ZNF548**	-0.92	1.38E-12	1.07E-11
**CAMKK2**	0.69	1.39E-12	1.07E-11
**LTB**	2.50	1.39E-12	1.08E-11
**SOCS7**	-0.71	1.39E-12	1.08E-11
**MT-ATP8**	3.23	1.39E-12	1.08E-11
**ANAPC2**	1.31	1.40E-12	1.08E-11
**CHCHD6**	1.03	1.40E-12	1.08E-11
**KCNAB1**	-1.47	1.41E-12	1.09E-11
**TAS2R30**	-7.70	1.41E-12	1.09E-11
**COL6A3**	3.24	1.42E-12	1.10E-11
**CLEC1A**	2.80	1.42E-12	1.10E-11
**CCHCR1**	1.06	1.43E-12	1.11E-11
**AP4S1**	-1.00	1.44E-12	1.11E-11
**PLEKHF1**	1.94	1.44E-12	1.12E-11
**SEMA3F**	1.00	1.47E-12	1.13E-11
**RP11-1263C18.1**	-3.06	1.47E-12	1.14E-11
**RP11-342K6.1**	-1.25	1.48E-12	1.14E-11
**NPM1P26**	-5.11	1.49E-12	1.15E-11
**PDIK1L**	-1.10	1.49E-12	1.15E-11
**RAB27B**	-1.66	1.50E-12	1.16E-11
**TDRD3**	-0.78	1.50E-12	1.16E-11
**TUT1**	1.37	1.51E-12	1.16E-11
**CLTB**	1.35	1.51E-12	1.17E-11
**ANKFY1**	-0.59	1.52E-12	1.17E-11
**SSBP2**	-0.98	1.52E-12	1.17E-11
**TMED1**	1.50	1.53E-12	1.18E-11
**DCAF13P3**	-4.40	1.54E-12	1.19E-11
**ANGEL1**	1.01	1.55E-12	1.20E-11
**UNC13D**	1.69	1.57E-12	1.21E-11
**PSMB4**	0.94	1.57E-12	1.21E-11
**TCF12**	-0.76	1.57E-12	1.21E-11
**MRPS7**	1.01	1.58E-12	1.22E-11
**CACNA1C**	2.54	1.59E-12	1.23E-11
**AL662800.1**	-2.37	1.60E-12	1.23E-11
**ZNF441**	-1.43	1.61E-12	1.24E-11
**SLPI**	1.79	1.64E-12	1.26E-11
**HENMT1**	1.49	1.64E-12	1.26E-11
**SUSD6**	0.88	1.64E-12	1.26E-11
**SLC9A7**	-1.46	1.65E-12	1.27E-11
**CYP4Z1**	-3.03	1.65E-12	1.27E-11
**RP11-29G8.3**	-3.89	1.66E-12	1.27E-11
**IL27RA**	2.09	1.66E-12	1.28E-11

**HAGHL**	2.48	1.67E-12	1.28E-11
**CALML5**	2.22	1.68E-12	1.29E-11
**MYO9B**	1.05	1.68E-12	1.29E-11
**C20orf196**	1.58	1.69E-12	1.29E-11
**CCDC39**	-2.00	1.69E-12	1.30E-11
**IL4I1**	4.52	1.69E-12	1.30E-11
**CLDN18**	-4.41	1.70E-12	1.30E-11
**RP5-821D11.7**	-1.07	1.71E-12	1.31E-11
**PLP1**	-2.04	1.72E-12	1.32E-11
**ANO7P1**	1.77	1.72E-12	1.32E-11
**SENP7**	-1.11	1.73E-12	1.33E-11
**RUNX3**	1.60	1.74E-12	1.33E-11
**HMGXB3**	0.63	1.74E-12	1.34E-11
**C17orf89**	1.14	1.75E-12	1.34E-11
**RP11-849H4.4**	-2.88	1.75E-12	1.34E-11
**BLNK**	1.53	1.77E-12	1.36E-11
**CXorf23**	-0.94	1.79E-12	1.37E-11
**ACTB**	1.03	1.79E-12	1.37E-11
**NOX4**	4.45	1.81E-12	1.38E-11
**SKIV2L**	0.80	1.81E-12	1.38E-11
**ZFP3**	-0.98	1.83E-12	1.40E-11
**RNF217-AS1**	-2.68	1.83E-12	1.40E-11
**DBX2**	-6.60	1.84E-12	1.40E-11
**RP11-65E22.2**	-3.30	1.84E-12	1.41E-11
**RMND5A**	-1.33	1.84E-12	1.41E-11
**TPP1**	1.37	1.85E-12	1.41E-11
**AC006460.2**	-3.95	1.86E-12	1.42E-11
**ZNF613**	-0.86	1.87E-12	1.43E-11
**ZNF879**	-1.13	1.87E-12	1.43E-11
**DMTF1**	-1.37	1.90E-12	1.45E-11
**ACTA2**	2.62	1.92E-12	1.46E-11
**DBNL**	0.91	1.92E-12	1.46E-11
**NSMCE2**	0.72	1.93E-12	1.47E-11
**AP000439.3**	-2.64	1.93E-12	1.47E-11
**HSD17B14**	2.59	1.95E-12	1.49E-11
**SLC52A2**	1.22	1.98E-12	1.51E-11
**SPRED2**	1.17	2.00E-12	1.52E-11
**PTBP1**	0.75	2.01E-12	1.53E-11
**POLQ**	-1.44	2.01E-12	1.53E-11
**SGIP1**	2.54	2.02E-12	1.53E-11
**XLOC_008613**	-5.40	2.02E-12	1.53E-11
**HSD3B1**	-10.02	2.05E-12	1.56E-11
**CRYZP1**	-4.48	2.05E-12	1.56E-11
**CERCAM**	2.97	2.05E-12	1.56E-11
**FANCL**	-0.97	2.06E-12	1.56E-11

**PCDH12**	2.90	2.07E-12	1.57E-11
**CYTL1**	3.37	2.11E-12	1.60E-11
**LL0XNC01-237H1.2**	-2.09	2.11E-12	1.60E-11
**FLJ37453**	1.30	2.15E-12	1.63E-11
**TMEM44**	2.38	2.15E-12	1.63E-11
**NPY5R**	-2.78	2.19E-12	1.66E-11
**POLR2G**	0.99	2.20E-12	1.67E-11
**FBXO33**	-0.81	2.20E-12	1.67E-11
**CLN6**	1.10	2.22E-12	1.69E-11
**PHB2**	0.85	2.23E-12	1.69E-11
**CPSF4**	0.78	2.24E-12	1.70E-11
**APEH**	0.86	2.25E-12	1.70E-11
**HAS3**	2.24	2.25E-12	1.71E-11
**C19orf33**	-3.22	2.28E-12	1.72E-11
**TIGD5**	1.00	2.28E-12	1.73E-11
**FOXP1**	0.77	2.32E-12	1.76E-11
**HSD3BP5**	-2.97	2.33E-12	1.76E-11
**DYNLRB1**	0.95	2.33E-12	1.77E-11
**PEX11G**	1.55	2.34E-12	1.77E-11
**SNHG6**	1.14	2.34E-12	1.77E-11
**ZNF876P**	-2.09	2.34E-12	1.77E-11
**RP11-504G3.4**	-3.75	2.35E-12	1.78E-11
**CEACAM19**	1.45	2.38E-12	1.80E-11
**FPGT-TNNI3K**	-3.31	2.38E-12	1.80E-11
**DOK1**	2.37	2.38E-12	1.80E-11
**CHUK**	-0.87	2.39E-12	1.80E-11
**FOXRED1**	1.20	2.40E-12	1.81E-11
**ZNHIT2**	1.13	2.41E-12	1.82E-11
**KRR1P1**	-4.78	2.41E-12	1.82E-11
**S100A9**	5.92	2.41E-12	1.82E-11
**CEP162**	-1.01	2.42E-12	1.83E-11
**AL133243.4**	-5.88	2.42E-12	1.83E-11
**OCA2**	-1.84	2.45E-12	1.85E-11
**ARF1**	0.86	2.45E-12	1.85E-11
**FJX1**	1.09	2.46E-12	1.85E-11
**SPICE1**	-0.91	2.46E-12	1.86E-11
**MEX3D**	1.00	2.46E-12	1.86E-11
**RP11-531F16.4**	-2.29	2.48E-12	1.86E-11
**RUNDC3A**	1.99	2.49E-12	1.88E-11
**PHC1**	1.53	2.51E-12	1.89E-11
**C1orf233**	1.76	2.51E-12	1.89E-11
**ZNF443**	-1.26	2.52E-12	1.90E-11
**C19orf54**	1.46	2.53E-12	1.90E-11
**DNAJB6**	0.95	2.55E-12	1.92E-11
**IGIP**	-1.07	2.58E-12	1.94E-11

**DCTN2**	0.77	2.60E-12	1.95E-11
**UBE2L6**	1.19	2.62E-12	1.97E-11
**ZNF687**	0.72	2.63E-12	1.97E-11
**MSC**	3.45	2.64E-12	1.98E-11
**CFAP36**	0.69	2.65E-12	1.99E-11
**FZD2**	3.94	2.66E-12	1.99E-11
**UBL4A**	0.80	2.69E-12	2.02E-11
**LINC00657**	-0.88	2.69E-12	2.02E-11
**CNN3**	1.63	2.70E-12	2.02E-11
**KIF17**	3.09	2.71E-12	2.03E-11
**SNORA63**	-3.77	2.71E-12	2.03E-11
**RP11-461L13.3**	-3.01	2.72E-12	2.04E-11
**CRKL**	-0.70	2.73E-12	2.05E-11
**AKR1B1**	1.61	2.74E-12	2.05E-11
**ASTE1**	-0.76	2.74E-12	2.05E-11
**KRT37**	3.97	2.75E-12	2.06E-11
**STK31**	-3.99	2.78E-12	2.08E-11
**RGMB-AS1**	-2.09	2.80E-12	2.10E-11
**DANT2**	-3.19	2.80E-12	2.10E-11
**EBF2**	4.47	2.81E-12	2.11E-11
**RP5-1014D13.2**	-2.10	2.83E-12	2.12E-11
**NUP43**	-1.06	2.84E-12	2.12E-11
**DDOST**	0.90	2.87E-12	2.14E-11
**SHOC2**	-0.77	2.87E-12	2.15E-11
**RTF1**	0.77	2.88E-12	2.15E-11
**PALLD**	1.52	2.90E-12	2.17E-11
**C1QA**	4.01	2.90E-12	2.17E-11
**CH17-76K2.6**	-4.67	2.93E-12	2.18E-11
**MARK3**	0.53	2.93E-12	2.19E-11
**SSR4**	1.07	2.93E-12	2.19E-11
**CNTNAP3**	-2.48	2.99E-12	2.23E-11
**SNHG14**	-1.26	3.00E-12	2.24E-11
**RNY5**	-9.52	3.00E-12	2.24E-11
**SDHA**	0.92	3.01E-12	2.25E-11
**HSPA12B**	2.47	3.06E-12	2.28E-11
**CAND1**	-0.85	3.06E-12	2.28E-11
**NDUFS1**	-0.93	3.08E-12	2.29E-11
**KLC2**	1.25	3.12E-12	2.33E-11
**KIAA1549L**	-1.72	3.14E-12	2.34E-11
**OSGIN1**	3.19	3.18E-12	2.37E-11
**RAB22A**	-0.71	3.21E-12	2.39E-11
**C4B**	4.91	3.22E-12	2.40E-11
**ZNF257**	-2.52	3.23E-12	2.41E-11
**FREM2**	-2.75	3.24E-12	2.41E-11
**JMJD7**	-3.23	3.27E-12	2.43E-11

**TSPAN12**	2.44	3.30E-12	2.45E-11
**USP47**	-0.78	3.30E-12	2.45E-11
**MRPS12**	1.05	3.31E-12	2.46E-11
**MEPCE**	1.00	3.32E-12	2.46E-11
**PANX2**	3.38	3.32E-12	2.47E-11
**ZNF606**	-1.03	3.34E-12	2.48E-11
**WNT5A**	1.99	3.38E-12	2.51E-11
**OR51E1**	6.53	3.42E-12	2.54E-11
**PCDH17**	3.49	3.43E-12	2.54E-11
**TOR1A**	0.76	3.43E-12	2.54E-11
**ST7L**	-1.03	3.43E-12	2.54E-11
**HOXC6**	1.39	3.49E-12	2.59E-11
**MRPL34**	1.16	3.50E-12	2.59E-11
**RP11-448G15.3**	-2.29	3.52E-12	2.61E-11
**DGAT2L6**	-10.05	3.52E-12	2.61E-11
**RP11-133K1.9**	-3.13	3.52E-12	2.61E-11
**QRSL1P3**	-5.08	3.54E-12	2.63E-11
**KLHDC4**	1.05	3.61E-12	2.67E-11
**FN3KRP**	1.11	3.61E-12	2.67E-11
**RP11-394B2.5**	-3.05	3.64E-12	2.69E-11
**PMS2P1**	0.84	3.64E-12	2.69E-11
**ASPSCR1**	1.10	3.67E-12	2.71E-11
**DPP8**	-0.80	3.67E-12	2.72E-11
**TDO2**	6.16	3.69E-12	2.73E-11
**RP11-115D19.1**	-2.90	3.70E-12	2.73E-11
**LINC00888**	2.32	3.71E-12	2.74E-11
**TRIM65**	0.85	3.71E-12	2.74E-11
**TOLLIP**	1.11	3.76E-12	2.78E-11
**MTMR7**	-1.73	3.76E-12	2.78E-11
**COPZ2**	1.49	3.77E-12	2.79E-11
**CCL5**	2.97	3.78E-12	2.79E-11
**GCAT**	1.37	3.79E-12	2.79E-11
**CD40**	1.44	3.80E-12	2.81E-11
**RN7SKP71**	-7.33	3.81E-12	2.81E-11
**UBE2D2**	0.79	3.82E-12	2.82E-11
**IQSEC1**	1.48	3.84E-12	2.84E-11
**MSI2**	-0.70	3.87E-12	2.85E-11
**STYX**	-1.08	3.89E-12	2.87E-11
**FCMR**	3.34	3.90E-12	2.87E-11
**TM4SF1**	2.48	3.90E-12	2.88E-11
**S100A4**	2.01	3.91E-12	2.88E-11
**CLEC12B**	-2.85	3.91E-12	2.88E-11
**TTC21B-AS1**	-4.45	3.99E-12	2.94E-11
**PTCD2**	-0.79	4.02E-12	2.96E-11
**AIFM2**	1.78	4.02E-12	2.96E-11

**KRBA1**	1.38	4.03E-12	2.97E-11
**LINC01550**	-1.30	4.05E-12	2.98E-11
**ZNF346**	0.71	4.07E-12	2.99E-11
**LAMP1**	0.77	4.10E-12	3.01E-11
**RP13-923O23.7**	-2.64	4.11E-12	3.02E-11
**TGM4**	-3.62	4.13E-12	3.04E-11
**RALGAPA2**	-0.98	4.15E-12	3.05E-11
**FDXR**	1.24	4.17E-12	3.06E-11
**SEMA6D**	1.88	4.19E-12	3.08E-11
**FLT1**	2.17	4.21E-12	3.09E-11
**EPHA5**	-2.55	4.25E-12	3.12E-11
**SAE1**	0.83	4.25E-12	3.12E-11
**POLDIP3**	0.57	4.25E-12	3.12E-11
**SLC12A7**	1.44	4.26E-12	3.12E-11
**C12orf45**	-1.01	4.26E-12	3.13E-11
**ZFYVE19**	0.95	4.27E-12	3.13E-11
**TACC3**	1.33	4.27E-12	3.13E-11
**C9orf131**	-4.80	4.27E-12	3.13E-11
**FAM162A**	1.08	4.28E-12	3.14E-11
**ZMYM6**	-0.72	4.34E-12	3.18E-11
**MTHFD1L**	1.73	4.34E-12	3.18E-11
**DLG2**	-1.27	4.35E-12	3.19E-11
**ZNF799**	-0.87	4.37E-12	3.20E-11
**UBL5**	1.20	4.38E-12	3.20E-11
**TAOK2**	0.87	4.38E-12	3.21E-11
**DHRS3**	0.91	4.41E-12	3.23E-11
**G1562**	-5.62	4.42E-12	3.23E-11
**ZNF776**	-0.98	4.43E-12	3.24E-11
**FCGR2A**	2.99	4.43E-12	3.24E-11
**ARL5B**	-1.11	4.44E-12	3.25E-11
**TTC5**	-0.76	4.45E-12	3.26E-11
**TXNDC12**	0.87	4.50E-12	3.29E-11
**SLC7A6**	-1.15	4.52E-12	3.30E-11
**FERMT3**	2.35	4.54E-12	3.32E-11
**MYCT1**	2.23	4.55E-12	3.32E-11
**FTOP1**	-2.45	4.59E-12	3.35E-11
**HIRA**	0.81	4.59E-12	3.35E-11
**ACTR1A**	0.71	4.67E-12	3.41E-11
**CSNK1G1**	-0.56	4.69E-12	3.42E-11
**PPIE**	0.73	4.73E-12	3.45E-11
**SCEL-AS1**	-7.57	4.74E-12	3.46E-11
**ELP2**	-0.68	4.74E-12	3.46E-11
**C1QB**	3.99	4.80E-12	3.50E-11
**NAA35**	-0.70	4.82E-12	3.51E-11
**LIMD2**	2.13	4.88E-12	3.56E-11

**RP11-515O17.2**	-7.34	4.92E-12	3.58E-11
**PTCD3**	-0.67	4.93E-12	3.59E-11
**AF131215.5**	-4.92	4.94E-12	3.60E-11
**CIRH1A**	1.02	5.03E-12	3.66E-11
**RPL10P3**	-3.27	5.06E-12	3.68E-11
**TRIM41**	0.92	5.09E-12	3.71E-11
**C9orf114**	0.85	5.11E-12	3.71E-11
**HIF1A**	1.29	5.12E-12	3.72E-11
**GLCCI1**	-1.06	5.13E-12	3.73E-11
**VARS**	0.94	5.15E-12	3.75E-11
**FAM184B**	-2.17	5.18E-12	3.76E-11
**RNF166**	1.48	5.21E-12	3.78E-11
**PPP2R3A**	-1.09	5.28E-12	3.84E-11
**NDFIP2**	-1.40	5.28E-12	3.84E-11
**RP1-239B22.5**	-2.09	5.29E-12	3.84E-11
**CDH6**	2.70	5.34E-12	3.88E-11
**RP11-50D9.3**	-3.58	5.37E-12	3.90E-11
**RP11-379B18.5**	-2.16	5.38E-12	3.90E-11
**PSME1**	0.93	5.43E-12	3.94E-11
**SPATA6**	-0.82	5.44E-12	3.95E-11
**ZNF430**	-0.91	5.51E-12	3.99E-11
**CTD-2619J13.17**	-1.91	5.55E-12	4.02E-11
**LINC00152**	3.40	5.63E-12	4.08E-11
**MT-ND4**	4.16	5.66E-12	4.10E-11
**DTNB**	1.11	5.67E-12	4.10E-11
**FRMD5**	-2.49	5.67E-12	4.11E-11
**CASKIN2**	1.32	5.69E-12	4.12E-11
**RASD2**	3.80	5.79E-12	4.19E-11
**TBC1D1**	1.28	5.82E-12	4.21E-11
**NEU3**	-0.89	5.84E-12	4.23E-11
**FRMD6-AS2**	-3.51	5.87E-12	4.25E-11
**SNORA26**	-4.35	5.89E-12	4.26E-11
**ARRDC3-AS1**	-1.87	5.98E-12	4.32E-11
**RP11-127L20.3**	-2.74	5.98E-12	4.32E-11
**AC073046.25**	-1.94	6.09E-12	4.40E-11
**CMTM4**	-0.98	6.10E-12	4.41E-11
**SDHC**	0.61	6.17E-12	4.46E-11
**AC074286.1**	-1.87	6.18E-12	4.46E-11
**TMF1**	-0.79	6.20E-12	4.48E-11
**C1orf54**	2.36	6.22E-12	4.49E-11
**LAPTM4A**	1.06	6.28E-12	4.53E-11
**TPRG1L**	0.96	6.34E-12	4.57E-11
**DGCR11**	-1.55	6.35E-12	4.58E-11
**G18355**	-7.78	6.37E-12	4.60E-11
**ADCY6**	1.26	6.41E-12	4.62E-11

**ABCA11P**	-2.47	6.45E-12	4.65E-11
**KIAA1429**	-0.75	6.45E-12	4.65E-11
**HNRNPL**	0.66	6.47E-12	4.66E-11
**HIST2H2BE**	-1.47	6.48E-12	4.67E-11
**AF127577.13**	-6.13	6.49E-12	4.68E-11
**GLTSCR2**	1.27	6.49E-12	4.68E-11
**BRF1**	1.05	6.52E-12	4.69E-11
**THRSP**	-7.17	6.60E-12	4.75E-11
**TBC1D12**	-0.89	6.62E-12	4.76E-11
**S100A8**	5.67	6.70E-12	4.82E-11
**MGRN1**	1.33	6.73E-12	4.84E-11
**SMIM10**	2.24	6.76E-12	4.86E-11
**HECTD4**	-1.14	6.78E-12	4.88E-11
**SLC16A2**	1.76	6.78E-12	4.88E-11
**TXNL4B**	0.87	6.81E-12	4.90E-11
**ARL16**	1.21	6.83E-12	4.90E-11
**UBE2V1**	-2.10	6.84E-12	4.92E-11
**SNORA42**	-6.20	6.86E-12	4.93E-11
**ZNF587**	-1.23	6.87E-12	4.93E-11
**RP11-893F2.18**	-3.06	6.91E-12	4.96E-11
**LRRC8B**	-1.53	6.91E-12	4.96E-11
**RP11-712P20.2**	-7.42	6.94E-12	4.98E-11
**EEF1B2P6**	-2.41	6.94E-12	4.98E-11
**BAZ2B**	-0.89	6.96E-12	4.99E-11
**MAP3K7**	-0.57	6.98E-12	5.00E-11
**CUTA**	0.91	7.01E-12	5.02E-11
**RP11-98L5.5**	-5.01	7.01E-12	5.03E-11
**CNST**	-1.10	7.07E-12	5.06E-11
**MED24**	0.72	7.12E-12	5.10E-11
**RP11-507K2.2**	-3.62	7.12E-12	5.10E-11
**ATP6V1A**	-0.93	7.18E-12	5.14E-11
**LRRC7**	-2.24	7.18E-12	5.14E-11
**FAM171A1**	1.95	7.22E-12	5.17E-11
**C9orf40**	1.05	7.34E-12	5.25E-11
**FBXO6**	1.05	7.34E-12	5.25E-11
**CAP2P1**	-3.40	7.40E-12	5.29E-11
**MTSS1L**	1.07	7.42E-12	5.30E-11
**MBD3**	0.94	7.46E-12	5.33E-11
**TM2D2**	1.04	7.47E-12	5.34E-11
**VPS16**	0.79	7.55E-12	5.40E-11
**SGK3**	-1.33	7.55E-12	5.40E-11
**PDSS1P1**	-7.53	7.65E-12	5.46E-11
**GPX3**	3.50	7.68E-12	5.49E-11
**ZNF385A**	2.26	7.71E-12	5.51E-11
**RP11-16P6.1**	-1.90	7.74E-12	5.53E-11

**AF131215.2**	-1.26	7.78E-12	5.55E-11
**RBPMS**	1.70	7.78E-12	5.55E-11
**NAALADL1**	2.06	7.79E-12	5.56E-11
**MYCBP2**	-1.33	7.81E-12	5.57E-11
**RBM17**	0.53	7.82E-12	5.58E-11
**G22259**	-2.14	7.83E-12	5.58E-11
**RP11-500G22.5**	-3.02	7.91E-12	5.64E-11
**RP11-864J10.4**	-3.00	7.92E-12	5.64E-11
**TTPAL**	-1.25	7.95E-12	5.66E-11
**INF2**	1.54	8.06E-12	5.74E-11
**HNRNPA1**	1.05	8.17E-12	5.81E-11
**BACH1-IT2**	-2.78	8.17E-12	5.82E-11
**BBC3**	1.22	8.17E-12	5.82E-11
**UNC5A**	5.42	8.19E-12	5.83E-11
**EWSR1**	0.61	8.19E-12	5.83E-11
**TSR2**	0.88	8.25E-12	5.87E-11
**RP11-454F8.2**	-3.39	8.26E-12	5.87E-11
**RP11-160H12.2**	-5.32	8.29E-12	5.89E-11
**MAB21L2**	-7.55	8.31E-12	5.90E-11
**RNF44**	1.37	8.41E-12	5.98E-11
**CDH11**	3.25	8.42E-12	5.98E-11
**RP11-588K22.2**	2.92	8.44E-12	6.00E-11
**ARPP19**	-0.81	8.50E-12	6.03E-11
**BMP3**	-5.08	8.51E-12	6.04E-11
**NAA16**	-0.76	8.55E-12	6.07E-11
**LEPR**	-2.29	8.68E-12	6.16E-11
**ZFP36L2**	0.98	8.74E-12	6.20E-11
**MT-ND3**	1.79	8.74E-12	6.20E-11
**IRF3**	1.35	8.76E-12	6.21E-11
**GOLGA6L5P**	2.62	8.81E-12	6.24E-11
**CEP89**	0.62	8.96E-12	6.35E-11
**RCN1**	1.65	9.01E-12	6.39E-11
**DUSP19**	-1.50	9.04E-12	6.41E-11
**CDC14B**	1.09	9.07E-12	6.43E-11
**MIR210HG**	1.83	9.11E-12	6.45E-11
**RBM10**	0.71	9.11E-12	6.45E-11
**AC063976.7**	-3.32	9.26E-12	6.55E-11
**C11orf24**	1.41	9.29E-12	6.58E-11
**MICALL2**	2.08	9.29E-12	6.58E-11
**CMTM6**	-1.08	9.31E-12	6.59E-11
**NAPG**	-0.76	9.39E-12	6.64E-11
**RP11-563E2.2**	-4.23	9.58E-12	6.77E-11
**KIF13B**	-1.01	9.61E-12	6.79E-11
**CITED2**	1.02	9.64E-12	6.81E-11
**ASB3**	-2.06	9.77E-12	6.90E-11

**GPRC5B**	2.00	9.77E-12	6.90E-11
**PLXNB2**	0.86	9.78E-12	6.91E-11
**RP11-620J15.1**	-7.55	9.82E-12	6.94E-11
**A4GALT**	2.43	9.83E-12	6.94E-11
**MPDU1**	0.79	9.95E-12	7.02E-11
**SLC9A6**	-0.79	9.95E-12	7.02E-11
**HNRNPAB**	0.95	1.00E-11	7.07E-11
**IGSF10**	-3.04	1.01E-11	7.09E-11
**RASGRP1**	-1.34	1.01E-11	7.10E-11
**ADSL**	0.75	1.01E-11	7.10E-11
**SCAMP1-AS1**	1.30	1.01E-11	7.13E-11
**ELL**	0.83	1.02E-11	7.17E-11
**FADS3**	3.12	1.05E-11	7.37E-11
**HDAC7**	1.19	1.05E-11	7.39E-11
**CHRM5**	-2.96	1.05E-11	7.39E-11
**RAB43**	2.44	1.05E-11	7.42E-11
**DGUOK**	0.62	1.06E-11	7.45E-11
**C9orf64**	1.16	1.06E-11	7.46E-11
**LRWD1**	1.27	1.06E-11	7.47E-11
**CNTN2**	-2.83	1.06E-11	7.48E-11
**EGF**	-2.15	1.07E-11	7.51E-11
**DOK3**	2.53	1.08E-11	7.59E-11
**ADPRH**	2.19	1.08E-11	7.59E-11
**SYMPK**	1.14	1.08E-11	7.61E-11
**MTBP**	-1.01	1.09E-11	7.64E-11
**ZC3H6**	-1.02	1.09E-11	7.69E-11
**NECAB3**	1.38	1.09E-11	7.69E-11
**COX6A1**	-0.91	1.09E-11	7.69E-11
**RP11-65L3.3**	-4.61	1.11E-11	7.77E-11
**SNORA2A**	-5.92	1.11E-11	7.79E-11
**SDCCAG3**	0.97	1.11E-11	7.80E-11
**SMG1P1**	-2.26	1.11E-11	7.80E-11
**HSD17B10**	1.13	1.13E-11	7.92E-11
**HPRT1**	1.27	1.13E-11	7.95E-11
**RNGTT**	-0.83	1.14E-11	8.00E-11
**FAM110B**	2.50	1.14E-11	8.01E-11
**RP5-1187M17.10**	2.19	1.14E-11	8.01E-11
**AGR3**	-8.35	1.14E-11	8.02E-11
**DCAF5**	0.57	1.15E-11	8.02E-11
**XLOC_000992**	-4.23	1.16E-11	8.09E-11
**LIN54**	-1.00	1.16E-11	8.10E-11
**CLCN7**	1.02	1.18E-11	8.23E-11
**SNORA74**	-7.05	1.18E-11	8.25E-11
**RP11-582E3.4**	-3.08	1.20E-11	8.41E-11
**MAN2B1**	1.14	1.21E-11	8.43E-11

**PRMT2**	0.74	1.21E-11	8.45E-11
**DNA2**	-1.11	1.21E-11	8.46E-11
**EMP2**	-1.12	1.24E-11	8.63E-11
**DNMBP-AS1**	-3.03	1.24E-11	8.65E-11
**OGFOD2**	-2.90	1.24E-11	8.66E-11
**CLDN16**	-1.65	1.25E-11	8.74E-11
**DIS3**	-0.78	1.25E-11	8.76E-11
**CCDC150P1**	-2.81	1.25E-11	8.76E-11
**PI3**	4.85	1.26E-11	8.78E-11
**ZNF90**	-1.43	1.26E-11	8.83E-11
**SMAD1**	0.86	1.27E-11	8.84E-11
**PDGFA**	1.31	1.28E-11	8.92E-11
**COX7A1**	2.66	1.28E-11	8.92E-11
**ARRDC1**	1.31	1.29E-11	9.01E-11
**REEP3**	-1.13	1.29E-11	9.02E-11
**ZNF524**	1.01	1.30E-11	9.05E-11
**PMS1**	-0.95	1.30E-11	9.07E-11
**PKIA-AS1**	-4.11	1.31E-11	9.12E-11
**NPY1R**	-2.32	1.31E-11	9.12E-11
**TNK2**	1.30	1.32E-11	9.18E-11
**ZXDA**	-1.10	1.32E-11	9.18E-11
**RPL18AP3**	1.13	1.33E-11	9.28E-11
**MARK1**	0.93	1.34E-11	9.33E-11
**ATP1B3-AS1**	-2.96	1.34E-11	9.34E-11
**COBLL1**	-1.06	1.35E-11	9.37E-11
**CCDC96**	1.66	1.36E-11	9.49E-11
**RP11-477D19.2**	-3.04	1.37E-11	9.51E-11
**KANTR**	-2.22	1.37E-11	9.51E-11
**TP73**	1.26	1.37E-11	9.51E-11
**KIF2A**	-0.76	1.37E-11	9.52E-11
**AC009505.2**	-3.75	1.37E-11	9.53E-11
**CLIC1**	0.91	1.38E-11	9.56E-11
**CPPED1**	0.93	1.38E-11	9.60E-11
**FOSL1**	2.81	1.38E-11	9.61E-11
**SAYSD1**	0.80	1.39E-11	9.63E-11
**YTHDF1**	0.64	1.39E-11	9.66E-11
**RP11-996F15.6**	-2.41	1.41E-11	9.80E-11
**NUDT5**	-0.59	1.42E-11	9.83E-11
**PWWP2B**	1.20	1.42E-11	9.83E-11
**ANP32A**	1.20	1.42E-11	9.84E-11
**RP1-30M3.6**	-2.64	1.42E-11	9.84E-11
**PRDM10**	-0.81	1.42E-11	9.84E-11
**IL6**	8.12	1.42E-11	9.87E-11
**HCFC1R1**	0.98	1.43E-11	9.91E-11
**PI4K2A**	0.84	1.43E-11	9.92E-11

**CLTC**	-0.79	1.43E-11	9.92E-11
**CPEB3**	-1.05	1.44E-11	9.95E-11
**NLGN1**	-1.87	1.46E-11	1.01E-10
**SLC2A6**	2.63	1.46E-11	1.01E-10
**COL1A1**	4.02	1.46E-11	1.01E-10
**COQ9**	0.77	1.46E-11	1.01E-10
**C1orf112**	-1.18	1.48E-11	1.02E-10
**FAT2**	-1.13	1.48E-11	1.02E-10
**DUSP15**	4.47	1.48E-11	1.02E-10
**ULK4**	-1.53	1.49E-11	1.03E-10
**GUCY1A3**	2.51	1.49E-11	1.03E-10
**C15orf39**	1.33	1.50E-11	1.04E-10
**CTC-448F2.4**	-7.06	1.51E-11	1.04E-10
**ZFYVE28**	1.20	1.51E-11	1.04E-10
**FAM102A**	1.40	1.51E-11	1.04E-10
**CTD-2382E5.2**	-3.53	1.53E-11	1.06E-10
**HID1**	2.14	1.53E-11	1.06E-10
**ZFPM1**	1.26	1.54E-11	1.06E-10
**KIF1A**	-2.96	1.55E-11	1.07E-10
**NELFCD**	0.61	1.56E-11	1.08E-10
**HABP4**	1.36	1.57E-11	1.08E-10
**ZNF618**	1.26	1.58E-11	1.09E-10
**ALG6**	-0.68	1.59E-11	1.09E-10
**IPP**	-0.80	1.59E-11	1.10E-10
**ARNTL**	-1.96	1.61E-11	1.11E-10
**G32140**	-2.91	1.61E-11	1.11E-10
**C9orf69**	0.87	1.61E-11	1.11E-10
**PAPD4**	-0.68	1.61E-11	1.11E-10
**HCN2**	1.97	1.62E-11	1.11E-10
**RP13-516M14.1**	2.21	1.62E-11	1.12E-10
**CTC-251D13.1**	-2.33	1.63E-11	1.12E-10
**ARAP1**	1.02	1.63E-11	1.12E-10
**SRFBP1**	-0.94	1.63E-11	1.12E-10
**RP11-252C24.3**	-7.48	1.63E-11	1.12E-10
**PUS1**	1.32	1.63E-11	1.12E-10
**RP11-1069G10.1**	-4.46	1.65E-11	1.13E-10
**FOXP1-IT1**	-3.13	1.65E-11	1.13E-10
**TAF6L**	0.91	1.65E-11	1.13E-10
**POU2F3**	-1.55	1.65E-11	1.14E-10
**CTD-2528L19.6**	-1.71	1.66E-11	1.14E-10
**RP11-57H12.5**	-3.27	1.66E-11	1.14E-10
**TAF2**	-0.57	1.66E-11	1.14E-10
**APPBP2**	-0.79	1.68E-11	1.15E-10
**NDC1**	-1.01	1.69E-11	1.16E-10
**CRLF3**	-0.76	1.69E-11	1.16E-10

**RP11-631M6.2**	3.30	1.69E-11	1.16E-10
**RPS6KA1**	0.83	1.69E-11	1.16E-10
**LDB3**	1.83	1.69E-11	1.16E-10
**NUDT13**	-1.41	1.70E-11	1.17E-10
**CCNE2**	-1.14	1.71E-11	1.17E-10
**RPL7P9**	1.41	1.72E-11	1.18E-10
**AC093110.3**	-2.27	1.72E-11	1.18E-10
**RRNAD1**	0.80	1.73E-11	1.18E-10
**MRPL24**	0.86	1.73E-11	1.18E-10
**ARMCX6**	1.23	1.73E-11	1.19E-10
**KIAA0930**	0.73	1.74E-11	1.19E-10
**PRND**	7.40	1.75E-11	1.20E-10
**LRRC37A15P**	-3.10	1.76E-11	1.20E-10
**ZNF600**	-1.11	1.76E-11	1.21E-10
**ZNF302**	-0.91	1.77E-11	1.21E-10
**RP11-343L5.2**	-3.08	1.77E-11	1.21E-10
**ATG14**	-0.80	1.78E-11	1.21E-10
**LSM14B**	0.57	1.79E-11	1.23E-10
**SCO1**	-0.51	1.83E-11	1.25E-10
**FBXO28**	-1.15	1.83E-11	1.25E-10
**MDGA2**	-2.33	1.84E-11	1.26E-10
**NSMCE1**	1.01	1.85E-11	1.26E-10
**KCTD21**	1.47	1.85E-11	1.26E-10
**SLC39A9**	-0.63	1.85E-11	1.27E-10
**SLC25A5**	1.03	1.86E-11	1.27E-10
**HSP90B2P**	-3.81	1.88E-11	1.28E-10
**SLC9A1**	1.73	1.88E-11	1.28E-10
**IREB2**	-0.75	1.89E-11	1.29E-10
**LY6G6C**	1.96	1.91E-11	1.30E-10
**SYVN1**	1.23	1.92E-11	1.31E-10
**KIF19**	4.97	1.93E-11	1.31E-10
**SMAD1-AS2**	-7.18	1.93E-11	1.32E-10
**CBL**	-0.84	1.94E-11	1.32E-10
**G10489**	-7.55	1.94E-11	1.32E-10
**PPP1R37**	0.87	1.95E-11	1.33E-10
**IL31RA**	-3.41	1.95E-11	1.33E-10
**RAPGEF2**	-0.69	1.95E-11	1.33E-10
**G40351**	-2.60	1.96E-11	1.33E-10
**SNORA14A**	-7.62	1.96E-11	1.33E-10
**ZDHHC18**	0.79	1.97E-11	1.34E-10
**GAK**	0.65	1.97E-11	1.34E-10
**THRA**	0.92	1.98E-11	1.34E-10
**BCHE**	-1.94	2.01E-11	1.37E-10
**ELAVL1**	0.51	2.01E-11	1.37E-10
**SPECC1L**	1.05	2.02E-11	1.37E-10

**SLK**	-0.80	2.02E-11	1.38E-10
**MLANA**	-2.13	2.04E-11	1.38E-10
**RP5-940J5.6**	-2.82	2.05E-11	1.39E-10
**CCAR1**	0.77	2.06E-11	1.40E-10
**ST3GAL4**	1.58	2.06E-11	1.40E-10
**VAT1L**	5.07	2.10E-11	1.43E-10
**RIC3**	-1.74	2.10E-11	1.43E-10
**TMEM101**	0.90	2.13E-11	1.45E-10
**SLC27A1**	1.31	2.13E-11	1.45E-10
**RP11-973N13.4**	-3.46	2.14E-11	1.45E-10
**TAF4B**	-1.07	2.16E-11	1.47E-10
**HOXD8**	2.13	2.18E-11	1.48E-10
**RAP2B**	0.89	2.21E-11	1.50E-10
**PMVK**	1.52	2.22E-11	1.50E-10
**ZNF607**	-1.07	2.22E-11	1.51E-10
**RP11-96H17.1**	-3.33	2.23E-11	1.51E-10
**CTBP2P8**	-3.79	2.24E-11	1.51E-10
**ARHGAP4**	2.38	2.26E-11	1.53E-10
**PGAP3**	0.73	2.26E-11	1.53E-10
**NAA30**	-0.95	2.27E-11	1.54E-10
**IFT80**	-1.03	2.28E-11	1.54E-10
**SH3BP5**	1.98	2.28E-11	1.55E-10
**RBM25**	1.16	2.30E-11	1.56E-10
**CNPY4**	1.35	2.31E-11	1.56E-10
**SUDS3**	-0.77	2.31E-11	1.56E-10
**PCIF1**	1.09	2.32E-11	1.57E-10
**CHMP1B2P**	-3.05	2.33E-11	1.57E-10
**PFDN6**	1.00	2.33E-11	1.57E-10
**PIGF**	-1.33	2.33E-11	1.57E-10
**FAM184A**	-1.92	2.36E-11	1.60E-10
**TNPO1**	-0.81	2.36E-11	1.60E-10
**DAD1**	1.16	2.38E-11	1.60E-10
**RCBTB2**	1.19	2.38E-11	1.61E-10
**ARL4AP1**	-7.41	2.38E-11	1.61E-10
**RP1-68D18.4**	-2.40	2.39E-11	1.61E-10
**HMGN2P5**	1.27	2.40E-11	1.62E-10
**CLIC6**	3.36	2.41E-11	1.62E-10
**ZNF256**	-1.34	2.42E-11	1.63E-10
**OTUD6B**	-0.87	2.44E-11	1.64E-10
**HP**	4.24	2.44E-11	1.65E-10
**G21031**	-5.25	2.46E-11	1.66E-10
**ATXN1**	-0.66	2.48E-11	1.67E-10
**DHRS4-AS1**	1.00	2.48E-11	1.67E-10
**SEC13**	0.85	2.48E-11	1.67E-10
**PTGFRN**	0.69	2.50E-11	1.68E-10

**RP11-775C24.5**	1.43	2.51E-11	1.69E-10
**RPS10**	-2.15	2.52E-11	1.69E-10
**INPP4B**	-1.24	2.52E-11	1.69E-10
**PROX2**	-2.90	2.53E-11	1.70E-10
**RP11-229D13.3**	-3.13	2.53E-11	1.70E-10
**RP11-363E7.4**	-1.41	2.54E-11	1.71E-10
**GREM2**	-2.68	2.56E-11	1.72E-10
**PLXDC1**	2.53	2.60E-11	1.75E-10
**DDX18P1**	-4.58	2.60E-11	1.75E-10
**SETD7**	-0.79	2.62E-11	1.76E-10
**DCDC2B**	-3.75	2.62E-11	1.76E-10
**RP11-206L10.9**	-2.76	2.64E-11	1.77E-10
**RAB42**	3.53	2.64E-11	1.77E-10
**RP11-3P17.4**	-3.73	2.64E-11	1.77E-10
**RP11-2H3.7**	-4.42	2.64E-11	1.77E-10
**MMACHC**	-0.98	2.65E-11	1.78E-10
**CLEC3B**	-2.49	2.65E-11	1.78E-10
**AC008937.2**	-7.23	2.67E-11	1.79E-10
**FAM175A**	-0.87	2.68E-11	1.80E-10
**ZNF24**	-0.82	2.69E-11	1.80E-10
**NAP1L4**	0.71	2.69E-11	1.80E-10
**ZGPAT**	1.51	2.69E-11	1.80E-10
**JMJD6**	1.04	2.70E-11	1.81E-10
**MRPL51**	1.16	2.70E-11	1.81E-10
**ZDHHC7**	0.62	2.71E-11	1.81E-10
**B3GNT2**	1.00	2.75E-11	1.84E-10
**LRRC16A**	-0.86	2.77E-11	1.85E-10
**TMEM79**	1.21	2.77E-11	1.85E-10
**RP11-503E24.2**	-2.67	2.77E-11	1.85E-10
**RUFY2**	-0.64	2.78E-11	1.86E-10
**CD300LG**	3.43	2.78E-11	1.86E-10
**FAM153A**	-3.18	2.81E-11	1.88E-10
**UBAP2L**	0.61	2.81E-11	1.88E-10
**RIPK4**	1.14	2.81E-11	1.88E-10
**SP4**	-1.41	2.82E-11	1.88E-10
**CRCT1**	2.20	2.82E-11	1.89E-10
**CCND1**	-1.60	2.83E-11	1.89E-10
**CTD-2501E16.2**	-5.14	2.86E-11	1.91E-10
**PIGS**	0.75	2.87E-11	1.92E-10
**USP19**	0.66	2.88E-11	1.92E-10
**THBD**	1.44	2.89E-11	1.93E-10
**GABRA4**	-4.57	2.90E-11	1.93E-10
**G2520**	-2.28	2.90E-11	1.93E-10
**MED22**	0.94	2.91E-11	1.94E-10
**RNU6-167P**	-7.23	2.92E-11	1.95E-10

**STAG2**	-0.95	2.94E-11	1.96E-10
**FUT3**	2.99	2.98E-11	1.98E-10
**POFUT2**	1.77	3.01E-11	2.00E-10
**COMMD1**	0.86	3.03E-11	2.02E-10
**SLC25A26**	0.64	3.03E-11	2.02E-10
**NT5DC3**	-1.15	3.03E-11	2.02E-10
**RINT1**	-0.63	3.04E-11	2.02E-10
**GEMIN5**	-0.75	3.04E-11	2.03E-10
**PCP4L1**	2.71	3.04E-11	2.03E-10
**ZDHHC13**	-0.92	3.09E-11	2.05E-10
**SMARCAD1**	-0.93	3.12E-11	2.08E-10
**RP5-916O11.3**	-7.46	3.16E-11	2.10E-10
**TFCP2**	0.73	3.16E-11	2.10E-10
**ALDH3B1**	1.86	3.19E-11	2.12E-10
**LCLAT1**	-0.83	3.20E-11	2.12E-10
**MSL2**	-0.53	3.20E-11	2.13E-10
**CCT7**	0.97	3.23E-11	2.15E-10
**SNORD12B**	-4.24	3.25E-11	2.16E-10
**RP11-218C14.5**	-7.23	3.25E-11	2.16E-10
**PDE6A**	-6.08	3.25E-11	2.16E-10
**OSBPL7**	2.19	3.26E-11	2.16E-10
**IPO13**	0.99	3.28E-11	2.17E-10
**HMBS**	1.26	3.28E-11	2.18E-10
**SLITRK6**	-1.40	3.29E-11	2.18E-10
**MRPL42**	-0.96	3.31E-11	2.19E-10
**KRT18P31**	-7.01	3.32E-11	2.20E-10
**CHST7**	2.07	3.32E-11	2.20E-10
**PDGFRB**	2.87	3.34E-11	2.21E-10
**SLC29A4**	2.08	3.36E-11	2.23E-10
**AC006116.19**	-4.28	3.39E-11	2.24E-10
**COL4A4**	3.91	3.42E-11	2.27E-10
**RER1**	0.65	3.43E-11	2.27E-10
**TMEM53**	1.30	3.45E-11	2.29E-10
**MAP3K6**	1.15	3.51E-11	2.33E-10
**ZNF213**	1.27	3.52E-11	2.33E-10
**SNRPB**	1.08	3.52E-11	2.33E-10
**DOK7**	-3.26	3.53E-11	2.33E-10
**ASB14**	-1.98	3.55E-11	2.35E-10
**WDR5B**	-0.98	3.58E-11	2.37E-10
**AMOTL1**	-0.73	3.59E-11	2.37E-10
**RP5-1042I8.7**	-1.40	3.61E-11	2.39E-10
**AC092171.2**	-3.34	3.62E-11	2.39E-10
**FRS3**	2.06	3.63E-11	2.40E-10
**KRT16P3**	3.82	3.64E-11	2.40E-10
**RP11-797A18.5**	-5.26	3.67E-11	2.42E-10

**CTTN**	0.59	3.68E-11	2.43E-10
**RC3H1-IT1**	-7.15	3.72E-11	2.46E-10
**HMGCL**	1.02	3.72E-11	2.46E-10
**RGS9**	-2.29	3.73E-11	2.46E-10
**IFNK**	-7.04	3.73E-11	2.46E-10
**TMEM104**	1.26	3.74E-11	2.47E-10
**SYNCRIP**	-0.85	3.75E-11	2.47E-10
**XLOC_007718**	-3.12	3.76E-11	2.48E-10
**AP4M1**	1.04	3.78E-11	2.49E-10
**ERCC4**	-0.89	3.79E-11	2.50E-10
**POLR1D**	0.83	3.80E-11	2.51E-10
**KLK10**	1.44	3.81E-11	2.51E-10
**CCDC62**	-2.21	3.82E-11	2.52E-10
**ZNRD1-AS1**	-2.00	3.85E-11	2.53E-10
**IRAK1BP1**	-1.01	3.86E-11	2.54E-10
**FMOD**	2.73	3.86E-11	2.54E-10
**TRNT1**	-0.78	3.87E-11	2.54E-10
**TMEM102**	1.02	3.93E-11	2.59E-10
**SLC16A9**	3.25	3.96E-11	2.60E-10
**RP4-625H18.2**	-2.51	3.96E-11	2.61E-10
**LINC00623**	2.81	4.00E-11	2.63E-10
**RP11-797A18.6**	-3.72	4.01E-11	2.64E-10
**CPSF2**	-0.81	4.02E-11	2.65E-10
**RP11-536O18.1**	4.28	4.03E-11	2.65E-10
**CDHR5**	-4.69	4.05E-11	2.66E-10
**FTO**	-0.79	4.06E-11	2.67E-10
**FOXO1**	-0.81	4.11E-11	2.70E-10
**RP11-44I10.3**	-2.83	4.11E-11	2.70E-10
**ACVRL1**	2.52	4.13E-11	2.71E-10
**RP11-196G11.5**	-1.00	4.14E-11	2.72E-10
**PPP6R2**	0.68	4.15E-11	2.72E-10
**COPRS**	1.27	4.15E-11	2.72E-10
**CHD6**	-0.74	4.18E-11	2.74E-10
**LZIC**	-0.77	4.18E-11	2.74E-10
**GS1-115G20.1**	-6.28	4.19E-11	2.75E-10
**CDYL2**	1.63	4.20E-11	2.75E-10
**ALKBH3-AS1**	-2.26	4.22E-11	2.77E-10
**MAPK12**	2.50	4.24E-11	2.78E-10
**KIAA1217**	1.08	4.24E-11	2.78E-10
**PSME2**	0.82	4.25E-11	2.79E-10
**TAPBPL**	0.94	4.28E-11	2.80E-10
**AVL9**	-0.60	4.29E-11	2.81E-10
**C22orf34**	3.15	4.29E-11	2.81E-10
**RPL24**	1.07	4.35E-11	2.84E-10
**DUSP1**	2.39	4.45E-11	2.91E-10

**HTRA1**	2.08	4.47E-11	2.92E-10
**G40986**	-7.65	4.47E-11	2.92E-10
**ADH5**	1.10	4.49E-11	2.93E-10
**NPTX2**	2.43	4.49E-11	2.94E-10
**NAT6**	1.56	4.52E-11	2.95E-10
**ZBTB32**	-3.10	4.53E-11	2.96E-10
**LMTK3**	1.66	4.55E-11	2.97E-10
**ABCG4**	5.74	4.58E-11	2.99E-10
**ELFN2**	-3.88	4.58E-11	2.99E-10
**ZNF594**	-1.57	4.59E-11	3.00E-10
**DGCR6L**	1.04	4.60E-11	3.00E-10
**ANKRD27**	-0.48	4.62E-11	3.01E-10
**SNORA71C**	-4.51	4.62E-11	3.02E-10
**ZSCAN16-AS1**	1.94	4.63E-11	3.02E-10
**IPO11**	-0.70	4.67E-11	3.05E-10
**NUMBL**	1.72	4.68E-11	3.05E-10
**RGS17**	-2.28	4.73E-11	3.09E-10
**GPAT3**	-1.72	4.77E-11	3.11E-10
**AC073333.8**	-3.76	4.82E-11	3.14E-10
**PABPN1**	1.21	4.84E-11	3.15E-10
**NOB1**	0.80	4.85E-11	3.16E-10
**EXOC3**	0.70	4.85E-11	3.16E-10
**B4GALT2**	1.07	4.87E-11	3.17E-10
**RP11-434P11.2**	-3.46	4.88E-11	3.18E-10
**FLJ20021**	1.53	4.90E-11	3.19E-10
**PLA2G15**	0.86	4.93E-11	3.21E-10
**RP11-405F3.5**	-7.12	4.93E-11	3.21E-10
**MATN2**	-1.10	4.93E-11	3.21E-10
**RP11-231E19.1**	-4.85	4.94E-11	3.22E-10
**RP11-506K6.4**	-2.55	4.95E-11	3.22E-10
**SEMA6C**	1.63	4.96E-11	3.22E-10
**LEPROT**	0.78	5.00E-11	3.25E-10
**RARRES3**	2.12	5.04E-11	3.27E-10
**TBX4**	4.90	5.04E-11	3.28E-10
**KIAA1468**	-0.80	5.05E-11	3.28E-10
**COX5A**	0.97	5.06E-11	3.29E-10
**CTC-327F10.4**	-2.25	5.15E-11	3.34E-10
**VCAM1**	3.14	5.15E-11	3.35E-10
**TRIM14**	1.22	5.20E-11	3.37E-10
**CCDC153**	1.53	5.21E-11	3.38E-10
**ZKSCAN8**	-0.83	5.23E-11	3.39E-10
**INTS5**	0.93	5.23E-11	3.39E-10
**MMGT1**	-0.78	5.35E-11	3.47E-10
**RP5-1142A6.2**	-3.23	5.36E-11	3.48E-10
**AVPR1A**	2.61	5.38E-11	3.48E-10

**TPMTP2**	-7.03	5.43E-11	3.52E-10
**MLK7-AS1**	-2.65	5.45E-11	3.53E-10
**MRM1**	1.26	5.46E-11	3.54E-10
**ZNF14**	-1.01	5.47E-11	3.54E-10
**JAG2**	0.89	5.51E-11	3.57E-10
**CHSY1**	0.95	5.55E-11	3.59E-10
**CTA-276F8.1**	-3.23	5.55E-11	3.59E-10
**TFEB**	1.55	5.57E-11	3.60E-10
**DCTPP1**	1.11	5.58E-11	3.61E-10
**XXyac-YX65C7_A.2**	1.91	5.59E-11	3.61E-10
**MAN1C1**	1.50	5.59E-11	3.62E-10
**IGHMBP2**	0.74	5.71E-11	3.69E-10
**RBP7**	1.61	5.75E-11	3.72E-10
**PAX8**	-2.73	5.76E-11	3.72E-10
**AC007319.1**	-2.54	5.78E-11	3.73E-10
**PTER**	-0.86	5.79E-11	3.74E-10
**RP11-89H19.1**	-7.26	5.84E-11	3.77E-10
**CHD3**	0.67	5.84E-11	3.77E-10
**TEX261**	0.80	5.84E-11	3.77E-10
**OPN4**	5.22	5.85E-11	3.77E-10
**WAS**	2.61	5.85E-11	3.78E-10
**ZNF827**	1.15	5.86E-11	3.78E-10
**PPIA**	1.09	5.93E-11	3.82E-10
**THAP7**	0.75	5.94E-11	3.83E-10
**TMEM161B-AS1**	-1.52	5.98E-11	3.85E-10
**CIAPIN1**	0.85	5.99E-11	3.86E-10
**ZNF527**	-0.96	6.02E-11	3.88E-10
**PEX11B**	0.70	6.02E-11	3.88E-10
**MSANTD3**	0.94	6.02E-11	3.88E-10
**B4GALT5**	1.12	6.04E-11	3.89E-10
**SH2B2**	1.77	6.05E-11	3.90E-10
**GGA1**	0.85	6.13E-11	3.95E-10
**RP11-395I6.3**	-1.70	6.23E-11	4.01E-10
**FUBP3**	0.66	6.23E-11	4.01E-10
**CCL2**	2.71	6.27E-11	4.04E-10
**IARS**	-0.87	6.28E-11	4.04E-10
**CREM**	1.36	6.29E-11	4.05E-10
**TSPAN1**	2.36	6.34E-11	4.08E-10
**CLIP2**	1.04	6.36E-11	4.09E-10
**CFAP97**	-0.95	6.38E-11	4.10E-10
**MACROD1**	1.12	6.41E-11	4.12E-10
**GLTSCR1**	2.21	6.42E-11	4.13E-10
**UBR4**	-0.76	6.44E-11	4.14E-10
**MME**	4.11	6.47E-11	4.16E-10
**PSAPL1**	-1.55	6.48E-11	4.16E-10

**G30952**	-3.74	6.51E-11	4.18E-10
**METTL8**	-0.95	6.52E-11	4.19E-10
**ITGA9-AS1**	-1.55	6.53E-11	4.19E-10
**C5orf30**	-0.98	6.55E-11	4.20E-10
**C19orf66**	1.29	6.66E-11	4.28E-10
**G26246**	-4.77	6.74E-11	4.33E-10
**SEC24A**	-0.81	6.75E-11	4.33E-10
**LRRC37A6P**	-2.36	6.80E-11	4.36E-10
**RP11-731D1.4**	-2.23	6.82E-11	4.37E-10
**WDR66**	2.59	6.89E-11	4.42E-10
**RP5-1112D6.8**	-2.13	6.91E-11	4.43E-10
**SMG1P7**	-1.80	6.92E-11	4.43E-10
**GATC**	-0.66	6.94E-11	4.44E-10
**KCNQ1**	2.50	7.01E-11	4.49E-10
**BRINP1**	3.94	7.01E-11	4.49E-10
**ZNF75BP**	-7.26	7.02E-11	4.49E-10
**RP11-824M15.3**	-4.16	7.05E-11	4.51E-10
**DCXR**	1.25	7.11E-11	4.55E-10
**RP11-143J12.3**	-3.59	7.11E-11	4.55E-10
**HOOK2**	1.30	7.14E-11	4.57E-10
**RP11-864I4.3**	-2.49	7.17E-11	4.58E-10
**RP11-370I10.12**	-5.68	7.21E-11	4.61E-10
**AWAT1**	-8.38	7.23E-11	4.63E-10
**GOLGA2**	0.73	7.25E-11	4.63E-10
**USP1**	-0.92	7.28E-11	4.65E-10
**NCAPH2**	0.73	7.33E-11	4.68E-10
**FOXE1**	2.69	7.35E-11	4.69E-10
**CSRNP1**	1.22	7.35E-11	4.70E-10
**PNPLA3**	-2.10	7.36E-11	4.70E-10
**AL109767.1**	-6.92	7.37E-11	4.70E-10
**MLKL**	1.49	7.50E-11	4.79E-10
**CSPG4P11**	-2.41	7.58E-11	4.84E-10
**PPP1R13L**	1.44	7.63E-11	4.87E-10
**WNT11**	2.53	7.63E-11	4.87E-10
**GTPBP10**	-0.85	7.67E-11	4.89E-10
**SLC2A12**	-1.84	7.69E-11	4.90E-10
**KCTD2**	0.84	7.71E-11	4.91E-10
**RDH16**	2.15	7.76E-11	4.95E-10
**RP11-274B21.14**	-1.77	7.81E-11	4.98E-10
**GNG7**	1.93	7.87E-11	5.02E-10
**EEF1A1P13**	-1.86	7.89E-11	5.03E-10
**XLOC_006645**	-1.88	7.96E-11	5.07E-10
**MAP3K3**	0.84	7.96E-11	5.07E-10
**GOLT1B**	-1.05	7.97E-11	5.08E-10
**ACSM6**	-4.39	7.98E-11	5.08E-10

**ANKRD50**	-1.06	8.02E-11	5.10E-10
**LINC01125**	-2.16	8.05E-11	5.12E-10
**PRSS36**	1.46	8.10E-11	5.15E-10
**CA9**	4.00	8.11E-11	5.16E-10
**SLC24A3**	1.02	8.13E-11	5.17E-10
**SPON1**	3.21	8.13E-11	5.17E-10
**PLA2G4A**	-1.03	8.13E-11	5.17E-10
**DENND4B**	1.38	8.17E-11	5.19E-10
**HAUS5**	0.87	8.19E-11	5.20E-10
**EEF1A1P19**	-1.97	8.19E-11	5.21E-10
**ALAD**	0.85	8.21E-11	5.22E-10
**ALK**	-3.32	8.24E-11	5.23E-10
**RP5-850O15.3**	-7.04	8.24E-11	5.23E-10
**STIM1**	0.66	8.26E-11	5.24E-10
**CHRD**	2.51	8.27E-11	5.25E-10
**SLMAP**	-0.65	8.28E-11	5.25E-10
**RHOBTB2**	1.55	8.32E-11	5.28E-10
**KRT8P38**	-3.03	8.46E-11	5.37E-10
**ARHGEF25**	2.31	8.47E-11	5.37E-10
**OLFM1**	1.58	8.51E-11	5.40E-10
**DSG1-AS1**	-3.30	8.53E-11	5.41E-10
**CNTNAP2**	3.70	8.53E-11	5.41E-10
**PIGBOS1**	1.15	8.56E-11	5.43E-10
**KLHL35**	1.56	8.59E-11	5.44E-10
**SLC2A13**	-1.56	8.60E-11	5.45E-10
**ZNF189**	-0.89	8.62E-11	5.46E-10
**IMP4**	0.73	8.71E-11	5.51E-10
**XLOC_012057**	3.64	8.86E-11	5.61E-10
**AF129075.5**	-2.47	8.89E-11	5.63E-10
**CLDN7**	2.98	8.90E-11	5.63E-10
**TLE3**	1.02	9.01E-11	5.70E-10
**SAV1**	0.84	9.09E-11	5.75E-10
**XYLT2**	0.78	9.15E-11	5.79E-10
**PPP4R2**	-0.81	9.15E-11	5.79E-10
**RP11-393I2.2**	-7.10	9.35E-11	5.91E-10
**AC009303.1**	-6.63	9.35E-11	5.91E-10
**PSEN1**	-0.59	9.38E-11	5.93E-10
**TMPO**	-0.90	9.39E-11	5.93E-10
**FBXL14**	1.23	9.49E-11	6.00E-10
**RRAGA**	0.75	9.59E-11	6.05E-10
**C6orf163**	-2.13	9.59E-11	6.05E-10
**DEAR**	-4.10	9.65E-11	6.09E-10
**CBX2**	1.28	9.68E-11	6.11E-10
**FZD3**	-1.03	9.69E-11	6.12E-10
**TSHZ3**	2.46	9.71E-11	6.13E-10

**BNIP3P39**	-5.86	9.83E-11	6.20E-10
**NKG7**	3.10	9.83E-11	6.20E-10
**YBX3**	0.93	9.84E-11	6.20E-10
**LRP4-AS1**	-2.85	9.97E-11	6.28E-10
**RP4-639F20.1**	2.20	9.99E-11	6.30E-10
**KRAS**	-0.76	1.00E-10	6.30E-10
**G22214**	3.13	1.00E-10	6.30E-10
**MRPL54**	1.15	1.00E-10	6.31E-10
**GSTCD**	-0.80	1.00E-10	6.32E-10
**SCARNA3**	-4.57	1.01E-10	6.33E-10
**MSANTD4**	-0.81	1.01E-10	6.34E-10
**SNU13**	0.84	1.01E-10	6.36E-10
**CCDC85C**	1.08	1.01E-10	6.37E-10
**SHISA8**	3.48	1.02E-10	6.39E-10
**POLM**	1.50	1.02E-10	6.40E-10
**CENPF**	-1.35	1.02E-10	6.40E-10
**ACTR5**	0.73	1.02E-10	6.41E-10
**RP11-27M24.2**	-4.29	1.02E-10	6.41E-10
**ARHGAP10**	-0.61	1.02E-10	6.41E-10
**THAP7-AS1**	1.66	1.02E-10	6.41E-10
**ZMIZ2**	1.37	1.02E-10	6.42E-10
**FXR2**	0.73	1.02E-10	6.43E-10
**BLVRB**	1.18	1.04E-10	6.51E-10
**SLC10A5**	-3.57	1.04E-10	6.54E-10
**CAP1**	0.93	1.05E-10	6.61E-10
**ALDH6A1**	-1.30	1.06E-10	6.65E-10
**DNAJC5**	0.81	1.07E-10	6.69E-10
**PSMC5**	0.76	1.07E-10	6.72E-10
**DNM1L**	-0.58	1.07E-10	6.74E-10
**KIAA1033**	-0.75	1.08E-10	6.75E-10
**TAS2R31**	-3.73	1.08E-10	6.79E-10
**MIER2**	0.78	1.08E-10	6.80E-10
**C17orf96**	1.80	1.09E-10	6.83E-10
**CTD-2371O3.3**	-3.29	1.09E-10	6.85E-10
**PET117**	-2.11	1.10E-10	6.88E-10
**RP11-293M10.2**	-6.92	1.10E-10	6.89E-10
**RP11-25C19.3**	-7.02	1.10E-10	6.89E-10
**HNRNPA3**	0.75	1.11E-10	6.92E-10
**ANKUB1**	-7.03	1.11E-10	6.93E-10
**ITGB2**	2.47	1.11E-10	6.93E-10
**ANKHD1-EIF4EBP3**	-2.57	1.11E-10	6.95E-10
**RPL27**	1.20	1.11E-10	6.97E-10
**BRI3BP**	-1.88	1.13E-10	7.07E-10
**FIBP**	0.72	1.13E-10	7.08E-10
**COX6B1**	0.93	1.14E-10	7.10E-10

**FOXF2**	2.29	1.15E-10	7.19E-10
**ZNF221**	-1.57	1.15E-10	7.21E-10
**C1QTNF1**	2.13	1.16E-10	7.23E-10
**ZNF791**	-0.63	1.16E-10	7.24E-10
**PSMG3**	0.83	1.16E-10	7.24E-10
**SPINK9**	5.21	1.16E-10	7.27E-10
**C16orf58**	0.84	1.17E-10	7.28E-10
**AC093495.4**	-2.28	1.18E-10	7.36E-10
**XLOC_008747**	-4.19	1.18E-10	7.38E-10
**COPG1**	0.61	1.18E-10	7.39E-10
**PRR36**	2.36	1.19E-10	7.43E-10
**OAZ1**	0.86	1.20E-10	7.51E-10
**RP11-778H2.1**	-7.29	1.22E-10	7.61E-10
**ITPRIP**	1.21	1.22E-10	7.61E-10
**STOM**	1.07	1.23E-10	7.69E-10
**ZMAT5**	1.22	1.24E-10	7.70E-10
**CMB9-55A18.1**	-4.19	1.25E-10	7.77E-10
**NUTF2**	0.92	1.25E-10	7.79E-10
**PLAT**	1.90	1.27E-10	7.90E-10
**FYB**	2.35	1.27E-10	7.92E-10
**INPPL1**	1.26	1.27E-10	7.92E-10
**PCDHGA11**	-1.23	1.27E-10	7.93E-10
**WFDC1**	3.54	1.27E-10	7.93E-10
**SH3D19**	-0.73	1.28E-10	7.95E-10
**RNASEH2A**	1.19	1.28E-10	7.98E-10
**EIF3J-AS1**	1.02	1.29E-10	8.03E-10
**SLC4A11**	2.39	1.30E-10	8.06E-10
**ATL1**	-1.00	1.30E-10	8.08E-10
**C15orf56**	-1.98	1.30E-10	8.09E-10
**UBE2E3**	0.81	1.31E-10	8.14E-10
**ZNF468**	-0.92	1.31E-10	8.17E-10
**ITGB6**	1.69	1.31E-10	8.17E-10
**ARGFXP2**	-3.70	1.32E-10	8.18E-10
**SRP72P2**	-6.92	1.33E-10	8.24E-10
**NFYC**	0.59	1.34E-10	8.29E-10
**ERVW-1**	-3.34	1.34E-10	8.31E-10
**FOXP2**	-1.42	1.34E-10	8.34E-10
**RP11-267M23.1**	-1.72	1.35E-10	8.36E-10
**CTC-444N24.8**	-1.13	1.35E-10	8.36E-10
**SLC35A3**	-0.74	1.35E-10	8.36E-10
**RGS20**	1.88	1.35E-10	8.37E-10
**AL450992.2**	-1.89	1.36E-10	8.44E-10
**RAB33B**	-0.87	1.37E-10	8.47E-10
**PRNCR1**	-4.66	1.37E-10	8.47E-10
**RP11-173A16.1**	-3.43	1.38E-10	8.53E-10

**INHBA**	3.24	1.38E-10	8.58E-10
**SOX15**	1.21	1.39E-10	8.61E-10
**RP3-368A4.5**	-1.88	1.39E-10	8.63E-10
**VAMP8**	1.15	1.39E-10	8.64E-10
**KIDINS220**	-0.54	1.40E-10	8.67E-10
**TEDDM1**	-3.11	1.41E-10	8.70E-10
**F8A1**	1.99	1.41E-10	8.72E-10
**GPC4**	1.85	1.41E-10	8.73E-10
**PSMC1**	0.99	1.42E-10	8.82E-10
**SFRP4**	6.35	1.43E-10	8.83E-10
**COL12A1**	3.07	1.44E-10	8.91E-10
**ID4**	-1.21	1.46E-10	9.00E-10
**ZBTB4**	0.74	1.47E-10	9.07E-10
**RP11-326C3.7**	3.14	1.48E-10	9.12E-10
**RP13-216E22.4**	-2.42	1.49E-10	9.19E-10
**RP11-631M21.7**	-7.14	1.51E-10	9.33E-10
**SLC27A2**	-4.28	1.51E-10	9.33E-10
**SVBP**	1.24	1.52E-10	9.38E-10
**ZC3H7B**	0.58	1.53E-10	9.44E-10
**Z73979.1**	4.88	1.53E-10	9.44E-10
**EPGN**	4.47	1.53E-10	9.47E-10
**RN7SL128P**	-7.33	1.54E-10	9.48E-10
**ZNF169**	-1.45	1.54E-10	9.53E-10
**C1orf95**	-1.49	1.55E-10	9.53E-10
**PRSS8**	1.11	1.55E-10	9.54E-10
**ADGRF4**	-1.31	1.55E-10	9.55E-10
**TEKT3**	-2.26	1.56E-10	9.61E-10
**WDR63**	-2.01	1.56E-10	9.63E-10
**MMP13**	4.18	1.57E-10	9.69E-10
**RP11-356K23.1**	-3.27	1.57E-10	9.70E-10
**MICALCL**	-1.43	1.57E-10	9.70E-10
**GNPDA1**	1.08	1.58E-10	9.71E-10
**TDRP**	1.54	1.58E-10	9.71E-10
**TOMM5**	-1.66	1.58E-10	9.71E-10
**ATP1B1P1**	-6.95	1.58E-10	9.72E-10
**RPL13A**	0.88	1.59E-10	9.76E-10
**AHCY**	0.91	1.59E-10	9.81E-10
**SETD8**	0.76	1.59E-10	9.81E-10
**GPR82**	-1.99	1.62E-10	9.95E-10
**DYRK3**	1.78	1.62E-10	9.95E-10
**USP46**	-0.76	1.62E-10	9.95E-10
**HPS5**	-0.61	1.62E-10	9.96E-10
**MOB3A**	1.13	1.62E-10	9.97E-10
**CDC73**	-0.74	1.62E-10	9.97E-10
**AC092641.2**	-6.94	1.64E-10	1.01E-09

**CLCA2**	1.21	1.65E-10	1.01E-09
**FASTKD3**	-0.83	1.65E-10	1.01E-09
**ZNF227**	-0.71	1.66E-10	1.02E-09
**LRRC75B**	1.65	1.67E-10	1.02E-09
**SOBP**	2.47	1.67E-10	1.03E-09
**RP11-466H18.1**	1.29	1.67E-10	1.03E-09
**MNT**	1.61	1.67E-10	1.03E-09
**CYCSP38**	-7.02	1.69E-10	1.04E-09
**LRRC19**	-2.93	1.69E-10	1.04E-09
**LINC00894**	-2.44	1.69E-10	1.04E-09
**RP11-395I14.2**	-3.09	1.69E-10	1.04E-09
**CCDC86**	1.23	1.69E-10	1.04E-09
**ODCP**	-3.57	1.71E-10	1.05E-09
**SRSF4**	0.68	1.71E-10	1.05E-09
**CASP16P**	-2.91	1.71E-10	1.05E-09
**EEF1DP3**	-1.81	1.71E-10	1.05E-09
**MIIP**	1.24	1.73E-10	1.06E-09
**CMB9-22P13.1**	2.99	1.73E-10	1.06E-09
**ACSBG1**	-3.74	1.74E-10	1.07E-09
**XLOC_008981**	-4.37	1.76E-10	1.07E-09
**AC012360.6**	-2.81	1.76E-10	1.08E-09
**CTB-33O18.1**	5.62	1.78E-10	1.09E-09
**EIF3B**	0.64	1.78E-10	1.09E-09
**KIZ-AS1**	-4.23	1.78E-10	1.09E-09
**ATP5F1P1**	-6.94	1.79E-10	1.09E-09
**RP3-496C20.1**	-4.60	1.80E-10	1.10E-09
**HOXC10**	2.52	1.80E-10	1.10E-09
**PHGDH**	1.52	1.80E-10	1.10E-09
**NT5M**	1.50	1.80E-10	1.10E-09
**CTD-2622I13.3**	-3.98	1.80E-10	1.10E-09
**LHFP**	1.75	1.81E-10	1.11E-09
**TSGA10**	-1.10	1.82E-10	1.11E-09
**POM121L9P**	4.48	1.82E-10	1.11E-09
**RAI14**	-1.23	1.83E-10	1.12E-09
**AC008277.1**	-6.02	1.84E-10	1.12E-09
**CTC-344H19.6**	-7.20	1.84E-10	1.12E-09
**RP11-166P13.4**	-4.86	1.84E-10	1.13E-09
**NPLOC4**	0.58	1.85E-10	1.13E-09
**PDDC1**	0.93	1.86E-10	1.13E-09
**C18orf8**	-0.57	1.87E-10	1.14E-09
**ZRANB2-AS1**	-4.93	1.88E-10	1.15E-09
**RP11-981P6.1**	-2.71	1.89E-10	1.15E-09
**E2F3**	0.91	1.89E-10	1.15E-09
**CCDC64B**	1.22	1.92E-10	1.17E-09
**PM20D2**	-0.97	1.94E-10	1.18E-09

**SNAPC4**	1.07	1.95E-10	1.19E-09
**RP11-61L19.1**	-3.80	1.95E-10	1.19E-09
**CTBP2**	0.85	1.95E-10	1.19E-09
**XLOC_005050**	-2.28	1.96E-10	1.19E-09
**HYAL4**	3.97	1.96E-10	1.19E-09
**CALHM2**	1.91	1.96E-10	1.19E-09
**ANKRD65**	1.72	1.97E-10	1.20E-09
**ZDHHC23**	-1.47	1.97E-10	1.20E-09
**RP1-283E3.8**	-2.38	1.98E-10	1.20E-09
**MICAL2**	-1.09	2.01E-10	1.22E-09
**CTB-92J24.2**	-2.00	2.03E-10	1.23E-09
**ELF3**	2.59	2.03E-10	1.23E-09
**C21orf91-OT1**	-6.87	2.03E-10	1.23E-09
**GRHL1**	-0.86	2.04E-10	1.24E-09
**EPHB1**	-1.63	2.04E-10	1.24E-09
**PCDHGB9P**	-4.23	2.04E-10	1.24E-09
**PLPP1**	1.08	2.05E-10	1.24E-09
**C3orf17**	-0.52	2.06E-10	1.25E-09
**CYP1A1**	-5.67	2.06E-10	1.25E-09
**CFLAR**	-0.57	2.06E-10	1.25E-09
**CRBN**	-0.73	2.06E-10	1.25E-09
**RP3-425C14.4**	-1.20	2.08E-10	1.26E-09
**AC009970.1**	-4.17	2.08E-10	1.26E-09
**CD1D**	4.60	2.09E-10	1.27E-09
**LCMT1**	0.86	2.10E-10	1.27E-09
**SH2B3**	1.97	2.10E-10	1.27E-09
**ABRA**	-4.14	2.12E-10	1.28E-09
**FAM207A**	1.37	2.12E-10	1.28E-09
**AP001429.1**	-4.02	2.12E-10	1.29E-09
**SMARCA4**	0.81	2.13E-10	1.29E-09
**RP1-224A6.9**	-3.11	2.13E-10	1.29E-09
**ANKRD28**	-0.59	2.14E-10	1.29E-09
**CD74**	1.99	2.14E-10	1.29E-09
**ZNF439**	-1.12	2.14E-10	1.30E-09
**ZNF829**	-0.92	2.14E-10	1.30E-09
**CTD-2024I7.13**	-7.14	2.15E-10	1.30E-09
**TMX2P1**	-0.87	2.15E-10	1.30E-09
**ASIC1**	2.18	2.16E-10	1.30E-09
**FBXL7**	2.53	2.16E-10	1.30E-09
**XLOC_000610**	-1.60	2.16E-10	1.31E-09
**MAP1LC3A**	1.37	2.17E-10	1.31E-09
**MMP19**	2.42	2.17E-10	1.31E-09
**RNVU1-18**	-7.15	2.19E-10	1.32E-09
**CTD-2530H12.2**	-3.64	2.19E-10	1.32E-09
**ERVH48-1**	-3.54	2.21E-10	1.33E-09

**GMPR2**	0.72	2.24E-10	1.35E-09
**CTD-2017D11.2**	-2.74	2.24E-10	1.35E-09
**ZNF725P**	-7.30	2.24E-10	1.35E-09
**XLOC_013541**	5.12	2.27E-10	1.37E-09
**AC008074.3**	-2.46	2.29E-10	1.38E-09
**ZC3HAV1L**	-1.12	2.29E-10	1.38E-09
**SLC26A5**	-2.54	2.30E-10	1.39E-09
**EDIL3**	-1.58	2.30E-10	1.39E-09
**GMPPB**	1.10	2.31E-10	1.39E-09
**PRPF19**	0.87	2.31E-10	1.39E-09
**RHOV**	1.26	2.33E-10	1.41E-09
**PDZD11**	0.95	2.34E-10	1.41E-09
**FBXL19**	1.13	2.35E-10	1.42E-09
**RP11-380G5.2**	-4.31	2.36E-10	1.42E-09
**RPS12P26**	-3.61	2.36E-10	1.42E-09
**NAA10**	1.17	2.36E-10	1.42E-09
**PP14571**	-3.20	2.37E-10	1.43E-09
**RP11-138A9.1**	-3.33	2.37E-10	1.43E-09
**RP11-52A20.2**	-4.01	2.37E-10	1.43E-09
**FLT3**	-2.38	2.42E-10	1.45E-09
**RAD9A**	1.48	2.42E-10	1.46E-09
**RP11-57H14.4**	-0.92	2.42E-10	1.46E-09
**HCRTR1**	-5.08	2.44E-10	1.47E-09
**RP11-400F19.12**	-3.42	2.44E-10	1.47E-09
**DENND5A**	1.24	2.44E-10	1.47E-09
**IKBIP**	1.77	2.44E-10	1.47E-09
**MCRS1**	0.85	2.45E-10	1.47E-09
**EXOC2**	-0.67	2.46E-10	1.48E-09
**AC006019.3**	-7.27	2.46E-10	1.48E-09
**SNORA75**	-4.52	2.46E-10	1.48E-09
**ST8SIA2**	3.95	2.48E-10	1.49E-09
**ADAMTS2**	2.95	2.53E-10	1.52E-09
**RASSF7**	1.10	2.54E-10	1.52E-09
**FAM227A**	-2.66	2.54E-10	1.53E-09
**ERVMER34-1**	2.24	2.56E-10	1.53E-09
**NDUFAF5**	-0.60	2.56E-10	1.54E-09
**RASA3**	1.62	2.58E-10	1.55E-09
**RP11-99A1.2**	-6.82	2.59E-10	1.55E-09
**DPAGT1**	1.08	2.59E-10	1.55E-09
**FGF11**	1.82	2.61E-10	1.56E-09
**RP11-16C1.2**	-3.90	2.62E-10	1.57E-09
**HERC4**	-0.71	2.63E-10	1.57E-09
**SYT2**	-4.47	2.64E-10	1.58E-09
**DCAF10**	-0.67	2.66E-10	1.59E-09
**LIPN**	-1.95	2.66E-10	1.59E-09

**HEMGN**	-6.83	2.67E-10	1.60E-09
**ANGPTL3**	-6.84	2.69E-10	1.61E-09
**ZNF518B**	-0.93	2.69E-10	1.61E-09
**RP11-74C1.4**	-4.15	2.69E-10	1.61E-09
**PDIA4**	0.97	2.71E-10	1.62E-09
**RP11-600F24.7**	-1.79	2.71E-10	1.62E-09
**G10911**	-2.30	2.72E-10	1.62E-09
**LEAP2**	-2.23	2.72E-10	1.63E-09
**QPCTL**	1.20	2.75E-10	1.64E-09
**IQSEC2**	1.40	2.76E-10	1.65E-09
**ETF1P2**	-6.76	2.76E-10	1.65E-09
**PRR33**	-2.92	2.77E-10	1.65E-09
**SEC61B**	1.03	2.77E-10	1.66E-09
**RP11-58K22.4**	-2.97	2.80E-10	1.67E-09
**C10orf35**	2.08	2.81E-10	1.67E-09
**ANKRD46**	-0.97	2.81E-10	1.67E-09
**ANLN**	-1.28	2.81E-10	1.68E-09
**FAM185A**	-0.78	2.82E-10	1.68E-09
**RP11-374P20.4**	-6.86	2.82E-10	1.68E-09
**CALB1**	-3.82	2.82E-10	1.68E-09
**SNX29P1**	-2.97	2.83E-10	1.69E-09
**POMT2**	1.03	2.84E-10	1.69E-09
**HEXIM2**	-1.88	2.84E-10	1.69E-09
**SPOP**	0.63	2.88E-10	1.72E-09
**RP11-797H7.5**	-3.85	2.90E-10	1.73E-09
**LRRK2**	-1.06	2.90E-10	1.73E-09
**GTPBP3**	1.33	2.90E-10	1.73E-09
**SNORA46**	-4.59	2.91E-10	1.73E-09
**ZNF33B**	-1.03	2.98E-10	1.77E-09
**EPDR1**	2.07	2.98E-10	1.78E-09
**RP11-157G21.2**	4.18	2.99E-10	1.78E-09
**PGLYRP4**	1.72	3.00E-10	1.79E-09
**KCND1**	2.79	3.03E-10	1.80E-09
**C1orf105**	-4.11	3.04E-10	1.81E-09
**USP20**	0.80	3.04E-10	1.81E-09
**CTTNBP2NL**	-0.74	3.06E-10	1.82E-09
**SPARC**	2.62	3.06E-10	1.82E-09
**TMEM8B**	1.17	3.13E-10	1.86E-09
**RP11-290L7.5**	-7.18	3.15E-10	1.87E-09
**ATP2B2**	-3.08	3.15E-10	1.87E-09
**LDHB**	1.38	3.18E-10	1.89E-09
**CCDC69**	1.24	3.18E-10	1.89E-09
**HOMEZ**	-0.68	3.19E-10	1.90E-09
**AC016747.3**	1.51	3.19E-10	1.90E-09
**CORO7**	1.60	3.19E-10	1.90E-09

**NPM1P37**	-4.41	3.20E-10	1.90E-09
**THOC7-AS1**	-4.45	3.20E-10	1.90E-09
**SNORD9**	-7.26	3.21E-10	1.90E-09
**LINC01426**	5.12	3.22E-10	1.91E-09
**RP3-425C14.5**	-3.38	3.23E-10	1.92E-09
**NAV1**	1.35	3.24E-10	1.92E-09
**RP11-33B1.1**	-1.38	3.26E-10	1.93E-09
**CCDC94**	0.86	3.30E-10	1.96E-09
**RP11-583F2.5**	-6.80	3.31E-10	1.96E-09
**SLC2A3P4**	-4.42	3.34E-10	1.98E-09
**RP11-22C11.1**	-5.10	3.36E-10	1.99E-09
**RP11-568G11.4**	-4.89	3.37E-10	2.00E-09
**AF230666.2**	-3.47	3.38E-10	2.00E-09
**CSRNP3**	-1.24	3.39E-10	2.01E-09
**TMEM260**	-0.91	3.41E-10	2.02E-09
**RP11-492I21.1**	-2.55	3.43E-10	2.03E-09
**SLC30A4**	-0.86	3.45E-10	2.04E-09
**BRPF3**	0.97	3.47E-10	2.05E-09
**MYRF**	-1.45	3.48E-10	2.06E-09
**DKK1**	3.32	3.49E-10	2.06E-09
**PFKFB4**	2.13	3.49E-10	2.07E-09
**MICA**	1.34	3.51E-10	2.07E-09
**IMPG1**	-2.61	3.51E-10	2.08E-09
**EID2**	0.92	3.52E-10	2.08E-09
**DARS2**	-0.74	3.57E-10	2.11E-09
**RP11-697H9.2**	-6.65	3.58E-10	2.11E-09
**MED19**	0.84	3.58E-10	2.11E-09
**PLEKHA3**	-0.82	3.58E-10	2.12E-09
**GTF3A**	0.69	3.59E-10	2.12E-09
**AC093323.3**	0.70	3.62E-10	2.13E-09
**RNF6**	-1.01	3.62E-10	2.13E-09
**MBD1**	0.69	3.62E-10	2.14E-09
**F2RL2**	2.53	3.64E-10	2.15E-09
**RP11-488L18.4**	-1.31	3.66E-10	2.16E-09
**AK2**	0.63	3.66E-10	2.16E-09
**RP1-224A6.8**	-6.81	3.68E-10	2.17E-09
**CHTF18**	1.44	3.70E-10	2.18E-09
**TBC1D19**	-1.14	3.75E-10	2.21E-09
**CPNE3**	-0.92	3.75E-10	2.21E-09
**FTH1P24**	-6.58	3.79E-10	2.23E-09
**AL133243.1**	-2.29	3.79E-10	2.23E-09
**ZNF569**	-0.98	3.83E-10	2.26E-09
**ZNF101P2**	-6.97	3.86E-10	2.27E-09
**DOK2**	2.84	3.89E-10	2.29E-09
**RTN4RL2**	2.24	3.90E-10	2.30E-09

**TMEM185A**	1.22	3.92E-10	2.31E-09
**DIDO1**	-0.55	3.92E-10	2.31E-09
**STAMBPL1**	-0.99	3.93E-10	2.31E-09
**COA4**	0.90	3.93E-10	2.31E-09
**ZFPL1**	1.02	3.94E-10	2.32E-09
**CD200**	2.18	3.94E-10	2.32E-09
**TAF1C**	1.57	3.97E-10	2.33E-09
**TMSB4XP4**	2.64	3.97E-10	2.34E-09
**TRIM23**	-0.90	3.99E-10	2.34E-09
**RP3-468K18.6**	-3.48	3.99E-10	2.34E-09
**ZFC3H1**	-1.20	3.99E-10	2.34E-09
**RP11-557J10.3**	-6.56	3.99E-10	2.35E-09
**WNT5A-AS1**	2.88	4.00E-10	2.35E-09
**RP4-718P11.1**	-6.81	4.01E-10	2.35E-09
**RUFY3**	-0.84	4.01E-10	2.35E-09
**RP11-4B16.4**	-3.62	4.02E-10	2.36E-09
**MSTO1**	1.23	4.02E-10	2.36E-09
**TAC4**	-4.08	4.05E-10	2.38E-09
**ANXA11**	0.76	4.06E-10	2.38E-09
**MISP**	3.76	4.06E-10	2.38E-09
**GRIK3**	3.42	4.12E-10	2.41E-09
**CTD-3220F14.2**	-6.61	4.16E-10	2.44E-09
**CCDC107**	1.88	4.16E-10	2.44E-09
**TLE2**	1.52	4.16E-10	2.44E-09
**TMEM14A**	1.19	4.17E-10	2.45E-09
**NOD2**	1.02	4.19E-10	2.46E-09
**CCDC15**	-1.13	4.21E-10	2.47E-09
**SP8**	-2.52	4.21E-10	2.47E-09
**KIF16B**	-0.75	4.22E-10	2.47E-09
**MIR944**	-6.81	4.27E-10	2.50E-09
**RNF39**	1.30	4.30E-10	2.52E-09
**HIGD1B**	2.86	4.32E-10	2.53E-09
**ECM1**	1.51	4.33E-10	2.53E-09
**RP11-141B14.1**	-2.76	4.34E-10	2.54E-09
**MOB1A**	-0.91	4.36E-10	2.55E-09
**DR1**	-0.85	4.36E-10	2.55E-09
**MTRNR2L3**	-6.70	4.36E-10	2.55E-09
**RP11-521I2.3**	-4.33	4.39E-10	2.56E-09
**SELENBP1**	1.23	4.39E-10	2.57E-09
**EIF4E3**	-0.81	4.40E-10	2.57E-09
**CINP**	0.78	4.40E-10	2.57E-09
**ANO1**	-1.05	4.41E-10	2.57E-09
**ADAM1B**	-3.24	4.44E-10	2.59E-09
**PLOD2**	1.72	4.44E-10	2.60E-09
**RP11-680F8.4**	-4.10	4.47E-10	2.61E-09

**LINC00094**	0.98	4.51E-10	2.63E-09
**MBOAT2**	-1.11	4.51E-10	2.63E-09
**CEP19**	1.56	4.53E-10	2.64E-09
**RP11-27I1.4**	-1.16	4.53E-10	2.64E-09
**G26658**	-3.09	4.54E-10	2.65E-09
**MT-CO1**	1.72	4.54E-10	2.65E-09
**RRAD**	3.16	4.58E-10	2.67E-09
**SNORD116-16**	-7.32	4.59E-10	2.68E-09
**C19orf48**	0.92	4.59E-10	2.68E-09
**AC093673.5**	2.46	4.61E-10	2.68E-09
**OSMR**	1.31	4.61E-10	2.69E-09
**FNBP1P1**	-1.68	4.62E-10	2.69E-09
**RP3-382I10.7**	-3.09	4.63E-10	2.70E-09
**ZNF235**	-0.88	4.63E-10	2.70E-09
**RP11-162G10.1**	-3.63	4.65E-10	2.71E-09
**SPTBN2**	-1.28	4.66E-10	2.71E-09
**ATP13A4-AS1**	-6.72	4.67E-10	2.72E-09
**TSPAN18**	2.26	4.70E-10	2.74E-09
**RP11-120K19.3**	-4.00	4.73E-10	2.75E-09
**COG3**	-0.58	4.73E-10	2.75E-09
**ZNF341**	1.03	4.74E-10	2.76E-09
**FAM25A**	-3.06	4.77E-10	2.77E-09
**HEATR6**	-0.57	4.77E-10	2.77E-09
**SOCS1**	2.25	4.77E-10	2.77E-09
**PPP2R5D**	0.52	4.77E-10	2.78E-09
**RGS4**	6.45	4.84E-10	2.82E-09
**TMEM230**	0.96	4.87E-10	2.83E-09
**XLOC_004197**	3.99	4.88E-10	2.84E-09
**PYM1**	0.96	5.01E-10	2.91E-09
**RP11-392B6.1**	-6.70	5.04E-10	2.93E-09
**RAD18**	-0.57	5.05E-10	2.93E-09
**SIX1**	5.56	5.05E-10	2.93E-09
**VTRNA2-1**	-6.93	5.06E-10	2.94E-09
**ZNF426**	-0.72	5.08E-10	2.95E-09
**ZNF546**	-1.40	5.08E-10	2.95E-09
**ANGPT1**	2.78	5.10E-10	2.96E-09
**ECH1**	1.30	5.11E-10	2.96E-09
**EFCAB6**	-1.27	5.12E-10	2.97E-09
**PIP5KL1**	1.87	5.15E-10	2.99E-09
**CTB-92J24.3**	-7.42	5.24E-10	3.04E-09
**LSM12**	0.82	5.27E-10	3.06E-09
**PLXNA4**	-1.80	5.28E-10	3.06E-09
**ZNF710**	0.77	5.31E-10	3.08E-09
**RP11-299J3.8**	-1.60	5.32E-10	3.08E-09
**RP11-97O12.6**	-2.20	5.33E-10	3.08E-09

**PCSK9**	2.41	5.33E-10	3.08E-09
**PCDHGA8**	-2.31	5.35E-10	3.09E-09
**FER1L5**	-2.82	5.35E-10	3.09E-09
**DNAJB1**	0.89	5.39E-10	3.12E-09
**MIF4GD**	0.90	5.42E-10	3.14E-09
**G10488**	-7.11	5.46E-10	3.16E-09
**PSMA2**	-1.07	5.48E-10	3.17E-09
**CCL19**	3.28	5.49E-10	3.17E-09
**TMEM68**	-0.82	5.50E-10	3.18E-09
**SLC45A1**	2.15	5.52E-10	3.19E-09
**AAMP**	0.78	5.54E-10	3.20E-09
**CEACAM5**	2.50	5.56E-10	3.21E-09
**AZIN1**	-0.83	5.56E-10	3.21E-09
**TBL1XR1-AS1**	-6.73	5.57E-10	3.21E-09
**C1orf27**	-0.60	5.61E-10	3.24E-09
**UNC5B**	2.10	5.65E-10	3.26E-09
**HELLPAR**	-2.34	5.67E-10	3.27E-09
**RBM12B-AS1**	-2.81	5.68E-10	3.28E-09
**RP11-568K15.1**	-1.15	5.68E-10	3.28E-09
**KBTBD7**	-0.81	5.69E-10	3.28E-09
**CPSF1**	1.04	5.70E-10	3.29E-09
**RGPD5**	-2.66	5.72E-10	3.30E-09
**TENM3**	2.20	5.73E-10	3.30E-09
**ZNF204P**	-1.37	5.74E-10	3.31E-09
**DUS2**	0.72	5.76E-10	3.32E-09
**MAP2K4**	-0.68	5.79E-10	3.34E-09
**UGT1A5**	-6.95	5.81E-10	3.35E-09
**EIF4EBP2**	-0.60	5.90E-10	3.40E-09
**DDX26B**	-1.12	5.93E-10	3.42E-09
**RP11-67L2.2**	-1.09	5.94E-10	3.42E-09
**FAHD1**	1.22	5.95E-10	3.43E-09
**PIK3CB**	-0.94	5.96E-10	3.43E-09
**RP11-457M11.7**	-3.65	5.99E-10	3.45E-09
**RP5-881P19.7**	-6.53	5.99E-10	3.45E-09
**WDR17**	-2.83	5.99E-10	3.45E-09
**MRPL14**	0.99	5.99E-10	3.45E-09
**UQCRH**	0.95	6.02E-10	3.46E-09
**BBS9**	-0.76	6.03E-10	3.47E-09
**RNU2-64P**	-7.87	6.05E-10	3.48E-09
**XLOC_010311**	-6.48	6.09E-10	3.50E-09
**RPS6KA5**	-1.32	6.18E-10	3.55E-09
**RP11-354M20.3**	-5.30	6.18E-10	3.55E-09
**PSMC4**	0.86	6.18E-10	3.55E-09
**KCTD13**	1.42	6.19E-10	3.56E-09
**B3GALT4**	0.74	6.21E-10	3.57E-09

**LRRC75A**	-1.67	6.23E-10	3.58E-09
**MYO5B**	-1.30	6.23E-10	3.58E-09
**RP11-214J9.1**	-3.95	6.24E-10	3.58E-09
**RP11-288C18.1**	-1.18	6.24E-10	3.58E-09
**DDX28**	0.84	6.30E-10	3.62E-09
**UST**	-1.40	6.31E-10	3.62E-09
**C22orf23**	1.13	6.31E-10	3.62E-09
**BAK1**	1.28	6.33E-10	3.63E-09
**MOCS1**	2.66	6.33E-10	3.63E-09
**DIRAS1**	1.92	6.34E-10	3.64E-09
**BEND5**	2.25	6.37E-10	3.65E-09
**ZNF624**	-0.91	6.40E-10	3.67E-09
**PHKG2**	0.99	6.45E-10	3.70E-09
**SOD2**	1.42	6.45E-10	3.70E-09
**AC026271.5**	1.16	6.47E-10	3.71E-09
**AL928654.7**	-6.91	6.49E-10	3.72E-09
**MYCBP2-AS2**	-6.88	6.54E-10	3.74E-09
**SNORD116-1**	-7.15	6.55E-10	3.75E-09
**PIAS4**	0.85	6.58E-10	3.76E-09
**CTD-2233K9.1**	-2.83	6.62E-10	3.79E-09
**COG7**	0.63	6.65E-10	3.81E-09
**THAP6**	-0.82	6.69E-10	3.83E-09
**CTD-2574D22.4**	-1.93	6.69E-10	3.83E-09
**NXT1**	0.86	6.73E-10	3.85E-09
**RP11-815J21.3**	-6.64	6.78E-10	3.88E-09
**DOCK9-AS1**	-6.61	6.82E-10	3.90E-09
**TIMM8B**	1.11	6.83E-10	3.91E-09
**NUP98**	-0.58	6.87E-10	3.93E-09
**KPNA6**	-0.63	6.89E-10	3.94E-09
**ADH6**	-3.04	6.91E-10	3.95E-09
**HSPA6**	1.69	7.01E-10	4.00E-09
**RPL7**	0.98	7.05E-10	4.03E-09
**VPS37D**	1.70	7.07E-10	4.04E-09
**SLC24A5**	-2.20	7.09E-10	4.05E-09
**MARCKS**	1.01	7.12E-10	4.06E-09
**FBXL13**	-1.72	7.17E-10	4.09E-09
**G30345**	-3.15	7.19E-10	4.10E-09
**RP11-552M11.4**	-3.42	7.20E-10	4.11E-09
**NENF**	0.97	7.23E-10	4.12E-09
**GSAP**	-1.12	7.38E-10	4.21E-09
**MYO1F**	1.97	7.39E-10	4.21E-09
**DNAJB12**	0.50	7.44E-10	4.24E-09
**RDH10-AS1**	-5.58	7.48E-10	4.26E-09
**NEMP1**	-1.09	7.51E-10	4.28E-09
**NAP1L4P1**	-3.18	7.56E-10	4.31E-09

**RP11-745A24.1**	-6.47	7.56E-10	4.31E-09
**KLHL26**	1.02	7.58E-10	4.32E-09
**KBTBD8**	-1.47	7.58E-10	4.32E-09
**IK**	0.80	7.60E-10	4.33E-09
**PCAT19**	2.52	7.60E-10	4.33E-09
**SLC25A16**	-0.76	7.64E-10	4.35E-09
**MKNK1**	0.97	7.67E-10	4.36E-09
**PARGP1**	-0.90	7.69E-10	4.37E-09
**RP11-530C5.4**	-6.44	7.74E-10	4.40E-09
**ZBTB20-AS1**	-4.22	7.77E-10	4.42E-09
**CREB3L1**	3.18	7.85E-10	4.46E-09
**SMYD5**	0.75	7.87E-10	4.47E-09
**NR5A2**	1.90	7.93E-10	4.51E-09
**MRPS31P5**	-2.14	7.97E-10	4.53E-09
**RSU1**	0.82	7.99E-10	4.54E-09
**TAS2R3**	-5.51	8.02E-10	4.55E-09
**RP11-575G13.2**	-6.76	8.03E-10	4.56E-09
**MIR421**	-6.73	8.03E-10	4.56E-09
**RPL23AP64**	-3.09	8.06E-10	4.57E-09
**RNF157**	2.07	8.08E-10	4.59E-09
**TBL3**	0.77	8.10E-10	4.60E-09
**CCDC120**	1.28	8.12E-10	4.61E-09
**COL23A1**	1.68	8.12E-10	4.61E-09
**EPN3**	1.30	8.14E-10	4.62E-09
**CXCR2**	1.26	8.19E-10	4.65E-09
**NADSYN1**	1.03	8.20E-10	4.65E-09
**CACNA2D2**	-1.59	8.20E-10	4.65E-09
**FAM217A**	-3.64	8.23E-10	4.67E-09
**HOXA11**	2.86	8.31E-10	4.71E-09
**RRP1**	0.94	8.31E-10	4.71E-09
**OACYLP**	5.73	8.32E-10	4.72E-09
**RP11-286E11.1**	-3.91	8.37E-10	4.74E-09
**FAM46C**	-1.99	8.42E-10	4.77E-09
**ACTN1**	1.82	8.54E-10	4.83E-09
**RP11-797H7.1**	-1.98	8.55E-10	4.84E-09
**RN7SL648P**	-5.75	8.57E-10	4.85E-09
**RP6-99M1.3**	-7.00	8.59E-10	4.86E-09
**DNAJC1**	0.82	8.62E-10	4.88E-09
**RP11-535C21.3**	-6.64	8.63E-10	4.88E-09
**C1GALT1P1**	-6.77	8.64E-10	4.89E-09
**LDOC1L**	0.99	8.66E-10	4.90E-09
**RP11-530C5.2**	-3.31	8.67E-10	4.90E-09
**CLN3**	-1.39	8.69E-10	4.91E-09
**CTD-3184A7.4**	1.64	8.71E-10	4.93E-09
**TAS2R15P**	-3.88	8.74E-10	4.94E-09

**ADRBK2**	-1.03	8.76E-10	4.95E-09
**FAM110A**	1.12	8.79E-10	4.97E-09
**KAT2B**	-0.90	8.81E-10	4.98E-09
**AVEN**	1.02	8.82E-10	4.98E-09
**DENND6B**	1.84	8.83E-10	4.99E-09
**E4F1**	0.87	8.85E-10	5.00E-09
**RP11-820I16.3**	-4.41	8.86E-10	5.00E-09
**PDS5A**	-0.66	8.87E-10	5.01E-09
**FAM134B**	-1.52	8.89E-10	5.02E-09
**SLC26A6**	1.62	8.93E-10	5.04E-09
**ZNF37BP**	-1.27	8.94E-10	5.04E-09
**CTA-398F10.2**	-3.06	8.95E-10	5.05E-09
**SYNE3**	-1.67	9.01E-10	5.08E-09
**DSTYK**	-0.66	9.05E-10	5.10E-09
**CRTAC1**	1.51	9.05E-10	5.10E-09
**AKAP13**	-0.79	9.17E-10	5.17E-09
**MTRR**	-0.75	9.19E-10	5.18E-09
**TMEM246-AS1**	-4.46	9.24E-10	5.21E-09
**CEP152**	-1.19	9.27E-10	5.22E-09
**HCCS**	0.69	9.28E-10	5.23E-09
**EFNB1**	0.82	9.38E-10	5.28E-09
**SNORD101**	-3.10	9.38E-10	5.28E-09
**ZNF45**	-0.67	9.42E-10	5.30E-09
**METTL20**	-0.93	9.42E-10	5.30E-09
**AFTPH**	-0.83	9.51E-10	5.35E-09
**GATM**	-1.64	9.65E-10	5.43E-09
**XLOC_008024**	-1.63	9.74E-10	5.48E-09
**SNX33**	0.86	9.76E-10	5.49E-09
**AC010240.2**	-4.53	9.86E-10	5.54E-09
**MITF**	-1.10	9.92E-10	5.58E-09
**ZNF230**	-0.86	9.95E-10	5.59E-09
**TRAPPC3**	0.70	1.00E-09	5.63E-09
**ZDHHC21**	-1.37	1.00E-09	5.64E-09
**KCNMB1**	2.02	1.01E-09	5.65E-09
**TEC**	-1.01	1.02E-09	5.72E-09
**PROSER2**	1.30	1.02E-09	5.73E-09
**KCNA1**	-6.85	1.02E-09	5.73E-09
**ADAM22**	-1.84	1.02E-09	5.74E-09
**ST3GAL3**	1.20	1.02E-09	5.74E-09
**PILRA**	2.76	1.03E-09	5.79E-09
**XLOC_013543**	4.26	1.04E-09	5.83E-09
**KAT5**	0.58	1.05E-09	5.86E-09
**AGAP2**	1.35	1.05E-09	5.87E-09
**RP11-204P2.3**	-3.18	1.06E-09	5.95E-09
**RP11-39M21.1**	-6.74	1.06E-09	5.95E-09

**TMEM185B**	0.67	1.06E-09	5.96E-09
**AC092811.1**	-4.13	1.06E-09	5.97E-09
**PTPN23**	1.47	1.07E-09	5.99E-09
**NR2F6**	1.01	1.09E-09	6.09E-09
**RASGRP3**	2.26	1.09E-09	6.10E-09
**KNOP1**	0.87	1.09E-09	6.12E-09
**DHX37**	0.73	1.09E-09	6.13E-09
**MUC16**	-7.04	1.10E-09	6.14E-09
**KLHL41**	-2.70	1.10E-09	6.14E-09
**EIF2B4**	0.78	1.10E-09	6.14E-09
**RP11-264L1.2**	-7.08	1.10E-09	6.15E-09
**MARK2P8**	-3.28	1.10E-09	6.15E-09
**XLOC_010389**	-3.92	1.10E-09	6.16E-09
**EPS15L1**	0.61	1.10E-09	6.16E-09
**MTND4P15**	-6.55	1.10E-09	6.18E-09
**PIDD1**	1.84	1.11E-09	6.18E-09
**NRM**	1.00	1.11E-09	6.21E-09
**AC003104.1**	-5.36	1.13E-09	6.31E-09
**DLEU2L**	-2.62	1.13E-09	6.32E-09
**CRTAP**	0.79	1.13E-09	6.33E-09
**XXYLT1**	1.02	1.14E-09	6.36E-09
**MOXD1**	1.60	1.14E-09	6.37E-09
**ZNF28**	-1.01	1.16E-09	6.46E-09
**GOLGA4**	-1.09	1.16E-09	6.49E-09
**NT5C2**	0.45	1.16E-09	6.49E-09
**SECTM1**	1.38	1.16E-09	6.50E-09
**RP11-203B9.4**	-2.33	1.17E-09	6.53E-09
**HS6ST3**	-3.79	1.17E-09	6.54E-09
**NHEG1**	-2.80	1.18E-09	6.56E-09
**RP11-337N6.1**	-3.73	1.18E-09	6.57E-09
**SLC22A18AS**	2.86	1.18E-09	6.59E-09
**RP11-140H17.2**	-6.62	1.19E-09	6.63E-09
**RP11-123O10.3**	-6.92	1.20E-09	6.70E-09
**CTD-2636A23.2**	-2.33	1.21E-09	6.74E-09
**IRAK2**	1.25	1.21E-09	6.74E-09
**FABP4**	5.15	1.21E-09	6.76E-09
**SNORD116-15**	-7.45	1.21E-09	6.77E-09
**DDAH2**	1.11	1.22E-09	6.77E-09
**SIPA1L2**	1.42	1.22E-09	6.81E-09
**THOC1**	-0.91	1.23E-09	6.84E-09
**RP13-487P22.1**	-4.21	1.23E-09	6.85E-09
**FAN1**	-0.95	1.24E-09	6.89E-09
**SARNP**	-2.47	1.24E-09	6.93E-09
**BMX**	2.45	1.25E-09	6.95E-09
**SURF2**	0.90	1.26E-09	7.00E-09

**SKP2**	-0.75	1.26E-09	7.01E-09
**TUBGCP2**	0.52	1.26E-09	7.01E-09
**MACROD2**	-1.43	1.26E-09	7.02E-09
**CD99L2**	1.29	1.26E-09	7.03E-09
**PITPNM2**	1.12	1.27E-09	7.08E-09
**GALNT1**	-1.18	1.28E-09	7.14E-09
**ZNF263**	0.48	1.29E-09	7.16E-09
**TIMM17A**	0.74	1.29E-09	7.17E-09
**RP4-671G15.2**	-3.36	1.29E-09	7.17E-09
**FNDC3A**	-0.75	1.30E-09	7.20E-09
**UHRF1BP1**	-0.65	1.30E-09	7.21E-09
**RP11-252A24.2**	-1.31	1.30E-09	7.22E-09
**PFDN1**	0.75	1.31E-09	7.27E-09
**RP11-33N16.2**	-6.26	1.32E-09	7.33E-09
**RBM4**	-1.19	1.32E-09	7.34E-09
**SNORA33**	-3.02	1.34E-09	7.41E-09
**FABP7**	-4.79	1.35E-09	7.51E-09
**STAT5A**	0.97	1.36E-09	7.52E-09
**SNAI1**	3.01	1.36E-09	7.56E-09
**ACAP2-IT1**	-3.27	1.37E-09	7.59E-09
**FGFR2**	-1.07	1.38E-09	7.63E-09
**LAMP5**	3.86	1.39E-09	7.69E-09
**G7822**	-2.48	1.40E-09	7.76E-09
**CDH13**	0.88	1.41E-09	7.81E-09
**SERPINA1**	3.34	1.42E-09	7.86E-09
**RP11-290C10.1**	-4.11	1.42E-09	7.89E-09
**MAPKAPK5**	-0.57	1.43E-09	7.91E-09
**ARPC3**	0.94	1.43E-09	7.91E-09
**AFF3**	-2.09	1.43E-09	7.92E-09
**TTC3P1**	-0.94	1.44E-09	7.98E-09
**PPP3CB-AS1**	-1.19	1.47E-09	8.11E-09
**DNAJB11**	0.78	1.47E-09	8.14E-09
**AP000577.2**	-3.66	1.48E-09	8.18E-09
**ALAS2**	-4.84	1.49E-09	8.23E-09
**COL4A5**	-0.89	1.50E-09	8.28E-09
**PDE10A**	2.37	1.50E-09	8.29E-09
**RP5-951N9.1**	-6.80	1.51E-09	8.36E-09
**RNASEH2B**	-0.76	1.53E-09	8.46E-09
**PRDM16**	2.28	1.53E-09	8.48E-09
**RBBP4P1**	-6.56	1.54E-09	8.52E-09
**HTATIP2**	0.95	1.55E-09	8.54E-09
**SPDYE1**	-2.81	1.55E-09	8.56E-09
**CD1C**	-2.62	1.55E-09	8.58E-09
**RN7SL16P**	-6.63	1.57E-09	8.65E-09
**AC025335.1**	-1.24	1.57E-09	8.65E-09

**SCART1**	-1.58	1.57E-09	8.65E-09
**HIF1AN**	-0.69	1.57E-09	8.68E-09
**BOP1**	1.52	1.58E-09	8.70E-09
**RP11-537E18.1**	-3.82	1.58E-09	8.70E-09
**TSEN54**	0.92	1.58E-09	8.70E-09
**KRT6C**	7.17	1.58E-09	8.73E-09
**TAS2R19**	-4.33	1.59E-09	8.74E-09
**GSG1**	-3.63	1.60E-09	8.79E-09
**PSMA6P1**	-3.66	1.60E-09	8.80E-09
**LRPPRC**	-0.60	1.60E-09	8.82E-09
**RP3-522J7.5**	-6.50	1.60E-09	8.82E-09
**PARVA**	1.00	1.61E-09	8.85E-09
**CARKD**	0.65	1.61E-09	8.89E-09
**SERPINF1**	2.30	1.62E-09	8.91E-09
**ACTR3B**	1.08	1.62E-09	8.91E-09
**ATP5HP4**	-4.23	1.62E-09	8.94E-09
**TBC1D9B**	0.54	1.63E-09	8.97E-09
**HSD3B7**	1.17	1.63E-09	8.98E-09
**CCR10**	3.64	1.63E-09	8.98E-09
**IL15RA**	1.55	1.63E-09	8.99E-09
**RP11-592B15.4**	-1.44	1.64E-09	9.00E-09
**STX7**	-0.63	1.64E-09	9.00E-09
**CERS5**	0.70	1.64E-09	9.00E-09
**RP3-472M2.2**	-6.49	1.64E-09	9.01E-09
**SNRPGP18**	-6.81	1.64E-09	9.01E-09
**CYP39A1**	-1.59	1.65E-09	9.05E-09
**CCDC130**	1.23	1.65E-09	9.05E-09
**HIST2H2BA**	-2.82	1.65E-09	9.08E-09
**GPC3**	2.13	1.65E-09	9.09E-09
**BMP2KL**	-6.86	1.67E-09	9.16E-09
**IRS4**	-3.53	1.68E-09	9.22E-09
**MPI**	0.81	1.68E-09	9.23E-09
**HBS1L**	-0.60	1.68E-09	9.23E-09
**SCARNA23**	-6.73	1.69E-09	9.25E-09
**NPNT**	1.45	1.71E-09	9.37E-09
**DNAJC19P5**	-2.16	1.71E-09	9.39E-09
**EIF1AX-AS1**	-4.28	1.71E-09	9.39E-09
**MMP3**	7.85	1.71E-09	9.40E-09
**RP11-23N2.4**	-2.21	1.72E-09	9.45E-09
**MATK**	2.18	1.72E-09	9.45E-09
**RP11-46O21.2**	-4.50	1.73E-09	9.48E-09
**MTMR8**	-1.22	1.74E-09	9.52E-09
**RP11-1094M14.11**	1.13	1.74E-09	9.53E-09
**NAT9**	1.11	1.76E-09	9.66E-09
**C1orf122**	1.72	1.79E-09	9.80E-09

**CASP1P2**	-1.99	1.79E-09	9.83E-09
**CTD-3014M21.1**	-3.66	1.81E-09	9.90E-09
**RP11-197K3.1**	-6.59	1.81E-09	9.92E-09
**G36147**	-8.95	1.82E-09	9.96E-09
**MPLKIP**	0.52	1.82E-09	9.96E-09
**PDS5B**	-0.71	1.82E-09	9.98E-09
**FMNL2**	1.20	1.83E-09	1.00E-08
**TMEM233**	5.00	1.83E-09	1.00E-08
**TCEA1P2**	-1.82	1.84E-09	1.01E-08
**MTND1P9**	-6.48	1.86E-09	1.02E-08
**B9D2**	1.57	1.87E-09	1.02E-08
**SMARCE1P1**	-6.55	1.87E-09	1.02E-08
**RP11-527N22.1**	3.55	1.89E-09	1.03E-08
**TRIM52-AS1**	1.01	1.89E-09	1.03E-08
**RP11-463O12.5**	-2.10	1.90E-09	1.04E-08
**KIFC3**	0.75	1.90E-09	1.04E-08
**FGD1**	0.89	1.93E-09	1.05E-08
**ENTPD1-AS1**	-0.94	1.93E-09	1.06E-08
**AADACL2**	-1.80	1.94E-09	1.06E-08
**TM2D1**	-0.71	1.95E-09	1.06E-08
**FAM120C**	-1.06	1.95E-09	1.06E-08
**FILIP1**	2.03	1.96E-09	1.07E-08
**MICALL1**	0.91	1.96E-09	1.07E-08
**ZNF614**	-0.89	1.96E-09	1.07E-08
**RAPGEF1**	0.83	1.96E-09	1.07E-08
**DHPS**	0.89	1.98E-09	1.08E-08
**LSM2**	0.96	1.99E-09	1.08E-08
**LINC01376**	-2.05	2.00E-09	1.09E-08
**LRIT2**	-2.33	2.00E-09	1.09E-08
**FOXRED2**	0.99	2.00E-09	1.09E-08
**FAM132B**	2.61	2.01E-09	1.09E-08
**RP11-519C12.1**	-6.62	2.01E-09	1.09E-08
**RNASET2**	1.39	2.01E-09	1.09E-08
**FBXO4**	0.86	2.01E-09	1.10E-08
**SLC7A4**	-2.35	2.02E-09	1.10E-08
**ARPC2**	0.69	2.02E-09	1.10E-08
**AP5Z1**	0.98	2.03E-09	1.11E-08
**CCDC146**	-1.27	2.04E-09	1.11E-08
**CNKSR1**	1.68	2.07E-09	1.12E-08
**SNORA70**	-4.46	2.07E-09	1.12E-08
**PDE7B**	2.07	2.09E-09	1.14E-08
**RP11-609N14.4**	-2.72	2.10E-09	1.14E-08
**ROBO3**	2.80	2.12E-09	1.15E-08
**NACC1**	0.93	2.12E-09	1.15E-08
**RP11-390P2.4**	1.66	2.14E-09	1.16E-08

**AC092168.2**	-3.69	2.14E-09	1.16E-08
**CDK5**	0.97	2.14E-09	1.16E-08
**RRM2P3**	-2.45	2.14E-09	1.16E-08
**COL18A1-AS2**	-6.70	2.14E-09	1.16E-08
**ISG20**	1.55	2.14E-09	1.16E-08
**TMEM98**	1.54	2.15E-09	1.17E-08
**INAFM2**	1.37	2.16E-09	1.17E-08
**RNU1-1**	-6.59	2.16E-09	1.17E-08
**APBB1**	1.19	2.16E-09	1.17E-08
**CLEC4E**	4.18	2.17E-09	1.18E-08
**CCRL2**	3.10	2.17E-09	1.18E-08
**MAP2K1**	0.78	2.17E-09	1.18E-08
**CYP4A11**	-4.31	2.17E-09	1.18E-08
**KLHL5**	1.34	2.18E-09	1.18E-08
**YTHDC2**	-0.72	2.18E-09	1.18E-08
**DPY19L4**	-0.81	2.18E-09	1.18E-08
**RNPEPL1**	1.05	2.19E-09	1.19E-08
**FAM83E**	2.43	2.21E-09	1.20E-08
**FZD1**	1.13	2.21E-09	1.20E-08
**AC005550.3**	-5.64	2.25E-09	1.22E-08
**CREB3L2**	1.20	2.25E-09	1.22E-08
**CLEC4G**	4.97	2.26E-09	1.22E-08
**KPNA1**	-0.64	2.26E-09	1.22E-08
**NUTM1**	-6.33	2.26E-09	1.22E-08
**STBD1**	2.07	2.26E-09	1.22E-08
**SMCR8**	-0.56	2.26E-09	1.22E-08
**MRPL45**	0.77	2.27E-09	1.23E-08
**CCDC162P**	-2.68	2.28E-09	1.23E-08
**RP11-28B23.1**	-5.31	2.28E-09	1.23E-08
**ST7-AS2**	-3.66	2.29E-09	1.24E-08
**DACT1**	2.75	2.29E-09	1.24E-08
**CD72**	2.87	2.30E-09	1.24E-08
**SRGAP2C**	-1.30	2.30E-09	1.24E-08
**NOMO1**	0.71	2.30E-09	1.24E-08
**PRKAB1**	0.76	2.30E-09	1.24E-08
**CCDC6**	-0.73	2.32E-09	1.25E-08
**RP1-198K11.5**	2.13	2.32E-09	1.25E-08
**SHC2**	1.56	2.33E-09	1.26E-08
**CD1E**	-2.45	2.34E-09	1.27E-08
**RP11-792A8.3**	-2.70	2.35E-09	1.27E-08
**IRX3**	1.81	2.36E-09	1.27E-08
**H6PD**	-0.64	2.36E-09	1.27E-08
**BRD4**	1.40	2.37E-09	1.28E-08
**TBX2-AS1**	2.81	2.40E-09	1.29E-08
**FAM73A**	-0.72	2.40E-09	1.30E-08

**BCR**	0.86	2.40E-09	1.30E-08
**PQBP1**	1.00	2.41E-09	1.30E-08
**WDR19**	-0.74	2.41E-09	1.30E-08
**SAMD12**	-1.76	2.41E-09	1.30E-08
**RASAL3**	1.79	2.43E-09	1.31E-08
**COG8**	-0.68	2.43E-09	1.31E-08
**PC**	1.59	2.44E-09	1.31E-08
**PHF13**	0.61	2.44E-09	1.31E-08
**DCLK2**	1.63	2.45E-09	1.32E-08
**CCDC14**	-1.27	2.45E-09	1.32E-08
**AC015923.1**	-6.42	2.46E-09	1.33E-08
**TRIM56**	1.03	2.47E-09	1.33E-08
**HYPK**	-2.17	2.48E-09	1.34E-08
**PEX10**	0.64	2.51E-09	1.35E-08
**C6orf222**	-3.29	2.53E-09	1.36E-08
**FAM225A**	4.61	2.53E-09	1.36E-08
**USP35**	1.56	2.53E-09	1.36E-08
**ZC3H12A**	1.19	2.54E-09	1.36E-08
**EFCAB1**	-1.69	2.57E-09	1.38E-08
**CERS2**	1.01	2.57E-09	1.38E-08
**HFE**	1.15	2.57E-09	1.38E-08
**TSPAN4**	1.53	2.57E-09	1.38E-08
**ZRANB1**	-0.93	2.57E-09	1.38E-08
**SCN7A**	-3.91	2.58E-09	1.38E-08
**MRPL43**	0.84	2.58E-09	1.38E-08
**MPZL3**	-1.29	2.58E-09	1.39E-08
**PHIP**	-0.78	2.59E-09	1.39E-08
**TCN1**	6.61	2.59E-09	1.39E-08
**PICK1**	0.88	2.60E-09	1.39E-08
**HEXDC**	1.34	2.62E-09	1.41E-08
**NANS**	0.66	2.63E-09	1.41E-08
**HNRNPRP1**	-4.56	2.63E-09	1.41E-08
**PRR16**	3.46	2.65E-09	1.42E-08
**ARFIP2**	0.67	2.65E-09	1.42E-08
**CFAP58-AS1**	2.00	2.66E-09	1.43E-08
**RP11-549L6.2**	-6.62	2.66E-09	1.43E-08
**RP11-478C6.6**	-2.74	2.69E-09	1.44E-08
**RP5-872K7.7**	-6.53	2.70E-09	1.44E-08
**AMPD2**	0.91	2.70E-09	1.44E-08
**FAM131B**	2.85	2.70E-09	1.45E-08
**RP11-430C7.2**	-6.62	2.71E-09	1.45E-08
**WDR86**	3.66	2.71E-09	1.45E-08
**TYRO3P**	-3.53	2.73E-09	1.46E-08
**FAM124A**	1.25	2.73E-09	1.46E-08
**RAB6B**	1.47	2.75E-09	1.47E-08

**RP11-709A23.1**	-2.64	2.75E-09	1.47E-08
**AP000462.2**	-6.96	2.75E-09	1.47E-08
**CTD-2525I3.6**	-6.14	2.78E-09	1.49E-08
**RN7SL338P**	-6.49	2.79E-09	1.49E-08
**RP11-504I13.2**	-7.00	2.80E-09	1.50E-08
**AL354808.2**	-6.63	2.80E-09	1.50E-08
**TMEM184C**	-0.59	2.83E-09	1.51E-08
**LIPH**	-1.73	2.83E-09	1.51E-08
**KIF7**	1.84	2.84E-09	1.52E-08
**ANK1**	2.06	2.86E-09	1.53E-08
**SPATA1**	-2.41	2.86E-09	1.53E-08
**ILF2P1**	-6.65	2.87E-09	1.53E-08
**IFT140**	0.82	2.89E-09	1.54E-08
**MTCH1**	0.56	2.91E-09	1.55E-08
**MTFMT**	0.57	2.92E-09	1.56E-08
**RNMT**	-0.68	2.92E-09	1.56E-08
**RP11-477E3.2**	-6.44	2.93E-09	1.56E-08
**DDX50P1**	-2.89	2.93E-09	1.56E-08
**ZKSCAN3**	-0.97	2.93E-09	1.56E-08
**WASH4P**	2.05	2.93E-09	1.57E-08
**CELF2-AS1**	-3.64	2.95E-09	1.58E-08
**TUBE1**	-0.94	2.96E-09	1.58E-08
**ERCC6L**	-1.18	2.99E-09	1.60E-08
**LINC00691**	-6.71	3.00E-09	1.60E-08
**TRMT13**	-0.93	3.03E-09	1.62E-08
**RP11-16K12.2**	-6.31	3.03E-09	1.62E-08
**TMEM56**	-4.50	3.04E-09	1.62E-08
**RP11-429P3.8**	-3.04	3.04E-09	1.62E-08
**PIBF1**	-0.84	3.04E-09	1.62E-08
**CLDN20**	-3.49	3.04E-09	1.62E-08
**ESYT2**	-0.70	3.06E-09	1.63E-08
**RPS11P5**	-2.20	3.07E-09	1.63E-08
**DHX38**	0.55	3.07E-09	1.64E-08
**SPDYA**	-2.29	3.11E-09	1.65E-08
**CLIP1-AS1**	-3.96	3.12E-09	1.66E-08
**BCL9**	0.82	3.16E-09	1.68E-08
**CYP2C18**	-1.46	3.17E-09	1.69E-08
**ZNF132**	-1.12	3.18E-09	1.69E-08
**RP4-814D15.1**	-6.54	3.21E-09	1.71E-08
**NAA40**	1.09	3.21E-09	1.71E-08
**ZNF813**	-0.88	3.22E-09	1.71E-08
**SNORA13**	-4.50	3.23E-09	1.71E-08
**TRABD2B**	1.52	3.23E-09	1.72E-08
**CCT7P1**	-2.38	3.24E-09	1.72E-08
**KATNAL2**	-1.45	3.25E-09	1.73E-08

**OASL**	4.22	3.28E-09	1.74E-08
**CITF22-92A6.1**	3.06	3.29E-09	1.75E-08
**ZNF469**	2.40	3.30E-09	1.75E-08
**FAM35CP**	-6.45	3.30E-09	1.75E-08
**RPGRIP1L**	-1.09	3.30E-09	1.75E-08
**DLGAP1-AS1**	1.65	3.31E-09	1.76E-08
**ZNF354C**	-1.16	3.31E-09	1.76E-08
**PYGM**	1.93	3.32E-09	1.76E-08
**RP11-295G20.2**	1.42	3.33E-09	1.76E-08
**AC025918.2**	-6.42	3.33E-09	1.77E-08
**CHST2**	2.51	3.33E-09	1.77E-08
**GS1-115G20.2**	-4.89	3.34E-09	1.77E-08
**XXbac-BPGBPG55C20.2**	-1.55	3.36E-09	1.78E-08
**CCDC126**	-1.10	3.37E-09	1.79E-08
**IPO7**	-0.75	3.37E-09	1.79E-08
**STARD7**	-0.63	3.38E-09	1.79E-08
**REXO1**	1.14	3.39E-09	1.80E-08
**CTD-2619J13.14**	1.07	3.39E-09	1.80E-08
**RP11-981G7.1**	-5.52	3.40E-09	1.80E-08
**C3AR1**	3.01	3.40E-09	1.80E-08
**RP11-96K19.4**	-2.24	3.41E-09	1.80E-08
**FARP2**	-0.60	3.43E-09	1.82E-08
**RP11-862L9.2**	-4.34	3.45E-09	1.82E-08
**ZNF793**	-1.06	3.45E-09	1.83E-08
**TYK2**	1.00	3.50E-09	1.85E-08
**2-Mar**	0.86	3.50E-09	1.85E-08
**AC002117.1**	-4.84	3.50E-09	1.85E-08
**RP11-274A11.4**	-3.03	3.54E-09	1.87E-08
**AC023632.1**	-6.22	3.56E-09	1.88E-08
**RP1-288H2.2**	-3.43	3.56E-09	1.88E-08
**PPBP**	-4.69	3.57E-09	1.89E-08
**G36254**	-2.95	3.57E-09	1.89E-08
**RP11-752D24.2**	-4.16	3.58E-09	1.89E-08
**TMEM232**	-1.75	3.59E-09	1.90E-08
**NDN**	1.88	3.61E-09	1.91E-08
**MKNK2**	0.95	3.63E-09	1.92E-08
**MED1**	-0.53	3.63E-09	1.92E-08
**MT-CYB**	3.41	3.64E-09	1.92E-08
**ZNF703**	1.54	3.65E-09	1.92E-08
**TYW5**	-1.02	3.69E-09	1.95E-08
**ZNF761**	-0.81	3.70E-09	1.95E-08
**ZNF41**	-0.67	3.78E-09	1.99E-08
**NRBF2P5**	-4.73	3.78E-09	1.99E-08
**COX20**	-1.36	3.81E-09	2.01E-08
**DMAP1**	1.08	3.84E-09	2.03E-08

**ARC**	2.57	3.85E-09	2.03E-08
**HOXB9**	4.73	3.85E-09	2.03E-08
**FBXL3**	-0.95	3.85E-09	2.03E-08
**KMT2E-AS1**	1.30	3.86E-09	2.03E-08
**ALG11**	-0.67	3.86E-09	2.03E-08
**ALKBH8**	-0.67	3.88E-09	2.04E-08
**KRT128P**	-6.88	3.89E-09	2.05E-08
**YWHAQ**	0.84	3.89E-09	2.05E-08
**ZNF860**	-1.24	3.89E-09	2.05E-08
**G39463**	-2.51	3.90E-09	2.05E-08
**RP11-536L3.4**	-4.06	3.90E-09	2.05E-08
**CPNE1**	1.27	3.90E-09	2.05E-08
**FGFRL1**	1.34	3.90E-09	2.05E-08
**RBM17P1**	-6.81	3.91E-09	2.05E-08
**SH3RF3-AS1**	2.14	3.91E-09	2.05E-08
**STX16**	1.24	3.92E-09	2.06E-08
**CRIP1**	-2.44	3.92E-09	2.06E-08
**BAALC**	1.82	3.93E-09	2.06E-08
**SPTLC3**	-1.20	3.94E-09	2.07E-08
**DCAF7**	0.63	3.94E-09	2.07E-08
**FBXW4**	0.89	3.95E-09	2.08E-08
**TCEB1**	0.94	3.96E-09	2.08E-08
**PRAF2**	2.49	3.97E-09	2.09E-08
**INTS6-AS1**	-1.95	3.98E-09	2.09E-08
**TNFRSF11B**	3.01	3.99E-09	2.09E-08
**ANKS6**	0.97	3.99E-09	2.09E-08
**AC093382.1**	-3.08	3.99E-09	2.09E-08
**CDC42-IT1**	-5.19	4.00E-09	2.10E-08
**VEGFC**	2.17	4.00E-09	2.10E-08
**G10141**	-3.96	4.01E-09	2.10E-08
**HNRNPA1P49**	-3.70	4.01E-09	2.10E-08
**RN7SL674P**	-6.38	4.02E-09	2.11E-08
**HOXA13**	2.04	4.03E-09	2.11E-08
**RP11-260M2.1**	-3.34	4.04E-09	2.12E-08
**ADI1**	0.67	4.06E-09	2.13E-08
**ERH**	0.97	4.06E-09	2.13E-08
**CTD-2651B20.7**	-5.14	4.07E-09	2.13E-08
**RP11-278C7.3**	-3.22	4.07E-09	2.13E-08
**RP11-677I18.3**	-2.44	4.07E-09	2.13E-08
**TNKS2**	-0.68	4.08E-09	2.13E-08
**TMEM219**	0.60	4.08E-09	2.14E-08
**CTA-384D8.36**	2.24	4.10E-09	2.14E-08
**ZNF436**	-0.71	4.13E-09	2.16E-08
**HIST1H4J**	-2.00	4.17E-09	2.18E-08
**TMEM37**	1.73	4.18E-09	2.19E-08

**ZBED8**	-1.31	4.18E-09	2.19E-08
**RP11-532M24.1**	-2.29	4.20E-09	2.20E-08
**SEH1L**	-0.73	4.21E-09	2.20E-08
**PDE4DIP**	-0.96	4.21E-09	2.20E-08
**LINC01527**	-3.11	4.21E-09	2.20E-08
**FAM193B**	1.41	4.22E-09	2.20E-08
**PYGB**	1.11	4.23E-09	2.21E-08
**RP11-44F14.7**	-3.67	4.23E-09	2.21E-08
**CTD-2310F14.1**	-2.70	4.25E-09	2.22E-08
**ENO1-IT1**	-4.07	4.25E-09	2.22E-08
**GLRX5**	0.97	4.27E-09	2.23E-08
**EXOC3-AS1**	1.09	4.27E-09	2.23E-08
**RP11-573D15.9**	-1.97	4.27E-09	2.23E-08
**UBA6**	-0.70	4.30E-09	2.24E-08
**PRDM4**	0.46	4.34E-09	2.27E-08
**TOMM34**	0.77	4.35E-09	2.27E-08
**RP1-80N2.4**	-2.03	4.38E-09	2.29E-08
**SNORA66**	-2.58	4.40E-09	2.30E-08
**SNCG**	1.59	4.41E-09	2.30E-08
**SYCP3**	-2.71	4.43E-09	2.31E-08
**RNU5D-1**	-6.99	4.43E-09	2.31E-08
**ISL1**	-3.61	4.47E-09	2.33E-08
**AL136115.1**	-3.58	4.47E-09	2.33E-08
**RP11-466F5.8**	-2.96	4.52E-09	2.36E-08
**P2RX7**	-1.16	4.54E-09	2.36E-08
**TOE1**	0.75	4.54E-09	2.37E-08
**SHISA4**	1.57	4.57E-09	2.38E-08
**ETHE1**	1.45	4.58E-09	2.39E-08
**MUS81**	1.13	4.61E-09	2.40E-08
**PSMB8**	1.05	4.62E-09	2.41E-08
**ZFAND1**	-0.73	4.63E-09	2.41E-08
**TNPO1P1**	-5.48	4.64E-09	2.41E-08
**EIF4H**	1.01	4.65E-09	2.42E-08
**LRP3**	1.31	4.68E-09	2.44E-08
**PLSCR1**	1.42	4.68E-09	2.44E-08
**METTL14**	-0.67	4.70E-09	2.44E-08
**RP11-1277A3.2**	-1.60	4.70E-09	2.44E-08
**DSG1**	-1.43	4.74E-09	2.47E-08
**PKDREJ**	-1.71	4.75E-09	2.47E-08
**NRROS**	2.19	4.76E-09	2.47E-08
**AC113189.5**	1.56	4.76E-09	2.48E-08
**CACNB2**	-1.77	4.77E-09	2.48E-08
**OGT**	-1.33	4.77E-09	2.48E-08
**SFR1P1**	-6.72	4.77E-09	2.48E-08
**ANKS1A**	0.62	4.78E-09	2.48E-08

**TM4SF20**	-6.23	4.83E-09	2.51E-08
**CXCR6**	-1.83	4.85E-09	2.52E-08
**BNIP3P8**	-6.35	4.85E-09	2.52E-08
**TEX264**	0.86	4.85E-09	2.52E-08
**MED6**	-0.54	4.87E-09	2.53E-08
**RP3-522J7.7**	-2.78	4.88E-09	2.53E-08
**CTC-527H23.1**	-6.12	4.93E-09	2.56E-08
**ADORA2BP1**	-2.91	4.93E-09	2.56E-08
**MEIOB**	-2.63	4.94E-09	2.56E-08
**AC009480.3**	-5.35	4.99E-09	2.59E-08
**PTRF**	0.99	5.01E-09	2.60E-08
**ZXDB**	-0.95	5.05E-09	2.62E-08
**RP11-513M16.7**	-2.64	5.06E-09	2.62E-08
**SEC63**	0.61	5.06E-09	2.62E-08
**GLIPR2**	1.68	5.09E-09	2.64E-08
**NRBF2**	1.09	5.10E-09	2.64E-08
**FAM78A**	2.17	5.13E-09	2.66E-08
**KIAA1755**	2.71	5.13E-09	2.66E-08
**KIF3C**	1.33	5.13E-09	2.66E-08
**PLEKHA2**	-0.81	5.14E-09	2.66E-08
**LARP1B**	-0.73	5.14E-09	2.66E-08
**RAPGEF6**	-0.80	5.16E-09	2.67E-08
**LCE1F**	2.26	5.16E-09	2.67E-08
**RP11-603K19.1**	-5.56	5.17E-09	2.67E-08
**ESPNL**	3.39	5.17E-09	2.68E-08
**SLC25A46**	-0.85	5.19E-09	2.69E-08
**EBF1**	2.08	5.19E-09	2.69E-08
**PROM2**	0.95	5.20E-09	2.69E-08
**BNIP3P26**	-6.55	5.20E-09	2.69E-08
**NCOA4**	-0.86	5.21E-09	2.69E-08
**RP1-117O3.2**	-6.30	5.22E-09	2.70E-08
**RHEB**	0.68	5.28E-09	2.73E-08
**ZNF267**	-0.87	5.30E-09	2.74E-08
**MTA1**	1.07	5.30E-09	2.74E-08
**UNC80**	-2.84	5.35E-09	2.76E-08
**FAM198B**	2.05	5.36E-09	2.77E-08
**DAAM2**	1.58	5.37E-09	2.77E-08
**C15orf62**	-1.11	5.38E-09	2.78E-08
**PGP**	0.98	5.39E-09	2.78E-08
**RN7SL12P**	-5.04	5.41E-09	2.79E-08
**RP11-800A3.7**	3.79	5.41E-09	2.79E-08
**FAM53C**	0.57	5.44E-09	2.81E-08
**TBXAS1**	2.62	5.45E-09	2.81E-08
**WDR25**	0.91	5.46E-09	2.81E-08
**KRBOX4**	0.76	5.48E-09	2.83E-08

**CCDC38**	-3.39	5.49E-09	2.83E-08
**GGA3**	0.75	5.50E-09	2.83E-08
**SLC30A10**	-5.38	5.50E-09	2.84E-08
**ADM5**	2.19	5.51E-09	2.84E-08
**PHBP12**	-4.21	5.51E-09	2.84E-08
**LINC00641**	-1.52	5.51E-09	2.84E-08
**TMEM242**	0.65	5.56E-09	2.86E-08
**CD2AP**	-0.96	5.58E-09	2.87E-08
**RP11-182L21.6**	1.03	5.58E-09	2.87E-08
**OPA1-AS1**	-5.31	5.58E-09	2.88E-08
**ENTPD6**	1.19	5.61E-09	2.89E-08
**CMTM7**	1.56	5.62E-09	2.89E-08
**RP11-152L20.3**	-3.93	5.62E-09	2.89E-08
**CDKN2AIP**	0.61	5.64E-09	2.90E-08
**RN7SL517P**	-6.39	5.65E-09	2.90E-08
**RP11-4B16.1**	-4.56	5.67E-09	2.91E-08
**UGDH**	0.88	5.69E-09	2.93E-08
**MTO1**	-0.47	5.69E-09	2.93E-08
**G32314**	1.93	5.70E-09	2.93E-08
**COBL**	-1.23	5.71E-09	2.94E-08
**RP11-390P2.2**	-4.95	5.72E-09	2.94E-08
**SARS**	0.69	5.73E-09	2.95E-08
**USMG5**	1.02	5.76E-09	2.96E-08
**SNORD116-8**	-6.83	5.77E-09	2.97E-08
**METTL13**	0.80	5.81E-09	2.98E-08
**SLC25A42**	1.02	5.83E-09	2.99E-08
**SENP1**	-0.60	5.83E-09	2.99E-08
**NHLH2**	-1.87	5.85E-09	3.01E-08
**SNORD112**	-2.66	5.90E-09	3.03E-08
**RP11-861A13.3**	-3.37	5.92E-09	3.04E-08
**CTHRC1**	3.17	5.95E-09	3.05E-08
**RP11-17E4.1**	-6.48	5.95E-09	3.05E-08
**ZNF718**	-2.01	5.97E-09	3.06E-08
**LINC00339**	0.83	5.99E-09	3.07E-08
**AC002400.1**	-6.52	6.01E-09	3.08E-08
**SV2A**	2.11	6.01E-09	3.08E-08
**CWF19L1**	-0.67	6.01E-09	3.08E-08
**PLEKHF2**	-1.12	6.01E-09	3.08E-08
**ALG9**	-0.78	6.01E-09	3.08E-08
**TNRC18**	1.10	6.02E-09	3.09E-08
**RP11-58G13.1**	-6.54	6.04E-09	3.09E-08
**RP11-278J6.5**	-6.43	6.07E-09	3.11E-08
**ACKR2**	2.95	6.10E-09	3.13E-08
**RPP25**	1.48	6.11E-09	3.13E-08
**PRKAG2-AS1**	2.03	6.16E-09	3.15E-08

**WASF2**	0.73	6.16E-09	3.16E-08
**RP5-991C6.4**	-6.46	6.22E-09	3.18E-08
**SYNDIG1**	5.02	6.25E-09	3.20E-08
**AFF2**	-2.13	6.30E-09	3.22E-08
**TADA1**	-0.76	6.32E-09	3.23E-08
**RP11-30L15.4**	-3.79	6.32E-09	3.23E-08
**USP38**	-0.96	6.36E-09	3.25E-08
**CRADD**	0.74	6.37E-09	3.26E-08
**CTC-559E9.6**	-1.66	6.40E-09	3.27E-08
**GALM**	1.60	6.43E-09	3.28E-08
**DMRT3**	5.85	6.45E-09	3.29E-08
**DPRXP1**	-6.21	6.54E-09	3.34E-08
**AC006994.1**	-5.33	6.54E-09	3.34E-08
**AC006277.3**	-3.35	6.59E-09	3.37E-08
**RP4-695O20.1**	-6.30	6.60E-09	3.37E-08
**TNK1**	0.85	6.61E-09	3.37E-08
**ARSD**	0.83	6.64E-09	3.39E-08
**GRHPR**	0.55	6.66E-09	3.40E-08
**ABHD14B**	1.01	6.67E-09	3.40E-08
**ZNF500**	0.81	6.67E-09	3.40E-08
**GPD1L**	-1.17	6.68E-09	3.41E-08
**KRT8P26**	-2.35	6.72E-09	3.43E-08
**KIF5B**	-0.89	6.74E-09	3.44E-08
**TTLL1**	1.09	6.74E-09	3.44E-08
**RBM22**	0.38	6.75E-09	3.44E-08
**CBX1**	0.71	6.77E-09	3.45E-08
**ZFAND2B**	0.71	6.78E-09	3.46E-08
**SLAMF8**	2.52	6.78E-09	3.46E-08
**RP11-326L2.1**	-4.31	6.81E-09	3.47E-08
**FCN3**	3.75	6.82E-09	3.47E-08
**RP11-188C12.2**	-2.51	6.82E-09	3.47E-08
**NKAPP1**	-1.80	6.83E-09	3.48E-08
**KRT42P**	2.70	6.84E-09	3.48E-08
**FRY**	-0.99	6.85E-09	3.49E-08
**KIAA0100**	-0.63	6.85E-09	3.49E-08
**MT-TM**	-7.94	6.85E-09	3.49E-08
**ZFYVE26**	-0.66	6.88E-09	3.50E-08
**RP11-731J8.1**	-6.34	6.89E-09	3.51E-08
**HYAL1**	1.26	6.90E-09	3.51E-08
**TNRC6C-AS1**	1.79	6.91E-09	3.51E-08
**XLOC_009000**	-6.31	6.91E-09	3.52E-08
**SERTM1**	-7.68	6.92E-09	3.52E-08
**PSMA5**	0.83	6.93E-09	3.52E-08
**MEF2D**	1.03	6.96E-09	3.54E-08
**PYROXD1**	-0.84	6.97E-09	3.54E-08

**AC006461.2**	-6.57	6.98E-09	3.55E-08
**KRT8P31**	-4.08	6.99E-09	3.55E-08
**ADCY3**	0.85	7.03E-09	3.57E-08
**SNRPGP2**	2.26	7.06E-09	3.58E-08
**RP11-382A20.3**	1.13	7.08E-09	3.59E-08
**STEAP1**	2.29	7.10E-09	3.60E-08
**JADE1**	-0.73	7.11E-09	3.61E-08
**SNORA45**	-4.79	7.12E-09	3.61E-08
**SYT7**	2.63	7.12E-09	3.62E-08
**TAF12**	0.78	7.14E-09	3.62E-08
**FN1**	2.72	7.15E-09	3.63E-08
**AGBL4**	-3.45	7.16E-09	3.63E-08
**ICAM1**	2.22	7.16E-09	3.63E-08
**CPD**	-0.87	7.19E-09	3.65E-08
**TNFRSF10D**	2.10	7.24E-09	3.67E-08
**MIER1**	-0.81	7.31E-09	3.71E-08
**KIAA1147**	-0.59	7.33E-09	3.71E-08
**ORMDL1**	-1.11	7.34E-09	3.72E-08
**XLOC_005426**	-3.76	7.35E-09	3.72E-08
**RP11-544A12.4**	-3.02	7.37E-09	3.73E-08
**DKFZP434I0714**	-0.99	7.43E-09	3.76E-08
**2-Mar**	1.66	7.49E-09	3.80E-08
**ZNF837**	1.01	7.51E-09	3.80E-08
**DBNDD1**	1.55	7.53E-09	3.81E-08
**RNPC3**	-1.33	7.55E-09	3.82E-08
**NKD2**	1.67	7.56E-09	3.82E-08
**CYSRT1**	1.46	7.58E-09	3.84E-08
**OMD**	-2.64	7.59E-09	3.84E-08
**IQSEC3**	4.51	7.59E-09	3.84E-08
**METTL21EP**	-4.82	7.61E-09	3.85E-08
**LINC01136**	-2.90	7.67E-09	3.88E-08
**ZNF219**	0.78	7.68E-09	3.88E-08
**MRPL21**	0.82	7.69E-09	3.89E-08
**ZNF701**	-0.78	7.75E-09	3.92E-08
**LILRB3**	3.86	7.80E-09	3.94E-08
**RP11-767L7.1**	-6.72	7.84E-09	3.96E-08
**G4403**	-2.29	7.84E-09	3.96E-08
**RP11-815J21.4**	-2.64	7.86E-09	3.97E-08
**SNX10**	2.24	7.88E-09	3.98E-08
**AC004893.11**	-1.36	7.90E-09	3.99E-08
**IRX5**	1.55	7.92E-09	4.00E-08
**G26457**	4.86	7.97E-09	4.02E-08
**E2F8**	-1.17	8.00E-09	4.04E-08
**CTD-2523D13.1**	-6.27	8.02E-09	4.05E-08
**ZNF350**	-0.69	8.04E-09	4.06E-08

**LAMA5-AS1**	-6.32	8.05E-09	4.06E-08
**G40866**	-4.17	8.06E-09	4.07E-08
**TBC1D30**	2.01	8.09E-09	4.08E-08
**MIR3619**	-6.38	8.09E-09	4.08E-08
**PTPMT1**	-1.51	8.12E-09	4.09E-08
**FOXC2**	3.67	8.13E-09	4.10E-08
**RP11-317F20.3**	-6.33	8.15E-09	4.11E-08
**GLIS3**	2.39	8.18E-09	4.12E-08
**CYSTM1**	1.28	8.21E-09	4.14E-08
**RP11-147L13.15**	1.47	8.23E-09	4.15E-08
**C1QL1**	3.85	8.24E-09	4.15E-08
**RP11-326G21.1**	2.01	8.24E-09	4.15E-08
**TAS2R13**	-6.34	8.32E-09	4.19E-08
**POLD2**	0.96	8.33E-09	4.19E-08
**SNORD105B**	-7.58	8.33E-09	4.19E-08
**CYP19A1**	3.35	8.34E-09	4.20E-08
**C1orf43**	0.70	8.37E-09	4.21E-08
**GABRD**	3.42	8.37E-09	4.21E-08
**PLCXD2**	-2.00	8.41E-09	4.23E-08
**NEFM**	7.73	8.42E-09	4.24E-08
**ITGA1**	1.93	8.42E-09	4.24E-08
**ATP9B**	-0.57	8.45E-09	4.25E-08
**HIST2H2AA4**	-3.11	8.56E-09	4.31E-08
**EFCAB7**	-0.67	8.57E-09	4.31E-08
**CORO1C**	-0.73	8.61E-09	4.33E-08
**HSD11B1**	-1.88	8.62E-09	4.33E-08
**RP11-365O16.6**	-1.84	8.64E-09	4.34E-08
**KLHL17**	1.75	8.64E-09	4.34E-08
**ADD3**	-1.20	8.66E-09	4.35E-08
**FUNDC2**	0.87	8.66E-09	4.35E-08
**B4GALNT4**	1.69	8.75E-09	4.40E-08
**BCLAF1**	-0.83	8.77E-09	4.41E-08
**SLC11A2**	-0.73	8.80E-09	4.42E-08
**C18orf32**	-0.89	8.83E-09	4.43E-08
**AFMID**	0.84	8.83E-09	4.43E-08
**ADTRP**	-1.68	8.83E-09	4.43E-08
**LSP1**	-1.26	8.84E-09	4.44E-08
**AL136531.1**	-4.74	8.87E-09	4.45E-08
**ZNF818P**	-1.94	8.95E-09	4.49E-08
**NOP10**	0.86	8.96E-09	4.49E-08
**FAM201A**	-2.02	9.11E-09	4.57E-08
**CAPN2**	1.25	9.13E-09	4.58E-08
**NDUFS5**	1.01	9.17E-09	4.60E-08
**ZBED2**	1.88	9.21E-09	4.61E-08
**PARD3B**	-0.88	9.22E-09	4.62E-08

**PIK3CD**	1.21	9.32E-09	4.67E-08
**PGGT1B**	-0.77	9.32E-09	4.67E-08
**KPTN**	1.52	9.33E-09	4.67E-08
**BTBD16**	-1.94	9.33E-09	4.67E-08
**ASB2**	1.84	9.35E-09	4.68E-08
**AC073626.2**	-6.26	9.39E-09	4.70E-08
**RAB8B**	-1.02	9.43E-09	4.72E-08
**RP11-950K24.2**	-6.32	9.45E-09	4.73E-08
**CCDC109B**	0.97	9.46E-09	4.73E-08
**RP5-1125A11.4**	-6.31	9.46E-09	4.73E-08
**RPAP3**	-0.55	9.48E-09	4.74E-08
**G25594**	-1.46	9.54E-09	4.77E-08
**RN7SKP69**	-6.16	9.56E-09	4.78E-08
**RP4-682C21.2**	-2.51	9.58E-09	4.79E-08
**CREB3L4**	0.93	9.61E-09	4.80E-08
**PSMB10**	1.40	9.64E-09	4.82E-08
**USP12-AS1**	-4.34	9.75E-09	4.87E-08
**DUSP9**	2.52	9.76E-09	4.88E-08
**PPP1R3F**	1.04	9.80E-09	4.90E-08
**AC074367.1**	-6.47	9.85E-09	4.92E-08
**ASH2L**	0.55	9.86E-09	4.92E-08
**TOR1AIP1**	-0.64	9.86E-09	4.92E-08
**RP11-687F6.4**	-6.39	9.89E-09	4.94E-08
**SNORD66**	-7.68	9.92E-09	4.95E-08
**CACYBPP1**	-6.38	9.92E-09	4.95E-08
**DCAF17**	-0.79	9.98E-09	4.98E-08
**PPP1R12A**	-0.71	9.99E-09	4.99E-08
**GS1-124K5.2**	-1.72	1.00E-08	5.01E-08
**ST3GAL1**	1.82	1.00E-08	5.01E-08
**ARAP1-AS2**	-3.46	1.01E-08	5.04E-08
**TOLLIP-AS1**	1.91	1.02E-08	5.07E-08
**GJA1P1**	-3.58	1.02E-08	5.10E-08
**WEE1**	-1.24	1.02E-08	5.10E-08
**RITA1**	0.80	1.03E-08	5.11E-08
**ASXL2**	-0.73	1.03E-08	5.12E-08
**FAM69C**	-1.74	1.03E-08	5.13E-08
**PCCA-AS1**	-4.62	1.03E-08	5.13E-08
**NUDCD3**	0.57	1.03E-08	5.13E-08
**SNORD67**	-6.63	1.03E-08	5.14E-08
**RP11-154H23.3**	-4.35	1.03E-08	5.14E-08
**URGCP**	0.66	1.04E-08	5.15E-08
**CHM**	-0.80	1.04E-08	5.18E-08
**JAK1**	-0.68	1.04E-08	5.18E-08
**RP11-440L14.1**	-1.83	1.04E-08	5.18E-08
**INPP5B**	-0.63	1.05E-08	5.20E-08

**MUTYH**	1.12	1.05E-08	5.20E-08
**RP11-635N19.2**	-4.58	1.05E-08	5.20E-08
**ABHD8**	1.07	1.05E-08	5.21E-08
**RP11-243J16.7**	-4.25	1.05E-08	5.23E-08
**RP11-1046B16.3**	-3.78	1.06E-08	5.25E-08
**KCNJ9**	-3.38	1.06E-08	5.26E-08
**ADNP2**	-0.65	1.06E-08	5.28E-08
**TSPAN17**	1.12	1.06E-08	5.29E-08
**MACC1-AS1**	-6.16	1.07E-08	5.32E-08
**RP11-135J2.3**	-5.19	1.07E-08	5.33E-08
**TNFRSF14**	1.68	1.08E-08	5.35E-08
**GPR61**	-2.50	1.08E-08	5.37E-08
**SNORA35**	-6.83	1.08E-08	5.39E-08
**FTLP6**	-6.34	1.09E-08	5.40E-08
**TST**	1.27	1.09E-08	5.40E-08
**RANGAP1**	0.83	1.09E-08	5.40E-08
**HGD**	-5.77	1.09E-08	5.41E-08
**TPBGL**	4.17	1.09E-08	5.42E-08
**snoU2_19**	-6.32	1.10E-08	5.45E-08
**PLXNC1**	-1.29	1.10E-08	5.48E-08
**RP11-53B2.2**	-3.68	1.11E-08	5.49E-08
**TCOF1**	0.78	1.11E-08	5.52E-08
**RP11-649G15.2**	-1.42	1.12E-08	5.54E-08
**RP11-44F14.6**	-1.63	1.12E-08	5.54E-08
**PRDX4**	1.22	1.12E-08	5.56E-08
**PIGN**	-0.94	1.13E-08	5.60E-08
**DDX18P5**	-6.31	1.13E-08	5.61E-08
**MAPK6PS3**	-6.43	1.13E-08	5.62E-08
**RP11-17G12.2**	-3.46	1.14E-08	5.64E-08
**EDEM1**	-0.66	1.14E-08	5.64E-08
**FGF7**	2.81	1.14E-08	5.64E-08
**G9222**	-3.84	1.14E-08	5.65E-08
**RNF41**	0.46	1.14E-08	5.65E-08
**RP11-391H12.8**	-3.67	1.15E-08	5.71E-08
**LMBR1L**	1.00	1.16E-08	5.74E-08
**RP11-462D18.2**	-6.48	1.17E-08	5.77E-08
**RP11-680F20.9**	-3.12	1.17E-08	5.77E-08
**MBOAT1**	-1.20	1.17E-08	5.78E-08
**RPS2P44**	-6.10	1.17E-08	5.80E-08
**RP11-52J3.3**	-6.23	1.17E-08	5.80E-08
**GAPDHP42**	-4.29	1.18E-08	5.82E-08
**ACAN**	4.40	1.18E-08	5.85E-08
**RP11-180P8.3**	-4.45	1.19E-08	5.86E-08
**G23773**	-3.75	1.19E-08	5.86E-08
**KLHDC1**	-0.96	1.19E-08	5.86E-08

**KCNJ1**	-3.64	1.19E-08	5.88E-08
**TTC39B**	-1.10	1.19E-08	5.88E-08
**RPS11**	0.79	1.20E-08	5.90E-08
**MFSD6**	-1.06	1.20E-08	5.92E-08
**EHD3**	1.42	1.20E-08	5.94E-08
**RP11-100G15.12**	-6.44	1.20E-08	5.94E-08
**HSD11B2**	2.42	1.21E-08	5.99E-08
**SYT11**	1.48	1.22E-08	6.01E-08
**ATXN2L**	1.18	1.22E-08	6.03E-08
**CCNC**	-0.76	1.22E-08	6.04E-08
**RP11-571I18.4**	-2.42	1.23E-08	6.06E-08
**BNIP1**	0.89	1.23E-08	6.07E-08
**AKR7A2**	0.63	1.23E-08	6.08E-08
**BAG5**	-0.82	1.24E-08	6.11E-08
**RP11-75A9.3**	-4.53	1.24E-08	6.13E-08
**RASSF8-AS1**	1.74	1.24E-08	6.13E-08
**JTB**	0.61	1.25E-08	6.14E-08
**VTI1A**	-0.57	1.25E-08	6.14E-08
**TMEM11**	0.71	1.25E-08	6.16E-08
**METAP2**	0.89	1.26E-08	6.20E-08
**G35951**	3.03	1.27E-08	6.24E-08
**FAM185BP**	-2.06	1.27E-08	6.25E-08
**LLGL1**	0.70	1.27E-08	6.25E-08
**ANXA6**	1.78	1.27E-08	6.26E-08
**GATAD1**	0.91	1.27E-08	6.26E-08
**SPHK2**	1.40	1.28E-08	6.30E-08
**RP11-210N13.1**	-6.09	1.28E-08	6.31E-08
**CTD-2561B21.5**	-4.45	1.28E-08	6.32E-08
**SLC25A39P1**	-6.27	1.29E-08	6.33E-08
**USP4**	0.53	1.29E-08	6.34E-08
**TFRC**	-1.46	1.29E-08	6.34E-08
**AATF**	0.67	1.29E-08	6.34E-08
**RP3-523K23.2**	-1.04	1.29E-08	6.36E-08
**SYF2**	0.85	1.30E-08	6.40E-08
**SLC36A3**	-3.47	1.30E-08	6.40E-08
**FCRLB**	1.75	1.30E-08	6.41E-08
**DCLRE1A**	-0.80	1.32E-08	6.49E-08
**FMO2**	3.06	1.33E-08	6.52E-08
**ETS2**	0.93	1.33E-08	6.53E-08
**RP13-93L13.2**	-6.34	1.34E-08	6.56E-08
**TFAP2A**	1.05	1.34E-08	6.60E-08
**FNDC4**	2.36	1.35E-08	6.61E-08
**RP11-982M15.5**	-6.13	1.35E-08	6.62E-08
**YIPF6**	-0.65	1.35E-08	6.65E-08
**ZNF816**	-1.11	1.36E-08	6.65E-08

**RP11-463D19.1**	-4.70	1.36E-08	6.66E-08
**RP11-449G16.1**	-4.21	1.39E-08	6.80E-08
**CYTH4**	2.51	1.39E-08	6.82E-08
**CTC-444N24.6**	-1.93	1.39E-08	6.82E-08
**DNM3**	-1.30	1.39E-08	6.83E-08
**RBM27**	-1.03	1.40E-08	6.86E-08
**MIF-AS1**	-3.30	1.42E-08	6.96E-08
**RAPGEF3**	1.58	1.43E-08	7.00E-08
**MIR573**	-2.44	1.43E-08	7.01E-08
**PRUNE**	0.62	1.43E-08	7.02E-08
**CA4**	4.07	1.45E-08	7.09E-08
**CDC42EP3P1**	-6.10	1.45E-08	7.09E-08
**CBARP**	1.48	1.45E-08	7.10E-08
**SMAGP**	0.93	1.45E-08	7.12E-08
**RHBDF1**	0.89	1.45E-08	7.12E-08
**TRIM16**	1.00	1.46E-08	7.12E-08
**RP11-276H19.1**	-1.27	1.46E-08	7.14E-08
**GAS7**	-1.00	1.46E-08	7.16E-08
**SLC35F1**	-1.95	1.47E-08	7.17E-08
**ZNF101**	-0.58	1.47E-08	7.17E-08
**AC016909.1**	-6.28	1.47E-08	7.19E-08
**FOXN3-AS2**	-6.38	1.47E-08	7.19E-08
**TMEM43**	-0.72	1.47E-08	7.19E-08
**RP13-39P12.2**	-2.20	1.48E-08	7.21E-08
**DDR1**	0.89	1.48E-08	7.24E-08
**PARPBP**	-1.12	1.49E-08	7.27E-08
**G28632**	-6.36	1.49E-08	7.28E-08
**TAS2R18**	-3.40	1.49E-08	7.29E-08
**RP11-17G12.3**	-6.26	1.49E-08	7.29E-08
**H2AFY2**	1.10	1.50E-08	7.31E-08
**ZNF844**	-0.74	1.50E-08	7.33E-08
**ZNF789**	-1.21	1.51E-08	7.36E-08
**RNF181**	0.74	1.51E-08	7.37E-08
**RP11-631N16.4**	-2.29	1.52E-08	7.40E-08
**MANEA**	-1.03	1.52E-08	7.41E-08
**PVT1**	1.64	1.52E-08	7.42E-08
**ULBP2**	4.26	1.52E-08	7.42E-08
**snoU109**	-1.89	1.52E-08	7.42E-08
**OR10AD1**	-6.38	1.53E-08	7.47E-08
**RP11-508N22.12**	-2.21	1.53E-08	7.48E-08
**RNU6-638P**	-6.25	1.54E-08	7.49E-08
**RP11-887P2.5**	-3.06	1.54E-08	7.53E-08
**STEAP4**	2.04	1.55E-08	7.54E-08
**RNU6-36P**	-6.21	1.55E-08	7.54E-08
**S100A14**	1.18	1.55E-08	7.54E-08

**KCNV1**	-6.13	1.55E-08	7.54E-08
**GBA**	1.14	1.55E-08	7.55E-08
**AL035610.2**	-6.27	1.56E-08	7.58E-08
**XPNPEP1**	-0.36	1.56E-08	7.59E-08
**GYLTL1B**	1.19	1.56E-08	7.60E-08
**XLOC_008614**	-2.44	1.56E-08	7.61E-08
**IRF1**	1.28	1.57E-08	7.62E-08
**DNAJC7**	0.49	1.57E-08	7.63E-08
**HSD17B13**	-4.66	1.58E-08	7.69E-08
**CTC-529I10.1**	-3.72	1.58E-08	7.71E-08
**AP000462.1**	-6.75	1.59E-08	7.74E-08
**MPST**	0.95	1.60E-08	7.77E-08
**GULP1**	-1.44	1.60E-08	7.77E-08
**PIGZ**	1.13	1.61E-08	7.84E-08
**MT-ND5**	3.70	1.61E-08	7.84E-08
**G13883**	4.92	1.62E-08	7.85E-08
**RP11-705O1.8**	-2.82	1.62E-08	7.85E-08
**WDTC1**	0.94	1.62E-08	7.85E-08
**RP11-812I20.2**	-6.16	1.63E-08	7.90E-08
**NFYC-AS1**	-2.08	1.64E-08	7.99E-08
**NRXN1**	-2.68	1.64E-08	7.99E-08
**THAP8**	0.96	1.65E-08	7.99E-08
**EEF2**	0.68	1.65E-08	8.00E-08
**RP11-8L18.2**	-3.43	1.65E-08	8.03E-08
**HPGDS**	-1.67	1.66E-08	8.05E-08
**RP11-255H23.5**	-5.13	1.67E-08	8.09E-08
**GIN1**	-0.89	1.67E-08	8.11E-08
**STEAP3**	1.23	1.68E-08	8.15E-08
**C5orf49**	-2.21	1.69E-08	8.18E-08
**DDX49**	0.67	1.69E-08	8.21E-08
**SLC22A15**	-0.99	1.70E-08	8.26E-08
**UBE2Q1**	0.47	1.71E-08	8.28E-08
**MAX**	0.51	1.71E-08	8.29E-08
**MYO3A**	-3.37	1.72E-08	8.34E-08
**FPGT**	-0.89	1.73E-08	8.37E-08
**ZNF568**	-0.64	1.74E-08	8.43E-08
**UGT1A6**	-2.06	1.74E-08	8.44E-08
**INSL6**	-4.51	1.75E-08	8.47E-08
**AC099684.1**	-4.64	1.76E-08	8.51E-08
**CKAP5**	-0.55	1.76E-08	8.54E-08
**TP53TG1**	0.89	1.77E-08	8.55E-08
**GIMAP8**	1.64	1.77E-08	8.57E-08
**NASP**	0.64	1.78E-08	8.61E-08
**COG6**	-0.72	1.78E-08	8.62E-08
**PRKRIRP7**	-2.23	1.79E-08	8.64E-08

**MFAP3L**	-1.25	1.79E-08	8.66E-08
**CTC-534A2.2**	-1.19	1.80E-08	8.70E-08
**MIS18A**	0.98	1.80E-08	8.70E-08
**OSCAR**	1.96	1.81E-08	8.77E-08
**SMYD4**	0.49	1.83E-08	8.86E-08
**SDAD1**	-0.63	1.84E-08	8.90E-08
**CHST10**	1.65	1.84E-08	8.92E-08
**RPL7L1P2**	-6.34	1.85E-08	8.92E-08
**AC007191.4**	-1.87	1.85E-08	8.92E-08
**AREG**	3.43	1.85E-08	8.93E-08
**RP11-210K20.5**	-6.23	1.85E-08	8.95E-08
**RP11-62G11.1**	-6.10	1.85E-08	8.95E-08
**AC004471.9**	-3.20	1.85E-08	8.95E-08
**FAM122A**	-0.65	1.86E-08	8.97E-08
**AF131215.4**	-6.29	1.86E-08	9.00E-08
**ADGRL3**	-2.40	1.87E-08	9.05E-08
**RP11-10L12.2**	-6.32	1.88E-08	9.05E-08
**FLAD1**	0.74	1.88E-08	9.09E-08
**HSF2**	-0.89	1.89E-08	9.13E-08
**PRR13**	0.93	1.90E-08	9.15E-08
**RP11-154D6.1**	-1.78	1.90E-08	9.16E-08
**RP11-388M20.1**	-3.56	1.90E-08	9.18E-08
**UBXN2B**	-0.93	1.91E-08	9.20E-08
**TPT1P10**	-6.17	1.91E-08	9.20E-08
**CPSF7**	0.63	1.91E-08	9.23E-08
**CTD-2562G15.3**	-3.53	1.92E-08	9.24E-08
**RP11-739N20.2**	-2.98	1.92E-08	9.25E-08
**FAM169A**	-1.82	1.93E-08	9.32E-08
**MYOM3**	1.88	1.95E-08	9.40E-08
**ANK2**	-1.80	1.95E-08	9.40E-08
**WDR53**	1.04	1.96E-08	9.43E-08
**ADCYAP1R1**	2.35	1.97E-08	9.47E-08
**COL18A1-AS1**	-3.85	1.97E-08	9.50E-08
**SNORA11**	-2.40	1.99E-08	9.57E-08
**SCAMP2**	0.62	2.00E-08	9.65E-08
**OSGEPL1**	-0.77	2.01E-08	9.65E-08
**SSRP1**	0.54	2.01E-08	9.68E-08
**SDE2**	-0.75	2.02E-08	9.71E-08
**KRT16P5**	4.36	2.02E-08	9.72E-08
**NAPA**	0.67	2.02E-08	9.73E-08
**TMED3**	0.76	2.03E-08	9.76E-08
**SMPD4**	0.70	2.04E-08	9.79E-08
**RPS21**	1.07	2.04E-08	9.79E-08
**THEGL**	-3.42	2.04E-08	9.83E-08
**DTL**	-1.01	2.05E-08	9.83E-08

**EXTL3**	0.71	2.05E-08	9.84E-08
**ADNP**	-0.52	2.05E-08	9.87E-08
**ZNF136**	-0.75	2.06E-08	9.89E-08
**ADGRB2**	1.66	2.06E-08	9.91E-08
**ROS1**	-5.96	2.07E-08	9.92E-08
**ADAMTS1**	1.59	2.07E-08	9.93E-08
**ITSN2**	-0.72	2.07E-08	9.95E-08
**SLC37A4**	0.78	2.08E-08	1.00E-07
**LGALS9B**	4.98	2.08E-08	1.00E-07
**MSH3**	-0.71	2.09E-08	1.00E-07
**DAAM1**	-0.92	2.10E-08	1.01E-07
**CWC27**	0.74	2.10E-08	1.01E-07
**POLR3GL**	1.04	2.10E-08	1.01E-07
**ANKRD54**	0.73	2.10E-08	1.01E-07
**TP73-AS1**	0.89	2.10E-08	1.01E-07
**XLOC_014080**	-2.65	2.11E-08	1.01E-07
**POC1B**	-0.64	2.11E-08	1.01E-07
**ZNRF3-IT1**	-6.06	2.12E-08	1.02E-07
**TP53INP1**	-0.77	2.12E-08	1.02E-07
**LINC00896**	-2.75	2.12E-08	1.02E-07
**NCL**	0.89	2.12E-08	1.02E-07
**CACNB3**	0.93	2.12E-08	1.02E-07
**TTC3-AS1**	-6.12	2.12E-08	1.02E-07
**PAAF1**	0.51	2.13E-08	1.02E-07
**XLOC_014097**	-2.72	2.13E-08	1.02E-07
**IGHG1**	6.44	2.13E-08	1.02E-07
**APLP1**	2.22	2.14E-08	1.02E-07
**ODF3L1**	-2.90	2.15E-08	1.03E-07
**AC009542.2**	-5.74	2.15E-08	1.03E-07
**HAO2**	-5.93	2.15E-08	1.03E-07
**ING2**	0.92	2.16E-08	1.03E-07
**NUDT4**	0.89	2.16E-08	1.03E-07
**RP11-278C7.5**	-2.78	2.16E-08	1.04E-07
**BOLA1**	0.75	2.17E-08	1.04E-07
**ZNF316**	0.88	2.18E-08	1.04E-07
**GDPD5**	2.19	2.19E-08	1.04E-07
**SFT2D1**	0.48	2.20E-08	1.05E-07
**MIR99A**	-6.09	2.21E-08	1.05E-07
**MMP24**	-1.71	2.21E-08	1.06E-07
**UHRF2P1**	-6.30	2.22E-08	1.06E-07
**RAB11FIP4**	-0.89	2.22E-08	1.06E-07
**EBF3**	1.66	2.23E-08	1.06E-07
**CLCF1**	2.00	2.23E-08	1.06E-07
**CD9**	0.89	2.23E-08	1.06E-07
**ZNF512B**	0.73	2.24E-08	1.07E-07

**SS18L1**	-0.74	2.24E-08	1.07E-07
**PITRM1-AS1**	-2.04	2.25E-08	1.07E-07
**CARNMT1**	-0.90	2.25E-08	1.07E-07
**TBCK**	-1.09	2.25E-08	1.07E-07
**SERINC5**	-0.72	2.25E-08	1.07E-07
**ADGB**	-3.17	2.25E-08	1.07E-07
**RFX1**	1.40	2.25E-08	1.07E-07
**FUZ**	0.92	2.25E-08	1.07E-07
**AC009120.5**	-1.84	2.25E-08	1.07E-07
**RP11-517I3.1**	-2.62	2.27E-08	1.08E-07
**RP4-794H19.1**	2.63	2.27E-08	1.08E-07
**SMTNL2**	2.93	2.28E-08	1.09E-07
**SORBS1**	-0.89	2.28E-08	1.09E-07
**LRRTM2**	-2.42	2.28E-08	1.09E-07
**COX5B**	0.87	2.31E-08	1.10E-07
**YEATS2-AS1**	-2.53	2.31E-08	1.10E-07
**RP11-410E4.1**	-2.81	2.32E-08	1.10E-07
**TFDP2**	-0.73	2.32E-08	1.11E-07
**DBN1**	1.89	2.33E-08	1.11E-07
**ABCC1**	0.74	2.33E-08	1.11E-07
**RP11-1D12.1**	-6.26	2.35E-08	1.12E-07
**KANK2**	1.46	2.36E-08	1.12E-07
**U3**	-1.74	2.37E-08	1.13E-07
**PKHD1**	-3.21	2.37E-08	1.13E-07
**PSG5**	-5.15	2.38E-08	1.13E-07
**RP11-160A9.3**	-6.25	2.38E-08	1.13E-07
**NATD1**	1.02	2.38E-08	1.13E-07
**RPL18**	0.83	2.38E-08	1.13E-07
**SIX2**	3.82	2.38E-08	1.13E-07
**SH3PXD2B**	1.13	2.39E-08	1.14E-07
**THUMPD1**	-0.59	2.40E-08	1.14E-07
**ABHD6**	-1.00	2.40E-08	1.14E-07
**ALS2CR12**	-2.67	2.40E-08	1.14E-07
**CTDP1**	0.78	2.40E-08	1.14E-07
**INPP5E**	0.99	2.42E-08	1.15E-07
**GPBP1**	-0.54	2.43E-08	1.15E-07
**RP11-289A15.1**	-4.63	2.44E-08	1.16E-07
**RMDN1**	-0.50	2.44E-08	1.16E-07
**RP11-45M22.3**	-4.86	2.47E-08	1.17E-07
**ZNF284**	-0.96	2.47E-08	1.17E-07
**FAM133B**	0.93	2.47E-08	1.17E-07
**PACS2**	0.77	2.48E-08	1.18E-07
**ENHO**	-3.21	2.49E-08	1.18E-07
**LARGE-AS1**	-6.23	2.50E-08	1.19E-07
**SYT14**	-3.72	2.50E-08	1.19E-07

**RP11-503N18.5**	-3.73	2.52E-08	1.19E-07
**RP11-95O2.1**	-3.05	2.52E-08	1.20E-07
**MIR920**	-6.50	2.52E-08	1.20E-07
**CNRIP1**	1.87	2.53E-08	1.20E-07
**CNOT4**	-0.65	2.53E-08	1.20E-07
**TMEM245**	-0.54	2.54E-08	1.20E-07
**RNU6-4P**	-5.11	2.54E-08	1.20E-07
**GAPDHP49**	-4.58	2.54E-08	1.20E-07
**TSPAN11**	2.00	2.54E-08	1.21E-07
**CCDC174**	0.47	2.55E-08	1.21E-07
**ZNF823**	-0.69	2.55E-08	1.21E-07
**TMEM240**	3.05	2.56E-08	1.21E-07
**NUDT21**	-0.81	2.56E-08	1.21E-07
**HOPX**	-1.45	2.56E-08	1.21E-07
**RPL7A**	0.83	2.57E-08	1.21E-07
**RP11-123O10.1**	-6.47	2.57E-08	1.22E-07
**IAH1**	0.74	2.57E-08	1.22E-07
**ADAM19**	2.32	2.58E-08	1.22E-07
**RP11-464C19.3**	-5.00	2.59E-08	1.22E-07
**PRSS35**	4.30	2.59E-08	1.23E-07
**SLCO4C1**	-5.29	2.59E-08	1.23E-07
**CADM4**	1.06	2.60E-08	1.23E-07
**MFSD1P1**	-5.84	2.60E-08	1.23E-07
**AC016831.7**	-3.01	2.61E-08	1.23E-07
**NOS2**	3.29	2.62E-08	1.24E-07
**C11orf42**	-4.27	2.63E-08	1.24E-07
**CYP20A1**	-0.62	2.63E-08	1.24E-07
**DOCK4-AS1**	-6.08	2.64E-08	1.25E-07
**RPL9P32**	-6.39	2.64E-08	1.25E-07
**HMGB1P14**	-5.09	2.64E-08	1.25E-07
**TBX6**	1.84	2.65E-08	1.25E-07
**NDUFB11**	0.77	2.66E-08	1.26E-07
**PPP1R13B**	0.63	2.67E-08	1.26E-07
**RP11-127B20.2**	-1.89	2.67E-08	1.26E-07
**MGST3**	0.76	2.70E-08	1.27E-07
**BOLA3**	1.08	2.70E-08	1.27E-07
**S1PR3**	1.70	2.70E-08	1.27E-07
**RP11-552C15.1**	-6.21	2.71E-08	1.28E-07
**GNLY**	2.84	2.73E-08	1.29E-07
**KRT8P46**	-2.40	2.74E-08	1.29E-07
**RP11-620J15.3**	1.65	2.75E-08	1.30E-07
**FCHSD2**	0.68	2.76E-08	1.30E-07
**CRNKL1**	-0.55	2.76E-08	1.30E-07
**RP11-16E23.4**	-5.12	2.76E-08	1.30E-07
**RP11-93G5.1**	-5.11	2.77E-08	1.30E-07

**XLOC_001195**	3.25	2.80E-08	1.32E-07
**AMN**	1.65	2.80E-08	1.32E-07
**BMP2K**	-0.88	2.83E-08	1.33E-07
**VTI1B**	-0.48	2.83E-08	1.33E-07
**KDM6B**	1.70	2.84E-08	1.34E-07
**RN7SKP230**	-3.73	2.84E-08	1.34E-07
**SGSM1**	-1.90	2.85E-08	1.34E-07
**FAM53B-AS1**	-5.96	2.86E-08	1.34E-07
**STRN3**	-0.62	2.86E-08	1.35E-07
**SMOC1**	2.40	2.86E-08	1.35E-07
**MBLAC1**	1.15	2.87E-08	1.35E-07
**GSTO2**	-1.01	2.87E-08	1.35E-07
**CSMD2**	3.39	2.87E-08	1.35E-07
**RP11-700J17.1**	-2.14	2.88E-08	1.35E-07
**LAMC1**	1.38	2.88E-08	1.35E-07
**FYTTD1**	-0.87	2.88E-08	1.36E-07
**PAX3**	-1.49	2.89E-08	1.36E-07
**SLC45A3**	1.62	2.89E-08	1.36E-07
**SMIM18**	-3.19	2.90E-08	1.36E-07
**AL590452.1**	-2.98	2.90E-08	1.36E-07
**NTF3**	2.01	2.91E-08	1.37E-07
**GPX7**	1.75	2.91E-08	1.37E-07
**WFIKKN2**	-4.11	2.93E-08	1.37E-07
**MTOR**	-0.61	2.93E-08	1.37E-07
**AC037445.1**	-6.19	2.93E-08	1.37E-07
**REPS2**	-0.95	2.95E-08	1.38E-07
**NAA20**	0.82	2.95E-08	1.38E-07
**EEF1A1P30**	-6.08	2.98E-08	1.40E-07
**TLE6**	2.95	2.98E-08	1.40E-07
**LINC00571**	-2.11	2.99E-08	1.40E-07
**EED**	-0.71	2.99E-08	1.40E-07
**HSPE1P13**	-6.25	3.00E-08	1.41E-07
**ZCCHC4**	-0.71	3.02E-08	1.41E-07
**G23772**	-6.09	3.03E-08	1.42E-07
**ZDHHC20**	-0.92	3.04E-08	1.42E-07
**AC068020.1**	-6.16	3.05E-08	1.43E-07
**SNORD99**	-4.60	3.05E-08	1.43E-07
**RP11-161I2.1**	-4.59	3.05E-08	1.43E-07
**KCNMB4**	-1.42	3.05E-08	1.43E-07
**CLK2**	1.01	3.08E-08	1.44E-07
**AP000695.6**	4.45	3.09E-08	1.45E-07
**RPL27A**	0.76	3.10E-08	1.45E-07
**CLNK**	-2.07	3.11E-08	1.45E-07
**CPED1**	-1.58	3.13E-08	1.46E-07
**LIMCH1**	1.06	3.13E-08	1.46E-07

**SPATA13**	-0.88	3.14E-08	1.47E-07
**RP11-164N3.3**	-5.88	3.15E-08	1.47E-07
**PLD1**	-0.94	3.15E-08	1.47E-07
**FKBPL**	1.20	3.17E-08	1.48E-07
**MAP2K5**	0.89	3.17E-08	1.48E-07
**DRG1**	0.68	3.17E-08	1.48E-07
**NRG4**	-1.23	3.19E-08	1.49E-07
**RP11-230C9.3**	-6.22	3.20E-08	1.49E-07
**CYP11A1**	4.32	3.20E-08	1.50E-07
**PQLC2**	0.78	3.21E-08	1.50E-07
**VTRNA1-1**	-6.24	3.22E-08	1.50E-07
**RP3-342P20.2**	-2.31	3.22E-08	1.50E-07
**PLK4**	-0.77	3.23E-08	1.51E-07
**PRKD3**	-0.67	3.24E-08	1.51E-07
**SLC2A3P2**	-6.08	3.27E-08	1.52E-07
**RP11-175O19.4**	-0.77	3.29E-08	1.54E-07
**ZNF43**	-1.30	3.30E-08	1.54E-07
**PLEKHH1**	-1.60	3.30E-08	1.54E-07
**RP11-557N21.1**	-4.54	3.31E-08	1.54E-07
**CD79B**	2.71	3.31E-08	1.54E-07
**PHF7**	0.92	3.32E-08	1.55E-07
**CUTC**	0.81	3.32E-08	1.55E-07
**RP3-323N1.2**	-6.25	3.32E-08	1.55E-07
**AMZ2**	0.74	3.35E-08	1.56E-07
**NEUROD2**	-3.29	3.35E-08	1.56E-07
**CYP1A2**	-7.42	3.37E-08	1.57E-07
**THEM6**	0.88	3.38E-08	1.57E-07
**RP1-5O6.4**	-6.04	3.38E-08	1.57E-07
**ATAD3A**	1.03	3.38E-08	1.58E-07
**GPR37L1**	-2.96	3.40E-08	1.58E-07
**CDH15**	4.18	3.40E-08	1.59E-07
**HSD17B6**	2.81	3.46E-08	1.61E-07
**SACM1L**	-0.64	3.46E-08	1.61E-07
**SPATA20**	1.34	3.48E-08	1.62E-07
**G4388**	-2.43	3.48E-08	1.62E-07
**GNL3L**	-0.63	3.49E-08	1.62E-07
**RP11-767L7.2**	-6.18	3.51E-08	1.63E-07
**FAM180B**	-2.84	3.51E-08	1.63E-07
**RP11-890B15.3**	-0.91	3.52E-08	1.64E-07
**EEF1A1P25**	-4.53	3.53E-08	1.64E-07
**HSD17B12**	-0.87	3.54E-08	1.65E-07
**DLGAP4**	0.79	3.55E-08	1.65E-07
**AL160175.1**	-5.88	3.58E-08	1.66E-07
**RP5-1033H22.2**	-2.39	3.59E-08	1.67E-07
**LINC01615**	4.41	3.59E-08	1.67E-07

**UBL4B**	4.94	3.61E-08	1.68E-07
**LINC00997**	1.65	3.61E-08	1.68E-07
**SNHG12**	-2.42	3.63E-08	1.68E-07
**ABCF1**	0.64	3.63E-08	1.69E-07
**MEX3A**	1.92	3.63E-08	1.69E-07
**HDAC3**	0.44	3.63E-08	1.69E-07
**RPLP1**	0.96	3.64E-08	1.69E-07
**TK1**	1.22	3.65E-08	1.69E-07
**RNF170**	-0.64	3.68E-08	1.71E-07
**LINC01137**	1.06	3.68E-08	1.71E-07
**LDHAL6A**	-2.56	3.70E-08	1.72E-07
**CTD-2339M3.1**	-6.07	3.70E-08	1.72E-07
**RP11-930P14.2**	1.98	3.72E-08	1.72E-07
**GLT1D1**	1.21	3.72E-08	1.73E-07
**TMEM26**	5.50	3.73E-08	1.73E-07
**S100A7**	4.97	3.74E-08	1.73E-07
**SNORD83A**	-3.68	3.75E-08	1.74E-07
**KMT2B**	1.35	3.76E-08	1.74E-07
**RHOA**	0.69	3.76E-08	1.74E-07
**BRD7P4**	-6.36	3.78E-08	1.75E-07
**SLAMF7**	1.87	3.78E-08	1.75E-07
**SRP54-AS1**	-1.70	3.78E-08	1.75E-07
**SLC35F2**	-0.80	3.79E-08	1.75E-07
**ABT1**	0.72	3.79E-08	1.75E-07
**RNF130**	1.13	3.79E-08	1.76E-07
**CPA6**	5.60	3.80E-08	1.76E-07
**TEK**	1.68	3.81E-08	1.76E-07
**ITPRIPL2**	-0.73	3.81E-08	1.76E-07
**RP11-709D24.8**	-3.18	3.82E-08	1.77E-07
**UBE2O**	0.63	3.84E-08	1.78E-07
**KDELC1**	1.44	3.85E-08	1.78E-07
**CTD-3157E16.1**	2.22	3.88E-08	1.80E-07
**HK2**	1.50	3.89E-08	1.80E-07
**ARTN**	3.58	3.89E-08	1.80E-07
**C6orf136**	1.12	3.90E-08	1.80E-07
**NPM1P43**	-6.21	3.91E-08	1.81E-07
**GPR1**	1.70	3.92E-08	1.81E-07
**LAT2**	1.85	3.93E-08	1.82E-07
**ZFAS1**	0.81	3.94E-08	1.82E-07
**RP5-1063M23.1**	-5.84	3.95E-08	1.83E-07
**RP11-150O12.3**	-2.89	3.95E-08	1.83E-07
**RP4-798A10.2**	-1.40	3.96E-08	1.83E-07
**RGS17P1**	-4.14	3.96E-08	1.83E-07
**SRGAP3-AS2**	-6.11	3.97E-08	1.84E-07
**RNU6-1275P**	-6.22	3.98E-08	1.84E-07

**TOP1MT**	0.80	4.00E-08	1.85E-07
**ABHD18**	-0.66	4.00E-08	1.85E-07
**TNIP3**	3.97	4.00E-08	1.85E-07
**HS1BP3**	0.72	4.03E-08	1.86E-07
**SCN9A**	-1.86	4.03E-08	1.86E-07
**AJUBA**	-0.77	4.03E-08	1.86E-07
**ADAMTS10**	2.29	4.04E-08	1.86E-07
**MYOM2**	-1.75	4.04E-08	1.86E-07
**AC116366.5**	-6.03	4.04E-08	1.87E-07
**KLHL42**	-0.60	4.05E-08	1.87E-07
**AP000962.2**	-3.69	4.06E-08	1.87E-07
**MMP17**	1.63	4.07E-08	1.87E-07
**RP11-631M6.3**	-4.66	4.07E-08	1.88E-07
**ARNT**	-0.47	4.07E-08	1.88E-07
**RP11-968A15.2**	-3.49	4.07E-08	1.88E-07
**FAM179A**	-2.72	4.08E-08	1.88E-07
**CALM2P2**	-2.38	4.09E-08	1.88E-07
**RP11-445J14.1**	-6.23	4.09E-08	1.88E-07
**AASDH**	-0.67	4.10E-08	1.89E-07
**CUL3**	-0.65	4.10E-08	1.89E-07
**HIST1H2AC**	-1.73	4.11E-08	1.89E-07
**ZNF35**	-0.72	4.11E-08	1.89E-07
**CTD-2666L21.2**	-2.59	4.12E-08	1.90E-07
**RP11-81A1.6**	-1.24	4.13E-08	1.90E-07
**RP11-506H20.2**	-6.14	4.14E-08	1.90E-07
**CCDC125**	-0.68	4.14E-08	1.91E-07
**PIGO**	0.57	4.15E-08	1.91E-07
**DSC1**	-1.62	4.17E-08	1.92E-07
**ABR**	0.65	4.18E-08	1.92E-07
**CD300A**	1.90	4.18E-08	1.92E-07
**GFOD2**	0.91	4.18E-08	1.92E-07
**CMKLR1**	2.38	4.19E-08	1.93E-07
**SS18**	-0.53	4.20E-08	1.93E-07
**SLC25A10**	1.23	4.20E-08	1.93E-07
**AGFG1**	-0.59	4.21E-08	1.93E-07
**RP11-126O1.4**	-5.02	4.22E-08	1.94E-07
**MESP2**	3.38	4.22E-08	1.94E-07
**CIAO1**	0.43	4.22E-08	1.94E-07
**NCOR1**	-0.57	4.23E-08	1.94E-07
**CENPI**	-1.10	4.24E-08	1.94E-07
**PRSS48**	-4.02	4.25E-08	1.95E-07
**KIF15**	-0.99	4.25E-08	1.95E-07
**MTUS2**	-1.98	4.27E-08	1.96E-07
**LINC00278**	-1.85	4.28E-08	1.96E-07
**RP11-849N15.3**	-2.97	4.31E-08	1.98E-07

**LPAR5**	1.10	4.31E-08	1.98E-07
**PDXP**	2.75	4.32E-08	1.98E-07
**GOLGA6L4**	2.65	4.34E-08	1.99E-07
**CTD-2201E9.1**	-5.37	4.35E-08	2.00E-07
**RP11-665C16.1**	-6.03	4.37E-08	2.00E-07
**GPR151**	-5.89	4.37E-08	2.00E-07
**C16orf45**	1.89	4.40E-08	2.02E-07
**MCM9**	-0.69	4.40E-08	2.02E-07
**C16orf91**	0.72	4.42E-08	2.03E-07
**MFAP3**	-0.79	4.42E-08	2.03E-07
**RP11-141B14.2**	-4.71	4.43E-08	2.03E-07
**SLC20A1**	1.35	4.43E-08	2.03E-07
**GRIN2D**	2.19	4.43E-08	2.03E-07
**MRFAP1L1**	0.65	4.44E-08	2.03E-07
**BIRC6-AS1**	-6.23	4.45E-08	2.03E-07
**BICD1**	1.70	4.46E-08	2.04E-07
**CACNG8**	4.14	4.46E-08	2.04E-07
**KRT18P37**	-5.94	4.47E-08	2.05E-07
**RP11-494M8.4**	-1.99	4.49E-08	2.06E-07
**RPL12P44**	-2.83	4.50E-08	2.06E-07
**TLR2**	2.02	4.51E-08	2.06E-07
**G23276**	-5.84	4.55E-08	2.08E-07
**MACROD2-AS1**	-6.52	4.56E-08	2.09E-07
**NDST3**	-3.21	4.58E-08	2.09E-07
**CH25H**	2.85	4.61E-08	2.11E-07
**SLC22A17**	1.29	4.64E-08	2.12E-07
**MUC15**	-1.48	4.65E-08	2.12E-07
**BMP7**	-1.24	4.65E-08	2.13E-07
**NOXA1**	1.84	4.66E-08	2.13E-07
**AC007317.1**	-6.28	4.67E-08	2.13E-07
**AC004076.5**	-2.11	4.69E-08	2.14E-07
**LINC01314**	-2.44	4.72E-08	2.16E-07
**FRG2HP**	-2.39	4.73E-08	2.16E-07
**RP11-307L3.4**	-5.91	4.73E-08	2.16E-07
**FUCA2**	1.31	4.73E-08	2.16E-07
**LCAT**	1.75	4.74E-08	2.16E-07
**UQCRBP1**	-6.02	4.75E-08	2.17E-07
**ACSBG2**	-3.44	4.75E-08	2.17E-07
**ZNF562**	-0.79	4.79E-08	2.19E-07
**TCAIM**	-0.78	4.83E-08	2.20E-07
**WDR49**	-5.99	4.86E-08	2.22E-07
**TXK**	-1.32	4.87E-08	2.22E-07
**GPX8**	1.78	4.88E-08	2.23E-07
**CTD-2623N2.3**	-1.91	4.90E-08	2.23E-07
**SNORA80B**	-6.25	4.92E-08	2.24E-07

**GOLM1**	1.13	4.93E-08	2.25E-07
**BRD8**	0.76	4.94E-08	2.25E-07
**RP11-1398P2.1**	3.07	4.94E-08	2.25E-07
**AC007690.1**	-4.45	4.95E-08	2.25E-07
**C9orf78**	0.75	4.95E-08	2.25E-07
**RP11-338I21.1**	-1.87	4.95E-08	2.25E-07
**SLX4IP**	-0.93	4.95E-08	2.26E-07
**AC006145.4**	-6.12	4.98E-08	2.27E-07
**WDR76**	-0.92	4.99E-08	2.27E-07
**CBX7**	0.85	4.99E-08	2.27E-07
**SRXN1**	2.56	5.01E-08	2.28E-07
**ACTRT3**	1.94	5.02E-08	2.28E-07
**RP11-167N5.5**	-5.97	5.03E-08	2.29E-07
**GHc-857G6.7**	-2.70	5.03E-08	2.29E-07
**AC016735.2**	-1.94	5.03E-08	2.29E-07
**STAC3**	1.84	5.05E-08	2.30E-07
**AF131215.1**	-6.04	5.05E-08	2.30E-07
**LARP7**	0.76	5.05E-08	2.30E-07
**RP5-1042K10.12**	-5.95	5.07E-08	2.31E-07
**5-Mar**	0.72	5.08E-08	2.31E-07
**RP11-1148O4.2**	-5.94	5.09E-08	2.31E-07
**ADAMTS16**	3.13	5.10E-08	2.32E-07
**REC114**	-5.94	5.10E-08	2.32E-07
**RBM14-RBM4**	-4.93	5.13E-08	2.33E-07
**RP11-315D13.1**	-6.09	5.14E-08	2.34E-07
**SF3A1**	0.52	5.15E-08	2.34E-07
**RERG**	2.03	5.16E-08	2.34E-07
**CCDC113**	-0.81	5.16E-08	2.34E-07
**MIR215**	-5.77	5.19E-08	2.35E-07
**CTD-3253I12.1**	-6.16	5.24E-08	2.38E-07
**LPAR4**	3.29	5.28E-08	2.40E-07
**RP11-526F3.1**	-6.15	5.28E-08	2.40E-07
**MAP9**	1.57	5.29E-08	2.40E-07
**SCCPDH**	-1.26	5.31E-08	2.41E-07
**PRKCQ-AS1**	2.88	5.32E-08	2.41E-07
**GRTP1-AS1**	-5.07	5.32E-08	2.41E-07
**SIRT2**	0.69	5.32E-08	2.41E-07
**CEP72**	-1.16	5.33E-08	2.42E-07
**XLOC_005563**	4.60	5.35E-08	2.42E-07
**RP11-508N12.2**	-5.98	5.36E-08	2.43E-07
**KLHL30**	3.06	5.37E-08	2.43E-07
**SEMA6A-AS1**	-2.19	5.40E-08	2.45E-07
**NEK6**	1.62	5.42E-08	2.45E-07
**KDM4A-AS1**	-2.14	5.44E-08	2.46E-07
**CTD-2227E11.1**	-2.79	5.44E-08	2.47E-07

**DUSP8**	1.84	5.45E-08	2.47E-07
**NFE2L3**	-1.02	5.45E-08	2.47E-07
**CDA**	2.10	5.46E-08	2.47E-07
**HOXA4**	1.86	5.48E-08	2.48E-07
**RP11-45M22.2**	-1.87	5.49E-08	2.48E-07
**LOXHD1**	2.96	5.53E-08	2.50E-07
**PRDX3P1**	-2.46	5.54E-08	2.51E-07
**SMG1P4**	-7.01	5.54E-08	2.51E-07
**RP11-1110F20.1**	-4.27	5.59E-08	2.53E-07
**UCHL5**	-0.76	5.61E-08	2.54E-07
**RP11-563J2.2**	-3.37	5.62E-08	2.54E-07
**C11orf45**	-1.41	5.62E-08	2.54E-07
**URB2**	-1.02	5.65E-08	2.55E-07
**MIR600HG**	-1.27	5.65E-08	2.56E-07
**RP11-855A2.2**	-3.75	5.67E-08	2.56E-07
**CCT8P1**	-1.94	5.68E-08	2.56E-07
**CYP4F22**	1.05	5.68E-08	2.56E-07
**MEF2C**	1.52	5.69E-08	2.57E-07
**KCND3**	-0.89	5.71E-08	2.58E-07
**RP11-365D23.4**	-6.11	5.73E-08	2.59E-07
**PINLYP**	1.41	5.73E-08	2.59E-07
**ENPP7P12**	-6.15	5.74E-08	2.59E-07
**BTF3P12**	-6.27	5.74E-08	2.59E-07
**METAP1**	0.53	5.75E-08	2.60E-07
**RP3-402G11.26**	2.08	5.76E-08	2.60E-07
**PPP2R5B**	0.80	5.78E-08	2.61E-07
**G35839**	-2.81	5.79E-08	2.61E-07
**VAC14-AS1**	-2.08	5.83E-08	2.63E-07
**COL19A1**	-3.65	5.86E-08	2.64E-07
**ZNF558**	-0.54	5.87E-08	2.65E-07
**G40216**	-3.35	5.92E-08	2.67E-07
**G9217**	-3.81	5.92E-08	2.67E-07
**SEMA3E**	-2.28	5.96E-08	2.69E-07
**TCTE3**	-1.52	6.00E-08	2.70E-07
**RP11-727F15.12**	-2.51	6.00E-08	2.71E-07
**WIPI1**	0.92	6.02E-08	2.71E-07
**RP11-608O8.2**	-4.23	6.04E-08	2.72E-07
**PARD6G**	-0.89	6.07E-08	2.73E-07
**RP11-420K10.1**	-6.10	6.07E-08	2.73E-07
**UST-AS1**	-6.10	6.08E-08	2.74E-07
**SLC12A9**	1.01	6.08E-08	2.74E-07
**MIOS**	-0.72	6.10E-08	2.75E-07
**CALU**	1.29	6.11E-08	2.75E-07
**RP11-35J10.7**	-3.72	6.12E-08	2.76E-07
**ALG1**	0.91	6.14E-08	2.76E-07

**RP11-691N7.6**	-2.77	6.14E-08	2.76E-07
**POLR3K**	0.92	6.15E-08	2.76E-07
**RBP1**	1.24	6.15E-08	2.77E-07
**EMG1**	-0.84	6.18E-08	2.78E-07
**RP11-572P18.1**	-1.19	6.23E-08	2.80E-07
**SNORD6**	-3.24	6.25E-08	2.81E-07
**AC013472.3**	-3.10	6.26E-08	2.81E-07
**GLS2**	-2.45	6.28E-08	2.82E-07
**PLA2G16**	2.63	6.29E-08	2.83E-07
**TMEM200B**	1.49	6.30E-08	2.83E-07
**G2584**	4.04	6.31E-08	2.84E-07
**XXbac-B562F10.11**	-3.45	6.31E-08	2.84E-07
**RP11-53B2.3**	-4.16	6.32E-08	2.84E-07
**PRKCZ**	0.92	6.32E-08	2.84E-07
**ING4**	0.66	6.33E-08	2.84E-07
**RP11-16E18.3**	-1.83	6.38E-08	2.86E-07
**CTD-2600H12.2**	-1.85	6.41E-08	2.88E-07
**RP11-508N12.3**	-6.04	6.45E-08	2.89E-07
**LINC-PINT**	-1.83	6.45E-08	2.89E-07
**RP11-185E12.2**	-6.04	6.45E-08	2.90E-07
**SPINT1**	0.85	6.48E-08	2.91E-07
**RP11-91J19.2**	-2.96	6.49E-08	2.91E-07
**GMEB2**	0.58	6.53E-08	2.93E-07
**G40865**	-3.13	6.53E-08	2.93E-07
**CYP46A1**	2.55	6.55E-08	2.94E-07
**RP11-298J20.4**	1.79	6.59E-08	2.96E-07
**RP11-576I22.2**	3.38	6.60E-08	2.96E-07
**PRF1**	1.94	6.61E-08	2.96E-07
**PI15**	3.17	6.63E-08	2.97E-07
**RP11-16E23.3**	-5.99	6.64E-08	2.97E-07
**NPTN-IT1**	-1.73	6.65E-08	2.98E-07
**LA16c-431H6.7**	-2.89	6.66E-08	2.98E-07
**AIF1**	2.14	6.66E-08	2.98E-07
**DSE**	-0.66	6.70E-08	3.00E-07
**GPT**	-1.61	6.72E-08	3.01E-07
**SCMH1**	0.85	6.72E-08	3.01E-07
**GTF2IRD2**	1.04	6.73E-08	3.01E-07
**ABCA10**	-1.90	6.77E-08	3.03E-07
**IDH3A**	0.63	6.78E-08	3.03E-07
**RP11-18H7.1**	-1.30	6.82E-08	3.05E-07
**LCE3A**	5.70	6.83E-08	3.06E-07
**RP1-30M3.5**	-1.90	6.84E-08	3.06E-07
**RPL23AP53**	-1.02	6.85E-08	3.06E-07
**ELP4**	-0.72	6.86E-08	3.07E-07
**HK3**	3.23	6.86E-08	3.07E-07

**HNRNPA1L2**	1.20	6.90E-08	3.08E-07
**RP11-6L6.4**	-5.88	6.92E-08	3.09E-07
**RP11-234A1.1**	-1.16	6.92E-08	3.09E-07
**TMEM209**	-0.81	6.94E-08	3.10E-07
**TP53TG5**	-1.67	6.98E-08	3.12E-07
**SHTN1**	-0.71	6.99E-08	3.12E-07
**ABCE1**	-0.83	7.00E-08	3.12E-07
**RP11-225N10.3**	-5.98	7.01E-08	3.13E-07
**LINC01296**	5.18	7.03E-08	3.14E-07
**VASH1**	1.85	7.04E-08	3.14E-07
**NAV2-AS5**	-6.26	7.04E-08	3.14E-07
**TAL1**	1.90	7.07E-08	3.16E-07
**PLCZ1**	-2.38	7.08E-08	3.16E-07
**RP11-212D19.5**	-5.81	7.10E-08	3.17E-07
**VIL1**	-5.74	7.15E-08	3.19E-07
**SNRPGP10**	1.71	7.15E-08	3.19E-07
**FOXCUT**	3.13	7.20E-08	3.21E-07
**SKINTL**	-5.89	7.20E-08	3.21E-07
**RPL7P57**	-6.25	7.20E-08	3.21E-07
**TRIM59**	-0.96	7.23E-08	3.22E-07
**RP11-573M3.2**	-6.11	7.25E-08	3.23E-07
**DUTP7**	-5.82	7.26E-08	3.24E-07
**CDKL1**	-1.52	7.28E-08	3.25E-07
**MLX**	0.54	7.29E-08	3.25E-07
**GOT2**	0.67	7.32E-08	3.26E-07
**UGT2A1**	-7.15	7.36E-08	3.28E-07
**AC007787.2**	-4.44	7.37E-08	3.28E-07
**TAGLN2P1**	-3.16	7.39E-08	3.29E-07
**RP11-345I18.4**	-5.96	7.39E-08	3.29E-07
**SMAP2**	0.94	7.42E-08	3.31E-07
**ATXN7L3B**	-0.52	7.47E-08	3.33E-07
**FCGR1B**	4.66	7.48E-08	3.33E-07
**CMIP**	1.11	7.48E-08	3.33E-07
**WSCD1**	1.89	7.49E-08	3.33E-07
**RP11-311B14.1**	-6.19	7.51E-08	3.34E-07
**ACTN4**	0.76	7.53E-08	3.35E-07
**RP11-512N4.2**	-6.10	7.57E-08	3.37E-07
**JRK**	-0.88	7.57E-08	3.37E-07
**RP11-498D10.5**	-2.63	7.59E-08	3.38E-07
**CTD-2540L5.9**	-5.90	7.60E-08	3.38E-07
**ADAMTSL4-AS1**	-1.85	7.60E-08	3.38E-07
**LINC00493**	-1.08	7.61E-08	3.38E-07
**TCEAL3**	1.05	7.62E-08	3.39E-07
**KIF26A**	1.45	7.62E-08	3.39E-07
**TET2-AS1**	-2.89	7.63E-08	3.39E-07

**PPP1R3G**	1.69	7.64E-08	3.40E-07
**RP11-748C4.1**	-6.11	7.66E-08	3.40E-07
**B3GAT2**	-1.90	7.69E-08	3.41E-07
**RP11-351I21.6**	-6.72	7.69E-08	3.42E-07
**SMG1P6**	-2.77	7.72E-08	3.43E-07
**HSPA8P15**	-4.15	7.73E-08	3.43E-07
**UBTD1**	0.97	7.75E-08	3.44E-07
**MT-ND4L**	2.53	7.76E-08	3.44E-07
**ATP5I**	-1.02	7.76E-08	3.45E-07
**TCAF2**	1.18	7.77E-08	3.45E-07
**RP11-180C16.1**	-4.53	7.80E-08	3.46E-07
**CHP2**	-2.25	7.81E-08	3.46E-07
**TCEA1P4**	-3.51	7.84E-08	3.48E-07
**ZNF117**	-1.95	7.90E-08	3.50E-07
**SMPD1**	0.68	7.93E-08	3.52E-07
**AC008746.12**	-2.78	7.93E-08	3.52E-07
**TREM2**	3.88	7.94E-08	3.52E-07
**CTD-2201E18.3**	1.28	7.94E-08	3.52E-07
**AP004289.1**	-6.11	7.96E-08	3.53E-07
**AP001046.6**	-2.90	7.97E-08	3.53E-07
**JOSD1**	0.56	8.00E-08	3.55E-07
**MYL3**	3.11	8.01E-08	3.55E-07
**SNORD91B**	-5.40	8.04E-08	3.56E-07
**RP11-124D2.7**	-3.55	8.06E-08	3.57E-07
**CTD-2619J13.16**	-1.80	8.08E-08	3.58E-07
**HPS6**	0.69	8.08E-08	3.58E-07
**MRPL27**	0.68	8.09E-08	3.58E-07
**ORC3**	-0.63	8.09E-08	3.58E-07
**AP003025.2**	-4.64	8.09E-08	3.58E-07
**DZIP1**	1.85	8.12E-08	3.59E-07
**TMEM175**	0.92	8.13E-08	3.60E-07
**NCOR2**	1.15	8.13E-08	3.60E-07
**PMS2**	-0.63	8.13E-08	3.60E-07
**PIK3R3**	1.96	8.14E-08	3.60E-07
**STAU1**	0.46	8.15E-08	3.61E-07
**ERCC2**	0.98	8.19E-08	3.62E-07
**ZNF154**	-1.78	8.20E-08	3.62E-07
**C6orf1**	0.81	8.21E-08	3.63E-07
**GRAP**	3.93	8.22E-08	3.63E-07
**RP11-369K16.1**	-2.38	8.22E-08	3.63E-07
**SGMS1**	-0.84	8.23E-08	3.64E-07
**SCARNA15**	-5.65	8.24E-08	3.64E-07
**snoU2-30**	-5.84	8.27E-08	3.65E-07
**CTC-250P20.1**	-5.89	8.27E-08	3.65E-07
**RP11-397P13.6**	-4.51	8.29E-08	3.66E-07

**MCOLN2**	-1.78	8.29E-08	3.66E-07
**STAM2**	-0.66	8.33E-08	3.68E-07
**RP11-215P8.2**	-2.84	8.34E-08	3.68E-07
**LINC01272**	2.59	8.34E-08	3.68E-07
**RP11-345P4.9**	0.94	8.35E-08	3.69E-07
**KIAA1211L**	1.16	8.36E-08	3.69E-07
**HHEX**	1.75	8.37E-08	3.69E-07
**PHTF1**	1.36	8.42E-08	3.71E-07
**RFX8**	3.23	8.46E-08	3.73E-07
**RP11-23J9.5**	-2.40	8.48E-08	3.74E-07
**FAM167A-AS1**	-4.72	8.55E-08	3.77E-07
**GOPC**	-0.59	8.59E-08	3.79E-07
**C17orf104**	-1.85	8.59E-08	3.79E-07
**RP11-642A1.1**	-6.00	8.63E-08	3.80E-07
**RP11-127I20.8**	-2.92	8.67E-08	3.82E-07
**RP5-1126H10.2**	-1.79	8.67E-08	3.82E-07
**NDUFAF1**	0.97	8.69E-08	3.83E-07
**CTD-2012K14.5**	-3.00	8.69E-08	3.83E-07
**AC079753.5**	-3.53	8.69E-08	3.83E-07
**RP11-123C21.2**	-6.06	8.72E-08	3.84E-07
**RN7SL502P**	-5.81	8.73E-08	3.84E-07
**RP11-513M16.8**	-1.42	8.73E-08	3.84E-07
**PRRX1**	2.15	8.78E-08	3.86E-07
**AC006019.4**	-6.51	8.78E-08	3.86E-07
**WNK3**	-1.45	8.79E-08	3.87E-07
**ETV7**	-1.14	8.79E-08	3.87E-07
**THSD4**	0.95	8.85E-08	3.89E-07
**ADH7**	2.81	8.88E-08	3.91E-07
**ZNF676**	-3.65	8.90E-08	3.91E-07
**RP4-734G22.3**	-1.94	8.95E-08	3.94E-07
**FCHO2**	-0.76	8.98E-08	3.95E-07
**MIPEP**	-0.81	8.99E-08	3.95E-07
**AL627171.2**	-3.11	9.11E-08	4.00E-07
**TCEB1P31**	-6.08	9.12E-08	4.01E-07
**TSC2**	0.65	9.15E-08	4.02E-07
**RP11-90O23.1**	-6.06	9.16E-08	4.02E-07
**RP11-117N2.2**	-5.89	9.17E-08	4.03E-07
**AC005540.3**	-3.66	9.17E-08	4.03E-07
**AC000123.4**	-1.76	9.19E-08	4.04E-07
**DIRC2**	-0.83	9.24E-08	4.06E-07
**ZNF367**	-1.10	9.26E-08	4.07E-07
**SMARCE1**	-0.58	9.28E-08	4.07E-07
**RP5-1021I20.6**	-5.94	9.29E-08	4.08E-07
**C11orf65**	-1.68	9.32E-08	4.09E-07
**HOXC12**	3.09	9.34E-08	4.10E-07

**VSIG10**	-0.96	9.36E-08	4.11E-07
**FAM43B**	2.86	9.39E-08	4.12E-07
**POLR3B**	-0.89	9.39E-08	4.12E-07
**RP11-101O6.2**	-5.76	9.40E-08	4.12E-07
**RP11-46A10.6**	-5.76	9.43E-08	4.13E-07
**CAMK2B**	-2.18	9.46E-08	4.15E-07
**NOP2**	0.79	9.47E-08	4.15E-07
**ITGAX**	2.48	9.47E-08	4.15E-07
**PDIA6**	0.80	9.48E-08	4.15E-07
**PHACTR2P1**	-3.62	9.48E-08	4.15E-07
**RP11-441O15.3**	-2.09	9.50E-08	4.16E-07
**GTPBP8**	-0.61	9.50E-08	4.16E-07
**MANBA**	0.65	9.51E-08	4.17E-07
**PDE9A**	2.49	9.57E-08	4.19E-07
**TNPO2**	0.62	9.60E-08	4.20E-07
**SULT1A1**	2.75	9.61E-08	4.21E-07
**RP11-152N13.16**	-2.99	9.63E-08	4.22E-07
**CYSLTR1**	-1.65	9.66E-08	4.23E-07
**LMNA**	0.82	9.66E-08	4.23E-07
**TAB3**	-0.68	9.66E-08	4.23E-07
**SERPINA9**	-2.73	9.71E-08	4.25E-07
**AC083949.1**	-5.91	9.73E-08	4.25E-07
**KIAA1462**	2.23	9.73E-08	4.26E-07
**RP11-3J10.7**	-6.02	9.74E-08	4.26E-07
**RP11-475E11.2**	-5.95	9.77E-08	4.27E-07
**RP11-474P2.7**	-4.80	9.78E-08	4.28E-07
**LONRF2**	-2.24	9.78E-08	4.28E-07
**CTA-243E7.1**	-2.26	9.82E-08	4.29E-07
**AAED1**	0.98	9.86E-08	4.31E-07
**AC092338.5**	-2.09	9.88E-08	4.32E-07
**GS1-165B14.2**	-6.30	9.97E-08	4.36E-07
**SNORD71**	-5.65	1.00E-07	4.37E-07
**RP11-303G3.6**	-2.65	1.01E-07	4.39E-07
**MILR1**	3.34	1.01E-07	4.39E-07
**ARHGAP42**	-0.92	1.01E-07	4.40E-07
**AL773572.7**	-6.06	1.01E-07	4.41E-07
**TMEM205**	0.95	1.01E-07	4.41E-07
**SEMA5B**	2.90	1.01E-07	4.41E-07
**G31307**	3.15	1.01E-07	4.42E-07
**BCL2L15**	2.74	1.01E-07	4.42E-07
**DYNLL1**	1.07	1.02E-07	4.43E-07
**RP11-670E13.6**	1.80	1.02E-07	4.43E-07
**DYNC1LI2**	-0.44	1.02E-07	4.44E-07
**AC000120.7**	-3.94	1.02E-07	4.44E-07
**C10orf113**	-4.45	1.02E-07	4.45E-07

**XLOC_005458**	-2.43	1.02E-07	4.45E-07
**RP4-677H15.4**	-5.96	1.02E-07	4.46E-07
**AF178030.2**	-4.03	1.03E-07	4.48E-07
**BNIP3P42**	-5.87	1.03E-07	4.48E-07
**SLC25A27**	-1.73	1.03E-07	4.49E-07
**AP001962.3**	-5.79	1.03E-07	4.50E-07
**CNOT10**	-0.48	1.03E-07	4.50E-07
**SNORA67**	-3.52	1.04E-07	4.52E-07
**PPIG**	0.81	1.04E-07	4.54E-07
**C19orf67**	-5.90	1.04E-07	4.55E-07
**NEDD1**	-0.70	1.05E-07	4.55E-07
**IGSF8**	0.88	1.05E-07	4.57E-07
**MED21**	-0.60	1.05E-07	4.57E-07
**HMGN1P8**	-3.43	1.05E-07	4.58E-07
**LYAR**	1.06	1.06E-07	4.61E-07
**FAM13A**	-0.90	1.06E-07	4.62E-07
**TOR2A**	1.08	1.06E-07	4.62E-07
**VIMP**	0.69	1.06E-07	4.63E-07
**RP11-797J4.1**	-6.05	1.06E-07	4.63E-07
**NPM1P30**	-5.95	1.07E-07	4.63E-07
**AC007787.3**	-4.39	1.07E-07	4.64E-07
**PGM5P2**	-2.28	1.07E-07	4.65E-07
**NKX1-2**	-3.08	1.07E-07	4.66E-07
**RP11-532F6.2**	-6.00	1.07E-07	4.66E-07
**ENDOU**	-1.17	1.07E-07	4.66E-07
**VIPR1-AS1**	-2.14	1.08E-07	4.68E-07
**LINC01521**	1.98	1.08E-07	4.69E-07
**MS4A7**	2.68	1.08E-07	4.69E-07
**FLJ37035**	-2.16	1.08E-07	4.69E-07
**PYDC1**	2.22	1.08E-07	4.70E-07
**RSPO1**	1.73	1.09E-07	4.72E-07
**WDR75**	-0.49	1.09E-07	4.72E-07
**RP11-359B20.1**	-5.95	1.09E-07	4.74E-07
**EPB41L4A**	-0.96	1.09E-07	4.74E-07
**GIT1**	0.83	1.09E-07	4.74E-07
**RP11-747H7.3**	-1.75	1.09E-07	4.75E-07
**AC110781.3**	-5.87	1.10E-07	4.77E-07
**SMPDL3A**	-0.92	1.10E-07	4.77E-07
**MTX1P1**	1.59	1.10E-07	4.78E-07
**G39039**	-2.65	1.10E-07	4.79E-07
**NOL11**	-0.57	1.11E-07	4.81E-07
**ADAMTSL4**	1.61	1.11E-07	4.81E-07
**RP11-74C1.2**	-3.32	1.11E-07	4.81E-07
**BBX**	-0.58	1.11E-07	4.81E-07
**ZMYM3**	0.65	1.11E-07	4.82E-07

**HBG2**	-6.40	1.12E-07	4.84E-07
**LINC01278**	1.41	1.12E-07	4.85E-07
**AC017048.2**	-6.29	1.12E-07	4.86E-07
**RP11-390D11.1**	-3.67	1.12E-07	4.86E-07
**SCFD1**	-0.64	1.12E-07	4.87E-07
**AK3P5**	-5.95	1.13E-07	4.90E-07
**AC098614.2**	2.06	1.13E-07	4.91E-07
**ARMCX2**	1.51	1.14E-07	4.92E-07
**AC007256.5**	-3.76	1.14E-07	4.94E-07
**ALPK2**	2.77	1.14E-07	4.95E-07
**RNU6-308P**	-6.08	1.14E-07	4.95E-07
**PRKCA-AS1**	-6.28	1.14E-07	4.95E-07
**RNU6-9**	-5.52	1.15E-07	4.97E-07
**HARS**	0.54	1.15E-07	4.97E-07
**DDX17**	-1.02	1.15E-07	4.98E-07
**SLC26A3**	-6.20	1.16E-07	5.01E-07
**MAP2K3**	1.14	1.16E-07	5.03E-07
**POLL**	0.97	1.17E-07	5.04E-07
**ASCC1**	0.56	1.17E-07	5.05E-07
**RP11-141M1.3**	-2.65	1.17E-07	5.05E-07
**RP11-45A17.4**	-3.20	1.17E-07	5.06E-07
**NODAL**	-3.07	1.17E-07	5.06E-07
**TAS2R14**	-2.18	1.17E-07	5.07E-07
**POLR2D**	-0.63	1.17E-07	5.07E-07
**AC007000.10**	-2.78	1.18E-07	5.08E-07
**IKZF4**	1.29	1.18E-07	5.09E-07
**CTC-287O8.1**	-5.96	1.18E-07	5.10E-07
**WDR11**	-0.44	1.19E-07	5.13E-07
**FAM78B**	1.63	1.19E-07	5.14E-07
**GUSBP9**	-2.57	1.19E-07	5.15E-07
**CD1A**	-1.81	1.19E-07	5.16E-07
**ZNF839**	-0.62	1.19E-07	5.16E-07
**SPATA21**	-2.81	1.20E-07	5.16E-07
**BATF2**	2.72	1.20E-07	5.16E-07
**USO1**	-0.72	1.21E-07	5.23E-07
**LAPTM5**	1.87	1.22E-07	5.25E-07
**LIN7A**	1.92	1.22E-07	5.25E-07
**BNIP3P10**	-5.67	1.22E-07	5.26E-07
**RP4-683L5.1**	-6.14	1.22E-07	5.27E-07
**RP11-384C4.6**	-6.06	1.23E-07	5.28E-07
**LINC01268**	-2.88	1.23E-07	5.31E-07
**RP1-168P16.3**	-6.11	1.23E-07	5.31E-07
**AARD**	3.24	1.23E-07	5.31E-07
**F7**	2.87	1.23E-07	5.31E-07
**ASF1A**	-0.96	1.24E-07	5.32E-07

**ARHGAP11A**	-0.94	1.24E-07	5.33E-07
**LL21NC02-21A1.1**	4.49	1.24E-07	5.35E-07
**TRIM27**	0.70	1.25E-07	5.36E-07
**STXBP5-AS1**	1.48	1.25E-07	5.36E-07
**HACD4**	-1.29	1.25E-07	5.39E-07
**CTD-3064M3.3**	2.55	1.25E-07	5.39E-07
**PTPN2P1**	-6.01	1.26E-07	5.40E-07
**GRPEL2**	-0.66	1.26E-07	5.41E-07
**MARVELD2**	-0.91	1.26E-07	5.43E-07
**CCDC88C**	1.04	1.26E-07	5.43E-07
**GRID2IP**	-1.61	1.27E-07	5.45E-07
**NHLH1**	-2.74	1.27E-07	5.45E-07
**CREB5**	-1.28	1.27E-07	5.45E-07
**FAM225B**	4.11	1.27E-07	5.47E-07
**ZBTB43**	-0.79	1.27E-07	5.47E-07
**RP11-355O1.7**	-6.05	1.27E-07	5.48E-07
**TMEM105**	3.07	1.28E-07	5.50E-07
**PEG3**	-1.77	1.28E-07	5.50E-07
**AC092597.3**	-3.62	1.29E-07	5.54E-07
**HLA-DRA**	1.99	1.29E-07	5.56E-07
**FAM160B2**	1.11	1.30E-07	5.56E-07
**MARK2**	0.73	1.30E-07	5.57E-07
**RP11-210K20.2**	-5.76	1.30E-07	5.59E-07
**TRAPPC6B**	-0.58	1.30E-07	5.60E-07
**RP11-504G3.2**	-5.65	1.31E-07	5.64E-07
**TRAPPC6A**	0.94	1.32E-07	5.65E-07
**SLC1A1**	1.47	1.32E-07	5.65E-07
**SLAIN1**	-1.33	1.32E-07	5.66E-07
**ALOX12**	-1.06	1.33E-07	5.70E-07
**RP11-330C7.3**	-5.58	1.33E-07	5.70E-07
**LOH12CR2**	2.24	1.33E-07	5.71E-07
**RP11-1057B6.1**	-4.42	1.33E-07	5.72E-07
**PNLDC1**	-5.86	1.34E-07	5.73E-07
**MIAT**	-1.54	1.34E-07	5.74E-07
**G1047**	2.40	1.34E-07	5.74E-07
**SLC25A11**	0.70	1.34E-07	5.74E-07
**OGDHL**	5.27	1.34E-07	5.76E-07
**DSTNP1**	-2.04	1.35E-07	5.77E-07
**G34552**	-5.67	1.35E-07	5.78E-07
**RP11-286N22.6**	-4.76	1.35E-07	5.79E-07
**G38913**	-3.87	1.35E-07	5.80E-07
**WRNIP1**	0.43	1.36E-07	5.81E-07
**RP11-495K9.5**	-3.76	1.36E-07	5.81E-07
**CENPBD1**	0.89	1.36E-07	5.82E-07
**G33445**	-0.84	1.36E-07	5.83E-07

**RNFT1P2**	-5.78	1.37E-07	5.87E-07
**NR4A1**	2.16	1.37E-07	5.88E-07
**SNORD116-19**	-5.79	1.38E-07	5.90E-07
**ZNF563**	-0.71	1.38E-07	5.90E-07
**NIP7P3**	-5.97	1.38E-07	5.91E-07
**RP11-285G1.9**	-5.78	1.39E-07	5.93E-07
**SEC14L6**	-4.35	1.39E-07	5.95E-07
**BACE2**	1.20	1.39E-07	5.96E-07
**CTC-339F2.2**	-2.07	1.39E-07	5.96E-07
**RP11-45L9.1**	-4.50	1.40E-07	5.97E-07
**FNIP2**	-0.75	1.40E-07	5.97E-07
**RP11-546J1.1**	-2.84	1.40E-07	5.97E-07
**RP11-474D14.2**	-3.35	1.40E-07	5.99E-07
**RP11-613D13.5**	-2.29	1.40E-07	5.99E-07
**AC079610.1**	-5.85	1.41E-07	6.04E-07
**AC009237.8**	-1.13	1.41E-07	6.04E-07
**ANPEP**	2.08	1.42E-07	6.05E-07
**FAM69B**	1.58	1.42E-07	6.07E-07
**SLC35D1**	-0.64	1.42E-07	6.08E-07
**SLC25A14P1**	-5.76	1.43E-07	6.10E-07
**HMGN2P3**	2.03	1.43E-07	6.10E-07
**UGT1A4**	-6.14	1.43E-07	6.10E-07
**RP11-385F7.1**	2.51	1.43E-07	6.10E-07
**RP11-458F8.4**	2.60	1.43E-07	6.11E-07
**CLIC4**	1.08	1.43E-07	6.12E-07
**RPS15A**	-0.85	1.44E-07	6.13E-07
**RP11-598F7.5**	6.24	1.44E-07	6.14E-07
**FAM117B**	-0.93	1.45E-07	6.18E-07
**C21orf91**	-1.14	1.45E-07	6.19E-07
**CPEB4**	-0.87	1.46E-07	6.21E-07
**CHCHD2**	0.80	1.46E-07	6.21E-07
**PAIP2**	0.76	1.47E-07	6.25E-07
**LECT1**	-5.97	1.47E-07	6.25E-07
**POM121C**	1.11	1.48E-07	6.29E-07
**CASKIN1**	1.67	1.48E-07	6.30E-07
**SERPINB13**	1.79	1.48E-07	6.30E-07
**G43621**	-11.32	1.48E-07	6.32E-07
**LINC01181**	4.09	1.48E-07	6.33E-07
**AC019097.7**	-1.81	1.49E-07	6.33E-07
**RB1CC1**	-0.67	1.49E-07	6.34E-07
**RP4-724E13.2**	-3.04	1.49E-07	6.34E-07
**RP11-539G18.1**	-6.14	1.49E-07	6.35E-07
**DRGX**	-5.19	1.49E-07	6.36E-07
**RP11-358B23.7**	-5.85	1.50E-07	6.36E-07
**SEZ6L**	-3.33	1.50E-07	6.39E-07

**TBC1D14**	-0.62	1.50E-07	6.39E-07
**ADGRG3**	4.03	1.50E-07	6.40E-07
**THAP2**	-1.00	1.51E-07	6.41E-07
**KCNH8**	-2.69	1.51E-07	6.42E-07
**FABP6**	3.59	1.51E-07	6.44E-07
**PLP2**	0.74	1.51E-07	6.44E-07
**ARHGAP27**	0.92	1.52E-07	6.44E-07
**PLK1**	1.23	1.52E-07	6.46E-07
**RP11-110I1.11**	-2.41	1.53E-07	6.52E-07
**THUMPD3-AS1**	-1.13	1.54E-07	6.54E-07
**RN7SL239P**	-5.86	1.54E-07	6.54E-07
**RP11-544A12.8**	-4.46	1.54E-07	6.54E-07
**RPL15P3**	1.14	1.54E-07	6.56E-07
**PLK3**	0.95	1.54E-07	6.56E-07
**SNORA19**	-5.72	1.55E-07	6.58E-07
**RP1-124C6.1**	-3.05	1.55E-07	6.58E-07
**FLJ46284**	-2.35	1.55E-07	6.59E-07
**SH3RF2**	-0.96	1.55E-07	6.59E-07
**FST**	2.18	1.56E-07	6.61E-07
**RNU6-1291P**	-6.38	1.56E-07	6.63E-07
**DNAH6**	-2.62	1.57E-07	6.65E-07
**RP11-21K12.2**	-1.91	1.57E-07	6.67E-07
**ARL11**	1.31	1.57E-07	6.67E-07
**TCEANC**	-0.79	1.58E-07	6.68E-07
**RP4-591N18.2**	-2.84	1.58E-07	6.69E-07
**RBBP4P2**	-4.65	1.58E-07	6.72E-07
**FAM222B**	0.94	1.59E-07	6.72E-07
**OLMALINC**	1.57	1.59E-07	6.73E-07
**AC011752.1**	-3.89	1.59E-07	6.73E-07
**RP11-368L12.1**	-2.14	1.59E-07	6.73E-07
**SNHG11**	0.98	1.59E-07	6.74E-07
**ZBTB46**	1.02	1.59E-07	6.75E-07
**PLXNB3**	1.49	1.60E-07	6.76E-07
**NUDT16**	0.77	1.60E-07	6.79E-07
**YTHDF3**	-0.58	1.61E-07	6.81E-07
**TMEM94**	0.80	1.61E-07	6.83E-07
**LDHAL6B**	-2.64	1.61E-07	6.83E-07
**PCDHA10**	-2.38	1.61E-07	6.84E-07
**ZNF830**	0.59	1.62E-07	6.85E-07
**MTMR6**	-0.85	1.62E-07	6.85E-07
**LAS1L**	0.60	1.62E-07	6.86E-07
**RP11-420A23.1**	-1.70	1.63E-07	6.88E-07
**C7orf31**	1.12	1.63E-07	6.90E-07
**PCDHB14**	0.78	1.63E-07	6.91E-07
**RP11-218D6.4**	-5.70	1.64E-07	6.93E-07

**UBE2CP1**	-5.89	1.64E-07	6.94E-07
**C17orf58**	0.97	1.64E-07	6.95E-07
**MIR3671**	-3.72	1.65E-07	6.98E-07
**CARD6**	1.74	1.65E-07	6.98E-07
**RAI1-AS1**	-5.76	1.65E-07	6.99E-07
**RNF7**	0.57	1.66E-07	7.00E-07
**DHX36**	-0.54	1.66E-07	7.00E-07
**CTD-2292M16.8**	-1.62	1.66E-07	7.00E-07
**CTD-2265D6.2**	-6.12	1.67E-07	7.07E-07
**ASRGL1**	3.32	1.67E-07	7.07E-07
**DDX23**	0.51	1.67E-07	7.07E-07
**SAPCD2**	1.19	1.68E-07	7.08E-07
**RP11-453N18.1**	-6.04	1.68E-07	7.08E-07
**FTO-IT1**	-2.64	1.68E-07	7.09E-07
**MSLN**	3.81	1.69E-07	7.12E-07
**KRT8**	3.84	1.69E-07	7.12E-07
**AC127904.2**	-2.03	1.69E-07	7.13E-07
**RP11-143I21.1**	-5.64	1.69E-07	7.13E-07
**RP11-467H10.2**	-5.69	1.69E-07	7.15E-07
**Z95704.1**	-5.78	1.70E-07	7.17E-07
**SNRNP70**	1.46	1.70E-07	7.19E-07
**RP11-709D24.6**	-5.74	1.71E-07	7.20E-07
**LUM**	2.76	1.71E-07	7.20E-07
**C5orf34**	-1.21	1.71E-07	7.20E-07
**RBM23**	0.38	1.71E-07	7.20E-07
**G35863**	-5.43	1.72E-07	7.23E-07
**RRP7A**	0.88	1.72E-07	7.24E-07
**RP11-402L5.1**	-5.84	1.72E-07	7.24E-07
**DOC2B**	1.88	1.73E-07	7.28E-07
**RP11-336A10.2**	-3.02	1.73E-07	7.30E-07
**CTC-448F2.6**	-1.88	1.74E-07	7.32E-07
**SMIM20**	1.04	1.74E-07	7.32E-07
**Z97634.3**	2.72	1.74E-07	7.33E-07
**FLVCR1-AS1**	2.50	1.74E-07	7.34E-07
**MED28P3**	-2.60	1.76E-07	7.40E-07
**DKK2**	-2.25	1.77E-07	7.46E-07
**GRWD1**	0.70	1.78E-07	7.49E-07
**LRRC25**	3.00	1.78E-07	7.49E-07
**RP13-36G14.4**	-1.34	1.79E-07	7.53E-07
**CD207**	-1.89	1.79E-07	7.55E-07
**SLC30A6**	-0.58	1.80E-07	7.56E-07
**PFDN5**	0.70	1.80E-07	7.56E-07
**POMGNT1**	0.73	1.80E-07	7.57E-07
**CCNJP2**	-6.14	1.80E-07	7.58E-07
**KIAA2026**	-0.51	1.80E-07	7.58E-07

**RAVER1**	1.24	1.82E-07	7.64E-07
**WASH2P**	0.96	1.82E-07	7.65E-07
**ADRM1**	0.65	1.82E-07	7.66E-07
**RP11-575A19.2**	-3.15	1.84E-07	7.73E-07
**CSNK1A1P1**	-5.73	1.84E-07	7.73E-07
**CH17-431G21.1**	2.79	1.84E-07	7.73E-07
**TAS2R5**	-2.11	1.85E-07	7.78E-07
**PLCB2**	2.18	1.85E-07	7.79E-07
**RP11-190D6.1**	-5.91	1.86E-07	7.81E-07
**RP11-142E9.1**	-1.47	1.87E-07	7.86E-07
**RNU4ATAC16P**	-5.83	1.87E-07	7.87E-07
**WDR43**	-0.77	1.88E-07	7.87E-07
**DFFA**	0.53	1.88E-07	7.89E-07
**ZNF585B**	-0.79	1.88E-07	7.89E-07
**RP11-154B12.3**	-5.65	1.89E-07	7.91E-07
**MRPS6**	0.96	1.89E-07	7.94E-07
**KIF13A**	-0.50	1.89E-07	7.95E-07
**GTF3C6**	0.87	1.90E-07	7.97E-07
**ZER1**	0.64	1.90E-07	7.97E-07
**ERBB3**	-0.73	1.90E-07	7.98E-07
**FFAR4**	-1.51	1.90E-07	7.99E-07
**DHRS4L2**	1.06	1.91E-07	7.99E-07
**MRPL11**	0.75	1.91E-07	7.99E-07
**RP11-96D1.10**	-5.45	1.91E-07	8.00E-07
**MSC-AS1**	2.09	1.91E-07	8.01E-07
**RP11-242O24.3**	-5.80	1.91E-07	8.01E-07
**RP11-35O15.2**	-5.75	1.91E-07	8.01E-07
**ZNF664**	0.75	1.92E-07	8.02E-07
**RP11-159D12.8**	-0.87	1.92E-07	8.03E-07
**SIAE**	0.74	1.92E-07	8.04E-07
**AP000350.6**	-7.00	1.92E-07	8.05E-07
**ZNRF2P1**	1.93	1.93E-07	8.07E-07
**ATP5G2**	0.75	1.95E-07	8.17E-07
**NELL2**	3.20	1.95E-07	8.18E-07
**RP11-295H24.4**	-1.57	1.96E-07	8.19E-07
**ATP1A1-AS1**	-1.31	1.96E-07	8.19E-07
**HNRNPA3P9**	-4.59	1.96E-07	8.19E-07
**PDIA3**	0.78	1.96E-07	8.19E-07
**ATG10-AS1**	-5.84	1.96E-07	8.19E-07
**RP11-584P21.4**	-5.73	1.96E-07	8.21E-07
**RASSF4**	1.86	1.96E-07	8.21E-07
**LINC00667**	-0.88	1.97E-07	8.22E-07
**C1QTNF3**	-1.85	1.97E-07	8.23E-07
**HS3ST1**	1.82	1.97E-07	8.26E-07
**AF064858.6**	2.57	1.98E-07	8.26E-07

**TRIM46**	2.68	1.98E-07	8.27E-07
**IL17RB**	-1.11	1.99E-07	8.30E-07
**RP11-864I4.1**	1.81	1.99E-07	8.31E-07
**RP11-815J21.1**	-3.49	2.00E-07	8.37E-07
**IFNAR2**	1.47	2.01E-07	8.38E-07
**MT-TS2**	-6.73	2.01E-07	8.39E-07
**RP11-474I11.8**	-5.82	2.02E-07	8.43E-07
**USP32**	-0.76	2.04E-07	8.51E-07
**U47924.29**	-5.66	2.04E-07	8.51E-07
**RP11-317F20.2**	-5.89	2.04E-07	8.53E-07
**FTSJ1**	0.59	2.05E-07	8.55E-07
**RP4-798P15.3**	-3.18	2.05E-07	8.56E-07
**RP11-170L3.4**	-5.73	2.06E-07	8.59E-07
**BPNT1**	0.57	2.07E-07	8.64E-07
**RP11-646I6.5**	-2.27	2.07E-07	8.66E-07
**RP3-425C14.6**	-5.37	2.09E-07	8.72E-07
**CTC-518B2.8**	2.26	2.09E-07	8.73E-07
**ADAM20**	-3.89	2.11E-07	8.79E-07
**ZNF713**	-1.10	2.12E-07	8.83E-07
**G34302**	-5.93	2.12E-07	8.85E-07
**U47924.27**	1.27	2.13E-07	8.87E-07
**RP11-416N2.4**	-2.45	2.13E-07	8.87E-07
**TTC30B**	-0.78	2.14E-07	8.90E-07
**ZCCHC7**	-0.47	2.14E-07	8.90E-07
**ABHD13**	-0.74	2.14E-07	8.91E-07
**KIAA0040**	0.92	2.15E-07	8.94E-07
**CTD-3157E16.2**	1.90	2.15E-07	8.94E-07
**MSMB**	-3.21	2.15E-07	8.95E-07
**BZRAP1-AS1**	-2.06	2.16E-07	8.98E-07
**FAR2**	-3.43	2.16E-07	8.98E-07
**RNU2-25P**	-5.75	2.16E-07	8.98E-07
**C9orf91**	1.04	2.16E-07	9.00E-07
**UROS**	0.70	2.17E-07	9.01E-07
**PRDX1**	1.00	2.17E-07	9.02E-07
**RP11-351O1.4**	-5.83	2.17E-07	9.02E-07
**G12186**	-5.87	2.18E-07	9.07E-07
**DLEC1**	-2.18	2.18E-07	9.08E-07
**MON1A**	1.01	2.18E-07	9.09E-07
**KB-1254G8.1**	-4.73	2.19E-07	9.09E-07
**EPHX4**	3.73	2.19E-07	9.10E-07
**LAMB2**	1.09	2.19E-07	9.11E-07
**RP4-564F22.5**	-5.70	2.19E-07	9.11E-07
**MMP2**	2.81	2.20E-07	9.14E-07
**LIPT2**	-1.48	2.20E-07	9.14E-07
**RP11-611O2.1**	-5.79	2.21E-07	9.18E-07

**PHF12**	0.84	2.21E-07	9.20E-07
**RP11-145M9.5**	-4.74	2.22E-07	9.21E-07
**DSP**	-1.02	2.22E-07	9.22E-07
**RP11-841C19.1**	-5.94	2.22E-07	9.22E-07
**TTC17**	-0.50	2.22E-07	9.22E-07
**PAPD5**	-0.61	2.22E-07	9.23E-07
**OSER1**	0.66	2.22E-07	9.23E-07
**ATAD1**	-0.63	2.24E-07	9.28E-07
**G23966**	3.68	2.24E-07	9.29E-07
**RP11-372M18.2**	-3.14	2.24E-07	9.30E-07
**TCTA**	0.69	2.25E-07	9.32E-07
**HEG1**	1.40	2.25E-07	9.33E-07
**LINC00961**	3.52	2.25E-07	9.33E-07
**ALS2CR11**	-2.06	2.26E-07	9.39E-07
**TRMT61A**	0.74	2.26E-07	9.39E-07
**LRRC75A-AS1**	0.69	2.26E-07	9.39E-07
**MIR553**	-5.54	2.27E-07	9.39E-07
**TGIF2**	0.84	2.27E-07	9.40E-07
**TRPM1**	-1.91	2.27E-07	9.41E-07
**ACVR2B**	-0.80	2.27E-07	9.42E-07
**KCMF1**	0.50	2.28E-07	9.45E-07
**ARHGAP25**	1.39	2.28E-07	9.46E-07
**HELB**	-0.99	2.30E-07	9.54E-07
**ZW10**	-0.73	2.30E-07	9.54E-07
**GRID1**	1.87	2.30E-07	9.54E-07
**RP11-147G7.3**	-5.36	2.30E-07	9.55E-07
**SBF2-AS1**	-0.89	2.31E-07	9.57E-07
**LEMD3**	-0.57	2.32E-07	9.59E-07
**AC005042.2**	-4.87	2.33E-07	9.63E-07
**NCCRP1**	1.21	2.33E-07	9.66E-07
**STIL**	-0.71	2.33E-07	9.66E-07
**PKD2L2**	-3.03	2.33E-07	9.66E-07
**RP11-350F16.1**	-5.72	2.33E-07	9.66E-07
**ZNF22**	1.05	2.34E-07	9.66E-07
**AC023271.1**	-5.58	2.34E-07	9.68E-07
**OFD1P17**	-5.72	2.34E-07	9.70E-07
**RP11-550F7.1**	-6.19	2.35E-07	9.70E-07
**IPO8**	-0.49	2.35E-07	9.72E-07
**PLEKHA8**	-0.80	2.35E-07	9.72E-07
**TMEM63B**	0.67	2.35E-07	9.73E-07
**MAFK**	0.82	2.35E-07	9.73E-07
**SH3BP1**	0.96	2.36E-07	9.73E-07
**RP4-657D16.3**	-2.07	2.36E-07	9.74E-07
**AGR2**	-2.61	2.36E-07	9.77E-07
**RP11-712B9.6**	-5.83	2.37E-07	9.78E-07

**LRRC4**	1.85	2.37E-07	9.80E-07
**CFDP1**	0.53	2.37E-07	9.80E-07
**LZTS1**	1.30	2.39E-07	9.85E-07
**SH2D2A**	2.73	2.39E-07	9.88E-07
**TIPARP**	-1.06	2.40E-07	9.89E-07
**RP11-114M5.1**	-4.18	2.40E-07	9.91E-07
**RCSD1**	1.76	2.40E-07	9.92E-07
**RP11-22H5.2**	-3.99	2.41E-07	9.93E-07
**ZNF517**	0.92	2.41E-07	9.95E-07
**GAREML**	1.50	2.41E-07	9.96E-07
**TMEM150C**	1.50	2.42E-07	9.97E-07
**EMC7**	0.72	2.42E-07	9.98E-07
**SMYD2**	0.56	2.42E-07	9.99E-07
**ARSJ**	1.76	2.42E-07	9.99E-07
**MAFG-AS1**	1.42	2.43E-07	1.00E-06
**AC097382.5**	-5.59	2.43E-07	1.00E-06
**RP11-115J16.1**	-5.99	2.43E-07	1.00E-06
**MTF2**	-0.52	2.44E-07	1.01E-06
**ATPIF1**	0.77	2.44E-07	1.01E-06
**TAZ**	1.17	2.46E-07	1.01E-06
**CTB-75G16.1**	-3.20	2.46E-07	1.01E-06
**RP11-467H10.1**	-2.28	2.46E-07	1.01E-06
**RP11-848P1.9**	-1.12	2.47E-07	1.02E-06
**RP11-323I15.5**	-4.32	2.48E-07	1.02E-06
**RP11-640M9.1**	1.97	2.48E-07	1.02E-06
**C14orf79**	0.99	2.49E-07	1.03E-06
**CD3E**	1.91	2.49E-07	1.03E-06
**ZC2HC1A**	-1.15	2.51E-07	1.03E-06
**SARDH**	3.05	2.52E-07	1.04E-06
**XLOC_000633**	-2.94	2.52E-07	1.04E-06
**CD163**	3.07	2.53E-07	1.04E-06
**TBCD**	0.53	2.53E-07	1.04E-06
**PAXBP1**	-1.12	2.53E-07	1.04E-06
**NMNAT3**	-1.13	2.54E-07	1.04E-06
**MTND1P21**	-5.82	2.55E-07	1.05E-06
**NFU1**	0.94	2.56E-07	1.05E-06
**RP5-968J1.1**	-2.67	2.56E-07	1.05E-06
**C10orf2**	0.83	2.56E-07	1.05E-06
**FRG2DP**	-3.82	2.56E-07	1.05E-06
**ATP5G3**	0.80	2.56E-07	1.05E-06
**RP11-283I3.6**	-0.94	2.57E-07	1.05E-06
**RP11-357N13.2**	-3.23	2.57E-07	1.06E-06
**RAD1**	-0.52	2.57E-07	1.06E-06
**KRT18P63**	-4.13	2.58E-07	1.06E-06
**LINC00681**	-3.08	2.58E-07	1.06E-06

**FCF1P5**	-5.72	2.58E-07	1.06E-06
**MSR1**	3.10	2.58E-07	1.06E-06
**SLC38A2**	-0.78	2.59E-07	1.06E-06
**POLR1B**	-0.80	2.59E-07	1.06E-06
**C16orf86**	2.03	2.59E-07	1.06E-06
**GK5**	-1.72	2.60E-07	1.07E-06
**AC009299.3**	-1.36	2.60E-07	1.07E-06
**RPS5**	0.83	2.62E-07	1.07E-06
**RP1-86D1.5**	-5.67	2.62E-07	1.08E-06
**GJC2**	1.56	2.63E-07	1.08E-06
**MBP**	-0.68	2.63E-07	1.08E-06
**AP001468.1**	-3.32	2.64E-07	1.08E-06
**SOCS2**	1.58	2.66E-07	1.09E-06
**CTD-2218G20.2**	-6.49	2.68E-07	1.10E-06
**RP11-426D19.1**	-4.38	2.68E-07	1.10E-06
**SSR4P1**	1.19	2.68E-07	1.10E-06
**G7242**	-1.49	2.70E-07	1.11E-06
**MAU2**	0.55	2.70E-07	1.11E-06
**EAF1-AS1**	-3.65	2.70E-07	1.11E-06
**VTRNA1-3**	-5.59	2.70E-07	1.11E-06
**COL16A1**	1.25	2.70E-07	1.11E-06
**EML5**	-1.63	2.71E-07	1.11E-06
**LINC00843**	-1.59	2.72E-07	1.11E-06
**LIMS1**	0.79	2.72E-07	1.12E-06
**EIF4EP1**	-5.65	2.73E-07	1.12E-06
**PSMB3**	0.80	2.73E-07	1.12E-06
**FBXO17**	0.92	2.73E-07	1.12E-06
**G33836**	-6.58	2.75E-07	1.12E-06
**EIF6**	0.84	2.75E-07	1.13E-06
**CDHR1**	-1.63	2.75E-07	1.13E-06
**ZC3H12B**	-1.46	2.76E-07	1.13E-06
**RP11-392O18.2**	-5.64	2.77E-07	1.13E-06
**CFAP43**	-1.74	2.77E-07	1.13E-06
**G24466**	-5.69	2.78E-07	1.14E-06
**NTAN1P2**	-2.29	2.79E-07	1.14E-06
**ADGRE5**	1.36	2.79E-07	1.14E-06
**MAGI1-AS1**	-5.88	2.79E-07	1.14E-06
**ZNFX1**	-0.55	2.80E-07	1.14E-06
**RP11-364L4.1**	-4.26	2.80E-07	1.15E-06
**RP11-71H17.1**	-5.67	2.80E-07	1.15E-06
**MMP7**	3.67	2.82E-07	1.15E-06
**MIR3128**	-5.71	2.85E-07	1.16E-06
**CTD-2012K14.6**	-2.62	2.85E-07	1.16E-06
**ST3GAL2**	1.24	2.87E-07	1.17E-06
**AC008592.7**	-5.64	2.88E-07	1.17E-06

**CCAR2**	0.52	2.88E-07	1.18E-06
**XRCC2**	-1.02	2.90E-07	1.18E-06
**SDK2**	1.54	2.90E-07	1.18E-06
**RP11-739L10.1**	-3.28	2.90E-07	1.18E-06
**CYTH1**	0.42	2.93E-07	1.19E-06
**OTUD4P1**	-5.86	2.93E-07	1.19E-06
**RP11-115H15.2**	-3.59	2.93E-07	1.19E-06
**WNK1**	-0.81	2.93E-07	1.20E-06
**GPRIN2**	-1.22	2.94E-07	1.20E-06
**RP1-91J24.3**	-5.61	2.94E-07	1.20E-06
**CETP**	2.02	2.95E-07	1.20E-06
**CHORDC1**	-0.96	2.96E-07	1.21E-06
**NR1I3**	-1.21	2.96E-07	1.21E-06
**LY6G5C**	2.52	2.96E-07	1.21E-06
**LBX2-AS1**	2.53	2.96E-07	1.21E-06
**WDHD1**	-0.79	2.97E-07	1.21E-06
**KDM3B**	-0.43	2.97E-07	1.21E-06
**HRCT1**	3.70	2.97E-07	1.21E-06
**MRGPRX2**	-5.75	2.98E-07	1.21E-06
**RP1-27K12.4**	-3.41	2.98E-07	1.21E-06
**RP11-507K2.6**	-3.31	2.99E-07	1.22E-06
**SNX18P7**	-2.07	2.99E-07	1.22E-06
**HMGA1**	0.86	2.99E-07	1.22E-06
**RHCG**	3.65	2.99E-07	1.22E-06
**NAGPA**	0.73	3.00E-07	1.22E-06
**G6PD**	1.13	3.01E-07	1.22E-06
**PTGES3P1**	1.05	3.01E-07	1.23E-06
**RP11-295B17.6**	-5.67	3.01E-07	1.23E-06
**LDB2**	1.87	3.02E-07	1.23E-06
**G19426**	-4.77	3.02E-07	1.23E-06
**RP11-160E2.6**	-2.38	3.03E-07	1.23E-06
**NANOGP5**	-5.66	3.05E-07	1.24E-06
**RP11-83B20.1**	-4.10	3.05E-07	1.24E-06
**VPS37A**	-0.52	3.08E-07	1.25E-06
**RN7SKP271**	-5.77	3.08E-07	1.25E-06
**HAUS1**	0.80	3.09E-07	1.26E-06
**RP11-380M21.1**	-3.80	3.09E-07	1.26E-06
**GON4L**	-0.40	3.11E-07	1.26E-06
**SNX14**	-0.60	3.11E-07	1.26E-06
**SLC25A38**	0.72	3.11E-07	1.26E-06
**AC093388.3**	-2.34	3.12E-07	1.27E-06
**RLIMP1**	-2.87	3.12E-07	1.27E-06
**FAM101B**	1.66	3.14E-07	1.27E-06
**SMCR5**	-4.61	3.14E-07	1.28E-06
**ALX1**	-1.60	3.14E-07	1.28E-06

**H3F3AP4**	1.80	3.15E-07	1.28E-06
**ZNF564**	-2.26	3.15E-07	1.28E-06
**FOCAD**	-0.74	3.15E-07	1.28E-06
**TRPC5**	-5.66	3.16E-07	1.28E-06
**NACC2**	0.76	3.17E-07	1.29E-06
**CNNM3**	0.75	3.18E-07	1.29E-06
**RPL13**	0.78	3.18E-07	1.29E-06
**SOWAHD**	2.65	3.19E-07	1.29E-06
**CBX5P1**	-5.45	3.19E-07	1.29E-06
**CTD-2583A14.8**	1.73	3.20E-07	1.30E-06
**GPT2**	0.89	3.21E-07	1.30E-06
**BCAP29**	-0.95	3.22E-07	1.31E-06
**PCYT2**	0.89	3.23E-07	1.31E-06
**GTF2IRD2B**	0.95	3.24E-07	1.31E-06
**RP11-643A5.3**	-2.75	3.25E-07	1.32E-06
**DCDC1**	-4.48	3.25E-07	1.32E-06
**IZUMO4**	1.41	3.25E-07	1.32E-06
**CCL22**	-1.90	3.26E-07	1.32E-06
**EIF1B**	0.76	3.27E-07	1.32E-06
**AC017048.1**	-5.93	3.28E-07	1.33E-06
**RBPMSLP**	-5.60	3.30E-07	1.34E-06
**RP11-525G13.2**	-2.52	3.31E-07	1.34E-06
**XLOC_007276**	-5.70	3.32E-07	1.34E-06
**ZNF572**	-1.18	3.33E-07	1.35E-06
**LOXL3**	2.00	3.33E-07	1.35E-06
**RP11-290F24.6**	-2.05	3.33E-07	1.35E-06
**SNORA30**	-5.59	3.34E-07	1.35E-06
**RP11-20B24.6**	-5.65	3.35E-07	1.35E-06
**TSC1**	-0.60	3.35E-07	1.36E-06
**KIF28P**	-3.83	3.35E-07	1.36E-06
**MSX1**	1.92	3.36E-07	1.36E-06
**RP11-280O24.3**	-5.73	3.36E-07	1.36E-06
**DOCK9**	-0.54	3.37E-07	1.36E-06
**ZCRB1**	0.82	3.37E-07	1.36E-06
**AC104058.1**	-5.89	3.38E-07	1.36E-06
**SHROOM3**	-1.35	3.38E-07	1.37E-06
**HGH1**	1.61	3.38E-07	1.37E-06
**PPIL6**	-1.29	3.38E-07	1.37E-06
**SPEN**	-0.75	3.38E-07	1.37E-06
**RP13-210D15.8**	-4.04	3.38E-07	1.37E-06
**C8orf33**	0.62	3.39E-07	1.37E-06
**RP11-801G16.2**	-5.85	3.39E-07	1.37E-06
**KIF1BP**	0.47	3.39E-07	1.37E-06
**ZFHX4**	1.53	3.40E-07	1.37E-06
**VASN**	1.48	3.40E-07	1.37E-06

**CLIC3**	1.15	3.41E-07	1.38E-06
**XLOC_001097**	-3.68	3.42E-07	1.38E-06
**LINC00969**	-1.72	3.42E-07	1.38E-06
**RP6-24A23.3**	-5.62	3.43E-07	1.38E-06
**TSPAN10**	1.75	3.43E-07	1.38E-06
**TRIM24**	-0.65	3.43E-07	1.38E-06
**G39123**	-7.63	3.43E-07	1.38E-06
**RP11-148B6.1**	-5.68	3.45E-07	1.39E-06
**BSDC1**	0.42	3.46E-07	1.39E-06
**WDR38**	-3.94	3.47E-07	1.40E-06
**GIGYF1**	0.97	3.47E-07	1.40E-06
**SH3BP5-AS1**	-1.44	3.47E-07	1.40E-06
**RGL4**	-1.87	3.47E-07	1.40E-06
**BAP1**	0.44	3.49E-07	1.41E-06
**RP11-192H23.4**	1.43	3.49E-07	1.41E-06
**KRT12**	-2.84	3.50E-07	1.41E-06
**SNX16**	-1.01	3.51E-07	1.41E-06
**CTD-2357A8.2**	-3.92	3.52E-07	1.42E-06
**CTD-2542C24.5**	-4.02	3.53E-07	1.42E-06
**SEPT7P9**	-2.71	3.53E-07	1.42E-06
**AC010877.1**	-5.92	3.56E-07	1.43E-06
**ZSCAN2**	1.24	3.57E-07	1.44E-06
**EGLN3P1**	-4.19	3.58E-07	1.44E-06
**CTSO**	1.20	3.59E-07	1.44E-06
**LHX2**	-3.71	3.61E-07	1.45E-06
**RP11-110I1.5**	-3.14	3.62E-07	1.46E-06
**CTD-2083E4.5**	-2.90	3.62E-07	1.46E-06
**RNF32**	-1.44	3.64E-07	1.46E-06
**LINC01619**	-1.67	3.64E-07	1.46E-06
**CAPG**	0.78	3.64E-07	1.46E-06
**CTD-3222D19.8**	-1.99	3.64E-07	1.46E-06
**CORO1A**	1.28	3.64E-07	1.46E-06
**MAL**	1.76	3.65E-07	1.47E-06
**CDCA5**	1.02	3.65E-07	1.47E-06
**AC008440.10**	-3.51	3.65E-07	1.47E-06
**ALOX12P2**	-2.09	3.68E-07	1.48E-06
**LINC01482**	4.04	3.69E-07	1.48E-06
**DCUN1D2-AS**	-5.50	3.71E-07	1.49E-06
**HNRNPMP1**	-5.67	3.72E-07	1.49E-06
**SERTAD4-AS1**	1.90	3.74E-07	1.50E-06
**RP11-534L20.4**	-5.68	3.75E-07	1.50E-06
**RAB15**	1.06	3.75E-07	1.50E-06
**VSIG2**	1.65	3.79E-07	1.52E-06
**ANXA5**	1.09	3.82E-07	1.53E-06
**SLC22A18**	1.05	3.82E-07	1.53E-06

**CECR2**	-2.29	3.83E-07	1.54E-06
**AC092484.1**	-5.60	3.84E-07	1.54E-06
**CLDN15**	1.21	3.84E-07	1.54E-06
**RNU6-780P**	-5.77	3.87E-07	1.55E-06
**HMGN1**	0.59	3.87E-07	1.55E-06
**KCNJ6**	4.94	3.89E-07	1.56E-06
**VWCE**	2.31	3.89E-07	1.56E-06
**CTD-2302E22.5**	-3.41	3.89E-07	1.56E-06
**VN1R1**	-1.66	3.90E-07	1.56E-06
**ADCY5**	1.50	3.91E-07	1.57E-06
**RP5-856G1.2**	-3.96	3.92E-07	1.57E-06
**G30143**	-4.59	3.92E-07	1.57E-06
**RP3-476K8.3**	-3.56	3.92E-07	1.57E-06
**RP11-322D14.1**	-5.75	3.93E-07	1.57E-06
**GUCD1**	0.47	3.95E-07	1.58E-06
**RP11-113K21.6**	-2.62	3.95E-07	1.58E-06
**LCP1**	-1.29	3.95E-07	1.58E-06
**GDF15**	3.59	3.96E-07	1.58E-06
**CTC-421K24.1**	-5.56	3.97E-07	1.59E-06
**HSFY3P**	-5.80	3.97E-07	1.59E-06
**AUP1**	0.60	3.97E-07	1.59E-06
**RP11-98I9.4**	-1.08	3.97E-07	1.59E-06
**ZACN**	-2.73	3.97E-07	1.59E-06
**ZNF208**	-2.92	4.00E-07	1.60E-06
**RP11-171G2.1**	-5.64	4.00E-07	1.60E-06
**C5orf51**	-0.58	4.01E-07	1.60E-06
**INTS1**	0.79	4.01E-07	1.60E-06
**FARS2**	0.51	4.02E-07	1.61E-06
**EEF1B2P7**	-5.69	4.03E-07	1.61E-06
**KCND3-IT1**	-5.72	4.04E-07	1.62E-06
**STIP1**	0.74	4.05E-07	1.62E-06
**XLOC_005574**	-2.32	4.06E-07	1.62E-06
**G11672**	-2.58	4.06E-07	1.62E-06
**FXYD3**	0.84	4.06E-07	1.62E-06
**LPAR6**	0.80	4.06E-07	1.62E-06
**G25499**	-2.64	4.08E-07	1.63E-06
**SLC25A40**	-0.69	4.09E-07	1.63E-06
**FKBP11**	1.32	4.09E-07	1.63E-06
**FNTAP2**	-5.78	4.09E-07	1.63E-06
**KLHDC7B**	3.51	4.11E-07	1.64E-06
**RP11-274A11.3**	-4.20	4.11E-07	1.64E-06
**KLHL21**	0.75	4.11E-07	1.64E-06
**OTUD1**	-0.96	4.13E-07	1.65E-06
**CETN2**	0.91	4.13E-07	1.65E-06
**RP11-111M22.3**	1.05	4.14E-07	1.65E-06

**SERPINE3**	-2.56	4.14E-07	1.65E-06
**RBMX2**	0.76	4.15E-07	1.66E-06
**OSBPL6**	-1.23	4.16E-07	1.66E-06
**RP11-319G9.1**	-5.90	4.17E-07	1.66E-06
**LARP6**	1.71	4.17E-07	1.66E-06
**OSBPL5**	0.77	4.17E-07	1.66E-06
**MRPL36**	0.87	4.18E-07	1.66E-06
**CHI3L2**	2.72	4.19E-07	1.67E-06
**LINC00893**	-1.96	4.20E-07	1.68E-06
**DLX6**	1.76	4.21E-07	1.68E-06
**PHF21A**	0.74	4.22E-07	1.68E-06
**RPL3P1**	-5.54	4.23E-07	1.68E-06
**RP11-383B4.4**	-3.85	4.23E-07	1.69E-06
**TCF15**	5.27	4.23E-07	1.69E-06
**THEM4**	0.97	4.23E-07	1.69E-06
**CAMTA2**	1.10	4.24E-07	1.69E-06
**GLDN**	-1.52	4.25E-07	1.69E-06
**CD4**	1.63	4.25E-07	1.69E-06
**PPCDC**	0.77	4.26E-07	1.70E-06
**DOK5**	2.92	4.27E-07	1.70E-06
**RP11-484D2.3**	-3.21	4.28E-07	1.70E-06
**AP004289.2**	-5.90	4.29E-07	1.71E-06
**NFIA-AS1**	-4.20	4.29E-07	1.71E-06
**ADGRG2**	-1.71	4.30E-07	1.71E-06
**LRRC41**	0.46	4.32E-07	1.72E-06
**RANBP6**	-0.74	4.33E-07	1.72E-06
**NUDT12**	-0.99	4.34E-07	1.72E-06
**SPNS2**	1.00	4.36E-07	1.73E-06
**TMEM140**	0.88	4.36E-07	1.73E-06
**RP11-484D2.2**	-2.52	4.38E-07	1.74E-06
**WDR73**	-0.97	4.41E-07	1.75E-06
**ABHD17C**	0.71	4.41E-07	1.75E-06
**DCUN1D4**	-0.58	4.41E-07	1.75E-06
**TTC14**	-1.05	4.41E-07	1.75E-06
**PFN1P2**	-2.34	4.42E-07	1.76E-06
**TPRG1-AS2**	-5.78	4.44E-07	1.76E-06
**PDZK1**	-3.78	4.44E-07	1.76E-06
**MSL1**	0.51	4.45E-07	1.77E-06
**ZDHHC14**	1.10	4.47E-07	1.77E-06
**G19170**	-2.76	4.48E-07	1.78E-06
**CTA-29F11.1**	1.67	4.48E-07	1.78E-06
**FAF1**	-0.49	4.48E-07	1.78E-06
**RP11-690I21.2**	-3.75	4.49E-07	1.78E-06
**ZNF528**	-1.00	4.50E-07	1.79E-06
**1-Mar**	-1.56	4.50E-07	1.79E-06

**G38458**	-3.44	4.52E-07	1.79E-06
**RP11-535A19.1**	-1.42	4.53E-07	1.80E-06
**G12735**	-2.06	4.54E-07	1.80E-06
**MT-TC**	-3.98	4.54E-07	1.80E-06
**LY96**	2.16	4.54E-07	1.80E-06
**ELMOD2**	-0.82	4.59E-07	1.82E-06
**KCNV2**	-3.96	4.59E-07	1.82E-06
**LY6H**	8.48	4.60E-07	1.82E-06
**MT-CO3**	0.93	4.62E-07	1.83E-06
**FEZ2**	0.61	4.64E-07	1.84E-06
**XLOC_011152**	-1.79	4.67E-07	1.85E-06
**HTR1F**	2.70	4.69E-07	1.86E-06
**PAXBP1-AS1**	-1.41	4.69E-07	1.86E-06
**GTF2H5**	0.76	4.71E-07	1.86E-06
**TMEM17**	0.73	4.71E-07	1.87E-06
**NCOA1**	-0.45	4.73E-07	1.88E-06
**ATE1**	-0.57	4.74E-07	1.88E-06
**AL133243.2**	-1.81	4.75E-07	1.88E-06
**AL161450.1**	-5.74	4.76E-07	1.88E-06
**MRTO4**	0.81	4.77E-07	1.89E-06
**C10orf71**	-4.49	4.78E-07	1.89E-06
**SCARNA18**	-5.32	4.78E-07	1.89E-06
**HLF**	-1.38	4.81E-07	1.90E-06
**TSEN2**	-0.71	4.82E-07	1.91E-06
**CTA-280A3.2**	-3.03	4.82E-07	1.91E-06
**AF131215.3**	-3.38	4.84E-07	1.91E-06
**RP11-113C12.4**	-4.59	4.86E-07	1.92E-06
**CTA-414D7.1**	-2.75	4.86E-07	1.92E-06
**RHOXF1-AS1**	-2.82	4.87E-07	1.93E-06
**FLNA**	0.73	4.89E-07	1.93E-06
**RP4-675C20.4**	-3.45	4.89E-07	1.93E-06
**RANBP1**	0.68	4.91E-07	1.94E-06
**EIF4BP5**	-5.77	4.91E-07	1.94E-06
**LDHAP2**	-5.61	4.91E-07	1.94E-06
**PUM2**	-0.65	4.92E-07	1.94E-06
**TAS2R63P**	-5.54	4.93E-07	1.95E-06
**SF3A3P1**	-5.62	4.98E-07	1.97E-06
**AC009961.3**	-1.14	4.99E-07	1.97E-06
**KIAA1658**	-5.70	4.99E-07	1.97E-06
**BMS1P7**	-3.70	5.00E-07	1.97E-06
**CTA-250D13.1**	-5.89	5.00E-07	1.97E-06
**RNU6-3P**	-5.39	5.01E-07	1.98E-06
**RP11-96C23.9**	-3.29	5.02E-07	1.98E-06
**PALB2**	-0.60	5.03E-07	1.98E-06
**RP11-258C19.4**	-2.38	5.03E-07	1.98E-06

**G26248**	-5.44	5.04E-07	1.99E-06
**SNORD117**	-5.11	5.05E-07	1.99E-06
**C1orf147**	-2.15	5.07E-07	2.00E-06
**LAP3**	0.79	5.07E-07	2.00E-06
**PLEKHS1**	3.90	5.08E-07	2.00E-06
**RP11-785H5.2**	-5.69	5.08E-07	2.00E-06
**RP11-33B1.3**	-4.56	5.08E-07	2.00E-06
**RP11-342K6.2**	-2.95	5.09E-07	2.01E-06
**PRIMPOL**	-0.61	5.10E-07	2.01E-06
**COL11A1**	4.70	5.10E-07	2.01E-06
**RP11-45M22.5**	-5.94	5.10E-07	2.01E-06
**UPB1**	-4.95	5.10E-07	2.01E-06
**AC007283.4**	-5.53	5.11E-07	2.01E-06
**LINC01013**	3.23	5.12E-07	2.02E-06
**CTD-2373N4.5**	-5.49	5.13E-07	2.02E-06
**RP11-195B17.1**	-2.92	5.14E-07	2.02E-06
**RP11-201O14.1**	-5.48	5.15E-07	2.03E-06
**SUGT1P2**	-3.62	5.16E-07	2.03E-06
**LOC100421166**	-5.59	5.16E-07	2.03E-06
**TARBP1**	-0.84	5.18E-07	2.04E-06
**WDPCP**	-1.18	5.19E-07	2.04E-06
**ZRANB3**	-0.72	5.19E-07	2.04E-06
**RP11-123C21.3**	-5.50	5.19E-07	2.04E-06
**TFR2**	1.19	5.20E-07	2.05E-06
**RP11-264L1.4**	-3.86	5.24E-07	2.06E-06
**BTBD3**	-0.78	5.24E-07	2.06E-06
**AC124997.1**	-5.77	5.26E-07	2.07E-06
**PRDX2P3**	-3.16	5.27E-07	2.07E-06
**RP11-541N10.3**	1.07	5.28E-07	2.08E-06
**C2CD5**	-0.61	5.30E-07	2.08E-06
**LRRC57**	-0.54	5.31E-07	2.09E-06
**RBX1**	0.69	5.35E-07	2.10E-06
**RP11-391L3.3**	-2.91	5.38E-07	2.11E-06
**RP11-325E14.5**	-5.69	5.39E-07	2.12E-06
**LINC00963**	0.91	5.39E-07	2.12E-06
**RP11-123N4.4**	-5.75	5.40E-07	2.12E-06
**DNM2**	0.59	5.40E-07	2.12E-06
**ZNF575**	1.49	5.40E-07	2.12E-06
**RP11-142L16.2**	-5.54	5.41E-07	2.12E-06
**EPHA3**	1.66	5.42E-07	2.13E-06
**PHRF1**	0.59	5.43E-07	2.13E-06
**AC005062.2**	-2.70	5.46E-07	2.14E-06
**RP11-829H16.5**	-5.81	5.47E-07	2.15E-06
**FKBP1AP2**	-5.64	5.48E-07	2.15E-06
**HAX1**	0.63	5.51E-07	2.16E-06

**WDR83OS**	-0.66	5.52E-07	2.16E-06
**MIR554**	-5.80	5.53E-07	2.17E-06
**RP11-25D3.1**	-5.58	5.54E-07	2.17E-06
**RGS19**	1.03	5.55E-07	2.18E-06
**SPTSSA**	1.10	5.55E-07	2.18E-06
**AC008074.5**	-5.50	5.56E-07	2.18E-06
**LYPD1**	4.38	5.57E-07	2.18E-06
**TNNI2**	-2.53	5.58E-07	2.19E-06
**COL21A1**	-1.16	5.60E-07	2.19E-06
**CWC25**	0.54	5.63E-07	2.21E-06
**C8orf58**	0.68	5.64E-07	2.21E-06
**FCF1P8**	-5.57	5.64E-07	2.21E-06
**RP11-272L14.2**	-2.46	5.64E-07	2.21E-06
**MIR3139**	-5.69	5.64E-07	2.21E-06
**EIF2S2P5**	-5.68	5.69E-07	2.23E-06
**THAP9**	-0.72	5.70E-07	2.23E-06
**RP11-804N13.1**	-4.24	5.71E-07	2.23E-06
**RP4-529N6.2**	2.87	5.73E-07	2.24E-06
**RP5-1136G13.2**	1.05	5.73E-07	2.24E-06
**AC010522.1**	-5.49	5.78E-07	2.26E-06
**RP11-257O5.2**	-1.45	5.78E-07	2.26E-06
**MKRN5P**	-4.21	5.79E-07	2.26E-06
**AC083875.2**	-5.55	5.79E-07	2.26E-06
**CTD-2186M15.3**	1.41	5.80E-07	2.27E-06
**DFNB31**	1.20	5.80E-07	2.27E-06
**EEF1A1P32**	-5.65	5.80E-07	2.27E-06
**DDX47**	-1.70	5.80E-07	2.27E-06
**HSPG2**	1.37	5.81E-07	2.27E-06
**EEF1A1P3**	-2.59	5.83E-07	2.28E-06
**RP11-701P16.2**	-2.70	5.85E-07	2.29E-06
**GANC**	-0.65	5.85E-07	2.29E-06
**MTUS1**	0.76	5.86E-07	2.29E-06
**LIFR**	-1.49	5.86E-07	2.29E-06
**PPIHP1**	-5.77	5.86E-07	2.29E-06
**PNCK**	3.04	5.87E-07	2.29E-06
**RP11-446N19.1**	-1.84	5.88E-07	2.30E-06
**G39805**	-5.59	5.91E-07	2.31E-06
**RGCC**	1.39	5.92E-07	2.31E-06
**SMCO4**	1.27	5.93E-07	2.31E-06
**INPP5A**	-0.64	5.93E-07	2.32E-06
**MYH10**	0.80	5.93E-07	2.32E-06
**BMS1P10**	1.69	5.94E-07	2.32E-06
**WDR91**	0.79	5.94E-07	2.32E-06
**LRRC61**	0.94	5.97E-07	2.33E-06
**VNN3**	3.75	5.98E-07	2.33E-06

**RP11-1023L17.1**	-1.48	5.98E-07	2.33E-06
**APAF1**	-0.86	5.98E-07	2.33E-06
**PRKCI**	-0.61	6.00E-07	2.34E-06
**AC008592.3**	1.96	6.00E-07	2.34E-06
**SLC7A5**	1.19	6.01E-07	2.34E-06
**FAM166A**	-5.86	6.07E-07	2.37E-06
**SPRR2A**	5.56	6.08E-07	2.37E-06
**PSG11**	-5.55	6.08E-07	2.37E-06
**AC008079.10**	-2.08	6.09E-07	2.37E-06
**RP11-317B17.4**	-5.38	6.09E-07	2.37E-06
**SCARNA11**	-5.32	6.11E-07	2.38E-06
**FOXF1**	3.44	6.13E-07	2.39E-06
**SIRT1**	-0.49	6.14E-07	2.39E-06
**ARHGAP32**	-0.71	6.15E-07	2.39E-06
**DRAM1**	1.38	6.15E-07	2.40E-06
**RP11-613F22.8**	-3.29	6.16E-07	2.40E-06
**SNORD82**	-5.91	6.16E-07	2.40E-06
**MIR3142HG**	-2.18	6.17E-07	2.40E-06
**DNAJB4**	-1.07	6.19E-07	2.41E-06
**STX8**	0.60	6.20E-07	2.42E-06
**MYBBP1A**	0.77	6.24E-07	2.43E-06
**RP13-1056D16.2**	-5.46	6.24E-07	2.43E-06
**RP11-1086F11.1**	-4.16	6.25E-07	2.43E-06
**SGOL1**	-1.25	6.28E-07	2.44E-06
**RIMKLA**	2.01	6.30E-07	2.45E-06
**RP11-166N17.1**	-4.15	6.33E-07	2.46E-06
**CACTIN**	0.65	6.34E-07	2.47E-06
**FLG**	-1.58	6.34E-07	2.47E-06
**ZNF746**	0.58	6.36E-07	2.47E-06
**RP11-565F19.2**	-1.52	6.38E-07	2.48E-06
**C12orf65**	0.49	6.38E-07	2.48E-06
**RP11-36N20.1**	-5.70	6.38E-07	2.48E-06
**G3030**	3.88	6.38E-07	2.48E-06
**FOXN3-AS1**	1.37	6.39E-07	2.48E-06
**DNAH14**	-1.56	6.39E-07	2.48E-06
**RP11-474P2.2**	-2.37	6.40E-07	2.49E-06
**NT5C1B**	-3.11	6.41E-07	2.49E-06
**FOXI3**	-5.96	6.41E-07	2.49E-06
**EIF3K**	0.57	6.42E-07	2.49E-06
**RP11-211G3.3**	-4.19	6.45E-07	2.50E-06
**RP11-44F21.3**	-3.30	6.45E-07	2.50E-06
**N4BP2L1**	-0.94	6.45E-07	2.51E-06
**LOR**	1.52	6.46E-07	2.51E-06
**RP11-627K11.3**	-5.59	6.48E-07	2.51E-06
**MDH2**	0.65	6.48E-07	2.52E-06

**SLCO2B1**	1.95	6.49E-07	2.52E-06
**G42703**	-5.64	6.50E-07	2.52E-06
**PGS1**	0.69	6.51E-07	2.52E-06
**CTD-2231E14.8**	-3.16	6.51E-07	2.53E-06
**UBXN10**	-1.55	6.54E-07	2.54E-06
**RP11-305L7.7**	-1.60	6.54E-07	2.54E-06
**RP11-798M19.3**	-2.29	6.55E-07	2.54E-06
**TUBA3D**	-2.23	6.55E-07	2.54E-06
**RP11-201O14.2**	-5.39	6.58E-07	2.55E-06
**PRADC1**	0.76	6.59E-07	2.56E-06
**SIDT2**	0.62	6.59E-07	2.56E-06
**ERI3-IT1**	-3.76	6.60E-07	2.56E-06
**XLOC_009790**	4.85	6.62E-07	2.57E-06
**RNA5SP20**	-5.78	6.63E-07	2.57E-06
**RP11-693M3.1**	-2.55	6.64E-07	2.57E-06
**CYP4F8**	-4.96	6.67E-07	2.58E-06
**SNRNP35**	0.71	6.68E-07	2.59E-06
**PPAT**	-0.65	6.72E-07	2.60E-06
**CTD-3094K11.1**	3.10	6.72E-07	2.60E-06
**RP11-389G6.4**	-5.43	6.72E-07	2.60E-06
**MAGOHB**	-0.58	6.73E-07	2.61E-06
**RP11-147L13.8**	-2.38	6.75E-07	2.61E-06
**RPL12P31**	-5.86	6.75E-07	2.61E-06
**AC093732.1**	-4.49	6.78E-07	2.62E-06
**RIT1**	0.54	6.79E-07	2.63E-06
**TSSK4**	-1.57	6.80E-07	2.63E-06
**AC073043.1**	-3.58	6.80E-07	2.63E-06
**RBMS1P1**	-3.56	6.81E-07	2.63E-06
**RPS20P4**	-5.73	6.81E-07	2.64E-06
**RP11-800A18.3**	-1.72	6.83E-07	2.64E-06
**TADA2B**	0.40	6.83E-07	2.64E-06
**FAM186B**	-2.25	6.83E-07	2.64E-06
**RP11-37B2.1**	-1.51	6.84E-07	2.64E-06
**G31829**	-5.31	6.86E-07	2.65E-06
**EIF3I**	0.75	6.87E-07	2.66E-06
**LRRC47**	0.54	6.89E-07	2.66E-06
**AC147651.4**	2.56	6.91E-07	2.67E-06
**CTA-972D3.2**	-5.48	6.91E-07	2.67E-06
**SNRK**	-0.68	6.92E-07	2.67E-06
**AP000437.3**	-2.09	6.93E-07	2.68E-06
**DDIT3**	0.99	6.94E-07	2.68E-06
**RP6-24A23.7**	-3.33	6.94E-07	2.68E-06
**INTS7**	-0.71	6.98E-07	2.70E-06
**AL391994.1**	-5.52	7.00E-07	2.70E-06
**TMPRSS11GP**	-3.48	7.00E-07	2.70E-06

**KLHL3**	1.10	7.01E-07	2.70E-06
**RNU6-1033P**	-5.80	7.01E-07	2.71E-06
**RP11-244G12.1**	-6.20	7.03E-07	2.71E-06
**LMNB2**	0.84	7.04E-07	2.72E-06
**MAD2L1BP**	0.61	7.05E-07	2.72E-06
**SLC9B1**	-1.34	7.05E-07	2.72E-06
**CTNND2**	-3.20	7.09E-07	2.74E-06
**MTAP**	-0.46	7.10E-07	2.74E-06
**RP11-264L1.3**	-6.03	7.14E-07	2.75E-06
**RP11-417B4.2**	-5.51	7.16E-07	2.76E-06
**CTD-2007L18.5**	-4.47	7.17E-07	2.76E-06
**ADAP1**	1.31	7.17E-07	2.76E-06
**ME3**	1.74	7.17E-07	2.76E-06
**SAAL1**	0.59	7.20E-07	2.77E-06
**RP11-546B15.1**	-3.10	7.20E-07	2.77E-06
**DDX58**	-0.91	7.22E-07	2.78E-06
**ZNF501**	-0.93	7.24E-07	2.79E-06
**CD302**	-1.32	7.24E-07	2.79E-06
**SCYL3**	-0.54	7.25E-07	2.79E-06
**MTHFD2L**	-1.09	7.25E-07	2.79E-06
**RP11-5N19.3**	-5.42	7.30E-07	2.81E-06
**POPDC2**	1.86	7.31E-07	2.81E-06
**AC010761.13**	-2.50	7.31E-07	2.81E-06
**RP4-802A10.1**	-4.89	7.31E-07	2.81E-06
**CTC-550B14.6**	-5.50	7.32E-07	2.82E-06
**PTCHD4**	3.96	7.33E-07	2.82E-06
**DOLK**	0.72	7.35E-07	2.83E-06
**CASZ1**	-0.84	7.35E-07	2.83E-06
**BTG2**	1.11	7.39E-07	2.84E-06
**CABP1**	1.85	7.39E-07	2.84E-06
**LRRC56**	1.14	7.41E-07	2.85E-06
**SHOX2**	2.29	7.43E-07	2.86E-06
**CDKL4**	-3.46	7.43E-07	2.86E-06
**MIRLET7F1**	-5.48	7.45E-07	2.86E-06
**RP11-103C16.2**	-3.20	7.45E-07	2.87E-06
**MRPL32**	0.61	7.46E-07	2.87E-06
**KCNC3**	1.82	7.46E-07	2.87E-06
**PALM2**	-1.92	7.47E-07	2.87E-06
**RP4-655J12.4**	-2.86	7.48E-07	2.87E-06
**AP001439.2**	-5.60	7.48E-07	2.87E-06
**FAM171B**	-1.37	7.49E-07	2.88E-06
**C12orf29**	-1.03	7.49E-07	2.88E-06
**CEP76**	-0.94	7.50E-07	2.88E-06
**SALL1**	3.64	7.53E-07	2.89E-06
**CTD-3234P18.6**	-2.46	7.55E-07	2.90E-06

**RP11-295M3.2**	-4.78	7.55E-07	2.90E-06
**SNORD116-24**	-5.57	7.58E-07	2.91E-06
**RAB40B**	0.59	7.59E-07	2.91E-06
**ADRA1D**	4.58	7.62E-07	2.92E-06
**RP11-274B21.10**	-3.37	7.64E-07	2.93E-06
**RNA5SP187**	-5.11	7.66E-07	2.94E-06
**RP11-529H22.1**	-5.51	7.67E-07	2.94E-06
**VKORC1L1**	-0.69	7.67E-07	2.94E-06
**SDPR**	1.50	7.70E-07	2.95E-06
**TM9SF3**	-0.64	7.73E-07	2.96E-06
**CECR1**	1.58	7.77E-07	2.98E-06
**RP11-15M15.1**	-5.79	7.78E-07	2.98E-06
**WIPI2**	0.68	7.80E-07	2.99E-06
**SLC9A9-AS1**	-5.47	7.82E-07	3.00E-06
**NOS1**	-2.04	7.82E-07	3.00E-06
**GRIP1**	-1.13	7.83E-07	3.00E-06
**SLC22A23**	0.92	7.83E-07	3.00E-06
**CTA-390C10.10**	-1.80	7.85E-07	3.01E-06
**EIF2S2P3**	3.52	7.85E-07	3.01E-06
**TMEM182**	1.45	7.86E-07	3.01E-06
**RP11-314N13.9**	-2.13	7.86E-07	3.01E-06
**KATNBL1**	-0.55	7.86E-07	3.01E-06
**PGAM1P6**	-5.44	7.88E-07	3.02E-06
**LONRF3**	-1.88	7.89E-07	3.02E-06
**LINC01359**	-2.57	7.89E-07	3.02E-06
**CCAT2**	-5.66	7.90E-07	3.02E-06
**RPL7P7**	-5.71	7.90E-07	3.02E-06
**RP1-102H19.7**	-5.45	7.92E-07	3.03E-06
**RPL7AP65**	-5.45	7.92E-07	3.03E-06
**RPL3P6**	-4.02	7.94E-07	3.04E-06
**VPS26AP1**	-5.43	7.94E-07	3.04E-06
**RNU6-6P**	-5.53	7.95E-07	3.04E-06
**ZNF514**	-0.94	7.97E-07	3.05E-06
**FAM195A**	1.58	7.97E-07	3.05E-06
**MORC2**	0.60	7.99E-07	3.05E-06
**RP11-39E3.5**	-5.42	7.99E-07	3.05E-06
**G5758**	-1.98	8.00E-07	3.06E-06
**ATL2**	-0.64	8.02E-07	3.07E-06
**RAD51AP1P1**	-5.60	8.03E-07	3.07E-06
**YBX1P6**	-1.87	8.03E-07	3.07E-06
**ROCK2**	-0.61	8.05E-07	3.07E-06
**EFCAB14-AS1**	-3.21	8.06E-07	3.08E-06
**ZNF280B**	-1.77	8.10E-07	3.09E-06
**C15orf59**	-1.37	8.10E-07	3.09E-06
**NOP56P3**	-5.43	8.10E-07	3.09E-06

**RMRPP4**	-5.60	8.10E-07	3.09E-06
**BAIAP2L2**	1.51	8.13E-07	3.10E-06
**RPL7P17**	-5.46	8.15E-07	3.11E-06
**RP11-927P21.4**	1.42	8.16E-07	3.12E-06
**IL16**	-1.14	8.18E-07	3.12E-06
**RP11-223P11.3**	-2.62	8.19E-07	3.12E-06
**Z83844.1**	-4.61	8.20E-07	3.13E-06
**ADA**	1.53	8.21E-07	3.13E-06
**TBX5-AS1**	-2.25	8.21E-07	3.13E-06
**RP11-406H23.5**	-5.68	8.24E-07	3.14E-06
**BTF3L4P1**	-5.46	8.26E-07	3.15E-06
**G9978**	-0.97	8.26E-07	3.15E-06
**POM121L14P**	-5.53	8.26E-07	3.15E-06
**IFIT3**	1.69	8.27E-07	3.15E-06
**RP5-855D21.1**	-1.86	8.31E-07	3.17E-06
**GPRIN1**	2.15	8.32E-07	3.17E-06
**RP1-41C23.1**	-5.61	8.33E-07	3.18E-06
**IGHM**	4.56	8.37E-07	3.19E-06
**LURAP1L-AS1**	-3.13	8.38E-07	3.19E-06
**ZNF225**	-0.70	8.42E-07	3.21E-06
**IMMP1L**	-0.69	8.45E-07	3.22E-06
**WBP4**	0.65	8.45E-07	3.22E-06
**CTD-2175A23.1**	-5.55	8.50E-07	3.24E-06
**ANKRD18B**	-1.47	8.51E-07	3.24E-06
**ICOSLG**	1.22	8.53E-07	3.25E-06
**CPM**	-0.87	8.57E-07	3.26E-06
**RRH**	-3.58	8.57E-07	3.26E-06
**AC068657.2**	-5.43	8.60E-07	3.27E-06
**MOGS**	0.66	8.61E-07	3.27E-06
**AL133262.1**	-5.48	8.64E-07	3.29E-06
**ZBED6CL**	1.06	8.65E-07	3.29E-06
**RP11-244N9.4**	-3.55	8.68E-07	3.30E-06
**IGSF21**	3.74	8.73E-07	3.32E-06
**EEF1GP2**	-5.88	8.74E-07	3.32E-06
**RN7SKP151**	-5.62	8.78E-07	3.34E-06
**CHRNA9**	2.61	8.79E-07	3.34E-06
**LINC01364**	-3.37	8.80E-07	3.35E-06
**RP11-554D20.2**	-4.54	8.82E-07	3.35E-06
**PRED57**	-5.55	8.82E-07	3.35E-06
**RP11-135A24.2**	-5.52	8.84E-07	3.36E-06
**STX1A**	1.52	8.94E-07	3.40E-06
**CTA-228A9.4**	-2.48	8.95E-07	3.40E-06
**RP11-365O16.5**	-5.45	8.96E-07	3.40E-06
**LYPLAL1**	-0.75	9.01E-07	3.42E-06
**RP11-453E17.4**	-5.47	9.03E-07	3.43E-06

**CTC-506B8.1**	-5.64	9.04E-07	3.43E-06
**IL36G**	1.77	9.04E-07	3.43E-06
**XLOC_012871**	-5.50	9.04E-07	3.43E-06
**RP11-176D17.3**	-3.50	9.04E-07	3.43E-06
**NRARP**	1.16	9.05E-07	3.43E-06
**CLCC1**	-0.54	9.05E-07	3.44E-06
**KCTD20**	-0.59	9.06E-07	3.44E-06
**CBLN3**	1.44	9.07E-07	3.44E-06
**ARF4**	0.76	9.10E-07	3.45E-06
**AC132872.2**	-2.31	9.12E-07	3.46E-06
**ICE2**	-0.57	9.12E-07	3.46E-06
**ZSCAN16**	-0.83	9.12E-07	3.46E-06
**VEGFA**	1.41	9.13E-07	3.46E-06
**RP11-295P9.12**	-3.32	9.13E-07	3.46E-06
**FDPSP2**	-5.75	9.13E-07	3.46E-06
**POU5F1P5**	-4.47	9.14E-07	3.46E-06
**RP1-261D10.1**	-5.59	9.16E-07	3.47E-06
**MESP1**	2.76	9.16E-07	3.47E-06
**TRIM21**	0.81	9.17E-07	3.48E-06
**ZNF707**	1.05	9.19E-07	3.48E-06
**SNORA31**	-2.18	9.19E-07	3.48E-06
**NIT1**	0.86	9.20E-07	3.48E-06
**STK17B**	-0.93	9.24E-07	3.50E-06
**G33562**	-1.63	9.24E-07	3.50E-06
**RPS3P2**	-5.22	9.26E-07	3.50E-06
**AC064852.4**	-5.37	9.27E-07	3.51E-06
**WDR78**	-0.92	9.28E-07	3.51E-06
**FOXP3**	1.82	9.29E-07	3.52E-06
**FETUB**	2.17	9.30E-07	3.52E-06
**RNU7-84P**	-5.34	9.31E-07	3.52E-06
**TUG1**	-0.55	9.34E-07	3.54E-06
**SH2D5**	3.70	9.40E-07	3.56E-06
**AC017101.10**	-5.68	9.43E-07	3.57E-06
**RP11-354E23.3**	-5.79	9.45E-07	3.58E-06
**EIF5A2P1**	-5.45	9.47E-07	3.58E-06
**SECISBP2**	-0.64	9.47E-07	3.58E-06
**RALGPS2**	-0.91	9.49E-07	3.59E-06
**CDCA4P4**	-2.01	9.51E-07	3.60E-06
**BIRC6-AS2**	-4.64	9.54E-07	3.61E-06
**DUSP22**	0.48	9.59E-07	3.63E-06
**RP11-471L13.3**	-3.63	9.68E-07	3.66E-06
**LRRC4C**	2.71	9.69E-07	3.66E-06
**HOXC4**	1.52	9.70E-07	3.66E-06
**SCARNA8**	-5.74	9.72E-07	3.67E-06
**TXNP5**	-5.81	9.74E-07	3.68E-06

**RP11-452J21.2**	-4.52	9.75E-07	3.68E-06
**RP11-363E6.3**	2.42	9.81E-07	3.71E-06
**TSLP**	1.89	9.84E-07	3.72E-06
**DSG4**	-3.14	9.85E-07	3.72E-06
**AC096921.2**	-5.45	9.86E-07	3.72E-06
**OSBPL11**	-0.55	9.86E-07	3.72E-06
**APOL4**	1.84	9.92E-07	3.74E-06
**GPR179**	-5.68	9.94E-07	3.75E-06
**AC067959.1**	-1.77	9.95E-07	3.75E-06
**RP11-673C5.1**	1.13	9.95E-07	3.75E-06
**PTH1R**	2.10	9.96E-07	3.76E-06
**CCL4**	4.08	9.99E-07	3.77E-06
**TPPP3**	-1.76	9.99E-07	3.77E-06
**ANKRD33B-AS1**	-5.45	1.00E-06	3.77E-06
**RNU6-1153P**	-5.32	1.00E-06	3.77E-06
**MYBL2**	1.19	1.00E-06	3.77E-06
**GLB1L**	0.98	1.00E-06	3.78E-06
**ABHD14A**	1.45	1.00E-06	3.78E-06
**G0S2**	4.92	1.00E-06	3.79E-06
**SUPT4H1P1**	-5.36	1.01E-06	3.80E-06
**SP140L**	1.70	1.01E-06	3.80E-06
**CTC-451P13.1**	-2.78	1.01E-06	3.80E-06
**RNU6-1334P**	-5.56	1.01E-06	3.80E-06
**RP3-406A7.3**	-5.38	1.01E-06	3.81E-06
**AP4E1**	-0.75	1.01E-06	3.81E-06
**RP4-569M23.4**	-5.58	1.01E-06	3.81E-06
**C17orf78**	-4.03	1.02E-06	3.83E-06
**AC005077.14**	2.63	1.02E-06	3.83E-06
**AC003989.4**	-5.41	1.02E-06	3.83E-06
**RP11-739P1.2**	-3.00	1.02E-06	3.84E-06
**NUTM2B-AS1**	-1.20	1.02E-06	3.84E-06
**RP11-39E3.4**	-5.64	1.02E-06	3.84E-06
**FAT3**	-1.86	1.02E-06	3.85E-06
**BID**	0.77	1.03E-06	3.86E-06
**RP5-884C9.3**	-5.49	1.03E-06	3.86E-06
**RP11-170M17.2**	2.28	1.03E-06	3.87E-06
**PLBD1**	1.17	1.03E-06	3.87E-06
**RP11-166B2.5**	-5.57	1.03E-06	3.88E-06
**RP5-1027G4.3**	-2.43	1.04E-06	3.91E-06
**ST13P10**	-5.63	1.04E-06	3.91E-06
**TPM3P1**	-5.86	1.04E-06	3.92E-06
**PALMD**	-0.92	1.04E-06	3.93E-06
**RNF144B**	-0.76	1.05E-06	3.93E-06
**HECTD2**	-0.77	1.05E-06	3.94E-06
**RP3-405J10.3**	-2.16	1.05E-06	3.95E-06

**AL591069.1**	-3.04	1.05E-06	3.95E-06
**FRMD6**	-0.76	1.05E-06	3.95E-06
**ELAC2**	0.50	1.05E-06	3.95E-06
**MIR103A1**	-5.44	1.05E-06	3.95E-06
**RP11-41O4.1**	-2.95	1.05E-06	3.96E-06
**KRT18P57**	-5.22	1.06E-06	3.96E-06
**ACTG1P14**	-4.13	1.06E-06	3.97E-06
**GAS8**	0.95	1.06E-06	3.98E-06
**RP11-90P5.1**	-5.56	1.06E-06	3.98E-06
**PNPLA5**	-4.46	1.06E-06	3.98E-06
**AIMP2**	0.61	1.06E-06	3.98E-06
**AC003958.2**	-3.88	1.06E-06	3.99E-06
**UFL1**	-0.69	1.07E-06	4.02E-06
**SEL1L**	-0.68	1.08E-06	4.04E-06
**USP6NL**	-0.76	1.08E-06	4.06E-06
**SRMP2**	-5.58	1.08E-06	4.06E-06
**RP11-718B12.5**	-3.69	1.08E-06	4.07E-06
**LINC01305**	3.23	1.09E-06	4.07E-06
**CRISPLD2**	1.72	1.09E-06	4.07E-06
**HSPA14**	-0.44	1.09E-06	4.08E-06
**RP11-49K24.5**	-5.57	1.10E-06	4.11E-06
**RP11-459F6.1**	-5.41	1.10E-06	4.12E-06
**RP1-12G14.6**	-5.39	1.10E-06	4.12E-06
**USP7**	-0.48	1.10E-06	4.13E-06
**MIR374B**	-5.44	1.10E-06	4.13E-06
**SRGAP2-AS1**	-3.36	1.10E-06	4.14E-06
**AK3P3**	-2.20	1.11E-06	4.16E-06
**AC019064.1**	-3.22	1.11E-06	4.16E-06
**KAZN**	0.83	1.11E-06	4.17E-06
**RP5-1057I20.6**	-3.32	1.12E-06	4.18E-06
**GAREM**	-1.03	1.12E-06	4.19E-06
**RP11-757G1.6**	-1.39	1.12E-06	4.19E-06
**RP11-158M2.3**	-2.35	1.12E-06	4.19E-06
**RP11-49O14.3**	-5.78	1.12E-06	4.19E-06
**CTD-2165H16.4**	-1.44	1.12E-06	4.21E-06
**LYRM4**	0.75	1.13E-06	4.21E-06
**CPE**	1.80	1.13E-06	4.22E-06
**NOC2L**	0.69	1.13E-06	4.22E-06
**VANGL2**	0.93	1.13E-06	4.23E-06
**AJ003147.11**	-2.51	1.13E-06	4.23E-06
**RP11-567F11.1**	-3.38	1.13E-06	4.24E-06
**RP11-52J3.2**	-5.18	1.14E-06	4.25E-06
**RP4-758J18.10**	2.09	1.14E-06	4.25E-06
**NEDD9**	1.15	1.14E-06	4.26E-06
**CHD7**	-0.73	1.14E-06	4.27E-06

**NLRP10**	-1.96	1.15E-06	4.28E-06
**RP11-437F6.1**	-5.64	1.15E-06	4.29E-06
**ZNF831**	-2.11	1.15E-06	4.31E-06
**RP11-1148L6.5**	0.70	1.15E-06	4.31E-06
**DAGLB**	0.49	1.16E-06	4.32E-06
**TRMT2A**	0.91	1.16E-06	4.32E-06
**EDRF1-AS1**	-2.41	1.16E-06	4.33E-06
**C2CD4C**	2.58	1.16E-06	4.35E-06
**CFAP20**	0.59	1.17E-06	4.35E-06
**MTRNR2L8**	-2.10	1.17E-06	4.35E-06
**RAB17**	2.00	1.17E-06	4.37E-06
**KIAA0196**	-0.39	1.17E-06	4.37E-06
**GRIA4**	-1.73	1.17E-06	4.38E-06
**ZMYM4**	-0.50	1.18E-06	4.40E-06
**AC000089.3**	-3.22	1.18E-06	4.40E-06
**HOXB8**	4.04	1.18E-06	4.40E-06
**AC003101.1**	-5.13	1.18E-06	4.41E-06
**CLDN23**	-1.54	1.18E-06	4.41E-06
**RPUSD3**	0.57	1.18E-06	4.41E-06
**RP11-172E10.1**	-5.58	1.19E-06	4.42E-06
**LINC00924**	6.20	1.19E-06	4.42E-06
**KIT**	-1.04	1.19E-06	4.43E-06
**PREPL**	-0.99	1.19E-06	4.44E-06
**CTB-113P19.1**	-2.31	1.19E-06	4.44E-06
**RP11-972P1.7**	-2.01	1.20E-06	4.46E-06
**CH17-262A2.1**	-4.15	1.20E-06	4.46E-06
**FOXK1**	-0.77	1.20E-06	4.47E-06
**RPL23AP63**	-5.58	1.20E-06	4.47E-06
**RPL23AP30**	-5.28	1.20E-06	4.47E-06
**SFRP1**	2.13	1.20E-06	4.48E-06
**HINT2**	0.75	1.20E-06	4.48E-06
**DPH6**	-0.89	1.20E-06	4.48E-06
**RP5-1086K13.1**	-2.42	1.20E-06	4.48E-06
**RP11-598F7.6**	5.80	1.21E-06	4.49E-06
**OR5P2**	-5.77	1.21E-06	4.50E-06
**FAM166B**	-1.84	1.21E-06	4.50E-06
**AL138706.1**	-5.39	1.22E-06	4.54E-06
**SLC9B2**	0.82	1.22E-06	4.55E-06
**RP11-27K13.3**	-2.55	1.22E-06	4.55E-06
**RP11-52I18.1**	-4.50	1.22E-06	4.56E-06
**MAPT-IT1**	-5.36	1.23E-06	4.56E-06
**RP11-144H23.2**	-5.52	1.23E-06	4.56E-06
**ODF2**	0.68	1.23E-06	4.56E-06
**RP11-48O20.5**	-5.29	1.23E-06	4.57E-06
**RP4-593M8.1**	-5.38	1.23E-06	4.59E-06

**NDOR1**	1.08	1.23E-06	4.59E-06
**RP11-685N10.1**	-2.50	1.23E-06	4.59E-06
**PHLDB3**	0.71	1.24E-06	4.59E-06
**HIST2H3C**	-5.46	1.24E-06	4.60E-06
**TMCO5A**	-5.33	1.24E-06	4.62E-06
**RP11-486O12.2**	-1.83	1.25E-06	4.63E-06
**PWAR6**	-1.50	1.25E-06	4.64E-06
**LL0XNC01-7P3.1**	-1.53	1.25E-06	4.65E-06
**SNORD14A**	-2.24	1.25E-06	4.66E-06
**NKAP**	0.48	1.25E-06	4.66E-06
**SH3BGR**	1.42	1.25E-06	4.66E-06
**RP11-106D4.2**	-2.48	1.26E-06	4.66E-06
**PDZD9**	-2.93	1.26E-06	4.67E-06
**RP11-795F19.1**	-2.14	1.26E-06	4.68E-06
**LXN**	0.99	1.26E-06	4.68E-06
**SFTPD**	1.26	1.26E-06	4.68E-06
**SLCO1A2**	-5.67	1.26E-06	4.69E-06
**STXBP3**	-0.65	1.27E-06	4.71E-06
**ZNF677**	-0.89	1.27E-06	4.71E-06
**RPSAP6**	-5.76	1.27E-06	4.72E-06
**CTD-2017F17.2**	4.76	1.28E-06	4.73E-06
**ZBTB26**	-0.75	1.28E-06	4.73E-06
**RIMS3**	1.40	1.28E-06	4.74E-06
**RP11-480I12.5**	1.82	1.28E-06	4.74E-06
**RP11-298I3.3**	-4.28	1.29E-06	4.77E-06
**ZNF10**	-0.92	1.29E-06	4.78E-06
**MAP4**	0.54	1.29E-06	4.78E-06
**ZNF226**	-0.66	1.29E-06	4.78E-06
**HS3ST4**	4.43	1.30E-06	4.81E-06
**RP11-575L7.4**	-2.33	1.30E-06	4.82E-06
**PRR34-AS1**	-1.25	1.30E-06	4.82E-06
**HACE1**	-0.54	1.30E-06	4.82E-06
**RP4-706A16.3**	-1.36	1.30E-06	4.83E-06
**PPIF**	0.85	1.31E-06	4.83E-06
**RUVBL1-AS1**	-5.58	1.31E-06	4.85E-06
**RERE**	0.84	1.31E-06	4.85E-06
**AB019441.29**	-2.26	1.31E-06	4.85E-06
**C19orf38**	3.26	1.31E-06	4.87E-06
**MRPS18A**	0.72	1.32E-06	4.88E-06
**SNORA9**	-3.05	1.32E-06	4.89E-06
**ING1**	0.58	1.32E-06	4.89E-06
**DERL1**	0.44	1.32E-06	4.89E-06
**COX6CP1**	-3.86	1.33E-06	4.92E-06
**DDX55**	0.77	1.34E-06	4.94E-06
**MALL**	1.03	1.34E-06	4.95E-06

**RP11-798G7.8**	-3.41	1.34E-06	4.96E-06
**RPEP3**	-5.54	1.34E-06	4.96E-06
**KNDC1**	2.83	1.35E-06	4.99E-06
**RP5-945I17.2**	-5.65	1.35E-06	4.99E-06
**PDF**	1.29	1.35E-06	5.00E-06
**NLRP3**	2.39	1.35E-06	5.01E-06
**MRPS15**	0.60	1.36E-06	5.01E-06
**OPLAH**	0.91	1.36E-06	5.01E-06
**GCNT2**	-0.98	1.36E-06	5.01E-06
**TSPEAR**	-2.58	1.36E-06	5.02E-06
**PGLYRP3**	1.30	1.37E-06	5.05E-06
**ZBTB48**	0.89	1.37E-06	5.07E-06
**RP11-799B12.2**	-3.98	1.37E-06	5.07E-06
**ACTA1**	-3.46	1.37E-06	5.08E-06
**CXCL17**	2.83	1.38E-06	5.08E-06
**PCDHB9**	1.24	1.38E-06	5.09E-06
**MAP3K14**	0.92	1.38E-06	5.09E-06
**RP11-158G18.1**	-5.62	1.38E-06	5.10E-06
**ZNF33BP1**	-3.90	1.39E-06	5.11E-06
**RP11-11C20.3**	-2.78	1.39E-06	5.11E-06
**HS3ST3A1**	3.13	1.39E-06	5.12E-06
**TUBB8P1**	-3.34	1.39E-06	5.13E-06
**TUSC5**	6.51	1.39E-06	5.13E-06
**MATN3**	2.66	1.39E-06	5.14E-06
**TANC2**	-0.80	1.40E-06	5.15E-06
**DGAT2**	-1.28	1.40E-06	5.15E-06
**RP4-575N6.4**	3.24	1.40E-06	5.17E-06
**AFAP1**	1.61	1.41E-06	5.18E-06
**PRPF40B**	0.90	1.41E-06	5.18E-06
**RNU6-1188P**	-5.67	1.41E-06	5.19E-06
**RP11-480I12.2**	-3.91	1.41E-06	5.19E-06
**RABEPK**	0.67	1.41E-06	5.19E-06
**RP11-479I16.2**	-5.38	1.41E-06	5.20E-06
**RP11-192H23.7**	-5.48	1.41E-06	5.21E-06
**ENOX1**	1.89	1.42E-06	5.21E-06
**QTRTD1**	-0.48	1.42E-06	5.21E-06
**SPSB1**	0.80	1.43E-06	5.25E-06
**ZBTB12**	1.67	1.43E-06	5.27E-06
**MAK**	-1.90	1.43E-06	5.28E-06
**SHF**	1.02	1.43E-06	5.28E-06
**CTB-31N19.3**	-2.49	1.43E-06	5.28E-06
**RYKP1**	-3.02	1.44E-06	5.29E-06
**CRAMP1**	0.59	1.44E-06	5.29E-06
**AC096574.4**	-3.60	1.44E-06	5.29E-06
**RP11-494K3.2**	-5.49	1.45E-06	5.32E-06

**RP11-613D13.8**	3.91	1.45E-06	5.32E-06
**LINC01481**	-1.23	1.45E-06	5.32E-06
**OR51E2**	5.02	1.45E-06	5.32E-06
**ACPP**	-1.16	1.45E-06	5.34E-06
**FAM76B**	-0.66	1.45E-06	5.35E-06
**PRSS22**	2.15	1.46E-06	5.35E-06
**RP11-299J3.6**	-3.03	1.46E-06	5.35E-06
**SCARNA4**	-3.46	1.46E-06	5.36E-06
**SNRPC**	0.82	1.46E-06	5.36E-06
**AC006042.8**	-2.24	1.46E-06	5.36E-06
**CHRM2**	-5.87	1.47E-06	5.39E-06
**TUBB2A**	1.17	1.47E-06	5.39E-06
**MTL5**	1.24	1.47E-06	5.39E-06
**GLA**	0.81	1.47E-06	5.40E-06
**PCDHB2**	1.76	1.47E-06	5.40E-06
**RGL1**	1.00	1.47E-06	5.41E-06
**RP4-681N20.5**	-2.66	1.47E-06	5.41E-06
**FGF14-AS2**	1.26	1.48E-06	5.43E-06
**SYK**	0.57	1.48E-06	5.43E-06
**ARMC9**	1.24	1.48E-06	5.44E-06
**CA15P1**	-4.54	1.49E-06	5.46E-06
**RP11-241J12.3**	-5.59	1.49E-06	5.47E-06
**DBT**	-0.72	1.49E-06	5.47E-06
**C7orf73**	0.85	1.49E-06	5.47E-06
**CTC-379B2.4**	-5.29	1.49E-06	5.47E-06
**MAZ**	0.80	1.49E-06	5.48E-06
**RP11-420A21.1**	-5.74	1.49E-06	5.48E-06
**RP11-386I14.3**	-5.28	1.49E-06	5.48E-06
**RP11-857B24.5**	-2.48	1.50E-06	5.48E-06
**VSIG10L**	1.26	1.50E-06	5.49E-06
**G35488**	-1.92	1.50E-06	5.49E-06
**AC018738.2**	3.84	1.50E-06	5.50E-06
**CNOT2**	-0.49	1.50E-06	5.50E-06
**HNRNPA1P68**	-5.51	1.50E-06	5.50E-06
**ZZEF1**	-0.59	1.50E-06	5.51E-06
**RP11-646I6.6**	-5.37	1.50E-06	5.51E-06
**PHKG1**	-1.21	1.51E-06	5.53E-06
**RTEL1P1**	-2.64	1.51E-06	5.54E-06
**AC108142.1**	4.61	1.51E-06	5.54E-06
**CFAP73**	-1.85	1.51E-06	5.54E-06
**COLCA2**	2.74	1.52E-06	5.56E-06
**MIR3648**	-3.23	1.52E-06	5.57E-06
**ZC3HC1**	0.62	1.52E-06	5.58E-06
**NONO**	0.52	1.53E-06	5.58E-06
**RP11-439L18.2**	-2.13	1.53E-06	5.59E-06

**RP11-39E3.3**	-5.29	1.54E-06	5.62E-06
**RP11-277L2.3**	2.09	1.54E-06	5.63E-06
**G34070**	-6.90	1.54E-06	5.63E-06
**SRI**	0.72	1.54E-06	5.64E-06
**L2HGDH**	-0.73	1.54E-06	5.64E-06
**XLOC_012011**	-1.79	1.54E-06	5.64E-06
**MUC6**	-3.34	1.54E-06	5.64E-06
**SYT1**	-1.89	1.54E-06	5.65E-06
**BLACAT1**	2.52	1.55E-06	5.65E-06
**RP11-164N3.2**	-5.38	1.55E-06	5.66E-06
**AIRN**	-5.12	1.55E-06	5.67E-06
**SPTY2D1**	-0.56	1.56E-06	5.69E-06
**RP11-295K2.3**	-4.27	1.56E-06	5.72E-06
**RP11-806H10.4**	4.39	1.57E-06	5.72E-06
**G24464**	-3.47	1.57E-06	5.72E-06
**CHRND**	-3.96	1.57E-06	5.74E-06
**SNORD116-18**	-5.73	1.57E-06	5.74E-06
**SLC2A1**	1.19	1.57E-06	5.75E-06
**RPE**	-0.71	1.58E-06	5.75E-06
**ELAVL4**	-2.03	1.59E-06	5.79E-06
**POC1A**	1.06	1.59E-06	5.79E-06
**CTD-2006C1.2**	-1.27	1.59E-06	5.79E-06
**BBS10**	-0.82	1.59E-06	5.81E-06
**SNORD3B-1**	-2.56	1.59E-06	5.81E-06
**DNAJB7**	-4.41	1.59E-06	5.81E-06
**TMEM72**	-5.39	1.59E-06	5.81E-06
**RP1-167F1.2**	-1.84	1.59E-06	5.82E-06
**LINC00987**	1.91	1.60E-06	5.84E-06
**TMEM171**	1.63	1.60E-06	5.85E-06
**ZNF485**	-0.90	1.61E-06	5.85E-06
**KB-431C1.5**	-2.06	1.61E-06	5.86E-06
**SLFNL1**	-2.31	1.61E-06	5.86E-06
**RP1-206D15.5**	-4.00	1.61E-06	5.87E-06
**AZGP1P1**	1.95	1.61E-06	5.88E-06
**XLOC_007628**	-5.30	1.62E-06	5.89E-06
**PCDHGB6**	-0.85	1.62E-06	5.91E-06
**RNU7-80P**	-5.44	1.62E-06	5.91E-06
**PAICSP1**	-2.41	1.62E-06	5.91E-06
**PARD6A**	1.51	1.63E-06	5.93E-06
**XBP1P1**	-5.15	1.63E-06	5.93E-06
**PATL1**	0.80	1.63E-06	5.93E-06
**RP11-337C18.9**	-1.41	1.63E-06	5.94E-06
**ZNF23**	-1.33	1.63E-06	5.94E-06
**GGT7**	0.80	1.63E-06	5.94E-06
**KLB**	-1.15	1.63E-06	5.94E-06

**AC007365.3**	-2.41	1.63E-06	5.95E-06
**CYP4B1**	-2.14	1.64E-06	5.97E-06
**TRO**	1.68	1.64E-06	5.97E-06
**GYPC**	1.53	1.64E-06	5.97E-06
**RP4-803J11.2**	-5.69	1.64E-06	5.97E-06
**RNU6-171P**	-5.68	1.64E-06	5.97E-06
**NRN1**	1.41	1.64E-06	5.98E-06
**RP11-338L22.2**	-5.86	1.65E-06	5.99E-06
**RP11-666F17.2**	-5.61	1.65E-06	5.99E-06
**QPRT**	1.45	1.65E-06	6.01E-06
**RNU1-124P**	-5.27	1.65E-06	6.01E-06
**GATA2**	1.96	1.65E-06	6.01E-06
**RP11-829H16.3**	-3.62	1.65E-06	6.01E-06
**RP11-467D18.2**	-5.23	1.66E-06	6.04E-06
**C9orf172**	1.82	1.67E-06	6.06E-06
**DOCK9-AS2**	1.18	1.67E-06	6.06E-06
**SNX1**	0.31	1.67E-06	6.07E-06
**RPS14P4**	5.62	1.67E-06	6.07E-06
**MBNL2**	-0.73	1.69E-06	6.12E-06
**CTD-2525I3.3**	-4.23	1.69E-06	6.13E-06
**RP11-123O10.4**	-2.20	1.69E-06	6.13E-06
**AC007279.2**	-5.45	1.69E-06	6.14E-06
**RP11-133M8.3**	-5.64	1.69E-06	6.14E-06
**CCKBR**	-3.03	1.70E-06	6.17E-06
**GOLGA8H**	-2.07	1.70E-06	6.18E-06
**EEF1B2P1**	-5.68	1.70E-06	6.18E-06
**KCTD11**	0.91	1.70E-06	6.18E-06
**ZNF275**	1.25	1.70E-06	6.19E-06
**ATAD3C**	2.50	1.71E-06	6.20E-06
**RWDD3**	-1.32	1.71E-06	6.21E-06
**RP11-413M3.4**	-4.14	1.72E-06	6.23E-06
**IL17D**	-1.31	1.72E-06	6.23E-06
**LINC00959**	1.08	1.73E-06	6.26E-06
**SEC62-AS1**	-5.09	1.73E-06	6.26E-06
**WDR3**	-0.64	1.73E-06	6.27E-06
**SPATA13-AS1**	-5.47	1.73E-06	6.27E-06
**RAP2C-AS1**	-1.24	1.73E-06	6.28E-06
**NDNF**	-1.37	1.73E-06	6.28E-06
**ALDH1B1**	1.77	1.73E-06	6.29E-06
**NDFIP1P1**	-4.71	1.74E-06	6.30E-06
**RP11-545G3.1**	-5.53	1.74E-06	6.31E-06
**RP11-456J20.1**	-5.51	1.75E-06	6.33E-06
**RPL12P15**	-5.52	1.75E-06	6.33E-06
**MT-TV**	-6.33	1.75E-06	6.34E-06
**LFNG**	-0.89	1.75E-06	6.36E-06

**RP11-158M2.2**	-5.56	1.76E-06	6.38E-06
**EPC1**	-0.55	1.76E-06	6.38E-06
**RP11-16P20.3**	-5.33	1.76E-06	6.38E-06
**ZNF134**	-0.64	1.76E-06	6.38E-06
**CTD-2649C14.2**	-2.43	1.76E-06	6.39E-06
**OR10AB1P**	-6.00	1.77E-06	6.40E-06
**DEDD**	0.43	1.77E-06	6.40E-06
**COL3A1**	2.71	1.77E-06	6.42E-06
**RAP1GAP**	1.45	1.77E-06	6.42E-06
**PLCB3**	0.71	1.78E-06	6.43E-06
**GBAP1**	1.87	1.79E-06	6.46E-06
**RP11-395G23.3**	2.17	1.79E-06	6.47E-06
**AC087163.2**	-2.25	1.79E-06	6.47E-06
**CDC27**	-0.56	1.79E-06	6.48E-06
**RP11-321A17.4**	-5.50	1.79E-06	6.48E-06
**AC079807.3**	-5.12	1.79E-06	6.48E-06
**FTSJ2**	0.61	1.80E-06	6.49E-06
**RP11-420K14.6**	-4.43	1.80E-06	6.49E-06
**XLOC_010736**	4.30	1.80E-06	6.50E-06
**XXyac-YRM2039.3**	-2.44	1.80E-06	6.50E-06
**IFT20**	0.65	1.80E-06	6.50E-06
**CASP4**	0.60	1.80E-06	6.52E-06
**CCR4**	-2.51	1.80E-06	6.52E-06
**AC010969.1**	-2.04	1.80E-06	6.52E-06
**NR2F1-AS1**	3.01	1.81E-06	6.53E-06
**USP49**	-0.75	1.81E-06	6.55E-06
**WDR6**	1.04	1.82E-06	6.56E-06
**RN7SKP9**	-4.98	1.82E-06	6.56E-06
**MIR26B**	-5.24	1.82E-06	6.56E-06
**AC005522.7**	-5.13	1.83E-06	6.59E-06
**RP11-254B13.4**	-5.52	1.83E-06	6.59E-06
**RP11-56B16.1**	-3.63	1.83E-06	6.60E-06
**TDGF1P6**	-5.35	1.83E-06	6.60E-06
**ASPHD1**	3.11	1.83E-06	6.61E-06
**RP11-483C6.1**	-3.75	1.84E-06	6.62E-06
**LINC01389**	-2.60	1.84E-06	6.62E-06
**AP001432.14**	-2.20	1.84E-06	6.64E-06
**KHDC1**	-1.71	1.84E-06	6.64E-06
**FGF13**	2.54	1.84E-06	6.64E-06
**RP11-142C4.4**	-3.27	1.84E-06	6.65E-06
**HERC2**	-0.62	1.85E-06	6.66E-06
**RP11-54O7.3**	2.77	1.85E-06	6.67E-06
**RP11-705O24.1**	-4.57	1.85E-06	6.68E-06
**ANXA2P2**	-1.17	1.86E-06	6.69E-06
**CRYBA1**	-5.15	1.86E-06	6.69E-06

**CAP2**	1.49	1.86E-06	6.72E-06
**TMEM110**	-1.72	1.87E-06	6.72E-06
**CTD-2368P22.1**	-1.44	1.87E-06	6.73E-06
**LYST-AS1**	-5.39	1.87E-06	6.74E-06
**RP11-178C3.6**	-3.57	1.87E-06	6.74E-06
**ATP8A2P2**	-4.64	1.87E-06	6.74E-06
**C1orf131**	0.42	1.87E-06	6.74E-06
**FOSL2**	0.67	1.87E-06	6.74E-06
**SLC10A7**	-0.69	1.87E-06	6.75E-06
**FAM95C**	-2.71	1.88E-06	6.76E-06
**B4GALT4-AS1**	-5.21	1.88E-06	6.77E-06
**G18874**	-3.24	1.88E-06	6.78E-06
**RP11-800A18.4**	-5.11	1.88E-06	6.78E-06
**RP11-348N5.7**	-1.78	1.89E-06	6.79E-06
**RP11-1174L13.2**	-5.47	1.89E-06	6.80E-06
**G36288**	-2.47	1.89E-06	6.80E-06
**RP11-782C8.2**	-3.45	1.89E-06	6.82E-06
**AC114812.9**	-4.61	1.90E-06	6.83E-06
**NFIA-AS2**	3.80	1.91E-06	6.86E-06
**CYB561D2**	0.70	1.91E-06	6.86E-06
**RP11-95G17.2**	-5.28	1.91E-06	6.86E-06
**GATB**	0.86	1.91E-06	6.86E-06
**MED27**	0.67	1.91E-06	6.88E-06
**NUMB**	0.29	1.92E-06	6.89E-06
**AC011406.2**	-5.28	1.92E-06	6.89E-06
**GAS1**	1.19	1.92E-06	6.90E-06
**MKL2**	-0.81	1.93E-06	6.94E-06
**G14609**	-3.39	1.93E-06	6.95E-06
**SNORD12C**	-3.74	1.94E-06	6.96E-06
**SNORA65**	-2.19	1.94E-06	6.96E-06
**ZNF207**	-0.60	1.94E-06	6.96E-06
**SLC33A1**	-0.48	1.94E-06	6.97E-06
**LPCAT4**	1.03	1.96E-06	7.02E-06
**IL18**	-0.95	1.96E-06	7.02E-06
**G14419**	1.69	1.96E-06	7.04E-06
**RND1**	2.24	1.97E-06	7.05E-06
**TRAPPC2**	-0.61	1.97E-06	7.07E-06
**ABHD1**	-2.47	1.99E-06	7.13E-06
**PCDHGC3**	0.72	1.99E-06	7.14E-06
**EPB41L4A-AS2**	1.22	1.99E-06	7.16E-06
**G5386**	1.75	2.00E-06	7.16E-06
**SNORD73**	-5.38	2.00E-06	7.17E-06
**CMTR2**	-0.52	2.00E-06	7.18E-06
**RPL21P32**	-4.15	2.00E-06	7.18E-06
**ZNF366**	1.41	2.00E-06	7.18E-06

**LINC00427**	-5.94	2.00E-06	7.19E-06
**G26054**	2.79	2.02E-06	7.23E-06
**LRRC59**	0.77	2.02E-06	7.26E-06
**COL4A6**	-0.67	2.03E-06	7.27E-06
**RP11-731D1.1**	-3.51	2.03E-06	7.27E-06
**VARS2**	0.74	2.03E-06	7.28E-06
**CTB-113P19.5**	-1.37	2.03E-06	7.28E-06
**MED17**	-0.47	2.03E-06	7.28E-06
**RP11-473O4.4**	-5.33	2.03E-06	7.29E-06
**AC139103.1**	-5.35	2.04E-06	7.30E-06
**CSRP2BP**	-0.54	2.04E-06	7.31E-06
**LINC00475**	1.94	2.05E-06	7.33E-06
**WDR41**	-0.57	2.05E-06	7.34E-06
**CCNH**	-0.44	2.05E-06	7.35E-06
**TTC4**	-1.44	2.05E-06	7.35E-06
**TRPV2**	1.16	2.06E-06	7.37E-06
**RP11-8L18.3**	-5.68	2.06E-06	7.38E-06
**MRPL18**	0.81	2.06E-06	7.38E-06
**VPS45**	-0.49	2.06E-06	7.38E-06
**RGL3**	2.53	2.07E-06	7.39E-06
**NOTCH3**	1.00	2.07E-06	7.40E-06
**IL37**	-1.75	2.07E-06	7.42E-06
**TCEAL4**	0.99	2.08E-06	7.44E-06
**RAB32**	1.22	2.08E-06	7.44E-06
**RP11-321N4.4**	-5.20	2.08E-06	7.44E-06
**TESC**	2.71	2.08E-06	7.45E-06
**AC026202.3**	-3.40	2.09E-06	7.46E-06
**RP11-697N18.1**	-5.69	2.09E-06	7.46E-06
**FADS6**	1.37	2.09E-06	7.47E-06
**MIR1262**	-5.36	2.09E-06	7.47E-06
**HTT**	-0.64	2.09E-06	7.47E-06
**LPP-AS1**	-5.30	2.09E-06	7.48E-06
**SF3B1**	-0.48	2.10E-06	7.49E-06
**NAV3**	-0.96	2.10E-06	7.49E-06
**TEX101**	2.60	2.10E-06	7.49E-06
**PCSK6**	0.88	2.10E-06	7.50E-06
**RN7SKP227**	-5.76	2.11E-06	7.52E-06
**DENND1B**	-0.73	2.11E-06	7.53E-06
**ZKSCAN7P1**	-5.28	2.11E-06	7.54E-06
**LILRA5**	4.11	2.12E-06	7.58E-06
**RP11-521B24.3**	2.78	2.13E-06	7.59E-06
**KIAA0141**	0.54	2.13E-06	7.59E-06
**RP4-535B20.1**	-5.08	2.13E-06	7.59E-06
**RP11-304F15.7**	-4.21	2.13E-06	7.59E-06
**SLC2A9**	1.29	2.13E-06	7.60E-06

**15-Sep**	0.78	2.14E-06	7.63E-06
**RP11-173G21.1**	-5.62	2.14E-06	7.63E-06
**ZNF555**	-0.74	2.15E-06	7.66E-06
**PTK6**	0.84	2.15E-06	7.67E-06
**CTB-107G13.1**	-3.22	2.15E-06	7.67E-06
**SERPINA10**	-5.43	2.16E-06	7.68E-06
**SLC25A3P2**	-5.40	2.16E-06	7.70E-06
**RP11-231D20.2**	1.84	2.17E-06	7.72E-06
**RP11-1109F11.3**	-2.30	2.17E-06	7.75E-06
**DEPDC1**	-1.03	2.18E-06	7.76E-06
**RP11-524O24.2**	-5.25	2.18E-06	7.76E-06
**MIR3174**	-5.43	2.18E-06	7.78E-06
**SPPL2B**	1.32	2.19E-06	7.79E-06
**RAB3GAP1**	-0.48	2.19E-06	7.80E-06
**RP11-295P9.2**	-5.35	2.19E-06	7.80E-06
**RP1-319M7.2**	-5.33	2.19E-06	7.80E-06
**MT1X**	1.68	2.19E-06	7.80E-06
**MGAM**	-2.63	2.19E-06	7.80E-06
**LINC00696**	-4.14	2.20E-06	7.82E-06
**CTC-428H11.2**	-2.95	2.20E-06	7.85E-06
**AC136289.1**	-2.29	2.21E-06	7.87E-06
**PRSS16**	1.09	2.22E-06	7.89E-06
**RP11-477J21.2**	-5.43	2.22E-06	7.90E-06
**MYL12A**	0.76	2.22E-06	7.91E-06
**RP1-184J9.2**	-5.29	2.23E-06	7.93E-06
**MIRLET7C**	-5.21	2.23E-06	7.95E-06
**SSH2**	-0.70	2.24E-06	7.97E-06
**TYSND1**	0.67	2.24E-06	7.98E-06
**CHAC1**	3.01	2.25E-06	7.99E-06
**ZBTB20**	-1.19	2.25E-06	8.00E-06
**NRG1**	-1.11	2.25E-06	8.01E-06
**LAGE3P1**	-5.43	2.25E-06	8.02E-06
**RP11-182M20.2**	-5.39	2.26E-06	8.03E-06
**RAB2B**	-0.40	2.26E-06	8.04E-06
**RP11-455J20.3**	-3.41	2.27E-06	8.07E-06
**PARP12**	0.75	2.27E-06	8.07E-06
**AC011742.3**	-2.94	2.27E-06	8.08E-06
**ENTPD3**	1.03	2.29E-06	8.12E-06
**TSC22D3**	1.18	2.29E-06	8.14E-06
**PRRG1**	0.91	2.29E-06	8.15E-06
**RP11-642D21.2**	-5.25	2.30E-06	8.16E-06
**TPI1P4**	-5.69	2.30E-06	8.18E-06
**HDAC8**	0.48	2.30E-06	8.18E-06
**RN7SL57P**	-5.37	2.31E-06	8.19E-06
**NAA15**	-0.62	2.31E-06	8.19E-06

**RP11-498M15.1**	-3.34	2.31E-06	8.22E-06
**MYB**	-1.19	2.32E-06	8.23E-06
**AIMP1P1**	-4.49	2.32E-06	8.24E-06
**BIN3**	0.63	2.33E-06	8.25E-06
**PTGS1**	-0.69	2.33E-06	8.27E-06
**RFX7**	-0.59	2.34E-06	8.30E-06
**BCKDHA**	-3.27	2.34E-06	8.31E-06
**CCR8**	-3.35	2.34E-06	8.31E-06
**C17orf82**	4.33	2.34E-06	8.31E-06
**LITAF**	0.50	2.35E-06	8.34E-06
**ZNF639**	-0.54	2.36E-06	8.38E-06
**SNX3**	0.72	2.36E-06	8.38E-06
**GLYR1**	0.42	2.37E-06	8.39E-06
**TATDN2P3**	-2.70	2.38E-06	8.43E-06
**ADAT2**	-1.00	2.38E-06	8.44E-06
**RP11-648O15.1**	-5.29	2.38E-06	8.45E-06
**DLEU1**	-0.66	2.39E-06	8.46E-06
**STK36**	0.97	2.39E-06	8.48E-06
**RP11-422P24.9**	-5.44	2.40E-06	8.48E-06
**ANKRD36**	-1.57	2.40E-06	8.48E-06
**GTF3C2-AS1**	-2.69	2.40E-06	8.49E-06
**RNU4-24P**	-5.32	2.40E-06	8.50E-06
**RP11-490B18.6**	-2.12	2.40E-06	8.51E-06
**G43312**	-3.68	2.40E-06	8.51E-06
**RP11-298J23.5**	-5.30	2.41E-06	8.54E-06
**PABPC1P1**	-3.97	2.42E-06	8.56E-06
**HEATR9**	-5.48	2.42E-06	8.56E-06
**RP11-30L15.6**	-3.83	2.43E-06	8.60E-06
**LIMS2**	1.47	2.43E-06	8.60E-06
**PCBD2**	-0.58	2.44E-06	8.62E-06
**RP11-535A19.2**	-1.41	2.44E-06	8.62E-06
**TOX2**	2.38	2.44E-06	8.62E-06
**HCG18**	-0.84	2.44E-06	8.62E-06
**RP5-890E16.2**	-1.72	2.44E-06	8.63E-06
**RPL12P29**	-5.14	2.44E-06	8.63E-06
**FAM98A**	0.66	2.44E-06	8.63E-06
**NPM1P29**	-3.77	2.44E-06	8.63E-06
**RN7SL577P**	-5.41	2.44E-06	8.63E-06
**BBS5**	1.07	2.44E-06	8.63E-06
**GORAB**	0.59	2.45E-06	8.65E-06
**SCARNA20**	-2.71	2.45E-06	8.67E-06
**AP000962.1**	-3.68	2.46E-06	8.67E-06
**SMURF2**	-0.70	2.46E-06	8.69E-06
**GLTSCR1L**	-0.54	2.46E-06	8.70E-06
**TRIB1**	-0.57	2.47E-06	8.71E-06

**EMX2**	1.20	2.47E-06	8.74E-06
**PRH2**	-5.37	2.48E-06	8.74E-06
**LILRB4**	3.29	2.48E-06	8.77E-06
**DNMT3B**	0.80	2.49E-06	8.78E-06
**FAM111B**	-1.05	2.49E-06	8.78E-06
**LMF1**	0.81	2.49E-06	8.79E-06
**CTD-2215E18.2**	-5.51	2.49E-06	8.79E-06
**G40138**	-5.51	2.49E-06	8.80E-06
**PIGB**	-0.51	2.50E-06	8.81E-06
**DYNC2LI1**	-0.50	2.50E-06	8.83E-06
**AC007563.1**	-5.68	2.51E-06	8.84E-06
**SNORD11**	-5.20	2.51E-06	8.85E-06
**KLK4**	4.32	2.51E-06	8.85E-06
**CEP78**	-0.60	2.51E-06	8.85E-06
**RP11-571F15.2**	-5.18	2.51E-06	8.86E-06
**RPL36AL**	0.74	2.51E-06	8.86E-06
**AC093788.1**	-5.44	2.51E-06	8.87E-06
**RP1-7G5.5**	-2.61	2.52E-06	8.88E-06
**ARMC6**	0.80	2.52E-06	8.90E-06
**CYCSP2**	-5.35	2.52E-06	8.90E-06
**AC108066.1**	-5.60	2.53E-06	8.91E-06
**EIF3J**	0.67	2.53E-06	8.92E-06
**FAM160A2**	0.67	2.54E-06	8.94E-06
**GFAP**	2.97	2.54E-06	8.94E-06
**IFNLR1**	-0.89	2.54E-06	8.94E-06
**RP11-343N15.5**	-0.84	2.56E-06	9.03E-06
**G40298**	-1.68	2.57E-06	9.07E-06
**AC093690.1**	-2.63	2.58E-06	9.10E-06
**GSTM3**	-1.32	2.59E-06	9.12E-06
**CCDC138**	-0.59	2.59E-06	9.13E-06
**NDUFB4P2**	-5.54	2.60E-06	9.14E-06
**RP11-517M22.1**	-5.29	2.60E-06	9.15E-06
**CTC-463A16.1**	1.23	2.60E-06	9.16E-06
**PDE6C**	-2.12	2.60E-06	9.16E-06
**RP11-63K6.1**	-5.16	2.60E-06	9.16E-06
**BNIP3P40**	-4.92	2.61E-06	9.17E-06
**UBFD1**	0.38	2.61E-06	9.17E-06
**SNORA2B**	-5.34	2.61E-06	9.17E-06
**DDI2**	-0.57	2.61E-06	9.19E-06
**RPL17P11**	-5.76	2.62E-06	9.21E-06
**IL10RA**	1.66	2.63E-06	9.24E-06
**RP11-182I10.2**	-5.31	2.64E-06	9.29E-06
**ADORA1**	2.93	2.64E-06	9.29E-06
**TATDN1P1**	-3.88	2.64E-06	9.29E-06
**RNA5SP123**	-5.20	2.65E-06	9.31E-06

**LCP2**	1.80	2.65E-06	9.31E-06
**TREM1**	3.52	2.66E-06	9.34E-06
**FHOD3**	-1.13	2.66E-06	9.36E-06
**LIN9**	-0.72	2.67E-06	9.37E-06
**BACH1-AS1**	-3.03	2.67E-06	9.38E-06
**RGAG4**	1.56	2.67E-06	9.39E-06
**KLF4P1**	-5.28	2.68E-06	9.40E-06
**TRIO**	-0.46	2.68E-06	9.41E-06
**SMC5-AS1**	-2.08	2.69E-06	9.43E-06
**RP4-631H13.6**	-2.40	2.69E-06	9.44E-06
**NDUFS8**	0.61	2.69E-06	9.45E-06
**RP11-277I20.3**	-5.55	2.69E-06	9.46E-06
**KLHL24**	-0.78	2.70E-06	9.46E-06
**WDR89**	-0.78	2.70E-06	9.46E-06
**UBAP1L**	-1.64	2.70E-06	9.46E-06
**RNF152**	-1.02	2.71E-06	9.51E-06
**AC010900.2**	-5.31	2.72E-06	9.53E-06
**RP11-703I16.3**	2.31	2.72E-06	9.54E-06
**RP11-278A23.2**	-5.48	2.72E-06	9.54E-06
**BCS1L**	0.84	2.73E-06	9.57E-06
**AC073635.5**	-2.47	2.73E-06	9.58E-06
**GPR85**	2.14	2.73E-06	9.58E-06
**SCNN1G**	1.55	2.73E-06	9.58E-06
**WDR74**	0.68	2.74E-06	9.59E-06
**ZFP28**	-0.85	2.74E-06	9.61E-06
**CYCSP52**	-4.98	2.75E-06	9.63E-06
**ZNF629**	0.38	2.75E-06	9.64E-06
**PIGHP1**	-2.06	2.76E-06	9.66E-06
**PAK1**	0.62	2.76E-06	9.66E-06
**GDPD2**	-1.10	2.76E-06	9.68E-06
**PSMB7**	0.74	2.77E-06	9.69E-06
**TUBG1P**	-3.38	2.77E-06	9.69E-06
**RP1-315G1.1**	-5.13	2.77E-06	9.70E-06
**RP1-145M24.1**	-5.16	2.78E-06	9.72E-06
**ARF1P2**	-5.71	2.78E-06	9.73E-06
**C20orf24**	0.93	2.79E-06	9.77E-06
**KNOP1P2**	-4.05	2.79E-06	9.78E-06
**FGFR1OP2**	-0.50	2.80E-06	9.78E-06
**RP5-1092A11.2**	-5.41	2.80E-06	9.79E-06
**ZMIZ1**	0.84	2.80E-06	9.80E-06
**ARIH2OS**	1.13	2.81E-06	9.82E-06
**RP11-415J8.7**	-3.63	2.81E-06	9.83E-06
**AP001350.4**	-1.98	2.82E-06	9.85E-06
**EIF2S2**	0.67	2.82E-06	9.86E-06
**TAS2R50**	-5.34	2.83E-06	9.89E-06

**MCUR1**	-0.72	2.83E-06	9.90E-06
**HMGB3P9**	-5.13	2.83E-06	9.90E-06
**MIR181A1HG**	-3.47	2.85E-06	9.95E-06
**RP11-173A8.2**	-3.00	2.85E-06	9.96E-06
**GLT8D2**	1.87	2.85E-06	9.97E-06
**ZNF530**	-0.72	2.86E-06	9.98E-06
**PRRT4**	2.20	2.86E-06	9.98E-06
**RNU6ATAC7P**	-5.19	2.86E-06	1.00E-05
**G38112**	-2.83	2.86E-06	1.00E-05
**TJP1**	-0.60	2.86E-06	1.00E-05
**AC137590.1**	-5.31	2.87E-06	1.00E-05
**BRD3**	0.69	2.87E-06	1.00E-05
**P2RY13**	-1.55	2.87E-06	1.00E-05
**PRR12**	1.57	2.88E-06	1.01E-05
**DUSP12**	0.55	2.88E-06	1.01E-05
**AC064850.4**	-5.49	2.89E-06	1.01E-05
**CARS-AS1**	-3.79	2.89E-06	1.01E-05
**HSCB**	0.78	2.90E-06	1.01E-05
**RNF213**	-0.67	2.90E-06	1.01E-05
**LINC01003**	0.82	2.90E-06	1.01E-05
**BRPF1**	0.47	2.91E-06	1.01E-05
**RP11-1260E13.4**	1.36	2.91E-06	1.02E-05
**ZDHHC9**	-0.60	2.91E-06	1.02E-05
**SNORA59A**	-2.10	2.92E-06	1.02E-05
**XLOC_008072**	-0.97	2.92E-06	1.02E-05
**NADK**	0.69	2.92E-06	1.02E-05
**JAM3**	1.13	2.93E-06	1.02E-05
**RP11-152H18.4**	-4.29	2.93E-06	1.02E-05
**RNU6-33P**	-4.97	2.94E-06	1.02E-05
**PRELID3A**	2.06	2.95E-06	1.03E-05
**IVD**	1.33	2.95E-06	1.03E-05
**RP11-573M3.3**	-3.67	2.95E-06	1.03E-05
**FOLR1**	3.36	2.96E-06	1.03E-05
**SUSD5**	-1.41	2.96E-06	1.03E-05
**ANKRD18EP**	-1.06	2.96E-06	1.03E-05
**CCPG1**	-0.57	2.97E-06	1.03E-05
**RBM48**	-0.45	2.97E-06	1.03E-05
**DEF6**	0.69	2.97E-06	1.03E-05
**KIAA1143**	-0.62	2.98E-06	1.04E-05
**RPRD1A**	-0.64	2.98E-06	1.04E-05
**RFWD3**	-0.56	2.99E-06	1.04E-05
**RNF38**	-0.53	2.99E-06	1.04E-05
**RP11-456P18.2**	-3.04	2.99E-06	1.04E-05
**TOPORS**	-0.62	3.00E-06	1.04E-05
**LINC00649**	-1.60	3.00E-06	1.04E-05

**RP4-591B8.2**	-5.19	3.00E-06	1.04E-05
**SENP8**	-0.95	3.01E-06	1.05E-05
**RP11-182L21.5**	-3.20	3.01E-06	1.05E-05
**CSPG4**	1.38	3.01E-06	1.05E-05
**RPSAP16**	-3.74	3.02E-06	1.05E-05
**CTD-2308G16.1**	-5.12	3.02E-06	1.05E-05
**GLRX3P2**	-5.49	3.02E-06	1.05E-05
**HSPA12A**	-0.75	3.02E-06	1.05E-05
**REPS1**	-0.51	3.02E-06	1.05E-05
**ZNF541**	1.80	3.03E-06	1.05E-05
**FAM178B**	1.90	3.03E-06	1.05E-05
**ZNF532**	0.81	3.03E-06	1.05E-05
**E2F2**	-0.82	3.03E-06	1.05E-05
**RP11-192M23.1**	-2.58	3.04E-06	1.05E-05
**SNORA55**	-4.10	3.04E-06	1.06E-05
**RP11-131L12.3**	-1.89	3.04E-06	1.06E-05
**MYL6BP1**	-5.29	3.05E-06	1.06E-05
**PSG6**	-5.59	3.05E-06	1.06E-05
**VPS36**	-0.77	3.06E-06	1.06E-05
**KCTD9P4**	-5.33	3.06E-06	1.06E-05
**CTC-459F4.3**	-0.79	3.06E-06	1.06E-05
**SAP30**	0.79	3.07E-06	1.07E-05
**CTD-2516F10.4**	-4.45	3.07E-06	1.07E-05
**RNU6ATAC16P**	-5.40	3.07E-06	1.07E-05
**NLRP3P**	-2.49	3.08E-06	1.07E-05
**RP3-399L15.3**	-1.82	3.08E-06	1.07E-05
**HMGB2**	0.89	3.09E-06	1.07E-05
**TTC25**	1.83	3.09E-06	1.07E-05
**RP11-619J20.1**	-3.91	3.09E-06	1.07E-05
**AMPH**	1.95	3.10E-06	1.08E-05
**TLK1**	-0.66	3.11E-06	1.08E-05
**RN7SKP64**	-5.23	3.11E-06	1.08E-05
**G31689**	-2.14	3.11E-06	1.08E-05
**RP11-452L6.1**	-1.01	3.11E-06	1.08E-05
**GALNT14**	1.42	3.11E-06	1.08E-05
**MTND4P14**	-5.62	3.11E-06	1.08E-05
**RP11-430L17.1**	-4.18	3.12E-06	1.08E-05
**GPR63**	-1.94	3.12E-06	1.08E-05
**RP11-474I11.7**	-4.21	3.12E-06	1.08E-05
**RP11-723D22.2**	-5.19	3.13E-06	1.08E-05
**RP11-722E23.2**	-1.02	3.14E-06	1.09E-05
**SMYD3**	1.20	3.14E-06	1.09E-05
**RP11-191A15.2**	-5.28	3.14E-06	1.09E-05
**XLOC_010695**	-3.28	3.15E-06	1.09E-05
**RP11-215P8.3**	-1.84	3.15E-06	1.09E-05

**PDCD4-AS1**	1.67	3.17E-06	1.10E-05
**AC115115.3**	-5.10	3.17E-06	1.10E-05
**SEC14L5**	-1.09	3.17E-06	1.10E-05
**TPGS2**	0.50	3.17E-06	1.10E-05
**CDY4P**	-5.57	3.18E-06	1.10E-05
**CELF2-AS2**	-5.27	3.19E-06	1.10E-05
**ABCA7**	0.97	3.19E-06	1.10E-05
**RWDD1**	0.55	3.20E-06	1.11E-05
**HBEGF**	1.24	3.20E-06	1.11E-05
**POMP**	0.76	3.20E-06	1.11E-05
**KB-1460A1.5**	-1.20	3.21E-06	1.11E-05
**CCDC159**	1.10	3.21E-06	1.11E-05
**RP11-354P11.3**	-3.83	3.21E-06	1.11E-05
**KB-1410C5.2**	-5.28	3.22E-06	1.11E-05
**PCDHGC4**	-1.87	3.22E-06	1.11E-05
**AC022173.2**	-5.23	3.22E-06	1.11E-05
**CDSN**	-2.38	3.22E-06	1.11E-05
**APOBEC3G**	1.55	3.24E-06	1.12E-05
**STK26**	-1.08	3.24E-06	1.12E-05
**MTND2P11**	-5.21	3.25E-06	1.12E-05
**BCL9L**	1.18	3.25E-06	1.12E-05
**RP11-74D7.1**	-5.42	3.25E-06	1.12E-05
**CSF2RB**	1.09	3.26E-06	1.12E-05
**SOS1-IT1**	-2.12	3.26E-06	1.12E-05
**PARVB**	1.35	3.26E-06	1.12E-05
**U2SURP**	-0.63	3.26E-06	1.13E-05
**TRIP4**	0.52	3.27E-06	1.13E-05
**NOL4L**	1.09	3.27E-06	1.13E-05
**PLA2G2A**	3.54	3.27E-06	1.13E-05
**CTD-2647L4.1**	-5.34	3.28E-06	1.13E-05
**C9orf41-AS1**	-2.15	3.28E-06	1.13E-05
**RN7SKP35**	-5.39	3.29E-06	1.13E-05
**TENM2**	0.81	3.29E-06	1.13E-05
**CD34**	1.51	3.30E-06	1.14E-05
**PRR34**	-1.44	3.31E-06	1.14E-05
**HNRNPA1P59**	-5.13	3.32E-06	1.15E-05
**RPL7AP2**	-4.26	3.33E-06	1.15E-05
**STAT5B**	0.37	3.33E-06	1.15E-05
**MIR566**	-5.17	3.34E-06	1.15E-05
**RP11-793A3.2**	-2.50	3.35E-06	1.15E-05
**CCND2**	-0.80	3.37E-06	1.16E-05
**AL139080.1**	-5.28	3.38E-06	1.16E-05
**UBE2E2**	0.94	3.38E-06	1.16E-05
**AC004837.5**	-3.22	3.39E-06	1.17E-05
**CTD-2213F21.2**	-2.07	3.40E-06	1.17E-05

**ZNRF3-AS1**	-4.06	3.40E-06	1.17E-05
**DMKN**	0.98	3.41E-06	1.17E-05
**SERPIND1**	-2.53	3.42E-06	1.18E-05
**COX7A2L**	0.67	3.42E-06	1.18E-05
**RP11-571F15.3**	-5.21	3.43E-06	1.18E-05
**TNS4**	1.16	3.44E-06	1.18E-05
**CTD-2385L22.2**	-2.61	3.44E-06	1.19E-05
**HLA-C**	1.62	3.44E-06	1.19E-05
**RP11-344A16.2**	-5.26	3.46E-06	1.19E-05
**RP11-73K9.3**	-2.00	3.46E-06	1.19E-05
**RP11-658F2.8**	-0.93	3.46E-06	1.19E-05
**C1orf21**	-0.54	3.46E-06	1.19E-05
**RASL10B**	2.60	3.46E-06	1.19E-05
**AC109826.1**	4.88	3.47E-06	1.19E-05
**RP5-981O7.2**	-3.02	3.48E-06	1.20E-05
**PKD1P6**	0.97	3.48E-06	1.20E-05
**XLOC_011805**	-5.13	3.50E-06	1.20E-05
**VCP**	0.51	3.50E-06	1.20E-05
**AC079949.1**	4.15	3.50E-06	1.20E-05
**CIPC**	-0.56	3.51E-06	1.21E-05
**RHCE**	1.51	3.52E-06	1.21E-05
**PIEZO1**	0.84	3.53E-06	1.21E-05
**MEP1B**	-3.98	3.53E-06	1.21E-05
**CILP2**	3.38	3.54E-06	1.22E-05
**C3orf18**	1.56	3.55E-06	1.22E-05
**MYLPF**	2.25	3.55E-06	1.22E-05
**PER1**	1.91	3.55E-06	1.22E-05
**XLOC_007999**	-5.11	3.55E-06	1.22E-05
**NCK1-AS1**	1.13	3.56E-06	1.22E-05
**RP11-348N5.9**	-2.67	3.56E-06	1.22E-05
**RP11-408O19.5**	-2.45	3.57E-06	1.22E-05
**CTD-3247F14.2**	-1.72	3.57E-06	1.23E-05
**HNRNPA1P35**	-2.88	3.57E-06	1.23E-05
**NPL**	1.36	3.58E-06	1.23E-05
**REV1**	-0.47	3.58E-06	1.23E-05
**RPL5P23**	-3.50	3.58E-06	1.23E-05
**RP4-597J3.1**	-5.24	3.59E-06	1.23E-05
**RP1-60N8.1**	-5.33	3.59E-06	1.23E-05
**MAGT1**	-0.62	3.59E-06	1.23E-05
**RP11-78I14.1**	-5.03	3.61E-06	1.24E-05
**HACD3**	-0.60	3.61E-06	1.24E-05
**RP5-875H18.10**	-5.55	3.62E-06	1.24E-05
**PSMD2**	0.60	3.62E-06	1.24E-05
**SRCAP**	-1.03	3.63E-06	1.25E-05
**INTS8**	-0.58	3.63E-06	1.25E-05

**SYNJ2BP**	-0.61	3.63E-06	1.25E-05
**KRT16P1**	4.85	3.64E-06	1.25E-05
**RP11-159D12.2**	-1.66	3.64E-06	1.25E-05
**GNA14**	1.48	3.65E-06	1.25E-05
**SFSWAP**	0.58	3.66E-06	1.25E-05
**RNU6-140P**	4.15	3.67E-06	1.26E-05
**CTD-3193O13.12**	-2.78	3.68E-06	1.26E-05
**FZD9**	1.84	3.70E-06	1.27E-05
**Metazoa_SRP**	-2.41	3.70E-06	1.27E-05
**IFNGR1**	0.69	3.70E-06	1.27E-05
**MLIP-AS1**	-3.19	3.72E-06	1.28E-05
**C2CD3**	-0.62	3.73E-06	1.28E-05
**RP5-1142A6.8**	-2.96	3.73E-06	1.28E-05
**G5143**	-2.63	3.74E-06	1.28E-05
**PEX26**	-0.45	3.76E-06	1.29E-05
**RP11-95J11.1**	-5.28	3.76E-06	1.29E-05
**SNORA59B**	-5.12	3.76E-06	1.29E-05
**CTD-2152M20.2**	-2.46	3.76E-06	1.29E-05
**RBBP6**	0.61	3.76E-06	1.29E-05
**RP3-403L10.3**	-4.97	3.77E-06	1.29E-05
**S100A12**	5.53	3.78E-06	1.29E-05
**PRG4**	-3.38	3.78E-06	1.29E-05
**CTC-329H14.1**	-5.15	3.79E-06	1.29E-05
**UBA52P8**	-2.81	3.79E-06	1.30E-05
**DPYS**	4.98	3.80E-06	1.30E-05
**MORN4**	0.97	3.80E-06	1.30E-05
**RP11-466F5.4**	-5.31	3.81E-06	1.30E-05
**G12163**	-1.84	3.82E-06	1.31E-05
**IL20**	3.67	3.83E-06	1.31E-05
**RP11-211G3.2**	-1.31	3.84E-06	1.31E-05
**THRB-IT1**	-2.63	3.85E-06	1.31E-05
**CXCL13**	8.34	3.86E-06	1.32E-05
**RP11-691H4.4**	-5.53	3.88E-06	1.32E-05
**RP3-329E20.2**	-5.19	3.88E-06	1.33E-05
**HTR2A**	2.78	3.88E-06	1.33E-05
**RP11-288H12.3**	-1.30	3.89E-06	1.33E-05
**CTC-360J11.4**	-5.13	3.89E-06	1.33E-05
**YBX1P3**	-5.22	3.90E-06	1.33E-05
**RP11-390B4.3**	-4.23	3.90E-06	1.33E-05
**PTCHD1**	-3.00	3.90E-06	1.33E-05
**RP11-560O20.1**	-5.32	3.91E-06	1.33E-05
**SLC5A2**	-2.65	3.91E-06	1.33E-05
**SNX12**	0.56	3.91E-06	1.33E-05
**RP11-863H1.1**	-2.76	3.92E-06	1.34E-05
**PHLDB1**	0.97	3.92E-06	1.34E-05

**TAS2R12**	-5.32	3.93E-06	1.34E-05
**ERGIC1**	0.46	3.93E-06	1.34E-05
**RP11-383G10.3**	-5.50	3.93E-06	1.34E-05
**RP11-227G15.11**	-2.28	3.94E-06	1.34E-05
**NPM1P25**	1.41	3.94E-06	1.34E-05
**XLOC_011429**	-5.23	3.94E-06	1.34E-05
**KIAA0226L**	2.28	3.94E-06	1.34E-05
**MRPL20**	0.54	3.95E-06	1.35E-05
**ARHGEF3**	0.55	3.95E-06	1.35E-05
**CARS**	0.55	3.96E-06	1.35E-05
**RP11-513O13.1**	3.15	3.97E-06	1.35E-05
**VN1R42P**	-4.77	3.98E-06	1.36E-05
**STT3A-AS1**	-5.05	3.98E-06	1.36E-05
**RP11-191A15.1**	-5.44	3.99E-06	1.36E-05
**ADRA2B**	2.08	3.99E-06	1.36E-05
**SORL1**	-1.03	4.00E-06	1.36E-05
**RP11-556O9.4**	-5.25	4.00E-06	1.36E-05
**RP11-466A19.5**	-5.30	4.02E-06	1.37E-05
**GHRLOS**	-2.27	4.03E-06	1.37E-05
**WNT5B**	1.23	4.03E-06	1.37E-05
**ATMIN**	-0.66	4.03E-06	1.37E-05
**RP11-635N19.3**	-1.76	4.03E-06	1.37E-05
**TNFRSF12A**	1.75	4.03E-06	1.37E-05
**CTD-2315M5.2**	-5.34	4.04E-06	1.38E-05
**AARS**	0.74	4.05E-06	1.38E-05
**NOMO2**	0.63	4.05E-06	1.38E-05
**RP1-118J21.5**	-4.09	4.05E-06	1.38E-05
**TMEM70**	0.65	4.06E-06	1.38E-05
**G16985**	-3.79	4.07E-06	1.38E-05
**SNORD58**	-5.01	4.07E-06	1.38E-05
**STOX2**	-0.76	4.08E-06	1.39E-05
**C1orf162**	1.50	4.08E-06	1.39E-05
**UFSP2**	-0.55	4.09E-06	1.39E-05
**RRAS2**	0.59	4.09E-06	1.39E-05
**GFM1**	-0.52	4.10E-06	1.40E-05
**FANCG**	0.81	4.11E-06	1.40E-05
**AP000925.2**	-4.39	4.12E-06	1.40E-05
**SNORD111B**	-5.16	4.12E-06	1.40E-05
**LILRA1**	3.55	4.12E-06	1.40E-05
**RP11-177A2.5**	-5.29	4.14E-06	1.41E-05
**CTD-2013N24.2**	-1.59	4.14E-06	1.41E-05
**PCK2**	0.75	4.15E-06	1.41E-05
**LVRN**	-2.16	4.15E-06	1.41E-05
**CTD-3154N5.1**	-5.20	4.15E-06	1.41E-05
**MKNK1-AS1**	-2.26	4.16E-06	1.41E-05

**GPR52**	-5.25	4.17E-06	1.42E-05
**JAM2**	1.17	4.18E-06	1.42E-05
**EDEM3**	-0.57	4.18E-06	1.42E-05
**RP11-179A7.2**	-5.30	4.18E-06	1.42E-05
**AIG1**	0.54	4.19E-06	1.42E-05
**CASP7**	0.76	4.19E-06	1.42E-05
**MICB**	1.90	4.21E-06	1.43E-05
**BMS1P4**	-1.61	4.21E-06	1.43E-05
**RP5-890E16.5**	-1.63	4.21E-06	1.43E-05
**TBC1D7**	0.50	4.21E-06	1.43E-05
**KLHL6**	1.77	4.21E-06	1.43E-05
**RP5-1065J22.4**	-3.71	4.21E-06	1.43E-05
**PARD3**	-0.86	4.22E-06	1.43E-05
**FOLR2**	2.80	4.22E-06	1.43E-05
**RP11-12A16.3**	-5.46	4.23E-06	1.44E-05
**RP3-425P12.4**	-2.79	4.23E-06	1.44E-05
**RP11-83B20.2**	-5.19	4.24E-06	1.44E-05
**DSC3**	-0.84	4.24E-06	1.44E-05
**RPS15AP10**	-1.94	4.25E-06	1.44E-05
**BBS7**	-0.77	4.25E-06	1.44E-05
**RP11-1102P22.2**	-4.45	4.25E-06	1.44E-05
**DNAJC25**	0.65	4.26E-06	1.44E-05
**SCLT1**	-0.58	4.27E-06	1.45E-05
**HOXC11**	2.38	4.28E-06	1.45E-05
**LL22NC03-N14H11.1**	6.33	4.30E-06	1.46E-05
**RNPEP**	0.52	4.30E-06	1.46E-05
**MB21D2**	-0.77	4.31E-06	1.46E-05
**RP11-81A1.4**	-5.53	4.32E-06	1.46E-05
**EPHA4**	-0.66	4.33E-06	1.46E-05
**RP11-83J16.1**	-3.47	4.33E-06	1.46E-05
**LARP4P**	-2.54	4.33E-06	1.47E-05
**SMAD1-AS1**	-5.31	4.36E-06	1.48E-05
**SMARCA5**	-0.53	4.36E-06	1.48E-05
**RNU6-676P**	-5.40	4.36E-06	1.48E-05
**RP11-84A19.4**	-4.02	4.37E-06	1.48E-05
**RP11-876N24.5**	-1.34	4.37E-06	1.48E-05
**RP11-77P6.2**	-1.24	4.38E-06	1.48E-05
**CD37**	1.70	4.38E-06	1.48E-05
**GIMAP2**	1.47	4.39E-06	1.48E-05
**DBP**	1.43	4.39E-06	1.49E-05
**RBBP8NL**	1.18	4.41E-06	1.49E-05
**G31075**	-3.30	4.41E-06	1.49E-05
**VWA2**	-1.02	4.41E-06	1.49E-05
**RP11-1060J15.3**	-5.39	4.42E-06	1.49E-05
**ASXL1**	0.51	4.42E-06	1.50E-05

**AL596137.1**	-5.33	4.43E-06	1.50E-05
**RP11-1299A16.3**	-1.32	4.46E-06	1.51E-05
**AC114812.10**	-4.71	4.47E-06	1.51E-05
**SHC4**	2.10	4.47E-06	1.51E-05
**XLOC_005024**	-4.56	4.50E-06	1.52E-05
**RNF141**	-0.71	4.51E-06	1.52E-05
**CTD-2066L21.3**	-3.24	4.52E-06	1.53E-05
**MRRF**	-0.42	4.54E-06	1.53E-05
**PLCD4**	1.12	4.57E-06	1.54E-05
**RP11-573N10.1**	-5.20	4.58E-06	1.55E-05
**RP11-494M8.1**	-5.49	4.58E-06	1.55E-05
**RP11-203H19.2**	-5.22	4.59E-06	1.55E-05
**CYP27A1**	1.42	4.59E-06	1.55E-05
**ITGA10**	2.14	4.60E-06	1.55E-05
**RP11-732A21.3**	2.11	4.60E-06	1.55E-05
**RP3-467L1.6**	-2.51	4.60E-06	1.55E-05
**IFI27**	3.18	4.61E-06	1.55E-05
**RP11-813F20.4**	-5.00	4.61E-06	1.56E-05
**CDC42P5**	-5.21	4.63E-06	1.56E-05
**RIMKLBP1**	-4.83	4.64E-06	1.56E-05
**MCCC1**	-0.75	4.64E-06	1.56E-05
**SPTY2D1-AS1**	-1.28	4.64E-06	1.56E-05
**PINX1**	0.88	4.64E-06	1.56E-05
**OPRL1**	2.01	4.64E-06	1.56E-05
**BMPR1APS1**	-5.03	4.64E-06	1.56E-05
**PTPRA**	0.38	4.65E-06	1.57E-05
**RP11-317P15.4**	3.91	4.66E-06	1.57E-05
**TMEM127**	0.57	4.66E-06	1.57E-05
**MIR3676**	-5.02	4.67E-06	1.57E-05
**MIR3175**	-5.04	4.67E-06	1.57E-05
**PPP1R1A**	4.66	4.69E-06	1.58E-05
**RP5-1057I20.5**	-5.40	4.69E-06	1.58E-05
**AF212831.2**	-5.97	4.71E-06	1.59E-05
**DLGAP4-AS1**	-2.05	4.71E-06	1.59E-05
**RPL13P6**	-4.98	4.72E-06	1.59E-05
**TMIGD3**	3.20	4.73E-06	1.59E-05
**RP11-162A12.3**	-2.34	4.73E-06	1.59E-05
**RP1-261D10.2**	-1.27	4.76E-06	1.60E-05
**SPRR2G**	3.37	4.77E-06	1.61E-05
**AC006159.5**	-4.24	4.78E-06	1.61E-05
**WNT4**	-1.40	4.78E-06	1.61E-05
**RP4-614O4.11**	-2.03	4.79E-06	1.61E-05
**ZFYVE1**	0.45	4.80E-06	1.61E-05
**RHOBTB3**	-1.10	4.80E-06	1.61E-05
**XK**	-1.23	4.80E-06	1.61E-05

**CTA-292E10.8**	-2.50	4.80E-06	1.62E-05
**HAUS8**	0.93	4.81E-06	1.62E-05
**GYG1**	0.86	4.81E-06	1.62E-05
**FBXL16**	1.12	4.82E-06	1.62E-05
**RP11-702F3.1**	-4.98	4.83E-06	1.62E-05
**RP11-777F6.3**	-2.48	4.83E-06	1.62E-05
**AOC1**	2.25	4.84E-06	1.63E-05
**TMEM74**	-1.70	4.84E-06	1.63E-05
**G5142**	-1.67	4.85E-06	1.63E-05
**MEG8**	-2.81	4.85E-06	1.63E-05
**CTB-176F20.3**	-1.77	4.87E-06	1.64E-05
**RP11-324I22.4**	-1.32	4.87E-06	1.64E-05
**ERCC3**	0.33	4.89E-06	1.64E-05
**FGF14-IT1**	-4.97	4.90E-06	1.65E-05
**RP11-203L2.3**	-5.01	4.91E-06	1.65E-05
**MMP25-AS1**	1.94	4.92E-06	1.65E-05
**RP11-396C23.4**	-2.96	4.92E-06	1.65E-05
**AF064858.11**	-2.47	4.93E-06	1.65E-05
**NRP1**	1.40	4.94E-06	1.66E-05
**CLTCL1**	0.81	4.95E-06	1.66E-05
**RN7SL413P**	-4.87	4.95E-06	1.66E-05
**RP11-35J10.4**	-5.67	4.95E-06	1.66E-05
**DNAJC30**	0.72	4.98E-06	1.67E-05
**CLDN2**	-2.07	4.99E-06	1.67E-05
**RP11-367J11.2**	-2.46	4.99E-06	1.67E-05
**ZEB2-AS1**	-3.20	4.99E-06	1.67E-05
**SDS**	2.91	5.00E-06	1.68E-05
**LCN2**	2.80	5.01E-06	1.68E-05
**SNRPA1**	0.69	5.03E-06	1.69E-05
**TMEM200A**	1.83	5.04E-06	1.69E-05
**AC005351.1**	-5.08	5.05E-06	1.69E-05
**RP11-665C16.5**	-5.41	5.05E-06	1.69E-05
**MXRA7**	1.24	5.05E-06	1.69E-05
**RP11-77K12.3**	-4.96	5.06E-06	1.70E-05
**CTD-2547G23.4**	-1.43	5.06E-06	1.70E-05
**RPL9P30**	-5.22	5.07E-06	1.70E-05
**CTD-2542L18.1**	-5.14	5.09E-06	1.71E-05
**MED11**	0.68	5.10E-06	1.71E-05
**RP1-97G4.1**	-5.33	5.10E-06	1.71E-05
**XLOC_013542**	5.46	5.11E-06	1.71E-05
**PPP4R3A**	-0.48	5.11E-06	1.71E-05
**MFN1**	-0.61	5.11E-06	1.71E-05
**LRRC37A**	-1.57	5.12E-06	1.71E-05
**XLOC_005369**	-2.99	5.12E-06	1.72E-05
**VCL**	0.55	5.12E-06	1.72E-05

**ACR**	3.59	5.12E-06	1.72E-05
**AOAH**	2.19	5.14E-06	1.72E-05
**IGHGP**	6.34	5.14E-06	1.72E-05
**KCTD6**	-0.63	5.15E-06	1.72E-05
**GLTSCR1-AS1**	-5.18	5.15E-06	1.72E-05
**RNU6-83P**	-5.33	5.16E-06	1.73E-05
**RPL37P2**	3.10	5.17E-06	1.73E-05
**RP11-268P4.6**	-5.07	5.18E-06	1.73E-05
**TFG**	0.41	5.18E-06	1.73E-05
**SH3BP5L**	0.75	5.19E-06	1.74E-05
**PRCP**	0.66	5.19E-06	1.74E-05
**RP1-161N10.1**	-5.06	5.19E-06	1.74E-05
**TGS1**	-0.56	5.20E-06	1.74E-05
**SLC35A1**	-0.66	5.20E-06	1.74E-05
**TFDP1**	0.49	5.21E-06	1.74E-05
**XLOC_004122**	-1.26	5.21E-06	1.74E-05
**TPI1P3**	-5.09	5.21E-06	1.74E-05
**SPATA17**	-1.52	5.22E-06	1.75E-05
**RP4-569M23.5**	-4.93	5.23E-06	1.75E-05
**GRAMD2**	0.82	5.23E-06	1.75E-05
**RN7SL653P**	-5.18	5.23E-06	1.75E-05
**XLOC_005377**	-5.16	5.25E-06	1.75E-05
**RP11-863P13.6**	-2.27	5.26E-06	1.76E-05
**PNPT1P1**	-5.07	5.27E-06	1.76E-05
**ACKR4**	1.40	5.28E-06	1.76E-05
**WASF1**	0.97	5.29E-06	1.77E-05
**RP11-334C17.3**	1.25	5.29E-06	1.77E-05
**C12orf40**	-5.34	5.30E-06	1.77E-05
**EPS8L1**	0.98	5.30E-06	1.77E-05
**RP11-2N1.1**	-5.46	5.31E-06	1.77E-05
**RP11-546M21.6**	-4.98	5.31E-06	1.77E-05
**CEP85L**	-1.00	5.31E-06	1.77E-05
**XLOC_003809**	3.45	5.33E-06	1.78E-05
**MTND4P32**	-5.27	5.34E-06	1.78E-05
**SNORA70G**	-5.33	5.34E-06	1.78E-05
**PLXNB1**	1.00	5.36E-06	1.79E-05
**DUXAP9**	4.19	5.38E-06	1.79E-05
**SDK1**	1.99	5.38E-06	1.79E-05
**VEZT**	-0.44	5.38E-06	1.80E-05
**GLB1**	0.67	5.40E-06	1.80E-05
**ARHGEF16**	1.18	5.41E-06	1.80E-05
**RN7SL333P**	-2.82	5.41E-06	1.80E-05
**AP000889.2**	-5.45	5.42E-06	1.80E-05
**MTCO3P12**	-1.24	5.43E-06	1.81E-05
**ARHGEF33**	-1.65	5.43E-06	1.81E-05

**ORMDL3**	0.56	5.43E-06	1.81E-05
**FRZB**	1.38	5.43E-06	1.81E-05
**RP11-327O17.2**	-4.95	5.43E-06	1.81E-05
**GALNS**	0.91	5.44E-06	1.81E-05
**HSD11B1L**	0.98	5.44E-06	1.81E-05
**RP11-481C4.1**	-5.38	5.46E-06	1.82E-05
**RP11-342K6.3**	-4.81	5.46E-06	1.82E-05
**SERTAD4**	0.88	5.47E-06	1.82E-05
**DLGAP3**	2.18	5.47E-06	1.82E-05
**ZBED5**	-0.55	5.50E-06	1.83E-05
**RNU6-402P**	-4.94	5.51E-06	1.83E-05
**G19261**	2.48	5.51E-06	1.83E-05
**COX15**	-0.45	5.52E-06	1.84E-05
**PRPF8**	-0.48	5.52E-06	1.84E-05
**GCSAML**	-2.56	5.53E-06	1.84E-05
**LINC01397**	4.50	5.53E-06	1.84E-05
**ACRC**	-1.51	5.54E-06	1.84E-05
**JARID2-AS1**	-2.30	5.54E-06	1.84E-05
**OR7E100P**	-5.21	5.55E-06	1.84E-05
**G15458**	-5.14	5.55E-06	1.85E-05
**ARVCF**	1.18	5.56E-06	1.85E-05
**SLC30A5**	-0.58	5.56E-06	1.85E-05
**RP11-258F1.2**	-4.05	5.59E-06	1.86E-05
**H2AFY**	0.45	5.59E-06	1.86E-05
**TXNDC11**	0.51	5.62E-06	1.87E-05
**GFRA1**	-2.00	5.62E-06	1.87E-05
**RPL6P27**	0.95	5.62E-06	1.87E-05
**LCE3C**	6.61	5.63E-06	1.87E-05
**HSPE1P27**	-5.14	5.64E-06	1.87E-05
**SLCO1C1**	3.02	5.64E-06	1.87E-05
**PLA2G4E-AS1**	-1.95	5.66E-06	1.88E-05
**ROR2**	1.96	5.67E-06	1.88E-05
**ATP10D**	-0.77	5.69E-06	1.89E-05
**RP11-115C21.4**	-5.10	5.69E-06	1.89E-05
**AC073069.2**	-5.11	5.70E-06	1.89E-05
**UGT1A2P**	-5.33	5.70E-06	1.89E-05
**MED12L**	-1.57	5.70E-06	1.89E-05
**XLOC_002925**	-4.17	5.72E-06	1.90E-05
**KRT16P2**	3.46	5.73E-06	1.90E-05
**IL24**	2.93	5.74E-06	1.90E-05
**CTC-303L1.2**	-4.15	5.74E-06	1.90E-05
**KCNA5**	2.97	5.75E-06	1.91E-05
**RP11-270F18.2**	-5.01	5.75E-06	1.91E-05
**CDX1**	4.74	5.76E-06	1.91E-05
**RP11-16L14.2**	-4.79	5.77E-06	1.91E-05

**RP11-413E1.4**	-3.40	5.77E-06	1.91E-05
**HHIPL1**	1.90	5.78E-06	1.92E-05
**CTA-299D3.8**	-5.58	5.78E-06	1.92E-05
**RASL11B**	1.45	5.79E-06	1.92E-05
**CCNYL1**	0.72	5.80E-06	1.92E-05
**RP11-15B17.4**	-4.22	5.80E-06	1.92E-05
**AL139333.1**	-4.83	5.80E-06	1.92E-05
**SLC8A2**	1.92	5.80E-06	1.92E-05
**NCAM1**	-1.77	5.81E-06	1.92E-05
**UBN1**	0.43	5.82E-06	1.93E-05
**FGF12**	2.60	5.82E-06	1.93E-05
**GS1-124K5.3**	-1.50	5.82E-06	1.93E-05
**NPR1**	1.57	5.82E-06	1.93E-05
**AC009495.3**	-3.21	5.84E-06	1.94E-05
**RP5-1049G16.4**	-5.02	5.85E-06	1.94E-05
**SNPH**	1.16	5.86E-06	1.94E-05
**NEK5**	-1.26	5.86E-06	1.94E-05
**AC106873.4**	-5.20	5.86E-06	1.94E-05
**RP5-979D14.1**	-5.32	5.87E-06	1.94E-05
**FAM133DP**	2.01	5.88E-06	1.94E-05
**MCM3AP-AS1**	-1.14	5.90E-06	1.95E-05
**RP11-762H8.1**	-4.91	5.90E-06	1.95E-05
**SSR3**	-0.63	5.90E-06	1.95E-05
**RP11-46I1.1**	-5.45	5.91E-06	1.95E-05
**AC013410.1**	-5.44	5.91E-06	1.95E-05
**RP11-752G15.8**	-4.35	5.92E-06	1.96E-05
**RPLP0**	0.77	5.93E-06	1.96E-05
**DPH2**	0.70	5.94E-06	1.96E-05
**RP11-493P1.2**	-3.59	5.94E-06	1.96E-05
**MIR1537**	-5.19	5.95E-06	1.97E-05
**OTX1**	1.22	5.95E-06	1.97E-05
**ITPK1-AS1**	-5.04	5.96E-06	1.97E-05
**RP11-51J9.5**	2.05	5.97E-06	1.97E-05
**CROCCP3**	-1.23	5.97E-06	1.97E-05
**SH3BP2**	-0.88	5.98E-06	1.97E-05
**RP11-71L14.3**	-1.63	5.98E-06	1.97E-05
**RP11-95P9.1**	-5.33	5.98E-06	1.98E-05
**POLR3G**	1.15	5.98E-06	1.98E-05
**RPS4XP17**	-3.27	5.99E-06	1.98E-05
**RAD21**	-0.45	6.00E-06	1.98E-05
**MEF2A**	-0.68	6.04E-06	1.99E-05
**RP11-111F5.8**	-5.71	6.04E-06	1.99E-05
**SFTPC**	-2.13	6.04E-06	1.99E-05
**ME1**	-1.18	6.04E-06	2.00E-05
**TMPRSS11A**	2.99	6.05E-06	2.00E-05

**RP11-458F8.1**	-2.07	6.05E-06	2.00E-05
**RP4-672N11.1**	-5.28	6.07E-06	2.00E-05
**RP11-12G12.7**	1.43	6.08E-06	2.01E-05
**ELOVL3**	-4.37	6.08E-06	2.01E-05
**VSIG4**	2.52	6.08E-06	2.01E-05
**CTC-550B14.7**	-1.34	6.10E-06	2.01E-05
**SHC3**	-1.60	6.11E-06	2.02E-05
**XLOC_009475**	-2.41	6.13E-06	2.02E-05
**AC068641.1**	-5.12	6.14E-06	2.02E-05
**VWFP1**	4.85	6.14E-06	2.03E-05
**NR1D1**	2.11	6.18E-06	2.04E-05
**CTC-487M23.6**	-1.51	6.22E-06	2.05E-05
**GPR161**	0.89	6.22E-06	2.05E-05
**RNF123**	0.65	6.23E-06	2.05E-05
**PAX6**	-0.96	6.24E-06	2.06E-05
**AB015752.3**	-4.91	6.25E-06	2.06E-05
**RP1-178F10.1**	-2.22	6.26E-06	2.06E-05
**GAS2L1**	0.71	6.26E-06	2.06E-05
**NCBP2**	0.48	6.27E-06	2.06E-05
**GGCX**	-0.49	6.27E-06	2.07E-05
**RP11-14I17.3**	-5.41	6.28E-06	2.07E-05
**CTBP1**	0.34	6.28E-06	2.07E-05
**MIR3140**	-5.28	6.28E-06	2.07E-05
**RNA5SP295**	-5.43	6.29E-06	2.07E-05
**TP53RK**	-0.70	6.30E-06	2.07E-05
**CAPN10-AS1**	-1.51	6.30E-06	2.07E-05
**KB-7G2.9**	-5.29	6.30E-06	2.08E-05
**RP11-554D20.1**	-5.17	6.31E-06	2.08E-05
**KB-1562D12.3**	-3.88	6.31E-06	2.08E-05
**RNU6-37P**	3.08	6.32E-06	2.08E-05
**TP53BP1**	-0.51	6.33E-06	2.08E-05
**RP11-26L20.3**	-5.32	6.33E-06	2.08E-05
**RSPO3**	2.94	6.35E-06	2.09E-05
**TDG**	0.45	6.35E-06	2.09E-05
**NDUFA3**	0.61	6.37E-06	2.09E-05
**STAR**	-0.89	6.38E-06	2.10E-05
**RP11-1055B8.1**	2.01	6.39E-06	2.10E-05
**C1orf159**	1.27	6.42E-06	2.11E-05
**AC073569.1**	-5.26	6.44E-06	2.12E-05
**RP11-661O13.1**	-2.54	6.44E-06	2.12E-05
**GNB5**	0.92	6.44E-06	2.12E-05
**RP1-56K13.5**	-4.52	6.46E-06	2.12E-05
**DNAJB3**	-4.30	6.46E-06	2.12E-05
**SELP**	1.88	6.47E-06	2.13E-05
**SLTM**	0.52	6.48E-06	2.13E-05

**RP11-346E8.1**	-5.36	6.49E-06	2.13E-05
**MRS2**	-0.49	6.50E-06	2.14E-05
**CTC-239J10.1**	-4.90	6.51E-06	2.14E-05
**RP11-325K4.3**	-2.12	6.51E-06	2.14E-05
**ARHGAP15**	1.39	6.52E-06	2.14E-05
**RP6-206I17.1**	1.41	6.53E-06	2.14E-05
**RP11-927P21.1**	-2.39	6.53E-06	2.14E-05
**GLOD4**	0.58	6.53E-06	2.14E-05
**RPL7P25**	-5.37	6.54E-06	2.15E-05
**SLC25A30**	-0.41	6.54E-06	2.15E-05
**RP11-263K4.1**	-5.29	6.57E-06	2.16E-05
**RP11-175K6.1**	2.32	6.57E-06	2.16E-05
**RP11-618N24.1**	-3.43	6.59E-06	2.16E-05
**FAM193A**	0.59	6.60E-06	2.17E-05
**CCNB2P1**	-5.21	6.65E-06	2.18E-05
**ZDHHC11**	-2.28	6.65E-06	2.18E-05
**WDR45B**	0.46	6.65E-06	2.18E-05
**CRTC3**	0.63	6.67E-06	2.19E-05
**VAV1**	1.68	6.67E-06	2.19E-05
**MTND5P26**	-5.48	6.68E-06	2.19E-05
**ZMYND11**	-0.46	6.69E-06	2.19E-05
**SAMD5**	-1.82	6.70E-06	2.20E-05
**TEX9**	-0.85	6.70E-06	2.20E-05
**SCN3B**	0.91	6.71E-06	2.20E-05
**CTD-2262B20.1**	-2.92	6.71E-06	2.20E-05
**TXNDC12-AS1**	-4.89	6.72E-06	2.20E-05
**PRPF40A**	-0.55	6.73E-06	2.20E-05
**AP001619.2**	-3.44	6.73E-06	2.21E-05
**G16026**	-2.98	6.75E-06	2.21E-05
**RNA5SP78**	-5.20	6.75E-06	2.21E-05
**DCTN4**	-0.51	6.77E-06	2.22E-05
**ABCC4**	-0.96	6.77E-06	2.22E-05
**PI4KAP2**	1.70	6.79E-06	2.22E-05
**RP11-240G22.5**	-3.51	6.79E-06	2.22E-05
**TCN2**	1.39	6.82E-06	2.23E-05
**AC104113.3**	-5.31	6.83E-06	2.23E-05
**AC093157.1**	-3.17	6.83E-06	2.24E-05
**RP11-151A6.4**	-1.93	6.83E-06	2.24E-05
**G42257**	-4.98	6.84E-06	2.24E-05
**KLF9**	1.05	6.85E-06	2.24E-05
**SRMS**	1.71	6.85E-06	2.24E-05
**RBKS**	1.12	6.86E-06	2.25E-05
**PKP3**	0.92	6.88E-06	2.25E-05
**RP11-65L3.2**	-2.51	6.88E-06	2.25E-05
**RP11-1012A1.10**	-5.10	6.89E-06	2.25E-05

**EIF2S3**	-0.58	6.90E-06	2.26E-05
**RP11-508M8.1**	-6.12	6.91E-06	2.26E-05
**VPS26BP1**	-4.15	6.93E-06	2.27E-05
**TCTEX1D1**	2.14	6.94E-06	2.27E-05
**MIR3659**	-5.20	6.95E-06	2.27E-05
**RP11-20B24.5**	-5.02	6.95E-06	2.27E-05
**RFC5**	-0.58	6.96E-06	2.27E-05
**KRT43P**	-5.43	6.98E-06	2.28E-05
**FANCF**	-0.63	7.00E-06	2.29E-05
**TSPAN9-IT1**	-5.22	7.01E-06	2.29E-05
**LPAR2**	0.66	7.03E-06	2.30E-05
**RP3-476K8.4**	-3.50	7.06E-06	2.30E-05
**TOP3B**	-2.99	7.06E-06	2.31E-05
**ACADVL**	0.75	7.07E-06	2.31E-05
**RP11-613F22.6**	-3.71	7.08E-06	2.31E-05
**SP2-AS1**	-1.06	7.08E-06	2.31E-05
**DENND1A**	0.78	7.08E-06	2.31E-05
**RP11-82O19.2**	-2.96	7.09E-06	2.31E-05
**G6708**	-1.04	7.10E-06	2.32E-05
**CFAP100**	-4.80	7.10E-06	2.32E-05
**SNORD114-20**	-5.48	7.11E-06	2.32E-05
**SCD**	1.28	7.12E-06	2.32E-05
**MAPKAP1**	0.38	7.12E-06	2.32E-05
**F12**	1.05	7.12E-06	2.32E-05
**AC016757.3**	2.62	7.13E-06	2.32E-05
**RP11-298E2.2**	-3.66	7.15E-06	2.33E-05
**G30728**	-2.04	7.15E-06	2.33E-05
**RFWD2**	-0.41	7.15E-06	2.33E-05
**SNORD63**	-2.15	7.15E-06	2.33E-05
**NAA50**	-0.61	7.16E-06	2.33E-05
**CFAP44-AS1**	-4.96	7.16E-06	2.33E-05
**CISH**	0.86	7.16E-06	2.33E-05
**CXorf57**	-0.91	7.17E-06	2.34E-05
**SRRM1P3**	-4.49	7.18E-06	2.34E-05
**AC092839.4**	-5.04	7.20E-06	2.35E-05
**SUN1**	-0.47	7.21E-06	2.35E-05
**JUP**	0.86	7.22E-06	2.35E-05
**LYRM7**	-0.81	7.22E-06	2.35E-05
**POLR3H**	0.51	7.22E-06	2.35E-05
**RP4-644L1.2**	-4.23	7.23E-06	2.36E-05
**CTD-2301A4.3**	-2.56	7.24E-06	2.36E-05
**NR1H3**	1.24	7.24E-06	2.36E-05
**CHST11**	1.48	7.25E-06	2.36E-05
**EDDM3CP**	-5.07	7.26E-06	2.36E-05
**RP11-981G7.6**	-2.06	7.26E-06	2.36E-05

**RP11-493G17.4**	-5.31	7.28E-06	2.37E-05
**CTD-3099C6.11**	1.53	7.28E-06	2.37E-05
**RP11-69E11.8**	-2.80	7.30E-06	2.38E-05
**UBA52**	0.61	7.31E-06	2.38E-05
**DDX52**	-0.60	7.32E-06	2.38E-05
**MYH15**	-1.87	7.32E-06	2.38E-05
**PPA2**	-0.53	7.34E-06	2.39E-05
**AC012613.2**	-5.29	7.34E-06	2.39E-05
**GAPDHP33**	-5.20	7.35E-06	2.39E-05
**HLA-DQA1**	-3.31	7.36E-06	2.39E-05
**POLR2A**	0.88	7.37E-06	2.40E-05
**RNU6-1327P**	-5.24	7.37E-06	2.40E-05
**RPS3P7**	-5.46	7.37E-06	2.40E-05
**PSMD3**	0.56	7.37E-06	2.40E-05
**EPHA8**	5.39	7.38E-06	2.40E-05
**RP11-87H9.3**	-2.24	7.38E-06	2.40E-05
**AC018755.16**	2.13	7.39E-06	2.40E-05
**XLOC_013178**	-4.94	7.42E-06	2.41E-05
**RNASEH1-AS1**	1.12	7.42E-06	2.41E-05
**RBPJ**	-0.47	7.42E-06	2.41E-05
**SNAPIN**	0.67	7.43E-06	2.41E-05
**AC130709.1**	-5.22	7.43E-06	2.41E-05
**PRR15L**	2.06	7.44E-06	2.42E-05
**RP11-354P11.2**	-5.24	7.45E-06	2.42E-05
**CTB-193M12.1**	-5.15	7.46E-06	2.42E-05
**FAM32A**	0.61	7.47E-06	2.43E-05
**ESRP2**	0.61	7.48E-06	2.43E-05
**PPIC**	0.95	7.49E-06	2.43E-05
**TMEM143**	0.96	7.49E-06	2.43E-05
**RPSAP21**	-3.62	7.50E-06	2.43E-05
**BMP4**	0.78	7.50E-06	2.43E-05
**KRT39**	-5.06	7.51E-06	2.44E-05
**KB-1205A7.1**	-5.31	7.51E-06	2.44E-05
**ADGRL1**	0.81	7.51E-06	2.44E-05
**RP11-51F16.1**	2.15	7.51E-06	2.44E-05
**BAHD1**	0.61	7.53E-06	2.44E-05
**RBP2**	-2.94	7.54E-06	2.45E-05
**MIR503HG**	3.11	7.61E-06	2.47E-05
**P2RY6**	2.51	7.65E-06	2.48E-05
**UBL7**	0.63	7.65E-06	2.48E-05
**TMC7**	1.24	7.66E-06	2.48E-05
**RP11-1129I3.1**	-4.92	7.66E-06	2.48E-05
**XXyac-YR38GF2.1**	-2.45	7.67E-06	2.49E-05
**MYLK-AS1**	-1.74	7.68E-06	2.49E-05
**SAMD14**	2.13	7.68E-06	2.49E-05

**RPS2P7**	-5.77	7.68E-06	2.49E-05
**TPD52**	-0.86	7.70E-06	2.49E-05
**DNM1P35**	-2.60	7.81E-06	2.53E-05
**NME9**	-1.65	7.81E-06	2.53E-05
**RP11-823P9.4**	-5.27	7.82E-06	2.53E-05
**MIR27B**	-1.82	7.83E-06	2.53E-05
**GPR3**	3.42	7.83E-06	2.54E-05
**IQCJ**	-6.18	7.84E-06	2.54E-05
**GCHFR**	-2.41	7.85E-06	2.54E-05
**RNU6-341P**	-5.06	7.85E-06	2.54E-05
**USP39**	0.37	7.86E-06	2.54E-05
**G11966**	-3.52	7.88E-06	2.55E-05
**RP1-80N2.3**	-1.60	7.89E-06	2.55E-05
**MIR194-1**	-5.04	7.89E-06	2.55E-05
**MTND5P28**	-3.03	7.90E-06	2.56E-05
**PCYT1A**	-0.62	7.90E-06	2.56E-05
**RP11-886P16.10**	-5.01	7.91E-06	2.56E-05
**DCLK1**	-1.67	7.91E-06	2.56E-05
**BARX2**	1.42	7.94E-06	2.57E-05
**RP11-705C15.3**	-1.58	7.95E-06	2.57E-05
**PRR15**	1.88	7.99E-06	2.58E-05
**CTPS2**	-0.58	7.99E-06	2.58E-05
**MTCYBP3**	-5.28	8.00E-06	2.59E-05
**VN1R14P**	-4.88	8.02E-06	2.59E-05
**FBXO22**	-0.53	8.02E-06	2.59E-05
**SNORD100**	-1.86	8.03E-06	2.60E-05
**RP11-535M15.1**	2.89	8.06E-06	2.60E-05
**RP11-769O8.1**	-4.79	8.09E-06	2.61E-05
**ZNF853**	1.27	8.11E-06	2.62E-05
**AC008746.5**	-2.61	8.11E-06	2.62E-05
**RP11-540O11.8**	-4.88	8.12E-06	2.62E-05
**SNX7**	0.79	8.14E-06	2.63E-05
**ATOX1**	0.44	8.15E-06	2.63E-05
**RP11-430H10.2**	3.99	8.17E-06	2.64E-05
**CA14**	-1.09	8.17E-06	2.64E-05
**ZNF880**	-1.02	8.17E-06	2.64E-05
**CTD-2522E6.4**	-4.79	8.18E-06	2.64E-05
**CTB-58E17.1**	0.89	8.18E-06	2.64E-05
**FOS**	2.35	8.18E-06	2.64E-05
**LYSMD3**	-0.61	8.19E-06	2.64E-05
**RBP4**	4.01	8.20E-06	2.64E-05
**RP11-571L19.8**	-1.27	8.21E-06	2.65E-05
**OLFM2**	0.85	8.22E-06	2.65E-05
**BNIP3P27**	-5.06	8.22E-06	2.65E-05
**NOP56**	0.64	8.23E-06	2.65E-05

**MAPK8IP1**	0.89	8.23E-06	2.65E-05
**RP11-552M11.8**	-1.34	8.25E-06	2.66E-05
**ARMCX5-GPRASP2**	-0.83	8.26E-06	2.66E-05
**WASH7P**	1.07	8.27E-06	2.67E-05
**RP11-342M21.2**	-5.26	8.27E-06	2.67E-05
**RP11-164P12.5**	-0.79	8.27E-06	2.67E-05
**ZCCHC24**	1.35	8.29E-06	2.67E-05
**ATP8B1**	-0.46	8.32E-06	2.68E-05
**RP11-39M21.2**	-3.25	8.36E-06	2.69E-05
**RP11-177N22.2**	-5.44	8.37E-06	2.70E-05
**CPB2**	-4.90	8.40E-06	2.71E-05
**CD1B**	-3.72	8.40E-06	2.71E-05
**UEVLD**	-0.86	8.40E-06	2.71E-05
**AVPI1**	0.90	8.43E-06	2.71E-05
**MTATP6P11**	-5.40	8.43E-06	2.71E-05
**BEND6**	2.13	8.47E-06	2.73E-05
**SAP18**	0.64	8.48E-06	2.73E-05
**LINC00710**	-3.15	8.52E-06	2.74E-05
**CENPCP1**	-5.28	8.55E-06	2.75E-05
**AURKB**	1.05	8.55E-06	2.75E-05
**FGFBP3**	1.04	8.55E-06	2.75E-05
**RP11-15I11.3**	-2.50	8.56E-06	2.76E-05
**FLJ13224**	-2.70	8.57E-06	2.76E-05
**AC007919.18**	-4.69	8.57E-06	2.76E-05
**CPSF6**	-0.51	8.58E-06	2.76E-05
**RP11-2C24.9**	-0.99	8.58E-06	2.76E-05
**INIP**	-0.48	8.59E-06	2.76E-05
**CTD-2192J16.20**	-1.14	8.61E-06	2.77E-05
**OR2I1P**	4.15	8.62E-06	2.77E-05
**TM9SF4**	0.50	8.62E-06	2.77E-05
**RP11-122A21.2**	-4.24	8.63E-06	2.77E-05
**MEIS1-AS2**	-4.90	8.63E-06	2.77E-05
**DNMBP**	-0.57	8.65E-06	2.78E-05
**RNVU1-13**	-3.65	8.67E-06	2.79E-05
**RP11-10E18.7**	-2.34	8.67E-06	2.79E-05
**CTC-436P18.4**	-2.94	8.70E-06	2.80E-05
**RPL29P19**	3.99	8.71E-06	2.80E-05
**RNF125**	-0.86	8.71E-06	2.80E-05
**XLOC_003480**	-3.56	8.71E-06	2.80E-05
**PMF1**	0.78	8.73E-06	2.80E-05
**RPL23AP11**	-5.05	8.73E-06	2.80E-05
**ZNF223**	-1.27	8.73E-06	2.80E-05
**RP11-501M7.1**	-5.32	8.76E-06	2.81E-05
**RP11-150O12.6**	3.58	8.79E-06	2.82E-05
**SEMA3F-AS1**	-1.87	8.80E-06	2.83E-05

**MKX**	2.68	8.81E-06	2.83E-05
**RP11-328C8.2**	-3.81	8.82E-06	2.83E-05
**PCSK1**	2.34	8.83E-06	2.83E-05
**CTC-459F4.1**	-1.58	8.84E-06	2.84E-05
**SNORA81**	-2.81	8.87E-06	2.85E-05
**CTA-392E5.1**	5.10	8.89E-06	2.85E-05
**CTD-2235C13.3**	-2.19	8.89E-06	2.85E-05
**RP11-793H13.12**	-4.91	8.92E-06	2.86E-05
**KRT73**	-2.90	8.93E-06	2.87E-05
**PEX6**	1.23	8.94E-06	2.87E-05
**FLRT2**	-0.86	8.94E-06	2.87E-05
**NLE1**	0.53	8.95E-06	2.87E-05
**AC006445.7**	-4.94	8.99E-06	2.88E-05
**AC144530.1**	-2.88	8.99E-06	2.88E-05
**FAU**	0.65	9.00E-06	2.89E-05
**G28021**	-4.91	9.01E-06	2.89E-05
**INPP5F**	-0.49	9.02E-06	2.89E-05
**DOCK10**	-0.75	9.04E-06	2.90E-05
**G38234**	-3.58	9.04E-06	2.90E-05
**AC002128.5**	-2.20	9.04E-06	2.90E-05
**HAPLN1**	-5.91	9.09E-06	2.91E-05
**C1QTNF9B**	-3.97	9.09E-06	2.91E-05
**PCED1B-AS1**	2.17	9.12E-06	2.92E-05
**ATRIP**	0.57	9.13E-06	2.92E-05
**LAMA4**	1.50	9.15E-06	2.93E-05
**E2F4**	0.56	9.15E-06	2.93E-05
**CATSPERG**	-1.57	9.19E-06	2.94E-05
**TUSC3**	1.15	9.23E-06	2.95E-05
**FRMD4A**	-0.70	9.24E-06	2.96E-05
**ADAMTS9**	1.46	9.25E-06	2.96E-05
**AC092687.5**	-5.27	9.25E-06	2.96E-05
**RP11-102M11.1**	-2.21	9.26E-06	2.96E-05
**SMU1**	-0.48	9.26E-06	2.96E-05
**FLG-AS1**	-1.38	9.29E-06	2.97E-05
**RP11-304F15.3**	-1.75	9.29E-06	2.97E-05
**SLC45A2**	-1.66	9.30E-06	2.97E-05
**AC016683.5**	-4.68	9.30E-06	2.98E-05
**RP11-359M6.1**	-4.05	9.33E-06	2.98E-05
**FAM3C2**	1.18	9.35E-06	2.99E-05
**RNU7-91P**	-5.35	9.36E-06	2.99E-05
**CTA-331F8.1**	-5.36	9.37E-06	3.00E-05
**MTIF3**	0.52	9.38E-06	3.00E-05
**RP11-166O4.4**	-5.13	9.39E-06	3.00E-05
**BUD31**	0.65	9.40E-06	3.00E-05
**MYEOV2**	0.77	9.40E-06	3.01E-05

**RP11-435O5.6**	-5.12	9.41E-06	3.01E-05
**DOT1L**	0.82	9.41E-06	3.01E-05
**RPL12P20**	-5.19	9.42E-06	3.01E-05
**RPL4P4**	-1.05	9.43E-06	3.01E-05
**HTR5BP**	-3.00	9.45E-06	3.02E-05
**RPL7AP15**	-5.22	9.48E-06	3.03E-05
**ENO1P3**	-3.32	9.54E-06	3.05E-05
**AC092171.4**	0.80	9.54E-06	3.05E-05
**PRR19**	1.95	9.57E-06	3.06E-05
**RP11-90D4.3**	-5.05	9.57E-06	3.06E-05
**EIF4BP7**	-1.23	9.58E-06	3.06E-05
**RP11-439H13.2**	-1.47	9.58E-06	3.06E-05
**RP11-622C24.2**	-2.84	9.63E-06	3.08E-05
**PAXIP1**	-0.47	9.64E-06	3.08E-05
**SNORA17**	-5.06	9.65E-06	3.08E-05
**RP11-777B9.1**	-5.05	9.65E-06	3.08E-05
**ADCYAP1**	5.14	9.66E-06	3.08E-05
**NCLP1**	-2.88	9.68E-06	3.09E-05
**FGD5**	1.33	9.68E-06	3.09E-05
**BUD13**	0.39	9.71E-06	3.10E-05
**RNU6-608P**	-5.17	9.72E-06	3.10E-05
**PABPC4L**	1.58	9.72E-06	3.10E-05
**TXNP6**	-5.16	9.74E-06	3.11E-05
**PTPN20B**	-2.00	9.75E-06	3.11E-05
**AP000473.8**	-2.04	9.76E-06	3.11E-05
**EFCAB5**	-2.23	9.76E-06	3.11E-05
**POLE2**	-0.75	9.77E-06	3.11E-05
**RP1-300G12.2**	-4.88	9.78E-06	3.12E-05
**LCNL1**	4.30	9.78E-06	3.12E-05
**VENTX**	2.97	9.80E-06	3.12E-05
**TMEM164**	-0.94	9.81E-06	3.13E-05
**ZFP91**	-0.50	9.83E-06	3.13E-05
**TMEM92**	2.53	9.86E-06	3.14E-05
**U6**	-2.62	9.88E-06	3.15E-05
**RP11-358B23.5**	-4.07	9.89E-06	3.15E-05
**C11orf54**	-0.77	9.89E-06	3.15E-05
**MIR548E**	-5.23	9.90E-06	3.15E-05
**FMNL1**	1.40	9.91E-06	3.15E-05
**HNRNPA1P76**	-3.24	9.94E-06	3.17E-05
**MSH4**	-2.93	9.96E-06	3.17E-05
**GDPD3**	1.09	9.98E-06	3.18E-05
**CTD-2104P17.1**	-4.08	9.98E-06	3.18E-05
**GLYATL1**	-2.25	9.99E-06	3.18E-05
**RP11-515O17.3**	-4.84	9.99E-06	3.18E-05
**ATF6**	-0.62	1.00E-05	3.18E-05

**EIF2AK1**	0.41	1.00E-05	3.18E-05
**BOC**	-0.94	1.00E-05	3.18E-05
**TSPAN7**	1.23	1.00E-05	3.19E-05
**SYCE3**	3.00	1.00E-05	3.19E-05
**HSPA8P18**	-5.22	1.01E-05	3.20E-05
**SPRR2D**	2.44	1.01E-05	3.20E-05
**ZNF131**	-0.44	1.01E-05	3.21E-05
**SLC38A1**	-0.76	1.01E-05	3.21E-05
**CCDC168**	-3.97	1.01E-05	3.21E-05
**RP11-216N14.7**	-3.62	1.01E-05	3.21E-05
**NIPAL3**	-0.76	1.01E-05	3.21E-05
**XLOC_000566**	-4.84	1.01E-05	3.21E-05
**TPRKBP2**	-5.06	1.01E-05	3.22E-05
**EID3**	-1.00	1.01E-05	3.22E-05
**RN7SL23P**	-5.32	1.01E-05	3.22E-05
**RP11-6O2.3**	-2.13	1.01E-05	3.22E-05
**RP11-517H2.6**	-4.75	1.01E-05	3.22E-05
**RBM26**	-0.56	1.01E-05	3.22E-05
**RP11-531A24.5**	-1.49	1.02E-05	3.23E-05
**G29706**	-5.14	1.02E-05	3.23E-05
**HN1**	0.71	1.02E-05	3.24E-05
**MIR5692C2**	-4.91	1.02E-05	3.24E-05
**RN7SL552P**	-5.08	1.02E-05	3.25E-05
**RPL18P10**	-4.14	1.03E-05	3.25E-05
**OSTF1**	0.56	1.03E-05	3.26E-05
**CEP295NL**	-2.80	1.03E-05	3.26E-05
**GBP4**	1.57	1.03E-05	3.26E-05
**HSD3BP1**	-5.07	1.03E-05	3.27E-05
**RP11-22B10.3**	-5.41	1.03E-05	3.27E-05
**MED8**	0.55	1.03E-05	3.28E-05
**KCND3-AS1**	-4.83	1.03E-05	3.28E-05
**EMILIN3**	-2.26	1.04E-05	3.28E-05
**RP11-863P13.4**	-1.38	1.04E-05	3.29E-05
**RPL12P11**	-5.16	1.04E-05	3.29E-05
**NDRG3**	0.51	1.04E-05	3.29E-05
**KCNK5**	2.56	1.04E-05	3.29E-05
**MTND4P23**	-5.26	1.04E-05	3.29E-05
**FBLN7**	1.70	1.04E-05	3.29E-05
**ZNF331**	-0.56	1.05E-05	3.31E-05
**RP11-482M8.1**	1.69	1.05E-05	3.31E-05
**STAP2**	0.74	1.05E-05	3.32E-05
**RP11-344N10.5**	-1.59	1.05E-05	3.32E-05
**ENPP7P4**	-2.67	1.05E-05	3.33E-05
**RP11-451F14.1**	-5.13	1.05E-05	3.33E-05
**snoZ5**	-5.07	1.05E-05	3.33E-05

**RP11-540O11.6**	-4.91	1.05E-05	3.33E-05
**PJA2**	-0.59	1.05E-05	3.34E-05
**CLCN1**	3.49	1.06E-05	3.35E-05
**CCDC74BP1**	-5.04	1.06E-05	3.35E-05
**CABLES1**	-0.62	1.06E-05	3.36E-05
**LZTR1**	0.94	1.06E-05	3.36E-05
**G11316**	-5.90	1.06E-05	3.36E-05
**DEF8**	0.55	1.06E-05	3.36E-05
**PIF1**	1.78	1.06E-05	3.36E-05
**ASB9P1**	-4.86	1.06E-05	3.37E-05
**PCOLCE2**	-1.81	1.07E-05	3.37E-05
**GHRL**	-1.86	1.07E-05	3.37E-05
**RPL7P52**	-5.18	1.07E-05	3.38E-05
**CTC-459M5.2**	-2.84	1.07E-05	3.38E-05
**IL2RA**	3.09	1.07E-05	3.38E-05
**TLR7**	2.37	1.07E-05	3.39E-05
**AC009009.1**	-5.28	1.07E-05	3.39E-05
**FAM160B1**	-0.50	1.07E-05	3.39E-05
**FAM149B1**	-0.38	1.07E-05	3.39E-05
**DIO1**	-2.65	1.07E-05	3.39E-05
**GPC5-AS2**	-5.10	1.08E-05	3.41E-05
**FIBIN**	1.53	1.08E-05	3.41E-05
**RP11-375I20.6**	-2.72	1.08E-05	3.41E-05
**N6AMT2**	0.72	1.08E-05	3.42E-05
**SERPINB10**	-1.39	1.08E-05	3.42E-05
**AC124914.3**	-4.34	1.08E-05	3.42E-05
**XPOTP1**	-5.21	1.08E-05	3.42E-05
**CCDC115**	0.58	1.08E-05	3.42E-05
**CTD-2095E4.3**	-2.02	1.08E-05	3.43E-05
**OR52I1**	-4.96	1.08E-05	3.43E-05
**RP11-161M6.2**	2.42	1.09E-05	3.43E-05
**BECN1**	0.42	1.09E-05	3.43E-05
**PSG1**	-5.37	1.09E-05	3.44E-05
**MUSK**	-2.52	1.09E-05	3.45E-05
**WWC2-AS2**	1.12	1.09E-05	3.45E-05
**RP11-439E19.9**	-2.73	1.09E-05	3.45E-05
**RP11-466A19.4**	-5.27	1.09E-05	3.45E-05
**HIST2H2AA3**	-2.72	1.09E-05	3.45E-05
**RP11-6E9.4**	-4.87	1.09E-05	3.45E-05
**CCDC149**	0.77	1.10E-05	3.46E-05
**RP11-537I16.2**	-5.30	1.10E-05	3.46E-05
**G24850**	-2.75	1.10E-05	3.46E-05
**LAD1**	0.80	1.10E-05	3.46E-05
**PPP1R26P1**	-1.93	1.10E-05	3.46E-05
**CTC-308K20.4**	-5.05	1.10E-05	3.47E-05

**TPM3P7**	-5.30	1.10E-05	3.47E-05
**DOLPP1**	0.48	1.10E-05	3.47E-05
**PLCD3**	0.78	1.10E-05	3.47E-05
**PREX1**	1.07	1.11E-05	3.49E-05
**RP11-493E3.1**	-4.65	1.11E-05	3.49E-05
**TRPM6**	1.57	1.11E-05	3.50E-05
**RP4-534N18.2**	-2.64	1.11E-05	3.51E-05
**RPL36AP15**	-3.52	1.11E-05	3.51E-05
**AXDND1**	-4.44	1.12E-05	3.51E-05
**RP11-397J20.1**	-5.05	1.12E-05	3.52E-05
**FAM53A**	1.31	1.12E-05	3.52E-05
**RP4-791M13.5**	-5.04	1.12E-05	3.52E-05
**GYG2**	1.52	1.12E-05	3.52E-05
**FAM49A**	1.31	1.12E-05	3.53E-05
**RAB21**	-0.56	1.12E-05	3.53E-05
**RP11-265F19.1**	-4.92	1.12E-05	3.53E-05
**GAS6-AS1**	-1.75	1.12E-05	3.53E-05
**LINC01311**	-1.53	1.12E-05	3.53E-05
**PIGU**	0.50	1.12E-05	3.54E-05
**RP5-1139B12.4**	-4.87	1.12E-05	3.54E-05
**OSTN**	-5.34	1.12E-05	3.54E-05
**H2AFZP3**	-4.88	1.13E-05	3.55E-05
**UBQLN1**	-0.53	1.13E-05	3.55E-05
**ZC3H8**	-0.53	1.13E-05	3.56E-05
**RP11-21M24.3**	-4.11	1.13E-05	3.56E-05
**CTD-2583P5.1**	-5.08	1.13E-05	3.56E-05
**RUVBL1**	0.56	1.13E-05	3.56E-05
**CEP63**	0.48	1.13E-05	3.56E-05
**RP11-802O23.3**	-4.99	1.13E-05	3.57E-05
**RNU5F-1**	-4.07	1.14E-05	3.57E-05
**RP11-44N11.1**	-2.09	1.14E-05	3.57E-05
**PTPN6**	0.66	1.14E-05	3.58E-05
**PRICKLE4**	1.30	1.14E-05	3.58E-05
**TSHR**	-2.19	1.14E-05	3.58E-05
**IFI6**	2.76	1.14E-05	3.59E-05
**EEF1A2**	1.67	1.14E-05	3.59E-05
**RP11-478C6.5**	-4.65	1.14E-05	3.60E-05
**WDR88**	-1.18	1.15E-05	3.62E-05
**FOLH1**	1.60	1.15E-05	3.62E-05
**HDAC11**	0.62	1.15E-05	3.63E-05
**FCER1G**	2.30	1.16E-05	3.63E-05
**SEPT7P6**	-5.22	1.16E-05	3.63E-05
**RABGGTB**	-0.55	1.16E-05	3.65E-05
**RP11-210K20.6**	-5.18	1.16E-05	3.66E-05
**CTD-2235C13.1**	-3.04	1.17E-05	3.66E-05

**PHOSPHO2**	-1.14	1.17E-05	3.68E-05
**AC010999.1**	-4.98	1.17E-05	3.68E-05
**ZNF621**	-0.59	1.17E-05	3.68E-05
**IL6ST**	-0.94	1.17E-05	3.69E-05
**GPAT2**	1.85	1.18E-05	3.69E-05
**RP13-476E20.1**	-1.90	1.18E-05	3.69E-05
**GAPDHP39**	-3.75	1.18E-05	3.70E-05
**G33837**	-5.72	1.18E-05	3.70E-05
**KLHL22**	0.64	1.18E-05	3.71E-05
**RP3-525L6.2**	-3.08	1.19E-05	3.72E-05
**FAT1**	-0.81	1.19E-05	3.72E-05
**S100A7A**	4.77	1.19E-05	3.72E-05
**TMOD3**	-0.61	1.19E-05	3.72E-05
**RP11-146N23.4**	-2.11	1.19E-05	3.73E-05
**AC012368.1**	-4.77	1.19E-05	3.73E-05
**FBXL19-AS1**	1.19	1.19E-05	3.73E-05
**ZNF696**	0.64	1.19E-05	3.73E-05
**ACO2**	0.51	1.19E-05	3.74E-05
**RP11-687E1.2**	-4.14	1.19E-05	3.74E-05
**SNORD116-13**	-4.94	1.20E-05	3.75E-05
**UBTF**	0.41	1.20E-05	3.76E-05
**RN7SL141P**	-5.01	1.20E-05	3.76E-05
**ALDOC**	0.92	1.20E-05	3.77E-05
**OBP2A**	3.38	1.20E-05	3.77E-05
**SMIM6**	-2.78	1.20E-05	3.77E-05
**AC012627.1**	-5.14	1.21E-05	3.78E-05
**SNHG5**	1.37	1.21E-05	3.79E-05
**UBE2A**	0.49	1.21E-05	3.79E-05
**PDHA1P1**	-4.32	1.22E-05	3.82E-05
**LINC01503**	1.16	1.22E-05	3.82E-05
**RNU6-942P**	-3.56	1.22E-05	3.82E-05
**CTD-2066L21.2**	-5.58	1.22E-05	3.83E-05
**XPO5**	-0.51	1.23E-05	3.83E-05
**TRMT5**	-0.48	1.23E-05	3.84E-05
**LINC00412**	-5.03	1.23E-05	3.84E-05
**ATP2C1**	-0.49	1.23E-05	3.85E-05
**PES1P2**	-5.36	1.23E-05	3.85E-05
**AC006539.3**	-4.85	1.23E-05	3.86E-05
**ISM1-AS1**	-4.87	1.23E-05	3.86E-05
**RN7SL188P**	-5.22	1.24E-05	3.86E-05
**WFDC21P**	-1.60	1.24E-05	3.87E-05
**LUC7L3**	1.11	1.24E-05	3.87E-05
**BAMBI**	1.70	1.24E-05	3.88E-05
**GPR79**	-4.03	1.24E-05	3.88E-05
**RP11-305O4.2**	-3.88	1.24E-05	3.88E-05

**SMC1A**	-0.47	1.24E-05	3.88E-05
**G11188**	-1.29	1.24E-05	3.88E-05
**RP5-855D21.2**	-2.08	1.25E-05	3.89E-05
**C8orf76**	-0.92	1.25E-05	3.89E-05
**TLR3**	-0.95	1.25E-05	3.90E-05
**PRAP1**	-2.79	1.25E-05	3.90E-05
**SEC24D**	0.85	1.25E-05	3.91E-05
**NUP107**	-0.40	1.25E-05	3.91E-05
**AQP5**	4.22	1.25E-05	3.91E-05
**CRLF1**	-1.09	1.26E-05	3.93E-05
**RP4-613B23.1**	-1.79	1.26E-05	3.93E-05
**NXPE2**	-4.87	1.26E-05	3.94E-05
**GPI**	0.62	1.26E-05	3.94E-05
**G1033**	-2.91	1.26E-05	3.94E-05
**PPP1R1B**	2.00	1.27E-05	3.97E-05
**CCDC184**	1.99	1.27E-05	3.97E-05
**OXSM**	0.50	1.27E-05	3.97E-05
**BHLHE22**	2.29	1.27E-05	3.97E-05
**RP11-407H12.8**	-6.67	1.27E-05	3.98E-05
**B3GALT5-AS1**	2.79	1.28E-05	3.98E-05
**PCDHGB3**	-1.06	1.28E-05	3.98E-05
**RPL7P11**	-5.18	1.28E-05	3.98E-05
**TBL1X**	0.57	1.28E-05	3.98E-05
**RBM26-AS1**	-1.17	1.28E-05	3.99E-05
**CXCL8**	4.41	1.28E-05	3.99E-05
**RP11-178L8.3**	-2.96	1.28E-05	4.00E-05
**CEP83**	-0.76	1.28E-05	4.00E-05
**CCDC28B**	1.27	1.29E-05	4.03E-05
**BAIAP3**	1.34	1.30E-05	4.04E-05
**RP11-388P9.2**	-2.25	1.30E-05	4.05E-05
**LPL**	1.72	1.30E-05	4.05E-05
**CASC3**	0.34	1.30E-05	4.05E-05
**RP11-316K19.3**	-5.00	1.30E-05	4.06E-05
**GLB1L2**	0.93	1.30E-05	4.06E-05
**CTC-450M9.1**	-2.88	1.30E-05	4.06E-05
**RP11-768F21.1**	5.46	1.30E-05	4.06E-05
**MIR365A**	-4.99	1.31E-05	4.06E-05
**COG4**	0.51	1.31E-05	4.08E-05
**RP11-1148L6.8**	1.05	1.31E-05	4.08E-05
**PTAFR**	1.19	1.31E-05	4.08E-05
**DDX51**	0.69	1.31E-05	4.08E-05
**RP11-950C14.10**	-2.37	1.31E-05	4.08E-05
**SMG7**	0.73	1.31E-05	4.09E-05
**LMX1B**	1.32	1.31E-05	4.09E-05
**NEK8**	0.88	1.31E-05	4.09E-05

**UBE2V2P4**	-4.97	1.32E-05	4.10E-05
**ANO5**	-1.84	1.32E-05	4.10E-05
**RP11-672A2.4**	3.12	1.32E-05	4.11E-05
**TMEM38A**	1.16	1.32E-05	4.12E-05
**PSEN2**	0.74	1.32E-05	4.12E-05
**CTC-327F10.1**	-5.31	1.33E-05	4.12E-05
**ALDH7A1P2**	-4.48	1.33E-05	4.12E-05
**RP11-22B23.1**	1.56	1.33E-05	4.12E-05
**MT1L**	2.03	1.33E-05	4.14E-05
**SQRDL**	0.58	1.33E-05	4.15E-05
**DIAPH2-AS1**	-2.15	1.34E-05	4.15E-05
**AC083843.4**	-4.15	1.34E-05	4.15E-05
**ANKRD61**	-1.82	1.34E-05	4.16E-05
**RP11-613C6.4**	-4.93	1.35E-05	4.19E-05
**C14orf1**	0.82	1.35E-05	4.19E-05
**PMAIP1**	1.12	1.35E-05	4.19E-05
**CYP27B1**	1.65	1.35E-05	4.19E-05
**C10orf55**	1.40	1.35E-05	4.19E-05
**IGSF9B**	-1.81	1.35E-05	4.19E-05
**KB-1184D12.1**	5.93	1.35E-05	4.20E-05
**CNGB3**	-3.98	1.35E-05	4.20E-05
**STRBP**	-0.72	1.35E-05	4.20E-05
**RNU6-433P**	-5.08	1.36E-05	4.21E-05
**RP11-359J14.2**	-2.20	1.36E-05	4.21E-05
**PTOV1-AS1**	0.72	1.36E-05	4.23E-05
**TMEM25**	0.68	1.37E-05	4.24E-05
**LZTFL1**	-0.61	1.37E-05	4.24E-05
**KLKP1**	2.78	1.37E-05	4.24E-05
**RN7SL425P**	-3.07	1.37E-05	4.24E-05
**RP11-257I8.2**	-5.90	1.37E-05	4.24E-05
**SEC13P1**	-4.85	1.37E-05	4.24E-05
**RRAGC**	0.66	1.37E-05	4.25E-05
**RP5-1042K10.10**	-3.38	1.37E-05	4.25E-05
**ACTG1P10**	-2.58	1.37E-05	4.26E-05
**MRPL53P1**	-3.67	1.37E-05	4.26E-05
**RP11-641A6.3**	-2.49	1.38E-05	4.28E-05
**AP000889.1**	-2.96	1.38E-05	4.28E-05
**CNTFR-AS1**	-5.05	1.38E-05	4.28E-05
**ASB7**	-0.55	1.38E-05	4.28E-05
**RP11-272D12.2**	-4.51	1.38E-05	4.29E-05
**P2RY1**	-0.78	1.38E-05	4.29E-05
**PLEKHN1**	0.93	1.39E-05	4.29E-05
**RP11-53O19.2**	-5.28	1.39E-05	4.29E-05
**PPIAP22**	0.91	1.39E-05	4.29E-05
**TNFSF13**	1.55	1.39E-05	4.30E-05

**RP11-2B6.2**	-2.77	1.39E-05	4.30E-05
**BAALC-AS2**	4.09	1.39E-05	4.31E-05
**RP11-403A21.3**	-4.41	1.39E-05	4.32E-05
**GRIK1**	-1.62	1.39E-05	4.32E-05
**MTND2P2**	-4.88	1.40E-05	4.32E-05
**KRT18P4**	-2.74	1.40E-05	4.32E-05
**RP5-1056H1.2**	-1.12	1.40E-05	4.33E-05
**FANCI**	-0.61	1.40E-05	4.33E-05
**RP11-259O2.2**	-2.51	1.40E-05	4.34E-05
**RP11-40C6.2**	-3.42	1.41E-05	4.35E-05
**CD160**	-1.87	1.41E-05	4.35E-05
**AC064874.1**	-4.98	1.41E-05	4.36E-05
**RP11-484O2.1**	-4.94	1.41E-05	4.36E-05
**PLS3-AS1**	-1.55	1.41E-05	4.36E-05
**CECR3**	-5.00	1.41E-05	4.36E-05
**SUCLG1**	0.74	1.41E-05	4.36E-05
**AQP3**	1.08	1.41E-05	4.37E-05
**U4**	-5.03	1.41E-05	4.37E-05
**CTD-2047H16.4**	-1.38	1.41E-05	4.37E-05
**HSPE1P11**	-4.77	1.41E-05	4.37E-05
**PRICKLE1**	1.36	1.41E-05	4.37E-05
**RP11-345P4.7**	-2.02	1.42E-05	4.38E-05
**GABRB3**	-2.67	1.42E-05	4.38E-05
**EEF1A1P11**	-1.27	1.42E-05	4.39E-05
**EPHX3**	0.85	1.42E-05	4.39E-05
**STAB1**	1.90	1.42E-05	4.39E-05
**HLCS-IT1**	-5.13	1.42E-05	4.40E-05
**C8orf17**	-5.17	1.42E-05	4.40E-05
**KLF13**	-0.59	1.43E-05	4.40E-05
**SPRED3**	1.17	1.43E-05	4.41E-05
**TTYH2**	1.04	1.43E-05	4.41E-05
**RPL7L1P12**	-4.12	1.43E-05	4.41E-05
**SLC4A1**	-4.01	1.43E-05	4.42E-05
**COQ10A**	0.91	1.43E-05	4.42E-05
**PEX3**	-0.73	1.44E-05	4.44E-05
**VRK1**	-0.58	1.44E-05	4.44E-05
**TAS2R46**	-4.11	1.44E-05	4.45E-05
**KRT35**	-6.72	1.44E-05	4.45E-05
**LRTOMT**	-0.83	1.45E-05	4.46E-05
**STAG3**	-1.30	1.45E-05	4.47E-05
**RP11-278C7.1**	-1.41	1.45E-05	4.47E-05
**FUBP1**	-0.58	1.45E-05	4.48E-05
**SLC35A2**	0.53	1.45E-05	4.48E-05
**PVR**	0.64	1.45E-05	4.48E-05
**HOXA7**	1.49	1.46E-05	4.50E-05

**RP11-242D8.2**	-5.06	1.46E-05	4.51E-05
**RP4-752I6.1**	-4.42	1.46E-05	4.51E-05
**DGKG**	-1.08	1.46E-05	4.51E-05
**NDUFAF4P3**	-2.14	1.47E-05	4.53E-05
**MRAS**	1.63	1.47E-05	4.53E-05
**RPL21P23**	-5.13	1.47E-05	4.53E-05
**COX5BP3**	-5.02	1.47E-05	4.53E-05
**ERICH1**	0.72	1.47E-05	4.53E-05
**CUL2**	-0.49	1.47E-05	4.54E-05
**PGM5P1**	-1.79	1.48E-05	4.56E-05
**RP11-454F8.3**	-5.09	1.48E-05	4.56E-05
**FHAD1**	3.53	1.48E-05	4.56E-05
**RP11-87H9.4**	-2.23	1.48E-05	4.57E-05
**RNU6-260P**	-5.17	1.48E-05	4.57E-05
**BRF2**	0.68	1.48E-05	4.57E-05
**DDX42**	0.31	1.48E-05	4.57E-05
**SLC9A8**	0.61	1.48E-05	4.57E-05
**RP11-303E16.9**	-2.97	1.48E-05	4.57E-05
**AP004290.1**	-4.69	1.49E-05	4.58E-05
**BTF3**	0.64	1.49E-05	4.58E-05
**DGKQ**	0.74	1.49E-05	4.58E-05
**CTD-2260A17.3**	-4.60	1.49E-05	4.59E-05
**C6orf3**	-1.05	1.50E-05	4.60E-05
**AC126614.1**	-5.17	1.50E-05	4.61E-05
**TLN1**	0.59	1.50E-05	4.63E-05
**RPL7L1P3**	-3.10	1.51E-05	4.64E-05
**MEMO1P1**	-1.43	1.51E-05	4.64E-05
**FLJ27354**	-1.38	1.51E-05	4.65E-05
**RN7SKP163**	-4.95	1.51E-05	4.65E-05
**WAC-AS1**	0.67	1.51E-05	4.65E-05
**HVCN1**	1.50	1.52E-05	4.67E-05
**SLC5A3**	-1.13	1.52E-05	4.68E-05
**RP5-1092A3.4**	-2.01	1.52E-05	4.68E-05
**SMG6-IT1**	-5.12	1.52E-05	4.68E-05
**CC2D2B**	-3.61	1.52E-05	4.68E-05
**RP11-113A11.1**	-4.99	1.53E-05	4.69E-05
**ISG15**	2.91	1.53E-05	4.69E-05
**RN7SL381P**	-4.06	1.53E-05	4.70E-05
**TTC31**	0.55	1.53E-05	4.70E-05
**SNORA70F**	-5.15	1.53E-05	4.71E-05
**KRT20**	-3.03	1.54E-05	4.72E-05
**CTD-2044J15.1**	-4.83	1.54E-05	4.72E-05
**TMPRSS7**	-2.69	1.54E-05	4.73E-05
**DDIT4L**	2.26	1.54E-05	4.74E-05
**CDKL2**	-3.37	1.54E-05	4.74E-05

**ASB8**	0.39	1.55E-05	4.75E-05
**BMP8A**	2.45	1.55E-05	4.75E-05
**RNU2-42P**	-5.06	1.55E-05	4.75E-05
**RP11-3K24.3**	-4.90	1.55E-05	4.76E-05
**HLA-DPB1**	1.23	1.56E-05	4.78E-05
**ADM2**	1.76	1.56E-05	4.78E-05
**BMP7-AS1**	-5.17	1.56E-05	4.79E-05
**RNU7-79P**	-4.99	1.56E-05	4.79E-05
**NDUFAF4P1**	-4.79	1.56E-05	4.79E-05
**RPL7P18**	3.57	1.56E-05	4.80E-05
**ENPP2**	1.03	1.57E-05	4.80E-05
**CDK12**	-0.44	1.57E-05	4.81E-05
**DDX56**	0.44	1.57E-05	4.81E-05
**TBATA**	-3.28	1.57E-05	4.81E-05
**GPN2**	0.41	1.57E-05	4.82E-05
**AL353662.1**	-4.94	1.57E-05	4.82E-05
**SLC16A1**	1.09	1.57E-05	4.82E-05
**SRSF1**	-0.48	1.57E-05	4.82E-05
**ELOVL6**	-1.11	1.57E-05	4.82E-05
**SLC30A9**	-0.57	1.58E-05	4.85E-05
**WISP1**	2.18	1.58E-05	4.85E-05
**XLOC_013547**	5.37	1.58E-05	4.86E-05
**AAR2**	0.53	1.59E-05	4.86E-05
**CHODL-AS1**	-2.64	1.59E-05	4.87E-05
**CYFIP2**	1.05	1.59E-05	4.87E-05
**RP11-94C24.8**	-2.33	1.59E-05	4.87E-05
**RP11-107K17.2**	-4.92	1.59E-05	4.88E-05
**RP11-490N5.3**	-5.11	1.59E-05	4.88E-05
**RP11-403H13.1**	-3.90	1.60E-05	4.89E-05
**CTD-2006C1.12**	-1.79	1.60E-05	4.89E-05
**RP11-775J23.2**	-3.89	1.60E-05	4.89E-05
**SCNN1A**	0.83	1.60E-05	4.89E-05
**RP11-330L19.2**	-5.01	1.60E-05	4.89E-05
**ABHD2**	-0.56	1.60E-05	4.89E-05
**CREG2**	3.19	1.60E-05	4.89E-05
**SGCE**	1.41	1.60E-05	4.90E-05
**RP5-997D24.3**	-5.27	1.60E-05	4.90E-05
**RP11-35J10.6**	-2.82	1.61E-05	4.92E-05
**SUV39H1**	0.70	1.62E-05	4.94E-05
**G12578**	-2.21	1.62E-05	4.95E-05
**TTLL7-IT1**	-5.34	1.62E-05	4.95E-05
**RP11-436M15.1**	-4.92	1.62E-05	4.96E-05
**RP11-139H15.6**	-2.81	1.62E-05	4.96E-05
**CTB-73N10.1**	-5.22	1.62E-05	4.97E-05
**SYT14P1**	-5.24	1.63E-05	4.97E-05

**RPL23AP18**	-4.86	1.63E-05	4.98E-05
**CAPN15**	0.75	1.63E-05	4.98E-05
**G10909**	-1.71	1.63E-05	4.99E-05
**AC007556.3**	-4.92	1.64E-05	5.00E-05
**EREG**	-1.62	1.64E-05	5.01E-05
**RP11-511I11.2**	-5.18	1.64E-05	5.01E-05
**RP11-56B16.6**	-4.84	1.64E-05	5.01E-05
**NOL10**	-0.40	1.64E-05	5.01E-05
**CCDC93**	-0.40	1.64E-05	5.02E-05
**CEP164**	0.70	1.64E-05	5.02E-05
**RP11-119D9.1**	-2.31	1.64E-05	5.02E-05
**MIR181B2**	-4.89	1.64E-05	5.02E-05
**TRAPPC13**	-0.63	1.65E-05	5.03E-05
**C9orf16**	0.84	1.65E-05	5.03E-05
**CBY1**	0.55	1.65E-05	5.04E-05
**RP11-554I8.2**	3.23	1.65E-05	5.05E-05
**ASCL2**	0.95	1.66E-05	5.06E-05
**KDM3A**	-0.64	1.66E-05	5.07E-05
**TIGD4**	-2.14	1.66E-05	5.07E-05
**JDP2**	0.77	1.66E-05	5.08E-05
**GATS**	-0.98	1.67E-05	5.08E-05
**RGS9BP**	-3.21	1.67E-05	5.08E-05
**NUP160**	-0.45	1.67E-05	5.09E-05
**CTC-360G5.9**	-1.59	1.67E-05	5.10E-05
**KCNH1**	2.30	1.67E-05	5.10E-05
**DYNLT3**	0.96	1.67E-05	5.10E-05
**PGBD1**	1.20	1.67E-05	5.10E-05
**G2927**	-4.85	1.67E-05	5.10E-05
**FSTL4**	-1.27	1.67E-05	5.11E-05
**RP11-214K3.20**	-3.35	1.68E-05	5.11E-05
**G7527**	-2.54	1.68E-05	5.11E-05
**TNFSF13B**	1.70	1.68E-05	5.11E-05
**SFR1**	0.58	1.68E-05	5.11E-05
**TTK**	-1.02	1.68E-05	5.12E-05
**STX18**	0.32	1.68E-05	5.12E-05
**RP11-505K9.1**	1.92	1.68E-05	5.13E-05
**GATA2-AS1**	2.56	1.68E-05	5.13E-05
**AP000254.8**	-0.95	1.68E-05	5.13E-05
**RP11-640B6.1**	-4.86	1.69E-05	5.13E-05
**CH507-42P11.8**	-3.58	1.69E-05	5.14E-05
**RP11-298J20.3**	-2.22	1.69E-05	5.14E-05
**SLC35G6**	-4.91	1.69E-05	5.15E-05
**ING3**	-0.50	1.69E-05	5.16E-05
**RP11-173P15.9**	-2.29	1.69E-05	5.16E-05
**RHBDD1**	-0.46	1.69E-05	5.16E-05

**ULBP1**	2.92	1.70E-05	5.17E-05
**EBLN3**	-0.45	1.70E-05	5.17E-05
**RMDN3**	0.60	1.70E-05	5.17E-05
**CCDC151**	2.22	1.70E-05	5.18E-05
**SHROOM4**	1.07	1.71E-05	5.20E-05
**RP11-269G24.8**	-5.04	1.71E-05	5.21E-05
**RP11-531A24.7**	-5.21	1.71E-05	5.21E-05
**RP11-932O9.7**	-3.94	1.72E-05	5.24E-05
**ACOT9**	0.75	1.72E-05	5.24E-05
**SETDB1**	-0.35	1.72E-05	5.24E-05
**MIR576**	-4.82	1.73E-05	5.25E-05
**LINC00592**	1.99	1.73E-05	5.26E-05
**SNORD53_SNORD92**	-4.25	1.73E-05	5.27E-05
**AP000998.2**	-3.05	1.73E-05	5.27E-05
**LRRFIP2**	-0.55	1.74E-05	5.28E-05
**YY1AP1**	-0.43	1.74E-05	5.28E-05
**PCSK2**	-1.50	1.74E-05	5.28E-05
**G18345**	-5.15	1.74E-05	5.29E-05
**LCT**	-4.00	1.74E-05	5.29E-05
**CTBS**	-0.70	1.74E-05	5.29E-05
**CD247**	1.56	1.74E-05	5.30E-05
**CALN1**	-1.68	1.75E-05	5.30E-05
**CTA-407F11.7**	-4.88	1.75E-05	5.31E-05
**MAPRE1**	0.56	1.75E-05	5.31E-05
**ANXA2R**	1.58	1.75E-05	5.32E-05
**RP11-378J18.6**	-4.77	1.76E-05	5.34E-05
**RP11-668G10.2**	-4.74	1.76E-05	5.34E-05
**G13086**	-2.27	1.76E-05	5.34E-05
**ROBO2**	-1.27	1.76E-05	5.34E-05
**CTD-2337I7.1**	-2.96	1.76E-05	5.35E-05
**CEP57**	-0.42	1.76E-05	5.35E-05
**MRPS6P2**	-4.90	1.76E-05	5.35E-05
**SPRR4**	-3.72	1.76E-05	5.35E-05
**IKZF5**	-0.60	1.76E-05	5.35E-05
**CHL1**	-1.50	1.77E-05	5.36E-05
**NDNL2**	0.58	1.77E-05	5.37E-05
**CTD-3185P2.1**	-1.61	1.77E-05	5.37E-05
**TERF2**	0.60	1.77E-05	5.37E-05
**CTSF**	0.81	1.78E-05	5.39E-05
**G25089**	-4.24	1.78E-05	5.40E-05
**TCF4-AS2**	-4.88	1.78E-05	5.40E-05
**RNF220**	0.45	1.78E-05	5.41E-05
**VDAC3**	0.51	1.79E-05	5.42E-05
**PROCR**	0.93	1.79E-05	5.42E-05
**SCG5**	2.02	1.79E-05	5.42E-05

**RP11-182I10.1**	-5.24	1.79E-05	5.42E-05
**RPS6P16**	-4.97	1.79E-05	5.43E-05
**TMED7**	-0.84	1.80E-05	5.45E-05
**CTD-3065B20.2**	-2.54	1.80E-05	5.45E-05
**TMEM55A**	-0.95	1.81E-05	5.48E-05
**HRH1**	0.98	1.81E-05	5.48E-05
**RP5-1132H15.1**	-2.73	1.81E-05	5.49E-05
**BHLHB9**	-0.69	1.81E-05	5.49E-05
**ABL1**	0.76	1.82E-05	5.50E-05
**RNU4-78P**	-4.63	1.82E-05	5.50E-05
**AC021078.1**	-5.13	1.82E-05	5.50E-05
**RP11-293A21.2**	-4.91	1.82E-05	5.51E-05
**RP11-474L23.3**	-5.14	1.82E-05	5.52E-05
**AGBL3**	-0.99	1.83E-05	5.53E-05
**RP11-701H24.8**	-4.69	1.83E-05	5.53E-05
**ODF3**	-4.86	1.84E-05	5.56E-05
**UBP1**	0.45	1.84E-05	5.56E-05
**AC094019.4**	-3.70	1.84E-05	5.57E-05
**PHF2P2**	-2.64	1.85E-05	5.58E-05
**HSP90B1**	0.80	1.85E-05	5.59E-05
**DNAJC19P7**	-5.21	1.86E-05	5.62E-05
**RP1-85F18.6**	-4.78	1.86E-05	5.62E-05
**SFTPB**	-5.02	1.86E-05	5.62E-05
**AC000123.3**	-2.06	1.86E-05	5.62E-05
**COL28A1**	-1.84	1.86E-05	5.62E-05
**MTCO3P2**	-4.93	1.86E-05	5.63E-05
**SERINC3**	-0.53	1.86E-05	5.63E-05
**RP11-83B20.5**	-5.20	1.86E-05	5.63E-05
**RP11-197P3.5**	-2.32	1.87E-05	5.65E-05
**RPSA**	0.66	1.87E-05	5.65E-05
**PSG7**	-2.21	1.87E-05	5.66E-05
**RTP4**	1.98	1.87E-05	5.66E-05
**ADAMTS18**	2.91	1.87E-05	5.67E-05
**UCKL1-AS1**	-2.79	1.88E-05	5.67E-05
**ZNF135**	-0.97	1.88E-05	5.68E-05
**WFDC12**	2.02	1.88E-05	5.68E-05
**AC010980.2**	1.97	1.88E-05	5.68E-05
**RP11-247A12.2**	2.40	1.88E-05	5.68E-05
**NR4A2**	1.14	1.89E-05	5.70E-05
**AC078864.2**	-4.85	1.89E-05	5.70E-05
**RPL7AP10**	-2.24	1.89E-05	5.70E-05
**LCE2A**	1.67	1.89E-05	5.71E-05
**RN7SL430P**	-5.01	1.89E-05	5.71E-05
**RP4-791M13.4**	-4.85	1.89E-05	5.71E-05
**PCDHGB5**	-0.73	1.89E-05	5.72E-05

**PDLIM3**	1.17	1.90E-05	5.72E-05
**RP11-207C16.4**	-4.99	1.90E-05	5.73E-05
**ITGAE**	0.59	1.91E-05	5.76E-05
**RNU4-35P**	-4.88	1.91E-05	5.76E-05
**NOD1**	0.66	1.91E-05	5.77E-05
**SKP1**	-0.48	1.92E-05	5.78E-05
**LYSMD2**	0.77	1.92E-05	5.79E-05
**RP11-553D4.2**	-4.88	1.92E-05	5.80E-05
**EIF4A2P2**	-4.01	1.93E-05	5.81E-05
**GRM4**	3.84	1.93E-05	5.82E-05
**STARD13-IT1**	-5.10	1.93E-05	5.83E-05
**GAPDHP61**	-4.25	1.94E-05	5.84E-05
**CTD-2201E9.2**	-4.99	1.94E-05	5.84E-05
**AC091132.1**	-2.87	1.94E-05	5.84E-05
**NCOA7-AS1**	-5.14	1.94E-05	5.85E-05
**FBXO44**	0.93	1.95E-05	5.87E-05
**SNORA57**	-4.75	1.95E-05	5.87E-05
**HMGB3P14**	-2.77	1.95E-05	5.88E-05
**PRO1804**	-2.11	1.95E-05	5.89E-05
**FAM222A-AS1**	-4.90	1.96E-05	5.90E-05
**PIK3IP1**	1.00	1.96E-05	5.90E-05
**RP11-401P9.1**	-4.63	1.96E-05	5.90E-05
**G3405**	-4.74	1.96E-05	5.91E-05
**SLC38A3**	3.61	1.96E-05	5.92E-05
**RP13-635I23.3**	-5.08	1.97E-05	5.92E-05
**ZSWIM6**	0.49	1.97E-05	5.93E-05
**LRRC40**	-0.67	1.97E-05	5.93E-05
**ARHGEF40**	0.96	1.97E-05	5.94E-05
**MIR571**	-4.83	1.97E-05	5.94E-05
**SDHCP3**	-4.73	1.97E-05	5.94E-05
**USP17L7**	-5.28	1.97E-05	5.94E-05
**TEN1**	-4.98	1.98E-05	5.95E-05
**RRP15**	-0.54	1.98E-05	5.95E-05
**DIRAS2**	-1.81	1.98E-05	5.97E-05
**RP11-305O4.3**	-4.95	1.99E-05	5.97E-05
**CHCHD4P5**	-5.13	1.99E-05	5.98E-05
**AC108218.1**	-5.10	1.99E-05	5.98E-05
**G25302**	-5.34	1.99E-05	5.99E-05
**AC019186.1**	-3.44	1.99E-05	5.99E-05
**PHF2**	0.32	1.99E-05	5.99E-05
**XYLT1**	-0.68	1.99E-05	5.99E-05
**LENEP**	-4.87	1.99E-05	5.99E-05
**XLOC_008642**	-3.44	1.99E-05	5.99E-05
**RNU6-652P**	-2.49	2.00E-05	6.02E-05
**C2orf88**	-1.18	2.01E-05	6.04E-05

**CHFR**	0.41	2.01E-05	6.04E-05
**L3MBTL1**	-1.38	2.01E-05	6.05E-05
**ZNF471**	-1.00	2.01E-05	6.05E-05
**TCEB1P19**	-2.18	2.01E-05	6.05E-05
**MYH16**	-2.38	2.01E-05	6.05E-05
**SNORA24**	-4.91	2.02E-05	6.06E-05
**NRIP1**	-0.69	2.02E-05	6.07E-05
**LINC00853**	2.72	2.03E-05	6.09E-05
**TLE1**	0.56	2.03E-05	6.10E-05
**PNRC1**	-0.63	2.04E-05	6.12E-05
**NCF1**	2.48	2.04E-05	6.12E-05
**KRT31**	-1.66	2.04E-05	6.12E-05
**AC009303.2**	2.32	2.05E-05	6.14E-05
**KIF26B**	1.24	2.05E-05	6.16E-05
**IL1R2**	-1.67	2.05E-05	6.16E-05
**RNF19A**	-0.53	2.06E-05	6.17E-05
**METTL3**	-0.71	2.06E-05	6.18E-05
**ARFGEF3**	-1.54	2.06E-05	6.19E-05
**LINC01612**	-6.36	2.06E-05	6.20E-05
**SH3GL1P2**	-3.34	2.07E-05	6.20E-05
**AC092614.2**	2.96	2.07E-05	6.22E-05
**C1orf194**	-3.55	2.08E-05	6.23E-05
**CTB-174O21.2**	-4.66	2.08E-05	6.23E-05
**DYNC1I2**	0.55	2.08E-05	6.24E-05
**USP13**	-0.53	2.08E-05	6.24E-05
**MIR5692A1**	-4.88	2.08E-05	6.25E-05
**RP11-98C1.2**	-4.66	2.09E-05	6.27E-05
**PON3**	-1.35	2.09E-05	6.27E-05
**CADM2**	-1.97	2.10E-05	6.29E-05
**ARNTL2-AS1**	-3.08	2.10E-05	6.29E-05
**HNF4A**	-4.65	2.10E-05	6.30E-05
**PIAS3**	0.62	2.10E-05	6.30E-05
**RN7SL418P**	-5.00	2.11E-05	6.31E-05
**RNA5SP88**	-4.90	2.11E-05	6.32E-05
**LAG3**	1.89	2.11E-05	6.32E-05
**SUOX**	1.05	2.11E-05	6.33E-05
**FAM58A**	0.62	2.12E-05	6.35E-05
**RP11-395N21.2**	-4.63	2.12E-05	6.35E-05
**AC007969.5**	1.20	2.12E-05	6.35E-05
**LDB1**	0.66	2.12E-05	6.35E-05
**ZBTB8OSP1**	-5.15	2.12E-05	6.35E-05
**JMJD8**	0.56	2.12E-05	6.35E-05
**ITGAV**	-0.77	2.12E-05	6.36E-05
**ZNF790-AS1**	-1.12	2.13E-05	6.37E-05
**ZSWIM8**	0.58	2.13E-05	6.37E-05

**LA16c-329F2.1**	-4.83	2.13E-05	6.38E-05
**RP11-506N2.1**	-5.53	2.13E-05	6.38E-05
**PSME4**	-0.54	2.13E-05	6.38E-05
**RP11-494O16.4**	-4.88	2.14E-05	6.39E-05
**GZMA**	2.55	2.14E-05	6.40E-05
**MIR4729**	-4.96	2.14E-05	6.41E-05
**RP11-711M9.2**	-5.13	2.14E-05	6.41E-05
**RP11-158L12.4**	-1.47	2.15E-05	6.42E-05
**PCBP2P1**	-3.87	2.15E-05	6.42E-05
**MIR5581**	-4.66	2.15E-05	6.42E-05
**CTD-2146O16.1**	-5.10	2.15E-05	6.43E-05
**DTX2**	0.91	2.16E-05	6.45E-05
**ORAI3**	0.65	2.16E-05	6.45E-05
**AL138751.1**	-5.03	2.16E-05	6.45E-05
**PLEKHG1**	-0.64	2.16E-05	6.47E-05
**CTD-2619J13.8**	2.55	2.17E-05	6.47E-05
**COL1A2**	2.54	2.17E-05	6.49E-05
**CLCN2**	0.99	2.17E-05	6.50E-05
**LACTB2-AS1**	-2.00	2.18E-05	6.50E-05
**PRKAG2**	0.49	2.18E-05	6.50E-05
**BACE1**	0.52	2.18E-05	6.51E-05
**SNORA60**	-2.72	2.18E-05	6.52E-05
**NDUFA3P1**	-5.07	2.18E-05	6.52E-05
**RP11-298C2.1**	-3.10	2.19E-05	6.53E-05
**CHAD**	-1.53	2.19E-05	6.54E-05
**CDHR2**	-1.87	2.19E-05	6.55E-05
**NPM1P9**	-5.02	2.20E-05	6.56E-05
**THRB-AS1**	-1.93	2.20E-05	6.56E-05
**NXPH3**	1.71	2.20E-05	6.57E-05
**PADI4**	-5.22	2.20E-05	6.57E-05
**TBC1D23**	-0.61	2.20E-05	6.58E-05
**FRG1**	0.70	2.21E-05	6.59E-05
**KRT8P12**	0.92	2.21E-05	6.59E-05
**FAM136A**	0.41	2.21E-05	6.59E-05
**RP11-573E11.2**	-5.21	2.21E-05	6.60E-05
**TMEM75**	-3.87	2.22E-05	6.61E-05
**RNU1-120P**	-3.76	2.22E-05	6.63E-05
**CARD16**	1.15	2.23E-05	6.64E-05
**CTC-338M12.6**	-2.79	2.23E-05	6.64E-05
**POMT1**	0.67	2.23E-05	6.64E-05
**CCR1**	2.40	2.23E-05	6.64E-05
**Z85986.1**	-5.10	2.23E-05	6.65E-05
**CCL24**	2.87	2.23E-05	6.66E-05
**RP11-767C1.2**	-1.70	2.24E-05	6.68E-05
**TYR**	-1.54	2.24E-05	6.68E-05

**RP11-404P21.5**	-3.11	2.26E-05	6.72E-05
**ARHGEF3-AS1**	-4.87	2.26E-05	6.72E-05
**RP11-89N17.4**	-4.67	2.26E-05	6.74E-05
**CTD-2516F10.2**	-1.62	2.26E-05	6.74E-05
**FPR1**	2.58	2.27E-05	6.75E-05
**RP11-21B21.4**	-3.20	2.28E-05	6.78E-05
**C7orf71**	-4.75	2.28E-05	6.78E-05
**RP11-325L12.3**	-4.86	2.28E-05	6.79E-05
**AC018804.6**	3.03	2.29E-05	6.81E-05
**MAP3K19**	-4.61	2.29E-05	6.81E-05
**RP11-320L11.2**	-1.20	2.29E-05	6.81E-05
**NRL**	1.15	2.29E-05	6.82E-05
**GS1-25M2.1**	-4.96	2.29E-05	6.83E-05
**RP11-118F19.1**	-2.50	2.30E-05	6.83E-05
**TMEM261**	0.69	2.30E-05	6.84E-05
**RP11-489G11.3**	-3.98	2.30E-05	6.84E-05
**RPL7P21**	-3.29	2.30E-05	6.84E-05
**UBAC1**	0.41	2.30E-05	6.84E-05
**OSBPL9P4**	-5.07	2.30E-05	6.85E-05
**NSRP1P1**	2.16	2.30E-05	6.85E-05
**EIF3D**	0.48	2.30E-05	6.85E-05
**MOGAT1**	-5.19	2.30E-05	6.85E-05
**DNASE2**	0.66	2.31E-05	6.86E-05
**COX10**	0.47	2.31E-05	6.86E-05
**SUMO2P8**	-4.63	2.31E-05	6.87E-05
**SNORD116-23**	-5.45	2.32E-05	6.89E-05
**RP11-310J24.3**	-5.06	2.32E-05	6.89E-05
**RN7SL805P**	-2.46	2.32E-05	6.89E-05
**RNU6-846P**	-5.16	2.32E-05	6.90E-05
**RP11-367G18.1**	-0.92	2.33E-05	6.91E-05
**SYN1**	2.25	2.33E-05	6.92E-05
**G24559**	-2.17	2.33E-05	6.93E-05
**S1PR5**	0.75	2.33E-05	6.94E-05
**GLCE**	-0.76	2.34E-05	6.94E-05
**COQ5**	0.57	2.34E-05	6.94E-05
**C9**	-2.69	2.34E-05	6.96E-05
**WDR59**	0.55	2.34E-05	6.96E-05
**RNU6-522P**	-4.94	2.34E-05	6.96E-05
**RNF31**	0.68	2.34E-05	6.96E-05
**HPCA**	-2.30	2.36E-05	7.00E-05
**IDO2**	-3.99	2.36E-05	7.00E-05
**RPLP0P6**	-0.82	2.36E-05	7.00E-05
**RP11-239C9.1**	-5.33	2.36E-05	7.02E-05
**GNAI1**	-0.71	2.36E-05	7.02E-05
**RP11-64K7.1**	-5.06	2.37E-05	7.03E-05

**NTF4**	1.10	2.37E-05	7.04E-05
**RPS26P21**	-4.73	2.37E-05	7.04E-05
**SLC39A11**	0.59	2.38E-05	7.05E-05
**CH17-373J23.1**	-3.41	2.38E-05	7.07E-05
**RSF1-IT2**	-2.58	2.38E-05	7.07E-05
**POLD4**	0.85	2.38E-05	7.07E-05
**GZMB**	1.97	2.38E-05	7.07E-05
**KISS1R**	3.19	2.38E-05	7.07E-05
**CABP4**	1.86	2.39E-05	7.09E-05
**SKIV2L2**	-0.58	2.40E-05	7.13E-05
**PCDHGA7**	-0.94	2.40E-05	7.13E-05
**ETV1**	2.15	2.41E-05	7.13E-05
**RP11-627K11.6**	-4.69	2.41E-05	7.14E-05
**SRGAP2**	-0.59	2.41E-05	7.14E-05
**RNF212B**	-2.12	2.41E-05	7.15E-05
**AC069282.6**	-1.09	2.42E-05	7.16E-05
**RP3-429O6.1**	-5.92	2.42E-05	7.16E-05
**RBM41**	-0.49	2.42E-05	7.16E-05
**KLHL2**	0.48	2.42E-05	7.18E-05
**RP11-128N14.4**	-4.57	2.42E-05	7.18E-05
**AL591377.1**	-4.89	2.43E-05	7.19E-05
**SH2D3A**	0.82	2.43E-05	7.21E-05
**SH3BP4**	0.49	2.44E-05	7.21E-05
**RP11-401L13.4**	-4.93	2.44E-05	7.23E-05
**BDKRB2**	0.86	2.44E-05	7.23E-05
**RP5-836N17.4**	-4.56	2.44E-05	7.23E-05
**GRIFIN**	-4.64	2.45E-05	7.26E-05
**AC018462.2**	-1.70	2.45E-05	7.27E-05
**MTURN**	-0.66	2.46E-05	7.28E-05
**CTC-458I2.2**	-2.58	2.46E-05	7.29E-05
**AC009518.8**	-4.15	2.46E-05	7.29E-05
**ELMOD1**	-1.35	2.47E-05	7.29E-05
**OGG1**	0.82	2.47E-05	7.30E-05
**IMMP1LP1**	-2.54	2.47E-05	7.30E-05
**CCL8**	3.93	2.47E-05	7.30E-05
**FAM83A-AS1**	2.29	2.47E-05	7.30E-05
**ZFP1**	-0.69	2.47E-05	7.31E-05
**RP5-1073F15.1**	-4.86	2.48E-05	7.32E-05
**RP11-677M14.6**	-3.55	2.48E-05	7.34E-05
**G16028**	-2.67	2.49E-05	7.35E-05
**TRPV6**	-1.51	2.50E-05	7.39E-05
**FAM102B**	-0.71	2.50E-05	7.40E-05
**CYP26A1**	7.51	2.51E-05	7.41E-05
**RP11-85K15.3**	-1.89	2.51E-05	7.43E-05
**LYG2**	-3.88	2.52E-05	7.44E-05

**ARPC1A**	0.49	2.52E-05	7.45E-05
**PYGO1**	-1.04	2.52E-05	7.46E-05
**KLHL9**	-0.45	2.53E-05	7.46E-05
**CTD-2381F24.1**	-3.98	2.53E-05	7.48E-05
**NFATC1**	0.99	2.54E-05	7.49E-05
**RNU6-633P**	-4.88	2.54E-05	7.49E-05
**SCO2**	0.87	2.55E-05	7.52E-05
**HIST2H4B**	-4.44	2.55E-05	7.52E-05
**POLHP1**	-4.54	2.55E-05	7.54E-05
**CTB-20D2.1**	-5.26	2.55E-05	7.54E-05
**RP11-117L5.3**	-4.80	2.56E-05	7.55E-05
**MIR5692A2**	-4.80	2.56E-05	7.55E-05
**CHMP7**	0.48	2.56E-05	7.56E-05
**XLOC_005041**	-2.96	2.56E-05	7.56E-05
**LY86**	1.95	2.56E-05	7.56E-05
**RP11-567L7.3**	-4.00	2.57E-05	7.59E-05
**CTD-2626G11.2**	-1.81	2.57E-05	7.59E-05
**SMARCAL1**	0.56	2.57E-05	7.59E-05
**OR5E1P**	-3.23	2.58E-05	7.60E-05
**TAS2R20**	-2.19	2.58E-05	7.61E-05
**RP4-539M6.21**	-4.95	2.58E-05	7.62E-05
**RP11-534L20.5**	-2.75	2.59E-05	7.62E-05
**HTRA3**	2.08	2.59E-05	7.63E-05
**DNAJC28**	-0.77	2.59E-05	7.64E-05
**C16orf59**	1.19	2.60E-05	7.66E-05
**ANKRD13C**	-0.63	2.61E-05	7.69E-05
**RP11-397P13.7**	-3.25	2.61E-05	7.69E-05
**KIF3B**	0.47	2.61E-05	7.69E-05
**GLB1L3**	1.04	2.61E-05	7.70E-05
**RP11-481F24.3**	-2.95	2.61E-05	7.70E-05
**MAPRE3**	0.60	2.61E-05	7.70E-05
**CDKN2C**	0.64	2.62E-05	7.71E-05
**RP11-723O4.7**	-3.62	2.62E-05	7.71E-05
**MS4A4A**	2.42	2.62E-05	7.71E-05
**LPCAT3**	0.76	2.62E-05	7.72E-05
**NAALADL2**	-1.11	2.62E-05	7.73E-05
**RP1-168P16.2**	-3.34	2.63E-05	7.74E-05
**RP11-18B3.2**	-4.67	2.63E-05	7.75E-05
**KCTD3**	-0.43	2.63E-05	7.76E-05
**RP11-47J17.3**	-5.18	2.64E-05	7.76E-05
**LRRN4**	-3.21	2.64E-05	7.78E-05
**THSD1**	0.90	2.65E-05	7.80E-05
**AC010733.5**	-1.43	2.65E-05	7.80E-05
**SLF1**	-0.60	2.65E-05	7.80E-05
**RP11-195F19.30**	-2.58	2.65E-05	7.80E-05

**GALNT5**	2.00	2.65E-05	7.81E-05
**REEP2**	1.53	2.66E-05	7.84E-05
**MAF**	0.61	2.66E-05	7.84E-05
**TMEM88**	2.38	2.67E-05	7.84E-05
**G1862**	-2.31	2.67E-05	7.84E-05
**OR7M1P**	-3.47	2.67E-05	7.85E-05
**VGLL3**	-1.21	2.67E-05	7.85E-05
**KCNN4**	2.82	2.67E-05	7.86E-05
**RP11-219D15.3**	-2.85	2.68E-05	7.87E-05
**RP4-564F22.7**	-3.19	2.68E-05	7.87E-05
**TMEM167A**	-0.66	2.68E-05	7.88E-05
**RPL13P4**	-4.69	2.68E-05	7.89E-05
**OR2A1-AS1**	-1.77	2.68E-05	7.89E-05
**YIPF1**	0.50	2.69E-05	7.89E-05
**MIR5579**	-4.89	2.69E-05	7.90E-05
**RP5-882C2.2**	1.20	2.69E-05	7.91E-05
**AC005042.4**	-1.48	2.70E-05	7.93E-05
**C1orf146**	-4.48	2.70E-05	7.94E-05
**CDRT4**	-2.51	2.72E-05	7.99E-05
**HLA-A**	1.08	2.73E-05	8.02E-05
**XLOC_013506**	-2.11	2.74E-05	8.04E-05
**RP11-47I22.1**	-2.01	2.74E-05	8.05E-05
**NUTM2G**	-1.55	2.74E-05	8.05E-05
**RNU6-444P**	-5.02	2.75E-05	8.07E-05
**RP13-735L24.1**	-1.69	2.75E-05	8.07E-05
**THG1L**	0.63	2.75E-05	8.08E-05
**FAM135B**	-4.59	2.76E-05	8.09E-05
**AC011247.3**	-3.42	2.76E-05	8.10E-05
**TEPP**	2.89	2.77E-05	8.12E-05
**RP11-165F24.3**	-1.76	2.77E-05	8.13E-05
**RP11-737O24.2**	-4.82	2.79E-05	8.19E-05
**PIGT**	0.72	2.79E-05	8.19E-05
**SOD1**	0.72	2.79E-05	8.19E-05
**RP13-1016M1.2**	4.72	2.79E-05	8.20E-05
**STEAP1B**	2.36	2.80E-05	8.20E-05
**AC060226.1**	-5.20	2.80E-05	8.20E-05
**RP11-158M2.5**	-3.35	2.80E-05	8.20E-05
**PDE7A**	0.84	2.80E-05	8.21E-05
**ZNRD1**	0.65	2.80E-05	8.22E-05
**FZD4**	1.25	2.81E-05	8.24E-05
**C3**	2.53	2.81E-05	8.25E-05
**LINC01547**	1.16	2.83E-05	8.29E-05
**RPL7AP30**	-1.53	2.83E-05	8.30E-05
**RP11-143K11.7**	-3.96	2.83E-05	8.30E-05
**GPR155**	-0.69	2.83E-05	8.30E-05

**RP11-403I13.4**	-3.30	2.83E-05	8.30E-05
**G40462**	3.14	2.84E-05	8.32E-05
**PLAA**	-0.48	2.84E-05	8.32E-05
**CTD-2544H17.1**	-5.33	2.85E-05	8.35E-05
**SERGEF**	0.45	2.85E-05	8.36E-05
**POU3F1**	0.83	2.85E-05	8.36E-05
**NHSL1**	-0.52	2.86E-05	8.37E-05
**SOX9**	1.80	2.86E-05	8.38E-05
**RP11-113I22.1**	-5.27	2.86E-05	8.38E-05
**RP11-225B17.2**	1.34	2.86E-05	8.38E-05
**LINC00472**	-1.87	2.86E-05	8.38E-05
**CASP6**	0.65	2.86E-05	8.38E-05
**MIR5009**	-4.82	2.86E-05	8.39E-05
**RP11-15L13.4**	-2.40	2.87E-05	8.39E-05
**RP11-216B9.8**	-5.06	2.87E-05	8.39E-05
**RP11-26J3.3**	-0.96	2.87E-05	8.39E-05
**TBK1**	-0.55	2.87E-05	8.41E-05
**RP11-161H23.10**	-3.00	2.88E-05	8.41E-05
**APTR**	0.65	2.88E-05	8.41E-05
**MIRLET7BHG**	-1.49	2.88E-05	8.42E-05
**PDHX**	-0.59	2.88E-05	8.42E-05
**RP11-782C8.5**	-3.32	2.88E-05	8.43E-05
**AC002467.7**	1.31	2.88E-05	8.44E-05
**DMBX1**	-5.82	2.89E-05	8.44E-05
**NR2C2AP**	0.65	2.89E-05	8.44E-05
**GNAO1**	1.07	2.89E-05	8.44E-05
**TRNAU1AP**	0.49	2.89E-05	8.45E-05
**EMC3**	0.47	2.89E-05	8.45E-05
**S100PBP**	-0.49	2.89E-05	8.45E-05
**MIR4505**	-4.52	2.89E-05	8.46E-05
**ATP5L2**	-2.76	2.89E-05	8.46E-05
**PEF1**	0.39	2.90E-05	8.48E-05
**GLRA1**	-4.94	2.90E-05	8.48E-05
**XLOC_007800**	-3.38	2.91E-05	8.50E-05
**CARF**	-0.75	2.92E-05	8.52E-05
**ZNF92P3**	-2.94	2.92E-05	8.53E-05
**BATF3**	2.35	2.92E-05	8.53E-05
**APOOL**	-0.68	2.92E-05	8.53E-05
**RNY4P17**	-3.79	2.92E-05	8.54E-05
**PAIP1P1**	-2.00	2.93E-05	8.55E-05
**XLOC_008269**	-4.70	2.93E-05	8.55E-05
**TMEM14C**	0.67	2.93E-05	8.55E-05
**NUP50**	-0.42	2.93E-05	8.56E-05
**G35698**	-1.61	2.93E-05	8.56E-05
**TPST2P1**	-4.83	2.94E-05	8.58E-05

**USP17L2**	-5.10	2.94E-05	8.58E-05
**ZSCAN22**	-0.67	2.95E-05	8.60E-05
**BACH1-IT3**	-4.58	2.95E-05	8.60E-05
**RNF138P1**	-4.74	2.95E-05	8.60E-05
**SCML1**	-0.74	2.95E-05	8.60E-05
**AC013429.5**	-4.94	2.95E-05	8.60E-05
**DHRS4**	0.96	2.95E-05	8.60E-05
**RP11-417E7.2**	4.72	2.95E-05	8.61E-05
**CTD-2017C7.3**	-4.93	2.96E-05	8.62E-05
**CTC-325H20.8**	-5.12	2.96E-05	8.63E-05
**COPZ1**	0.51	2.98E-05	8.68E-05
**SNORD72**	-4.02	2.98E-05	8.68E-05
**GSC**	3.21	2.98E-05	8.70E-05
**EMBP1**	-1.89	2.98E-05	8.70E-05
**FAM89A**	0.92	2.98E-05	8.70E-05
**RP11-325L12.6**	4.20	2.99E-05	8.71E-05
**FBXO11**	-0.41	3.00E-05	8.73E-05
**AP001055.6**	2.38	3.00E-05	8.73E-05
**PSG9**	-4.63	3.00E-05	8.74E-05
**RANP3**	-4.62	3.00E-05	8.75E-05
**C16orf62**	0.32	3.02E-05	8.78E-05
**MRPL23**	0.51	3.02E-05	8.80E-05
**ZNF350-AS1**	-2.98	3.02E-05	8.81E-05
**SLC44A4**	2.70	3.03E-05	8.83E-05
**AC011431.1**	-5.12	3.03E-05	8.83E-05
**C17orf80**	-0.52	3.03E-05	8.83E-05
**AC016738.4**	-2.60	3.04E-05	8.84E-05
**AGFG2**	-0.49	3.04E-05	8.85E-05
**RP5-943J3.2**	2.42	3.04E-05	8.86E-05
**ZNF100**	-0.90	3.05E-05	8.87E-05
**RP11-90L1.8**	1.05	3.05E-05	8.87E-05
**RPS15AP12**	-3.09	3.05E-05	8.88E-05
**AC012065.5**	-4.00	3.06E-05	8.90E-05
**RP3-355L5.3**	-4.53	3.06E-05	8.90E-05
**SFRP2**	2.56	3.06E-05	8.90E-05
**MIR1255A**	-5.04	3.06E-05	8.90E-05
**ADAM8**	1.29	3.06E-05	8.91E-05
**RAB3IP**	-0.86	3.07E-05	8.92E-05
**RP3-406A7.5**	-4.54	3.07E-05	8.93E-05
**ATP6V1B2**	0.51	3.07E-05	8.93E-05
**RP11-100G15.3**	-4.32	3.08E-05	8.94E-05
**RP11-2J18.1**	-2.19	3.08E-05	8.95E-05
**RP11-689P11.3**	-4.51	3.08E-05	8.95E-05
**ARHGAP17**	0.78	3.08E-05	8.96E-05
**RP11-178L8.9**	-2.30	3.09E-05	8.97E-05

**ENY2**	0.52	3.09E-05	8.98E-05
**PPIAP2**	-4.76	3.10E-05	9.00E-05
**CR1**	2.71	3.10E-05	9.00E-05
**VAX2**	3.36	3.10E-05	9.00E-05
**ASB6**	0.51	3.10E-05	9.00E-05
**ZNF385D-AS2**	-6.14	3.10E-05	9.00E-05
**GNG5**	0.53	3.10E-05	9.00E-05
**SLC13A4**	-1.28	3.10E-05	9.01E-05
**C1orf158**	-6.48	3.11E-05	9.02E-05
**RP11-276H1.3**	-2.98	3.11E-05	9.04E-05
**GTF2IP4**	-0.90	3.11E-05	9.04E-05
**LARP4B**	-0.50	3.12E-05	9.05E-05
**AP000462.3**	-4.90	3.12E-05	9.05E-05
**SNORD59A**	-3.38	3.13E-05	9.08E-05
**RP11-631N16.2**	-1.21	3.13E-05	9.08E-05
**UCP2**	1.45	3.13E-05	9.09E-05
**RP11-411G7.2**	-5.09	3.13E-05	9.09E-05
**RORB**	-2.09	3.13E-05	9.09E-05
**XLOC_004631**	4.92	3.13E-05	9.09E-05
**CDC42EP2**	1.24	3.14E-05	9.12E-05
**IZUMO1**	-2.34	3.14E-05	9.12E-05
**RNU1-100P**	-5.01	3.15E-05	9.13E-05
**MTND5P2**	-4.90	3.15E-05	9.14E-05
**GAPDHP60**	-3.17	3.15E-05	9.15E-05
**FOPNL**	-0.69	3.15E-05	9.15E-05
**PVRL1**	0.87	3.16E-05	9.16E-05
**CYP2J2**	-1.32	3.16E-05	9.16E-05
**RP11-486P11.1**	-4.65	3.17E-05	9.18E-05
**HSD3BP4**	-3.51	3.17E-05	9.18E-05
**GPR174**	-3.22	3.18E-05	9.20E-05
**STRAP**	0.59	3.18E-05	9.21E-05
**RPL31P52**	-4.42	3.19E-05	9.23E-05
**THSD7A**	1.15	3.19E-05	9.24E-05
**NCAPG**	-0.91	3.19E-05	9.24E-05
**BAG1**	0.52	3.19E-05	9.25E-05
**ENPP7P10**	-3.66	3.20E-05	9.26E-05
**RP11-67K19.3**	-5.00	3.20E-05	9.28E-05
**ITPKB-AS1**	-4.83	3.21E-05	9.29E-05
**SPATA20P1**	5.52	3.21E-05	9.30E-05
**AATK**	1.28	3.21E-05	9.30E-05
**UACA**	0.65	3.21E-05	9.31E-05
**NDUFB8P2**	-4.78	3.22E-05	9.32E-05
**XIST**	-4.63	3.22E-05	9.32E-05
**AC002551.1**	-4.73	3.22E-05	9.33E-05
**RPL7P46**	-4.70	3.22E-05	9.34E-05

**CCDC28A**	0.58	3.23E-05	9.34E-05
**CBR4**	-0.56	3.23E-05	9.34E-05
**RP11-409K20.6**	-4.91	3.23E-05	9.35E-05
**NPTXR**	1.20	3.23E-05	9.35E-05
**G29699**	-3.81	3.24E-05	9.39E-05
**AF011889.5**	-2.32	3.25E-05	9.39E-05
**RP11-5G9.5**	-2.07	3.25E-05	9.41E-05
**CMAS**	0.56	3.25E-05	9.41E-05
**ANAPC7**	0.52	3.25E-05	9.41E-05
**RP11-70D24.1**	-2.52	3.27E-05	9.45E-05
**MRPL50**	-0.59	3.27E-05	9.46E-05
**KRT19**	3.89	3.27E-05	9.46E-05
**G25248**	-2.14	3.27E-05	9.46E-05
**MIR1296**	-4.79	3.28E-05	9.47E-05
**DAGLA**	1.74	3.28E-05	9.48E-05
**ZNF516**	-0.49	3.28E-05	9.48E-05
**RP11-326I11.5**	-1.86	3.28E-05	9.49E-05
**FAM106DP**	-4.88	3.28E-05	9.49E-05
**RP11-447H19.4**	-4.86	3.29E-05	9.51E-05
**SNORD127**	-4.99	3.30E-05	9.52E-05
**BRIX1**	-0.57	3.30E-05	9.52E-05
**RP11-103J8.1**	-4.50	3.30E-05	9.52E-05
**TFAP2A-AS1**	-1.38	3.30E-05	9.53E-05
**AR**	-1.19	3.30E-05	9.53E-05
**FYN**	0.69	3.31E-05	9.55E-05
**RPL23AP83**	-4.65	3.31E-05	9.55E-05
**TPM1**	1.07	3.31E-05	9.55E-05
**C17orf99**	2.59	3.31E-05	9.55E-05
**EFCAB10**	-1.79	3.31E-05	9.56E-05
**PGM3**	-0.50	3.31E-05	9.56E-05
**HHIP**	-3.61	3.31E-05	9.56E-05
**ANKRD13B**	1.05	3.32E-05	9.57E-05
**MARVELD3**	0.96	3.33E-05	9.61E-05
**PAX8-AS1**	-2.33	3.34E-05	9.63E-05
**DGKD**	0.80	3.34E-05	9.64E-05
**RP11-106E7.1**	-4.71	3.35E-05	9.66E-05
**WISP3**	1.40	3.36E-05	9.68E-05
**RP11-423H2.1**	1.44	3.36E-05	9.69E-05
**ZHX1**	-0.68	3.36E-05	9.69E-05
**PLEKHG4**	0.90	3.36E-05	9.70E-05
**SNRPG**	0.78	3.37E-05	9.71E-05
**RP11-1B20.1**	-4.81	3.37E-05	9.71E-05
**RP11-354P11.8**	-4.77	3.37E-05	9.71E-05
**AC019070.1**	-5.07	3.37E-05	9.72E-05
**EAF2**	1.38	3.38E-05	9.73E-05

**CPEB2**	-0.79	3.38E-05	9.75E-05
**PRKAB2**	-0.66	3.39E-05	9.76E-05
**CYB561**	0.53	3.39E-05	9.78E-05
**RP11-296O14.2**	-4.80	3.40E-05	9.80E-05
**MT1A**	2.82	3.40E-05	9.81E-05
**AF064858.8**	-1.59	3.41E-05	9.82E-05
**CH17-340M24.3**	1.56	3.41E-05	9.83E-05
**ETAA1**	-0.53	3.42E-05	9.86E-05
**TOR1B**	0.50	3.42E-05	9.86E-05
**AC004893.10**	-3.96	3.43E-05	9.87E-05
**UBQLN2**	0.52	3.43E-05	9.88E-05
**CEACAM21**	3.09	3.43E-05	9.89E-05
**TGIF1P1**	-4.54	3.44E-05	9.89E-05
**SGSM2**	0.85	3.44E-05	9.90E-05
**CTBP1-AS**	-1.70	3.44E-05	9.90E-05
**GPBP1L1**	-0.49	3.44E-05	9.91E-05
**PRC1-AS1**	-2.45	3.44E-05	9.91E-05
**NEK9**	0.39	3.45E-05	9.92E-05
**PRRT2**	2.92	3.45E-05	9.94E-05
**TPSB2**	2.58	3.45E-05	9.94E-05
**RP11-705C15.2**	-1.24	3.45E-05	9.94E-05
**TNF**	-1.32	3.46E-05	9.94E-05
**FBXL17**	-0.47	3.46E-05	9.94E-05
**CTB-178M22.1**	-4.70	3.46E-05	9.95E-05
**RP11-337A23.5**	-4.36	3.46E-05	9.95E-05
**CTD-2651B20.4**	-4.84	3.47E-05	9.99E-05
**RP11-35G22.1**	-2.25	3.48E-05	1.00E-04
**RP11-371E8.4**	-4.67	3.48E-05	1.00E-04
**RP11-97C16.1**	-1.23	3.48E-05	1.00E-04
**RP11-174F8.1**	-4.79	3.49E-05	1.00E-04
**RP11-481G8.2**	-4.70	3.49E-05	1.00E-04
**THRB**	-0.71	3.49E-05	1.00E-04
**STK39**	1.01	3.50E-05	1.00E-04
**TMEM256P2**	-4.69	3.50E-05	1.01E-04
**ARHGAP33**	1.90	3.50E-05	1.01E-04
**NAPRT**	0.88	3.51E-05	1.01E-04
**RP11-864N7.2**	-1.41	3.51E-05	1.01E-04
**XLOC_009724**	-4.90	3.51E-05	1.01E-04
**RP4-777O23.2**	-4.58	3.51E-05	1.01E-04
**RPS3AP5**	-2.00	3.51E-05	1.01E-04
**KCNRG**	-1.70	3.52E-05	1.01E-04
**RP11-686O6.2**	0.98	3.52E-05	1.01E-04
**RP11-424M24.3**	-4.55	3.54E-05	1.02E-04
**NANOS1**	0.92	3.54E-05	1.02E-04
**PPM1M**	0.77	3.54E-05	1.02E-04

**G35257**	-2.52	3.54E-05	1.02E-04
**RP11-575L7.8**	-3.39	3.55E-05	1.02E-04
**TC2N**	-0.59	3.55E-05	1.02E-04
**WHAMM**	0.45	3.55E-05	1.02E-04
**PDE4B**	1.19	3.55E-05	1.02E-04
**LINC00933**	1.20	3.56E-05	1.02E-04
**ANTXR1**	0.80	3.57E-05	1.02E-04
**RP11-64D24.2**	-4.87	3.57E-05	1.02E-04
**DKK3**	0.79	3.60E-05	1.03E-04
**RP11-498C9.4**	-4.08	3.60E-05	1.03E-04
**NDFIP1**	0.61	3.60E-05	1.03E-04
**LINC00032**	-3.15	3.61E-05	1.04E-04
**CPT2**	0.72	3.61E-05	1.04E-04
**G25631**	-1.96	3.62E-05	1.04E-04
**RNU6-595P**	-3.97	3.62E-05	1.04E-04
**ZMYND19P1**	-4.48	3.62E-05	1.04E-04
**RN7SL844P**	-4.72	3.62E-05	1.04E-04
**POU5F1P4**	-4.81	3.62E-05	1.04E-04
**AC092066.1**	0.90	3.62E-05	1.04E-04
**TMEM180**	-0.74	3.63E-05	1.04E-04
**CTB-186G2.4**	-4.71	3.64E-05	1.04E-04
**AC069213.4**	-4.80	3.64E-05	1.04E-04
**HOXA3**	1.33	3.66E-05	1.05E-04
**ARG1**	1.66	3.66E-05	1.05E-04
**GTF2IP7**	-1.66	3.67E-05	1.05E-04
**RP11-10C24.2**	-1.50	3.68E-05	1.05E-04
**PCBP2**	0.66	3.68E-05	1.05E-04
**SEC14L1P1**	-1.38	3.68E-05	1.06E-04
**OR55B1P**	-4.88	3.72E-05	1.07E-04
**DUTP2**	-4.87	3.73E-05	1.07E-04
**RP11-255H23.4**	1.50	3.73E-05	1.07E-04
**NIPSNAP1**	0.62	3.73E-05	1.07E-04
**AC069155.1**	-3.76	3.74E-05	1.07E-04
**HMGN2P35**	-4.95	3.74E-05	1.07E-04
**HSPA8P19**	-3.33	3.74E-05	1.07E-04
**RP11-329J18.5**	-2.70	3.75E-05	1.07E-04
**RP11-109D20.1**	-4.98	3.76E-05	1.07E-04
**RN7SL242P**	-4.67	3.76E-05	1.08E-04
**RPL21P5**	-3.00	3.76E-05	1.08E-04
**SH3GLB1**	-0.52	3.77E-05	1.08E-04
**ERMP1**	-0.61	3.77E-05	1.08E-04
**GABARAPL1**	0.85	3.78E-05	1.08E-04
**ARG2**	1.23	3.78E-05	1.08E-04
**CLDND1**	-0.61	3.78E-05	1.08E-04
**RP11-401P9.7**	-2.40	3.79E-05	1.08E-04

**ITGB5-AS1**	-3.15	3.79E-05	1.08E-04
**AC092839.3**	-4.81	3.79E-05	1.08E-04
**DCC**	-3.10	3.79E-05	1.08E-04
**RP11-120K19.4**	-2.88	3.79E-05	1.08E-04
**PPP2CB**	0.45	3.80E-05	1.09E-04
**PRELID1P3**	-4.77	3.80E-05	1.09E-04
**RPL22**	0.57	3.81E-05	1.09E-04
**TRIT1**	-0.55	3.81E-05	1.09E-04
**G28650**	-2.38	3.81E-05	1.09E-04
**CMBL**	1.22	3.82E-05	1.09E-04
**RP11-379F4.7**	-1.15	3.83E-05	1.09E-04
**RP11-144C15.2**	-4.39	3.83E-05	1.09E-04
**RP11-574K11.8**	-2.72	3.83E-05	1.09E-04
**SPINK7**	2.92	3.83E-05	1.09E-04
**RP11-592N21.1**	-1.44	3.83E-05	1.09E-04
**PDPK2P**	1.02	3.84E-05	1.10E-04
**C1orf101**	-1.26	3.84E-05	1.10E-04
**RNY4**	-5.21	3.84E-05	1.10E-04
**GSTA3**	-1.97	3.84E-05	1.10E-04
**RAG2**	-4.69	3.85E-05	1.10E-04
**DTWD2**	-0.74	3.85E-05	1.10E-04
**TTTY10**	-1.82	3.85E-05	1.10E-04
**SNORA11B**	-4.77	3.85E-05	1.10E-04
**PTPRT**	-1.83	3.85E-05	1.10E-04
**RNU6V**	-4.69	3.86E-05	1.10E-04
**MRC2**	1.26	3.86E-05	1.10E-04
**CTD-2135D7.2**	-4.36	3.88E-05	1.11E-04
**RPS6KB2**	0.70	3.89E-05	1.11E-04
**RP11-62C3.8**	-4.77	3.89E-05	1.11E-04
**CTD-2349P21.10**	-2.07	3.89E-05	1.11E-04
**RNF216**	0.28	3.90E-05	1.11E-04
**SELK**	0.71	3.90E-05	1.11E-04
**B4GAT1**	0.60	3.90E-05	1.11E-04
**GULOP**	-2.57	3.91E-05	1.11E-04
**AC004461.4**	-2.84	3.92E-05	1.12E-04
**ZSCAN18**	-0.74	3.92E-05	1.12E-04
**MORC4**	-0.46	3.92E-05	1.12E-04
**FCN1**	1.89	3.93E-05	1.12E-04
**PTPRB**	1.17	3.93E-05	1.12E-04
**HLA-F**	1.06	3.93E-05	1.12E-04
**PEX5L**	-2.53	3.93E-05	1.12E-04
**RP11-56H7.2**	-4.83	3.94E-05	1.12E-04
**TRIM55**	-4.90	3.95E-05	1.12E-04
**RPL31P20**	-4.87	3.95E-05	1.12E-04
**AMN1**	-0.73	3.97E-05	1.13E-04

**CH17-264L24.1**	-1.94	3.97E-05	1.13E-04
**SNORD103B**	-4.83	3.99E-05	1.14E-04
**RP11-254F7.2**	1.08	3.99E-05	1.14E-04
**AC093620.5**	-1.62	4.00E-05	1.14E-04
**CTA-204B4.2**	1.15	4.00E-05	1.14E-04
**RP4-794I6.4**	1.65	4.00E-05	1.14E-04
**ZNF195**	-0.51	4.00E-05	1.14E-04
**RP11-286N3.1**	-4.88	4.01E-05	1.14E-04
**CTD-2012K14.7**	-3.76	4.01E-05	1.14E-04
**CBX3P3**	-4.72	4.01E-05	1.14E-04
**ZNF691**	0.78	4.01E-05	1.14E-04
**CSNK1G3**	-0.46	4.02E-05	1.14E-04
**CTD-2313N18.7**	-3.97	4.02E-05	1.14E-04
**HNRNPA1P33**	-1.29	4.03E-05	1.15E-04
**UBLCP1**	0.57	4.03E-05	1.15E-04
**LIPA**	0.84	4.04E-05	1.15E-04
**SLC7A7**	1.54	4.04E-05	1.15E-04
**Z98881.1**	-2.75	4.04E-05	1.15E-04
**CLCN3**	-0.63	4.05E-05	1.15E-04
**OR2A7**	-2.06	4.05E-05	1.15E-04
**AC024560.3**	-1.08	4.05E-05	1.15E-04
**RP11-717A5.1**	-4.87	4.06E-05	1.15E-04
**SLC30A2**	1.44	4.06E-05	1.15E-04
**AC007078.4**	-4.37	4.07E-05	1.16E-04
**RP11-849I19.2**	-2.86	4.08E-05	1.16E-04
**PCDHGA10**	-0.82	4.09E-05	1.16E-04
**RNA5SP297**	-4.84	4.09E-05	1.16E-04
**DPYD-AS2**	-4.84	4.09E-05	1.16E-04
**AC008280.1**	-5.07	4.10E-05	1.16E-04
**SIRPAP1**	-4.30	4.10E-05	1.16E-04
**RP11-478B9.3**	-5.34	4.10E-05	1.16E-04
**CAPS2**	-0.81	4.10E-05	1.16E-04
**RP11-209D14.4**	-4.69	4.11E-05	1.17E-04
**RP11-936I5.1**	-4.13	4.11E-05	1.17E-04
**DOC2GP**	-2.32	4.12E-05	1.17E-04
**CLEC5A**	-1.11	4.12E-05	1.17E-04
**GEMIN8**	0.50	4.13E-05	1.17E-04
**RP11-269G24.7**	-4.79	4.13E-05	1.17E-04
**RP11-593F23.1**	-2.73	4.14E-05	1.17E-04
**BCAS2P2**	-3.14	4.14E-05	1.18E-04
**RSPH1**	-1.07	4.14E-05	1.18E-04
**PAPL**	1.41	4.15E-05	1.18E-04
**MSH2**	-0.58	4.15E-05	1.18E-04
**RN7SL138P**	1.41	4.16E-05	1.18E-04
**EML2**	0.66	4.17E-05	1.18E-04

**AC000003.2**	-4.23	4.17E-05	1.18E-04
**UCHL1**	1.71	4.17E-05	1.18E-04
**RP11-435F17.3**	1.78	4.18E-05	1.19E-04
**ADIPOR2**	0.55	4.18E-05	1.19E-04
**RP11-252C15.1**	-3.06	4.19E-05	1.19E-04
**RP11-21M24.2**	-3.64	4.19E-05	1.19E-04
**VRK3**	0.44	4.20E-05	1.19E-04
**MTND5P15**	-4.74	4.20E-05	1.19E-04
**FAM212A**	0.64	4.21E-05	1.19E-04
**RP11-357N13.1**	-4.36	4.21E-05	1.19E-04
**WBP11**	0.40	4.23E-05	1.20E-04
**DDX3P1**	-4.88	4.23E-05	1.20E-04
**PIP5K1P1**	-4.65	4.24E-05	1.20E-04
**ZNF20**	-1.34	4.24E-05	1.20E-04
**FBXO36-IT1**	-4.74	4.25E-05	1.20E-04
**ZNF547**	-0.82	4.25E-05	1.20E-04
**G30001**	-1.21	4.26E-05	1.21E-04
**RP11-22H5.1**	-4.90	4.27E-05	1.21E-04
**CENPBD1P1**	0.61	4.28E-05	1.21E-04
**KRT85**	-8.24	4.28E-05	1.21E-04
**RP11-37C7.3**	-2.75	4.29E-05	1.21E-04
**SNORD45**	-4.92	4.29E-05	1.21E-04
**ZMYM5**	-0.41	4.29E-05	1.21E-04
**AL139318.1**	-4.77	4.30E-05	1.22E-04
**TSPAN16**	-3.41	4.30E-05	1.22E-04
**G39254**	2.47	4.30E-05	1.22E-04
**PCDHB11**	-1.47	4.30E-05	1.22E-04
**CCAT1**	-4.82	4.31E-05	1.22E-04
**RP11-505K9.3**	-4.77	4.32E-05	1.22E-04
**MLLT10**	-0.46	4.33E-05	1.22E-04
**RP11-1007J8.1**	-4.57	4.33E-05	1.22E-04
**RP11-902B17.1**	-1.00	4.33E-05	1.23E-04
**RN7SL801P**	-5.07	4.33E-05	1.23E-04
**RP11-426C22.4**	2.55	4.34E-05	1.23E-04
**SIGLEC6**	-2.80	4.34E-05	1.23E-04
**AC105760.3**	-2.53	4.34E-05	1.23E-04
**PPP2R5C**	-0.47	4.34E-05	1.23E-04
**SOS1**	-0.48	4.35E-05	1.23E-04
**TCF3P1**	-4.79	4.36E-05	1.23E-04
**XLOC_004198**	3.44	4.36E-05	1.23E-04
**RP11-537H15.3**	-2.51	4.37E-05	1.23E-04
**LANCL3**	-0.83	4.37E-05	1.24E-04
**VSTM4**	1.28	4.38E-05	1.24E-04
**NEURL4**	0.80	4.39E-05	1.24E-04
**GTF2IP1**	-0.70	4.39E-05	1.24E-04

**RP11-737O24.1**	-4.81	4.39E-05	1.24E-04
**GALNT7**	1.15	4.39E-05	1.24E-04
**RP11-318C2.1**	-2.88	4.40E-05	1.24E-04
**ACTBP13**	-3.47	4.40E-05	1.24E-04
**LRRC34**	-1.08	4.40E-05	1.24E-04
**G31549**	-1.76	4.40E-05	1.24E-04
**SNORD116-14**	-4.56	4.41E-05	1.24E-04
**SLC52A3**	1.54	4.42E-05	1.25E-04
**RP3-468O1.6**	-4.83	4.42E-05	1.25E-04
**RP11-139K4.2**	-4.93	4.43E-05	1.25E-04
**STPG2**	-1.78	4.43E-05	1.25E-04
**RP4-568F9.6**	-4.51	4.44E-05	1.25E-04
**RNU4-48P**	-4.83	4.44E-05	1.25E-04
**TIFAB**	-2.15	4.44E-05	1.25E-04
**G2450**	-1.68	4.45E-05	1.26E-04
**TEAD4**	0.71	4.47E-05	1.26E-04
**MKI67**	-0.94	4.48E-05	1.26E-04
**HTR1DP1**	-2.75	4.48E-05	1.26E-04
**RP11-53B2.1**	-3.73	4.48E-05	1.26E-04
**RP11-274B21.3**	1.33	4.49E-05	1.27E-04
**G11854**	-4.63	4.50E-05	1.27E-04
**DCUN1D5**	0.45	4.50E-05	1.27E-04
**RPS3P1**	-4.69	4.51E-05	1.27E-04
**G41367**	-1.67	4.53E-05	1.28E-04
**TSPYL1**	-0.54	4.53E-05	1.28E-04
**PTTG4P**	-4.57	4.53E-05	1.28E-04
**ECT2**	-0.67	4.54E-05	1.28E-04
**RN7SL806P**	-4.56	4.54E-05	1.28E-04
**MEG9**	2.77	4.55E-05	1.28E-04
**RN7SL47P**	-4.77	4.55E-05	1.28E-04
**JPX**	-0.49	4.57E-05	1.29E-04
**NGFR**	1.71	4.57E-05	1.29E-04
**LLPHP2**	-4.95	4.59E-05	1.29E-04
**CTD-3032H12.1**	-3.06	4.60E-05	1.30E-04
**RP11-44N22.3**	1.76	4.61E-05	1.30E-04
**RPL7P14**	-4.83	4.61E-05	1.30E-04
**TMED11P**	-3.04	4.61E-05	1.30E-04
**AC087501.1**	-4.40	4.61E-05	1.30E-04
**MAMDC4**	-1.54	4.63E-05	1.30E-04
**HIST2H3A**	-4.43	4.63E-05	1.30E-04
**TAF9BP1**	-3.27	4.65E-05	1.31E-04
**SLC44A1**	-0.63	4.66E-05	1.31E-04
**ZNF337**	-1.00	4.67E-05	1.31E-04
**OSBPL10**	0.64	4.68E-05	1.32E-04
**RP11-244M2.1**	-1.10	4.69E-05	1.32E-04

**CLUH**	0.65	4.69E-05	1.32E-04
**SPECC1**	0.92	4.69E-05	1.32E-04
**RP11-543P15.3**	-4.76	4.69E-05	1.32E-04
**SAMD4A**	-1.10	4.70E-05	1.32E-04
**ZNF461**	-0.57	4.70E-05	1.32E-04
**AP5S1**	0.62	4.70E-05	1.32E-04
**XPC**	-0.48	4.70E-05	1.32E-04
**G29778**	-1.29	4.71E-05	1.32E-04
**RP5-894A10.2**	-1.21	4.71E-05	1.32E-04
**XPO6**	0.38	4.72E-05	1.33E-04
**CTD-2270P14.5**	-1.17	4.72E-05	1.33E-04
**RP11-454L9.2**	-2.17	4.74E-05	1.33E-04
**RP11-83J16.3**	-4.56	4.74E-05	1.33E-04
**C2orf47**	-0.46	4.74E-05	1.33E-04
**C1orf111**	-3.29	4.74E-05	1.33E-04
**RP3-415N12.1**	-3.09	4.74E-05	1.33E-04
**MORF4L2**	0.55	4.74E-05	1.33E-04
**RP11-220D10.1**	-5.02	4.75E-05	1.33E-04
**RN7SL246P**	-4.82	4.75E-05	1.33E-04
**MAST2**	0.80	4.76E-05	1.34E-04
**SSH1**	0.53	4.76E-05	1.34E-04
**ABCD4**	0.65	4.76E-05	1.34E-04
**FBXO21**	-0.48	4.76E-05	1.34E-04
**HMGB3P10**	-2.39	4.77E-05	1.34E-04
**KRT73-AS1**	-4.63	4.77E-05	1.34E-04
**GREM1**	2.70	4.77E-05	1.34E-04
**IL9**	-5.16	4.77E-05	1.34E-04
**GPKOW**	0.46	4.77E-05	1.34E-04
**SNHG9**	-1.11	4.78E-05	1.34E-04
**LSM1**	0.65	4.78E-05	1.34E-04
**CEBPG**	-0.58	4.78E-05	1.34E-04
**ADAP2**	0.95	4.78E-05	1.34E-04
**METTL21C**	-5.00	4.79E-05	1.34E-04
**SBK1**	1.72	4.80E-05	1.35E-04
**RNA5SP383**	3.08	4.80E-05	1.35E-04
**SASH3**	1.73	4.81E-05	1.35E-04
**PACRGL**	-0.46	4.83E-05	1.35E-04
**CBX3P5**	-4.61	4.83E-05	1.36E-04
**TSPO2**	-3.56	4.84E-05	1.36E-04
**MTX2**	0.49	4.85E-05	1.36E-04
**XLOC_002545**	-4.92	4.85E-05	1.36E-04
**RP11-864I4.4**	-1.95	4.86E-05	1.36E-04
**RP11-384K6.6**	1.22	4.86E-05	1.36E-04
**TLE4**	-0.67	4.88E-05	1.37E-04
**PLPPR5**	-5.13	4.88E-05	1.37E-04

**CARD14**	0.89	4.88E-05	1.37E-04
**LINC00871**	-4.51	4.88E-05	1.37E-04
**RP11-133K1.7**	-4.51	4.89E-05	1.37E-04
**MMP27**	-1.90	4.89E-05	1.37E-04
**TIGD1**	-0.97	4.91E-05	1.37E-04
**SNORD116-6**	-4.78	4.91E-05	1.37E-04
**TSC22D1**	0.77	4.91E-05	1.38E-04
**CTD-2081C10.5**	-4.90	4.92E-05	1.38E-04
**RP11-264L1.1**	-3.35	4.92E-05	1.38E-04
**RPS20P35**	-4.47	4.94E-05	1.38E-04
**RP11-873E20.1**	-1.56	4.94E-05	1.38E-04
**CTC-425O23.5**	-1.74	4.95E-05	1.39E-04
**VN1R83P**	-1.95	4.95E-05	1.39E-04
**GAPDHP27**	-4.83	4.97E-05	1.39E-04
**TMEM177**	0.52	4.97E-05	1.39E-04
**RP11-517P14.2**	2.13	4.97E-05	1.39E-04
**ADGRE2**	1.13	4.97E-05	1.39E-04
**SLC19A1**	1.31	4.98E-05	1.39E-04
**RP11-275F13.3**	-3.56	4.98E-05	1.39E-04
**DUXAP7**	-4.54	4.98E-05	1.39E-04
**DUS4L**	-0.55	4.99E-05	1.40E-04
**FKBP7**	1.32	4.99E-05	1.40E-04
**C1QBP**	0.58	4.99E-05	1.40E-04
**ATHL1**	2.08	5.00E-05	1.40E-04
**SLAIN2**	-0.54	5.00E-05	1.40E-04
**RP1-137K2.2**	-3.96	5.01E-05	1.40E-04
**AC018742.1**	-5.32	5.01E-05	1.40E-04
**SNORA48**	-2.72	5.02E-05	1.40E-04
**SNRPF**	0.76	5.02E-05	1.40E-04
**RP11-144C15.1**	-4.87	5.02E-05	1.40E-04
**NCF4**	1.75	5.03E-05	1.40E-04
**KRT8P36**	-4.39	5.03E-05	1.40E-04
**HSPE1P8**	-4.85	5.03E-05	1.40E-04
**CTD-3222D19.4**	-3.66	5.03E-05	1.40E-04
**SNORD69**	-2.70	5.03E-05	1.40E-04
**RPL39L**	0.89	5.03E-05	1.40E-04
**GRAMD1C**	-0.92	5.03E-05	1.41E-04
**RP11-346D14.1**	-2.39	5.03E-05	1.41E-04
**ETV4**	1.08	5.03E-05	1.41E-04
**IFNA20P**	-2.00	5.03E-05	1.41E-04
**RP11-274B21.4**	1.23	5.05E-05	1.41E-04
**AL627171.1**	-4.49	5.05E-05	1.41E-04
**DPYD-AS1**	-4.42	5.06E-05	1.41E-04
**RPS13P2**	-2.10	5.06E-05	1.41E-04
**G10908**	-1.71	5.07E-05	1.41E-04

**NPM1P19**	-4.63	5.07E-05	1.42E-04
**CTD-2206N4.4**	-2.19	5.08E-05	1.42E-04
**SLC35F4**	-4.50	5.09E-05	1.42E-04
**TRNP1**	1.08	5.09E-05	1.42E-04
**NONOP2**	-1.04	5.10E-05	1.42E-04
**RNU4-22P**	-4.41	5.10E-05	1.42E-04
**JAGN1**	0.51	5.10E-05	1.42E-04
**SNORD20**	-2.48	5.11E-05	1.42E-04
**NDUFB4P1**	-3.74	5.11E-05	1.43E-04
**RP11-252I14.2**	-4.83	5.12E-05	1.43E-04
**OR5P3**	-3.53	5.12E-05	1.43E-04
**KCNQ1OT1**	-1.35	5.12E-05	1.43E-04
**ARL5A**	-0.92	5.13E-05	1.43E-04
**MAFTRR**	-1.48	5.13E-05	1.43E-04
**SUMO1**	0.64	5.13E-05	1.43E-04
**CD300LF**	3.05	5.14E-05	1.43E-04
**ARHGEF9-IT1**	-4.80	5.14E-05	1.43E-04
**SNX32**	-1.52	5.14E-05	1.43E-04
**PPIL4**	-0.42	5.16E-05	1.44E-04
**FLNB-AS1**	-1.44	5.17E-05	1.44E-04
**RPL23AP23**	-4.80	5.17E-05	1.44E-04
**KRT8P29**	-4.63	5.17E-05	1.44E-04
**PYCR2**	0.77	5.18E-05	1.44E-04
**MIR30E**	-4.61	5.18E-05	1.44E-04
**SMG5**	0.54	5.18E-05	1.44E-04
**RNF149**	0.52	5.18E-05	1.44E-04
**CTC-297N7.10**	-4.70	5.18E-05	1.44E-04
**RP11-131M11.2**	-1.07	5.18E-05	1.44E-04
**RP3-509L4.3**	-4.77	5.19E-05	1.44E-04
**EBAG9P1**	-3.15	5.19E-05	1.45E-04
**MCMDC2**	-1.01	5.20E-05	1.45E-04
**HSF2BP**	1.31	5.20E-05	1.45E-04
**RNU6-466P**	-4.72	5.20E-05	1.45E-04
**SHE**	1.11	5.22E-05	1.45E-04
**RP11-950C14.3**	-3.20	5.23E-05	1.45E-04
**TMPRSS11CP**	-4.93	5.23E-05	1.46E-04
**TIMP2**	1.35	5.24E-05	1.46E-04
**AC022538.1**	-5.05	5.24E-05	1.46E-04
**LDHAP7**	-4.75	5.25E-05	1.46E-04
**SPRYD7**	0.65	5.25E-05	1.46E-04
**AMIGO2**	1.49	5.26E-05	1.46E-04
**RP11-461L13.5**	-3.54	5.26E-05	1.46E-04
**FSIP2-AS1**	-2.67	5.27E-05	1.47E-04
**DNAH7**	-1.61	5.28E-05	1.47E-04
**RNU6-142P**	-4.90	5.28E-05	1.47E-04

**RP11-415J8.3**	-1.32	5.28E-05	1.47E-04
**MCL1**	0.42	5.28E-05	1.47E-04
**XLOC_007686**	-1.16	5.29E-05	1.47E-04
**CTD-3199J23.6**	1.87	5.29E-05	1.47E-04
**RP11-448G4.4**	-4.76	5.29E-05	1.47E-04
**CPNE6**	-3.47	5.30E-05	1.47E-04
**RN7SKP70**	-2.43	5.30E-05	1.47E-04
**RP11-164N3.1**	-4.72	5.30E-05	1.47E-04
**SON**	-0.53	5.31E-05	1.48E-04
**IL11RA**	-1.19	5.33E-05	1.48E-04
**HES1**	0.90	5.34E-05	1.48E-04
**AC092574.2**	-2.40	5.35E-05	1.49E-04
**DICER1-AS1**	1.14	5.35E-05	1.49E-04
**RP11-787I22.3**	-2.86	5.36E-05	1.49E-04
**CSNK1G2-AS1**	-5.07	5.36E-05	1.49E-04
**MAMLD1**	1.24	5.37E-05	1.49E-04
**RP11-399O19.9**	-1.46	5.38E-05	1.49E-04
**CTSW**	1.38	5.38E-05	1.49E-04
**RP11-305K5.1**	-1.23	5.38E-05	1.49E-04
**HAGLROS**	4.86	5.39E-05	1.50E-04
**RP11-505K9.5**	-2.49	5.39E-05	1.50E-04
**RP11-57H12.2**	-5.35	5.40E-05	1.50E-04
**ULK3**	0.78	5.41E-05	1.50E-04
**MTMR1**	-0.56	5.41E-05	1.50E-04
**VDAC2**	0.53	5.41E-05	1.50E-04
**WASF3-AS1**	-4.81	5.42E-05	1.50E-04
**RP11-1074O12.1**	-4.64	5.44E-05	1.51E-04
**RP11-996F15.5**	-1.33	5.46E-05	1.51E-04
**RP1-309F20.3**	-1.72	5.47E-05	1.52E-04
**SEC16B**	-2.91	5.48E-05	1.52E-04
**RP11-697N18.4**	-2.76	5.48E-05	1.52E-04
**RNU6-107P**	-4.83	5.49E-05	1.52E-04
**RP11-849F2.9**	1.85	5.50E-05	1.52E-04
**NXPE1**	-3.89	5.50E-05	1.52E-04
**RP13-20L14.10**	-1.92	5.51E-05	1.53E-04
**BANF1P3**	-2.54	5.52E-05	1.53E-04
**FREM1**	-1.28	5.52E-05	1.53E-04
**EBI3**	3.03	5.52E-05	1.53E-04
**CA12**	0.44	5.55E-05	1.54E-04
**ARHGAP29**	-0.81	5.55E-05	1.54E-04
**TAS2R6P**	-3.55	5.56E-05	1.54E-04
**CSE1L**	-0.63	5.57E-05	1.54E-04
**LRRC37A2**	-1.12	5.58E-05	1.54E-04
**SLC9A5**	1.41	5.58E-05	1.55E-04
**RASA4CP**	1.17	5.59E-05	1.55E-04

**TDGF1P5**	-4.38	5.59E-05	1.55E-04
**RP11-277I20.2**	-4.73	5.59E-05	1.55E-04
**CTD-2630F21.1**	1.32	5.61E-05	1.55E-04
**FAM131C**	1.51	5.61E-05	1.55E-04
**RP11-419K12.1**	-4.49	5.61E-05	1.55E-04
**EIF3EP1**	-1.07	5.61E-05	1.55E-04
**C6orf47**	0.46	5.61E-05	1.55E-04
**GPR180**	-0.74	5.61E-05	1.55E-04
**DDX11L1**	-4.49	5.61E-05	1.55E-04
**SNORD14E**	-4.30	5.62E-05	1.55E-04
**DPY30**	0.68	5.62E-05	1.55E-04
**RP11-55K22.2**	-4.47	5.62E-05	1.55E-04
**HINFP**	0.58	5.63E-05	1.56E-04
**SNORD126**	-4.80	5.63E-05	1.56E-04
**MORC2-AS1**	-2.89	5.65E-05	1.56E-04
**UBE2D3P2**	-4.90	5.66E-05	1.57E-04
**RCOR3**	-0.54	5.67E-05	1.57E-04
**KLHL32**	-2.80	5.68E-05	1.57E-04
**RP11-894P9.2**	-4.41	5.69E-05	1.57E-04
**CSPG4P8**	3.14	5.70E-05	1.58E-04
**RP11-421L21.2**	-2.47	5.70E-05	1.58E-04
**ATP11A-AS1**	-3.51	5.71E-05	1.58E-04
**SLC25A2**	-3.25	5.71E-05	1.58E-04
**HEATR4**	-1.66	5.71E-05	1.58E-04
**RN7SL555P**	-5.44	5.71E-05	1.58E-04
**H2AFZP4**	-4.76	5.71E-05	1.58E-04
**RGS22**	-1.69	5.72E-05	1.58E-04
**RP4-700A9.1**	-4.58	5.72E-05	1.58E-04
**GTSE1**	1.13	5.72E-05	1.58E-04
**FAM19A2**	-1.32	5.75E-05	1.59E-04
**RP3-508I15.9**	-1.07	5.75E-05	1.59E-04
**EFS**	0.60	5.75E-05	1.59E-04
**KLHL36**	-0.29	5.76E-05	1.59E-04
**LTF**	3.76	5.76E-05	1.59E-04
**CYP27C1**	1.03	5.77E-05	1.59E-04
**ATP6V1G1**	0.63	5.77E-05	1.59E-04
**NKX3-1**	1.51	5.78E-05	1.59E-04
**RP11-554D14.6**	-4.75	5.78E-05	1.60E-04
**ITGB1**	0.88	5.80E-05	1.60E-04
**CBWD6**	-1.07	5.81E-05	1.60E-04
**CARD8-AS1**	1.52	5.81E-05	1.60E-04
**DNAJC3**	-0.53	5.81E-05	1.60E-04
**AC011385.1**	-2.46	5.82E-05	1.61E-04
**CTD-2349P21.9**	-3.02	5.83E-05	1.61E-04
**RAB40AL**	-4.66	5.84E-05	1.61E-04

**GOLIM4**	0.74	5.85E-05	1.61E-04
**MIR3145**	-4.58	5.87E-05	1.62E-04
**RP11-286O18.1**	-4.66	5.88E-05	1.62E-04
**EEF1E1**	-0.93	5.90E-05	1.63E-04
**BCL11A**	-0.87	5.90E-05	1.63E-04
**CTD-2173L22.4**	-1.23	5.91E-05	1.63E-04
**UBASH3B**	-0.93	5.92E-05	1.63E-04
**SYTL5**	2.74	5.93E-05	1.63E-04
**RP11-195C7.1**	-1.83	5.93E-05	1.63E-04
**G2592**	-1.63	5.93E-05	1.63E-04
**RP11-849F2.5**	-0.99	5.94E-05	1.64E-04
**RP11-290D2.5**	-4.55	5.94E-05	1.64E-04
**CTB-167B5.1**	-4.54	5.95E-05	1.64E-04
**OR5P1P**	-3.48	5.95E-05	1.64E-04
**KRT8P44**	-5.13	5.96E-05	1.64E-04
**ABHD5**	-1.03	5.97E-05	1.65E-04
**RP11-434D9.1**	-3.43	5.98E-05	1.65E-04
**XLOC_005768**	-4.98	5.98E-05	1.65E-04
**RPA2**	0.74	5.99E-05	1.65E-04
**HMGB1P8**	-4.64	6.00E-05	1.65E-04
**RP11-776B3.1**	-4.63	6.00E-05	1.65E-04
**ZNF578**	-1.67	6.01E-05	1.66E-04
**PRKAG1**	0.54	6.02E-05	1.66E-04
**AQP4**	-5.32	6.02E-05	1.66E-04
**RP11-108K3.4**	-4.87	6.03E-05	1.66E-04
**TRIM37**	-0.46	6.04E-05	1.66E-04
**RBM3**	0.67	6.04E-05	1.66E-04
**C17orf67**	1.20	6.05E-05	1.66E-04
**RP11-220I1.4**	-4.92	6.05E-05	1.67E-04
**LINC00238**	-3.86	6.05E-05	1.67E-04
**XLOC_009001**	-4.66	6.05E-05	1.67E-04
**FADS2**	-3.90	6.06E-05	1.67E-04
**AC010642.1**	-1.40	6.08E-05	1.67E-04
**MIR31HG**	5.46	6.08E-05	1.67E-04
**NCKAP5L**	1.15	6.09E-05	1.67E-04
**PRDX2**	0.81	6.09E-05	1.67E-04
**RN7SKP235**	-4.87	6.11E-05	1.68E-04
**ABCB10**	-0.37	6.11E-05	1.68E-04
**RFC1**	-0.46	6.11E-05	1.68E-04
**TBC1D16**	0.42	6.11E-05	1.68E-04
**G28648**	-1.26	6.11E-05	1.68E-04
**RP11-285G1.14**	-3.40	6.12E-05	1.68E-04
**ANAPC10P1**	-4.77	6.12E-05	1.68E-04
**CTC-296K1.3**	2.65	6.13E-05	1.68E-04
**ST3GAL1P1**	-4.62	6.13E-05	1.68E-04

**RPS15AP11**	-3.63	6.13E-05	1.68E-04
**RP11-297A16.4**	1.15	6.13E-05	1.69E-04
**RNU6-198P**	-4.93	6.14E-05	1.69E-04
**ZDHHC15**	-0.90	6.15E-05	1.69E-04
**SNORD53**	-4.84	6.15E-05	1.69E-04
**TP63**	-0.65	6.15E-05	1.69E-04
**ZNF577**	-0.89	6.16E-05	1.69E-04
**RP11-758H9.2**	1.13	6.16E-05	1.69E-04
**KCTD9P5**	-5.23	6.17E-05	1.70E-04
**RP11-332O19.2**	-4.60	6.17E-05	1.70E-04
**RP11-678G15.2**	-4.68	6.19E-05	1.70E-04
**KRT71**	-6.58	6.20E-05	1.70E-04
**GATM-AS1**	-4.52	6.21E-05	1.70E-04
**OSTCP8**	-4.50	6.21E-05	1.71E-04
**RNF214**	0.47	6.22E-05	1.71E-04
**G38113**	-2.16	6.24E-05	1.71E-04
**RP11-20I23.5**	-2.96	6.24E-05	1.71E-04
**ATP5G1P6**	-1.85	6.25E-05	1.71E-04
**LRRC26**	3.73	6.26E-05	1.72E-04
**GBA2**	0.68	6.27E-05	1.72E-04
**EXOC6**	-0.55	6.28E-05	1.72E-04
**MTMR3**	-0.56	6.28E-05	1.72E-04
**PSG4**	-2.92	6.28E-05	1.72E-04
**RN7SL851P**	-4.58	6.29E-05	1.72E-04
**RP11-80H5.7**	-3.15	6.29E-05	1.72E-04
**RP1-257A7.4**	-2.14	6.29E-05	1.73E-04
**AC008850.3**	-2.70	6.30E-05	1.73E-04
**RP11-480A16.1**	-1.87	6.30E-05	1.73E-04
**TRAPPC5**	-1.18	6.30E-05	1.73E-04
**ZCCHC6**	-0.62	6.31E-05	1.73E-04
**AP000892.6**	2.37	6.31E-05	1.73E-04
**G23133**	-1.01	6.31E-05	1.73E-04
**TIMP3**	1.38	6.32E-05	1.73E-04
**G33706**	-2.47	6.33E-05	1.74E-04
**G5880**	-5.45	6.35E-05	1.74E-04
**EIF4BP2**	-4.98	6.35E-05	1.74E-04
**PCNXL3**	0.72	6.35E-05	1.74E-04
**RP1-128M12.3**	-2.61	6.35E-05	1.74E-04
**LINC01465**	-1.19	6.36E-05	1.74E-04
**CRY1**	-0.96	6.36E-05	1.74E-04
**IPO5P1**	-1.25	6.36E-05	1.74E-04
**DCPS**	0.55	6.37E-05	1.74E-04
**SSB**	0.69	6.37E-05	1.75E-04
**CTD-3193K9.1**	-4.67	6.37E-05	1.75E-04
**GLRX2**	-0.68	6.38E-05	1.75E-04

**RP11-168F9.2**	-3.80	6.38E-05	1.75E-04
**UBA3**	-0.54	6.38E-05	1.75E-04
**CENPT**	0.89	6.39E-05	1.75E-04
**RP11-443B20.1**	-1.34	6.39E-05	1.75E-04
**NLN**	-0.53	6.40E-05	1.75E-04
**RP11-697H9.5**	-4.55	6.40E-05	1.75E-04
**G15374**	-1.55	6.40E-05	1.75E-04
**AC007461.2**	-4.84	6.40E-05	1.75E-04
**RP11-73E6.2**	-4.68	6.40E-05	1.75E-04
**G3632**	-1.48	6.41E-05	1.75E-04
**KLRC4-KLRK1**	-4.81	6.42E-05	1.75E-04
**TYRO3**	-0.75	6.42E-05	1.76E-04
**AC109631.1**	-4.32	6.43E-05	1.76E-04
**LRRC69**	-1.44	6.43E-05	1.76E-04
**USP5**	0.54	6.44E-05	1.76E-04
**CLHC1**	-0.92	6.44E-05	1.76E-04
**ST6GAL2**	-2.31	6.45E-05	1.76E-04
**DIABLO**	-0.90	6.46E-05	1.77E-04
**EXD2**	0.36	6.46E-05	1.77E-04
**DBF4**	-0.58	6.47E-05	1.77E-04
**ITM2BP1**	-4.23	6.48E-05	1.77E-04
**RP11-417L19.6**	-2.10	6.49E-05	1.77E-04
**RAB4B**	1.02	6.49E-05	1.77E-04
**RP11-61L19.3**	-1.07	6.50E-05	1.78E-04
**ENO4**	-1.65	6.50E-05	1.78E-04
**RNU6-946P**	-4.89	6.51E-05	1.78E-04
**RP11-401P9.4**	-0.99	6.51E-05	1.78E-04
**RP11-526K21.2**	-4.77	6.51E-05	1.78E-04
**AC107081.5**	-1.95	6.52E-05	1.78E-04
**GRIN3B**	2.34	6.53E-05	1.78E-04
**PAPOLA**	-0.36	6.54E-05	1.78E-04
**KBTBD12**	-2.32	6.55E-05	1.79E-04
**LRRC9**	-4.35	6.55E-05	1.79E-04
**WASL**	-0.47	6.55E-05	1.79E-04
**NCKAP5-IT1**	-4.53	6.55E-05	1.79E-04
**NPEPL1**	0.97	6.56E-05	1.79E-04
**ETF1P1**	-4.61	6.56E-05	1.79E-04
**RP11-757C15.4**	-4.69	6.56E-05	1.79E-04
**HEXA-AS1**	-1.18	6.57E-05	1.79E-04
**HYOU1**	0.54	6.58E-05	1.79E-04
**STX1B**	1.12	6.59E-05	1.80E-04
**WDR83**	0.88	6.59E-05	1.80E-04
**RPL10AP1**	-4.43	6.59E-05	1.80E-04
**CNNM4**	0.98	6.60E-05	1.80E-04
**SNAPC1**	0.67	6.60E-05	1.80E-04

**MIR3657**	-4.95	6.61E-05	1.80E-04
**RP11-706C16.7**	2.73	6.61E-05	1.80E-04
**SLC29A2**	1.06	6.61E-05	1.80E-04
**APOBR**	1.06	6.65E-05	1.81E-04
**SLC17A9**	2.15	6.65E-05	1.81E-04
**FAM229B**	1.04	6.67E-05	1.82E-04
**FKBP9**	0.61	6.67E-05	1.82E-04
**RNASE1**	2.09	6.67E-05	1.82E-04
**C18orf65**	2.27	6.68E-05	1.82E-04
**RP11-112J3.16**	-3.42	6.71E-05	1.83E-04
**RN7SL526P**	-4.75	6.71E-05	1.83E-04
**MIR98**	-4.60	6.72E-05	1.83E-04
**NAPA-AS1**	-1.71	6.73E-05	1.83E-04
**RP11-76C10.6**	-4.88	6.74E-05	1.83E-04
**TRMO**	-0.41	6.74E-05	1.84E-04
**PLS1**	-0.75	6.75E-05	1.84E-04
**GUSBP1**	-0.98	6.75E-05	1.84E-04
**RP5-1182A14.5**	-4.77	6.76E-05	1.84E-04
**EHMT1-IT1**	-4.71	6.76E-05	1.84E-04
**RP11-452J6.2**	-3.41	6.76E-05	1.84E-04
**DCBLD2**	-0.55	6.77E-05	1.84E-04
**G9105**	4.30	6.77E-05	1.84E-04
**USP16**	-0.53	6.78E-05	1.84E-04
**RP4-555D20.2**	-4.25	6.79E-05	1.85E-04
**CTB-46B19.2**	-1.94	6.81E-05	1.85E-04
**AC073641.2**	-4.57	6.81E-05	1.85E-04
**YPEL2**	-0.46	6.81E-05	1.85E-04
**RP11-214O14.1**	-4.60	6.82E-05	1.86E-04
**LL22NC03-80A10.6**	-1.02	6.82E-05	1.86E-04
**TLCD2**	0.99	6.84E-05	1.86E-04
**CMPK1**	-0.54	6.84E-05	1.86E-04
**HNRNPA1P45**	-4.91	6.84E-05	1.86E-04
**POP7**	0.58	6.84E-05	1.86E-04
**RP11-196O2.1**	-4.79	6.85E-05	1.86E-04
**RNY3**	-4.89	6.86E-05	1.86E-04
**FAM83H**	0.72	6.86E-05	1.86E-04
**XLOC_006923**	-2.36	6.88E-05	1.87E-04
**RP11-536C5.7**	-1.77	6.89E-05	1.87E-04
**AC009299.1**	-4.67	6.90E-05	1.88E-04
**ATP5J**	0.61	6.91E-05	1.88E-04
**LINC01537**	1.65	6.91E-05	1.88E-04
**RPL4P5**	-1.73	6.91E-05	1.88E-04
**RP11-423F24.3**	-2.61	6.92E-05	1.88E-04
**RP11-367O10.1**	-4.88	6.92E-05	1.88E-04
**PROK1**	3.89	6.93E-05	1.88E-04

**NEK11**	-0.54	6.94E-05	1.88E-04
**ACAD9**	-0.61	6.94E-05	1.88E-04
**KALRN**	0.99	6.95E-05	1.89E-04
**RP11-1365D11.1**	-2.13	6.96E-05	1.89E-04
**G8112**	1.15	6.96E-05	1.89E-04
**RP11-516A11.1**	-4.42	6.97E-05	1.89E-04
**OR5BK1P**	-4.48	6.98E-05	1.90E-04
**PUDP**	0.58	6.99E-05	1.90E-04
**RP11-60C6.8**	-4.61	6.99E-05	1.90E-04
**WDR48**	-0.37	6.99E-05	1.90E-04
**RNU6-960P**	-4.87	7.02E-05	1.91E-04
**MYH14**	-1.07	7.03E-05	1.91E-04
**RNY4P7**	-4.75	7.03E-05	1.91E-04
**G23048**	-2.62	7.05E-05	1.91E-04
**MAN2B2**	0.50	7.05E-05	1.91E-04
**AC010149.4**	-4.37	7.06E-05	1.91E-04
**NDUFA3P2**	-4.61	7.07E-05	1.92E-04
**CLDN3**	2.27	7.07E-05	1.92E-04
**IYD**	-3.86	7.08E-05	1.92E-04
**HOXA2**	-1.54	7.09E-05	1.92E-04
**TSPAN14**	0.59	7.10E-05	1.92E-04
**PSMA3-AS1**	-0.66	7.10E-05	1.93E-04
**RPSAP19**	-1.59	7.10E-05	1.93E-04
**SUPT6H**	0.36	7.12E-05	1.93E-04
**AL596220.1**	-4.71	7.13E-05	1.93E-04
**YWHAZP3**	-1.98	7.13E-05	1.93E-04
**RP11-420B22.1**	-4.43	7.13E-05	1.93E-04
**SPATA33**	0.81	7.14E-05	1.94E-04
**RNU6-1310P**	-4.75	7.15E-05	1.94E-04
**ALDH1A3**	1.49	7.16E-05	1.94E-04
**HNRNPA1P36**	-4.65	7.16E-05	1.94E-04
**RP11-245J9.6**	-2.74	7.16E-05	1.94E-04
**COL2A1**	-1.93	7.16E-05	1.94E-04
**MCCD1**	-4.80	7.17E-05	1.94E-04
**XLOC_005303**	3.91	7.17E-05	1.94E-04
**EDN1**	1.05	7.19E-05	1.95E-04
**RP11-114B7.6**	4.24	7.19E-05	1.95E-04
**YARS**	0.51	7.20E-05	1.95E-04
**SMARCA2**	-0.40	7.21E-05	1.95E-04
**RP11-525G12.1**	-4.55	7.21E-05	1.95E-04
**APBB1IP**	1.55	7.22E-05	1.95E-04
**AC090804.1**	-3.23	7.22E-05	1.95E-04
**RAD51B**	-0.61	7.22E-05	1.95E-04
**PACSIN2**	0.32	7.24E-05	1.96E-04
**RP11-583F24.7**	-4.03	7.25E-05	1.96E-04

**MTOR-AS1**	-4.50	7.25E-05	1.96E-04
**NCBP2-AS1**	-3.10	7.26E-05	1.96E-04
**ALOX5AP**	1.61	7.26E-05	1.97E-04
**PI4KAP1**	2.14	7.27E-05	1.97E-04
**CTD-2269F5.1**	-1.34	7.27E-05	1.97E-04
**AC010886.2**	-5.06	7.27E-05	1.97E-04
**AMMECR1-IT1**	-4.55	7.28E-05	1.97E-04
**RP11-968A15.8**	-2.91	7.28E-05	1.97E-04
**ZNF354B**	-0.58	7.28E-05	1.97E-04
**RBM45**	-0.47	7.28E-05	1.97E-04
**AMACR**	1.26	7.28E-05	1.97E-04
**TPH1**	-1.91	7.29E-05	1.97E-04
**RPL17P17**	-4.06	7.29E-05	1.97E-04
**RP11-394B5.2**	-4.73	7.30E-05	1.97E-04
**PIEZO2**	-1.36	7.30E-05	1.97E-04
**ATXN7L2**	0.77	7.31E-05	1.98E-04
**C19orf71**	-1.21	7.31E-05	1.98E-04
**5S_rRNA**	-4.51	7.32E-05	1.98E-04
**G32308**	4.34	7.32E-05	1.98E-04
**RP11-552M6.1**	-4.64	7.33E-05	1.98E-04
**AL133243.3**	-4.55	7.34E-05	1.98E-04
**RP11-650J17.2**	-4.60	7.35E-05	1.98E-04
**CD58**	-0.63	7.35E-05	1.99E-04
**CAMK4**	-1.40	7.35E-05	1.99E-04
**RP11-401L13.7**	-4.96	7.36E-05	1.99E-04
**ADGRE4P**	-2.11	7.36E-05	1.99E-04
**GDNF**	1.79	7.38E-05	1.99E-04
**RP11-401E9.3**	-4.62	7.39E-05	1.99E-04
**YIPF4**	-0.41	7.40E-05	2.00E-04
**RP11-197N18.7**	-3.80	7.40E-05	2.00E-04
**SNORA25**	-3.93	7.41E-05	2.00E-04
**RP11-105N14.1**	-1.36	7.41E-05	2.00E-04
**RP11-98D18.3**	3.11	7.45E-05	2.01E-04
**XLOC_013496**	-4.88	7.45E-05	2.01E-04
**NFKBIA**	0.81	7.46E-05	2.01E-04
**RP4-620E11.4**	-3.82	7.47E-05	2.02E-04
**RP11-933H2.4**	-1.91	7.48E-05	2.02E-04
**CYTIP**	-1.08	7.49E-05	2.02E-04
**RN7SL751P**	-2.74	7.49E-05	2.02E-04
**G12194**	-2.64	7.49E-05	2.02E-04
**RPL30P2**	-4.73	7.50E-05	2.02E-04
**RP11-217L21.1**	-4.60	7.50E-05	2.02E-04
**RP11-380M21.3**	-4.81	7.51E-05	2.03E-04
**ZNF442**	-0.82	7.53E-05	2.03E-04
**PAOX**	0.85	7.53E-05	2.03E-04

**TPSAB1**	2.26	7.54E-05	2.03E-04
**RP11-1250I15.1**	-4.69	7.54E-05	2.03E-04
**RP11-81A22.4**	0.71	7.55E-05	2.03E-04
**AC137932.5**	-2.03	7.55E-05	2.04E-04
**CTD-2555A7.3**	-2.83	7.56E-05	2.04E-04
**XXbac-B476C20.11**	-4.31	7.57E-05	2.04E-04
**SLC1A7**	1.93	7.58E-05	2.04E-04
**CPAMD8**	2.23	7.58E-05	2.04E-04
**RP5-965G21.5**	-4.41	7.60E-05	2.05E-04
**AIM2**	2.48	7.60E-05	2.05E-04
**RP11-256L6.2**	-4.32	7.61E-05	2.05E-04
**RP11-689J19.1**	-4.79	7.64E-05	2.06E-04
**SSBP3-AS1**	-1.75	7.64E-05	2.06E-04
**AC092162.1**	-2.14	7.66E-05	2.06E-04
**LINC01094**	1.15	7.67E-05	2.06E-04
**RP11-413G15.1**	-4.75	7.68E-05	2.07E-04
**CTB-102L5.7**	-3.53	7.70E-05	2.07E-04
**G6426**	-2.17	7.70E-05	2.07E-04
**USF1P1**	-4.42	7.70E-05	2.07E-04
**RPS20**	0.66	7.70E-05	2.07E-04
**RP11-676J12.7**	1.84	7.72E-05	2.08E-04
**TUBG1**	0.69	7.73E-05	2.08E-04
**AP001626.1**	1.96	7.73E-05	2.08E-04
**RASSF6**	-1.16	7.73E-05	2.08E-04
**NQO1**	1.04	7.74E-05	2.08E-04
**RP11-20L24.1**	-4.62	7.74E-05	2.08E-04
**HMGN2P27**	-4.68	7.75E-05	2.08E-04
**RNU6-759P**	-4.62	7.76E-05	2.09E-04
**RP11-342M1.3**	-1.67	7.77E-05	2.09E-04
**CA3**	-1.28	7.78E-05	2.09E-04
**IST1**	0.22	7.80E-05	2.10E-04
**RP11-114F3.5**	-3.73	7.82E-05	2.10E-04
**RP11-231E4.5**	-2.44	7.82E-05	2.10E-04
**AL160011.1**	-4.58	7.83E-05	2.10E-04
**SLC39A8**	1.43	7.83E-05	2.11E-04
**CTA-276F8.2**	-4.57	7.83E-05	2.11E-04
**RP11-379P15.1**	-4.96	7.84E-05	2.11E-04
**TRIM3**	0.72	7.85E-05	2.11E-04
**RP11-438L19.1**	-1.71	7.85E-05	2.11E-04
**RP11-111F16.2**	1.99	7.87E-05	2.11E-04
**RP11-511H9.3**	-2.64	7.88E-05	2.12E-04
**RP11-83A16.1**	-4.70	7.88E-05	2.12E-04
**XLOC_007993**	-1.17	7.89E-05	2.12E-04
**G32188**	1.79	7.89E-05	2.12E-04
**TCP11L1**	-0.53	7.89E-05	2.12E-04

**CTD-3126B10.2**	-2.48	7.92E-05	2.13E-04
**MCM5**	0.58	7.95E-05	2.13E-04
**SP140**	-1.33	7.95E-05	2.13E-04
**AC022819.2**	-4.70	7.95E-05	2.13E-04
**MEIS2**	0.61	7.95E-05	2.13E-04
**HSPB7**	1.24	7.96E-05	2.14E-04
**CCDC122**	-0.72	7.98E-05	2.14E-04
**SUV420H1**	-0.41	7.98E-05	2.14E-04
**CCNI**	0.43	7.99E-05	2.14E-04
**PDZD4**	0.85	7.99E-05	2.14E-04
**YBEY**	0.76	7.99E-05	2.14E-04
**MNS1**	1.09	8.02E-05	2.15E-04
**RP11-196G18.22**	-1.02	8.03E-05	2.15E-04
**GLRX3**	0.50	8.03E-05	2.16E-04
**PKD1L3**	-2.00	8.04E-05	2.16E-04
**IRF5**	0.76	8.05E-05	2.16E-04
**RP11-365F18.3**	-4.32	8.06E-05	2.16E-04
**XKR4**	-2.59	8.06E-05	2.16E-04
**RP11-392P7.7**	-4.85	8.07E-05	2.16E-04
**RP11-353H3.1**	-4.58	8.11E-05	2.17E-04
**PTPRVP**	-3.06	8.11E-05	2.17E-04
**RP11-111K18.2**	-3.07	8.12E-05	2.18E-04
**LINC01588**	-0.88	8.12E-05	2.18E-04
**FAM134A**	0.38	8.12E-05	2.18E-04
**TTC13**	-0.52	8.13E-05	2.18E-04
**RP11-234K24.3**	-3.02	8.13E-05	2.18E-04
**NECAP1**	-0.39	8.13E-05	2.18E-04
**FIGNL1**	-0.59	8.13E-05	2.18E-04
**MAP3K7CL**	1.13	8.13E-05	2.18E-04
**CTA-217C2.2**	-2.33	8.14E-05	2.18E-04
**CELF3**	-4.53	8.15E-05	2.18E-04
**KDELC1P1**	-4.65	8.17E-05	2.19E-04
**INSIG2**	-0.55	8.17E-05	2.19E-04
**CERKL**	1.87	8.18E-05	2.19E-04
**NRAS**	-0.57	8.20E-05	2.19E-04
**THSD4-AS1**	-3.13	8.21E-05	2.20E-04
**CTB-174D11.1**	-2.55	8.21E-05	2.20E-04
**RP11-474B12.1**	-2.07	8.22E-05	2.20E-04
**AP001205.1**	-2.93	8.23E-05	2.20E-04
**UBA5**	-0.34	8.23E-05	2.20E-04
**URB1**	-0.54	8.23E-05	2.20E-04
**TMEM223**	0.58	8.23E-05	2.20E-04
**RN7SL284P**	-4.50	8.24E-05	2.20E-04
**RP11-332L8.1**	-4.57	8.25E-05	2.21E-04
**RNF185**	0.51	8.26E-05	2.21E-04

**RP11-57K17.1**	-3.18	8.30E-05	2.22E-04
**RHOD**	0.73	8.33E-05	2.23E-04
**SV2C**	-1.48	8.34E-05	2.23E-04
**RP11-259F16.3**	-4.54	8.34E-05	2.23E-04
**MIR5692C1**	-4.68	8.35E-05	2.23E-04
**FAM21FP**	1.22	8.35E-05	2.23E-04
**CTD-2515H24.4**	-4.83	8.35E-05	2.23E-04
**CLPTM1L**	0.36	8.35E-05	2.23E-04
**RP11-413H22.3**	-2.65	8.36E-05	2.24E-04
**AC006372.4**	3.54	8.37E-05	2.24E-04
**RP11-454H13.1**	-4.54	8.37E-05	2.24E-04
**RP1-121G13.3**	-3.64	8.38E-05	2.24E-04
**RP1-20B11.2**	-2.55	8.39E-05	2.24E-04
**RP1-256G22.2**	-2.34	8.40E-05	2.25E-04
**GRIA1**	2.16	8.41E-05	2.25E-04
**FRG1BP**	0.82	8.41E-05	2.25E-04
**RP11-474P2.4**	-4.18	8.43E-05	2.25E-04
**CTD-2540B15.9**	-4.57	8.46E-05	2.26E-04
**COX6CP10**	-4.63	8.46E-05	2.26E-04
**CHTF8**	0.46	8.47E-05	2.26E-04
**RP11-95H11.1**	-4.83	8.48E-05	2.26E-04
**RP5-956O18.2**	-3.24	8.49E-05	2.27E-04
**GPR157**	-0.73	8.49E-05	2.27E-04
**FLII**	0.40	8.50E-05	2.27E-04
**RP11-641C17.3**	-4.71	8.50E-05	2.27E-04
**HAP1**	2.05	8.52E-05	2.28E-04
**SRD5A3**	0.82	8.52E-05	2.28E-04
**PDC**	-3.78	8.53E-05	2.28E-04
**HERC6**	1.11	8.54E-05	2.28E-04
**AC074117.10**	0.86	8.56E-05	2.28E-04
**HSPE1P26**	-4.24	8.56E-05	2.28E-04
**ITGAM**	1.58	8.56E-05	2.28E-04
**RP3-391O22.1**	-4.59	8.57E-05	2.29E-04
**ENTPD2**	1.22	8.57E-05	2.29E-04
**RP11-47G11.2**	-3.04	8.59E-05	2.29E-04
**RP11-405L18.4**	-4.19	8.60E-05	2.29E-04
**AC010226.4**	-1.31	8.61E-05	2.30E-04
**ACSM3**	-1.97	8.62E-05	2.30E-04
**GAPDHS**	-2.29	8.62E-05	2.30E-04
**PLA2G7**	-1.70	8.62E-05	2.30E-04
**ZNF346-IT1**	-2.15	8.65E-05	2.31E-04
**GCNT1P3**	-3.81	8.65E-05	2.31E-04
**AF127577.11**	-2.69	8.66E-05	2.31E-04
**P2RX2**	-4.69	8.66E-05	2.31E-04
**LINC00511**	0.73	8.66E-05	2.31E-04

**CTC-277H1.7**	1.88	8.67E-05	2.31E-04
**STRA6**	3.13	8.68E-05	2.31E-04
**RP11-15A1.2**	-3.01	8.68E-05	2.31E-04
**RNU6-681P**	-4.57	8.70E-05	2.32E-04
**H2AFZP1**	-4.64	8.70E-05	2.32E-04
**RP11-834C11.8**	-2.57	8.71E-05	2.32E-04
**SNAP47-AS1**	-4.54	8.72E-05	2.32E-04
**TMCO1**	0.52	8.72E-05	2.32E-04
**TUB**	-0.92	8.73E-05	2.33E-04
**MAST3**	-0.65	8.76E-05	2.33E-04
**DNAJC6**	-0.91	8.77E-05	2.33E-04
**RP11-248J18.3**	-1.08	8.77E-05	2.34E-04
**SPAG4**	2.10	8.78E-05	2.34E-04
**G3769**	-5.08	8.83E-05	2.35E-04
**MZT1P1**	-4.63	8.83E-05	2.35E-04
**STON2**	-0.63	8.84E-05	2.35E-04
**C15orf61**	-0.55	8.84E-05	2.35E-04
**AC012513.4**	-4.43	8.85E-05	2.36E-04
**RN7SKP237**	-4.55	8.86E-05	2.36E-04
**RHBDL1**	1.66	8.87E-05	2.36E-04
**G22682**	-2.30	8.88E-05	2.36E-04
**ESYT3**	-0.91	8.88E-05	2.36E-04
**LRP11**	-0.40	8.88E-05	2.36E-04
**SLC25A36**	-0.59	8.89E-05	2.36E-04
**EIF2AK2**	-0.68	8.89E-05	2.36E-04
**WDFY1**	-0.50	8.89E-05	2.36E-04
**RAD9B**	-1.16	8.89E-05	2.36E-04
**PSMD12**	-0.55	8.90E-05	2.37E-04
**TRIM63**	-1.33	8.91E-05	2.37E-04
**ADORA2A-AS1**	-2.28	8.92E-05	2.37E-04
**SNORA43**	-4.42	8.92E-05	2.37E-04
**XLOC_000828**	-4.57	8.93E-05	2.37E-04
**CDK5RAP3**	0.83	8.93E-05	2.37E-04
**RP11-374F3.5**	-4.58	8.94E-05	2.38E-04
**RP11-255P5.2**	-4.19	8.94E-05	2.38E-04
**RP11-354E23.5**	-3.21	8.94E-05	2.38E-04
**DZIP1L**	-1.19	8.95E-05	2.38E-04
**DNAJA4**	-0.55	8.95E-05	2.38E-04
**RP11-1149O23.2**	-1.34	8.95E-05	2.38E-04
**PLA2G12A**	-0.48	8.95E-05	2.38E-04
**SLC4A5**	-1.44	8.97E-05	2.38E-04
**RP11-327F22.4**	-3.20	8.98E-05	2.38E-04
**CTA-797E19.2**	-4.66	8.98E-05	2.39E-04
**RP1-199J3.7**	-3.59	8.98E-05	2.39E-04
**SWSAP1**	0.83	8.99E-05	2.39E-04

**ARHGAP26-AS1**	-4.80	9.00E-05	2.39E-04
**TMEM41A**	-0.33	9.00E-05	2.39E-04
**LSINCT5**	-4.69	9.01E-05	2.39E-04
**RP11-118E18.1**	-4.60	9.02E-05	2.39E-04
**CTD-2024I7.1**	-4.28	9.02E-05	2.39E-04
**FOXO4**	0.54	9.03E-05	2.40E-04
**RP11-1250I15.2**	-4.44	9.04E-05	2.40E-04
**SNX19**	-0.47	9.06E-05	2.40E-04
**RP11-567M16.5**	-4.47	9.07E-05	2.41E-04
**NDUFS5P1**	-4.51	9.08E-05	2.41E-04
**RP11-10N23.2**	-1.10	9.09E-05	2.41E-04
**DGCR2**	0.45	9.11E-05	2.42E-04
**ELMSAN1**	-0.65	9.12E-05	2.42E-04
**CCDC181**	-1.72	9.13E-05	2.42E-04
**INO80E**	0.99	9.13E-05	2.42E-04
**CTD-2566J3.2**	-4.96	9.14E-05	2.42E-04
**PNOC**	-4.63	9.16E-05	2.43E-04
**IL12RB1**	1.69	9.16E-05	2.43E-04
**TRAT1**	-2.13	9.17E-05	2.43E-04
**HSPD1P9**	-5.14	9.17E-05	2.43E-04
**RPL6**	0.60	9.17E-05	2.43E-04
**RP11-103B5.4**	-2.17	9.20E-05	2.44E-04
**RPL11**	0.64	9.21E-05	2.44E-04
**KRT18P59**	-2.30	9.21E-05	2.44E-04
**HOXB6**	2.14	9.21E-05	2.44E-04
**RP11-80H5.9**	-2.30	9.21E-05	2.44E-04
**AC003989.3**	-3.41	9.22E-05	2.44E-04
**C5AR2**	1.39	9.24E-05	2.45E-04
**SPAG16**	-0.71	9.24E-05	2.45E-04
**RP3-495K2.3**	-4.68	9.24E-05	2.45E-04
**CTD-3088G3.4**	-2.67	9.24E-05	2.45E-04
**RP11-523H24.3**	-2.55	9.25E-05	2.45E-04
**ZBTB20-AS4**	-2.32	9.26E-05	2.45E-04
**G2449**	-3.09	9.26E-05	2.45E-04
**ZNF559-ZNF177**	-3.72	9.28E-05	2.46E-04
**RP11-1079K10.3**	-3.64	9.28E-05	2.46E-04
**CRHBP**	2.51	9.29E-05	2.46E-04
**GDAP1L1**	-2.97	9.29E-05	2.46E-04
**HNRNPA1P14**	-4.51	9.30E-05	2.46E-04
**ANXA8L2**	0.82	9.30E-05	2.46E-04
**AC092155.4**	-4.69	9.31E-05	2.46E-04
**POGLUT1**	-0.53	9.31E-05	2.46E-04
**RP11-83M16.6**	-3.85	9.32E-05	2.46E-04
**VAMP4**	-0.45	9.32E-05	2.47E-04
**RP5-908M14.9**	1.28	9.32E-05	2.47E-04

**OGFOD1P1**	-4.52	9.32E-05	2.47E-04
**SGPP1**	-0.86	9.32E-05	2.47E-04
**TSEN15P2**	-4.28	9.33E-05	2.47E-04
**ISLR**	1.73	9.34E-05	2.47E-04
**RP3-417G15.1**	-1.17	9.35E-05	2.47E-04
**PRAM1**	2.15	9.36E-05	2.47E-04
**DPRXP3**	-4.43	9.37E-05	2.48E-04
**ADPRHL1**	1.10	9.38E-05	2.48E-04
**AC024257.1**	-4.28	9.38E-05	2.48E-04
**HDGF**	0.53	9.38E-05	2.48E-04
**RPS10P16**	-4.20	9.42E-05	2.49E-04
**RP11-521L9.2**	-4.44	9.45E-05	2.50E-04
**ZBTB2**	-0.53	9.49E-05	2.51E-04
**MIR640**	-4.79	9.50E-05	2.51E-04
**RP11-264J4.8**	-4.24	9.50E-05	2.51E-04
**RNU6-1267P**	-3.14	9.51E-05	2.51E-04
**U8**	-3.34	9.51E-05	2.51E-04
**PSTPIP1**	1.27	9.52E-05	2.52E-04
**RP11-329A14.1**	-4.49	9.52E-05	2.52E-04
**CBX6**	0.75	9.53E-05	2.52E-04
**LINC01132**	1.81	9.55E-05	2.52E-04
**CTB-49A3.5**	-2.87	9.57E-05	2.53E-04
**RASSF3**	0.51	9.57E-05	2.53E-04
**BCAN**	-1.59	9.58E-05	2.53E-04
**FAM174B**	0.78	9.59E-05	2.53E-04
**RPS5P2**	-4.47	9.60E-05	2.53E-04
**XLOC_013940**	-2.72	9.60E-05	2.53E-04
**RP11-240E2.2**	-3.01	9.61E-05	2.54E-04
**SH3GL3**	1.90	9.61E-05	2.54E-04
**ST8SIA4**	1.40	9.61E-05	2.54E-04
**RPLP1P6**	1.38	9.61E-05	2.54E-04
**RP11-15E18.5**	-4.42	9.62E-05	2.54E-04
**RBM38**	0.85	9.64E-05	2.54E-04
**LL21NC02-1C16.2**	2.09	9.65E-05	2.54E-04
**GPC6**	1.88	9.67E-05	2.55E-04
**ATP13A5**	-1.42	9.69E-05	2.55E-04
**RP1-21O18.3**	-4.52	9.70E-05	2.56E-04
**RP11-442G21.2**	-2.96	9.72E-05	2.56E-04
**LINC00282**	-4.16	9.75E-05	2.57E-04
**RP11-104E19.1**	-4.56	9.75E-05	2.57E-04
**G13446**	-4.79	9.76E-05	2.57E-04
**COL27A1**	1.00	9.77E-05	2.58E-04
**RP11-16C1.1**	-4.60	9.77E-05	2.58E-04
**EXOSC1**	0.54	9.78E-05	2.58E-04
**METTL9**	0.55	9.79E-05	2.58E-04

**DPPA2P1**	-4.50	9.80E-05	2.58E-04
**VIM**	1.00	9.81E-05	2.58E-04
**ARL13A**	-1.70	9.81E-05	2.59E-04
**SNAPC3**	-0.43	9.81E-05	2.59E-04
**COX6CP14**	-4.71	9.82E-05	2.59E-04
**GVQW1**	-1.33	9.82E-05	2.59E-04
**DKFZp779M0652**	3.98	9.83E-05	2.59E-04
**COQ10BP2**	-4.54	9.83E-05	2.59E-04
**RP4-669H2.1**	-2.50	9.85E-05	2.59E-04
**RHOXF2**	-4.83	9.87E-05	2.60E-04
**TP53**	0.44	9.89E-05	2.61E-04
**ZBTB7C**	0.78	9.91E-05	2.61E-04
**RP11-100G15.4**	-4.67	9.95E-05	2.62E-04
**PTDSS1**	-0.50	9.96E-05	2.62E-04
**RN7SL173P**	-4.61	9.96E-05	2.62E-04
**AC012314.8**	-1.62	9.97E-05	2.62E-04
**MAPK1IP1L**	-0.43	9.97E-05	2.62E-04
**ZNF670**	-0.68	9.98E-05	2.63E-04
**TDP2**	0.68	9.99E-05	2.63E-04
**PTCH1**	-0.76	9.99E-05	2.63E-04
**SCN4A**	3.85	1.00E-04	2.63E-04
**RP11-452H21.4**	-1.65	1.00E-04	2.63E-04
**RP11-321C24.4**	-4.43	1.00E-04	2.63E-04
**RP11-271C24.3**	-1.80	1.00E-04	2.63E-04
**RP11-18B16.2**	5.06	1.00E-04	2.63E-04
**RP11-169K17.2**	-4.76	1.00E-04	2.64E-04
**RP11-219B4.5**	-4.24	1.00E-04	2.64E-04
**ALDOB**	-2.32	1.00E-04	2.64E-04
**XLOC_007629**	-4.60	1.01E-04	2.64E-04
**AC017104.6**	2.37	1.01E-04	2.65E-04
**CAPRIN2**	-0.73	1.01E-04	2.65E-04
**G10910**	-1.86	1.01E-04	2.65E-04
**RP11-708J19.3**	-2.30	1.01E-04	2.65E-04
**RNU7-18P**	-4.45	1.01E-04	2.65E-04
**KLHL7**	-0.52	1.01E-04	2.66E-04
**LSMEM2**	-4.48	1.01E-04	2.66E-04
**KIF11**	-0.71	1.01E-04	2.66E-04
**SNORD91A**	-4.46	1.02E-04	2.67E-04
**APBA1**	-0.56	1.02E-04	2.67E-04
**SMG1P5**	-1.50	1.02E-04	2.67E-04
**RP11-361L15.5**	-2.21	1.02E-04	2.67E-04
**KDM5C-IT1**	-4.42	1.02E-04	2.68E-04
**ADAM15**	0.54	1.02E-04	2.68E-04
**AC034220.3**	-1.38	1.02E-04	2.68E-04
**ASPN**	1.48	1.02E-04	2.68E-04

**G15373**	-1.72	1.02E-04	2.69E-04
**WWP1**	-0.53	1.02E-04	2.69E-04
**AP000560.1**	-4.69	1.02E-04	2.69E-04
**PDCL3P5**	-1.94	1.02E-04	2.69E-04
**RP11-12O16.1**	-4.85	1.02E-04	2.69E-04
**RPS12P20**	-4.54	1.03E-04	2.69E-04
**RP11-322D14.2**	-4.46	1.03E-04	2.70E-04
**RP11-162A12.2**	1.09	1.03E-04	2.71E-04
**RP11-96D1.5**	-2.30	1.03E-04	2.71E-04
**ARHGAP44**	0.93	1.04E-04	2.72E-04
**PSMD8P1**	-4.21	1.04E-04	2.73E-04
**MMAA**	-0.65	1.04E-04	2.73E-04
**RP3-426I6.5**	-2.43	1.04E-04	2.73E-04
**RNU6-523P**	-4.46	1.04E-04	2.73E-04
**GJB7**	-2.13	1.04E-04	2.73E-04
**AC105461.1**	-4.74	1.05E-04	2.74E-04
**EPB42**	-2.87	1.05E-04	2.75E-04
**ENSA**	0.48	1.05E-04	2.75E-04
**CEP112**	0.98	1.05E-04	2.75E-04
**RNY3P7**	-3.79	1.05E-04	2.75E-04
**RP5-875H18.4**	-4.39	1.05E-04	2.76E-04
**LINC00920**	0.99	1.05E-04	2.76E-04
**ITPRIPL1**	0.96	1.05E-04	2.76E-04
**RP11-434E6.4**	0.90	1.05E-04	2.76E-04
**RP11-343C2.10**	-4.43	1.05E-04	2.76E-04
**GCK**	3.25	1.05E-04	2.76E-04
**RP11-425D17.2**	-3.36	1.05E-04	2.76E-04
**SMO**	0.69	1.06E-04	2.77E-04
**RP1-206D15.6**	-1.12	1.06E-04	2.77E-04
**RP11-530N7.2**	-3.77	1.06E-04	2.77E-04
**GTF3C1**	-0.43	1.06E-04	2.77E-04
**RP11-138H8.2**	-4.39	1.06E-04	2.77E-04
**APBB3**	1.20	1.06E-04	2.77E-04
**SUPT16HP1**	-3.20	1.06E-04	2.78E-04
**PKDCC**	1.69	1.06E-04	2.78E-04
**RP11-486B10.3**	-4.34	1.06E-04	2.78E-04
**CYYR1-AS1**	-2.44	1.06E-04	2.78E-04
**GAB1**	-0.63	1.06E-04	2.78E-04
**MB**	2.46	1.06E-04	2.79E-04
**RP11-1191J2.4**	-4.67	1.07E-04	2.79E-04
**XXbac-B444P24.13**	-1.91	1.07E-04	2.79E-04
**RPSAP12**	-1.68	1.07E-04	2.80E-04
**G40985**	-3.23	1.07E-04	2.80E-04
**METTL21AP1**	-4.22	1.07E-04	2.80E-04
**INPP1**	0.54	1.07E-04	2.80E-04

**MC5R**	-4.82	1.07E-04	2.81E-04
**RPL35P3**	-4.34	1.08E-04	2.81E-04
**CTA-246H3.11**	-2.14	1.08E-04	2.81E-04
**OMG**	-1.93	1.08E-04	2.82E-04
**ZIK1**	-0.82	1.08E-04	2.82E-04
**RAB25**	0.74	1.08E-04	2.82E-04
**RP11-325K4.2**	-1.88	1.08E-04	2.82E-04
**C1orf195**	-3.49	1.08E-04	2.82E-04
**TMEM97**	-1.79	1.08E-04	2.83E-04
**MAP3K9**	-0.88	1.08E-04	2.83E-04
**CTD-2366F13.2**	-2.04	1.08E-04	2.83E-04
**BISPR**	1.80	1.08E-04	2.83E-04
**CAV1**	0.64	1.09E-04	2.84E-04
**TATDN2P2**	-1.00	1.09E-04	2.84E-04
**MIR1276**	-4.61	1.09E-04	2.85E-04
**PRTFDC1**	1.16	1.09E-04	2.85E-04
**RP1-274L7.4**	-3.77	1.09E-04	2.85E-04
**MIR100HG**	-1.95	1.09E-04	2.85E-04
**RP11-479G22.8**	1.08	1.09E-04	2.85E-04
**RP11-188P20.3**	-2.03	1.09E-04	2.86E-04
**MTND5P14**	-4.54	1.09E-04	2.86E-04
**RP11-21M7.2**	-4.64	1.09E-04	2.86E-04
**SNAPC5**	0.49	1.09E-04	2.86E-04
**ATP6V1B1**	2.73	1.10E-04	2.86E-04
**RP11-342F17.1**	-4.75	1.10E-04	2.86E-04
**RP11-204K16.1**	-4.55	1.10E-04	2.87E-04
**GAPDHP47**	-4.61	1.10E-04	2.87E-04
**APOBEC3B**	1.40	1.10E-04	2.88E-04
**RAC2**	1.66	1.10E-04	2.88E-04
**RGPD8**	-1.01	1.10E-04	2.88E-04
**HNRNPA1P21**	-2.23	1.10E-04	2.88E-04
**FAM118B**	0.54	1.11E-04	2.89E-04
**AC004237.1**	-2.27	1.11E-04	2.89E-04
**PRR5L**	1.41	1.11E-04	2.89E-04
**C4orf27**	0.54	1.11E-04	2.89E-04
**ZNF449**	-0.78	1.11E-04	2.89E-04
**RP5-1029F21.2**	-4.16	1.11E-04	2.89E-04
**RP5-1115A15.1**	-2.00	1.11E-04	2.90E-04
**COX7A2**	0.68	1.11E-04	2.90E-04
**RP11-434E6.2**	-5.23	1.11E-04	2.90E-04
**RP11-224O19.4**	-4.62	1.11E-04	2.90E-04
**MYADM**	1.15	1.12E-04	2.91E-04
**EXOC7**	0.29	1.12E-04	2.91E-04
**AC006372.5**	4.28	1.12E-04	2.92E-04
**RP11-396F22.1**	2.33	1.12E-04	2.93E-04

**OR10J2P**	-4.72	1.13E-04	2.93E-04
**SORCS3**	-3.22	1.13E-04	2.93E-04
**ZNF285**	-1.49	1.13E-04	2.93E-04
**AKR1A1**	0.64	1.13E-04	2.93E-04
**GCNT1P1**	-4.49	1.13E-04	2.94E-04
**AC078852.1**	-4.58	1.13E-04	2.94E-04
**CTD-2340D6.1**	-4.54	1.13E-04	2.94E-04
**HIST3H2A**	0.80	1.13E-04	2.94E-04
**RAPGEF4-AS1**	-3.34	1.13E-04	2.94E-04
**RP11-139H14.5**	-2.86	1.13E-04	2.94E-04
**CADPS2**	0.90	1.13E-04	2.94E-04
**RNF222**	0.99	1.13E-04	2.94E-04
**SATB2-AS1**	2.55	1.13E-04	2.94E-04
**MS4A6A**	1.89	1.13E-04	2.95E-04
**RP11-389C8.2**	1.05	1.13E-04	2.95E-04
**RPS15AP36**	-4.39	1.13E-04	2.95E-04
**CHST15**	0.74	1.13E-04	2.95E-04
**RP11-39K24.4**	-3.72	1.14E-04	2.96E-04
**RP11-235E17.5**	-3.33	1.14E-04	2.96E-04
**CDAN1**	0.64	1.14E-04	2.96E-04
**KCNIP3**	0.95	1.14E-04	2.97E-04
**RP11-347C12.3**	4.23	1.14E-04	2.97E-04
**G2523**	-4.03	1.14E-04	2.97E-04
**RP3-510H16.3**	-4.33	1.15E-04	2.98E-04
**RP11-466A19.3**	-4.48	1.15E-04	2.98E-04
**CFAP52**	-2.84	1.15E-04	2.98E-04
**GAPDHP51**	-4.70	1.15E-04	2.98E-04
**KIF20B**	-0.74	1.15E-04	2.98E-04
**TRIM38**	0.45	1.15E-04	2.99E-04
**RN7SL362P**	-4.54	1.15E-04	2.99E-04
**G36840**	-1.76	1.15E-04	2.99E-04
**AC011753.5**	-3.23	1.15E-04	2.99E-04
**SEC62**	0.63	1.15E-04	3.00E-04
**PTRH2**	0.44	1.15E-04	3.00E-04
**KCNQ4**	1.95	1.15E-04	3.00E-04
**LINC01362**	-4.06	1.16E-04	3.00E-04
**AC107072.2**	-4.23	1.16E-04	3.00E-04
**RP11-749I16.3**	-3.67	1.16E-04	3.01E-04
**PSMA4**	0.63	1.16E-04	3.01E-04
**KLHL29**	-0.68	1.16E-04	3.01E-04
**SPEF2**	-1.00	1.16E-04	3.01E-04
**RNF144A**	0.85	1.16E-04	3.01E-04
**RP1-12G14.9**	-1.91	1.16E-04	3.01E-04
**KIAA0125**	-4.83	1.16E-04	3.01E-04
**RP11-272L13.4**	-4.78	1.16E-04	3.01E-04

**RP11-536K7.3**	1.76	1.16E-04	3.01E-04
**RP11-133N21.10**	-2.13	1.16E-04	3.02E-04
**UTAT33**	-2.13	1.17E-04	3.03E-04
**HSP90AB1**	0.56	1.17E-04	3.03E-04
**CDC42EP4**	0.64	1.17E-04	3.03E-04
**LINC01620**	-4.56	1.17E-04	3.03E-04
**RP11-109L13.1**	4.70	1.17E-04	3.03E-04
**RAD17P1**	-5.15	1.17E-04	3.04E-04
**XLOC_009777**	-3.19	1.18E-04	3.05E-04
**RP11-425L10.1**	-0.92	1.18E-04	3.06E-04
**CNOT7**	-0.50	1.18E-04	3.06E-04
**CTB-152G17.5**	-4.43	1.18E-04	3.06E-04
**MAML2**	-0.58	1.18E-04	3.06E-04
**PDE5A**	0.79	1.18E-04	3.06E-04
**RP11-360D2.2**	-4.50	1.18E-04	3.06E-04
**RP11-713M15.1**	-3.26	1.18E-04	3.06E-04
**RP11-401P9.6**	-4.24	1.18E-04	3.07E-04
**RP11-180P8.4**	-4.54	1.18E-04	3.07E-04
**PCDHB4**	1.25	1.19E-04	3.07E-04
**7SK**	-2.93	1.19E-04	3.07E-04
**RP11-115A14.1**	-4.49	1.19E-04	3.08E-04
**SEPT7P1**	-4.46	1.19E-04	3.08E-04
**SLC35E2**	-0.87	1.19E-04	3.09E-04
**RP11-649A18.3**	-4.43	1.19E-04	3.09E-04
**RP3-431P23.5**	3.69	1.19E-04	3.09E-04
**TRIM39**	0.46	1.19E-04	3.09E-04
**RP11-1275H24.2**	-1.76	1.20E-04	3.10E-04
**PHBP2**	-3.41	1.20E-04	3.10E-04
**NIPAL2**	-0.67	1.20E-04	3.10E-04
**AC008268.1**	-4.84	1.20E-04	3.10E-04
**TMEM184A**	1.03	1.20E-04	3.10E-04
**MIR181A2**	-4.55	1.20E-04	3.11E-04
**RP11-554A11.4**	2.29	1.20E-04	3.11E-04
**RP11-80I15.4**	-2.91	1.20E-04	3.11E-04
**MIR604**	-4.38	1.20E-04	3.11E-04
**RP11-439H8.4**	-4.44	1.20E-04	3.11E-04
**PPP4R1**	0.47	1.20E-04	3.12E-04
**RNF182**	2.03	1.20E-04	3.12E-04
**RNU6-126P**	-4.16	1.21E-04	3.13E-04
**TMED6**	-1.45	1.21E-04	3.13E-04
**RP11-804H8.6**	-1.50	1.21E-04	3.13E-04
**GRIN2A**	-2.05	1.21E-04	3.13E-04
**CDH19**	-1.31	1.21E-04	3.14E-04
**SPINK1**	-1.94	1.21E-04	3.14E-04
**PPP2R1B**	-0.43	1.21E-04	3.14E-04

**RP11-1007O24.3**	-1.06	1.21E-04	3.14E-04
**MAP3K12**	1.06	1.21E-04	3.14E-04
**G41235**	-2.79	1.22E-04	3.15E-04
**RP11-654A16.1**	-4.89	1.22E-04	3.16E-04
**RP11-183I6.2**	-4.52	1.22E-04	3.16E-04
**MIR548H1**	-4.59	1.22E-04	3.16E-04
**SNORA72**	-2.83	1.22E-04	3.16E-04
**SLC25A43**	-0.51	1.22E-04	3.16E-04
**RP11-493E12.2**	-2.02	1.22E-04	3.16E-04
**ZCCHC9**	0.50	1.22E-04	3.16E-04
**RP11-274B21.1**	-1.47	1.23E-04	3.18E-04
**G2899**	-2.18	1.23E-04	3.18E-04
**PRR4**	-1.66	1.23E-04	3.18E-04
**MAMSTR**	1.24	1.23E-04	3.18E-04
**CTC-348L5.1**	-3.44	1.23E-04	3.19E-04
**RP11-438F14.3**	-5.25	1.24E-04	3.19E-04
**CAMKV**	-4.25	1.24E-04	3.19E-04
**RP11-650J17.1**	-4.50	1.24E-04	3.20E-04
**CTD-3096P4.1**	-4.55	1.24E-04	3.20E-04
**MCCC1-AS1**	-2.99	1.24E-04	3.21E-04
**FUT2**	1.01	1.25E-04	3.23E-04
**UBE2FP1**	-1.20	1.25E-04	3.23E-04
**C1orf53**	1.32	1.25E-04	3.23E-04
**FRMD4B**	-0.51	1.25E-04	3.23E-04
**HTRA4**	2.54	1.25E-04	3.23E-04
**RP1-266L20.2**	1.79	1.25E-04	3.23E-04
**RPL10A**	0.59	1.25E-04	3.23E-04
**PLA2G5**	2.75	1.26E-04	3.24E-04
**UBE2G2**	0.42	1.26E-04	3.24E-04
**RP11-545I5.3**	-0.79	1.26E-04	3.24E-04
**RP4-814D15.2**	-2.18	1.26E-04	3.24E-04
**RNU6-1237P**	-4.51	1.26E-04	3.25E-04
**RNU6-943P**	-4.56	1.26E-04	3.25E-04
**AC006557.1**	-4.74	1.26E-04	3.26E-04
**RP1-142L7.5**	-2.13	1.26E-04	3.26E-04
**AC007036.6**	-2.06	1.27E-04	3.26E-04
**AC007879.5**	-4.30	1.27E-04	3.26E-04
**CCR9**	-4.58	1.27E-04	3.27E-04
**LINC01300**	-2.50	1.27E-04	3.27E-04
**CTD-2027I19.2**	-2.16	1.27E-04	3.27E-04
**FAM175B**	-0.52	1.27E-04	3.28E-04
**RP11-97N19.3**	-4.27	1.27E-04	3.28E-04
**SIGLEC9**	2.41	1.27E-04	3.28E-04
**PCDHB6**	1.54	1.28E-04	3.30E-04
**RPL36AP33**	-4.64	1.28E-04	3.30E-04

**CTSK**	1.63	1.28E-04	3.30E-04
**BPGM**	0.56	1.28E-04	3.30E-04
**XLOC_010601**	-4.80	1.28E-04	3.30E-04
**GABRP**	2.99	1.29E-04	3.31E-04
**ARHGAP26-IT1**	-4.72	1.29E-04	3.32E-04
**RP1-78O14.1**	2.38	1.29E-04	3.32E-04
**RP11-732M18.4**	-4.29	1.29E-04	3.33E-04
**G25046**	-4.27	1.29E-04	3.33E-04
**TMEM108-AS1**	-3.24	1.29E-04	3.33E-04
**CACNA2D3-AS1**	-4.45	1.30E-04	3.34E-04
**CRB2**	-1.93	1.30E-04	3.35E-04
**ENTPD8**	-4.49	1.30E-04	3.35E-04
**HGSNAT**	0.45	1.30E-04	3.36E-04
**COX8A**	-0.66	1.30E-04	3.36E-04
**EIF3L**	-0.55	1.31E-04	3.36E-04
**RP11-553K8.5**	-4.44	1.31E-04	3.36E-04
**AP000662.9**	-4.30	1.31E-04	3.36E-04
**RNU6-1157P**	-4.46	1.31E-04	3.37E-04
**TVP23C**	-1.09	1.31E-04	3.37E-04
**E2F7**	-0.87	1.31E-04	3.37E-04
**SIGLEC7**	3.99	1.31E-04	3.37E-04
**RPS24P16**	-4.66	1.31E-04	3.37E-04
**PUM1**	-0.34	1.31E-04	3.38E-04
**RP11-227G15.10**	1.96	1.31E-04	3.38E-04
**SAP130**	0.44	1.31E-04	3.38E-04
**UGCG**	-0.66	1.32E-04	3.39E-04
**DCHS2**	-1.66	1.32E-04	3.40E-04
**RPL23AP55**	-4.31	1.32E-04	3.40E-04
**GPR150**	1.75	1.32E-04	3.40E-04
**MAGEH1**	0.97	1.32E-04	3.40E-04
**APOBEC3F**	1.19	1.32E-04	3.40E-04
**PANO1**	1.83	1.32E-04	3.40E-04
**ARSE**	1.83	1.32E-04	3.40E-04
**RP11-423O2.2**	-4.49	1.32E-04	3.40E-04
**AC004540.5**	2.22	1.32E-04	3.40E-04
**SNORD85**	-4.66	1.32E-04	3.41E-04
**CRACR2B**	2.22	1.33E-04	3.42E-04
**CKB**	0.92	1.33E-04	3.42E-04
**ZDHHC4**	0.70	1.33E-04	3.42E-04
**CTD-2308L22.1**	1.15	1.33E-04	3.42E-04
**RP11-884K10.7**	-0.71	1.33E-04	3.42E-04
**RNU1-2**	-2.90	1.33E-04	3.42E-04
**ZIC1**	2.12	1.33E-04	3.43E-04
**ISLR2**	2.17	1.33E-04	3.43E-04
**RN7SL285P**	-4.50	1.34E-04	3.44E-04

**MOCS2**	0.52	1.34E-04	3.44E-04
**LA16c-380H5.3**	-4.47	1.34E-04	3.44E-04
**ZNF887P**	-1.29	1.34E-04	3.44E-04
**CTA-243E7.4**	-4.27	1.34E-04	3.44E-04
**RPL27P12**	-4.68	1.34E-04	3.44E-04
**MTND2P5**	-4.49	1.34E-04	3.44E-04
**FGR**	1.35	1.34E-04	3.45E-04
**RP11-477J21.6**	-4.16	1.34E-04	3.45E-04
**HMGB1P23**	-4.48	1.34E-04	3.45E-04
**PRMT1P1**	-4.61	1.34E-04	3.45E-04
**RP3-337H4.6**	-2.27	1.34E-04	3.45E-04
**RCOR2**	1.34	1.35E-04	3.45E-04
**RN7SKP95**	-4.70	1.35E-04	3.46E-04
**AC067940.1**	-4.52	1.35E-04	3.46E-04
**CCDC84**	-1.07	1.35E-04	3.46E-04
**MXD3**	1.00	1.35E-04	3.46E-04
**PHC1P1**	1.70	1.35E-04	3.47E-04
**NMD3P1**	-2.63	1.35E-04	3.47E-04
**NHLRC4**	2.32	1.35E-04	3.47E-04
**PTMAP3**	-4.53	1.35E-04	3.47E-04
**G32422**	-2.58	1.35E-04	3.47E-04
**C18orf15**	-4.60	1.36E-04	3.48E-04
**CTC-137K3.1**	-4.26	1.36E-04	3.48E-04
**CD80**	-3.27	1.36E-04	3.48E-04
**SNRPGP5**	-4.31	1.36E-04	3.49E-04
**DESI2**	-0.56	1.36E-04	3.49E-04
**SNORA3**	-2.35	1.36E-04	3.49E-04
**LCMT2**	-0.37	1.36E-04	3.50E-04
**RN7SL109P**	-4.28	1.37E-04	3.50E-04
**CCDC59**	0.60	1.37E-04	3.51E-04
**RP11-397O4.1**	-4.47	1.37E-04	3.51E-04
**RP11-410L14.1**	-3.13	1.37E-04	3.51E-04
**TBX21**	2.53	1.37E-04	3.51E-04
**RNY5P5**	-4.61	1.37E-04	3.52E-04
**KCNK10**	2.54	1.38E-04	3.53E-04
**SEC24B-AS1**	-0.99	1.38E-04	3.53E-04
**TAGAP**	-0.93	1.38E-04	3.54E-04
**CES4A**	-1.17	1.38E-04	3.54E-04
**USP21**	0.70	1.38E-04	3.54E-04
**MIR5706**	-4.62	1.39E-04	3.55E-04
**RP11-46D1.2**	-4.49	1.39E-04	3.55E-04
**HOXB13**	8.66	1.39E-04	3.55E-04
**CTD-2293H3.1**	-2.48	1.39E-04	3.55E-04
**AC006116.20**	-1.39	1.39E-04	3.56E-04
**IFT52**	0.60	1.39E-04	3.56E-04

**COL8A2**	1.14	1.39E-04	3.56E-04
**CTC-398G3.2**	-4.38	1.39E-04	3.57E-04
**TFAP4**	0.68	1.40E-04	3.57E-04
**RP11-193I22.2**	-4.42	1.40E-04	3.58E-04
**RP11-48B3.5**	1.41	1.40E-04	3.58E-04
**AP000719.1**	-4.49	1.40E-04	3.58E-04
**RP11-48B14.1**	-1.80	1.40E-04	3.58E-04
**RP1-168P16.1**	-4.78	1.40E-04	3.59E-04
**SEC14L4**	-3.47	1.41E-04	3.60E-04
**ALAS1**	0.73	1.41E-04	3.60E-04
**KCNG3**	-1.62	1.41E-04	3.60E-04
**GMNN**	-0.68	1.41E-04	3.60E-04
**NANOGP2**	-3.79	1.41E-04	3.61E-04
**RP11-50B3.4**	-2.64	1.42E-04	3.62E-04
**PRPS1P2**	-1.53	1.42E-04	3.62E-04
**SLC35E3**	0.56	1.42E-04	3.62E-04
**RP11-67L3.2**	-2.27	1.42E-04	3.62E-04
**MIR567**	-4.33	1.42E-04	3.63E-04
**RP11-386I23.1**	-2.80	1.42E-04	3.63E-04
**VWDE**	-2.68	1.42E-04	3.63E-04
**RP11-308D16.2**	-1.07	1.42E-04	3.64E-04
**SLC27A4**	0.67	1.42E-04	3.64E-04
**RPL5P11**	-4.75	1.43E-04	3.64E-04
**OR2A9P**	-2.16	1.43E-04	3.65E-04
**AMD1**	-0.54	1.43E-04	3.66E-04
**RP11-296K13.4**	-3.15	1.43E-04	3.66E-04
**TIMELESS**	0.71	1.43E-04	3.66E-04
**RP11-78O7.1**	-4.35	1.44E-04	3.67E-04
**AC110299.5**	-3.33	1.44E-04	3.67E-04
**NFE2L2**	-0.56	1.44E-04	3.68E-04
**AC073326.3**	-3.79	1.44E-04	3.68E-04
**KHDRBS1**	0.41	1.44E-04	3.68E-04
**RP11-295D4.4**	-2.25	1.44E-04	3.68E-04
**NBPF10**	-0.50	1.44E-04	3.69E-04
**NR6A1**	-0.76	1.45E-04	3.69E-04
**CCDC170**	1.56	1.45E-04	3.69E-04
**IL17B**	1.99	1.45E-04	3.69E-04
**RP4-718D20.3**	-4.50	1.45E-04	3.69E-04
**ACSF3**	0.54	1.45E-04	3.69E-04
**RP11-452N17.1**	-2.07	1.45E-04	3.70E-04
**LINC00565**	2.98	1.45E-04	3.70E-04
**CTD-2215E18.1**	-4.49	1.45E-04	3.71E-04
**COPS8**	0.48	1.46E-04	3.71E-04
**ENPP3**	-1.34	1.46E-04	3.71E-04
**CCK**	4.61	1.46E-04	3.71E-04

**AC106900.6**	-4.69	1.46E-04	3.72E-04
**AC015987.1**	-2.37	1.46E-04	3.72E-04
**MFAP4**	1.62	1.46E-04	3.72E-04
**KB-1090H4.2**	-4.55	1.46E-04	3.72E-04
**RPL15P18**	-4.63	1.46E-04	3.72E-04
**SETP20**	-3.20	1.47E-04	3.74E-04
**CALB2**	2.30	1.47E-04	3.74E-04
**ITIH2**	-2.16	1.47E-04	3.74E-04
**RP5-1154E9.7**	-4.81	1.47E-04	3.74E-04
**RP11-89N17.2**	-4.76	1.48E-04	3.76E-04
**PTMA**	0.67	1.48E-04	3.76E-04
**RP11-261C10.4**	-4.58	1.48E-04	3.76E-04
**CNTD2**	2.47	1.48E-04	3.77E-04
**SDC2**	1.22	1.48E-04	3.77E-04
**RP3-388M5.9**	-1.76	1.48E-04	3.77E-04
**EEF1A1P16**	-4.59	1.48E-04	3.78E-04
**RN7SKP30**	-3.28	1.48E-04	3.78E-04
**MIR4644**	-4.54	1.49E-04	3.79E-04
**AC126544.4**	-2.21	1.49E-04	3.79E-04
**MIR4714**	-4.48	1.49E-04	3.80E-04
**EVA1C**	0.69	1.49E-04	3.80E-04
**UTP14C**	-0.42	1.49E-04	3.80E-04
**DCUN1D3**	-0.44	1.50E-04	3.81E-04
**MED26**	0.59	1.50E-04	3.81E-04
**RP11-499O7.7**	-3.50	1.50E-04	3.81E-04
**RP11-10L12.1**	-4.49	1.50E-04	3.82E-04
**IRF6**	-0.59	1.50E-04	3.82E-04
**CTD-2587H19.3**	-3.34	1.50E-04	3.82E-04
**KDM5C**	0.36	1.50E-04	3.82E-04
**TRMT11**	-0.51	1.50E-04	3.82E-04
**AC007362.3**	-3.27	1.50E-04	3.82E-04
**MEFV**	2.17	1.50E-04	3.82E-04
**MIR550A1**	-4.66	1.51E-04	3.83E-04
**RP11-109I13.2**	-3.42	1.51E-04	3.83E-04
**ZNF728**	-3.74	1.51E-04	3.83E-04
**CWC22**	-0.43	1.51E-04	3.83E-04
**G28383**	-2.15	1.51E-04	3.84E-04
**RPL21P132**	-4.24	1.51E-04	3.84E-04
**DIP2A-IT1**	-3.61	1.51E-04	3.84E-04
**RP11-214K3.21**	-3.08	1.51E-04	3.84E-04
**GPSM2**	-0.49	1.51E-04	3.84E-04
**RP11-114F3.4**	-4.20	1.51E-04	3.85E-04
**TAS2R4**	-1.79	1.52E-04	3.85E-04
**OSBPL10-AS1**	-2.63	1.52E-04	3.85E-04
**RP3-486I3.7**	-3.30	1.52E-04	3.86E-04

**RP11-325E5.4**	-4.51	1.52E-04	3.86E-04
**GLT6D1**	-4.50	1.52E-04	3.86E-04
**TOB2**	0.38	1.52E-04	3.87E-04
**RP11-49K24.4**	-4.16	1.52E-04	3.87E-04
**RP13-991F5.2**	-4.54	1.52E-04	3.87E-04
**PLET1**	-2.63	1.53E-04	3.88E-04
**ZNF727**	-1.86	1.53E-04	3.89E-04
**RP11-329J18.3**	-2.41	1.53E-04	3.89E-04
**PAFAH1B2**	-0.46	1.53E-04	3.89E-04
**RP11-1055B8.10**	-4.31	1.53E-04	3.90E-04
**RP11-649A18.4**	-4.09	1.53E-04	3.90E-04
**FGF14-AS1**	-4.60	1.54E-04	3.90E-04
**NAMPT**	0.77	1.54E-04	3.90E-04
**RP11-307P5.1**	-4.70	1.54E-04	3.90E-04
**PPP1R7**	0.47	1.54E-04	3.90E-04
**ST6GALNAC4**	1.59	1.54E-04	3.90E-04
**RP11-57A1.1**	-2.76	1.54E-04	3.90E-04
**ABCB8**	0.56	1.54E-04	3.91E-04
**FBXO31**	0.38	1.54E-04	3.91E-04
**GATAD2B**	-0.64	1.54E-04	3.91E-04
**G10617**	-3.15	1.54E-04	3.91E-04
**DUOXA1**	0.77	1.54E-04	3.91E-04
**LSM11**	-0.50	1.54E-04	3.91E-04
**RP11-356J5.4**	-4.63	1.55E-04	3.93E-04
**RP11-754B17.1**	-2.54	1.55E-04	3.93E-04
**C8orf44**	-1.12	1.55E-04	3.93E-04
**CTC-499B15.6**	-3.56	1.55E-04	3.94E-04
**GAPDHP59**	-4.53	1.56E-04	3.94E-04
**RP11-211N11.5**	-2.66	1.56E-04	3.95E-04
**ST20**	-1.55	1.56E-04	3.95E-04
**KIAA0196-AS1**	-2.97	1.56E-04	3.95E-04
**PCDHAC2**	2.25	1.56E-04	3.95E-04
**RP11-84C10.3**	-4.73	1.56E-04	3.96E-04
**RP11-466F5.3**	-3.05	1.57E-04	3.97E-04
**MN1**	1.41	1.57E-04	3.98E-04
**PAQR8**	-0.91	1.58E-04	4.00E-04
**KIAA1614-AS1**	-4.38	1.58E-04	4.00E-04
**RP11-843A23.1**	-2.70	1.58E-04	4.01E-04
**CERS6-AS1**	-4.20	1.58E-04	4.01E-04
**AQP1**	1.19	1.58E-04	4.01E-04
**AC005288.1**	-4.61	1.59E-04	4.02E-04
**TAS1R3**	-1.54	1.59E-04	4.02E-04
**AC002401.1**	-4.52	1.59E-04	4.03E-04
**RN7SL65P**	-4.77	1.59E-04	4.03E-04
**AC011242.5**	-4.62	1.59E-04	4.03E-04

**VPRBP**	-0.39	1.60E-04	4.04E-04
**MBTPS1**	0.33	1.60E-04	4.04E-04
**FERMT2**	1.19	1.60E-04	4.04E-04
**RP11-420K8.1**	-3.16	1.60E-04	4.05E-04
**ZHX2**	0.56	1.60E-04	4.05E-04
**RP11-11M20.2**	-4.51	1.60E-04	4.05E-04
**SFMBT2**	0.96	1.60E-04	4.06E-04
**DNAJC27-AS1**	-1.01	1.60E-04	4.06E-04
**LINC00652**	-2.15	1.60E-04	4.06E-04
**ACP6**	-0.89	1.60E-04	4.06E-04
**SNORD113-4**	-4.44	1.61E-04	4.06E-04
**CTC-338M12.2**	-4.71	1.61E-04	4.07E-04
**GAL**	-4.05	1.61E-04	4.07E-04
**ARHGAP9**	1.42	1.61E-04	4.07E-04
**RP4-569M23.2**	-1.45	1.61E-04	4.07E-04
**SEZ6**	-2.32	1.61E-04	4.07E-04
**LSAMP**	-0.89	1.61E-04	4.07E-04
**KCTD5**	0.54	1.61E-04	4.07E-04
**CTD-2132N18.2**	1.58	1.62E-04	4.09E-04
**EIF4E2P1**	-4.53	1.62E-04	4.09E-04
**FAM60BP**	-4.41	1.62E-04	4.09E-04
**RP11-557J10.4**	-2.67	1.62E-04	4.09E-04
**PWWP2A**	-0.35	1.62E-04	4.10E-04
**KIAA0895L**	1.21	1.62E-04	4.10E-04
**RP13-455A7.1**	-3.69	1.62E-04	4.10E-04
**RP11-703M24.5**	-4.73	1.63E-04	4.11E-04
**RP11-423G4.10**	-4.16	1.63E-04	4.11E-04
**MIR544B**	-4.49	1.63E-04	4.11E-04
**RP11-480C16.1**	-3.08	1.63E-04	4.11E-04
**LINC00365**	-2.50	1.63E-04	4.11E-04
**SYNPR**	4.19	1.63E-04	4.11E-04
**KARSP1**	-4.25	1.63E-04	4.12E-04
**CTD-2371O3.2**	-3.17	1.63E-04	4.13E-04
**AC001226.7**	-4.03	1.64E-04	4.13E-04
**FARP1**	1.00	1.64E-04	4.13E-04
**ICAM5**	2.17	1.64E-04	4.15E-04
**RP1-80N2.2**	-2.36	1.64E-04	4.15E-04
**RNU6-817P**	-3.11	1.65E-04	4.15E-04
**RP11-68I3.11**	-4.04	1.65E-04	4.16E-04
**RP11-467L19.14**	-4.57	1.65E-04	4.16E-04
**TMEM99**	-1.13	1.65E-04	4.17E-04
**DCP1B**	0.54	1.66E-04	4.18E-04
**SCG3**	-2.01	1.66E-04	4.18E-04
**CDCA4P1**	-3.08	1.66E-04	4.18E-04
**2-Sep**	0.56	1.66E-04	4.18E-04

**RNU1-87P**	-4.72	1.66E-04	4.18E-04
**RN7SKP158**	-4.66	1.66E-04	4.18E-04
**RP11-299P2.2**	-4.58	1.66E-04	4.19E-04
**MINA**	-0.41	1.66E-04	4.19E-04
**TAS2R43**	-6.03	1.66E-04	4.19E-04
**RP11-401F2.4**	-4.43	1.66E-04	4.19E-04
**RP11-66H6.3**	-4.31	1.66E-04	4.19E-04
**XLOC_003804**	3.28	1.67E-04	4.20E-04
**AIG1P1**	-4.56	1.67E-04	4.20E-04
**MROH2A**	-1.45	1.67E-04	4.21E-04
**CTD-2049O4.1**	-3.03	1.67E-04	4.21E-04
**FDPSP7**	-4.63	1.67E-04	4.21E-04
**SCARNA16**	-4.44	1.67E-04	4.21E-04
**LATS2-AS1**	-4.44	1.67E-04	4.21E-04
**TRIM60P17**	-4.64	1.67E-04	4.22E-04
**MTCO3P11**	-3.62	1.67E-04	4.22E-04
**TNKS1BP1**	0.50	1.68E-04	4.24E-04
**RP1-228P16.1**	-2.59	1.68E-04	4.24E-04
**AC003090.1**	-2.91	1.69E-04	4.25E-04
**RHOBTB1**	0.77	1.69E-04	4.25E-04
**RCL1**	-0.51	1.69E-04	4.25E-04
**RP11-613M10.9**	-2.78	1.69E-04	4.26E-04
**RP11-430H12.2**	-4.14	1.69E-04	4.26E-04
**AFAP1-AS1**	-2.52	1.69E-04	4.26E-04
**DPH7**	0.77	1.69E-04	4.26E-04
**TACR2**	-1.03	1.69E-04	4.27E-04
**RP11-734K21.2**	-3.26	1.70E-04	4.27E-04
**RPL23AP35**	-4.54	1.70E-04	4.27E-04
**RAP1A**	0.52	1.70E-04	4.27E-04
**RP11-216L13.19**	-1.84	1.70E-04	4.27E-04
**ALOX15**	-1.67	1.70E-04	4.27E-04
**POMZP3**	1.33	1.70E-04	4.28E-04
**C19orf81**	1.68	1.70E-04	4.28E-04
**PJA1**	0.55	1.70E-04	4.28E-04
**SETP10**	-4.62	1.70E-04	4.28E-04
**RP11-881M11.8**	-4.50	1.70E-04	4.28E-04
**TMEM258**	0.53	1.70E-04	4.28E-04
**XLOC_003406**	-1.64	1.71E-04	4.29E-04
**SLC4A9**	-3.47	1.71E-04	4.29E-04
**G1561**	-1.90	1.71E-04	4.29E-04
**ZNF345**	-0.46	1.71E-04	4.30E-04
**RP11-177C12.1**	-0.98	1.71E-04	4.30E-04
**CH17-302M23.1**	-2.45	1.71E-04	4.30E-04
**RPL34-AS1**	-1.48	1.71E-04	4.30E-04
**G23007**	-2.61	1.71E-04	4.30E-04

**AC007285.7**	-3.07	1.71E-04	4.31E-04
**BBS4**	0.52	1.71E-04	4.31E-04
**RNY4P8**	-4.43	1.72E-04	4.31E-04
**HCP5**	-0.74	1.72E-04	4.31E-04
**RGN**	1.71	1.72E-04	4.33E-04
**NDUFA6**	0.69	1.72E-04	4.33E-04
**COQ6**	-0.98	1.72E-04	4.33E-04
**LELP1**	-4.44	1.72E-04	4.33E-04
**SNX18P9**	-4.63	1.73E-04	4.34E-04
**NAALAD2**	-1.42	1.73E-04	4.34E-04
**RP11-20I20.2**	-4.30	1.73E-04	4.34E-04
**SLURP1**	1.26	1.73E-04	4.35E-04
**RP4-535B20.4**	-4.54	1.73E-04	4.35E-04
**AL353698.1**	-4.58	1.73E-04	4.35E-04
**G1920**	-4.58	1.73E-04	4.35E-04
**RP11-28G8.1**	-2.10	1.73E-04	4.35E-04
**RP11-50D9.4**	-1.94	1.74E-04	4.36E-04
**RP11-31F19.1**	-4.78	1.74E-04	4.36E-04
**FDPSP3**	-4.33	1.74E-04	4.37E-04
**WNT8B**	-4.11	1.74E-04	4.37E-04
**KLRAP1**	-1.11	1.74E-04	4.37E-04
**RN7SL535P**	-2.47	1.74E-04	4.38E-04
**DDAH1**	-1.02	1.75E-04	4.39E-04
**RHOQ**	0.49	1.75E-04	4.39E-04
**TPRA1**	0.38	1.75E-04	4.40E-04
**RN7SKP97**	-3.33	1.76E-04	4.41E-04
**PCCA**	-0.50	1.76E-04	4.41E-04
**CTD-2201E9.3**	-4.69	1.76E-04	4.41E-04
**N6AMT1**	-0.88	1.76E-04	4.42E-04
**HMGN1P2**	-4.43	1.77E-04	4.43E-04
**NHS**	-0.57	1.77E-04	4.43E-04
**ZFYVE27**	0.63	1.77E-04	4.43E-04
**ATP1B1**	1.09	1.77E-04	4.43E-04
**LTBP1**	1.45	1.77E-04	4.43E-04
**VN1R81P**	-1.83	1.77E-04	4.43E-04
**PSPC1**	0.37	1.77E-04	4.43E-04
**GAS6-AS2**	1.14	1.77E-04	4.44E-04
**EBNA1BP2**	0.58	1.77E-04	4.44E-04
**PSMA6**	-0.65	1.77E-04	4.44E-04
**RP11-500G10.5**	-4.22	1.78E-04	4.45E-04
**CDK15**	-1.22	1.78E-04	4.45E-04
**UPF3AP3**	1.70	1.78E-04	4.46E-04
**TMED5**	-0.66	1.78E-04	4.47E-04
**PLEKHA8P1**	1.09	1.79E-04	4.48E-04
**RP11-298D21.1**	-3.15	1.79E-04	4.48E-04

**RP11-930O11.3**	-3.52	1.79E-04	4.49E-04
**RNU6-853P**	-4.83	1.80E-04	4.50E-04
**MSRB3**	1.03	1.80E-04	4.50E-04
**TOMM70A**	-0.48	1.80E-04	4.50E-04
**D2HGDH**	1.07	1.80E-04	4.50E-04
**RP11-34A14.3**	-4.19	1.80E-04	4.50E-04
**RP11-44D5.1**	-4.18	1.80E-04	4.50E-04
**RP11-429E11.3**	-4.75	1.80E-04	4.51E-04
**RP11-213G2.3**	-0.79	1.80E-04	4.51E-04
**CPNE9**	-2.16	1.81E-04	4.52E-04
**PPP1R8P1**	-4.15	1.81E-04	4.53E-04
**RPS18P9**	0.62	1.82E-04	4.54E-04
**RBMXL1**	-0.50	1.82E-04	4.55E-04
**SPOCK2**	1.31	1.82E-04	4.55E-04
**AC078852.2**	-4.47	1.82E-04	4.55E-04
**AC004067.5**	-1.16	1.82E-04	4.55E-04
**RP11-474G23.3**	-3.19	1.82E-04	4.56E-04
**RBBP4**	-0.34	1.82E-04	4.56E-04
**COL26A1**	2.66	1.82E-04	4.56E-04
**RP11-309L24.10**	-2.13	1.82E-04	4.56E-04
**CHCHD4**	0.54	1.83E-04	4.57E-04
**CDKL5**	-0.45	1.83E-04	4.57E-04
**RP11-231I16.1**	-1.66	1.83E-04	4.57E-04
**C2orf69**	-0.61	1.83E-04	4.57E-04
**AC007308.6**	-2.50	1.83E-04	4.58E-04
**CAMK2N2**	3.07	1.83E-04	4.59E-04
**RP11-596D21.1**	-1.86	1.84E-04	4.59E-04
**MARCKSL1P1**	-3.14	1.84E-04	4.59E-04
**AADACP1**	-2.50	1.84E-04	4.60E-04
**GALNT8**	-1.79	1.84E-04	4.60E-04
**PARP1**	0.44	1.85E-04	4.61E-04
**MBLAC2**	-0.68	1.85E-04	4.62E-04
**AC131571.2**	-2.62	1.85E-04	4.63E-04
**STARD13**	1.01	1.85E-04	4.63E-04
**RP11-818O24.3**	-1.99	1.86E-04	4.63E-04
**ALG13**	-0.67	1.86E-04	4.63E-04
**RNA5S9**	-4.85	1.86E-04	4.64E-04
**ARID4B**	0.44	1.86E-04	4.64E-04
**RPS3AP35**	-4.48	1.86E-04	4.64E-04
**RP11-67L14.1**	-4.68	1.86E-04	4.65E-04
**AC004022.8**	-3.67	1.86E-04	4.65E-04
**ZNHIT6**	-0.41	1.86E-04	4.65E-04
**CTC-218B8.3**	-4.48	1.86E-04	4.65E-04
**RNU6-254P**	-4.25	1.87E-04	4.66E-04
**AIF1L**	-0.82	1.87E-04	4.66E-04

**HSPA8P3**	-4.47	1.87E-04	4.67E-04
**RP11-116K4.1**	-3.23	1.87E-04	4.68E-04
**RP11-165D6.1**	-3.74	1.88E-04	4.68E-04
**GCM2**	-4.77	1.88E-04	4.68E-04
**G41001**	1.99	1.88E-04	4.68E-04
**RN7SL146P**	-4.52	1.88E-04	4.69E-04
**FAM64A**	0.82	1.88E-04	4.69E-04
**RP4-539M6.20**	-2.38	1.88E-04	4.69E-04
**AC092798.2**	-2.65	1.89E-04	4.70E-04
**UPK3B**	-1.88	1.89E-04	4.71E-04
**SNRPGP14**	-2.23	1.89E-04	4.71E-04
**SRSF11**	0.71	1.89E-04	4.71E-04
**APOC1**	-2.85	1.89E-04	4.72E-04
**SLC25A15P5**	-4.30	1.90E-04	4.74E-04
**ACTL7A**	-4.58	1.90E-04	4.74E-04
**HLA-B**	0.84	1.90E-04	4.74E-04
**ELOVL7**	-0.98	1.90E-04	4.74E-04
**RP11-1379J22.2**	-1.54	1.90E-04	4.74E-04
**IQCJ-SCHIP1-AS1**	-4.61	1.90E-04	4.75E-04
**ZNF649-AS1**	-4.33	1.90E-04	4.75E-04
**GUCY1B2**	-5.77	1.91E-04	4.75E-04
**RP11-440D17.4**	-1.54	1.91E-04	4.75E-04
**POP1**	-0.65	1.91E-04	4.76E-04
**RP11-216N14.5**	-2.45	1.91E-04	4.76E-04
**MCTS2P**	-1.11	1.91E-04	4.77E-04
**RP11-347C18.5**	-1.56	1.91E-04	4.77E-04
**RP11-1396O13.1**	-4.52	1.91E-04	4.77E-04
**SRPX**	1.25	1.92E-04	4.77E-04
**TMEM181**	-0.39	1.92E-04	4.77E-04
**HMGN2P30**	-4.51	1.92E-04	4.78E-04
**MARK3P1**	-4.53	1.92E-04	4.79E-04
**RP11-839G9.1**	-2.44	1.93E-04	4.81E-04
**G15649**	-2.51	1.93E-04	4.81E-04
**BX649597.1**	-3.28	1.93E-04	4.81E-04
**RP11-274J7.2**	-4.70	1.93E-04	4.81E-04
**LAMA3**	-0.48	1.93E-04	4.81E-04
**RP11-159H22.2**	-2.03	1.93E-04	4.82E-04
**TBP**	0.50	1.94E-04	4.83E-04
**PTPRU**	0.78	1.94E-04	4.84E-04
**CPB2-AS1**	-2.22	1.94E-04	4.84E-04
**BNIP3P24**	-3.34	1.95E-04	4.84E-04
**NDUFAF3**	0.52	1.95E-04	4.86E-04
**AC016745.3**	-3.26	1.95E-04	4.86E-04
**VPS51**	0.50	1.95E-04	4.86E-04
**RP11-394O2.3**	-2.80	1.96E-04	4.88E-04

**MAP4K2**	0.51	1.96E-04	4.88E-04
**RP11-255C15.3**	-2.16	1.96E-04	4.88E-04
**AP4B1**	0.64	1.96E-04	4.88E-04
**INO80B**	-1.84	1.97E-04	4.89E-04
**RP11-687F6.5**	-2.87	1.97E-04	4.90E-04
**UBE2Q1-AS1**	-3.20	1.97E-04	4.90E-04
**NEK4**	-0.43	1.97E-04	4.90E-04
**RP11-379J5.5**	-2.59	1.97E-04	4.91E-04
**LIX1L**	0.93	1.98E-04	4.91E-04
**RP11-299P2.1**	-4.33	1.98E-04	4.91E-04
**RP11-15F12.3**	-4.66	1.98E-04	4.91E-04
**RP11-33E12.2**	-1.32	1.98E-04	4.91E-04
**CTD-2027I19.3**	-2.10	1.98E-04	4.93E-04
**ZNF736P6Y**	-5.62	1.98E-04	4.93E-04
**OR5P4P**	-4.63	1.98E-04	4.93E-04
**MIR101-1**	-4.30	1.98E-04	4.93E-04
**RP11-382A20.1**	-4.35	1.98E-04	4.93E-04
**GSPT1**	-0.41	1.98E-04	4.93E-04
**C17orf50**	-2.33	1.99E-04	4.93E-04
**G40352**	-3.27	1.99E-04	4.94E-04
**NOP9**	-0.33	1.99E-04	4.94E-04
**AL160291.1**	-4.44	1.99E-04	4.95E-04
**G29948**	1.79	1.99E-04	4.95E-04
**G34276**	-1.34	1.99E-04	4.95E-04
**RP11-367J11.3**	-1.38	1.99E-04	4.95E-04
**RNU2-46P**	-4.48	2.00E-04	4.95E-04
**LINC00678**	-4.34	2.00E-04	4.95E-04
**MSRB1**	0.95	2.00E-04	4.95E-04
**XLOC_001134**	-4.27	2.00E-04	4.96E-04
**RP11-421E14.2**	-1.45	2.00E-04	4.96E-04
**RP11-278C7.2**	-4.17	2.00E-04	4.97E-04
**CDC7**	-0.92	2.00E-04	4.97E-04
**RP11-397E7.1**	-4.30	2.00E-04	4.97E-04
**RAP1GDS1**	-0.45	2.00E-04	4.97E-04
**SCML2P2**	-1.83	2.01E-04	4.99E-04
**RP11-296A18.5**	-2.58	2.01E-04	4.99E-04
**RP11-115D19.3**	-4.26	2.01E-04	4.99E-04
**PABPN1L**	-4.26	2.02E-04	5.01E-04
**NINL**	0.94	2.02E-04	5.01E-04
**NOSTRIN**	1.30	2.02E-04	5.01E-04
**RP11-184I16.4**	-3.09	2.02E-04	5.01E-04
**GIT2**	-0.51	2.02E-04	5.01E-04
**HSPA2**	0.87	2.03E-04	5.02E-04
**KLF3P1**	-4.26	2.03E-04	5.03E-04
**RP11-305E6.4**	-1.23	2.03E-04	5.03E-04

**PSMD10**	0.62	2.03E-04	5.04E-04
**FAM46B**	1.00	2.04E-04	5.05E-04
**AC009506.1**	1.17	2.04E-04	5.05E-04
**TSSC1**	0.49	2.04E-04	5.06E-04
**RP11-20O24.1**	-4.42	2.04E-04	5.06E-04
**AL590762.11**	-3.22	2.04E-04	5.06E-04
**STON1**	1.05	2.04E-04	5.06E-04
**RP5-1100E15.4**	-4.31	2.05E-04	5.07E-04
**RPL9P9**	3.46	2.05E-04	5.07E-04
**RP11-305L7.6**	2.55	2.05E-04	5.08E-04
**XLOC_008000**	-4.38	2.06E-04	5.09E-04
**RP11-480D4.7**	-3.47	2.06E-04	5.11E-04
**NHEJ1**	-3.25	2.07E-04	5.13E-04
**AC023137.2**	-2.94	2.07E-04	5.13E-04
**RP4-733B9.1**	-4.65	2.07E-04	5.13E-04
**RP11-139H15.7**	-3.05	2.07E-04	5.13E-04
**DRC3**	-0.97	2.07E-04	5.13E-04
**STX2**	1.08	2.08E-04	5.14E-04
**RP11-14I17.1**	-2.76	2.08E-04	5.14E-04
**RNU6-495P**	-3.95	2.08E-04	5.14E-04
**RN7SL67P**	-4.49	2.08E-04	5.14E-04
**ZNF480**	-0.53	2.08E-04	5.14E-04
**RP4-537K23.4**	-4.23	2.08E-04	5.15E-04
**TAF11**	0.37	2.08E-04	5.15E-04
**ALMS1-IT1**	-1.21	2.08E-04	5.15E-04
**RP11-266L9.5**	-1.52	2.08E-04	5.15E-04
**C1orf50**	0.41	2.08E-04	5.15E-04
**RP11-138I1.3**	-1.37	2.09E-04	5.17E-04
**ACTR1B**	0.45	2.09E-04	5.17E-04
**LRRC27**	0.75	2.09E-04	5.17E-04
**SPDYE5**	-1.48	2.09E-04	5.17E-04
**AC140076.1**	-4.12	2.09E-04	5.17E-04
**RP4-697P8.3**	-4.55	2.09E-04	5.17E-04
**AC024568.1**	-4.48	2.10E-04	5.18E-04
**MIR491**	-4.34	2.10E-04	5.18E-04
**RP11-968O1.5**	-1.58	2.10E-04	5.18E-04
**UPK3BP1**	-4.20	2.10E-04	5.20E-04
**MANEAL**	1.05	2.11E-04	5.21E-04
**SPATA9**	-1.45	2.11E-04	5.21E-04
**ACAD11**	-3.90	2.11E-04	5.21E-04
**PCDHGA6**	-0.70	2.11E-04	5.21E-04
**AC004987.9**	1.51	2.12E-04	5.23E-04
**CNTN6**	3.37	2.12E-04	5.24E-04
**MYO1G**	1.25	2.12E-04	5.24E-04
**RP5-1021I20.4**	-4.25	2.12E-04	5.24E-04

**ALOX12P1**	-4.44	2.12E-04	5.24E-04
**BMS1P20**	-2.30	2.12E-04	5.24E-04
**RP1-59D14.8**	-4.51	2.13E-04	5.25E-04
**TGFB2**	-0.99	2.13E-04	5.26E-04
**RP11-178L8.5**	-4.92	2.13E-04	5.26E-04
**VWA9**	0.37	2.13E-04	5.27E-04
**RN7SL153P**	-4.42	2.13E-04	5.27E-04
**AKAP8L**	0.62	2.14E-04	5.29E-04
**TTC1**	0.54	2.15E-04	5.31E-04
**PCDH1**	0.50	2.15E-04	5.31E-04
**RP11-184J23.2**	-4.83	2.16E-04	5.32E-04
**RP11-270M14.4**	-3.58	2.16E-04	5.33E-04
**RP11-386I14.2**	-2.09	2.17E-04	5.35E-04
**MCEE**	0.56	2.17E-04	5.35E-04
**RNU7-70P**	-4.19	2.17E-04	5.35E-04
**RNU7-16P**	-4.48	2.17E-04	5.36E-04
**RP11-73G16.1**	-4.31	2.17E-04	5.36E-04
**CCT5P1**	-4.33	2.18E-04	5.36E-04
**RP4-603I14.3**	-4.39	2.18E-04	5.37E-04
**RP11-191L9.5**	-3.47	2.18E-04	5.37E-04
**ZBTB5**	-0.38	2.18E-04	5.37E-04
**RP5-1065J22.2**	-4.07	2.18E-04	5.37E-04
**TMEM201**	0.71	2.18E-04	5.38E-04
**RP11-724M22.1**	-3.24	2.18E-04	5.38E-04
**ACSL1**	-1.26	2.18E-04	5.38E-04
**AC019117.1**	-3.27	2.19E-04	5.39E-04
**RP11-512M8.11**	-3.43	2.19E-04	5.39E-04
**RP11-154F14.3**	-4.58	2.19E-04	5.40E-04
**SNORD78**	-1.72	2.19E-04	5.40E-04
**NCF1C**	2.29	2.19E-04	5.40E-04
**CTC-250P20.2**	-3.53	2.19E-04	5.40E-04
**RP5-1023B21.1**	-4.39	2.19E-04	5.40E-04
**KIF22**	0.44	2.20E-04	5.41E-04
**CTD-2566J3.1**	-3.80	2.20E-04	5.41E-04
**PICALM**	-0.34	2.20E-04	5.43E-04
**HNRNPA1P50**	-2.33	2.20E-04	5.43E-04
**RAD50**	-0.37	2.21E-04	5.43E-04
**MIR4762**	-4.57	2.21E-04	5.44E-04
**CABLES2**	0.66	2.21E-04	5.44E-04
**IGF2BP3**	-1.39	2.21E-04	5.45E-04
**RNU1-109P**	-4.43	2.22E-04	5.46E-04
**PDXDC2P**	-1.15	2.22E-04	5.47E-04
**CHMP4BP1**	-2.47	2.22E-04	5.47E-04
**RN7SKP292**	-2.92	2.22E-04	5.47E-04
**SGF29**	0.50	2.23E-04	5.48E-04

**G32334**	-3.01	2.24E-04	5.50E-04
**TMEM107**	0.70	2.24E-04	5.50E-04
**RP11-403E24.1**	-4.21	2.24E-04	5.51E-04
**PGAM1P11**	-4.13	2.24E-04	5.51E-04
**LRFN5**	-1.51	2.24E-04	5.51E-04
**RPL31P58**	-4.19	2.25E-04	5.52E-04
**NEDD4**	-0.57	2.25E-04	5.53E-04
**RP3-507I15.2**	-4.45	2.25E-04	5.53E-04
**PSMB6**	0.66	2.25E-04	5.54E-04
**RP11-1006G14.1**	-4.42	2.25E-04	5.54E-04
**RP11-674I16.2**	-4.55	2.25E-04	5.54E-04
**RP11-505C13.1**	-4.54	2.26E-04	5.55E-04
**LINC00852**	-1.12	2.26E-04	5.55E-04
**G35150**	-4.04	2.26E-04	5.55E-04
**XLOC_007830**	-4.17	2.26E-04	5.56E-04
**AP1G2**	0.78	2.26E-04	5.57E-04
**AC015726.1**	-4.46	2.27E-04	5.57E-04
**MFI2**	2.65	2.27E-04	5.57E-04
**FOXN4**	-4.73	2.27E-04	5.57E-04
**PSMB1**	0.55	2.27E-04	5.57E-04
**FAM46A**	-0.62	2.27E-04	5.59E-04
**ZNF146**	-0.46	2.28E-04	5.59E-04
**GOLGA6L3**	4.31	2.28E-04	5.59E-04
**MRPL23-AS1**	-2.30	2.28E-04	5.60E-04
**RP11-818O24.2**	-2.96	2.29E-04	5.62E-04
**NTRK1**	3.88	2.29E-04	5.62E-04
**SPOCK1**	2.01	2.29E-04	5.63E-04
**SNORA1**	-4.49	2.29E-04	5.63E-04
**FIP1L1**	-0.48	2.29E-04	5.63E-04
**INVS**	-0.46	2.30E-04	5.64E-04
**RP11-118E18.4**	-3.96	2.30E-04	5.65E-04
**GNE**	0.68	2.31E-04	5.68E-04
**PARP9**	0.83	2.32E-04	5.69E-04
**LRP2**	-3.36	2.32E-04	5.69E-04
**FAM35A**	-0.47	2.32E-04	5.70E-04
**GGNBP2**	0.35	2.32E-04	5.70E-04
**HNRNPA3P5**	3.11	2.32E-04	5.70E-04
**RP11-761I4.4**	-2.47	2.33E-04	5.72E-04
**RP11-331F4.4**	-2.91	2.33E-04	5.72E-04
**RP3-510D11.2**	1.26	2.33E-04	5.72E-04
**FAM218A**	-1.31	2.33E-04	5.72E-04
**RP11-57H14.5**	-3.17	2.33E-04	5.72E-04
**MDC1**	0.62	2.34E-04	5.73E-04
**CYCSP24**	-4.21	2.34E-04	5.73E-04
**GZMM**	2.11	2.34E-04	5.74E-04

**G30853**	-3.73	2.34E-04	5.75E-04
**TYRP1**	-1.63	2.35E-04	5.75E-04
**VWC2L**	-4.80	2.35E-04	5.75E-04
**AC007679.4**	-4.54	2.35E-04	5.76E-04
**RP11-111H13.1**	-2.81	2.35E-04	5.76E-04
**YOD1**	-1.26	2.35E-04	5.76E-04
**CDC14A**	-0.62	2.36E-04	5.78E-04
**KBTBD6**	-0.54	2.36E-04	5.79E-04
**MIR4774**	-4.15	2.36E-04	5.79E-04
**ACBD4**	0.69	2.36E-04	5.79E-04
**RP11-378G13.2**	-4.21	2.36E-04	5.79E-04
**TMEM191C**	1.84	2.36E-04	5.79E-04
**ADCK3**	0.58	2.36E-04	5.79E-04
**CTD-2116N17.1**	-4.34	2.37E-04	5.80E-04
**BNIP3P25**	-3.60	2.37E-04	5.80E-04
**BRD9P2**	-3.20	2.37E-04	5.80E-04
**ZNF552**	-0.46	2.37E-04	5.80E-04
**RBM28**	-0.57	2.37E-04	5.80E-04
**SPG20**	-0.52	2.37E-04	5.81E-04
**TMEM254**	-0.70	2.38E-04	5.82E-04
**VLDLR-AS1**	1.86	2.38E-04	5.82E-04
**PDXK**	0.61	2.38E-04	5.82E-04
**RP11-9H20.2**	-3.37	2.38E-04	5.82E-04
**C1QBPP2**	-2.05	2.38E-04	5.83E-04
**RP11-271C24.2**	-3.90	2.38E-04	5.83E-04
**RN7SL30P**	-4.49	2.38E-04	5.83E-04
**ZMAT2**	0.61	2.38E-04	5.83E-04
**HSD3BP2**	-6.46	2.38E-04	5.84E-04
**RP11-104N10.2**	-1.19	2.39E-04	5.84E-04
**RP11-426J5.3**	-1.81	2.39E-04	5.84E-04
**RP11-417F21.1**	-2.53	2.39E-04	5.85E-04
**PHF20L1**	-0.48	2.39E-04	5.86E-04
**RP1-63M2.5**	-4.39	2.40E-04	5.87E-04
**ABCA13**	-1.98	2.40E-04	5.88E-04
**AGRP**	-2.41	2.40E-04	5.88E-04
**G10846**	-1.46	2.40E-04	5.88E-04
**DDIAS**	-0.76	2.41E-04	5.88E-04
**PCNXL2**	-0.70	2.41E-04	5.89E-04
**TLN2**	-0.86	2.41E-04	5.89E-04
**PDCL3**	0.65	2.42E-04	5.91E-04
**AC008671.1**	-4.10	2.42E-04	5.91E-04
**KRT16P6**	3.25	2.42E-04	5.91E-04
**RP1-140J1.4**	-4.69	2.42E-04	5.91E-04
**DISP1**	-0.61	2.42E-04	5.91E-04
**EHD1**	0.43	2.42E-04	5.91E-04

**RCAN2**	1.11	2.42E-04	5.91E-04
**METTL2B**	-0.46	2.42E-04	5.91E-04
**RP11-458I7.1**	-4.44	2.42E-04	5.92E-04
**DUSP14**	0.93	2.42E-04	5.92E-04
**TCEB3B**	-4.23	2.43E-04	5.93E-04
**MIR548V**	-3.92	2.43E-04	5.94E-04
**RP5-1147A1.2**	-4.42	2.43E-04	5.94E-04
**RP11-166B2.3**	-1.74	2.43E-04	5.94E-04
**RP5-1077I2.3**	-2.58	2.44E-04	5.95E-04
**AC092755.4**	-4.22	2.44E-04	5.95E-04
**BCDIN3D-AS1**	-1.78	2.44E-04	5.96E-04
**CTC-325H20.7**	-3.94	2.44E-04	5.96E-04
**RP11-711G10.1**	-4.46	2.45E-04	5.98E-04
**C6orf120**	-0.50	2.45E-04	5.99E-04
**FTH1P25**	-4.70	2.45E-04	5.99E-04
**ZSCAN32**	-1.49	2.46E-04	6.00E-04
**LINC00467**	0.73	2.46E-04	6.01E-04
**MTM1**	-0.46	2.46E-04	6.01E-04
**ZNF324B**	-0.51	2.46E-04	6.01E-04
**RP11-115D19.2**	-3.34	2.46E-04	6.01E-04
**SOX2**	1.48	2.46E-04	6.01E-04
**SLC8A1-AS1**	-2.21	2.47E-04	6.02E-04
**MCM10**	-0.72	2.47E-04	6.02E-04
**RP4-541C22.5**	-1.70	2.47E-04	6.03E-04
**TIMM9P2**	-1.88	2.47E-04	6.04E-04
**MIR9-3HG**	1.74	2.48E-04	6.04E-04
**RN7SKP50**	-4.42	2.48E-04	6.04E-04
**RP11-108P20.2**	-4.26	2.48E-04	6.05E-04
**RP11-1000B6.8**	-2.10	2.49E-04	6.06E-04
**RP11-110H1.9**	-4.30	2.49E-04	6.06E-04
**PTPRE**	0.71	2.49E-04	6.08E-04
**TRIM16L**	0.76	2.50E-04	6.10E-04
**ADGRG1**	0.62	2.51E-04	6.11E-04
**CTB-179K24.3**	-2.15	2.51E-04	6.11E-04
**MTND5P32**	-4.49	2.51E-04	6.11E-04
**BTG3**	0.53	2.51E-04	6.12E-04
**CBWD3**	-0.97	2.51E-04	6.12E-04
**THEMIS3P**	-4.10	2.51E-04	6.13E-04
**XLOC_009920**	3.47	2.51E-04	6.13E-04
**CXCL11**	3.09	2.52E-04	6.14E-04
**KDM1B**	-0.45	2.52E-04	6.15E-04
**RP5-1107A17.2**	-4.34	2.52E-04	6.15E-04
**INSM2**	-3.14	2.53E-04	6.17E-04
**OAS1**	1.00	2.53E-04	6.17E-04
**B4GALNT1**	1.91	2.54E-04	6.18E-04

**CNDP1**	-3.24	2.54E-04	6.18E-04
**OXCT1**	-0.62	2.54E-04	6.18E-04
**CHRNB1**	0.52	2.54E-04	6.18E-04
**RNU6-307P**	-4.32	2.54E-04	6.20E-04
**TRIM71**	-4.93	2.55E-04	6.21E-04
**RP4-705D16.3**	-2.73	2.55E-04	6.21E-04
**AC068587.2**	-4.11	2.55E-04	6.22E-04
**CAB39**	-0.47	2.55E-04	6.22E-04
**RP11-93I21.3**	-2.33	2.56E-04	6.22E-04
**PRDX3**	0.57	2.56E-04	6.22E-04
**IFT46**	0.45	2.56E-04	6.23E-04
**PPFIA1**	-0.27	2.56E-04	6.24E-04
**LINC00305**	-4.36	2.56E-04	6.24E-04
**XLOC_000647**	0.90	2.57E-04	6.25E-04
**PIK3CG**	-1.05	2.57E-04	6.25E-04
**RPS14P8**	-3.37	2.57E-04	6.25E-04
**RP11-184A2.2**	-2.04	2.57E-04	6.26E-04
**TSPYL6**	-3.66	2.58E-04	6.26E-04
**RP11-429G19.3**	-0.90	2.58E-04	6.27E-04
**RP11-144I2.1**	-1.96	2.58E-04	6.27E-04
**HOXD13**	3.22	2.58E-04	6.27E-04
**AP000936.1**	-2.16	2.58E-04	6.28E-04
**COMMD10**	-0.42	2.59E-04	6.29E-04
**RPL6P12**	-4.51	2.59E-04	6.29E-04
**RP11-132G19.3**	-4.31	2.59E-04	6.29E-04
**RP11-269G24.2**	-4.35	2.59E-04	6.29E-04
**XLOC_009603**	-4.01	2.59E-04	6.29E-04
**FUK**	0.61	2.59E-04	6.29E-04
**AC007557.2**	-4.32	2.60E-04	6.31E-04
**RNA5SP33**	-4.55	2.60E-04	6.31E-04
**APELA**	1.96	2.60E-04	6.31E-04
**RP11-592N21.2**	-4.21	2.60E-04	6.31E-04
**RP11-43D2.2**	-4.53	2.60E-04	6.31E-04
**TLR10**	-1.49	2.60E-04	6.32E-04
**RAB27A**	0.55	2.60E-04	6.32E-04
**CTC-327F10.5**	-1.44	2.60E-04	6.32E-04
**ANO3**	-1.30	2.61E-04	6.33E-04
**CTC-487M23.8**	-4.16	2.61E-04	6.33E-04
**PLCG1-AS1**	-1.47	2.61E-04	6.33E-04
**HOXC-AS3**	1.79	2.61E-04	6.34E-04
**RPL23AP52**	-4.31	2.61E-04	6.34E-04
**SFTPD-AS1**	-4.10	2.61E-04	6.34E-04
**GLRB**	-1.63	2.61E-04	6.34E-04
**RP11-584P21.2**	-2.14	2.61E-04	6.35E-04
**DCAKD**	0.60	2.62E-04	6.35E-04

**WFS1**	0.61	2.62E-04	6.36E-04
**CTD-2301A4.1**	-4.33	2.62E-04	6.36E-04
**TRAM2**	0.82	2.62E-04	6.36E-04
**GNL2P1**	-4.46	2.62E-04	6.36E-04
**HNRNPH1P1**	-2.43	2.62E-04	6.37E-04
**RP11-589F5.3**	-4.73	2.63E-04	6.37E-04
**ZC3H12D**	-1.17	2.63E-04	6.38E-04
**RN7SL258P**	-4.21	2.63E-04	6.38E-04
**CD46**	-0.36	2.63E-04	6.38E-04
**AC109642.1**	2.58	2.63E-04	6.39E-04
**G24512**	-4.34	2.64E-04	6.40E-04
**CTC-425O23.2**	-2.10	2.64E-04	6.40E-04
**G25498**	-2.13	2.65E-04	6.41E-04
**CCDC68**	-1.17	2.65E-04	6.41E-04
**G43485**	-4.92	2.65E-04	6.42E-04
**ZMYM4-AS1**	-3.21	2.65E-04	6.42E-04
**SNX25**	-0.42	2.65E-04	6.42E-04
**LTBP4**	1.05	2.65E-04	6.42E-04
**AC144525.1**	-4.31	2.65E-04	6.42E-04
**PFAS**	0.59	2.66E-04	6.44E-04
**AC108463.2**	4.48	2.66E-04	6.44E-04
**RP11-799B12.1**	-1.29	2.66E-04	6.45E-04
**XLOC_012841**	1.82	2.66E-04	6.45E-04
**PGRMC2**	-0.59	2.66E-04	6.45E-04
**RP1-168L15.5**	-2.88	2.66E-04	6.45E-04
**RP11-394I13.2**	-2.50	2.66E-04	6.45E-04
**AC026740.1**	-1.37	2.66E-04	6.46E-04
**SPATA2**	0.54	2.67E-04	6.46E-04
**RP11-166B2.8**	-2.07	2.68E-04	6.48E-04
**UBE2B**	0.55	2.68E-04	6.50E-04
**NEK3**	0.63	2.68E-04	6.50E-04
**RP11-327F22.6**	-1.68	2.69E-04	6.50E-04
**RP13-216E22.5**	-4.11	2.69E-04	6.51E-04
**CTD-2373H9.5**	-4.14	2.69E-04	6.52E-04
**PPARGC1B**	-0.95	2.70E-04	6.53E-04
**OLFML3**	1.72	2.70E-04	6.53E-04
**RP11-363J20.1**	-3.59	2.70E-04	6.54E-04
**UBE2FP2**	-4.38	2.70E-04	6.54E-04
**CBR3-AS1**	-1.00	2.71E-04	6.55E-04
**RP11-151A6.6**	-3.46	2.71E-04	6.56E-04
**RPL7P49**	-3.22	2.72E-04	6.58E-04
**CTC-429L19.3**	-2.41	2.72E-04	6.59E-04
**CTD-2024F21.1**	-4.08	2.73E-04	6.60E-04
**CTD-2587H19.2**	-4.27	2.73E-04	6.60E-04
**AC087239.1**	-3.45	2.73E-04	6.60E-04

**HIST2H4A**	-1.88	2.73E-04	6.61E-04
**SHISA9**	3.04	2.73E-04	6.61E-04
**TFAM**	-0.51	2.73E-04	6.61E-04
**SERPINB4**	3.71	2.74E-04	6.62E-04
**ITCH-IT1**	-2.88	2.74E-04	6.63E-04
**RMI1**	-0.58	2.74E-04	6.63E-04
**PISD**	0.39	2.74E-04	6.63E-04
**RP5-862P8.2**	-0.94	2.75E-04	6.64E-04
**AC012485.1**	-4.14	2.75E-04	6.65E-04
**CTD-2542C24.9**	-4.20	2.76E-04	6.67E-04
**ARFGAP2**	0.37	2.76E-04	6.67E-04
**PKIA**	-0.84	2.76E-04	6.68E-04
**RIOK1**	0.50	2.76E-04	6.68E-04
**ACMSD**	-3.02	2.76E-04	6.68E-04
**RP11-385F5.4**	-4.03	2.77E-04	6.68E-04
**MIR23B**	-1.72	2.77E-04	6.68E-04
**RNU6-282P**	-4.06	2.77E-04	6.68E-04
**G36324**	-2.76	2.77E-04	6.70E-04
**TGDS**	-0.34	2.77E-04	6.70E-04
**RNU6-1142P**	-4.41	2.77E-04	6.70E-04
**G25511**	-2.14	2.78E-04	6.72E-04
**ZNF157**	-1.92	2.79E-04	6.73E-04
**AC011290.5**	-1.88	2.79E-04	6.73E-04
**PRDX2P1**	-4.31	2.79E-04	6.74E-04
**UBOX5-AS1**	-1.83	2.79E-04	6.74E-04
**CTD-3113P16.11**	-1.51	2.79E-04	6.74E-04
**KLHDC8A**	3.30	2.80E-04	6.75E-04
**CASC1**	-1.03	2.80E-04	6.76E-04
**KCNJ10**	-2.80	2.80E-04	6.76E-04
**RP4-654C18.1**	-3.52	2.81E-04	6.77E-04
**PMCHL1**	-5.49	2.81E-04	6.77E-04
**RNF139**	-0.50	2.81E-04	6.78E-04
**RNA5SP44**	-4.17	2.81E-04	6.78E-04
**RP11-99A14.1**	-4.42	2.81E-04	6.78E-04
**NUP35**	-0.54	2.81E-04	6.79E-04
**G41064**	-4.17	2.81E-04	6.79E-04
**CAPZA3**	-2.14	2.81E-04	6.79E-04
**RAB5CP1**	-2.03	2.81E-04	6.79E-04
**AC097662.2**	-1.24	2.82E-04	6.79E-04
**AL050321.1**	-4.26	2.82E-04	6.79E-04
**UGT3A1**	-4.22	2.82E-04	6.79E-04
**AC079354.6**	-4.45	2.82E-04	6.79E-04
**CSRP3**	-4.69	2.82E-04	6.79E-04
**AC012531.25**	-1.62	2.82E-04	6.79E-04
**XXbac-B444P24.10**	-1.65	2.82E-04	6.80E-04

**ZBTB20-AS3**	-3.08	2.82E-04	6.80E-04
**TXNL4A**	0.43	2.83E-04	6.82E-04
**AC012358.7**	-3.20	2.83E-04	6.82E-04
**ENPP7P5**	-4.44	2.83E-04	6.83E-04
**XLOC_001124**	-4.44	2.84E-04	6.84E-04
**NR3C1**	-0.51	2.84E-04	6.84E-04
**AC073215.1**	-4.22	2.85E-04	6.86E-04
**USP43**	0.87	2.85E-04	6.87E-04
**RP5-974N19.1**	-4.48	2.85E-04	6.87E-04
**CTD-2555A7.2**	3.37	2.85E-04	6.87E-04
**GLT8D1**	0.51	2.85E-04	6.87E-04
**C1orf109**	0.40	2.85E-04	6.87E-04
**RNU6-856P**	-4.50	2.86E-04	6.89E-04
**PRIM2**	-0.49	2.86E-04	6.89E-04
**MIR548O2**	-4.45	2.86E-04	6.89E-04
**RNU7-48P**	-4.40	2.86E-04	6.90E-04
**LNX2**	-0.54	2.86E-04	6.90E-04
**OSM**	3.18	2.87E-04	6.92E-04
**AC092535.3**	-2.40	2.88E-04	6.94E-04
**RNU4-42P**	-4.02	2.89E-04	6.95E-04
**CXCL1**	3.13	2.89E-04	6.95E-04
**NAB2**	0.46	2.89E-04	6.96E-04
**AP000525.9**	4.49	2.89E-04	6.96E-04
**ANKS3**	0.93	2.90E-04	6.97E-04
**AC006539.1**	-4.20	2.90E-04	6.98E-04
**XIAP**	-0.42	2.91E-04	7.00E-04
**CTD-2145A24.3**	-2.01	2.91E-04	7.00E-04
**RPL17P25**	-4.52	2.91E-04	7.00E-04
**RP11-255E6.5**	-4.42	2.91E-04	7.00E-04
**SNORA16B**	-3.46	2.91E-04	7.00E-04
**RNU6-577P**	-4.40	2.91E-04	7.00E-04
**SAMD11**	1.85	2.92E-04	7.01E-04
**RPS4XP14**	-3.08	2.92E-04	7.01E-04
**RPL5P14**	-4.54	2.92E-04	7.01E-04
**RPL37A**	0.60	2.92E-04	7.02E-04
**RP1-134O19.3**	-4.41	2.92E-04	7.03E-04
**RP11-119F19.5**	-0.90	2.93E-04	7.03E-04
**PLEK**	1.43	2.93E-04	7.04E-04
**C6orf132**	0.74	2.94E-04	7.05E-04
**RP11-266L9.8**	1.14	2.94E-04	7.06E-04
**RFX5**	0.59	2.94E-04	7.06E-04
**AC024475.1**	-4.42	2.94E-04	7.07E-04
**MIR627**	-4.27	2.94E-04	7.07E-04
**RP11-88I18.2**	-2.12	2.95E-04	7.08E-04
**NANOGP11**	-4.42	2.95E-04	7.08E-04

**ADIPOR1**	0.49	2.95E-04	7.09E-04
**PLLP**	-1.05	2.95E-04	7.09E-04
**PTTG1**	0.74	2.96E-04	7.10E-04
**HMGB3P21**	-4.34	2.96E-04	7.10E-04
**RP5-1099D15.1**	-2.10	2.96E-04	7.10E-04
**G25988**	-1.80	2.96E-04	7.11E-04
**RP11-361D15.2**	-2.95	2.96E-04	7.11E-04
**SLC2A10**	1.63	2.96E-04	7.11E-04
**TAS2R64P**	-5.74	2.96E-04	7.11E-04
**GTF2F2**	-0.76	2.96E-04	7.11E-04
**RP11-169K17.3**	-2.58	2.96E-04	7.11E-04
**LINC00271**	-1.50	2.97E-04	7.12E-04
**RNU6-701P**	-3.26	2.97E-04	7.13E-04
**AC016821.1**	-4.08	2.97E-04	7.13E-04
**SYCE1L**	0.94	2.97E-04	7.13E-04
**AKR1C2**	0.82	2.97E-04	7.13E-04
**GRK5-IT1**	-4.26	2.97E-04	7.13E-04
**TBX3**	0.83	2.97E-04	7.13E-04
**RP11-624D20.1**	-4.49	2.98E-04	7.14E-04
**ZNF93**	-0.75	2.98E-04	7.15E-04
**RNU6-548P**	-4.40	2.98E-04	7.15E-04
**RNU6-1005P**	-4.16	2.99E-04	7.16E-04
**PSG2**	-4.04	2.99E-04	7.16E-04
**MAPKBP1**	-0.61	2.99E-04	7.16E-04
**EPC2**	-0.40	2.99E-04	7.17E-04
**CTD-2358C21.5**	-3.02	2.99E-04	7.18E-04
**SUMO2P15**	-4.59	3.00E-04	7.18E-04
**RASA4**	1.18	3.00E-04	7.19E-04
**NR3C2**	-0.82	3.00E-04	7.19E-04
**NEK7**	-0.60	3.00E-04	7.19E-04
**AP003041.2**	-3.98	3.00E-04	7.20E-04
**C1orf216**	0.69	3.00E-04	7.20E-04
**RP11-333J10.3**	-3.21	3.00E-04	7.20E-04
**ZNF767P**	-0.92	3.00E-04	7.20E-04
**AP1B1**	0.44	3.00E-04	7.20E-04
**RN7SL733P**	-4.31	3.00E-04	7.20E-04
**SLC34A2**	4.01	3.00E-04	7.20E-04
**BMS1P5**	1.14	3.01E-04	7.21E-04
**RPL21P121**	-4.17	3.01E-04	7.22E-04
**PGLYRP2**	1.99	3.01E-04	7.22E-04
**RP11-322E11.5**	2.90	3.02E-04	7.23E-04
**MAGI1**	-0.43	3.03E-04	7.24E-04
**JTBP1**	-4.59	3.03E-04	7.25E-04
**FBXO8**	-0.54	3.03E-04	7.25E-04
**C20orf195**	1.97	3.03E-04	7.25E-04

**CTD-3239E11.2**	-3.50	3.03E-04	7.26E-04
**G28649**	-1.45	3.03E-04	7.26E-04
**XLOC_008100**	2.10	3.04E-04	7.27E-04
**CENPE**	-0.77	3.04E-04	7.27E-04
**RN7SKP124**	-4.28	3.04E-04	7.27E-04
**TMEM170B**	-1.10	3.04E-04	7.27E-04
**RP11-363H12.1**	-4.41	3.05E-04	7.29E-04
**KRT79**	-3.59	3.05E-04	7.30E-04
**TACC2**	-0.69	3.05E-04	7.31E-04
**RP1-265C24.5**	-4.19	3.06E-04	7.32E-04
**RNU5E-4P**	-4.07	3.06E-04	7.32E-04
**OVCH2**	-1.69	3.06E-04	7.32E-04
**RN7SKP287**	-3.26	3.06E-04	7.33E-04
**RP11-91P24.6**	-2.28	3.07E-04	7.33E-04
**WDR33**	-0.36	3.07E-04	7.34E-04
**SLC38A6**	-0.73	3.07E-04	7.35E-04
**RP11-291L19.1**	-3.98	3.08E-04	7.35E-04
**SCGB3A2**	-1.64	3.08E-04	7.37E-04
**DHX8**	-0.38	3.08E-04	7.37E-04
**FOXD2-AS1**	2.07	3.08E-04	7.37E-04
**TOMM22**	0.46	3.09E-04	7.38E-04
**C7orf60**	-0.57	3.09E-04	7.38E-04
**ZBTB11-AS1**	-0.69	3.09E-04	7.39E-04
**AMICA1**	1.34	3.09E-04	7.40E-04
**MRPL49**	0.42	3.10E-04	7.40E-04
**RP11-574O16.1**	-4.47	3.10E-04	7.41E-04
**DIP2C**	0.56	3.10E-04	7.41E-04
**G15375**	-0.99	3.10E-04	7.41E-04
**RP11-567F11.2**	-4.42	3.11E-04	7.42E-04
**RN7SL459P**	-4.86	3.12E-04	7.45E-04
**TMEM19**	-0.72	3.12E-04	7.46E-04
**ATG10-IT1**	-4.56	3.12E-04	7.46E-04
**GBE1**	0.55	3.13E-04	7.48E-04
**RP11-248E9.5**	-4.72	3.13E-04	7.48E-04
**ABLIM3**	1.02	3.14E-04	7.49E-04
**KRT18P15**	-4.10	3.14E-04	7.49E-04
**ZSCAN5D**	-4.34	3.14E-04	7.49E-04
**RP11-332H17.1**	-4.48	3.14E-04	7.50E-04
**RP11-313P13.5**	-3.67	3.15E-04	7.51E-04
**RP11-1124B17.1**	-3.94	3.16E-04	7.53E-04
**XLOC_010205**	-4.02	3.16E-04	7.53E-04
**CTC-529G1.1**	-2.39	3.16E-04	7.53E-04
**STAC2**	3.30	3.16E-04	7.53E-04
**RP11-65N13.6**	-4.10	3.16E-04	7.55E-04
**KRT41P**	-4.40	3.17E-04	7.55E-04

**SNORD74**	-4.40	3.17E-04	7.56E-04
**OR7E47P**	-1.36	3.17E-04	7.57E-04
**RP11-42I10.1**	2.10	3.17E-04	7.57E-04
**RP11-504P24.3**	-1.02	3.18E-04	7.58E-04
**ITFG1**	-0.41	3.18E-04	7.58E-04
**AL031594.1**	-4.45	3.18E-04	7.58E-04
**XLOC_013984**	5.10	3.18E-04	7.58E-04
**ATP6V1G1P6**	-4.38	3.18E-04	7.58E-04
**LMO4**	0.48	3.18E-04	7.59E-04
**ZNF616**	-0.50	3.19E-04	7.60E-04
**CCDC13**	1.65	3.19E-04	7.60E-04
**U51244.2**	-3.74	3.19E-04	7.60E-04
**RP11-707O23.1**	-2.86	3.19E-04	7.60E-04
**PLEKHG4B**	1.70	3.19E-04	7.61E-04
**SNORD70**	-3.04	3.20E-04	7.62E-04
**SNRPB2**	0.65	3.20E-04	7.64E-04
**BLZF1**	-0.50	3.21E-04	7.64E-04
**CHMP4A**	1.43	3.21E-04	7.64E-04
**RPS23P5**	-4.16	3.21E-04	7.64E-04
**PLA1A**	2.54	3.21E-04	7.64E-04
**AP000593.7**	-4.07	3.21E-04	7.65E-04
**TMEM131**	-0.45	3.21E-04	7.65E-04
**DNMT1**	0.43	3.21E-04	7.66E-04
**RP11-7M10.2**	-4.18	3.22E-04	7.66E-04
**EEF1A1**	0.55	3.22E-04	7.67E-04
**ADPGK-AS1**	-1.79	3.22E-04	7.67E-04
**RP11-386M24.6**	-2.25	3.22E-04	7.67E-04
**RP11-382D8.3**	-3.38	3.22E-04	7.67E-04
**AC067956.1**	-4.48	3.23E-04	7.68E-04
**RP11-84A12.1**	-4.32	3.23E-04	7.68E-04
**RP11-173P15.7**	-4.30	3.23E-04	7.69E-04
**PLBD1-AS1**	1.36	3.23E-04	7.69E-04
**ASH1L-IT1**	-4.14	3.25E-04	7.73E-04
**ATG2A**	0.57	3.25E-04	7.73E-04
**RP11-384M20.1**	-3.47	3.25E-04	7.73E-04
**CCDC18**	-0.80	3.25E-04	7.74E-04
**ERBB2**	0.51	3.25E-04	7.74E-04
**PCDHB19P**	-1.79	3.26E-04	7.75E-04
**RN7SL20P**	-4.00	3.26E-04	7.75E-04
**CCDC88B**	1.08	3.26E-04	7.76E-04
**RPARP-AS1**	-0.92	3.26E-04	7.76E-04
**PHLPP1**	-0.46	3.26E-04	7.76E-04
**FGD2**	1.02	3.26E-04	7.76E-04
**SUPT20H**	-0.46	3.26E-04	7.76E-04
**MYEF2**	-0.78	3.27E-04	7.77E-04

**AP001434.2**	4.10	3.27E-04	7.77E-04
**HMGXB4**	0.33	3.27E-04	7.78E-04
**ACHE**	1.59	3.28E-04	7.79E-04
**ANGPTL7**	-3.07	3.28E-04	7.79E-04
**SDHDP2**	-4.53	3.29E-04	7.81E-04
**RP11-177N22.3**	-4.40	3.29E-04	7.81E-04
**UNC79**	1.60	3.29E-04	7.82E-04
**MYLK-AS2**	-4.31	3.29E-04	7.82E-04
**SNHG24**	-1.91	3.29E-04	7.82E-04
**RFX2**	-1.14	3.29E-04	7.83E-04
**ZNF689**	0.54	3.29E-04	7.83E-04
**UTP11L**	0.43	3.29E-04	7.83E-04
**BDH1**	0.55	3.30E-04	7.83E-04
**GRIN1**	1.89	3.30E-04	7.84E-04
**LRRC37A7P**	-1.60	3.30E-04	7.84E-04
**IL22RA1**	-0.57	3.30E-04	7.84E-04
**HOTAIR**	2.37	3.30E-04	7.84E-04
**MCU**	-0.67	3.30E-04	7.85E-04
**AC006014.1**	2.09	3.30E-04	7.85E-04
**RP11-506H21.5**	-1.89	3.30E-04	7.85E-04
**RP11-708B6.2**	-4.42	3.31E-04	7.86E-04
**AP000695.4**	3.80	3.31E-04	7.86E-04
**RP11-109N23.5**	-4.04	3.31E-04	7.86E-04
**MIR1273C**	-4.24	3.31E-04	7.87E-04
**RP4-744I24.4**	-3.43	3.31E-04	7.87E-04
**XLOC_001066**	-4.47	3.32E-04	7.87E-04
**RP11-522L3.5**	-4.50	3.32E-04	7.87E-04
**RP11-861E21.2**	-1.60	3.32E-04	7.88E-04
**ZWILCH**	-0.49	3.32E-04	7.88E-04
**RP11-669C19.1**	-4.74	3.32E-04	7.88E-04
**TCF7L2**	0.74	3.32E-04	7.89E-04
**RP13-463N16.6**	3.60	3.32E-04	7.89E-04
**RAB3A**	1.09	3.33E-04	7.89E-04
**RP11-547D24.1**	3.41	3.33E-04	7.90E-04
**RP11-298I3.1**	-1.92	3.33E-04	7.90E-04
**HS3ST5**	-3.26	3.33E-04	7.90E-04
**RP11-196G11.4**	-1.89	3.34E-04	7.91E-04
**XLOC_002995**	-2.83	3.34E-04	7.91E-04
**RP11-797D24.3**	-2.17	3.34E-04	7.91E-04
**LINC00839**	1.38	3.34E-04	7.91E-04
**KATNB1**	0.64	3.34E-04	7.92E-04
**XLOC_014106**	-4.04	3.34E-04	7.93E-04
**CHST13**	3.78	3.35E-04	7.93E-04
**RP11-256K9.1**	-4.73	3.35E-04	7.93E-04
**NEDD8**	-0.53	3.35E-04	7.94E-04

**RP11-388P9.3**	-4.36	3.36E-04	7.96E-04
**AC007899.3**	-4.46	3.36E-04	7.96E-04
**CSRNP2**	0.35	3.36E-04	7.97E-04
**RNF175**	1.44	3.37E-04	7.98E-04
**TNFAIP6**	1.88	3.37E-04	7.98E-04
**RNU7-40P**	3.48	3.37E-04	7.99E-04
**RP11-40B20.1**	-4.21	3.38E-04	8.00E-04
**CTD-2659N19.9**	-4.39	3.38E-04	8.00E-04
**XLOC_013981**	-1.87	3.39E-04	8.02E-04
**CDH8**	2.03	3.39E-04	8.02E-04
**COL10A1**	2.21	3.39E-04	8.03E-04
**RP11-411A19.5**	-4.28	3.39E-04	8.03E-04
**GEMIN4**	-0.49	3.40E-04	8.04E-04
**TNNI3K**	-4.16	3.40E-04	8.04E-04
**NNT**	-0.35	3.40E-04	8.05E-04
**RNU6-606P**	-4.14	3.40E-04	8.05E-04
**RP11-642D21.1**	-3.97	3.40E-04	8.05E-04
**TRIM68**	0.47	3.40E-04	8.06E-04
**C6orf99**	-1.92	3.41E-04	8.06E-04
**HDDC2**	0.44	3.41E-04	8.07E-04
**GSG2**	-0.98	3.41E-04	8.08E-04
**DCLRE1B**	0.54	3.41E-04	8.08E-04
**ACTG1**	0.67	3.42E-04	8.09E-04
**RP5-916O11.2**	-3.53	3.42E-04	8.09E-04
**RNU7-3P**	-4.19	3.42E-04	8.09E-04
**THBS2**	1.26	3.42E-04	8.09E-04
**PRR3**	0.62	3.42E-04	8.09E-04
**POLE**	-0.53	3.42E-04	8.10E-04
**RP11-383D22.1**	-4.52	3.43E-04	8.10E-04
**PIPSL**	-1.88	3.43E-04	8.11E-04
**CTC-422A18.2**	-4.21	3.44E-04	8.13E-04
**AC083822.2**	-4.27	3.44E-04	8.13E-04
**G10021**	-2.39	3.44E-04	8.13E-04
**RPL17P19**	-4.36	3.44E-04	8.14E-04
**RP11-401N16.2**	-4.13	3.45E-04	8.16E-04
**KLLN**	0.73	3.45E-04	8.16E-04
**RAD17**	-0.37	3.45E-04	8.16E-04
**PDE8B**	-0.88	3.46E-04	8.17E-04
**RP11-91H12.4**	-4.33	3.46E-04	8.17E-04
**SUMO2P16**	-4.44	3.46E-04	8.18E-04
**RP11-179B2.2**	-1.77	3.47E-04	8.19E-04
**FBLL1**	1.90	3.47E-04	8.19E-04
**RP11-62C3.6**	-4.60	3.47E-04	8.21E-04
**G36939**	-3.44	3.48E-04	8.21E-04
**HSD17B1**	-0.65	3.49E-04	8.24E-04

**RPL7AP31**	-2.67	3.49E-04	8.24E-04
**MIR374A**	-4.33	3.49E-04	8.24E-04
**HRH3**	3.86	3.49E-04	8.25E-04
**AVIL**	-0.84	3.49E-04	8.25E-04
**RP3-405J10.5**	-4.29	3.50E-04	8.26E-04
**C6orf183**	-1.76	3.50E-04	8.26E-04
**KCTD9**	-0.38	3.50E-04	8.27E-04
**RP5-1107A17.3**	-4.04	3.52E-04	8.30E-04
**G16357**	1.56	3.52E-04	8.31E-04
**RP11-229P13.2**	-2.63	3.52E-04	8.32E-04
**TMC4**	1.36	3.53E-04	8.33E-04
**G34274**	-2.10	3.53E-04	8.33E-04
**COX7BP2**	-4.43	3.53E-04	8.33E-04
**MYO5A**	-0.52	3.54E-04	8.34E-04
**AC010329.1**	-4.11	3.54E-04	8.35E-04
**RP3-508I15.14**	-4.05	3.54E-04	8.36E-04
**XPA**	-0.34	3.54E-04	8.36E-04
**PPARA**	-0.81	3.55E-04	8.37E-04
**ZMPSTE24**	-0.51	3.55E-04	8.38E-04
**RP1-257I20.14**	-1.64	3.55E-04	8.38E-04
**ATPAF2**	0.45	3.56E-04	8.39E-04
**LRRC37A11P**	-1.47	3.56E-04	8.40E-04
**MIR222**	-4.31	3.56E-04	8.40E-04
**PLCG2**	0.44	3.56E-04	8.40E-04
**PRMT7**	0.51	3.57E-04	8.41E-04
**VN2R10P**	-4.06	3.57E-04	8.42E-04
**RNU1-73P**	-4.01	3.57E-04	8.43E-04
**RNU6-331P**	-4.26	3.58E-04	8.43E-04
**ASPG**	0.96	3.58E-04	8.43E-04
**RNU6-387P**	-2.80	3.59E-04	8.45E-04
**EMCN**	1.26	3.59E-04	8.47E-04
**RP5-1120P11.4**	-4.33	3.60E-04	8.48E-04
**RP11-678G15.1**	-3.50	3.60E-04	8.48E-04
**DDX31**	-0.55	3.60E-04	8.48E-04
**GTF2IP13**	1.34	3.61E-04	8.50E-04
**RP11-255G12.2**	-1.28	3.62E-04	8.52E-04
**RPL34P22**	-3.04	3.62E-04	8.53E-04
**KDM4B**	0.41	3.62E-04	8.53E-04
**AXIN2**	-0.62	3.62E-04	8.53E-04
**SFXN2**	-0.87	3.62E-04	8.53E-04
**MAGOH2P**	-1.36	3.62E-04	8.54E-04
**RP11-399C16.3**	-3.00	3.64E-04	8.56E-04
**RP11-167P22.3**	-4.32	3.64E-04	8.56E-04
**RP11-104O19.4**	-2.51	3.64E-04	8.56E-04
**HELZ2**	0.80	3.64E-04	8.56E-04

**PAK6**	0.62	3.64E-04	8.57E-04
**AC009487.6**	-1.58	3.64E-04	8.57E-04
**HOXD1**	3.39	3.65E-04	8.59E-04
**ENPP7P1**	-4.17	3.65E-04	8.59E-04
**RTL1**	-4.40	3.65E-04	8.59E-04
**RPL5P24**	-4.62	3.65E-04	8.59E-04
**OCLM**	-1.77	3.65E-04	8.60E-04
**GUSBP4**	-1.15	3.66E-04	8.60E-04
**FAXDC2**	-0.98	3.66E-04	8.61E-04
**RP11-74E22.6**	-2.35	3.66E-04	8.61E-04
**AC007377.1**	-4.67	3.66E-04	8.62E-04
**DNLZ**	-2.01	3.66E-04	8.62E-04
**TMEM2**	0.88	3.67E-04	8.63E-04
**KIAA1614**	0.82	3.67E-04	8.64E-04
**TNFAIP2**	1.34	3.68E-04	8.64E-04
**WBSCR17**	2.08	3.68E-04	8.64E-04
**POLI**	-0.60	3.68E-04	8.66E-04
**RP4-714D9.2**	-4.18	3.68E-04	8.66E-04
**KANK1**	-0.52	3.68E-04	8.66E-04
**RP11-109P14.8**	-4.27	3.69E-04	8.67E-04
**MOGAT2**	-3.51	3.69E-04	8.67E-04
**NRIP2**	1.02	3.70E-04	8.69E-04
**RP11-382A20.7**	-1.81	3.70E-04	8.69E-04
**OR7E11P**	-4.49	3.70E-04	8.69E-04
**RP11-383J24.2**	-2.00	3.70E-04	8.69E-04
**KRT8P3**	-4.03	3.70E-04	8.69E-04
**G42644**	-2.31	3.70E-04	8.70E-04
**RP11-5P18.1**	-4.35	3.71E-04	8.71E-04
**CUX1**	0.50	3.71E-04	8.72E-04
**NRF1**	0.45	3.72E-04	8.74E-04
**AC093375.1**	-2.55	3.73E-04	8.76E-04
**AL157392.1**	-4.26	3.73E-04	8.76E-04
**TCF7L1-IT1**	-4.71	3.73E-04	8.77E-04
**CSTA**	0.88	3.73E-04	8.77E-04
**METTL21B**	0.49	3.73E-04	8.77E-04
**RP5-1057J7.7**	-1.42	3.73E-04	8.77E-04
**RP11-664D1.1**	-3.90	3.74E-04	8.77E-04
**RNU6-122P**	-4.11	3.75E-04	8.79E-04
**ASAP1**	0.44	3.75E-04	8.80E-04
**RP11-329J18.4**	-3.97	3.75E-04	8.80E-04
**RP5-1099C19.2**	-2.73	3.75E-04	8.81E-04
**XLOC_005452**	1.92	3.76E-04	8.81E-04
**PARD6G-AS1**	-1.17	3.76E-04	8.83E-04
**XLOC_008043**	-4.48	3.76E-04	8.83E-04
**AKR1C1**	0.80	3.76E-04	8.83E-04

**MIR938**	-4.22	3.76E-04	8.83E-04
**PFKM**	0.47	3.76E-04	8.83E-04
**RP11-843P14.1**	-2.00	3.77E-04	8.85E-04
**AC027612.6**	0.75	3.79E-04	8.89E-04
**MTCO1P19**	-4.40	3.79E-04	8.89E-04
**RP11-29B2.6**	-3.98	3.80E-04	8.90E-04
**KRT40**	-3.23	3.80E-04	8.90E-04
**DDX60L**	-0.81	3.80E-04	8.91E-04
**HOXD10**	1.33	3.80E-04	8.92E-04
**SNORD114-21**	-4.43	3.81E-04	8.94E-04
**NOTUM**	2.32	3.82E-04	8.95E-04
**AL590762.10**	-3.95	3.82E-04	8.95E-04
**CKMT1B**	0.62	3.82E-04	8.95E-04
**PSRC1**	1.04	3.82E-04	8.96E-04
**RPN1**	0.52	3.82E-04	8.96E-04
**IL18BP**	1.02	3.82E-04	8.96E-04
**TMEM169**	-1.08	3.83E-04	8.97E-04
**XLOC_005514**	-4.44	3.84E-04	8.99E-04
**RNY3P1**	-4.49	3.84E-04	8.99E-04
**SNORD37**	-4.34	3.84E-04	9.00E-04
**RN7SL431P**	-3.87	3.84E-04	9.00E-04
**ELF1**	-0.43	3.84E-04	9.00E-04
**PHACTR2-AS1**	-3.50	3.84E-04	9.01E-04
**ADAMDEC1**	4.35	3.85E-04	9.01E-04
**MAP2**	-0.81	3.85E-04	9.01E-04
**RPL37AP1**	-4.17	3.85E-04	9.02E-04
**SNORD111**	-3.89	3.85E-04	9.02E-04
**IQCH**	-0.78	3.86E-04	9.03E-04
**TRPM4**	0.70	3.86E-04	9.03E-04
**BRWD1-IT1**	-3.89	3.88E-04	9.07E-04
**RNU6-223P**	-4.22	3.88E-04	9.09E-04
**CTD-2376I4.2**	1.85	3.88E-04	9.09E-04
**RP4-761J14.8**	-2.14	3.89E-04	9.10E-04
**RAB11FIP2**	-0.56	3.89E-04	9.10E-04
**ADAMTS13**	1.27	3.89E-04	9.10E-04
**RP11-946P6.6**	-1.83	3.89E-04	9.11E-04
**TCF19**	0.44	3.89E-04	9.11E-04
**SLC9A9**	-0.51	3.90E-04	9.12E-04
**ZNF593**	-4.18	3.90E-04	9.12E-04
**RNF135**	0.36	3.90E-04	9.13E-04
**ACKR3**	1.21	3.90E-04	9.13E-04
**SCARNA24**	-4.21	3.91E-04	9.14E-04
**RP11-139F4.2**	-4.25	3.92E-04	9.17E-04
**RP11-759F5.1**	-4.47	3.92E-04	9.17E-04
**AC092295.4**	-4.18	3.92E-04	9.18E-04

**PTENP1**	-1.71	3.92E-04	9.18E-04
**PILRB**	-1.54	3.93E-04	9.20E-04
**RNU6-915P**	-4.09	3.93E-04	9.20E-04
**RP11-1E4.1**	-3.13	3.93E-04	9.20E-04
**SLC22A10**	-4.56	3.94E-04	9.20E-04
**OVOL1-AS1**	-0.99	3.94E-04	9.21E-04
**NAP1L2**	-1.08	3.94E-04	9.21E-04
**SSBP1**	0.47	3.95E-04	9.23E-04
**RP11-454F8.4**	-1.89	3.97E-04	9.28E-04
**RP11-370K11.1**	-4.26	3.98E-04	9.29E-04
**GNPNAT1**	-0.51	3.98E-04	9.29E-04
**CFL1P1**	1.46	3.98E-04	9.31E-04
**RP11-10J21.2**	-2.90	3.99E-04	9.33E-04
**AQP11**	-1.33	3.99E-04	9.33E-04
**RP3-388E23.2**	-1.95	4.00E-04	9.34E-04
**SLITRK5**	-2.51	4.00E-04	9.34E-04
**B3GNT7**	1.13	4.00E-04	9.34E-04
**G26207**	-3.33	4.00E-04	9.35E-04
**GHR**	-0.55	4.01E-04	9.37E-04
**CATSPER2**	-1.39	4.02E-04	9.38E-04
**RP11-630D6.5**	-4.21	4.02E-04	9.38E-04
**RP11-58K22.5**	-4.04	4.02E-04	9.40E-04
**CTD-3105H18.7**	-4.12	4.03E-04	9.40E-04
**RN7SL15P**	-2.46	4.03E-04	9.40E-04
**RP5-837J1.4**	-4.25	4.03E-04	9.41E-04
**RPS6KB1**	-0.45	4.03E-04	9.41E-04
**RP11-163E9.1**	-2.18	4.03E-04	9.41E-04
**IFI27L1**	0.76	4.04E-04	9.43E-04
**TIMM21**	0.38	4.04E-04	9.44E-04
**CTD-2609K8.3**	-3.87	4.04E-04	9.44E-04
**URI1**	-0.43	4.04E-04	9.44E-04
**LGI1**	-2.40	4.05E-04	9.45E-04
**KCNA3**	-2.35	4.05E-04	9.45E-04
**PSMD8**	0.48	4.05E-04	9.45E-04
**VPS9D1-AS1**	1.85	4.05E-04	9.46E-04
**RP4-647C14.3**	-4.22	4.05E-04	9.46E-04
**PCDHB10**	0.80	4.07E-04	9.48E-04
**HNRNPA3P11**	-4.24	4.07E-04	9.50E-04
**SYP**	0.75	4.08E-04	9.51E-04
**RP11-460N20.7**	-2.49	4.08E-04	9.51E-04
**RP11-235E17.4**	-3.98	4.08E-04	9.52E-04
**RP11-93H24.3**	-1.67	4.08E-04	9.52E-04
**RPL4P3**	-4.39	4.09E-04	9.53E-04
**MTCO1P15**	-4.12	4.10E-04	9.55E-04
**AFG3L2**	-0.40	4.10E-04	9.55E-04

**C8orf46**	-1.61	4.10E-04	9.55E-04
**RP11-1029M24.4**	-4.19	4.11E-04	9.58E-04
**G10661**	3.38	4.12E-04	9.59E-04
**NEFH**	1.57	4.12E-04	9.59E-04
**RAX2**	-4.18	4.12E-04	9.60E-04
**GRM8**	3.52	4.12E-04	9.60E-04
**RNU6-69P**	-4.74	4.12E-04	9.61E-04
**RNU6-862P**	-4.22	4.13E-04	9.62E-04
**AL355531.2**	-4.36	4.13E-04	9.63E-04
**GABBR2**	-2.99	4.16E-04	9.68E-04
**GBGT1**	1.46	4.16E-04	9.69E-04
**IL1A**	-1.43	4.16E-04	9.69E-04
**RP11-285J16.1**	-2.07	4.16E-04	9.69E-04
**LA16c-60H5.7**	5.42	4.17E-04	9.70E-04
**CYP3A5**	-1.38	4.17E-04	9.70E-04
**FAM13B**	-0.39	4.17E-04	9.71E-04
**RPL6P2**	-4.53	4.17E-04	9.71E-04
**RP11-46I1.2**	-4.38	4.17E-04	9.71E-04
**XXbac-B476C20.14**	-4.12	4.17E-04	9.72E-04
**AC073410.1**	-1.90	4.18E-04	9.72E-04
**OTOP1**	2.07	4.18E-04	9.72E-04
**RP11-543N12.1**	-3.97	4.18E-04	9.73E-04
**KB-1958F4.1**	1.77	4.18E-04	9.73E-04
**AC026904.1**	2.26	4.18E-04	9.73E-04
**ATF7**	0.59	4.18E-04	9.74E-04
**AP000662.4**	-1.57	4.19E-04	9.76E-04
**STK32B**	1.41	4.19E-04	9.76E-04
**RP11-522L3.11**	-4.31	4.19E-04	9.76E-04
**G3148**	-2.69	4.20E-04	9.76E-04
**HNRNPA1P15**	-4.66	4.20E-04	9.77E-04
**RNU4-62P**	5.07	4.20E-04	9.78E-04
**PTPRZ1**	-0.90	4.21E-04	9.78E-04
**AC016738.3**	-4.02	4.21E-04	9.78E-04
**RP11-1197K16.2**	-4.38	4.21E-04	9.78E-04
**WNT6**	1.22	4.21E-04	9.80E-04
**SHPK**	0.62	4.22E-04	9.81E-04
**HYI**	0.88	4.22E-04	9.81E-04
**RP11-574F21.2**	-2.35	4.23E-04	9.82E-04
**ATP6V0E1P1**	-3.02	4.23E-04	9.83E-04
**KCTD18**	0.38	4.23E-04	9.83E-04
**TFPI2**	2.47	4.23E-04	9.84E-04
**RP11-325F22.2**	-2.23	4.23E-04	9.84E-04
**SLC44A3**	0.79	4.24E-04	9.85E-04
**RP11-597M17.1**	-4.66	4.24E-04	9.85E-04
**RP11-286E11.2**	-2.55	4.25E-04	9.87E-04

**AP001596.6**	1.99	4.25E-04	9.87E-04
**RP1-122P22.4**	2.67	4.25E-04	9.88E-04
**YPEL5**	0.50	4.25E-04	9.88E-04
**XLOC_007934**	-4.33	4.26E-04	9.89E-04
**MIR320E**	2.10	4.26E-04	9.89E-04
**RNU6-588P**	-4.45	4.26E-04	9.90E-04
**TMEM42**	0.60	4.26E-04	9.90E-04
**RP11-692D12.1**	-1.20	4.26E-04	9.90E-04
**RP11-21A7A.4**	-4.05	4.26E-04	9.90E-04
**SLC6A7**	3.82	4.26E-04	9.90E-04
**RP5-1142J19.1**	1.41	4.27E-04	9.90E-04
**CTD-2154B17.2**	-4.04	4.27E-04	9.91E-04
**TDRD1**	-3.19	4.27E-04	9.91E-04
**FDX1P1**	-4.04	4.27E-04	9.92E-04
**SUPT3H**	0.71	4.28E-04	9.93E-04
**TWIST2**	1.24	4.28E-04	9.94E-04
**SLC22A1**	-1.58	4.28E-04	9.94E-04
**HADHA**	0.38	4.30E-04	9.97E-04
**HID1-AS1**	3.18	4.30E-04	9.98E-04
**RP11-58O9.2**	1.38	4.31E-04	9.99E-04
**LANCL1**	-0.59	4.31E-04	9.99E-04
**RP11-21C4.1**	4.07	4.31E-04	1.00E-03
**AC114296.1**	-4.65	4.31E-04	1.00E-03
**ARHGAP6**	1.21	4.32E-04	1.00E-03
**ST6GAL1**	0.65	4.32E-04	1.00E-03
**RP11-128N14.5**	-2.83	4.32E-04	1.00E-03
**G32185**	3.58	4.32E-04	1.00E-03
**GALT**	-0.89	4.33E-04	1.00E-03
**HOXB4**	1.66	4.33E-04	1.00E-03
**RP11-47L3.1**	2.06	4.34E-04	1.01E-03
**UQCRB**	0.51	4.35E-04	1.01E-03
**ASGR1**	0.86	4.36E-04	1.01E-03
**NPRL2**	0.53	4.36E-04	1.01E-03
**RNU6-375P**	-4.34	4.36E-04	1.01E-03
**FLJ22447**	1.22	4.36E-04	1.01E-03
**RP1-79C4.4**	2.34	4.37E-04	1.01E-03
**RP11-720L3.1**	-4.10	4.37E-04	1.01E-03
**RP11-384L8.2**	-4.22	4.38E-04	1.01E-03
**PALM**	1.00	4.38E-04	1.02E-03
**MS4A14**	2.32	4.38E-04	1.02E-03
**APOO**	0.68	4.39E-04	1.02E-03
**RN7SL182P**	-4.05	4.40E-04	1.02E-03
**RNU6-128P**	-4.41	4.41E-04	1.02E-03
**RP11-164P12.3**	-1.52	4.42E-04	1.02E-03
**TK2**	0.51	4.42E-04	1.02E-03

**CGRRF1**	0.59	4.43E-04	1.03E-03
**RP11-686G8.5**	-4.23	4.43E-04	1.03E-03
**ASTN2-AS1**	-4.44	4.44E-04	1.03E-03
**PHACTR2**	-0.65	4.44E-04	1.03E-03
**BTBD8**	-1.25	4.45E-04	1.03E-03
**AC005082.12**	-1.39	4.45E-04	1.03E-03
**MT-TL1**	-4.60	4.45E-04	1.03E-03
**ITGA11**	1.67	4.46E-04	1.03E-03
**ADPGK**	0.50	4.46E-04	1.03E-03
**UGT1A3**	-4.01	4.46E-04	1.03E-03
**LIMK2**	0.44	4.46E-04	1.03E-03
**CTB-187L3.1**	-3.80	4.46E-04	1.03E-03
**FAM192A**	0.26	4.46E-04	1.03E-03
**RP11-445L13__B.3**	-4.16	4.47E-04	1.03E-03
**POLD1**	0.55	4.48E-04	1.04E-03
**G24802**	-3.81	4.48E-04	1.04E-03
**CTB-13F3.1**	1.81	4.48E-04	1.04E-03
**KDM8**	0.80	4.48E-04	1.04E-03
**CA5B**	-0.62	4.48E-04	1.04E-03
**KMT2E**	-0.46	4.49E-04	1.04E-03
**AF213884.3**	-4.15	4.49E-04	1.04E-03
**RNU5A-6P**	-4.44	4.49E-04	1.04E-03
**LINC00982**	2.74	4.49E-04	1.04E-03
**RP4-673M15.1**	1.35	4.50E-04	1.04E-03
**RN7SL36P**	-4.10	4.50E-04	1.04E-03
**AC007551.1**	-4.03	4.50E-04	1.04E-03
**LMBRD1**	-0.52	4.51E-04	1.04E-03
**TSSC2**	1.59	4.51E-04	1.04E-03
**CENPJ**	-0.55	4.52E-04	1.04E-03
**ZNF318**	-0.43	4.55E-04	1.05E-03
**RP11-151N17.1**	-1.47	4.55E-04	1.05E-03
**RP4-669P10.16**	-4.07	4.56E-04	1.05E-03
**KB-1732A1.1**	1.06	4.56E-04	1.05E-03
**AC079834.2**	-4.30	4.56E-04	1.05E-03
**AK4**	-0.61	4.57E-04	1.06E-03
**MEX3B**	1.42	4.58E-04	1.06E-03
**DIO2**	1.81	4.58E-04	1.06E-03
**C9orf116**	1.74	4.59E-04	1.06E-03
**RP11-89N17.1**	-3.36	4.61E-04	1.06E-03
**FEZ1**	1.02	4.61E-04	1.06E-03
**MTND1P32**	-3.95	4.61E-04	1.07E-03
**RP11-59H7.1**	-4.08	4.62E-04	1.07E-03
**XLOC_005079**	2.81	4.62E-04	1.07E-03
**AC003682.16**	-3.95	4.63E-04	1.07E-03
**MEIS3P1**	1.60	4.63E-04	1.07E-03

**CTC-543D15.8**	2.23	4.63E-04	1.07E-03
**AC007365.4**	-4.08	4.64E-04	1.07E-03
**RP11-1017G21.3**	-4.16	4.64E-04	1.07E-03
**RP3-354N19.3**	-3.48	4.64E-04	1.07E-03
**FAM107B**	-0.86	4.64E-04	1.07E-03
**RP11-138H8.8**	-2.14	4.65E-04	1.07E-03
**GCNT4**	-0.73	4.65E-04	1.07E-03
**AGER**	1.12	4.66E-04	1.07E-03
**MKKS**	0.39	4.66E-04	1.07E-03
**NPEPPS**	0.45	4.67E-04	1.08E-03
**FXN**	0.69	4.67E-04	1.08E-03
**XLOC_011550**	-4.45	4.68E-04	1.08E-03
**CTD-2576D5.4**	-4.14	4.68E-04	1.08E-03
**MIR143**	3.44	4.68E-04	1.08E-03
**CTH**	0.95	4.68E-04	1.08E-03
**RN7SL650P**	-2.23	4.68E-04	1.08E-03
**POLR2I**	-0.65	4.69E-04	1.08E-03
**PDCL2**	-3.79	4.69E-04	1.08E-03
**G35258**	-3.33	4.70E-04	1.08E-03
**RALGPS1**	-0.78	4.70E-04	1.08E-03
**TMEM191B**	2.70	4.70E-04	1.08E-03
**HMGCR**	-0.81	4.71E-04	1.09E-03
**AC007292.3**	-1.59	4.71E-04	1.09E-03
**CHGA**	-2.19	4.73E-04	1.09E-03
**IKZF3**	-1.02	4.73E-04	1.09E-03
**RP11-48G14.1**	-4.11	4.73E-04	1.09E-03
**G18243**	2.87	4.74E-04	1.09E-03
**NKX6-2**	3.04	4.74E-04	1.09E-03
**CYB5RL**	-0.82	4.74E-04	1.09E-03
**UPF3AP2**	1.98	4.74E-04	1.09E-03
**GS1-124K5.12**	0.64	4.74E-04	1.09E-03
**SCFD2**	-0.48	4.75E-04	1.09E-03
**CTD-2083E4.4**	-1.48	4.76E-04	1.10E-03
**ANP32AP1**	-3.92	4.77E-04	1.10E-03
**G26757**	-1.44	4.77E-04	1.10E-03
**LTA4H**	-0.49	4.77E-04	1.10E-03
**GPR107**	0.37	4.77E-04	1.10E-03
**RP11-693N9.2**	1.55	4.79E-04	1.10E-03
**AC005841.1**	-1.89	4.79E-04	1.10E-03
**MIR597**	-4.45	4.79E-04	1.10E-03
**AC009120.11**	-3.04	4.80E-04	1.10E-03
**AADACL2-AS1**	-1.66	4.82E-04	1.11E-03
**VDAC1P11**	-4.49	4.83E-04	1.11E-03
**ACSM5**	1.50	4.83E-04	1.11E-03
**XLOC_001023**	-0.78	4.83E-04	1.11E-03

**MAOA**	-0.84	4.83E-04	1.11E-03
**CTD-2256P15.2**	0.94	4.83E-04	1.11E-03
**G25515**	-4.05	4.83E-04	1.11E-03
**RPS10P3**	-1.51	4.84E-04	1.11E-03
**GOT1**	0.63	4.85E-04	1.12E-03
**RP11-416L21.2**	4.59	4.85E-04	1.12E-03
**RP11-384F7.2**	-4.20	4.86E-04	1.12E-03
**VN1R107P**	-3.58	4.86E-04	1.12E-03
**RP11-799D4.4**	0.87	4.86E-04	1.12E-03
**ITGAL**	1.21	4.87E-04	1.12E-03
**AC004053.1**	-4.09	4.87E-04	1.12E-03
**RP11-119H12.4**	-4.24	4.87E-04	1.12E-03
**CEBPZ**	-0.56	4.87E-04	1.12E-03
**USP17L1**	-4.12	4.88E-04	1.12E-03
**LINC01623**	-4.11	4.88E-04	1.12E-03
**RP11-755F10.1**	1.61	4.88E-04	1.12E-03
**TPRN**	0.53	4.89E-04	1.12E-03
**LINC01119**	2.42	4.89E-04	1.12E-03
**FARSA-AS1**	-3.26	4.90E-04	1.12E-03
**SLC9A9-AS2**	-4.08	4.90E-04	1.13E-03
**ZNF697**	0.85	4.90E-04	1.13E-03
**CACNA1S**	-3.17	4.91E-04	1.13E-03
**PTPRQ**	-3.79	4.91E-04	1.13E-03
**HMGB3**	0.55	4.92E-04	1.13E-03
**GGH**	0.96	4.92E-04	1.13E-03
**CTD-2201G3.1**	-1.91	4.92E-04	1.13E-03
**PLCL1**	0.93	4.93E-04	1.13E-03
**HIF1AP1**	-4.21	4.93E-04	1.13E-03
**XLOC_005110**	-1.84	4.93E-04	1.13E-03
**PDLIM1P4**	-1.89	4.94E-04	1.13E-03
**PPM1N**	1.04	4.94E-04	1.13E-03
**RP11-88H12.2**	-3.32	4.94E-04	1.13E-03
**RNU6-1266P**	-2.87	4.94E-04	1.13E-03
**UBL5P2**	-3.72	4.95E-04	1.14E-03
**ADAMTS7P4**	2.88	4.95E-04	1.14E-03
**RNU5E-2P**	-4.44	4.95E-04	1.14E-03
**AP002954.3**	-1.87	4.96E-04	1.14E-03
**MIR2467**	-4.25	4.98E-04	1.14E-03
**AC092782.1**	-4.28	4.98E-04	1.14E-03
**DLL1**	0.80	4.98E-04	1.14E-03
**MIR591**	-4.20	4.98E-04	1.14E-03
**GLRX**	1.22	4.98E-04	1.14E-03
**RP11-72I8.1**	-2.61	4.98E-04	1.14E-03
**ZNF487**	-0.64	4.99E-04	1.14E-03
**LRRC55**	1.60	4.99E-04	1.14E-03

**AC090571.1**	-3.98	4.99E-04	1.14E-03
**AC005154.8**	-2.97	5.00E-04	1.15E-03
**RUFY1**	0.33	5.00E-04	1.15E-03
**RP11-83B20.6**	-4.24	5.01E-04	1.15E-03
**RP11-507J18.5**	-3.79	5.01E-04	1.15E-03
**AC007563.5**	-3.31	5.01E-04	1.15E-03
**RP11-809C18.3**	-2.07	5.03E-04	1.15E-03
**MIR563**	-4.13	5.03E-04	1.15E-03
**HIST1H2AM**	-4.47	5.03E-04	1.15E-03
**AP001258.5**	-4.31	5.04E-04	1.15E-03
**LMOD3**	-1.89	5.04E-04	1.15E-03
**CTB-105N12.2**	-2.98	5.04E-04	1.16E-03
**RP3-388N13.5**	-4.00	5.05E-04	1.16E-03
**RP4-751H13.7**	-3.12	5.06E-04	1.16E-03
**RP11-432B6.1**	-4.06	5.06E-04	1.16E-03
**TNFRSF8**	1.43	5.06E-04	1.16E-03
**ZNF85**	-0.52	5.07E-04	1.16E-03
**MSL3**	0.44	5.07E-04	1.16E-03
**RIPK3**	0.72	5.07E-04	1.16E-03
**CTB-131B5.2**	-3.52	5.08E-04	1.16E-03
**RNU7-101P**	-4.48	5.08E-04	1.16E-03
**CACNA2D4**	1.05	5.09E-04	1.17E-03
**RP11-196B3.1**	-3.91	5.09E-04	1.17E-03
**CDCA2**	-0.79	5.10E-04	1.17E-03
**ANKRD16**	0.47	5.11E-04	1.17E-03
**CCDC172**	-4.94	5.11E-04	1.17E-03
**TH2LCRR**	-2.33	5.11E-04	1.17E-03
**NPIPB6**	-2.46	5.12E-04	1.17E-03
**RN7SL113P**	-2.51	5.13E-04	1.17E-03
**RP11-5L12.1**	-4.38	5.13E-04	1.17E-03
**G7721**	-4.92	5.13E-04	1.17E-03
**ZNF70**	-0.52	5.13E-04	1.17E-03
**NUTF2P8**	-4.21	5.13E-04	1.17E-03
**RP11-659P15.2**	-4.24	5.14E-04	1.18E-03
**PPFIA4**	1.37	5.14E-04	1.18E-03
**MEG3**	1.93	5.14E-04	1.18E-03
**RP11-58A11.2**	-1.79	5.14E-04	1.18E-03
**PRSS51**	-2.05	5.14E-04	1.18E-03
**MIR3684**	-4.16	5.15E-04	1.18E-03
**SPATA5L1**	0.44	5.15E-04	1.18E-03
**G34278**	-4.10	5.15E-04	1.18E-03
**C6orf10**	-3.46	5.16E-04	1.18E-03
**TBCC**	0.51	5.16E-04	1.18E-03
**KRT28**	-7.55	5.16E-04	1.18E-03
**RP11-427L15.2**	-1.62	5.17E-04	1.18E-03

**RP1-267L14.3**	-3.89	5.17E-04	1.18E-03
**PEBP1P2**	-3.98	5.17E-04	1.18E-03
**RP11-894J14.2**	-4.09	5.18E-04	1.18E-03
**HDLBP**	0.39	5.18E-04	1.18E-03
**RP11-18M17.1**	-2.32	5.18E-04	1.18E-03
**TCEAL3-AS1**	-3.81	5.18E-04	1.18E-03
**RPS27P16**	-2.69	5.20E-04	1.19E-03
**DNAJC11**	0.34	5.20E-04	1.19E-03
**SLC35E1**	0.32	5.20E-04	1.19E-03
**EFCAB6-AS1**	-4.36	5.20E-04	1.19E-03
**NPW**	1.77	5.21E-04	1.19E-03
**HYAL3**	1.43	5.21E-04	1.19E-03
**SRSF12**	-0.96	5.22E-04	1.19E-03
**SETP5**	-4.07	5.22E-04	1.19E-03
**EMC8**	0.48	5.22E-04	1.19E-03
**RP11-235E17.6**	-1.92	5.23E-04	1.19E-03
**ZFP92**	1.60	5.23E-04	1.19E-03
**NFIC**	0.91	5.24E-04	1.20E-03
**GS1-259H13.7**	-2.47	5.25E-04	1.20E-03
**CACNB4**	-1.40	5.25E-04	1.20E-03
**RP11-367H5.8**	-4.20	5.25E-04	1.20E-03
**RP5-1198O20.4**	1.38	5.25E-04	1.20E-03
**RPL23AP17**	-4.43	5.26E-04	1.20E-03
**RP11-648O15.2**	-4.33	5.26E-04	1.20E-03
**CYP3A4**	-1.75	5.26E-04	1.20E-03
**RP11-131L12.4**	-2.04	5.26E-04	1.20E-03
**MOB4**	-0.64	5.27E-04	1.20E-03
**C5orf22**	-0.46	5.27E-04	1.20E-03
**RP11-83M16.5**	-3.69	5.27E-04	1.20E-03
**KB-1562D12.2**	-4.07	5.28E-04	1.20E-03
**CCL17**	-2.18	5.28E-04	1.20E-03
**MRPS21P9**	-3.89	5.29E-04	1.21E-03
**AP000473.6**	-4.31	5.29E-04	1.21E-03
**XLOC_011789**	-4.61	5.29E-04	1.21E-03
**IL20RA**	-0.78	5.30E-04	1.21E-03
**RP11-519G16.3**	-2.48	5.30E-04	1.21E-03
**S100A6**	1.02	5.30E-04	1.21E-03
**POLR3E**	-0.51	5.30E-04	1.21E-03
**RP11-415F23.4**	-3.45	5.31E-04	1.21E-03
**AC055764.1**	-3.99	5.31E-04	1.21E-03
**CDK2**	-0.37	5.32E-04	1.21E-03
**ANKRD30BP3**	-2.77	5.32E-04	1.21E-03
**PPT1**	0.57	5.32E-04	1.21E-03
**UNKL**	0.67	5.33E-04	1.21E-03
**ZNF565**	-0.54	5.33E-04	1.21E-03

**RP11-219B4.6**	-4.19	5.33E-04	1.21E-03
**AC019181.2**	-2.31	5.33E-04	1.21E-03
**RBM33**	-0.54	5.34E-04	1.22E-03
**MTCO2P2**	-4.33	5.34E-04	1.22E-03
**AL050316.1**	-4.19	5.35E-04	1.22E-03
**RP11-676J12.4**	-2.94	5.35E-04	1.22E-03
**COA6**	0.64	5.36E-04	1.22E-03
**AC016907.3**	-4.15	5.36E-04	1.22E-03
**ACTG1P3**	-2.23	5.36E-04	1.22E-03
**RP11-567P19.1**	-3.83	5.36E-04	1.22E-03
**STX11**	0.76	5.36E-04	1.22E-03
**TMEM40**	0.58	5.36E-04	1.22E-03
**RP11-110H1.4**	-3.39	5.37E-04	1.22E-03
**CTC-429P9.2**	-0.67	5.37E-04	1.22E-03
**OR2T10**	-4.99	5.37E-04	1.22E-03
**ATG16L1**	-0.43	5.37E-04	1.22E-03
**EDAR**	1.67	5.38E-04	1.22E-03
**FMO5**	-0.99	5.38E-04	1.22E-03
**TGFBR2**	0.68	5.38E-04	1.22E-03
**TRUB2**	0.42	5.38E-04	1.22E-03
**MID2**	-0.64	5.38E-04	1.23E-03
**RPL7P32**	-3.28	5.39E-04	1.23E-03
**DDX11L8**	-4.28	5.39E-04	1.23E-03
**TOMM40L**	0.51	5.40E-04	1.23E-03
**VPS33B**	0.42	5.40E-04	1.23E-03
**CTDSP2**	0.35	5.40E-04	1.23E-03
**PRRX2-AS1**	-4.32	5.40E-04	1.23E-03
**CEP120**	-0.43	5.41E-04	1.23E-03
**RP11-267J23.1**	-4.22	5.41E-04	1.23E-03
**SDR39U1**	1.00	5.41E-04	1.23E-03
**C10orf71-AS1**	-4.53	5.41E-04	1.23E-03
**RP11-10A14.9**	-4.22	5.42E-04	1.23E-03
**RPL24P8**	-1.78	5.43E-04	1.23E-03
**POLR3DP1**	-2.52	5.44E-04	1.24E-03
**RP11-93O14.3**	-2.86	5.44E-04	1.24E-03
**SDCBPP1**	-4.49	5.44E-04	1.24E-03
**SNORD56**	-2.77	5.44E-04	1.24E-03
**MAGEA11**	-4.70	5.45E-04	1.24E-03
**G26051**	-2.53	5.45E-04	1.24E-03
**TATDN2P1**	-4.15	5.45E-04	1.24E-03
**GDPD1**	-0.78	5.45E-04	1.24E-03
**DDC**	-3.79	5.45E-04	1.24E-03
**RP13-923O23.6**	-4.03	5.45E-04	1.24E-03
**HMGN1P10**	-4.41	5.46E-04	1.24E-03
**AC139100.4**	-2.26	5.46E-04	1.24E-03

**RP11-551L14.7**	-4.11	5.46E-04	1.24E-03
**RBM20**	1.06	5.46E-04	1.24E-03
**LINC01372**	-1.80	5.47E-04	1.24E-03
**NTM-AS1**	-3.80	5.47E-04	1.24E-03
**OR1K1**	-4.39	5.48E-04	1.24E-03
**RP13-467H17.1**	-2.09	5.48E-04	1.24E-03
**CTA-315H11.2**	-4.21	5.48E-04	1.24E-03
**PRH1**	-2.81	5.49E-04	1.25E-03
**CES5A**	-2.03	5.49E-04	1.25E-03
**PCSK4**	2.03	5.49E-04	1.25E-03
**TRIM67**	-1.89	5.50E-04	1.25E-03
**ITPKB-IT1**	-3.90	5.50E-04	1.25E-03
**ZNF843**	0.76	5.50E-04	1.25E-03
**CTC-806A22.1**	-4.40	5.50E-04	1.25E-03
**RN7SL75P**	-3.93	5.50E-04	1.25E-03
**DHX9**	-0.39	5.50E-04	1.25E-03
**DHX16**	0.35	5.50E-04	1.25E-03
**AC007050.17**	-3.49	5.52E-04	1.25E-03
**HMGB3P4**	-2.17	5.54E-04	1.26E-03
**RNY4P4**	-4.09	5.55E-04	1.26E-03
**AC004870.5**	4.43	5.55E-04	1.26E-03
**ZNF394**	-0.38	5.55E-04	1.26E-03
**GMFB**	-0.56	5.55E-04	1.26E-03
**CTD-2046J7.1**	-4.47	5.55E-04	1.26E-03
**AL049758.2**	-4.53	5.56E-04	1.26E-03
**HIATL1**	-0.40	5.56E-04	1.26E-03
**ZDHHC17**	-0.47	5.57E-04	1.26E-03
**PDE4C**	2.14	5.57E-04	1.26E-03
**TNRC18P1**	-2.26	5.58E-04	1.27E-03
**REEP5**	-0.39	5.58E-04	1.27E-03
**VENTXP6**	-4.39	5.59E-04	1.27E-03
**RNU6-789P**	-4.20	5.59E-04	1.27E-03
**PGM2L1**	1.00	5.59E-04	1.27E-03
**RP11-108M9.3**	-2.89	5.60E-04	1.27E-03
**TOB1**	0.62	5.60E-04	1.27E-03
**AL358815.1**	-4.08	5.60E-04	1.27E-03
**RP11-592P9.3**	-4.18	5.60E-04	1.27E-03
**AC003080.4**	-4.38	5.60E-04	1.27E-03
**FGFR4**	1.50	5.61E-04	1.27E-03
**YWHAZP4**	-4.13	5.61E-04	1.27E-03
**MYB-AS1**	-4.57	5.62E-04	1.27E-03
**DIRC1**	5.01	5.62E-04	1.27E-03
**EEF1A1P5**	-0.79	5.63E-04	1.27E-03
**MIR590**	-1.77	5.64E-04	1.28E-03
**AC004449.6**	-2.51	5.64E-04	1.28E-03

**RP11-674I16.1**	-4.02	5.64E-04	1.28E-03
**HPD**	2.59	5.64E-04	1.28E-03
**CCZ1**	-0.77	5.64E-04	1.28E-03
**RHEBP3**	-4.26	5.64E-04	1.28E-03
**HNRNPA1P71**	-4.36	5.64E-04	1.28E-03
**WNT16**	-1.07	5.65E-04	1.28E-03
**TPST1**	1.01	5.66E-04	1.28E-03
**RNU4-54P**	-4.03	5.66E-04	1.28E-03
**AP000473.5**	-1.58	5.67E-04	1.28E-03
**FAM188B**	-1.37	5.67E-04	1.28E-03
**AC004054.1**	-4.35	5.67E-04	1.28E-03
**L3MBTL4-AS1**	-1.90	5.68E-04	1.28E-03
**RPS3AP6**	-0.72	5.68E-04	1.29E-03
**RP11-141C7.4**	-4.33	5.69E-04	1.29E-03
**CYCSP40**	-4.17	5.71E-04	1.29E-03
**GARS**	0.53	5.71E-04	1.29E-03
**RPL34P21**	-4.10	5.71E-04	1.29E-03
**RP11-349G13.3**	-3.39	5.72E-04	1.29E-03
**BOLA3-AS1**	0.96	5.72E-04	1.29E-03
**PDLIM5**	-0.45	5.72E-04	1.29E-03
**EYA4**	3.58	5.73E-04	1.29E-03
**G5664**	-4.40	5.73E-04	1.30E-03
**SUB1P1**	-1.95	5.74E-04	1.30E-03
**AC009495.4**	-3.48	5.75E-04	1.30E-03
**PSMB5**	0.56	5.75E-04	1.30E-03
**RN7SKP23**	-1.35	5.75E-04	1.30E-03
**EEF2KMT**	0.68	5.75E-04	1.30E-03
**AC005392.13**	-4.23	5.76E-04	1.30E-03
**C7orf57**	2.31	5.76E-04	1.30E-03
**MIATNB**	-0.90	5.77E-04	1.30E-03
**RP11-328C8.4**	-4.51	5.77E-04	1.30E-03
**AC009303.3**	-3.93	5.78E-04	1.30E-03
**RP11-561O23.8**	-2.31	5.78E-04	1.31E-03
**XLOC_005510**	2.53	5.78E-04	1.31E-03
**CTC-244M17.1**	-3.88	5.79E-04	1.31E-03
**SRL**	1.22	5.79E-04	1.31E-03
**TRBC2**	1.35	5.80E-04	1.31E-03
**XLOC_005515**	-4.48	5.80E-04	1.31E-03
**AC091729.9**	0.87	5.80E-04	1.31E-03
**XLOC_014082**	-0.84	5.81E-04	1.31E-03
**C4orf45**	-3.86	5.82E-04	1.31E-03
**SOAT1**	-2.36	5.82E-04	1.31E-03
**G26709**	-3.94	5.82E-04	1.31E-03
**CD244**	1.89	5.82E-04	1.31E-03
**LINC01420**	0.48	5.82E-04	1.31E-03

**XLOC_000839**	-3.92	5.83E-04	1.32E-03
**HAS2**	-1.12	5.84E-04	1.32E-03
**ARNTL2**	-0.70	5.84E-04	1.32E-03
**RP11-1277A3.3**	1.90	5.86E-04	1.32E-03
**RP11-197M22.2**	-2.01	5.86E-04	1.32E-03
**RP11-459O16.1**	-4.72	5.87E-04	1.32E-03
**RP11-483H20.4**	-2.27	5.87E-04	1.32E-03
**NTPCR**	0.42	5.87E-04	1.32E-03
**BBS1**	-1.37	5.87E-04	1.32E-03
**CTD-2363C16.2**	-3.30	5.88E-04	1.33E-03
**TEX29**	2.07	5.90E-04	1.33E-03
**RP11-344F13.1**	-4.23	5.91E-04	1.33E-03
**SLC43A3**	0.79	5.91E-04	1.33E-03
**LINC00176**	1.13	5.91E-04	1.33E-03
**RP11-48B3.3**	-1.73	5.91E-04	1.33E-03
**LAMTOR3P2**	-1.97	5.91E-04	1.33E-03
**RP11-6B19.1**	-2.79	5.91E-04	1.33E-03
**RP5-1011O1.2**	-4.18	5.92E-04	1.33E-03
**LINC01552**	-3.77	5.92E-04	1.33E-03
**CD274**	1.13	5.92E-04	1.33E-03
**TMEM189**	-0.63	5.93E-04	1.34E-03
**KRT74**	-4.69	5.93E-04	1.34E-03
**RFC2**	0.53	5.93E-04	1.34E-03
**LLGL2**	0.67	5.93E-04	1.34E-03
**RPS16P5**	-4.09	5.93E-04	1.34E-03
**EEF1A1P6**	-0.84	5.94E-04	1.34E-03
**DHX29**	-0.49	5.94E-04	1.34E-03
**MIR210**	1.99	5.94E-04	1.34E-03
**AC013448.2**	-4.28	5.94E-04	1.34E-03
**G555**	-2.63	5.94E-04	1.34E-03
**CDCP1**	-0.46	5.95E-04	1.34E-03
**RP11-96H17.3**	-2.02	5.95E-04	1.34E-03
**CMTR1**	0.33	5.95E-04	1.34E-03
**RP11-996F15.2**	-1.22	5.96E-04	1.34E-03
**WHAMMP2**	1.02	5.96E-04	1.34E-03
**RNF144A-AS1**	1.74	5.97E-04	1.34E-03
**FBXW12**	-2.00	5.97E-04	1.34E-03
**XLOC_007865**	2.24	5.97E-04	1.34E-03
**MIR342**	-4.18	5.98E-04	1.35E-03
**ERCC1**	0.42	5.99E-04	1.35E-03
**METTL5**	0.47	5.99E-04	1.35E-03
**CDH10**	-3.37	6.01E-04	1.35E-03
**RPL7P16**	2.43	6.01E-04	1.35E-03
**PTPRN**	3.33	6.02E-04	1.35E-03
**RNU6-773P**	-4.30	6.02E-04	1.35E-03

**SEMA4A**	0.58	6.02E-04	1.35E-03
**CH507-42P11.7**	-1.00	6.02E-04	1.35E-03
**RP11-502N13.2**	-2.70	6.02E-04	1.35E-03
**RP11-474C8.8**	-2.39	6.04E-04	1.36E-03
**TAF7L**	-1.15	6.04E-04	1.36E-03
**ARHGAP11B**	-0.75	6.05E-04	1.36E-03
**XLOC_007925**	-1.32	6.05E-04	1.36E-03
**RNA5SP493**	-4.38	6.06E-04	1.36E-03
**LINC01614**	6.68	6.07E-04	1.36E-03
**HSPE1P25**	-4.00	6.07E-04	1.36E-03
**RP11-667M19.2**	-3.24	6.08E-04	1.37E-03
**RNU6-1289P**	-4.31	6.08E-04	1.37E-03
**C10orf131**	-2.24	6.08E-04	1.37E-03
**KCNJ4**	3.92	6.08E-04	1.37E-03
**RNFT2**	-1.24	6.09E-04	1.37E-03
**RAB3D**	-0.65	6.09E-04	1.37E-03
**POM121**	0.63	6.09E-04	1.37E-03
**FTH1P4**	-4.12	6.10E-04	1.37E-03
**RPL23AP58**	-2.72	6.10E-04	1.37E-03
**STX19**	-0.74	6.10E-04	1.37E-03
**DUTP6**	-1.26	6.11E-04	1.37E-03
**ADGRA2**	1.24	6.13E-04	1.38E-03
**AL356776.1**	-2.58	6.13E-04	1.38E-03
**FCF1**	0.36	6.14E-04	1.38E-03
**RNU6-613P**	-4.47	6.14E-04	1.38E-03
**KRTAP3-2**	-5.42	6.14E-04	1.38E-03
**UBE2G1**	0.34	6.15E-04	1.38E-03
**LRRC2-AS1**	-4.15	6.15E-04	1.38E-03
**ZNF574**	0.46	6.16E-04	1.38E-03
**RP4-800G7.3**	-2.00	6.16E-04	1.38E-03
**RP1-278C19.8**	-4.43	6.16E-04	1.38E-03
**AC018693.6**	-4.08	6.16E-04	1.38E-03
**LCK**	1.24	6.17E-04	1.39E-03
**RP11-932O9.10**	-2.07	6.18E-04	1.39E-03
**RNU6-1171P**	-4.18	6.18E-04	1.39E-03
**NEGR1**	1.57	6.18E-04	1.39E-03
**RNA5SP283**	3.63	6.19E-04	1.39E-03
**TNFRSF18**	-0.89	6.19E-04	1.39E-03
**G35511**	-1.49	6.20E-04	1.39E-03
**LRAT**	-1.66	6.20E-04	1.39E-03
**MTFR1L**	0.49	6.21E-04	1.39E-03
**ADAT3**	0.74	6.22E-04	1.39E-03
**RP11-708J19.1**	1.13	6.22E-04	1.39E-03
**MTA3**	0.36	6.22E-04	1.39E-03
**ENC1**	0.81	6.22E-04	1.40E-03

**RP11-503L23.1**	-4.43	6.22E-04	1.40E-03
**SPRYD4**	0.44	6.23E-04	1.40E-03
**AL357673.1**	0.75	6.23E-04	1.40E-03
**BCL2L2-PABPN1**	-2.42	6.24E-04	1.40E-03
**HOXD-AS2**	1.71	6.25E-04	1.40E-03
**C1QL4**	-2.28	6.26E-04	1.40E-03
**CTD-2218G20.1**	-4.50	6.26E-04	1.40E-03
**RP5-836J3.1**	-3.56	6.26E-04	1.40E-03
**ALKBH3**	0.36	6.28E-04	1.41E-03
**RP11-406H23.2**	-2.58	6.29E-04	1.41E-03
**NSUN6**	-0.52	6.29E-04	1.41E-03
**MIR561**	-4.14	6.29E-04	1.41E-03
**SIK1**	-4.17	6.29E-04	1.41E-03
**AC018867.1**	-3.25	6.29E-04	1.41E-03
**LINC01208**	-4.25	6.29E-04	1.41E-03
**XLOC_004915**	-1.94	6.30E-04	1.41E-03
**XLOC_008282**	3.09	6.32E-04	1.41E-03
**ALOX12-AS1**	-1.00	6.32E-04	1.41E-03
**ECHDC3**	0.79	6.32E-04	1.41E-03
**CTC-412M14.5**	-3.52	6.32E-04	1.41E-03
**RNU6-645P**	-4.32	6.32E-04	1.41E-03
**CRLS1**	0.28	6.32E-04	1.42E-03
**RPL7P26**	-3.89	6.33E-04	1.42E-03
**RNU6-1282P**	-4.20	6.33E-04	1.42E-03
**PWWP2AP1**	-4.12	6.33E-04	1.42E-03
**NACAD**	1.31	6.33E-04	1.42E-03
**RRS1**	0.72	6.34E-04	1.42E-03
**AC069257.8**	-2.78	6.36E-04	1.42E-03
**C11orf30**	-0.42	6.37E-04	1.43E-03
**RNU7-196P**	-4.29	6.37E-04	1.43E-03
**AC007036.4**	-2.17	6.38E-04	1.43E-03
**FGFR1OP**	-0.70	6.38E-04	1.43E-03
**CLK3**	0.43	6.39E-04	1.43E-03
**RN7SKP56**	-4.18	6.41E-04	1.43E-03
**RP11-583F2.2**	-3.33	6.41E-04	1.43E-03
**CTD-3162L10.3**	-4.20	6.41E-04	1.43E-03
**RELL2**	1.20	6.42E-04	1.44E-03
**CTD-2001C12.1**	-1.86	6.43E-04	1.44E-03
**G42895**	-1.64	6.44E-04	1.44E-03
**GREB1**	-1.12	6.46E-04	1.45E-03
**HMGN2P38**	-4.26	6.46E-04	1.45E-03
**RP11-73G16.2**	-1.93	6.47E-04	1.45E-03
**XLOC_004697**	-1.93	6.47E-04	1.45E-03
**RPS2P39**	-4.19	6.47E-04	1.45E-03
**HS6ST1P1**	2.75	6.48E-04	1.45E-03

**AL356741.1**	-4.16	6.49E-04	1.45E-03
**CFAP69**	-0.84	6.49E-04	1.45E-03
**TAF5L**	0.30	6.49E-04	1.45E-03
**XKRX**	-1.04	6.49E-04	1.45E-03
**Z98941.1**	-4.40	6.49E-04	1.45E-03
**HLA-DQB2**	-1.17	6.52E-04	1.46E-03
**CTC-523E23.11**	1.41	6.53E-04	1.46E-03
**RNU6-1305P**	-3.86	6.54E-04	1.46E-03
**ACSL6**	-2.56	6.54E-04	1.46E-03
**TMEM139**	1.44	6.55E-04	1.46E-03
**VPS39**	0.32	6.55E-04	1.46E-03
**AKAP6**	-0.85	6.56E-04	1.46E-03
**KLHDC9**	0.98	6.56E-04	1.47E-03
**CTC-490G23.6**	-3.82	6.57E-04	1.47E-03
**TRPC1**	-0.65	6.57E-04	1.47E-03
**C11orf94**	-1.73	6.57E-04	1.47E-03
**AKAP17BP**	-2.89	6.57E-04	1.47E-03
**G354**	1.75	6.58E-04	1.47E-03
**AC022400.1**	-3.38	6.59E-04	1.47E-03
**SLC25A45**	1.05	6.60E-04	1.47E-03
**TDRD15**	-4.13	6.61E-04	1.48E-03
**AOC4P**	2.85	6.62E-04	1.48E-03
**NOBOX**	-4.15	6.63E-04	1.48E-03
**EDA**	-0.59	6.63E-04	1.48E-03
**TREH**	-2.92	6.63E-04	1.48E-03
**RP11-321F6.2**	-4.47	6.63E-04	1.48E-03
**ABCA9-AS1**	-4.06	6.64E-04	1.48E-03
**F2RL1**	-0.59	6.65E-04	1.48E-03
**XLOC_007868**	-2.02	6.65E-04	1.48E-03
**RP11-15A1.9**	-2.26	6.65E-04	1.48E-03
**RREB1**	-0.49	6.65E-04	1.48E-03
**MIR193B**	-4.24	6.66E-04	1.49E-03
**C10orf90**	-1.25	6.67E-04	1.49E-03
**CLYBL-AS2**	-4.04	6.67E-04	1.49E-03
**ZNF605**	-0.52	6.68E-04	1.49E-03
**MSS51**	-1.13	6.69E-04	1.49E-03
**RP11-689E3.2**	-4.17	6.69E-04	1.49E-03
**ADH1C**	3.50	6.70E-04	1.49E-03
**AL450226.2**	-3.11	6.70E-04	1.49E-03
**RPL23AP72**	-4.05	6.71E-04	1.50E-03
**RNY4P25**	-3.93	6.71E-04	1.50E-03
**RP11-120K24.4**	-2.66	6.73E-04	1.50E-03
**CTC-510F12.2**	2.34	6.74E-04	1.50E-03
**CCDC30**	-0.80	6.75E-04	1.50E-03
**HEXB**	0.55	6.76E-04	1.51E-03

**RPL5P32**	-4.26	6.76E-04	1.51E-03
**RN7SL748P**	-4.09	6.76E-04	1.51E-03
**LPAR3**	-0.67	6.76E-04	1.51E-03
**PAPD7**	-0.41	6.76E-04	1.51E-03
**TRIM6**	1.30	6.77E-04	1.51E-03
**RP11-392M9.2**	-4.09	6.77E-04	1.51E-03
**SLC36A1**	0.67	6.77E-04	1.51E-03
**AC009245.3**	-1.58	6.77E-04	1.51E-03
**CPS1**	-0.72	6.78E-04	1.51E-03
**LRP1-AS**	-2.97	6.79E-04	1.51E-03
**ABCC11**	-1.43	6.80E-04	1.51E-03
**RWDD2A**	0.49	6.80E-04	1.51E-03
**AC078864.1**	-3.89	6.80E-04	1.51E-03
**PSMC1P7**	-2.57	6.80E-04	1.51E-03
**OXCT2P1**	2.90	6.81E-04	1.52E-03
**RNU6-212P**	-4.24	6.81E-04	1.52E-03
**PRKG2**	-1.39	6.82E-04	1.52E-03
**HLA-K**	1.83	6.82E-04	1.52E-03
**MIR497HG**	-1.78	6.84E-04	1.52E-03
**GTF2I**	-0.33	6.86E-04	1.53E-03
**KB-1980E6.3**	4.25	6.86E-04	1.53E-03
**RP11-440G5.2**	2.81	6.86E-04	1.53E-03
**ARFGAP3**	0.46	6.86E-04	1.53E-03
**IRX2**	0.45	6.86E-04	1.53E-03
**RP11-394I13.1**	2.28	6.88E-04	1.53E-03
**DIO3OS**	1.47	6.88E-04	1.53E-03
**XXbac-BPG252P9.9**	-2.67	6.89E-04	1.53E-03
**LMAN2L**	0.54	6.89E-04	1.53E-03
**PIGFP2**	-4.41	6.89E-04	1.53E-03
**CEACAM6**	2.24	6.89E-04	1.53E-03
**CDC20**	0.83	6.91E-04	1.54E-03
**AC010883.5**	-2.20	6.91E-04	1.54E-03
**RP4-635A23.3**	-4.14	6.91E-04	1.54E-03
**RP11-324E6.6**	-4.03	6.93E-04	1.54E-03
**AC016700.2**	-4.19	6.93E-04	1.54E-03
**BIN1**	1.09	6.94E-04	1.54E-03
**CTNS**	0.51	6.94E-04	1.54E-03
**RP11-388K2.1**	-4.41	6.95E-04	1.54E-03
**TRIM62**	0.73	6.95E-04	1.54E-03
**EIF3CL**	-1.11	6.95E-04	1.54E-03
**TSG101**	0.42	6.97E-04	1.55E-03
**CTA-339C12.1**	-2.88	6.98E-04	1.55E-03
**RP4-657D16.6**	-4.13	6.98E-04	1.55E-03
**RP11-177G23.1**	-2.25	6.98E-04	1.55E-03
**COX7CP1**	-3.81	6.98E-04	1.55E-03

**RP4-673D20.6**	-1.98	6.99E-04	1.55E-03
**HLA-W**	-3.26	6.99E-04	1.55E-03
**MIR4449**	-4.12	6.99E-04	1.55E-03
**RP11-757F18.3**	-1.93	7.00E-04	1.55E-03
**RP11-557C18.3**	-3.31	7.01E-04	1.56E-03
**6-Sep**	0.89	7.02E-04	1.56E-03
**RP11-203M5.2**	-4.15	7.02E-04	1.56E-03
**LINC00167**	-2.54	7.02E-04	1.56E-03
**WNT10B**	-0.97	7.03E-04	1.56E-03
**RP1-149A16.3**	-2.66	7.03E-04	1.56E-03
**MIEF1**	0.31	7.03E-04	1.56E-03
**OSBPL1A**	0.55	7.04E-04	1.56E-03
**PRELID2**	-0.79	7.04E-04	1.56E-03
**NCF1B**	2.21	7.06E-04	1.57E-03
**RGS1**	1.55	7.06E-04	1.57E-03
**RP11-38J22.6**	-4.35	7.06E-04	1.57E-03
**RP11-177B4.1**	-1.70	7.06E-04	1.57E-03
**CTD-2314B22.2**	4.66	7.06E-04	1.57E-03
**C15orf53**	-4.20	7.07E-04	1.57E-03
**RPL21P136**	-2.47	7.07E-04	1.57E-03
**RP11-435J9.2**	-2.43	7.07E-04	1.57E-03
**PHLDA1**	0.59	7.07E-04	1.57E-03
**LINC00664**	-2.33	7.08E-04	1.57E-03
**KPRP**	1.19	7.08E-04	1.57E-03
**AC006946.12**	-2.77	7.08E-04	1.57E-03
**AC017074.2**	-1.13	7.09E-04	1.57E-03
**RP13-228J13.8**	-4.00	7.10E-04	1.57E-03
**OXTR**	1.81	7.10E-04	1.57E-03
**SNURF**	-2.73	7.13E-04	1.58E-03
**GPR37**	0.75	7.14E-04	1.58E-03
**PCYOX1**	-0.45	7.14E-04	1.58E-03
**MAST4-AS1**	-1.05	7.15E-04	1.58E-03
**PPM1B**	-0.44	7.16E-04	1.59E-03
**MTATP6P1**	3.05	7.17E-04	1.59E-03
**RP11-673F18.1**	-3.73	7.18E-04	1.59E-03
**DSCR9**	-3.03	7.19E-04	1.59E-03
**RP11-713N11.5**	-4.50	7.20E-04	1.59E-03
**C6orf203**	0.43	7.20E-04	1.59E-03
**PXK**	-0.34	7.22E-04	1.60E-03
**ZNF609**	-0.32	7.22E-04	1.60E-03
**ISPD**	-0.70	7.22E-04	1.60E-03
**AC002310.10**	-4.33	7.23E-04	1.60E-03
**C1orf61**	-2.61	7.24E-04	1.60E-03
**RCBTB2P1**	-3.72	7.24E-04	1.60E-03
**AC008073.9**	-4.32	7.24E-04	1.60E-03

**RN7SL809P**	-2.41	7.25E-04	1.60E-03
**RP4-784A16.4**	-4.35	7.25E-04	1.61E-03
**CXCL12**	1.56	7.25E-04	1.61E-03
**FAM26F**	1.90	7.25E-04	1.61E-03
**MFAP1**	0.53	7.27E-04	1.61E-03
**CTNNAL1**	-0.59	7.27E-04	1.61E-03
**RP1-138B7.4**	-3.92	7.27E-04	1.61E-03
**RP11-219B17.3**	-1.48	7.29E-04	1.61E-03
**AC078883.3**	-1.55	7.29E-04	1.61E-03
**SMIM11A**	-4.05	7.30E-04	1.62E-03
**RP11-478C6.2**	-3.61	7.31E-04	1.62E-03
**AC061992.2**	3.25	7.31E-04	1.62E-03
**RP11-679B19.2**	-4.39	7.31E-04	1.62E-03
**BEST2**	-1.50	7.31E-04	1.62E-03
**XLOC_007955**	3.03	7.31E-04	1.62E-03
**PKMYT1**	0.80	7.32E-04	1.62E-03
**MYO1A**	-1.68	7.32E-04	1.62E-03
**IBSP**	6.19	7.32E-04	1.62E-03
**RP11-347E10.1**	-4.03	7.33E-04	1.62E-03
**ABHD11**	0.61	7.33E-04	1.62E-03
**RP11-310E22.4**	-2.80	7.33E-04	1.62E-03
**MIR545**	-4.06	7.33E-04	1.62E-03
**FAM86JP**	-0.93	7.35E-04	1.62E-03
**IKBKAP**	-0.54	7.35E-04	1.63E-03
**G35786**	-2.99	7.36E-04	1.63E-03
**RNU6-57P**	-4.24	7.37E-04	1.63E-03
**RP11-2N1.2**	-3.55	7.39E-04	1.63E-03
**NXT2**	-0.64	7.39E-04	1.63E-03
**PADI1**	1.35	7.39E-04	1.63E-03
**MLXIP**	0.82	7.40E-04	1.63E-03
**YRDCP1**	-3.95	7.40E-04	1.63E-03
**TRIM25**	-0.41	7.40E-04	1.64E-03
**RP11-15I20.1**	-4.16	7.41E-04	1.64E-03
**RP11-87H9.5**	-4.20	7.42E-04	1.64E-03
**ATP8B3**	1.23	7.42E-04	1.64E-03
**ILK**	-1.85	7.42E-04	1.64E-03
**RP11-69L16.4**	-3.89	7.43E-04	1.64E-03
**RP5-832C2.5**	1.02	7.43E-04	1.64E-03
**PODXL2**	0.73	7.43E-04	1.64E-03
**WDR12**	-0.40	7.44E-04	1.64E-03
**RNU1-27P**	-4.48	7.44E-04	1.64E-03
**DGCR8**	0.44	7.45E-04	1.64E-03
**RP3-329A5.8**	-1.80	7.45E-04	1.64E-03
**RP11-324D17.1**	-5.08	7.45E-04	1.65E-03
**PRKCA**	-0.64	7.46E-04	1.65E-03

**RP11-111A22.1**	-2.10	7.46E-04	1.65E-03
**MIR219A2**	-4.18	7.47E-04	1.65E-03
**AC007163.3**	3.54	7.47E-04	1.65E-03
**CTD-2215E18.3**	-4.24	7.48E-04	1.65E-03
**FAM124B**	0.95	7.49E-04	1.65E-03
**RP11-1109F11.5**	-3.78	7.50E-04	1.65E-03
**CTB-77H17.1**	-4.12	7.50E-04	1.66E-03
**CTD-2284J15.1**	-1.02	7.50E-04	1.66E-03
**NDUFA5P11**	-4.10	7.50E-04	1.66E-03
**RPLP1P13**	-4.18	7.50E-04	1.66E-03
**RP11-1149M10.1**	-3.06	7.51E-04	1.66E-03
**RP11-578O24.2**	-1.51	7.51E-04	1.66E-03
**CNOT10-AS1**	-3.23	7.52E-04	1.66E-03
**RP11-452L6.6**	1.92	7.53E-04	1.66E-03
**RP4-791M13.3**	-2.19	7.53E-04	1.66E-03
**IL7**	-0.80	7.54E-04	1.66E-03
**CHL1-AS1**	-1.95	7.54E-04	1.66E-03
**HOXC-AS1**	1.08	7.56E-04	1.67E-03
**DBH-AS1**	3.58	7.58E-04	1.67E-03
**FTH1P1**	-3.93	7.58E-04	1.67E-03
**MIR644A**	-4.02	7.59E-04	1.67E-03
**PLAC8L1**	-1.30	7.60E-04	1.68E-03
**RP11-77G23.2**	-4.08	7.60E-04	1.68E-03
**XLOC_002780**	-4.07	7.61E-04	1.68E-03
**KLRD1**	1.42	7.62E-04	1.68E-03
**GSTM3P2**	-4.34	7.62E-04	1.68E-03
**RP11-134K13.4**	-1.94	7.63E-04	1.68E-03
**OVOL1**	-0.79	7.63E-04	1.68E-03
**LINC01560**	0.64	7.65E-04	1.68E-03
**PVRIG**	-3.46	7.65E-04	1.68E-03
**RP11-1017G21.4**	-2.07	7.65E-04	1.69E-03
**LIN7B**	0.75	7.65E-04	1.69E-03
**ARPC5**	0.45	7.66E-04	1.69E-03
**RP13-941N14.1**	-2.37	7.67E-04	1.69E-03
**RNU6-540P**	-3.94	7.67E-04	1.69E-03
**C10orf91**	1.87	7.69E-04	1.69E-03
**YWHAB**	0.36	7.69E-04	1.69E-03
**AC004945.1**	-4.36	7.71E-04	1.70E-03
**ZNF705E**	-2.98	7.71E-04	1.70E-03
**CTD-2313N18.8**	-4.27	7.72E-04	1.70E-03
**GLYATL1P4**	-2.85	7.73E-04	1.70E-03
**KRTAP3-3**	-5.64	7.73E-04	1.70E-03
**TRAF3IP1**	0.42	7.74E-04	1.70E-03
**RP11-769O8.3**	-2.96	7.74E-04	1.70E-03
**RP11-89K11.1**	2.22	7.77E-04	1.71E-03

**RP11-1016B18.1**	-4.66	7.77E-04	1.71E-03
**LINC01424**	-3.32	7.78E-04	1.71E-03
**RP4-665N4.8**	-2.41	7.78E-04	1.71E-03
**SPRTN**	-0.46	7.78E-04	1.71E-03
**CCDC148**	1.72	7.79E-04	1.71E-03
**RP11-527N22.2**	2.23	7.80E-04	1.72E-03
**AC000003.1**	-4.22	7.80E-04	1.72E-03
**SRBD1**	-0.56	7.81E-04	1.72E-03
**RP11-392O17.1**	2.04	7.81E-04	1.72E-03
**RP11-465B22.3**	1.13	7.81E-04	1.72E-03
**TRAK2**	-0.55	7.81E-04	1.72E-03
**RASA4DP**	2.08	7.81E-04	1.72E-03
**AC006160.5**	-2.27	7.82E-04	1.72E-03
**MPRIP-AS1**	-1.66	7.83E-04	1.72E-03
**EXOC4**	-0.39	7.84E-04	1.72E-03
**PUSL1**	0.57	7.84E-04	1.72E-03
**NSUN4**	0.30	7.85E-04	1.72E-03
**PRDM5**	-0.79	7.87E-04	1.73E-03
**DEFB109P1B**	-4.25	7.88E-04	1.73E-03
**CTD-2326C4.1**	-4.44	7.88E-04	1.73E-03
**G24514**	-1.76	7.88E-04	1.73E-03
**JCHAIN**	-3.17	7.88E-04	1.73E-03
**KIAA1644**	1.27	7.88E-04	1.73E-03
**VWA3A**	1.48	7.89E-04	1.73E-03
**AC007566.10**	-1.54	7.89E-04	1.73E-03
**RP11-495P10.2**	2.76	7.90E-04	1.73E-03
**RP11-318K15.2**	-3.83	7.90E-04	1.73E-03
**XLOC_006578**	-4.57	7.90E-04	1.74E-03
**ZNF839P1**	-3.03	7.91E-04	1.74E-03
**TRAF3**	0.38	7.91E-04	1.74E-03
**RP11-241J12.1**	-4.37	7.91E-04	1.74E-03
**CTD-2314G24.2**	-2.41	7.92E-04	1.74E-03
**HINT3**	-0.56	7.93E-04	1.74E-03
**G3524**	-2.57	7.93E-04	1.74E-03
**RP3-508I15.19**	-3.23	7.93E-04	1.74E-03
**GPR17**	-1.55	7.94E-04	1.74E-03
**C10orf76**	0.33	7.94E-04	1.74E-03
**RP11-349G13.1**	-2.43	7.95E-04	1.75E-03
**GATA3**	-0.73	7.97E-04	1.75E-03
**MTRF1LP2**	-1.91	7.98E-04	1.75E-03
**GBP1P1**	1.42	7.99E-04	1.75E-03
**RP11-386I14.4**	-1.56	7.99E-04	1.75E-03
**RP5-1125A11.6**	-1.69	7.99E-04	1.75E-03
**SEC14L3**	-4.55	7.99E-04	1.75E-03
**LRP1B**	-1.40	7.99E-04	1.75E-03

**RP11-423O2.1**	-2.84	8.01E-04	1.76E-03
**RP11-132J14.3**	-3.93	8.01E-04	1.76E-03
**SMARCC1**	-0.37	8.02E-04	1.76E-03
**G40766**	-3.33	8.03E-04	1.76E-03
**RP5-1037N22.2**	-4.17	8.03E-04	1.76E-03
**RP11-680L20.1**	-4.40	8.04E-04	1.76E-03
**LSM8**	-0.37	8.04E-04	1.76E-03
**SLC25A18**	-1.89	8.04E-04	1.76E-03
**MIR3611**	-4.30	8.05E-04	1.76E-03
**XLOC_002996**	-1.87	8.05E-04	1.77E-03
**FAM151B**	-0.77	8.07E-04	1.77E-03
**AC096775.2**	-4.20	8.08E-04	1.77E-03
**G28331**	-1.14	8.08E-04	1.77E-03
**ZNF25**	-0.54	8.08E-04	1.77E-03
**PTGER4**	0.93	8.09E-04	1.77E-03
**RNASEH2B-AS1**	-1.72	8.11E-04	1.78E-03
**LRRC37A9P**	-3.86	8.11E-04	1.78E-03
**RP11-174O3.3**	-4.29	8.11E-04	1.78E-03
**MIR4482-1**	-2.26	8.11E-04	1.78E-03
**RP11-407N8.6**	-4.03	8.13E-04	1.78E-03
**RP11-529F4.1**	-1.26	8.13E-04	1.78E-03
**LY6D**	-0.61	8.14E-04	1.78E-03
**PSMC1P9**	-1.90	8.14E-04	1.78E-03
**CTD-2553L13.7**	-3.25	8.15E-04	1.79E-03
**AP000223.42**	-1.73	8.16E-04	1.79E-03
**LINC01497**	-3.53	8.16E-04	1.79E-03
**FCF1P1**	-2.85	8.17E-04	1.79E-03
**CORO6**	1.34	8.18E-04	1.79E-03
**MASTL**	-0.45	8.18E-04	1.79E-03
**AC005154.7**	-1.86	8.18E-04	1.79E-03
**RP11-723D22.3**	2.48	8.19E-04	1.79E-03
**CTB-174D11.3**	-3.88	8.20E-04	1.79E-03
**RP11-552F3.4**	-4.11	8.20E-04	1.80E-03
**RP6-109B7.5**	-1.04	8.20E-04	1.80E-03
**RP11-603B24.1**	3.61	8.21E-04	1.80E-03
**CD44**	-0.35	8.23E-04	1.80E-03
**BX470102.3**	-1.64	8.23E-04	1.80E-03
**RP11-448G15.1**	-4.06	8.23E-04	1.80E-03
**RP11-456H18.2**	-2.16	8.24E-04	1.80E-03
**RNU6-893P**	-3.92	8.25E-04	1.81E-03
**RP11-328D5.1**	-4.45	8.26E-04	1.81E-03
**RP11-54O7.17**	2.05	8.26E-04	1.81E-03
**HSPD1P1**	-4.17	8.26E-04	1.81E-03
**RP11-569G9.7**	-2.23	8.27E-04	1.81E-03
**SLC35D3**	-4.26	8.27E-04	1.81E-03

**ABHD12**	0.54	8.29E-04	1.81E-03
**AP003774.4**	3.06	8.29E-04	1.81E-03
**N4BP1**	0.36	8.30E-04	1.81E-03
**STMN1P1**	-3.93	8.30E-04	1.81E-03
**RN7SL434P**	-3.86	8.31E-04	1.82E-03
**RP11-477H21.2**	-1.38	8.31E-04	1.82E-03
**PRSS37**	-2.80	8.31E-04	1.82E-03
**RP1-134E15.3**	-1.99	8.32E-04	1.82E-03
**RP11-543B16.1**	-3.87	8.32E-04	1.82E-03
**LIN28A**	-3.90	8.32E-04	1.82E-03
**PTPN20A**	-2.10	8.32E-04	1.82E-03
**RN7SL225P**	-4.42	8.32E-04	1.82E-03
**KB-1208A12.3**	-1.01	8.33E-04	1.82E-03
**HMGB1P7**	-3.92	8.34E-04	1.82E-03
**CARD11**	1.49	8.34E-04	1.82E-03
**MKRN9P**	-3.80	8.35E-04	1.82E-03
**RP5-1065P14.3**	-4.04	8.35E-04	1.82E-03
**LILRA6**	2.13	8.36E-04	1.82E-03
**RP11-383I23.2**	-3.20	8.36E-04	1.83E-03
**AC010642.2**	-3.66	8.37E-04	1.83E-03
**RMST**	-4.41	8.37E-04	1.83E-03
**AC090420.1**	-4.11	8.38E-04	1.83E-03
**CTC-471J1.11**	-2.81	8.39E-04	1.83E-03
**FBXO38**	-0.31	8.39E-04	1.83E-03
**MED4-AS1**	-3.62	8.39E-04	1.83E-03
**RN7SL330P**	-4.21	8.40E-04	1.83E-03
**RFXAP**	0.72	8.41E-04	1.84E-03
**BTF3P6**	-4.02	8.41E-04	1.84E-03
**RP11-930O11.2**	-4.10	8.42E-04	1.84E-03
**RP11-250B2.5**	-2.19	8.42E-04	1.84E-03
**PLEKHM1**	0.39	8.42E-04	1.84E-03
**RP11-358D17.2**	-1.50	8.42E-04	1.84E-03
**HMGCS1**	-1.37	8.43E-04	1.84E-03
**SFXN4**	0.58	8.44E-04	1.84E-03
**RP11-292B8.1**	-1.34	8.46E-04	1.84E-03
**RP11-679C8.2**	-1.84	8.46E-04	1.84E-03
**SLFN11**	1.34	8.48E-04	1.85E-03
**FBXL8**	0.77	8.48E-04	1.85E-03
**BATF**	1.37	8.49E-04	1.85E-03
**ZNF300**	-0.71	8.49E-04	1.85E-03
**CTC-487M23.7**	-1.60	8.49E-04	1.85E-03
**TCEA1**	-0.48	8.49E-04	1.85E-03
**RP11-92K15.1**	-3.91	8.49E-04	1.85E-03
**FOXJ3**	-0.34	8.49E-04	1.85E-03
**RP11-96K19.5**	-2.63	8.50E-04	1.85E-03

**KLF8**	-0.72	8.50E-04	1.85E-03
**OR9L1P**	-4.09	8.50E-04	1.85E-03
**RP11-131K5.2**	-4.02	8.50E-04	1.85E-03
**URB1-AS1**	0.84	8.51E-04	1.85E-03
**RP11-131K5.1**	-3.87	8.51E-04	1.85E-03
**RP11-90M2.5**	-3.00	8.51E-04	1.85E-03
**RP11-428C19.4**	-4.27	8.51E-04	1.86E-03
**TRIL**	1.30	8.52E-04	1.86E-03
**RP11-697E14.2**	-4.04	8.54E-04	1.86E-03
**CTC-484P3.3**	-3.99	8.54E-04	1.86E-03
**ACOT2**	1.13	8.54E-04	1.86E-03
**CTD-2353F22.1**	-3.18	8.55E-04	1.86E-03
**G8227**	-1.80	8.55E-04	1.86E-03
**RP11-174G6.5**	-1.16	8.56E-04	1.86E-03
**RP5-1184F4.5**	-3.27	8.56E-04	1.86E-03
**AC130689.5**	-4.19	8.56E-04	1.86E-03
**RP11-459E5.1**	-2.22	8.57E-04	1.87E-03
**AC011239.1**	-3.75	8.58E-04	1.87E-03
**SNAP29**	0.54	8.59E-04	1.87E-03
**SLC22A13**	-3.84	8.59E-04	1.87E-03
**PATL2**	-1.30	8.60E-04	1.87E-03
**RNF13**	-0.54	8.60E-04	1.87E-03
**SULT1C4**	1.30	8.62E-04	1.88E-03
**SOCS5P4**	-1.79	8.62E-04	1.88E-03
**ATP6V1E1**	0.54	8.62E-04	1.88E-03
**ITPKA**	1.88	8.63E-04	1.88E-03
**CENPU**	-0.89	8.64E-04	1.88E-03
**IL10RB-AS1**	1.82	8.64E-04	1.88E-03
**RP11-222K16.2**	2.60	8.65E-04	1.88E-03
**FAM47E**	-1.19	8.66E-04	1.88E-03
**RP3-510L9.1**	-3.40	8.66E-04	1.88E-03
**RP11-363L24.3**	-2.69	8.66E-04	1.88E-03
**CNIH4**	-0.37	8.66E-04	1.88E-03
**RNVU1-15**	-4.04	8.66E-04	1.88E-03
**LDAH**	-0.49	8.67E-04	1.89E-03
**DKFZP434L187**	-4.40	8.68E-04	1.89E-03
**CCL28**	0.82	8.70E-04	1.89E-03
**LMNTD2**	1.10	8.71E-04	1.89E-03
**AC012066.1**	-3.80	8.73E-04	1.90E-03
**RN7SKP274**	-4.07	8.77E-04	1.91E-03
**SNIP1**	-0.38	8.78E-04	1.91E-03
**G30917**	-3.30	8.79E-04	1.91E-03
**G43342**	-4.48	8.80E-04	1.91E-03
**RP4-539M6.14**	-4.97	8.81E-04	1.91E-03
**RP11-6B6.3**	-2.63	8.82E-04	1.92E-03

**MIR125B2**	-2.74	8.83E-04	1.92E-03
**SAMM50P1**	-4.03	8.83E-04	1.92E-03
**RP11-500M8.4**	-4.15	8.84E-04	1.92E-03
**CTD-2216M2.1**	-4.13	8.84E-04	1.92E-03
**MTRNR2L1**	-4.34	8.84E-04	1.92E-03
**TTC33**	-0.54	8.86E-04	1.92E-03
**RNU6-874P**	-4.13	8.86E-04	1.93E-03
**TECRL**	-4.51	8.87E-04	1.93E-03
**OXNAD1**	-0.43	8.89E-04	1.93E-03
**SEPHS1P1**	-2.68	8.90E-04	1.93E-03
**DLGAP1**	-1.08	8.90E-04	1.93E-03
**RP11-376O6.2**	-3.87	8.91E-04	1.93E-03
**RNU7-124P**	-4.30	8.93E-04	1.94E-03
**E2F3-IT1**	-4.01	8.93E-04	1.94E-03
**RP11-342L8.2**	-3.79	8.94E-04	1.94E-03
**RP11-129I19.2**	-2.40	8.95E-04	1.94E-03
**RELT**	0.87	8.95E-04	1.94E-03
**ZNF669**	-0.56	8.95E-04	1.94E-03
**ZP1**	-2.29	8.96E-04	1.95E-03
**ZNF224**	-0.55	8.97E-04	1.95E-03
**RN7SL671P**	-4.17	8.98E-04	1.95E-03
**ZBTB47**	0.80	8.98E-04	1.95E-03
**MIR548K**	-4.10	8.99E-04	1.95E-03
**C14orf28**	-0.58	8.99E-04	1.95E-03
**RP11-61J19.4**	1.22	9.00E-04	1.95E-03
**IQCB1**	-0.54	9.00E-04	1.95E-03
**RP11-843P14.2**	-3.36	9.00E-04	1.95E-03
**RP11-89F17.5**	-3.70	9.01E-04	1.95E-03
**RP11-275I14.4**	-0.86	9.02E-04	1.96E-03
**XLOC_008618**	-1.11	9.02E-04	1.96E-03
**PATE4**	-4.32	9.03E-04	1.96E-03
**RP11-180O5.2**	-3.07	9.04E-04	1.96E-03
**FAM134C**	-0.27	9.05E-04	1.96E-03
**RP11-351O1.3**	-4.47	9.05E-04	1.96E-03
**SLC6A2**	-1.65	9.05E-04	1.96E-03
**RNU6-1**	-2.36	9.06E-04	1.96E-03
**MEX3C**	-0.39	9.06E-04	1.96E-03
**RP11-158M2.6**	-3.78	9.07E-04	1.97E-03
**RP11-589B3.6**	-3.84	9.07E-04	1.97E-03
**C11orf74**	0.67	9.09E-04	1.97E-03
**EMP3**	1.09	9.10E-04	1.97E-03
**RPL23AP36**	-4.41	9.10E-04	1.97E-03
**ABLIM1**	-0.55	9.12E-04	1.98E-03
**ANTXRLP1**	-3.82	9.13E-04	1.98E-03
**MIR4742**	-4.33	9.13E-04	1.98E-03

**ST6GALNAC3**	1.15	9.14E-04	1.98E-03
**AC079779.4**	-4.04	9.15E-04	1.98E-03
**RNU6-479P**	-4.14	9.15E-04	1.98E-03
**RP11-804H8.7**	-3.77	9.15E-04	1.98E-03
**ZNF576**	0.48	9.15E-04	1.98E-03
**COQ4**	0.42	9.15E-04	1.98E-03
**MEGF6**	0.70	9.16E-04	1.98E-03
**TUBGCP5**	-0.35	9.16E-04	1.98E-03
**RP11-454K7.3**	-3.03	9.16E-04	1.98E-03
**RSL24D1P8**	-2.74	9.16E-04	1.98E-03
**GZF1**	-0.33	9.17E-04	1.98E-03
**PKD2L1**	3.34	9.17E-04	1.99E-03
**VNN2**	1.97	9.18E-04	1.99E-03
**SNORA36B**	-4.15	9.18E-04	1.99E-03
**MTCO2P11**	-3.85	9.18E-04	1.99E-03
**RNU6-1301P**	-4.16	9.18E-04	1.99E-03
**CRY2**	-0.65	9.18E-04	1.99E-03
**RP11-92F20.1**	-4.04	9.19E-04	1.99E-03
**CTB-119C2.1**	-1.72	9.19E-04	1.99E-03
**CTD-2537I9.12**	2.54	9.20E-04	1.99E-03
**AC092635.1**	-4.48	9.21E-04	1.99E-03
**RP11-19E11.1**	2.47	9.21E-04	1.99E-03
**WHAMMP3**	1.12	9.22E-04	1.99E-03
**RP5-875H3.2**	-3.93	9.23E-04	2.00E-03
**RP11-18C24.8**	-3.31	9.23E-04	2.00E-03
**AC004381.6**	-0.52	9.23E-04	2.00E-03
**RP11-360I2.1**	-3.94	9.23E-04	2.00E-03
**TXNL1**	0.35	9.24E-04	2.00E-03
**ZC3H11B**	-2.56	9.26E-04	2.00E-03
**CTD-2114J12.1**	-4.42	9.27E-04	2.01E-03
**ENPP1**	-1.23	9.28E-04	2.01E-03
**ATP6V1C1**	-0.37	9.29E-04	2.01E-03
**LINC01010**	1.05	9.29E-04	2.01E-03
**RP11-319G6.3**	-3.97	9.29E-04	2.01E-03
**ETV3**	-0.51	9.30E-04	2.01E-03
**PLEKHA6**	-1.08	9.30E-04	2.01E-03
**MAPK8**	-0.36	9.30E-04	2.01E-03
**XLOC_011471**	-4.29	9.31E-04	2.01E-03
**RP11-147L13.14**	-2.81	9.32E-04	2.01E-03
**RNU6-1050P**	-4.01	9.32E-04	2.01E-03
**RP11-46H11.11**	-1.54	9.33E-04	2.01E-03
**RP11-408I18.9**	-3.81	9.33E-04	2.02E-03
**RP11-400L8.2**	-1.62	9.33E-04	2.02E-03
**SFXN5**	0.53	9.33E-04	2.02E-03
**RN7SL706P**	-4.31	9.33E-04	2.02E-03

**BCL10**	-0.40	9.33E-04	2.02E-03
**RP11-380I10.2**	-3.98	9.34E-04	2.02E-03
**AP000302.58**	-3.79	9.35E-04	2.02E-03
**MIR3126**	-4.09	9.36E-04	2.02E-03
**RP11-384C4.7**	-3.12	9.37E-04	2.02E-03
**IFIT1B**	-4.07	9.39E-04	2.03E-03
**MIR2278**	-4.55	9.40E-04	2.03E-03
**RP11-803P9.1**	-1.94	9.44E-04	2.04E-03
**RP11-689B22.2**	-2.80	9.44E-04	2.04E-03
**NFIL3**	0.86	9.44E-04	2.04E-03
**PPIAP11**	-1.93	9.44E-04	2.04E-03
**PDK2**	0.60	9.44E-04	2.04E-03
**PTGES2-AS1**	-2.48	9.44E-04	2.04E-03
**RP11-29H23.7**	-2.74	9.45E-04	2.04E-03
**CCT3**	0.52	9.46E-04	2.04E-03
**NLRP12**	2.46	9.46E-04	2.04E-03
**RP11-312B8.2**	-3.93	9.47E-04	2.04E-03
**XLOC_003662**	-3.34	9.47E-04	2.04E-03
**AC138965.1**	-4.20	9.48E-04	2.04E-03
**ORC5**	-0.38	9.48E-04	2.05E-03
**G29811**	-2.50	9.49E-04	2.05E-03
**RP11-416A17.1**	-3.99	9.49E-04	2.05E-03
**RP11-983P16.2**	-1.37	9.50E-04	2.05E-03
**MACROD2-IT1**	-3.67	9.50E-04	2.05E-03
**REC8**	1.12	9.51E-04	2.05E-03
**LAMP3**	-0.94	9.52E-04	2.05E-03
**UBE2Q2**	0.47	9.52E-04	2.05E-03
**AC018463.4**	-3.80	9.53E-04	2.05E-03
**HLA-DQB1**	-2.89	9.53E-04	2.06E-03
**ANKRD44-IT1**	-2.15	9.56E-04	2.06E-03
**RNA5SP256**	-4.19	9.56E-04	2.06E-03
**CTD-2560C21.1**	-4.14	9.57E-04	2.06E-03
**RP11-503P10.1**	-2.62	9.58E-04	2.06E-03
**AC017083.2**	-3.88	9.58E-04	2.06E-03
**BCAR3**	-0.74	9.58E-04	2.06E-03
**ARID1B**	-0.38	9.59E-04	2.07E-03
**RP13-270P17.1**	-2.02	9.59E-04	2.07E-03
**MVK**	0.76	9.59E-04	2.07E-03
**MIR4766**	-3.95	9.60E-04	2.07E-03
**RP11-47F1.1**	-4.45	9.60E-04	2.07E-03
**RP11-416A14.1**	-4.09	9.61E-04	2.07E-03
**LRRC15**	2.57	9.62E-04	2.07E-03
**KCNK15**	1.41	9.62E-04	2.07E-03
**AP000487.4**	-3.97	9.62E-04	2.07E-03
**CTC-518B2.9**	-3.96	9.63E-04	2.07E-03

**RP11-90E5.1**	-4.03	9.63E-04	2.07E-03
**HLA-L**	-1.45	9.64E-04	2.08E-03
**AC105316.1**	-4.09	9.64E-04	2.08E-03
**RN7SL328P**	-2.43	9.65E-04	2.08E-03
**SGOL1-AS1**	-1.78	9.66E-04	2.08E-03
**MIR623**	-2.42	9.66E-04	2.08E-03
**CDC42BPB**	0.29	9.66E-04	2.08E-03
**RP11-582J16.4**	-0.83	9.66E-04	2.08E-03
**RPS20P15**	-3.96	9.67E-04	2.08E-03
**WDR31**	-0.56	9.67E-04	2.08E-03
**HOXB2**	1.44	9.68E-04	2.08E-03
**DUOX2**	0.98	9.69E-04	2.08E-03
**RN7SL532P**	-3.84	9.70E-04	2.09E-03
**CXCL9**	3.25	9.71E-04	2.09E-03
**RP11-545M17.2**	-4.02	9.72E-04	2.09E-03
**LDLR**	0.90	9.72E-04	2.09E-03
**RNU6-8**	-2.07	9.73E-04	2.09E-03
**RP11-114F10.3**	-1.42	9.73E-04	2.09E-03
**RP11-462G22.2**	-3.30	9.73E-04	2.09E-03
**EXOSC3P1**	-2.12	9.74E-04	2.09E-03
**ZBTB16**	-1.53	9.75E-04	2.10E-03
**snR65**	-4.10	9.75E-04	2.10E-03
**WNT10A**	-0.90	9.75E-04	2.10E-03
**RP11-434H6.7**	-1.21	9.75E-04	2.10E-03
**RP11-255A11.4**	-4.06	9.75E-04	2.10E-03
**APOA1-AS**	-3.85	9.77E-04	2.10E-03
**MIR1972-1**	-4.07	9.77E-04	2.10E-03
**RPL26P35**	-3.71	9.77E-04	2.10E-03
**OLAH**	-4.45	9.79E-04	2.10E-03
**RNU6-151P**	-3.93	9.81E-04	2.11E-03
**RN7SL566P**	-3.94	9.82E-04	2.11E-03
**RP1-68D18.3**	-4.07	9.82E-04	2.11E-03
**IRX1**	1.07	9.83E-04	2.11E-03
**FAM105A**	-0.70	9.83E-04	2.11E-03
**SNORD119**	-3.67	9.83E-04	2.11E-03
**RP4-796I17.5**	-3.95	9.85E-04	2.12E-03
**RP11-612J15.1**	-4.19	9.85E-04	2.12E-03
**RNU6-720P**	-3.62	9.86E-04	2.12E-03
**SERAC1**	-0.44	9.86E-04	2.12E-03
**MUT**	-0.49	9.86E-04	2.12E-03
**CDK2AP2P2**	2.69	9.87E-04	2.12E-03
**RP5-827C21.2**	-4.13	9.87E-04	2.12E-03
**MIR1256**	-4.14	9.87E-04	2.12E-03
**RBM19**	0.39	9.89E-04	2.12E-03
**RP11-487E1.2**	-4.13	9.89E-04	2.12E-03

**INPP5J**	1.08	9.90E-04	2.12E-03
**RP11-260E18.1**	-3.84	9.90E-04	2.13E-03
**PIP4K2C**	0.43	9.90E-04	2.13E-03
**CTD-2256P15.4**	-3.62	9.91E-04	2.13E-03
**SUMO4**	-1.78	9.91E-04	2.13E-03
**RP11-195C7.3**	-3.86	9.91E-04	2.13E-03
**TVP23CP2**	-3.93	9.92E-04	2.13E-03
**ISPD-AS1**	-2.50	9.93E-04	2.13E-03
**AF131215.9**	-0.84	9.93E-04	2.13E-03
**RPS4XP22**	-2.37	9.93E-04	2.13E-03
**SLC7A14**	-3.04	9.94E-04	2.13E-03
**WDR82**	0.32	9.94E-04	2.13E-03
**ERV3-1**	-1.52	9.95E-04	2.13E-03
**ATG13**	0.40	9.95E-04	2.13E-03
**HAS1**	-1.59	9.96E-04	2.14E-03
**PTPN2P2**	-4.09	9.96E-04	2.14E-03
**AC007875.2**	-4.47	9.97E-04	2.14E-03
**NUS1P1**	1.76	9.97E-04	2.14E-03
**RP11-506B6.6**	-1.81	9.98E-04	2.14E-03
**SLC4A8**	1.88	9.99E-04	2.14E-03
**RNU6-315P**	-3.74	1.00E-03	2.14E-03
**FAXC**	1.71	1.00E-03	2.14E-03
**RP11-907D1.3**	-4.12	1.00E-03	2.15E-03
**RTFDC1**	0.41	1.00E-03	2.15E-03
**AC007161.5**	-2.75	1.00E-03	2.15E-03
**AC090286.2**	-3.87	1.00E-03	2.15E-03
**PUS3**	0.47	1.00E-03	2.15E-03
**FAM168B**	0.28	1.00E-03	2.15E-03
**TRPM2**	1.24	1.00E-03	2.15E-03
**N4BP3**	0.45	1.00E-03	2.15E-03
**RP11-45A17.3**	-1.37	1.00E-03	2.15E-03
**BRSK1**	2.07	1.01E-03	2.15E-03
**CTD-2540F13.2**	1.78	1.01E-03	2.15E-03
**RP11-259N19.1**	1.33	1.01E-03	2.15E-03
**GK-AS1**	-2.34	1.01E-03	2.15E-03
**SCNN1B**	0.82	1.01E-03	2.16E-03
**QRFP**	-1.27	1.01E-03	2.16E-03
**SOCS2-AS1**	1.47	1.01E-03	2.16E-03
**RP11-844G16.3**	-4.12	1.01E-03	2.16E-03
**BTN3A2**	0.93	1.01E-03	2.16E-03
**RPL12P24**	-4.05	1.01E-03	2.17E-03
**MIR339**	3.14	1.01E-03	2.17E-03
**CTC-471J1.2**	-0.78	1.01E-03	2.17E-03
**RP11-20G13.3**	-3.75	1.01E-03	2.17E-03
**RP11-394B2.6**	-3.94	1.02E-03	2.17E-03

**AC007557.3**	-3.92	1.02E-03	2.18E-03
**AC109992.1**	-4.22	1.02E-03	2.18E-03
**VNN1**	1.64	1.02E-03	2.18E-03
**MRPS9**	-0.47	1.02E-03	2.18E-03
**BLID**	-4.03	1.02E-03	2.18E-03
**GAPT**	-1.12	1.02E-03	2.18E-03
**RP11-383C5.3**	-3.65	1.02E-03	2.18E-03
**RP11-477G18.2**	-3.38	1.02E-03	2.19E-03
**ASTL**	-1.48	1.02E-03	2.19E-03
**MAGI2**	0.67	1.02E-03	2.19E-03
**ZBTB40-IT1**	-3.10	1.03E-03	2.19E-03
**EEF1A1P9**	-1.50	1.03E-03	2.19E-03
**RPL3P4**	0.76	1.03E-03	2.19E-03
**AL161645.1**	-2.90	1.03E-03	2.20E-03
**OR7E94P**	-2.56	1.03E-03	2.20E-03
**ARID1A**	0.53	1.03E-03	2.20E-03
**CASP5**	2.99	1.03E-03	2.20E-03
**G36170**	-1.78	1.03E-03	2.21E-03
**PLEKHB1**	1.79	1.04E-03	2.21E-03
**RP6-149D17.1**	-3.90	1.04E-03	2.22E-03
**PUM3**	-0.41	1.04E-03	2.22E-03
**CTD-2062A1.2**	-3.23	1.04E-03	2.22E-03
**G34279**	-4.32	1.04E-03	2.22E-03
**YRDC**	0.53	1.04E-03	2.22E-03
**THNSL1**	-0.66	1.04E-03	2.22E-03
**CTC-232P5.3**	-3.36	1.04E-03	2.22E-03
**RP11-82L18.2**	-1.32	1.04E-03	2.22E-03
**RNU6-202P**	-4.10	1.04E-03	2.23E-03
**SIM2**	-1.44	1.04E-03	2.23E-03
**RP11-435O5.4**	-3.78	1.04E-03	2.23E-03
**RGS13**	-1.66	1.04E-03	2.23E-03
**NPIPB11**	1.33	1.05E-03	2.23E-03
**GTF2IP14**	-1.42	1.05E-03	2.24E-03
**MIR580**	-4.06	1.05E-03	2.24E-03
**STS**	-0.90	1.05E-03	2.24E-03
**RP5-875O13.1**	-3.94	1.05E-03	2.24E-03
**CUZD1**	-1.09	1.05E-03	2.24E-03
**AP000563.2**	-4.11	1.05E-03	2.24E-03
**RNF223**	1.03	1.05E-03	2.24E-03
**RP11-3K24.2**	-3.92	1.05E-03	2.25E-03
**ARRDC1-AS1**	0.75	1.05E-03	2.25E-03
**CTB-131B5.5**	-1.61	1.05E-03	2.25E-03
**PARVG**	1.42	1.05E-03	2.25E-03
**RP11-100N20.1**	-4.58	1.05E-03	2.25E-03
**GXYLT1P4**	-4.14	1.05E-03	2.25E-03

**RP11-802F5.1**	-3.89	1.06E-03	2.25E-03
**RP11-188D8.1**	-2.51	1.06E-03	2.25E-03
**ANKRD20A3**	-3.66	1.06E-03	2.25E-03
**ZNF625**	-2.06	1.06E-03	2.25E-03
**NAV2-IT1**	-4.17	1.06E-03	2.26E-03
**THNSL2**	1.38	1.06E-03	2.26E-03
**LINC00272**	-3.95	1.06E-03	2.26E-03
**CPEB3_ribozyme**	-3.92	1.06E-03	2.26E-03
**RN7SL127P**	-3.17	1.06E-03	2.26E-03
**PPIAP10**	-4.10	1.06E-03	2.27E-03
**ST8SIA6**	-0.89	1.06E-03	2.27E-03
**C3orf62**	0.53	1.07E-03	2.27E-03
**RNU6-314P**	-4.32	1.07E-03	2.27E-03
**PABPC5-AS1**	-4.03	1.07E-03	2.27E-03
**UBE2Q2P1**	0.93	1.07E-03	2.27E-03
**EXOSC6**	-0.50	1.07E-03	2.27E-03
**ECE2**	0.74	1.07E-03	2.28E-03
**PAWR**	-0.50	1.07E-03	2.28E-03
**FBXO42**	-0.39	1.07E-03	2.28E-03
**CTA-268H5.12**	-2.62	1.07E-03	2.29E-03
**XXbac-B476C20.13**	-2.64	1.08E-03	2.29E-03
**SELPLG**	1.32	1.08E-03	2.29E-03
**CSDC2**	1.58	1.08E-03	2.30E-03
**ENTPD1**	0.74	1.08E-03	2.30E-03
**RP11-863P13.5**	-1.86	1.08E-03	2.30E-03
**PTPRM**	0.73	1.08E-03	2.30E-03
**RP11-476I15.5**	-4.00	1.08E-03	2.30E-03
**RP11-53B2.5**	-3.64	1.08E-03	2.30E-03
**AC004159.1**	-4.01	1.08E-03	2.30E-03
**GS1-251I9.3**	-4.36	1.08E-03	2.30E-03
**RP11-147L13.11**	0.49	1.08E-03	2.30E-03
**ENTPD5**	-0.40	1.08E-03	2.30E-03
**RP11-546K22.1**	-2.58	1.08E-03	2.30E-03
**GALNTL6**	-1.27	1.08E-03	2.31E-03
**SPRY2**	0.48	1.08E-03	2.31E-03
**CTD-2562G15.2**	-3.76	1.08E-03	2.31E-03
**MIR2909**	-4.09	1.09E-03	2.31E-03
**SETP17**	-3.68	1.09E-03	2.31E-03
**RP11-893F2.14**	-4.02	1.09E-03	2.31E-03
**TCERG1**	-0.44	1.09E-03	2.31E-03
**G42447**	-3.77	1.09E-03	2.31E-03
**C8orf37**	-0.64	1.09E-03	2.31E-03
**PRICKLE2-AS2**	-3.94	1.09E-03	2.31E-03
**RP5-1000K24.2**	-0.78	1.09E-03	2.31E-03
**HS3ST6**	-0.94	1.09E-03	2.31E-03

**HNRNPCP2**	1.03	1.09E-03	2.31E-03
**RP11-640N11.2**	-3.94	1.09E-03	2.31E-03
**TCEB1P33**	-2.90	1.09E-03	2.31E-03
**RP11-275I4.1**	-3.20	1.09E-03	2.31E-03
**G42437**	-4.83	1.09E-03	2.31E-03
**EIF4G2**	0.36	1.09E-03	2.32E-03
**CTC-490G23.2**	4.54	1.09E-03	2.32E-03
**CLIP1**	-0.47	1.09E-03	2.32E-03
**ACAT1**	-0.55	1.09E-03	2.32E-03
**LINC00426**	-1.21	1.09E-03	2.32E-03
**MIR620**	-4.41	1.09E-03	2.32E-03
**CPEB2-AS1**	-1.24	1.09E-03	2.32E-03
**PRMT5-AS1**	-2.53	1.10E-03	2.33E-03
**RN7SL482P**	-4.02	1.10E-03	2.34E-03
**SUPT16H**	-0.37	1.10E-03	2.34E-03
**DUTP1**	-2.73	1.10E-03	2.34E-03
**KRBA2**	-0.89	1.10E-03	2.34E-03
**RNU6-1285P**	-3.86	1.10E-03	2.34E-03
**HCAR1**	-1.75	1.10E-03	2.34E-03
**GLYCTK-AS1**	-2.03	1.10E-03	2.35E-03
**AL136419.6**	1.41	1.10E-03	2.35E-03
**RP11-560A15.6**	-4.32	1.10E-03	2.35E-03
**RP11-24B13.1**	-4.37	1.10E-03	2.35E-03
**RP11-1113L8.1**	-4.11	1.11E-03	2.35E-03
**XDH**	0.99	1.11E-03	2.35E-03
**THEM5**	0.92	1.11E-03	2.35E-03
**SPATA12**	-1.39	1.11E-03	2.35E-03
**AC024569.1**	-3.85	1.11E-03	2.35E-03
**RP11-715F3.2**	-1.95	1.11E-03	2.35E-03
**AC013410.2**	-3.69	1.11E-03	2.35E-03
**HSD3BP3**	-4.21	1.11E-03	2.35E-03
**RP11-271K11.5**	-1.78	1.11E-03	2.36E-03
**RP11-76C10.3**	-4.38	1.11E-03	2.36E-03
**ZNF174**	0.48	1.11E-03	2.36E-03
**KANSL1-AS1**	-1.63	1.12E-03	2.37E-03
**SMS**	0.51	1.12E-03	2.37E-03
**ZBTB20-AS2**	-4.14	1.12E-03	2.37E-03
**NGEF**	-0.64	1.12E-03	2.37E-03
**RP11-85G20.2**	-4.18	1.12E-03	2.37E-03
**RP11-611O2.6**	-2.94	1.12E-03	2.37E-03
**TSHZ1**	0.36	1.12E-03	2.38E-03
**RP11-319G9.5**	-2.73	1.12E-03	2.38E-03
**HAUS4**	-0.59	1.12E-03	2.38E-03
**EDC3**	0.50	1.12E-03	2.38E-03
**GPER1**	0.83	1.12E-03	2.38E-03

**RP11-90P5.5**	-4.02	1.12E-03	2.38E-03
**LINC01139**	1.69	1.12E-03	2.38E-03
**OR7E12P**	2.54	1.12E-03	2.39E-03
**FAM216A**	-0.64	1.12E-03	2.39E-03
**RP11-53I6.4**	-1.57	1.13E-03	2.39E-03
**RP11-46A10.8**	-2.34	1.13E-03	2.39E-03
**AC096732.2**	-4.18	1.13E-03	2.40E-03
**CBX3**	0.50	1.13E-03	2.40E-03
**BANF1P4**	-3.91	1.13E-03	2.40E-03
**PAGR1**	0.76	1.13E-03	2.40E-03
**RP11-864J10.2**	-3.91	1.13E-03	2.40E-03
**PARM1**	0.90	1.13E-03	2.40E-03
**RP11-406H21.2**	-3.89	1.13E-03	2.40E-03
**CDK17**	-0.34	1.13E-03	2.40E-03
**SERBP1P6**	-1.91	1.14E-03	2.41E-03
**TONSL**	0.58	1.14E-03	2.41E-03
**CCDC157**	1.05	1.14E-03	2.41E-03
**SLC1A6**	-1.03	1.14E-03	2.41E-03
**S1PR4**	1.47	1.14E-03	2.41E-03
**SNORD7**	-2.67	1.14E-03	2.42E-03
**MROH6**	0.65	1.14E-03	2.42E-03
**KRT13**	1.90	1.14E-03	2.42E-03
**RP11-138P22.1**	-3.94	1.14E-03	2.42E-03
**TIMM8A**	-0.60	1.14E-03	2.42E-03
**RP11-2K6.2**	-3.97	1.15E-03	2.42E-03
**FABP5P5**	-4.05	1.15E-03	2.43E-03
**UTP23**	-0.43	1.15E-03	2.43E-03
**LRRC8D**	0.44	1.15E-03	2.43E-03
**MYO19**	-0.49	1.15E-03	2.43E-03
**ZNF766**	-0.44	1.15E-03	2.43E-03
**MFHAS1**	0.57	1.15E-03	2.43E-03
**PLAGL2**	0.61	1.15E-03	2.43E-03
**C10orf25**	1.34	1.15E-03	2.44E-03
**RP11-12J10.4**	-2.19	1.15E-03	2.44E-03
**RNU7-113P**	-4.05	1.15E-03	2.44E-03
**JAG1**	0.34	1.15E-03	2.44E-03
**TTC23L**	-1.29	1.16E-03	2.44E-03
**RP11-313J2.1**	-1.42	1.16E-03	2.45E-03
**ABCA8**	-1.23	1.16E-03	2.45E-03
**DDHD1**	-0.68	1.16E-03	2.45E-03
**AC092291.2**	-4.18	1.16E-03	2.45E-03
**LINC01006**	0.86	1.16E-03	2.45E-03
**MTND6P5**	-3.98	1.16E-03	2.46E-03
**GTF2IP9**	-2.34	1.16E-03	2.46E-03
**PLAC1**	-2.42	1.16E-03	2.46E-03

**RP11-119F7.3**	-4.05	1.16E-03	2.46E-03
**AC004112.4**	-1.73	1.16E-03	2.46E-03
**GRHL2**	-0.49	1.16E-03	2.46E-03
**CTD-2313J17.1**	-3.71	1.16E-03	2.46E-03
**RP1-253P7.4**	-1.46	1.16E-03	2.46E-03
**EDA2R**	1.23	1.17E-03	2.47E-03
**RP11-5C23.2**	-1.58	1.17E-03	2.47E-03
**UVSSA**	-0.75	1.17E-03	2.47E-03
**SNORD121A**	-4.03	1.17E-03	2.47E-03
**IQCK**	0.65	1.17E-03	2.47E-03
**IL5**	-3.81	1.17E-03	2.47E-03
**RP11-419I17.1**	-3.51	1.17E-03	2.47E-03
**MASP1**	1.24	1.17E-03	2.47E-03
**EPB41L4A-AS1**	0.48	1.17E-03	2.48E-03
**C8G**	1.75	1.17E-03	2.48E-03
**C1orf174**	0.35	1.17E-03	2.48E-03
**RP11-382A20.5**	-4.29	1.17E-03	2.48E-03
**SNORA27**	-4.20	1.17E-03	2.48E-03
**RP11-171I2.2**	-2.65	1.17E-03	2.48E-03
**SMC2**	-0.42	1.17E-03	2.48E-03
**IMPAD1**	-0.47	1.17E-03	2.48E-03
**MRPS10P1**	-4.06	1.18E-03	2.48E-03
**ITGB7**	-0.79	1.18E-03	2.48E-03
**RSU1P2**	-4.10	1.18E-03	2.48E-03
**AL049647.1**	-3.91	1.18E-03	2.48E-03
**RNU6-998P**	-3.68	1.18E-03	2.49E-03
**LAMC3**	1.32	1.18E-03	2.49E-03
**SYAP1**	-0.44	1.18E-03	2.49E-03
**RP11-338K17.10**	-1.93	1.18E-03	2.49E-03
**EIF4EBP1P1**	-3.80	1.18E-03	2.49E-03
**RP11-1085N6.2**	-2.74	1.18E-03	2.49E-03
**RNU6ATAC24P**	-3.67	1.18E-03	2.49E-03
**RP11-434D12.1**	-1.27	1.18E-03	2.49E-03
**RP11-184M15.1**	2.09	1.18E-03	2.50E-03
**KRT7**	2.68	1.18E-03	2.50E-03
**EPX**	-1.38	1.19E-03	2.50E-03
**STAG3L1**	1.21	1.19E-03	2.50E-03
**RN7SKP40**	-3.88	1.19E-03	2.50E-03
**CTD-3018O17.6**	-4.16	1.19E-03	2.51E-03
**UBR5-AS1**	-0.77	1.19E-03	2.51E-03
**TES**	0.34	1.19E-03	2.51E-03
**RP11-81M19.3**	1.67	1.19E-03	2.51E-03
**PRR26**	-1.50	1.19E-03	2.52E-03
**ERICH3**	-4.00	1.20E-03	2.52E-03
**RP11-632P5.1**	-1.64	1.20E-03	2.52E-03

**POU1F1**	-3.75	1.20E-03	2.52E-03
**RP13-638C3.2**	-3.03	1.20E-03	2.52E-03
**OR52W1**	-3.85	1.20E-03	2.52E-03
**DFFB**	0.88	1.20E-03	2.52E-03
**LACTB**	0.41	1.20E-03	2.52E-03
**RPL17P40**	-4.00	1.20E-03	2.53E-03
**G2136**	-2.17	1.20E-03	2.53E-03
**SNORD116-2**	-3.79	1.20E-03	2.53E-03
**ARHGAP23**	0.55	1.20E-03	2.54E-03
**PRKRIR**	-0.42	1.21E-03	2.54E-03
**RP4-704D21.2**	-4.02	1.21E-03	2.54E-03
**RP5-867C24.5**	-2.62	1.21E-03	2.54E-03
**UGP2**	0.32	1.21E-03	2.55E-03
**RP11-319G6.1**	-0.72	1.21E-03	2.55E-03
**KCNMA1-AS1**	-2.97	1.21E-03	2.55E-03
**TRBV29OR9-2**	-3.23	1.21E-03	2.55E-03
**RBBP8P1**	-4.00	1.21E-03	2.55E-03
**C2CD4B**	1.40	1.21E-03	2.55E-03
**KB-1930G5.4**	-2.09	1.21E-03	2.55E-03
**CTD-2012J19.1**	-3.71	1.21E-03	2.55E-03
**LACE1**	-0.52	1.21E-03	2.55E-03
**YAP1**	-0.25	1.21E-03	2.55E-03
**RNU6-787P**	-3.73	1.21E-03	2.55E-03
**GCH1**	-0.60	1.21E-03	2.56E-03
**AP006621.5**	0.75	1.21E-03	2.56E-03
**RP11-104N10.1**	-1.51	1.22E-03	2.56E-03
**SMG6**	0.37	1.22E-03	2.56E-03
**SMC1B**	-1.61	1.22E-03	2.56E-03
**OR5C1**	-4.01	1.22E-03	2.56E-03
**GPR173**	1.17	1.22E-03	2.56E-03
**CCDC152**	-0.81	1.22E-03	2.57E-03
**CD200R1**	-1.40	1.22E-03	2.57E-03
**CHRNA2**	-2.06	1.22E-03	2.57E-03
**RNU6ATAC12P**	-3.97	1.22E-03	2.57E-03
**RP3-348I23.2**	-3.62	1.22E-03	2.57E-03
**XLOC_013463**	-4.36	1.22E-03	2.58E-03
**DDX11L9**	-3.75	1.23E-03	2.58E-03
**G1997**	-1.34	1.23E-03	2.58E-03
**IFT22**	0.63	1.23E-03	2.58E-03
**FRAT1**	0.65	1.23E-03	2.59E-03
**RNU6-82P**	-2.76	1.23E-03	2.59E-03
**RP11-763F8.1**	-4.19	1.23E-03	2.59E-03
**RP5-1142A6.10**	-1.63	1.23E-03	2.59E-03
**FAM200B**	-0.41	1.24E-03	2.60E-03
**TCFL5**	0.30	1.24E-03	2.60E-03

**RP11-368P15.2**	-3.50	1.24E-03	2.60E-03
**RP5-966M1.7**	-1.95	1.24E-03	2.60E-03
**NXPE4**	-4.00	1.25E-03	2.62E-03
**RN7SL206P**	-3.95	1.25E-03	2.62E-03
**SLC37A2**	0.99	1.25E-03	2.62E-03
**TTC26**	-0.48	1.25E-03	2.62E-03
**LRRC2**	-1.12	1.25E-03	2.62E-03
**G10656**	1.44	1.25E-03	2.62E-03
**OTOF**	-2.19	1.25E-03	2.62E-03
**FNDC5**	-1.52	1.25E-03	2.63E-03
**C19orf52**	0.40	1.25E-03	2.63E-03
**MMAB**	0.52	1.25E-03	2.63E-03
**DNAH2**	-1.40	1.25E-03	2.63E-03
**RPS2P55**	-3.93	1.25E-03	2.63E-03
**MIR4787**	2.37	1.25E-03	2.63E-03
**MIR3193**	-4.03	1.25E-03	2.63E-03
**STRIP2**	1.10	1.25E-03	2.63E-03
**C9orf47**	-1.75	1.26E-03	2.64E-03
**RP5-966M1.4**	-3.95	1.26E-03	2.64E-03
**RP11-102N12.3**	1.51	1.26E-03	2.64E-03
**RP11-83A24.2**	-0.71	1.26E-03	2.64E-03
**RP11-285A1.1**	-2.63	1.26E-03	2.65E-03
**RPS7P15**	-3.90	1.26E-03	2.65E-03
**TEX10**	-0.30	1.26E-03	2.65E-03
**IL5RA**	-1.79	1.26E-03	2.65E-03
**SEPHS1**	0.33	1.26E-03	2.65E-03
**DCTN1-AS1**	-2.35	1.26E-03	2.65E-03
**RP11-190D6.2**	-3.99	1.27E-03	2.66E-03
**EIF4G1**	0.39	1.27E-03	2.66E-03
**CTD-3222D19.9**	-1.65	1.27E-03	2.66E-03
**RP11-506H20.1**	-1.76	1.27E-03	2.66E-03
**BBS2**	-0.41	1.27E-03	2.67E-03
**RP4-620E11.8**	-1.21	1.27E-03	2.67E-03
**CTD-2623N2.11**	-3.78	1.27E-03	2.67E-03
**USP27X**	0.43	1.27E-03	2.67E-03
**SLC41A2**	-1.17	1.27E-03	2.67E-03
**RNU7-96P**	-4.01	1.27E-03	2.67E-03
**ZNF276**	0.72	1.28E-03	2.67E-03
**G40864**	-2.65	1.28E-03	2.67E-03
**MPO**	4.02	1.28E-03	2.68E-03
**DBF4B**	1.01	1.28E-03	2.68E-03
**HABP2**	4.25	1.28E-03	2.68E-03
**G10181**	-1.07	1.28E-03	2.68E-03
**PPP2R2A**	0.41	1.28E-03	2.68E-03
**RP11-519G16.5**	-1.61	1.28E-03	2.68E-03

**ACRBP**	1.52	1.28E-03	2.68E-03
**CTD-2184D3.1**	-4.16	1.28E-03	2.68E-03
**XLOC_003378**	1.63	1.28E-03	2.68E-03
**CTB-133G6.1**	-2.45	1.28E-03	2.68E-03
**RNU7-107P**	-4.14	1.28E-03	2.68E-03
**APOBEC4**	-3.15	1.28E-03	2.68E-03
**AC009948.5**	-0.67	1.28E-03	2.69E-03
**CDC42BPA**	-0.45	1.28E-03	2.69E-03
**ATF2**	-0.42	1.28E-03	2.69E-03
**ZBTB18**	-0.41	1.28E-03	2.69E-03
**GAPDHP62**	-3.80	1.28E-03	2.69E-03
**TIAF1**	-1.16	1.28E-03	2.69E-03
**RP11-77H9.8**	-3.55	1.28E-03	2.69E-03
**SH2D4A**	0.69	1.28E-03	2.69E-03
**LRCH4**	0.73	1.29E-03	2.69E-03
**ZFHX4-AS1**	4.05	1.29E-03	2.70E-03
**RP11-312P12.3**	-3.97	1.29E-03	2.70E-03
**ZNF165**	-0.77	1.29E-03	2.70E-03
**RP5-907D15.4**	4.34	1.29E-03	2.70E-03
**CTB-189B5.3**	-3.37	1.29E-03	2.70E-03
**RP11-647O20.1**	-3.69	1.29E-03	2.70E-03
**RP11-147L13.7**	-2.21	1.29E-03	2.70E-03
**IDI2**	-4.16	1.29E-03	2.71E-03
**MIR125B1**	-4.01	1.30E-03	2.71E-03
**RP11-305E17.4**	-3.83	1.30E-03	2.71E-03
**KCNA4**	-2.37	1.30E-03	2.71E-03
**CFL1P3**	-3.98	1.30E-03	2.72E-03
**CTC-303L1.1**	-2.59	1.30E-03	2.72E-03
**HNRNPC**	0.42	1.30E-03	2.72E-03
**RPL5P13**	-4.20	1.30E-03	2.73E-03
**MAP6**	1.19	1.30E-03	2.73E-03
**RP11-576C2.1**	-2.95	1.30E-03	2.73E-03
**TMEM254-AS1**	-0.78	1.30E-03	2.73E-03
**AP000266.7**	-3.21	1.30E-03	2.73E-03
**RP11-231L11.3**	-1.97	1.30E-03	2.73E-03
**RP11-75C10.6**	-1.89	1.31E-03	2.73E-03
**RP11-243J18.2**	-3.76	1.31E-03	2.73E-03
**EIF1P7**	-3.96	1.31E-03	2.73E-03
**SC22CB-1D7.1**	-3.84	1.31E-03	2.73E-03
**JPH2**	1.12	1.31E-03	2.73E-03
**RP11-197N18.8**	-1.57	1.31E-03	2.74E-03
**ANKRD13A**	-0.41	1.31E-03	2.74E-03
**RP11-406A9.2**	-4.28	1.31E-03	2.74E-03
**KCNJ2-AS1**	0.88	1.31E-03	2.75E-03
**POMGNT2**	0.40	1.31E-03	2.75E-03

**SNORA62**	-3.56	1.32E-03	2.75E-03
**RP11-506O24.2**	-2.29	1.32E-03	2.75E-03
**VASH2**	-0.97	1.32E-03	2.75E-03
**DXO**	0.64	1.32E-03	2.76E-03
**NLGN1-AS1**	-4.39	1.32E-03	2.76E-03
**EBLN2**	-1.07	1.32E-03	2.76E-03
**CTB-139P11.2**	-3.57	1.32E-03	2.76E-03
**RP5-892K4.1**	-3.57	1.33E-03	2.77E-03
**RP11-753H16.5**	-2.49	1.33E-03	2.77E-03
**RP11-756J15.2**	-3.67	1.33E-03	2.77E-03
**RP11-798G7.6**	-2.23	1.33E-03	2.77E-03
**RNU6-435P**	-4.16	1.33E-03	2.77E-03
**RP11-486G15.1**	-4.06	1.33E-03	2.77E-03
**NVL**	-0.39	1.33E-03	2.77E-03
**VEPH1**	-0.87	1.33E-03	2.77E-03
**EXO1**	-0.69	1.33E-03	2.77E-03
**CT45A11P**	-3.81	1.33E-03	2.77E-03
**PMP2**	-2.36	1.33E-03	2.78E-03
**RNU6-766P**	-4.05	1.33E-03	2.78E-03
**PTPN1**	0.31	1.34E-03	2.79E-03
**RP11-758N13.3**	-3.72	1.34E-03	2.79E-03
**LINC01058**	-1.67	1.34E-03	2.79E-03
**MIR3162**	-3.95	1.34E-03	2.79E-03
**PTGES3P2**	-2.85	1.34E-03	2.80E-03
**RP11-3D4.4**	-3.47	1.34E-03	2.80E-03
**GPR141**	-1.53	1.34E-03	2.80E-03
**SREBF2**	0.51	1.34E-03	2.80E-03
**SMIM10L2B**	0.57	1.35E-03	2.81E-03
**EN2**	1.97	1.35E-03	2.81E-03
**CKMT2-AS1**	0.50	1.35E-03	2.81E-03
**TMTC2**	-0.43	1.35E-03	2.81E-03
**RP11-420K14.1**	-4.76	1.35E-03	2.81E-03
**OR9I3P**	-4.23	1.35E-03	2.81E-03
**RN7SL166P**	-3.45	1.35E-03	2.82E-03
**RP11-530C5.1**	-3.14	1.35E-03	2.82E-03
**ACA59**	-3.13	1.35E-03	2.82E-03
**ASAH1**	-0.49	1.35E-03	2.82E-03
**CKS1B**	0.61	1.35E-03	2.82E-03
**XLOC_012881**	4.39	1.35E-03	2.82E-03
**SLC6A11**	0.97	1.35E-03	2.82E-03
**EXT2**	0.42	1.35E-03	2.82E-03
**RP5-1110E20.1**	-3.81	1.35E-03	2.82E-03
**KB-1410C5.1**	-3.59	1.35E-03	2.82E-03
**KCNIP4-IT1**	-4.82	1.36E-03	2.83E-03
**RP11-352M15.2**	-0.41	1.36E-03	2.83E-03

**RP5-1100H13.3**	-4.08	1.36E-03	2.83E-03
**CCDC121**	-0.64	1.36E-03	2.83E-03
**RP11-120M18.2**	-1.77	1.36E-03	2.83E-03
**RP11-79N23.1**	-1.55	1.36E-03	2.84E-03
**RP11-91I20.4**	-2.82	1.36E-03	2.84E-03
**MIR301A**	-4.22	1.36E-03	2.84E-03
**RP11-74M13.4**	-2.89	1.36E-03	2.84E-03
**ZNF214**	-0.89	1.36E-03	2.84E-03
**TAPT1-AS1**	-1.09	1.36E-03	2.84E-03
**CTD-2033D15.2**	2.59	1.36E-03	2.84E-03
**BLOC1S5**	-0.35	1.37E-03	2.85E-03
**BAZ2A**	0.45	1.37E-03	2.85E-03
**TECPR2**	0.38	1.37E-03	2.85E-03
**EPRS**	-0.45	1.37E-03	2.85E-03
**HDAC6**	0.39	1.37E-03	2.85E-03
**RPUSD4**	0.29	1.37E-03	2.85E-03
**MCTP1**	0.72	1.37E-03	2.85E-03
**MYBL1**	-0.64	1.37E-03	2.85E-03
**CHN2**	-0.80	1.37E-03	2.85E-03
**CYBB**	1.45	1.37E-03	2.86E-03
**CTD-3064H18.4**	-1.85	1.38E-03	2.86E-03
**RNU5B-3P**	-3.71	1.38E-03	2.86E-03
**CTB-184G21.3**	-3.16	1.38E-03	2.86E-03
**RP11-154J22.1**	-1.64	1.38E-03	2.87E-03
**RP11-91A18.4**	-3.54	1.38E-03	2.87E-03
**AZIN1-AS1**	-0.91	1.38E-03	2.87E-03
**C14orf105**	-3.16	1.38E-03	2.87E-03
**SPRR3**	9.08	1.38E-03	2.87E-03
**GAPDHP46**	-4.08	1.38E-03	2.87E-03
**RP13-488H8.1**	-4.11	1.38E-03	2.87E-03
**AC020629.1**	-3.86	1.38E-03	2.87E-03
**CD38**	2.07	1.38E-03	2.87E-03
**WIPF1**	1.07	1.38E-03	2.87E-03
**RP11-378J18.8**	-0.82	1.38E-03	2.87E-03
**ZNF774**	-1.16	1.38E-03	2.88E-03
**RP4-616B8.6**	-3.95	1.39E-03	2.88E-03
**RP11-767N6.7**	-0.68	1.39E-03	2.88E-03
**CTD-2026K11.6**	-1.39	1.39E-03	2.89E-03
**XLOC_005448**	-3.46	1.39E-03	2.89E-03
**RPL21P28**	1.16	1.39E-03	2.89E-03
**MGAT5B**	1.85	1.39E-03	2.89E-03
**AC108059.4**	-4.32	1.39E-03	2.89E-03
**SREBF1**	0.63	1.39E-03	2.90E-03
**MYL6P4**	-4.11	1.39E-03	2.90E-03
**RNU6-446P**	-3.80	1.39E-03	2.90E-03

**SERPINB7**	-0.97	1.40E-03	2.90E-03
**CTD-3128G10.7**	3.69	1.40E-03	2.90E-03
**CYCSP4**	-4.14	1.40E-03	2.90E-03
**KCP**	1.64	1.40E-03	2.90E-03
**RP11-261P13.6**	-2.51	1.40E-03	2.90E-03
**RP11-1H15.2**	-4.14	1.40E-03	2.90E-03
**RAB39A**	-2.20	1.40E-03	2.91E-03
**PTHLH**	1.42	1.40E-03	2.91E-03
**RP11-16L21.7**	-4.05	1.40E-03	2.91E-03
**CTC-353G13.1**	-3.05	1.40E-03	2.91E-03
**RN7SL501P**	-3.79	1.40E-03	2.91E-03
**RP11-568G11.5**	-4.19	1.40E-03	2.91E-03
**ENTHD1**	-2.52	1.41E-03	2.92E-03
**XLOC_002793**	-1.72	1.41E-03	2.92E-03
**RP11-737O24.3**	-2.28	1.41E-03	2.92E-03
**SEMA3G**	1.26	1.41E-03	2.92E-03
**KTN1-AS1**	-0.53	1.41E-03	2.92E-03
**FLJ46066**	-3.00	1.41E-03	2.93E-03
**FAM83H-AS1**	-0.62	1.41E-03	2.93E-03
**DHX33**	0.45	1.41E-03	2.93E-03
**AC018878.3**	-4.04	1.41E-03	2.93E-03
**KMO**	-1.41	1.41E-03	2.93E-03
**BIN2**	1.06	1.41E-03	2.93E-03
**FAM73B**	0.49	1.41E-03	2.93E-03
**TAF1B**	-0.46	1.41E-03	2.93E-03
**RBM43P1**	-2.10	1.42E-03	2.93E-03
**RP11-562A8.4**	-1.38	1.42E-03	2.94E-03
**WBP2NL**	-0.98	1.42E-03	2.94E-03
**GRIK1-AS1**	-3.95	1.42E-03	2.94E-03
**CSTL1**	-4.46	1.42E-03	2.94E-03
**RPL39P5**	-3.06	1.42E-03	2.94E-03
**PF4**	-3.29	1.42E-03	2.95E-03
**SYT6**	-2.75	1.42E-03	2.95E-03
**WDFY4**	-1.17	1.42E-03	2.95E-03
**HNRNPA1P62**	-3.81	1.42E-03	2.95E-03
**RP11-473O4.1**	-2.73	1.42E-03	2.95E-03
**SLC5A9**	-0.96	1.42E-03	2.95E-03
**ZBED3**	0.97	1.42E-03	2.95E-03
**RNU6-1136P**	-3.81	1.42E-03	2.95E-03
**OTUD6B-AS1**	-0.54	1.42E-03	2.95E-03
**AC006262.10**	-3.94	1.43E-03	2.95E-03
**RP11-479F13.1**	-3.60	1.43E-03	2.96E-03
**RP11-731J8.2**	-3.48	1.43E-03	2.96E-03
**FNDC9**	-3.95	1.43E-03	2.96E-03
**PRKD1**	0.93	1.43E-03	2.96E-03

**RNU6-360P**	-3.83	1.43E-03	2.96E-03
**SLC35F3**	1.63	1.43E-03	2.96E-03
**G35954**	-3.99	1.43E-03	2.96E-03
**RPL31P60**	-4.04	1.43E-03	2.96E-03
**CEP126**	-0.62	1.43E-03	2.96E-03
**XLOC_012036**	-3.01	1.43E-03	2.96E-03
**RP11-813P10.1**	-4.08	1.43E-03	2.96E-03
**BCAS1**	1.39	1.43E-03	2.97E-03
**FAM126A**	-0.70	1.43E-03	2.97E-03
**CDC42SE2**	-0.44	1.43E-03	2.97E-03
**C3orf84**	-3.93	1.43E-03	2.97E-03
**CEP164P1**	-2.84	1.44E-03	2.97E-03
**GRIK5**	0.97	1.44E-03	2.97E-03
**AC092038.1**	-3.86	1.44E-03	2.98E-03
**AASDHPPT**	-0.44	1.44E-03	2.98E-03
**RP3-426I6.2**	-2.53	1.44E-03	2.99E-03
**MTCO2P19**	-4.13	1.44E-03	2.99E-03
**AC005740.5**	-1.68	1.45E-03	2.99E-03
**AP000487.6**	-1.91	1.45E-03	2.99E-03
**RN7SKP154**	-4.02	1.45E-03	2.99E-03
**TUBA3E**	-3.67	1.45E-03	2.99E-03
**GOLPH3L**	-0.28	1.45E-03	2.99E-03
**SNORA36**	-3.58	1.45E-03	2.99E-03
**RP3-514P16.1**	-3.71	1.45E-03	3.00E-03
**CCDC117**	-0.35	1.45E-03	3.00E-03
**AC105402.4**	-4.07	1.45E-03	3.00E-03
**STK17A**	0.48	1.45E-03	3.00E-03
**CNN1**	1.39	1.45E-03	3.00E-03
**RP11-830F9.5**	1.15	1.45E-03	3.00E-03
**RNU6-195P**	-3.47	1.45E-03	3.01E-03
**TFPI**	1.19	1.45E-03	3.01E-03
**CD300C**	2.07	1.46E-03	3.01E-03
**DOC2A**	-2.33	1.46E-03	3.01E-03
**ANKRD53**	1.35	1.46E-03	3.01E-03
**RXFP1**	2.60	1.46E-03	3.01E-03
**RN7SL505P**	-3.22	1.46E-03	3.01E-03
**RP11-463C14.1**	-4.00	1.46E-03	3.01E-03
**SNAI3-AS1**	1.41	1.46E-03	3.01E-03
**PGM2**	0.60	1.46E-03	3.01E-03
**COMMD9**	0.28	1.46E-03	3.02E-03
**CNOT6**	-0.35	1.46E-03	3.03E-03
**RP11-354B3.1**	-3.86	1.47E-03	3.03E-03
**U7**	-4.01	1.47E-03	3.03E-03
**RP11-509J21.2**	-2.68	1.47E-03	3.03E-03
**HSP90AB2P**	-1.93	1.47E-03	3.03E-03

**FKBP3**	0.42	1.47E-03	3.03E-03
**RP11-394B2.7**	-3.91	1.47E-03	3.03E-03
**MYOC**	-2.28	1.47E-03	3.03E-03
**SPIN1**	-0.32	1.47E-03	3.03E-03
**LGALS9C**	5.44	1.47E-03	3.03E-03
**MFF**	-0.29	1.47E-03	3.04E-03
**G39617**	-3.99	1.47E-03	3.04E-03
**G41369**	-2.15	1.47E-03	3.04E-03
**RP11-775L16.1**	3.80	1.47E-03	3.04E-03
**SUSD2**	-0.85	1.47E-03	3.04E-03
**SMCO3**	1.45	1.48E-03	3.05E-03
**LL22NC03-86G7.1**	-0.73	1.48E-03	3.05E-03
**HBP1**	-0.53	1.48E-03	3.05E-03
**VRK2**	-0.39	1.48E-03	3.05E-03
**RP1-180M12.1**	-4.04	1.48E-03	3.05E-03
**TMEM135**	-0.58	1.48E-03	3.05E-03
**RP3-520B18.1**	-3.77	1.48E-03	3.06E-03
**RP11-132M7.3**	-1.69	1.48E-03	3.06E-03
**HCK**	1.09	1.48E-03	3.06E-03
**NUDCD1**	-0.43	1.49E-03	3.06E-03
**AC069394.1**	-3.90	1.49E-03	3.06E-03
**SLC15A4**	0.33	1.49E-03	3.07E-03
**RP11-436H11.5**	-4.16	1.49E-03	3.07E-03
**BNIP3P16**	-3.88	1.49E-03	3.07E-03
**RP11-382J24.2**	-3.53	1.49E-03	3.07E-03
**G19573**	2.44	1.49E-03	3.07E-03
**RP3-455J7.4**	3.09	1.49E-03	3.07E-03
**RP11-82K18.2**	-2.80	1.49E-03	3.08E-03
**MIR3146**	-3.95	1.49E-03	3.08E-03
**ABHD16A**	-1.13	1.50E-03	3.08E-03
**MIR3929**	-3.85	1.50E-03	3.09E-03
**RP11-257O5.4**	-2.28	1.50E-03	3.09E-03
**CYP7B1**	-0.60	1.50E-03	3.09E-03
**CTA-85E5.6**	-4.47	1.50E-03	3.09E-03
**USMG5P1**	-3.84	1.50E-03	3.09E-03
**RNU1-16P**	-3.41	1.51E-03	3.10E-03
**RP11-209D20.2**	-3.68	1.51E-03	3.10E-03
**CTB-2L9.1**	-3.92	1.51E-03	3.11E-03
**AC073063.10**	-2.48	1.51E-03	3.11E-03
**RBM44**	-1.34	1.51E-03	3.11E-03
**RP3-389A20.5**	-3.99	1.51E-03	3.11E-03
**PCDHGA9**	-0.47	1.51E-03	3.11E-03
**RN7SL441P**	-1.87	1.51E-03	3.12E-03
**RP11-520H14.7**	-2.00	1.51E-03	3.12E-03
**RP11-367H1.1**	-2.18	1.52E-03	3.12E-03

**XLOC_001188**	2.49	1.52E-03	3.12E-03
**RNU4ATAC11P**	-4.04	1.52E-03	3.13E-03
**GPATCH11**	-0.51	1.52E-03	3.13E-03
**SGK494**	-1.34	1.52E-03	3.13E-03
**RP11-40C11.2**	-3.97	1.53E-03	3.14E-03
**LINC01443**	1.88	1.53E-03	3.14E-03
**C3orf80**	3.11	1.53E-03	3.15E-03
**RP11-733O18.1**	1.92	1.53E-03	3.15E-03
**PECR**	-1.85	1.53E-03	3.15E-03
**RP5-1147A1.1**	-3.73	1.53E-03	3.15E-03
**FAM167A**	-0.66	1.53E-03	3.15E-03
**XLOC_010591**	-3.08	1.53E-03	3.15E-03
**OPN1SW**	1.15	1.53E-03	3.16E-03
**GPAM**	-0.55	1.54E-03	3.16E-03
**RP4-710M16.2**	-4.15	1.54E-03	3.16E-03
**DQX1**	0.96	1.54E-03	3.17E-03
**HCG27**	-1.25	1.54E-03	3.17E-03
**MALRD1**	-2.26	1.54E-03	3.17E-03
**RP5-965G21.6**	-1.07	1.54E-03	3.17E-03
**C7orf43**	0.73	1.54E-03	3.17E-03
**CTD-2353F22.2**	-1.53	1.54E-03	3.17E-03
**RP11-449L23.3**	-3.98	1.54E-03	3.17E-03
**CA11**	0.74	1.54E-03	3.17E-03
**PRKRA**	-0.35	1.54E-03	3.18E-03
**RP11-468H14.2**	-2.33	1.55E-03	3.18E-03
**RN7SL473P**	-1.72	1.55E-03	3.18E-03
**PTPRJ**	-0.57	1.55E-03	3.18E-03
**POLR2B**	-0.38	1.55E-03	3.18E-03
**CHIC2**	0.56	1.55E-03	3.18E-03
**RN7SL396P**	-3.89	1.55E-03	3.19E-03
**RNY1P4**	-3.38	1.55E-03	3.19E-03
**RP1-161P9.5**	-2.83	1.55E-03	3.19E-03
**ANGPTL6**	1.45	1.55E-03	3.19E-03
**LINC00174**	0.89	1.56E-03	3.20E-03
**LINC01260**	2.66	1.56E-03	3.20E-03
**RPL21P1**	-2.99	1.56E-03	3.20E-03
**MEOX2-AS1**	2.66	1.56E-03	3.20E-03
**CTD-2313J17.5**	-1.45	1.56E-03	3.20E-03
**ACTG1P20**	-1.53	1.56E-03	3.20E-03
**GGACT**	0.73	1.56E-03	3.20E-03
**CGGBP1**	-0.34	1.56E-03	3.20E-03
**MRPS33**	0.52	1.57E-03	3.22E-03
**RP11-93M12.1**	-3.69	1.57E-03	3.22E-03
**RP11-98L12.2**	-4.25	1.57E-03	3.22E-03
**CTC-503J8.4**	-3.47	1.57E-03	3.22E-03

**RP11-96D1.3**	-2.84	1.57E-03	3.22E-03
**RN7SL688P**	-3.65	1.57E-03	3.23E-03
**CTD-2154B17.3**	-3.97	1.57E-03	3.23E-03
**CRHR1**	2.28	1.57E-03	3.23E-03
**AC012593.1**	-3.98	1.57E-03	3.23E-03
**WDR93**	1.58	1.58E-03	3.23E-03
**RP4-635E18.8**	-1.29	1.58E-03	3.23E-03
**ZNF404**	-0.91	1.58E-03	3.24E-03
**CD209**	2.01	1.58E-03	3.24E-03
**ZNF277**	-0.51	1.58E-03	3.24E-03
**HEXIM1**	0.57	1.58E-03	3.24E-03
**BCORL1**	0.87	1.58E-03	3.24E-03
**AC019181.3**	-2.10	1.58E-03	3.24E-03
**RP13-578N3.3**	-2.27	1.58E-03	3.24E-03
**RP11-576N17.3**	-4.31	1.58E-03	3.25E-03
**AC006547.13**	0.96	1.58E-03	3.25E-03
**RP3-355L5.4**	-1.29	1.58E-03	3.25E-03
**RNA5SP201**	-3.72	1.58E-03	3.25E-03
**RP11-514F3.4**	-4.05	1.58E-03	3.25E-03
**AC003991.3**	-1.92	1.58E-03	3.25E-03
**RNU6-199P**	-4.08	1.59E-03	3.25E-03
**RP11-881M11.4**	-3.93	1.59E-03	3.25E-03
**KAT6B**	-0.37	1.59E-03	3.25E-03
**RP11-351D16.3**	1.05	1.59E-03	3.25E-03
**CCDC144B**	-1.42	1.59E-03	3.26E-03
**KB-1742H10.3**	-1.34	1.59E-03	3.26E-03
**SLC22A25**	-4.23	1.60E-03	3.27E-03
**RPS2P41**	-3.03	1.60E-03	3.27E-03
**WHSC1**	-0.31	1.60E-03	3.27E-03
**RNA5SP221**	-3.78	1.60E-03	3.27E-03
**SPACA6P-AS**	-3.86	1.60E-03	3.28E-03
**RP11-93B14.6**	-1.00	1.60E-03	3.28E-03
**RP11-130C19.3**	-2.10	1.60E-03	3.28E-03
**IGFBP7-AS1**	1.95	1.60E-03	3.29E-03
**RP11-320P11.1**	-3.41	1.60E-03	3.29E-03
**MRPL50P2**	-4.07	1.61E-03	3.29E-03
**RNF11B**	-4.04	1.61E-03	3.29E-03
**RP11-574K11.29**	-1.19	1.61E-03	3.29E-03
**FRAT2**	0.71	1.61E-03	3.29E-03
**RP11-426J5.1**	-4.25	1.61E-03	3.29E-03
**RP11-506M13.3**	-1.23	1.61E-03	3.29E-03
**EQTN**	-3.79	1.61E-03	3.30E-03
**G26744**	-1.46	1.61E-03	3.30E-03
**AC012360.4**	-2.62	1.61E-03	3.30E-03
**SDHCP2**	-3.86	1.61E-03	3.30E-03

**RP5-1029F21.4**	-2.89	1.61E-03	3.30E-03
**AC097658.1**	-0.85	1.61E-03	3.30E-03
**RP11-115J16.2**	-4.19	1.61E-03	3.30E-03
**RNU6-1190P**	-3.89	1.62E-03	3.31E-03
**GRAMD1B**	1.07	1.62E-03	3.31E-03
**C1D**	0.49	1.62E-03	3.31E-03
**RP11-303E16.2**	0.72	1.62E-03	3.31E-03
**TMEM63C**	-1.69	1.62E-03	3.32E-03
**RNU6-177P**	-4.24	1.62E-03	3.32E-03
**RALA**	0.39	1.62E-03	3.32E-03
**RP4-803A2.2**	-3.68	1.62E-03	3.32E-03
**RP11-728K20.2**	-2.69	1.62E-03	3.32E-03
**XRCC6**	0.44	1.62E-03	3.32E-03
**BLMH**	-0.76	1.62E-03	3.32E-03
**RP11-667M19.4**	-2.02	1.62E-03	3.32E-03
**RP11-766H1.1**	-3.75	1.63E-03	3.32E-03
**MYCBP**	-0.48	1.63E-03	3.33E-03
**LRRN3**	-1.26	1.63E-03	3.33E-03
**RP11-160A9.2**	-3.13	1.63E-03	3.33E-03
**STRADA**	-1.08	1.63E-03	3.33E-03
**RNU6-1026P**	-3.96	1.64E-03	3.35E-03
**IMMTP1**	-3.95	1.64E-03	3.35E-03
**XLOC_005302**	-3.85	1.64E-03	3.35E-03
**RP11-136C24.2**	-3.70	1.64E-03	3.35E-03
**CLEC1B**	-3.90	1.64E-03	3.36E-03
**RP11-331F9.3**	-3.08	1.64E-03	3.36E-03
**FSCN3**	-2.02	1.64E-03	3.36E-03
**RP1-39G22.4**	-3.64	1.65E-03	3.36E-03
**NADK2**	-0.46	1.65E-03	3.37E-03
**CTB-134F13.1**	-3.43	1.65E-03	3.37E-03
**TGOLN2**	0.39	1.65E-03	3.38E-03
**RP11-890B15.2**	-2.29	1.65E-03	3.38E-03
**CYP2S1**	0.99	1.66E-03	3.39E-03
**XLOC_014192**	-2.46	1.66E-03	3.39E-03
**RP11-158M2.4**	-3.10	1.66E-03	3.39E-03
**CTD-2383M3.1**	-1.24	1.66E-03	3.40E-03
**RP11-262H14.3**	-5.76	1.67E-03	3.40E-03
**RNU6-481P**	2.64	1.67E-03	3.40E-03
**XLOC_008895**	-4.38	1.67E-03	3.40E-03
**G5833**	-2.05	1.67E-03	3.40E-03
**RP11-399K21.12**	-3.65	1.67E-03	3.40E-03
**LRRC39**	-0.90	1.67E-03	3.41E-03
**RP11-408P14.1**	-0.90	1.67E-03	3.41E-03
**PAPOLB**	-3.94	1.67E-03	3.41E-03
**RP11-127I20.7**	-4.18	1.67E-03	3.41E-03

**NBPF1**	-0.70	1.67E-03	3.41E-03
**VPS26B**	-0.32	1.67E-03	3.41E-03
**PYHIN1**	1.80	1.67E-03	3.41E-03
**RP11-511I11.1**	-3.94	1.67E-03	3.41E-03
**PDK3**	-0.65	1.68E-03	3.42E-03
**CLCN6**	0.59	1.68E-03	3.43E-03
**VPS33A**	-0.36	1.68E-03	3.43E-03
**RP11-834C11.10**	-2.42	1.68E-03	3.44E-03
**RP4-694A7.2**	-4.05	1.69E-03	3.44E-03
**KLHL2P1**	1.66	1.69E-03	3.44E-03
**TXNIP**	0.61	1.69E-03	3.44E-03
**HLA-DQB1-AS1**	-4.77	1.69E-03	3.44E-03
**TMEM241**	0.52	1.69E-03	3.44E-03
**UPK3A**	4.41	1.69E-03	3.44E-03
**RP3-325F22.3**	-1.73	1.69E-03	3.44E-03
**MMP10**	4.08	1.69E-03	3.44E-03
**SCHLAP1**	-3.75	1.69E-03	3.44E-03
**GPSM1**	0.57	1.69E-03	3.44E-03
**MTND4P26**	-2.78	1.69E-03	3.45E-03
**KLHDC7A**	3.72	1.69E-03	3.45E-03
**HNRNPU**	-0.36	1.69E-03	3.45E-03
**RP11-713P17.3**	-0.87	1.69E-03	3.45E-03
**RP3-408N23.4**	-2.66	1.69E-03	3.45E-03
**PRKAR2A**	-0.38	1.70E-03	3.46E-03
**ADAR**	-0.35	1.70E-03	3.46E-03
**RP11-139E19.3**	-3.93	1.70E-03	3.46E-03
**CHRAC1**	0.43	1.70E-03	3.46E-03
**RP11-806L2.6**	-2.53	1.70E-03	3.46E-03
**C9orf84**	-1.23	1.70E-03	3.46E-03
**RP11-19E18.2**	-2.58	1.70E-03	3.46E-03
**SRGAP3-AS3**	-2.18	1.70E-03	3.47E-03
**RN7SL187P**	-3.54	1.71E-03	3.48E-03
**IDE**	-0.95	1.71E-03	3.48E-03
**AC004057.1**	1.24	1.71E-03	3.48E-03
**RP11-137L10.5**	-3.59	1.71E-03	3.48E-03
**RP11-493E12.1**	-3.56	1.71E-03	3.48E-03
**CTD-2171N6.1**	3.52	1.71E-03	3.49E-03
**PSG10P**	-3.99	1.71E-03	3.49E-03
**SLC26A4**	1.38	1.71E-03	3.49E-03
**AC093818.1**	-1.19	1.71E-03	3.49E-03
**ADAM20P1**	-1.94	1.72E-03	3.49E-03
**RP11-449H3.1**	-3.78	1.72E-03	3.50E-03
**CTD-2248H3.1**	-1.93	1.72E-03	3.50E-03
**TRMT2B**	-0.53	1.72E-03	3.50E-03
**LINC01167**	-3.95	1.72E-03	3.50E-03

**RP11-413H22.2**	-4.04	1.72E-03	3.50E-03
**ADAM17**	-0.34	1.72E-03	3.50E-03
**RP11-972P1.10**	-3.90	1.72E-03	3.50E-03
**RP11-346M5.1**	-4.03	1.72E-03	3.50E-03
**RXRG**	-1.04	1.72E-03	3.51E-03
**RP11-632K20.2**	-2.18	1.72E-03	3.51E-03
**HNRNPCP6**	-3.99	1.72E-03	3.51E-03
**RP11-831F12.2**	-3.91	1.73E-03	3.51E-03
**RP3-341D10.1**	-2.43	1.73E-03	3.51E-03
**FAM49B**	0.42	1.73E-03	3.51E-03
**SRGAP3**	-0.78	1.73E-03	3.51E-03
**ACVR1C**	1.95	1.73E-03	3.51E-03
**XLOC_008611**	-3.69	1.73E-03	3.51E-03
**GNB2L1**	0.41	1.73E-03	3.52E-03
**RPS3AP44**	-3.89	1.73E-03	3.52E-03
**ITFG1-AS1**	-2.48	1.73E-03	3.52E-03
**GPN3**	0.51	1.73E-03	3.52E-03
**G27476**	-2.57	1.73E-03	3.52E-03
**AC083900.1**	-2.02	1.73E-03	3.52E-03
**AC018890.6**	-1.92	1.73E-03	3.52E-03
**PDE6G**	1.77	1.73E-03	3.52E-03
**NOM1**	-0.39	1.74E-03	3.53E-03
**RP13-93L13.1**	-4.02	1.74E-03	3.54E-03
**KLHL8**	-0.32	1.74E-03	3.54E-03
**NCS1**	-0.53	1.74E-03	3.55E-03
**RP11-848P1.3**	1.14	1.75E-03	3.55E-03
**ABCB7**	-0.30	1.75E-03	3.55E-03
**TRAJ38**	-4.06	1.75E-03	3.55E-03
**PDE1C**	1.48	1.75E-03	3.56E-03
**CSF3R**	1.75	1.75E-03	3.56E-03
**RP11-26H16.1**	-3.90	1.75E-03	3.56E-03
**RP3-395P12.2**	-3.94	1.75E-03	3.56E-03
**RP13-1032I1.7**	0.74	1.75E-03	3.56E-03
**RN7SL370P**	-3.56	1.76E-03	3.56E-03
**SNORD41**	-3.71	1.76E-03	3.57E-03
**POU2AF1**	2.10	1.76E-03	3.57E-03
**RP11-62F24.1**	-4.25	1.76E-03	3.57E-03
**AC093267.1**	-4.02	1.76E-03	3.57E-03
**PDZRN3-AS1**	-3.71	1.76E-03	3.57E-03
**APOBEC2**	-1.27	1.76E-03	3.58E-03
**XLOC_010312**	-3.80	1.76E-03	3.58E-03
**NUP188**	-0.31	1.77E-03	3.58E-03
**RP11-32B5.1**	-1.63	1.77E-03	3.58E-03
**ACTG2**	1.44	1.77E-03	3.59E-03
**TMA7**	-0.74	1.77E-03	3.59E-03

**PPP1R26-AS1**	1.28	1.77E-03	3.60E-03
**NPHP3-ACAD11**	-3.47	1.78E-03	3.60E-03
**RP11-651K21.1**	-3.72	1.78E-03	3.61E-03
**ZBTB7B**	0.48	1.78E-03	3.61E-03
**MIR3927**	-3.84	1.78E-03	3.61E-03
**RP11-120J1.1**	-1.91	1.78E-03	3.61E-03
**RP11-732A21.2**	-3.84	1.78E-03	3.61E-03
**ALPK1**	-0.36	1.78E-03	3.61E-03
**C1orf115**	0.96	1.78E-03	3.61E-03
**RP11-345J18.2**	-2.16	1.78E-03	3.62E-03
**CTD-2270P14.2**	-1.60	1.78E-03	3.62E-03
**MYO15B**	1.18	1.78E-03	3.62E-03
**CDS1**	-0.61	1.78E-03	3.62E-03
**RP11-460N11.2**	-3.11	1.79E-03	3.62E-03
**ZNF486**	-0.65	1.79E-03	3.63E-03
**RP11-730G20.2**	-2.30	1.79E-03	3.63E-03
**ASB15**	-3.99	1.79E-03	3.64E-03
**PGPEP1**	0.88	1.79E-03	3.64E-03
**XLOC_002554**	1.22	1.79E-03	3.64E-03
**RP11-219E7.2**	-4.23	1.80E-03	3.64E-03
**TMEM150B**	2.36	1.80E-03	3.64E-03
**AC107021.1**	-3.56	1.80E-03	3.64E-03
**CD53**	1.27	1.80E-03	3.65E-03
**SLC12A8**	1.11	1.80E-03	3.65E-03
**RPL21P3**	-2.98	1.80E-03	3.65E-03
**RP11-473O4.3**	-2.08	1.80E-03	3.65E-03
**RP11-731K22.1**	-3.98	1.80E-03	3.65E-03
**OR10A3**	-3.50	1.80E-03	3.65E-03
**AC100830.5**	-2.13	1.80E-03	3.66E-03
**NEURL3**	2.72	1.81E-03	3.66E-03
**RP11-676J12.9**	-1.57	1.81E-03	3.66E-03
**RNU1-28P**	-3.28	1.81E-03	3.66E-03
**USP6**	-2.10	1.81E-03	3.67E-03
**RP11-571I18.2**	-3.88	1.81E-03	3.68E-03
**PCBP2-OT1**	-2.86	1.82E-03	3.68E-03
**AKNA**	0.75	1.82E-03	3.68E-03
**RP11-490E15.2**	-3.86	1.82E-03	3.68E-03
**ABCC5-AS1**	-2.68	1.82E-03	3.69E-03
**IP6K2**	0.40	1.82E-03	3.69E-03
**XPR1**	0.53	1.82E-03	3.69E-03
**MIR3939**	-3.58	1.82E-03	3.69E-03
**PHF19**	0.54	1.82E-03	3.69E-03
**NUTM2D**	1.08	1.83E-03	3.69E-03
**CTD-2006K23.1**	3.19	1.83E-03	3.70E-03
**AC009237.10**	-3.85	1.83E-03	3.70E-03

**RP11-42F12.1**	-3.87	1.83E-03	3.70E-03
**YRDCP2**	-3.65	1.83E-03	3.70E-03
**ELAVL3**	3.58	1.83E-03	3.70E-03
**MYO1D**	0.32	1.83E-03	3.70E-03
**ZBTB10**	-0.72	1.83E-03	3.70E-03
**RP11-574K11.27**	-3.24	1.83E-03	3.71E-03
**RP11-543H12.1**	-3.97	1.83E-03	3.71E-03
**MORF4L2P1**	-4.03	1.83E-03	3.71E-03
**RP11-659E9.4**	-2.06	1.83E-03	3.71E-03
**RP11-276H1.2**	-3.52	1.84E-03	3.71E-03
**ESYT1**	0.34	1.84E-03	3.71E-03
**CTD-2595P9.2**	-4.12	1.84E-03	3.71E-03
**G5146**	-0.92	1.84E-03	3.72E-03
**LINC00704**	2.15	1.84E-03	3.73E-03
**AC069154.4**	-3.92	1.85E-03	3.73E-03
**RP11-413E1.2**	-3.56	1.85E-03	3.73E-03
**LINC01004**	-1.15	1.85E-03	3.73E-03
**CARD10**	0.92	1.85E-03	3.73E-03
**RN7SL864P**	-3.85	1.85E-03	3.74E-03
**METTL18**	-0.54	1.85E-03	3.74E-03
**RP1-37M3.8**	-2.97	1.85E-03	3.74E-03
**LLNLR-285B5.1**	-3.04	1.85E-03	3.75E-03
**RP11-304F15.6**	-3.76	1.85E-03	3.75E-03
**SASH1**	-0.47	1.85E-03	3.75E-03
**EIF2S1**	-0.44	1.85E-03	3.75E-03
**AC007773.1**	-3.73	1.86E-03	3.76E-03
**HSPA1B**	0.61	1.86E-03	3.76E-03
**SLC2A2**	-3.94	1.86E-03	3.76E-03
**AC016730.1**	-3.49	1.86E-03	3.76E-03
**MTND5P11**	-3.25	1.87E-03	3.77E-03
**RP11-121C2.3**	-3.91	1.87E-03	3.77E-03
**CARNS1**	-1.20	1.87E-03	3.77E-03
**PDGFD**	1.47	1.87E-03	3.77E-03
**RP5-1092L12.2**	-4.21	1.87E-03	3.77E-03
**KIF21B**	0.94	1.87E-03	3.77E-03
**RP11-314N13.10**	-1.33	1.87E-03	3.78E-03
**HOXC5**	-2.28	1.87E-03	3.78E-03
**COMMD3**	-0.47	1.88E-03	3.79E-03
**C1orf220**	-2.26	1.88E-03	3.80E-03
**RP1-142L7.9**	-3.11	1.88E-03	3.80E-03
**LINC01585**	-2.58	1.88E-03	3.80E-03
**RP11-680G24.6**	-1.83	1.88E-03	3.80E-03
**LINC00456**	-3.95	1.89E-03	3.81E-03
**EIF4A1P8**	-3.29	1.89E-03	3.81E-03
**RNU4-21P**	-4.08	1.89E-03	3.81E-03

**RP11-345K20.2**	-1.75	1.89E-03	3.81E-03
**XLOC_009342**	-2.21	1.89E-03	3.81E-03
**ACRV1**	1.64	1.89E-03	3.82E-03
**RPS29P22**	-3.89	1.89E-03	3.82E-03
**IRF8**	1.19	1.89E-03	3.82E-03
**LINC00886**	-1.16	1.90E-03	3.83E-03
**CTD-2014D20.1**	-2.17	1.90E-03	3.83E-03
**CTD-2589H19.6**	-1.48	1.90E-03	3.83E-03
**POLR2M**	-0.51	1.90E-03	3.83E-03
**RP11-7F17.3**	1.63	1.90E-03	3.83E-03
**DNAJC19P6**	-4.07	1.90E-03	3.83E-03
**RNU6-1278P**	-4.03	1.90E-03	3.84E-03
**RN7SL442P**	-4.08	1.90E-03	3.84E-03
**RP4-760C5.3**	-3.04	1.91E-03	3.84E-03
**RNU6-1102P**	-4.42	1.91E-03	3.85E-03
**GAPDHP32**	-4.19	1.91E-03	3.85E-03
**RN7SL68P**	-1.90	1.91E-03	3.85E-03
**CTC-329D1.2**	0.95	1.91E-03	3.85E-03
**YES1**	-0.41	1.91E-03	3.85E-03
**RP11-766F14.2**	-3.68	1.92E-03	3.86E-03
**XLOC_011383**	-4.08	1.92E-03	3.87E-03
**CYBRD1**	-0.90	1.92E-03	3.87E-03
**AC104655.3**	-1.35	1.92E-03	3.87E-03
**F8**	0.66	1.92E-03	3.87E-03
**RWDD4P1**	-2.29	1.92E-03	3.87E-03
**GOSR1**	-0.27	1.93E-03	3.88E-03
**DKC1**	0.43	1.93E-03	3.88E-03
**AKAP2**	-2.34	1.93E-03	3.88E-03
**RP11-369F10.2**	-4.05	1.93E-03	3.89E-03
**RP11-356I2.4**	-0.71	1.93E-03	3.89E-03
**CTC-359D24.3**	-0.91	1.93E-03	3.89E-03
**PCAT2**	-3.86	1.93E-03	3.90E-03
**PIGCP1**	-0.68	1.94E-03	3.90E-03
**FBXO34**	-0.31	1.94E-03	3.90E-03
**GCC2-AS1**	2.17	1.94E-03	3.90E-03
**RP11-79M19.2**	-3.65	1.94E-03	3.90E-03
**OLIG3**	-4.18	1.94E-03	3.90E-03
**TLR8**	1.62	1.95E-03	3.92E-03
**PNPLA8**	-0.47	1.95E-03	3.92E-03
**RP11-482L11.1**	-4.06	1.95E-03	3.92E-03
**RP11-122C5.3**	-3.97	1.95E-03	3.92E-03
**GLYATL1P2**	-3.82	1.95E-03	3.92E-03
**SRP9P1**	-1.31	1.95E-03	3.92E-03
**RP11-226M10.3**	-1.81	1.95E-03	3.92E-03
**MED20**	0.40	1.95E-03	3.93E-03

**TMTC4**	-0.42	1.95E-03	3.93E-03
**RNA5SP165**	-3.82	1.95E-03	3.93E-03
**IL36RN**	0.96	1.95E-03	3.93E-03
**HINT1**	0.49	1.95E-03	3.93E-03
**SNORD114-1**	-3.94	1.96E-03	3.94E-03
**PCDHGA4**	-0.93	1.96E-03	3.94E-03
**TRIM35**	-0.60	1.96E-03	3.94E-03
**WDR70**	0.33	1.96E-03	3.94E-03
**SLC12A5**	-1.38	1.96E-03	3.94E-03
**G22304**	-4.25	1.96E-03	3.94E-03
**ABCB9**	0.64	1.96E-03	3.94E-03
**FAM83C**	0.73	1.96E-03	3.95E-03
**POLR1A**	-0.43	1.97E-03	3.95E-03
**GOT2P2**	-3.94	1.97E-03	3.95E-03
**TNFRSF10C**	1.70	1.97E-03	3.95E-03
**C11orf71**	-0.52	1.97E-03	3.95E-03
**CDC42P4**	-2.56	1.97E-03	3.96E-03
**AC090625.1**	-3.99	1.97E-03	3.96E-03
**PXYLP1**	-0.46	1.97E-03	3.96E-03
**JPH4**	1.25	1.97E-03	3.96E-03
**CPB1**	3.36	1.97E-03	3.97E-03
**ATP2A3**	1.95	1.97E-03	3.97E-03
**RNU6-377P**	-3.64	1.98E-03	3.97E-03
**PELO**	0.36	1.98E-03	3.97E-03
**PRR13P1**	-2.38	1.98E-03	3.97E-03
**RP11-1236K1.8**	-3.90	1.98E-03	3.98E-03
**RNU4-47P**	1.97	1.98E-03	3.98E-03
**ADAMTS14**	1.34	1.98E-03	3.98E-03
**AC016712.2**	-4.07	1.98E-03	3.98E-03
**LINC00954**	-1.46	1.98E-03	3.98E-03
**RP3-497J21.1**	-3.97	1.99E-03	3.99E-03
**NMUR1**	1.73	1.99E-03	3.99E-03
**RNU6-189P**	-3.85	1.99E-03	4.00E-03
**RNU6-1043P**	-3.68	1.99E-03	4.00E-03
**CTD-3128G10.6**	-1.72	1.99E-03	4.00E-03
**TBC1D9**	0.82	1.99E-03	4.00E-03
**MIR23A**	-1.44	2.00E-03	4.01E-03
**MIR4442**	1.60	2.00E-03	4.01E-03
**RP11-169K17.4**	-3.05	2.00E-03	4.02E-03
**UBE2V1P1**	-2.26	2.00E-03	4.02E-03
**LIAS**	0.47	2.00E-03	4.02E-03
**PROCA1**	0.98	2.00E-03	4.02E-03
**RND3**	0.58	2.01E-03	4.02E-03
**RELN**	-1.33	2.01E-03	4.03E-03
**FCHSD1**	0.92	2.01E-03	4.04E-03

**G25047**	-3.86	2.01E-03	4.04E-03
**RNU6-1209P**	-3.69	2.01E-03	4.04E-03
**CDC42EP3**	0.68	2.01E-03	4.04E-03
**FOXR1**	-3.94	2.01E-03	4.04E-03
**RP11-129B9.1**	-3.51	2.02E-03	4.04E-03
**BNIP3P22**	-3.80	2.02E-03	4.04E-03
**SLC35A5**	-0.44	2.02E-03	4.05E-03
**ZAR1**	-3.73	2.02E-03	4.05E-03
**RP11-379F4.8**	-3.16	2.02E-03	4.05E-03
**MIR3942**	-3.62	2.02E-03	4.06E-03
**IL1F10**	-1.04	2.02E-03	4.06E-03
**GZMH**	1.85	2.03E-03	4.06E-03
**RP11-295I5.3**	-1.26	2.03E-03	4.06E-03
**POU6F2**	3.18	2.03E-03	4.07E-03
**HIP1R**	0.50	2.03E-03	4.07E-03
**NMD3**	-0.33	2.03E-03	4.07E-03
**WWTR1-AS1**	1.46	2.03E-03	4.08E-03
**SNORD96B**	-3.88	2.03E-03	4.08E-03
**CTD-2553L13.4**	-3.90	2.04E-03	4.08E-03
**HERC2P10**	-2.39	2.04E-03	4.08E-03
**RN7SKP198**	-3.86	2.04E-03	4.08E-03
**RP11-356J5.13**	-2.71	2.04E-03	4.08E-03
**AC064836.3**	-3.68	2.04E-03	4.08E-03
**YWHAEP7**	-4.13	2.04E-03	4.09E-03
**LINC00314**	-3.98	2.04E-03	4.10E-03
**FAM89B**	-0.58	2.05E-03	4.10E-03
**AP000908.1**	-4.02	2.05E-03	4.11E-03
**CTB-51E13.1**	-3.82	2.05E-03	4.11E-03
**RPL26P19**	-1.06	2.05E-03	4.11E-03
**C2orf15**	-0.81	2.06E-03	4.12E-03
**RP11-94C24.13**	-3.51	2.06E-03	4.12E-03
**RP4-800G7.1**	-2.73	2.06E-03	4.12E-03
**REEP6**	1.18	2.06E-03	4.12E-03
**TRA2B**	-0.28	2.06E-03	4.12E-03
**AC011899.9**	2.04	2.06E-03	4.12E-03
**SMARCA5-AS1**	-3.02	2.06E-03	4.13E-03
**NDUFA13**	-1.02	2.06E-03	4.13E-03
**HNRNPK**	0.38	2.06E-03	4.13E-03
**GNG4**	2.13	2.06E-03	4.13E-03
**CITF22-92A6.2**	-1.94	2.06E-03	4.13E-03
**CTD-2003C8.2**	-1.92	2.07E-03	4.15E-03
**RP11-529H20.3**	-3.74	2.07E-03	4.15E-03
**FAM220A**	0.35	2.07E-03	4.15E-03
**AP3M1**	-0.35	2.07E-03	4.15E-03
**RN7SL382P**	-2.94	2.08E-03	4.15E-03

**DENR**	0.39	2.08E-03	4.15E-03
**KCNN3**	1.04	2.08E-03	4.16E-03
**RP11-384C4.2**	-1.73	2.08E-03	4.17E-03
**AC133528.2**	0.57	2.09E-03	4.17E-03
**XLOC_010725**	-2.44	2.09E-03	4.17E-03
**ORC1**	-0.61	2.09E-03	4.17E-03
**RP1-140C12.2**	-2.52	2.09E-03	4.17E-03
**LINC00998**	0.64	2.09E-03	4.17E-03
**RPL7AP66**	-1.84	2.09E-03	4.17E-03
**CDC40**	-0.35	2.09E-03	4.17E-03
**MRPL44**	0.41	2.09E-03	4.18E-03
**AC011431.2**	-3.83	2.09E-03	4.18E-03
**RNU6-786P**	-3.58	2.09E-03	4.19E-03
**RNU6-378P**	-4.05	2.09E-03	4.19E-03
**TDRD10**	1.24	2.10E-03	4.19E-03
**RP11-91P24.5**	-2.01	2.10E-03	4.19E-03
**RP11-108K3.5**	-4.05	2.10E-03	4.19E-03
**ARHGAP39**	0.71	2.10E-03	4.19E-03
**AC002542.2**	-3.84	2.10E-03	4.19E-03
**CTPS1**	0.53	2.10E-03	4.20E-03
**RP1-278E11.3**	-4.12	2.10E-03	4.20E-03
**XLOC_007053**	2.05	2.10E-03	4.20E-03
**PXT1**	-3.90	2.10E-03	4.20E-03
**RP11-64B16.4**	-1.70	2.10E-03	4.20E-03
**LINS1**	-0.34	2.10E-03	4.20E-03
**ZFAT**	-0.38	2.10E-03	4.20E-03
**AC017104.2**	-1.67	2.11E-03	4.21E-03
**RP11-53I6.1**	-4.05	2.11E-03	4.21E-03
**G19165**	-3.75	2.11E-03	4.21E-03
**GDA**	2.69	2.11E-03	4.22E-03
**KIAA0408**	-3.54	2.11E-03	4.22E-03
**CRYBB1**	2.53	2.11E-03	4.22E-03
**AC093627.10**	0.93	2.11E-03	4.22E-03
**RP11-248J23.6**	-1.91	2.12E-03	4.22E-03
**CTD-2376I20.1**	-3.82	2.12E-03	4.22E-03
**MRPL1**	0.44	2.12E-03	4.23E-03
**AL353671.1**	-3.46	2.12E-03	4.23E-03
**CHST9**	-1.80	2.12E-03	4.23E-03
**KIF3A**	-0.39	2.12E-03	4.23E-03
**KLK15**	-3.95	2.12E-03	4.23E-03
**DLX1**	1.93	2.12E-03	4.23E-03
**LPIN3**	0.87	2.12E-03	4.24E-03
**RPL29P11**	-1.13	2.13E-03	4.24E-03
**PPT2-EGFL8**	-2.28	2.13E-03	4.25E-03
**WDYHV1**	0.41	2.13E-03	4.25E-03

**HSDL1**	0.28	2.13E-03	4.25E-03
**RP5-1163L11.2**	-4.03	2.13E-03	4.25E-03
**RP11-424G14.1**	-1.39	2.13E-03	4.25E-03
**IFT74**	-0.36	2.14E-03	4.26E-03
**AC009473.1**	-1.82	2.14E-03	4.26E-03
**CTD-2530N21.5**	-3.32	2.14E-03	4.26E-03
**RP4-742C19.13**	-1.30	2.14E-03	4.26E-03
**TCF4-AS1**	-2.76	2.14E-03	4.27E-03
**CC2D1B**	0.31	2.14E-03	4.27E-03
**ADGRF1**	3.65	2.14E-03	4.27E-03
**AL591479.1**	0.88	2.14E-03	4.28E-03
**MIR4473**	-3.81	2.14E-03	4.28E-03
**LRFN1**	1.11	2.14E-03	4.28E-03
**MURC**	1.79	2.15E-03	4.28E-03
**PTPDC1**	-0.48	2.15E-03	4.28E-03
**RP11-342A23.2**	-3.33	2.15E-03	4.28E-03
**COLEC11**	2.58	2.15E-03	4.28E-03
**RP11-422P24.12**	1.38	2.16E-03	4.30E-03
**FICD**	-0.48	2.16E-03	4.30E-03
**GSDMA**	0.88	2.16E-03	4.30E-03
**DLGAP1-AS2**	1.31	2.16E-03	4.30E-03
**PLCB1-IT1**	-3.90	2.16E-03	4.30E-03
**ACTG1P18**	-4.06	2.16E-03	4.30E-03
**TRAJ18**	-3.74	2.16E-03	4.31E-03
**MIR4459**	-4.08	2.16E-03	4.31E-03
**ATP6V0A1**	-0.34	2.17E-03	4.31E-03
**S100B**	-0.82	2.17E-03	4.31E-03
**ZNF326**	-0.43	2.17E-03	4.32E-03
**CTD-2555O16.4**	-1.05	2.17E-03	4.32E-03
**RP11-576C12.1**	-1.88	2.17E-03	4.33E-03
**RP5-1165K10.2**	-1.76	2.17E-03	4.33E-03
**HDAC5**	0.45	2.17E-03	4.33E-03
**RNF111**	-0.32	2.18E-03	4.33E-03
**RP11-208G20.3**	-3.76	2.18E-03	4.33E-03
**BRSK2**	-1.24	2.18E-03	4.33E-03
**AP000487.5**	-1.14	2.18E-03	4.34E-03
**ZNF419**	-0.51	2.18E-03	4.35E-03
**ARMC4**	2.53	2.19E-03	4.35E-03
**MIR643**	-3.88	2.19E-03	4.35E-03
**ANKRD30A**	-3.57	2.19E-03	4.35E-03
**LINC01270**	2.19	2.19E-03	4.36E-03
**CCNL2P1**	-3.71	2.19E-03	4.36E-03
**NDUFA12**	0.55	2.19E-03	4.37E-03
**CTD-2587H24.10**	-2.71	2.20E-03	4.37E-03
**RP4-591C20.9**	0.63	2.20E-03	4.37E-03

**AC008269.2**	-2.60	2.20E-03	4.37E-03
**RP11-478C6.4**	-3.41	2.20E-03	4.38E-03
**TCTN2**	-0.52	2.20E-03	4.38E-03
**RP11-467P9.1**	-2.05	2.20E-03	4.38E-03
**G2591**	-2.04	2.21E-03	4.39E-03
**DGCR6**	0.77	2.21E-03	4.39E-03
**FCGR1C**	4.93	2.21E-03	4.39E-03
**RP1-249I4.2**	-2.66	2.21E-03	4.39E-03
**MIR562**	-3.72	2.21E-03	4.39E-03
**FOXM1**	0.70	2.21E-03	4.40E-03
**DPH1**	0.46	2.21E-03	4.40E-03
**DNAH9**	-0.80	2.21E-03	4.40E-03
**RNU6-425P**	-3.69	2.21E-03	4.40E-03
**RNU6-155P**	-3.83	2.21E-03	4.40E-03
**LRRC37BP1**	0.41	2.21E-03	4.40E-03
**TPT1**	0.54	2.21E-03	4.40E-03
**RNU6-553P**	-3.97	2.22E-03	4.40E-03
**LINC01589**	-1.66	2.22E-03	4.41E-03
**G5850**	-5.94	2.22E-03	4.42E-03
**RP11-316M21.6**	-2.05	2.22E-03	4.42E-03
**WFDC2**	1.78	2.22E-03	4.42E-03
**CNTNAP4**	4.07	2.22E-03	4.42E-03
**TRIM52**	-0.53	2.23E-03	4.42E-03
**XLOC_008050**	-3.71	2.23E-03	4.42E-03
**RN7SL503P**	-3.60	2.23E-03	4.42E-03
**G36466**	-3.50	2.23E-03	4.43E-03
**SLC25A19**	0.86	2.23E-03	4.43E-03
**SMIM19**	-0.49	2.23E-03	4.43E-03
**TRMT10A**	-0.71	2.23E-03	4.43E-03
**MATR3**	-0.38	2.23E-03	4.43E-03
**RP11-306O1.2**	-4.37	2.23E-03	4.44E-03
**AC093381.2**	-3.87	2.24E-03	4.44E-03
**SCAANT1**	-2.41	2.24E-03	4.44E-03
**RP11-459D22.1**	-1.44	2.24E-03	4.44E-03
**RP3-337H4.8**	-1.45	2.24E-03	4.44E-03
**TCF4**	0.58	2.24E-03	4.44E-03
**MIR1285-1**	-3.59	2.24E-03	4.44E-03
**AC004593.3**	-2.66	2.24E-03	4.45E-03
**GLP1R**	2.68	2.24E-03	4.45E-03
**RP11-468E2.11**	-0.83	2.24E-03	4.45E-03
**RN7SL168P**	-3.77	2.25E-03	4.46E-03
**STT3A**	0.37	2.25E-03	4.46E-03
**RP11-267M23.3**	-1.94	2.25E-03	4.46E-03
**GTF2IRD1**	0.42	2.25E-03	4.47E-03
**RORA-AS2**	-3.72	2.25E-03	4.47E-03

**RFTN1P1**	2.36	2.25E-03	4.47E-03
**RP5-1121H13.4**	-3.58	2.25E-03	4.47E-03
**AMD1P1**	-2.68	2.25E-03	4.47E-03
**LURAP1**	-0.85	2.26E-03	4.47E-03
**ZC3H7A**	0.33	2.26E-03	4.47E-03
**ZNF862**	0.47	2.26E-03	4.47E-03
**RP11-84C13.2**	-2.67	2.26E-03	4.48E-03
**RP11-923I11.6**	2.25	2.26E-03	4.49E-03
**RP11-386M24.3**	-3.66	2.27E-03	4.50E-03
**ADRA2C**	1.33	2.27E-03	4.50E-03
**VSTM5**	0.95	2.27E-03	4.50E-03
**PTN**	1.19	2.27E-03	4.50E-03
**RPL7AP4**	-4.00	2.27E-03	4.51E-03
**AL365202.1**	-3.78	2.27E-03	4.51E-03
**IGSF3**	0.48	2.27E-03	4.51E-03
**UTS2B**	-1.84	2.28E-03	4.51E-03
**AC090616.2**	-1.39	2.28E-03	4.52E-03
**PSMG3-AS1**	0.94	2.28E-03	4.52E-03
**RP11-1415C14.3**	-1.03	2.28E-03	4.52E-03
**SCAPER**	-0.43	2.28E-03	4.52E-03
**RP11-480N24.4**	-3.15	2.28E-03	4.52E-03
**MFSD2A**	-0.74	2.28E-03	4.52E-03
**HSD3B2**	-3.90	2.29E-03	4.53E-03
**CAST**	-0.30	2.29E-03	4.54E-03
**RP11-487I5.4**	-2.84	2.29E-03	4.54E-03
**G39251**	-1.57	2.29E-03	4.54E-03
**MIR3143**	-3.89	2.30E-03	4.55E-03
**EXT1**	0.39	2.30E-03	4.55E-03
**CERS1**	2.33	2.30E-03	4.55E-03
**RP11-196B3.3**	-3.60	2.30E-03	4.56E-03
**CMTM8**	0.96	2.30E-03	4.56E-03
**AC004549.6**	-3.71	2.30E-03	4.56E-03
**RP11-417J8.3**	-3.19	2.31E-03	4.56E-03
**SCD5**	-1.01	2.31E-03	4.57E-03
**HCN3**	0.85	2.31E-03	4.58E-03
**C11orf49**	-0.44	2.31E-03	4.58E-03
**SNHG1**	-0.73	2.32E-03	4.59E-03
**WASH1**	1.00	2.32E-03	4.59E-03
**SLIT2-IT1**	-3.75	2.32E-03	4.59E-03
**MPND**	0.57	2.32E-03	4.59E-03
**RNU6-1141P**	-3.70	2.32E-03	4.60E-03
**RP11-556O9.2**	-1.91	2.32E-03	4.60E-03
**RPL19P14**	-4.01	2.33E-03	4.60E-03
**MAP2K6**	-0.59	2.33E-03	4.60E-03
**RP11-9L18.3**	-3.31	2.33E-03	4.60E-03

**RP11-670E13.3**	-3.96	2.33E-03	4.60E-03
**CTD-2536I1.2**	1.83	2.33E-03	4.61E-03
**LST1**	1.02	2.33E-03	4.61E-03
**ADAMTS3**	1.10	2.33E-03	4.61E-03
**GOLPH3**	0.33	2.33E-03	4.61E-03
**TAF9P3**	-2.22	2.33E-03	4.62E-03
**AC004540.4**	2.08	2.34E-03	4.62E-03
**PALM3**	1.53	2.34E-03	4.62E-03
**XLOC_014046**	-1.28	2.34E-03	4.62E-03
**RNA5SP210**	-3.65	2.34E-03	4.62E-03
**SLC30A1**	-0.56	2.34E-03	4.63E-03
**RNU6-1185P**	-4.04	2.34E-03	4.63E-03
**CCBE1**	-1.25	2.34E-03	4.63E-03
**PID1**	-1.06	2.35E-03	4.64E-03
**CCDC65**	-0.81	2.35E-03	4.64E-03
**RNU6-1034P**	-4.45	2.35E-03	4.64E-03
**ARHGEF10**	-0.54	2.35E-03	4.65E-03
**AC104654.2**	4.60	2.35E-03	4.65E-03
**RBMXP4**	1.26	2.36E-03	4.66E-03
**TUBBP5**	2.13	2.36E-03	4.66E-03
**RN7SL683P**	-3.76	2.36E-03	4.66E-03
**RP11-142O6.1**	-1.98	2.36E-03	4.66E-03
**FLJ33360**	-2.70	2.36E-03	4.67E-03
**INSM1**	-1.72	2.37E-03	4.67E-03
**TULP1**	-0.86	2.37E-03	4.68E-03
**FLJ31356**	-0.96	2.37E-03	4.68E-03
**AC091814.2**	-3.76	2.37E-03	4.68E-03
**C10orf11**	1.33	2.37E-03	4.68E-03
**RP11-568J23.4**	-3.83	2.37E-03	4.69E-03
**AC114812.8**	-2.92	2.37E-03	4.69E-03
**RP11-136H19.1**	-2.23	2.38E-03	4.69E-03
**SPTA1**	2.11	2.38E-03	4.70E-03
**RNU6-762P**	-3.59	2.38E-03	4.70E-03
**IGKV3-20**	3.66	2.38E-03	4.71E-03
**TTC22**	0.63	2.38E-03	4.71E-03
**EPHB6**	-0.81	2.39E-03	4.71E-03
**SPAG5**	-0.48	2.39E-03	4.71E-03
**RP11-27G14.4**	-0.95	2.39E-03	4.71E-03
**RP4-566D2.1**	-4.18	2.39E-03	4.71E-03
**DANCR**	0.52	2.39E-03	4.71E-03
**AC133644.2**	2.58	2.39E-03	4.73E-03
**RP11-680F20.5**	-2.83	2.40E-03	4.73E-03
**NAGA**	0.55	2.40E-03	4.73E-03
**ALOX12B**	0.87	2.40E-03	4.73E-03
**RPP14**	-0.34	2.40E-03	4.73E-03

**UBL3**	-0.68	2.40E-03	4.73E-03
**TIMM9**	0.38	2.40E-03	4.74E-03
**RNU2-68P**	3.05	2.40E-03	4.74E-03
**AC093166.4**	-3.77	2.40E-03	4.74E-03
**AC005330.2**	3.20	2.40E-03	4.74E-03
**FAM25BP**	-1.81	2.40E-03	4.74E-03
**RP11-118M9.3**	-1.77	2.41E-03	4.74E-03
**CLEC4D**	4.04	2.41E-03	4.75E-03
**TACR1**	0.82	2.41E-03	4.75E-03
**MRPS18B**	0.38	2.41E-03	4.75E-03
**bP-2171C21.6**	2.10	2.41E-03	4.75E-03
**ZNF462**	-0.53	2.41E-03	4.75E-03
**CNBP**	0.49	2.41E-03	4.75E-03
**TRIM22**	0.85	2.41E-03	4.75E-03
**MED18**	0.47	2.42E-03	4.76E-03
**RP3-449O17.1**	0.98	2.42E-03	4.76E-03
**ABHD10**	-0.45	2.42E-03	4.76E-03
**RP11-230L22.4**	-3.70	2.42E-03	4.77E-03
**PRR11**	0.78	2.42E-03	4.78E-03
**RP11-481A20.11**	1.70	2.42E-03	4.78E-03
**RP11-4L24.4**	-1.03	2.43E-03	4.78E-03
**HMG20A**	-0.27	2.43E-03	4.78E-03
**HSPBAP1**	-0.43	2.43E-03	4.78E-03
**IPO9**	-0.26	2.43E-03	4.78E-03
**CENPW**	0.76	2.43E-03	4.78E-03
**RNVU1-6**	-2.89	2.43E-03	4.79E-03
**CTD-2562J15.4**	-3.38	2.43E-03	4.79E-03
**MIR4637**	-3.91	2.43E-03	4.79E-03
**HULC**	-3.52	2.43E-03	4.79E-03
**DNAI1**	-3.52	2.44E-03	4.80E-03
**RP11-277P12.20**	1.18	2.44E-03	4.80E-03
**KRT18**	1.85	2.44E-03	4.80E-03
**UBE3AP2**	-4.18	2.44E-03	4.81E-03
**GCC2**	-0.42	2.44E-03	4.81E-03
**HYKK**	-0.62	2.45E-03	4.82E-03
**RN7SL55P**	-3.66	2.45E-03	4.82E-03
**RP11-2E11.6**	-3.94	2.45E-03	4.82E-03
**AC079922.3**	1.63	2.45E-03	4.82E-03
**AC105398.3**	-2.03	2.45E-03	4.82E-03
**AL163193.1**	-3.76	2.45E-03	4.83E-03
**DSCAS**	-1.77	2.45E-03	4.83E-03
**RP11-694I15.7**	-0.88	2.46E-03	4.83E-03
**RP11-390F4.3**	-1.29	2.46E-03	4.84E-03
**RP11-365F18.6**	-3.51	2.46E-03	4.84E-03
**CPT1C**	0.58	2.46E-03	4.84E-03

**PAPPA2**	-1.67	2.46E-03	4.85E-03
**SIMC1**	-0.42	2.47E-03	4.85E-03
**BCL7C**	0.46	2.47E-03	4.85E-03
**MAGEF1**	0.43	2.47E-03	4.85E-03
**COPS7B**	0.53	2.47E-03	4.86E-03
**RP11-720N19.1**	-3.58	2.47E-03	4.86E-03
**KRT72**	-1.71	2.47E-03	4.86E-03
**MAT1A**	-1.50	2.47E-03	4.86E-03
**NT5CP1**	-3.97	2.48E-03	4.87E-03
**C1orf229**	1.64	2.48E-03	4.87E-03
**LINC00116**	0.68	2.48E-03	4.87E-03
**WNT2**	2.05	2.48E-03	4.87E-03
**CEP70**	-0.53	2.48E-03	4.88E-03
**MIR1278**	-3.74	2.48E-03	4.88E-03
**RP13-890H12.2**	-1.23	2.48E-03	4.88E-03
**RNA5SP37**	-1.48	2.48E-03	4.88E-03
**RP11-301G23.1**	-3.79	2.48E-03	4.88E-03
**ABI3BP**	-1.13	2.49E-03	4.89E-03
**XLOC_010023**	-1.47	2.49E-03	4.89E-03
**EXTL3-AS1**	-1.30	2.49E-03	4.89E-03
**RP11-476B13.2**	-3.02	2.49E-03	4.89E-03
**XLOC_011448**	-1.09	2.49E-03	4.89E-03
**FABP9**	-7.39	2.49E-03	4.89E-03
**OR2T11**	-4.71	2.50E-03	4.91E-03
**RPS26P52**	-3.30	2.51E-03	4.92E-03
**RP5-966M1.5**	-3.60	2.51E-03	4.92E-03
**RP5-827C21.6**	-2.72	2.51E-03	4.92E-03
**B3GNTL1**	-0.63	2.51E-03	4.93E-03
**AC008937.3**	-3.82	2.51E-03	4.93E-03
**SLC45A4**	-0.71	2.51E-03	4.93E-03
**CTD-2013N17.1**	-3.37	2.51E-03	4.93E-03
**5-Sep**	0.54	2.51E-03	4.93E-03
**AP001476.2**	3.74	2.51E-03	4.93E-03
**ZADH2**	-0.28	2.52E-03	4.94E-03
**CIT**	-0.61	2.52E-03	4.95E-03
**ARNT2**	0.86	2.52E-03	4.95E-03
**GPC5**	-2.85	2.52E-03	4.95E-03
**XLOC_014083**	-2.50	2.52E-03	4.95E-03
**MIR942**	-3.77	2.52E-03	4.95E-03
**RP11-202G18.1**	-3.67	2.52E-03	4.95E-03
**RAB11FIP1P1**	-1.49	2.52E-03	4.95E-03
**XLOC_013531**	-3.31	2.52E-03	4.95E-03
**LSM3**	0.54	2.53E-03	4.96E-03
**LSG1**	-0.24	2.53E-03	4.96E-03
**RP11-407N17.4**	-1.68	2.53E-03	4.96E-03

**RP11-303E16.3**	-2.90	2.53E-03	4.96E-03
**RN7SL192P**	-2.72	2.53E-03	4.96E-03
**CDH18**	-2.11	2.53E-03	4.96E-03
**GBP5**	1.18	2.53E-03	4.97E-03
**RNU6-494P**	-4.06	2.54E-03	4.97E-03
**AC009411.2**	-3.83	2.54E-03	4.97E-03
**BOLL**	-3.09	2.54E-03	4.98E-03
**KIAA1841**	-0.57	2.54E-03	4.98E-03
**PEBP1**	0.47	2.54E-03	4.98E-03
**MIR4669**	-3.64	2.54E-03	4.98E-03
**RP11-141M1.1**	-3.55	2.54E-03	4.98E-03
**EID2B**	1.17	2.55E-03	4.99E-03
**LINC01558**	2.06	2.55E-03	4.99E-03
**AF131215.6**	-3.80	2.55E-03	4.99E-03
**EOMES**	1.74	2.55E-03	5.00E-03
**AC005251.3**	-3.68	2.56E-03	5.01E-03
**RP11-95I19.2**	-3.53	2.56E-03	5.02E-03
**RNU6-574P**	-2.20	2.56E-03	5.02E-03
**SZT2**	-0.51	2.56E-03	5.02E-03
**RP11-96C23.14**	-3.73	2.56E-03	5.02E-03
**FGF17**	-1.88	2.56E-03	5.02E-03
**PRDM6**	-1.26	2.56E-03	5.03E-03
**SLC35B1**	0.37	2.57E-03	5.03E-03
**UPF2**	0.36	2.57E-03	5.03E-03
**RP11-267J23.4**	-3.54	2.57E-03	5.03E-03
**ANKRD18CP**	-2.58	2.57E-03	5.03E-03
**MTAPP2**	-3.57	2.57E-03	5.04E-03
**DDX11L10**	-2.31	2.57E-03	5.04E-03
**MIR603**	-3.67	2.58E-03	5.05E-03
**RPS4XP6**	-3.16	2.58E-03	5.05E-03
**PPP2R2B**	-0.72	2.58E-03	5.05E-03
**RP11-36I17.2**	-2.62	2.58E-03	5.05E-03
**USP30**	-0.39	2.58E-03	5.05E-03
**AL844165.1**	-3.69	2.58E-03	5.05E-03
**RP1-167A14.2**	-1.48	2.58E-03	5.05E-03
**RP11-45F15.2**	-3.72	2.58E-03	5.06E-03
**FAP**	1.72	2.59E-03	5.06E-03
**WLS**	-0.36	2.59E-03	5.07E-03
**RP11-5N11.2**	-3.85	2.59E-03	5.07E-03
**LINC00582**	-3.94	2.59E-03	5.07E-03
**G31252**	-2.08	2.59E-03	5.08E-03
**RP11-12M5.4**	-3.37	2.60E-03	5.08E-03
**C20orf194**	0.51	2.60E-03	5.08E-03
**RP11-275I4.2**	2.99	2.60E-03	5.08E-03
**RP1-122O8.7**	-3.41	2.60E-03	5.09E-03

**MED28P7**	-3.75	2.60E-03	5.09E-03
**CSPG4P13**	2.77	2.60E-03	5.09E-03
**RP11-332M2.1**	0.57	2.60E-03	5.09E-03
**NPM1P41**	-4.07	2.60E-03	5.09E-03
**CNR1**	1.15	2.61E-03	5.10E-03
**RP11-64K12.9**	-3.56	2.61E-03	5.10E-03
**TTI2**	0.37	2.61E-03	5.10E-03
**RP11-108A14.1**	-2.39	2.61E-03	5.10E-03
**AC017028.7**	-3.76	2.61E-03	5.11E-03
**CCDC64**	-0.77	2.61E-03	5.11E-03
**RP1-40G4P.1**	-2.13	2.61E-03	5.11E-03
**OR7E130P**	-3.90	2.62E-03	5.11E-03
**DAW1**	-2.31	2.62E-03	5.12E-03
**FGFR1**	1.15	2.62E-03	5.12E-03
**ARMC2**	-0.60	2.62E-03	5.12E-03
**AC013468.1**	-3.71	2.62E-03	5.12E-03
**XLOC_001595**	-1.87	2.62E-03	5.12E-03
**RP11-153F10.2**	-1.51	2.62E-03	5.12E-03
**RP11-86H7.6**	-3.64	2.62E-03	5.13E-03
**SLC5A6**	0.58	2.62E-03	5.13E-03
**LRG1**	1.82	2.62E-03	5.13E-03
**ZDHHC20P4**	-2.60	2.63E-03	5.13E-03
**TMEM45B**	0.74	2.63E-03	5.13E-03
**DLGAP2**	-1.55	2.63E-03	5.13E-03
**XLOC_013958**	-3.89	2.63E-03	5.13E-03
**RP11-525A16.4**	-1.91	2.63E-03	5.13E-03
**AC079804.1**	-3.84	2.63E-03	5.14E-03
**CKAP2L**	-0.74	2.63E-03	5.14E-03
**RP11-295I5.4**	-3.54	2.63E-03	5.14E-03
**COL7A1**	0.81	2.63E-03	5.14E-03
**RP11-123C21.1**	-4.01	2.63E-03	5.14E-03
**ATPAF1**	0.31	2.63E-03	5.14E-03
**KRT17P3**	-4.25	2.63E-03	5.14E-03
**NPCDR1**	-3.73	2.63E-03	5.14E-03
**NTN5**	2.12	2.64E-03	5.14E-03
**CTD-2006H14.2**	-0.81	2.64E-03	5.15E-03
**IKBKE**	0.65	2.64E-03	5.15E-03
**RPS20P14**	-2.64	2.64E-03	5.16E-03
**CKAP2P1**	-3.77	2.64E-03	5.16E-03
**ACCSL**	-1.83	2.64E-03	5.16E-03
**PPHLN1**	0.21	2.64E-03	5.16E-03
**CR392039.1**	-4.25	2.64E-03	5.16E-03
**RP11-1113N2.4**	-3.70	2.65E-03	5.16E-03
**RP11-781A6.1**	-3.62	2.65E-03	5.17E-03
**MGC16275**	-0.96	2.65E-03	5.18E-03

**RNY3P6**	-3.79	2.66E-03	5.18E-03
**RP1-164F3.8**	-4.06	2.66E-03	5.19E-03
**HACD1**	0.75	2.66E-03	5.19E-03
**RP11-4K3__A.3**	-1.70	2.66E-03	5.20E-03
**AP001172.2**	-3.74	2.67E-03	5.20E-03
**SLC16A14**	-0.83	2.67E-03	5.20E-03
**CLN8**	-0.44	2.67E-03	5.21E-03
**RP11-644F5.11**	0.93	2.67E-03	5.21E-03
**LILRA2**	1.88	2.67E-03	5.21E-03
**RP11-68L18.1**	-1.98	2.67E-03	5.21E-03
**TUFT1**	-0.49	2.67E-03	5.21E-03
**GAPLINC**	1.76	2.68E-03	5.22E-03
**OSTC**	0.51	2.68E-03	5.23E-03
**RP11-157E16.1**	-3.81	2.68E-03	5.23E-03
**PDK1**	-0.64	2.68E-03	5.23E-03
**NR2C1**	-0.38	2.68E-03	5.23E-03
**PGAM1P4**	-3.96	2.68E-03	5.23E-03
**AC004862.6**	-3.86	2.69E-03	5.23E-03
**CTC-268N12.2**	-2.55	2.69E-03	5.23E-03
**RP11-481J2.4**	-1.10	2.69E-03	5.24E-03
**ANKRD36C**	-0.94	2.69E-03	5.24E-03
**RAET1L**	1.07	2.70E-03	5.26E-03
**CTD-2010I16.1**	-1.29	2.70E-03	5.26E-03
**CCDC150**	-0.93	2.70E-03	5.26E-03
**IL2RG**	1.00	2.70E-03	5.26E-03
**XXbac-B461K10.4**	-2.72	2.70E-03	5.27E-03
**MLC1**	-1.13	2.71E-03	5.27E-03
**AC006077.4**	-3.74	2.71E-03	5.27E-03
**KRT18P62**	-3.82	2.71E-03	5.27E-03
**METTL7B**	-3.27	2.71E-03	5.28E-03
**OIT3**	2.53	2.72E-03	5.30E-03
**RP11-1112G13.3**	-3.86	2.72E-03	5.30E-03
**MIR1539**	-2.02	2.72E-03	5.30E-03
**FAM204BP**	-2.89	2.72E-03	5.30E-03
**USP48**	-0.26	2.73E-03	5.31E-03
**GAS5**	0.53	2.73E-03	5.31E-03
**ZNF433**	-0.52	2.73E-03	5.31E-03
**RP11-72B4.2**	-2.46	2.73E-03	5.31E-03
**RP11-649E7.7**	-2.96	2.73E-03	5.31E-03
**FAF2**	-0.31	2.73E-03	5.31E-03
**RP11-1017G21.5**	0.78	2.73E-03	5.32E-03
**RP11-331F4.5**	-1.43	2.74E-03	5.32E-03
**SERBP1P3**	3.04	2.74E-03	5.32E-03
**RPL35AP31**	-3.78	2.74E-03	5.32E-03
**SNORA64**	-2.98	2.74E-03	5.33E-03

**BMS1P18**	3.64	2.74E-03	5.34E-03
**AC013267.1**	-3.71	2.75E-03	5.34E-03
**RP3-508I15.10**	-1.74	2.75E-03	5.34E-03
**TSPY26P**	0.94	2.75E-03	5.34E-03
**LACC1**	-0.62	2.75E-03	5.34E-03
**UBE2V2P3**	-2.43	2.75E-03	5.34E-03
**RP11-393I2.4**	-0.95	2.75E-03	5.35E-03
**MEI1**	1.40	2.75E-03	5.35E-03
**UQCC2**	0.51	2.75E-03	5.35E-03
**HAUS2**	-0.38	2.75E-03	5.35E-03
**CCR3**	-1.56	2.75E-03	5.36E-03
**RP11-488C13.1**	2.17	2.76E-03	5.36E-03
**SNRPGP1**	-3.67	2.76E-03	5.36E-03
**RP3-388M5.8**	-3.61	2.76E-03	5.36E-03
**XLOC_002956**	-3.19	2.76E-03	5.37E-03
**CBX3P4**	-3.75	2.76E-03	5.37E-03
**CDH7**	-4.25	2.76E-03	5.37E-03
**CTD-2283N19.1**	-1.97	2.76E-03	5.37E-03
**RPS2P28**	-4.11	2.77E-03	5.38E-03
**CCDC67**	-2.28	2.77E-03	5.38E-03
**B4GALT6**	-0.54	2.77E-03	5.38E-03
**RP11-145E17.2**	-1.05	2.77E-03	5.38E-03
**TOMM22P2**	-3.35	2.78E-03	5.39E-03
**AF131217.1**	-1.33	2.78E-03	5.39E-03
**RNU6-958P**	-3.77	2.78E-03	5.40E-03
**AC009994.2**	-3.55	2.78E-03	5.40E-03
**KIF18A**	-0.71	2.78E-03	5.41E-03
**SIK2**	-0.35	2.79E-03	5.41E-03
**G231**	1.60	2.79E-03	5.41E-03
**DNASE1L1**	0.83	2.79E-03	5.41E-03
**VN1R80P**	-2.25	2.79E-03	5.41E-03
**TNP1**	-3.89	2.79E-03	5.41E-03
**LAMA1**	1.10	2.79E-03	5.42E-03
**RP11-161M6.6**	-1.39	2.79E-03	5.42E-03
**XLOC_007860**	-2.68	2.79E-03	5.42E-03
**RP11-739N10.1**	-3.95	2.79E-03	5.42E-03
**ANXA2P1**	-3.38	2.80E-03	5.43E-03
**RNU6-807P**	-2.83	2.80E-03	5.43E-03
**ST13P15**	-1.92	2.80E-03	5.43E-03
**RN7SL390P**	-4.12	2.80E-03	5.43E-03
**NPM1**	0.55	2.80E-03	5.44E-03
**TIMM9P1**	-2.67	2.80E-03	5.44E-03
**NDRG2**	0.51	2.80E-03	5.44E-03
**RP11-370I10.6**	-3.78	2.80E-03	5.44E-03
**RP1-167G20.1**	-3.51	2.81E-03	5.44E-03

**SRPK3**	-1.23	2.81E-03	5.45E-03
**SLC22A5**	-0.70	2.81E-03	5.46E-03
**POPDC3**	3.62	2.82E-03	5.46E-03
**NAE1**	-0.38	2.82E-03	5.47E-03
**GRIN2B**	-1.81	2.82E-03	5.47E-03
**AC114812.5**	-3.57	2.82E-03	5.47E-03
**XLOC_005552**	3.97	2.82E-03	5.47E-03
**RP11-253E3.1**	-3.63	2.82E-03	5.47E-03
**RP11-834C11.4**	0.75	2.82E-03	5.47E-03
**DEPDC4**	-1.04	2.82E-03	5.47E-03
**MRPL37P1**	-1.33	2.83E-03	5.48E-03
**RP5-827C21.4**	1.24	2.83E-03	5.48E-03
**OAS2**	1.99	2.83E-03	5.49E-03
**TMC5**	-1.28	2.83E-03	5.49E-03
**AC017002.1**	3.66	2.83E-03	5.49E-03
**RP5-951N9.2**	-3.49	2.83E-03	5.49E-03
**PTOV1**	0.82	2.83E-03	5.49E-03
**MRPL40**	0.48	2.84E-03	5.51E-03
**MIR3134**	-3.79	2.84E-03	5.51E-03
**CDK19**	-0.33	2.85E-03	5.52E-03
**G34551**	-0.99	2.85E-03	5.53E-03
**PTK2B**	-0.52	2.85E-03	5.53E-03
**MRPS18AP1**	-3.61	2.86E-03	5.53E-03
**RP11-344B2.2**	-3.98	2.86E-03	5.53E-03
**TPPP2**	-3.38	2.86E-03	5.53E-03
**LINC01032**	-3.20	2.86E-03	5.54E-03
**TEX11**	3.46	2.86E-03	5.54E-03
**RHOF**	-1.02	2.86E-03	5.54E-03
**POC1B-GALNT4**	-2.95	2.86E-03	5.54E-03
**LA16c-395F10.1**	-3.68	2.86E-03	5.55E-03
**RPS7P11**	-0.97	2.87E-03	5.55E-03
**RPL21P8**	-4.07	2.87E-03	5.55E-03
**SFRP5**	-1.71	2.87E-03	5.56E-03
**XLOC_012351**	-2.70	2.87E-03	5.56E-03
**DDI1**	-3.48	2.87E-03	5.56E-03
**RP11-30K9.4**	-2.50	2.87E-03	5.56E-03
**INO80**	-0.29	2.87E-03	5.57E-03
**RNU6-101P**	-4.02	2.88E-03	5.57E-03
**RP11-317B17.3**	-3.47	2.88E-03	5.57E-03
**RP5-1024G6.7**	-2.22	2.88E-03	5.57E-03
**PKI55**	-0.68	2.88E-03	5.57E-03
**RTEL1**	-1.43	2.88E-03	5.58E-03
**XXbac-BPGBPG55C20.3**	-2.85	2.88E-03	5.58E-03
**LRSAM1**	0.43	2.89E-03	5.59E-03
**BRAP**	-0.31	2.89E-03	5.59E-03

**RP1-209B5.2**	-3.88	2.89E-03	5.59E-03
**CUL4A**	-0.26	2.90E-03	5.61E-03
**RP1-43O17.2**	2.45	2.91E-03	5.63E-03
**RP11-782C8.1**	-1.43	2.91E-03	5.63E-03
**RNU7-38P**	-3.81	2.91E-03	5.64E-03
**WWC2-AS1**	-3.89	2.91E-03	5.64E-03
**RP11-728F11.4**	-3.74	2.92E-03	5.64E-03
**CTD-2380F24.1**	-2.94	2.92E-03	5.66E-03
**MCMBP**	-0.36	2.92E-03	5.66E-03
**DLEU7**	-1.30	2.93E-03	5.66E-03
**RP11-1246C19.1**	0.79	2.93E-03	5.66E-03
**RP11-266K4.13**	-3.01	2.93E-03	5.67E-03
**MIR3153**	-3.54	2.93E-03	5.67E-03
**RP11-79H23.3**	-1.06	2.93E-03	5.67E-03
**JADE2**	0.38	2.93E-03	5.67E-03
**AC105053.3**	-3.58	2.94E-03	5.68E-03
**KBTBD2**	0.29	2.94E-03	5.68E-03
**CTD-3025N20.3**	-1.25	2.94E-03	5.69E-03
**RP11-94P14.1**	-3.72	2.94E-03	5.69E-03
**ABCB1**	1.20	2.94E-03	5.69E-03
**ZNF620**	-0.66	2.94E-03	5.69E-03
**RNU6-509P**	-3.13	2.95E-03	5.70E-03
**CTC-268N12.3**	-3.57	2.95E-03	5.70E-03
**AL355490.2**	-3.64	2.95E-03	5.70E-03
**SDR9C7**	0.70	2.95E-03	5.70E-03
**IP6K1**	0.32	2.95E-03	5.71E-03
**WDR62**	0.69	2.95E-03	5.71E-03
**RP11-787D18.1**	-3.34	2.96E-03	5.71E-03
**ADAMTS9-AS2**	-0.85	2.96E-03	5.71E-03
**AL121652.1**	-3.72	2.96E-03	5.71E-03
**CTD-2012K14.2**	-3.73	2.96E-03	5.71E-03
**CLDN8**	-1.11	2.96E-03	5.72E-03
**G8119**	-4.03	2.96E-03	5.72E-03
**KCNJ2**	-0.49	2.96E-03	5.72E-03
**ARHGAP19**	-0.46	2.96E-03	5.72E-03
**AREL1**	-0.31	2.97E-03	5.73E-03
**AL035422.1**	-3.74	2.97E-03	5.73E-03
**HTR7P1**	-0.66	2.97E-03	5.73E-03
**RP11-34P13.13**	-2.22	2.97E-03	5.73E-03
**DGKE**	-0.47	2.98E-03	5.75E-03
**RP13-672B3.5**	-3.49	2.98E-03	5.76E-03
**RP1-187B23.1**	-2.15	2.98E-03	5.76E-03
**RP11-809N15.2**	-3.53	2.99E-03	5.76E-03
**AL627309.1**	1.61	2.99E-03	5.77E-03
**RNU6-767P**	-3.73	2.99E-03	5.77E-03

**RP11-702L15.4**	-3.21	2.99E-03	5.77E-03
**GUCY1A2**	1.30	3.00E-03	5.79E-03
**NEURL1B**	-0.47	3.00E-03	5.79E-03
**DRC1**	3.04	3.01E-03	5.81E-03
**NHS-AS1**	-3.92	3.01E-03	5.81E-03
**SNRK-AS1**	-2.06	3.01E-03	5.81E-03
**RP11-70D24.2**	-2.42	3.02E-03	5.82E-03
**SIM1**	-1.25	3.02E-03	5.82E-03
**AC114763.1**	-2.60	3.02E-03	5.82E-03
**KRBOX1**	-1.36	3.02E-03	5.83E-03
**MIR4305**	-3.23	3.02E-03	5.83E-03
**RP11-805J14.3**	-3.63	3.02E-03	5.83E-03
**TRBV30**	-3.35	3.03E-03	5.84E-03
**RP11-224O19.5**	-3.68	3.03E-03	5.84E-03
**AL441988.1**	-3.86	3.03E-03	5.85E-03
**HPDL**	0.88	3.03E-03	5.85E-03
**TLCD1**	-0.75	3.03E-03	5.85E-03
**SOX8**	1.59	3.03E-03	5.85E-03
**PCNXL4**	-0.33	3.03E-03	5.85E-03
**MS4A4E**	-2.03	3.04E-03	5.85E-03
**RNA5SP118**	-2.97	3.04E-03	5.87E-03
**LRRC37B**	-0.45	3.05E-03	5.87E-03
**TAS2R10**	-3.67	3.05E-03	5.87E-03
**ZIM2**	-3.93	3.05E-03	5.88E-03
**RNU6-769P**	-3.73	3.06E-03	5.89E-03
**OTUD7A**	-0.67	3.06E-03	5.89E-03
**HEATR5A**	-0.38	3.07E-03	5.91E-03
**RP11-3L21.2**	-3.99	3.07E-03	5.91E-03
**AC022558.1**	-1.86	3.07E-03	5.91E-03
**RNU6-1214P**	-3.79	3.07E-03	5.91E-03
**AC121251.1**	-3.73	3.07E-03	5.91E-03
**AL031320.1**	-1.61	3.07E-03	5.92E-03
**AC093616.4**	1.54	3.07E-03	5.92E-03
**RN7SKP253**	-3.38	3.08E-03	5.92E-03
**EFCAB9**	-3.89	3.08E-03	5.93E-03
**SMAD3**	0.46	3.08E-03	5.94E-03
**PITPNM3**	-0.58	3.09E-03	5.95E-03
**AC005048.1**	-3.57	3.09E-03	5.95E-03
**FRY-AS1**	-1.91	3.09E-03	5.95E-03
**LA16c-352F7.1**	-3.61	3.09E-03	5.95E-03
**RP11-690P14.4**	-2.58	3.10E-03	5.96E-03
**LOC440461**	2.22	3.10E-03	5.96E-03
**RP11-662G23.1**	5.08	3.10E-03	5.96E-03
**LRRC3**	0.71	3.10E-03	5.96E-03
**XLOC_006312**	-3.97	3.10E-03	5.97E-03

**DNM1P51**	-1.91	3.10E-03	5.97E-03
**TLK1P1**	-3.73	3.11E-03	5.98E-03
**RP11-144O23.22**	-3.49	3.11E-03	5.98E-03
**RP11-834C11.11**	-2.09	3.11E-03	5.99E-03
**RUNDC1**	0.25	3.11E-03	5.99E-03
**OLA1P1**	-2.13	3.12E-03	5.99E-03
**CTD-2330K9.2**	-3.83	3.12E-03	6.00E-03
**RNU6-1016P**	-1.30	3.12E-03	6.00E-03
**IRX4**	0.62	3.12E-03	6.00E-03
**RP11-61L23.2**	1.31	3.12E-03	6.00E-03
**RP5-965F6.2**	-2.03	3.12E-03	6.00E-03
**PCDHGB4**	-0.80	3.13E-03	6.01E-03
**SLC28A2**	-2.01	3.13E-03	6.02E-03
**RP11-74K11.2**	-3.74	3.13E-03	6.02E-03
**KCNJ11**	-1.24	3.13E-03	6.02E-03
**RNU6-1062P**	-3.78	3.14E-03	6.03E-03
**HEATR3**	-0.39	3.14E-03	6.04E-03
**RNU6-531P**	-1.61	3.14E-03	6.04E-03
**CROT**	0.36	3.14E-03	6.04E-03
**IL17RD**	-0.57	3.15E-03	6.05E-03
**METTL7A**	0.68	3.15E-03	6.05E-03
**RP1-228P16.7**	-3.64	3.15E-03	6.05E-03
**RP11-642A1.2**	-2.09	3.15E-03	6.05E-03
**RP11-141C7.3**	-3.16	3.15E-03	6.05E-03
**TROAP**	0.74	3.15E-03	6.06E-03
**SNHG10**	-0.70	3.15E-03	6.06E-03
**HGF**	1.46	3.15E-03	6.06E-03
**NLK**	-0.31	3.15E-03	6.06E-03
**G3707**	-1.10	3.15E-03	6.06E-03
**CHAF1A**	0.35	3.16E-03	6.06E-03
**CTD-2623N2.5**	-3.42	3.16E-03	6.07E-03
**RP11-298D21.2**	-3.87	3.16E-03	6.07E-03
**TTC30A**	-0.50	3.16E-03	6.07E-03
**OR7E7P**	-1.47	3.16E-03	6.07E-03
**AC017028.1**	-3.41	3.16E-03	6.08E-03
**OFCC1**	-3.93	3.17E-03	6.08E-03
**SNORD64**	-3.98	3.17E-03	6.09E-03
**RP11-458D21.1**	-1.04	3.17E-03	6.09E-03
**RP11-111G23.1**	-1.40	3.17E-03	6.09E-03
**PAX9**	2.15	3.17E-03	6.10E-03
**POLG**	0.36	3.18E-03	6.10E-03
**KLC1**	-0.54	3.18E-03	6.10E-03
**AC074375.1**	-3.46	3.18E-03	6.10E-03
**RP11-575F12.3**	1.37	3.18E-03	6.10E-03
**CTC-370J7.1**	-3.45	3.18E-03	6.10E-03

**MIR26A1**	-3.90	3.18E-03	6.10E-03
**RPUSD2**	0.46	3.18E-03	6.11E-03
**RP11-274B21.12**	1.44	3.19E-03	6.11E-03
**RP11-365H22.2**	-1.71	3.19E-03	6.12E-03
**LAMA5**	0.60	3.19E-03	6.12E-03
**RP11-556H2.4**	-4.07	3.19E-03	6.12E-03
**RP11-147L13.12**	-0.73	3.19E-03	6.12E-03
**MIR3679**	-3.87	3.19E-03	6.12E-03
**OSGIN2**	-0.65	3.20E-03	6.13E-03
**RP11-770G2.2**	-1.97	3.20E-03	6.14E-03
**CMC1**	0.39	3.20E-03	6.14E-03
**CDC42P1**	-3.33	3.20E-03	6.14E-03
**SENCR**	1.43	3.21E-03	6.15E-03
**AC007557.4**	-3.81	3.21E-03	6.15E-03
**RPL36AP43**	-2.83	3.21E-03	6.15E-03
**DRD1**	2.29	3.21E-03	6.15E-03
**CCDC110**	-0.87	3.21E-03	6.15E-03
**NMU**	-0.80	3.21E-03	6.16E-03
**KCNH3**	-1.49	3.21E-03	6.16E-03
**G39823**	-1.47	3.21E-03	6.16E-03
**CYP4X1**	1.02	3.22E-03	6.17E-03
**BPIFB9P**	-1.99	3.22E-03	6.18E-03
**MRPL12**	0.87	3.22E-03	6.18E-03
**OVCH1-AS1**	-4.05	3.23E-03	6.19E-03
**HLA-DMB**	0.90	3.23E-03	6.20E-03
**HADHAP1**	-2.97	3.23E-03	6.20E-03
**RP11-353M9.1**	-3.45	3.24E-03	6.20E-03
**RN7SL752P**	-3.44	3.24E-03	6.20E-03
**ACSL4**	0.60	3.24E-03	6.20E-03
**OVCH1**	-2.77	3.24E-03	6.21E-03
**RP11-77A13.1**	-3.79	3.24E-03	6.21E-03
**CTD-2015G9.2**	2.51	3.24E-03	6.22E-03
**SPAG17**	1.43	3.25E-03	6.22E-03
**WI2-8325B5.1**	-3.93	3.25E-03	6.22E-03
**NFATC2IP**	0.32	3.25E-03	6.23E-03
**RP11-10J21.3**	-4.04	3.25E-03	6.23E-03
**MIR3978**	-3.55	3.26E-03	6.24E-03
**CCDC88A**	0.60	3.26E-03	6.24E-03
**RP11-123K19.1**	1.63	3.26E-03	6.24E-03
**SLC51A**	-0.60	3.26E-03	6.24E-03
**CR769776.1**	-4.15	3.26E-03	6.25E-03
**BTG1**	0.54	3.27E-03	6.26E-03
**AC005162.5**	1.81	3.27E-03	6.26E-03
**RP11-159F24.3**	-0.96	3.27E-03	6.26E-03
**RP4-728D4.3**	-1.85	3.27E-03	6.26E-03

**RP5-935K16.2**	-3.16	3.27E-03	6.26E-03
**CYB5R2**	0.41	3.27E-03	6.26E-03
**NFX1**	-0.29	3.27E-03	6.26E-03
**RN7SL481P**	-1.89	3.27E-03	6.26E-03
**RP11-500C11.3**	1.51	3.27E-03	6.27E-03
**MT1H**	1.30	3.28E-03	6.27E-03
**THTPA**	0.76	3.28E-03	6.27E-03
**RP4-816N1.6**	-2.80	3.28E-03	6.27E-03
**RP11-665E10.4**	-3.75	3.28E-03	6.27E-03
**AC069513.4**	-2.87	3.28E-03	6.27E-03
**AF224669.3**	-3.91	3.28E-03	6.28E-03
**CTD-3037G24.3**	-3.29	3.28E-03	6.28E-03
**ZMAT3**	-0.44	3.28E-03	6.28E-03
**RP11-43F13.3**	3.47	3.28E-03	6.28E-03
**RP11-214N15.5**	-3.08	3.28E-03	6.28E-03
**RP11-60A8.1**	-1.22	3.28E-03	6.28E-03
**G18341**	-3.72	3.29E-03	6.29E-03
**PARP16**	0.33	3.29E-03	6.29E-03
**AP002884.1**	-3.85	3.29E-03	6.29E-03
**RP5-1063M23.2**	-3.19	3.29E-03	6.29E-03
**EFNA4**	0.53	3.29E-03	6.30E-03
**C19orf73**	1.09	3.29E-03	6.30E-03
**AC009237.11**	-1.10	3.30E-03	6.30E-03
**RP11-787D18.2**	-4.04	3.30E-03	6.30E-03
**UFL1-AS1**	-2.42	3.30E-03	6.31E-03
**ENPP7P6**	-2.51	3.30E-03	6.31E-03
**THYN1**	0.48	3.30E-03	6.31E-03
**RP4-717I23.2**	-3.49	3.30E-03	6.31E-03
**RPL7AP3**	-3.51	3.30E-03	6.31E-03
**NARF-IT1**	-1.67	3.30E-03	6.31E-03
**HIAT1**	-0.41	3.31E-03	6.31E-03
**RNU6-795P**	-3.62	3.31E-03	6.32E-03
**TNNC1**	-0.87	3.31E-03	6.32E-03
**PRPF4**	0.30	3.31E-03	6.32E-03
**AC135178.1**	-0.89	3.31E-03	6.32E-03
**CT62**	1.40	3.31E-03	6.32E-03
**RP11-714G18.1**	-1.69	3.31E-03	6.32E-03
**CFAP221**	2.01	3.31E-03	6.32E-03
**RP1-234P15.4**	-1.09	3.31E-03	6.33E-03
**RP11-48G14.3**	-1.82	3.32E-03	6.33E-03
**AC093642.5**	-0.84	3.32E-03	6.33E-03
**PRR13P3**	-3.97	3.32E-03	6.34E-03
**LRRC14**	0.48	3.32E-03	6.34E-03
**RP1-142L7.8**	-3.23	3.33E-03	6.35E-03
**NADK2-AS1**	-1.83	3.33E-03	6.35E-03

**SLC47A1P1**	-3.33	3.33E-03	6.36E-03
**ART5**	2.33	3.33E-03	6.36E-03
**RN7SL608P**	-2.72	3.34E-03	6.37E-03
**TKFC**	0.33	3.34E-03	6.38E-03
**RYBP**	-0.31	3.34E-03	6.38E-03
**CHCHD4P2**	-4.18	3.35E-03	6.38E-03
**MTND1P8**	-3.86	3.35E-03	6.39E-03
**ARHGEF26-AS1**	-1.29	3.35E-03	6.39E-03
**MAGEA10**	-3.63	3.35E-03	6.39E-03
**RP11-734K23.9**	-2.06	3.35E-03	6.39E-03
**ZNF583**	-0.63	3.35E-03	6.39E-03
**RP11-1166P10.1**	0.94	3.35E-03	6.40E-03
**MIR211**	-3.68	3.35E-03	6.40E-03
**RP4-753P9.3**	-3.18	3.35E-03	6.40E-03
**DDX19B**	0.44	3.36E-03	6.41E-03
**PUS7**	-0.51	3.36E-03	6.41E-03
**G27121**	-1.18	3.36E-03	6.41E-03
**MAP4K1**	0.87	3.36E-03	6.41E-03
**AC005682.6**	1.06	3.36E-03	6.42E-03
**GRP**	3.36	3.37E-03	6.42E-03
**RPS20P31**	-3.62	3.37E-03	6.42E-03
**SNORA15**	-2.81	3.37E-03	6.42E-03
**RPL31P7**	-3.74	3.37E-03	6.42E-03
**NME7**	0.49	3.37E-03	6.43E-03
**IPO4**	-1.20	3.38E-03	6.44E-03
**NDUFA5P2**	-3.88	3.38E-03	6.44E-03
**CDCA4P2**	-3.81	3.38E-03	6.45E-03
**TMEM86B**	1.12	3.39E-03	6.46E-03
**EFHC1**	-0.65	3.39E-03	6.46E-03
**CTD-2006O16.2**	-4.18	3.39E-03	6.46E-03
**C17orf53**	0.64	3.39E-03	6.46E-03
**NAV2-AS4**	-3.51	3.39E-03	6.46E-03
**CTD-3216D2.5**	-3.60	3.40E-03	6.47E-03
**SUMO2P14**	-4.08	3.40E-03	6.47E-03
**LA16c-390E6.3**	-3.47	3.40E-03	6.48E-03
**AC008752.3**	-3.60	3.40E-03	6.48E-03
**MIR4753**	-3.92	3.41E-03	6.49E-03
**RP11-15N24.4**	-1.07	3.42E-03	6.50E-03
**LRR1**	-0.55	3.42E-03	6.50E-03
**AC012501.3**	-3.94	3.42E-03	6.50E-03
**RP13-152O15.5**	4.79	3.42E-03	6.51E-03
**RN7SL787P**	-3.85	3.42E-03	6.51E-03
**RN7SL834P**	-1.41	3.42E-03	6.51E-03
**NDUFC1**	0.47	3.42E-03	6.51E-03
**RPL9P2**	-2.62	3.42E-03	6.52E-03

**MTND5P25**	-3.55	3.43E-03	6.52E-03
**FKBP4P1**	-3.67	3.43E-03	6.52E-03
**RP11-314B1.2**	-3.99	3.43E-03	6.53E-03
**CFAP46**	-1.44	3.43E-03	6.53E-03
**RP11-438N5.4**	-3.87	3.43E-03	6.53E-03
**SMC6**	-0.34	3.43E-03	6.54E-03
**EGLN3-AS1**	-3.30	3.44E-03	6.54E-03
**RIOK2**	-0.38	3.44E-03	6.54E-03
**LATS2**	0.60	3.44E-03	6.55E-03
**RP3-512E2.2**	-3.44	3.45E-03	6.56E-03
**G38643**	-4.04	3.45E-03	6.56E-03
**AC005517.3**	-1.55	3.45E-03	6.56E-03
**BLCAP**	0.34	3.45E-03	6.56E-03
**ACTR3BP4**	-3.56	3.45E-03	6.56E-03
**TTC19**	-0.34	3.45E-03	6.57E-03
**SULT2B1**	0.57	3.46E-03	6.57E-03
**ADCY1**	0.73	3.46E-03	6.57E-03
**AC137932.4**	-1.18	3.46E-03	6.57E-03
**RP11-848G14.2**	3.07	3.46E-03	6.58E-03
**SRP19**	-0.35	3.46E-03	6.58E-03
**TMEM216**	0.51	3.46E-03	6.58E-03
**RP11-302M6.5**	-3.49	3.47E-03	6.59E-03
**RPL36AP13**	-3.53	3.47E-03	6.59E-03
**ANKHD1**	-1.01	3.47E-03	6.60E-03
**PROZ**	-3.32	3.47E-03	6.60E-03
**FTHL17**	-3.58	3.47E-03	6.60E-03
**ZNF322**	-0.56	3.47E-03	6.60E-03
**RNU6-750P**	-3.85	3.47E-03	6.60E-03
**RAI1**	0.77	3.48E-03	6.61E-03
**RNU4-59P**	-3.88	3.48E-03	6.61E-03
**CREBZF**	-0.49	3.48E-03	6.61E-03
**XLOC_009826**	-3.49	3.48E-03	6.62E-03
**NANOGP4**	-3.86	3.49E-03	6.63E-03
**TYW3**	-0.47	3.49E-03	6.63E-03
**RP11-254I22.3**	-3.65	3.49E-03	6.63E-03
**RP11-20B24.7**	1.83	3.49E-03	6.63E-03
**RP11-292F22.5**	-4.04	3.49E-03	6.64E-03
**ANKRD49**	-0.40	3.50E-03	6.64E-03
**G33755**	3.04	3.50E-03	6.64E-03
**RP11-90D11.1**	-3.86	3.50E-03	6.65E-03
**FIGF**	1.72	3.50E-03	6.65E-03
**ANKRD31**	1.11	3.50E-03	6.65E-03
**QRSL1**	-0.29	3.50E-03	6.65E-03
**RPL12**	0.50	3.50E-03	6.65E-03
**REN**	1.86	3.50E-03	6.65E-03

**RP11-252K23.2**	-1.82	3.51E-03	6.66E-03
**CETN3**	0.50	3.51E-03	6.66E-03
**AC011747.4**	-2.57	3.51E-03	6.66E-03
**RP1-128O3.5**	-3.97	3.51E-03	6.66E-03
**SUDS3P1**	-1.25	3.52E-03	6.67E-03
**TNNT3**	1.25	3.52E-03	6.67E-03
**EDN3**	-1.84	3.52E-03	6.68E-03
**PCDHB15**	0.71	3.52E-03	6.68E-03
**MIR4802**	-3.65	3.52E-03	6.68E-03
**CHKA**	0.80	3.52E-03	6.68E-03
**LCE1E**	1.72	3.53E-03	6.69E-03
**AC010731.4**	-4.13	3.53E-03	6.70E-03
**TSTD2**	-0.33	3.53E-03	6.70E-03
**RNU7-151P**	-3.79	3.53E-03	6.70E-03
**TMED7-TICAM2**	-3.69	3.54E-03	6.72E-03
**UBA52P5**	-3.50	3.54E-03	6.72E-03
**LUC7L**	0.72	3.54E-03	6.72E-03
**MIR3666**	-3.72	3.55E-03	6.73E-03
**LINC01189**	-4.14	3.55E-03	6.73E-03
**HNRNPF**	0.42	3.55E-03	6.74E-03
**WFDC3**	2.09	3.56E-03	6.74E-03
**TMEM266**	-0.73	3.56E-03	6.76E-03
**TMEM57**	0.41	3.57E-03	6.76E-03
**RP11-286N3.2**	-2.90	3.57E-03	6.76E-03
**RNU6-1079P**	-3.60	3.57E-03	6.76E-03
**XLOC_008357**	-2.16	3.57E-03	6.76E-03
**CTD-3126B10.4**	2.03	3.57E-03	6.77E-03
**MIR5693**	-3.70	3.58E-03	6.78E-03
**SAP30L-AS1**	-0.93	3.58E-03	6.78E-03
**RP11-386G11.10**	-2.22	3.58E-03	6.79E-03
**G33057**	1.80	3.58E-03	6.79E-03
**ZNF29P**	-2.88	3.59E-03	6.80E-03
**AC106053.1**	-3.55	3.59E-03	6.80E-03
**G7758**	-4.50	3.59E-03	6.81E-03
**LINC01140**	1.22	3.59E-03	6.81E-03
**ANO10**	0.37	3.60E-03	6.81E-03
**RBPJL**	-3.42	3.60E-03	6.82E-03
**BHLHE41**	1.04	3.60E-03	6.82E-03
**PTPN7**	1.11	3.60E-03	6.82E-03
**AC020900.2**	-3.91	3.60E-03	6.83E-03
**LINC01116**	1.12	3.61E-03	6.84E-03
**RP11-83M16.3**	-3.61	3.61E-03	6.84E-03
**G17634**	-6.15	3.61E-03	6.84E-03
**XLOC_008883**	-3.30	3.61E-03	6.84E-03
**FTLP8**	-3.79	3.62E-03	6.85E-03

**LGI2**	1.60	3.62E-03	6.85E-03
**GLS**	-0.52	3.62E-03	6.85E-03
**CDK10**	0.90	3.62E-03	6.86E-03
**EPHA1**	0.52	3.62E-03	6.86E-03
**AMER2**	-2.93	3.63E-03	6.86E-03
**RP11-438F14.10**	-4.53	3.63E-03	6.87E-03
**RNU6-1025P**	-3.65	3.63E-03	6.87E-03
**PDCD5**	0.40	3.64E-03	6.88E-03
**RP11-267N12.2**	-3.65	3.64E-03	6.89E-03
**RP11-540O11.7**	-2.61	3.64E-03	6.89E-03
**ZNF764**	0.46	3.64E-03	6.89E-03
**GLE1**	-0.25	3.64E-03	6.89E-03
**EFCAB14**	-0.30	3.64E-03	6.89E-03
**TRAJ10**	-4.07	3.64E-03	6.89E-03
**SMC3**	-0.39	3.65E-03	6.90E-03
**RP11-66H6.4**	-1.53	3.65E-03	6.90E-03
**RP11-798K23.5**	2.04	3.65E-03	6.91E-03
**L3MBTL3**	0.41	3.65E-03	6.91E-03
**NDUFAF4P4**	-3.42	3.65E-03	6.91E-03
**RPL13AP6**	-1.85	3.65E-03	6.91E-03
**RBM34**	-1.02	3.65E-03	6.91E-03
**SDAD1P4**	-4.03	3.66E-03	6.92E-03
**RNU6-306P**	-3.68	3.66E-03	6.92E-03
**CYP4A22**	-3.15	3.66E-03	6.93E-03
**RN7SKP185**	-3.79	3.67E-03	6.93E-03
**HIGD1AP14**	-3.49	3.67E-03	6.94E-03
**RP4-778K6.1**	-4.12	3.67E-03	6.94E-03
**CTC-429P9.1**	0.58	3.67E-03	6.94E-03
**TMSB4XP8**	0.83	3.67E-03	6.94E-03
**MADCAM1**	1.54	3.67E-03	6.94E-03
**RP11-445N18.7**	-2.09	3.67E-03	6.94E-03
**XLOC_005539**	-3.68	3.67E-03	6.94E-03
**AC005387.3**	1.98	3.68E-03	6.95E-03
**IGBP1P1**	-3.47	3.68E-03	6.95E-03
**GPX1P1**	2.11	3.68E-03	6.96E-03
**AP001628.6**	-3.17	3.68E-03	6.96E-03
**RP11-107M16.2**	-2.04	3.69E-03	6.97E-03
**MLF1**	-0.74	3.69E-03	6.97E-03
**BNIP3P29**	-4.15	3.69E-03	6.97E-03
**HDAC9**	-0.87	3.69E-03	6.97E-03
**AL035610.1**	-1.95	3.70E-03	6.98E-03
**ACTR3C**	-0.85	3.70E-03	6.98E-03
**RP11-715H19.2**	2.71	3.71E-03	7.01E-03
**RNF168**	-0.35	3.71E-03	7.01E-03
**TUSC7**	-3.99	3.71E-03	7.01E-03

**RP11-206L10.3**	-2.83	3.71E-03	7.01E-03
**RP11-879F14.2**	1.75	3.72E-03	7.02E-03
**RNU1-85P**	-2.88	3.72E-03	7.02E-03
**AARS2**	0.47	3.72E-03	7.02E-03
**RP11-346C16.1**	-3.53	3.72E-03	7.03E-03
**GLRA3**	-1.71	3.72E-03	7.03E-03
**KCNK2**	-1.46	3.73E-03	7.04E-03
**RP11-333E13.2**	-1.87	3.73E-03	7.04E-03
**MRPS31P4**	-1.28	3.73E-03	7.05E-03
**RPL5P30**	-1.36	3.74E-03	7.05E-03
**MYRFL**	-1.28	3.74E-03	7.05E-03
**RP11-155N3.4**	-3.59	3.74E-03	7.06E-03
**EPN2**	-0.35	3.74E-03	7.06E-03
**LIFR-AS1**	-0.94	3.74E-03	7.06E-03
**DUSP28**	-0.56	3.75E-03	7.07E-03
**OSGEP**	0.50	3.75E-03	7.07E-03
**AC008065.1**	-2.48	3.75E-03	7.07E-03
**UBE2E1**	0.42	3.75E-03	7.07E-03
**C5orf66**	-1.12	3.75E-03	7.07E-03
**G39634**	1.56	3.75E-03	7.08E-03
**SMIM8**	-0.54	3.76E-03	7.09E-03
**RP11-68I3.7**	-3.94	3.76E-03	7.09E-03
**RP11-875O11.1**	-1.96	3.76E-03	7.09E-03
**LINC00578**	2.33	3.76E-03	7.09E-03
**SAMM50**	0.37	3.76E-03	7.10E-03
**RP11-551G24.2**	-3.27	3.76E-03	7.10E-03
**PRIM1**	-0.47	3.77E-03	7.10E-03
**RBP5**	1.31	3.77E-03	7.10E-03
**RP11-637A17.2**	-2.00	3.77E-03	7.10E-03
**MX2**	1.13	3.77E-03	7.10E-03
**AC005086.1**	-3.37	3.77E-03	7.10E-03
**RP11-17A1.3**	-2.63	3.77E-03	7.10E-03
**BUB1B**	-0.52	3.77E-03	7.10E-03
**RP11-347C12.1**	1.81	3.77E-03	7.11E-03
**PTGES3**	0.36	3.77E-03	7.11E-03
**THBS4**	1.87	3.77E-03	7.11E-03
**AC093838.4**	0.68	3.78E-03	7.11E-03
**ARHGAP18**	0.66	3.78E-03	7.12E-03
**ZNF385D-AS1**	-4.02	3.78E-03	7.12E-03
**NUTM2B**	-1.52	3.78E-03	7.12E-03
**GALR2**	1.96	3.79E-03	7.13E-03
**BPIFC**	-0.65	3.79E-03	7.14E-03
**MKS1**	0.37	3.79E-03	7.15E-03
**CCDC3**	-0.66	3.79E-03	7.15E-03
**WFDC5**	0.53	3.80E-03	7.15E-03

**NTNG2**	1.20	3.80E-03	7.15E-03
**AC005256.1**	4.15	3.80E-03	7.16E-03
**TRAJ12**	-3.67	3.80E-03	7.16E-03
**G22345**	-2.10	3.81E-03	7.17E-03
**SAMD11P1**	-3.63	3.81E-03	7.17E-03
**CTC-277H1.6**	-1.33	3.81E-03	7.17E-03
**FBXL2**	-0.80	3.81E-03	7.17E-03
**LINC01229**	-1.58	3.81E-03	7.18E-03
**RP11-585P4.5**	0.97	3.81E-03	7.18E-03
**PWAR1**	-3.66	3.82E-03	7.19E-03
**LTV1P1**	-3.81	3.82E-03	7.19E-03
**DHRS2**	-3.04	3.82E-03	7.20E-03
**G32324**	-3.80	3.83E-03	7.20E-03
**LA16c-390H2.4**	-1.95	3.83E-03	7.20E-03
**RP11-144A16.8**	-3.52	3.83E-03	7.20E-03
**RNA5SP179**	-3.65	3.83E-03	7.21E-03
**CTD-2619J13.19**	-1.29	3.83E-03	7.21E-03
**XLOC_012736**	-1.02	3.83E-03	7.21E-03
**XLOC_005502**	-4.09	3.83E-03	7.21E-03
**RP11-350F16.2**	-4.26	3.84E-03	7.22E-03
**RP11-310P5.1**	-3.87	3.84E-03	7.22E-03
**SULT1E1**	-1.02	3.84E-03	7.23E-03
**RP11-676J12.8**	-1.53	3.84E-03	7.23E-03
**RNU6-979P**	-3.53	3.84E-03	7.23E-03
**RP11-43D4.2**	-3.59	3.84E-03	7.23E-03
**RP11-326I11.3**	0.85	3.85E-03	7.23E-03
**RP4-800M22.2**	-2.37	3.85E-03	7.24E-03
**RP4-813D12.3**	-1.83	3.86E-03	7.25E-03
**AC017078.1**	-3.77	3.86E-03	7.25E-03
**AP000857.2**	-3.90	3.86E-03	7.25E-03
**RP4-622L5.7**	-3.63	3.86E-03	7.25E-03
**RP11-701H24.5**	-2.41	3.86E-03	7.25E-03
**FLVCR1**	-0.41	3.86E-03	7.25E-03
**SMIM7**	-0.27	3.86E-03	7.25E-03
**RNU6-1318P**	-3.42	3.86E-03	7.26E-03
**ZFHX2**	-0.96	3.86E-03	7.26E-03
**SLC50A1**	0.55	3.87E-03	7.26E-03
**LINC01012**	-1.22	3.87E-03	7.26E-03
**RP11-16N11.2**	-0.95	3.87E-03	7.28E-03
**SPDYE2**	-1.64	3.87E-03	7.28E-03
**RP11-649A18.12**	0.93	3.87E-03	7.28E-03
**RN7SKP74**	-2.69	3.87E-03	7.28E-03
**RP11-43N5.1**	-2.46	3.88E-03	7.28E-03
**SLC8A3**	-1.57	3.88E-03	7.28E-03
**PAK7**	-2.27	3.88E-03	7.29E-03

**XXbac-BPG154L12.4**	-3.48	3.88E-03	7.29E-03
**CCDC74A**	0.83	3.88E-03	7.29E-03
**IGHEP1**	-5.44	3.88E-03	7.29E-03
**AC005534.6**	-3.80	3.88E-03	7.29E-03
**XLOC_004156**	-4.21	3.88E-03	7.29E-03
**RP11-193H22.2**	-3.59	3.89E-03	7.30E-03
**RNU6-422P**	-3.61	3.90E-03	7.31E-03
**ZNF473**	-0.41	3.90E-03	7.32E-03
**DRG2**	0.32	3.90E-03	7.32E-03
**CAPRIN1**	-0.29	3.90E-03	7.32E-03
**C21orf62**	-1.33	3.90E-03	7.32E-03
**MYBPC3**	-2.00	3.90E-03	7.33E-03
**AC007679.3**	-3.80	3.91E-03	7.33E-03
**RP11-293K19.1**	-3.55	3.91E-03	7.33E-03
**CTA-351J1.1**	-3.86	3.91E-03	7.34E-03
**RN7SKP173**	-2.81	3.91E-03	7.34E-03
**SLC22A16**	2.80	3.91E-03	7.35E-03
**CTD-2515H24.3**	-3.85	3.92E-03	7.35E-03
**RP11-462P6.1**	-3.53	3.92E-03	7.35E-03
**RP11-334E6.3**	-3.49	3.92E-03	7.35E-03
**RP1-191J18.66**	1.52	3.92E-03	7.35E-03
**RP11-701H24.3**	-1.42	3.92E-03	7.35E-03
**RNU6-74P**	-3.85	3.92E-03	7.35E-03
**HS1BP3-IT1**	-2.03	3.92E-03	7.36E-03
**VIPR2**	1.62	3.93E-03	7.38E-03
**AC004812.1**	-3.59	3.94E-03	7.38E-03
**CFAP53**	0.87	3.95E-03	7.40E-03
**ITGB3BP**	-0.42	3.95E-03	7.40E-03
**RP11-796E2.4**	0.75	3.95E-03	7.41E-03
**RP11-369G6.2**	-4.22	3.95E-03	7.41E-03
**PRKX-AS1**	-3.40	3.95E-03	7.41E-03
**POLRMTP1**	-2.34	3.95E-03	7.41E-03
**Vault**	-3.92	3.96E-03	7.42E-03
**RYK**	-0.29	3.96E-03	7.42E-03
**RP11-637C24.5**	-3.92	3.96E-03	7.43E-03
**KCNQ3**	1.05	3.97E-03	7.44E-03
**ELAC1**	-0.34	3.97E-03	7.44E-03
**CTD-2011F17.2**	-3.44	3.97E-03	7.44E-03
**MIR4782**	-3.64	3.97E-03	7.45E-03
**RP11-216B9.9**	-1.71	3.98E-03	7.45E-03
**CENPUP2**	-3.50	3.98E-03	7.46E-03
**AC090952.5**	-3.69	3.98E-03	7.46E-03
**HOXC13**	0.60	3.98E-03	7.46E-03
**RPL21P65**	-3.41	3.99E-03	7.47E-03
**ANKRD39**	-0.53	3.99E-03	7.47E-03

**SPG7**	0.47	3.99E-03	7.47E-03
**GSE1**	-0.40	3.99E-03	7.48E-03
**LA16c-360H6.1**	-1.55	3.99E-03	7.48E-03
**CRABP1**	-1.18	3.99E-03	7.48E-03
**RP11-780K2.1**	-3.77	3.99E-03	7.48E-03
**G36605**	0.64	4.00E-03	7.49E-03
**NACA3P**	-1.85	4.00E-03	7.50E-03
**SUZ12P1**	-0.70	4.01E-03	7.50E-03
**RCC2P4**	-3.69	4.01E-03	7.51E-03
**APOPT1**	0.33	4.01E-03	7.51E-03
**H1FX-AS1**	1.27	4.02E-03	7.52E-03
**RP3-466I7.1**	-3.60	4.02E-03	7.53E-03
**ST8SIA5**	2.64	4.02E-03	7.53E-03
**WIPF2**	0.30	4.02E-03	7.53E-03
**RNU6-879P**	-3.76	4.02E-03	7.53E-03
**NAALADL2-AS3**	-3.71	4.02E-03	7.53E-03
**RP11-377D9.3**	-1.51	4.02E-03	7.53E-03
**ZNF44**	-0.30	4.03E-03	7.53E-03
**PEMT**	0.44	4.03E-03	7.54E-03
**OR2AO1P**	-2.92	4.03E-03	7.54E-03
**RP4-671O14.5**	-4.14	4.03E-03	7.54E-03
**RP11-231C14.4**	-1.25	4.03E-03	7.54E-03
**AP3S1**	0.41	4.03E-03	7.54E-03
**GGN**	1.11	4.03E-03	7.54E-03
**G895**	1.24	4.03E-03	7.54E-03
**RP11-433P17.1**	-3.70	4.03E-03	7.55E-03
**CTD-2340D6.2**	-3.73	4.04E-03	7.55E-03
**STYXL1**	0.60	4.04E-03	7.55E-03
**G38114**	-1.81	4.04E-03	7.56E-03
**LRRC37A4P**	-1.67	4.04E-03	7.56E-03
**G10695**	-1.11	4.04E-03	7.56E-03
**OPA3**	0.36	4.05E-03	7.56E-03
**ELMO1-AS1**	-3.96	4.05E-03	7.57E-03
**RP11-540B6.6**	-0.79	4.06E-03	7.58E-03
**RP11-588L15.2**	-2.87	4.06E-03	7.59E-03
**MIR570**	-1.45	4.06E-03	7.59E-03
**ZSCAN12**	-0.45	4.06E-03	7.59E-03
**RP11-496H15.2**	-2.94	4.06E-03	7.60E-03
**RP11-142C4.6**	-1.04	4.07E-03	7.61E-03
**MRPL33**	0.54	4.08E-03	7.63E-03
**WSCD2**	1.47	4.09E-03	7.64E-03
**PTCD1**	-0.54	4.09E-03	7.65E-03
**KLF12**	-0.69	4.09E-03	7.65E-03
**GCNT6**	-3.40	4.10E-03	7.66E-03
**CTD-2538G9.5**	-2.37	4.10E-03	7.66E-03

**RN7SL359P**	-4.30	4.10E-03	7.66E-03
**RP11-164H5.1**	-3.99	4.10E-03	7.66E-03
**AHDC1**	0.74	4.10E-03	7.67E-03
**RP11-1099M24.6**	-0.95	4.10E-03	7.67E-03
**SNORD88A**	-2.66	4.11E-03	7.67E-03
**AC007099.1**	-3.69	4.11E-03	7.67E-03
**RP11-807C20.2**	-1.33	4.11E-03	7.67E-03
**AC130352.1**	-4.09	4.11E-03	7.67E-03
**TYMS**	0.62	4.11E-03	7.68E-03
**CTC-232P5.4**	-3.64	4.11E-03	7.68E-03
**MIR648**	-3.68	4.12E-03	7.69E-03
**CTD-2047H16.3**	-1.82	4.12E-03	7.69E-03
**C10orf95**	-1.88	4.12E-03	7.70E-03
**G554**	-3.09	4.12E-03	7.70E-03
**G31765**	0.86	4.13E-03	7.71E-03
**EIF2A**	-0.47	4.13E-03	7.71E-03
**RP5-1159O4.2**	-1.05	4.13E-03	7.71E-03
**RNU6-1176P**	-3.48	4.13E-03	7.71E-03
**EEF1GP1**	-4.12	4.13E-03	7.71E-03
**Z98044.1**	-3.42	4.13E-03	7.72E-03
**MIR4427**	-3.63	4.13E-03	7.72E-03
**MIR199A1**	-2.69	4.14E-03	7.73E-03
**RNU6-584P**	-3.48	4.15E-03	7.74E-03
**RP11-129B9.2**	-3.09	4.15E-03	7.74E-03
**RP11-69H7.4**	-3.80	4.15E-03	7.74E-03
**CTB-193M12.3**	-1.53	4.15E-03	7.74E-03
**TTC7A**	0.44	4.15E-03	7.75E-03
**RP11-588L15.1**	-3.94	4.15E-03	7.75E-03
**CHDH**	0.99	4.16E-03	7.75E-03
**MIR30C1**	-3.54	4.16E-03	7.76E-03
**P2RY12**	-1.75	4.16E-03	7.77E-03
**C1QTNF3-AMACR**	-3.40	4.17E-03	7.77E-03
**HSPA4**	-0.47	4.17E-03	7.77E-03
**RP11-527F13.1**	-2.66	4.17E-03	7.78E-03
**RP11-330M2.4**	-2.04	4.18E-03	7.79E-03
**DENND1C**	0.52	4.18E-03	7.79E-03
**RP11-616M17.1**	-2.54	4.18E-03	7.79E-03
**DDX55P1**	2.72	4.18E-03	7.79E-03
**RP11-93O14.1**	-3.65	4.18E-03	7.80E-03
**CACHD1**	0.63	4.19E-03	7.81E-03
**NFKBID**	0.60	4.19E-03	7.81E-03
**TATDN2**	0.41	4.19E-03	7.81E-03
**RP11-713H12.2**	-3.51	4.20E-03	7.82E-03
**PAN3-AS1**	-0.87	4.20E-03	7.83E-03
**RPS10P14**	-3.81	4.20E-03	7.83E-03

**KB-1460A1.1**	-1.49	4.20E-03	7.83E-03
**BTLA**	-1.54	4.20E-03	7.83E-03
**SLC34A3**	-1.53	4.21E-03	7.84E-03
**NAPSB**	0.83	4.21E-03	7.84E-03
**RP3-471C18.2**	-3.77	4.21E-03	7.85E-03
**MIR30B**	-3.86	4.21E-03	7.85E-03
**RP11-556O9.3**	-3.21	4.22E-03	7.85E-03
**SLC35G2**	1.17	4.22E-03	7.85E-03
**RPP30**	-0.26	4.22E-03	7.86E-03
**RP11-709A23.2**	-1.60	4.22E-03	7.86E-03
**RNU6-490P**	-3.66	4.22E-03	7.87E-03
**RNU6-975P**	-3.56	4.23E-03	7.87E-03
**BTNL8**	-3.69	4.23E-03	7.87E-03
**AKTIP**	-0.43	4.23E-03	7.88E-03
**RP11-101E7.2**	-2.91	4.23E-03	7.88E-03
**GGTA1P**	0.80	4.23E-03	7.88E-03
**SCOCP1**	-2.54	4.24E-03	7.89E-03
**ABCC2**	-0.61	4.24E-03	7.89E-03
**RP11-1060G2.2**	-3.46	4.24E-03	7.89E-03
**RP11-449L13.2**	-3.32	4.24E-03	7.89E-03
**AC010967.2**	-3.25	4.24E-03	7.89E-03
**RP11-644F5.10**	-1.45	4.24E-03	7.90E-03
**G38278**	-3.29	4.25E-03	7.90E-03
**CACNA1I**	-1.73	4.25E-03	7.91E-03
**PPM1E**	-1.44	4.25E-03	7.91E-03
**GLIPR1L1**	-1.33	4.25E-03	7.91E-03
**RNU7-160P**	-3.69	4.26E-03	7.92E-03
**HMGN3**	0.43	4.26E-03	7.92E-03
**RNASEK**	1.09	4.26E-03	7.92E-03
**G32126**	-1.70	4.26E-03	7.93E-03
**HNRNPKP4**	-3.33	4.26E-03	7.93E-03
**MORF4L2-AS1**	-1.27	4.26E-03	7.93E-03
**EP400NL**	0.73	4.26E-03	7.93E-03
**RP11-403P17.6**	-1.80	4.26E-03	7.93E-03
**RECQL5**	0.55	4.27E-03	7.94E-03
**SFXN3**	0.44	4.27E-03	7.94E-03
**RP1-197B17.5**	-1.55	4.27E-03	7.95E-03
**AC024937.6**	-1.62	4.27E-03	7.95E-03
**PIP4K2B**	0.22	4.28E-03	7.96E-03
**GAPDHP22**	-3.64	4.29E-03	7.98E-03
**RPL34P6**	-2.14	4.30E-03	7.99E-03
**RP11-578F21.2**	-3.28	4.30E-03	7.99E-03
**ERO1B**	-0.52	4.30E-03	7.99E-03
**RP5-1065J22.8**	0.95	4.31E-03	8.01E-03
**POLR3C**	0.28	4.32E-03	8.02E-03

**COLQ**	0.94	4.32E-03	8.03E-03
**RP11-428C19.5**	-3.44	4.32E-03	8.03E-03
**RP5-1125A11.7**	-0.91	4.32E-03	8.03E-03
**SERPINB5**	-0.67	4.32E-03	8.03E-03
**HMGB1**	0.47	4.33E-03	8.04E-03
**LINC01534**	-0.69	4.33E-03	8.04E-03
**RP11-13K12.5**	2.10	4.33E-03	8.05E-03
**NR2E3**	-1.70	4.33E-03	8.05E-03
**SNORD116**	-3.52	4.34E-03	8.06E-03
**RP11-395N3.2**	-3.13	4.34E-03	8.06E-03
**RPS3AP2**	-3.43	4.34E-03	8.06E-03
**MTMR9LP**	1.10	4.34E-03	8.06E-03
**FAM86KP**	-2.91	4.34E-03	8.06E-03
**VPS11**	0.27	4.34E-03	8.06E-03
**MIR3973**	-3.61	4.35E-03	8.07E-03
**PCDHGA3**	-0.85	4.35E-03	8.07E-03
**VN1R85P**	-4.01	4.35E-03	8.07E-03
**RP11-762H8.2**	-1.19	4.35E-03	8.08E-03
**FHDC1**	0.52	4.35E-03	8.08E-03
**CDC42P6**	-1.45	4.35E-03	8.08E-03
**TNS3**	0.75	4.35E-03	8.08E-03
**RP11-449L23.2**	-1.80	4.36E-03	8.09E-03
**GSTZ1**	0.28	4.36E-03	8.10E-03
**CYB561A3**	-0.56	4.36E-03	8.10E-03
**PPIEL**	-1.45	4.37E-03	8.10E-03
**TTC12**	-0.58	4.37E-03	8.10E-03
**CCDC180**	-1.47	4.37E-03	8.10E-03
**RP3-509I19.6**	-2.99	4.37E-03	8.11E-03
**VSIG1**	1.08	4.37E-03	8.11E-03
**NABP1**	0.71	4.38E-03	8.12E-03
**EOGT**	0.47	4.38E-03	8.13E-03
**PHBP15**	-2.59	4.38E-03	8.13E-03
**G39546**	3.17	4.38E-03	8.13E-03
**KB-1958F4.2**	-3.88	4.38E-03	8.13E-03
**SNED1**	0.96	4.39E-03	8.14E-03
**SWAP70**	-0.35	4.39E-03	8.14E-03
**CTD-2561B21.4**	-3.34	4.39E-03	8.14E-03
**RNPS1P1**	-3.47	4.39E-03	8.14E-03
**RP3-467L1.4**	-1.80	4.39E-03	8.15E-03
**RGPD3**	-1.12	4.39E-03	8.15E-03
**SKI**	0.54	4.40E-03	8.16E-03
**RP11-266L9.3**	-2.50	4.40E-03	8.16E-03
**UPK1A-AS1**	-3.69	4.41E-03	8.17E-03
**OTOGL**	-1.85	4.41E-03	8.17E-03
**RNU6-135P**	-3.71	4.41E-03	8.17E-03

**AC010243.1**	-3.57	4.41E-03	8.18E-03
**RP11-162A23.5**	-3.86	4.42E-03	8.18E-03
**RP11-357H14.16**	-3.65	4.42E-03	8.19E-03
**G36510**	-1.21	4.43E-03	8.20E-03
**TTC39A-AS1**	-2.30	4.43E-03	8.21E-03
**RP4-713B5.2**	-2.09	4.43E-03	8.21E-03
**AK9**	-0.45	4.43E-03	8.21E-03
**SEC1P**	1.46	4.43E-03	8.21E-03
**BZW2**	-0.36	4.44E-03	8.22E-03
**RN7SL463P**	-3.66	4.44E-03	8.23E-03
**KIAA1586**	-0.44	4.44E-03	8.23E-03
**FAM3D**	1.46	4.45E-03	8.24E-03
**FAM72C**	-2.08	4.45E-03	8.24E-03
**LEKR1**	-0.94	4.45E-03	8.25E-03
**CCR6**	-3.40	4.45E-03	8.25E-03
**CABIN1**	0.42	4.46E-03	8.25E-03
**LARP1P1**	-3.70	4.46E-03	8.25E-03
**RP11-806L2.2**	-3.13	4.46E-03	8.27E-03
**RP11-102G14.1**	-1.53	4.46E-03	8.27E-03
**INPP5D**	0.57	4.47E-03	8.28E-03
**LINC00596**	-3.40	4.47E-03	8.28E-03
**RBPMS-AS1**	1.84	4.47E-03	8.28E-03
**MIR548Q**	-3.56	4.47E-03	8.28E-03
**TMEM163**	1.81	4.48E-03	8.29E-03
**XLOC_005411**	-3.38	4.48E-03	8.29E-03
**KCNE3**	1.15	4.48E-03	8.30E-03
**STPG1**	0.47	4.49E-03	8.31E-03
**CDKN2AIPNL**	0.42	4.49E-03	8.31E-03
**MCM3AP**	-0.28	4.49E-03	8.32E-03
**RP11-295D4.5**	-1.74	4.50E-03	8.32E-03
**XLOC_001223**	-1.41	4.50E-03	8.32E-03
**G41963**	2.79	4.50E-03	8.32E-03
**ZNF137P**	-0.68	4.50E-03	8.33E-03
**KCNT2**	-1.10	4.50E-03	8.33E-03
**SNAP23**	0.31	4.50E-03	8.33E-03
**HOXA10**	0.77	4.50E-03	8.33E-03
**SRSF8**	0.39	4.50E-03	8.33E-03
**RN7SL587P**	1.85	4.51E-03	8.33E-03
**PIRT**	2.48	4.51E-03	8.34E-03
**ENOSF1**	-0.81	4.51E-03	8.34E-03
**RP11-445F6.2**	-3.59	4.52E-03	8.36E-03
**TKTL1**	2.12	4.52E-03	8.36E-03
**GINM1**	0.38	4.53E-03	8.37E-03
**INTS12**	0.27	4.53E-03	8.37E-03
**POU6F1**	0.55	4.53E-03	8.37E-03

**RP11-925D8.3**	-3.91	4.53E-03	8.38E-03
**ZNF630-AS1**	-4.00	4.54E-03	8.38E-03
**RP11-407B7.1**	-1.89	4.54E-03	8.40E-03
**FEM1A**	-0.64	4.54E-03	8.40E-03
**AC011999.1**	-3.41	4.54E-03	8.40E-03
**EFHB**	-1.79	4.55E-03	8.41E-03
**LINC00310**	1.79	4.56E-03	8.42E-03
**MAATS1**	-1.09	4.56E-03	8.42E-03
**NTRK2**	0.80	4.56E-03	8.43E-03
**LINC00857**	1.68	4.56E-03	8.43E-03
**ZNF285B**	-1.91	4.56E-03	8.43E-03
**SLC2A11**	0.65	4.56E-03	8.43E-03
**FAM86EP**	-0.65	4.57E-03	8.44E-03
**RP11-415F23.2**	-1.18	4.57E-03	8.44E-03
**SPSB2**	0.63	4.57E-03	8.44E-03
**RP11-452K12.6**	-3.69	4.58E-03	8.46E-03
**RPS2P5**	1.47	4.58E-03	8.46E-03
**ABI2**	-0.36	4.58E-03	8.46E-03
**RRM1-AS1**	-3.67	4.59E-03	8.47E-03
**RP11-113A11.3**	-3.47	4.59E-03	8.47E-03
**FCGR3B**	1.62	4.59E-03	8.47E-03
**CTD-3065J16.9**	1.24	4.59E-03	8.48E-03
**RP11-300D11.3**	-3.39	4.59E-03	8.48E-03
**DNAH3**	-0.59	4.60E-03	8.48E-03
**TSSK6**	0.76	4.60E-03	8.50E-03
**INCA1**	0.79	4.61E-03	8.50E-03
**RTP3**	-3.39	4.61E-03	8.50E-03
**RN7SL414P**	-3.47	4.61E-03	8.51E-03
**RNU6-1226P**	-3.73	4.61E-03	8.51E-03
**WFDC13**	-3.38	4.61E-03	8.52E-03
**RP11-84D1.1**	-1.98	4.62E-03	8.52E-03
**CTD-2506P8.6**	0.94	4.62E-03	8.52E-03
**ATP2B3**	-2.01	4.62E-03	8.53E-03
**CTB-180A7.6**	-3.33	4.63E-03	8.55E-03
**GDI2P2**	-2.02	4.63E-03	8.55E-03
**AMOT**	-0.56	4.64E-03	8.56E-03
**AL359542.1**	-3.49	4.64E-03	8.56E-03
**NLRP1**	0.53	4.64E-03	8.56E-03
**RP4-737E23.2**	1.33	4.64E-03	8.57E-03
**RPL23AP70**	-3.77	4.65E-03	8.57E-03
**RP11-312O7.2**	-0.68	4.65E-03	8.57E-03
**GPHN**	-0.36	4.65E-03	8.58E-03
**GINS1**	-0.44	4.66E-03	8.59E-03
**G25440**	-3.55	4.66E-03	8.59E-03
**RP11-319E16.2**	-3.97	4.66E-03	8.59E-03

**MIR455**	-3.60	4.66E-03	8.60E-03
**UBXN11**	0.79	4.66E-03	8.60E-03
**SLC38A11**	1.53	4.66E-03	8.60E-03
**KRT1**	1.16	4.66E-03	8.60E-03
**CIDECP**	0.57	4.67E-03	8.60E-03
**ARR3**	-1.08	4.67E-03	8.60E-03
**LA16c-380H5.5**	-2.74	4.67E-03	8.60E-03
**MIRLET7D**	-1.38	4.67E-03	8.60E-03
**INTS9**	0.30	4.67E-03	8.61E-03
**PDCL3P6**	-3.60	4.68E-03	8.62E-03
**GPR88**	3.30	4.68E-03	8.62E-03
**ZIC4**	1.69	4.68E-03	8.62E-03
**LY75**	-0.81	4.68E-03	8.62E-03
**UTP3**	0.35	4.68E-03	8.63E-03
**LINC00323**	1.95	4.68E-03	8.63E-03
**AC090427.1**	-3.61	4.69E-03	8.63E-03
**G2608**	4.04	4.69E-03	8.63E-03
**CYTH3**	0.33	4.69E-03	8.64E-03
**PITX2**	1.66	4.70E-03	8.65E-03
**RP11-390E23.6**	-0.50	4.70E-03	8.65E-03
**ATP5F1P7**	-3.60	4.70E-03	8.66E-03
**RP11-174G6.1**	-1.26	4.70E-03	8.66E-03
**PNN**	0.44	4.71E-03	8.67E-03
**OR7E91P**	1.20	4.72E-03	8.68E-03
**bP-2189O9.2**	1.99	4.72E-03	8.68E-03
**MIR3138**	-3.34	4.72E-03	8.68E-03
**ZNF783**	0.49	4.72E-03	8.69E-03
**RNA5SP395**	-3.61	4.72E-03	8.70E-03
**RP11-434H6.6**	2.33	4.72E-03	8.70E-03
**AL136218.1**	-3.91	4.73E-03	8.70E-03
**RP11-486M23.1**	-3.92	4.74E-03	8.72E-03
**U2AF1L4**	1.21	4.74E-03	8.72E-03
**CCR5**	1.30	4.74E-03	8.72E-03
**XLOC_003787**	-1.63	4.74E-03	8.73E-03
**STKLD1**	-0.86	4.74E-03	8.73E-03
**RP11-497D6.5**	-3.75	4.75E-03	8.73E-03
**COL11A2**	1.01	4.75E-03	8.74E-03
**RP11-446H18.5**	-1.85	4.75E-03	8.75E-03
**KRT18P60**	-3.66	4.75E-03	8.75E-03
**BCL9P1**	-3.86	4.76E-03	8.75E-03
**CTD-2287O16.4**	-3.48	4.76E-03	8.76E-03
**CYP2T3P**	-3.90	4.76E-03	8.76E-03
**CTC-548K16.6**	3.33	4.76E-03	8.76E-03
**NPIPA5**	2.03	4.76E-03	8.76E-03
**UTP14A**	0.43	4.76E-03	8.76E-03

**LL21NC02-1C16.1**	2.20	4.77E-03	8.77E-03
**NSDHL**	0.81	4.77E-03	8.78E-03
**CTA-243E7.2**	-2.95	4.77E-03	8.78E-03
**SNORA4**	-2.98	4.78E-03	8.78E-03
**ACTG1P1**	-1.59	4.78E-03	8.79E-03
**RP11-290L1.4**	-3.30	4.78E-03	8.79E-03
**AC073257.2**	4.14	4.78E-03	8.79E-03
**RP11-205A8.3**	-3.74	4.78E-03	8.79E-03
**RP11-158H5.7**	-0.53	4.78E-03	8.80E-03
**MIR4697HG**	-1.07	4.79E-03	8.80E-03
**PLB1**	-0.61	4.79E-03	8.81E-03
**HLCS**	-0.92	4.80E-03	8.81E-03
**KCNJ5**	0.95	4.80E-03	8.82E-03
**TRAJ39**	-3.22	4.80E-03	8.82E-03
**RP11-268I9.1**	-3.49	4.80E-03	8.83E-03
**HARS2**	0.30	4.81E-03	8.84E-03
**LGALS8-AS1**	-1.94	4.81E-03	8.84E-03
**CRYZL1**	-0.31	4.81E-03	8.85E-03
**RPL7L1P9**	-3.45	4.82E-03	8.85E-03
**RP11-434B12.1**	-0.85	4.82E-03	8.86E-03
**MANSC1**	0.35	4.83E-03	8.88E-03
**RP3-333A15.2**	-1.12	4.84E-03	8.88E-03
**DBIL5P2**	-1.43	4.84E-03	8.89E-03
**RPL23AP82**	0.66	4.84E-03	8.89E-03
**FAM153C**	-1.90	4.85E-03	8.90E-03
**MIR558**	-3.70	4.85E-03	8.90E-03
**RBM22P4**	-3.52	4.85E-03	8.90E-03
**UGGT2**	-0.53	4.85E-03	8.91E-03
**RP1-193H18.2**	-0.63	4.85E-03	8.91E-03
**HMGN2P20**	-3.61	4.86E-03	8.93E-03
**RP11-732M18.2**	-3.71	4.87E-03	8.93E-03
**PACRG-AS3**	-3.45	4.87E-03	8.94E-03
**AL590084.1**	-3.40	4.88E-03	8.95E-03
**CLVS2**	-2.49	4.88E-03	8.96E-03
**ADAM11**	0.79	4.88E-03	8.96E-03
**GPHA2**	-3.48	4.88E-03	8.96E-03
**MYOZ3**	1.53	4.88E-03	8.96E-03
**RP11-109N23.4**	-1.78	4.88E-03	8.96E-03
**APOBEC3H**	2.28	4.89E-03	8.97E-03
**LA16c-313D11.12**	-0.75	4.89E-03	8.97E-03
**ATAT1**	0.66	4.89E-03	8.97E-03
**MYO18B**	-3.02	4.89E-03	8.97E-03
**ATP5B**	0.35	4.90E-03	8.99E-03
**SMIM5**	0.61	4.90E-03	8.99E-03
**RNU1-134P**	-3.23	4.91E-03	9.00E-03

**CTD-2561J22.2**	-1.01	4.91E-03	9.01E-03
**TCEB3C**	-3.97	4.92E-03	9.03E-03
**SNORD62**	-3.52	4.92E-03	9.03E-03
**RP11-313A24.1**	-2.60	4.93E-03	9.05E-03
**RP11-507K13.6**	-1.50	4.94E-03	9.06E-03
**ARMCX5**	-0.41	4.94E-03	9.06E-03
**CA5BP1**	0.58	4.94E-03	9.06E-03
**RP11-113K21.1**	-3.55	4.95E-03	9.07E-03
**NECAP1P2**	-3.28	4.95E-03	9.08E-03
**AC013448.1**	-3.73	4.95E-03	9.08E-03
**MIR3136**	-3.45	4.96E-03	9.09E-03
**TFF3**	1.22	4.96E-03	9.09E-03
**snoR442**	-2.59	4.96E-03	9.09E-03
**RP11-434H14.1**	-3.54	4.96E-03	9.09E-03
**HNRNPA3P7**	-3.63	4.96E-03	9.09E-03
**AC084290.2**	-3.74	4.96E-03	9.10E-03
**APOM**	-0.61	4.97E-03	9.11E-03
**CCL7**	4.14	4.97E-03	9.11E-03
**SLC25A23**	0.57	4.97E-03	9.11E-03
**RP11-613C6.2**	-3.33	4.98E-03	9.12E-03
**LIPI**	-2.94	4.99E-03	9.14E-03
**G22291**	-1.47	4.99E-03	9.14E-03
**CTD-3252C9.4**	-0.96	4.99E-03	9.14E-03
**PSPH**	0.57	4.99E-03	9.14E-03
**OR7E128P**	-1.46	4.99E-03	9.15E-03
**SERPINI2**	-3.47	5.00E-03	9.15E-03
**STXBP5L**	-1.75	5.00E-03	9.16E-03
**RP11-638I2.2**	-1.31	5.00E-03	9.16E-03
**TRAJ34**	-3.77	5.00E-03	9.16E-03
**RN7SL208P**	-1.62	5.00E-03	9.16E-03
**GID4**	-0.33	5.01E-03	9.17E-03
**LMOD2**	-2.83	5.01E-03	9.17E-03
**PIN4**	0.50	5.02E-03	9.18E-03
**AC016644.1**	-3.36	5.02E-03	9.19E-03
**RP1-244F24.1**	1.76	5.02E-03	9.19E-03
**CTD-2506J14.1**	-3.44	5.02E-03	9.19E-03
**GYPB**	-3.94	5.02E-03	9.19E-03
**RN7SL788P**	-3.16	5.03E-03	9.20E-03
**WDSUB1**	-0.52	5.03E-03	9.20E-03
**C11orf80**	-0.44	5.03E-03	9.21E-03
**AC009411.1**	-3.73	5.03E-03	9.21E-03
**MAGEL2**	2.52	5.03E-03	9.21E-03
**RP11-76C10.4**	-3.96	5.05E-03	9.23E-03
**CTA-363E6.2**	-3.66	5.05E-03	9.23E-03
**RNU6-268P**	-3.66	5.06E-03	9.26E-03

**RNF4**	0.22	5.06E-03	9.26E-03
**RP1-267D11.1**	-3.48	5.06E-03	9.26E-03
**DBI**	0.61	5.06E-03	9.26E-03
**RNU6-619P**	-3.34	5.06E-03	9.26E-03
**MCFD2**	0.33	5.07E-03	9.27E-03
**FAM45A**	0.35	5.09E-03	9.31E-03
**WNK4**	-1.12	5.09E-03	9.31E-03
**BCRP8**	-3.52	5.10E-03	9.33E-03
**RP11-352B15.2**	-3.38	5.10E-03	9.33E-03
**MIR221**	-1.90	5.11E-03	9.35E-03
**RNA5SP129**	-3.84	5.11E-03	9.35E-03
**BICC1**	1.21	5.12E-03	9.36E-03
**SYNGR3**	1.28	5.12E-03	9.36E-03
**TSPAN6**	0.82	5.12E-03	9.37E-03
**G4061**	-1.10	5.12E-03	9.37E-03
**LHCGR**	-2.96	5.12E-03	9.37E-03
**RCAN1**	0.62	5.12E-03	9.37E-03
**AGK**	-0.28	5.12E-03	9.37E-03
**C6orf106**	0.34	5.13E-03	9.38E-03
**RP11-863P13.3**	2.86	5.13E-03	9.38E-03
**FLRT1**	-1.52	5.13E-03	9.38E-03
**CTD-2547L24.4**	2.24	5.14E-03	9.39E-03
**RP11-160H22.3**	-3.76	5.14E-03	9.40E-03
**CTB-12O2.1**	-3.59	5.14E-03	9.40E-03
**MTSS1**	-0.28	5.14E-03	9.40E-03
**STX18-IT1**	-2.58	5.15E-03	9.41E-03
**RP5-994D16.9**	-0.67	5.15E-03	9.41E-03
**RP11-366L5.1**	-0.86	5.15E-03	9.42E-03
**RN7SL308P**	-3.25	5.16E-03	9.42E-03
**RPL5P4**	-1.18	5.16E-03	9.42E-03
**RTN4R**	-0.58	5.16E-03	9.43E-03
**RN7SL263P**	-2.36	5.16E-03	9.43E-03
**G35259**	-2.18	5.17E-03	9.44E-03
**SLC4A1AP**	0.31	5.18E-03	9.45E-03
**RP11-426J5.2**	-3.41	5.18E-03	9.46E-03
**ITPKB**	0.40	5.18E-03	9.47E-03
**SNORD115**	-3.52	5.19E-03	9.48E-03
**CYP2C19**	-3.74	5.19E-03	9.48E-03
**CTA-276O3.4**	-2.56	5.19E-03	9.48E-03
**RP11-198M15.1**	-3.33	5.21E-03	9.51E-03
**RP6-24A23.8**	-3.58	5.21E-03	9.51E-03
**FAM24B**	-1.13	5.21E-03	9.51E-03
**ATF3**	1.17	5.22E-03	9.53E-03
**RP11-624M8.1**	2.62	5.23E-03	9.54E-03
**RNF165**	-0.75	5.23E-03	9.54E-03

**AC005162.4**	-3.61	5.24E-03	9.55E-03
**EI24**	0.41	5.24E-03	9.56E-03
**NUSAP1**	-0.54	5.24E-03	9.57E-03
**RP11-343B5.1**	1.71	5.25E-03	9.57E-03
**SHMT1P1**	-3.32	5.25E-03	9.59E-03
**NDE1**	0.31	5.25E-03	9.59E-03
**LINC01430**	-3.71	5.26E-03	9.59E-03
**RPL12P13**	-3.67	5.26E-03	9.59E-03
**RP3-521E19.3**	-3.63	5.26E-03	9.59E-03
**NUTF2P6**	2.31	5.26E-03	9.59E-03
**RP11-1072C15.1**	-3.50	5.26E-03	9.60E-03
**GTF3AP2**	-3.55	5.27E-03	9.60E-03
**PIN4P1**	-3.55	5.27E-03	9.60E-03
**RP11-404P21.6**	-3.78	5.27E-03	9.61E-03
**YTHDF3-AS1**	0.94	5.28E-03	9.63E-03
**G21880**	-3.03	5.28E-03	9.63E-03
**F11R**	0.35	5.29E-03	9.64E-03
**AF121897.4**	-3.75	5.29E-03	9.64E-03
**MAST4-IT1**	-3.79	5.29E-03	9.64E-03
**MIR550A3**	-3.63	5.29E-03	9.64E-03
**RP11-186B7.4**	-3.21	5.29E-03	9.64E-03
**HDDC3**	0.52	5.29E-03	9.65E-03
**RP11-415F23.3**	-1.63	5.29E-03	9.65E-03
**ASIC2**	-1.64	5.30E-03	9.65E-03
**CEP170P1**	-1.23	5.30E-03	9.65E-03
**PIPOX**	-0.98	5.30E-03	9.66E-03
**SCAP**	0.34	5.30E-03	9.66E-03
**G6277**	-0.62	5.31E-03	9.67E-03
**RP11-100L22.1**	2.52	5.32E-03	9.68E-03
**RAN**	0.48	5.32E-03	9.69E-03
**SLC35E4**	0.61	5.32E-03	9.69E-03
**ANKRD20A5P**	2.00	5.33E-03	9.70E-03
**DPEP2**	1.34	5.33E-03	9.71E-03
**LINC01213**	-2.09	5.33E-03	9.72E-03
**RP13-46H24.1**	-2.12	5.33E-03	9.72E-03
**C20orf202**	1.11	5.34E-03	9.73E-03
**CHCHD1**	0.41	5.34E-03	9.73E-03
**XLOC_007614**	-2.17	5.34E-03	9.73E-03
**RP11-375N9.2**	-3.71	5.35E-03	9.75E-03
**ZNF595**	-0.75	5.37E-03	9.78E-03
**RNA5SP238**	-3.94	5.37E-03	9.78E-03
**C1orf74**	-0.53	5.38E-03	9.80E-03
**PIGL**	-0.74	5.39E-03	9.82E-03
**RNU7-140P**	-3.22	5.39E-03	9.82E-03
**RNGTTP1**	-3.37	5.40E-03	9.83E-03

**CKM**	-1.68	5.40E-03	9.83E-03
**GS1-114I9.3**	-3.18	5.40E-03	9.83E-03
**HDAC1P2**	-1.66	5.40E-03	9.83E-03
**RSRC1**	-0.37	5.41E-03	9.84E-03
**CYP3A43**	-3.52	5.41E-03	9.84E-03
**LINC00968**	1.89	5.41E-03	9.85E-03
**TCTEX1D4**	-1.71	5.41E-03	9.85E-03
**RP11-570L15.1**	-3.62	5.42E-03	9.85E-03
**CTD-2086O20.1**	-3.89	5.42E-03	9.85E-03
**RPS12P31**	-3.30	5.43E-03	9.87E-03
**RNA5SP260**	-3.54	5.43E-03	9.87E-03
**ZMYND8**	0.30	5.43E-03	9.88E-03
**RP11-303G3.9**	-3.94	5.43E-03	9.88E-03
**PVRL3**	-0.83	5.44E-03	9.89E-03
**XLOC_002788**	-1.77	5.44E-03	9.89E-03
**RNU5E-3P**	-4.05	5.45E-03	9.90E-03
**YWHABP2**	-1.78	5.45E-03	9.91E-03
**RPL12P41**	-3.37	5.45E-03	9.91E-03
**GRM7-AS1**	-2.11	5.46E-03	9.92E-03
**NPM1P24**	-3.46	5.46E-03	9.93E-03
**YAP1P1**	-1.84	5.46E-03	9.93E-03
**IGFL2**	-0.84	5.47E-03	9.94E-03
**UXS1**	0.38	5.47E-03	9.94E-03
**LINC00640**	-0.52	5.47E-03	9.94E-03
**RP11-692P14.1**	-3.76	5.47E-03	9.95E-03
**FAM72D**	-0.88	5.48E-03	9.96E-03
**EMC2**	0.34	5.49E-03	9.98E-03
**LDHAP3**	-3.72	5.49E-03	9.98E-03
**RP11-353N4.2**	-2.96	5.49E-03	9.99E-03
**RP11-513D5.2**	-3.25	5.50E-03	9.99E-03
**G10019**	-1.67	5.50E-03	9.99E-03
**GABPA**	-0.42	5.50E-03	9.99E-03
**G32202**	-2.46	5.50E-03	1.00E-02
**RN7SKP92**	-3.63	5.50E-03	1.00E-02
**MGP**	1.09	5.51E-03	1.00E-02
**WNK2**	-1.21	5.51E-03	1.00E-02
**POFUT1**	0.27	5.51E-03	1.00E-02
**RP11-426C22.5**	1.24	5.52E-03	1.00E-02
**FBXO36**	-0.51	5.52E-03	1.00E-02
**B4GALT4**	-0.27	5.52E-03	1.00E-02
**LINC01153**	-4.11	5.52E-03	1.00E-02
**RP11-615I2.1**	2.95	5.53E-03	1.00E-02
**RP11-408A13.4**	-1.53	5.53E-03	1.00E-02
**RP11-234K19.1**	-1.92	5.53E-03	1.00E-02
**CTD-2323K18.1**	-1.66	5.53E-03	1.00E-02

**AL137846.1**	-3.15	5.54E-03	1.00E-02
**CD28**	1.57	5.54E-03	1.01E-02
**G28011**	-3.88	5.56E-03	1.01E-02
**CFAP161**	-1.73	5.56E-03	1.01E-02
**RP11-73G16.3**	-3.61	5.56E-03	1.01E-02
**LINCR-0001**	-1.41	5.57E-03	1.01E-02
**RP1-71H19.2**	-3.43	5.57E-03	1.01E-02
**SLC25A3**	0.34	5.58E-03	1.01E-02
**RP11-14I17.2**	-1.86	5.58E-03	1.01E-02
**RP11-390K5.3**	-3.69	5.58E-03	1.01E-02
**SLC6A6P1**	-3.84	5.59E-03	1.01E-02
**ABCC9**	1.09	5.59E-03	1.01E-02
**FAM21EP**	1.03	5.59E-03	1.01E-02
**SPNS1**	1.59	5.59E-03	1.01E-02
**GS1-124K5.10**	-1.10	5.60E-03	1.02E-02
**LDHAL6FP**	-3.49	5.60E-03	1.02E-02
**XLOC_012720**	-3.33	5.60E-03	1.02E-02
**RP11-61K9.2**	-2.36	5.61E-03	1.02E-02
**SF3A3**	0.30	5.61E-03	1.02E-02
**XLOC_007556**	-0.95	5.61E-03	1.02E-02
**RP11-363J17.1**	-3.66	5.61E-03	1.02E-02
**MT-TH**	-4.37	5.61E-03	1.02E-02
**VSTM2L**	-1.27	5.62E-03	1.02E-02
**PRRG2**	0.53	5.62E-03	1.02E-02
**AC009014.3**	-1.98	5.62E-03	1.02E-02
**RNU6-1201P**	-3.34	5.63E-03	1.02E-02
**ARL10**	-0.69	5.63E-03	1.02E-02
**ERICH6**	-1.84	5.63E-03	1.02E-02
**ABCA4**	-1.32	5.63E-03	1.02E-02
**AC096579.13**	-1.57	5.64E-03	1.02E-02
**RP11-666A8.8**	-1.90	5.64E-03	1.02E-02
**XLOC_000757**	-2.68	5.64E-03	1.02E-02
**AC012370.2**	-1.91	5.64E-03	1.02E-02
**KARS**	0.33	5.65E-03	1.02E-02
**ERN2**	2.51	5.65E-03	1.02E-02
**NFAM1**	1.05	5.65E-03	1.02E-02
**LINC00539**	-1.03	5.66E-03	1.03E-02
**RP1-67K17.4**	-2.14	5.67E-03	1.03E-02
**IQCD**	0.88	5.67E-03	1.03E-02
**TGIF2P1**	-2.35	5.67E-03	1.03E-02
**PRKAA1**	-0.34	5.67E-03	1.03E-02
**XLOC_003364**	-2.44	5.68E-03	1.03E-02
**RP11-6I2.3**	3.21	5.68E-03	1.03E-02
**CTC-281F24.1**	-1.32	5.68E-03	1.03E-02
**LIPM**	-0.88	5.68E-03	1.03E-02

**IGKV3-15**	7.19	5.68E-03	1.03E-02
**U91328.2**	-3.67	5.68E-03	1.03E-02
**EPSTI1**	1.45	5.69E-03	1.03E-02
**RP11-121A14.3**	-3.17	5.69E-03	1.03E-02
**SNRNP48**	-0.31	5.70E-03	1.03E-02
**XLOC_005479**	-1.18	5.70E-03	1.03E-02
**RNA5SP345**	-3.55	5.70E-03	1.03E-02
**CTC-507E2.2**	-3.43	5.70E-03	1.03E-02
**IGFL1**	2.41	5.71E-03	1.03E-02
**CDC23**	-0.33	5.71E-03	1.03E-02
**MIR5192**	-3.60	5.71E-03	1.03E-02
**TAF13P2**	-3.28	5.72E-03	1.03E-02
**MMP12**	-2.99	5.72E-03	1.03E-02
**CANX**	-0.36	5.72E-03	1.04E-02
**UBA7**	0.62	5.73E-03	1.04E-02
**RNU6-455P**	-3.59	5.73E-03	1.04E-02
**HES7**	2.46	5.73E-03	1.04E-02
**NINJ2**	0.83	5.73E-03	1.04E-02
**G43442**	2.86	5.74E-03	1.04E-02
**MIRLET7A1**	-1.53	5.74E-03	1.04E-02
**CEP95**	-0.48	5.75E-03	1.04E-02
**CTD-2650P22.2**	1.42	5.75E-03	1.04E-02
**DHX34**	0.50	5.76E-03	1.04E-02
**GPRASP2**	-0.46	5.76E-03	1.04E-02
**EEF1GP4**	-2.62	5.76E-03	1.04E-02
**HAVCR2**	1.00	5.77E-03	1.04E-02
**DCK**	-0.51	5.77E-03	1.04E-02
**CTD-2291D10.4**	-2.43	5.77E-03	1.04E-02
**HSD17B1P1**	0.82	5.77E-03	1.04E-02
**RP11-59O6.3**	-3.62	5.77E-03	1.04E-02
**PIM2**	0.55	5.77E-03	1.04E-02
**AC097721.1**	-2.69	5.78E-03	1.04E-02
**MMP28**	-0.66	5.78E-03	1.04E-02
**RP11-53B2.4**	-2.69	5.78E-03	1.05E-02
**RP4-778K6.3**	-1.61	5.79E-03	1.05E-02
**HAAO**	0.61	5.79E-03	1.05E-02
**RP11-44F14.5**	-1.80	5.79E-03	1.05E-02
**PRODH**	-1.07	5.80E-03	1.05E-02
**ADCY10P1**	-0.90	5.81E-03	1.05E-02
**TLR5**	0.80	5.81E-03	1.05E-02
**LINC00865**	1.85	5.81E-03	1.05E-02
**OR52B5P**	-3.59	5.81E-03	1.05E-02
**PCGF6**	0.44	5.81E-03	1.05E-02
**NCR1**	2.43	5.81E-03	1.05E-02
**NPY6R**	-2.98	5.83E-03	1.05E-02

**SETP21**	-2.48	5.83E-03	1.05E-02
**CTC-571O20.1**	-3.58	5.83E-03	1.05E-02
**ZHX1-C8orf76**	-3.39	5.83E-03	1.05E-02
**SNHG17**	0.76	5.84E-03	1.05E-02
**GJA6P**	-3.56	5.84E-03	1.05E-02
**POLE3**	0.40	5.85E-03	1.06E-02
**LHFPL2**	0.90	5.85E-03	1.06E-02
**RP11-145A3.1**	2.33	5.85E-03	1.06E-02
**DDX43P1**	-4.02	5.85E-03	1.06E-02
**LINC01073**	-2.05	5.85E-03	1.06E-02
**FERP1**	-3.63	5.85E-03	1.06E-02
**CASC19**	-3.24	5.85E-03	1.06E-02
**RP11-1023L17.2**	-1.30	5.86E-03	1.06E-02
**RP11-810D13.1**	-2.18	5.86E-03	1.06E-02
**RPL29P24**	-2.74	5.86E-03	1.06E-02
**RP11-192N10.2**	-3.52	5.86E-03	1.06E-02
**XLOC_007506**	-2.31	5.87E-03	1.06E-02
**CAPN3**	-1.03	5.88E-03	1.06E-02
**AP3S2**	0.35	5.88E-03	1.06E-02
**RP11-108L7.14**	-3.09	5.88E-03	1.06E-02
**AC092964.1**	-3.20	5.89E-03	1.06E-02
**SLX4**	0.55	5.89E-03	1.06E-02
**TUBB4BP2**	-3.70	5.89E-03	1.06E-02
**KRT17P1**	-4.26	5.89E-03	1.06E-02
**DTX2P1**	-0.99	5.90E-03	1.06E-02
**RP11-380L11.4**	2.09	5.90E-03	1.06E-02
**LDLRAD2**	0.98	5.91E-03	1.07E-02
**MIR4755**	-3.14	5.91E-03	1.07E-02
**SCUBE1**	-1.32	5.91E-03	1.07E-02
**UBE2D4**	-0.34	5.92E-03	1.07E-02
**NRON**	-3.51	5.92E-03	1.07E-02
**CTAGE6**	-2.50	5.93E-03	1.07E-02
**RP11-129K12.1**	-2.44	5.93E-03	1.07E-02
**MIR4269**	-3.46	5.94E-03	1.07E-02
**G22256**	-1.23	5.94E-03	1.07E-02
**CPEB1**	-1.13	5.94E-03	1.07E-02
**TAPBP**	0.27	5.94E-03	1.07E-02
**ACER3**	0.58	5.95E-03	1.07E-02
**RS1**	-3.48	5.95E-03	1.07E-02
**LCE1A**	0.99	5.95E-03	1.07E-02
**RPS12P27**	-2.20	5.95E-03	1.07E-02
**MVB12B**	0.50	5.97E-03	1.08E-02
**CYP17A1**	-1.25	5.97E-03	1.08E-02
**BX088645.3**	-1.73	5.98E-03	1.08E-02
**LGALS12**	3.88	5.98E-03	1.08E-02

**ADAM32**	-0.96	5.99E-03	1.08E-02
**SEMA4F**	0.67	5.99E-03	1.08E-02
**RP11-70D24.3**	-2.24	5.99E-03	1.08E-02
**CTD-2370N5.3**	-3.47	5.99E-03	1.08E-02
**CTC-470E21.1**	-3.67	6.00E-03	1.08E-02
**GUCY2C**	-0.71	6.00E-03	1.08E-02
**LMX1A**	-2.07	6.01E-03	1.08E-02
**VAC14**	0.33	6.01E-03	1.08E-02
**RP11-342M3.5**	-3.92	6.01E-03	1.08E-02
**PLGLB2**	-3.20	6.02E-03	1.08E-02
**DHDH**	2.28	6.02E-03	1.08E-02
**TOX4P1**	-2.49	6.02E-03	1.08E-02
**ARHGEF19**	0.55	6.03E-03	1.09E-02
**ZBTB8A**	0.50	6.04E-03	1.09E-02
**G28007**	-3.27	6.04E-03	1.09E-02
**ID2-AS1**	-0.73	6.06E-03	1.09E-02
**DAB2**	1.08	6.06E-03	1.09E-02
**ABCC10**	0.49	6.06E-03	1.09E-02
**MLLT4-AS1**	0.91	6.07E-03	1.09E-02
**DNMT3A**	0.37	6.07E-03	1.09E-02
**IFITM9P**	-3.50	6.07E-03	1.09E-02
**MIR103A2**	1.31	6.08E-03	1.09E-02
**KLHL10**	-1.94	6.08E-03	1.09E-02
**CTD-2545H1.2**	-2.13	6.08E-03	1.09E-02
**TMEM132E**	1.49	6.08E-03	1.09E-02
**WRAP73**	0.41	6.08E-03	1.09E-02
**G303**	-1.92	6.08E-03	1.09E-02
**COL14A1**	1.17	6.10E-03	1.10E-02
**RP11-845C23.2**	-3.51	6.10E-03	1.10E-02
**CIDEC**	3.76	6.11E-03	1.10E-02
**SCHIP1**	1.15	6.11E-03	1.10E-02
**KBTBD3**	-0.42	6.11E-03	1.10E-02
**RP11-310E22.5**	-3.56	6.13E-03	1.10E-02
**RP11-203M5.7**	-1.45	6.13E-03	1.10E-02
**SMARCE1P6**	-2.57	6.13E-03	1.10E-02
**RP11-795J1.2**	-3.71	6.13E-03	1.10E-02
**CLEC16A**	-0.32	6.14E-03	1.10E-02
**RP13-638C3.3**	-1.74	6.14E-03	1.10E-02
**TAP2**	0.46	6.14E-03	1.10E-02
**CCDC17**	-0.99	6.15E-03	1.10E-02
**RNU6-593P**	-3.41	6.15E-03	1.11E-02
**DTHD1**	-1.97	6.15E-03	1.11E-02
**RP11-423E7.2**	-2.29	6.16E-03	1.11E-02
**SEPSECS-AS1**	-1.23	6.18E-03	1.11E-02
**HIST2H3DP1**	-2.69	6.18E-03	1.11E-02

**HMGN2P46**	-1.11	6.18E-03	1.11E-02
**C11orf57**	-0.27	6.19E-03	1.11E-02
**CEP68**	-0.31	6.19E-03	1.11E-02
**PRPSAP2**	0.36	6.19E-03	1.11E-02
**SNORD92**	-3.24	6.20E-03	1.11E-02
**ADGRE3**	-2.69	6.20E-03	1.11E-02
**RHPN1-AS1**	1.21	6.20E-03	1.11E-02
**PSORS1C2**	0.76	6.21E-03	1.11E-02
**AP006621.6**	-1.46	6.21E-03	1.12E-02
**AACS**	-0.43	6.21E-03	1.12E-02
**RP11-54H7.4**	-1.52	6.22E-03	1.12E-02
**CTAGE9**	-2.16	6.22E-03	1.12E-02
**SEC63P1**	-2.30	6.22E-03	1.12E-02
**DPY19L2P2**	-0.63	6.22E-03	1.12E-02
**APRT**	0.44	6.22E-03	1.12E-02
**XLOC_000972**	-1.74	6.22E-03	1.12E-02
**RTCB**	0.33	6.23E-03	1.12E-02
**RP11-13N12.1**	-3.71	6.23E-03	1.12E-02
**MIR579**	-3.99	6.23E-03	1.12E-02
**RP11-325E5.1**	-3.51	6.24E-03	1.12E-02
**SAMD9**	-0.69	6.25E-03	1.12E-02
**MTF1**	0.38	6.25E-03	1.12E-02
**TRUB1**	-0.40	6.25E-03	1.12E-02
**PSAT1**	-0.71	6.26E-03	1.12E-02
**G5617**	-1.80	6.27E-03	1.13E-02
**CDADC1**	0.35	6.27E-03	1.13E-02
**NAV2**	0.67	6.27E-03	1.13E-02
**DNAH12**	1.45	6.28E-03	1.13E-02
**RP11-5G9.6**	-3.36	6.28E-03	1.13E-02
**C15orf52**	0.87	6.28E-03	1.13E-02
**PHBP7**	-3.96	6.29E-03	1.13E-02
**IGKV2-24**	-3.88	6.29E-03	1.13E-02
**GEMIN8P4**	1.33	6.30E-03	1.13E-02
**RP11-793A3.1**	-3.34	6.31E-03	1.13E-02
**G39214**	-0.84	6.31E-03	1.13E-02
**SNHG15**	0.60	6.31E-03	1.13E-02
**RP11-862L9.3**	-2.63	6.32E-03	1.13E-02
**BHMT**	-1.46	6.32E-03	1.13E-02
**AP001469.7**	-1.61	6.33E-03	1.13E-02
**CDCA7**	-0.45	6.33E-03	1.13E-02
**SNX22**	-1.05	6.33E-03	1.14E-02
**AC084117.3**	1.38	6.34E-03	1.14E-02
**CRIPAK**	0.65	6.34E-03	1.14E-02
**RP11-720D4.2**	-3.38	6.34E-03	1.14E-02
**SERPINA13P**	-3.77	6.34E-03	1.14E-02

**MMP8**	4.14	6.35E-03	1.14E-02
**XLOC_004166**	-1.58	6.36E-03	1.14E-02
**SPATA7**	-0.49	6.36E-03	1.14E-02
**FLRT3**	-0.83	6.36E-03	1.14E-02
**RP11-57H12.3**	-1.23	6.36E-03	1.14E-02
**RP11-367N14.3**	-1.66	6.37E-03	1.14E-02
**RP1-292L20.3**	1.28	6.38E-03	1.14E-02
**ENPP7P8**	-3.48	6.38E-03	1.14E-02
**PDCD6IPP1**	-2.44	6.38E-03	1.14E-02
**DPEP1**	4.21	6.38E-03	1.14E-02
**RP11-400N13.3**	2.86	6.38E-03	1.14E-02
**RASAL2-AS1**	-0.89	6.38E-03	1.14E-02
**SNORD1B**	-3.25	6.39E-03	1.14E-02
**GS1-279B7.1**	-2.71	6.39E-03	1.14E-02
**GPR146**	0.81	6.39E-03	1.14E-02
**STEAP2**	-0.73	6.39E-03	1.15E-02
**RPL23AP40**	-3.41	6.40E-03	1.15E-02
**SLC25A6P3**	-3.51	6.40E-03	1.15E-02
**RP11-15A1.8**	-1.10	6.40E-03	1.15E-02
**RP11-502F1.1**	-2.43	6.41E-03	1.15E-02
**RP11-343K8.3**	-3.12	6.42E-03	1.15E-02
**TVP23CP1**	-3.42	6.42E-03	1.15E-02
**RP11-296P7.4**	-3.08	6.42E-03	1.15E-02
**EPB41L4B**	-0.58	6.43E-03	1.15E-02
**CD3EAP**	0.59	6.43E-03	1.15E-02
**RNA5SP233**	-3.54	6.43E-03	1.15E-02
**SLC39A2**	0.64	6.43E-03	1.15E-02
**USP44**	-0.80	6.44E-03	1.15E-02
**RPS5P3**	-3.41	6.44E-03	1.15E-02
**G26933**	-2.10	6.44E-03	1.15E-02
**XLOC_012700**	3.83	6.45E-03	1.15E-02
**AC084809.3**	-3.14	6.45E-03	1.15E-02
**RN7SL189P**	-3.77	6.45E-03	1.15E-02
**G22924**	-2.83	6.45E-03	1.15E-02
**TBX18**	-0.98	6.45E-03	1.15E-02
**RP11-346C16.2**	-3.39	6.46E-03	1.15E-02
**AKR1D1P1**	-3.44	6.46E-03	1.16E-02
**IGF1R**	-0.45	6.46E-03	1.16E-02
**PBOV1**	-3.48	6.48E-03	1.16E-02
**RP11-108M9.4**	-1.35	6.49E-03	1.16E-02
**XLOC_011005**	-4.02	6.49E-03	1.16E-02
**RP11-557F20.2**	-3.35	6.49E-03	1.16E-02
**ETV5-AS1**	-2.76	6.49E-03	1.16E-02
**RP1-228H13.5**	0.78	6.50E-03	1.16E-02
**LENG8**	1.04	6.50E-03	1.16E-02

**RHPN1**	1.07	6.50E-03	1.16E-02
**MIR608**	-3.72	6.51E-03	1.16E-02
**ACAT2**	-0.97	6.51E-03	1.16E-02
**RP11-27G24.3**	-2.01	6.52E-03	1.17E-02
**AC097721.2**	-1.06	6.52E-03	1.17E-02
**XLOC_000697**	1.16	6.52E-03	1.17E-02
**CTB-152G17.4**	-3.73	6.52E-03	1.17E-02
**RP11-803D5.1**	2.51	6.52E-03	1.17E-02
**RP13-452N2.1**	-2.38	6.53E-03	1.17E-02
**LINC01298**	-3.71	6.53E-03	1.17E-02
**SRGAP3-AS1**	-3.73	6.53E-03	1.17E-02
**RP11-109N23.6**	-1.51	6.53E-03	1.17E-02
**RN7SL254P**	-3.69	6.53E-03	1.17E-02
**SMIM1**	1.17	6.54E-03	1.17E-02
**RP11-250B2.4**	-2.45	6.54E-03	1.17E-02
**TCEB2P4**	-1.86	6.54E-03	1.17E-02
**RP11-236P24.1**	-3.56	6.54E-03	1.17E-02
**ARIH2**	0.22	6.54E-03	1.17E-02
**FAM156B**	1.07	6.55E-03	1.17E-02
**RP11-263C24.3**	-3.35	6.56E-03	1.17E-02
**RN7SL745P**	-3.01	6.56E-03	1.17E-02
**FCGR2B**	1.20	6.56E-03	1.17E-02
**CLPB**	-0.40	6.56E-03	1.17E-02
**RP5-827E24.1**	-3.48	6.56E-03	1.17E-02
**RP11-342M1.7**	-3.64	6.57E-03	1.17E-02
**ZCCHC18**	1.40	6.57E-03	1.17E-02
**FAM76A**	-0.49	6.57E-03	1.17E-02
**G11773**	1.81	6.57E-03	1.17E-02
**TOP2A**	-0.57	6.58E-03	1.17E-02
**UBC**	0.41	6.58E-03	1.17E-02
**FLVCR2**	0.35	6.58E-03	1.17E-02
**ZFX-AS1**	-3.35	6.58E-03	1.17E-02
**ZNF252P-AS1**	-1.31	6.58E-03	1.17E-02
**AC005757.6**	-3.56	6.58E-03	1.18E-02
**DNAJC27**	-0.39	6.59E-03	1.18E-02
**PNPLA4**	0.47	6.60E-03	1.18E-02
**AC096772.6**	-0.51	6.60E-03	1.18E-02
**RP11-3B7.1**	1.52	6.60E-03	1.18E-02
**LGR5**	-1.73	6.61E-03	1.18E-02
**XLOC_004201**	2.01	6.61E-03	1.18E-02
**SGK2**	-2.17	6.61E-03	1.18E-02
**ZNRF2P3**	-3.09	6.61E-03	1.18E-02
**EIF4HP1**	-0.92	6.62E-03	1.18E-02
**LINC01206**	3.56	6.62E-03	1.18E-02
**RP11-485G7.6**	-1.51	6.62E-03	1.18E-02

**CTC-367J11.1**	-3.31	6.62E-03	1.18E-02
**RP11-1012A1.7**	-3.29	6.62E-03	1.18E-02
**RP11-332H18.3**	-1.63	6.63E-03	1.18E-02
**RN7SL179P**	-3.43	6.63E-03	1.18E-02
**MIR378H**	-2.09	6.64E-03	1.18E-02
**AC104781.1**	-3.56	6.64E-03	1.18E-02
**RP11-638I2.10**	-3.51	6.64E-03	1.18E-02
**IL21R**	1.26	6.64E-03	1.18E-02
**COX7A2P1**	-2.27	6.65E-03	1.18E-02
**SEC23B**	-0.36	6.65E-03	1.19E-02
**CTC-459F4.6**	-2.21	6.66E-03	1.19E-02
**CTC-479C5.11**	-3.30	6.66E-03	1.19E-02
**AC010091.1**	-3.44	6.66E-03	1.19E-02
**PPP1R1AP1**	-3.41	6.67E-03	1.19E-02
**CAAP1**	-0.33	6.67E-03	1.19E-02
**ZNF202**	0.35	6.67E-03	1.19E-02
**RN7SL45P**	-3.40	6.68E-03	1.19E-02
**RP3-412A9.16**	-1.32	6.68E-03	1.19E-02
**RP11-407G23.7**	-1.44	6.68E-03	1.19E-02
**C1GALT1**	-0.39	6.68E-03	1.19E-02
**ENDOV**	0.45	6.68E-03	1.19E-02
**LBP**	8.17	6.68E-03	1.19E-02
**RP11-887P2.3**	1.04	6.68E-03	1.19E-02
**CTD-3096M3.2**	-2.61	6.69E-03	1.19E-02
**PTPRK**	-0.36	6.69E-03	1.19E-02
**CORO2B**	0.81	6.70E-03	1.19E-02
**AC009948.7**	-3.07	6.70E-03	1.19E-02
**RN7SL351P**	-3.49	6.70E-03	1.19E-02
**RP11-1072C15.4**	-1.83	6.70E-03	1.19E-02
**PTPN20CP**	-3.70	6.70E-03	1.19E-02
**EFCAB11**	0.41	6.71E-03	1.19E-02
**PI4K2B**	-0.51	6.71E-03	1.19E-02
**QKI**	-0.34	6.71E-03	1.19E-02
**AKIRIN1**	0.31	6.72E-03	1.20E-02
**MARS2**	-0.47	6.72E-03	1.20E-02
**RP11-80P20.3**	-0.77	6.72E-03	1.20E-02
**OPTN**	0.26	6.73E-03	1.20E-02
**LINC01090**	-3.41	6.73E-03	1.20E-02
**USPL1**	-0.34	6.74E-03	1.20E-02
**OR52T1P**	-3.38	6.74E-03	1.20E-02
**DDX27**	0.28	6.74E-03	1.20E-02
**7-Mar**	-0.45	6.74E-03	1.20E-02
**XLOC_005513**	-4.01	6.74E-03	1.20E-02
**RP1-45I4.2**	-3.25	6.75E-03	1.20E-02
**C19orf12**	0.25	6.75E-03	1.20E-02

**PTP4A2P2**	1.83	6.75E-03	1.20E-02
**RP11-146I2.1**	-1.47	6.76E-03	1.20E-02
**RP11-573G6.4**	-2.02	6.77E-03	1.20E-02
**CAMK1D**	-0.45	6.77E-03	1.20E-02
**C9orf163**	-1.33	6.77E-03	1.20E-02
**GNAI3**	-0.41	6.79E-03	1.21E-02
**OBP2B**	3.61	6.79E-03	1.21E-02
**CRNN**	1.08	6.79E-03	1.21E-02
**RP11-66N24.7**	-3.17	6.79E-03	1.21E-02
**GLI3**	0.42	6.79E-03	1.21E-02
**Z83826.1**	-1.50	6.80E-03	1.21E-02
**RP11-246E12.2**	-1.70	6.80E-03	1.21E-02
**SNX24**	-0.38	6.80E-03	1.21E-02
**LINC00211**	2.57	6.81E-03	1.21E-02
**CTC-435M10.10**	-3.15	6.81E-03	1.21E-02
**AC016831.6**	-3.26	6.82E-03	1.21E-02
**CTB-60B18.10**	1.84	6.82E-03	1.21E-02
**ATAD3B**	0.82	6.83E-03	1.21E-02
**EEF1A1P24**	-3.47	6.83E-03	1.21E-02
**MIR3680-1**	-2.70	6.84E-03	1.22E-02
**TAF5**	-0.35	6.85E-03	1.22E-02
**RLBP1**	-2.66	6.85E-03	1.22E-02
**HOXA-AS3**	-1.23	6.86E-03	1.22E-02
**IZUMO2**	-3.70	6.86E-03	1.22E-02
**RP11-147I3.1**	-1.28	6.87E-03	1.22E-02
**RP11-403P17.2**	-3.67	6.87E-03	1.22E-02
**SYT16**	-3.13	6.88E-03	1.22E-02
**RP5-849H19.2**	-2.27	6.88E-03	1.22E-02
**MEOX2**	0.84	6.88E-03	1.22E-02
**PCDHB16**	0.69	6.88E-03	1.22E-02
**RNF138**	-0.50	6.89E-03	1.22E-02
**G28332**	-1.23	6.89E-03	1.22E-02
**BRCC3P1**	-2.44	6.91E-03	1.23E-02
**XLOC_002943**	-1.38	6.91E-03	1.23E-02
**RP5-884G6.2**	0.91	6.91E-03	1.23E-02
**RNU4-87P**	-3.56	6.91E-03	1.23E-02
**RP11-214K3.18**	-3.38	6.91E-03	1.23E-02
**G39828**	1.76	6.91E-03	1.23E-02
**RP11-274J7.3**	-3.25	6.91E-03	1.23E-02
**RP11-1348G14.5**	-1.80	6.91E-03	1.23E-02
**UBXN7-AS1**	-3.23	6.92E-03	1.23E-02
**EFNA5**	-0.38	6.92E-03	1.23E-02
**AL158147.2**	-2.52	6.92E-03	1.23E-02
**RN7SKP179**	-3.25	6.92E-03	1.23E-02
**AC090286.4**	-3.19	6.93E-03	1.23E-02

**EGOT**	-1.58	6.93E-03	1.23E-02
**RP11-271K21.12**	-2.32	6.93E-03	1.23E-02
**SFPQ**	0.37	6.94E-03	1.23E-02
**AJAP1**	1.09	6.94E-03	1.23E-02
**RP11-98J23.2**	-1.60	6.94E-03	1.23E-02
**HMGB3P24**	-2.06	6.94E-03	1.23E-02
**HNRNPCP7**	-1.22	6.95E-03	1.23E-02
**RP11-121L10.2**	-1.95	6.96E-03	1.23E-02
**LRGUK**	-1.02	6.97E-03	1.24E-02
**RPL36AP41**	-3.38	6.97E-03	1.24E-02
**PP7080**	0.38	6.97E-03	1.24E-02
**RP11-504P24.5**	-3.34	6.97E-03	1.24E-02
**RP11-443O13.3**	-2.07	6.98E-03	1.24E-02
**AL121952.1**	-3.55	6.98E-03	1.24E-02
**RP4-800M22.4**	-3.87	6.98E-03	1.24E-02
**RP11-141M1.4**	-3.24	6.99E-03	1.24E-02
**HDGFP1**	-3.37	6.99E-03	1.24E-02
**OR9Q1**	-2.79	7.01E-03	1.24E-02
**FGF9**	1.53	7.01E-03	1.24E-02
**WISP2**	1.48	7.01E-03	1.24E-02
**FGF1**	1.37	7.02E-03	1.24E-02
**AC116533.1**	-3.56	7.02E-03	1.24E-02
**CTC-510F12.3**	2.52	7.02E-03	1.24E-02
**RAB44**	-1.21	7.02E-03	1.24E-02
**PARG**	-0.37	7.02E-03	1.24E-02
**MIR548AA1**	-3.69	7.02E-03	1.24E-02
**RP11-54A4.2**	-1.91	7.03E-03	1.25E-02
**PHBP9**	-1.13	7.03E-03	1.25E-02
**AC017083.1**	-3.18	7.04E-03	1.25E-02
**SHBG**	2.83	7.04E-03	1.25E-02
**IGF2-AS**	5.39	7.05E-03	1.25E-02
**MOAP1**	0.34	7.05E-03	1.25E-02
**RP11-467J12.2**	-3.24	7.05E-03	1.25E-02
**HACL1**	-0.66	7.06E-03	1.25E-02
**EVPL**	0.45	7.07E-03	1.25E-02
**RP11-616M22.3**	-3.55	7.07E-03	1.25E-02
**RP11-56B16.5**	-2.26	7.07E-03	1.25E-02
**SLC22A6**	-3.29	7.07E-03	1.25E-02
**RP11-381E24.1**	-1.54	7.08E-03	1.25E-02
**NCBP3**	-0.27	7.08E-03	1.25E-02
**RNU7-71P**	-3.79	7.08E-03	1.25E-02
**USP51**	0.53	7.09E-03	1.25E-02
**CTD-2281E23.1**	-3.57	7.09E-03	1.26E-02
**CALCRL**	0.73	7.09E-03	1.26E-02
**RP11-412D9.4**	1.10	7.10E-03	1.26E-02

**XLOC_013181**	-1.14	7.10E-03	1.26E-02
**RP11-266K4.9**	-1.15	7.10E-03	1.26E-02
**VN1R84P**	-3.98	7.12E-03	1.26E-02
**MIR3909**	-3.37	7.12E-03	1.26E-02
**G7957**	-3.40	7.12E-03	1.26E-02
**RNU6-658P**	-2.84	7.13E-03	1.26E-02
**GS1-174L6.4**	3.14	7.13E-03	1.26E-02
**LINC00517**	3.06	7.13E-03	1.26E-02
**LINC00866**	2.28	7.15E-03	1.27E-02
**METTL2A**	-0.34	7.16E-03	1.27E-02
**G9801**	-1.47	7.16E-03	1.27E-02
**RN7SL696P**	-3.26	7.17E-03	1.27E-02
**RPL23AP4**	-3.62	7.17E-03	1.27E-02
**EZR-AS1**	-3.08	7.17E-03	1.27E-02
**TARSL2**	0.40	7.18E-03	1.27E-02
**AC007091.1**	-3.38	7.18E-03	1.27E-02
**RP11-212I21.4**	-1.17	7.20E-03	1.27E-02
**SPDYE12P**	1.21	7.21E-03	1.27E-02
**RN7SKP178**	-3.87	7.21E-03	1.27E-02
**RP11-370I10.11**	-2.39	7.21E-03	1.27E-02
**RP11-146D12.2**	-2.30	7.21E-03	1.27E-02
**ADGRV1**	-1.16	7.22E-03	1.28E-02
**ATP8A2**	1.22	7.22E-03	1.28E-02
**RP11-421M1.8**	-1.30	7.22E-03	1.28E-02
**KBTBD11**	1.06	7.22E-03	1.28E-02
**MED14**	-0.33	7.23E-03	1.28E-02
**SPCS1**	0.42	7.23E-03	1.28E-02
**LINC00482**	1.67	7.24E-03	1.28E-02
**LRRTM4**	3.25	7.24E-03	1.28E-02
**RP11-793H13.3**	-3.31	7.25E-03	1.28E-02
**RP11-690I21.1**	-2.62	7.25E-03	1.28E-02
**RN7SKP184**	-3.23	7.25E-03	1.28E-02
**RP5-1142J19.2**	-2.18	7.25E-03	1.28E-02
**GOLT1A**	2.45	7.25E-03	1.28E-02
**CTD-2161E19.1**	-2.99	7.25E-03	1.28E-02
**RNU6-583P**	-3.65	7.26E-03	1.28E-02
**G25496**	-1.10	7.26E-03	1.28E-02
**RPSAP36**	-1.52	7.26E-03	1.28E-02
**RP11-299M14.2**	-3.52	7.26E-03	1.28E-02
**RP11-585F1.6**	-3.45	7.26E-03	1.28E-02
**RECQL**	-0.42	7.28E-03	1.28E-02
**SYT12**	0.97	7.28E-03	1.29E-02
**RP11-272L13.2**	-3.63	7.29E-03	1.29E-02
**RNU6-881P**	-3.36	7.30E-03	1.29E-02
**RNU6-1127P**	-3.56	7.30E-03	1.29E-02

**IPO5**	-0.27	7.31E-03	1.29E-02
**GLTSCR2-AS1**	-1.32	7.31E-03	1.29E-02
**ANKS4B**	-3.75	7.32E-03	1.29E-02
**ARID3B**	0.49	7.32E-03	1.29E-02
**AL355794.1**	-3.82	7.33E-03	1.29E-02
**AC002064.5**	-3.49	7.33E-03	1.29E-02
**MARS**	0.36	7.33E-03	1.29E-02
**LINC00162**	0.80	7.33E-03	1.29E-02
**MIR1972-2**	-3.51	7.33E-03	1.29E-02
**RN7SKP37**	-3.83	7.34E-03	1.29E-02
**MIR4419A**	-3.33	7.35E-03	1.30E-02
**ZNF155**	-0.64	7.35E-03	1.30E-02
**XLOC_012436**	-1.68	7.35E-03	1.30E-02
**RP11-616K22.2**	-3.12	7.37E-03	1.30E-02
**NDUFB6**	0.53	7.37E-03	1.30E-02
**ANTXR2**	0.97	7.38E-03	1.30E-02
**SEL1L3**	0.54	7.38E-03	1.30E-02
**MRGPRX3**	3.57	7.39E-03	1.30E-02
**MIR661**	-3.58	7.39E-03	1.30E-02
**METTL1**	0.64	7.39E-03	1.30E-02
**DYNC1LI1**	-0.43	7.39E-03	1.30E-02
**TIGD3**	1.46	7.40E-03	1.31E-02
**RP11-136F16.2**	-3.36	7.40E-03	1.31E-02
**RP11-324F21.1**	-3.42	7.41E-03	1.31E-02
**MEDAG**	1.17	7.41E-03	1.31E-02
**FAM182B**	0.91	7.41E-03	1.31E-02
**STX17-AS1**	-0.81	7.41E-03	1.31E-02
**XLOC_011484**	-2.36	7.42E-03	1.31E-02
**LILRA4**	-2.22	7.42E-03	1.31E-02
**PPP2R2C**	0.55	7.42E-03	1.31E-02
**PTPRS**	-0.35	7.42E-03	1.31E-02
**RP11-798L4.1**	-2.31	7.43E-03	1.31E-02
**RP3-337O18.9**	2.78	7.43E-03	1.31E-02
**RP11-568K15.2**	-2.14	7.43E-03	1.31E-02
**FAR1-IT1**	-2.67	7.44E-03	1.31E-02
**RP11-747H12.5**	-3.88	7.44E-03	1.31E-02
**RP11-50I19.1**	-3.29	7.44E-03	1.31E-02
**XLOC_009885**	-2.25	7.45E-03	1.31E-02
**PRR22**	1.07	7.45E-03	1.31E-02
**AURKAPS1**	-2.45	7.45E-03	1.31E-02
**CDK11A**	-1.20	7.46E-03	1.32E-02
**LINC01170**	1.59	7.47E-03	1.32E-02
**OR2G6**	-4.14	7.48E-03	1.32E-02
**G14803**	-2.52	7.49E-03	1.32E-02
**RP11-96C23.10**	-3.49	7.50E-03	1.32E-02

**RP5-1101C3.1**	-1.86	7.52E-03	1.32E-02
**CORIN**	-0.88	7.52E-03	1.33E-02
**NTSR1**	1.42	7.52E-03	1.33E-02
**ARL17A**	-0.94	7.53E-03	1.33E-02
**RNU7-119P**	-3.84	7.54E-03	1.33E-02
**EHMT1**	0.28	7.54E-03	1.33E-02
**AC083843.2**	-2.93	7.55E-03	1.33E-02
**TIA1**	-0.37	7.56E-03	1.33E-02
**BMS1P9**	1.85	7.56E-03	1.33E-02
**MYEOV**	1.17	7.57E-03	1.33E-02
**RPS26P3**	-3.11	7.57E-03	1.33E-02
**LINC00992**	1.04	7.57E-03	1.33E-02
**RP11-570L14.1**	-3.81	7.58E-03	1.33E-02
**AC073283.7**	-2.09	7.58E-03	1.33E-02
**MTND1P3**	-2.99	7.59E-03	1.34E-02
**CICP16**	-1.52	7.61E-03	1.34E-02
**BVES**	0.89	7.62E-03	1.34E-02
**IFT81**	-0.36	7.62E-03	1.34E-02
**RP11-707P17.1**	-3.62	7.63E-03	1.34E-02
**DIS3L**	-0.38	7.63E-03	1.34E-02
**GS1-393G12.12**	1.22	7.64E-03	1.34E-02
**TMEM167B**	-0.42	7.65E-03	1.35E-02
**OR5K2**	-3.47	7.65E-03	1.35E-02
**HOXA9**	1.07	7.66E-03	1.35E-02
**PLA2R1**	-0.79	7.66E-03	1.35E-02
**RP11-753H16.3**	-3.11	7.67E-03	1.35E-02
**GAS8-AS1**	-2.33	7.67E-03	1.35E-02
**FAM96AP2**	1.18	7.67E-03	1.35E-02
**PIP5K1A**	-0.29	7.67E-03	1.35E-02
**CTD-3065B20.3**	-1.91	7.67E-03	1.35E-02
**AC079250.1**	-0.82	7.67E-03	1.35E-02
**A1CF**	-2.73	7.68E-03	1.35E-02
**FOXK2**	0.27	7.68E-03	1.35E-02
**RPSAP50**	-3.68	7.68E-03	1.35E-02
**ALG1L10P**	-1.65	7.68E-03	1.35E-02
**TRIM45**	-0.73	7.69E-03	1.35E-02
**RP5-1028L10.1**	-3.65	7.69E-03	1.35E-02
**RN7SL220P**	-1.46	7.70E-03	1.35E-02
**RP11-159F24.5**	-1.73	7.71E-03	1.35E-02
**CDK20**	0.59	7.72E-03	1.36E-02
**C2orf82**	1.32	7.72E-03	1.36E-02
**SERPINB9**	1.31	7.72E-03	1.36E-02
**RP11-112L18.1**	-3.41	7.72E-03	1.36E-02
**SCAND2P**	-0.66	7.73E-03	1.36E-02
**SLC6A14**	1.14	7.74E-03	1.36E-02

**CCDC114**	-1.11	7.74E-03	1.36E-02
**RP11-33O4.2**	-2.06	7.75E-03	1.36E-02
**RP11-173B14.4**	-1.29	7.75E-03	1.36E-02
**BEND7P1**	-3.38	7.75E-03	1.36E-02
**RP11-304C12.5**	-1.79	7.75E-03	1.36E-02
**ERP29**	0.45	7.76E-03	1.36E-02
**RP11-17G2.1**	-3.95	7.76E-03	1.36E-02
**SPATC1L**	1.44	7.77E-03	1.36E-02
**CIART**	1.68	7.79E-03	1.37E-02
**CHCHD3**	0.27	7.79E-03	1.37E-02
**RAB30**	-0.43	7.80E-03	1.37E-02
**RP11-121M22.1**	-1.24	7.80E-03	1.37E-02
**MSI1**	1.80	7.80E-03	1.37E-02
**KSR2**	-1.28	7.81E-03	1.37E-02
**RP11-382A20.4**	-3.38	7.81E-03	1.37E-02
**RP11-10J21.6**	-3.18	7.81E-03	1.37E-02
**TXNRD3**	-0.33	7.82E-03	1.37E-02
**RN7SKP110**	-3.60	7.82E-03	1.37E-02
**CACNA1D**	-0.76	7.83E-03	1.37E-02
**RP4-561L24.3**	-1.09	7.84E-03	1.38E-02
**PRMT3**	-0.38	7.84E-03	1.38E-02
**KB-1572G7.3**	-3.29	7.84E-03	1.38E-02
**TIGAR**	0.63	7.84E-03	1.38E-02
**SUV420H2**	0.68	7.84E-03	1.38E-02
**XLOC_005280**	-3.50	7.84E-03	1.38E-02
**MFAP5**	-1.26	7.85E-03	1.38E-02
**AC007131.2**	-3.72	7.86E-03	1.38E-02
**G29539**	1.77	7.86E-03	1.38E-02
**ST7**	-0.30	7.86E-03	1.38E-02
**MESTP1**	-3.68	7.87E-03	1.38E-02
**RP11-573G6.6**	-1.98	7.87E-03	1.38E-02
**MFN2**	0.26	7.87E-03	1.38E-02
**AC003088.1**	-3.52	7.87E-03	1.38E-02
**ILDR2**	-1.70	7.87E-03	1.38E-02
**LRRN2**	1.41	7.88E-03	1.38E-02
**EFCAB8**	-1.90	7.88E-03	1.38E-02
**PIK3C2B**	-0.44	7.88E-03	1.38E-02
**RPLP2**	0.44	7.88E-03	1.38E-02
**RP3-400B16.2**	-3.53	7.89E-03	1.38E-02
**CCL3**	4.02	7.89E-03	1.38E-02
**TGFBR3L**	1.17	7.90E-03	1.38E-02
**ARL6IP6**	-0.41	7.92E-03	1.39E-02
**KRT10**	1.04	7.93E-03	1.39E-02
**RP11-1094M14.8**	1.46	7.93E-03	1.39E-02
**AC097467.2**	-3.71	7.93E-03	1.39E-02

**CST6**	-0.90	7.93E-03	1.39E-02
**RNU6-301P**	-2.19	7.93E-03	1.39E-02
**CTA-407F11.8**	-2.61	7.94E-03	1.39E-02
**RP11-336A10.4**	2.77	7.94E-03	1.39E-02
**G26249**	-1.90	7.95E-03	1.39E-02
**RIMBP3**	1.61	7.96E-03	1.39E-02
**KRTAP1-3**	-6.19	7.99E-03	1.40E-02
**ACTL10**	0.75	7.99E-03	1.40E-02
**IMMT**	0.22	8.00E-03	1.40E-02
**GLYATL1P1**	-2.21	8.00E-03	1.40E-02
**CTD-2568A17.1**	-2.69	8.00E-03	1.40E-02
**RP11-286H14.8**	-1.50	8.01E-03	1.40E-02
**RP11-7F18.2**	-1.38	8.01E-03	1.40E-02
**GJA1**	0.59	8.02E-03	1.40E-02
**KLK8**	0.57	8.02E-03	1.40E-02
**RNU7-11P**	-3.67	8.02E-03	1.40E-02
**CTC-499B15.8**	-1.76	8.02E-03	1.40E-02
**RP11-98D18.1**	-3.50	8.04E-03	1.41E-02
**NNAT**	-0.91	8.04E-03	1.41E-02
**MPTX1**	-3.14	8.05E-03	1.41E-02
**CTB-78O21.1**	-2.36	8.05E-03	1.41E-02
**MRO**	1.94	8.05E-03	1.41E-02
**MIP**	-2.36	8.05E-03	1.41E-02
**AL603965.1**	-1.33	8.06E-03	1.41E-02
**GCLM**	0.62	8.06E-03	1.41E-02
**RP11-768G7.2**	-3.86	8.06E-03	1.41E-02
**RP4-735C1.4**	-2.46	8.06E-03	1.41E-02
**CTD-2231E14.5**	-3.27	8.06E-03	1.41E-02
**AC007880.1**	-3.19	8.07E-03	1.41E-02
**TTC21A**	0.74	8.07E-03	1.41E-02
**NCAPG2**	-0.39	8.07E-03	1.41E-02
**RP11-87E22.2**	-3.43	8.08E-03	1.41E-02
**ALDH3A1**	0.90	8.08E-03	1.41E-02
**RP11-356O9.1**	-3.12	8.09E-03	1.42E-02
**RPL18AP2**	-3.28	8.10E-03	1.42E-02
**RP11-295H24.3**	-3.50	8.10E-03	1.42E-02
**FOXP1-AS1**	-1.70	8.11E-03	1.42E-02
**CYCSP10**	-1.81	8.11E-03	1.42E-02
**RP11-16P20.4**	-3.04	8.11E-03	1.42E-02
**RP11-1021O19.2**	-3.38	8.11E-03	1.42E-02
**RP11-356O9.2**	-3.77	8.12E-03	1.42E-02
**MIR200B**	-2.84	8.12E-03	1.42E-02
**RNU6-689P**	-3.71	8.13E-03	1.42E-02
**RP11-770J1.7**	-3.25	8.13E-03	1.42E-02
**AP3B2**	0.86	8.14E-03	1.42E-02

**RRP8**	-0.27	8.15E-03	1.42E-02
**PTGER3**	-0.74	8.15E-03	1.42E-02
**RNU6-897P**	-3.58	8.15E-03	1.42E-02
**SIGMAR1**	0.35	8.15E-03	1.42E-02
**RPL21P110**	-3.49	8.17E-03	1.43E-02
**ZNF239**	1.27	8.17E-03	1.43E-02
**GNPAT**	-0.50	8.18E-03	1.43E-02
**RP1-12G14.7**	-0.76	8.19E-03	1.43E-02
**RABGAP1**	-0.27	8.20E-03	1.43E-02
**MMP21**	-1.92	8.20E-03	1.43E-02
**RP11-85O21.2**	-3.52	8.21E-03	1.43E-02
**LRRC37A17P**	-0.63	8.21E-03	1.43E-02
**PCDHB18P**	0.60	8.22E-03	1.44E-02
**RP11-284J1.1**	-2.37	8.22E-03	1.44E-02
**RP11-76C10.5**	-3.46	8.22E-03	1.44E-02
**RP11-770J1.5**	-3.60	8.22E-03	1.44E-02
**RP11-394O4.5**	1.41	8.23E-03	1.44E-02
**VPS4B**	-0.37	8.23E-03	1.44E-02
**ADAD1P2**	-3.45	8.23E-03	1.44E-02
**RP11-432I5.4**	-3.43	8.24E-03	1.44E-02
**ST13P11**	-2.71	8.24E-03	1.44E-02
**RP11-731D1.3**	-3.39	8.25E-03	1.44E-02
**PRMT5**	0.37	8.25E-03	1.44E-02
**RP11-626H12.2**	4.81	8.25E-03	1.44E-02
**AL049539.1**	-3.22	8.27E-03	1.44E-02
**RPGRIP1**	-0.85	8.28E-03	1.45E-02
**RFT1**	-0.24	8.29E-03	1.45E-02
**RP11-555K12.1**	-2.04	8.30E-03	1.45E-02
**PEPD**	0.43	8.30E-03	1.45E-02
**B3GNT4**	0.55	8.30E-03	1.45E-02
**RP11-710E1.2**	-3.10	8.30E-03	1.45E-02
**RN7SL857P**	-3.66	8.30E-03	1.45E-02
**SNORA18**	-3.23	8.31E-03	1.45E-02
**MIR593**	-3.55	8.31E-03	1.45E-02
**STRC**	-1.38	8.32E-03	1.45E-02
**AL353763.1**	-3.75	8.32E-03	1.45E-02
**AC009229.6**	-2.34	8.33E-03	1.45E-02
**RP11-368N21.5**	1.29	8.33E-03	1.45E-02
**RP11-762I7.4**	-1.29	8.33E-03	1.45E-02
**G36172**	-1.26	8.33E-03	1.45E-02
**RP11-85I21.1**	-2.95	8.34E-03	1.45E-02
**YPEL5P2**	-2.18	8.34E-03	1.45E-02
**RP11-88G17.6**	-3.22	8.35E-03	1.46E-02
**RP11-44F21.4**	-3.81	8.35E-03	1.46E-02
**CTD-2245F17.3**	-0.84	8.35E-03	1.46E-02

**RP11-1002K11.1**	-0.69	8.35E-03	1.46E-02
**GDE1**	0.43	8.35E-03	1.46E-02
**RP11-316J7.4**	-3.66	8.36E-03	1.46E-02
**TRAM2-AS1**	0.66	8.37E-03	1.46E-02
**RP11-417N10.3**	-2.99	8.37E-03	1.46E-02
**G5109**	-2.04	8.38E-03	1.46E-02
**RP11-272P10.2**	-3.16	8.38E-03	1.46E-02
**NSUN7**	-0.62	8.38E-03	1.46E-02
**RNU6-100P**	-3.06	8.38E-03	1.46E-02
**RNU2-39P**	-3.22	8.39E-03	1.46E-02
**RPL38**	0.43	8.39E-03	1.46E-02
**ST18**	-2.07	8.40E-03	1.46E-02
**EXOG**	0.71	8.42E-03	1.47E-02
**RP11-144G7.2**	-2.39	8.42E-03	1.47E-02
**RP11-476C8.2**	-2.21	8.42E-03	1.47E-02
**LYG1**	-1.57	8.42E-03	1.47E-02
**IFIT1P1**	-3.28	8.42E-03	1.47E-02
**FAM81B**	-1.84	8.43E-03	1.47E-02
**RNU6-541P**	-3.25	8.43E-03	1.47E-02
**RP11-56F10.3**	-2.90	8.43E-03	1.47E-02
**CFAP58**	0.77	8.43E-03	1.47E-02
**XLOC_003663**	-1.55	8.43E-03	1.47E-02
**TUBB2B**	0.95	8.44E-03	1.47E-02
**RP11-235P11.1**	-3.65	8.44E-03	1.47E-02
**CD226**	-1.12	8.45E-03	1.47E-02
**RP11-211A18.1**	-3.47	8.45E-03	1.47E-02
**RP11-1078H9.2**	-3.60	8.46E-03	1.47E-02
**RPL31P2**	-3.76	8.47E-03	1.47E-02
**RP11-322E11.3**	-2.71	8.48E-03	1.48E-02
**PCNPP1**	-1.30	8.48E-03	1.48E-02
**HRK**	2.18	8.49E-03	1.48E-02
**CTD-2568A17.8**	-2.33	8.49E-03	1.48E-02
**CXCL5**	2.67	8.49E-03	1.48E-02
**LINC00240**	-1.21	8.49E-03	1.48E-02
**CHKB-AS1**	1.20	8.50E-03	1.48E-02
**RP11-662B19.2**	-3.04	8.50E-03	1.48E-02
**XLOC_005392**	-3.40	8.50E-03	1.48E-02
**MSH5**	-1.14	8.51E-03	1.48E-02
**RP11-351I21.7**	-3.10	8.51E-03	1.48E-02
**RP11-1060J15.4**	-3.46	8.51E-03	1.48E-02
**DDX24**	0.23	8.51E-03	1.48E-02
**AC025442.3**	-3.28	8.51E-03	1.48E-02
**NUBPL**	-0.33	8.51E-03	1.48E-02
**CTD-2555K7.2**	-2.24	8.52E-03	1.48E-02
**RP11-426K3.1**	-2.44	8.52E-03	1.48E-02

**LPGAT1**	-0.37	8.53E-03	1.48E-02
**XLOC_013942**	-3.56	8.53E-03	1.48E-02
**ANKLE2**	0.23	8.54E-03	1.49E-02
**PSMD5-AS1**	0.64	8.54E-03	1.49E-02
**RP11-49C9.2**	-3.62	8.55E-03	1.49E-02
**PROM1**	2.14	8.56E-03	1.49E-02
**CTD-2616J11.16**	-3.42	8.56E-03	1.49E-02
**WWTR1**	0.38	8.56E-03	1.49E-02
**C21orf2**	0.57	8.56E-03	1.49E-02
**CCDC142**	0.63	8.57E-03	1.49E-02
**GLUD1P2**	1.38	8.58E-03	1.49E-02
**CTD-2547H18.1**	-3.14	8.60E-03	1.49E-02
**RRS1-AS1**	-1.50	8.60E-03	1.50E-02
**HNRNPA1P1**	-3.33	8.61E-03	1.50E-02
**AGAP1-IT1**	-2.11	8.61E-03	1.50E-02
**RP11-380M21.4**	-1.35	8.61E-03	1.50E-02
**KRT77**	-1.01	8.62E-03	1.50E-02
**LAMTOR3**	-0.44	8.62E-03	1.50E-02
**XLOC_011151**	-3.11	8.62E-03	1.50E-02
**QARS**	0.34	8.63E-03	1.50E-02
**THSD7B**	-1.81	8.63E-03	1.50E-02
**RP11-762I7.5**	-2.75	8.63E-03	1.50E-02
**GAPDHP2**	-1.93	8.63E-03	1.50E-02
**KCNC1**	1.63	8.64E-03	1.50E-02
**RP4-665J23.1**	1.14	8.64E-03	1.50E-02
**RP11-308D16.1**	-3.54	8.65E-03	1.50E-02
**COL4A3**	1.18	8.66E-03	1.50E-02
**RP5-933K21.3**	1.66	8.66E-03	1.50E-02
**HOXB3**	0.91	8.66E-03	1.50E-02
**RN7SKP115**	-3.34	8.66E-03	1.50E-02
**SLC6A17**	-0.91	8.66E-03	1.50E-02
**AL590431.1**	-1.24	8.66E-03	1.50E-02
**G11315**	-3.86	8.66E-03	1.50E-02
**CSMD1**	-1.26	8.67E-03	1.51E-02
**IQUB**	-1.14	8.67E-03	1.51E-02
**RP11-166P13.3**	0.63	8.68E-03	1.51E-02
**SETD1B**	0.67	8.68E-03	1.51E-02
**ZNF608**	0.70	8.69E-03	1.51E-02
**ZNF730**	-2.02	8.69E-03	1.51E-02
**SNHG4**	-0.66	8.69E-03	1.51E-02
**ADGRE1**	2.82	8.69E-03	1.51E-02
**LGALS7B**	0.95	8.69E-03	1.51E-02
**RP11-212F11.1**	-3.07	8.70E-03	1.51E-02
**RP11-165M1.3**	-3.17	8.70E-03	1.51E-02
**ACTR2**	0.36	8.70E-03	1.51E-02

**RP11-66B24.7**	2.45	8.71E-03	1.51E-02
**NLRP9**	-3.39	8.71E-03	1.51E-02
**RFC3**	-0.37	8.71E-03	1.51E-02
**AL138498.1**	-3.64	8.71E-03	1.51E-02
**CELSR3-AS1**	1.60	8.71E-03	1.51E-02
**RNU5F-3P**	-3.50	8.71E-03	1.51E-02
**MID1**	0.47	8.72E-03	1.51E-02
**CTD-2319I12.10**	-2.65	8.72E-03	1.51E-02
**AC091801.1**	2.76	8.72E-03	1.51E-02
**RP13-228J13.6**	-3.25	8.73E-03	1.51E-02
**RP11-274E6.1**	-3.78	8.73E-03	1.51E-02
**LA16c-OS12.2**	2.01	8.74E-03	1.51E-02
**XLOC_011523**	-2.65	8.74E-03	1.52E-02
**THUMPD2**	0.31	8.75E-03	1.52E-02
**LRRCC1**	-0.51	8.75E-03	1.52E-02
**CEACAMP10**	-2.59	8.76E-03	1.52E-02
**RP11-256L6.3**	-1.42	8.76E-03	1.52E-02
**CYP4F12**	-0.72	8.77E-03	1.52E-02
**DMC1**	-0.77	8.78E-03	1.52E-02
**RP11-727A23.4**	-1.21	8.78E-03	1.52E-02
**GABRB1**	-3.27	8.79E-03	1.52E-02
**AC079781.7**	-3.04	8.79E-03	1.52E-02
**OR7E38P**	-0.67	8.79E-03	1.52E-02
**RP11-91J3.3**	5.11	8.79E-03	1.52E-02
**XLOC_002773**	-3.52	8.80E-03	1.53E-02
**UQCR10**	0.41	8.80E-03	1.53E-02
**RP11-350N15.5**	0.80	8.80E-03	1.53E-02
**SEMA6A**	0.62	8.80E-03	1.53E-02
**GS1-293C5.1**	-3.48	8.81E-03	1.53E-02
**MTCO1P29**	-3.51	8.81E-03	1.53E-02
**IRAK4**	-0.30	8.82E-03	1.53E-02
**RP11-587D21.1**	-1.62	8.83E-03	1.53E-02
**RP11-2I17.1**	-3.74	8.83E-03	1.53E-02
**BUD13P1**	-0.96	8.83E-03	1.53E-02
**EIF2B5**	0.25	8.83E-03	1.53E-02
**ARL8B**	-0.37	8.84E-03	1.53E-02
**SNORD19**	-1.31	8.84E-03	1.53E-02
**OR7E89P**	-2.67	8.85E-03	1.53E-02
**GPR39**	-1.07	8.86E-03	1.53E-02
**VIT**	1.12	8.86E-03	1.53E-02
**SMIM15**	-0.41	8.88E-03	1.54E-02
**IMPDH2**	0.39	8.89E-03	1.54E-02
**SERF1B**	1.12	8.89E-03	1.54E-02
**KDM4A**	-0.21	8.90E-03	1.54E-02
**CHST12**	0.41	8.90E-03	1.54E-02

**NOP58**	0.33	8.91E-03	1.54E-02
**RN7SKP244**	-3.35	8.91E-03	1.54E-02
**RP11-540O11.4**	-2.98	8.91E-03	1.54E-02
**EIF4A1P11**	-3.34	8.92E-03	1.54E-02
**TRPC3**	2.15	8.93E-03	1.54E-02
**RP3-405J10.2**	1.64	8.93E-03	1.54E-02
**CROCC2**	2.44	8.93E-03	1.54E-02
**ENTPD3-AS1**	-0.69	8.93E-03	1.54E-02
**TRAJ14**	-3.19	8.93E-03	1.54E-02
**XLOC_002712**	-3.81	8.93E-03	1.54E-02
**DUSP7**	0.46	8.93E-03	1.55E-02
**KDM2A**	0.27	8.94E-03	1.55E-02
**TXLNG**	-0.34	8.94E-03	1.55E-02
**RP11-455F5.4**	-3.19	8.94E-03	1.55E-02
**SNX18P15**	-3.47	8.95E-03	1.55E-02
**RP11-345P4.6**	-1.72	8.96E-03	1.55E-02
**CTD-2349P21.6**	-2.04	8.96E-03	1.55E-02
**HEPHL1**	2.01	8.97E-03	1.55E-02
**GPR160**	0.78	8.98E-03	1.55E-02
**CHMP2B**	-0.44	8.99E-03	1.55E-02
**CTA-440B3.1**	-3.29	8.99E-03	1.55E-02
**FOXJ1**	-0.86	9.00E-03	1.56E-02
**SPRR2E**	1.13	9.00E-03	1.56E-02
**ABCB6**	-1.34	9.01E-03	1.56E-02
**RP11-411B10.8**	-3.08	9.01E-03	1.56E-02
**SMARCA1**	0.45	9.02E-03	1.56E-02
**RP11-669E14.6**	-1.35	9.02E-03	1.56E-02
**CCNB3**	-0.74	9.03E-03	1.56E-02
**AP000857.3**	-3.52	9.03E-03	1.56E-02
**CTD-3222D19.2**	-3.44	9.03E-03	1.56E-02
**MT1JP**	4.30	9.03E-03	1.56E-02
**GRM1**	-1.52	9.04E-03	1.56E-02
**LINC00659**	2.57	9.05E-03	1.56E-02
**CHODL**	-0.67	9.05E-03	1.56E-02
**RP11-697E22.2**	-2.96	9.06E-03	1.57E-02
**RN7SL739P**	-3.58	9.07E-03	1.57E-02
**GPATCH1**	-0.30	9.07E-03	1.57E-02
**RP11-597M17.3**	-3.40	9.08E-03	1.57E-02
**NPIPB3**	1.29	9.09E-03	1.57E-02
**CADM3**	-1.07	9.10E-03	1.57E-02
**RP11-315D16.4**	-2.53	9.11E-03	1.57E-02
**UBE2E1-AS1**	2.39	9.11E-03	1.57E-02
**COX6B1P3**	-3.25	9.11E-03	1.57E-02
**RP11-212I21.3**	-2.16	9.11E-03	1.57E-02
**SIAH1**	0.35	9.11E-03	1.57E-02

**XLOC_003405**	-1.04	9.12E-03	1.57E-02
**C20orf203**	-1.73	9.12E-03	1.57E-02
**RPL21**	0.43	9.12E-03	1.57E-02
**RPS6P22**	-3.69	9.12E-03	1.58E-02
**SUN2**	0.36	9.13E-03	1.58E-02
**MED28P1**	-2.90	9.13E-03	1.58E-02
**CMPK2**	1.44	9.13E-03	1.58E-02
**RNA5SP195**	-2.98	9.14E-03	1.58E-02
**RNU6-514P**	-3.33	9.14E-03	1.58E-02
**SAP30L**	-0.25	9.14E-03	1.58E-02
**NMT2**	0.32	9.15E-03	1.58E-02
**PPP1R3C**	0.92	9.15E-03	1.58E-02
**C16orf46**	1.24	9.15E-03	1.58E-02
**AGGF1**	-0.31	9.16E-03	1.58E-02
**RP11-532F6.5**	-2.56	9.16E-03	1.58E-02
**CBR1**	0.56	9.16E-03	1.58E-02
**PPFIA3**	0.50	9.16E-03	1.58E-02
**HCG11**	-0.58	9.18E-03	1.58E-02
**MUC21**	-4.25	9.19E-03	1.58E-02
**PIGX**	0.37	9.19E-03	1.59E-02
**MIR505**	-3.37	9.19E-03	1.59E-02
**AC007881.4**	-3.44	9.20E-03	1.59E-02
**GFRA2**	1.05	9.20E-03	1.59E-02
**LINC01572**	-0.89	9.21E-03	1.59E-02
**RAVER2**	-0.34	9.21E-03	1.59E-02
**OVOL3**	-1.81	9.22E-03	1.59E-02
**RP11-468E2.10**	-1.02	9.22E-03	1.59E-02
**EIF3A**	0.29	9.22E-03	1.59E-02
**RP4-765C7.2**	-3.16	9.23E-03	1.59E-02
**RP11-1099M24.9**	-3.13	9.23E-03	1.59E-02
**CTB-58E17.2**	-3.20	9.23E-03	1.59E-02
**TMPRSS11E**	-1.60	9.23E-03	1.59E-02
**MALSU1**	0.30	9.24E-03	1.59E-02
**RP3-475N16.1**	-0.86	9.24E-03	1.59E-02
**RP11-1096G20.5**	-1.41	9.24E-03	1.59E-02
**WI2-89927D4.1**	-3.60	9.24E-03	1.59E-02
**ATP6V1E2**	0.52	9.25E-03	1.59E-02
**CTD-2373J6.2**	-1.82	9.25E-03	1.59E-02
**RP11-230C9.2**	-1.24	9.27E-03	1.60E-02
**RN7SL49P**	-2.61	9.27E-03	1.60E-02
**ABCA6**	-1.03	9.28E-03	1.60E-02
**PARP11**	0.44	9.28E-03	1.60E-02
**RP11-76G10.1**	-3.50	9.28E-03	1.60E-02
**CTD-2384B9.1**	-3.50	9.29E-03	1.60E-02
**RP11-119J18.1**	-1.08	9.29E-03	1.60E-02

**RP4-769N13.7**	-2.37	9.30E-03	1.60E-02
**RNU6-1048P**	-1.98	9.30E-03	1.60E-02
**XLOC_013950**	1.66	9.31E-03	1.60E-02
**FAM95B1**	-3.30	9.31E-03	1.60E-02
**CICP3**	-3.23	9.31E-03	1.60E-02
**ZNF330**	0.25	9.31E-03	1.60E-02
**RP11-63I8.1**	-2.63	9.32E-03	1.60E-02
**RP11-247I13.11**	-2.86	9.32E-03	1.60E-02
**SCN3A**	-0.98	9.33E-03	1.61E-02
**RP11-10O17.1**	-1.33	9.33E-03	1.61E-02
**GALNT12**	0.74	9.33E-03	1.61E-02
**RP11-90J7.3**	-2.88	9.33E-03	1.61E-02
**PLCB1**	0.82	9.34E-03	1.61E-02
**RP4-809F4.1**	-3.31	9.36E-03	1.61E-02
**LLPH**	-0.29	9.36E-03	1.61E-02
**RP11-782C8.3**	-3.12	9.36E-03	1.61E-02
**NDUFV2-AS1**	-0.52	9.37E-03	1.61E-02
**WNT9A**	0.81	9.37E-03	1.61E-02
**PCYOX1L**	0.54	9.38E-03	1.61E-02
**PHF8**	-0.31	9.38E-03	1.61E-02
**RP11-583F2.1**	-2.23	9.38E-03	1.61E-02
**KRTAP17-1**	-6.28	9.39E-03	1.62E-02
**RNU6-930P**	-3.30	9.39E-03	1.62E-02
**SCAF4**	0.41	9.41E-03	1.62E-02
**RPS15AP34**	-3.45	9.42E-03	1.62E-02
**AC013264.2**	-3.79	9.43E-03	1.62E-02
**TMOD2**	-0.39	9.43E-03	1.62E-02
**BCL7A**	0.42	9.43E-03	1.62E-02
**PCDHB3**	-0.85	9.43E-03	1.62E-02
**AKR1C6P**	-3.05	9.44E-03	1.62E-02
**RP11-428P16.3**	-3.20	9.45E-03	1.63E-02
**MIR1284**	-3.57	9.45E-03	1.63E-02
**SNORD113**	-3.45	9.46E-03	1.63E-02
**ZIC2**	1.51	9.48E-03	1.63E-02
**CTD-2066L21.1**	-2.44	9.48E-03	1.63E-02
**P2RY14**	-0.86	9.48E-03	1.63E-02
**RP11-794A8.1**	-3.19	9.48E-03	1.63E-02
**KRT80**	0.56	9.48E-03	1.63E-02
**KRTDAP**	-0.59	9.49E-03	1.63E-02
**XLOC_006299**	-3.50	9.49E-03	1.63E-02
**AQP4-AS1**	-3.72	9.50E-03	1.63E-02
**RP11-599B13.9**	0.82	9.50E-03	1.63E-02
**TDRD12**	-2.41	9.51E-03	1.63E-02
**FDXACB1**	-0.57	9.51E-03	1.63E-02
**RP11-481H12.1**	1.40	9.51E-03	1.63E-02

**RP11-30G8.1**	-3.73	9.52E-03	1.64E-02
**CTC-524C5.2**	-0.89	9.52E-03	1.64E-02
**XPO7**	-0.24	9.53E-03	1.64E-02
**MIR3176**	-3.61	9.54E-03	1.64E-02
**IL12RB2**	0.65	9.54E-03	1.64E-02
**MIR1280**	-3.23	9.54E-03	1.64E-02
**HNRNPH3**	0.47	9.54E-03	1.64E-02
**MIR4319**	-3.57	9.54E-03	1.64E-02
**RP1-102E24.10**	-1.01	9.55E-03	1.64E-02
**NDST1**	0.43	9.55E-03	1.64E-02
**BFSP2**	-2.90	9.56E-03	1.64E-02
**GLIDR**	0.60	9.56E-03	1.64E-02
**MAP1LC3B**	0.46	9.57E-03	1.64E-02
**RP4-655J12.5**	-3.15	9.57E-03	1.64E-02
**XLOC_014355**	-3.12	9.57E-03	1.64E-02
**MIR3926-2**	-3.45	9.57E-03	1.64E-02
**C1QTNF4**	1.27	9.57E-03	1.64E-02
**FANCE**	0.48	9.58E-03	1.64E-02
**SSXP10**	-3.37	9.59E-03	1.65E-02
**ECEL1P3**	-3.28	9.60E-03	1.65E-02
**RNA5SP354**	-3.13	9.61E-03	1.65E-02
**RP11-344N10.4**	-3.08	9.62E-03	1.65E-02
**RP11-110I1.12**	-0.71	9.62E-03	1.65E-02
**FLJ16779**	2.03	9.62E-03	1.65E-02
**MDM1**	-0.46	9.63E-03	1.65E-02
**EIF2S3L**	-2.03	9.63E-03	1.65E-02
**PDYN**	-3.41	9.63E-03	1.65E-02
**UBE2SP2**	-3.62	9.64E-03	1.65E-02
**G8340**	-1.52	9.65E-03	1.65E-02
**RP11-444A22.1**	-3.47	9.65E-03	1.66E-02
**AC118278.1**	-1.33	9.65E-03	1.66E-02
**UNC119B**	0.33	9.66E-03	1.66E-02
**IGHG3**	2.75	9.67E-03	1.66E-02
**ARRDC4**	-0.42	9.67E-03	1.66E-02
**KRT32**	-2.39	9.67E-03	1.66E-02
**RP11-554F20.1**	-1.33	9.68E-03	1.66E-02
**LINC00900**	-0.43	9.68E-03	1.66E-02
**HNRNPKP1**	-3.43	9.68E-03	1.66E-02
**RN7SL615P**	-3.28	9.69E-03	1.66E-02
**FOSB**	1.83	9.69E-03	1.66E-02
**RP11-711K1.7**	-2.16	9.70E-03	1.66E-02
**DPYD-IT1**	-3.13	9.70E-03	1.66E-02
**AP001471.1**	3.31	9.70E-03	1.66E-02
**RP11-399J13.2**	0.85	9.72E-03	1.67E-02
**G4303**	-2.03	9.72E-03	1.67E-02

**RP11-181K3.4**	-1.34	9.73E-03	1.67E-02
**ZSCAN9**	0.43	9.74E-03	1.67E-02
**KLK1**	-0.75	9.75E-03	1.67E-02
**RPL23AP14**	-3.37	9.76E-03	1.67E-02
**AL365502.1**	-3.44	9.76E-03	1.67E-02
**RP11-1105O14.1**	-3.64	9.77E-03	1.67E-02
**10-Sep**	-0.40	9.78E-03	1.68E-02
**CTC-459M5.1**	-2.17	9.79E-03	1.68E-02
**ASB4**	-1.39	9.79E-03	1.68E-02
**FXYD2**	-2.56	9.80E-03	1.68E-02
**SLC13A2**	2.40	9.80E-03	1.68E-02
**TRAP1**	0.31	9.80E-03	1.68E-02
**MSH6**	-0.32	9.81E-03	1.68E-02
**CTD-2006C1.13**	-2.03	9.81E-03	1.68E-02
**IDH1-AS1**	1.65	9.81E-03	1.68E-02
**LINC00327**	-0.73	9.81E-03	1.68E-02
**CTD-2547L16.1**	-1.11	9.81E-03	1.68E-02
**RP11-451H23.1**	-3.12	9.81E-03	1.68E-02
**PROSER3**	0.60	9.82E-03	1.68E-02
**ANO8**	0.55	9.82E-03	1.68E-02
**RNF152P1**	-2.18	9.82E-03	1.68E-02
**RNU6-431P**	-2.93	9.83E-03	1.68E-02
**AC079753.4**	-3.40	9.83E-03	1.68E-02
**COX6B1P5**	-2.29	9.84E-03	1.68E-02
**RP11-565J7.1**	-2.94	9.84E-03	1.68E-02
**PTPN12**	0.36	9.85E-03	1.69E-02
**XRCC5**	-0.36	9.85E-03	1.69E-02
**SEMA3B**	-0.98	9.85E-03	1.69E-02
**AC104699.1**	-3.34	9.86E-03	1.69E-02
**SIT1**	1.40	9.88E-03	1.69E-02
**MAPK15**	-1.26	9.88E-03	1.69E-02
**RP11-740D6.3**	-3.13	9.88E-03	1.69E-02
**HNRNPA1P54**	-3.65	9.88E-03	1.69E-02
**RP11-394I13.3**	-2.53	9.89E-03	1.69E-02
**RP11-220C2.1**	-2.10	9.89E-03	1.69E-02
**MAPK8IP3**	0.62	9.89E-03	1.69E-02
**PLPP4**	1.72	9.89E-03	1.69E-02
**CHCHD2P6**	1.68	9.90E-03	1.69E-02
**RP11-214K3.22**	-3.16	9.90E-03	1.69E-02
**PSMA2P1**	-2.86	9.90E-03	1.69E-02
**TSPEAR-AS2**	-1.73	9.92E-03	1.70E-02
**RP11-177G23.2**	-2.94	9.92E-03	1.70E-02
**RSG1**	0.58	9.92E-03	1.70E-02
**GTF2E2**	0.27	9.93E-03	1.70E-02
**RPL7L1P8**	-3.19	9.94E-03	1.70E-02

**RP1-128O3.6**	-3.61	9.95E-03	1.70E-02
**RP11-800A3.4**	0.85	9.95E-03	1.70E-02
**RP5-1100I6.1**	-3.15	9.96E-03	1.70E-02
**AC016712.1**	-4.79	9.97E-03	1.70E-02
**RGPD2**	-1.63	9.97E-03	1.70E-02
**SARM1**	-0.61	9.97E-03	1.71E-02
**C10orf62**	-3.13	9.98E-03	1.71E-02
**PRDM1**	-0.56	9.99E-03	1.71E-02
**CRYAB**	0.54	9.99E-03	1.71E-02
**MYO1H**	1.25	9.99E-03	1.71E-02
**ZSCAN4**	-1.61	1.00E-02	1.71E-02
**CDC26**	0.42	1.00E-02	1.71E-02
**RP11-43F13.1**	0.51	1.00E-02	1.71E-02
**C1QTNF9B-AS1**	-2.30	1.00E-02	1.71E-02
**RP11-384B12.3**	-3.43	1.00E-02	1.71E-02
**CEACAMP8**	-3.28	1.00E-02	1.71E-02
**CTB-3M24.2**	-2.97	1.00E-02	1.71E-02
**LTV1**	-0.44	1.00E-02	1.71E-02
**RP11-780M14.1**	-3.57	1.00E-02	1.71E-02
**RP11-153M7.3**	2.17	1.00E-02	1.71E-02
**RP11-49K24.3**	-1.52	1.01E-02	1.72E-02
**RP11-154P18.2**	1.79	1.01E-02	1.72E-02
**TRA2A**	0.29	1.01E-02	1.72E-02
**RP13-15M17.1**	-2.97	1.01E-02	1.72E-02
**RP11-420B22.2**	-3.22	1.01E-02	1.72E-02
**CTD-3037G24.4**	-2.29	1.01E-02	1.72E-02
**XLOC_014114**	2.42	1.01E-02	1.72E-02
**RP11-285F7.2**	0.69	1.01E-02	1.72E-02
**UBE2D3P3**	-2.73	1.01E-02	1.73E-02
**RP11-640M9.2**	-0.64	1.01E-02	1.73E-02
**GPC2**	-0.96	1.01E-02	1.73E-02
**PMS2P11**	-3.26	1.01E-02	1.73E-02
**P2RY2**	0.75	1.01E-02	1.73E-02
**OR9I1**	-3.32	1.01E-02	1.73E-02
**RP11-431N15.2**	-3.07	1.02E-02	1.73E-02
**MRI1**	0.66	1.02E-02	1.73E-02
**RN7SL426P**	-2.67	1.02E-02	1.73E-02
**CD79A**	1.90	1.02E-02	1.74E-02
**RP11-299G20.5**	1.23	1.02E-02	1.74E-02
**RP11-11M20.4**	-0.94	1.02E-02	1.74E-02
**MTCYBP18**	-3.12	1.02E-02	1.74E-02
**FNBP1**	-0.22	1.02E-02	1.74E-02
**RPS6P25**	-1.70	1.02E-02	1.74E-02
**AC005307.3**	-2.53	1.02E-02	1.74E-02
**MIEF2**	0.32	1.02E-02	1.75E-02

**COLGALT2**	-1.13	1.02E-02	1.75E-02
**RP11-574K11.24**	0.73	1.02E-02	1.75E-02
**RWDD4P2**	0.54	1.02E-02	1.75E-02
**FAM71C**	-3.14	1.02E-02	1.75E-02
**AURKA**	0.59	1.02E-02	1.75E-02
**AC140061.12**	-3.61	1.03E-02	1.75E-02
**RP11-715J22.2**	-3.35	1.03E-02	1.75E-02
**ANKRD66**	-3.64	1.03E-02	1.75E-02
**EIF4EP3**	-3.29	1.03E-02	1.75E-02
**RP11-157H4.1**	-3.34	1.03E-02	1.75E-02
**RP1-40E16.9**	-3.02	1.03E-02	1.75E-02
**PRR9**	-2.89	1.03E-02	1.75E-02
**bP-21264C1.1**	-4.24	1.03E-02	1.75E-02
**XLOC_011990**	-1.81	1.03E-02	1.75E-02
**GLDCP1**	-3.29	1.03E-02	1.75E-02
**MFSD11**	-0.28	1.03E-02	1.76E-02
**DNAJC18**	0.84	1.03E-02	1.76E-02
**RP11-547C13.1**	-3.43	1.03E-02	1.76E-02
**RP11-201E8.1**	-3.26	1.03E-02	1.76E-02
**PRPF38A**	0.22	1.04E-02	1.76E-02
**DNAJC9-AS1**	-1.53	1.04E-02	1.76E-02
**NRG2**	0.66	1.04E-02	1.77E-02
**PRDX6**	0.41	1.04E-02	1.77E-02
**FAM111A**	-0.46	1.04E-02	1.77E-02
**XLOC_007753**	-3.19	1.04E-02	1.77E-02
**OPCML**	1.61	1.04E-02	1.77E-02
**RP6-159A1.4**	-1.30	1.04E-02	1.77E-02
**ITCH-AS1**	-3.23	1.04E-02	1.77E-02
**TRAIP**	0.46	1.04E-02	1.78E-02
**LINC00392**	-2.36	1.04E-02	1.78E-02
**CTD-2210P15.3**	-3.60	1.05E-02	1.78E-02
**HMGA2**	2.26	1.05E-02	1.78E-02
**GS1-166A23.2**	-2.30	1.05E-02	1.78E-02
**LY86-AS1**	-2.03	1.05E-02	1.78E-02
**RPL35P5**	-1.77	1.05E-02	1.78E-02
**XLOC_013424**	-0.63	1.05E-02	1.79E-02
**LINC01105**	-3.72	1.05E-02	1.79E-02
**AHI1**	-0.49	1.05E-02	1.79E-02
**G33410**	-1.22	1.05E-02	1.79E-02
**C10orf107**	2.22	1.05E-02	1.79E-02
**ADIPOQ**	3.91	1.05E-02	1.79E-02
**RP4-555D20.1**	-3.48	1.05E-02	1.79E-02
**SNORD116-25**	-3.54	1.05E-02	1.79E-02
**GSX2**	-1.26	1.05E-02	1.79E-02
**THEMIS**	-1.21	1.05E-02	1.79E-02

**RNU6-1143P**	-3.33	1.05E-02	1.79E-02
**XLOC_000964**	-3.92	1.05E-02	1.79E-02
**CBWD7**	-1.71	1.05E-02	1.80E-02
**FAM198A**	0.94	1.06E-02	1.80E-02
**CEACAM4**	2.87	1.06E-02	1.80E-02
**CMB9-22P13.2**	-3.60	1.06E-02	1.80E-02
**CCT5**	0.40	1.06E-02	1.80E-02
**RNU6-181P**	-3.35	1.06E-02	1.80E-02
**PRR13P2**	-2.15	1.06E-02	1.80E-02
**ENTHD2**	0.78	1.06E-02	1.80E-02
**DYNLL2**	0.31	1.06E-02	1.80E-02
**FADS1**	-2.28	1.06E-02	1.80E-02
**RP11-485M7.2**	-2.39	1.06E-02	1.80E-02
**ZNF648**	-2.61	1.06E-02	1.80E-02
**RNU6-1045P**	-3.45	1.06E-02	1.80E-02
**CYP2R1**	-0.37	1.06E-02	1.80E-02
**CTD-2530H12.8**	2.17	1.06E-02	1.81E-02
**RBBP8**	-0.43	1.06E-02	1.81E-02
**RN7SKP273**	-3.26	1.06E-02	1.81E-02
**RP1-137D17.2**	1.66	1.06E-02	1.81E-02
**IFI44**	1.31	1.06E-02	1.81E-02
**G25971**	-2.52	1.06E-02	1.81E-02
**RGS11**	1.38	1.06E-02	1.81E-02
**RNU6-79P**	-3.32	1.07E-02	1.81E-02
**YBX1P7**	-3.33	1.07E-02	1.81E-02
**PPP4R4**	-1.06	1.07E-02	1.81E-02
**G16011**	-1.26	1.07E-02	1.81E-02
**AC002044.4**	3.00	1.07E-02	1.81E-02
**TOX4**	0.26	1.07E-02	1.82E-02
**TBCE**	-0.35	1.07E-02	1.82E-02
**HOXB-AS3**	-2.62	1.07E-02	1.82E-02
**XLOC_014038**	-1.45	1.07E-02	1.82E-02
**CD300LB**	2.25	1.07E-02	1.82E-02
**RP11-6J24.3**	-2.20	1.07E-02	1.82E-02
**CXorf38**	-0.37	1.07E-02	1.82E-02
**RPS4XP3**	-3.65	1.07E-02	1.82E-02
**RP11-266J6.2**	-3.65	1.07E-02	1.82E-02
**LPIN2**	0.58	1.08E-02	1.83E-02
**RP11-467L13.7**	0.62	1.08E-02	1.83E-02
**RP11-83N9.5**	0.83	1.08E-02	1.83E-02
**RP11-392A14.9**	-3.00	1.08E-02	1.83E-02
**GJD4**	2.29	1.08E-02	1.83E-02
**FRMD7**	-1.82	1.08E-02	1.83E-02
**CTB-1144G6.5**	-3.58	1.08E-02	1.84E-02
**TSPAN31**	0.35	1.08E-02	1.84E-02

**RP4-736H5.3**	-3.21	1.08E-02	1.84E-02
**MMP16**	-1.13	1.08E-02	1.84E-02
**ZSCAN23**	-1.40	1.08E-02	1.84E-02
**ABHD11-AS1**	1.03	1.08E-02	1.84E-02
**RP11-345N11.1**	-3.30	1.08E-02	1.84E-02
**AC097455.1**	-3.39	1.09E-02	1.84E-02
**PLEC**	-0.49	1.09E-02	1.84E-02
**XLOC_004671**	-3.22	1.09E-02	1.84E-02
**RP11-135A1.2**	-3.07	1.09E-02	1.84E-02
**RP11-314C9.1**	-3.01	1.09E-02	1.84E-02
**SNAP25**	1.85	1.09E-02	1.85E-02
**RP11-849F2.7**	-2.31	1.09E-02	1.85E-02
**PTPRR**	1.73	1.09E-02	1.85E-02
**CYP2C9**	-2.36	1.09E-02	1.85E-02
**RN7SL684P**	-2.58	1.09E-02	1.85E-02
**DDX21**	-0.46	1.09E-02	1.86E-02
**NARSP2**	-3.29	1.09E-02	1.86E-02
**AC159540.14**	-3.80	1.09E-02	1.86E-02
**PHBP21**	-3.02	1.10E-02	1.86E-02
**RPL7AP60**	-1.71	1.10E-02	1.86E-02
**XLOC_005549**	-3.26	1.10E-02	1.86E-02
**NYNRIN**	-0.48	1.10E-02	1.86E-02
**RP11-391M7.3**	-3.27	1.10E-02	1.86E-02
**IGDCC4**	1.05	1.10E-02	1.86E-02
**G36295**	-1.95	1.10E-02	1.86E-02
**GPR33**	-3.32	1.10E-02	1.86E-02
**WBP1L**	0.35	1.10E-02	1.86E-02
**RNFT1**	-0.43	1.10E-02	1.86E-02
**RP11-317G6.1**	-3.20	1.10E-02	1.86E-02
**CTD-2653D5.1**	-2.30	1.10E-02	1.86E-02
**RP3-453C12.15**	-2.99	1.10E-02	1.86E-02
**ZNF71**	0.33	1.10E-02	1.86E-02
**SERINC1**	-0.40	1.10E-02	1.86E-02
**RP11-390K5.6**	-1.20	1.10E-02	1.86E-02
**TRIM2**	-0.57	1.10E-02	1.87E-02
**COX10-AS1**	-0.45	1.10E-02	1.87E-02
**RP11-394J1.2**	-3.45	1.10E-02	1.87E-02
**ATG12P2**	-3.02	1.10E-02	1.87E-02
**G34171**	-1.36	1.10E-02	1.87E-02
**TNNI3**	-1.10	1.10E-02	1.87E-02
**GLTP**	0.44	1.10E-02	1.87E-02
**HLA-DRB1**	1.17	1.10E-02	1.87E-02
**CELSR3**	0.68	1.10E-02	1.87E-02
**GINS4**	0.66	1.11E-02	1.88E-02
**1-Dec**	2.26	1.11E-02	1.88E-02

**HELQ**	-0.28	1.11E-02	1.88E-02
**SPIDR**	0.27	1.11E-02	1.88E-02
**PSG8**	-3.60	1.11E-02	1.88E-02
**ITGA6**	0.42	1.11E-02	1.88E-02
**CECR7**	-0.62	1.11E-02	1.88E-02
**CYCSP29**	-3.63	1.11E-02	1.88E-02
**DOCK3**	-0.59	1.11E-02	1.88E-02
**ZNF667**	-0.85	1.11E-02	1.88E-02
**RP11-470P21.2**	-2.70	1.11E-02	1.88E-02
**CXCL10**	2.43	1.11E-02	1.88E-02
**RP11-421F16.3**	-0.42	1.11E-02	1.88E-02
**AC008993.2**	-3.12	1.11E-02	1.88E-02
**AP000356.1**	2.03	1.11E-02	1.88E-02
**FBXO48**	-0.41	1.11E-02	1.88E-02
**XLOC_008957**	2.51	1.11E-02	1.88E-02
**LRRC23**	0.58	1.11E-02	1.89E-02
**RPL36A**	-0.90	1.11E-02	1.89E-02
**MRPL13**	0.46	1.12E-02	1.89E-02
**ADGRB3**	-1.02	1.12E-02	1.89E-02
**GATAD2A**	0.29	1.12E-02	1.89E-02
**RNU6-1306P**	-3.55	1.12E-02	1.89E-02
**ROPN1L**	2.12	1.12E-02	1.89E-02
**SAMD12-AS1**	-3.10	1.12E-02	1.89E-02
**RP11-656D10.3**	1.27	1.12E-02	1.89E-02
**TMEM178A**	1.31	1.12E-02	1.89E-02
**TMEM253**	-1.51	1.12E-02	1.90E-02
**RRM1**	-0.41	1.12E-02	1.90E-02
**PSMC3IP**	0.52	1.12E-02	1.90E-02
**TMX3**	-0.39	1.12E-02	1.90E-02
**AP001619.3**	-2.24	1.12E-02	1.90E-02
**RP5-967N21.11**	-1.47	1.12E-02	1.90E-02
**AC006946.17**	-1.51	1.12E-02	1.90E-02
**CPOX**	0.30	1.12E-02	1.90E-02
**CEP170**	-0.37	1.12E-02	1.90E-02
**XLOC_014400**	-3.38	1.12E-02	1.90E-02
**G39784**	-1.88	1.13E-02	1.90E-02
**WDR64**	-3.25	1.13E-02	1.91E-02
**RN7SL663P**	-3.06	1.13E-02	1.91E-02
**GLYCTK**	0.76	1.13E-02	1.91E-02
**BMS1P15**	2.44	1.13E-02	1.91E-02
**RP1-251I12.1**	-3.58	1.13E-02	1.91E-02
**ARHGAP21**	-0.38	1.13E-02	1.91E-02
**RP11-134G8.5**	0.88	1.13E-02	1.91E-02
**RP5-823G15.5**	-2.48	1.13E-02	1.91E-02
**SEPP1**	0.56	1.13E-02	1.91E-02

**HTR1B**	1.85	1.13E-02	1.91E-02
**SRRD**	-0.32	1.13E-02	1.91E-02
**RN7SL735P**	-3.46	1.13E-02	1.91E-02
**C15orf40**	-0.26	1.13E-02	1.92E-02
**ANKRD62P1-PARP4P3**	-1.77	1.13E-02	1.92E-02
**RP11-400K9.4**	0.72	1.14E-02	1.92E-02
**AC007318.5**	0.60	1.14E-02	1.92E-02
**RP11-244O19.1**	0.64	1.14E-02	1.92E-02
**FAM132A**	-1.05	1.14E-02	1.92E-02
**AMMECR1L**	0.21	1.14E-02	1.92E-02
**RP3-522P13.3**	-3.40	1.14E-02	1.92E-02
**EEF1B2P8**	-3.09	1.14E-02	1.92E-02
**RPL15P2**	-2.90	1.14E-02	1.92E-02
**RNU6-883P**	-2.95	1.14E-02	1.92E-02
**RP11-36C20.1**	-1.31	1.14E-02	1.92E-02
**BIK**	0.77	1.14E-02	1.92E-02
**RP11-430B1.1**	-1.62	1.14E-02	1.92E-02
**AMPD1**	-3.43	1.14E-02	1.92E-02
**OXT**	2.32	1.14E-02	1.93E-02
**RP4-651E10.4**	-2.02	1.14E-02	1.93E-02
**SLC7A10**	2.87	1.15E-02	1.93E-02
**HSD17B11**	0.62	1.15E-02	1.93E-02
**ST3GAL5-AS1**	-1.42	1.15E-02	1.93E-02
**RP3-406P24.4**	-2.97	1.15E-02	1.94E-02
**CTD-2349P21.12**	-3.18	1.15E-02	1.94E-02
**LLNLR-304A6.1**	-2.93	1.15E-02	1.94E-02
**RPS18P12**	-2.07	1.15E-02	1.94E-02
**ATG12**	-0.29	1.15E-02	1.94E-02
**RP11-889L3.1**	-1.48	1.15E-02	1.94E-02
**SLC35E1P1**	-1.68	1.15E-02	1.94E-02
**CTC-260E6.6**	-1.31	1.15E-02	1.94E-02
**RN7SL738P**	-2.62	1.15E-02	1.94E-02
**RP11-411B10.2**	-3.08	1.15E-02	1.94E-02
**BCO1**	-1.06	1.15E-02	1.94E-02
**G34275**	-1.51	1.15E-02	1.95E-02
**RNU6-876P**	-2.81	1.15E-02	1.95E-02
**AE000661.37**	2.47	1.15E-02	1.95E-02
**G8412**	-0.76	1.16E-02	1.95E-02
**ZNF488**	-0.70	1.16E-02	1.95E-02
**ADAMTS17**	-0.52	1.16E-02	1.95E-02
**RP11-327F22.1**	-3.33	1.16E-02	1.95E-02
**RNU7-108P**	-3.53	1.16E-02	1.95E-02
**RNU1-51P**	-3.76	1.16E-02	1.95E-02
**CCDC188**	1.28	1.16E-02	1.96E-02
**TSPAN3**	0.40	1.16E-02	1.96E-02

**AC005477.1**	-3.20	1.16E-02	1.96E-02
**ATP11C**	-0.39	1.16E-02	1.96E-02
**RP11-361F15.5**	-3.87	1.16E-02	1.96E-02
**MSMO1**	-1.10	1.16E-02	1.96E-02
**CTD-2158P22.1**	-3.28	1.16E-02	1.96E-02
**RP5-1028L10.2**	-3.04	1.16E-02	1.96E-02
**ALG1L11P**	-3.06	1.16E-02	1.96E-02
**RP11-730K11.1**	-3.22	1.16E-02	1.96E-02
**KLKB1**	1.87	1.16E-02	1.96E-02
**PIGH**	-0.28	1.17E-02	1.96E-02
**MBD4**	-0.25	1.17E-02	1.96E-02
**RSPO2**	4.43	1.17E-02	1.96E-02
**RP11-379F4.1**	-2.71	1.17E-02	1.96E-02
**AL360176.1**	-3.14	1.17E-02	1.97E-02
**RP11-127B20.3**	-1.28	1.17E-02	1.97E-02
**RP11-1319K7.1**	-1.05	1.17E-02	1.97E-02
**SNRPGP6**	-3.78	1.17E-02	1.97E-02
**CTD-2526A2.2**	-1.67	1.17E-02	1.97E-02
**NKTR**	-0.66	1.17E-02	1.97E-02
**ZNF859P**	-3.64	1.17E-02	1.97E-02
**AC105381.1**	-3.06	1.17E-02	1.97E-02
**FLNB**	0.29	1.17E-02	1.97E-02
**RNU6-1010P**	2.42	1.17E-02	1.97E-02
**CTD-2240J17.2**	-3.55	1.17E-02	1.97E-02
**KCNK7**	-0.64	1.17E-02	1.97E-02
**SENP3-EIF4A1**	-2.24	1.17E-02	1.97E-02
**POLR2C**	0.29	1.17E-02	1.97E-02
**RNU6-987P**	-3.43	1.17E-02	1.97E-02
**G31097**	0.99	1.17E-02	1.97E-02
**CTC-260E6.3**	-3.12	1.17E-02	1.97E-02
**SLC6A13**	-3.07	1.17E-02	1.97E-02
**RP11-730A19.5**	-1.43	1.17E-02	1.98E-02
**RP11-115C21.2**	0.54	1.17E-02	1.98E-02
**RP11-446F17.3**	-3.19	1.18E-02	1.98E-02
**RP11-383G6.3**	-1.62	1.18E-02	1.98E-02
**MIR645**	-2.23	1.18E-02	1.98E-02
**RP11-769N19.2**	-3.27	1.18E-02	1.98E-02
**RP11-446E9.1**	-1.67	1.18E-02	1.98E-02
**AADAT**	0.91	1.18E-02	1.98E-02
**RNA5SP168**	-3.28	1.18E-02	1.98E-02
**ZNF286A**	-0.39	1.18E-02	1.98E-02
**NBEAL2**	0.61	1.18E-02	1.98E-02
**GNAT1**	-3.19	1.18E-02	1.98E-02
**GSTT2**	2.18	1.18E-02	1.98E-02
**G33852**	-0.80	1.18E-02	1.98E-02

**RNU6-343P**	-3.21	1.18E-02	1.98E-02
**LRRC42**	0.27	1.18E-02	1.99E-02
**RP11-83M16.4**	-3.20	1.18E-02	1.99E-02
**ZNF416**	-0.34	1.18E-02	1.99E-02
**C9orf170**	1.49	1.18E-02	1.99E-02
**AC090696.2**	-3.48	1.18E-02	1.99E-02
**C4orf33**	-0.36	1.18E-02	1.99E-02
**EIF2AK4**	0.21	1.18E-02	1.99E-02
**RP11-744O11.2**	-2.81	1.18E-02	1.99E-02
**PODN**	1.05	1.18E-02	1.99E-02
**TM9SF2**	-0.37	1.18E-02	1.99E-02
**C8orf44-SGK3**	-3.22	1.19E-02	1.99E-02
**MPPED1**	-1.50	1.19E-02	1.99E-02
**IFT88**	-0.30	1.19E-02	2.00E-02
**MKRN3**	-2.60	1.19E-02	2.00E-02
**CBLC**	0.41	1.19E-02	2.00E-02
**DPRXP5**	-3.28	1.19E-02	2.00E-02
**RPN2**	0.32	1.19E-02	2.00E-02
**RP11-395N3.1**	-3.29	1.19E-02	2.00E-02
**RNA5SP364**	-3.83	1.19E-02	2.00E-02
**TRAJ30**	-3.35	1.19E-02	2.00E-02
**RP11-217B7.3**	-1.65	1.19E-02	2.00E-02
**ZNF329**	-0.32	1.19E-02	2.01E-02
**SYNE1-AS1**	-2.83	1.19E-02	2.01E-02
**ARHGEF5**	-0.51	1.19E-02	2.01E-02
**RP3-395M20.2**	-1.86	1.19E-02	2.01E-02
**ZSCAN20**	-0.42	1.19E-02	2.01E-02
**RPL4P6**	-1.16	1.20E-02	2.01E-02
**RP11-14N7.2**	2.77	1.20E-02	2.01E-02
**RP11-219A15.1**	-2.75	1.20E-02	2.01E-02
**KCNAB2**	0.58	1.20E-02	2.01E-02
**RP11-383C6.2**	-3.85	1.20E-02	2.01E-02
**HLA-DQA2**	1.06	1.20E-02	2.02E-02
**RP11-473C19.1**	-3.42	1.20E-02	2.02E-02
**SSSCA1-AS1**	0.76	1.20E-02	2.02E-02
**RPL7P10**	-3.41	1.20E-02	2.02E-02
**PCSK5**	0.89	1.20E-02	2.02E-02
**BEAN1-AS1**	-3.33	1.20E-02	2.02E-02
**RP11-53B2.6**	-2.24	1.21E-02	2.02E-02
**RBM17P4**	-1.64	1.21E-02	2.03E-02
**ZNF680P1**	-3.17	1.21E-02	2.03E-02
**PNPT1**	-0.31	1.21E-02	2.03E-02
**GKN2**	-3.62	1.21E-02	2.03E-02
**MIR151B**	-3.33	1.21E-02	2.03E-02
**CTD-2267D19.1**	-1.38	1.21E-02	2.03E-02

**RP11-809O17.1**	1.39	1.21E-02	2.04E-02
**RP11-386M24.9**	-3.63	1.21E-02	2.04E-02
**AC011243.1**	-3.30	1.21E-02	2.04E-02
**HK1**	0.34	1.22E-02	2.04E-02
**RNA5SP491**	-3.50	1.22E-02	2.04E-02
**ZNF423**	0.96	1.22E-02	2.04E-02
**DDX46**	0.31	1.22E-02	2.04E-02
**RP11-427P5.3**	-2.29	1.22E-02	2.04E-02
**RP11-146F11.1**	-0.75	1.22E-02	2.04E-02
**FASTKD1**	-0.42	1.22E-02	2.04E-02
**SIGLEC8**	-2.09	1.22E-02	2.05E-02
**RSPRY1**	-0.20	1.22E-02	2.05E-02
**CSNK1G2P1**	-1.82	1.22E-02	2.05E-02
**RP11-596C23.2**	-1.68	1.22E-02	2.05E-02
**RNU5E-10P**	-3.03	1.23E-02	2.05E-02
**ACOT12**	-3.18	1.23E-02	2.06E-02
**KRT38**	-2.39	1.23E-02	2.06E-02
**XLOC_002840**	-1.03	1.23E-02	2.06E-02
**PPT2**	0.38	1.23E-02	2.06E-02
**RP11-538P18.1**	-3.29	1.23E-02	2.06E-02
**XLOC_004672**	1.37	1.23E-02	2.06E-02
**LINC00639**	1.08	1.23E-02	2.06E-02
**NPIPB7**	-1.96	1.23E-02	2.07E-02
**ZNF274**	-0.30	1.23E-02	2.07E-02
**RP11-439A17.10**	-1.32	1.23E-02	2.07E-02
**G909**	2.39	1.23E-02	2.07E-02
**MRPL57P8**	-3.08	1.23E-02	2.07E-02
**AIM1**	-0.64	1.24E-02	2.07E-02
**RP11-326N17.1**	-3.58	1.24E-02	2.07E-02
**LIMD1-AS1**	-1.44	1.24E-02	2.07E-02
**CTD-2206G10.2**	-3.45	1.24E-02	2.07E-02
**G25497**	-1.45	1.24E-02	2.07E-02
**CAMK2D**	-0.33	1.24E-02	2.07E-02
**RP1-111B22.3**	-2.13	1.24E-02	2.08E-02
**GS1-124K5.11**	-0.81	1.24E-02	2.08E-02
**RNA5SP304**	-3.64	1.24E-02	2.08E-02
**MTRNR2L4**	-2.97	1.24E-02	2.08E-02
**RP3-431P23.2**	-3.32	1.24E-02	2.08E-02
**ASF1B**	0.55	1.24E-02	2.08E-02
**PRDX3P4**	-2.31	1.24E-02	2.08E-02
**RN7SL174P**	-3.16	1.24E-02	2.08E-02
**FITM2**	-0.55	1.24E-02	2.08E-02
**OPN3**	0.41	1.25E-02	2.08E-02
**RPL36AP29**	-3.22	1.25E-02	2.08E-02
**RYR3**	-0.89	1.25E-02	2.08E-02

**PLIN1**	2.74	1.25E-02	2.08E-02
**RN7SL610P**	-3.09	1.25E-02	2.09E-02
**SUPV3L1**	-0.21	1.25E-02	2.09E-02
**KCTD16**	-1.43	1.25E-02	2.09E-02
**AC019201.1**	-3.57	1.25E-02	2.09E-02
**AC116614.1**	2.82	1.25E-02	2.09E-02
**RP11-796A5.3**	-1.80	1.25E-02	2.09E-02
**THOC7**	0.40	1.25E-02	2.09E-02
**SERHL**	0.96	1.25E-02	2.09E-02
**YWHAZP2**	-1.69	1.25E-02	2.09E-02
**CTD-2026K11.2**	-2.27	1.25E-02	2.09E-02
**SERPINB2**	-0.70	1.25E-02	2.09E-02
**DRAXIN**	1.39	1.25E-02	2.09E-02
**RP11-67L3.4**	-1.23	1.25E-02	2.10E-02
**DSCC1**	-0.60	1.25E-02	2.10E-02
**ATRX**	-0.32	1.25E-02	2.10E-02
**RP11-661G16.1**	-3.50	1.26E-02	2.10E-02
**TMEM35**	1.13	1.26E-02	2.10E-02
**RP11-182N22.8**	-3.31	1.26E-02	2.10E-02
**RP11-245D16.4**	0.89	1.26E-02	2.10E-02
**RNU2-63P**	-3.28	1.26E-02	2.10E-02
**DYRK2**	-0.28	1.26E-02	2.11E-02
**RP5-967N21.7**	-3.38	1.26E-02	2.11E-02
**ZNF395**	0.37	1.26E-02	2.11E-02
**SNORD116-26**	-3.48	1.26E-02	2.11E-02
**MESTIT1**	-2.58	1.26E-02	2.11E-02
**NR2F2-AS1**	1.33	1.27E-02	2.11E-02
**NMRK1**	0.33	1.27E-02	2.12E-02
**CHRM1**	-1.07	1.27E-02	2.12E-02
**CBFA2T2**	-0.35	1.27E-02	2.12E-02
**UGT8**	1.02	1.27E-02	2.12E-02
**LINC00908**	-1.06	1.27E-02	2.12E-02
**DMBT1**	-1.94	1.27E-02	2.12E-02
**PTP4A2**	0.35	1.27E-02	2.12E-02
**RP11-876N24.2**	-3.14	1.27E-02	2.12E-02
**VN1R21P**	-1.64	1.27E-02	2.13E-02
**MIR454**	-3.34	1.27E-02	2.13E-02
**AP000892.4**	-3.17	1.27E-02	2.13E-02
**G10252**	1.20	1.27E-02	2.13E-02
**RNF103**	-0.28	1.27E-02	2.13E-02
**CFD**	-0.98	1.27E-02	2.13E-02
**HSBP1**	0.35	1.28E-02	2.13E-02
**RPS3AP29**	-2.20	1.28E-02	2.13E-02
**OSBP2**	0.48	1.28E-02	2.13E-02
**RP11-405M12.3**	1.84	1.28E-02	2.13E-02

**G26935**	-3.13	1.28E-02	2.13E-02
**FAM120B**	-0.28	1.28E-02	2.13E-02
**SLC5A4**	2.20	1.28E-02	2.14E-02
**RP6-42F4.1**	-0.46	1.28E-02	2.14E-02
**TMEM59L**	2.22	1.28E-02	2.14E-02
**SEC31A**	0.22	1.28E-02	2.14E-02
**PRICKLE2-AS1**	-1.96	1.28E-02	2.14E-02
**FAM114A1**	0.37	1.28E-02	2.14E-02
**CCNO**	0.71	1.28E-02	2.14E-02
**SPATA22**	-3.89	1.28E-02	2.14E-02
**CYP1B1-AS1**	-1.03	1.28E-02	2.14E-02
**RP1-92C4.1**	-3.56	1.28E-02	2.14E-02
**TMSB15B**	0.70	1.28E-02	2.14E-02
**EBP**	0.63	1.28E-02	2.14E-02
**APOBEC3D**	1.06	1.29E-02	2.14E-02
**AC139887.4**	-1.97	1.29E-02	2.14E-02
**NKAPL**	0.67	1.29E-02	2.14E-02
**RP11-504I13.3**	-1.69	1.29E-02	2.14E-02
**IGKV1-22**	-3.40	1.29E-02	2.14E-02
**RPL23AP50**	-2.92	1.29E-02	2.15E-02
**C1orf116**	-0.47	1.29E-02	2.15E-02
**RNU6-628P**	-3.36	1.29E-02	2.15E-02
**HYI-AS1**	1.49	1.29E-02	2.15E-02
**ZBTB40**	-0.31	1.29E-02	2.15E-02
**RP11-13K12.1**	1.73	1.29E-02	2.15E-02
**CGNL1**	-0.72	1.29E-02	2.15E-02
**KHNYN**	-0.21	1.29E-02	2.15E-02
**RP11-1105G2.3**	-1.25	1.29E-02	2.15E-02
**AC007879.1**	-3.22	1.29E-02	2.15E-02
**RPS10P18**	-3.30	1.29E-02	2.15E-02
**PCDH11Y**	-2.13	1.29E-02	2.15E-02
**GALR3**	2.87	1.29E-02	2.16E-02
**INSRR**	3.30	1.29E-02	2.16E-02
**NFKBIZ**	-0.51	1.30E-02	2.16E-02
**RP11-798G7.7**	-1.70	1.30E-02	2.16E-02
**LINC01128**	0.34	1.30E-02	2.16E-02
**RP11-390G14.1**	-2.24	1.30E-02	2.16E-02
**WI2-85898F10.2**	-2.03	1.30E-02	2.16E-02
**COL4A3BP**	-0.31	1.30E-02	2.16E-02
**RP11-554A11.8**	-3.26	1.30E-02	2.17E-02
**ARL13B**	-0.36	1.30E-02	2.17E-02
**RNU5A-8P**	-3.70	1.30E-02	2.17E-02
**AGAP10P**	1.48	1.31E-02	2.17E-02
**KRT8P33**	-0.99	1.31E-02	2.17E-02
**ST8SIA6-AS1**	1.61	1.31E-02	2.17E-02

**RN7SL838P**	-3.19	1.31E-02	2.17E-02
**SCGB1D5P**	-3.69	1.31E-02	2.18E-02
**PHKB**	-0.21	1.31E-02	2.18E-02
**RP11-252C24.2**	-3.54	1.31E-02	2.18E-02
**G43233**	-1.25	1.31E-02	2.18E-02
**RP11-346C20.4**	3.13	1.31E-02	2.18E-02
**ST3GAL5**	-0.58	1.31E-02	2.18E-02
**RAB28**	-0.31	1.31E-02	2.18E-02
**RP11-47J17.2**	-2.01	1.31E-02	2.18E-02
**RNU6-432P**	-3.61	1.31E-02	2.18E-02
**RP11-311P8.2**	-1.65	1.31E-02	2.18E-02
**AC004453.8**	-0.97	1.31E-02	2.18E-02
**CCNA2**	-0.52	1.31E-02	2.18E-02
**SNORA51**	-1.40	1.31E-02	2.18E-02
**LRRC18**	-1.49	1.31E-02	2.18E-02
**CGREF1**	0.78	1.31E-02	2.18E-02
**ANKRD26P4**	-2.27	1.32E-02	2.19E-02
**RP11-357P18.2**	-3.21	1.32E-02	2.19E-02
**RN7SKP269**	-2.07	1.32E-02	2.19E-02
**CTD-2373J6.1**	-1.57	1.32E-02	2.19E-02
**KLRK1**	-1.56	1.32E-02	2.19E-02
**ZC2HC1B**	-3.03	1.32E-02	2.19E-02
**LINC01344**	-2.15	1.32E-02	2.19E-02
**RP11-428G5.7**	-3.27	1.32E-02	2.19E-02
**AC114969.1**	-3.51	1.32E-02	2.20E-02
**CTD-2162K18.4**	-3.04	1.32E-02	2.20E-02
**RP11-541M12.6**	-1.73	1.32E-02	2.20E-02
**RP11-324O2.3**	-1.42	1.32E-02	2.20E-02
**RP11-50D16.4**	-2.14	1.32E-02	2.20E-02
**G32279**	2.70	1.32E-02	2.20E-02
**G24237**	-0.84	1.32E-02	2.20E-02
**KIFC1**	0.51	1.32E-02	2.20E-02
**AP2B1**	0.29	1.32E-02	2.20E-02
**FAAH**	0.53	1.32E-02	2.20E-02
**MIR3135B**	-3.15	1.33E-02	2.20E-02
**RP11-156L14.1**	-2.98	1.33E-02	2.20E-02
**RP11-650K20.2**	-3.14	1.33E-02	2.20E-02
**GYPA**	-3.11	1.33E-02	2.20E-02
**AC000124.1**	-3.14	1.33E-02	2.21E-02
**CASD1**	-0.38	1.33E-02	2.21E-02
**LARS**	-0.32	1.33E-02	2.21E-02
**GOLGA2P7**	0.73	1.33E-02	2.21E-02
**DMXL2**	-0.61	1.33E-02	2.21E-02
**SDR16C6P**	-3.25	1.33E-02	2.21E-02
**RNU6-5P**	-2.93	1.33E-02	2.21E-02

**RNU4-30P**	-3.27	1.33E-02	2.22E-02
**RP11-138I17.1**	-2.93	1.34E-02	2.22E-02
**RP11-483P21.6**	-2.36	1.34E-02	2.22E-02
**RP11-539E19.2**	-3.38	1.34E-02	2.22E-02
**SLN**	1.56	1.34E-02	2.22E-02
**RP5-837M10.4**	-2.17	1.34E-02	2.22E-02
**LYPD2**	-0.89	1.34E-02	2.22E-02
**AC010884.1**	-2.81	1.34E-02	2.23E-02
**UBAP2**	-0.24	1.34E-02	2.23E-02
**RP11-488L18.3**	-1.36	1.34E-02	2.23E-02
**LINC01100**	-4.01	1.34E-02	2.23E-02
**TAF4**	0.31	1.34E-02	2.23E-02
**NEK10**	-1.28	1.34E-02	2.23E-02
**CTD-2197I11.1**	-2.23	1.34E-02	2.23E-02
**IMPDH1P11**	-3.33	1.34E-02	2.23E-02
**RP11-420L9.2**	1.67	1.34E-02	2.23E-02
**RP11-516C1.1**	-3.06	1.34E-02	2.23E-02
**MBIP**	-0.33	1.34E-02	2.23E-02
**SULT4A1**	2.65	1.34E-02	2.23E-02
**GDAP1**	0.45	1.35E-02	2.23E-02
**RP11-1105G2.4**	-2.89	1.35E-02	2.23E-02
**RP11-618L22.1**	-2.28	1.35E-02	2.24E-02
**MCM3**	0.33	1.35E-02	2.24E-02
**PTTG3P**	-3.26	1.35E-02	2.24E-02
**CTB-43E15.1**	-1.53	1.35E-02	2.24E-02
**ENO3**	0.80	1.35E-02	2.24E-02
**HCG25**	-1.12	1.35E-02	2.24E-02
**ERVMER61-1**	-2.51	1.35E-02	2.25E-02
**ZNF503-AS1**	1.18	1.35E-02	2.25E-02
**RPS20P10**	-3.29	1.35E-02	2.25E-02
**DISC1**	0.66	1.35E-02	2.25E-02
**DLG5**	-0.31	1.35E-02	2.25E-02
**XLOC_002770**	-3.29	1.36E-02	2.25E-02
**GAD1**	-2.27	1.36E-02	2.25E-02
**CDC20P1**	0.81	1.36E-02	2.25E-02
**CTAGE3P**	-3.19	1.36E-02	2.25E-02
**LINC01127**	0.93	1.36E-02	2.25E-02
**RPS18**	0.42	1.36E-02	2.25E-02
**RP11-115N12.1**	-2.46	1.36E-02	2.25E-02
**AZGP1**	0.70	1.36E-02	2.25E-02
**CTB-55O6.8**	0.74	1.36E-02	2.25E-02
**SRSF3**	0.29	1.36E-02	2.26E-02
**RN7SL213P**	-3.02	1.36E-02	2.26E-02
**GRSF1**	-0.24	1.36E-02	2.26E-02
**CYP51A1**	-0.54	1.36E-02	2.26E-02

**HHIP-AS1**	-1.92	1.36E-02	2.26E-02
**XLOC_010945**	1.59	1.37E-02	2.26E-02
**RP11-181K12.2**	-2.77	1.37E-02	2.26E-02
**ATP5G1**	0.46	1.37E-02	2.26E-02
**RP13-497K6.1**	-3.40	1.37E-02	2.26E-02
**ULBP3**	1.30	1.37E-02	2.27E-02
**IL33**	0.98	1.37E-02	2.27E-02
**HLA-H**	1.34	1.37E-02	2.27E-02
**MIR4677**	-3.23	1.37E-02	2.27E-02
**XLOC_000648**	1.59	1.37E-02	2.27E-02
**RP11-83B20.4**	-3.46	1.37E-02	2.27E-02
**KB-1907C4.2**	-3.52	1.37E-02	2.27E-02
**AC023481.1**	-3.15	1.37E-02	2.27E-02
**POM121L6P**	-2.41	1.38E-02	2.28E-02
**OR52B6**	-3.10	1.38E-02	2.28E-02
**INSIG1**	-1.49	1.38E-02	2.28E-02
**INTS3**	0.37	1.38E-02	2.28E-02
**LINC01142**	3.23	1.38E-02	2.29E-02
**RP1-251M9.2**	-2.96	1.38E-02	2.29E-02
**C16orf71**	-1.00	1.38E-02	2.29E-02
**LINC01068**	-2.54	1.38E-02	2.29E-02
**MIR302C**	-3.19	1.38E-02	2.29E-02
**PLPPR4**	0.77	1.38E-02	2.29E-02
**ZNF90P1**	-1.31	1.39E-02	2.29E-02
**MIR141**	-3.14	1.39E-02	2.30E-02
**SMN1**	0.41	1.39E-02	2.30E-02
**IBA57**	-0.34	1.39E-02	2.30E-02
**AC073551.1**	-3.37	1.39E-02	2.30E-02
**HMGN3-AS1**	-0.54	1.39E-02	2.30E-02
**FAM215B**	2.24	1.39E-02	2.30E-02
**AL391384.1**	-3.26	1.39E-02	2.30E-02
**FAM155A**	1.94	1.39E-02	2.30E-02
**LINC00343**	-3.64	1.39E-02	2.31E-02
**RP11-61F12.1**	-2.95	1.39E-02	2.31E-02
**RP11-391L3.5**	-1.52	1.39E-02	2.31E-02
**SLCO4A1-AS1**	-0.83	1.39E-02	2.31E-02
**BRI3P1**	-2.23	1.40E-02	2.31E-02
**AC003973.3**	-1.42	1.40E-02	2.31E-02
**MYBPHL**	-3.09	1.40E-02	2.31E-02
**RP11-327E2.5**	1.14	1.40E-02	2.31E-02
**SMYD3-IT1**	-2.06	1.40E-02	2.31E-02
**CDK1**	-0.57	1.40E-02	2.31E-02
**RP11-428P16.2**	-1.38	1.40E-02	2.32E-02
**RN7SKP171**	-3.52	1.40E-02	2.32E-02
**RP11-84C10.4**	-1.66	1.40E-02	2.32E-02

**G34929**	1.58	1.40E-02	2.32E-02
**RABGAP1L-IT1**	-1.56	1.40E-02	2.32E-02
**FAM92A1**	-0.46	1.40E-02	2.32E-02
**MIR4524B**	-3.02	1.40E-02	2.32E-02
**MX1**	1.30	1.40E-02	2.32E-02
**RN7SL600P**	1.24	1.41E-02	2.33E-02
**NOTCH1**	0.43	1.41E-02	2.33E-02
**STT3B**	-0.30	1.41E-02	2.33E-02
**SMG8**	-0.37	1.41E-02	2.33E-02
**RP11-1029J19.4**	1.54	1.41E-02	2.33E-02
**RPL32P1**	-1.94	1.41E-02	2.33E-02
**RP11-374A4.1**	-3.47	1.41E-02	2.33E-02
**RNU6-42P**	-3.35	1.41E-02	2.33E-02
**CD6**	0.85	1.41E-02	2.33E-02
**ALDH1A2**	-0.70	1.41E-02	2.33E-02
**AC069257.6**	-2.92	1.41E-02	2.34E-02
**RP11-288I21.1**	-1.07	1.41E-02	2.34E-02
**RPL26P9**	-3.41	1.41E-02	2.34E-02
**AC006273.5**	1.11	1.41E-02	2.34E-02
**TUBB**	0.35	1.42E-02	2.34E-02
**RP11-253E3.3**	0.57	1.42E-02	2.34E-02
**NUDT19P5**	2.04	1.42E-02	2.34E-02
**AC007386.3**	-3.37	1.42E-02	2.34E-02
**NDUFAF6**	-0.27	1.42E-02	2.34E-02
**RP11-802D6.1**	2.04	1.42E-02	2.34E-02
**RP11-545E17.3**	0.77	1.42E-02	2.34E-02
**RP11-323F24.4**	-3.33	1.42E-02	2.35E-02
**AP001258.4**	-0.35	1.42E-02	2.35E-02
**ARHGAP28**	0.86	1.42E-02	2.35E-02
**RP5-864K19.6**	-2.45	1.42E-02	2.35E-02
**RNU6-1147P**	-3.22	1.42E-02	2.35E-02
**LAMTOR5P1**	-2.49	1.42E-02	2.35E-02
**XLOC_014397**	-2.13	1.42E-02	2.35E-02
**RP11-536C5.2**	-3.00	1.42E-02	2.35E-02
**RP11-10N16.3**	-1.06	1.43E-02	2.35E-02
**RP11-782C8.4**	-3.26	1.43E-02	2.36E-02
**ECD**	-0.25	1.43E-02	2.36E-02
**RPEP4**	-1.65	1.43E-02	2.36E-02
**XXbac-B135H6.18**	1.10	1.43E-02	2.36E-02
**FBXO30**	-0.27	1.43E-02	2.36E-02
**C19orf18**	-1.09	1.43E-02	2.36E-02
**CTD-2571E19.3**	-3.68	1.43E-02	2.36E-02
**MYOT**	-1.26	1.43E-02	2.36E-02
**C9orf66**	0.97	1.43E-02	2.36E-02
**SLC7A1**	0.46	1.43E-02	2.36E-02

**RP5-998H6.2**	-3.21	1.43E-02	2.36E-02
**ZNF83**	-0.53	1.43E-02	2.36E-02
**G3616**	-1.11	1.43E-02	2.37E-02
**AC079613.1**	-3.46	1.43E-02	2.37E-02
**SLC25A48**	-1.15	1.44E-02	2.37E-02
**FKBP4**	0.42	1.44E-02	2.37E-02
**RP1-172B20.6**	-2.02	1.44E-02	2.37E-02
**RPS6KL1**	0.64	1.44E-02	2.37E-02
**KCNMA1**	-0.57	1.44E-02	2.37E-02
**RP11-293G6__A.2**	-3.52	1.44E-02	2.37E-02
**FAM86GP**	1.36	1.44E-02	2.37E-02
**RNU6-1156P**	-2.95	1.44E-02	2.38E-02
**RP11-439E19.6**	-3.27	1.44E-02	2.38E-02
**RP11-837J7.3**	-1.36	1.44E-02	2.38E-02
**AC016722.4**	-1.31	1.44E-02	2.38E-02
**RPL39P36**	-1.21	1.44E-02	2.38E-02
**RP11-323F24.3**	-2.93	1.44E-02	2.38E-02
**RP11-799M12.2**	-1.70	1.44E-02	2.38E-02
**BANF1P2**	2.11	1.44E-02	2.38E-02
**XLOC_013962**	-1.90	1.45E-02	2.38E-02
**RPS3AP25**	-2.46	1.45E-02	2.38E-02
**TLL2**	1.28	1.45E-02	2.39E-02
**RNU6-414P**	-2.96	1.45E-02	2.39E-02
**RP11-159F24.6**	1.10	1.45E-02	2.39E-02
**FGF22**	-0.52	1.45E-02	2.39E-02
**KIF2C**	0.54	1.45E-02	2.40E-02
**RPP38**	-0.34	1.45E-02	2.40E-02
**MIR624**	-2.87	1.45E-02	2.40E-02
**XLOC_009723**	-2.09	1.45E-02	2.40E-02
**RNU4-9P**	-3.41	1.45E-02	2.40E-02
**RN7SL449P**	-3.38	1.46E-02	2.40E-02
**KRTAP12-1**	-5.72	1.46E-02	2.40E-02
**ROCK1**	0.26	1.46E-02	2.41E-02
**XLOC_004069**	-3.46	1.46E-02	2.41E-02
**LYRM1**	-0.34	1.46E-02	2.41E-02
**RP11-480G7.2**	-2.94	1.46E-02	2.41E-02
**PPP6C**	-0.35	1.46E-02	2.41E-02
**PIGA**	-0.36	1.46E-02	2.41E-02
**RTCA**	0.24	1.46E-02	2.41E-02
**TRIB3**	-0.40	1.46E-02	2.41E-02
**ANKZF1**	0.62	1.46E-02	2.41E-02
**IGLV5-52**	-3.28	1.47E-02	2.42E-02
**HKDC1**	1.35	1.47E-02	2.42E-02
**TCEB3-AS1**	-0.85	1.47E-02	2.42E-02
**XLOC_014105**	-2.47	1.47E-02	2.42E-02

**RP11-1103G16.1**	-2.76	1.47E-02	2.42E-02
**PARP8**	-0.36	1.47E-02	2.42E-02
**DHTKD1**	-0.26	1.47E-02	2.42E-02
**DEGS1**	-0.49	1.47E-02	2.42E-02
**RPL12P42**	-2.82	1.47E-02	2.42E-02
**AC009480.1**	-2.85	1.47E-02	2.42E-02
**MUC20P1**	-2.33	1.47E-02	2.42E-02
**RP11-88I18.3**	-3.33	1.47E-02	2.42E-02
**BANP**	0.40	1.47E-02	2.42E-02
**C2orf61**	-1.67	1.48E-02	2.43E-02
**RCCD1**	0.52	1.48E-02	2.43E-02
**CTD-3224K15.2**	-2.35	1.48E-02	2.43E-02
**TMPRSS2**	2.01	1.48E-02	2.43E-02
**XLOC_005040**	-3.20	1.48E-02	2.43E-02
**ANKRD20A14P**	1.58	1.48E-02	2.43E-02
**MIR4432HG**	-3.23	1.48E-02	2.43E-02
**RP11-91I20.2**	-3.02	1.48E-02	2.43E-02
**KCNG1**	1.46	1.48E-02	2.44E-02
**SUZ12**	-0.29	1.48E-02	2.44E-02
**MIR4500HG**	-1.77	1.48E-02	2.44E-02
**RASGEF1A**	0.77	1.48E-02	2.44E-02
**BNIP3P11**	-1.26	1.48E-02	2.44E-02
**LRRC46**	-1.04	1.48E-02	2.44E-02
**AC016700.6**	-3.16	1.48E-02	2.44E-02
**AL096677.1**	-3.23	1.49E-02	2.44E-02
**CTD-2616J11.10**	-3.30	1.49E-02	2.45E-02
**GNAL**	0.56	1.49E-02	2.45E-02
**CPLX2**	-2.90	1.49E-02	2.45E-02
**RP11-244F12.3**	-1.44	1.49E-02	2.45E-02
**LOXL1-AS1**	0.48	1.49E-02	2.45E-02
**SDC4**	-0.49	1.49E-02	2.45E-02
**BDH2**	-0.34	1.49E-02	2.45E-02
**G30965**	2.13	1.49E-02	2.46E-02
**RP11-10A14.4**	-1.56	1.50E-02	2.46E-02
**ANKRD10**	0.42	1.50E-02	2.46E-02
**LINC00293**	-3.94	1.50E-02	2.46E-02
**RRN3**	-0.33	1.50E-02	2.46E-02
**PIK3AP1**	1.09	1.50E-02	2.46E-02
**CH507-9B2.3**	-1.11	1.50E-02	2.46E-02
**THEG**	-1.00	1.50E-02	2.46E-02
**RP11-440L14.4**	-1.44	1.50E-02	2.46E-02
**RP11-430B1.2**	1.20	1.50E-02	2.46E-02
**RP11-802O23.4**	-3.04	1.50E-02	2.46E-02
**CD5**	-0.91	1.50E-02	2.47E-02
**XX-DJ76P10__A.2**	-3.33	1.50E-02	2.47E-02

**RNU4-31P**	-3.03	1.50E-02	2.47E-02
**MORN3**	-0.48	1.50E-02	2.47E-02
**CHCHD4P4**	-3.36	1.50E-02	2.47E-02
**RP11-432I13.6**	-1.90	1.51E-02	2.47E-02
**RP11-384O8.1**	1.02	1.51E-02	2.47E-02
**FUT1**	0.59	1.51E-02	2.48E-02
**PCDH9**	-1.07	1.51E-02	2.48E-02
**RNU6-194P**	1.94	1.51E-02	2.48E-02
**XLOC_004724**	-3.29	1.51E-02	2.48E-02
**TRMT2B-AS1**	-3.34	1.51E-02	2.48E-02
**RP11-288E14.2**	-2.77	1.51E-02	2.48E-02
**RP11-761N21.2**	-0.79	1.51E-02	2.48E-02
**TRAJ19**	-3.05	1.51E-02	2.48E-02
**KLHL38**	2.38	1.51E-02	2.48E-02
**GPR35**	1.00	1.51E-02	2.48E-02
**RP1-47A17.1**	-2.57	1.51E-02	2.49E-02
**RNU6-406P**	-4.36	1.52E-02	2.49E-02
**OSGEPL1-AS1**	-1.07	1.52E-02	2.49E-02
**TMBIM6**	0.32	1.52E-02	2.49E-02
**CTB-89H12.4**	-0.74	1.52E-02	2.49E-02
**WDR82P2**	-3.62	1.52E-02	2.49E-02
**ANKRD20A4**	-2.32	1.52E-02	2.49E-02
**RP5-1097P24.1**	-2.31	1.52E-02	2.49E-02
**GAS2**	-0.98	1.52E-02	2.49E-02
**RP11-102M11.2**	-2.70	1.52E-02	2.50E-02
**ATP11A**	-0.28	1.52E-02	2.50E-02
**HMGB1P44**	-3.09	1.52E-02	2.50E-02
**KAZALD1**	0.65	1.52E-02	2.50E-02
**XLOC_012016**	-1.94	1.53E-02	2.50E-02
**AOC2**	0.70	1.53E-02	2.50E-02
**GNPDA2**	-0.38	1.53E-02	2.50E-02
**MTND4LP7**	-3.55	1.53E-02	2.50E-02
**RP5-991G20.2**	-1.36	1.53E-02	2.50E-02
**AC098617.1**	-3.30	1.53E-02	2.51E-02
**ADGRA3**	-0.30	1.53E-02	2.51E-02
**PBX3**	-0.38	1.53E-02	2.51E-02
**TRPA1**	1.61	1.53E-02	2.51E-02
**Z69666.2**	-1.19	1.53E-02	2.51E-02
**AC002486.3**	-3.45	1.53E-02	2.51E-02
**XLOC_003803**	-2.05	1.53E-02	2.51E-02
**RP11-75C10.9**	-2.84	1.53E-02	2.51E-02
**FAM133A**	1.66	1.53E-02	2.51E-02
**DHDDS**	0.28	1.53E-02	2.52E-02
**ICOS**	-1.36	1.54E-02	2.52E-02
**PKNOX2-AS1**	-3.35	1.54E-02	2.52E-02

**UBA2**	-0.33	1.54E-02	2.52E-02
**PMS2P3**	-0.55	1.54E-02	2.52E-02
**RNU7-109P**	-3.12	1.54E-02	2.52E-02
**XLOC_005053**	-2.06	1.54E-02	2.52E-02
**ARPC3P1**	-1.76	1.54E-02	2.52E-02
**RP11-77O7.1**	-2.92	1.54E-02	2.52E-02
**MIR3941**	-2.51	1.54E-02	2.52E-02
**PRPS2**	-0.34	1.54E-02	2.52E-02
**ANKRD6**	0.64	1.54E-02	2.52E-02
**LINC00614**	-1.82	1.54E-02	2.52E-02
**CYSLTR2**	-1.00	1.54E-02	2.53E-02
**RPS10P2**	-2.30	1.54E-02	2.53E-02
**VPS35**	-0.29	1.54E-02	2.53E-02
**OR2T2**	-3.59	1.54E-02	2.53E-02
**SMARCE1P2**	-3.32	1.54E-02	2.53E-02
**POP5**	0.43	1.54E-02	2.53E-02
**SUMF1**	0.31	1.55E-02	2.53E-02
**RNA5SP48**	-3.20	1.55E-02	2.53E-02
**RP11-28H5.2**	-1.51	1.55E-02	2.53E-02
**RP11-293M10.6**	-0.73	1.55E-02	2.53E-02
**SIX4**	1.77	1.55E-02	2.53E-02
**RP11-90B9.3**	-1.31	1.55E-02	2.53E-02
**GPR84**	2.00	1.55E-02	2.53E-02
**RP11-134E15.1**	-3.42	1.55E-02	2.54E-02
**CTB-147N14.6**	1.44	1.55E-02	2.54E-02
**HSPA8P16**	-3.40	1.55E-02	2.54E-02
**CRISPLD1**	1.09	1.55E-02	2.54E-02
**GNL2**	0.27	1.55E-02	2.54E-02
**RNU6-644P**	-3.59	1.56E-02	2.54E-02
**AC012668.2**	-3.39	1.56E-02	2.54E-02
**RP11-210H10__A.1**	-2.96	1.56E-02	2.55E-02
**TWSG1**	0.40	1.56E-02	2.55E-02
**PON1**	-1.89	1.56E-02	2.55E-02
**RP11-466A19.6**	-2.97	1.56E-02	2.55E-02
**RP11-307L14.2**	-2.98	1.56E-02	2.55E-02
**GRIP2**	-0.86	1.56E-02	2.55E-02
**RNA5SP18**	2.44	1.56E-02	2.56E-02
**FCER2**	-2.00	1.57E-02	2.56E-02
**PRR7**	0.59	1.57E-02	2.56E-02
**ASNSP5**	-3.14	1.57E-02	2.56E-02
**RP11-280G9.1**	-3.54	1.57E-02	2.57E-02
**RP13-516M14.10**	1.58	1.57E-02	2.57E-02
**G27456**	-1.01	1.57E-02	2.57E-02
**ANKRD52**	0.51	1.57E-02	2.57E-02
**ARSB**	0.67	1.57E-02	2.57E-02

**hsa-mir-150**	-3.27	1.57E-02	2.57E-02
**RP11-834C11.15**	-1.41	1.57E-02	2.57E-02
**EML1**	-0.37	1.58E-02	2.58E-02
**HNRNPR**	-0.29	1.58E-02	2.58E-02
**DKKL1**	2.58	1.58E-02	2.58E-02
**OR10A6**	-2.89	1.58E-02	2.58E-02
**SALL4**	1.42	1.58E-02	2.58E-02
**FAS**	-0.51	1.58E-02	2.58E-02
**RP11-326C3.11**	1.03	1.58E-02	2.58E-02
**RNA5SP494**	-2.79	1.58E-02	2.58E-02
**RNU6-615P**	-3.68	1.58E-02	2.59E-02
**AC074212.6**	1.31	1.58E-02	2.59E-02
**ILF2**	0.30	1.58E-02	2.59E-02
**AC017035.2**	2.38	1.58E-02	2.59E-02
**TMEM56-RWDD3**	-1.40	1.58E-02	2.59E-02
**NWD2**	-0.95	1.58E-02	2.59E-02
**RTKN**	0.35	1.58E-02	2.59E-02
**AQP2**	3.75	1.58E-02	2.59E-02
**RP11-527J8.1**	-0.98	1.59E-02	2.59E-02
**CTD-2337A12.1**	-1.04	1.59E-02	2.59E-02
**HCG22**	2.88	1.59E-02	2.59E-02
**BTNL9**	1.04	1.59E-02	2.59E-02
**RP11-317N8.3**	-2.10	1.59E-02	2.60E-02
**ITGAD**	1.88	1.59E-02	2.60E-02
**CYB5B**	0.28	1.59E-02	2.60E-02
**SUMO2**	0.40	1.60E-02	2.61E-02
**ALG5**	0.38	1.60E-02	2.61E-02
**TIFA**	-0.47	1.60E-02	2.61E-02
**RP1-292B18.1**	-3.05	1.60E-02	2.61E-02
**RP11-109A6.3**	-0.99	1.60E-02	2.61E-02
**ZNF32**	0.38	1.60E-02	2.61E-02
**AC002066.1**	-1.80	1.60E-02	2.61E-02
**RPSAP72**	-3.23	1.60E-02	2.62E-02
**SCPEP1**	0.43	1.60E-02	2.62E-02
**FAM90A2P**	-2.59	1.60E-02	2.62E-02
**HIPK1-AS1**	-0.94	1.61E-02	2.62E-02
**LYSMD1**	0.28	1.61E-02	2.62E-02
**RASA2-IT1**	-1.72	1.61E-02	2.62E-02
**ODC1**	0.75	1.61E-02	2.62E-02
**HNRNPA1P29**	-3.23	1.61E-02	2.63E-02
**USP32P1**	-2.67	1.61E-02	2.63E-02
**E2F1**	0.46	1.61E-02	2.63E-02
**AC078842.4**	-3.30	1.61E-02	2.63E-02
**RP11-455O6.5**	-2.76	1.61E-02	2.63E-02
**XLOC_002710**	-2.89	1.61E-02	2.63E-02

**LA16c-431H6.6**	-1.21	1.61E-02	2.63E-02
**RIMKLB**	0.45	1.62E-02	2.63E-02
**SAXO1**	-1.31	1.62E-02	2.64E-02
**SLC26A2**	-0.67	1.62E-02	2.64E-02
**RP13-977J11.6**	-2.69	1.62E-02	2.64E-02
**RP11-219B4.7**	-2.32	1.62E-02	2.64E-02
**RP11-776A13.3**	-2.76	1.62E-02	2.64E-02
**RP11-550A5.2**	-2.51	1.63E-02	2.65E-02
**SLC22A8**	-2.88	1.63E-02	2.65E-02
**GMCL1**	-0.32	1.63E-02	2.65E-02
**RAB5B**	-0.29	1.63E-02	2.66E-02
**DFFBP1**	-2.25	1.63E-02	2.66E-02
**AC073621.2**	1.67	1.63E-02	2.66E-02
**RP11-338K17.6**	-2.02	1.63E-02	2.66E-02
**AMMECR1**	-0.44	1.63E-02	2.66E-02
**CTSG**	1.47	1.63E-02	2.66E-02
**ANKRD22**	-0.41	1.63E-02	2.66E-02
**CTD-2043I16.1**	-3.60	1.63E-02	2.66E-02
**SLC17A7**	0.95	1.63E-02	2.66E-02
**RP11-578B16.1**	-3.55	1.63E-02	2.66E-02
**RIMS1**	-1.89	1.63E-02	2.66E-02
**DSCAML1**	-1.43	1.63E-02	2.66E-02
**SMKR1**	1.46	1.63E-02	2.66E-02
**MIR1180**	-2.90	1.63E-02	2.66E-02
**RP11-810P12.7**	2.14	1.63E-02	2.66E-02
**ATP6V0CP1**	-2.21	1.64E-02	2.67E-02
**CTD-2008L17.1**	2.64	1.64E-02	2.67E-02
**XIAP-AS1**	-1.68	1.64E-02	2.67E-02
**CHRNB4**	1.96	1.64E-02	2.67E-02
**XLOC_009863**	-3.42	1.64E-02	2.67E-02
**RP11-351M16.4**	-2.08	1.64E-02	2.67E-02
**RPL32P3**	-0.65	1.64E-02	2.67E-02
**CTB-3M24.3**	-3.36	1.64E-02	2.67E-02
**AC026366.1**	-3.10	1.64E-02	2.67E-02
**ESRG**	1.35	1.64E-02	2.67E-02
**RP11-135A24.4**	-2.08	1.64E-02	2.67E-02
**RNU7-4P**	-2.95	1.64E-02	2.67E-02
**RP11-817I4.1**	1.02	1.64E-02	2.68E-02
**STAT4**	0.93	1.64E-02	2.68E-02
**MYADML**	-3.04	1.65E-02	2.68E-02
**CTB-171A8.1**	2.24	1.65E-02	2.68E-02
**CLSTN2**	-0.80	1.65E-02	2.68E-02
**RP11-196H14.2**	-3.55	1.65E-02	2.68E-02
**SIRPB2**	1.17	1.65E-02	2.68E-02
**TNFSF4**	0.98	1.65E-02	2.68E-02

**MIR936**	-1.52	1.65E-02	2.69E-02
**GJB3**	-0.42	1.65E-02	2.69E-02
**CD59**	0.44	1.65E-02	2.69E-02
**HMGA1P5**	2.04	1.65E-02	2.69E-02
**SLC12A6**	-0.28	1.65E-02	2.69E-02
**SLC31A1**	-0.45	1.66E-02	2.69E-02
**RP11-43A14.1**	-2.40	1.66E-02	2.69E-02
**RNU6-890P**	-1.69	1.66E-02	2.69E-02
**RP11-574F11.3**	-2.32	1.66E-02	2.70E-02
**LINC01218**	-3.65	1.66E-02	2.70E-02
**AL359922.1**	-2.58	1.66E-02	2.70E-02
**BABAM1**	0.32	1.66E-02	2.70E-02
**RNU6-936P**	-3.13	1.66E-02	2.70E-02
**XLOC_004677**	-1.71	1.66E-02	2.70E-02
**RP11-439K3.3**	-3.01	1.66E-02	2.70E-02
**TMEM50B**	-0.24	1.66E-02	2.70E-02
**PCDHB5**	0.86	1.66E-02	2.70E-02
**DEFB109P1**	-3.36	1.66E-02	2.70E-02
**PRPF18**	-0.29	1.66E-02	2.71E-02
**MTRF1LP1**	-2.20	1.67E-02	2.71E-02
**RP1-41C23.4**	-1.37	1.67E-02	2.71E-02
**RP11-932O9.9**	-2.29	1.67E-02	2.71E-02
**FDCSP**	4.20	1.67E-02	2.71E-02
**G15372**	-1.21	1.67E-02	2.71E-02
**YTHDC1**	0.26	1.67E-02	2.71E-02
**RP1-140K8.5**	1.48	1.67E-02	2.71E-02
**RP11-103J8.2**	-3.20	1.67E-02	2.71E-02
**DNHD1**	-0.74	1.67E-02	2.71E-02
**G26247**	-1.35	1.67E-02	2.71E-02
**KRTAP24-1**	-6.56	1.67E-02	2.72E-02
**RP11-567M16.1**	1.28	1.67E-02	2.72E-02
**IMPA1**	0.38	1.67E-02	2.72E-02
**RP4-756H11.5**	0.35	1.67E-02	2.72E-02
**SNORD116-29**	-3.66	1.67E-02	2.72E-02
**RP11-379C10.4**	-2.85	1.67E-02	2.72E-02
**RNU6-859P**	-3.75	1.67E-02	2.72E-02
**SUSD1**	0.64	1.67E-02	2.72E-02
**FOXC1**	1.12	1.68E-02	2.72E-02
**FOXH1**	-1.62	1.68E-02	2.72E-02
**RPL7AP64**	-0.72	1.68E-02	2.72E-02
**AP000704.5**	-2.68	1.68E-02	2.72E-02
**CTD-2228K2.2**	-3.22	1.68E-02	2.72E-02
**RSPH3**	-0.29	1.68E-02	2.72E-02
**CARS2**	0.36	1.68E-02	2.72E-02
**RP11-573D15.8**	1.37	1.68E-02	2.72E-02

**RP11-420A6.2**	-1.47	1.68E-02	2.72E-02
**RP11-776H12.1**	-2.47	1.68E-02	2.72E-02
**IQCG**	-0.29	1.68E-02	2.73E-02
**RP11-122K13.7**	-2.23	1.68E-02	2.73E-02
**XLOC_005569**	-3.48	1.68E-02	2.73E-02
**PSTK**	-0.39	1.68E-02	2.73E-02
**METTL4**	-0.30	1.68E-02	2.73E-02
**AHSA1**	0.35	1.68E-02	2.73E-02
**RP11-381K20.2**	-1.35	1.68E-02	2.73E-02
**GOLGA6L9**	-1.62	1.68E-02	2.73E-02
**RNU6-399P**	-3.31	1.68E-02	2.73E-02
**LLNLR-276H7.1**	-3.13	1.68E-02	2.73E-02
**RP11-370A5.1**	-1.61	1.69E-02	2.74E-02
**NQO2**	0.60	1.69E-02	2.74E-02
**AC092377.1**	-3.46	1.69E-02	2.74E-02
**RP11-144F15.1**	-2.07	1.69E-02	2.74E-02
**RN7SL300P**	-3.02	1.69E-02	2.74E-02
**CSMD3**	-3.22	1.69E-02	2.74E-02
**G32993**	1.24	1.69E-02	2.74E-02
**UMODL1**	-1.53	1.69E-02	2.74E-02
**IL9RP3**	-2.05	1.69E-02	2.74E-02
**MVD**	0.85	1.69E-02	2.74E-02
**FAM27E2**	-3.50	1.69E-02	2.74E-02
**KB-1639H6.2**	-3.32	1.69E-02	2.74E-02
**RN7SKP296**	-1.57	1.69E-02	2.74E-02
**RP11-830F9.6**	1.96	1.69E-02	2.74E-02
**P2RY10**	-0.97	1.70E-02	2.75E-02
**OR1F12**	-1.91	1.70E-02	2.75E-02
**TSSK3**	-1.53	1.70E-02	2.75E-02
**PER2**	-0.63	1.70E-02	2.75E-02
**RP11-211N8.2**	-1.06	1.70E-02	2.75E-02
**C1orf210**	0.45	1.70E-02	2.75E-02
**AC016745.1**	-2.42	1.70E-02	2.75E-02
**RP1-278O22.2**	-1.74	1.70E-02	2.76E-02
**TMEM141**	0.41	1.70E-02	2.76E-02
**RP11-44D5.2**	-3.20	1.70E-02	2.76E-02
**LINC01102**	-2.75	1.70E-02	2.76E-02
**THAP11**	0.31	1.70E-02	2.76E-02
**RP11-544I20.2**	-1.20	1.71E-02	2.76E-02
**SDR42E2**	-1.46	1.71E-02	2.76E-02
**G42897**	-0.83	1.71E-02	2.77E-02
**PCDHA7**	2.28	1.71E-02	2.77E-02
**RP11-158H5.2**	-1.21	1.71E-02	2.77E-02
**PCDHA9**	-2.88	1.71E-02	2.77E-02
**G3BP2**	-0.33	1.71E-02	2.77E-02

**ZSCAN31**	0.80	1.71E-02	2.77E-02
**RP11-517C16.4**	-2.13	1.71E-02	2.77E-02
**RP11-227D13.4**	-2.79	1.71E-02	2.77E-02
**CTD-2553L13.5**	-2.20	1.71E-02	2.77E-02
**LMAN1**	0.40	1.71E-02	2.78E-02
**FAM66B**	-0.83	1.71E-02	2.78E-02
**PELI1**	-0.37	1.71E-02	2.78E-02
**RP11-80H18.3**	-1.91	1.71E-02	2.78E-02
**CLDN12**	-0.29	1.72E-02	2.78E-02
**TMED10**	-0.31	1.72E-02	2.78E-02
**SNRPCP9**	-3.41	1.72E-02	2.78E-02
**RNU6-517P**	-3.30	1.72E-02	2.78E-02
**RP11-95I19.1**	-2.74	1.72E-02	2.78E-02
**CMB9-94B1.2**	-2.47	1.72E-02	2.79E-02
**EEF1A1P10**	-2.86	1.72E-02	2.79E-02
**RP11-432N13.2**	-3.41	1.72E-02	2.79E-02
**AC114808.2**	-3.02	1.72E-02	2.79E-02
**RP11-424C20.2**	0.87	1.73E-02	2.79E-02
**CTC-281F24.5**	0.85	1.73E-02	2.79E-02
**MIR548A3**	-3.39	1.73E-02	2.79E-02
**KCTD9P1**	-3.30	1.73E-02	2.80E-02
**RNU6-875P**	-3.09	1.73E-02	2.80E-02
**BNIP3P5**	-2.73	1.73E-02	2.80E-02
**DENND2D**	-0.43	1.73E-02	2.80E-02
**RBFA**	-0.38	1.73E-02	2.80E-02
**RP11-726G1.1**	-1.01	1.73E-02	2.80E-02
**TRAJ21**	-3.06	1.73E-02	2.80E-02
**CSPG4P12**	1.06	1.73E-02	2.81E-02
**RP4-781K5.6**	-2.29	1.73E-02	2.81E-02
**LYPLA1**	-0.61	1.74E-02	2.81E-02
**CTD-2373N4.3**	-1.05	1.74E-02	2.81E-02
**IGLON5**	1.35	1.74E-02	2.81E-02
**ADH1A**	-2.71	1.74E-02	2.81E-02
**RP11-335G20.7**	-2.09	1.74E-02	2.82E-02
**MIR326**	-2.94	1.74E-02	2.82E-02
**HTR4**	-1.90	1.75E-02	2.82E-02
**RNU6-335P**	-2.85	1.75E-02	2.83E-02
**CD177**	2.81	1.75E-02	2.83E-02
**MIR4513**	-3.22	1.75E-02	2.83E-02
**NPTN**	0.26	1.75E-02	2.83E-02
**CTA-384D8.31**	3.19	1.75E-02	2.83E-02
**G15987**	-2.76	1.75E-02	2.83E-02
**BCAP31P2**	2.49	1.75E-02	2.83E-02
**CTD-2541M15.3**	-1.93	1.75E-02	2.83E-02
**PPME1**	0.27	1.75E-02	2.83E-02

**KRTAP8-1**	-3.25	1.75E-02	2.83E-02
**AC096649.2**	-3.27	1.75E-02	2.83E-02
**RP11-572F4.1**	-2.94	1.75E-02	2.83E-02
**RP11-114F10.2**	-1.37	1.75E-02	2.84E-02
**RP1-15D23.2**	-2.00	1.76E-02	2.84E-02
**RP11-473M20.16**	-0.81	1.76E-02	2.84E-02
**IGFBP5**	-0.69	1.76E-02	2.84E-02
**AGAP5**	-1.74	1.76E-02	2.84E-02
**RP11-242F11.2**	-3.26	1.76E-02	2.84E-02
**CTD-2619J13.3**	-1.02	1.76E-02	2.84E-02
**ARHGEF37**	-0.44	1.76E-02	2.84E-02
**TSPYL4**	0.26	1.76E-02	2.84E-02
**RP11-428G2.1**	-3.78	1.76E-02	2.84E-02
**RAB12**	-0.32	1.76E-02	2.84E-02
**SNORD93**	-2.59	1.76E-02	2.84E-02
**NUP85**	0.25	1.76E-02	2.84E-02
**RP11-327J17.2**	1.96	1.76E-02	2.85E-02
**RP11-242D8.3**	-2.69	1.76E-02	2.85E-02
**NENFP1**	-3.09	1.76E-02	2.85E-02
**USP27X-AS1**	0.56	1.76E-02	2.85E-02
**UNC13A**	1.63	1.76E-02	2.85E-02
**ACTR8**	-0.25	1.76E-02	2.85E-02
**MTERF4**	0.27	1.77E-02	2.85E-02
**LCA5**	0.66	1.77E-02	2.85E-02
**BCL2A1**	1.00	1.77E-02	2.85E-02
**KDM4E**	-2.12	1.77E-02	2.85E-02
**RP11-110G21.1**	0.40	1.77E-02	2.86E-02
**G12733**	2.35	1.77E-02	2.86E-02
**CTSV**	-0.64	1.77E-02	2.86E-02
**NDUFB4**	0.39	1.77E-02	2.86E-02
**CD3D**	1.20	1.77E-02	2.86E-02
**FAM214A**	-0.40	1.77E-02	2.86E-02
**ADRA1B**	1.83	1.77E-02	2.86E-02
**RN7SKP134**	-3.08	1.77E-02	2.86E-02
**NBPF16**	-0.72	1.77E-02	2.86E-02
**G42267**	-2.75	1.77E-02	2.86E-02
**RP11-367B6.2**	-3.02	1.78E-02	2.86E-02
**RP11-734K21.5**	-1.15	1.78E-02	2.86E-02
**AC104534.2**	-1.32	1.78E-02	2.87E-02
**PPP1R3D**	0.32	1.78E-02	2.87E-02
**MIR4252**	-3.27	1.78E-02	2.87E-02
**MEIS3P2**	1.24	1.78E-02	2.87E-02
**RN7SL506P**	-3.17	1.78E-02	2.88E-02
**STOX1**	-0.83	1.78E-02	2.88E-02
**Z98049.1**	-1.63	1.78E-02	2.88E-02

**RP11-775D22.3**	-1.65	1.79E-02	2.88E-02
**CCDC57**	0.57	1.79E-02	2.88E-02
**RHEBP2**	1.82	1.79E-02	2.88E-02
**IGKV6-21**	-2.43	1.79E-02	2.89E-02
**LINC01621**	1.48	1.79E-02	2.89E-02
**C1orf145**	-0.92	1.79E-02	2.89E-02
**CTTNBP2**	-0.61	1.79E-02	2.89E-02
**CTD-2325P2.3**	1.69	1.79E-02	2.89E-02
**MYOZ1**	1.33	1.79E-02	2.89E-02
**RP11-555K12.2**	-1.39	1.80E-02	2.89E-02
**RP11-73M18.10**	-1.22	1.80E-02	2.89E-02
**RP11-85B7.2**	-1.72	1.80E-02	2.90E-02
**RP11-461A8.5**	-2.79	1.80E-02	2.90E-02
**RNU6-711P**	-2.37	1.80E-02	2.90E-02
**ARL6IP1**	-0.36	1.80E-02	2.90E-02
**AC005954.3**	-3.55	1.80E-02	2.90E-02
**AC009229.5**	-2.17	1.80E-02	2.90E-02
**RPL39P26**	-2.85	1.80E-02	2.90E-02
**RP11-589N15.2**	-0.89	1.80E-02	2.90E-02
**RP11-24B13.2**	-2.10	1.80E-02	2.90E-02
**DCAF13**	-0.31	1.80E-02	2.90E-02
**RP11-457M11.6**	-3.19	1.80E-02	2.90E-02
**RP11-973H7.3**	-0.59	1.80E-02	2.90E-02
**RP11-665C16.6**	-2.24	1.80E-02	2.91E-02
**ADAMTS9-AS1**	-1.09	1.80E-02	2.91E-02
**LINC01354**	1.45	1.80E-02	2.91E-02
**TLR6**	-0.59	1.81E-02	2.91E-02
**RP11-20O24.4**	-0.65	1.81E-02	2.91E-02
**RP11-489M13.1**	-3.19	1.81E-02	2.91E-02
**XLOC_007894**	-2.02	1.81E-02	2.91E-02
**KB-1615E4.2**	-3.24	1.81E-02	2.91E-02
**INHA**	1.03	1.81E-02	2.92E-02
**ARMC10**	-0.27	1.81E-02	2.92E-02
**CFAP47**	-2.76	1.81E-02	2.92E-02
**LRRC37A5P**	1.93	1.81E-02	2.92E-02
**RPL23AP95**	-2.28	1.81E-02	2.92E-02
**RNU6ATAC23P**	-3.40	1.81E-02	2.92E-02
**GATSL2**	-1.38	1.81E-02	2.92E-02
**CTD-2330K9.3**	-2.52	1.81E-02	2.92E-02
**ISCA2**	0.30	1.82E-02	2.92E-02
**RP11-181K12.1**	-2.58	1.82E-02	2.92E-02
**HIBADH**	0.45	1.82E-02	2.93E-02
**CTD-2515H24.2**	-1.27	1.82E-02	2.93E-02
**RP11-572M11.3**	-1.37	1.82E-02	2.93E-02
**CWF19L2**	0.35	1.82E-02	2.93E-02

**LRRC49**	-0.42	1.82E-02	2.93E-02
**G353**	1.70	1.82E-02	2.93E-02
**IGKV1-17**	5.82	1.82E-02	2.93E-02
**RP11-1100L3.8**	2.90	1.82E-02	2.93E-02
**AC062029.1**	0.70	1.82E-02	2.93E-02
**G22346**	-1.55	1.83E-02	2.94E-02
**CLIC5**	1.05	1.83E-02	2.94E-02
**RP11-73M18.11**	-1.54	1.83E-02	2.94E-02
**CFAP61**	-1.38	1.83E-02	2.94E-02
**MTMR2**	-0.25	1.83E-02	2.94E-02
**RP11-380O24.1**	-3.02	1.83E-02	2.94E-02
**RBMS3-AS1**	-3.48	1.83E-02	2.94E-02
**RP11-293M10.1**	-2.37	1.83E-02	2.94E-02
**RP11-303E16.7**	-2.85	1.83E-02	2.94E-02
**IGKC**	2.05	1.83E-02	2.94E-02
**RP11-108L7.15**	1.31	1.83E-02	2.94E-02
**FAM138B**	-3.12	1.83E-02	2.94E-02
**CFAP57**	-0.98	1.83E-02	2.95E-02
**PLXNA1**	0.28	1.83E-02	2.95E-02
**RP11-12J10.3**	-1.34	1.83E-02	2.95E-02
**CCL14**	1.23	1.84E-02	2.95E-02
**HIST2H2BD**	1.62	1.84E-02	2.95E-02
**HLA-G**	-1.99	1.84E-02	2.96E-02
**AC145343.2**	3.18	1.84E-02	2.96E-02
**AC004019.13**	-2.61	1.84E-02	2.96E-02
**AC016735.1**	-2.84	1.84E-02	2.96E-02
**RP11-756H20.1**	-3.41	1.84E-02	2.96E-02
**EEF1D**	-0.26	1.84E-02	2.96E-02
**RP1-168L15.6**	-1.76	1.84E-02	2.96E-02
**AF011889.2**	-1.81	1.85E-02	2.97E-02
**USP14**	-0.29	1.85E-02	2.97E-02
**KATNBL1P2**	-2.88	1.85E-02	2.97E-02
**CTA-444M12.3**	-3.00	1.85E-02	2.97E-02
**CTD-2574D22.2**	-0.76	1.85E-02	2.97E-02
**RP11-174N3.4**	-2.89	1.85E-02	2.97E-02
**RP11-73O6.3**	-2.55	1.85E-02	2.97E-02
**G25266**	-3.26	1.85E-02	2.97E-02
**CLDN10**	2.98	1.85E-02	2.98E-02
**XLOC_008674**	-1.86	1.85E-02	2.98E-02
**RAET1G**	0.72	1.86E-02	2.98E-02
**RP11-476H24.1**	-3.38	1.86E-02	2.98E-02
**RPL39P40**	2.10	1.86E-02	2.98E-02
**RTN1**	-0.81	1.86E-02	2.98E-02
**RP11-45F15.1**	-3.45	1.86E-02	2.98E-02
**RGPD6**	-1.69	1.86E-02	2.98E-02

**RP11-333J10.2**	-2.47	1.86E-02	2.98E-02
**RNU6-188P**	-3.06	1.86E-02	2.98E-02
**CAMKK1**	0.52	1.86E-02	2.98E-02
**AC015849.13**	-1.00	1.86E-02	2.98E-02
**ALG1L13P**	-1.69	1.86E-02	2.98E-02
**SGCZ**	-2.58	1.86E-02	2.99E-02
**RP11-640I2.1**	-2.91	1.86E-02	2.99E-02
**KCNK12**	1.20	1.86E-02	2.99E-02
**MRPS10P2**	-2.77	1.86E-02	2.99E-02
**TMEM220**	-0.35	1.87E-02	2.99E-02
**RYR2**	-0.72	1.87E-02	2.99E-02
**NDUFA11**	-0.42	1.87E-02	2.99E-02
**TCEAL1**	0.42	1.87E-02	2.99E-02
**SIAH2**	0.33	1.87E-02	2.99E-02
**MYOF**	0.25	1.87E-02	2.99E-02
**TACSTD2**	0.37	1.87E-02	3.00E-02
**RPSAP13**	-2.28	1.87E-02	3.00E-02
**KDSR**	-0.27	1.87E-02	3.00E-02
**RP11-13N13.6**	-2.31	1.87E-02	3.00E-02
**RP11-809N8.6**	-3.32	1.87E-02	3.01E-02
**LEO1**	-0.37	1.87E-02	3.01E-02
**RNU1-63P**	-2.89	1.88E-02	3.01E-02
**HNRNPA1P66**	-3.13	1.88E-02	3.01E-02
**PPFIBP2**	0.26	1.88E-02	3.01E-02
**ARRDC3**	-0.43	1.88E-02	3.01E-02
**CACNA1B**	1.49	1.88E-02	3.01E-02
**SUCLG2P2**	-2.74	1.88E-02	3.01E-02
**MIR548O**	-2.07	1.88E-02	3.01E-02
**SRPX2**	1.12	1.88E-02	3.01E-02
**FAM155B**	2.35	1.88E-02	3.01E-02
**CTC-529I10.2**	-0.91	1.88E-02	3.02E-02
**RNU7-110P**	-3.35	1.88E-02	3.02E-02
**RP11-570L15.2**	-3.42	1.88E-02	3.02E-02
**HP1BP3**	-0.23	1.88E-02	3.02E-02
**RP11-788M5.4**	-3.16	1.89E-02	3.02E-02
**RP11-715J22.6**	-1.44	1.89E-02	3.02E-02
**RP11-1252D15.1**	1.17	1.89E-02	3.02E-02
**MIR1271**	-2.86	1.89E-02	3.02E-02
**MIR4480**	-2.99	1.89E-02	3.03E-02
**POLR3A**	-0.29	1.89E-02	3.03E-02
**AL022345.10**	-2.12	1.89E-02	3.03E-02
**XLOC_002876**	-3.09	1.89E-02	3.03E-02
**RP5-837J1.6**	-2.15	1.89E-02	3.03E-02
**CSTF2**	0.31	1.89E-02	3.03E-02
**RP11-185E8.2**	1.53	1.90E-02	3.03E-02

**MGC32805**	1.67	1.90E-02	3.04E-02
**G22509**	1.77	1.90E-02	3.04E-02
**MOV10**	-0.31	1.90E-02	3.04E-02
**RP11-87N24.1**	-3.65	1.90E-02	3.04E-02
**EMX1**	-1.32	1.90E-02	3.04E-02
**RP5-1052I5.2**	-3.45	1.90E-02	3.04E-02
**SORD**	-0.62	1.90E-02	3.04E-02
**RNU6-808P**	-2.82	1.90E-02	3.04E-02
**DERA**	0.30	1.90E-02	3.05E-02
**CSTF1**	-0.23	1.91E-02	3.05E-02
**CPEB1-AS1**	-2.98	1.91E-02	3.05E-02
**G13548**	-2.54	1.91E-02	3.05E-02
**RPRD1B**	-0.18	1.91E-02	3.05E-02
**DDX20**	-0.26	1.91E-02	3.05E-02
**DBH**	2.60	1.91E-02	3.05E-02
**RP11-343H19.1**	-1.77	1.91E-02	3.05E-02
**CACYBPP2**	-1.58	1.91E-02	3.06E-02
**AIM1L**	0.48	1.91E-02	3.06E-02
**SNORD56B**	-3.42	1.91E-02	3.06E-02
**RP11-355N15.1**	-3.10	1.91E-02	3.06E-02
**ATP5G2P3**	-2.83	1.91E-02	3.06E-02
**KB-1396H2.2**	-2.46	1.91E-02	3.06E-02
**RP11-462G12.1**	1.65	1.91E-02	3.06E-02
**CCDC74B**	0.97	1.91E-02	3.06E-02
**SYF2P2**	-2.91	1.92E-02	3.07E-02
**SNX18**	-0.30	1.92E-02	3.07E-02
**LINC00907**	-1.53	1.92E-02	3.07E-02
**RP11-421N8.1**	-2.06	1.92E-02	3.07E-02
**RP11-317N8.4**	-2.99	1.92E-02	3.07E-02
**RP5-1073O3.2**	2.30	1.92E-02	3.07E-02
**RP11-429E11.2**	1.21	1.92E-02	3.08E-02
**RP11-254A24.2**	-1.82	1.92E-02	3.08E-02
**Z95704.5**	-1.31	1.93E-02	3.08E-02
**RNA5SP300**	-3.10	1.93E-02	3.08E-02
**TMEM147-AS1**	-0.69	1.93E-02	3.08E-02
**RP11-244H3.1**	1.10	1.93E-02	3.08E-02
**XLOC_008109**	1.55	1.93E-02	3.08E-02
**LINC01607**	2.03	1.93E-02	3.09E-02
**RCOR1**	-0.25	1.93E-02	3.09E-02
**PCYT1B**	1.07	1.93E-02	3.09E-02
**MTCP1**	-0.90	1.93E-02	3.09E-02
**PET100**	-0.65	1.93E-02	3.09E-02
**STAT2**	0.27	1.93E-02	3.09E-02
**YEATS2**	0.33	1.94E-02	3.09E-02
**RP4-740C4.7**	-1.18	1.94E-02	3.09E-02

**G43427**	-2.40	1.94E-02	3.10E-02
**ZNF429**	-0.47	1.94E-02	3.10E-02
**ERHP2**	-2.69	1.94E-02	3.10E-02
**AC005152.3**	2.31	1.94E-02	3.10E-02
**COL6A5**	-1.33	1.94E-02	3.10E-02
**RP11-468O2.1**	-1.63	1.94E-02	3.10E-02
**PIGK**	-0.37	1.94E-02	3.10E-02
**AC110769.3**	-2.29	1.94E-02	3.10E-02
**SERPINA11**	1.79	1.94E-02	3.10E-02
**RP11-872D17.8**	-2.32	1.95E-02	3.11E-02
**RP11-82L20.1**	-3.63	1.95E-02	3.11E-02
**ATP6V0E2**	0.55	1.95E-02	3.11E-02
**RPS26P28**	-2.66	1.95E-02	3.11E-02
**RP11-134G8.7**	0.98	1.95E-02	3.11E-02
**GSTP1P1**	-2.86	1.95E-02	3.11E-02
**AC073957.15**	-3.26	1.95E-02	3.11E-02
**RP11-923I11.4**	-3.50	1.95E-02	3.11E-02
**RP11-85G20.1**	-3.24	1.95E-02	3.11E-02
**GPRC5D**	-1.17	1.95E-02	3.12E-02
**FBN3**	-2.00	1.95E-02	3.12E-02
**HSPE1P28**	-2.09	1.95E-02	3.12E-02
**USP2-AS1**	1.64	1.96E-02	3.12E-02
**RP11-529E10.7**	-1.30	1.96E-02	3.12E-02
**RNU1-91P**	-2.42	1.96E-02	3.12E-02
**RPL23AP86**	-2.75	1.96E-02	3.12E-02
**RP11-111A21.1**	-2.18	1.96E-02	3.13E-02
**RP11-563J2.3**	-1.35	1.96E-02	3.13E-02
**RP1-100J12.1**	-2.81	1.96E-02	3.13E-02
**GM2A**	0.33	1.96E-02	3.13E-02
**LA16c-361A3.3**	-1.12	1.97E-02	3.14E-02
**RP11-83B20.3**	-2.53	1.97E-02	3.14E-02
**RNA5SP203**	-2.45	1.97E-02	3.14E-02
**PITHD1**	0.34	1.97E-02	3.14E-02
**AC104772.1**	-3.90	1.97E-02	3.14E-02
**RP11-347C18.1**	-2.37	1.97E-02	3.15E-02
**AC108488.4**	1.12	1.97E-02	3.15E-02
**XLOC_011382**	-2.45	1.98E-02	3.15E-02
**TTL**	0.29	1.98E-02	3.16E-02
**LINC01569**	0.81	1.98E-02	3.16E-02
**BOD1L1**	-0.28	1.98E-02	3.16E-02
**CTD-2227C6.2**	-3.18	1.98E-02	3.16E-02
**H3F3B**	0.48	1.98E-02	3.16E-02
**AC138647.1**	-1.96	1.99E-02	3.17E-02
**IGFBP6**	1.05	1.99E-02	3.17E-02
**SLC22A3**	-0.38	1.99E-02	3.17E-02

**Z83001.1**	-1.31	1.99E-02	3.18E-02
**RP11-63M22.1**	-1.21	1.99E-02	3.18E-02
**AC008781.7**	-2.76	1.99E-02	3.18E-02
**ADHFE1**	-0.86	2.00E-02	3.18E-02
**TBC1D24**	-0.40	2.00E-02	3.18E-02
**PRSS3**	0.57	2.00E-02	3.18E-02
**AC104457.1**	-3.00	2.00E-02	3.18E-02
**MIR3157**	-3.30	2.00E-02	3.19E-02
**G26252**	-2.79	2.00E-02	3.19E-02
**RP11-57B24.1**	-3.05	2.00E-02	3.19E-02
**APH1B**	-0.46	2.01E-02	3.20E-02
**RNASE2**	1.57	2.01E-02	3.20E-02
**FBP2**	-3.55	2.01E-02	3.20E-02
**C20orf144**	-1.22	2.01E-02	3.20E-02
**G34082**	2.41	2.01E-02	3.20E-02
**RP11-542B15.1**	2.96	2.01E-02	3.20E-02
**AC074338.4**	-1.58	2.01E-02	3.20E-02
**RP11-424M21.1**	-1.23	2.02E-02	3.21E-02
**RP11-575H3.1**	-3.15	2.02E-02	3.22E-02
**RP11-510M2.2**	-2.44	2.02E-02	3.22E-02
**ISCA1**	0.34	2.02E-02	3.22E-02
**HMGB1P3**	-2.89	2.02E-02	3.22E-02
**G358**	-1.13	2.02E-02	3.22E-02
**RNF114**	0.22	2.02E-02	3.22E-02
**MBD5**	-0.36	2.02E-02	3.22E-02
**HERPUD2**	0.20	2.03E-02	3.23E-02
**SEMA3A**	1.15	2.03E-02	3.23E-02
**CNTNAP3P5**	-3.53	2.03E-02	3.23E-02
**RP11-466A19.8**	-1.60	2.03E-02	3.23E-02
**AC005159.1**	-3.43	2.03E-02	3.23E-02
**RP11-91P24.1**	-1.30	2.03E-02	3.24E-02
**RNA5SP219**	-1.44	2.03E-02	3.24E-02
**ZNF286B**	-0.42	2.04E-02	3.24E-02
**RP11-397E7.4**	1.12	2.04E-02	3.24E-02
**SMIM10L1**	0.48	2.04E-02	3.24E-02
**CTD-2302E22.6**	-1.53	2.04E-02	3.25E-02
**RPS3A**	-0.39	2.04E-02	3.25E-02
**TMEM206**	0.41	2.05E-02	3.26E-02
**ZNF511**	0.50	2.05E-02	3.26E-02
**PPCS**	0.32	2.05E-02	3.26E-02
**GOLGA2P5**	-0.78	2.05E-02	3.26E-02
**RP13-514E23.1**	-2.79	2.05E-02	3.27E-02
**LRTM1**	-3.12	2.05E-02	3.27E-02
**OSTCP2**	-3.35	2.05E-02	3.27E-02
**HIGD1AP16**	-2.27	2.05E-02	3.27E-02

**EHD4-AS1**	-1.45	2.05E-02	3.27E-02
**RP11-712L6.8**	-2.96	2.06E-02	3.27E-02
**DDX50**	-0.28	2.06E-02	3.27E-02
**PCNPP5**	-2.96	2.06E-02	3.28E-02
**CHEK2**	0.39	2.06E-02	3.28E-02
**PRSS30P**	2.02	2.06E-02	3.28E-02
**DYNAP**	2.53	2.06E-02	3.28E-02
**CDCA4**	0.36	2.06E-02	3.28E-02
**RP11-473M20.9**	1.09	2.07E-02	3.29E-02
**KLC3**	0.55	2.07E-02	3.29E-02
**POLR3D**	0.28	2.07E-02	3.29E-02
**MAB21L3**	-0.53	2.07E-02	3.29E-02
**CTD-2574D22.3**	-0.86	2.07E-02	3.29E-02
**CD48**	1.08	2.07E-02	3.29E-02
**RP11-351A11.1**	-1.64	2.07E-02	3.29E-02
**LINC01018**	-1.20	2.07E-02	3.29E-02
**FZD5**	0.66	2.07E-02	3.30E-02
**RP11-469H8.6**	2.48	2.07E-02	3.30E-02
**RP11-227G15.9**	-2.09	2.08E-02	3.30E-02
**RP11-248G5.9**	-1.68	2.08E-02	3.30E-02
**SRSF6**	0.38	2.08E-02	3.30E-02
**NPPC**	1.39	2.08E-02	3.30E-02
**AC005752.10**	-1.47	2.08E-02	3.30E-02
**RP11-277P12.9**	-1.71	2.08E-02	3.30E-02
**MRPL52**	0.24	2.08E-02	3.30E-02
**RP11-687M24.7**	-2.69	2.08E-02	3.30E-02
**RP11-443F16.1**	-2.31	2.08E-02	3.30E-02
**RP11-253A20.1**	-1.52	2.08E-02	3.30E-02
**NAAA**	0.38	2.08E-02	3.31E-02
**SCUBE2**	0.62	2.08E-02	3.31E-02
**RP11-162A12.4**	-1.84	2.08E-02	3.31E-02
**RNU2-7P**	-2.47	2.08E-02	3.31E-02
**RPL13AP20**	1.31	2.09E-02	3.31E-02
**RP11-932O9.8**	-2.11	2.09E-02	3.31E-02
**RPL15P4**	-3.33	2.09E-02	3.31E-02
**PPP1R16A**	0.45	2.09E-02	3.31E-02
**RP11-248M19.1**	-1.15	2.09E-02	3.32E-02
**CHI3L1**	1.47	2.09E-02	3.32E-02
**KTN1**	-0.37	2.09E-02	3.32E-02
**SSTR1**	1.20	2.09E-02	3.32E-02
**CTC-497E21.5**	-2.13	2.09E-02	3.32E-02
**GLG1**	-0.26	2.09E-02	3.32E-02
**AC063932.1**	-3.40	2.09E-02	3.32E-02
**FRMPD2**	2.46	2.09E-02	3.32E-02
**G17366**	1.52	2.09E-02	3.32E-02

**RP4-781K5.9**	-3.07	2.10E-02	3.33E-02
**CTA-384D8.35**	1.57	2.10E-02	3.33E-02
**MTCYBP23**	-3.41	2.10E-02	3.33E-02
**IFFO2**	-0.46	2.10E-02	3.33E-02
**PADI3**	-1.84	2.10E-02	3.33E-02
**GTF2IP20**	0.64	2.10E-02	3.34E-02
**CASC10**	0.51	2.11E-02	3.34E-02
**ANKRD12**	0.36	2.11E-02	3.34E-02
**RP11-667K14.8**	-1.88	2.11E-02	3.35E-02
**C2orf72**	1.54	2.11E-02	3.35E-02
**RP11-259G18.3**	-5.37	2.11E-02	3.35E-02
**NOL6**	0.35	2.11E-02	3.35E-02
**MTCO3P15**	-3.30	2.12E-02	3.35E-02
**EGLN2**	-0.59	2.12E-02	3.35E-02
**OTUD7B**	-0.24	2.12E-02	3.36E-02
**ANO7**	1.15	2.12E-02	3.36E-02
**PABPC1P3**	-1.17	2.12E-02	3.36E-02
**SOX11**	2.19	2.12E-02	3.36E-02
**TSKS**	2.20	2.12E-02	3.36E-02
**FBXO32**	0.50	2.12E-02	3.36E-02
**ESRP1**	-0.35	2.12E-02	3.36E-02
**TRDC**	1.39	2.12E-02	3.36E-02
**LINC01060**	2.64	2.12E-02	3.36E-02
**EEF1A1P39**	-3.36	2.12E-02	3.36E-02
**MICAL1**	0.38	2.12E-02	3.36E-02
**ZNF536**	-1.42	2.12E-02	3.36E-02
**AC008703.1**	-2.92	2.12E-02	3.36E-02
**NHLRC1**	0.46	2.12E-02	3.36E-02
**GS1-259H13.2**	-0.97	2.12E-02	3.36E-02
**SPERT**	2.52	2.12E-02	3.36E-02
**MIR32**	-2.90	2.12E-02	3.37E-02
**CPN2**	3.40	2.12E-02	3.37E-02
**AMDHD1**	-0.84	2.13E-02	3.37E-02
**NOL7**	-0.41	2.13E-02	3.37E-02
**RP11-91P24.3**	-2.85	2.13E-02	3.37E-02
**AC011239.2**	-1.41	2.13E-02	3.37E-02
**RP11-12A20.12**	-3.25	2.13E-02	3.37E-02
**CTD-2521M24.6**	1.66	2.13E-02	3.38E-02
**DEFB108B**	-2.95	2.13E-02	3.38E-02
**MTND4P19**	-2.89	2.13E-02	3.38E-02
**PLCE1-AS2**	-3.14	2.13E-02	3.38E-02
**AC110084.1**	-1.64	2.13E-02	3.38E-02
**RNA5SP385**	-2.30	2.13E-02	3.38E-02
**XLOC_002945**	-3.03	2.14E-02	3.38E-02
**RP11-686D22.4**	1.62	2.14E-02	3.38E-02

**SATB2**	0.61	2.14E-02	3.38E-02
**CTB-31O20.8**	-2.09	2.14E-02	3.39E-02
**ZP3**	0.63	2.14E-02	3.39E-02
**RP11-686O6.1**	-2.84	2.14E-02	3.39E-02
**C4BPA**	2.60	2.14E-02	3.39E-02
**TBPL1**	-0.27	2.14E-02	3.39E-02
**SCARNA14**	-2.98	2.14E-02	3.39E-02
**AC079466.1**	-3.25	2.14E-02	3.39E-02
**TEFM**	-0.34	2.15E-02	3.40E-02
**LINC01159**	-2.88	2.15E-02	3.40E-02
**PCDHA4**	1.68	2.15E-02	3.40E-02
**RP11-403I13.7**	0.74	2.15E-02	3.40E-02
**CTC-1337H24.3**	-2.42	2.15E-02	3.40E-02
**TUBAP4**	-3.18	2.15E-02	3.41E-02
**MIR3682**	-1.25	2.15E-02	3.41E-02
**RN7SL364P**	1.74	2.16E-02	3.41E-02
**RP6-91H8.3**	2.66	2.16E-02	3.41E-02
**RP11-603J24.14**	-2.84	2.16E-02	3.41E-02
**RP11-282O18.7**	-2.19	2.16E-02	3.41E-02
**XLOC_010475**	-3.05	2.16E-02	3.42E-02
**G11405**	-2.05	2.16E-02	3.42E-02
**GPR75-ASB3**	-2.75	2.16E-02	3.42E-02
**ZNF740**	0.22	2.16E-02	3.42E-02
**AC005076.5**	1.03	2.16E-02	3.42E-02
**G32346**	-3.07	2.17E-02	3.42E-02
**RNU6-216P**	-2.35	2.17E-02	3.42E-02
**IGDCC3**	1.90	2.17E-02	3.43E-02
**LRRFIP1P1**	1.28	2.17E-02	3.43E-02
**AEBP2**	-0.32	2.17E-02	3.43E-02
**ZBTB14**	-0.23	2.17E-02	3.43E-02
**MRPS23**	0.28	2.17E-02	3.43E-02
**FUCA1P1**	-3.08	2.17E-02	3.44E-02
**RP1-302G2.5**	1.60	2.17E-02	3.44E-02
**RPL17**	-0.43	2.18E-02	3.44E-02
**SNORA73**	-1.86	2.18E-02	3.44E-02
**TMEM190**	-2.65	2.18E-02	3.44E-02
**LINC01237**	-1.27	2.18E-02	3.44E-02
**RP11-63L7.5**	-2.86	2.18E-02	3.44E-02
**DIO3**	1.29	2.18E-02	3.44E-02
**DRICH1**	-1.27	2.18E-02	3.44E-02
**RP13-122B23.8**	2.33	2.18E-02	3.45E-02
**RNU6-1177P**	-2.86	2.18E-02	3.45E-02
**RP11-3D4.2**	-1.12	2.18E-02	3.45E-02
**RP11-484D2.5**	-1.27	2.19E-02	3.45E-02
**RP11-148L24.1**	-1.89	2.19E-02	3.46E-02

**SIRPG**	1.87	2.19E-02	3.46E-02
**RPL21P54**	-3.10	2.19E-02	3.46E-02
**RP11-284B18.3**	-1.43	2.19E-02	3.46E-02
**YAF2**	-0.30	2.19E-02	3.46E-02
**RSU1P3**	-2.91	2.20E-02	3.47E-02
**UBE2D1**	0.39	2.20E-02	3.47E-02
**RNU6-571P**	-2.81	2.20E-02	3.47E-02
**MAT2B**	0.33	2.20E-02	3.47E-02
**RP11-258C19.7**	-0.60	2.20E-02	3.47E-02
**RP11-384A12.1**	-3.06	2.20E-02	3.47E-02
**SDHAF3**	0.44	2.20E-02	3.47E-02
**FAM189A2**	-0.74	2.20E-02	3.47E-02
**RN7SL629P**	-2.54	2.20E-02	3.48E-02
**PHF24**	-0.79	2.20E-02	3.48E-02
**RP11-712L6.5**	0.77	2.20E-02	3.48E-02
**ZDHHC2**	-0.87	2.21E-02	3.48E-02
**CFHR1**	5.74	2.21E-02	3.49E-02
**TFP1**	-1.34	2.21E-02	3.49E-02
**AC007246.3**	-0.37	2.21E-02	3.49E-02
**AC002398.11**	-3.54	2.21E-02	3.49E-02
**RPS15AP1**	1.09	2.21E-02	3.49E-02
**HSPA7**	0.82	2.21E-02	3.49E-02
**PGAM1P5**	-2.12	2.21E-02	3.49E-02
**RP11-219O3.2**	-3.37	2.21E-02	3.49E-02
**SYT13**	1.99	2.21E-02	3.49E-02
**VTA1**	-0.32	2.21E-02	3.49E-02
**ARHGAP40**	-0.63	2.22E-02	3.50E-02
**ENTPD4**	-0.31	2.22E-02	3.50E-02
**FAM104A**	-0.26	2.22E-02	3.50E-02
**C3orf67-AS1**	-3.47	2.22E-02	3.50E-02
**OR2A3P**	-2.66	2.22E-02	3.50E-02
**STX16-NPEPL1**	-1.43	2.22E-02	3.50E-02
**SCN5A**	-1.24	2.22E-02	3.50E-02
**G264**	0.95	2.22E-02	3.50E-02
**RSPH10B**	-2.47	2.22E-02	3.50E-02
**KCNT1**	1.30	2.22E-02	3.50E-02
**RP11-18H21.1**	2.79	2.22E-02	3.50E-02
**GLUD1**	-0.33	2.22E-02	3.50E-02
**RP11-6N17.4**	0.52	2.22E-02	3.51E-02
**C15orf41**	-0.36	2.23E-02	3.51E-02
**RP11-165M1.2**	-2.72	2.23E-02	3.51E-02
**BZW1**	0.39	2.23E-02	3.51E-02
**AC015933.2**	-3.27	2.23E-02	3.51E-02
**CAT**	-0.55	2.23E-02	3.51E-02
**PHKA1P1**	-1.69	2.23E-02	3.52E-02

**ZPBP**	-1.53	2.23E-02	3.52E-02
**AC005534.8**	-2.11	2.23E-02	3.52E-02
**RP11-196E1.3**	-2.47	2.23E-02	3.52E-02
**ALG9-IT1**	-2.82	2.23E-02	3.52E-02
**CTD-2165H16.3**	-2.74	2.24E-02	3.52E-02
**RP11-452G18.1**	-2.86	2.24E-02	3.53E-02
**RP11-340F14.6**	-1.00	2.24E-02	3.53E-02
**RN7SL724P**	-1.98	2.24E-02	3.53E-02
**RP11-655M14.13**	-1.06	2.24E-02	3.53E-02
**CD180**	1.22	2.24E-02	3.53E-02
**RP11-734J24.1**	-2.05	2.24E-02	3.53E-02
**KLK6**	2.01	2.25E-02	3.54E-02
**RPL23AP10**	-2.25	2.25E-02	3.54E-02
**ERC1**	-0.29	2.25E-02	3.55E-02
**RP11-282E4.1**	-2.14	2.25E-02	3.55E-02
**RP11-30K9.1**	-2.63	2.25E-02	3.55E-02
**NDUFA1**	0.40	2.25E-02	3.55E-02
**RP11-126F18.2**	-2.37	2.25E-02	3.55E-02
**ZFYVE21**	0.26	2.26E-02	3.56E-02
**RP11-208G20.2**	-3.10	2.26E-02	3.56E-02
**LINC01107**	-3.15	2.26E-02	3.56E-02
**TRPM5**	-2.58	2.26E-02	3.56E-02
**ZNF233**	-0.87	2.26E-02	3.56E-02
**RP11-478B9.1**	-3.40	2.27E-02	3.57E-02
**MXRA5Y**	1.24	2.27E-02	3.57E-02
**RP5-1153D9.5**	-2.93	2.27E-02	3.57E-02
**RP11-25B7.1**	-2.34	2.27E-02	3.57E-02
**RAP1AP**	-2.81	2.27E-02	3.57E-02
**MIR3164**	-2.86	2.27E-02	3.57E-02
**SESN1**	-0.38	2.27E-02	3.57E-02
**DPY19L1P2**	-1.82	2.27E-02	3.58E-02
**CPQ**	0.57	2.27E-02	3.58E-02
**G18713**	1.74	2.27E-02	3.58E-02
**PRED62**	2.39	2.27E-02	3.58E-02
**XLOC_005339**	-2.48	2.28E-02	3.58E-02
**ZNF251**	0.35	2.28E-02	3.58E-02
**NSA2**	0.46	2.28E-02	3.59E-02
**HIST1H2APS3**	-3.30	2.28E-02	3.59E-02
**RP11-837J7.4**	-2.78	2.28E-02	3.59E-02
**MIR3137**	-3.25	2.28E-02	3.59E-02
**ARCN1**	0.24	2.28E-02	3.59E-02
**AQP7**	1.65	2.29E-02	3.60E-02
**XLOC_012879**	2.12	2.29E-02	3.60E-02
**MGAT4A**	-0.48	2.29E-02	3.60E-02
**RP11-764K9.1**	-2.93	2.29E-02	3.60E-02

**RP11-505K9.4**	-1.38	2.29E-02	3.60E-02
**RP11-499E18.1**	1.08	2.29E-02	3.61E-02
**CCNA1**	2.49	2.30E-02	3.61E-02
**A4GNT**	-1.71	2.30E-02	3.61E-02
**ETV5**	0.51	2.30E-02	3.61E-02
**RP11-359G22.2**	2.97	2.30E-02	3.61E-02
**OAS3**	0.60	2.30E-02	3.61E-02
**RP11-21B23.2**	-0.89	2.30E-02	3.61E-02
**RAX**	1.43	2.30E-02	3.61E-02
**RACGAP1**	-0.33	2.30E-02	3.62E-02
**RP11-333E1.1**	-1.21	2.30E-02	3.62E-02
**RN7SKP217**	-3.15	2.30E-02	3.62E-02
**RP11-204C16.4**	-1.97	2.30E-02	3.62E-02
**RP11-713P17.5**	-3.65	2.30E-02	3.62E-02
**PGAM4**	-2.16	2.30E-02	3.62E-02
**RP11-944L7.4**	-0.94	2.31E-02	3.63E-02
**RP11-304L19.2**	-2.93	2.31E-02	3.63E-02
**HNRNPA1P37**	-3.16	2.31E-02	3.63E-02
**KL**	0.80	2.31E-02	3.63E-02
**RP11-370A5.2**	2.16	2.31E-02	3.63E-02
**KCNS3**	0.61	2.31E-02	3.63E-02
**CTC-526N19.1**	-0.47	2.31E-02	3.63E-02
**AC096574.5**	-2.73	2.31E-02	3.63E-02
**OVOS2**	1.28	2.31E-02	3.63E-02
**RNU6-288P**	-3.15	2.31E-02	3.64E-02
**RP11-497H17.1**	-1.41	2.32E-02	3.64E-02
**SORBS2**	0.65	2.32E-02	3.64E-02
**FAM103A2P**	1.67	2.32E-02	3.64E-02
**SMAP1**	0.33	2.32E-02	3.65E-02
**RP11-31H5.2**	-3.31	2.32E-02	3.65E-02
**CTC-459F4.9**	-0.86	2.32E-02	3.65E-02
**RP11-106D4.3**	-2.70	2.32E-02	3.65E-02
**SIRT4**	0.53	2.33E-02	3.65E-02
**RERG-AS1**	-2.91	2.33E-02	3.66E-02
**AC104986.1**	-2.61	2.33E-02	3.66E-02
**XLOC_010144**	-3.36	2.33E-02	3.66E-02
**ZC3H13**	0.26	2.34E-02	3.67E-02
**KIF14**	-0.56	2.34E-02	3.67E-02
**PAK1IP1**	0.40	2.34E-02	3.67E-02
**SPDYE21P**	-1.04	2.34E-02	3.67E-02
**RP11-338L18.1**	-3.25	2.34E-02	3.67E-02
**TMEM120B**	-0.39	2.34E-02	3.68E-02
**AC006028.11**	-3.25	2.34E-02	3.68E-02
**NPIPB5**	0.97	2.34E-02	3.68E-02
**AMZ1**	-1.05	2.34E-02	3.68E-02

**AC007790.4**	-1.60	2.34E-02	3.68E-02
**G42642**	-0.65	2.34E-02	3.68E-02
**KRT16P4**	1.31	2.35E-02	3.68E-02
**G40762**	-0.55	2.35E-02	3.68E-02
**GRK6P1**	-1.63	2.35E-02	3.68E-02
**CSTF3**	-0.29	2.35E-02	3.68E-02
**RP11-556H2.2**	-3.43	2.35E-02	3.68E-02
**ASPRV1**	0.68	2.35E-02	3.68E-02
**XLOC_000974**	-1.74	2.35E-02	3.68E-02
**FAM81A**	0.61	2.35E-02	3.68E-02
**TGFA**	-0.42	2.35E-02	3.68E-02
**ARL15**	0.36	2.35E-02	3.68E-02
**SMNDC1**	-0.25	2.35E-02	3.68E-02
**C15orf59-AS1**	-1.73	2.35E-02	3.69E-02
**RP11-214K3.23**	-2.00	2.35E-02	3.69E-02
**GS1-358P8.4**	-0.50	2.35E-02	3.69E-02
**PNRC2P1**	-2.70	2.36E-02	3.69E-02
**CTB-60B18.12**	-2.01	2.36E-02	3.69E-02
**SNAP91**	-3.12	2.36E-02	3.70E-02
**FKBP9P1**	0.78	2.36E-02	3.70E-02
**ZNF692**	0.73	2.36E-02	3.70E-02
**C8orf59P2**	-3.02	2.36E-02	3.70E-02
**RP11-115N4.1**	2.09	2.36E-02	3.71E-02
**RP11-505P4.7**	-2.95	2.36E-02	3.71E-02
**UCA1**	-0.95	2.37E-02	3.71E-02
**RP11-719K4.7**	-3.26	2.37E-02	3.71E-02
**KRTAP12-2**	-4.89	2.37E-02	3.71E-02
**AC083875.1**	-3.00	2.37E-02	3.71E-02
**RNU6-344P**	-3.44	2.37E-02	3.71E-02
**MCF2**	-2.56	2.37E-02	3.71E-02
**ELF4**	0.37	2.37E-02	3.71E-02
**DCX**	-2.19	2.37E-02	3.72E-02
**TMEM9B**	-0.34	2.37E-02	3.72E-02
**FIGNL2**	1.53	2.37E-02	3.72E-02
**LCTL**	1.93	2.38E-02	3.72E-02
**TSACC**	-1.52	2.38E-02	3.72E-02
**PRC1**	-0.32	2.38E-02	3.72E-02
**SLC6A1**	1.51	2.38E-02	3.72E-02
**AL117334.1**	-3.14	2.38E-02	3.73E-02
**LRCH2**	0.95	2.38E-02	3.73E-02
**SEMA7A**	0.44	2.38E-02	3.73E-02
**C14orf142**	0.35	2.38E-02	3.73E-02
**LPXN**	0.58	2.39E-02	3.74E-02
**IL1R1**	0.39	2.39E-02	3.74E-02
**RP11-530N7.3**	-1.50	2.39E-02	3.74E-02

**ABRACL**	0.50	2.39E-02	3.74E-02
**AC024704.2**	-3.20	2.39E-02	3.74E-02
**RP11-361H10.5**	-2.83	2.39E-02	3.75E-02
**RP13-143G15.4**	-1.49	2.39E-02	3.75E-02
**G36656**	4.13	2.39E-02	3.75E-02
**B4GALT3**	0.23	2.40E-02	3.75E-02
**RHOA-IT1**	-1.32	2.40E-02	3.75E-02
**HNF1A**	-2.92	2.40E-02	3.76E-02
**AC005488.11**	-2.92	2.40E-02	3.76E-02
**FNTA**	-0.19	2.40E-02	3.76E-02
**THRA1/BTR**	-2.62	2.41E-02	3.77E-02
**RP11-74E22.3**	1.17	2.41E-02	3.77E-02
**CTD-2007H13.1**	-2.84	2.41E-02	3.77E-02
**AC009274.6**	-2.28	2.41E-02	3.77E-02
**RP11-10O17.3**	-2.08	2.41E-02	3.77E-02
**CTD-2203K17.1**	1.30	2.41E-02	3.77E-02
**MIR425**	-3.08	2.41E-02	3.78E-02
**PLA2G6**	0.66	2.41E-02	3.78E-02
**KB-1205A7.2**	-2.34	2.42E-02	3.78E-02
**ZNF665**	-0.44	2.42E-02	3.78E-02
**UBAC2-AS1**	-0.59	2.42E-02	3.79E-02
**RP11-38G5.2**	-2.80	2.42E-02	3.79E-02
**RP11-227D13.2**	-3.01	2.43E-02	3.80E-02
**TUBB8P7**	1.90	2.43E-02	3.80E-02
**GBAS**	0.31	2.43E-02	3.80E-02
**RP11-687M24.8**	-2.72	2.43E-02	3.80E-02
**RP13-104F24.2**	-1.00	2.43E-02	3.80E-02
**RP11-255P5.3**	-3.08	2.43E-02	3.80E-02
**RP11-745L13.2**	-2.83	2.43E-02	3.80E-02
**CENPM**	0.70	2.43E-02	3.81E-02
**RNF133**	-2.70	2.43E-02	3.81E-02
**ZNF529-AS1**	-0.64	2.44E-02	3.81E-02
**CARD18**	-0.80	2.44E-02	3.81E-02
**RPL35P2**	0.91	2.44E-02	3.82E-02
**LINC00847**	0.61	2.44E-02	3.82E-02
**RP11-25K19.1**	1.38	2.45E-02	3.82E-02
**DLC1**	0.68	2.45E-02	3.82E-02
**METAP1D**	0.49	2.45E-02	3.82E-02
**AC007381.3**	-1.04	2.45E-02	3.82E-02
**DLAT**	-0.34	2.45E-02	3.83E-02
**AC026150.8**	-1.31	2.45E-02	3.83E-02
**XXbac-BPG252P9.10**	-3.05	2.45E-02	3.83E-02
**RP11-337N6.2**	-1.07	2.45E-02	3.83E-02
**MIR708**	-1.95	2.45E-02	3.83E-02
**DDX3P2**	-3.32	2.45E-02	3.83E-02

**MAGEE1**	0.36	2.45E-02	3.83E-02
**RP11-855A2.1**	0.99	2.46E-02	3.84E-02
**RP11-817O13.8**	0.65	2.46E-02	3.84E-02
**SAMSN1**	1.11	2.46E-02	3.84E-02
**CTC-215O4.4**	-1.36	2.46E-02	3.84E-02
**RP11-134D3.1**	-3.06	2.46E-02	3.85E-02
**RASAL1**	0.52	2.46E-02	3.85E-02
**G2447**	-1.42	2.47E-02	3.85E-02
**SERP2**	0.93	2.47E-02	3.85E-02
**RP11-461L13.4**	-2.87	2.47E-02	3.85E-02
**CENPA**	-0.68	2.47E-02	3.86E-02
**PNMA6A**	2.01	2.47E-02	3.86E-02
**RP11-97N19.2**	-2.62	2.47E-02	3.86E-02
**RNU6-10P**	-2.89	2.47E-02	3.86E-02
**CTD-2350J17.1**	-3.24	2.47E-02	3.86E-02
**RAE1**	0.20	2.47E-02	3.86E-02
**CCDC43**	-0.33	2.47E-02	3.86E-02
**METTL12**	-0.64	2.48E-02	3.87E-02
**MIR5010**	-1.78	2.48E-02	3.87E-02
**RP11-629N8.4**	-3.02	2.48E-02	3.87E-02
**XLOC_011506**	-2.76	2.48E-02	3.88E-02
**C2orf44**	-0.32	2.49E-02	3.88E-02
**RP11-175B9.2**	-1.56	2.49E-02	3.88E-02
**ARMCX3**	0.48	2.49E-02	3.89E-02
**LINGO2**	-1.87	2.49E-02	3.89E-02
**RP11-318C24.1**	-2.89	2.49E-02	3.89E-02
**GFPT2**	0.86	2.49E-02	3.89E-02
**G15477**	-3.81	2.49E-02	3.89E-02
**PTPN2**	0.19	2.49E-02	3.89E-02
**RP11-672L10.2**	4.53	2.49E-02	3.89E-02
**TRAJ29**	-3.28	2.49E-02	3.89E-02
**CTD-2547L16.3**	-1.95	2.50E-02	3.89E-02
**XLOC_011549**	-3.63	2.50E-02	3.89E-02
**DEK**	-0.35	2.50E-02	3.90E-02
**RP11-305M3.2**	1.57	2.50E-02	3.90E-02
**AC187652.1**	1.97	2.50E-02	3.90E-02
**RP11-538D16.2**	-2.61	2.50E-02	3.90E-02
**NANP**	-0.34	2.50E-02	3.90E-02
**CTD-3051D23.1**	-2.13	2.50E-02	3.90E-02
**RN7SL698P**	-2.30	2.50E-02	3.90E-02
**RP11-251G23.5**	-0.62	2.50E-02	3.91E-02
**ERICH6B**	-2.67	2.51E-02	3.91E-02
**PPP3CC**	0.36	2.51E-02	3.91E-02
**LINC00504**	-0.74	2.51E-02	3.91E-02
**RP11-349F21.2**	-3.04	2.51E-02	3.91E-02

**RP11-2E17.2**	-2.71	2.51E-02	3.92E-02
**G8296**	-2.78	2.51E-02	3.92E-02
**RHOH**	0.91	2.51E-02	3.92E-02
**RP11-1020A11.1**	-1.33	2.51E-02	3.92E-02
**MAB21L1**	-1.33	2.51E-02	3.92E-02
**AC018643.4**	-2.90	2.52E-02	3.92E-02
**RNU6-476P**	-2.51	2.52E-02	3.92E-02
**RP11-30L3.2**	-1.61	2.52E-02	3.92E-02
**AMIGO1**	-0.52	2.52E-02	3.93E-02
**EPHX2**	-0.47	2.52E-02	3.93E-02
**AC079922.2**	-2.79	2.52E-02	3.93E-02
**NISCH**	0.39	2.52E-02	3.93E-02
**CTGLF11P**	-3.17	2.52E-02	3.93E-02
**TAGLN3**	-2.66	2.53E-02	3.93E-02
**RPL30P13**	-2.99	2.53E-02	3.94E-02
**DNAH10OS**	0.98	2.53E-02	3.94E-02
**RNU2-38P**	-1.98	2.53E-02	3.94E-02
**SUSD4**	0.82	2.53E-02	3.94E-02
**G11194**	-1.70	2.53E-02	3.94E-02
**RP5-965G21.4**	1.38	2.53E-02	3.94E-02
**KRTAP1-1**	-4.67	2.53E-02	3.94E-02
**PHF5CP**	-2.79	2.53E-02	3.95E-02
**ANKRD24**	0.94	2.53E-02	3.95E-02
**TMPRSS3**	1.52	2.54E-02	3.95E-02
**SLC2A1-AS1**	-0.83	2.54E-02	3.95E-02
**RN7SL412P**	-2.20	2.54E-02	3.96E-02
**RECQL4**	0.55	2.54E-02	3.96E-02
**NTM**	0.82	2.54E-02	3.96E-02
**CDRT1**	-1.21	2.54E-02	3.96E-02
**RP11-54C4.3**	0.58	2.54E-02	3.96E-02
**UBE2F-SCLY**	-2.84	2.55E-02	3.96E-02
**YTHDF2**	0.26	2.55E-02	3.97E-02
**LINC01054**	-2.36	2.55E-02	3.97E-02
**TRAPPC12-AS1**	-0.80	2.55E-02	3.97E-02
**MGC45922**	-1.87	2.55E-02	3.97E-02
**RNU6-611P**	1.56	2.56E-02	3.98E-02
**CTB-35F21.1**	-2.89	2.56E-02	3.98E-02
**OLR1**	2.80	2.56E-02	3.98E-02
**RP11-400F19.18**	-0.84	2.56E-02	3.98E-02
**CLUHP3**	-0.56	2.56E-02	3.99E-02
**CXCR3**	1.40	2.56E-02	3.99E-02
**KLK12**	-0.94	2.57E-02	3.99E-02
**RP11-475D10.4**	-3.46	2.57E-02	3.99E-02
**CPT1A**	0.45	2.57E-02	3.99E-02
**SYTL4**	0.90	2.57E-02	3.99E-02

**RP11-77G23.5**	-2.74	2.57E-02	4.00E-02
**RN7SKP160**	-1.61	2.57E-02	4.00E-02
**ATG4A**	0.39	2.57E-02	4.00E-02
**GCKR**	2.09	2.57E-02	4.00E-02
**CTD-2012K14.8**	1.36	2.58E-02	4.01E-02
**PDE3B**	-0.56	2.58E-02	4.01E-02
**SLFN12L**	-0.86	2.58E-02	4.01E-02
**AC110926.4**	-2.62	2.58E-02	4.01E-02
**TNPO3**	-0.18	2.58E-02	4.02E-02
**RP11-247C2.2**	1.87	2.59E-02	4.02E-02
**RP11-815J4.6**	1.72	2.59E-02	4.02E-02
**ST6GALNAC5**	1.17	2.59E-02	4.02E-02
**AC006116.12**	-2.50	2.59E-02	4.02E-02
**TXNDC9**	-0.41	2.59E-02	4.03E-02
**RP11-863K10.2**	-3.77	2.59E-02	4.03E-02
**RP3-508I15.20**	-1.40	2.59E-02	4.03E-02
**PDZD7**	0.99	2.59E-02	4.03E-02
**SH3YL1**	-0.57	2.59E-02	4.03E-02
**G32306**	-1.93	2.59E-02	4.03E-02
**CERS4**	0.51	2.60E-02	4.03E-02
**CTNND1**	-0.26	2.60E-02	4.03E-02
**IKBKB**	0.36	2.60E-02	4.03E-02
**RP11-588D3.1**	-3.18	2.60E-02	4.03E-02
**RN7SL350P**	-3.00	2.60E-02	4.03E-02
**RP11-305O6.3**	-1.26	2.60E-02	4.04E-02
**RP11-423H2.3**	1.31	2.60E-02	4.04E-02
**RP11-361I14.2**	-1.98	2.60E-02	4.04E-02
**CTC-542B22.2**	-0.98	2.60E-02	4.04E-02
**SCLY**	-0.80	2.60E-02	4.04E-02
**AC066692.3**	-2.19	2.60E-02	4.05E-02
**MPPED2**	1.18	2.61E-02	4.05E-02
**AC004980.9**	-3.03	2.61E-02	4.05E-02
**AKT3-IT1**	-2.37	2.61E-02	4.05E-02
**EIF3KP1**	-2.91	2.61E-02	4.05E-02
**MTND1P11**	-2.97	2.61E-02	4.05E-02
**ICA1L**	-0.57	2.61E-02	4.05E-02
**AC000036.4**	-3.30	2.61E-02	4.05E-02
**RNU6-118P**	-1.85	2.61E-02	4.05E-02
**RP11-250B2.3**	-1.28	2.61E-02	4.05E-02
**AC016909.2**	-3.33	2.61E-02	4.06E-02
**RP11-492M23.2**	-2.74	2.61E-02	4.06E-02
**FCF1P2**	0.37	2.61E-02	4.06E-02
**NTAN1**	0.53	2.61E-02	4.06E-02
**DAP3**	0.25	2.61E-02	4.06E-02
**ARHGAP30**	0.58	2.62E-02	4.06E-02

**C5orf17**	3.27	2.62E-02	4.06E-02
**RP11-282O18.3**	-0.58	2.62E-02	4.06E-02
**DYM**	-0.17	2.62E-02	4.06E-02
**AP001172.3**	-3.09	2.62E-02	4.06E-02
**FIG4**	-0.27	2.62E-02	4.06E-02
**SP9**	2.31	2.62E-02	4.07E-02
**G19546**	1.39	2.62E-02	4.07E-02
**CRIP2**	0.59	2.63E-02	4.07E-02
**FAM204CP**	-2.91	2.63E-02	4.07E-02
**RP11-517P14.7**	-1.96	2.63E-02	4.08E-02
**VIPAS39**	0.22	2.63E-02	4.08E-02
**RP11-353N14.4**	-2.90	2.63E-02	4.08E-02
**RPS2P45**	-1.60	2.63E-02	4.08E-02
**CKS2**	0.53	2.63E-02	4.08E-02
**RP11-283G6.3**	1.22	2.63E-02	4.08E-02
**RNU6-519P**	-2.90	2.64E-02	4.09E-02
**RBBP9**	-0.36	2.64E-02	4.09E-02
**CTC-254B4.1**	-2.44	2.64E-02	4.09E-02
**C9orf3**	0.38	2.64E-02	4.09E-02
**LEP**	3.51	2.64E-02	4.10E-02
**PRRC1**	-0.31	2.64E-02	4.10E-02
**TUBAP2**	-1.89	2.65E-02	4.10E-02
**RP11-472N13.3**	1.81	2.65E-02	4.10E-02
**SLC16A13**	0.61	2.65E-02	4.10E-02
**RP1-97D16.1**	-2.45	2.65E-02	4.10E-02
**ZNF335**	0.42	2.65E-02	4.10E-02
**SNORD105**	-3.50	2.65E-02	4.10E-02
**RP11-287J9.1**	-3.28	2.65E-02	4.11E-02
**CTD-2560E9.3**	-2.35	2.65E-02	4.11E-02
**RP1-77H15.1**	-1.88	2.65E-02	4.11E-02
**RP11-286N22.14**	-2.58	2.65E-02	4.11E-02
**RP11-716H6.1**	-3.40	2.66E-02	4.12E-02
**C1orf204**	-0.64	2.66E-02	4.12E-02
**LIPE-AS1**	1.01	2.66E-02	4.12E-02
**XLOC_008224**	-3.53	2.66E-02	4.12E-02
**RP11-212E4.1**	2.71	2.66E-02	4.12E-02
**ZNF32-AS1**	-1.39	2.66E-02	4.12E-02
**INE1**	-0.97	2.66E-02	4.12E-02
**NEURL2**	-1.14	2.66E-02	4.12E-02
**FOXA1**	1.92	2.66E-02	4.12E-02
**TWISTNB**	-0.33	2.66E-02	4.12E-02
**KB-226F1.1**	-2.73	2.66E-02	4.12E-02
**SIPA1L3**	-0.34	2.67E-02	4.13E-02
**FOXN2**	-0.39	2.67E-02	4.13E-02
**RPL12P9**	-3.03	2.67E-02	4.14E-02

**PLAGL1**	-0.43	2.68E-02	4.14E-02
**PRSS21**	1.28	2.68E-02	4.15E-02
**LINC00311**	2.17	2.68E-02	4.15E-02
**RP11-87C12.2**	-3.01	2.68E-02	4.15E-02
**RP11-561N12.8**	-3.80	2.68E-02	4.15E-02
**NBEAP1**	-1.69	2.68E-02	4.15E-02
**G37023**	2.73	2.68E-02	4.15E-02
**RAB9B**	-0.66	2.68E-02	4.16E-02
**C9orf38**	-1.15	2.69E-02	4.16E-02
**IMPG2**	-0.72	2.69E-02	4.16E-02
**FAM86C2P**	0.42	2.69E-02	4.16E-02
**RPS6**	0.34	2.69E-02	4.16E-02
**POT1**	-0.31	2.69E-02	4.17E-02
**XRCC6BP1**	-0.44	2.69E-02	4.17E-02
**PRKG1**	0.71	2.69E-02	4.17E-02
**KTI12**	0.39	2.70E-02	4.17E-02
**NUDT2**	0.53	2.70E-02	4.17E-02
**RNY4P19**	-2.62	2.70E-02	4.17E-02
**RP11-540A21.2**	-0.87	2.70E-02	4.18E-02
**TAF1D**	-0.34	2.70E-02	4.18E-02
**PCDH18**	1.04	2.70E-02	4.18E-02
**CTD-3074O7.5**	-0.60	2.70E-02	4.18E-02
**PDCD2**	-0.28	2.70E-02	4.18E-02
**CCL23**	1.65	2.70E-02	4.18E-02
**RP11-166O4.6**	-0.95	2.70E-02	4.18E-02
**RP11-1123I8.1**	-1.59	2.70E-02	4.18E-02
**CCDC173**	-0.91	2.70E-02	4.18E-02
**FZD6**	-0.34	2.71E-02	4.18E-02
**TDRKH**	0.52	2.71E-02	4.19E-02
**RN7SL589P**	2.07	2.71E-02	4.19E-02
**SERPINB11**	-2.97	2.71E-02	4.19E-02
**SOST**	4.72	2.71E-02	4.19E-02
**SDC4P**	-3.37	2.72E-02	4.20E-02
**RP11-69M1.6**	-1.11	2.72E-02	4.21E-02
**RPL36AP48**	-2.85	2.72E-02	4.21E-02
**SRD5A1**	-0.62	2.72E-02	4.21E-02
**LINC01057**	0.90	2.72E-02	4.21E-02
**FAM222A**	-0.54	2.72E-02	4.21E-02
**USP24P1**	-3.15	2.72E-02	4.21E-02
**RN7SKP283**	-2.93	2.72E-02	4.21E-02
**POLR2J2**	-1.13	2.72E-02	4.21E-02
**ZNF849P**	-2.66	2.73E-02	4.21E-02
**RP11-38H17.1**	3.31	2.73E-02	4.22E-02
**CD163L1**	0.96	2.73E-02	4.22E-02
**RCBTB1**	-0.35	2.73E-02	4.22E-02

**RP11-242J7.1**	-2.59	2.73E-02	4.22E-02
**AC012314.19**	-2.31	2.73E-02	4.22E-02
**RNU6-249P**	-3.17	2.73E-02	4.22E-02
**ZNF74**	0.35	2.73E-02	4.22E-02
**LRP1**	0.72	2.73E-02	4.22E-02
**AC002366.3**	-2.85	2.73E-02	4.22E-02
**CTNNA3**	-1.22	2.74E-02	4.23E-02
**C3orf79**	-2.17	2.74E-02	4.23E-02
**LINC01158**	-1.17	2.74E-02	4.23E-02
**SNUPN**	0.27	2.74E-02	4.23E-02
**RP11-378E13.4**	-3.44	2.74E-02	4.23E-02
**FAM174A**	0.42	2.74E-02	4.23E-02
**RNF8**	0.28	2.74E-02	4.24E-02
**LINGO3**	1.05	2.74E-02	4.24E-02
**HNRNPLP2**	0.98	2.75E-02	4.24E-02
**RP11-486I11.2**	-0.90	2.75E-02	4.24E-02
**RP11-291L22.9**	0.65	2.75E-02	4.24E-02
**AOX2P**	2.63	2.75E-02	4.24E-02
**G32019**	-1.94	2.75E-02	4.25E-02
**OCIAD1-AS1**	-1.48	2.75E-02	4.25E-02
**TEP1**	-0.35	2.75E-02	4.25E-02
**RP11-195F19.5**	-2.61	2.75E-02	4.25E-02
**ACACA**	-0.39	2.75E-02	4.25E-02
**G19654**	-2.06	2.75E-02	4.25E-02
**RP11-19G24.1**	-1.98	2.76E-02	4.25E-02
**RP11-521B24.4**	0.38	2.76E-02	4.26E-02
**CACNA1G**	-0.67	2.76E-02	4.26E-02
**USP36**	0.36	2.76E-02	4.26E-02
**KB-1517D11.2**	-3.17	2.76E-02	4.26E-02
**MICU2**	-0.37	2.77E-02	4.27E-02
**TPT1P4**	1.56	2.77E-02	4.27E-02
**RNU6-1262P**	-2.54	2.77E-02	4.27E-02
**RP11-513G19.1**	-1.31	2.77E-02	4.27E-02
**MMEL1**	1.33	2.77E-02	4.27E-02
**TM7SF3**	-0.32	2.77E-02	4.27E-02
**ZFP42**	2.69	2.77E-02	4.28E-02
**CTD-2363C16.1**	1.62	2.77E-02	4.28E-02
**RP11-335O4.3**	1.15	2.77E-02	4.28E-02
**SBF1**	0.32	2.78E-02	4.28E-02
**RP11-817I4.2**	1.40	2.78E-02	4.28E-02
**GNG2**	0.65	2.78E-02	4.28E-02
**FLT3LG**	1.81	2.78E-02	4.29E-02
**KIAA1107**	-0.56	2.78E-02	4.29E-02
**RP11-326L17.1**	-1.61	2.78E-02	4.29E-02
**TRAJ26**	-3.38	2.79E-02	4.30E-02

**LL0XNC01-116E7.1**	-3.36	2.79E-02	4.30E-02
**NTN4**	0.77	2.79E-02	4.30E-02
**RP11-180M15.4**	-2.59	2.79E-02	4.30E-02
**MTND4P20**	-2.09	2.79E-02	4.30E-02
**CTD-3075F15.1**	1.84	2.79E-02	4.31E-02
**PHYHD1**	-0.37	2.80E-02	4.31E-02
**RP11-1038A11.1**	-2.31	2.80E-02	4.31E-02
**CASQ2**	0.76	2.80E-02	4.31E-02
**AC012363.13**	2.60	2.80E-02	4.31E-02
**XLOC_001065**	1.59	2.80E-02	4.32E-02
**CALM1**	0.39	2.80E-02	4.32E-02
**RP4-598P13.1**	1.85	2.80E-02	4.32E-02
**RNU6-1228P**	-2.97	2.80E-02	4.32E-02
**DBIL5P**	-0.74	2.80E-02	4.32E-02
**FOXD4L1**	-1.92	2.80E-02	4.32E-02
**CD101**	-0.56	2.80E-02	4.32E-02
**RP11-439K3.1**	1.80	2.80E-02	4.32E-02
**SCARB1**	0.29	2.80E-02	4.32E-02
**G30960**	-1.24	2.81E-02	4.32E-02
**G29896**	-1.61	2.81E-02	4.32E-02
**XLOC_011384**	0.98	2.81E-02	4.33E-02
**C9orf129**	-2.85	2.81E-02	4.33E-02
**RNU6-26P**	-1.70	2.81E-02	4.33E-02
**DOK4**	-0.33	2.81E-02	4.33E-02
**XLOC_008971**	-2.53	2.81E-02	4.33E-02
**G32319**	1.87	2.81E-02	4.33E-02
**SERPINB9P1**	1.63	2.82E-02	4.34E-02
**SEL1L2**	-2.84	2.82E-02	4.34E-02
**RPS23P6**	1.49	2.82E-02	4.34E-02
**HERC5**	0.66	2.82E-02	4.34E-02
**COQ7**	0.25	2.82E-02	4.34E-02
**RP11-295M18.6**	2.07	2.82E-02	4.34E-02
**RP11-43D4.3**	-2.28	2.82E-02	4.34E-02
**KIRREL-IT1**	1.40	2.82E-02	4.35E-02
**MROH7**	-1.22	2.83E-02	4.36E-02
**BUB3**	-0.21	2.83E-02	4.36E-02
**AL008721.1**	-2.82	2.83E-02	4.36E-02
**TAX1BP1**	-0.30	2.84E-02	4.36E-02
**PYCARD**	0.43	2.84E-02	4.37E-02
**RHOT1P3**	-3.34	2.84E-02	4.37E-02
**CDHR3**	-0.68	2.84E-02	4.37E-02
**RP11-33N14.3**	-1.56	2.84E-02	4.37E-02
**RALBP1**	0.28	2.84E-02	4.37E-02
**RP1-272L16.1**	2.00	2.84E-02	4.38E-02
**G119**	-2.32	2.85E-02	4.38E-02

**KIAA1191**	0.22	2.85E-02	4.38E-02
**XLOC_002300**	2.66	2.85E-02	4.38E-02
**RP11-359E10.1**	1.00	2.85E-02	4.38E-02
**G8972**	-1.42	2.85E-02	4.38E-02
**IGHV1-2**	-3.28	2.85E-02	4.39E-02
**GSDMB**	-0.85	2.85E-02	4.39E-02
**AL591668.1**	-3.05	2.85E-02	4.39E-02
**RP11-449P15.2**	-0.96	2.86E-02	4.40E-02
**GOLGA8N**	-0.70	2.86E-02	4.40E-02
**AC091878.1**	1.59	2.86E-02	4.40E-02
**SH3GL1P1**	-0.73	2.86E-02	4.40E-02
**RIPPLY3**	1.67	2.86E-02	4.40E-02
**AFM**	-2.90	2.86E-02	4.40E-02
**RNA5SP371**	-2.87	2.86E-02	4.40E-02
**HLA-DRB6**	1.87	2.86E-02	4.40E-02
**DHH**	1.59	2.86E-02	4.40E-02
**RP4-740C4.5**	0.64	2.87E-02	4.41E-02
**NTN1**	-0.71	2.87E-02	4.41E-02
**C9orf9**	0.37	2.87E-02	4.41E-02
**FIBCD1**	2.19	2.87E-02	4.41E-02
**ULK2**	0.22	2.87E-02	4.41E-02
**DKK4**	2.13	2.87E-02	4.42E-02
**RP4-609E1.2**	-2.97	2.87E-02	4.42E-02
**AL022345.7**	-2.77	2.87E-02	4.42E-02
**JAZF1**	-0.37	2.88E-02	4.42E-02
**XG**	-0.62	2.88E-02	4.42E-02
**AL133458.1**	-1.20	2.88E-02	4.42E-02
**PLGLB1**	-1.14	2.88E-02	4.42E-02
**HSPE1P22**	-2.95	2.88E-02	4.42E-02
**RP11-395P13.7**	-3.21	2.88E-02	4.42E-02
**RP11-438N5.2**	-2.85	2.88E-02	4.43E-02
**RBFOX3**	-1.25	2.89E-02	4.44E-02
**GSTT2B**	1.97	2.89E-02	4.44E-02
**XLOC_005347**	-2.90	2.89E-02	4.45E-02
**MAST4**	-0.35	2.89E-02	4.45E-02
**XLOC_007698**	-2.24	2.90E-02	4.45E-02
**AP001601.2**	-3.10	2.90E-02	4.45E-02
**SLC22A24**	-2.96	2.90E-02	4.45E-02
**OR13D3P**	-2.86	2.90E-02	4.46E-02
**C1QL3**	-1.11	2.90E-02	4.46E-02
**AL513547.1**	-2.64	2.90E-02	4.46E-02
**ZCWPW1**	0.39	2.90E-02	4.46E-02
**XLOC_001153**	-1.54	2.91E-02	4.46E-02
**FATE1**	6.60	2.91E-02	4.47E-02
**ZNF32-AS2**	-0.99	2.91E-02	4.47E-02

**ZBED3-AS1**	-1.12	2.91E-02	4.47E-02
**RP11-275N1.1**	-3.06	2.91E-02	4.47E-02
**RP11-707M3.3**	-1.04	2.91E-02	4.47E-02
**CASQ1**	-1.31	2.92E-02	4.48E-02
**BOLA2P2**	-1.88	2.92E-02	4.48E-02
**CTD-2201E9.4**	-2.27	2.92E-02	4.48E-02
**TIPIN**	-0.40	2.92E-02	4.48E-02
**IGHE**	-4.64	2.92E-02	4.48E-02
**P4HA1**	0.59	2.92E-02	4.49E-02
**SMLR1**	-2.14	2.92E-02	4.49E-02
**EEF1A1P12**	-0.92	2.93E-02	4.50E-02
**BUB1**	-0.55	2.93E-02	4.50E-02
**DNAJC21**	-0.28	2.93E-02	4.50E-02
**LINC00483**	-2.65	2.93E-02	4.50E-02
**MIR320B2**	-2.08	2.94E-02	4.50E-02
**RP11-452G18.2**	-2.73	2.94E-02	4.51E-02
**ID2**	-0.47	2.94E-02	4.51E-02
**GAPDHP38**	-2.77	2.94E-02	4.51E-02
**KRT25**	-4.36	2.94E-02	4.51E-02
**XLOC_014276**	-2.14	2.94E-02	4.51E-02
**AP001189.4**	1.37	2.94E-02	4.51E-02
**PPIL3**	0.47	2.94E-02	4.51E-02
**SAXO2**	-1.08	2.94E-02	4.51E-02
**TMEM100**	0.87	2.94E-02	4.51E-02
**PIGR**	-2.34	2.95E-02	4.52E-02
**KLHL13**	0.73	2.95E-02	4.52E-02
**RP11-153I24.4**	-1.91	2.95E-02	4.52E-02
**AC080125.1**	-3.54	2.95E-02	4.52E-02
**ECEL1**	1.77	2.95E-02	4.52E-02
**RP11-573D15.1**	1.58	2.95E-02	4.52E-02
**DEPDC7**	0.46	2.95E-02	4.53E-02
**RP11-435O5.5**	-1.75	2.95E-02	4.53E-02
**MYOZ2**	-1.61	2.95E-02	4.53E-02
**FAM186A**	-1.65	2.95E-02	4.53E-02
**RP11-347C12.10**	0.67	2.96E-02	4.53E-02
**LINC00242**	1.14	2.96E-02	4.53E-02
**SYNJ2**	0.37	2.96E-02	4.53E-02
**RP11-568J23.6**	-2.53	2.96E-02	4.54E-02
**RP11-775D22.2**	1.55	2.96E-02	4.54E-02
**RP11-235C23.5**	-2.86	2.96E-02	4.54E-02
**RP11-49O14.2**	-1.29	2.96E-02	4.54E-02
**LLNLR-307A6.1**	-2.60	2.97E-02	4.55E-02
**DTWD1**	-0.25	2.97E-02	4.55E-02
**FAR1**	-0.28	2.97E-02	4.55E-02
**SMTNL1**	-1.15	2.97E-02	4.55E-02

**FGFBP2**	-1.03	2.98E-02	4.56E-02
**XLOC_009602**	1.72	2.98E-02	4.56E-02
**TUBAL3**	-1.13	2.98E-02	4.56E-02
**LINC01266**	-1.35	2.98E-02	4.56E-02
**RP11-466A19.1**	-1.24	2.98E-02	4.56E-02
**AC006042.7**	-1.57	2.98E-02	4.56E-02
**CYP4F11**	1.89	2.98E-02	4.57E-02
**EIF1AX**	-0.37	2.98E-02	4.57E-02
**SLC9A4**	1.15	2.98E-02	4.57E-02
**ESPNP**	-3.10	2.99E-02	4.57E-02
**RP11-703I16.1**	0.43	2.99E-02	4.58E-02
**HMMR**	-0.51	2.99E-02	4.58E-02
**RP11-1018N14.5**	1.63	2.99E-02	4.58E-02
**RP11-20E24.1**	-1.01	2.99E-02	4.58E-02
**RP11-395L14.18**	-0.57	2.99E-02	4.58E-02
**RP11-181E10.3**	2.00	3.00E-02	4.59E-02
**RP11-315O8.1**	-3.40	3.00E-02	4.59E-02
**MPV17L**	0.97	3.00E-02	4.59E-02
**SH3D21**	0.87	3.00E-02	4.59E-02
**XLOC_004673**	1.05	3.00E-02	4.60E-02
**RP11-641C17.4**	-2.74	3.00E-02	4.60E-02
**IL12A-AS1**	-2.64	3.00E-02	4.60E-02
**RP11-266A24.1**	-2.90	3.00E-02	4.60E-02
**MTTP**	1.24	3.00E-02	4.60E-02
**RP5-902P8.12**	-1.46	3.01E-02	4.60E-02
**TSC22D1-AS1**	1.03	3.01E-02	4.60E-02
**FBN2**	1.46	3.01E-02	4.61E-02
**RP3-335E1.1**	-2.77	3.01E-02	4.61E-02
**ZNF320**	0.33	3.01E-02	4.61E-02
**RP11-1112J20.2**	-1.58	3.01E-02	4.61E-02
**AJ011932.1**	2.20	3.01E-02	4.61E-02
**RGS10**	0.43	3.02E-02	4.62E-02
**RP11-360O19.5**	-2.33	3.02E-02	4.62E-02
**LNX1**	-0.49	3.02E-02	4.62E-02
**RP11-316N24.2**	-2.68	3.03E-02	4.63E-02
**CCDC58**	0.31	3.03E-02	4.64E-02
**G2582**	-0.91	3.03E-02	4.64E-02
**RP11-660M5.1**	-2.71	3.03E-02	4.64E-02
**RNU6-28P**	-3.03	3.04E-02	4.64E-02
**CTA-992D9.11**	-3.08	3.04E-02	4.64E-02
**DENND3**	0.52	3.04E-02	4.64E-02
**KDELR3**	0.69	3.04E-02	4.65E-02
**G40005**	-2.51	3.04E-02	4.65E-02
**AD001527.7**	1.91	3.04E-02	4.65E-02
**RP11-8L8.2**	2.67	3.04E-02	4.65E-02

**RP11-12L8.1**	-1.95	3.04E-02	4.65E-02
**RNU7-169P**	-3.10	3.04E-02	4.65E-02
**RNU6-470P**	-4.82	3.05E-02	4.66E-02
**RP11-122G18.7**	-1.95	3.05E-02	4.66E-02
**TAS1R1**	1.25	3.05E-02	4.66E-02
**RP11-278A23.1**	-1.07	3.05E-02	4.66E-02
**FDFT1**	0.44	3.05E-02	4.66E-02
**PXDNL**	0.82	3.05E-02	4.66E-02
**CDKL3**	-0.86	3.05E-02	4.66E-02
**RNA5SP379**	-3.05	3.05E-02	4.66E-02
**SPDYE3**	0.54	3.05E-02	4.67E-02
**LA16c-380F5.1**	-1.32	3.06E-02	4.67E-02
**RP11-108K3.1**	-2.53	3.06E-02	4.68E-02
**ANKRD34A**	0.98	3.06E-02	4.68E-02
**RPAP1**	0.30	3.07E-02	4.68E-02
**RP11-577H5.1**	-1.04	3.07E-02	4.68E-02
**AAGAB**	0.23	3.07E-02	4.68E-02
**G26815**	2.27	3.07E-02	4.69E-02
**AL162151.3**	-0.82	3.07E-02	4.70E-02
**DIRC3-AS1**	-1.51	3.08E-02	4.70E-02
**RP13-225O21.2**	-1.38	3.08E-02	4.70E-02
**RP11-798M19.6**	0.65	3.08E-02	4.70E-02
**SLC16A1-AS1**	0.65	3.08E-02	4.70E-02
**RP11-484D2.4**	-1.10	3.08E-02	4.71E-02
**AC068134.10**	-1.31	3.08E-02	4.71E-02
**SYNC**	0.50	3.09E-02	4.71E-02
**CLPX**	-0.28	3.09E-02	4.71E-02
**SH3PXD2A-AS1**	0.52	3.09E-02	4.71E-02
**TNFSF11**	-1.21	3.09E-02	4.72E-02
**FBXW4P1**	-0.82	3.09E-02	4.72E-02
**SLC46A2**	-0.60	3.09E-02	4.72E-02
**BCO2**	-0.71	3.10E-02	4.72E-02
**POLH**	-0.23	3.10E-02	4.73E-02
**UBXN8**	0.36	3.10E-02	4.73E-02
**NOP14-AS1**	0.31	3.10E-02	4.73E-02
**IGBP1**	0.30	3.11E-02	4.74E-02
**MYL4**	2.33	3.11E-02	4.74E-02
**TNFRSF17**	-2.03	3.11E-02	4.74E-02
**MIR3681HG**	1.98	3.11E-02	4.74E-02
**FAAHP1**	1.67	3.11E-02	4.74E-02
**UGDH-AS1**	-0.55	3.11E-02	4.75E-02
**RP11-13J8.1**	1.72	3.11E-02	4.75E-02
**ANKRD30B**	-3.04	3.11E-02	4.75E-02
**RP11-648L3.1**	-3.07	3.11E-02	4.75E-02
**TESK2**	0.31	3.11E-02	4.75E-02

**RP11-649E7.8**	-1.57	3.12E-02	4.75E-02
**OGFRP1**	-0.80	3.12E-02	4.75E-02
**RP11-466A19.7**	-1.67	3.12E-02	4.76E-02
**CEP170B**	0.41	3.12E-02	4.76E-02
**SEPW1**	0.31	3.12E-02	4.76E-02
**SYNRG**	-0.22	3.13E-02	4.77E-02
**AC108456.1**	-2.07	3.13E-02	4.77E-02
**G10743**	-2.84	3.13E-02	4.77E-02
**PLCXD2-AS1**	-2.74	3.13E-02	4.77E-02
**PDGFRA**	1.10	3.13E-02	4.77E-02
**RP11-18B3.3**	-2.78	3.13E-02	4.78E-02
**RP11-231P20.5**	-2.62	3.14E-02	4.78E-02
**RP3-477O4.5**	-2.66	3.14E-02	4.78E-02
**RP11-554E23.4**	-1.72	3.14E-02	4.78E-02
**G2589**	-1.46	3.14E-02	4.79E-02
**YY1P1**	-2.80	3.14E-02	4.79E-02
**CYP2D8P**	-1.06	3.14E-02	4.79E-02
**RP11-263C24.1**	-1.42	3.14E-02	4.79E-02
**AC093642.3**	-1.43	3.14E-02	4.79E-02
**CTD-2020K17.4**	1.30	3.15E-02	4.79E-02
**PPP3CB**	0.23	3.15E-02	4.80E-02
**HIGD1AP18**	-2.62	3.15E-02	4.80E-02
**NT5DC1**	-0.30	3.15E-02	4.80E-02
**RPL39P6**	-2.74	3.15E-02	4.80E-02
**RP11-644C3.1**	-2.25	3.15E-02	4.80E-02
**MXD1**	0.37	3.15E-02	4.80E-02
**RP11-946L16.2**	-2.64	3.15E-02	4.80E-02
**G34192**	1.10	3.15E-02	4.80E-02
**PFDN4**	0.39	3.16E-02	4.81E-02
**ROR1-AS1**	1.19	3.16E-02	4.81E-02
**RP11-819M15.1**	-2.33	3.16E-02	4.81E-02
**RP1-265C24.8**	-1.61	3.16E-02	4.82E-02
**G10801**	2.16	3.17E-02	4.82E-02
**RP11-686G8.1**	-2.46	3.17E-02	4.82E-02
**NOS1AP**	-0.72	3.17E-02	4.83E-02
**MIR149**	-1.41	3.17E-02	4.83E-02
**AC000068.5**	-0.90	3.17E-02	4.83E-02
**RP11-384P7.7**	-1.48	3.17E-02	4.83E-02
**PTP4A2P1**	2.02	3.18E-02	4.84E-02
**RP11-156G14.6**	-3.17	3.18E-02	4.84E-02
**RP11-424I19.2**	2.09	3.18E-02	4.84E-02
**YWHAQP6**	-1.49	3.18E-02	4.84E-02
**PITRM1**	0.23	3.18E-02	4.84E-02
**G5265**	-0.93	3.18E-02	4.84E-02
**RP11-445N18.5**	-1.88	3.18E-02	4.84E-02

**FOXJ2**	0.28	3.18E-02	4.84E-02
**CTC-435M10.12**	-2.09	3.19E-02	4.85E-02
**RNU6-981P**	-3.02	3.19E-02	4.86E-02
**PTGDR2**	-0.98	3.19E-02	4.86E-02
**DNM1P47**	-1.50	3.19E-02	4.86E-02
**COL9A2**	0.87	3.20E-02	4.87E-02
**KRTAP5-7**	-3.14	3.20E-02	4.87E-02
**TRAJ17**	-3.23	3.20E-02	4.87E-02
**IBA57-AS1**	-1.06	3.20E-02	4.87E-02
**RP11-218C14.8**	-0.96	3.20E-02	4.87E-02
**RNASE6**	0.97	3.20E-02	4.87E-02
**HAS2-AS1**	-1.06	3.20E-02	4.87E-02
**THAP1**	-0.36	3.20E-02	4.87E-02
**TRAPPC4**	0.32	3.21E-02	4.88E-02
**SNORD11B**	-2.12	3.21E-02	4.88E-02
**ZNF317**	-0.18	3.21E-02	4.88E-02
**RP11-460N20.4**	-1.36	3.21E-02	4.88E-02
**MTHFSD**	0.29	3.21E-02	4.88E-02
**SPAG5-AS1**	-1.15	3.21E-02	4.88E-02
**TEX12**	-1.61	3.21E-02	4.88E-02
**ZNF502**	-0.36	3.21E-02	4.89E-02
**RP11-1084A12.2**	-3.05	3.21E-02	4.89E-02
**RP11-33I11.2**	-1.69	3.22E-02	4.90E-02
**RP11-477N3.1**	-0.67	3.22E-02	4.90E-02
**ASS1P12**	-0.78	3.22E-02	4.90E-02
**RNU6-228P**	-3.22	3.23E-02	4.90E-02
**RP11-190A12.8**	-0.85	3.23E-02	4.90E-02
**RP11-237N19.3**	-3.08	3.23E-02	4.91E-02
**CASS4**	-0.73	3.23E-02	4.91E-02
**TOB2P1**	1.49	3.23E-02	4.91E-02
**LRCH3**	-0.23	3.23E-02	4.91E-02
**PPP1R14D**	-1.59	3.23E-02	4.91E-02
**RP11-266O8.1**	-2.66	3.24E-02	4.92E-02
**CTD-3018O17.5**	-1.07	3.24E-02	4.92E-02
**MIR3685**	-1.54	3.24E-02	4.92E-02
**MMP23B**	1.30	3.24E-02	4.92E-02
**RP11-44F14.2**	1.46	3.24E-02	4.92E-02
**RP11-666A8.9**	-2.01	3.24E-02	4.92E-02
**KPNA7**	-2.89	3.24E-02	4.92E-02
**ST13**	0.31	3.24E-02	4.92E-02
**SNX31**	1.71	3.24E-02	4.92E-02
**AC092620.3**	-2.10	3.24E-02	4.93E-02
**RP11-458J1.1**	0.47	3.25E-02	4.93E-02
**RP11-757G1.5**	-1.00	3.25E-02	4.94E-02
**CTC-391G2.1**	-2.93	3.25E-02	4.94E-02

**RP11-12A20.7**	-1.58	3.25E-02	4.94E-02
**RP11-440I14.3**	-1.91	3.25E-02	4.94E-02
**RP11-524O1.4**	-2.73	3.26E-02	4.95E-02
**RP11-139H15.5**	-1.60	3.26E-02	4.95E-02
**ORMDL2**	0.37	3.26E-02	4.95E-02
**ZNF217**	-0.20	3.26E-02	4.95E-02
**LINC00867**	2.99	3.26E-02	4.95E-02
**WDR77**	-0.30	3.26E-02	4.95E-02
**TMEM30A**	-0.35	3.26E-02	4.95E-02
**RPS6KC1**	-0.28	3.27E-02	4.96E-02
**AC092902.1**	-2.89	3.27E-02	4.96E-02
**LINC01252**	1.02	3.27E-02	4.96E-02
**HMGN1P12**	-1.76	3.27E-02	4.96E-02
**RP11-490D19.8**	-2.00	3.27E-02	4.97E-02
**CTD-2231H16.1**	1.92	3.27E-02	4.97E-02
**HOXB-AS1**	1.80	3.27E-02	4.97E-02
**ARL1**	-0.32	3.27E-02	4.97E-02
**XLOC_014066**	2.08	3.28E-02	4.98E-02
**UBTD2**	0.33	3.28E-02	4.98E-02
**AC010967.3**	-3.26	3.28E-02	4.98E-02
**TGFBI**	0.69	3.28E-02	4.98E-02
**TRGV2**	-2.71	3.28E-02	4.98E-02
**RP11-659P15.1**	-2.59	3.28E-02	4.98E-02
**RP11-120D5.1**	-0.86	3.29E-02	4.99E-02
**LRRN4CL**	-0.66	3.29E-02	4.99E-02
**CDKN3**	0.64	3.29E-02	4.99E-02
**GTPBP4**	0.32	3.29E-02	4.99E-02
**RP11-390M11.1**	-3.18	3.29E-02	4.99E-02
**ELMOD3**	-0.46	3.30E-02	5.00E-02
**SORD2P**	-1.13	3.30E-02	5.00E-02
**RP11-482G13.1**	-1.56	3.30E-02	5.00E-02
**ZNF736P9Y**	-1.20	3.30E-02	5.01E-02
**MCM4**	-0.31	3.30E-02	5.01E-02
**THPO**	1.22	3.31E-02	5.01E-02
**TIMM8AP1**	1.72	3.31E-02	5.02E-02
**NOA1**	0.22	3.31E-02	5.02E-02
**LLNLR-470E3.1**	2.69	3.31E-02	5.02E-02
**OSMR-AS1**	0.66	3.31E-02	5.02E-02
**MIR4731**	-2.71	3.31E-02	5.02E-02
**ACOX3**	0.33	3.32E-02	5.03E-02
**YBX1P4**	-1.82	3.32E-02	5.03E-02
**RP1-266L20.9**	-0.84	3.32E-02	5.03E-02
**RP11-1020A11.2**	-0.44	3.32E-02	5.03E-02
**FAM96A**	0.35	3.32E-02	5.04E-02
**RNU6-404P**	-2.55	3.33E-02	5.04E-02

**RP1-257C22.2**	-0.78	3.33E-02	5.04E-02
**RP13-13A3.1**	-3.34	3.33E-02	5.04E-02
**CYP21A2**	1.57	3.33E-02	5.04E-02
**RP11-1396O13.2**	-2.28	3.33E-02	5.05E-02
**STX18-AS1**	-0.59	3.33E-02	5.05E-02
**RP5-1042K10.13**	-1.67	3.34E-02	5.05E-02
**NOXRED1**	-0.60	3.34E-02	5.06E-02
**PCMTD2**	-0.30	3.34E-02	5.06E-02
**EGR2**	-0.58	3.34E-02	5.06E-02
**RP11-12D24.6**	-1.65	3.34E-02	5.06E-02
**RP11-680F20.10**	-1.97	3.34E-02	5.06E-02
**CTD-2290C23.1**	-3.06	3.34E-02	5.06E-02
**RNU6-911P**	-2.62	3.34E-02	5.06E-02
**RP11-310I24.1**	-2.98	3.34E-02	5.07E-02
**GALNT4**	0.85	3.35E-02	5.07E-02
**AC073130.3**	-1.35	3.35E-02	5.07E-02
**C11orf16**	-1.41	3.35E-02	5.08E-02
**MOCOS**	-0.47	3.36E-02	5.08E-02
**PCDHGC5**	-0.96	3.36E-02	5.08E-02
**KIF9**	-0.33	3.36E-02	5.08E-02
**CIRBP-AS1**	-0.87	3.36E-02	5.08E-02
**PACRG**	-0.69	3.36E-02	5.08E-02
**EIF2B5-IT1**	-2.32	3.36E-02	5.08E-02
**AIRE**	-0.97	3.36E-02	5.08E-02
**TMEM92-AS1**	2.46	3.36E-02	5.09E-02
**MIR205HG**	0.57	3.37E-02	5.10E-02
**DLG3-AS1**	-1.97	3.37E-02	5.10E-02
**PIH1D2**	-0.59	3.37E-02	5.10E-02
**PCDH10**	-1.20	3.38E-02	5.11E-02
**G10989**	-2.81	3.38E-02	5.11E-02
**RP11-119K6.6**	-1.97	3.38E-02	5.11E-02
**RN7SL767P**	-1.69	3.38E-02	5.11E-02
**CERS3-AS1**	-0.52	3.38E-02	5.11E-02
**RP11-463O9.1**	-1.94	3.38E-02	5.12E-02
**MAFIP**	1.58	3.38E-02	5.12E-02
**RP11-196G18.3**	1.31	3.38E-02	5.12E-02
**RP5-1136A10.1**	-3.00	3.38E-02	5.12E-02
**ALDH1L2**	0.64	3.39E-02	5.12E-02
**CBLN4**	-2.63	3.39E-02	5.12E-02
**DYNLRB2**	-0.64	3.39E-02	5.12E-02
**G13084**	-1.15	3.39E-02	5.13E-02
**AC135050.5**	-2.03	3.39E-02	5.13E-02
**AEN**	0.59	3.39E-02	5.13E-02
**RP11-541P9.3**	-2.53	3.39E-02	5.13E-02
**ITIH1**	2.32	3.40E-02	5.14E-02

**RP11-705O24.2**	-3.23	3.40E-02	5.14E-02
**RAB11A**	-0.34	3.40E-02	5.14E-02
**HSPB3**	1.00	3.41E-02	5.15E-02
**RP11-449J21.3**	-1.06	3.41E-02	5.15E-02
**GNGT2**	1.08	3.41E-02	5.15E-02
**ABCC3**	-0.60	3.41E-02	5.15E-02
**FLJ21408**	-1.23	3.41E-02	5.15E-02
**SHH**	-2.21	3.41E-02	5.15E-02
**HTR2B**	-0.98	3.41E-02	5.15E-02
**LA16c-329F2.2**	-1.62	3.41E-02	5.16E-02
**ZEB1-AS1**	0.60	3.42E-02	5.16E-02
**DEGS2**	0.44	3.42E-02	5.16E-02
**ARL17B**	-1.10	3.42E-02	5.16E-02
**RP11-90P16.1**	-3.03	3.42E-02	5.17E-02
**XLOC_010436**	-3.51	3.42E-02	5.17E-02
**NKILA**	1.29	3.43E-02	5.18E-02
**CDHR4**	-1.18	3.43E-02	5.18E-02
**NUDT17**	0.56	3.43E-02	5.19E-02
**ACAP1**	0.56	3.44E-02	5.19E-02
**AC009495.2**	-1.47	3.44E-02	5.19E-02
**ARHGAP23P1**	2.18	3.44E-02	5.19E-02
**GPR135**	-0.89	3.44E-02	5.19E-02
**AC005785.2**	-1.09	3.44E-02	5.19E-02
**XLOC_001804**	-1.65	3.44E-02	5.19E-02
**NKAIN2**	-1.15	3.44E-02	5.20E-02
**MIR2355**	-3.08	3.45E-02	5.20E-02
**SCAMP1**	-0.34	3.45E-02	5.20E-02
**PIFO**	-0.65	3.45E-02	5.20E-02
**MIR4639**	-2.97	3.45E-02	5.21E-02
**RN7SL867P**	-1.54	3.45E-02	5.21E-02
**MPC2**	-0.45	3.45E-02	5.21E-02
**TRAJ37**	-3.01	3.45E-02	5.21E-02
**RP11-114H23.2**	-2.85	3.45E-02	5.21E-02
**TRAF3IP2-AS1**	-0.56	3.46E-02	5.22E-02
**GNB3**	1.29	3.46E-02	5.22E-02
**IGFN1**	2.06	3.46E-02	5.22E-02
**RNU2-37P**	-2.94	3.46E-02	5.22E-02
**RP5-855D21.3**	-0.97	3.46E-02	5.22E-02
**CHIT1**	-1.45	3.46E-02	5.23E-02
**RP11-451O13.1**	-3.72	3.46E-02	5.23E-02
**RP3-340B19.3**	-2.50	3.46E-02	5.23E-02
**TTC28**	0.55	3.47E-02	5.23E-02
**SHANK2**	1.22	3.47E-02	5.23E-02
**AC097461.4**	-1.89	3.47E-02	5.23E-02
**RP11-568J23.8**	-2.08	3.47E-02	5.24E-02

**RP5-994D16.3**	-0.92	3.47E-02	5.24E-02
**RP11-34F20.7**	-1.41	3.47E-02	5.24E-02
**G1730**	1.34	3.48E-02	5.25E-02
**RN7SL306P**	-2.63	3.48E-02	5.25E-02
**UPK1B**	0.92	3.48E-02	5.25E-02
**AC026188.1**	-2.02	3.48E-02	5.25E-02
**EXOC3L4**	-1.00	3.48E-02	5.25E-02
**SPATA6L**	-0.74	3.48E-02	5.26E-02
**RN7SL569P**	-2.68	3.48E-02	5.26E-02
**TREML1**	1.42	3.49E-02	5.26E-02
**RP11-1379J22.5**	1.22	3.49E-02	5.26E-02
**FBXO9**	0.27	3.49E-02	5.26E-02
**ITIH5**	0.75	3.49E-02	5.26E-02
**B3GAT1**	0.71	3.49E-02	5.27E-02
**CTD-3010D24.3**	-2.08	3.50E-02	5.27E-02
**MAML1**	0.28	3.50E-02	5.27E-02
**RNU2-27P**	-0.80	3.50E-02	5.28E-02
**KB-1507C5.3**	-1.30	3.50E-02	5.28E-02
**MINOS1P2**	-2.79	3.50E-02	5.28E-02
**RN7SL233P**	2.01	3.51E-02	5.29E-02
**ILDR1**	-0.75	3.51E-02	5.29E-02
**ADGRG7**	-2.98	3.51E-02	5.29E-02
**PDIA3P1**	-0.40	3.51E-02	5.29E-02
**SLITRK2**	-0.62	3.51E-02	5.30E-02
**RP11-580I16.2**	-1.25	3.52E-02	5.30E-02
**PGM5-AS1**	1.44	3.52E-02	5.30E-02
**SLC26A10**	1.95	3.52E-02	5.30E-02
**VWA5A**	0.27	3.52E-02	5.30E-02
**RP11-90C4.1**	-1.08	3.52E-02	5.31E-02
**ACTR3**	-0.34	3.52E-02	5.31E-02
**C1orf198**	0.28	3.52E-02	5.31E-02
**SRRM2-AS1**	-0.71	3.52E-02	5.31E-02
**LINC00693**	1.43	3.52E-02	5.31E-02
**RP11-545A16.1**	-3.24	3.53E-02	5.32E-02
**RNU6-1112P**	-2.94	3.53E-02	5.32E-02
**RP11-175B9.3**	-0.66	3.53E-02	5.32E-02
**SLC18A3**	10.62	3.53E-02	5.32E-02
**HMGA1P2**	-1.46	3.53E-02	5.32E-02
**WRB**	0.29	3.53E-02	5.32E-02
**RNU6-196P**	-2.74	3.53E-02	5.32E-02
**RP11-1055B8.3**	-0.83	3.53E-02	5.32E-02
**ARFIP1**	-0.34	3.53E-02	5.32E-02
**ABHD15**	0.30	3.54E-02	5.33E-02
**RP11-180M15.6**	-1.21	3.54E-02	5.33E-02
**HORMAD2-AS1**	-1.95	3.54E-02	5.33E-02

**RPS17P13**	-3.11	3.54E-02	5.33E-02
**RN7SKP285**	-3.05	3.54E-02	5.33E-02
**MBD2**	0.25	3.54E-02	5.33E-02
**SFTPA1**	1.74	3.54E-02	5.33E-02
**PARP14**	-0.45	3.54E-02	5.33E-02
**AC007750.5**	2.05	3.55E-02	5.34E-02
**CCNJ**	-0.28	3.55E-02	5.34E-02
**RP11-73B2.6**	-1.00	3.55E-02	5.34E-02
**TLL1**	-0.69	3.55E-02	5.34E-02
**ARMCX4**	-0.39	3.55E-02	5.35E-02
**PLA2G4C**	0.72	3.55E-02	5.35E-02
**PLPP5**	0.28	3.55E-02	5.35E-02
**GCN1**	-0.20	3.56E-02	5.35E-02
**ZNF773**	-0.29	3.56E-02	5.35E-02
**SERPINB12**	-0.56	3.56E-02	5.35E-02
**CTD-3032H12.2**	-0.90	3.56E-02	5.36E-02
**PRPH**	1.45	3.56E-02	5.36E-02
**MAPK14**	0.25	3.56E-02	5.36E-02
**CTB-193M12.5**	0.37	3.57E-02	5.37E-02
**CTA-14H9.5**	-0.60	3.57E-02	5.37E-02
**RP11-815I9.4**	0.41	3.57E-02	5.38E-02
**ANKRD10-IT1**	-0.57	3.57E-02	5.38E-02
**RP11-589P10.5**	0.64	3.57E-02	5.38E-02
**RP11-404O13.4**	-3.01	3.57E-02	5.38E-02
**TBCAP2**	-3.29	3.57E-02	5.38E-02
**AC002044.3**	-1.45	3.58E-02	5.38E-02
**SNORD38**	-1.28	3.58E-02	5.38E-02
**RP11-758N13.1**	2.03	3.58E-02	5.38E-02
**CES3**	0.70	3.58E-02	5.38E-02
**RN7SKP26**	-2.82	3.58E-02	5.39E-02
**KLHL25**	0.32	3.58E-02	5.39E-02
**ANO9**	-0.65	3.58E-02	5.39E-02
**B3GNT3**	1.13	3.59E-02	5.39E-02
**ATP12A**	1.52	3.59E-02	5.39E-02
**HAUS6P3**	-2.91	3.59E-02	5.39E-02
**MLEC**	-0.27	3.59E-02	5.40E-02
**PADI2**	1.42	3.59E-02	5.40E-02
**S100G**	-2.60	3.59E-02	5.40E-02
**MIR599**	-3.12	3.59E-02	5.40E-02
**XLOC_009944**	2.05	3.60E-02	5.41E-02
**BTN2A3P**	0.49	3.60E-02	5.41E-02
**RP11-326C3.2**	1.78	3.61E-02	5.42E-02
**LINC01494**	-2.74	3.61E-02	5.42E-02
**PAIP1**	0.29	3.61E-02	5.42E-02
**HAR1A**	1.57	3.61E-02	5.42E-02

**SLC26A7**	-1.42	3.61E-02	5.43E-02
**ZFAND6**	0.33	3.61E-02	5.43E-02
**RP11-31H5.3**	-0.77	3.62E-02	5.43E-02
**CTNNBIP1**	0.39	3.62E-02	5.43E-02
**RPS3AP32**	-2.94	3.62E-02	5.43E-02
**RRP12**	0.41	3.62E-02	5.43E-02
**NDUFA7**	-0.86	3.62E-02	5.43E-02
**AC000078.5**	-1.55	3.62E-02	5.44E-02
**ATP4A**	-1.51	3.62E-02	5.44E-02
**RP11-91P24.7**	-1.35	3.62E-02	5.44E-02
**PHTF2**	-0.25	3.62E-02	5.44E-02
**HTR3A**	1.38	3.62E-02	5.44E-02
**ZNF300P1**	-0.77	3.62E-02	5.44E-02
**RP11-117L5.1**	-1.30	3.62E-02	5.44E-02
**RNA5SP155**	-2.80	3.63E-02	5.45E-02
**FFAR2**	0.58	3.63E-02	5.45E-02
**KLHDC2**	0.21	3.63E-02	5.45E-02
**AC006378.2**	-0.74	3.63E-02	5.45E-02
**RP11-77M5.1**	-2.53	3.63E-02	5.45E-02
**ERI2**	-0.33	3.63E-02	5.46E-02
**FSTL5**	2.76	3.63E-02	5.46E-02
**DUOX1**	0.46	3.64E-02	5.46E-02
**TPO**	1.21	3.64E-02	5.46E-02
**KLRB1**	0.89	3.64E-02	5.46E-02
**RNU6-1283P**	-2.91	3.64E-02	5.47E-02
**FASN**	-1.05	3.65E-02	5.47E-02
**RN7SL302P**	-3.28	3.65E-02	5.47E-02
**RP11-333O1.1**	1.55	3.65E-02	5.47E-02
**GSKIP**	-0.39	3.65E-02	5.47E-02
**EIF4EBP3**	-1.24	3.65E-02	5.48E-02
**AC104451.2**	-2.37	3.65E-02	5.48E-02
**XLOC_010952**	-2.17	3.65E-02	5.48E-02
**LGR6**	0.47	3.66E-02	5.49E-02
**XLOC_007971**	-2.44	3.66E-02	5.49E-02
**RP11-385D13.3**	0.87	3.66E-02	5.49E-02
**LA16c-380A1.1**	1.41	3.66E-02	5.50E-02
**RNU4-49P**	-2.87	3.67E-02	5.50E-02
**RP11-356J5.12**	0.76	3.67E-02	5.50E-02
**RP11-344N10.2**	-2.49	3.67E-02	5.51E-02
**C2orf40**	0.97	3.67E-02	5.51E-02
**RNU6-724P**	-3.05	3.67E-02	5.51E-02
**RP3-395M20.3**	-1.21	3.67E-02	5.51E-02
**LINC00702**	0.65	3.67E-02	5.51E-02
**SLC4A4**	-0.82	3.68E-02	5.52E-02
**KAT2A**	0.50	3.68E-02	5.52E-02

**RP11-57B24.2**	-2.75	3.68E-02	5.52E-02
**CTC-453G23.5**	-1.49	3.68E-02	5.52E-02
**AKAP10**	-0.14	3.68E-02	5.52E-02
**RP11-70L8.5**	-1.07	3.68E-02	5.52E-02
**RP11-404G16.2**	-2.69	3.68E-02	5.52E-02
**AC004156.3**	1.30	3.68E-02	5.52E-02
**AC063980.3**	3.91	3.68E-02	5.52E-02
**RFX3-AS1**	-0.83	3.69E-02	5.53E-02
**RP11-632C17__A.1**	-0.66	3.69E-02	5.54E-02
**EIF1AXP2**	-2.75	3.70E-02	5.54E-02
**RP11-30O15.1**	-3.49	3.70E-02	5.55E-02
**KRTAP26-1**	-5.29	3.70E-02	5.55E-02
**GAPDHP70**	-2.96	3.70E-02	5.55E-02
**CTC-241N9.1**	0.56	3.71E-02	5.55E-02
**RP1-95L4.2**	-2.22	3.71E-02	5.56E-02
**RP11-399E6.4**	-1.80	3.71E-02	5.56E-02
**MANSC4**	-1.82	3.71E-02	5.56E-02
**MTCO1P28**	-2.09	3.71E-02	5.56E-02
**SND1-IT1**	-1.20	3.71E-02	5.56E-02
**XLOC_004054**	2.66	3.71E-02	5.56E-02
**MIR30A**	-2.93	3.72E-02	5.57E-02
**RP3-508I15.22**	-1.62	3.72E-02	5.57E-02
**ANKRD36B**	-0.79	3.72E-02	5.57E-02
**HOXA10-AS**	1.89	3.72E-02	5.57E-02
**HSF5**	-2.09	3.73E-02	5.58E-02
**RP11-485M7.1**	-1.33	3.73E-02	5.59E-02
**RP11-662M24.1**	-2.91	3.73E-02	5.59E-02
**HSP90AA5P**	-3.08	3.73E-02	5.59E-02
**RPS24P8**	-1.67	3.73E-02	5.59E-02
**KIAA1324**	1.44	3.74E-02	5.60E-02
**RP11-104L21.3**	-1.05	3.74E-02	5.60E-02
**SLC39A14**	0.58	3.74E-02	5.60E-02
**RP1-69D17.4**	-1.38	3.74E-02	5.60E-02
**RNU1-60P**	-1.35	3.74E-02	5.60E-02
**PPM1J**	-0.61	3.75E-02	5.61E-02
**TM9SF1**	0.39	3.75E-02	5.62E-02
**CTD-2001J20.1**	-2.77	3.75E-02	5.62E-02
**CTC-366B18.4**	-0.55	3.76E-02	5.62E-02
**OR2H2**	-2.78	3.76E-02	5.62E-02
**IL1RL2**	-0.40	3.76E-02	5.62E-02
**CNNM2**	-0.28	3.76E-02	5.63E-02
**XPOT**	-0.28	3.76E-02	5.63E-02
**RABGEF1**	0.36	3.76E-02	5.63E-02
**AC004012.1**	-2.64	3.76E-02	5.63E-02
**POU4F1**	1.48	3.76E-02	5.63E-02

**UCN**	1.43	3.76E-02	5.63E-02
**CTD-2525I3.5**	-1.07	3.76E-02	5.63E-02
**ALG1L**	1.04	3.76E-02	5.63E-02
**G5482**	1.59	3.77E-02	5.63E-02
**GPRC5A**	0.96	3.78E-02	5.65E-02
**TMEM220-AS1**	0.76	3.78E-02	5.65E-02
**RP11-146G7.2**	-2.80	3.78E-02	5.65E-02
**RP11-789C17.3**	-2.68	3.78E-02	5.66E-02
**CTSS**	0.74	3.78E-02	5.66E-02
**G23688**	-2.79	3.79E-02	5.66E-02
**RP4-604A21.1**	-3.11	3.79E-02	5.67E-02
**RP11-562A8.1**	-1.19	3.79E-02	5.67E-02
**RP3-461P17.10**	-2.09	3.79E-02	5.67E-02
**LRIG1**	-0.33	3.79E-02	5.67E-02
**AC006195.2**	-2.22	3.80E-02	5.68E-02
**AC143336.1**	-3.01	3.80E-02	5.68E-02
**NOP16**	0.34	3.80E-02	5.68E-02
**TFB1M**	-0.28	3.80E-02	5.68E-02
**AC138744.2**	1.65	3.80E-02	5.69E-02
**RPL31**	0.34	3.81E-02	5.69E-02
**RP11-243M5.5**	-3.12	3.81E-02	5.69E-02
**AC011524.1**	-1.18	3.81E-02	5.69E-02
**RP11-887P2.6**	-1.60	3.81E-02	5.69E-02
**TUBBP9**	-3.07	3.81E-02	5.69E-02
**UBR7**	-0.24	3.81E-02	5.69E-02
**LINC00898**	1.09	3.81E-02	5.70E-02
**MFI2-AS1**	1.18	3.81E-02	5.70E-02
**SERBP1**	0.34	3.82E-02	5.70E-02
**LHFPL3-AS2**	-2.82	3.82E-02	5.70E-02
**KCTD4**	-0.97	3.82E-02	5.70E-02
**RP11-114G22.1**	-1.98	3.82E-02	5.70E-02
**BTBD19**	0.83	3.82E-02	5.71E-02
**RP11-570G20.1**	-2.58	3.82E-02	5.71E-02
**RP11-173M1.5**	-2.86	3.82E-02	5.71E-02
**H19**	1.30	3.83E-02	5.71E-02
**RP11-192P3.4**	1.71	3.83E-02	5.72E-02
**RP11-175P13.3**	-1.09	3.83E-02	5.72E-02
**RP11-292F9.1**	-3.03	3.83E-02	5.72E-02
**SRSF5**	0.53	3.83E-02	5.72E-02
**CREBL2**	-0.31	3.83E-02	5.72E-02
**PNMA5**	-2.16	3.83E-02	5.73E-02
**RPTOR**	-0.30	3.84E-02	5.73E-02
**UXT-AS1**	-1.71	3.84E-02	5.73E-02
**RP11-274A11.5**	-1.90	3.84E-02	5.73E-02
**CECR9**	1.71	3.84E-02	5.73E-02

**CHAMP1**	0.31	3.84E-02	5.73E-02
**AC006548.28**	-1.77	3.84E-02	5.74E-02
**AC124944.5**	-2.68	3.85E-02	5.74E-02
**RP11-319G9.3**	-1.05	3.85E-02	5.75E-02
**XLOC_002561**	0.80	3.85E-02	5.75E-02
**AC019118.4**	-2.41	3.85E-02	5.75E-02
**RP11-114N19.3**	-1.14	3.85E-02	5.75E-02
**LLNLR-245B6.1**	-0.51	3.85E-02	5.75E-02
**ADAM9**	0.34	3.85E-02	5.75E-02
**SCGB2A1**	1.97	3.86E-02	5.76E-02
**RP11-15F12.1**	-1.05	3.87E-02	5.77E-02
**SFXN1**	0.22	3.87E-02	5.77E-02
**HOXD11**	1.32	3.87E-02	5.77E-02
**ZNF192P1**	-1.04	3.87E-02	5.77E-02
**BSPRY**	0.44	3.87E-02	5.77E-02
**XLOC_002897**	-2.53	3.87E-02	5.77E-02
**G18534**	-0.78	3.87E-02	5.78E-02
**ICA1**	0.51	3.88E-02	5.78E-02
**RP11-346J10.2**	-1.81	3.88E-02	5.78E-02
**MTDH**	-0.25	3.88E-02	5.79E-02
**FAM66A**	-1.17	3.88E-02	5.79E-02
**KRTAP4-3**	-5.49	3.88E-02	5.79E-02
**LGALS2**	-0.65	3.88E-02	5.79E-02
**CTD-2561J22.5**	-1.11	3.88E-02	5.79E-02
**CCDC166**	-3.20	3.89E-02	5.79E-02
**YWHAG**	0.25	3.89E-02	5.80E-02
**NDUFB3**	0.36	3.89E-02	5.80E-02
**RN7SL529P**	-1.36	3.89E-02	5.81E-02
**ADARB1**	0.39	3.90E-02	5.81E-02
**CTD-2515H24.1**	-2.87	3.90E-02	5.82E-02
**IL1RAP**	-0.32	3.90E-02	5.82E-02
**NMNAT1P1**	-2.90	3.91E-02	5.82E-02
**ADCY9**	0.32	3.91E-02	5.83E-02
**DRP2**	-1.14	3.91E-02	5.83E-02
**NOX1**	-0.60	3.91E-02	5.83E-02
**G8228**	-1.60	3.91E-02	5.83E-02
**ENGASE**	0.62	3.91E-02	5.83E-02
**AP000240.9**	-0.73	3.91E-02	5.83E-02
**TSSC1-IT1**	-1.76	3.91E-02	5.83E-02
**NSF**	-0.23	3.92E-02	5.84E-02
**RNU6-647P**	-3.19	3.92E-02	5.84E-02
**NOX5**	0.75	3.92E-02	5.84E-02
**CTD-2319I12.2**	1.11	3.92E-02	5.85E-02
**G35308**	-2.54	3.92E-02	5.85E-02
**UFSP1**	0.56	3.92E-02	5.85E-02

**RP11-564D11.3**	-1.05	3.93E-02	5.85E-02
**G8321**	-1.92	3.93E-02	5.85E-02
**TMEM87B**	-0.23	3.93E-02	5.85E-02
**CSF1R**	0.73	3.93E-02	5.85E-02
**RP11-165D7.5**	0.93	3.93E-02	5.85E-02
**RP11-764D10.2**	-1.10	3.93E-02	5.86E-02
**RPL35AP32**	-2.22	3.93E-02	5.86E-02
**RP11-894P9.1**	-0.77	3.93E-02	5.86E-02
**AC004967.7**	0.50	3.94E-02	5.86E-02
**LINC01293**	-2.34	3.94E-02	5.86E-02
**NWD1**	1.40	3.94E-02	5.86E-02
**RP11-65M17.3**	-2.82	3.94E-02	5.86E-02
**PACRG-AS1**	-2.64	3.94E-02	5.87E-02
**RNU6-51P**	2.11	3.94E-02	5.87E-02
**RP5-937E21.8**	-1.74	3.94E-02	5.87E-02
**RP11-1415C14.4**	-2.89	3.94E-02	5.87E-02
**RP13-392I16.1**	-3.98	3.95E-02	5.87E-02
**HRH4**	-1.62	3.95E-02	5.88E-02
**TRPV4**	0.39	3.95E-02	5.88E-02
**RAD17P2**	-1.55	3.95E-02	5.88E-02
**ETF1**	-0.27	3.95E-02	5.88E-02
**APC2**	0.64	3.95E-02	5.88E-02
**RP11-20I23.13**	-2.04	3.96E-02	5.89E-02
**IL20RB**	-0.45	3.96E-02	5.89E-02
**AC003002.6**	-1.82	3.97E-02	5.91E-02
**RP11-650L12.1**	-1.15	3.97E-02	5.91E-02
**RAP1B**	0.25	3.97E-02	5.91E-02
**PHACTR1**	-0.38	3.98E-02	5.92E-02
**RP11-215C7.3**	-2.78	3.98E-02	5.92E-02
**MIR5188**	-2.07	3.98E-02	5.92E-02
**CTD-2553L13.10**	0.56	3.98E-02	5.92E-02
**CDC5L**	-0.26	3.98E-02	5.92E-02
**CBLB**	0.46	3.99E-02	5.93E-02
**LEPROTL1**	0.28	3.99E-02	5.93E-02
**RP11-178C3.2**	-2.60	3.99E-02	5.93E-02
**RBM15B**	0.23	3.99E-02	5.94E-02
**CITF22-49E9.3**	1.30	3.99E-02	5.94E-02
**RPS7P3**	-2.08	3.99E-02	5.94E-02
**LYPLA1P3**	-2.02	3.99E-02	5.94E-02
**RP11-544M22.3**	-2.05	4.00E-02	5.94E-02
**MKI67P1**	-3.45	4.00E-02	5.94E-02
**CENPP**	-0.35	4.00E-02	5.94E-02
**PRDM15**	-0.29	4.00E-02	5.95E-02
**AC106753.1**	2.15	4.00E-02	5.95E-02
**SIGLEC1**	1.02	4.01E-02	5.96E-02

**RP4-791C19.1**	-2.61	4.01E-02	5.96E-02
**DSCAM**	-3.07	4.01E-02	5.96E-02
**XLOC_002674**	1.80	4.01E-02	5.96E-02
**KLHL14**	1.18	4.01E-02	5.97E-02
**TMEM198B**	0.43	4.02E-02	5.97E-02
**HMGB1P10**	0.71	4.02E-02	5.97E-02
**HLTF**	-0.31	4.02E-02	5.97E-02
**CTB-1144G6.6**	-1.25	4.03E-02	5.99E-02
**AL353791.1**	-1.12	4.03E-02	5.99E-02
**AC074212.5**	1.64	4.03E-02	6.00E-02
**PHOX2A**	-2.79	4.04E-02	6.00E-02
**FUT7**	1.22	4.04E-02	6.00E-02
**AURKC**	0.85	4.04E-02	6.00E-02
**AC016910.1**	-1.60	4.04E-02	6.00E-02
**RP4-604K5.3**	-1.09	4.04E-02	6.01E-02
**RP11-1134I14.4**	1.65	4.05E-02	6.01E-02
**EEF1B2**	0.34	4.05E-02	6.01E-02
**PPIL2**	0.28	4.05E-02	6.01E-02
**RP11-498D10.3**	-2.05	4.05E-02	6.02E-02
**CCNB1**	0.46	4.05E-02	6.02E-02
**RP11-716H6.2**	-2.87	4.05E-02	6.02E-02
**CTD-3224K15.3**	-2.44	4.05E-02	6.02E-02
**RP11-167N4.4**	-2.37	4.06E-02	6.03E-02
**CYP51A1P3**	-0.95	4.06E-02	6.03E-02
**RP11-452H21.1**	-2.47	4.06E-02	6.03E-02
**RP11-304L19.1**	-1.44	4.07E-02	6.04E-02
**AC008991.1**	-2.10	4.07E-02	6.04E-02
**H2AFV**	-0.23	4.07E-02	6.04E-02
**ATP8B2**	0.64	4.07E-02	6.05E-02
**DNER**	1.49	4.07E-02	6.05E-02
**RP13-349O20.2**	1.72	4.07E-02	6.05E-02
**SNORD104**	-1.49	4.07E-02	6.05E-02
**ABCC5**	0.48	4.08E-02	6.05E-02
**ELL3**	-0.74	4.08E-02	6.05E-02
**OFD1**	-0.23	4.08E-02	6.05E-02
**RP11-264B14.1**	-2.71	4.08E-02	6.05E-02
**RP11-867G23.10**	2.31	4.08E-02	6.05E-02
**RP3-449M8.6**	-1.50	4.08E-02	6.05E-02
**GLYATL2**	1.73	4.08E-02	6.06E-02
**TECRP1**	-0.70	4.08E-02	6.06E-02
**RP11-173A6.2**	-1.41	4.09E-02	6.06E-02
**PACERR**	-2.15	4.09E-02	6.06E-02
**LINC01030**	-2.92	4.09E-02	6.06E-02
**ARRDC5**	1.89	4.09E-02	6.06E-02
**RP3-521E19.2**	-2.40	4.09E-02	6.07E-02

**UFM1**	-0.28	4.09E-02	6.07E-02
**SYNDIG1L**	1.62	4.09E-02	6.07E-02
**RP11-402L5.2**	-2.80	4.10E-02	6.08E-02
**ECI2**	-0.38	4.10E-02	6.08E-02
**LRRC14B**	-2.78	4.10E-02	6.08E-02
**G38775**	0.80	4.10E-02	6.08E-02
**RP11-404P21.9**	-1.18	4.10E-02	6.08E-02
**RP11-120C12.3**	2.14	4.10E-02	6.09E-02
**RP3-395M20.12**	0.61	4.10E-02	6.09E-02
**RP11-299L17.1**	-3.06	4.11E-02	6.09E-02
**NAV2-AS3**	-2.46	4.11E-02	6.09E-02
**RP11-85A1.3**	1.00	4.11E-02	6.09E-02
**AC078899.1**	-1.90	4.11E-02	6.09E-02
**RP11-621K7.1**	-2.89	4.11E-02	6.09E-02
**AC007009.2**	-2.94	4.11E-02	6.09E-02
**AC023590.1**	-1.74	4.11E-02	6.09E-02
**LINC00479**	-2.55	4.11E-02	6.10E-02
**RP11-667M19.1**	-1.77	4.11E-02	6.10E-02
**VN1R108P**	-1.18	4.12E-02	6.10E-02
**LSAMP-AS1**	-1.70	4.12E-02	6.10E-02
**MUC12**	-1.67	4.12E-02	6.11E-02
**NIPAL4**	0.33	4.12E-02	6.11E-02
**OR10V2P**	-2.57	4.12E-02	6.11E-02
**KLRC4**	-3.03	4.12E-02	6.11E-02
**MTHFR**	0.25	4.12E-02	6.11E-02
**SLFN5**	0.51	4.13E-02	6.12E-02
**FBXO40**	-1.54	4.13E-02	6.12E-02
**CTA-360L10.1**	-2.03	4.13E-02	6.13E-02
**KRTAP10-4**	-4.20	4.14E-02	6.13E-02
**MCM8-AS1**	-1.85	4.14E-02	6.14E-02
**SVILP1**	-0.96	4.14E-02	6.14E-02
**RP11-775C24.4**	-2.71	4.15E-02	6.15E-02
**RP11-425D10.10**	1.07	4.15E-02	6.15E-02
**RP11-561N12.1**	-3.13	4.15E-02	6.15E-02
**RP11-230F18.6**	-1.05	4.16E-02	6.16E-02
**ENKUR**	1.54	4.16E-02	6.16E-02
**KRTAP16-1**	-4.55	4.16E-02	6.16E-02
**SH3RF1**	0.31	4.16E-02	6.17E-02
**PRDM8**	0.97	4.16E-02	6.17E-02
**FTH1P3**	-2.10	4.17E-02	6.18E-02
**RP11-340A13.2**	-3.16	4.17E-02	6.18E-02
**BTBD10P2**	-0.93	4.17E-02	6.18E-02
**RP11-778D9.13**	-1.67	4.17E-02	6.18E-02
**KCNMB2-AS1**	-1.92	4.18E-02	6.18E-02
**DHCR24**	-0.45	4.18E-02	6.19E-02

**RP11-509J21.4**	-2.21	4.18E-02	6.19E-02
**RP11-486A14.1**	-1.82	4.18E-02	6.19E-02
**ART1**	-2.82	4.18E-02	6.19E-02
**RP5-902P8.10**	-2.01	4.18E-02	6.19E-02
**KRT24**	6.17	4.18E-02	6.19E-02
**MFSD4**	0.80	4.18E-02	6.19E-02
**XLOC_005099**	-1.79	4.18E-02	6.19E-02
**AC006947.1**	-1.75	4.19E-02	6.20E-02
**RP11-522B15.3**	1.22	4.19E-02	6.20E-02
**RP11-10L12.4**	-0.52	4.19E-02	6.20E-02
**RP11-168K11.2**	-2.01	4.19E-02	6.21E-02
**RP11-624L4.1**	0.87	4.20E-02	6.21E-02
**MECP2**	0.29	4.20E-02	6.21E-02
**B3GALT5**	1.39	4.20E-02	6.21E-02
**SSR1**	-0.24	4.20E-02	6.21E-02
**AGPS**	-0.27	4.20E-02	6.22E-02
**ZNHIT3**	0.21	4.21E-02	6.22E-02
**RP11-403A21.1**	-0.97	4.21E-02	6.23E-02
**ARF4-AS1**	-1.85	4.21E-02	6.23E-02
**RPL39**	-0.35	4.21E-02	6.23E-02
**HMGN2P8**	-2.42	4.21E-02	6.23E-02
**AC062017.1**	0.79	4.21E-02	6.23E-02
**RP11-218M22.1**	-0.46	4.21E-02	6.23E-02
**GLYATL1P3**	-1.69	4.21E-02	6.23E-02
**CTD-2184C24.2**	-1.30	4.21E-02	6.23E-02
**FAM66C**	-0.74	4.22E-02	6.24E-02
**RNU6-1284P**	-2.59	4.22E-02	6.24E-02
**HOXB5**	1.26	4.22E-02	6.24E-02
**RP11-676J12.6**	1.71	4.23E-02	6.25E-02
**WTAPP1**	1.64	4.23E-02	6.25E-02
**RNU4-8P**	-2.65	4.23E-02	6.25E-02
**RP11-214N1.1**	-3.16	4.23E-02	6.25E-02
**TRAF3IP3**	-0.58	4.23E-02	6.25E-02
**RP11-641C17.2**	-2.48	4.23E-02	6.25E-02
**LACTB2**	-0.46	4.23E-02	6.25E-02
**IGLV1-51**	-1.93	4.23E-02	6.25E-02
**DLK2**	0.55	4.23E-02	6.26E-02
**ASAH2C**	-2.58	4.23E-02	6.26E-02
**RP11-462B18.3**	-1.88	4.23E-02	6.26E-02
**G22375**	-0.79	4.24E-02	6.26E-02
**PPP1CB**	-0.35	4.24E-02	6.27E-02
**MRPS24**	-1.24	4.24E-02	6.27E-02
**AC009061.1**	1.45	4.24E-02	6.27E-02
**RP13-554M15.5**	-1.69	4.24E-02	6.27E-02
**SPDYE18**	-0.94	4.25E-02	6.28E-02

**ANKRD1**	-2.21	4.25E-02	6.28E-02
**FLCN**	0.29	4.25E-02	6.28E-02
**EVI2A**	0.78	4.26E-02	6.29E-02
**RP11-352G18.2**	-1.17	4.26E-02	6.29E-02
**EYA1**	-1.42	4.26E-02	6.29E-02
**RP5-901A4.1**	-1.42	4.26E-02	6.30E-02
**RP4-758J24.4**	-2.83	4.26E-02	6.30E-02
**CEL**	0.90	4.27E-02	6.30E-02
**LINC01317**	-1.26	4.27E-02	6.30E-02
**RP11-497H16.4**	1.32	4.27E-02	6.30E-02
**CD8A**	0.75	4.27E-02	6.30E-02
**ASCL4**	-1.31	4.27E-02	6.30E-02
**G30934**	-1.18	4.27E-02	6.31E-02
**TRIM29**	0.41	4.28E-02	6.31E-02
**PLEKHG7**	-1.33	4.28E-02	6.31E-02
**RPL23**	0.31	4.28E-02	6.32E-02
**CTD-2128A3.2**	2.05	4.28E-02	6.32E-02
**RPSAP9**	1.17	4.28E-02	6.32E-02
**RNA5SP290**	-2.66	4.29E-02	6.33E-02
**CTD-2047H16.2**	1.39	4.29E-02	6.33E-02
**KITLG**	-0.64	4.29E-02	6.33E-02
**WDR90**	0.54	4.29E-02	6.33E-02
**LINC01287**	-2.47	4.29E-02	6.33E-02
**OR2AE1**	-2.49	4.29E-02	6.34E-02
**FAM173B**	-0.48	4.29E-02	6.34E-02
**RPS15AP16**	0.99	4.29E-02	6.34E-02
**EP300**	-0.29	4.30E-02	6.34E-02
**ADAMTS8**	0.84	4.30E-02	6.34E-02
**DENND6A-AS1**	-1.37	4.31E-02	6.35E-02
**AC079610.2**	-2.65	4.31E-02	6.35E-02
**SYCN**	-1.14	4.31E-02	6.35E-02
**GABRB2**	-1.10	4.31E-02	6.35E-02
**RP11-161H23.9**	-3.01	4.31E-02	6.35E-02
**MIR1273F**	-2.80	4.31E-02	6.36E-02
**RP11-3N13.2**	-3.23	4.31E-02	6.36E-02
**LSM1P1**	-3.22	4.31E-02	6.36E-02
**NRD1**	-0.32	4.31E-02	6.36E-02
**BTBD9**	-0.21	4.32E-02	6.38E-02
**RP3-477O4.14**	-1.44	4.33E-02	6.38E-02
**UTP18**	-0.27	4.33E-02	6.38E-02
**MYO3B**	0.83	4.33E-02	6.38E-02
**ASNS**	-0.32	4.33E-02	6.38E-02
**TSKU**	0.30	4.33E-02	6.38E-02
**DGKI**	0.69	4.33E-02	6.39E-02
**RP11-181D18.4**	-1.56	4.33E-02	6.39E-02

**LINC00574**	1.97	4.33E-02	6.39E-02
**C9orf147**	-1.03	4.34E-02	6.39E-02
**GBP1**	0.39	4.34E-02	6.39E-02
**MRAP**	-1.56	4.34E-02	6.40E-02
**RP11-802E16.3**	1.10	4.34E-02	6.40E-02
**ABCC6**	0.77	4.35E-02	6.41E-02
**RP11-627G18.2**	-2.66	4.35E-02	6.42E-02
**RP11-108M9.6**	-1.74	4.35E-02	6.42E-02
**MZB1**	1.48	4.36E-02	6.42E-02
**GGT3P**	1.90	4.37E-02	6.44E-02
**RP11-324E6.10**	-1.48	4.37E-02	6.44E-02
**SLC25A44**	0.25	4.38E-02	6.45E-02
**AC109333.10**	-1.47	4.38E-02	6.45E-02
**RN7SL21P**	-2.05	4.38E-02	6.45E-02
**RP11-51O6.1**	-0.51	4.39E-02	6.46E-02
**G7666**	-0.79	4.39E-02	6.46E-02
**NCOA6**	0.30	4.39E-02	6.46E-02
**PPARG**	1.35	4.39E-02	6.46E-02
**GPR20**	1.11	4.39E-02	6.47E-02
**SLC25A21**	-0.85	4.39E-02	6.47E-02
**XLOC_005560**	1.19	4.39E-02	6.47E-02
**ZNF550**	-0.32	4.40E-02	6.47E-02
**AGBL1**	-2.79	4.40E-02	6.47E-02
**DSG2-AS1**	-1.70	4.40E-02	6.48E-02
**RNU6-539P**	-1.86	4.40E-02	6.48E-02
**ESF1**	0.37	4.40E-02	6.48E-02
**CRK**	0.20	4.41E-02	6.49E-02
**RAB28P5**	-1.88	4.41E-02	6.49E-02
**AC108448.2**	-1.77	4.41E-02	6.49E-02
**G8320**	-1.08	4.41E-02	6.49E-02
**MPHOSPH10**	0.24	4.41E-02	6.50E-02
**AC007392.3**	1.37	4.41E-02	6.50E-02
**RPL35P6**	-2.53	4.41E-02	6.50E-02
**FAM41C**	-2.74	4.42E-02	6.50E-02
**RN7SL144P**	-2.81	4.42E-02	6.51E-02
**CC2D2A**	-0.37	4.42E-02	6.51E-02
**NR1I2**	-2.46	4.42E-02	6.51E-02
**GLUD2**	0.68	4.43E-02	6.52E-02
**BTN1A1**	-2.11	4.43E-02	6.52E-02
**CTD-2325B11.1**	-2.96	4.43E-02	6.52E-02
**TPT1-AS1**	-0.52	4.43E-02	6.53E-02
**OR7A19P**	-1.93	4.44E-02	6.53E-02
**TRMT44**	-0.36	4.44E-02	6.54E-02
**RNU7-49P**	-2.10	4.45E-02	6.54E-02
**RNU7-163P**	-2.03	4.45E-02	6.54E-02

**G31519**	-0.99	4.45E-02	6.55E-02
**ZNF807**	-2.11	4.45E-02	6.55E-02
**RP11-320M2.1**	1.26	4.45E-02	6.55E-02
**RP11-816J6.3**	-1.31	4.46E-02	6.55E-02
**OVOL2**	0.49	4.46E-02	6.56E-02
**G38700**	0.89	4.46E-02	6.56E-02
**REV3L-IT1**	-1.36	4.46E-02	6.56E-02
**FZD10**	0.51	4.46E-02	6.57E-02
**LRRC71**	-1.43	4.47E-02	6.57E-02
**MAPT-AS1**	-2.68	4.47E-02	6.57E-02
**FAM228A**	1.43	4.47E-02	6.57E-02
**LA16c-306A4.1**	-1.80	4.47E-02	6.58E-02
**RP11-111F5.4**	1.25	4.47E-02	6.58E-02
**C1orf189**	-1.71	4.48E-02	6.59E-02
**TUBB4A**	-0.82	4.48E-02	6.59E-02
**DHX40**	-0.29	4.49E-02	6.59E-02
**RP11-739N20.3**	-2.78	4.49E-02	6.60E-02
**ALDH8A1**	-1.17	4.49E-02	6.60E-02
**PRORSD1P**	0.78	4.49E-02	6.60E-02
**STAB2**	1.14	4.49E-02	6.60E-02
**ACYP1**	0.43	4.49E-02	6.60E-02
**RP1-278O22.1**	-0.93	4.49E-02	6.61E-02
**CCNY**	0.22	4.50E-02	6.61E-02
**KRT34**	2.11	4.50E-02	6.61E-02
**G11279**	-3.02	4.50E-02	6.61E-02
**WNT3A**	-0.52	4.50E-02	6.61E-02
**TRG-AS1**	0.98	4.50E-02	6.62E-02
**AC074011.2**	1.74	4.51E-02	6.63E-02
**RP11-74D7.3**	-1.63	4.51E-02	6.63E-02
**AL359832.1**	-2.71	4.52E-02	6.64E-02
**ZNF396**	0.34	4.52E-02	6.64E-02
**ZNF559**	-0.42	4.52E-02	6.64E-02
**NHSL2**	0.62	4.52E-02	6.64E-02
**CTC-236F12.4**	-0.86	4.52E-02	6.65E-02
**CH507-9B2.5**	-1.38	4.53E-02	6.65E-02
**RP11-147L13.13**	-0.45	4.53E-02	6.66E-02
**PMS2P7**	0.95	4.54E-02	6.67E-02
**RP11-90K6.1**	-2.84	4.54E-02	6.67E-02
**CTB-134H23.3**	-2.69	4.54E-02	6.67E-02
**CDKN2B-AS1**	1.38	4.54E-02	6.67E-02
**RP1-151F17.1**	0.59	4.54E-02	6.67E-02
**GCOM2**	-1.46	4.55E-02	6.68E-02
**GRPEL1**	0.26	4.55E-02	6.68E-02
**FGD5-AS1**	-0.22	4.55E-02	6.68E-02
**TWF1P1**	-1.03	4.55E-02	6.69E-02

**EIF3M**	-0.24	4.55E-02	6.69E-02
**MIS18A-AS1**	-1.08	4.56E-02	6.69E-02
**IDNK**	0.28	4.56E-02	6.69E-02
**RP11-727A23.10**	-1.73	4.56E-02	6.70E-02
**RP1-131F15.2**	-2.01	4.56E-02	6.70E-02
**WDR92**	0.33	4.56E-02	6.70E-02
**RPS4XP11**	-2.36	4.57E-02	6.70E-02
**RP11-88E10.4**	1.17	4.57E-02	6.71E-02
**RNU6-828P**	-2.48	4.57E-02	6.71E-02
**RP11-680C21.1**	1.25	4.57E-02	6.71E-02
**RP11-872J21.3**	1.11	4.57E-02	6.71E-02
**PCSK1N**	1.39	4.57E-02	6.71E-02
**SMAD9**	-0.47	4.57E-02	6.71E-02
**MIR4740**	2.35	4.58E-02	6.71E-02
**AHCYL2**	-0.30	4.58E-02	6.71E-02
**RP11-848P1.4**	-1.32	4.58E-02	6.71E-02
**ITGB2-AS1**	1.12	4.58E-02	6.71E-02
**RP13-726E6.2**	-1.56	4.58E-02	6.71E-02
**AUTS2**	0.45	4.58E-02	6.71E-02
**ALDH5A1**	-0.42	4.58E-02	6.71E-02
**RP11-235E17.3**	-2.08	4.58E-02	6.72E-02
**LKAAEAR1**	1.75	4.58E-02	6.72E-02
**DESI1**	0.29	4.58E-02	6.72E-02
**KRTAP9-3**	-4.70	4.58E-02	6.72E-02
**LINC01036**	-2.77	4.58E-02	6.72E-02
**CTC-336P14.1**	-1.08	4.58E-02	6.72E-02
**RP4-678D15.1**	-3.23	4.58E-02	6.72E-02
**ABLIM2**	0.44	4.59E-02	6.73E-02
**RP11-972P1.11**	-1.32	4.59E-02	6.73E-02
**CTD-2270F17.1**	-2.94	4.59E-02	6.73E-02
**RP11-145A3.2**	2.33	4.59E-02	6.73E-02
**MUCL1**	1.45	4.59E-02	6.74E-02
**AK5**	0.63	4.60E-02	6.74E-02
**SLC22A31**	-1.69	4.60E-02	6.74E-02
**PABPC1P7**	-1.73	4.60E-02	6.74E-02
**RP5-1132H15.3**	-1.25	4.60E-02	6.75E-02
**RSBN1L**	-0.22	4.60E-02	6.75E-02
**CELP**	-2.19	4.60E-02	6.75E-02
**RP11-90L20.2**	-1.32	4.60E-02	6.75E-02
**RP11-44F14.10**	1.38	4.61E-02	6.75E-02
**AKR7A3**	0.73	4.61E-02	6.75E-02
**TMEM246**	-0.38	4.61E-02	6.75E-02
**RNA5SP160**	-1.72	4.61E-02	6.75E-02
**FAM57A**	-0.30	4.61E-02	6.75E-02
**RNA5SP268**	-2.50	4.61E-02	6.76E-02

**HAUS1P1**	-1.98	4.61E-02	6.76E-02
**MRPL15**	0.35	4.61E-02	6.76E-02
**RPS27L**	0.33	4.61E-02	6.76E-02
**RBBP7**	-0.31	4.62E-02	6.76E-02
**DENND2A**	0.74	4.62E-02	6.76E-02
**RP11-54I5.1**	-1.22	4.62E-02	6.76E-02
**AC012363.7**	-2.47	4.62E-02	6.77E-02
**NHP2P1**	-1.72	4.62E-02	6.77E-02
**MGEA5**	-0.27	4.62E-02	6.77E-02
**KIAA1522**	0.52	4.62E-02	6.77E-02
**SEPT14P12**	-2.37	4.63E-02	6.78E-02
**RP5-1048B16.1**	-3.25	4.63E-02	6.78E-02
**CTB-181H17.1**	-1.14	4.63E-02	6.78E-02
**RP11-138A9.2**	-0.94	4.63E-02	6.78E-02
**STK35**	-0.28	4.63E-02	6.78E-02
**KRTAP21-2**	-3.07	4.63E-02	6.78E-02
**RPL21P106**	-3.25	4.63E-02	6.78E-02
**CTD-2571L23.8**	-1.71	4.64E-02	6.79E-02
**XLOC_008668**	-2.16	4.64E-02	6.79E-02
**CMSS1**	0.35	4.64E-02	6.79E-02
**RNU6-866P**	-3.07	4.64E-02	6.79E-02
**CAB39P1**	-2.77	4.64E-02	6.79E-02
**CTD-3076O17.2**	-1.52	4.64E-02	6.79E-02
**AC012501.2**	-3.23	4.64E-02	6.79E-02
**CDC37L1**	-0.22	4.64E-02	6.80E-02
**ACSS1**	-0.35	4.64E-02	6.80E-02
**G5264**	-0.72	4.65E-02	6.80E-02
**IGKV1OR2-108**	-2.27	4.65E-02	6.81E-02
**TMIGD1**	-2.32	4.66E-02	6.81E-02
**RPL23AP67**	-2.03	4.66E-02	6.81E-02
**ZNF324**	-0.22	4.66E-02	6.82E-02
**GNG3**	-2.33	4.66E-02	6.82E-02
**ANXA2**	0.25	4.67E-02	6.83E-02
**RP11-119F19.4**	-0.64	4.68E-02	6.84E-02
**RP11-202D1.3**	-2.69	4.68E-02	6.84E-02
**RP3-453C12.8**	-2.59	4.68E-02	6.84E-02
**CTD-2639E6.4**	1.62	4.68E-02	6.84E-02
**ELOVL4**	-0.48	4.68E-02	6.84E-02
**PLA2G3**	0.55	4.68E-02	6.85E-02
**RP11-307P22.1**	-1.44	4.68E-02	6.85E-02
**RP3-467D16.3**	-2.55	4.69E-02	6.85E-02
**AC078883.4**	-1.38	4.69E-02	6.86E-02
**SLC30A3**	2.21	4.69E-02	6.86E-02
**RP4-568C11.4**	-0.54	4.69E-02	6.86E-02
**RP4-785G19.2**	-2.69	4.69E-02	6.86E-02

**TMEM95**	-1.48	4.69E-02	6.86E-02
**PMP22**	0.54	4.70E-02	6.87E-02
**G263**	-0.99	4.70E-02	6.87E-02
**DSTNP2**	0.54	4.70E-02	6.87E-02
**MAPK9**	0.18	4.70E-02	6.87E-02
**TGM7**	2.32	4.70E-02	6.87E-02
**MGME1**	-0.22	4.71E-02	6.88E-02
**IGKV2-23**	-3.05	4.71E-02	6.88E-02
**AC007000.11**	-0.87	4.71E-02	6.88E-02
**RP11-782C8.6**	-2.26	4.71E-02	6.88E-02
**ATP2A2**	0.33	4.71E-02	6.89E-02
**OR52H1**	-3.10	4.71E-02	6.89E-02
**LRRC73**	0.84	4.72E-02	6.89E-02
**SLC25A33**	0.42	4.72E-02	6.90E-02
**AP001059.7**	-2.56	4.72E-02	6.90E-02
**RP11-63E9.1**	1.78	4.72E-02	6.90E-02
**COX16**	-0.38	4.72E-02	6.90E-02
**MPL**	1.03	4.72E-02	6.90E-02
**CTD-2647E9.3**	-2.33	4.72E-02	6.90E-02
**RP11-318A15.8**	-2.61	4.73E-02	6.90E-02
**CTC-457E21.1**	-3.17	4.73E-02	6.91E-02
**TTLL9**	-1.17	4.73E-02	6.91E-02
**GFI1B**	-1.59	4.73E-02	6.91E-02
**FECH**	0.26	4.73E-02	6.91E-02
**RP11-447H19.3**	-2.94	4.73E-02	6.91E-02
**RP11-17P16.2**	-1.55	4.73E-02	6.91E-02
**GAPDHP74**	-3.16	4.74E-02	6.92E-02
**MIR2117HG**	-3.26	4.74E-02	6.92E-02
**CTD-2331H12.7**	-1.40	4.74E-02	6.92E-02
**RP11-415J8.5**	-2.19	4.74E-02	6.92E-02
**NBEA**	-0.76	4.75E-02	6.93E-02
**XRRA1**	-0.56	4.75E-02	6.93E-02
**CTC-518B2.10**	-1.84	4.75E-02	6.93E-02
**SPACA6P**	-0.55	4.75E-02	6.93E-02
**LINC00548**	-1.48	4.75E-02	6.93E-02
**ZCCHC8**	-0.21	4.75E-02	6.93E-02
**ASPHD2**	-0.55	4.75E-02	6.94E-02
**RP11-186N15.3**	-0.86	4.76E-02	6.94E-02
**RP11-629B11.5**	1.00	4.77E-02	6.96E-02
**RP11-567M16.2**	-0.88	4.77E-02	6.97E-02
**CTD-2256P15.1**	-2.19	4.77E-02	6.97E-02
**RNF219-AS1**	-1.91	4.77E-02	6.97E-02
**BAG2**	0.71	4.78E-02	6.97E-02
**AC007563.3**	-2.10	4.78E-02	6.97E-02
**AC093609.1**	-0.60	4.78E-02	6.97E-02

**TMIE**	0.76	4.78E-02	6.97E-02
**RN7SL368P**	2.13	4.78E-02	6.97E-02
**AL591893.1**	-1.65	4.78E-02	6.98E-02
**KIAA1211**	-0.67	4.79E-02	6.99E-02
**TEKT5**	1.35	4.80E-02	7.00E-02
**CTC-512J12.7**	-2.08	4.80E-02	7.00E-02
**XLOC_008277**	-1.63	4.80E-02	7.01E-02
**HDHD2**	-0.20	4.80E-02	7.01E-02
**RN7SKP16**	-1.59	4.80E-02	7.01E-02
**RPL12P25**	-1.74	4.82E-02	7.03E-02
**SNCA-AS1**	-2.53	4.82E-02	7.03E-02
**CHCHD2P2**	-1.28	4.82E-02	7.03E-02
**CDK14**	0.62	4.82E-02	7.04E-02
**CROCC**	0.45	4.83E-02	7.04E-02
**RNU4-23P**	-2.41	4.83E-02	7.04E-02
**KDELR2**	0.30	4.83E-02	7.04E-02
**AP000640.2**	-2.47	4.83E-02	7.04E-02
**MST1**	0.65	4.83E-02	7.04E-02
**KLHL22-IT1**	1.99	4.83E-02	7.05E-02
**LINC00906**	-1.23	4.84E-02	7.06E-02
**PHLDB2**	0.44	4.84E-02	7.06E-02
**AC006994.2**	-1.63	4.84E-02	7.06E-02
**RNU6-1257P**	-2.87	4.84E-02	7.06E-02
**AC020550.7**	-1.98	4.84E-02	7.06E-02
**CTC-203F4.2**	1.05	4.84E-02	7.06E-02
**RABL3**	-0.26	4.85E-02	7.06E-02
**RNU6-748P**	-2.73	4.85E-02	7.06E-02
**PIK3R5**	0.71	4.85E-02	7.07E-02
**IGFBP3**	0.87	4.85E-02	7.07E-02
**ACAD10**	0.27	4.86E-02	7.08E-02
**TOM1L1**	-0.31	4.86E-02	7.08E-02
**RP11-325L12.5**	-2.07	4.86E-02	7.08E-02
**GUSBP2**	-0.79	4.86E-02	7.08E-02
**RP11-779O18.2**	-3.33	4.86E-02	7.08E-02
**KDM2B**	-0.21	4.86E-02	7.08E-02
**MIR762HG**	0.79	4.86E-02	7.08E-02
**ZP2**	-2.76	4.87E-02	7.09E-02
**FOXI1**	2.49	4.87E-02	7.09E-02
**AC007386.4**	-2.29	4.87E-02	7.10E-02
**DHRS7B**	0.27	4.87E-02	7.10E-02
**1-Sep**	0.46	4.87E-02	7.10E-02
**SMIM10L2A**	0.41	4.88E-02	7.10E-02
**HMGB1P19**	-2.56	4.88E-02	7.10E-02
**RP11-296A18.6**	-1.27	4.88E-02	7.11E-02
**DPT**	1.02	4.88E-02	7.11E-02

**SPRN**	0.57	4.88E-02	7.11E-02
**RP11-154D17.1**	-1.44	4.88E-02	7.11E-02
**LINC01106**	0.69	4.88E-02	7.11E-02
**RP11-408A13.3**	-1.63	4.89E-02	7.11E-02
**G8753**	-0.80	4.89E-02	7.12E-02
**EPHA10**	1.22	4.89E-02	7.12E-02
**BMPR1APS2**	-1.97	4.90E-02	7.13E-02
**RBM11**	-1.10	4.90E-02	7.13E-02
**RSRP1**	-0.68	4.90E-02	7.14E-02
**SLC22A9**	-2.70	4.90E-02	7.14E-02
**OXCT1-AS1**	-0.96	4.90E-02	7.14E-02
**SLC26A4-AS1**	1.63	4.91E-02	7.14E-02
**RP11-63G10.3**	-1.61	4.91E-02	7.14E-02
**AC093627.12**	-2.71	4.91E-02	7.14E-02
**MFSD12**	-0.49	4.91E-02	7.14E-02
**RP11-179A10.1**	-2.48	4.91E-02	7.15E-02
**OR52H2P**	-2.73	4.91E-02	7.15E-02
**CALR4P**	-2.39	4.92E-02	7.15E-02
**MLPH**	0.45	4.93E-02	7.17E-02
**RP11-236P24.3**	-2.63	4.93E-02	7.17E-02
**RPL15**	0.29	4.94E-02	7.18E-02
**LINC01587**	-1.75	4.94E-02	7.18E-02
**RP11-618M23.5**	-2.34	4.94E-02	7.18E-02
**KCNIP2**	0.97	4.95E-02	7.20E-02
**COPA**	0.20	4.95E-02	7.21E-02
**RP11-418J17.3**	-2.36	4.96E-02	7.21E-02
**RP11-484L8.1**	-1.71	4.96E-02	7.21E-02
**RP11-359B12.2**	-0.51	4.96E-02	7.21E-02
**RP11-96D1.8**	-1.07	4.96E-02	7.22E-02
**ITGBL1**	0.97	4.96E-02	7.22E-02
**ATG10**	-0.38	4.97E-02	7.22E-02
**ACOT11**	0.63	4.97E-02	7.23E-02
**RP11-742D12.2**	-2.81	4.97E-02	7.23E-02
**RP11-643M14.1**	-1.75	4.98E-02	7.24E-02
**LA16c-380F5.3**	1.09	4.98E-02	7.24E-02
**RPS15AP24**	-2.07	4.99E-02	7.25E-02
**RP4-782L23.2**	-2.36	4.99E-02	7.25E-02
**RPL31P49**	-2.61	4.99E-02	7.25E-02
**PRRG3**	-0.77	4.99E-02	7.26E-02
**EIF4BP6**	-0.46	5.00E-02	7.26E-02
**RP11-652G5.1**	-2.59	5.00E-02	7.26E-02
**KRTAP11-1**	-3.17	5.00E-02	7.27E-02
**SOWAHB**	-0.52	5.00E-02	7.27E-02
**UQCRFS1P1**	0.62	5.00E-02	7.27E-02
**NPC1**	-0.22	5.01E-02	7.28E-02

**RPL13P12**	-1.76	5.01E-02	7.28E-02
**MTMR12**	-0.25	5.01E-02	7.29E-02
**DISC1-IT1**	-2.60	5.02E-02	7.29E-02
**AGT**	2.48	5.02E-02	7.30E-02
**G35852**	-2.78	5.02E-02	7.30E-02
**AL356475.1**	1.17	5.02E-02	7.30E-02
**ARMC12**	1.47	5.02E-02	7.30E-02
**AC005775.2**	1.71	5.02E-02	7.30E-02
**DOPEY2**	-0.44	5.03E-02	7.30E-02
**AC098795.1**	-2.39	5.03E-02	7.31E-02
**SLC26A1**	-0.71	5.03E-02	7.31E-02
**LINC01269**	-1.34	5.04E-02	7.31E-02
**PCNT**	-0.26	5.04E-02	7.31E-02
**RP11-142C4.5**	-1.24	5.04E-02	7.32E-02
**TCEANC2**	-0.21	5.04E-02	7.32E-02
**PKD1P1**	-2.56	5.04E-02	7.32E-02
**RP4-646N3.1**	-2.10	5.05E-02	7.33E-02
**RP11-107F6.4**	-2.52	5.05E-02	7.33E-02
**IGF2R**	-0.22	5.05E-02	7.33E-02
**RP13-726E6.1**	-2.75	5.05E-02	7.33E-02
**RP11-620J15.2**	1.70	5.05E-02	7.33E-02
**TRPM2-AS**	-1.73	5.05E-02	7.34E-02
**RP11-184B22.2**	-2.35	5.05E-02	7.34E-02
**GTF2IP23**	-0.82	5.06E-02	7.34E-02
**KCNC4-AS1**	-2.31	5.06E-02	7.35E-02
**HSPA1A**	0.36	5.06E-02	7.35E-02
**RP11-109A6.2**	-1.81	5.06E-02	7.35E-02
**DMRTA1**	-1.48	5.06E-02	7.35E-02
**RN7SL398P**	-1.97	5.07E-02	7.36E-02
**TMA16**	-0.24	5.07E-02	7.36E-02
**CTD-2315E11.1**	1.90	5.07E-02	7.36E-02
**WWC3-AS1**	-1.47	5.07E-02	7.36E-02
**RNU6-678P**	-2.11	5.08E-02	7.37E-02
**RP11-73M11.3**	-2.56	5.08E-02	7.37E-02
**RAB37**	0.82	5.08E-02	7.37E-02
**AC005831.1**	1.57	5.08E-02	7.37E-02
**CTD-2561J22.1**	-2.58	5.08E-02	7.37E-02
**MMP25**	0.82	5.09E-02	7.39E-02
**HIC2**	0.39	5.10E-02	7.39E-02
**XLOC_010005**	-3.30	5.10E-02	7.40E-02
**GTF2H2B**	-1.19	5.10E-02	7.40E-02
**MIR331**	-2.19	5.10E-02	7.40E-02
**RP11-299H21.1**	0.74	5.11E-02	7.42E-02
**CCNG1**	-0.34	5.11E-02	7.42E-02
**MFAP1P1**	1.50	5.12E-02	7.42E-02

**C10orf105**	-1.03	5.12E-02	7.42E-02
**CTD-2561B21.3**	-2.59	5.12E-02	7.43E-02
**HSP90AB4P**	1.10	5.12E-02	7.43E-02
**G36482**	0.74	5.12E-02	7.43E-02
**RP11-160H22.5**	-1.34	5.12E-02	7.43E-02
**H3F3AP6**	-0.64	5.12E-02	7.43E-02
**C19orf44**	0.36	5.13E-02	7.44E-02
**AC002310.17**	-1.99	5.13E-02	7.44E-02
**RP11-661G16.2**	-2.88	5.14E-02	7.45E-02
**EXOSC10**	-0.13	5.14E-02	7.45E-02
**PHKA2-AS1**	1.16	5.15E-02	7.47E-02
**EVPLL**	-1.34	5.16E-02	7.47E-02
**TIGD7**	-0.76	5.16E-02	7.48E-02
**RP11-360N9.2**	2.23	5.16E-02	7.48E-02
**FBXW11P1**	-1.36	5.16E-02	7.48E-02
**RPL23AP90**	-2.06	5.16E-02	7.48E-02
**RP11-459F6.3**	-0.95	5.17E-02	7.49E-02
**NPR3**	-0.64	5.17E-02	7.49E-02
**CTD-2054N24.2**	1.04	5.17E-02	7.50E-02
**RP11-385G16.1**	-2.54	5.18E-02	7.50E-02
**TAP1**	0.29	5.18E-02	7.50E-02
**AC097493.1**	-2.35	5.18E-02	7.50E-02
**RP11-71E19.1**	-2.33	5.18E-02	7.51E-02
**RP11-615I2.3**	-1.53	5.18E-02	7.51E-02
**CNTN1**	-1.10	5.18E-02	7.51E-02
**GPR183**	0.67	5.18E-02	7.51E-02
**PLSCR4**	0.55	5.19E-02	7.52E-02
**SPDEF**	2.30	5.20E-02	7.53E-02
**RP11-159H22.1**	-2.62	5.20E-02	7.53E-02
**LRRTM3**	-2.54	5.20E-02	7.53E-02
**GGNBP1**	-2.59	5.20E-02	7.53E-02
**RNU7-28P**	-2.43	5.20E-02	7.54E-02
**F10**	0.73	5.21E-02	7.54E-02
**FAM84B**	-0.37	5.21E-02	7.54E-02
**GGT8P**	-2.76	5.21E-02	7.54E-02
**RP11-371I20.2**	1.87	5.21E-02	7.54E-02
**FAM27E3**	-1.27	5.21E-02	7.54E-02
**RP11-473M20.5**	0.98	5.21E-02	7.54E-02
**PCDHGA5**	-0.47	5.21E-02	7.54E-02
**STAC**	-0.77	5.22E-02	7.55E-02
**RP11-266N13.2**	-2.52	5.22E-02	7.55E-02
**FALEC**	-0.81	5.22E-02	7.56E-02
**ZNF385B**	-1.15	5.22E-02	7.56E-02
**RP11-662B19.1**	-2.12	5.22E-02	7.56E-02
**SP100**	0.23	5.23E-02	7.57E-02

**LMO7**	-0.34	5.23E-02	7.57E-02
**CTC-429P9.3**	-0.38	5.23E-02	7.57E-02
**CTD-2376I4.1**	1.64	5.23E-02	7.57E-02
**BTF3L4**	-0.27	5.23E-02	7.57E-02
**FAM83D**	-0.42	5.24E-02	7.58E-02
**G30412**	-1.34	5.24E-02	7.58E-02
**HSBP1P1**	-2.57	5.24E-02	7.58E-02
**RBPJP7**	-1.96	5.24E-02	7.58E-02
**ANAPC15**	0.40	5.24E-02	7.58E-02
**RP11-334L9.1**	-1.97	5.24E-02	7.59E-02
**AC100848.1**	-2.98	5.25E-02	7.59E-02
**RMDN2-AS1**	-1.65	5.25E-02	7.59E-02
**HMGB3P8**	-1.08	5.25E-02	7.59E-02
**RP11-680F20.6**	-0.78	5.25E-02	7.60E-02
**PRTG**	0.86	5.25E-02	7.60E-02
**RP11-289I10.2**	1.55	5.25E-02	7.60E-02
**ATP5H**	0.27	5.25E-02	7.60E-02
**OR9I2P**	-2.76	5.26E-02	7.61E-02
**RP11-488I20.8**	-3.21	5.26E-02	7.61E-02
**CLDN17**	1.56	5.27E-02	7.61E-02
**AC005754.8**	-0.85	5.27E-02	7.62E-02
**CTA-113A6.1**	-1.84	5.27E-02	7.62E-02
**ESPN**	0.62	5.27E-02	7.62E-02
**DHX35**	0.20	5.27E-02	7.62E-02
**RP11-365P13.5**	-1.24	5.28E-02	7.63E-02
**RP11-261C10.7**	2.95	5.28E-02	7.64E-02
**CTC-209L16.1**	-2.32	5.28E-02	7.64E-02
**C21orf62-AS1**	-0.66	5.29E-02	7.64E-02
**KRTAP4-1**	-5.07	5.29E-02	7.65E-02
**RNU6-372P**	-3.08	5.29E-02	7.65E-02
**TMPRSS13**	0.40	5.29E-02	7.65E-02
**AP000560.3**	-0.73	5.30E-02	7.65E-02
**RP11-404O13.5**	-2.05	5.30E-02	7.66E-02
**ERICD**	1.25	5.30E-02	7.66E-02
**RP11-517I3.2**	0.42	5.31E-02	7.67E-02
**SLC7A11-AS1**	-1.60	5.31E-02	7.67E-02
**SALRNA2**	-1.91	5.31E-02	7.67E-02
**RP11-297K8.2**	-2.37	5.32E-02	7.68E-02
**WEE2-AS1**	0.61	5.32E-02	7.68E-02
**SPINK4**	-1.78	5.32E-02	7.68E-02
**GOLGA8R**	0.66	5.32E-02	7.69E-02
**QSOX2**	-0.25	5.32E-02	7.69E-02
**KRTAP2-4**	-4.45	5.33E-02	7.70E-02
**LINC00637**	-2.69	5.33E-02	7.70E-02
**RP11-81A22.5**	0.78	5.33E-02	7.70E-02

**CLCP2**	-2.42	5.34E-02	7.71E-02
**IGHV3-49**	4.87	5.34E-02	7.71E-02
**DIO2-AS1**	-1.28	5.34E-02	7.71E-02
**RP11-383C5.5**	-1.42	5.34E-02	7.71E-02
**HBQ1**	-2.43	5.34E-02	7.71E-02
**RP11-1055B8.2**	-2.36	5.34E-02	7.71E-02
**RP11-87N3.6**	-2.64	5.34E-02	7.71E-02
**ABCF2**	0.25	5.34E-02	7.71E-02
**RP11-958F21.1**	-1.87	5.34E-02	7.71E-02
**RP11-25G10.2**	-2.54	5.34E-02	7.71E-02
**PIP5K1B**	0.93	5.34E-02	7.72E-02
**RP11-547D23.1**	-1.77	5.35E-02	7.72E-02
**RN7SL707P**	-2.38	5.35E-02	7.72E-02
**ATRAID**	0.27	5.35E-02	7.72E-02
**DNAJC12**	0.98	5.36E-02	7.73E-02
**RAD52**	-0.41	5.36E-02	7.73E-02
**STAG3L5P**	-0.65	5.36E-02	7.74E-02
**DPY19L3**	-0.29	5.36E-02	7.74E-02
**LINC00526**	0.43	5.36E-02	7.74E-02
**RBM18**	-0.25	5.37E-02	7.74E-02
**RP11-723J4.3**	-1.77	5.37E-02	7.74E-02
**CTC-308K20.3**	-2.35	5.37E-02	7.75E-02
**RP11-423E7.1**	-2.10	5.37E-02	7.75E-02
**G16361**	1.63	5.38E-02	7.76E-02
**RP11-96D1.7**	-1.87	5.38E-02	7.76E-02
**CTD-3035D6.1**	-2.55	5.38E-02	7.76E-02
**CICP26**	-1.93	5.39E-02	7.77E-02
**CTC-351M12.1**	0.56	5.39E-02	7.77E-02
**G25119**	-3.36	5.40E-02	7.79E-02
**CTC-428G20.6**	-0.95	5.40E-02	7.79E-02
**METTL6**	-0.23	5.40E-02	7.79E-02
**FGF10**	1.41	5.40E-02	7.79E-02
**ALDH18A1**	0.22	5.41E-02	7.79E-02
**RP4-737E23.7**	1.37	5.41E-02	7.79E-02
**NUCB2**	0.34	5.41E-02	7.80E-02
**AC007391.2**	-2.94	5.41E-02	7.80E-02
**AC068896.1**	-2.69	5.42E-02	7.81E-02
**RN7SL108P**	-3.08	5.42E-02	7.81E-02
**HN1L**	0.29	5.42E-02	7.81E-02
**AKAP1**	0.29	5.42E-02	7.82E-02
**ZNF211**	-0.34	5.42E-02	7.82E-02
**CNTNAP3B**	-0.84	5.43E-02	7.82E-02
**AC141586.5**	0.51	5.43E-02	7.82E-02
**IARS2**	-0.20	5.43E-02	7.83E-02
**STARD9**	-0.51	5.44E-02	7.84E-02

**RP11-84A14.4**	-1.27	5.44E-02	7.84E-02
**RP11-15A1.4**	-0.98	5.44E-02	7.84E-02
**RP11-19G24.2**	-2.69	5.44E-02	7.85E-02
**G15152**	1.64	5.45E-02	7.85E-02
**LNX1-AS2**	-2.03	5.45E-02	7.85E-02
**GTF2IRD2P1**	1.73	5.46E-02	7.86E-02
**CLMP**	-1.22	5.46E-02	7.87E-02
**RP11-265N7.2**	-2.80	5.46E-02	7.87E-02
**TRIM17**	0.61	5.46E-02	7.87E-02
**RP11-839D17.3**	1.35	5.46E-02	7.87E-02
**SNX6P1**	-3.59	5.46E-02	7.87E-02
**G41381**	-1.30	5.46E-02	7.87E-02
**XLOC_012288**	-1.40	5.47E-02	7.87E-02
**RPL5P34**	-1.00	5.47E-02	7.88E-02
**RP11-47J17.1**	-2.05	5.47E-02	7.88E-02
**AC000068.10**	-1.67	5.47E-02	7.88E-02
**LINC01545**	-2.01	5.48E-02	7.89E-02
**RCN1P2**	0.92	5.48E-02	7.89E-02
**RP11-750H9.5**	0.93	5.49E-02	7.90E-02
**KRT2**	-1.13	5.49E-02	7.91E-02
**RP11-511H23.2**	-0.79	5.49E-02	7.91E-02
**AC108861.1**	-2.61	5.49E-02	7.91E-02
**RP5-1061H20.4**	-0.95	5.50E-02	7.92E-02
**TUBB8P2**	-2.10	5.50E-02	7.92E-02
**LINC01183**	-0.79	5.50E-02	7.92E-02
**G12029**	1.84	5.50E-02	7.92E-02
**SESTD1**	-0.44	5.51E-02	7.92E-02
**CPSF4L**	-1.40	5.51E-02	7.94E-02
**ZNF296**	0.63	5.52E-02	7.94E-02
**RP3-337H4.9**	-2.44	5.52E-02	7.94E-02
**TNFSF8**	1.05	5.52E-02	7.94E-02
**RP5-875O13.7**	-4.04	5.52E-02	7.95E-02
**XLOC_011533**	-1.92	5.53E-02	7.96E-02
**KRTAP4-7**	-4.49	5.53E-02	7.96E-02
**SETD9**	0.50	5.53E-02	7.96E-02
**RP5-1041C10.3**	-1.14	5.54E-02	7.97E-02
**SH3PXD2A**	0.24	5.54E-02	7.97E-02
**XLOC_001189**	-1.84	5.54E-02	7.97E-02
**FZD7**	0.47	5.54E-02	7.97E-02
**KRTAP10-10**	-3.62	5.55E-02	7.98E-02
**PGLYRP1**	-1.48	5.55E-02	7.98E-02
**DUSP10**	-0.27	5.55E-02	7.99E-02
**SNTG2**	-0.59	5.56E-02	7.99E-02
**STMN1**	0.34	5.56E-02	8.00E-02
**RP11-712B9.5**	-0.86	5.56E-02	8.00E-02

**NIM1K**	0.60	5.56E-02	8.00E-02
**RN7SL558P**	-1.33	5.56E-02	8.00E-02
**KLRG1**	0.88	5.56E-02	8.00E-02
**AMZ2P1**	0.46	5.56E-02	8.00E-02
**RP11-145E17.3**	-1.98	5.57E-02	8.00E-02
**AC105921.5**	-2.81	5.57E-02	8.01E-02
**XLOC_013952**	-1.72	5.57E-02	8.01E-02
**CTD-3138B18.4**	-2.33	5.57E-02	8.01E-02
**G12464**	-2.42	5.57E-02	8.01E-02
**MIR143HG**	-0.81	5.58E-02	8.02E-02
**KRTAP1-5**	-3.58	5.58E-02	8.02E-02
**RP11-242C19.2**	-1.24	5.58E-02	8.02E-02
**RP5-1120P11.3**	-2.25	5.58E-02	8.03E-02
**AC009951.2**	-2.62	5.59E-02	8.03E-02
**ZAP70**	0.53	5.59E-02	8.03E-02
**FBXL5**	-0.22	5.59E-02	8.03E-02
**RP11-474D1.3**	-2.59	5.59E-02	8.04E-02
**PKP4**	-0.22	5.60E-02	8.05E-02
**RP11-545A16.3**	-1.75	5.60E-02	8.05E-02
**XXbac-BPG55C20.7**	2.62	5.60E-02	8.05E-02
**SYT5**	1.25	5.61E-02	8.06E-02
**SRRM1**	0.29	5.61E-02	8.06E-02
**XLOC_013955**	-1.68	5.61E-02	8.07E-02
**EP300-AS1**	0.56	5.62E-02	8.07E-02
**GS1-39E22.2**	-2.74	5.62E-02	8.07E-02
**DUBR**	-0.42	5.62E-02	8.07E-02
**RP11-142M10.2**	-1.58	5.62E-02	8.08E-02
**ZRANB2**	-0.37	5.63E-02	8.08E-02
**RP11-680A11.5**	0.95	5.63E-02	8.08E-02
**RP11-423O2.3**	-2.66	5.63E-02	8.08E-02
**MIR3192**	-1.95	5.63E-02	8.08E-02
**PCDHB8**	1.06	5.63E-02	8.09E-02
**AC007389.3**	-1.02	5.63E-02	8.09E-02
**PEG10**	0.61	5.63E-02	8.09E-02
**SEC23IP**	-0.14	5.63E-02	8.09E-02
**RP11-438L7.3**	-1.60	5.64E-02	8.09E-02
**GLYAT**	1.30	5.64E-02	8.09E-02
**XXYLT1-AS2**	-1.09	5.65E-02	8.11E-02
**VAMP1**	-0.49	5.65E-02	8.11E-02
**BTN3A3**	0.44	5.65E-02	8.12E-02
**RP11-273B20.1**	1.16	5.66E-02	8.12E-02
**DNAJC14**	-0.23	5.66E-02	8.13E-02
**CNOT8**	0.22	5.67E-02	8.13E-02
**HSD52**	-2.49	5.67E-02	8.14E-02
**RP11-29H23.6**	-2.39	5.67E-02	8.14E-02

**KRTAP19-1**	-4.75	5.67E-02	8.15E-02
**RP1-101K10.6**	-1.67	5.68E-02	8.15E-02
**CTC-436P18.1**	-1.84	5.68E-02	8.16E-02
**G33806**	1.38	5.69E-02	8.16E-02
**RP11-404P21.1**	-2.27	5.69E-02	8.16E-02
**MICAL3**	-0.31	5.69E-02	8.17E-02
**RP11-16E12.1**	-1.16	5.69E-02	8.17E-02
**G16040**	-1.71	5.70E-02	8.17E-02
**AC026703.2**	-2.41	5.70E-02	8.18E-02
**LPCAT2**	0.27	5.70E-02	8.18E-02
**CCT6P1**	-0.38	5.71E-02	8.19E-02
**PHF11**	0.23	5.72E-02	8.20E-02
**CPNE4**	-1.00	5.72E-02	8.21E-02
**ALG2**	0.19	5.72E-02	8.21E-02
**HBM**	-2.56	5.72E-02	8.21E-02
**UPK1A**	1.55	5.72E-02	8.21E-02
**SETMAR**	0.30	5.73E-02	8.22E-02
**ANAPC13**	0.29	5.73E-02	8.22E-02
**RASGEF1C**	1.06	5.73E-02	8.22E-02
**RP5-1024G6.2**	-1.78	5.73E-02	8.22E-02
**PAQR4**	0.38	5.74E-02	8.23E-02
**CTD-2302E22.4**	-1.57	5.74E-02	8.23E-02
**TMEM106A**	0.73	5.74E-02	8.23E-02
**RP3-398G3.5**	-2.37	5.74E-02	8.23E-02
**RNU6-880P**	-2.87	5.74E-02	8.24E-02
**RNA5SP282**	-2.00	5.74E-02	8.24E-02
**HCST**	1.07	5.75E-02	8.24E-02
**ATG9B**	0.79	5.75E-02	8.24E-02
**RP5-968D22.1**	-2.46	5.75E-02	8.24E-02
**RHBG**	-0.51	5.75E-02	8.24E-02
**DDX1**	0.25	5.76E-02	8.25E-02
**C5orf63**	-0.56	5.76E-02	8.26E-02
**RP4-781B1.5**	1.48	5.76E-02	8.26E-02
**MIR1913**	-2.48	5.76E-02	8.26E-02
**TNFRSF9**	-1.12	5.77E-02	8.27E-02
**PTGER1**	1.60	5.77E-02	8.27E-02
**AL953854.2**	-1.96	5.77E-02	8.27E-02
**SGCA**	0.79	5.77E-02	8.27E-02
**RP11-216L13.21**	-2.32	5.77E-02	8.27E-02
**CLEC18B**	1.21	5.78E-02	8.28E-02
**XLOC_002759**	-3.03	5.78E-02	8.28E-02
**RP5-1028K7.2**	1.60	5.78E-02	8.29E-02
**CTD-2537I9.13**	-1.51	5.79E-02	8.29E-02
**CXCR2P1**	-2.05	5.79E-02	8.29E-02
**RNU6-640P**	-2.55	5.79E-02	8.30E-02

**DEFB109P3**	-1.82	5.79E-02	8.30E-02
**ZNF812**	1.50	5.80E-02	8.30E-02
**UNC93A**	0.60	5.80E-02	8.31E-02
**LRRC17**	0.74	5.80E-02	8.31E-02
**MIR152**	-2.88	5.80E-02	8.31E-02
**PRNP**	0.29	5.81E-02	8.33E-02
**AC005189.6**	-2.40	5.82E-02	8.34E-02
**SUPT7L**	0.26	5.82E-02	8.34E-02
**AMFR**	0.19	5.82E-02	8.34E-02
**G6795**	-1.23	5.83E-02	8.34E-02
**RP11-429J17.7**	-0.86	5.83E-02	8.35E-02
**LSM14A**	0.24	5.83E-02	8.35E-02
**RP11-582J16.5**	-0.56	5.83E-02	8.35E-02
**GUSBP3**	-2.07	5.83E-02	8.35E-02
**VTN**	-0.98	5.84E-02	8.36E-02
**EIF2B1**	0.20	5.84E-02	8.36E-02
**CTB-102L5.8**	-1.42	5.84E-02	8.36E-02
**RP11-428J1.4**	0.67	5.84E-02	8.36E-02
**PRKCH**	-0.19	5.85E-02	8.37E-02
**RP11-1167A19.6**	-2.46	5.85E-02	8.38E-02
**CTD-2619J13.27**	-2.78	5.86E-02	8.38E-02
**RSL24D1P6**	-0.97	5.86E-02	8.38E-02
**PSMC6**	-0.25	5.86E-02	8.39E-02
**VCPKMT**	-0.30	5.87E-02	8.39E-02
**RPS19**	0.32	5.87E-02	8.40E-02
**RP11-888D10.3**	2.03	5.87E-02	8.40E-02
**MARK2P13**	-1.27	5.87E-02	8.40E-02
**SLC12A1**	-1.39	5.88E-02	8.41E-02
**RP11-1007I13.4**	-2.71	5.88E-02	8.41E-02
**DCAF8**	-0.26	5.88E-02	8.41E-02
**AC006946.16**	-0.81	5.88E-02	8.41E-02
**RETSAT**	-0.31	5.88E-02	8.41E-02
**XLOC_014369**	1.58	5.89E-02	8.42E-02
**SLC28A3**	-0.39	5.89E-02	8.42E-02
**CMC2**	-0.31	5.89E-02	8.42E-02
**YY1**	-0.16	5.89E-02	8.42E-02
**SYCP2**	-0.64	5.89E-02	8.43E-02
**ZSWIM3**	-0.34	5.89E-02	8.43E-02
**KCTD8**	-1.59	5.89E-02	8.43E-02
**FAM91A3P**	-1.69	5.89E-02	8.43E-02
**RNU6-1095P**	-2.72	5.90E-02	8.44E-02
**RP11-206F17.2**	-1.80	5.91E-02	8.45E-02
**GDF11**	0.43	5.91E-02	8.45E-02
**G27754**	0.95	5.91E-02	8.45E-02
**RP11-304L19.11**	-0.74	5.91E-02	8.45E-02

**G12734**	1.84	5.91E-02	8.45E-02
**AL162431.1**	-1.49	5.92E-02	8.46E-02
**TLE1P1**	1.65	5.92E-02	8.46E-02
**TRMU**	0.35	5.92E-02	8.46E-02
**RP11-180P8.1**	-0.76	5.92E-02	8.46E-02
**LLNLR-304A6.2**	-2.18	5.92E-02	8.47E-02
**AC011288.2**	2.69	5.93E-02	8.47E-02
**USP50**	-1.14	5.93E-02	8.47E-02
**C14orf159**	0.18	5.93E-02	8.48E-02
**RNU6-1165P**	-1.93	5.93E-02	8.48E-02
**DEFB4A**	2.20	5.93E-02	8.48E-02
**KRT36**	-1.40	5.94E-02	8.49E-02
**LCE2B**	-0.61	5.95E-02	8.51E-02
**RP11-488P3.1**	-1.06	5.96E-02	8.52E-02
**TGFA-IT1**	-2.37	5.96E-02	8.52E-02
**RP11-386M24.4**	1.01	5.97E-02	8.52E-02
**CROCCP2**	0.55	5.97E-02	8.53E-02
**DZIP3**	-0.37	5.97E-02	8.53E-02
**RP5-1007H16.1**	-1.64	5.97E-02	8.53E-02
**AL354764.1**	-2.69	5.97E-02	8.53E-02
**DTD2**	-0.30	5.98E-02	8.53E-02
**G26059**	-1.46	5.98E-02	8.54E-02
**AC013461.1**	-0.22	5.98E-02	8.54E-02
**NKIRAS1**	-0.23	5.98E-02	8.54E-02
**ROR1**	0.41	5.98E-02	8.55E-02
**LIG4**	-0.31	5.98E-02	8.55E-02
**RBP3**	-2.45	5.98E-02	8.55E-02
**ASB16**	-0.61	5.99E-02	8.55E-02
**SYNJ1**	-0.27	5.99E-02	8.55E-02
**RP11-79L9.2**	-1.04	5.99E-02	8.55E-02
**C8orf34-AS1**	1.62	5.99E-02	8.56E-02
**TPI1P1**	0.75	6.00E-02	8.56E-02
**RP11-531F16.3**	-1.11	6.00E-02	8.57E-02
**CNOT11**	0.16	6.00E-02	8.57E-02
**FOXP4-AS1**	-1.31	6.00E-02	8.57E-02
**SNCA**	-0.35	6.01E-02	8.58E-02
**LYPD6**	-0.77	6.01E-02	8.58E-02
**PTPRC**	-0.67	6.01E-02	8.58E-02
**NIP7**	0.31	6.01E-02	8.58E-02
**RP11-714L20.1**	2.02	6.02E-02	8.59E-02
**CTC-454I21.3**	-2.22	6.02E-02	8.59E-02
**AP4B1-AS1**	-1.05	6.02E-02	8.59E-02
**CCNG2**	0.40	6.02E-02	8.59E-02
**SS18L2**	0.32	6.03E-02	8.60E-02
**RP11-473O4.5**	-1.00	6.03E-02	8.60E-02

**CTD-2196E14.3**	-1.40	6.03E-02	8.60E-02
**SYT10**	-2.37	6.03E-02	8.61E-02
**GFPT1**	-0.28	6.03E-02	8.61E-02
**CENPN**	-0.37	6.03E-02	8.61E-02
**PCDHB7**	0.80	6.03E-02	8.61E-02
**COQ3**	0.33	6.04E-02	8.61E-02
**LRCOL1**	2.22	6.04E-02	8.61E-02
**RP11-435B5.4**	0.74	6.04E-02	8.62E-02
**MIR3177**	-2.53	6.04E-02	8.62E-02
**PLPP2**	-0.51	6.04E-02	8.62E-02
**RP11-374M1.9**	-2.44	6.05E-02	8.63E-02
**RP4-592A1.2**	-1.06	6.05E-02	8.63E-02
**EGR3**	-0.39	6.06E-02	8.64E-02
**RNU6-388P**	-1.91	6.06E-02	8.64E-02
**LA16c-425C2.1**	-1.60	6.06E-02	8.64E-02
**RP11-756P10.3**	-1.04	6.06E-02	8.64E-02
**SYNPO2L**	1.09	6.06E-02	8.64E-02
**HCP5B**	-2.31	6.06E-02	8.65E-02
**GAP43**	1.11	6.07E-02	8.65E-02
**TRAFD1**	0.24	6.07E-02	8.65E-02
**RNF24**	-0.32	6.07E-02	8.65E-02
**RP11-96O20.5**	-2.01	6.07E-02	8.66E-02
**GNAQ**	0.30	6.08E-02	8.66E-02
**RP11-479O9.3**	-1.19	6.08E-02	8.66E-02
**XLOC_014268**	-1.95	6.08E-02	8.67E-02
**MIR148B**	-2.31	6.09E-02	8.67E-02
**CTA-989H11.1**	-0.94	6.09E-02	8.67E-02
**OR2A42**	-2.62	6.09E-02	8.67E-02
**RP11-661A12.8**	-1.54	6.09E-02	8.68E-02
**C15orf57**	-0.43	6.10E-02	8.69E-02
**MIR5587**	-2.50	6.10E-02	8.69E-02
**G43247**	1.69	6.10E-02	8.69E-02
**KLF11**	0.27	6.10E-02	8.70E-02
**RP11-874G11.1**	-2.33	6.10E-02	8.70E-02
**MIR760**	-2.80	6.10E-02	8.70E-02
**ANXA4**	0.22	6.11E-02	8.71E-02
**SIRPB3P**	-2.66	6.11E-02	8.71E-02
**SCGB3A1**	2.12	6.12E-02	8.71E-02
**PARN**	0.17	6.12E-02	8.71E-02
**RP11-72I2.2**	-2.48	6.13E-02	8.72E-02
**XLOC_007998**	-1.91	6.13E-02	8.73E-02
**AC007271.3**	-2.22	6.13E-02	8.74E-02
**KATNA1**	0.17	6.13E-02	8.74E-02
**ZNF582-AS1**	0.88	6.14E-02	8.74E-02
**CD164**	-0.23	6.14E-02	8.74E-02

**DAG1**	-0.23	6.15E-02	8.75E-02
**NME5**	0.90	6.15E-02	8.76E-02
**RP11-182J23.1**	-1.98	6.15E-02	8.76E-02
**EHBP1**	-0.28	6.16E-02	8.76E-02
**RPS3AP3**	-1.89	6.16E-02	8.78E-02
**PPP1R1C**	0.79	6.16E-02	8.78E-02
**SEC31B**	-0.76	6.17E-02	8.78E-02
**RN7SL347P**	-2.27	6.17E-02	8.78E-02
**RN7SKP243**	-2.36	6.17E-02	8.78E-02
**RP11-768G7.1**	-2.61	6.18E-02	8.79E-02
**DUSP16**	-0.34	6.18E-02	8.79E-02
**XLOC_001200**	-0.79	6.18E-02	8.80E-02
**G7468**	2.00	6.19E-02	8.81E-02
**CTC-471F3.5**	-0.72	6.19E-02	8.81E-02
**AC104532.4**	-1.32	6.19E-02	8.81E-02
**XLOC_010336**	-0.77	6.19E-02	8.81E-02
**SNX18P12**	-2.86	6.19E-02	8.81E-02
**SUB1P4**	-2.11	6.19E-02	8.81E-02
**NXF3**	2.08	6.19E-02	8.81E-02
**HMGB1P5**	0.52	6.20E-02	8.81E-02
**RP11-461G12.2**	-2.60	6.20E-02	8.82E-02
**CEACAM7**	0.88	6.20E-02	8.82E-02
**RP11-236F9.2**	-1.77	6.21E-02	8.83E-02
**RP11-267M23.6**	-2.21	6.21E-02	8.83E-02
**CTD-2332E11.2**	-2.59	6.21E-02	8.84E-02
**CCDC79**	-1.45	6.21E-02	8.84E-02
**C4orf47**	0.78	6.21E-02	8.84E-02
**LINC00999**	-2.19	6.22E-02	8.85E-02
**G22271**	-0.80	6.22E-02	8.85E-02
**G10272**	-0.64	6.23E-02	8.85E-02
**OR7E15P**	-2.87	6.23E-02	8.86E-02
**XLOC_010693**	-1.75	6.23E-02	8.86E-02
**OLFM5P**	-1.46	6.23E-02	8.86E-02
**SLC22A4**	0.65	6.23E-02	8.86E-02
**IL18RAP**	1.07	6.24E-02	8.87E-02
**AL049542.1**	-2.29	6.24E-02	8.87E-02
**CSPG4P9**	-1.32	6.25E-02	8.88E-02
**LINGO1-AS1**	-3.26	6.25E-02	8.88E-02
**RNU6-1099P**	-1.58	6.25E-02	8.88E-02
**FAM60A**	-0.26	6.25E-02	8.89E-02
**XLOC_012115**	-2.52	6.25E-02	8.89E-02
**OTOA**	1.99	6.25E-02	8.89E-02
**CCDC112**	0.30	6.25E-02	8.89E-02
**XLOC_009607**	-1.48	6.26E-02	8.90E-02
**AP001062.7**	-0.57	6.26E-02	8.90E-02

**C22orf15**	-1.54	6.26E-02	8.90E-02
**SPNS3**	0.87	6.27E-02	8.91E-02
**RNU6-589P**	-3.08	6.27E-02	8.91E-02
**HMGB1P41**	-1.28	6.28E-02	8.92E-02
**RP1-95L4.4**	0.57	6.28E-02	8.92E-02
**RP11-142L1.1**	-1.44	6.28E-02	8.93E-02
**CTD-2078B5.2**	1.89	6.29E-02	8.93E-02
**CXorf21**	0.72	6.29E-02	8.93E-02
**XLOC_008986**	-1.19	6.29E-02	8.93E-02
**ADGRG4**	-1.71	6.29E-02	8.93E-02
**MTCYBP21**	-1.40	6.29E-02	8.93E-02
**ERAP2**	-1.12	6.29E-02	8.94E-02
**RP11-295D4.7**	-2.01	6.29E-02	8.94E-02
**RP11-211A18.2**	-1.50	6.29E-02	8.94E-02
**RP11-96D1.6**	0.75	6.30E-02	8.95E-02
**PPARGC1A**	-0.69	6.30E-02	8.95E-02
**LRRC10B**	1.47	6.31E-02	8.96E-02
**RP11-359D14.2**	-1.64	6.31E-02	8.96E-02
**FAM35DP**	0.55	6.31E-02	8.96E-02
**SUMO2P18**	-2.66	6.31E-02	8.96E-02
**FRRS1L**	-1.70	6.31E-02	8.96E-02
**CTD-2318O12.1**	-1.37	6.31E-02	8.96E-02
**G25097**	-2.21	6.31E-02	8.96E-02
**M6PR**	0.22	6.32E-02	8.98E-02
**OR2A1**	-1.96	6.33E-02	8.98E-02
**RP11-353N14.7**	-2.09	6.33E-02	8.98E-02
**CHAF1B**	0.35	6.33E-02	8.99E-02
**CNTD1**	-0.94	6.33E-02	8.99E-02
**G24578**	3.77	6.33E-02	8.99E-02
**ASTN1**	-1.29	6.34E-02	9.00E-02
**CLSTN3**	0.78	6.35E-02	9.01E-02
**FAM25G**	-1.95	6.35E-02	9.01E-02
**RP11-646E18.2**	-1.34	6.35E-02	9.01E-02
**KLF3-AS1**	-0.57	6.35E-02	9.01E-02
**HSP90AA2P**	-1.63	6.36E-02	9.02E-02
**TPX2**	0.39	6.36E-02	9.02E-02
**AC010525.2**	-2.85	6.36E-02	9.02E-02
**RPS4XP16**	-0.78	6.37E-02	9.03E-02
**SELT**	-0.30	6.37E-02	9.04E-02
**RP11-230C9.4**	-1.76	6.37E-02	9.04E-02
**CEP41**	0.36	6.39E-02	9.07E-02
**DCHS1**	0.64	6.40E-02	9.07E-02
**RP11-359M6.2**	1.91	6.40E-02	9.08E-02
**RP11-211N8.6**	-2.73	6.40E-02	9.08E-02
**PNMAL1**	0.90	6.40E-02	9.08E-02

**UBE4B**	-0.21	6.41E-02	9.08E-02
**TPTE2P3**	-2.63	6.41E-02	9.09E-02
**CS**	0.23	6.41E-02	9.09E-02
**EXOSC2**	-0.20	6.41E-02	9.09E-02
**RP11-1000B6.5**	-0.75	6.42E-02	9.10E-02
**G22194**	-1.33	6.42E-02	9.10E-02
**AC018804.1**	-2.45	6.42E-02	9.10E-02
**FRA10AC1**	0.26	6.42E-02	9.11E-02
**RP11-830F9.7**	-1.84	6.43E-02	9.11E-02
**RP4-633I8.3**	-1.55	6.43E-02	9.11E-02
**AC007238.1**	-1.19	6.43E-02	9.11E-02
**FOXL2**	1.68	6.43E-02	9.11E-02
**LSM5**	-0.27	6.43E-02	9.11E-02
**OR7E116P**	1.76	6.43E-02	9.12E-02
**HLA-Z**	-2.38	6.44E-02	9.12E-02
**PAICS**	-0.22	6.44E-02	9.12E-02
**RP11-401P9.5**	1.00	6.44E-02	9.12E-02
**RP1-292B18.3**	-2.27	6.45E-02	9.14E-02
**RNU6-877P**	1.70	6.46E-02	9.15E-02
**AC010525.4**	1.82	6.46E-02	9.15E-02
**RP11-329B9.4**	1.31	6.46E-02	9.15E-02
**RP11-616M22.12**	-2.58	6.46E-02	9.15E-02
**ASAH2B**	-0.36	6.46E-02	9.15E-02
**AC074366.3**	-2.17	6.46E-02	9.16E-02
**FBLN2**	0.79	6.47E-02	9.16E-02
**C11orf1**	-0.36	6.47E-02	9.16E-02
**RN7SL833P**	-2.73	6.47E-02	9.16E-02
**RP3-437C15.1**	-2.25	6.47E-02	9.16E-02
**XAF1**	0.72	6.47E-02	9.16E-02
**RP11-346J10.1**	-2.08	6.47E-02	9.17E-02
**MPP3**	0.58	6.48E-02	9.17E-02
**MIR548AC**	-1.99	6.48E-02	9.17E-02
**IGLV2-14**	-2.88	6.48E-02	9.18E-02
**RP11-204L24.2**	-1.47	6.48E-02	9.18E-02
**VTA1P1**	-1.88	6.49E-02	9.18E-02
**IDI2-AS1**	-1.58	6.49E-02	9.18E-02
**PCDHGA2**	-0.52	6.49E-02	9.19E-02
**NOVA1**	-0.78	6.49E-02	9.19E-02
**RP11-789C1.2**	-3.02	6.49E-02	9.19E-02
**RP11-347I19.7**	-1.58	6.50E-02	9.19E-02
**RP11-67C2.2**	-1.65	6.50E-02	9.21E-02
**CTD-2329K10.1**	-1.08	6.51E-02	9.22E-02
**CCDC105**	-2.37	6.51E-02	9.22E-02
**PTRH1**	-1.04	6.51E-02	9.22E-02
**MIR4767**	1.84	6.52E-02	9.22E-02

**CA2**	0.66	6.52E-02	9.22E-02
**IL34**	-0.40	6.53E-02	9.24E-02
**ITPR1**	0.54	6.53E-02	9.24E-02
**SPDYE16**	-1.10	6.53E-02	9.24E-02
**G31400**	-1.98	6.53E-02	9.24E-02
**RP11-44N21.1**	-0.76	6.53E-02	9.25E-02
**RN7SL753P**	-1.75	6.54E-02	9.25E-02
**RP11-465K4.4**	-2.49	6.54E-02	9.25E-02
**AL390776.1**	-2.67	6.55E-02	9.26E-02
**C16orf95**	0.94	6.56E-02	9.28E-02
**AP5B1**	-0.31	6.56E-02	9.28E-02
**MIR574**	-2.37	6.57E-02	9.29E-02
**LINC01160**	-1.53	6.57E-02	9.29E-02
**MIR4296**	-2.78	6.57E-02	9.29E-02
**RP11-328J14.1**	-2.27	6.57E-02	9.29E-02
**RP1-317E23.3**	-0.68	6.57E-02	9.30E-02
**IL18R1**	-0.37	6.57E-02	9.30E-02
**MMS19**	-0.16	6.58E-02	9.30E-02
**ZCCHC5**	2.73	6.58E-02	9.31E-02
**RPL24P2**	-1.68	6.58E-02	9.31E-02
**C9orf152**	0.81	6.58E-02	9.31E-02
**RP11-129G17.2**	-2.77	6.59E-02	9.31E-02
**GPR158**	-1.18	6.59E-02	9.32E-02
**TUBA4B**	-1.30	6.59E-02	9.32E-02
**NOLC1**	0.28	6.59E-02	9.32E-02
**WBP1**	0.60	6.59E-02	9.32E-02
**UNC50**	-0.18	6.59E-02	9.32E-02
**HAUS7**	-0.95	6.60E-02	9.32E-02
**RP11-111J6.2**	-1.12	6.60E-02	9.33E-02
**GNRHR**	-1.28	6.60E-02	9.33E-02
**CTD-3220F14.1**	-1.61	6.60E-02	9.33E-02
**RP11-452D12.1**	1.36	6.60E-02	9.33E-02
**GIMAP5**	-0.88	6.60E-02	9.33E-02
**RP11-153F5.3**	-2.56	6.60E-02	9.33E-02
**RP11-264J4.10**	2.06	6.61E-02	9.33E-02
**RPS7P10**	-1.05	6.61E-02	9.34E-02
**RP11-680F8.3**	0.60	6.61E-02	9.34E-02
**CTD-3092A11.1**	-0.58	6.61E-02	9.34E-02
**RPL7P15**	-2.85	6.62E-02	9.35E-02
**RLN1**	-1.46	6.62E-02	9.35E-02
**RBM24**	0.98	6.62E-02	9.35E-02
**RP11-474O21.5**	0.89	6.62E-02	9.35E-02
**ZCCHC10**	-0.29	6.62E-02	9.35E-02
**RP11-353N4.6**	1.44	6.62E-02	9.36E-02
**OR6L2P**	-1.32	6.63E-02	9.36E-02

**RP11-236L14.2**	1.57	6.63E-02	9.36E-02
**AC004051.2**	-2.12	6.63E-02	9.36E-02
**RP11-388C12.1**	1.52	6.64E-02	9.37E-02
**RP11-148K1.10**	-1.82	6.64E-02	9.37E-02
**RP11-562A8.5**	-1.20	6.65E-02	9.38E-02
**RP11-384B12.2**	-2.13	6.65E-02	9.39E-02
**AC099326.1**	-2.42	6.65E-02	9.39E-02
**RP4-694B14.8**	-1.10	6.65E-02	9.39E-02
**RP11-1102P22.3**	-1.15	6.65E-02	9.39E-02
**RNF212**	-0.54	6.65E-02	9.39E-02
**SERBP1P5**	-0.80	6.65E-02	9.39E-02
**RP11-329N15.3**	-1.16	6.66E-02	9.40E-02
**DAB1-AS1**	-2.77	6.66E-02	9.40E-02
**RP11-287D1.3**	-1.65	6.66E-02	9.41E-02
**RP11-885B4.2**	-2.02	6.66E-02	9.41E-02
**TMEM80**	0.38	6.67E-02	9.41E-02
**RP11-304L19.3**	-1.19	6.67E-02	9.41E-02
**C6orf201**	-1.21	6.67E-02	9.42E-02
**RP11-70P17.1**	-1.09	6.67E-02	9.42E-02
**PIR**	0.43	6.67E-02	9.42E-02
**MIR4796**	-2.71	6.67E-02	9.42E-02
**RP11-707O23.5**	-4.50	6.68E-02	9.42E-02
**CDH1**	-0.28	6.68E-02	9.42E-02
**RP11-452H21.2**	-1.96	6.68E-02	9.43E-02
**NLRP6**	1.18	6.68E-02	9.43E-02
**BCAP31P1**	2.03	6.69E-02	9.43E-02
**NUP54**	-0.24	6.69E-02	9.43E-02
**APTX**	0.16	6.69E-02	9.43E-02
**SRPRB**	0.27	6.69E-02	9.43E-02
**TTLL13P**	-2.35	6.69E-02	9.44E-02
**RP11-386J22.3**	-0.90	6.69E-02	9.44E-02
**PPP3CA**	-0.25	6.71E-02	9.46E-02
**XXbac-BPG27H4.8**	1.04	6.71E-02	9.46E-02
**AF064860.7**	1.56	6.71E-02	9.47E-02
**LINC00632**	-0.77	6.71E-02	9.47E-02
**FAM87A**	0.75	6.72E-02	9.47E-02
**AC009831.1**	-2.74	6.72E-02	9.47E-02
**RP11-320N21.1**	-0.81	6.72E-02	9.48E-02
**GTDC1**	0.24	6.72E-02	9.48E-02
**STRA8**	-1.86	6.72E-02	9.48E-02
**HSPD1P6**	1.40	6.73E-02	9.48E-02
**ZG16B**	0.80	6.73E-02	9.48E-02
**SH3TC2**	-0.52	6.73E-02	9.48E-02
**AC107983.4**	1.46	6.73E-02	9.49E-02
**CMP21-97G8.1**	-1.68	6.73E-02	9.49E-02

**AP1AR**	-0.28	6.73E-02	9.49E-02
**TUBA8**	-1.57	6.74E-02	9.49E-02
**NUDCD2**	0.20	6.74E-02	9.50E-02
**SIGLEC18P**	-2.74	6.74E-02	9.50E-02
**TPTE2P1**	1.15	6.74E-02	9.50E-02
**SGCB**	0.37	6.74E-02	9.51E-02
**RP11-225H22.4**	1.54	6.75E-02	9.51E-02
**G31401**	-1.34	6.75E-02	9.51E-02
**SYNE4**	-0.74	6.75E-02	9.51E-02
**RP11-428O18.6**	-1.34	6.75E-02	9.51E-02
**BEND7**	0.62	6.76E-02	9.52E-02
**TMEM69**	0.22	6.76E-02	9.53E-02
**TEX21P**	-1.12	6.76E-02	9.53E-02
**IL10**	1.56	6.76E-02	9.53E-02
**RP11-455I9.1**	-1.54	6.76E-02	9.53E-02
**RP11-250B2.6**	-0.69	6.78E-02	9.55E-02
**CLK4**	-0.35	6.78E-02	9.55E-02
**IER3IP1**	-0.37	6.78E-02	9.55E-02
**RP11-234B24.2**	-1.94	6.78E-02	9.55E-02
**RP11-706P11.2**	1.36	6.78E-02	9.55E-02
**RP11-332H18.7**	-1.92	6.79E-02	9.56E-02
**CUBNP3**	-1.78	6.79E-02	9.56E-02
**LINC00958**	-0.60	6.79E-02	9.57E-02
**MGAT4EP**	-2.58	6.80E-02	9.57E-02
**AC093822.1**	-2.55	6.81E-02	9.58E-02
**LA16c-312E8.4**	1.57	6.81E-02	9.59E-02
**AC006129.2**	1.09	6.81E-02	9.59E-02
**RP11-417L19.4**	1.56	6.81E-02	9.59E-02
**TCP10L**	-1.22	6.82E-02	9.60E-02
**MSTN**	-1.10	6.82E-02	9.60E-02
**TMEM248**	-0.17	6.82E-02	9.60E-02
**RP11-747H7.1**	-2.86	6.82E-02	9.61E-02
**AC016559.1**	-1.74	6.83E-02	9.61E-02
**CAPN6**	1.09	6.83E-02	9.61E-02
**VWA8-AS1**	-1.27	6.83E-02	9.61E-02
**RP11-247I13.3**	-1.60	6.83E-02	9.61E-02
**KSR1**	0.36	6.83E-02	9.61E-02
**RP11-553K23.2**	-1.70	6.83E-02	9.61E-02
**KIF20A**	0.53	6.84E-02	9.62E-02
**RP13-204A15.5**	-2.75	6.84E-02	9.62E-02
**C1GALT1C1L**	0.74	6.84E-02	9.62E-02
**GYPE**	0.74	6.84E-02	9.63E-02
**XLOC_012747**	-1.91	6.84E-02	9.63E-02
**RP11-32P22.1**	-2.32	6.84E-02	9.63E-02
**LRRC3B**	-2.21	6.84E-02	9.63E-02

**G35671**	-1.00	6.84E-02	9.63E-02
**AC004017.1**	-1.20	6.85E-02	9.63E-02
**HSPE1P2**	-2.40	6.85E-02	9.64E-02
**CTD-2007H13.3**	-0.79	6.85E-02	9.64E-02
**RP11-385D13.1**	-0.96	6.85E-02	9.64E-02
**DOK6**	0.79	6.86E-02	9.65E-02
**POU3F3**	-0.66	6.87E-02	9.66E-02
**CENPK**	-0.47	6.87E-02	9.66E-02
**RP11-325I22.3**	1.36	6.87E-02	9.66E-02
**MCTP2**	-0.31	6.88E-02	9.67E-02
**RP11-462L8.1**	2.67	6.88E-02	9.67E-02
**BNIP3P17**	-2.51	6.88E-02	9.67E-02
**FAM90A20P**	-1.65	6.88E-02	9.67E-02
**RSPH4A**	-0.62	6.88E-02	9.68E-02
**AL035706.1**	-2.53	6.88E-02	9.68E-02
**PHF5A**	0.31	6.89E-02	9.68E-02
**RP11-162G10.5**	-1.04	6.89E-02	9.68E-02
**KRTAP10-7**	-3.65	6.89E-02	9.69E-02
**EMILIN2**	0.68	6.89E-02	9.69E-02
**HNRNPLP1**	1.35	6.90E-02	9.69E-02
**SETP11**	-2.49	6.90E-02	9.70E-02
**RP11-374M1.2**	-1.06	6.91E-02	9.71E-02
**RNU6-761P**	-2.34	6.91E-02	9.71E-02
**MTND2P28**	3.28	6.91E-02	9.71E-02
**SLC23A3**	-1.23	6.92E-02	9.72E-02
**MIR1254-1**	-1.08	6.93E-02	9.73E-02
**G35143**	-2.21	6.93E-02	9.73E-02
**RP11-227L6.1**	-1.72	6.93E-02	9.73E-02
**C5orf56**	-0.61	6.93E-02	9.74E-02
**ZSCAN5A**	-0.25	6.93E-02	9.74E-02
**CLUHP6**	-2.68	6.93E-02	9.74E-02
**CYP8B1**	1.27	6.94E-02	9.74E-02
**NPHP1**	0.52	6.94E-02	9.75E-02
**LINC00205**	0.49	6.94E-02	9.75E-02
**ACSS2**	-0.51	6.94E-02	9.75E-02
**MIR4312**	-2.43	6.94E-02	9.75E-02
**IMMP2L**	-0.26	6.95E-02	9.76E-02
**SNX2**	0.28	6.95E-02	9.76E-02
**SH2D6**	-2.21	6.95E-02	9.77E-02
**RNA5SP372**	-2.61	6.96E-02	9.77E-02
**RP11-463I20.3**	-2.71	6.96E-02	9.78E-02
**RP11-304F15.4**	-0.93	6.96E-02	9.78E-02
**S1PR2**	0.30	6.96E-02	9.78E-02
**KB-226F1.2**	-1.19	6.97E-02	9.78E-02
**TMEM252**	-1.04	6.97E-02	9.78E-02

**RP5-839B4.8**	-2.17	6.97E-02	9.79E-02
**CTB-52I2.4**	-1.19	6.98E-02	9.79E-02
**CD22**	1.44	6.98E-02	9.80E-02
**OPTC**	-2.99	6.98E-02	9.80E-02
**LCN12**	-2.20	6.98E-02	9.80E-02
**TPK1**	0.56	6.99E-02	9.81E-02
**TTC36**	-1.20	6.99E-02	9.81E-02
**E2F3P1**	-2.57	7.00E-02	9.82E-02
**XLOC_006941**	1.68	7.00E-02	9.82E-02
**G2112**	-1.24	7.01E-02	9.83E-02
**FBXO16**	1.14	7.01E-02	9.84E-02
**RP11-150C16.1**	-0.80	7.01E-02	9.84E-02
**RNU6-1294P**	-2.37	7.01E-02	9.84E-02
**RARG**	-0.27	7.02E-02	9.85E-02
**ARSH**	1.33	7.02E-02	9.86E-02
**CLDN11**	-0.80	7.03E-02	9.87E-02
**TM7SF2**	0.41	7.03E-02	9.87E-02
**GRAMD3**	-0.24	7.03E-02	9.87E-02
**LRRC28**	-0.20	7.03E-02	9.87E-02
**RP11-135F9.4**	-0.79	7.04E-02	9.87E-02
**EIF3F**	0.21	7.04E-02	9.87E-02
**NBL1**	0.28	7.04E-02	9.87E-02
**XXYLT1-AS1**	-2.26	7.05E-02	9.89E-02
**RP11-107F6.3**	0.68	7.06E-02	9.90E-02
**JAKMIP2**	-0.78	7.06E-02	9.90E-02
**SAMD13**	-0.76	7.07E-02	9.91E-02
**GPRIN3**	0.54	7.07E-02	9.92E-02
**AL022341.3**	-1.20	7.08E-02	9.92E-02
**USP18**	0.69	7.08E-02	9.93E-02
**NARS**	-0.21	7.08E-02	9.93E-02
**RNU6-437P**	1.72	7.08E-02	9.93E-02
**XLOC_001286**	-1.33	7.09E-02	9.94E-02
**VTCN1**	1.29	7.09E-02	9.94E-02
**RRP1B**	-0.19	7.09E-02	9.94E-02
**CDR1**	-1.91	7.10E-02	9.95E-02
**CTA-345G4.1**	-2.79	7.10E-02	9.95E-02
**CYP2B6**	-3.47	7.10E-02	9.95E-02
**RP11-108K14.12**	-1.10	7.10E-02	9.95E-02
**G42176**	-1.61	7.10E-02	9.96E-02
**RP11-635L1.3**	-1.24	7.10E-02	9.96E-02
**TMEM196**	2.04	7.11E-02	9.96E-02
**STRCP1**	-0.96	7.11E-02	9.97E-02
**BNIP3P30**	-2.43	7.12E-02	9.98E-02
**ZBTB49**	0.31	7.12E-02	9.98E-02
**RARS**	-0.21	7.12E-02	9.98E-02

**EIF2D**	0.19	7.13E-02	9.98E-02
**CTC-297N7.1**	-2.53	7.13E-02	9.99E-02
**LINC01347**	0.93	7.13E-02	9.99E-02
**RP11-533E19.7**	-1.50	7.14E-02	1.00E-01
**GRM7**	-1.37	7.14E-02	1.00E-01
**LEXM**	-0.69	7.14E-02	1.00E-01
**RNU6-1251P**	-2.58	7.14E-02	1.00E-01
**H3F3AP1**	-2.57	7.15E-02	1.00E-01
**XXbac-BPG299F13.17**	0.90	7.15E-02	1.00E-01
**MIR652**	-2.64	7.15E-02	1.00E-01
**RP11-478B9.2**	-2.40	7.16E-02	1.00E-01
**G28013**	-2.49	7.16E-02	1.00E-01
**RP13-131K19.1**	-1.67	7.17E-02	1.00E-01
**P4HA2-AS1**	-1.64	7.18E-02	1.01E-01
**PQLC2L**	0.69	7.18E-02	1.01E-01
**FTLP3**	1.28	7.19E-02	1.01E-01
**NUP93**	-0.21	7.19E-02	1.01E-01
**FMO1**	0.99	7.20E-02	1.01E-01
**RP11-63G10.2**	-1.96	7.20E-02	1.01E-01
**G31022**	0.67	7.20E-02	1.01E-01
**RN7SL119P**	-1.47	7.21E-02	1.01E-01
**LINC01543**	-2.39	7.21E-02	1.01E-01
**RP11-117L5.4**	-2.20	7.22E-02	1.01E-01
**MIR568**	-0.74	7.22E-02	1.01E-01
**XLOC_013095**	2.16	7.22E-02	1.01E-01
**PYROXD2**	0.68	7.22E-02	1.01E-01
**RP11-338K17.5**	-0.87	7.22E-02	1.01E-01
**FAM66E**	-1.93	7.23E-02	1.01E-01
**RPL41P2**	-0.75	7.23E-02	1.01E-01
**FAM87B**	-0.82	7.23E-02	1.01E-01
**KDF1**	0.28	7.23E-02	1.01E-01
**RNU6-213P**	-2.29	7.24E-02	1.01E-01
**RP11-464F9.20**	-0.58	7.24E-02	1.01E-01
**TEX38**	-1.42	7.24E-02	1.01E-01
**XLOC_014010**	-0.99	7.26E-02	1.02E-01
**RMND1**	0.18	7.26E-02	1.02E-01
**ELMO1**	0.48	7.27E-02	1.02E-01
**AKR7A2P1**	-1.33	7.27E-02	1.02E-01
**RP11-665E10.2**	-2.68	7.27E-02	1.02E-01
**G826**	1.10	7.27E-02	1.02E-01
**STK38**	-0.23	7.28E-02	1.02E-01
**XLOC_008719**	-2.04	7.28E-02	1.02E-01
**RN7SL487P**	-2.35	7.28E-02	1.02E-01
**OCRL**	-0.15	7.28E-02	1.02E-01
**PRSS56**	-2.33	7.28E-02	1.02E-01

**HAMP**	1.08	7.29E-02	1.02E-01
**GTF2H3**	-0.35	7.29E-02	1.02E-01
**RP1-63M2.7**	-1.41	7.30E-02	1.02E-01
**TMEM59**	0.25	7.30E-02	1.02E-01
**AC002064.4**	-0.85	7.30E-02	1.02E-01
**AC007228.11**	-0.69	7.30E-02	1.02E-01
**LAMA2**	0.77	7.30E-02	1.02E-01
**RP11-680H20.1**	-0.99	7.31E-02	1.02E-01
**CTD-2293H3.2**	-1.40	7.31E-02	1.02E-01
**KB-1460A1.2**	-1.99	7.31E-02	1.02E-01
**ADO**	-0.20	7.31E-02	1.02E-01
**NKX2-5**	5.94	7.31E-02	1.02E-01
**RP5-1071N3.1**	-1.18	7.31E-02	1.02E-01
**NCKAP1L**	0.59	7.32E-02	1.02E-01
**KRTAP4-9**	-4.94	7.33E-02	1.02E-01
**TRDN**	-1.27	7.33E-02	1.02E-01
**TMC3-AS1**	-1.64	7.33E-02	1.02E-01
**PKP1**	0.37	7.33E-02	1.02E-01
**RN7SL288P**	-2.85	7.33E-02	1.02E-01
**AC005702.1**	1.06	7.33E-02	1.02E-01
**DACT3-AS1**	-1.10	7.34E-02	1.02E-01
**RP11-466F5.10**	-2.44	7.34E-02	1.02E-01
**AP000265.1**	-2.42	7.34E-02	1.03E-01
**RP11-713N11.4**	-2.40	7.34E-02	1.03E-01
**C16orf89**	1.34	7.35E-02	1.03E-01
**GTPBP1**	0.20	7.35E-02	1.03E-01
**RNU6-516P**	1.31	7.36E-02	1.03E-01
**RP11-798G7.5**	-1.26	7.36E-02	1.03E-01
**C1GALT1C1**	0.32	7.36E-02	1.03E-01
**HOXA1**	-0.38	7.36E-02	1.03E-01
**RNA5SP317**	-1.15	7.37E-02	1.03E-01
**CA8**	0.66	7.37E-02	1.03E-01
**RP11-59D5__B.2**	1.62	7.38E-02	1.03E-01
**NFIX**	0.37	7.38E-02	1.03E-01
**GDF6**	1.31	7.39E-02	1.03E-01
**AADACL4**	-2.40	7.39E-02	1.03E-01
**POSTN**	0.93	7.39E-02	1.03E-01
**PTEN**	-0.21	7.40E-02	1.03E-01
**SLFNL1-AS1**	-0.82	7.40E-02	1.03E-01
**CLDN10-AS1**	1.99	7.40E-02	1.03E-01
**API5**	-0.23	7.41E-02	1.03E-01
**RP11-173P15.10**	-1.40	7.42E-02	1.04E-01
**RP13-415G19.2**	-0.92	7.42E-02	1.04E-01
**RP11-274B21.5**	-1.67	7.42E-02	1.04E-01
**C2orf71**	-0.60	7.42E-02	1.04E-01

**RP11-119F7.5**	-0.71	7.42E-02	1.04E-01
**CCDC36**	0.68	7.42E-02	1.04E-01
**BMS1P11**	1.59	7.42E-02	1.04E-01
**AF064858.10**	-1.83	7.43E-02	1.04E-01
**CYP4F35P**	3.28	7.43E-02	1.04E-01
**WDR86-AS1**	1.68	7.43E-02	1.04E-01
**ZNF295-AS1**	-1.33	7.44E-02	1.04E-01
**PCAT1**	-0.63	7.44E-02	1.04E-01
**RP1-34B20.21**	-1.15	7.44E-02	1.04E-01
**AC007292.6**	-1.29	7.44E-02	1.04E-01
**AIMP1**	0.26	7.44E-02	1.04E-01
**RP11-671J11.5**	-1.68	7.45E-02	1.04E-01
**MCF2L**	-0.29	7.45E-02	1.04E-01
**XLOC_005371**	-2.52	7.46E-02	1.04E-01
**KCNG2**	0.83	7.46E-02	1.04E-01
**RP11-266K22.2**	-2.19	7.46E-02	1.04E-01
**RNU11-6P**	-2.27	7.46E-02	1.04E-01
**RN7SKP180**	-1.86	7.47E-02	1.04E-01
**TMEM128**	-0.23	7.47E-02	1.04E-01
**LINC00471**	-0.70	7.47E-02	1.04E-01
**VIM-AS1**	0.77	7.48E-02	1.04E-01
**KIFC2**	0.76	7.48E-02	1.04E-01
**ITGA8**	-0.61	7.48E-02	1.04E-01
**HIF1A-AS2**	-0.74	7.48E-02	1.04E-01
**EXO5**	0.42	7.48E-02	1.04E-01
**OARD1**	0.23	7.48E-02	1.04E-01
**RHEBL1**	-0.49	7.48E-02	1.04E-01
**S100A5**	-0.90	7.48E-02	1.04E-01
**PRKAR1AP**	-2.52	7.48E-02	1.04E-01
**XLOC_009740**	-1.12	7.49E-02	1.04E-01
**RP1-102G20.5**	-1.49	7.49E-02	1.04E-01
**CTB-131K11.1**	-0.21	7.49E-02	1.04E-01
**RP1-149A16.16**	-1.18	7.50E-02	1.04E-01
**ZFP37**	-0.47	7.50E-02	1.04E-01
**AC087650.1**	-3.84	7.51E-02	1.05E-01
**AF186192.5**	1.09	7.51E-02	1.05E-01
**RP11-1084A12.1**	-1.16	7.51E-02	1.05E-01
**RP11-1084J3.1**	-2.47	7.52E-02	1.05E-01
**BIRC3**	0.68	7.52E-02	1.05E-01
**PRR25**	-1.75	7.52E-02	1.05E-01
**MYZAP**	0.41	7.53E-02	1.05E-01
**RLN2**	-1.17	7.54E-02	1.05E-01
**MIR4512**	-2.32	7.55E-02	1.05E-01
**MNX1**	1.30	7.55E-02	1.05E-01
**RP11-713P17.4**	-1.53	7.55E-02	1.05E-01

**AC007228.9**	-0.76	7.56E-02	1.05E-01
**RNF126P1**	-1.90	7.56E-02	1.05E-01
**MST1R**	0.34	7.56E-02	1.05E-01
**YLPM1**	-0.22	7.56E-02	1.05E-01
**SLFN13**	0.55	7.57E-02	1.05E-01
**CEP83-AS1**	0.99	7.57E-02	1.05E-01
**LYZ**	0.88	7.57E-02	1.05E-01
**RP13-228J13.1**	-1.11	7.57E-02	1.05E-01
**RP5-867C24.1**	0.96	7.58E-02	1.06E-01
**KB-1836B5.1**	-0.90	7.58E-02	1.06E-01
**ANXA3**	0.72	7.60E-02	1.06E-01
**SDHAF4**	0.36	7.60E-02	1.06E-01
**RP11-345J4.5**	-2.16	7.60E-02	1.06E-01
**RP11-792A8.1**	-1.73	7.60E-02	1.06E-01
**XLOC_000986**	-2.11	7.60E-02	1.06E-01
**LINC01144**	0.51	7.61E-02	1.06E-01
**RP11-212I21.2**	1.52	7.61E-02	1.06E-01
**RUNX1T1**	0.64	7.61E-02	1.06E-01
**SLC47A2**	0.79	7.61E-02	1.06E-01
**H3F3C**	-1.56	7.62E-02	1.06E-01
**METTL22**	0.31	7.62E-02	1.06E-01
**RP11-216M21.1**	1.36	7.62E-02	1.06E-01
**SV2B**	-0.85	7.62E-02	1.06E-01
**RP11-307C19.1**	-1.79	7.63E-02	1.06E-01
**RP11-94A24.1**	1.42	7.63E-02	1.06E-01
**PPP2R5E**	-0.21	7.63E-02	1.06E-01
**RP11-46C24.7**	0.37	7.64E-02	1.06E-01
**FUT11**	0.36	7.64E-02	1.06E-01
**RP4-806M20.3**	-2.26	7.64E-02	1.06E-01
**RPL12P38**	-1.38	7.64E-02	1.06E-01
**RP11-669M16.1**	-1.21	7.65E-02	1.06E-01
**RN7SL821P**	-2.15	7.65E-02	1.06E-01
**RLTPR**	0.72	7.65E-02	1.06E-01
**A2M-AS1**	0.69	7.66E-02	1.07E-01
**RP11-43N16.4**	0.80	7.66E-02	1.07E-01
**THAP5P1**	-1.97	7.66E-02	1.07E-01
**RP11-428F8.2**	-2.31	7.67E-02	1.07E-01
**FTH1P16**	-1.33	7.67E-02	1.07E-01
**RP11-165A20.3**	-1.67	7.67E-02	1.07E-01
**RP11-20G13.1**	0.60	7.67E-02	1.07E-01
**METTL15**	-0.21	7.67E-02	1.07E-01
**AC011363.1**	-2.50	7.67E-02	1.07E-01
**G36839**	-0.75	7.68E-02	1.07E-01
**XLOC_014129**	1.33	7.69E-02	1.07E-01
**LA16c-321D4.2**	-1.26	7.69E-02	1.07E-01

**DNASE1**	-0.54	7.69E-02	1.07E-01
**RXFP4**	-2.05	7.70E-02	1.07E-01
**ZNF213-AS1**	-0.46	7.70E-02	1.07E-01
**SCN8A**	-0.67	7.70E-02	1.07E-01
**RP1-29C18.10**	-2.33	7.70E-02	1.07E-01
**AP000344.3**	-0.93	7.70E-02	1.07E-01
**TCTEX1D2**	-0.66	7.70E-02	1.07E-01
**SLA2**	-0.70	7.70E-02	1.07E-01
**XLOC_011150**	-1.42	7.71E-02	1.07E-01
**GRHL3**	0.36	7.71E-02	1.07E-01
**GRPEL2-AS1**	-2.36	7.71E-02	1.07E-01
**OAZ3**	0.71	7.72E-02	1.07E-01
**RNU7-171P**	-1.93	7.72E-02	1.07E-01
**CTD-2325P2.4**	-1.02	7.73E-02	1.07E-01
**RNU6-882P**	-1.08	7.73E-02	1.07E-01
**RP11-64C12.1**	-1.50	7.73E-02	1.07E-01
**MIR548L**	-2.32	7.73E-02	1.07E-01
**HMGN2P15**	1.04	7.74E-02	1.07E-01
**UQCC3**	0.28	7.74E-02	1.07E-01
**CTD-2525P14.5**	-0.88	7.74E-02	1.07E-01
**AC004510.3**	-2.24	7.74E-02	1.08E-01
**LCE6A**	0.68	7.75E-02	1.08E-01
**LSS**	0.29	7.75E-02	1.08E-01
**TUBGCP3**	-0.19	7.75E-02	1.08E-01
**RP11-259O2.3**	-1.39	7.75E-02	1.08E-01
**PARP4P1**	-2.17	7.75E-02	1.08E-01
**MPP4**	-0.92	7.76E-02	1.08E-01
**PSMG1**	0.29	7.76E-02	1.08E-01
**G19262**	2.21	7.76E-02	1.08E-01
**SNORD113-1**	-3.39	7.76E-02	1.08E-01
**FCGR2C**	-0.81	7.77E-02	1.08E-01
**RP11-930O11.1**	-1.48	7.77E-02	1.08E-01
**RP11-44N12.5**	-1.10	7.77E-02	1.08E-01
**CFH**	-0.71	7.77E-02	1.08E-01
**PDCD10**	-0.26	7.78E-02	1.08E-01
**HSPE1P3**	-1.75	7.79E-02	1.08E-01
**MARCO**	0.86	7.80E-02	1.08E-01
**G14986**	-1.44	7.80E-02	1.08E-01
**MYLK**	0.59	7.80E-02	1.08E-01
**RP1-90J20.8**	-1.77	7.80E-02	1.08E-01
**CYS1**	0.66	7.80E-02	1.08E-01
**RP11-359I18.5**	1.21	7.80E-02	1.08E-01
**CDIPT-AS1**	1.59	7.80E-02	1.08E-01
**IKZF1**	-0.59	7.80E-02	1.08E-01
**ATP1B3**	0.28	7.81E-02	1.08E-01

**G41823**	-0.47	7.82E-02	1.08E-01
**RP11-567O16.1**	-2.41	7.82E-02	1.08E-01
**PEX11A**	-0.47	7.82E-02	1.08E-01
**TRIM9**	-0.58	7.82E-02	1.09E-01
**RP11-20I23.2**	-0.78	7.82E-02	1.09E-01
**DLG5-AS1**	-0.39	7.83E-02	1.09E-01
**STK3**	-0.18	7.83E-02	1.09E-01
**RP11-195E2.1**	-2.01	7.83E-02	1.09E-01
**LINC00863**	0.46	7.83E-02	1.09E-01
**PIGV**	0.22	7.83E-02	1.09E-01
**AC009228.1**	-2.09	7.83E-02	1.09E-01
**ASUN**	0.25	7.84E-02	1.09E-01
**DPYSL2**	-0.40	7.84E-02	1.09E-01
**RP1-93H18.1**	-0.42	7.84E-02	1.09E-01
**MRPS27**	-0.19	7.85E-02	1.09E-01
**G43605**	2.14	7.85E-02	1.09E-01
**KRTAP2-2**	-4.32	7.85E-02	1.09E-01
**LIMS1-AS1**	-0.98	7.86E-02	1.09E-01
**RP3-322G13.7**	-1.24	7.87E-02	1.09E-01
**RPL12P35**	-1.71	7.88E-02	1.09E-01
**IQCC**	-0.30	7.88E-02	1.09E-01
**HNMT**	0.50	7.89E-02	1.09E-01
**KANSL1**	-0.29	7.89E-02	1.09E-01
**RP11-471B22.2**	-0.69	7.90E-02	1.10E-01
**RP1-159A19.4**	-1.30	7.91E-02	1.10E-01
**PHF20**	0.17	7.91E-02	1.10E-01
**RN7SL230P**	-1.62	7.91E-02	1.10E-01
**RP11-356K23.2**	-1.40	7.91E-02	1.10E-01
**CDX4**	-0.94	7.91E-02	1.10E-01
**RAG1**	0.58	7.91E-02	1.10E-01
**G14548**	0.98	7.92E-02	1.10E-01
**FAM157C**	-1.17	7.92E-02	1.10E-01
**RP5-844F9.1**	-1.71	7.93E-02	1.10E-01
**CTD-2215L10.1**	-2.13	7.93E-02	1.10E-01
**USH1G**	0.56	7.93E-02	1.10E-01
**LINC01579**	1.35	7.93E-02	1.10E-01
**MUC19**	-2.06	7.93E-02	1.10E-01
**NAT8**	-2.28	7.93E-02	1.10E-01
**BRI3BPP1**	-1.76	7.93E-02	1.10E-01
**RNU6-742P**	-2.36	7.93E-02	1.10E-01
**RP11-981G7.3**	-2.06	7.94E-02	1.10E-01
**HEXDC-IT1**	1.09	7.94E-02	1.10E-01
**SMIM14**	0.31	7.94E-02	1.10E-01
**RP11-74C13.3**	-2.30	7.94E-02	1.10E-01
**AC098820.3**	-1.13	7.94E-02	1.10E-01

**MRGPRF-AS1**	1.47	7.94E-02	1.10E-01
**KIF9-AS1**	0.56	7.95E-02	1.10E-01
**RP11-526I2.5**	-0.88	7.97E-02	1.10E-01
**B3GLCT**	0.34	7.97E-02	1.10E-01
**G18316**	1.08	7.97E-02	1.10E-01
**ARHGEF9**	-0.19	7.99E-02	1.11E-01
**CLEC4F**	-1.18	7.99E-02	1.11E-01
**FOSL1P1**	-2.39	7.99E-02	1.11E-01
**DUSP3**	0.16	7.99E-02	1.11E-01
**RP11-617F23.2**	-0.73	8.00E-02	1.11E-01
**G26976**	-1.48	8.00E-02	1.11E-01
**RNU6-623P**	-2.32	8.00E-02	1.11E-01
**PIK3CD-AS1**	-1.69	8.01E-02	1.11E-01
**LINC00092**	0.78	8.01E-02	1.11E-01
**DNAJC10**	-0.25	8.02E-02	1.11E-01
**HRAT92**	-0.97	8.02E-02	1.11E-01
**TBC1D20**	0.16	8.02E-02	1.11E-01
**GDF10**	1.10	8.02E-02	1.11E-01
**CNTN4-AS2**	-1.93	8.03E-02	1.11E-01
**XLOC_004107**	1.14	8.04E-02	1.11E-01
**LINC01625**	-0.70	8.04E-02	1.11E-01
**PPP1R3E**	-0.37	8.04E-02	1.11E-01
**ZBTB12P1**	-1.21	8.04E-02	1.11E-01
**AC007365.1**	-1.33	8.04E-02	1.11E-01
**SLC5A12**	1.90	8.05E-02	1.11E-01
**FBXO18**	0.14	8.05E-02	1.11E-01
**G33524**	-1.53	8.05E-02	1.11E-01
**PUS10**	-0.26	8.06E-02	1.11E-01
**LINC00884**	0.98	8.06E-02	1.11E-01
**AC004988.1**	1.60	8.07E-02	1.12E-01
**KB-1458E12.1**	-2.10	8.07E-02	1.12E-01
**ADAMTSL1**	0.98	8.08E-02	1.12E-01
**RP5-906C1.1**	-1.34	8.08E-02	1.12E-01
**TGM3**	0.50	8.09E-02	1.12E-01
**KRT8P39**	-0.69	8.09E-02	1.12E-01
**TMSB15A**	1.49	8.10E-02	1.12E-01
**USP22**	-0.14	8.10E-02	1.12E-01
**SNRPGP15**	-0.74	8.11E-02	1.12E-01
**GABPB1-AS1**	-0.60	8.11E-02	1.12E-01
**GPR55**	-0.93	8.11E-02	1.12E-01
**RPS8**	0.29	8.11E-02	1.12E-01
**XLOC_007805**	-2.10	8.11E-02	1.12E-01
**LINC00543**	2.18	8.12E-02	1.12E-01
**RP11-171I2.3**	-1.82	8.12E-02	1.12E-01
**FAUP1**	-1.14	8.12E-02	1.12E-01

**SKA3**	-0.35	8.12E-02	1.12E-01
**DCDC2**	1.23	8.13E-02	1.12E-01
**PANK2**	0.16	8.13E-02	1.12E-01
**RP11-1334A24.6**	1.47	8.15E-02	1.13E-01
**TMEM60**	0.27	8.15E-02	1.13E-01
**ZDHHC3**	-0.19	8.15E-02	1.13E-01
**DUSP4**	0.55	8.17E-02	1.13E-01
**C22orf39**	-0.23	8.17E-02	1.13E-01
**EFTUD1P1**	-2.37	8.17E-02	1.13E-01
**TEKT2**	1.23	8.18E-02	1.13E-01
**EGLN3**	0.41	8.18E-02	1.13E-01
**SPATS2**	0.24	8.20E-02	1.13E-01
**PTPRF**	0.30	8.20E-02	1.13E-01
**PRKG1-AS1**	-1.77	8.21E-02	1.13E-01
**IL1RN**	-0.30	8.21E-02	1.13E-01
**ONECUT2**	-1.79	8.22E-02	1.13E-01
**IL23A**	0.81	8.22E-02	1.14E-01
**RPL23AP92**	-2.51	8.24E-02	1.14E-01
**LINC00675**	-0.68	8.24E-02	1.14E-01
**IFIT1**	0.64	8.25E-02	1.14E-01
**ZNF788**	-0.51	8.25E-02	1.14E-01
**CWC15**	0.24	8.26E-02	1.14E-01
**ZNF597**	-0.27	8.26E-02	1.14E-01
**RP11-141C7.5**	-1.29	8.26E-02	1.14E-01
**RWDD2B**	0.31	8.26E-02	1.14E-01
**CTB-111F10.1**	-2.87	8.26E-02	1.14E-01
**RP11-57C19.6**	1.42	8.26E-02	1.14E-01
**DIAPH3**	-0.40	8.27E-02	1.14E-01
**RP11-782C8.7**	-2.58	8.27E-02	1.14E-01
**LINC00957**	0.45	8.28E-02	1.14E-01
**FLJ20306**	-0.79	8.28E-02	1.14E-01
**C8orf74**	0.54	8.29E-02	1.14E-01
**CLINT1**	-0.19	8.29E-02	1.14E-01
**CTD-3214H19.6**	-0.98	8.29E-02	1.14E-01
**DTX4**	-0.60	8.29E-02	1.14E-01
**MZT1**	-0.38	8.29E-02	1.14E-01
**EIF3H**	0.24	8.30E-02	1.14E-01
**RAB43P1**	1.16	8.30E-02	1.14E-01
**RP11-3D4.3**	-0.79	8.30E-02	1.15E-01
**ZNF804A**	1.18	8.30E-02	1.15E-01
**RP11-349F21.5**	-2.41	8.31E-02	1.15E-01
**RAD51-AS1**	-0.51	8.31E-02	1.15E-01
**TEX41**	-0.70	8.31E-02	1.15E-01
**CTC-340I23.2**	-2.76	8.32E-02	1.15E-01
**ZNF3**	0.16	8.32E-02	1.15E-01

**DTNA**	0.51	8.33E-02	1.15E-01
**RTKN2**	-0.56	8.33E-02	1.15E-01
**LRFN2**	1.18	8.34E-02	1.15E-01
**RRAGB**	-0.22	8.34E-02	1.15E-01
**RP11-243M5.3**	-2.65	8.35E-02	1.15E-01
**ARPC3P5**	-1.83	8.35E-02	1.15E-01
**PRMT6**	0.34	8.35E-02	1.15E-01
**MSX2**	0.40	8.35E-02	1.15E-01
**C14orf93**	0.22	8.36E-02	1.15E-01
**PPP1R14C**	-0.34	8.36E-02	1.15E-01
**RNU6-1297P**	-2.71	8.36E-02	1.15E-01
**G37296**	0.59	8.36E-02	1.15E-01
**KRTAP4-6**	-4.10	8.36E-02	1.15E-01
**NDUFV3**	-0.27	8.37E-02	1.15E-01
**RP11-410L14.2**	0.62	8.37E-02	1.15E-01
**RP11-401F2.3**	1.72	8.38E-02	1.15E-01
**RP11-156E8.1**	-0.80	8.38E-02	1.15E-01
**RNF112**	0.78	8.38E-02	1.16E-01
**ACTBL2**	-3.57	8.38E-02	1.16E-01
**ILKAP**	0.20	8.39E-02	1.16E-01
**SLC13A5**	-0.92	8.40E-02	1.16E-01
**GJB5**	-0.35	8.40E-02	1.16E-01
**CYP4F26P**	-0.70	8.41E-02	1.16E-01
**CASC15**	0.54	8.41E-02	1.16E-01
**ZNF385D**	-0.61	8.41E-02	1.16E-01
**MGC27382**	1.78	8.41E-02	1.16E-01
**MYPN**	-1.15	8.41E-02	1.16E-01
**EIF4A1P2**	-1.39	8.42E-02	1.16E-01
**AC007405.4**	-1.64	8.42E-02	1.16E-01
**EXOC5P1**	-1.99	8.42E-02	1.16E-01
**RP11-1C8.5**	-2.29	8.43E-02	1.16E-01
**MIR4706**	-2.31	8.43E-02	1.16E-01
**RPL29P12**	-1.40	8.43E-02	1.16E-01
**WDR45BP1**	-2.25	8.43E-02	1.16E-01
**RP11-486L19.2**	-0.76	8.44E-02	1.16E-01
**RCC2**	-0.18	8.44E-02	1.16E-01
**NEAT1**	-0.92	8.45E-02	1.16E-01
**CDK5RAP2**	-0.16	8.45E-02	1.16E-01
**CRYBB2P1**	-0.41	8.45E-02	1.16E-01
**GRIA3**	0.82	8.46E-02	1.16E-01
**RP4-714D9.4**	-2.40	8.46E-02	1.17E-01
**RP11-867G23.3**	-0.91	8.47E-02	1.17E-01
**TLX2**	-1.35	8.48E-02	1.17E-01
**CTC-344H19.4**	-2.37	8.48E-02	1.17E-01
**CTC-378H22.2**	1.56	8.48E-02	1.17E-01

**NFASC**	-0.38	8.49E-02	1.17E-01
**CTB-66B24.1**	-1.61	8.49E-02	1.17E-01
**LINC00276**	-2.48	8.50E-02	1.17E-01
**FUT8-AS1**	1.00	8.50E-02	1.17E-01
**AK4P4**	-1.64	8.50E-02	1.17E-01
**SNORD38A**	-3.05	8.51E-02	1.17E-01
**MICD**	-1.80	8.51E-02	1.17E-01
**RP11-203F10.6**	-1.75	8.51E-02	1.17E-01
**RP6-191P20.4**	-1.00	8.52E-02	1.17E-01
**TPM3P6**	-0.70	8.52E-02	1.17E-01
**RP1-232P20.1**	0.57	8.52E-02	1.17E-01
**RP3-428L16.2**	0.48	8.52E-02	1.17E-01
**C2CD4A**	0.87	8.53E-02	1.17E-01
**CTD-3020H12.4**	-0.98	8.54E-02	1.17E-01
**RP11-181G12.2**	0.89	8.54E-02	1.17E-01
**PCA3**	-1.35	8.54E-02	1.17E-01
**LRRN1**	-0.61	8.55E-02	1.18E-01
**RP11-253M7.3**	-2.15	8.55E-02	1.18E-01
**RP11-855A2.5**	-1.61	8.55E-02	1.18E-01
**RP11-1260E13.3**	-2.17	8.55E-02	1.18E-01
**RP1-45C12.1**	1.07	8.57E-02	1.18E-01
**IL15**	0.60	8.57E-02	1.18E-01
**CD8BP**	-1.28	8.57E-02	1.18E-01
**CTB-50L17.14**	-1.98	8.58E-02	1.18E-01
**AC016739.2**	0.98	8.58E-02	1.18E-01
**RP11-379F4.9**	-1.26	8.58E-02	1.18E-01
**AC012146.7**	-0.96	8.59E-02	1.18E-01
**STEAP3-AS1**	0.88	8.59E-02	1.18E-01
**TMCC1-AS1**	-0.70	8.60E-02	1.18E-01
**RP11-667K14.5**	-2.12	8.60E-02	1.18E-01
**HNRNPH2**	0.30	8.61E-02	1.18E-01
**RBMS3-AS3**	-1.40	8.61E-02	1.18E-01
**DDX11**	0.57	8.61E-02	1.18E-01
**RP11-223A3.1**	1.10	8.62E-02	1.18E-01
**PCSK7**	0.31	8.63E-02	1.19E-01
**GTF2F2P1**	-2.14	8.63E-02	1.19E-01
**TSPAN8**	-0.82	8.64E-02	1.19E-01
**LZTS1-AS1**	-1.92	8.64E-02	1.19E-01
**SRSF7**	0.28	8.64E-02	1.19E-01
**HPS3**	-0.19	8.65E-02	1.19E-01
**AMD1P3**	-2.27	8.66E-02	1.19E-01
**AC074117.13**	-2.10	8.66E-02	1.19E-01
**GUCA1B**	0.73	8.66E-02	1.19E-01
**AL353662.2**	-2.39	8.66E-02	1.19E-01
**RP1-99E18.2**	-1.72	8.67E-02	1.19E-01

**RP13-20L14.6**	0.55	8.67E-02	1.19E-01
**OR2B8P**	-2.66	8.68E-02	1.19E-01
**DHRS11**	0.29	8.68E-02	1.19E-01
**INCENP**	0.21	8.68E-02	1.19E-01
**ADCY10**	-0.83	8.68E-02	1.19E-01
**DCTN5**	0.16	8.68E-02	1.19E-01
**MUC5B**	0.98	8.69E-02	1.19E-01
**RP3-426I6.6**	-1.07	8.69E-02	1.19E-01
**WDR20**	-0.13	8.69E-02	1.19E-01
**G674**	1.58	8.70E-02	1.19E-01
**MAT2A**	-0.43	8.70E-02	1.20E-01
**ADSS**	-0.23	8.71E-02	1.20E-01
**ZMYND12**	0.56	8.71E-02	1.20E-01
**SCOC-AS1**	-0.56	8.71E-02	1.20E-01
**RP11-247L20.3**	1.75	8.72E-02	1.20E-01
**KRTAP9-9**	-4.47	8.72E-02	1.20E-01
**STAG3L4**	-0.37	8.72E-02	1.20E-01
**MTND2P26**	-2.08	8.72E-02	1.20E-01
**PWRN1**	-2.61	8.72E-02	1.20E-01
**RP11-526I2.1**	-1.72	8.73E-02	1.20E-01
**RNU6-1231P**	-1.71	8.73E-02	1.20E-01
**RP11-319E16.1**	-2.29	8.73E-02	1.20E-01
**RP13-33H18.1**	-1.05	8.73E-02	1.20E-01
**DBNDD2**	-0.60	8.74E-02	1.20E-01
**RP11-155O18.6**	-1.02	8.74E-02	1.20E-01
**IFIT2**	0.41	8.74E-02	1.20E-01
**RP11-528A10.1**	-1.79	8.74E-02	1.20E-01
**C5orf58**	-1.29	8.75E-02	1.20E-01
**AC118138.2**	-1.54	8.75E-02	1.20E-01
**RP1-149A16.17**	-1.19	8.76E-02	1.20E-01
**PSD4**	-0.28	8.76E-02	1.20E-01
**MPHOSPH6**	0.29	8.76E-02	1.20E-01
**RFFL**	0.22	8.76E-02	1.20E-01
**AC011551.3**	-1.37	8.76E-02	1.20E-01
**DDN**	1.21	8.77E-02	1.20E-01
**RPL13AP7**	-1.27	8.78E-02	1.20E-01
**RP11-2H3.6**	-1.84	8.78E-02	1.20E-01
**NECAB1**	-0.45	8.78E-02	1.21E-01
**CTD-2236F14.1**	-2.15	8.79E-02	1.21E-01
**DDX19A**	0.19	8.79E-02	1.21E-01
**TSTD1**	-0.34	8.79E-02	1.21E-01
**RP11-697N18.3**	-0.83	8.79E-02	1.21E-01
**RP11-552F3.9**	0.76	8.79E-02	1.21E-01
**TTC39A**	0.35	8.79E-02	1.21E-01
**FLJ42969**	1.36	8.80E-02	1.21E-01

**ANKRD45**	1.23	8.80E-02	1.21E-01
**AC007277.3**	-1.50	8.81E-02	1.21E-01
**RP11-89K10.1**	-0.89	8.81E-02	1.21E-01
**CCDC91**	-0.22	8.81E-02	1.21E-01
**KRTAP4-4**	-3.90	8.82E-02	1.21E-01
**RP3-525N10.2**	-1.59	8.82E-02	1.21E-01
**PIK3CD-AS2**	0.83	8.82E-02	1.21E-01
**RP11-392P7.6**	-0.46	8.82E-02	1.21E-01
**KRT78**	0.52	8.82E-02	1.21E-01
**XLOC_007725**	-0.95	8.82E-02	1.21E-01
**AC074391.1**	1.67	8.83E-02	1.21E-01
**ASS1P10**	-1.31	8.84E-02	1.21E-01
**RPS23P1**	-1.74	8.84E-02	1.21E-01
**AC005083.1**	-0.62	8.84E-02	1.21E-01
**AL353626.1**	-2.56	8.85E-02	1.21E-01
**RTCA-AS1**	-0.61	8.86E-02	1.21E-01
**TMX1**	-0.30	8.86E-02	1.21E-01
**XXyac-YX65C7_A.3**	-1.99	8.86E-02	1.21E-01
**TMEM38B**	-0.40	8.86E-02	1.21E-01
**PRDM2**	0.19	8.87E-02	1.22E-01
**KCTD19**	-1.00	8.88E-02	1.22E-01
**CYB561D1**	-0.26	8.88E-02	1.22E-01
**RP11-61L19.2**	0.68	8.88E-02	1.22E-01
**KPNA2**	0.37	8.88E-02	1.22E-01
**RP11-429P3.5**	-1.37	8.89E-02	1.22E-01
**MUM1L1**	-1.39	8.89E-02	1.22E-01
**STAG3L5P-PVRIG2P-PILRE**	-2.17	8.89E-02	1.22E-01
**MIR200A**	-1.38	8.90E-02	1.22E-01
**G32522**	1.65	8.91E-02	1.22E-01
**CDK8**	-0.20	8.91E-02	1.22E-01
**SPRY4-IT1**	1.64	8.92E-02	1.22E-01
**SH3GLB2**	0.31	8.92E-02	1.22E-01
**SATL1**	-2.27	8.93E-02	1.22E-01
**XLOC_010453**	-1.64	8.93E-02	1.22E-01
**BCAT1**	0.75	8.94E-02	1.22E-01
**SLC16A6P1**	-1.93	8.94E-02	1.22E-01
**ANOS1**	-0.44	8.94E-02	1.22E-01
**MRPS18C**	-0.24	8.94E-02	1.22E-01
**RP4-800F24.1**	-1.98	8.95E-02	1.22E-01
**AC114765.1**	-1.81	8.95E-02	1.23E-01
**IGHEP2**	-1.06	8.96E-02	1.23E-01
**CTD-2012J19.2**	-1.29	8.96E-02	1.23E-01
**LAT**	1.22	8.96E-02	1.23E-01
**ZNF271P**	0.26	8.96E-02	1.23E-01
**PLEKHH2**	-0.43	8.96E-02	1.23E-01

**PSMB8-AS1**	0.42	8.97E-02	1.23E-01
**RNU6-529P**	0.68	8.97E-02	1.23E-01
**RP11-753B14.1**	-2.60	8.97E-02	1.23E-01
**XLOC_007420**	-1.95	8.97E-02	1.23E-01
**PPP4R1-AS1**	-1.15	8.97E-02	1.23E-01
**GID8**	0.21	8.97E-02	1.23E-01
**DDTL**	0.61	8.97E-02	1.23E-01
**NEIL2**	0.27	8.97E-02	1.23E-01
**RP11-686D22.10**	1.74	8.98E-02	1.23E-01
**G4674**	1.21	8.99E-02	1.23E-01
**NUPL2**	0.18	8.99E-02	1.23E-01
**AP000253.1**	-1.08	9.00E-02	1.23E-01
**PROSER2-AS1**	-0.87	9.00E-02	1.23E-01
**LAX1**	-0.83	9.00E-02	1.23E-01
**TMCO6**	0.30	9.00E-02	1.23E-01
**CTC-232P5.1**	-0.82	9.00E-02	1.23E-01
**RP11-324I22.3**	1.53	9.02E-02	1.23E-01
**AC073130.1**	-1.55	9.02E-02	1.23E-01
**ALG8**	-0.24	9.02E-02	1.23E-01
**HERPUD1**	0.22	9.03E-02	1.23E-01
**RP6-191P20.3**	-2.01	9.03E-02	1.23E-01
**G25251**	3.52	9.03E-02	1.24E-01
**NCR3**	1.39	9.04E-02	1.24E-01
**RP11-268J15.5**	0.85	9.04E-02	1.24E-01
**MTFP1**	1.04	9.04E-02	1.24E-01
**IGKV4-1**	1.77	9.06E-02	1.24E-01
**CDH23**	-0.48	9.06E-02	1.24E-01
**AC093627.9**	-0.44	9.06E-02	1.24E-01
**RP11-182J1.5**	-2.36	9.07E-02	1.24E-01
**RP3-405J10.4**	-1.41	9.07E-02	1.24E-01
**P4HTM**	0.35	9.09E-02	1.24E-01
**AC002398.13**	1.50	9.10E-02	1.24E-01
**RP11-639E23.1**	-1.80	9.10E-02	1.24E-01
**AC005229.1**	0.40	9.10E-02	1.24E-01
**RBM14**	0.31	9.11E-02	1.24E-01
**IGLV3-21**	-2.85	9.12E-02	1.25E-01
**AL133493.2**	1.32	9.12E-02	1.25E-01
**RP11-276H7.3**	1.02	9.12E-02	1.25E-01
**CDH17**	1.20	9.12E-02	1.25E-01
**MSL3P1**	1.47	9.13E-02	1.25E-01
**ITLN2**	1.37	9.13E-02	1.25E-01
**ARL14**	1.84	9.13E-02	1.25E-01
**TDRD5**	-0.87	9.13E-02	1.25E-01
**XXcos-LUCA16.1**	-1.26	9.14E-02	1.25E-01
**VEZF1**	-0.18	9.14E-02	1.25E-01

**MIR589**	-1.34	9.14E-02	1.25E-01
**AC018712.2**	-2.34	9.15E-02	1.25E-01
**ATP5F1P5**	-0.59	9.15E-02	1.25E-01
**AC007405.6**	0.99	9.15E-02	1.25E-01
**ASLP1**	1.10	9.16E-02	1.25E-01
**AC004691.5**	-2.07	9.16E-02	1.25E-01
**UBXN2A**	-0.19	9.16E-02	1.25E-01
**LINC00342**	-0.55	9.16E-02	1.25E-01
**NR2E1**	1.61	9.17E-02	1.25E-01
**ZNF18**	-0.19	9.17E-02	1.25E-01
**AP000432.1**	-0.43	9.18E-02	1.25E-01
**RP11-814H16.2**	-2.41	9.19E-02	1.25E-01
**RP11-339B21.10**	1.48	9.19E-02	1.25E-01
**G15105**	-2.05	9.20E-02	1.26E-01
**RP11-156P1.3**	-0.33	9.20E-02	1.26E-01
**RP5-1056L3.3**	-0.77	9.20E-02	1.26E-01
**GLI4**	0.43	9.20E-02	1.26E-01
**MOCS3**	0.18	9.21E-02	1.26E-01
**RP11-57A19.2**	-1.82	9.21E-02	1.26E-01
**GP1BA**	-0.60	9.21E-02	1.26E-01
**CRIPT**	0.29	9.21E-02	1.26E-01
**C6orf223**	1.17	9.22E-02	1.26E-01
**RP11-368P15.1**	-2.68	9.22E-02	1.26E-01
**AC007204.1**	-2.15	9.22E-02	1.26E-01
**UPF3BP3**	1.16	9.22E-02	1.26E-01
**RP11-168J18.6**	-1.55	9.23E-02	1.26E-01
**KRTAP4-12**	-4.62	9.23E-02	1.26E-01
**RP11-624C23.1**	-1.96	9.24E-02	1.26E-01
**NEXN-AS1**	0.93	9.24E-02	1.26E-01
**RAB33A**	0.87	9.24E-02	1.26E-01
**G38698**	1.03	9.24E-02	1.26E-01
**RP11-288A5.2**	-1.62	9.25E-02	1.26E-01
**HOOK1**	-0.34	9.25E-02	1.26E-01
**UBE2N**	0.27	9.26E-02	1.26E-01
**RP11-88H9.2**	0.49	9.26E-02	1.26E-01
**RP11-426C22.1**	-1.64	9.26E-02	1.26E-01
**PRKACB**	0.34	9.26E-02	1.26E-01
**RP11-110I1.14**	-1.35	9.27E-02	1.26E-01
**RP11-305L7.1**	1.73	9.27E-02	1.26E-01
**AC009095.4**	-0.76	9.27E-02	1.26E-01
**G39662**	1.27	9.27E-02	1.26E-01
**RP11-111M22.2**	-0.64	9.28E-02	1.27E-01
**CCL1**	-2.58	9.28E-02	1.27E-01
**RINL**	0.46	9.29E-02	1.27E-01
**RNU2-33P**	-2.11	9.30E-02	1.27E-01

**GSTA1**	1.97	9.30E-02	1.27E-01
**RP11-203I2.1**	-0.92	9.30E-02	1.27E-01
**RP11-373N22.3**	-1.17	9.31E-02	1.27E-01
**RP11-314N13.3**	-0.91	9.31E-02	1.27E-01
**LA16c-312E8.2**	-1.90	9.31E-02	1.27E-01
**FKBP1C**	1.14	9.31E-02	1.27E-01
**FUNDC1**	0.30	9.32E-02	1.27E-01
**INSL3**	1.62	9.32E-02	1.27E-01
**MAPRE2**	-0.24	9.32E-02	1.27E-01
**AC008686.1**	-1.40	9.32E-02	1.27E-01
**U82695.9**	-1.39	9.32E-02	1.27E-01
**KERA**	1.42	9.32E-02	1.27E-01
**AP000351.10**	-1.44	9.32E-02	1.27E-01
**DYNC1I1**	-0.34	9.32E-02	1.27E-01
**SEC22A**	-0.18	9.33E-02	1.27E-01
**HSF4**	0.75	9.33E-02	1.27E-01
**C9orf106**	-0.83	9.33E-02	1.27E-01
**RP1-197B17.7**	-1.08	9.33E-02	1.27E-01
**ZNF19**	-0.32	9.34E-02	1.27E-01
**DNAH1**	-0.53	9.34E-02	1.27E-01
**AC008697.1**	-1.18	9.35E-02	1.27E-01
**G30338**	2.18	9.35E-02	1.27E-01
**OSTM1**	0.28	9.35E-02	1.27E-01
**CCDC191**	-0.33	9.35E-02	1.27E-01
**FAM189A1**	1.13	9.36E-02	1.27E-01
**AC092159.2**	-1.33	9.36E-02	1.27E-01
**HCFC1-AS1**	-2.23	9.36E-02	1.27E-01
**AC004000.1**	2.42	9.36E-02	1.27E-01
**LA16c-360A4.1**	-1.48	9.36E-02	1.28E-01
**RPS3AP40**	-2.39	9.38E-02	1.28E-01
**UTS2**	-2.10	9.38E-02	1.28E-01
**CTD-3179P9.1**	-2.10	9.39E-02	1.28E-01
**RPTN**	0.60	9.40E-02	1.28E-01
**CTD-2184D3.7**	-1.31	9.40E-02	1.28E-01
**RNU6-272P**	-2.32	9.40E-02	1.28E-01
**SLC18B1**	0.21	9.40E-02	1.28E-01
**GJA3**	0.70	9.41E-02	1.28E-01
**ELOVL5**	-1.09	9.41E-02	1.28E-01
**G22807**	0.73	9.41E-02	1.28E-01
**ARL9**	0.48	9.41E-02	1.28E-01
**C8orf31**	1.10	9.41E-02	1.28E-01
**RP11-290D2.3**	-0.75	9.42E-02	1.28E-01
**NEK1**	-0.23	9.42E-02	1.28E-01
**GOT2P3**	-1.47	9.42E-02	1.28E-01
**G23125**	-0.91	9.42E-02	1.28E-01

**CTA-113A6.2**	-2.14	9.43E-02	1.28E-01
**NAP1L5**	0.39	9.43E-02	1.28E-01
**CTB-49A3.4**	-1.94	9.43E-02	1.28E-01
**BCL2L13**	-0.19	9.43E-02	1.28E-01
**RP11-977B10.2**	1.00	9.43E-02	1.28E-01
**BPHL**	0.23	9.43E-02	1.28E-01
**HILS1**	-0.86	9.44E-02	1.28E-01
**SFTA1P**	2.67	9.44E-02	1.28E-01
**SUPT4H1**	0.22	9.45E-02	1.28E-01
**SCNN1D**	0.71	9.45E-02	1.29E-01
**RPL23AP87**	-1.38	9.45E-02	1.29E-01
**RP11-425D17.1**	-1.28	9.45E-02	1.29E-01
**RP11-295H24.5**	-1.38	9.45E-02	1.29E-01
**CRIP3**	-1.30	9.46E-02	1.29E-01
**PCBP1-AS1**	-0.33	9.46E-02	1.29E-01
**TGFBR1**	0.32	9.47E-02	1.29E-01
**ZNF75A**	-0.27	9.47E-02	1.29E-01
**AC159540.1**	0.76	9.47E-02	1.29E-01
**SLC25A5-AS1**	-0.42	9.48E-02	1.29E-01
**CTD-2529O21.1**	-1.63	9.48E-02	1.29E-01
**CTAGE15**	-1.96	9.49E-02	1.29E-01
**XLOC_013995**	-1.17	9.49E-02	1.29E-01
**SLC22A20**	0.76	9.50E-02	1.29E-01
**VAV3**	-0.35	9.50E-02	1.29E-01
**GFI1**	0.77	9.50E-02	1.29E-01
**SNX9**	0.23	9.50E-02	1.29E-01
**RP11-295P9.6**	-1.75	9.51E-02	1.29E-01
**CTD-2307P3.1**	-2.16	9.51E-02	1.29E-01
**TIAL1**	0.21	9.51E-02	1.29E-01
**CFP**	-0.55	9.52E-02	1.29E-01
**GPR149**	-1.67	9.52E-02	1.29E-01
**BNC2**	-0.44	9.52E-02	1.29E-01
**RP11-440I14.2**	-1.09	9.52E-02	1.29E-01
**HHAT**	0.27	9.52E-02	1.29E-01
**AC104777.4**	-2.26	9.53E-02	1.29E-01
**ATP6V0C**	1.07	9.53E-02	1.29E-01
**RP11-327F22.2**	-1.40	9.53E-02	1.30E-01
**CNKSR3**	-0.26	9.54E-02	1.30E-01
**CSGALNACT2**	0.30	9.55E-02	1.30E-01
**MIR5699**	-2.33	9.56E-02	1.30E-01
**BOLA2B**	0.69	9.56E-02	1.30E-01
**AL713999.1**	1.46	9.56E-02	1.30E-01
**TNNT1**	0.53	9.56E-02	1.30E-01
**XLOC_006637**	2.43	9.57E-02	1.30E-01
**TRAF5**	0.38	9.57E-02	1.30E-01

**MPEG1**	0.69	9.57E-02	1.30E-01
**CASP8**	-0.20	9.57E-02	1.30E-01
**HMCN2**	0.61	9.58E-02	1.30E-01
**HS3ST2**	0.87	9.59E-02	1.30E-01
**AC079781.8**	-1.24	9.59E-02	1.30E-01
**SLC25A12**	-0.20	9.59E-02	1.30E-01
**ASAH2**	-0.70	9.59E-02	1.30E-01
**CD83**	-0.51	9.60E-02	1.30E-01
**AC016894.1**	-1.48	9.61E-02	1.31E-01
**RP11-16O9.2**	-2.21	9.61E-02	1.31E-01
**BSNDP2**	-2.14	9.62E-02	1.31E-01
**CAMLG**	0.23	9.62E-02	1.31E-01
**G34382**	1.53	9.63E-02	1.31E-01
**RAB8A**	0.16	9.63E-02	1.31E-01
**RP1-267L14.6**	-1.58	9.64E-02	1.31E-01
**DLGAP1-AS5**	-1.73	9.64E-02	1.31E-01
**RP11-715J22.4**	-1.49	9.64E-02	1.31E-01
**CDCA8**	0.30	9.65E-02	1.31E-01
**SCOC**	-0.28	9.65E-02	1.31E-01
**PCDHA13**	-1.60	9.65E-02	1.31E-01
**AHSP**	-2.17	9.66E-02	1.31E-01
**RN7SL812P**	-1.58	9.66E-02	1.31E-01
**RMDN2**	-0.35	9.66E-02	1.31E-01
**XLOC_000152**	0.69	9.67E-02	1.31E-01
**ELOVL1**	0.33	9.67E-02	1.31E-01
**IGHA2**	-1.81	9.67E-02	1.31E-01
**KRTAP5-AS1**	1.77	9.68E-02	1.31E-01
**BCL6**	0.25	9.69E-02	1.31E-01
**RP11-549L6.3**	-1.40	9.69E-02	1.31E-01
**EXTL1**	0.86	9.70E-02	1.32E-01
**TTC39C-AS1**	-0.97	9.70E-02	1.32E-01
**NOL8**	-0.17	9.71E-02	1.32E-01
**RP11-1110J8.1**	-2.23	9.72E-02	1.32E-01
**LINC00672**	-0.83	9.72E-02	1.32E-01
**CTD-2026D20.3**	0.36	9.72E-02	1.32E-01
**G3911**	1.43	9.73E-02	1.32E-01
**RP11-340I6.8**	-1.33	9.73E-02	1.32E-01
**ZNRF2**	-0.23	9.74E-02	1.32E-01
**PDCD6IP**	-0.22	9.74E-02	1.32E-01
**CYP2E1**	-0.49	9.74E-02	1.32E-01
**G4754**	0.48	9.74E-02	1.32E-01
**RP3-340N1.2**	-2.34	9.75E-02	1.32E-01
**SYCP1**	2.29	9.75E-02	1.32E-01
**BANCR**	2.04	9.75E-02	1.32E-01
**ARPIN**	-0.16	9.75E-02	1.32E-01

**RP11-269F20.1**	-1.14	9.76E-02	1.32E-01
**TNFRSF13C**	0.55	9.76E-02	1.32E-01
**AP000688.14**	-0.69	9.76E-02	1.32E-01
**CTD-2199O4.6**	-1.89	9.77E-02	1.32E-01
**TOMM7**	0.31	9.77E-02	1.32E-01
**ZMAT1**	0.72	9.77E-02	1.32E-01
**SPESP1**	-0.77	9.79E-02	1.33E-01
**RP11-77E14.2**	-1.81	9.79E-02	1.33E-01
**RP11-546K22.3**	-0.84	9.80E-02	1.33E-01
**ISM1**	0.44	9.80E-02	1.33E-01
**RP11-334C17.6**	-1.01	9.80E-02	1.33E-01
**IGLV3-1**	-2.59	9.81E-02	1.33E-01
**CNIH2**	-0.49	9.81E-02	1.33E-01
**CTD-2184D3.3**	0.61	9.81E-02	1.33E-01
**CTA-223H9.9**	-1.17	9.81E-02	1.33E-01
**RPS24P12**	-2.10	9.82E-02	1.33E-01
**KRT82**	-3.32	9.82E-02	1.33E-01
**TBC1D8**	0.24	9.82E-02	1.33E-01
**TMEM117**	0.28	9.83E-02	1.33E-01
**RP11-214K3.19**	-0.95	9.83E-02	1.33E-01
**CLGN**	1.25	9.84E-02	1.33E-01
**PCDHGB1**	-0.61	9.84E-02	1.33E-01
**RP11-57G10.8**	-1.65	9.85E-02	1.33E-01
**LCA5L**	-0.59	9.85E-02	1.33E-01
**C10orf88**	0.17	9.86E-02	1.34E-01
**SRRM2**	0.38	9.86E-02	1.34E-01
**AMPD3**	-0.24	9.86E-02	1.34E-01
**RAB6A**	0.21	9.87E-02	1.34E-01
**AC002306.1**	-1.48	9.87E-02	1.34E-01
**GPRASP1**	-0.35	9.87E-02	1.34E-01
**KCNH5**	-1.31	9.89E-02	1.34E-01
**ST13P19**	-1.59	9.89E-02	1.34E-01
**RP11-599B13.8**	1.51	9.90E-02	1.34E-01
**RP11-115D19.4**	-1.23	9.90E-02	1.34E-01
**RP11-685M7.3**	-0.75	9.90E-02	1.34E-01
**KCTD21-AS1**	0.60	9.90E-02	1.34E-01
**RP11-326C3.16**	-1.01	9.91E-02	1.34E-01
**RP11-382D12.2**	1.23	9.91E-02	1.34E-01
**AC011330.5**	-1.03	9.91E-02	1.34E-01
**KRTAP8-2P**	-2.73	9.92E-02	1.34E-01
**RP11-513D5.5**	-2.10	9.92E-02	1.34E-01
**AC011330.13**	-0.78	9.92E-02	1.34E-01
**RP11-16B13.1**	-1.50	9.92E-02	1.34E-01
**DEPTOR**	0.27	9.93E-02	1.34E-01
**RNU6-137P**	-0.86	9.93E-02	1.34E-01

**ZNF16**	-0.18	9.94E-02	1.34E-01
**CTC-296K1.4**	1.73	9.95E-02	1.35E-01
**CUL1**	-0.12	9.95E-02	1.35E-01
**MINCR**	-0.41	9.95E-02	1.35E-01
**DNAJA3**	0.17	9.95E-02	1.35E-01
**CDYL**	-0.14	9.95E-02	1.35E-01
**RPL23AP81**	-1.75	9.96E-02	1.35E-01
**AC004231.2**	-0.67	9.96E-02	1.35E-01
**RP11-361M10.3**	-1.16	9.96E-02	1.35E-01
**ITFG2**	0.25	9.96E-02	1.35E-01
**ADNP-AS1**	-0.48	9.96E-02	1.35E-01
**RP11-543P15.1**	0.39	9.97E-02	1.35E-01
**SLC44A5**	-1.18	9.97E-02	1.35E-01
**MCPH1-AS1**	0.53	9.97E-02	1.35E-01
**APOA1**	-1.64	9.98E-02	1.35E-01
**G3694**	-1.48	9.98E-02	1.35E-01
**EIF4G3**	-0.24	9.98E-02	1.35E-01
**LINC00484**	-1.69	9.99E-02	1.35E-01
**RP11-545P7.9**	-1.43	9.99E-02	1.35E-01
**MTND6P4**	1.01	1.00E-01	1.35E-01
**AC097724.3**	0.91	1.00E-01	1.35E-01
**FHIT**	-0.38	1.00E-01	1.35E-01
**RP11-148B18.1**	-0.88	1.00E-01	1.35E-01
**CTD-2555O16.2**	-0.46	1.00E-01	1.35E-01
**LINC00840**	1.00	1.00E-01	1.36E-01
**MYH7B**	-0.63	1.00E-01	1.36E-01
**COQ2**	-0.28	1.00E-01	1.36E-01
**CRYGS**	-0.50	1.00E-01	1.36E-01
**NUDT15**	0.29	1.00E-01	1.36E-01
**SMCO2**	-0.74	1.00E-01	1.36E-01
**G37636**	-0.96	1.01E-01	1.36E-01
**GGA2**	0.18	1.01E-01	1.36E-01
**NPAS1**	-0.47	1.01E-01	1.36E-01
**RP11-133N21.7**	-1.02	1.01E-01	1.36E-01
**PRB4**	-1.56	1.01E-01	1.36E-01
**SCML4**	-0.73	1.01E-01	1.36E-01
**KCNE1**	-0.79	1.01E-01	1.36E-01
**RP3-465N24.6**	0.80	1.01E-01	1.36E-01
**RBMS2**	-0.24	1.01E-01	1.36E-01
**RPL23AP7**	0.36	1.01E-01	1.36E-01
**LEFTY2**	2.31	1.01E-01	1.36E-01
**RP11-451N19.3**	-2.23	1.01E-01	1.36E-01
**RP11-330O11.3**	-0.92	1.01E-01	1.36E-01
**AC007292.4**	-1.40	1.01E-01	1.36E-01
**LHFPL4**	0.79	1.01E-01	1.36E-01

**CTD-2231E14.2**	0.65	1.01E-01	1.37E-01
**ZNF99**	-1.22	1.01E-01	1.37E-01
**C8orf59**	-0.25	1.01E-01	1.37E-01
**RPF2**	-0.25	1.02E-01	1.37E-01
**XBP1**	0.22	1.02E-01	1.37E-01
**RPL12P4**	-0.43	1.02E-01	1.37E-01
**GTF2IRD1P1**	-0.99	1.02E-01	1.37E-01
**CD52**	-0.49	1.02E-01	1.37E-01
**NUGGC**	-0.83	1.02E-01	1.37E-01
**PRCD**	0.48	1.02E-01	1.37E-01
**FABP2**	-2.18	1.02E-01	1.37E-01
**RP11-640A1.3**	-2.68	1.02E-01	1.38E-01
**HS2ST1**	-0.26	1.02E-01	1.38E-01
**WNT3**	0.29	1.02E-01	1.38E-01
**OSBPL2**	-0.16	1.02E-01	1.38E-01
**RP11-517C16.2**	-0.61	1.02E-01	1.38E-01
**FMO4**	0.38	1.02E-01	1.38E-01
**RP11-4L24.3**	1.66	1.02E-01	1.38E-01
**FAM206A**	-0.17	1.02E-01	1.38E-01
**MIR130A**	-1.99	1.02E-01	1.38E-01
**AF127936.9**	-0.53	1.02E-01	1.38E-01
**MAPK4**	0.83	1.02E-01	1.38E-01
**XLOC_008062**	1.60	1.02E-01	1.38E-01
**NXPE3**	0.28	1.02E-01	1.38E-01
**SPTBN4**	0.60	1.02E-01	1.38E-01
**XLOC_009911**	-1.84	1.02E-01	1.38E-01
**EIF5A2**	-0.32	1.02E-01	1.38E-01
**RP11-1D12.2**	-1.05	1.02E-01	1.38E-01
**RPF1**	0.25	1.02E-01	1.38E-01
**TTLL10**	-1.17	1.03E-01	1.38E-01
**RP11-708L7.9**	-2.10	1.03E-01	1.38E-01
**SH3KBP1**	-0.34	1.03E-01	1.38E-01
**CH17-335B8.4**	-0.84	1.03E-01	1.38E-01
**RMRPP3**	-1.39	1.03E-01	1.39E-01
**TMX4**	-0.27	1.03E-01	1.39E-01
**MIR548AT**	-1.81	1.03E-01	1.39E-01
**ANKRD40**	-0.16	1.03E-01	1.39E-01
**PNMA3**	1.00	1.03E-01	1.39E-01
**MORN2**	0.32	1.03E-01	1.39E-01
**ALOXE3**	-0.45	1.03E-01	1.39E-01
**FNDC8**	1.23	1.03E-01	1.39E-01
**HAND2-AS1**	-0.76	1.03E-01	1.39E-01
**RP11-85B7.5**	-1.66	1.03E-01	1.39E-01
**RP11-281O15.2**	-1.90	1.03E-01	1.39E-01
**FANCA**	-0.41	1.03E-01	1.39E-01

**ATP2A1**	-0.70	1.03E-01	1.39E-01
**MNAT1**	-0.20	1.03E-01	1.39E-01
**ZNF786**	0.25	1.03E-01	1.39E-01
**SPTLC1P1**	-1.50	1.03E-01	1.39E-01
**AP000692.10**	0.85	1.03E-01	1.39E-01
**RP11-478P10.1**	-2.02	1.03E-01	1.39E-01
**ARID4A**	0.21	1.03E-01	1.39E-01
**AC005262.4**	-2.15	1.03E-01	1.39E-01
**LPAR1**	-0.57	1.03E-01	1.39E-01
**C14orf119**	0.24	1.03E-01	1.39E-01
**AC005042.5**	-1.01	1.03E-01	1.39E-01
**XLOC_005405**	-2.00	1.04E-01	1.39E-01
**AC010148.1**	-0.98	1.04E-01	1.39E-01
**KIAA1524**	-0.33	1.04E-01	1.39E-01
**PRAC2**	6.22	1.04E-01	1.39E-01
**RP11-840I19.5**	-0.84	1.04E-01	1.40E-01
**SERPINA3**	1.19	1.04E-01	1.40E-01
**UBA6-AS1**	-0.33	1.04E-01	1.40E-01
**RP11-817O13.9**	-1.39	1.04E-01	1.40E-01
**RP11-290F5.1**	-1.44	1.04E-01	1.40E-01
**AHSG**	1.58	1.04E-01	1.40E-01
**ATP1A2**	0.75	1.04E-01	1.40E-01
**SDC1**	0.23	1.04E-01	1.40E-01
**DMTN**	0.33	1.04E-01	1.40E-01
**CLC**	-2.19	1.04E-01	1.40E-01
**MYO7A**	0.77	1.04E-01	1.40E-01
**MPC1**	-0.23	1.04E-01	1.40E-01
**CDC42SE1**	0.18	1.04E-01	1.40E-01
**XLOC_012332**	-1.19	1.04E-01	1.40E-01
**AC104653.1**	0.90	1.04E-01	1.40E-01
**C12orf56**	0.56	1.04E-01	1.40E-01
**G14007**	-1.97	1.04E-01	1.40E-01
**HCG4P7**	1.11	1.04E-01	1.41E-01
**PPRC1**	0.31	1.04E-01	1.41E-01
**IRGM**	-1.86	1.04E-01	1.41E-01
**XLOC_014405**	-1.97	1.05E-01	1.41E-01
**LRRC36**	-0.78	1.05E-01	1.41E-01
**RN7SL81P**	-1.52	1.05E-01	1.41E-01
**FAM227B**	-0.29	1.05E-01	1.41E-01
**RP4-635E18.7**	-1.11	1.05E-01	1.41E-01
**RP11-212P7.1**	-1.32	1.05E-01	1.41E-01
**TMEM44-AS1**	0.47	1.05E-01	1.41E-01
**CTD-3105H18.8**	-1.72	1.05E-01	1.41E-01
**AK3**	-0.28	1.05E-01	1.41E-01
**FAM122B**	0.31	1.05E-01	1.41E-01

**GPM6A**	-0.70	1.05E-01	1.41E-01
**TRIAP1**	0.33	1.05E-01	1.41E-01
**SLC25A37**	0.33	1.05E-01	1.41E-01
**USP28**	0.25	1.05E-01	1.42E-01
**BTRC**	-0.18	1.06E-01	1.42E-01
**CD2**	0.71	1.06E-01	1.42E-01
**AC099850.1**	0.45	1.06E-01	1.42E-01
**RP11-36B6.1**	-2.05	1.06E-01	1.42E-01
**PTK2**	0.15	1.06E-01	1.42E-01
**REP15**	-1.28	1.06E-01	1.42E-01
**CTC-444N24.13**	-1.22	1.06E-01	1.42E-01
**CSF3**	1.49	1.06E-01	1.42E-01
**TMEM231**	0.26	1.06E-01	1.42E-01
**TMEM147**	0.26	1.06E-01	1.42E-01
**KRTAP9-8**	-4.52	1.06E-01	1.42E-01
**FAM163A**	1.78	1.06E-01	1.42E-01
**GMDS-AS1**	-0.54	1.06E-01	1.42E-01
**CTC-338M12.5**	-1.26	1.06E-01	1.43E-01
**RP11-219E7.3**	-1.78	1.06E-01	1.43E-01
**IDH1**	0.48	1.06E-01	1.43E-01
**SLC24A1**	0.21	1.06E-01	1.43E-01
**RNU6-824P**	-1.30	1.06E-01	1.43E-01
**KRTAP9-4**	-4.22	1.06E-01	1.43E-01
**SETD6**	-0.27	1.07E-01	1.43E-01
**RP11-376P6.3**	-1.32	1.07E-01	1.43E-01
**RP11-1038A11.2**	-2.16	1.07E-01	1.43E-01
**ABCB4**	-0.41	1.07E-01	1.43E-01
**RORC**	-0.32	1.07E-01	1.43E-01
**AC107218.3**	-1.54	1.07E-01	1.43E-01
**RP3-424M6.4**	0.45	1.07E-01	1.43E-01
**PRSS42**	-1.87	1.07E-01	1.43E-01
**RP11-392A14.8**	-1.62	1.07E-01	1.43E-01
**TMEM256**	-0.38	1.07E-01	1.43E-01
**PLPPR1**	-1.29	1.07E-01	1.43E-01
**RP11-277P12.10**	-1.04	1.07E-01	1.43E-01
**CST7**	0.70	1.07E-01	1.43E-01
**RP11-116O18.3**	-2.18	1.07E-01	1.44E-01
**TRAJ23**	-2.13	1.07E-01	1.44E-01
**CTB-25B13.12**	0.45	1.07E-01	1.44E-01
**RANP1**	-1.48	1.07E-01	1.44E-01
**RP11-506O24.1**	-0.84	1.07E-01	1.44E-01
**GAL3ST4**	-0.32	1.07E-01	1.44E-01
**RNA5SP343**	-2.39	1.07E-01	1.44E-01
**RP11-289H16.1**	-1.60	1.07E-01	1.44E-01
**CTC-436P18.5**	-0.82	1.07E-01	1.44E-01

**BCKDHB**	-0.35	1.07E-01	1.44E-01
**CTB-161M19.4**	-0.94	1.07E-01	1.44E-01
**SLC2A14**	-1.56	1.07E-01	1.44E-01
**RP11-490O6.2**	-0.86	1.07E-01	1.44E-01
**RNU6-32P**	-1.99	1.07E-01	1.44E-01
**CISD1**	0.29	1.07E-01	1.44E-01
**RP11-262H14.11**	-1.06	1.07E-01	1.44E-01
**AC002519.8**	-1.60	1.08E-01	1.44E-01
**RP3-324O17.7**	-1.07	1.08E-01	1.44E-01
**ISOC1**	-0.33	1.08E-01	1.44E-01
**NDUFAB1**	0.25	1.08E-01	1.44E-01
**XLOC_005945**	-2.38	1.08E-01	1.44E-01
**FAHD2A**	0.25	1.08E-01	1.44E-01
**RP11-475C16.1**	0.30	1.08E-01	1.44E-01
**ARF6**	0.23	1.08E-01	1.44E-01
**RP11-44F21.5**	0.90	1.08E-01	1.45E-01
**RP11-647K16.1**	-0.60	1.08E-01	1.45E-01
**WTAP**	0.16	1.08E-01	1.45E-01
**RPS2P32**	0.73	1.08E-01	1.45E-01
**MUM1**	0.27	1.08E-01	1.45E-01
**CCDC178**	1.72	1.08E-01	1.45E-01
**FH**	-0.25	1.08E-01	1.45E-01
**ACOXL**	1.11	1.08E-01	1.45E-01
**BHMT2**	0.68	1.08E-01	1.45E-01
**FXNP2**	-2.08	1.08E-01	1.45E-01
**RP11-336K24.12**	-1.00	1.08E-01	1.45E-01
**RP11-686D22.5**	1.60	1.08E-01	1.45E-01
**SIGLEC5**	1.89	1.08E-01	1.45E-01
**ANXA7**	0.21	1.08E-01	1.45E-01
**RP11-2N1.3**	-2.28	1.08E-01	1.45E-01
**RP11-33N14.5**	-1.35	1.08E-01	1.45E-01
**XLOC_011393**	-2.29	1.08E-01	1.45E-01
**FABP3**	0.52	1.08E-01	1.45E-01
**FAM205C**	-1.40	1.08E-01	1.45E-01
**LINC01535**	-0.96	1.08E-01	1.45E-01
**CTD-2026K11.5**	0.77	1.08E-01	1.45E-01
**GLI2**	0.59	1.08E-01	1.45E-01
**RP11-787D11.1**	-2.39	1.08E-01	1.45E-01
**ZNF534**	-1.50	1.09E-01	1.45E-01
**CTB-50L17.16**	1.45	1.09E-01	1.46E-01
**RNU6-322P**	-0.95	1.09E-01	1.46E-01
**MYCN**	0.74	1.09E-01	1.46E-01
**NTNG1**	1.05	1.09E-01	1.46E-01
**CTB-96E2.6**	1.31	1.09E-01	1.46E-01
**RP4-682C21.5**	-1.97	1.09E-01	1.46E-01

**RP11-388C12.5**	-1.97	1.09E-01	1.46E-01
**CTD-2650P22.1**	1.24	1.09E-01	1.46E-01
**SNX5**	-0.12	1.09E-01	1.46E-01
**NKX2-8**	1.52	1.09E-01	1.46E-01
**DLX2**	1.23	1.09E-01	1.46E-01
**TM6SF1**	-0.58	1.09E-01	1.46E-01
**AC090617.1**	-1.68	1.09E-01	1.46E-01
**RP11-85D18.1**	-1.50	1.09E-01	1.46E-01
**AC073284.4**	-1.15	1.09E-01	1.46E-01
**LINC01480**	1.48	1.09E-01	1.46E-01
**RP11-10J21.4**	-1.12	1.09E-01	1.46E-01
**CTD-3064H18.2**	-1.95	1.09E-01	1.46E-01
**PAX5**	1.80	1.09E-01	1.46E-01
**RFPL3S**	-1.32	1.09E-01	1.46E-01
**ALDH9A1**	0.26	1.09E-01	1.46E-01
**TBX5**	-0.71	1.09E-01	1.46E-01
**NLGN3**	0.45	1.09E-01	1.46E-01
**LGALS3**	0.30	1.09E-01	1.46E-01
**KLRF1**	0.84	1.09E-01	1.46E-01
**AC092301.3**	-1.16	1.09E-01	1.47E-01
**CA3-AS1**	-0.57	1.09E-01	1.47E-01
**RP11-17E2.2**	-1.84	1.09E-01	1.47E-01
**FBXW11**	-0.14	1.10E-01	1.47E-01
**SLC36A4**	-0.27	1.10E-01	1.47E-01
**RP11-229O3.1**	1.27	1.10E-01	1.47E-01
**RP11-64C1.1**	3.34	1.10E-01	1.47E-01
**PDGFC**	-0.37	1.10E-01	1.47E-01
**RP11-259K5.1**	-2.37	1.10E-01	1.47E-01
**RN7SL145P**	-1.38	1.10E-01	1.47E-01
**ZNF521**	0.49	1.10E-01	1.47E-01
**G25674**	-2.29	1.10E-01	1.47E-01
**CDV3**	-0.21	1.10E-01	1.47E-01
**ICAM3**	-0.71	1.10E-01	1.47E-01
**RP11-1070A24.1**	-2.06	1.10E-01	1.47E-01
**RP11-181C21.4**	1.21	1.10E-01	1.47E-01
**TPCN2**	0.25	1.10E-01	1.47E-01
**RP11-2H8.4**	-0.68	1.10E-01	1.47E-01
**CES5AP1**	-1.94	1.10E-01	1.47E-01
**CTD-3064M3.4**	-1.45	1.10E-01	1.47E-01
**AC091633.3**	-1.15	1.10E-01	1.47E-01
**RPL5**	0.26	1.10E-01	1.47E-01
**CUL4B**	-0.18	1.10E-01	1.47E-01
**RP11-344B2.3**	-1.36	1.10E-01	1.47E-01
**EEF1DP4**	-1.12	1.10E-01	1.48E-01
**MIR34AHG**	0.51	1.10E-01	1.48E-01

**ANO6**	-0.23	1.10E-01	1.48E-01
**RP11-574K11.32**	-1.38	1.10E-01	1.48E-01
**USP8**	-0.15	1.10E-01	1.48E-01
**G13124**	-0.55	1.11E-01	1.48E-01
**CKMT1A**	0.27	1.11E-01	1.48E-01
**TMIGD2**	1.76	1.11E-01	1.48E-01
**UBXN4**	0.17	1.11E-01	1.48E-01
**RP11-157P1.4**	-0.56	1.11E-01	1.48E-01
**TXLNA**	-0.14	1.11E-01	1.48E-01
**G35422**	-0.93	1.11E-01	1.48E-01
**STK32A**	-0.61	1.11E-01	1.48E-01
**IFI44L**	0.96	1.11E-01	1.49E-01
**RP11-417N10.5**	-2.03	1.11E-01	1.49E-01
**APITD1**	-0.76	1.11E-01	1.49E-01
**RP11-167H9.5**	-1.08	1.11E-01	1.49E-01
**AC010150.1**	-1.27	1.11E-01	1.49E-01
**LINC00294**	-0.30	1.11E-01	1.49E-01
**THUMPD3**	-0.13	1.11E-01	1.49E-01
**bP-2171C21.5**	1.90	1.11E-01	1.49E-01
**UBE3C**	-0.17	1.11E-01	1.49E-01
**SNHG23**	0.74	1.11E-01	1.49E-01
**PPIAP6**	-2.18	1.11E-01	1.49E-01
**YPEL4**	-0.52	1.12E-01	1.49E-01
**NBPF14**	-0.52	1.12E-01	1.49E-01
**RP11-477L16.2**	-2.16	1.12E-01	1.49E-01
**IL17RE**	0.28	1.12E-01	1.49E-01
**TAF9B**	-0.26	1.12E-01	1.49E-01
**ADRB1**	-0.75	1.12E-01	1.49E-01
**RP11-473M20.11**	1.01	1.12E-01	1.49E-01
**RP11-126L15.4**	-0.61	1.12E-01	1.49E-01
**PSAT1P3**	1.12	1.12E-01	1.49E-01
**KNSTRN**	-0.44	1.12E-01	1.50E-01
**KARSP2**	-1.25	1.12E-01	1.50E-01
**RP5-1039K5.12**	0.40	1.12E-01	1.50E-01
**RIMS4**	-1.19	1.12E-01	1.50E-01
**RP3-439F8.1**	-0.53	1.12E-01	1.50E-01
**RP11-330M19.1**	-1.88	1.13E-01	1.50E-01
**CTA-747E2.10**	-0.86	1.13E-01	1.50E-01
**PAM**	0.51	1.13E-01	1.50E-01
**RTN3**	-0.20	1.13E-01	1.50E-01
**G31903**	-1.10	1.13E-01	1.50E-01
**TSPAN32**	0.57	1.13E-01	1.50E-01
**RAB3C**	0.83	1.13E-01	1.51E-01
**CTC-260E6.4**	-1.13	1.13E-01	1.51E-01
**XLOC_011615**	-1.27	1.13E-01	1.51E-01

**NGDN**	0.21	1.13E-01	1.51E-01
**WNT7A**	0.51	1.13E-01	1.51E-01
**CCDC60**	1.41	1.13E-01	1.51E-01
**RN7SL99P**	-2.39	1.13E-01	1.51E-01
**AC011816.1**	-2.09	1.13E-01	1.51E-01
**TEX26-AS1**	1.12	1.13E-01	1.51E-01
**CEP85**	-0.23	1.13E-01	1.51E-01
**RP13-20L14.4**	-1.37	1.13E-01	1.51E-01
**CYP2T1P**	0.43	1.13E-01	1.51E-01
**RP11-1281K21.1**	-2.40	1.13E-01	1.51E-01
**EIF3E**	-0.28	1.13E-01	1.51E-01
**MIR2116**	-1.74	1.13E-01	1.51E-01
**AC093901.1**	0.97	1.13E-01	1.51E-01
**MIR3118-2**	-1.24	1.13E-01	1.51E-01
**ALOX15B**	-1.30	1.13E-01	1.51E-01
**SH3GL2**	-1.98	1.14E-01	1.51E-01
**GLO1**	-0.37	1.14E-01	1.52E-01
**HSD17B8**	0.34	1.14E-01	1.52E-01
**EFNB3**	0.31	1.14E-01	1.52E-01
**PLAC9**	0.53	1.14E-01	1.52E-01
**RP13-128O4.3**	0.83	1.14E-01	1.52E-01
**WSB2**	0.19	1.14E-01	1.52E-01
**RP11-332H18.5**	-1.54	1.14E-01	1.52E-01
**LY6K**	0.54	1.14E-01	1.52E-01
**RPL34P18**	-1.91	1.14E-01	1.52E-01
**NEU2**	-0.69	1.14E-01	1.52E-01
**HIBCH**	0.25	1.14E-01	1.52E-01
**AL133318.1**	-1.19	1.14E-01	1.52E-01
**GDF5**	1.26	1.14E-01	1.52E-01
**RP11-417J8.6**	-1.01	1.14E-01	1.52E-01
**CCDC25**	0.22	1.14E-01	1.52E-01
**TUBA4A**	0.33	1.14E-01	1.52E-01
**CSNK2A3**	-0.94	1.14E-01	1.53E-01
**G4640**	-1.60	1.14E-01	1.53E-01
**BACH2**	-0.33	1.14E-01	1.53E-01
**RP11-599J14.2**	-0.58	1.14E-01	1.53E-01
**RP11-1114A5.4**	0.55	1.14E-01	1.53E-01
**HMGB3P32**	-1.27	1.15E-01	1.53E-01
**RP11-498C9.12**	-1.39	1.15E-01	1.53E-01
**RP11-252A24.3**	-0.79	1.15E-01	1.53E-01
**SOAT2**	0.90	1.15E-01	1.53E-01
**SLC5A10**	0.74	1.15E-01	1.53E-01
**RP11-432J24.2**	-1.69	1.15E-01	1.53E-01
**RP11-568J23.5**	-1.01	1.15E-01	1.53E-01
**AC079630.4**	0.46	1.15E-01	1.53E-01

**CTC-559E9.8**	-0.50	1.15E-01	1.53E-01
**CTB-50L17.5**	-2.19	1.15E-01	1.53E-01
**RP11-737O24.5**	-1.69	1.15E-01	1.53E-01
**RP11-651L5.2**	-1.07	1.15E-01	1.53E-01
**TFCP2L1**	0.49	1.15E-01	1.53E-01
**PRRT3**	0.24	1.15E-01	1.53E-01
**RGPD4**	-1.96	1.15E-01	1.53E-01
**P2RX5-TAX1BP3**	-0.52	1.15E-01	1.53E-01
**ALG1L2**	0.79	1.15E-01	1.54E-01
**MYL12B**	0.23	1.15E-01	1.54E-01
**AQP10**	1.38	1.15E-01	1.54E-01
**CR848007.2**	-1.15	1.15E-01	1.54E-01
**KLHL34**	-0.75	1.15E-01	1.54E-01
**WDR26**	-0.23	1.15E-01	1.54E-01
**SPA17**	-0.42	1.15E-01	1.54E-01
**WFIKKN1**	-0.61	1.15E-01	1.54E-01
**NPFFR1**	0.83	1.15E-01	1.54E-01
**MST1L**	-0.87	1.16E-01	1.54E-01
**ERLIN1**	0.16	1.16E-01	1.54E-01
**AP000438.2**	-2.07	1.16E-01	1.54E-01
**NOL12**	-0.66	1.16E-01	1.54E-01
**RAD54L**	-0.44	1.16E-01	1.54E-01
**BACE1-AS**	0.43	1.16E-01	1.54E-01
**CCIN**	1.54	1.16E-01	1.54E-01
**STK24-AS1**	-0.92	1.16E-01	1.54E-01
**RP11-603J24.7**	-0.79	1.16E-01	1.54E-01
**ZNF75D**	-0.18	1.16E-01	1.54E-01
**ATP6V1D**	0.24	1.16E-01	1.54E-01
**PSD2**	0.85	1.16E-01	1.54E-01
**RP11-1055B8.4**	-0.71	1.16E-01	1.54E-01
**CXXC4**	0.73	1.16E-01	1.54E-01
**ARHGEF34P**	-0.58	1.16E-01	1.54E-01
**RP13-631K18.2**	-1.79	1.16E-01	1.55E-01
**LINC00444**	-1.30	1.16E-01	1.55E-01
**CH507-42P11.3**	-2.20	1.16E-01	1.55E-01
**DYNLL1P4**	-2.15	1.16E-01	1.55E-01
**RNU6-1279P**	-2.36	1.16E-01	1.55E-01
**RP11-381E24.4**	-2.01	1.16E-01	1.55E-01
**KB-1991G8.1**	-0.62	1.16E-01	1.55E-01
**DMBT1P1**	-2.02	1.16E-01	1.55E-01
**GNG5P3**	-1.97	1.16E-01	1.55E-01
**FLJ38576**	-1.99	1.16E-01	1.55E-01
**LINGO4**	-0.72	1.16E-01	1.55E-01
**AC007881.1**	-1.58	1.16E-01	1.55E-01
**AC144831.3**	-0.47	1.17E-01	1.55E-01

**RP1-37N7.1**	-0.87	1.17E-01	1.55E-01
**ZNF561-AS1**	-0.32	1.17E-01	1.55E-01
**RP11-227F19.5**	-1.31	1.17E-01	1.55E-01
**RP11-166B2.7**	-1.70	1.17E-01	1.55E-01
**HNRNPA1P53**	-1.69	1.17E-01	1.55E-01
**ZNF474**	-0.82	1.17E-01	1.55E-01
**RP11-699L21.1**	-1.56	1.17E-01	1.56E-01
**ARSF**	0.61	1.17E-01	1.56E-01
**RP11-752G15.10**	-1.84	1.17E-01	1.56E-01
**ERMAP**	-0.17	1.17E-01	1.56E-01
**RP11-544L8__B.4**	1.59	1.17E-01	1.56E-01
**AC002116.7**	-1.14	1.17E-01	1.56E-01
**C2orf54**	0.29	1.17E-01	1.56E-01
**AKR1C3**	0.35	1.17E-01	1.56E-01
**PLCH1**	0.80	1.17E-01	1.56E-01
**RP11-648L3.2**	-1.86	1.17E-01	1.56E-01
**RP4-775C13.1**	-1.57	1.17E-01	1.56E-01
**CAPZA2**	-0.27	1.17E-01	1.56E-01
**PLGRKT**	0.29	1.17E-01	1.56E-01
**LINC00942**	1.10	1.17E-01	1.56E-01
**RP3-441A12.1**	-1.61	1.17E-01	1.56E-01
**CTC-453G23.4**	-1.62	1.17E-01	1.56E-01
**NIFK-AS1**	0.24	1.17E-01	1.56E-01
**KCNIP1**	1.24	1.17E-01	1.56E-01
**RP11-213G2.5**	1.43	1.17E-01	1.56E-01
**CUBN**	-0.50	1.17E-01	1.56E-01
**RP4-781K5.4**	-0.73	1.17E-01	1.56E-01
**RP11-497H16.9**	-2.71	1.18E-01	1.56E-01
**RP4-773N10.4**	-0.28	1.18E-01	1.56E-01
**RNU7-75P**	-1.09	1.18E-01	1.56E-01
**RNU6-1042P**	-2.40	1.18E-01	1.56E-01
**AC005488.1**	1.23	1.18E-01	1.56E-01
**RP11-227G15.6**	-2.03	1.18E-01	1.56E-01
**ATP6V1H**	0.21	1.18E-01	1.56E-01
**DRAIC**	1.36	1.18E-01	1.57E-01
**DGKB**	1.09	1.18E-01	1.57E-01
**SRRM5**	-0.86	1.18E-01	1.57E-01
**AP001056.1**	1.31	1.18E-01	1.57E-01
**PTPN22**	0.53	1.18E-01	1.57E-01
**AC093724.2**	0.65	1.18E-01	1.57E-01
**RP3-395M20.8**	0.56	1.18E-01	1.57E-01
**CTA-963H5.5**	0.58	1.18E-01	1.57E-01
**G1876**	-1.09	1.18E-01	1.57E-01
**ITK**	-0.59	1.18E-01	1.57E-01
**KCNA7**	0.83	1.18E-01	1.57E-01

**RSL1D1**	0.23	1.18E-01	1.57E-01
**MTHFD1**	-0.19	1.18E-01	1.57E-01
**AC117395.1**	0.87	1.19E-01	1.57E-01
**AC006077.3**	-0.82	1.19E-01	1.57E-01
**RP3-402G11.28**	0.78	1.19E-01	1.57E-01
**KRTAP4-5**	-3.38	1.19E-01	1.57E-01
**AC011477.1**	-1.74	1.19E-01	1.58E-01
**ADCY7**	-0.24	1.19E-01	1.58E-01
**RP11-219E7.4**	-1.21	1.19E-01	1.58E-01
**AC016708.2**	-0.71	1.19E-01	1.58E-01
**IFT27**	0.28	1.19E-01	1.58E-01
**ZNF334**	-0.68	1.19E-01	1.58E-01
**G23532**	-2.58	1.19E-01	1.58E-01
**SLIT1**	-0.76	1.19E-01	1.58E-01
**RP3-395M20.7**	-1.95	1.19E-01	1.58E-01
**COX6B2**	0.97	1.20E-01	1.59E-01
**G5909**	-0.93	1.20E-01	1.59E-01
**RP11-582E3.2**	-1.81	1.20E-01	1.59E-01
**AP000347.2**	0.59	1.20E-01	1.59E-01
**FLJ26245**	-2.36	1.20E-01	1.59E-01
**FGF14**	-0.62	1.20E-01	1.59E-01
**RP11-1070N10.3**	-1.17	1.20E-01	1.59E-01
**TCF7**	-0.40	1.20E-01	1.59E-01
**BST1**	0.48	1.20E-01	1.59E-01
**RP11-51J9.4**	-1.25	1.20E-01	1.59E-01
**XXbac-B444P24.14**	-1.04	1.20E-01	1.59E-01
**SLC48A1**	0.22	1.20E-01	1.59E-01
**RNU7-135P**	-1.23	1.20E-01	1.59E-01
**RP5-1024N4.4**	-1.23	1.20E-01	1.59E-01
**RPS13**	0.27	1.20E-01	1.59E-01
**SLC22A7**	-1.80	1.20E-01	1.59E-01
**PPIAP29**	-0.64	1.20E-01	1.59E-01
**RP4-545K15.5**	-0.69	1.20E-01	1.59E-01
**DZANK1**	0.35	1.20E-01	1.59E-01
**DCAF12**	0.15	1.20E-01	1.59E-01
**MIRLET7DHG**	-1.16	1.20E-01	1.59E-01
**G39812**	-0.76	1.20E-01	1.59E-01
**RP11-894J14.5**	-0.64	1.20E-01	1.59E-01
**RP3-522J7.6**	-1.46	1.20E-01	1.59E-01
**G26573**	0.95	1.20E-01	1.59E-01
**RP11-848P1.7**	-0.74	1.20E-01	1.59E-01
**IGHV3-7**	-2.11	1.20E-01	1.59E-01
**B4GALT1-AS1**	-0.50	1.20E-01	1.59E-01
**RP11-130L8.3**	1.05	1.20E-01	1.60E-01
**RP11-209M4.1**	-1.63	1.20E-01	1.60E-01

**RAD51C**	0.20	1.20E-01	1.60E-01
**RP11-956J14.1**	-2.22	1.20E-01	1.60E-01
**RP11-495P10.3**	-1.50	1.20E-01	1.60E-01
**GGT1**	0.74	1.20E-01	1.60E-01
**HSH2D**	0.91	1.20E-01	1.60E-01
**SCUBE3**	-0.51	1.21E-01	1.60E-01
**RP11-486O13.4**	-1.96	1.21E-01	1.60E-01
**XLOC_010305**	-1.28	1.21E-01	1.60E-01
**ENO1-AS1**	-1.17	1.21E-01	1.60E-01
**RP11-227D13.1**	1.42	1.21E-01	1.60E-01
**HECW1**	-0.83	1.21E-01	1.60E-01
**RP11-409I10.2**	-1.48	1.21E-01	1.60E-01
**RASA4B**	1.16	1.21E-01	1.60E-01
**RNU6-1337P**	-1.85	1.21E-01	1.60E-01
**RP11-172H24.4**	1.01	1.21E-01	1.60E-01
**RP11-619I22.1**	-1.94	1.21E-01	1.60E-01
**RYR1**	-0.34	1.21E-01	1.60E-01
**LIX1**	1.09	1.21E-01	1.60E-01
**ZFR**	-0.20	1.21E-01	1.60E-01
**XXbac-B135H6.15**	1.04	1.21E-01	1.60E-01
**GPC6-AS1**	-2.07	1.21E-01	1.61E-01
**RHOT1P1**	1.54	1.21E-01	1.61E-01
**OXER1**	0.53	1.21E-01	1.61E-01
**RP11-326K13.4**	0.41	1.21E-01	1.61E-01
**CRAT**	-1.17	1.21E-01	1.61E-01
**TMEM65**	-0.20	1.21E-01	1.61E-01
**RBMX2P3**	1.32	1.22E-01	1.61E-01
**NETO1**	-1.05	1.22E-01	1.61E-01
**TSIX**	-1.87	1.22E-01	1.61E-01
**GBP3**	0.42	1.22E-01	1.61E-01
**RP11-109N23.1**	-1.23	1.22E-01	1.61E-01
**NCEH1**	0.42	1.22E-01	1.61E-01
**PNMA2**	0.45	1.22E-01	1.61E-01
**C6orf52**	-0.51	1.22E-01	1.61E-01
**AP003064.1**	-1.47	1.22E-01	1.62E-01
**SETD5**	0.25	1.22E-01	1.62E-01
**CEACAM1**	0.81	1.22E-01	1.62E-01
**BIRC5**	0.43	1.22E-01	1.62E-01
**CYP7A1**	-1.12	1.22E-01	1.62E-01
**CTB-58E17.3**	0.71	1.22E-01	1.62E-01
**DUSP8P5**	0.61	1.22E-01	1.62E-01
**RP11-106M7.1**	1.36	1.22E-01	1.62E-01
**AC007879.2**	-1.58	1.23E-01	1.62E-01
**TRAC**	0.64	1.23E-01	1.62E-01
**RNU6-1280P**	-1.40	1.23E-01	1.62E-01

**CAPZA1**	-0.20	1.23E-01	1.62E-01
**RP11-15J10.1**	-0.94	1.23E-01	1.62E-01
**RP1-28O10.1**	1.19	1.23E-01	1.62E-01
**RP11-392O17.2**	-0.78	1.23E-01	1.62E-01
**ABALON**	-0.83	1.23E-01	1.62E-01
**DNAAF1**	-0.82	1.23E-01	1.62E-01
**RP4-585I14.3**	-1.82	1.23E-01	1.62E-01
**GABRE**	-0.64	1.23E-01	1.63E-01
**RGS6**	-0.69	1.23E-01	1.63E-01
**RP11-673E1.1**	-1.28	1.23E-01	1.63E-01
**DCST2**	-0.71	1.23E-01	1.63E-01
**RP4-799P18.2**	1.96	1.23E-01	1.63E-01
**ANKRD23**	-0.59	1.23E-01	1.63E-01
**SKAP1**	0.63	1.23E-01	1.63E-01
**AL021917.1**	-1.98	1.23E-01	1.63E-01
**MIR647**	1.31	1.23E-01	1.63E-01
**HDAC2**	0.16	1.23E-01	1.63E-01
**HMGB1P24**	-0.97	1.23E-01	1.63E-01
**RP11-203J24.8**	-0.78	1.23E-01	1.63E-01
**BHLHA15**	-1.66	1.24E-01	1.63E-01
**ZNF382**	-0.46	1.24E-01	1.63E-01
**TTC23**	0.13	1.24E-01	1.63E-01
**RP11-108O10.2**	-1.52	1.24E-01	1.63E-01
**G39803**	-0.82	1.24E-01	1.63E-01
**FAM26D**	-3.02	1.24E-01	1.63E-01
**TAF9**	0.25	1.24E-01	1.63E-01
**RP11-214K3.5**	-1.89	1.24E-01	1.63E-01
**GPCPD1**	0.21	1.24E-01	1.64E-01
**RP11-304F15.5**	-2.08	1.24E-01	1.64E-01
**AC069363.1**	-2.28	1.24E-01	1.64E-01
**TMEM192**	0.18	1.24E-01	1.64E-01
**WSB1**	0.32	1.24E-01	1.64E-01
**TTC27**	-0.22	1.24E-01	1.64E-01
**B3GALNT1**	0.31	1.24E-01	1.64E-01
**YJEFN3**	0.93	1.24E-01	1.64E-01
**TBC1D3F**	-1.00	1.24E-01	1.64E-01
**ATP1A4**	-1.99	1.24E-01	1.64E-01
**RP11-214O1.3**	-1.43	1.24E-01	1.64E-01
**RNU4ATAC18P**	-0.99	1.24E-01	1.64E-01
**CECR6**	-0.51	1.24E-01	1.64E-01
**ARHGAP31-AS1**	-0.77	1.24E-01	1.64E-01
**AC010504.2**	-0.94	1.24E-01	1.64E-01
**RP11-392P7.1**	-1.86	1.24E-01	1.64E-01
**RP11-512N21.3**	-1.98	1.25E-01	1.64E-01
**TDGF1**	1.36	1.25E-01	1.64E-01

**SYNPO2**	0.52	1.25E-01	1.65E-01
**SMIM17**	-1.16	1.25E-01	1.65E-01
**PPA1**	0.29	1.25E-01	1.65E-01
**NDUFB5**	0.26	1.25E-01	1.65E-01
**PRR13P5**	1.00	1.25E-01	1.65E-01
**RPS26**	0.61	1.25E-01	1.65E-01
**MIR33B**	-2.05	1.25E-01	1.65E-01
**G16043**	-1.34	1.25E-01	1.65E-01
**CNBD2**	-0.81	1.25E-01	1.65E-01
**LRRIQ1**	0.83	1.25E-01	1.65E-01
**RP4-669B10.3**	-2.76	1.25E-01	1.65E-01
**AC009120.4**	-1.62	1.25E-01	1.65E-01
**CTD-2313J17.7**	-1.04	1.25E-01	1.65E-01
**RP11-20I20.4**	0.75	1.25E-01	1.65E-01
**HRASLS**	-0.56	1.25E-01	1.65E-01
**SUMO2P17**	1.01	1.25E-01	1.65E-01
**NAPSA**	0.68	1.25E-01	1.65E-01
**MEF2C-AS1**	-0.79	1.26E-01	1.66E-01
**SDHAP1**	-0.41	1.26E-01	1.66E-01
**ZNF747**	-0.24	1.26E-01	1.66E-01
**AQP7P1**	1.29	1.26E-01	1.66E-01
**WARS2**	-0.19	1.26E-01	1.66E-01
**RPSAP53**	-2.99	1.26E-01	1.66E-01
**NDUFB1P2**	-1.36	1.26E-01	1.66E-01
**FSTL1**	0.62	1.26E-01	1.66E-01
**RP11-454E5.4**	0.38	1.26E-01	1.66E-01
**KIF18B**	0.37	1.26E-01	1.66E-01
**KCNJ12**	-0.35	1.26E-01	1.66E-01
**CTD-2047H16.5**	-1.62	1.26E-01	1.66E-01
**SYCE2**	-0.84	1.26E-01	1.66E-01
**MAS1L**	-1.33	1.26E-01	1.66E-01
**PIK3R6**	0.53	1.26E-01	1.66E-01
**SIGLEC11**	1.48	1.26E-01	1.66E-01
**G13690**	-1.45	1.26E-01	1.66E-01
**AC008391.1**	-0.88	1.26E-01	1.67E-01
**CACNA1G-AS1**	-1.12	1.26E-01	1.67E-01
**RP11-125O18.1**	4.81	1.26E-01	1.67E-01
**AC007312.3**	-1.74	1.26E-01	1.67E-01
**LINC00562**	-0.73	1.26E-01	1.67E-01
**RP11-274B21.2**	0.42	1.26E-01	1.67E-01
**KB-1471A8.1**	-1.32	1.26E-01	1.67E-01
**AC092620.2**	-0.72	1.27E-01	1.67E-01
**CTC-461F20.1**	-1.92	1.27E-01	1.67E-01
**NUB1**	0.15	1.27E-01	1.67E-01
**AC018647.3**	0.82	1.27E-01	1.67E-01

**RPS29**	0.35	1.27E-01	1.67E-01
**CPA2**	1.13	1.27E-01	1.67E-01
**MIR193BHG**	-0.32	1.27E-01	1.67E-01
**RP11-707P17.2**	-0.88	1.27E-01	1.67E-01
**RP4-657E11.10**	-0.45	1.27E-01	1.67E-01
**RAPGEF5**	-0.30	1.27E-01	1.67E-01
**EBAG9**	0.17	1.27E-01	1.67E-01
**NALT1**	0.79	1.27E-01	1.67E-01
**LINC00598**	-0.78	1.27E-01	1.67E-01
**GPR15**	-1.53	1.27E-01	1.67E-01
**HUS1B**	0.88	1.27E-01	1.68E-01
**CCDC47**	-0.22	1.27E-01	1.68E-01
**SDHD**	-0.28	1.27E-01	1.68E-01
**MEIS1**	-0.34	1.27E-01	1.68E-01
**AC011385.2**	-1.64	1.27E-01	1.68E-01
**AC068522.4**	-1.00	1.27E-01	1.68E-01
**RP11-1094M14.5**	-1.31	1.27E-01	1.68E-01
**XLOC_002220**	-1.67	1.27E-01	1.68E-01
**RP11-252K23.1**	-1.16	1.28E-01	1.68E-01
**ABCA2**	0.27	1.28E-01	1.68E-01
**LINC01024**	0.31	1.28E-01	1.68E-01
**KCNH7**	-1.09	1.28E-01	1.68E-01
**ZNF890P**	-2.02	1.28E-01	1.68E-01
**CYP4A22-AS1**	1.36	1.28E-01	1.68E-01
**RP11-973F15.2**	-1.58	1.28E-01	1.68E-01
**XLOC_004632**	1.16	1.28E-01	1.68E-01
**TBC1D29**	-1.64	1.28E-01	1.68E-01
**ACOT8**	-0.24	1.28E-01	1.68E-01
**BCORP1**	-1.27	1.28E-01	1.68E-01
**RP11-480I12.9**	-0.81	1.28E-01	1.68E-01
**CRHR2**	-0.79	1.28E-01	1.69E-01
**RP11-700H6.1**	-1.18	1.28E-01	1.69E-01
**RN7SL630P**	-2.24	1.28E-01	1.69E-01
**RP11-800A3.3**	-1.46	1.28E-01	1.69E-01
**RP11-455F5.6**	1.21	1.28E-01	1.69E-01
**RP11-759A24.3**	1.70	1.28E-01	1.69E-01
**AC005562.1**	-0.85	1.28E-01	1.69E-01
**AC003682.17**	1.66	1.28E-01	1.69E-01
**FEM1C**	-0.20	1.29E-01	1.69E-01
**AC016586.1**	-0.67	1.29E-01	1.69E-01
**RAI2**	0.56	1.29E-01	1.69E-01
**PZP**	-1.01	1.29E-01	1.69E-01
**MTIF2**	0.17	1.29E-01	1.69E-01
**NLRP2**	0.98	1.29E-01	1.69E-01
**GABRR2**	-1.20	1.29E-01	1.69E-01

**RP11-57H14.3**	-2.09	1.29E-01	1.69E-01
**SPC25**	0.41	1.29E-01	1.70E-01
**EPB41L2**	0.40	1.29E-01	1.70E-01
**ERCC5**	-0.15	1.29E-01	1.70E-01
**MEI4**	-1.15	1.29E-01	1.70E-01
**TNRC6A**	-0.16	1.29E-01	1.70E-01
**TUSC8**	1.83	1.29E-01	1.70E-01
**CH17-174L20.1**	-2.02	1.29E-01	1.70E-01
**RP11-439L18.1**	1.31	1.29E-01	1.70E-01
**SEPT7P3**	-2.68	1.29E-01	1.70E-01
**CTD-2015B23.2**	-1.71	1.29E-01	1.70E-01
**AC073415.2**	-0.81	1.29E-01	1.70E-01
**ADIRF-AS1**	-0.51	1.29E-01	1.70E-01
**ASS1P2**	-0.88	1.29E-01	1.70E-01
**ARHGEF35**	0.65	1.29E-01	1.70E-01
**MIR3677**	-1.49	1.29E-01	1.70E-01
**SPRR2B**	1.53	1.29E-01	1.70E-01
**BBOX1-AS1**	-0.60	1.29E-01	1.70E-01
**RP11-176H8.3**	-1.35	1.29E-01	1.70E-01
**RP5-1039K5.19**	-0.36	1.29E-01	1.70E-01
**RP11-213G2.2**	-2.13	1.30E-01	1.70E-01
**RP11-4C20.3**	-1.72	1.30E-01	1.70E-01
**DDX39B**	0.57	1.30E-01	1.70E-01
**WBSCR27**	0.87	1.30E-01	1.70E-01
**RP11-622A1.2**	-2.05	1.30E-01	1.70E-01
**XLOC_010367**	1.48	1.30E-01	1.70E-01
**FYCO1**	-0.20	1.30E-01	1.70E-01
**DCLRE1C**	-0.16	1.30E-01	1.71E-01
**AUNIP**	-0.45	1.30E-01	1.71E-01
**AC018717.1**	-2.81	1.30E-01	1.71E-01
**LCE1D**	1.09	1.30E-01	1.71E-01
**RP11-362L22.1**	-1.58	1.30E-01	1.71E-01
**PKP2**	0.54	1.30E-01	1.71E-01
**G41428**	-1.56	1.30E-01	1.71E-01
**SARS2**	-0.61	1.30E-01	1.71E-01
**RAB38**	0.24	1.30E-01	1.71E-01
**RP11-351C21.2**	-0.75	1.30E-01	1.71E-01
**TMCC2**	0.38	1.30E-01	1.71E-01
**WEE2**	-0.88	1.30E-01	1.71E-01
**CHRNA3**	0.87	1.30E-01	1.71E-01
**RPL4**	0.24	1.31E-01	1.71E-01
**ZSWIM5**	-0.32	1.31E-01	1.71E-01
**WASF3**	0.32	1.31E-01	1.71E-01
**RAB28P2**	-1.63	1.31E-01	1.72E-01
**GCA**	0.31	1.31E-01	1.72E-01

**RP11-318A15.2**	-1.42	1.31E-01	1.72E-01
**RP11-119B16.2**	0.69	1.31E-01	1.72E-01
**RPL7AP11**	-1.40	1.31E-01	1.72E-01
**RP11-284P20.3**	-1.98	1.31E-01	1.72E-01
**COMMD2**	-0.24	1.31E-01	1.72E-01
**TMEM27**	-0.58	1.31E-01	1.72E-01
**LAMC2**	-0.40	1.31E-01	1.72E-01
**AC012074.2**	-0.62	1.31E-01	1.72E-01
**TNNT2**	-0.50	1.31E-01	1.72E-01
**RP5-963E22.6**	1.56	1.31E-01	1.72E-01
**OSBP**	-0.15	1.31E-01	1.72E-01
**INSR**	0.22	1.31E-01	1.73E-01
**CLEC4GP1**	1.31	1.32E-01	1.73E-01
**TMEM9B-AS1**	-0.42	1.32E-01	1.73E-01
**CTD-2035E11.3**	-1.36	1.32E-01	1.73E-01
**RP11-36B15.1**	-1.03	1.32E-01	1.73E-01
**RP11-354M1.2**	-1.18	1.32E-01	1.73E-01
**RP1-28H20.3**	-0.89	1.32E-01	1.73E-01
**KLHL7-AS1**	-0.73	1.32E-01	1.73E-01
**AC007041.2**	-1.59	1.32E-01	1.73E-01
**XLOC_009868**	0.73	1.32E-01	1.73E-01
**DCAF6**	0.13	1.32E-01	1.73E-01
**VCAN-AS1**	-1.38	1.32E-01	1.73E-01
**RNU6-1091P**	-1.35	1.32E-01	1.73E-01
**ADAM33**	0.62	1.32E-01	1.73E-01
**RP11-266L9.6**	-0.78	1.32E-01	1.73E-01
**AC093642.1**	-1.06	1.32E-01	1.73E-01
**RN7SL403P**	-1.66	1.32E-01	1.73E-01
**ALMS1P**	-1.27	1.32E-01	1.74E-01
**RP11-1C8.6**	-1.05	1.32E-01	1.74E-01
**CFAP157**	-0.40	1.32E-01	1.74E-01
**CBLN2**	-1.67	1.32E-01	1.74E-01
**ZNF34**	-0.24	1.32E-01	1.74E-01
**CYFIP1**	0.12	1.33E-01	1.74E-01
**SOX30**	1.05	1.33E-01	1.74E-01
**KRT18P5**	-1.17	1.33E-01	1.74E-01
**PCNAP4**	-1.48	1.33E-01	1.74E-01
**RP11-589M4.4**	-2.03	1.33E-01	1.74E-01
**RPS7**	0.26	1.33E-01	1.74E-01
**RARS2**	0.14	1.33E-01	1.74E-01
**G32232**	1.57	1.33E-01	1.74E-01
**CTD-2349P21.11**	-1.46	1.33E-01	1.74E-01
**KIF6**	-0.74	1.33E-01	1.74E-01
**DYRK4**	0.18	1.33E-01	1.74E-01
**SLC35B3**	0.19	1.33E-01	1.74E-01

**CTB-41I6.2**	2.05	1.33E-01	1.74E-01
**C11orf87**	-1.03	1.33E-01	1.74E-01
**RP4-605O3.4**	-0.45	1.33E-01	1.74E-01
**LRRC8E**	-0.34	1.33E-01	1.74E-01
**SF3B3**	-0.15	1.33E-01	1.74E-01
**PRKX**	-0.18	1.33E-01	1.74E-01
**G32932**	-1.34	1.33E-01	1.74E-01
**AP002381.2**	-1.66	1.33E-01	1.74E-01
**PDZRN3**	0.43	1.33E-01	1.74E-01
**G30301**	-2.21	1.33E-01	1.74E-01
**C11orf85**	-1.12	1.33E-01	1.74E-01
**RPH3AL**	0.35	1.33E-01	1.74E-01
**NPM1P6**	-0.74	1.33E-01	1.74E-01
**USP2**	-0.42	1.33E-01	1.74E-01
**AC093668.2**	0.86	1.33E-01	1.75E-01
**RPS15AP6**	-1.44	1.33E-01	1.75E-01
**MTHFD2**	0.33	1.33E-01	1.75E-01
**FCRL5**	1.45	1.33E-01	1.75E-01
**RP11-352D13.6**	-1.55	1.34E-01	1.75E-01
**ART3**	-0.86	1.34E-01	1.75E-01
**CNR2**	-1.64	1.34E-01	1.75E-01
**MAN2C1**	0.37	1.34E-01	1.75E-01
**ITIH6**	1.26	1.34E-01	1.75E-01
**ZNF587P1**	-1.18	1.34E-01	1.75E-01
**ZEB2**	0.41	1.34E-01	1.75E-01
**RECK**	-0.57	1.34E-01	1.75E-01
**RP11-626E13.1**	-2.07	1.34E-01	1.75E-01
**C3orf14**	0.26	1.34E-01	1.75E-01
**RP1-228P16.8**	-0.64	1.34E-01	1.75E-01
**RP11-375H19.2**	-1.05	1.34E-01	1.75E-01
**CAPN14**	-0.49	1.34E-01	1.75E-01
**ANKRD20A17P**	1.93	1.34E-01	1.76E-01
**CICP22**	-1.85	1.34E-01	1.76E-01
**GSPT2**	0.30	1.34E-01	1.76E-01
**SHISA3**	1.28	1.34E-01	1.76E-01
**NCK1**	-0.22	1.34E-01	1.76E-01
**TOB1-AS1**	-0.61	1.34E-01	1.76E-01
**PGM5P4**	-0.86	1.34E-01	1.76E-01
**RP11-517O13.3**	-0.93	1.34E-01	1.76E-01
**CDS2**	-0.19	1.34E-01	1.76E-01
**XLOC_005778**	1.29	1.35E-01	1.76E-01
**RP11-482E14.1**	-1.02	1.35E-01	1.76E-01
**RP11-717D12.1**	-0.97	1.35E-01	1.76E-01
**RP11-471B22.3**	-0.57	1.35E-01	1.76E-01
**XLOC_007942**	-0.81	1.35E-01	1.76E-01

**ZNF287**	-0.22	1.35E-01	1.76E-01
**RP1-140A9.1**	-0.70	1.35E-01	1.76E-01
**P2RX5**	-1.91	1.35E-01	1.76E-01
**ATP5J2**	0.21	1.35E-01	1.76E-01
**CTB-96E2.10**	-1.79	1.35E-01	1.76E-01
**RPS3AP12**	-1.13	1.35E-01	1.76E-01
**TCEB3**	-0.17	1.35E-01	1.76E-01
**CTD-2192J16.15**	-0.37	1.35E-01	1.76E-01
**RP11-120M18.5**	1.84	1.35E-01	1.77E-01
**RNU2-28P**	-0.81	1.35E-01	1.77E-01
**DDT**	-0.77	1.35E-01	1.77E-01
**SERF2**	0.23	1.35E-01	1.77E-01
**CLCA3P**	-1.35	1.35E-01	1.77E-01
**XLOC_007109**	-1.38	1.35E-01	1.77E-01
**TRHDE-AS1**	-1.00	1.35E-01	1.77E-01
**RP11-465N4.5**	-0.86	1.35E-01	1.77E-01
**BMPR1B**	0.33	1.36E-01	1.77E-01
**UAP1**	0.31	1.36E-01	1.77E-01
**KCTD14**	1.13	1.36E-01	1.77E-01
**DPP10-AS1**	-2.15	1.36E-01	1.77E-01
**HSP90AA1**	0.29	1.36E-01	1.77E-01
**RP11-736K20.5**	-0.67	1.36E-01	1.77E-01
**RP5-1050E16.2**	-2.01	1.36E-01	1.78E-01
**ST6GALNAC2**	-0.27	1.36E-01	1.78E-01
**HSD17B4**	-0.21	1.36E-01	1.78E-01
**AC090945.1**	-0.87	1.36E-01	1.78E-01
**SLC6A15**	-0.41	1.36E-01	1.78E-01
**ZNF259P1**	-1.05	1.36E-01	1.78E-01
**RP11-867G2.6**	-1.35	1.36E-01	1.78E-01
**RP11-21L23.4**	-1.77	1.36E-01	1.78E-01
**BTD**	0.27	1.36E-01	1.78E-01
**RP11-861E21.1**	-1.51	1.36E-01	1.78E-01
**C1orf228**	0.88	1.36E-01	1.78E-01
**SNRPCP19**	-1.55	1.36E-01	1.78E-01
**RNA5SP507**	-1.98	1.36E-01	1.78E-01
**SLFN12**	0.40	1.36E-01	1.78E-01
**G9256**	-1.22	1.37E-01	1.78E-01
**RP11-231G3.1**	-0.62	1.37E-01	1.78E-01
**CSNK2A2**	-0.28	1.37E-01	1.79E-01
**XLOC_005724**	0.39	1.37E-01	1.79E-01
**ST7-AS1**	0.55	1.37E-01	1.79E-01
**EVI2B**	0.56	1.37E-01	1.79E-01
**GNS**	0.24	1.37E-01	1.79E-01
**MCFD2P1**	-1.41	1.37E-01	1.79E-01
**RP11-316M1.3**	-1.58	1.37E-01	1.79E-01

**FILIP1L**	0.27	1.37E-01	1.79E-01
**CTD-2319I12.4**	0.83	1.37E-01	1.79E-01
**RP1-241P17.4**	1.03	1.37E-01	1.79E-01
**TRAJ5**	-2.46	1.37E-01	1.79E-01
**CTD-2336O2.3**	-1.04	1.37E-01	1.79E-01
**CEP135**	-0.21	1.37E-01	1.79E-01
**DGKZP1**	0.61	1.37E-01	1.79E-01
**RP4-728D4.2**	-0.77	1.37E-01	1.79E-01
**ANAPC10**	-0.20	1.37E-01	1.79E-01
**LRIT3**	-0.80	1.37E-01	1.79E-01
**MUC4**	-0.91	1.37E-01	1.79E-01
**RP11-667K14.3**	-0.42	1.37E-01	1.79E-01
**CAPN9**	0.99	1.37E-01	1.79E-01
**GAPDHP1**	-1.25	1.37E-01	1.79E-01
**RPL7P19**	-1.50	1.37E-01	1.79E-01
**LOX**	0.67	1.37E-01	1.79E-01
**XLOC_008033**	-0.43	1.37E-01	1.79E-01
**XLOC_011537**	-1.93	1.38E-01	1.79E-01
**RP11-506F3.1**	-1.45	1.38E-01	1.79E-01
**SH2D1A**	0.68	1.38E-01	1.79E-01
**DOCK11**	-0.43	1.38E-01	1.80E-01
**RP11-10J21.5**	-2.05	1.38E-01	1.80E-01
**RP11-395P16.1**	-2.00	1.38E-01	1.80E-01
**DYDC2**	1.08	1.38E-01	1.80E-01
**CTB-26E19.1**	-2.02	1.38E-01	1.80E-01
**RPL21P123**	-1.18	1.38E-01	1.80E-01
**RFWD2P1**	-2.16	1.38E-01	1.80E-01
**RP11-77H9.2**	-0.59	1.38E-01	1.80E-01
**AC098824.6**	-0.96	1.38E-01	1.80E-01
**LINC01504**	-0.59	1.38E-01	1.80E-01
**GXYLT1P6**	-1.55	1.38E-01	1.80E-01
**CPA4**	0.36	1.38E-01	1.80E-01
**EIF4HP2**	-0.87	1.38E-01	1.80E-01
**TMEM86A**	0.42	1.38E-01	1.80E-01
**TAS2R67P**	-1.45	1.38E-01	1.81E-01
**LARGE**	0.24	1.38E-01	1.81E-01
**C11orf58**	0.20	1.39E-01	1.81E-01
**ASS1P1**	-1.69	1.39E-01	1.81E-01
**EEF1B2P3**	-0.35	1.39E-01	1.81E-01
**RP11-44F21.2**	-1.72	1.39E-01	1.81E-01
**AC145124.2**	0.74	1.39E-01	1.81E-01
**FTH1P7**	1.27	1.39E-01	1.81E-01
**RP11-464D20.6**	-0.83	1.39E-01	1.81E-01
**CTC-499B15.7**	-1.98	1.39E-01	1.81E-01
**NUDT11**	-0.43	1.39E-01	1.81E-01

**ADAM21**	-0.98	1.39E-01	1.81E-01
**RP11-73M7.6**	0.58	1.39E-01	1.81E-01
**C21orf59**	0.21	1.39E-01	1.81E-01
**RP11-158H5.8**	-0.87	1.39E-01	1.81E-01
**COL6A4P1**	1.17	1.39E-01	1.81E-01
**RP11-673E1.3**	0.75	1.39E-01	1.81E-01
**MOBP**	-2.02	1.39E-01	1.81E-01
**ZNF662**	-0.31	1.39E-01	1.82E-01
**TJP2**	0.23	1.40E-01	1.82E-01
**CYP21A1P**	-1.80	1.40E-01	1.82E-01
**FBXO45**	0.39	1.40E-01	1.82E-01
**LINC00605**	1.56	1.40E-01	1.82E-01
**FANCD2OS**	-1.80	1.40E-01	1.82E-01
**TDH**	1.33	1.40E-01	1.82E-01
**AC011498.1**	-1.10	1.40E-01	1.82E-01
**E2F6**	-0.18	1.40E-01	1.82E-01
**EPYC**	5.94	1.40E-01	1.82E-01
**RP11-403I13.9**	-1.75	1.40E-01	1.82E-01
**FREM3**	-0.82	1.40E-01	1.82E-01
**MC1R**	0.61	1.40E-01	1.82E-01
**TATDN1**	-0.19	1.40E-01	1.83E-01
**RP11-343H5.4**	1.59	1.40E-01	1.83E-01
**RP11-599B13.7**	1.24	1.40E-01	1.83E-01
**RP11-371A22.1**	0.30	1.40E-01	1.83E-01
**AC005519.4**	-0.67	1.40E-01	1.83E-01
**LRIF1**	0.25	1.40E-01	1.83E-01
**CACNA1C-AS4**	-1.51	1.41E-01	1.83E-01
**CLDN19**	-1.60	1.41E-01	1.83E-01
**RP11-54O15.3**	-1.62	1.41E-01	1.83E-01
**CD5L**	-1.33	1.41E-01	1.83E-01
**FRG1JP**	-0.58	1.41E-01	1.83E-01
**RP11-13J10.1**	-1.49	1.41E-01	1.83E-01
**RP11-276H7.2**	0.59	1.41E-01	1.83E-01
**RP11-177H13.2**	-0.73	1.41E-01	1.83E-01
**R3HCC1L**	0.15	1.41E-01	1.83E-01
**CRNDE**	0.24	1.41E-01	1.83E-01
**FAM90A1**	0.67	1.41E-01	1.83E-01
**G3670**	1.19	1.41E-01	1.83E-01
**IRS1**	-0.36	1.41E-01	1.83E-01
**L3MBTL2**	0.15	1.41E-01	1.84E-01
**RP11-588H23.3**	-0.83	1.41E-01	1.84E-01
**RP11-973H7.1**	1.63	1.41E-01	1.84E-01
**RP11-501C14.9**	-1.05	1.41E-01	1.84E-01
**RP11-258F22.2**	-1.88	1.41E-01	1.84E-01
**RP11-499F3.2**	-1.87	1.41E-01	1.84E-01

**AC106786.1**	1.03	1.41E-01	1.84E-01
**CTB-49A3.2**	0.64	1.41E-01	1.84E-01
**CECR5-AS1**	-0.66	1.42E-01	1.84E-01
**SMC2-AS1**	-0.80	1.42E-01	1.84E-01
**GGTA2P**	-0.84	1.42E-01	1.84E-01
**ARHGEF39**	-0.45	1.42E-01	1.84E-01
**VAV3-AS1**	-1.57	1.42E-01	1.84E-01
**MIR26A2**	-1.21	1.42E-01	1.85E-01
**RP1-290F12.3**	-2.37	1.42E-01	1.85E-01
**AP001412.1**	-1.42	1.42E-01	1.85E-01
**AC027612.1**	1.00	1.42E-01	1.85E-01
**G6796**	-0.76	1.42E-01	1.85E-01
**RNU6-833P**	-1.86	1.42E-01	1.85E-01
**PTH2R**	1.11	1.42E-01	1.85E-01
**G26809**	-1.33	1.42E-01	1.85E-01
**RP11-73M18.8**	0.18	1.42E-01	1.85E-01
**RNU6ATAC18P**	-1.30	1.42E-01	1.85E-01
**RNU1-67P**	1.11	1.42E-01	1.85E-01
**AC002480.3**	1.28	1.42E-01	1.85E-01
**RP11-274H2.3**	-1.30	1.43E-01	1.85E-01
**AP1S2**	-0.25	1.43E-01	1.85E-01
**ASB16-AS1**	0.31	1.43E-01	1.85E-01
**MIR4804**	-1.93	1.43E-01	1.85E-01
**ETV2**	0.37	1.43E-01	1.86E-01
**ESR1**	-0.51	1.43E-01	1.86E-01
**FGFR3**	-0.27	1.43E-01	1.86E-01
**XLOC_003839**	-2.52	1.43E-01	1.86E-01
**ATP6V1G1P4**	-1.36	1.43E-01	1.86E-01
**SIRT5**	-0.21	1.43E-01	1.86E-01
**CTD-3064M3.7**	1.28	1.43E-01	1.86E-01
**CHRNB2**	-0.45	1.43E-01	1.86E-01
**RNA5SP194**	-2.20	1.43E-01	1.86E-01
**AP3B1**	-0.17	1.43E-01	1.86E-01
**KIAA0319**	0.44	1.43E-01	1.86E-01
**NUTM2A-AS1**	-0.42	1.43E-01	1.86E-01
**NALCN**	0.66	1.43E-01	1.86E-01
**CSDE1**	-0.19	1.43E-01	1.86E-01
**VPS53**	-0.21	1.44E-01	1.86E-01
**AC006116.17**	-1.88	1.44E-01	1.86E-01
**RP13-228J13.5**	-1.48	1.44E-01	1.86E-01
**OGDH**	0.17	1.44E-01	1.87E-01
**RP11-468E2.5**	0.80	1.44E-01	1.87E-01
**C9orf50**	1.50	1.44E-01	1.87E-01
**XLOC_007690**	0.58	1.44E-01	1.87E-01
**CLCNKB**	1.06	1.44E-01	1.87E-01

**SEC22C**	0.19	1.44E-01	1.87E-01
**RP11-43F13.4**	0.78	1.44E-01	1.87E-01
**MCF2L2**	-0.40	1.44E-01	1.87E-01
**BCCIP**	0.19	1.44E-01	1.87E-01
**MIR5194**	-1.58	1.44E-01	1.87E-01
**AF196970.3**	-2.28	1.44E-01	1.87E-01
**RP5-903G2.2**	0.71	1.44E-01	1.87E-01
**RP11-159L20.2**	-1.63	1.44E-01	1.87E-01
**RP11-661A12.9**	-0.78	1.44E-01	1.87E-01
**XLOC_004229**	-2.35	1.44E-01	1.87E-01
**GOLGA1**	-0.14	1.44E-01	1.87E-01
**GART**	0.16	1.44E-01	1.87E-01
**RP11-10A14.3**	0.89	1.44E-01	1.87E-01
**XLOC_013499**	0.42	1.45E-01	1.88E-01
**KB-1125A3.12**	-0.91	1.45E-01	1.88E-01
**GAPDHP52**	-1.22	1.45E-01	1.88E-01
**NLRC5**	-0.30	1.45E-01	1.88E-01
**LINC01451**	-0.93	1.45E-01	1.88E-01
**RP11-485M7.3**	0.67	1.45E-01	1.88E-01
**GNRHR2**	0.37	1.45E-01	1.88E-01
**AC012442.6**	-0.63	1.45E-01	1.88E-01
**AC034243.1**	-2.01	1.45E-01	1.88E-01
**AATBC**	-0.58	1.45E-01	1.88E-01
**DPY19L1P1**	-0.37	1.45E-01	1.88E-01
**RP11-462G2.2**	-1.79	1.45E-01	1.88E-01
**GPR171**	-0.79	1.45E-01	1.88E-01
**RPS19P3**	0.80	1.45E-01	1.88E-01
**SLCO4A1**	-0.30	1.45E-01	1.89E-01
**RP11-5N11.5**	-2.24	1.45E-01	1.89E-01
**KAT8**	0.17	1.45E-01	1.89E-01
**NAALADL2-AS2**	-1.83	1.46E-01	1.89E-01
**AC012363.8**	-1.33	1.46E-01	1.89E-01
**YME1L1**	-0.18	1.46E-01	1.89E-01
**HIST2H2BC**	-0.59	1.46E-01	1.89E-01
**NBPF2P**	-0.73	1.46E-01	1.89E-01
**XRCC6P2**	-1.46	1.46E-01	1.89E-01
**MIR581**	-1.39	1.46E-01	1.89E-01
**RP11-230B22.1**	-0.78	1.46E-01	1.89E-01
**HNRNPA3P12**	-1.62	1.46E-01	1.89E-01
**G12287**	-0.92	1.46E-01	1.89E-01
**USP9Y**	-1.53	1.46E-01	1.89E-01
**RP11-1277A3.1**	0.43	1.46E-01	1.89E-01
**POLR2KP1**	-1.35	1.46E-01	1.89E-01
**SYTL1**	0.30	1.46E-01	1.89E-01
**RP11-561N12.5**	-1.34	1.46E-01	1.90E-01

**KIF25**	0.82	1.46E-01	1.90E-01
**RP11-387H17.4**	-0.80	1.46E-01	1.90E-01
**C15orf54**	1.08	1.46E-01	1.90E-01
**SYNGR1**	0.25	1.46E-01	1.90E-01
**MIR378A**	-1.79	1.46E-01	1.90E-01
**AC010890.1**	-0.85	1.47E-01	1.90E-01
**WDR37**	0.15	1.47E-01	1.90E-01
**RGP1**	-0.14	1.47E-01	1.90E-01
**HLA-T**	-2.22	1.47E-01	1.90E-01
**FAM21C**	0.18	1.47E-01	1.90E-01
**MROH9**	-0.97	1.47E-01	1.90E-01
**EAPP**	0.26	1.47E-01	1.90E-01
**RP11-366L20.2**	1.04	1.47E-01	1.90E-01
**INMT**	0.52	1.47E-01	1.90E-01
**ARMCX1**	0.27	1.47E-01	1.90E-01
**SFI1**	-0.28	1.47E-01	1.90E-01
**RP11-349N19.2**	1.28	1.47E-01	1.91E-01
**CALM2P3**	-1.69	1.47E-01	1.91E-01
**FBXL18**	0.29	1.47E-01	1.91E-01
**G24100**	-0.91	1.47E-01	1.91E-01
**XLOC_004195**	-0.66	1.47E-01	1.91E-01
**L1TD1**	1.33	1.47E-01	1.91E-01
**ABHD3**	-0.26	1.48E-01	1.91E-01
**MKRN2**	0.16	1.48E-01	1.91E-01
**IFNAR1**	-0.16	1.48E-01	1.91E-01
**ZKSCAN5**	0.11	1.48E-01	1.91E-01
**AC004840.8**	-1.73	1.48E-01	1.91E-01
**LARS2**	0.15	1.48E-01	1.91E-01
**CTD-2002J20.1**	-1.01	1.48E-01	1.91E-01
**XXbac-BPG299F13.14**	-1.44	1.48E-01	1.91E-01
**RP11-553A21.3**	-0.39	1.48E-01	1.92E-01
**AC115522.3**	-0.38	1.48E-01	1.92E-01
**LINC01411**	1.05	1.48E-01	1.92E-01
**TCHP**	-0.22	1.48E-01	1.92E-01
**RP11-283G6.4**	-0.54	1.48E-01	1.92E-01
**MRPS22**	-0.16	1.48E-01	1.92E-01
**RP11-114H23.1**	-1.60	1.48E-01	1.92E-01
**RP11-809N8.4**	1.37	1.48E-01	1.92E-01
**RP3-400B16.1**	0.85	1.48E-01	1.92E-01
**LINC00518**	-0.69	1.49E-01	1.92E-01
**TRAF1**	-0.34	1.49E-01	1.92E-01
**GALNT13**	-0.74	1.49E-01	1.92E-01
**XCL2**	1.38	1.49E-01	1.92E-01
**BTBD7P1**	-0.90	1.49E-01	1.92E-01
**RP11-200A1.1**	-1.26	1.49E-01	1.92E-01

**PNO1**	-0.21	1.49E-01	1.92E-01
**RP11-368J21.3**	1.26	1.49E-01	1.92E-01
**G27536**	-1.14	1.49E-01	1.93E-01
**SPOCK3**	-2.20	1.49E-01	1.93E-01
**SPIN2B**	0.28	1.49E-01	1.93E-01
**RP3-510O8.4**	-1.42	1.49E-01	1.93E-01
**LYRM5**	-0.29	1.49E-01	1.93E-01
**RSAD2**	0.97	1.49E-01	1.93E-01
**KIF23**	-0.29	1.49E-01	1.93E-01
**TAOK3**	0.12	1.49E-01	1.93E-01
**PSMA6P2**	-1.52	1.49E-01	1.93E-01
**AC024084.1**	-2.17	1.50E-01	1.93E-01
**RP11-358B23.6**	-1.09	1.50E-01	1.93E-01
**KRTAP3-1**	-2.56	1.50E-01	1.93E-01
**CTB-39G8.3**	-1.15	1.50E-01	1.93E-01
**KIF5C**	-0.41	1.50E-01	1.93E-01
**AC002310.14**	0.36	1.50E-01	1.94E-01
**STRIP1**	0.13	1.50E-01	1.94E-01
**SNRPE**	0.27	1.50E-01	1.94E-01
**ADK**	-0.22	1.50E-01	1.94E-01
**RP11-677M14.7**	-0.61	1.50E-01	1.94E-01
**AF131215.8**	-0.55	1.50E-01	1.94E-01
**CCDC108**	-1.63	1.50E-01	1.94E-01
**RP11-1182P23.5**	-1.61	1.50E-01	1.94E-01
**POLR1E**	0.17	1.50E-01	1.94E-01
**KLF14**	-1.15	1.50E-01	1.94E-01
**RP11-44M6.7**	0.94	1.50E-01	1.94E-01
**AC156455.1**	1.00	1.50E-01	1.94E-01
**CTD-3162L10.1**	-0.77	1.50E-01	1.94E-01
**LINC00520**	1.12	1.50E-01	1.94E-01
**LINC01239**	-1.13	1.50E-01	1.94E-01
**RP11-84C10.2**	-1.05	1.50E-01	1.94E-01
**MOG**	-2.45	1.50E-01	1.94E-01
**SEPT7-AS1**	0.36	1.51E-01	1.94E-01
**G35131**	1.46	1.51E-01	1.95E-01
**LINC00113**	-1.31	1.51E-01	1.95E-01
**TUBA1A**	0.28	1.51E-01	1.95E-01
**BCL2L14**	-0.72	1.51E-01	1.95E-01
**XLOC_011980**	5.73	1.51E-01	1.95E-01
**TADA2A**	-0.19	1.51E-01	1.95E-01
**RP11-806O11.1**	-0.95	1.51E-01	1.95E-01
**COG2**	-0.10	1.51E-01	1.95E-01
**CCNB2**	0.30	1.51E-01	1.95E-01
**RP11-146F11.5**	-0.95	1.51E-01	1.95E-01
**LINC01343**	0.64	1.51E-01	1.95E-01

**ZNF2**	-0.21	1.51E-01	1.95E-01
**RP11-736K20.4**	0.65	1.51E-01	1.95E-01
**RP5-1148A21.3**	0.34	1.51E-01	1.95E-01
**CTSE**	-1.24	1.51E-01	1.95E-01
**RP11-338N10.3**	-1.95	1.51E-01	1.95E-01
**ZBP1**	0.91	1.51E-01	1.95E-01
**PCDHB12**	0.47	1.51E-01	1.95E-01
**RP11-217B1.2**	-0.69	1.51E-01	1.95E-01
**SEC61A2**	-0.36	1.51E-01	1.95E-01
**SPTB**	-0.45	1.51E-01	1.95E-01
**ADAMTS6**	0.56	1.51E-01	1.95E-01
**MYOM1**	0.49	1.51E-01	1.95E-01
**ATOH7**	1.06	1.51E-01	1.95E-01
**C18orf63**	-2.20	1.51E-01	1.95E-01
**RP11-432F4.2**	-1.71	1.51E-01	1.95E-01
**DLX3**	0.36	1.52E-01	1.96E-01
**OR8R1P**	-1.87	1.52E-01	1.96E-01
**MROH3P**	-0.77	1.52E-01	1.96E-01
**PSMB2**	0.21	1.52E-01	1.96E-01
**KMT2D**	-0.38	1.52E-01	1.96E-01
**PTPRD-AS1**	1.31	1.52E-01	1.96E-01
**SLIT2**	0.55	1.52E-01	1.96E-01
**RP11-38M8.1**	1.44	1.52E-01	1.96E-01
**G3630**	0.45	1.52E-01	1.96E-01
**G23016**	-1.06	1.52E-01	1.96E-01
**AQP6**	1.39	1.52E-01	1.96E-01
**HNRNPH1**	-0.25	1.52E-01	1.96E-01
**SHCBP1**	0.33	1.52E-01	1.96E-01
**OXCT2**	-0.75	1.52E-01	1.96E-01
**ADAM10**	-0.17	1.52E-01	1.96E-01
**AACSP1**	0.97	1.52E-01	1.96E-01
**A1BG**	-1.36	1.52E-01	1.96E-01
**METTL17**	0.27	1.52E-01	1.96E-01
**RP4-584D14.6**	-1.67	1.52E-01	1.96E-01
**IDI1**	-0.41	1.52E-01	1.96E-01
**LNP1**	0.41	1.52E-01	1.97E-01
**G30008**	1.31	1.53E-01	1.97E-01
**TPSG1**	-1.06	1.53E-01	1.97E-01
**RP11-553A10.1**	1.09	1.53E-01	1.97E-01
**XRCC4**	0.26	1.53E-01	1.97E-01
**RP11-264B17.2**	-0.75	1.53E-01	1.97E-01
**RRP36**	0.20	1.53E-01	1.97E-01
**RP11-619A14.3**	1.05	1.53E-01	1.97E-01
**RABIF**	0.22	1.53E-01	1.97E-01
**RP11-529K1.2**	-0.51	1.53E-01	1.97E-01

**MGARP**	-1.16	1.53E-01	1.97E-01
**MYLK4**	-0.32	1.53E-01	1.97E-01
**RP11-50C13.1**	-0.42	1.53E-01	1.97E-01
**LENG8-AS1**	-0.48	1.53E-01	1.97E-01
**TOM1L2**	0.26	1.53E-01	1.97E-01
**USP10**	-0.12	1.53E-01	1.97E-01
**LINC01191**	-1.63	1.53E-01	1.97E-01
**AC046143.2**	-0.90	1.53E-01	1.97E-01
**KRTAP19-5**	-3.67	1.53E-01	1.97E-01
**VPS52**	0.19	1.53E-01	1.98E-01
**C14orf37**	-0.27	1.53E-01	1.98E-01
**GPR65**	0.75	1.53E-01	1.98E-01
**MEAF6**	0.16	1.53E-01	1.98E-01
**CTD-2270L9.4**	1.09	1.53E-01	1.98E-01
**ZEB1**	0.46	1.53E-01	1.98E-01
**LINC00302**	0.54	1.53E-01	1.98E-01
**G35720**	1.10	1.54E-01	1.98E-01
**RP11-326N17.2**	-1.98	1.54E-01	1.98E-01
**FRMPD3**	-0.80	1.54E-01	1.98E-01
**AGAP1**	0.20	1.54E-01	1.98E-01
**IGBP1-AS2**	-1.46	1.54E-01	1.98E-01
**IGLV1-40**	2.73	1.54E-01	1.98E-01
**LMO3**	-0.73	1.54E-01	1.98E-01
**RPL13AP25**	-0.32	1.54E-01	1.98E-01
**ALOX5**	0.44	1.54E-01	1.98E-01
**RP4-601P9.2**	-1.47	1.54E-01	1.98E-01
**LINC01336**	-0.51	1.54E-01	1.98E-01
**NKRF**	-0.16	1.54E-01	1.98E-01
**NOL4**	-1.33	1.54E-01	1.98E-01
**RP11-107N15.1**	0.83	1.54E-01	1.98E-01
**G41319**	0.38	1.54E-01	1.98E-01
**RP11-294N21.3**	-1.37	1.54E-01	1.99E-01
**XLOC_009705**	-1.62	1.54E-01	1.99E-01
**CCNI2**	-0.74	1.54E-01	1.99E-01
**C19orf35**	0.79	1.54E-01	1.99E-01
**ZNF266**	-0.21	1.54E-01	1.99E-01
**RAB11B-AS1**	0.34	1.55E-01	1.99E-01
**RP11-159M11.2**	-1.12	1.55E-01	1.99E-01
**RANP8**	-1.47	1.55E-01	1.99E-01
**WWOX**	0.20	1.55E-01	1.99E-01
**NUS1**	0.28	1.55E-01	1.99E-01
**SNW1**	0.19	1.55E-01	1.99E-01
**FUT4**	0.39	1.55E-01	1.99E-01
**HMCN1**	-0.51	1.55E-01	1.99E-01
**RP11-467I17.1**	1.41	1.55E-01	1.99E-01

**RP11-711K1.8**	-2.22	1.55E-01	2.00E-01
**SIGLEC15**	0.64	1.55E-01	2.00E-01
**GCGR**	1.00	1.55E-01	2.00E-01
**SNX15**	-0.64	1.55E-01	2.00E-01
**C14orf169**	0.21	1.55E-01	2.00E-01
**LGALS7**	1.39	1.55E-01	2.00E-01
**HLA-DOA**	0.53	1.55E-01	2.00E-01
**NDUFA6-AS1**	-0.38	1.56E-01	2.00E-01
**LPO**	-1.45	1.56E-01	2.00E-01
**CTD-2555C10.3**	0.78	1.56E-01	2.00E-01
**GALNT16**	-0.52	1.56E-01	2.00E-01
**PON2**	-0.18	1.56E-01	2.00E-01
**CBLN1**	1.39	1.56E-01	2.00E-01
**PPP1R21**	-0.17	1.56E-01	2.00E-01
**LETM1**	-0.20	1.56E-01	2.00E-01
**CAMK1G**	0.90	1.56E-01	2.00E-01
**TJP3**	0.36	1.56E-01	2.00E-01
**KRT27**	-2.09	1.56E-01	2.00E-01
**FERMT1**	-0.22	1.56E-01	2.00E-01
**KRT87P**	1.41	1.56E-01	2.00E-01
**LINC01352**	0.97	1.56E-01	2.00E-01
**MTND4P12**	-1.19	1.56E-01	2.00E-01
**AP000692.9**	0.57	1.56E-01	2.01E-01
**AC105339.1**	-1.89	1.56E-01	2.01E-01
**CTD-2192J16.11**	1.03	1.56E-01	2.01E-01
**CCL16**	1.31	1.56E-01	2.01E-01
**CTC-559E9.1**	-0.37	1.56E-01	2.01E-01
**AC068580.5**	0.60	1.56E-01	2.01E-01
**RP11-738E22.3**	-0.58	1.56E-01	2.01E-01
**ADGRD2**	-0.89	1.56E-01	2.01E-01
**CAD**	0.19	1.57E-01	2.01E-01
**KIAA0907**	-0.35	1.57E-01	2.01E-01
**OLFML2A**	0.21	1.57E-01	2.01E-01
**SYT15**	-0.39	1.57E-01	2.01E-01
**EWSAT1**	-0.90	1.57E-01	2.01E-01
**RP11-302K17.3**	-1.80	1.57E-01	2.02E-01
**PHACTR3**	-0.53	1.57E-01	2.02E-01
**KCNMA1-AS3**	-1.96	1.57E-01	2.02E-01
**RP5-907C10.3**	-0.51	1.57E-01	2.02E-01
**RP4-575N6.5**	1.46	1.57E-01	2.02E-01
**CAMK2A**	-0.53	1.57E-01	2.02E-01
**ANXA8L1**	-0.60	1.57E-01	2.02E-01
**RP11-514P8.2**	-1.39	1.57E-01	2.02E-01
**EMX2OS**	-0.37	1.57E-01	2.02E-01
**AC144449.1**	-1.32	1.57E-01	2.02E-01

**CTB-37A13.1**	-1.96	1.57E-01	2.02E-01
**RP3-527G5.1**	1.41	1.57E-01	2.02E-01
**ARL4AP2**	-1.06	1.57E-01	2.02E-01
**AP1M2**	0.26	1.57E-01	2.02E-01
**XLOC_010439**	-1.68	1.57E-01	2.02E-01
**OSER1-AS1**	0.26	1.58E-01	2.02E-01
**AGAP7P**	1.41	1.58E-01	2.03E-01
**URAHP**	0.82	1.58E-01	2.03E-01
**KRTAP10-2**	-2.34	1.58E-01	2.03E-01
**AGBL5-AS1**	1.20	1.58E-01	2.03E-01
**SLC26A9**	0.42	1.58E-01	2.03E-01
**CTD-2201I18.1**	-0.76	1.58E-01	2.03E-01
**PAFAH2**	-0.25	1.58E-01	2.03E-01
**RP11-95I16.4**	-1.49	1.58E-01	2.03E-01
**RN7SL80P**	-1.82	1.58E-01	2.03E-01
**ZMAT4**	1.84	1.58E-01	2.03E-01
**APOE**	0.38	1.59E-01	2.04E-01
**NME2**	0.51	1.59E-01	2.04E-01
**AC013463.2**	-1.09	1.59E-01	2.04E-01
**ALDH1A1**	-0.48	1.59E-01	2.04E-01
**FOCAD-AS1**	-1.22	1.59E-01	2.04E-01
**AC004947.2**	0.82	1.59E-01	2.04E-01
**PKNOX1**	0.18	1.59E-01	2.04E-01
**FBXW8**	0.18	1.59E-01	2.04E-01
**SPRR2F**	1.61	1.59E-01	2.04E-01
**RP3-393E18.2**	1.14	1.59E-01	2.04E-01
**AC092652.1**	-1.14	1.59E-01	2.05E-01
**HMGN1P28**	-2.05	1.59E-01	2.05E-01
**MAPT**	-0.47	1.60E-01	2.05E-01
**NANOGNBP3**	0.89	1.60E-01	2.05E-01
**RP11-613M10.6**	0.25	1.60E-01	2.05E-01
**RP5-1065P14.2**	1.97	1.60E-01	2.05E-01
**RP13-631K18.3**	-1.39	1.60E-01	2.05E-01
**KRTAP5-8**	-1.76	1.60E-01	2.05E-01
**EFR3B**	0.44	1.60E-01	2.05E-01
**RP5-1057I20.2**	0.75	1.60E-01	2.05E-01
**RP4-798A10.7**	-0.82	1.60E-01	2.05E-01
**RN7SL775P**	-1.06	1.60E-01	2.05E-01
**RP11-881L2.1**	-0.72	1.60E-01	2.05E-01
**G32307**	-1.00	1.60E-01	2.05E-01
**TMPRSS12**	0.91	1.60E-01	2.05E-01
**ST13P6**	-0.82	1.60E-01	2.06E-01
**TMEM106C**	-0.20	1.61E-01	2.06E-01
**RP5-1107A17.4**	-0.93	1.61E-01	2.06E-01
**RP11-367G18.2**	-1.67	1.61E-01	2.06E-01

**ULK4P2**	-1.33	1.61E-01	2.06E-01
**TTC9B**	0.79	1.61E-01	2.06E-01
**RP11-989E6.13**	0.76	1.61E-01	2.06E-01
**RP11-399K21.14**	0.86	1.61E-01	2.06E-01
**AIDA**	0.16	1.61E-01	2.06E-01
**EXTL2**	-0.25	1.61E-01	2.06E-01
**OLFML1**	0.54	1.61E-01	2.06E-01
**CTB-113D17.1**	-0.75	1.61E-01	2.06E-01
**TNFAIP3**	0.31	1.61E-01	2.06E-01
**CTD-2382E5.6**	-0.87	1.61E-01	2.06E-01
**C1orf134**	0.36	1.61E-01	2.06E-01
**NR4A3**	-0.49	1.61E-01	2.06E-01
**SH3BGRL**	0.26	1.61E-01	2.06E-01
**SSUH2**	0.92	1.61E-01	2.06E-01
**G35542**	-1.23	1.61E-01	2.07E-01
**CDT1**	0.27	1.61E-01	2.07E-01
**OR7C1**	-1.09	1.62E-01	2.07E-01
**RP11-334C17.5**	-0.61	1.62E-01	2.07E-01
**RP11-147L13.2**	0.94	1.62E-01	2.07E-01
**RP5-858L17.1**	-0.60	1.62E-01	2.07E-01
**MIR200C**	-1.72	1.62E-01	2.07E-01
**RP11-713C5.1**	-1.25	1.62E-01	2.08E-01
**LRRC1**	-0.21	1.62E-01	2.08E-01
**HMGB3P22**	-0.85	1.62E-01	2.08E-01
**VILL**	0.23	1.62E-01	2.08E-01
**LINC01224**	-0.65	1.62E-01	2.08E-01
**G35130**	1.22	1.62E-01	2.08E-01
**RP11-433J8.1**	-1.21	1.63E-01	2.08E-01
**CYP2D6**	-0.60	1.63E-01	2.08E-01
**TANGO6**	0.19	1.63E-01	2.08E-01
**CTC-529P8.1**	-1.08	1.63E-01	2.08E-01
**RP11-378A12.1**	-0.85	1.63E-01	2.08E-01
**RP11-632F7.3**	-1.55	1.63E-01	2.08E-01
**TUBA3FP**	1.24	1.63E-01	2.08E-01
**XLOC_012692**	0.38	1.63E-01	2.08E-01
**ARID3C**	-0.81	1.63E-01	2.08E-01
**SLC10A2**	-1.56	1.63E-01	2.08E-01
**RP11-12A2.1**	1.34	1.63E-01	2.09E-01
**SNCAIP**	0.50	1.63E-01	2.09E-01
**APBB2**	-0.17	1.63E-01	2.09E-01
**RP5-1185I7.1**	1.30	1.63E-01	2.09E-01
**PAX1**	1.55	1.63E-01	2.09E-01
**XLOC_014271**	-0.82	1.63E-01	2.09E-01
**RP11-342I1.2**	-0.67	1.63E-01	2.09E-01
**IL17RA**	0.17	1.64E-01	2.09E-01

**EEF1DP7**	-0.76	1.64E-01	2.09E-01
**SVIL-AS1**	0.18	1.64E-01	2.09E-01
**KCTD1**	0.27	1.64E-01	2.09E-01
**RP11-136C24.3**	0.90	1.64E-01	2.09E-01
**C4orf32**	0.28	1.64E-01	2.10E-01
**CSDAP1**	-1.99	1.64E-01	2.10E-01
**G39827**	1.17	1.64E-01	2.10E-01
**RP11-610J23.1**	-1.86	1.64E-01	2.10E-01
**RP11-311H10.4**	-1.83	1.64E-01	2.10E-01
**CAV3**	-1.94	1.64E-01	2.10E-01
**SPC24**	-0.30	1.64E-01	2.10E-01
**RP4-612C19.1**	-1.77	1.64E-01	2.10E-01
**AC138035.2**	-0.82	1.64E-01	2.10E-01
**RP11-83A24.1**	-0.75	1.64E-01	2.10E-01
**ARMC1**	-0.20	1.64E-01	2.10E-01
**SDHAF2**	0.19	1.64E-01	2.10E-01
**RNU6-703P**	1.51	1.64E-01	2.10E-01
**AC010761.8**	-0.34	1.65E-01	2.10E-01
**RP11-442J21.2**	1.71	1.65E-01	2.10E-01
**WDR27**	-0.38	1.65E-01	2.11E-01
**CTD-2530H12.4**	1.11	1.65E-01	2.11E-01
**AL022344.7**	1.02	1.65E-01	2.11E-01
**FTH1P2**	0.88	1.65E-01	2.11E-01
**RP11-229P13.25**	-0.99	1.65E-01	2.11E-01
**G113**	1.44	1.65E-01	2.11E-01
**CTD-2002H8.2**	-0.52	1.65E-01	2.11E-01
**PLA2G10**	-0.83	1.65E-01	2.11E-01
**HLA-DRB5**	-1.42	1.65E-01	2.11E-01
**EIF4EP2**	-0.67	1.65E-01	2.11E-01
**RP11-795F19.5**	-0.76	1.65E-01	2.11E-01
**RP11-454C18.1**	-1.05	1.65E-01	2.11E-01
**G11342**	-0.78	1.65E-01	2.11E-01
**SPDYC**	-1.59	1.66E-01	2.12E-01
**MED4**	-0.16	1.66E-01	2.12E-01
**CCER2**	1.24	1.66E-01	2.12E-01
**CTD-2538C1.2**	-0.81	1.66E-01	2.12E-01
**RNU7-162P**	-1.46	1.66E-01	2.12E-01
**CTC-338M12.4**	0.44	1.66E-01	2.12E-01
**RN7SL277P**	-1.59	1.66E-01	2.12E-01
**RP5-1009E24.8**	1.05	1.66E-01	2.13E-01
**AC068831.16**	-0.52	1.66E-01	2.13E-01
**RASSF5**	-0.26	1.66E-01	2.13E-01
**GGT6**	-0.24	1.67E-01	2.13E-01
**RP11-536C12.1**	-0.94	1.67E-01	2.13E-01
**SMIM11B**	-1.17	1.67E-01	2.13E-01

**PSPHP1**	3.29	1.67E-01	2.13E-01
**SLC6A20**	0.76	1.67E-01	2.13E-01
**RP11-529A4.7**	2.07	1.67E-01	2.13E-01
**GPBAR1**	-0.58	1.67E-01	2.13E-01
**SMG1P2**	0.33	1.67E-01	2.13E-01
**MAP3K14-AS1**	-0.54	1.67E-01	2.13E-01
**SVEP1**	-0.72	1.67E-01	2.13E-01
**RP1-20C7.6**	-0.90	1.67E-01	2.13E-01
**FBXO15**	0.80	1.67E-01	2.13E-01
**CTC-563A5.2**	-1.89	1.67E-01	2.13E-01
**RP11-359K18.4**	0.67	1.67E-01	2.13E-01
**RP11-79P5.10**	-1.86	1.67E-01	2.13E-01
**CLU**	0.52	1.67E-01	2.14E-01
**RP13-36G14.3**	-1.68	1.67E-01	2.14E-01
**MIR635**	0.53	1.67E-01	2.14E-01
**COX18**	-0.14	1.68E-01	2.14E-01
**AC092291.1**	-2.21	1.68E-01	2.14E-01
**CTD-2228K2.7**	0.61	1.68E-01	2.14E-01
**IKBKGP1**	1.11	1.68E-01	2.14E-01
**CTB-191K22.6**	-1.14	1.68E-01	2.14E-01
**MUC1**	1.28	1.68E-01	2.14E-01
**ADAM28**	0.51	1.68E-01	2.14E-01
**CDH2**	-0.69	1.68E-01	2.14E-01
**RP11-329B9.3**	1.09	1.68E-01	2.14E-01
**TP53TG3D**	1.30	1.68E-01	2.15E-01
**AGMO**	-0.86	1.68E-01	2.15E-01
**RNA5SP159**	-1.94	1.68E-01	2.15E-01
**RPL9P3**	-2.00	1.68E-01	2.15E-01
**RP1-199J3.6**	1.19	1.68E-01	2.15E-01
**HNRNPA1P27**	-0.48	1.68E-01	2.15E-01
**TNNC2**	0.43	1.68E-01	2.15E-01
**IGKV2-28**	2.65	1.68E-01	2.15E-01
**LINC01563**	1.47	1.68E-01	2.15E-01
**MAPK10**	-0.44	1.69E-01	2.15E-01
**RPL37P3**	-1.26	1.69E-01	2.15E-01
**DUSP2**	0.32	1.69E-01	2.15E-01
**ASPH**	-0.19	1.69E-01	2.15E-01
**CTD-2132N18.4**	0.81	1.69E-01	2.15E-01
**RPSAP70**	0.96	1.69E-01	2.15E-01
**GALNT3**	-0.23	1.69E-01	2.15E-01
**ASB9**	0.44	1.69E-01	2.15E-01
**CTD-2006M22.2**	0.83	1.69E-01	2.16E-01
**CTD-2538C1.3**	-1.29	1.69E-01	2.16E-01
**TPSD1**	1.48	1.69E-01	2.16E-01
**PTPRH**	-0.55	1.69E-01	2.16E-01

**ZDHHC5**	0.19	1.69E-01	2.16E-01
**FAM228B**	0.26	1.69E-01	2.16E-01
**RP11-379H18.1**	-0.22	1.69E-01	2.16E-01
**RP11-285G1.2**	-1.41	1.69E-01	2.16E-01
**TEF**	0.42	1.69E-01	2.16E-01
**POP4**	0.20	1.69E-01	2.16E-01
**G25761**	-1.03	1.69E-01	2.16E-01
**TMEM178B**	-0.48	1.70E-01	2.16E-01
**RP11-973D8.5**	-1.00	1.70E-01	2.16E-01
**DACH2**	-1.12	1.70E-01	2.16E-01
**EMC3-AS1**	-0.38	1.70E-01	2.16E-01
**CD70**	-0.88	1.70E-01	2.16E-01
**G24602**	-1.47	1.70E-01	2.17E-01
**LSM6**	0.18	1.70E-01	2.17E-01
**NAPB**	-0.16	1.70E-01	2.17E-01
**G26856**	-0.68	1.70E-01	2.17E-01
**RP6-74O6.6**	-0.66	1.70E-01	2.17E-01
**CATSPER3**	1.02	1.70E-01	2.17E-01
**RNF43**	-0.24	1.70E-01	2.17E-01
**CACNG4**	1.26	1.70E-01	2.17E-01
**AC004448.5**	1.35	1.70E-01	2.17E-01
**RP11-775B15.2**	-1.59	1.70E-01	2.17E-01
**PPID**	-0.22	1.70E-01	2.17E-01
**C22orf24**	-1.39	1.71E-01	2.17E-01
**TTTY15**	-1.09	1.71E-01	2.17E-01
**XLOC_008786**	-2.25	1.71E-01	2.17E-01
**TRH**	-1.39	1.71E-01	2.17E-01
**PIP4K2A**	0.25	1.71E-01	2.17E-01
**SDCBP**	0.25	1.71E-01	2.17E-01
**TXLNGY**	-1.47	1.71E-01	2.18E-01
**CTD-2192J16.21**	-0.88	1.71E-01	2.18E-01
**BCAS3**	-0.15	1.71E-01	2.18E-01
**RP11-136K14.1**	-1.29	1.71E-01	2.18E-01
**RP13-884E18.4**	-1.69	1.71E-01	2.18E-01
**TFAP2E**	0.35	1.71E-01	2.18E-01
**C11orf70**	-0.32	1.72E-01	2.18E-01
**RP11-627G18.1**	-2.04	1.72E-01	2.18E-01
**RP3-460G2.2**	-1.68	1.72E-01	2.18E-01
**KLF5**	-0.28	1.72E-01	2.19E-01
**BDNF-AS**	-0.33	1.72E-01	2.19E-01
**AL137059.1**	-0.70	1.72E-01	2.19E-01
**IGFL1P1**	0.77	1.72E-01	2.19E-01
**ENSAP2**	-1.22	1.72E-01	2.19E-01
**RP11-404P21.3**	-1.46	1.72E-01	2.19E-01
**ESD**	0.23	1.72E-01	2.19E-01

**C8orf48**	-0.41	1.72E-01	2.19E-01
**CARD17**	-0.54	1.72E-01	2.19E-01
**XLOC_014104**	-0.46	1.72E-01	2.19E-01
**RP11-565F19.4**	-0.96	1.72E-01	2.19E-01
**RP4-669P10.19**	-1.28	1.72E-01	2.19E-01
**RP5-857K21.4**	-1.40	1.73E-01	2.19E-01
**AGPAT4-IT1**	0.72	1.73E-01	2.20E-01
**AC005532.5**	-0.81	1.73E-01	2.20E-01
**HNRNPA1P22**	-1.89	1.73E-01	2.20E-01
**PHKA2**	-0.23	1.73E-01	2.20E-01
**TTPA**	1.32	1.73E-01	2.20E-01
**CTD-3035K23.6**	-1.55	1.73E-01	2.20E-01
**MSANTD1**	-0.59	1.73E-01	2.20E-01
**FKSG61**	-1.44	1.73E-01	2.20E-01
**ARL6IP4**	0.84	1.73E-01	2.20E-01
**RP11-80I15.1**	-0.79	1.73E-01	2.20E-01
**C2orf68**	0.14	1.73E-01	2.20E-01
**CNIH1**	-0.20	1.73E-01	2.20E-01
**ICT1**	0.23	1.73E-01	2.20E-01
**RP11-165E7.1**	-1.26	1.73E-01	2.20E-01
**AC016999.2**	-0.93	1.73E-01	2.20E-01
**CALCOCO2**	-0.14	1.73E-01	2.20E-01
**CNPY2**	-0.35	1.73E-01	2.20E-01
**RP11-491H19.1**	-1.94	1.73E-01	2.20E-01
**ROPN1B**	0.55	1.73E-01	2.20E-01
**UCN2**	0.46	1.73E-01	2.20E-01
**RP11-45M22.4**	-1.45	1.73E-01	2.20E-01
**SRP9**	-0.26	1.73E-01	2.20E-01
**GCOM1**	-0.46	1.74E-01	2.21E-01
**RP11-384K6.8**	-1.34	1.74E-01	2.21E-01
**XLOC_001242**	-1.22	1.74E-01	2.21E-01
**JRKL**	-0.20	1.74E-01	2.21E-01
**ARHGAP20**	0.52	1.74E-01	2.21E-01
**PCDH15**	-1.27	1.74E-01	2.21E-01
**CSNK2A1**	-0.17	1.74E-01	2.21E-01
**SLC16A12**	0.72	1.74E-01	2.21E-01
**RP11-307C12.12**	-1.23	1.74E-01	2.21E-01
**FAM104B**	0.22	1.74E-01	2.21E-01
**LINC01250**	-2.29	1.74E-01	2.21E-01
**MYLK2**	1.52	1.74E-01	2.21E-01
**LINC01402**	-1.41	1.74E-01	2.21E-01
**CTB-55O6.12**	0.42	1.74E-01	2.21E-01
**LINC01089**	-0.50	1.74E-01	2.21E-01
**CTD-2193G5.1**	-1.32	1.74E-01	2.21E-01
**AC093627.8**	1.63	1.74E-01	2.22E-01

**RP11-498E2.9**	-0.33	1.75E-01	2.22E-01
**RP1-286D6.5**	-0.51	1.75E-01	2.22E-01
**RAB5CP2**	-1.69	1.75E-01	2.22E-01
**GKAP1**	-0.32	1.75E-01	2.22E-01
**MECR**	0.29	1.75E-01	2.22E-01
**RP11-259P15.4**	-1.37	1.75E-01	2.22E-01
**LRRC38**	1.59	1.75E-01	2.22E-01
**SERPINC1**	-1.10	1.75E-01	2.22E-01
**SPIRE2**	0.53	1.75E-01	2.22E-01
**SMIM22**	0.89	1.75E-01	2.22E-01
**NUTM2E**	1.19	1.75E-01	2.22E-01
**COL5A1-AS1**	-1.75	1.75E-01	2.22E-01
**RP11-530A18.1**	-1.37	1.75E-01	2.22E-01
**CABP7**	-1.13	1.75E-01	2.22E-01
**MLIP-IT1**	-0.50	1.75E-01	2.22E-01
**ZNF674-AS1**	-0.29	1.75E-01	2.23E-01
**TNIK**	-0.36	1.75E-01	2.23E-01
**IRS2**	-0.24	1.76E-01	2.23E-01
**AC083899.3**	0.44	1.76E-01	2.23E-01
**KB-1562D12.1**	-0.38	1.76E-01	2.23E-01
**RP11-560J1.2**	0.44	1.76E-01	2.23E-01
**GBA3**	-1.72	1.76E-01	2.23E-01
**RP11-419C23.1**	1.75	1.76E-01	2.23E-01
**EIF2S2P4**	-0.48	1.76E-01	2.23E-01
**G8456**	0.78	1.76E-01	2.23E-01
**G41767**	-1.22	1.76E-01	2.23E-01
**RP11-20B7.1**	1.19	1.76E-01	2.24E-01
**LINC00476**	0.27	1.76E-01	2.24E-01
**CCDC89**	-0.41	1.76E-01	2.24E-01
**NDST2**	-0.48	1.76E-01	2.24E-01
**ETV6**	0.18	1.76E-01	2.24E-01
**DHCR7**	0.38	1.77E-01	2.24E-01
**XLOC_008935**	1.07	1.77E-01	2.24E-01
**KRT83**	-2.59	1.77E-01	2.24E-01
**ALG1L7P**	-1.06	1.77E-01	2.24E-01
**LPP-AS2**	0.26	1.77E-01	2.24E-01
**G32333**	0.98	1.77E-01	2.24E-01
**RNU6-310P**	-1.65	1.77E-01	2.24E-01
**RP11-169E6.1**	-0.74	1.77E-01	2.25E-01
**LARS2-AS1**	-0.93	1.77E-01	2.25E-01
**AC067742.1**	-1.82	1.77E-01	2.25E-01
**SLC6A16**	0.55	1.77E-01	2.25E-01
**RP11-216B9.6**	-0.44	1.77E-01	2.25E-01
**CTC-559E9.9**	1.08	1.77E-01	2.25E-01
**RP11-367E12.4**	-0.66	1.77E-01	2.25E-01

**RP11-850A17.1**	1.10	1.77E-01	2.25E-01
**RN7SL181P**	0.64	1.77E-01	2.25E-01
**MLXIPL**	0.86	1.77E-01	2.25E-01
**AC140479.1**	1.16	1.78E-01	2.25E-01
**RP11-674N23.4**	-1.64	1.78E-01	2.25E-01
**RP13-580F15.2**	0.87	1.78E-01	2.25E-01
**CFL2**	0.33	1.78E-01	2.25E-01
**TMEM132D**	-1.19	1.78E-01	2.25E-01
**SUCLG2-AS1**	-0.48	1.78E-01	2.25E-01
**BMPER**	0.79	1.78E-01	2.25E-01
**AL669831.1**	-1.64	1.78E-01	2.25E-01
**RSPH10B2**	-1.66	1.78E-01	2.25E-01
**RP13-714J12.1**	-1.18	1.78E-01	2.25E-01
**FAM98B**	0.21	1.78E-01	2.25E-01
**RP11-773H22.4**	-1.02	1.78E-01	2.26E-01
**RP11-396C23.2**	0.42	1.78E-01	2.26E-01
**SRSF9P1**	0.83	1.78E-01	2.26E-01
**ABCA9**	-0.60	1.78E-01	2.26E-01
**TMED4**	-0.13	1.78E-01	2.26E-01
**HDX**	-0.38	1.78E-01	2.26E-01
**BORCS5**	-0.23	1.78E-01	2.26E-01
**RP11-87H9.2**	0.33	1.78E-01	2.26E-01
**TASP1**	-0.12	1.78E-01	2.26E-01
**LDHAP4**	0.50	1.78E-01	2.26E-01
**FAM122C**	0.27	1.78E-01	2.26E-01
**TSTD3**	-0.39	1.79E-01	2.26E-01
**PKLR**	-1.00	1.79E-01	2.26E-01
**C16orf54**	-0.60	1.79E-01	2.26E-01
**LINC00607**	-0.74	1.79E-01	2.26E-01
**RP4-604G5.3**	1.16	1.79E-01	2.26E-01
**RP11-402G3.5**	-1.64	1.79E-01	2.27E-01
**RP11-671M22.4**	-1.78	1.79E-01	2.27E-01
**RNU6-653P**	-2.05	1.79E-01	2.27E-01
**TCEAL7**	0.56	1.79E-01	2.27E-01
**FNBP1L**	-0.19	1.79E-01	2.27E-01
**CRACR2A**	0.73	1.79E-01	2.27E-01
**TRGV5**	1.23	1.79E-01	2.27E-01
**OR2A13P**	-1.48	1.79E-01	2.27E-01
**TXNRD1**	0.22	1.79E-01	2.27E-01
**ATP9A**	0.21	1.79E-01	2.27E-01
**RP5-1024G6.5**	-0.56	1.79E-01	2.27E-01
**MIPEPP1**	-1.75	1.79E-01	2.27E-01
**NBPF11**	-0.45	1.80E-01	2.27E-01
**NDUFA10**	0.16	1.80E-01	2.28E-01
**AC005082.1**	-1.19	1.80E-01	2.28E-01

**RP11-667M19.10**	1.09	1.80E-01	2.28E-01
**TBC1D2B**	0.19	1.80E-01	2.28E-01
**PDCD11**	-0.17	1.80E-01	2.28E-01
**GABRA2**	-1.05	1.80E-01	2.28E-01
**RP4-737E23.6**	1.25	1.80E-01	2.28E-01
**FUT6**	1.04	1.80E-01	2.28E-01
**RP4-545C24.1**	-0.64	1.80E-01	2.28E-01
**SMIM24**	1.23	1.80E-01	2.28E-01
**SLC25A20**	0.35	1.80E-01	2.28E-01
**AC004019.10**	-1.98	1.80E-01	2.28E-01
**RP11-348P10.2**	-0.40	1.80E-01	2.28E-01
**LINC00921**	0.35	1.80E-01	2.28E-01
**MGAT2**	0.80	1.81E-01	2.28E-01
**LINC01597**	1.37	1.81E-01	2.29E-01
**RP11-354E23.4**	-0.82	1.81E-01	2.29E-01
**CRYBB3**	-0.68	1.81E-01	2.29E-01
**PNPLA1**	-0.32	1.81E-01	2.29E-01
**KIAA0430**	-0.13	1.81E-01	2.29E-01
**ARL2BP**	0.32	1.81E-01	2.29E-01
**BTNL10**	-1.05	1.81E-01	2.29E-01
**CSF2**	-1.16	1.81E-01	2.29E-01
**RP11-49I11.2**	-1.10	1.82E-01	2.30E-01
**RP1-67A8.3**	-1.46	1.82E-01	2.30E-01
**G25462**	-1.95	1.82E-01	2.30E-01
**RP11-196B3.2**	-0.92	1.82E-01	2.30E-01
**SLC26A8**	-0.82	1.82E-01	2.30E-01
**CTB-3M24.1**	-1.33	1.82E-01	2.30E-01
**RFPL1S**	0.93	1.82E-01	2.30E-01
**RASGRF1**	0.73	1.82E-01	2.30E-01
**RP11-65L3.4**	-0.94	1.82E-01	2.30E-01
**RP4-671O14.6**	-0.62	1.82E-01	2.30E-01
**RP5-1180D12.1**	0.95	1.82E-01	2.30E-01
**PDE11A**	0.88	1.82E-01	2.30E-01
**LTA**	1.33	1.82E-01	2.30E-01
**ARL4A**	0.29	1.82E-01	2.30E-01
**DND1P1**	-0.46	1.82E-01	2.30E-01
**ZHX3**	-0.22	1.82E-01	2.30E-01
**RNF207**	0.59	1.82E-01	2.30E-01
**C4BPB**	1.25	1.82E-01	2.30E-01
**AP001347.6**	0.58	1.82E-01	2.30E-01
**RNU4-51P**	-1.31	1.82E-01	2.31E-01
**PRB2**	1.05	1.82E-01	2.31E-01
**XLOC_005550**	-1.12	1.83E-01	2.31E-01
**BMS1P1**	-0.94	1.83E-01	2.31E-01
**CTD-2666L21.1**	-0.75	1.83E-01	2.31E-01

**CDC6**	-0.23	1.83E-01	2.31E-01
**AC005682.5**	0.51	1.83E-01	2.31E-01
**RP3-333A15.1**	-0.83	1.83E-01	2.31E-01
**FAM107A**	0.51	1.83E-01	2.31E-01
**RP11-326K13.5**	-1.25	1.83E-01	2.31E-01
**RP4-800M22.1**	0.49	1.83E-01	2.32E-01
**RP11-479O9.4**	0.38	1.83E-01	2.32E-01
**HCG2040054**	-1.25	1.84E-01	2.32E-01
**RP11-380J14.1**	1.23	1.84E-01	2.32E-01
**RP11-98D18.9**	0.93	1.84E-01	2.32E-01
**RP11-196G11.3**	1.28	1.84E-01	2.32E-01
**RP11-863K10.4**	-0.44	1.84E-01	2.32E-01
**PEBP1P3**	-1.76	1.84E-01	2.32E-01
**RP11-855O10.2**	1.52	1.84E-01	2.32E-01
**ACTN3**	-1.35	1.84E-01	2.32E-01
**TAF1A**	-0.28	1.84E-01	2.32E-01
**RP11-720N19.2**	-1.18	1.84E-01	2.32E-01
**CPNE8**	0.24	1.84E-01	2.32E-01
**G11629**	-0.81	1.84E-01	2.33E-01
**RP11-678G14.2**	-0.84	1.84E-01	2.33E-01
**AC064875.2**	1.48	1.84E-01	2.33E-01
**ARL14EP**	-0.17	1.85E-01	2.33E-01
**PLA2G4E**	0.26	1.85E-01	2.33E-01
**DPY19L2P3**	-0.45	1.85E-01	2.33E-01
**MARK2P17**	-1.85	1.85E-01	2.33E-01
**G26596**	1.24	1.85E-01	2.33E-01
**DCST1**	-0.64	1.85E-01	2.33E-01
**LINC00612**	0.54	1.85E-01	2.33E-01
**RNLS**	-0.23	1.85E-01	2.33E-01
**RP4-800J21.3**	-0.98	1.85E-01	2.33E-01
**AC016734.2**	-0.84	1.85E-01	2.33E-01
**G25045**	1.19	1.85E-01	2.33E-01
**TMEM211**	1.27	1.85E-01	2.34E-01
**TMEM183A**	0.15	1.85E-01	2.34E-01
**NRCAM**	0.40	1.85E-01	2.34E-01
**SPIN3**	0.42	1.85E-01	2.34E-01
**NKAIN4**	1.08	1.85E-01	2.34E-01
**LINC01484**	-2.05	1.85E-01	2.34E-01
**IGFL3**	-0.48	1.85E-01	2.34E-01
**RP11-845M18.6**	1.18	1.85E-01	2.34E-01
**RNU6-190P**	-1.21	1.85E-01	2.34E-01
**MAGI2-AS3**	0.43	1.85E-01	2.34E-01
**CTD-2410N18.3**	-0.36	1.86E-01	2.34E-01
**WDR97**	-0.67	1.86E-01	2.34E-01
**RP11-523H20.3**	0.98	1.86E-01	2.34E-01

**MIR4484**	-1.55	1.86E-01	2.34E-01
**TSPYL5**	0.30	1.86E-01	2.34E-01
**AC015849.19**	-0.54	1.86E-01	2.34E-01
**TCTN1**	-0.18	1.86E-01	2.34E-01
**RP11-21G15.1**	0.70	1.86E-01	2.34E-01
**FAHD2CP**	0.42	1.86E-01	2.34E-01
**AC004980.10**	-0.79	1.86E-01	2.34E-01
**HNRNPA1P4**	-1.70	1.86E-01	2.35E-01
**RP11-350D17.3**	1.70	1.86E-01	2.35E-01
**USP30-AS1**	-1.25	1.86E-01	2.35E-01
**G27759**	0.62	1.86E-01	2.35E-01
**PGRMC1**	-0.32	1.86E-01	2.35E-01
**FAM196B**	-0.59	1.86E-01	2.35E-01
**RP11-118E18.2**	-1.74	1.86E-01	2.35E-01
**CD8B**	0.54	1.86E-01	2.35E-01
**RNA5SP465**	-1.45	1.86E-01	2.35E-01
**RP11-485G7.5**	1.29	1.86E-01	2.35E-01
**WIPF3**	0.33	1.87E-01	2.35E-01
**COL6A4P2**	1.46	1.87E-01	2.35E-01
**SERHL2**	-0.58	1.87E-01	2.35E-01
**MUC20**	0.63	1.87E-01	2.35E-01
**TAF13**	-0.24	1.87E-01	2.35E-01
**RP11-437J2.3**	-1.82	1.87E-01	2.35E-01
**RP11-95M15.2**	-1.18	1.87E-01	2.35E-01
**ENPP5**	0.53	1.87E-01	2.36E-01
**SERTAD2**	-0.18	1.87E-01	2.36E-01
**DDX10P1**	-0.77	1.87E-01	2.36E-01
**LINC00937**	0.57	1.87E-01	2.36E-01
**HRSP12**	0.29	1.88E-01	2.36E-01
**RP1-151F17.2**	0.38	1.88E-01	2.36E-01
**RUNDC3A-AS1**	0.55	1.88E-01	2.36E-01
**RP11-196G11.6**	-0.51	1.88E-01	2.36E-01
**RP11-139K1.2**	-1.06	1.88E-01	2.36E-01
**TBC1D8B**	0.29	1.88E-01	2.37E-01
**ZC3H4**	0.17	1.88E-01	2.37E-01
**MIRLET7G**	-1.41	1.88E-01	2.37E-01
**AC000111.6**	-0.95	1.88E-01	2.37E-01
**AC130469.1**	1.27	1.88E-01	2.37E-01
**AC073657.1**	-0.79	1.88E-01	2.37E-01
**RP4-758J18.7**	0.54	1.88E-01	2.37E-01
**TMCC3**	-0.22	1.88E-01	2.37E-01
**MIR4653**	0.72	1.88E-01	2.37E-01
**RP11-409K20.7**	-0.45	1.88E-01	2.37E-01
**SLC37A3**	0.17	1.88E-01	2.37E-01
**KANSL2**	0.11	1.88E-01	2.37E-01

**RP11-383F6.1**	-0.88	1.88E-01	2.37E-01
**RN7SL388P**	-0.95	1.88E-01	2.37E-01
**RP11-192H23.8**	0.75	1.89E-01	2.37E-01
**KRT84**	1.87	1.89E-01	2.38E-01
**RP11-778D9.4**	-1.13	1.89E-01	2.38E-01
**AC091492.2**	-1.55	1.89E-01	2.38E-01
**PSCA**	-0.49	1.89E-01	2.38E-01
**RP11-457M11.5**	-0.63	1.89E-01	2.38E-01
**CTC-455F18.1**	1.38	1.89E-01	2.38E-01
**AGGF1P2**	-1.14	1.89E-01	2.38E-01
**CXorf65**	1.01	1.89E-01	2.38E-01
**CAPN10**	0.32	1.89E-01	2.38E-01
**CNGA1**	-0.34	1.89E-01	2.38E-01
**SH3BGRL2**	-0.32	1.89E-01	2.38E-01
**TRMT10B**	-0.20	1.89E-01	2.38E-01
**G39837**	-1.15	1.90E-01	2.38E-01
**CALM2**	-0.20	1.90E-01	2.39E-01
**GTF2B**	-0.21	1.90E-01	2.39E-01
**PPM1H**	0.41	1.90E-01	2.39E-01
**TESC-AS1**	-1.80	1.90E-01	2.39E-01
**KBTBD4**	-0.18	1.90E-01	2.39E-01
**AHSA2**	-0.53	1.90E-01	2.39E-01
**ANKRD20A9P**	2.96	1.90E-01	2.39E-01
**GPR137B**	-0.17	1.90E-01	2.39E-01
**ADARB2**	-0.88	1.90E-01	2.39E-01
**XLOC_010292**	-0.37	1.90E-01	2.39E-01
**GOLGA8B**	-0.64	1.90E-01	2.39E-01
**MTCO1P12**	1.36	1.90E-01	2.39E-01
**RP11-85I17.2**	-0.78	1.90E-01	2.39E-01
**KRTAP10-3**	-3.79	1.90E-01	2.39E-01
**RP11-358L22.3**	-0.34	1.91E-01	2.40E-01
**XLOC_014122**	-1.05	1.91E-01	2.40E-01
**NRSN2-AS1**	0.36	1.91E-01	2.40E-01
**G18793**	0.73	1.91E-01	2.40E-01
**KRTAP4-2**	-3.60	1.91E-01	2.40E-01
**ZNF778**	-0.17	1.91E-01	2.40E-01
**RNU6-892P**	-1.23	1.91E-01	2.40E-01
**CCDC87**	-0.63	1.91E-01	2.40E-01
**RP11-33O4.1**	-0.43	1.91E-01	2.40E-01
**RNA5SP227**	-1.61	1.91E-01	2.40E-01
**AC024896.1**	1.18	1.91E-01	2.40E-01
**RP11-627K11.1**	-1.18	1.91E-01	2.40E-01
**BTNL2**	-1.35	1.91E-01	2.40E-01
**SLC16A6**	-0.28	1.91E-01	2.40E-01
**RP11-443P15.2**	-0.48	1.91E-01	2.40E-01

**GOSR2**	-0.16	1.91E-01	2.40E-01
**NELL1**	0.89	1.91E-01	2.40E-01
**MPRIPP1**	1.06	1.91E-01	2.40E-01
**LCE1B**	0.49	1.92E-01	2.41E-01
**ZC2HC1C**	0.40	1.92E-01	2.41E-01
**LAMP2**	-0.18	1.92E-01	2.41E-01
**AC003986.6**	-0.82	1.92E-01	2.41E-01
**RP13-188A5.1**	0.26	1.92E-01	2.41E-01
**LINC00462**	-0.70	1.92E-01	2.41E-01
**RP11-158I9.5**	-1.83	1.92E-01	2.41E-01
**EIF1B-AS1**	-0.41	1.92E-01	2.41E-01
**RP1-118J21.25**	-1.79	1.92E-01	2.41E-01
**SCGB1B2P**	1.81	1.92E-01	2.41E-01
**SVOPL**	-0.78	1.92E-01	2.41E-01
**ATRNL1**	-0.61	1.92E-01	2.41E-01
**OR1Q1**	-1.86	1.92E-01	2.41E-01
**SRGAP1**	-0.38	1.92E-01	2.42E-01
**LETM2**	-0.38	1.92E-01	2.42E-01
**AC104695.4**	-1.48	1.92E-01	2.42E-01
**RP11-463J10.2**	-1.07	1.92E-01	2.42E-01
**NPIPB4**	0.52	1.92E-01	2.42E-01
**RP1-102G20.4**	-1.47	1.92E-01	2.42E-01
**R3HDM2**	0.22	1.92E-01	2.42E-01
**SLC35B4**	0.21	1.92E-01	2.42E-01
**DAB1**	0.87	1.93E-01	2.42E-01
**RP11-66N11.8**	0.43	1.93E-01	2.42E-01
**RP11-438B23.2**	-1.21	1.93E-01	2.42E-01
**CYB5A**	-0.34	1.93E-01	2.42E-01
**ETFDH**	-0.19	1.93E-01	2.42E-01
**AC012594.1**	-1.44	1.93E-01	2.42E-01
**G480**	0.61	1.93E-01	2.42E-01
**DIRC3**	-0.62	1.93E-01	2.42E-01
**SMC4**	-0.19	1.93E-01	2.42E-01
**RP11-803B1.2**	-1.48	1.93E-01	2.42E-01
**GDPGP1**	0.43	1.93E-01	2.43E-01
**RAB10**	0.20	1.93E-01	2.43E-01
**XLOC_000641**	1.14	1.93E-01	2.43E-01
**GEMIN2**	-0.13	1.94E-01	2.43E-01
**ERP27**	0.74	1.94E-01	2.43E-01
**SHANK1**	0.49	1.94E-01	2.43E-01
**CTD-2033C11.1**	-0.75	1.94E-01	2.43E-01
**CHST8**	0.88	1.94E-01	2.43E-01
**RP11-574K11.5**	0.75	1.94E-01	2.43E-01
**OR7E62P**	0.42	1.94E-01	2.44E-01
**ITGB1P1**	0.51	1.94E-01	2.44E-01

**U52111.14**	0.88	1.94E-01	2.44E-01
**PTGES**	0.28	1.94E-01	2.44E-01
**KRTAP2-1**	-3.89	1.95E-01	2.44E-01
**INTS4P2**	-0.80	1.95E-01	2.44E-01
**RP11-641D5.2**	1.45	1.95E-01	2.44E-01
**RP11-32B5.7**	-0.89	1.95E-01	2.44E-01
**MIR320D1**	1.31	1.95E-01	2.44E-01
**EIF5AP3**	-1.25	1.95E-01	2.44E-01
**LRRC31**	-1.67	1.95E-01	2.44E-01
**BDNF**	-0.39	1.95E-01	2.45E-01
**RP11-713D19.1**	-1.09	1.95E-01	2.45E-01
**SPRED1**	0.21	1.95E-01	2.45E-01
**SLC6A9**	-0.36	1.95E-01	2.45E-01
**HCG14**	-1.06	1.95E-01	2.45E-01
**ARID5B**	0.18	1.95E-01	2.45E-01
**UQCC1**	-0.13	1.95E-01	2.45E-01
**AGXT**	-0.98	1.95E-01	2.45E-01
**AC005086.3**	-1.24	1.96E-01	2.45E-01
**PERP**	-0.26	1.96E-01	2.45E-01
**SMPD4P1**	-1.77	1.96E-01	2.45E-01
**CALML3-AS1**	0.34	1.96E-01	2.45E-01
**SIAH3**	-1.10	1.96E-01	2.45E-01
**RP4-612B15.3**	0.37	1.96E-01	2.45E-01
**XXyac-YR29IB3.1**	-2.04	1.96E-01	2.45E-01
**ATP10B**	-0.21	1.96E-01	2.45E-01
**PROSC**	0.19	1.96E-01	2.45E-01
**MIR646HG**	-0.52	1.96E-01	2.45E-01
**P2RX6P**	-0.93	1.96E-01	2.46E-01
**RPS11P6**	-0.63	1.96E-01	2.46E-01
**LINC01395**	-0.67	1.96E-01	2.46E-01
**HILPDA**	0.46	1.96E-01	2.46E-01
**C7orf55**	0.47	1.96E-01	2.46E-01
**DNAAF2**	0.19	1.96E-01	2.46E-01
**TOX**	0.58	1.96E-01	2.46E-01
**RXRA**	-0.15	1.96E-01	2.46E-01
**RP3-339A18.6**	0.91	1.96E-01	2.46E-01
**XLOC_009493**	-1.00	1.97E-01	2.46E-01
**AC006126.3**	-0.96	1.97E-01	2.47E-01
**C2orf70**	1.31	1.97E-01	2.47E-01
**UQCRFS1**	0.19	1.97E-01	2.47E-01
**UBL7-AS1**	0.20	1.97E-01	2.47E-01
**TMEM110-MUSTN1**	-0.93	1.97E-01	2.47E-01
**UGT1A10**	-1.31	1.97E-01	2.47E-01
**RP11-112J1.1**	-1.02	1.97E-01	2.47E-01
**CLDND2**	0.77	1.97E-01	2.47E-01

**RP11-478K15.6**	-1.87	1.97E-01	2.47E-01
**G40303**	-0.84	1.97E-01	2.47E-01
**ENOX2**	0.14	1.97E-01	2.47E-01
**PDZRN4**	-0.60	1.98E-01	2.47E-01
**BSN**	-0.46	1.98E-01	2.48E-01
**MAP6D1**	0.26	1.98E-01	2.48E-01
**RP11-311F12.1**	0.53	1.98E-01	2.48E-01
**BPIFB4**	6.19	1.98E-01	2.48E-01
**APCDD1**	0.18	1.98E-01	2.48E-01
**ASNSP1**	-2.73	1.98E-01	2.48E-01
**RP11-582E3.6**	-0.21	1.98E-01	2.48E-01
**UCP1**	-1.41	1.98E-01	2.48E-01
**NSFP1**	-1.87	1.98E-01	2.48E-01
**RP11-532N4.2**	-2.51	1.98E-01	2.48E-01
**SUMO1P3**	-0.95	1.98E-01	2.48E-01
**RP4-584D14.5**	-0.41	1.98E-01	2.48E-01
**RP11-398K22.12**	-0.26	1.99E-01	2.49E-01
**RP3-340B19.5**	-1.25	1.99E-01	2.49E-01
**PGM5P3-AS1**	-1.17	1.99E-01	2.49E-01
**ZKSCAN2**	0.22	1.99E-01	2.49E-01
**PGR**	-0.64	1.99E-01	2.49E-01
**RP11-455F5.5**	-1.46	1.99E-01	2.49E-01
**THAP9-AS1**	-0.26	1.99E-01	2.49E-01
**C19orf57**	0.30	1.99E-01	2.49E-01
**RP4-630A11.3**	-1.83	1.99E-01	2.49E-01
**MRPS35**	-0.20	1.99E-01	2.49E-01
**TFIP11**	0.15	1.99E-01	2.49E-01
**AC009961.5**	1.04	2.00E-01	2.50E-01
**FMR1-AS1**	-1.16	2.00E-01	2.50E-01
**RPL7P1**	-0.39	2.00E-01	2.50E-01
**RP11-245C17.2**	-1.14	2.00E-01	2.50E-01
**XLOC_013591**	0.80	2.00E-01	2.50E-01
**RP11-528A10.2**	-1.31	2.00E-01	2.50E-01
**C11orf52**	-1.39	2.00E-01	2.50E-01
**RP11-269G24.4**	-0.85	2.00E-01	2.50E-01
**SCRN1**	0.30	2.00E-01	2.50E-01
**CNTN4**	-0.50	2.00E-01	2.50E-01
**AC016629.3**	-1.32	2.00E-01	2.50E-01
**MS4A1**	-1.00	2.00E-01	2.50E-01
**TMPRSS15**	-1.32	2.00E-01	2.50E-01
**C1orf106**	-0.23	2.00E-01	2.50E-01
**ANOS2P**	-0.68	2.00E-01	2.51E-01
**RP11-556H2.3**	-1.34	2.00E-01	2.51E-01
**OCSTAMP**	-1.39	2.00E-01	2.51E-01
**SEC22B**	-0.21	2.00E-01	2.51E-01

**TPRKB**	0.24	2.01E-01	2.51E-01
**NBR1**	-0.14	2.01E-01	2.51E-01
**ZNF491**	-0.44	2.01E-01	2.51E-01
**AC023115.2**	-1.08	2.01E-01	2.51E-01
**G15537**	0.80	2.01E-01	2.51E-01
**CTD-2034I4.2**	-2.09	2.01E-01	2.51E-01
**RP11-641D5.1**	-0.37	2.01E-01	2.51E-01
**CTD-2553C6.1**	-1.46	2.01E-01	2.51E-01
**RP11-958N24.1**	0.36	2.01E-01	2.51E-01
**RP11-222K16.1**	0.91	2.01E-01	2.51E-01
**PMS2CL**	0.22	2.01E-01	2.51E-01
**TICRR**	-0.31	2.01E-01	2.51E-01
**RPL36**	0.19	2.01E-01	2.51E-01
**G41741**	1.24	2.01E-01	2.51E-01
**RANBP3L**	0.77	2.01E-01	2.51E-01
**NOV**	0.37	2.01E-01	2.52E-01
**DARS**	0.21	2.01E-01	2.52E-01
**RP4-717I23.3**	-0.44	2.02E-01	2.52E-01
**SPDYE17**	-1.73	2.02E-01	2.52E-01
**RP11-372B4.3**	-0.57	2.02E-01	2.52E-01
**AC017028.8**	-1.76	2.02E-01	2.52E-01
**RP11-652L8.4**	1.36	2.02E-01	2.52E-01
**PER3**	-0.47	2.02E-01	2.52E-01
**TGFBRAP1**	-0.13	2.02E-01	2.52E-01
**LINC00887**	0.90	2.02E-01	2.52E-01
**ARHGEF18**	0.21	2.02E-01	2.52E-01
**AC104667.3**	1.35	2.02E-01	2.52E-01
**MT-TP**	0.44	2.02E-01	2.53E-01
**BCOR**	-0.15	2.02E-01	2.53E-01
**CEACAM3**	1.14	2.03E-01	2.53E-01
**RAB40C**	0.25	2.03E-01	2.53E-01
**DPPA2P4**	0.97	2.03E-01	2.53E-01
**SMG7-AS1**	-0.54	2.03E-01	2.53E-01
**NUTM2HP**	0.93	2.03E-01	2.53E-01
**HS6ST2**	-0.63	2.03E-01	2.53E-01
**MCF2L-AS1**	0.74	2.03E-01	2.53E-01
**EMP1**	-0.17	2.03E-01	2.53E-01
**FAM21A**	0.41	2.03E-01	2.53E-01
**KLF1**	1.42	2.03E-01	2.53E-01
**TMEM61**	0.72	2.03E-01	2.53E-01
**RBPJP5**	-1.48	2.03E-01	2.53E-01
**RP11-428K3.1**	-0.52	2.03E-01	2.53E-01
**CUL9**	-0.18	2.03E-01	2.54E-01
**AC008985.1**	-1.00	2.03E-01	2.54E-01
**RP11-707G18.1**	-1.02	2.03E-01	2.54E-01

**CCND2-AS1**	1.31	2.03E-01	2.54E-01
**RP11-775C24.3**	-1.13	2.03E-01	2.54E-01
**ZDHHC20-IT1**	-1.19	2.03E-01	2.54E-01
**SLC4A7**	-0.26	2.03E-01	2.54E-01
**SMURF1**	-0.13	2.03E-01	2.54E-01
**CYP4F3**	-0.38	2.03E-01	2.54E-01
**SPTSSB**	-0.36	2.04E-01	2.54E-01
**RNU2-51P**	-1.66	2.04E-01	2.54E-01
**G12085**	1.31	2.04E-01	2.54E-01
**C6orf89**	-0.10	2.04E-01	2.54E-01
**PNLIPRP3**	0.61	2.04E-01	2.54E-01
**CADM3-AS1**	-0.60	2.04E-01	2.54E-01
**ACAP3**	0.27	2.04E-01	2.54E-01
**AC009133.12**	0.22	2.04E-01	2.54E-01
**PXMP4**	-0.35	2.04E-01	2.54E-01
**RP1-39G22.7**	-0.29	2.04E-01	2.54E-01
**TTC28-AS1**	0.21	2.04E-01	2.54E-01
**RGPD1**	-0.89	2.04E-01	2.55E-01
**UHRF2**	-0.18	2.04E-01	2.55E-01
**RAB26**	-0.51	2.04E-01	2.55E-01
**RP11-554A11.7**	-1.50	2.04E-01	2.55E-01
**TANK**	-0.16	2.04E-01	2.55E-01
**RP3-512B11.3**	0.28	2.05E-01	2.55E-01
**C7**	0.75	2.05E-01	2.55E-01
**APOD**	0.54	2.05E-01	2.55E-01
**RP11-490G8.1**	-1.20	2.05E-01	2.55E-01
**RP11-452K12.4**	-0.85	2.05E-01	2.55E-01
**SCGB2B2**	-0.58	2.05E-01	2.55E-01
**ST3GAL4-AS1**	0.44	2.05E-01	2.55E-01
**RRP7BP**	-0.42	2.05E-01	2.55E-01
**TRIM4**	0.13	2.05E-01	2.56E-01
**RNU7-41P**	1.05	2.05E-01	2.56E-01
**COL22A1**	1.29	2.05E-01	2.56E-01
**JPH1**	0.43	2.05E-01	2.56E-01
**LINC00393**	-0.51	2.06E-01	2.56E-01
**G28010**	-0.73	2.06E-01	2.56E-01
**PGM5**	0.34	2.06E-01	2.56E-01
**RP11-367F23.2**	0.66	2.06E-01	2.56E-01
**TCTE1**	0.83	2.06E-01	2.56E-01
**RP3-395C13.1**	-1.17	2.06E-01	2.56E-01
**RP11-367F23.1**	1.15	2.06E-01	2.56E-01
**ZFY-AS1**	-1.09	2.06E-01	2.56E-01
**MIR4999**	-1.59	2.06E-01	2.56E-01
**LRMP**	-0.58	2.06E-01	2.57E-01
**RAB18**	-0.20	2.06E-01	2.57E-01

**FAM157B**	-1.25	2.06E-01	2.57E-01
**KRTAP4-8**	-3.26	2.06E-01	2.57E-01
**DISC1FP1**	-1.24	2.06E-01	2.57E-01
**MBL1P**	0.63	2.06E-01	2.57E-01
**ZNF365**	-0.61	2.07E-01	2.57E-01
**AC005592.2**	1.23	2.07E-01	2.57E-01
**HCN1**	0.71	2.07E-01	2.57E-01
**RP11-486G15.2**	0.78	2.07E-01	2.57E-01
**RP1-202O8.2**	-0.78	2.07E-01	2.57E-01
**MKRN1**	0.13	2.07E-01	2.57E-01
**SGOL2**	-0.30	2.07E-01	2.58E-01
**PDCD1**	0.85	2.07E-01	2.58E-01
**RP11-242D8.1**	-0.36	2.07E-01	2.58E-01
**FZD10-AS1**	0.33	2.07E-01	2.58E-01
**POMC**	0.37	2.07E-01	2.58E-01
**YES1P1**	-0.65	2.07E-01	2.58E-01
**SLC6A6**	0.26	2.07E-01	2.58E-01
**RP11-713N11.6**	-1.05	2.07E-01	2.58E-01
**CCDC136**	0.40	2.08E-01	2.58E-01
**CACNA1F**	-0.64	2.08E-01	2.58E-01
**FBLN5**	0.52	2.08E-01	2.58E-01
**HLX-AS1**	1.33	2.08E-01	2.58E-01
**CPZ**	1.02	2.08E-01	2.59E-01
**RP11-92K15.3**	-0.61	2.08E-01	2.59E-01
**ECM2**	-0.44	2.08E-01	2.59E-01
**G3639**	1.47	2.08E-01	2.59E-01
**DHX57**	-0.11	2.08E-01	2.59E-01
**RP13-942N8.1**	-0.42	2.08E-01	2.59E-01
**PLCB4**	-0.47	2.08E-01	2.59E-01
**MIR3178**	-1.23	2.08E-01	2.59E-01
**RPL31P11**	-1.72	2.08E-01	2.59E-01
**LCMT1-AS1**	0.61	2.08E-01	2.59E-01
**RP11-365N19.2**	-0.95	2.08E-01	2.59E-01
**RP11-402P6.9**	-1.19	2.08E-01	2.59E-01
**HIGD1C**	-1.62	2.08E-01	2.59E-01
**MUC7**	1.99	2.08E-01	2.59E-01
**CCDC163P**	0.65	2.08E-01	2.59E-01
**AC021188.4**	0.65	2.09E-01	2.59E-01
**PPIAP30**	-0.95	2.09E-01	2.59E-01
**RP11-1148L6.9**	-0.54	2.09E-01	2.60E-01
**RP1-179N16.6**	-0.96	2.09E-01	2.60E-01
**SARAF**	-0.19	2.09E-01	2.60E-01
**CTD-2575K13.6**	-1.05	2.09E-01	2.60E-01
**MIR5690**	-0.84	2.09E-01	2.60E-01
**AP003068.23**	0.39	2.09E-01	2.60E-01

**TIAM2**	-0.28	2.09E-01	2.60E-01
**SPINT4**	-1.79	2.09E-01	2.60E-01
**ZNF526**	0.17	2.09E-01	2.60E-01
**RP11-505P4.6**	-0.89	2.09E-01	2.60E-01
**HLA-F-AS1**	-0.39	2.10E-01	2.61E-01
**RP3-331H24.6**	-0.71	2.10E-01	2.61E-01
**RPE65**	1.36	2.10E-01	2.61E-01
**TSC22D2**	0.16	2.10E-01	2.61E-01
**TGM5**	0.33	2.10E-01	2.61E-01
**PXN-AS1**	-0.30	2.10E-01	2.61E-01
**TSNARE1**	0.22	2.10E-01	2.61E-01
**RP11-461A8.4**	-0.79	2.10E-01	2.61E-01
**TPTE2**	-1.32	2.10E-01	2.61E-01
**EIF1AXP1**	-0.44	2.10E-01	2.61E-01
**RP11-178F10.1**	1.36	2.10E-01	2.61E-01
**ASIC3**	0.39	2.10E-01	2.61E-01
**RP5-882O7.1**	1.01	2.10E-01	2.61E-01
**AC129492.6**	-0.62	2.10E-01	2.61E-01
**MRPL39**	-0.22	2.10E-01	2.61E-01
**AC005255.3**	-0.72	2.10E-01	2.61E-01
**RP11-455F5.3**	0.41	2.10E-01	2.61E-01
**ZNF709**	-0.66	2.10E-01	2.61E-01
**PFN1P8**	1.04	2.11E-01	2.62E-01
**GPS2**	0.50	2.11E-01	2.62E-01
**G12754**	-0.86	2.11E-01	2.62E-01
**MYO16**	-0.51	2.11E-01	2.62E-01
**MYRIP**	0.46	2.11E-01	2.62E-01
**RP11-752L20.3**	-0.43	2.11E-01	2.62E-01
**ANKRD18DP**	-1.20	2.11E-01	2.62E-01
**RP11-46H11.12**	0.77	2.11E-01	2.62E-01
**G34428**	0.34	2.11E-01	2.62E-01
**TMC1**	0.74	2.11E-01	2.62E-01
**CTD-2366F13.1**	0.40	2.11E-01	2.62E-01
**RP11-355B11.2**	-0.40	2.11E-01	2.62E-01
**CTD-2270N23.1**	-0.80	2.11E-01	2.62E-01
**RAF1**	-0.11	2.11E-01	2.62E-01
**AC007285.6**	-0.51	2.11E-01	2.62E-01
**FAM129A**	-0.22	2.11E-01	2.62E-01
**GCLC**	-0.21	2.11E-01	2.62E-01
**RP11-122C5.2**	-1.83	2.11E-01	2.62E-01
**CTB-174D11.2**	-1.28	2.12E-01	2.63E-01
**RP11-700H6.4**	-0.77	2.12E-01	2.63E-01
**ANKRD62**	-1.64	2.12E-01	2.63E-01
**XLOC_006931**	-0.90	2.12E-01	2.63E-01
**KB-1460A1.3**	-1.37	2.12E-01	2.63E-01

**DHFR**	0.34	2.12E-01	2.63E-01
**RPL37P23**	-1.35	2.12E-01	2.63E-01
**PTGES3L**	0.73	2.12E-01	2.63E-01
**TOMM20**	-0.17	2.12E-01	2.63E-01
**RP11-474N24.6**	1.16	2.12E-01	2.64E-01
**MIR4258**	1.21	2.13E-01	2.64E-01
**RP11-392O1.4**	-1.17	2.13E-01	2.64E-01
**USP42**	0.12	2.13E-01	2.64E-01
**LINC01611**	-1.47	2.13E-01	2.64E-01
**XLOC_005361**	-0.61	2.13E-01	2.64E-01
**RP11-44F14.8**	0.61	2.13E-01	2.65E-01
**PPFIA2**	-0.58	2.14E-01	2.65E-01
**AP000251.3**	-1.07	2.14E-01	2.65E-01
**RPSAP41**	-1.59	2.14E-01	2.65E-01
**CTD-2228K2.1**	-1.45	2.14E-01	2.65E-01
**SIGLEC16**	0.60	2.14E-01	2.65E-01
**LINC00960**	1.55	2.14E-01	2.65E-01
**AC137934.1**	0.72	2.14E-01	2.65E-01
**TRIM32**	0.15	2.14E-01	2.65E-01
**NRAV**	-0.19	2.14E-01	2.65E-01
**GSG1L**	-1.07	2.14E-01	2.65E-01
**RP11-286B14.1**	0.65	2.14E-01	2.65E-01
**AP000688.29**	-0.74	2.14E-01	2.66E-01
**TTC6**	-1.62	2.14E-01	2.66E-01
**ZRANB2-AS2**	-0.46	2.14E-01	2.66E-01
**CTB-47B11.3**	-1.25	2.14E-01	2.66E-01
**G30962**	1.50	2.14E-01	2.66E-01
**RP11-261C10.5**	-1.34	2.14E-01	2.66E-01
**RP11-436D23.1**	-1.47	2.14E-01	2.66E-01
**LINC00491**	1.31	2.14E-01	2.66E-01
**ADRB2**	0.41	2.15E-01	2.66E-01
**STK24**	-0.15	2.15E-01	2.66E-01
**CEBPB-AS1**	-0.50	2.15E-01	2.66E-01
**EIF2S2P2**	-0.93	2.15E-01	2.66E-01
**IER3**	0.32	2.15E-01	2.66E-01
**PKD1P5**	0.91	2.15E-01	2.66E-01
**VWA5B2**	-0.76	2.15E-01	2.66E-01
**G22216**	0.94	2.15E-01	2.66E-01
**TF**	-0.85	2.15E-01	2.66E-01
**ZMYND10**	-0.43	2.15E-01	2.67E-01
**EGLN1**	0.17	2.15E-01	2.67E-01
**HAL**	0.41	2.15E-01	2.67E-01
**G39379**	-0.57	2.16E-01	2.67E-01
**LINC01219**	-0.78	2.16E-01	2.67E-01
**RHNO1**	-0.17	2.16E-01	2.67E-01

**PROKR1**	-1.56	2.16E-01	2.67E-01
**RNASE4**	0.62	2.16E-01	2.67E-01
**SIGLEC22P**	-1.23	2.16E-01	2.67E-01
**RP11-169K16.8**	-0.84	2.16E-01	2.67E-01
**MAL2**	-0.24	2.16E-01	2.67E-01
**ASH1L-AS1**	0.31	2.16E-01	2.68E-01
**OPN1LW**	1.25	2.16E-01	2.68E-01
**CFL1P5**	-1.25	2.16E-01	2.68E-01
**RN7SL846P**	-1.76	2.16E-01	2.68E-01
**ANKK1**	-0.38	2.16E-01	2.68E-01
**CTD-2192J16.26**	-0.84	2.16E-01	2.68E-01
**XLOC_012002**	-1.01	2.16E-01	2.68E-01
**ECHDC1**	0.21	2.16E-01	2.68E-01
**CTD-2195B23.3**	-0.84	2.17E-01	2.68E-01
**FAM45B**	0.81	2.17E-01	2.68E-01
**C8orf34**	0.69	2.17E-01	2.68E-01
**DDHD2**	-0.12	2.17E-01	2.68E-01
**S100A13**	0.34	2.17E-01	2.68E-01
**RP3-323A16.1**	-1.55	2.17E-01	2.68E-01
**TGFB2-AS1**	-0.52	2.17E-01	2.68E-01
**IGLV3-19**	-1.78	2.17E-01	2.69E-01
**PRICKLE2**	0.28	2.17E-01	2.69E-01
**RUNDC3B**	-0.41	2.17E-01	2.69E-01
**CCDC24**	-0.48	2.17E-01	2.69E-01
**CYP2C8**	-0.94	2.17E-01	2.69E-01
**C9orf153**	-1.07	2.17E-01	2.69E-01
**DENND5B**	-0.28	2.17E-01	2.69E-01
**RP11-307C18.1**	-0.71	2.17E-01	2.69E-01
**RP11-78A19.4**	-1.11	2.17E-01	2.69E-01
**AC099048.1**	1.02	2.17E-01	2.69E-01
**RP11-706O15.3**	-1.97	2.17E-01	2.69E-01
**RP11-977G19.12**	-0.56	2.17E-01	2.69E-01
**RP11-263K19.4**	-0.74	2.18E-01	2.69E-01
**GTF2IP5**	-0.65	2.18E-01	2.69E-01
**SLC39A6**	-0.29	2.18E-01	2.69E-01
**ABCD2**	-0.70	2.18E-01	2.69E-01
**RP11-159N11.4**	-0.45	2.18E-01	2.69E-01
**CCDC154**	0.78	2.18E-01	2.69E-01
**XLOC_001048**	-1.26	2.18E-01	2.69E-01
**CTD-2260A17.2**	0.30	2.18E-01	2.69E-01
**RP11-793H13.11**	0.31	2.18E-01	2.69E-01
**RP11-367N14.2**	0.76	2.18E-01	2.70E-01
**SKOR1**	0.45	2.18E-01	2.70E-01
**RP11-301L8.2**	-1.09	2.18E-01	2.70E-01
**TNK2-AS1**	-0.47	2.18E-01	2.70E-01

**RP11-248J18.2**	0.39	2.18E-01	2.70E-01
**RP11-697N18.2**	-1.56	2.18E-01	2.70E-01
**RP11-1078H9.5**	-0.94	2.18E-01	2.70E-01
**KIAA1683**	-0.46	2.18E-01	2.70E-01
**KCNAB3**	0.49	2.18E-01	2.70E-01
**KCNJ3**	-1.02	2.19E-01	2.70E-01
**RP11-10C8.2**	-0.94	2.19E-01	2.70E-01
**XLOC_002654**	0.95	2.19E-01	2.70E-01
**TAS2R62P**	-1.39	2.19E-01	2.70E-01
**G26828**	0.61	2.19E-01	2.70E-01
**C16orf70**	0.15	2.19E-01	2.71E-01
**COX11**	-0.15	2.19E-01	2.71E-01
**CX3CR1**	-0.36	2.19E-01	2.71E-01
**NYAP2**	-1.55	2.19E-01	2.71E-01
**SAMD9L**	-0.38	2.19E-01	2.71E-01
**RNVU1-19**	-2.97	2.19E-01	2.71E-01
**BRINP2**	-0.99	2.19E-01	2.71E-01
**ZSCAN1**	0.88	2.19E-01	2.71E-01
**SH2D1B**	0.95	2.19E-01	2.71E-01
**DAPL1**	0.31	2.20E-01	2.71E-01
**AC083862.6**	-1.48	2.20E-01	2.72E-01
**RP11-792A8.4**	-0.82	2.20E-01	2.72E-01
**RP11-177H2.1**	0.56	2.20E-01	2.72E-01
**GEMIN6**	0.20	2.20E-01	2.72E-01
**CNNM1**	-0.31	2.20E-01	2.72E-01
**XLOC_001447**	-0.79	2.20E-01	2.72E-01
**CTB-31O20.2**	-0.45	2.20E-01	2.72E-01
**SSPN**	0.38	2.20E-01	2.72E-01
**RP11-113K21.4**	-1.15	2.20E-01	2.72E-01
**ATXN1L**	-0.13	2.20E-01	2.72E-01
**ZNF454**	0.49	2.20E-01	2.72E-01
**XLOC_007879**	-0.66	2.20E-01	2.72E-01
**SH2D7**	-1.01	2.20E-01	2.72E-01
**EPCAM**	0.45	2.21E-01	2.72E-01
**RP11-290O12.2**	0.80	2.21E-01	2.72E-01
**XLOC_008667**	-0.51	2.21E-01	2.73E-01
**EIF1P6**	-0.70	2.21E-01	2.73E-01
**RN7SL268P**	-1.36	2.21E-01	2.73E-01
**RP11-867G2.4**	-1.58	2.21E-01	2.73E-01
**AC007966.1**	-0.96	2.21E-01	2.73E-01
**CTD-2600O9.1**	-0.49	2.21E-01	2.73E-01
**MTERF3**	-0.17	2.21E-01	2.73E-01
**RP11-689K5.3**	-0.74	2.21E-01	2.73E-01
**CTC-429P9.5**	0.21	2.21E-01	2.73E-01
**PACSIN1**	1.17	2.21E-01	2.73E-01

**MIR4477B**	-0.56	2.21E-01	2.73E-01
**MLYCD**	-0.17	2.21E-01	2.73E-01
**AC073254.1**	-0.34	2.21E-01	2.73E-01
**CTA-212A2.1**	0.72	2.22E-01	2.74E-01
**ADGRL2**	0.27	2.22E-01	2.74E-01
**SNCB**	1.39	2.22E-01	2.74E-01
**CTD-3247H4.2**	-0.79	2.22E-01	2.74E-01
**RP11-231P20.2**	1.01	2.22E-01	2.74E-01
**TRGV10**	-1.52	2.22E-01	2.74E-01
**KC6**	-1.41	2.22E-01	2.74E-01
**RP4-687K1.2**	-1.27	2.22E-01	2.74E-01
**AC005104.3**	-0.62	2.22E-01	2.74E-01
**AC104532.3**	-1.35	2.22E-01	2.74E-01
**G29700**	-1.07	2.23E-01	2.75E-01
**CTD-2561B21.10**	-1.39	2.23E-01	2.75E-01
**CTD-3092A11.2**	0.40	2.23E-01	2.75E-01
**KLHL31**	-0.39	2.23E-01	2.75E-01
**AC016700.5**	0.71	2.23E-01	2.75E-01
**KB-1410C5.5**	0.33	2.23E-01	2.75E-01
**PRDM11**	0.15	2.23E-01	2.75E-01
**UBE2C**	0.27	2.23E-01	2.75E-01
**ITGB1BP2**	-0.61	2.23E-01	2.75E-01
**GNB1L**	0.31	2.23E-01	2.75E-01
**RP11-218E20.5**	-1.31	2.23E-01	2.75E-01
**RP11-65J3.14**	-1.10	2.23E-01	2.75E-01
**FHL1**	-0.23	2.23E-01	2.75E-01
**POLR3F**	-0.14	2.23E-01	2.75E-01
**G41661**	-0.47	2.23E-01	2.76E-01
**ATP2C2**	0.22	2.23E-01	2.76E-01
**CTD-2024P10.2**	-0.90	2.24E-01	2.76E-01
**RP11-10L7.1**	1.08	2.24E-01	2.76E-01
**XKR5**	-0.65	2.24E-01	2.76E-01
**GSDMC**	0.31	2.24E-01	2.76E-01
**CTD-2510F5.4**	-0.39	2.24E-01	2.76E-01
**PHF10**	-0.13	2.24E-01	2.76E-01
**RP11-693J15.5**	-1.33	2.24E-01	2.76E-01
**FAM212B-AS1**	-1.11	2.24E-01	2.76E-01
**DCAF4L1**	-0.65	2.24E-01	2.76E-01
**APOBEC3A**	0.96	2.24E-01	2.77E-01
**C16orf74**	0.42	2.24E-01	2.77E-01
**PLEKHB2**	-0.13	2.24E-01	2.77E-01
**RP11-477I4.4**	0.62	2.24E-01	2.77E-01
**G3945**	-0.74	2.24E-01	2.77E-01
**KCTD12**	-0.29	2.25E-01	2.77E-01
**CTC-498J12.3**	-0.84	2.25E-01	2.77E-01

**AC093585.6**	-0.92	2.25E-01	2.77E-01
**CHMP3**	0.14	2.25E-01	2.77E-01
**CH17-353B19.1**	0.70	2.25E-01	2.77E-01
**AMOTL2**	-0.22	2.25E-01	2.77E-01
**RP11-51J9.6**	-0.64	2.25E-01	2.77E-01
**GABRG3**	0.85	2.25E-01	2.77E-01
**RNU6-469P**	1.13	2.25E-01	2.77E-01
**GIPR**	0.43	2.25E-01	2.78E-01
**HLA-DPA1**	0.41	2.25E-01	2.78E-01
**FEN1**	0.24	2.25E-01	2.78E-01
**TMEM213**	1.52	2.26E-01	2.78E-01
**KIFAP3**	-0.12	2.26E-01	2.78E-01
**RP11-357G3.2**	-1.61	2.26E-01	2.78E-01
**RP11-348B17.1**	-1.03	2.26E-01	2.78E-01
**RP11-16F15.2**	-1.09	2.26E-01	2.78E-01
**CTA-253N17.1**	0.52	2.26E-01	2.78E-01
**XLOC_005482**	1.46	2.26E-01	2.78E-01
**SESN3**	0.25	2.26E-01	2.78E-01
**RPS26P11**	1.09	2.26E-01	2.78E-01
**KCNMB2**	-1.03	2.26E-01	2.79E-01
**RARB**	0.34	2.27E-01	2.79E-01
**ZKSCAN7**	0.34	2.27E-01	2.79E-01
**G26250**	-0.59	2.27E-01	2.79E-01
**EMC6**	0.68	2.27E-01	2.79E-01
**NET1**	-0.19	2.27E-01	2.79E-01
**CTD-2246P4.1**	-0.90	2.27E-01	2.79E-01
**G8154**	-0.43	2.27E-01	2.79E-01
**XLOC_005542**	-0.57	2.27E-01	2.79E-01
**RP11-274H2.2**	-1.02	2.27E-01	2.79E-01
**RUSC2**	0.17	2.27E-01	2.80E-01
**SLC30A8**	-1.72	2.27E-01	2.80E-01
**PSMC1P1**	0.33	2.27E-01	2.80E-01
**STX3**	0.11	2.27E-01	2.80E-01
**CLDN4**	0.21	2.27E-01	2.80E-01
**RHPN2**	0.93	2.27E-01	2.80E-01
**MLLT10P1**	-1.02	2.28E-01	2.80E-01
**ME2**	-0.22	2.28E-01	2.80E-01
**ASB5**	-1.78	2.28E-01	2.80E-01
**TP53BP2**	-0.10	2.28E-01	2.80E-01
**FAM3C**	0.22	2.28E-01	2.80E-01
**UQCRHL**	0.28	2.28E-01	2.80E-01
**AP000688.1**	-1.33	2.28E-01	2.80E-01
**RNU6-1333P**	-1.57	2.28E-01	2.80E-01
**TMEM87A**	-0.11	2.28E-01	2.80E-01
**TEX22**	-0.58	2.28E-01	2.80E-01

**ZDHHC4P1**	-1.47	2.28E-01	2.80E-01
**TRABD2A**	0.48	2.28E-01	2.81E-01
**RBM39**	-0.15	2.28E-01	2.81E-01
**RIPK1**	-0.12	2.28E-01	2.81E-01
**RP11-1348G14.8**	-0.54	2.29E-01	2.81E-01
**CTB-113P19.3**	-0.67	2.29E-01	2.81E-01
**RP11-315O6.1**	0.70	2.29E-01	2.81E-01
**PCMT1**	0.14	2.29E-01	2.81E-01
**RN7SL559P**	-0.79	2.29E-01	2.81E-01
**RP11-38G5.4**	-0.87	2.29E-01	2.81E-01
**KIAA2012**	-1.12	2.29E-01	2.81E-01
**KIF1C**	0.17	2.29E-01	2.81E-01
**COPS3**	-0.17	2.29E-01	2.81E-01
**GABRR1**	1.17	2.29E-01	2.81E-01
**RABL2B**	0.21	2.29E-01	2.82E-01
**GAL3ST3**	-1.39	2.29E-01	2.82E-01
**WDFY3-AS2**	-0.27	2.29E-01	2.82E-01
**G11276**	0.72	2.29E-01	2.82E-01
**RP11-109G23.3**	-1.26	2.29E-01	2.82E-01
**VAPA**	0.14	2.30E-01	2.82E-01
**C6**	0.92	2.30E-01	2.82E-01
**AC018816.3**	-0.65	2.30E-01	2.82E-01
**GPR137C**	0.37	2.30E-01	2.82E-01
**ANG**	-0.25	2.30E-01	2.82E-01
**CTA-221G9.10**	0.95	2.30E-01	2.82E-01
**CCDC116**	0.67	2.30E-01	2.82E-01
**TEX2**	-0.15	2.30E-01	2.82E-01
**HSPD1P10**	-1.15	2.30E-01	2.82E-01
**CRB1**	-0.82	2.30E-01	2.82E-01
**RP5-837J1.1**	-1.37	2.30E-01	2.82E-01
**RP11-603J24.5**	-0.88	2.30E-01	2.83E-01
**POU5F1P6**	1.24	2.30E-01	2.83E-01
**RP11-456I15.2**	-0.86	2.30E-01	2.83E-01
**CP**	-0.64	2.30E-01	2.83E-01
**RANGRF**	0.79	2.30E-01	2.83E-01
**RP11-710F7.3**	-1.19	2.30E-01	2.83E-01
**RP11-293B20.2**	1.23	2.31E-01	2.83E-01
**RP11-502I4.3**	0.38	2.31E-01	2.83E-01
**RNU4-14P**	-1.52	2.31E-01	2.83E-01
**F13A1**	0.62	2.31E-01	2.83E-01
**RP11-537A6.9**	-1.05	2.31E-01	2.84E-01
**KRT86**	-1.53	2.31E-01	2.84E-01
**AC005740.6**	-0.60	2.31E-01	2.84E-01
**RP11-274B21.9**	0.91	2.31E-01	2.84E-01
**JKAMP**	-0.16	2.31E-01	2.84E-01

**SLC43A1**	0.31	2.31E-01	2.84E-01
**RP11-679B19.1**	-0.50	2.31E-01	2.84E-01
**RN7SKP239**	-1.16	2.31E-01	2.84E-01
**RP11-20I23.8**	-0.81	2.31E-01	2.84E-01
**RP11-317N8.5**	0.45	2.32E-01	2.84E-01
**CHD5**	-0.62	2.32E-01	2.84E-01
**ZNF717**	-0.36	2.32E-01	2.84E-01
**AC118344.1**	-1.20	2.32E-01	2.84E-01
**ITGA2**	-0.26	2.32E-01	2.84E-01
**XLOC_011979**	6.00	2.32E-01	2.85E-01
**FAT4**	-0.50	2.32E-01	2.85E-01
**AKAP3**	-0.36	2.32E-01	2.85E-01
**RP11-444E17.6**	-0.99	2.32E-01	2.85E-01
**DHODH**	0.17	2.32E-01	2.85E-01
**RN7SL336P**	-0.55	2.32E-01	2.85E-01
**GNL3**	0.18	2.32E-01	2.85E-01
**SLC25A4**	0.18	2.32E-01	2.85E-01
**C21orf58**	-0.33	2.32E-01	2.85E-01
**FOXD2**	0.59	2.32E-01	2.85E-01
**BCDIN3D**	0.18	2.32E-01	2.85E-01
**G3900**	-1.16	2.32E-01	2.85E-01
**G39807**	0.63	2.32E-01	2.85E-01
**PNMAL2**	0.30	2.33E-01	2.85E-01
**LINC01578**	-0.23	2.33E-01	2.85E-01
**FBLN1**	0.51	2.33E-01	2.86E-01
**CTB-50L17.9**	0.66	2.33E-01	2.86E-01
**BMP8B**	0.29	2.33E-01	2.86E-01
**LINC00941**	0.87	2.33E-01	2.86E-01
**SPSB3**	0.52	2.33E-01	2.86E-01
**RP11-489E7.4**	0.87	2.33E-01	2.86E-01
**RP11-199F11.2**	-0.54	2.33E-01	2.86E-01
**ISM2**	-1.26	2.33E-01	2.86E-01
**IGKV3D-15**	6.25	2.33E-01	2.86E-01
**RP11-61A14.4**	1.06	2.33E-01	2.86E-01
**CELF2**	-0.26	2.34E-01	2.86E-01
**CCL18**	0.96	2.34E-01	2.87E-01
**PSENEN**	-0.22	2.34E-01	2.87E-01
**RN7SL730P**	-0.98	2.34E-01	2.87E-01
**PRADC1P1**	-0.86	2.34E-01	2.87E-01
**SLC40A1**	0.29	2.34E-01	2.87E-01
**GFOD1-AS1**	-1.14	2.34E-01	2.87E-01
**AP001462.6**	0.58	2.34E-01	2.87E-01
**NBR2**	0.25	2.35E-01	2.88E-01
**CCDC58P5**	-1.34	2.35E-01	2.88E-01
**CAMKMT**	-0.22	2.35E-01	2.88E-01

**SPATA25**	-0.47	2.35E-01	2.88E-01
**PLIN4**	0.87	2.35E-01	2.88E-01
**RP5-1091N2.9**	1.18	2.35E-01	2.88E-01
**COPG2**	-0.21	2.35E-01	2.88E-01
**CBY3**	-0.97	2.35E-01	2.89E-01
**HMGN1P36**	-0.68	2.35E-01	2.89E-01
**bP-2189O9.3**	1.38	2.36E-01	2.89E-01
**ZNF542P**	0.20	2.36E-01	2.89E-01
**NFE2**	-0.38	2.36E-01	2.89E-01
**SIK3**	-0.19	2.36E-01	2.89E-01
**YBX1P2**	-0.60	2.36E-01	2.89E-01
**RP11-130F10.1**	-0.45	2.36E-01	2.89E-01
**AKT3**	-0.30	2.36E-01	2.89E-01
**AL353671.2**	1.34	2.36E-01	2.89E-01
**SPATS2L**	0.15	2.36E-01	2.89E-01
**RP11-379K17.4**	-1.45	2.36E-01	2.90E-01
**ZNF589**	-0.17	2.36E-01	2.90E-01
**CCNDBP1**	-0.17	2.37E-01	2.90E-01
**RP5-884C9.2**	-0.31	2.37E-01	2.90E-01
**SLCO2A1**	0.37	2.37E-01	2.90E-01
**RP3-453I5.2**	-1.40	2.37E-01	2.90E-01
**DLG3**	-0.15	2.37E-01	2.90E-01
**AP000442.1**	-0.46	2.37E-01	2.90E-01
**GPR62**	0.48	2.37E-01	2.90E-01
**ARMT1**	-0.19	2.37E-01	2.90E-01
**ZNF582**	-0.25	2.37E-01	2.90E-01
**LLNLF-158E9.1**	-1.30	2.37E-01	2.90E-01
**CXorf56**	-0.16	2.37E-01	2.90E-01
**XLOC_014272**	-1.01	2.37E-01	2.90E-01
**RNF17**	-1.00	2.37E-01	2.90E-01
**RP11-110I1.13**	1.02	2.37E-01	2.90E-01
**G6008**	-0.67	2.37E-01	2.90E-01
**WASF4P**	-1.30	2.37E-01	2.91E-01
**RP11-64C12.3**	-1.00	2.37E-01	2.91E-01
**RGS2**	-0.34	2.37E-01	2.91E-01
**QPCT**	-0.26	2.37E-01	2.91E-01
**HRASLS5**	-0.69	2.37E-01	2.91E-01
**CTC-325H20.4**	0.26	2.37E-01	2.91E-01
**GDF9**	-0.41	2.38E-01	2.91E-01
**PKMP3**	-0.58	2.38E-01	2.91E-01
**TERF1**	0.11	2.38E-01	2.91E-01
**RP11-338E21.2**	0.93	2.38E-01	2.91E-01
**XCL1**	0.97	2.38E-01	2.91E-01
**NUDT10**	-0.43	2.38E-01	2.92E-01
**CYP2F2P**	-0.70	2.38E-01	2.92E-01

**CACNA1C-AS1**	1.23	2.38E-01	2.92E-01
**TRPC2**	0.96	2.38E-01	2.92E-01
**RP11-253M7.1**	0.67	2.38E-01	2.92E-01
**RP1-102G20.2**	-1.23	2.38E-01	2.92E-01
**PDHB**	0.20	2.38E-01	2.92E-01
**LINC00189**	-0.94	2.38E-01	2.92E-01
**ABCA3**	0.28	2.39E-01	2.92E-01
**RP3-461F17.3**	-0.71	2.39E-01	2.92E-01
**AC006272.1**	-1.69	2.39E-01	2.92E-01
**ABCG5**	-0.70	2.39E-01	2.92E-01
**MAP10**	0.29	2.39E-01	2.92E-01
**REXO2**	-0.13	2.39E-01	2.93E-01
**RP5-966M1.6**	-0.72	2.39E-01	2.93E-01
**CDC123**	0.16	2.39E-01	2.93E-01
**RP11-77K12.9**	0.30	2.39E-01	2.93E-01
**RANP4**	-0.77	2.39E-01	2.93E-01
**NDC80**	0.25	2.39E-01	2.93E-01
**MAFA**	-0.84	2.40E-01	2.93E-01
**RP11-672L10.6**	-0.58	2.40E-01	2.93E-01
**GALNT11**	-0.17	2.40E-01	2.93E-01
**FBXW10**	-0.81	2.40E-01	2.93E-01
**RPL7P24**	-1.38	2.40E-01	2.93E-01
**RN7SL789P**	-0.94	2.40E-01	2.93E-01
**PCDHA12**	-1.20	2.40E-01	2.93E-01
**RP11-17P16.1**	-0.64	2.40E-01	2.93E-01
**RP1-152L7.5**	0.38	2.40E-01	2.93E-01
**BEND3**	-0.25	2.40E-01	2.93E-01
**ARMC10P1**	0.58	2.40E-01	2.94E-01
**ORAOV1**	0.21	2.40E-01	2.94E-01
**FBXO5**	-0.22	2.40E-01	2.94E-01
**G1159**	0.62	2.40E-01	2.94E-01
**WI2-3658N16.1**	0.35	2.40E-01	2.94E-01
**RP11-61E11.2**	-1.16	2.40E-01	2.94E-01
**GXYLT2**	-0.50	2.40E-01	2.94E-01
**PHYHIPL**	-0.46	2.41E-01	2.94E-01
**AC004951.6**	-0.58	2.41E-01	2.94E-01
**RP11-435F17.1**	-1.26	2.41E-01	2.94E-01
**KRTAP5-1**	-1.93	2.41E-01	2.94E-01
**RP11-1060G2.1**	-1.10	2.41E-01	2.94E-01
**RP11-267N12.1**	-1.29	2.41E-01	2.94E-01
**RBM7**	-0.14	2.41E-01	2.94E-01
**YY2**	0.29	2.41E-01	2.95E-01
**UBE2L4**	-1.13	2.41E-01	2.95E-01
**SLC23A2**	0.14	2.41E-01	2.95E-01
**CHRNA4**	-1.14	2.41E-01	2.95E-01

**RP11-73E17.2**	-0.28	2.41E-01	2.95E-01
**MICU1**	0.18	2.42E-01	2.95E-01
**FRG2JP**	-1.34	2.42E-01	2.95E-01
**FUT8**	0.29	2.42E-01	2.95E-01
**AC005537.2**	0.54	2.42E-01	2.95E-01
**AC005329.7**	0.99	2.42E-01	2.95E-01
**CTD-2503O16.4**	-0.97	2.42E-01	2.95E-01
**RP11-363E6.4**	-0.56	2.42E-01	2.95E-01
**CBX3P2**	0.30	2.42E-01	2.95E-01
**AC016582.2**	-0.72	2.42E-01	2.96E-01
**SLCO5A1**	-0.81	2.42E-01	2.96E-01
**DPP10**	-0.72	2.42E-01	2.96E-01
**DBR1**	0.15	2.42E-01	2.96E-01
**MIR361**	-1.84	2.42E-01	2.96E-01
**MGAT4C**	0.75	2.43E-01	2.96E-01
**RN7SL34P**	-0.86	2.43E-01	2.96E-01
**RP11-464F9.22**	-0.59	2.43E-01	2.96E-01
**ACOT13**	-0.13	2.43E-01	2.96E-01
**RNU4-25P**	-1.59	2.43E-01	2.96E-01
**HAPLN2**	0.96	2.43E-01	2.96E-01
**TMEM263**	0.29	2.43E-01	2.96E-01
**WWTR1-IT1**	0.66	2.43E-01	2.97E-01
**RP5-1116H23.3**	-0.76	2.43E-01	2.97E-01
**RNF2**	-0.18	2.43E-01	2.97E-01
**ZNF888**	-0.31	2.43E-01	2.97E-01
**MCEMP1**	1.29	2.43E-01	2.97E-01
**ANKMY2**	0.16	2.44E-01	2.97E-01
**XLOC_003397**	-1.56	2.44E-01	2.97E-01
**RP11-7F17.5**	0.74	2.44E-01	2.97E-01
**TMEM47**	0.31	2.44E-01	2.97E-01
**EMC1**	-0.10	2.44E-01	2.97E-01
**SLC1A3**	-0.25	2.44E-01	2.97E-01
**IPO9-AS1**	-0.80	2.44E-01	2.97E-01
**EFTUD2**	0.11	2.44E-01	2.98E-01
**RP11-46J23.1**	-0.56	2.44E-01	2.98E-01
**RP11-999E24.3**	-0.35	2.44E-01	2.98E-01
**RP11-66N24.4**	-0.57	2.44E-01	2.98E-01
**TSPAN33**	0.36	2.44E-01	2.98E-01
**UTY**	-1.32	2.44E-01	2.98E-01
**HDC**	0.65	2.44E-01	2.98E-01
**PSMD1**	0.16	2.45E-01	2.98E-01
**NIF3L1**	0.15	2.45E-01	2.98E-01
**KLHL4**	-0.51	2.45E-01	2.98E-01
**CTD-2547L24.3**	-0.43	2.45E-01	2.99E-01
**DIAPH3-AS2**	-1.62	2.45E-01	2.99E-01

**EFTUD1**	0.14	2.45E-01	2.99E-01
**RP13-582O9.7**	0.62	2.45E-01	2.99E-01
**PNPLA7**	0.35	2.45E-01	2.99E-01
**SYN2**	0.56	2.46E-01	3.00E-01
**RP11-11N7.4**	-1.04	2.46E-01	3.00E-01
**VAPB**	-0.15	2.46E-01	3.00E-01
**LMNB1**	-0.19	2.46E-01	3.00E-01
**FGF18**	0.87	2.46E-01	3.00E-01
**RP13-554M15.2**	0.74	2.46E-01	3.00E-01
**FAM151A**	0.62	2.46E-01	3.00E-01
**RNASE3**	-0.97	2.46E-01	3.00E-01
**LINC01277**	0.71	2.46E-01	3.00E-01
**RP11-432J22.2**	-0.47	2.46E-01	3.00E-01
**MCTS1**	0.15	2.46E-01	3.00E-01
**FAM71F2**	-0.46	2.46E-01	3.00E-01
**RP11-796E10.1**	-0.88	2.46E-01	3.00E-01
**SLC35G5**	0.76	2.47E-01	3.01E-01
**KIAA0355**	0.09	2.47E-01	3.01E-01
**MRVI1-AS1**	-0.64	2.47E-01	3.01E-01
**CHRFAM7A**	-0.87	2.47E-01	3.01E-01
**NANOS3**	0.93	2.47E-01	3.01E-01
**ZNF133**	0.14	2.47E-01	3.01E-01
**CTD-2095E4.4**	-0.64	2.47E-01	3.01E-01
**XLOC_011520**	1.24	2.47E-01	3.01E-01
**RP1-182O16.2**	-1.19	2.47E-01	3.01E-01
**NUPR1L**	-0.59	2.47E-01	3.01E-01
**RNU6-1053P**	-1.02	2.47E-01	3.01E-01
**AL109947.1**	-1.50	2.47E-01	3.01E-01
**CTA-221G9.12**	-0.33	2.47E-01	3.01E-01
**SMIM2-AS1**	-0.72	2.47E-01	3.01E-01
**CCL13**	0.71	2.47E-01	3.01E-01
**SPIN4-AS1**	1.48	2.48E-01	3.02E-01
**TMEM51-AS1**	0.39	2.48E-01	3.02E-01
**DAPK1**	0.33	2.48E-01	3.02E-01
**GOLGA8I**	-0.93	2.48E-01	3.02E-01
**ACP1**	0.14	2.48E-01	3.02E-01
**CYP51A1-AS1**	0.67	2.48E-01	3.02E-01
**NLRC4**	0.41	2.48E-01	3.02E-01
**C17orf107**	0.28	2.48E-01	3.02E-01
**RNA5SP217**	-1.18	2.48E-01	3.03E-01
**TRBV20-1**	1.03	2.49E-01	3.03E-01
**RP3-370M22.8**	-1.43	2.49E-01	3.03E-01
**CTD-2553L13.9**	0.64	2.49E-01	3.03E-01
**TRAJ20**	-1.73	2.49E-01	3.03E-01
**CAMK1**	0.53	2.49E-01	3.03E-01

**FNBP4**	0.22	2.49E-01	3.03E-01
**ZDHHC16**	0.17	2.49E-01	3.03E-01
**SLC25A30-AS1**	-1.26	2.49E-01	3.03E-01
**CPSF3**	-0.13	2.49E-01	3.03E-01
**CTD-3193K9.11**	1.35	2.49E-01	3.03E-01
**RP11-4C20.4**	0.98	2.49E-01	3.03E-01
**HSPB9**	0.73	2.49E-01	3.03E-01
**ZNF497**	0.38	2.49E-01	3.03E-01
**RP11-760L24.1**	-1.08	2.49E-01	3.03E-01
**RP5-1068E13.7**	-0.33	2.49E-01	3.03E-01
**PARS2**	-0.20	2.49E-01	3.03E-01
**C1orf168**	-1.26	2.49E-01	3.04E-01
**CTD-3105H18.11**	-1.23	2.49E-01	3.04E-01
**XLOC_010295**	6.05	2.49E-01	3.04E-01
**RP11-353N4.4**	-1.05	2.50E-01	3.04E-01
**NUDT7**	-0.28	2.50E-01	3.04E-01
**CPT1B**	0.69	2.50E-01	3.04E-01
**TRIQK**	-0.21	2.50E-01	3.04E-01
**ZNF140**	0.15	2.50E-01	3.04E-01
**NCAPD2**	0.15	2.50E-01	3.04E-01
**RP13-16H11.5**	-1.17	2.50E-01	3.04E-01
**TMEM18**	0.15	2.50E-01	3.04E-01
**SLC36A2**	-1.11	2.50E-01	3.04E-01
**RN7SKP78**	0.64	2.50E-01	3.04E-01
**FAM204A**	-0.12	2.50E-01	3.04E-01
**GAST**	1.91	2.51E-01	3.05E-01
**PROK2**	0.97	2.51E-01	3.05E-01
**BET1**	0.19	2.51E-01	3.05E-01
**CTA-292E10.6**	-0.29	2.51E-01	3.05E-01
**DLX4**	0.39	2.51E-01	3.05E-01
**CTB-129P6.7**	-0.71	2.51E-01	3.06E-01
**RP11-465B22.8**	-0.63	2.51E-01	3.06E-01
**INHBE**	0.78	2.51E-01	3.06E-01
**RP11-109D9.4**	1.01	2.51E-01	3.06E-01
**COL17A1**	-0.20	2.52E-01	3.06E-01
**VBP1**	-0.19	2.52E-01	3.06E-01
**XXbac-BPG157A10.21**	0.83	2.52E-01	3.06E-01
**LINC00377**	-1.70	2.52E-01	3.06E-01
**ANKDD1A**	0.38	2.52E-01	3.06E-01
**CLIC2**	0.39	2.52E-01	3.06E-01
**AL022476.2**	-0.56	2.52E-01	3.06E-01
**CCDC73**	-0.77	2.52E-01	3.06E-01
**FABP7P1**	0.87	2.52E-01	3.06E-01
**C1DP1**	-1.64	2.52E-01	3.06E-01
**FKBP5**	0.33	2.52E-01	3.06E-01

**ZNF415**	0.36	2.52E-01	3.06E-01
**CASC18**	-0.77	2.52E-01	3.07E-01
**C1orf52**	0.10	2.52E-01	3.07E-01
**G32125**	0.56	2.52E-01	3.07E-01
**SLC25A32**	-0.17	2.52E-01	3.07E-01
**SHMT1**	-0.17	2.52E-01	3.07E-01
**AIFM3**	-0.54	2.53E-01	3.07E-01
**SPTBN5**	-0.38	2.53E-01	3.07E-01
**SUMO2P19**	-1.26	2.53E-01	3.07E-01
**SPEF1**	-0.69	2.53E-01	3.07E-01
**UNC5D**	-0.84	2.53E-01	3.07E-01
**RP3-473B4.3**	-1.00	2.53E-01	3.08E-01
**GCNT3**	0.71	2.53E-01	3.08E-01
**TMEM151A**	1.09	2.53E-01	3.08E-01
**RP5-940J5.3**	-1.58	2.53E-01	3.08E-01
**NBPF20**	0.24	2.53E-01	3.08E-01
**ATP1A3**	0.74	2.53E-01	3.08E-01
**PAPLN**	-0.34	2.54E-01	3.08E-01
**SLC20A2**	0.15	2.54E-01	3.08E-01
**HTR3B**	1.30	2.54E-01	3.08E-01
**NCAM2**	-0.41	2.54E-01	3.08E-01
**SLC4A10**	0.88	2.54E-01	3.08E-01
**CASK**	-0.13	2.54E-01	3.08E-01
**UQCRC2**	0.18	2.54E-01	3.08E-01
**RP11-166B2.1**	0.77	2.54E-01	3.08E-01
**DDX12P**	0.36	2.54E-01	3.08E-01
**RP11-128A17.2**	-1.30	2.54E-01	3.09E-01
**RP11-611L7.2**	-0.62	2.54E-01	3.09E-01
**AC006014.8**	-1.09	2.54E-01	3.09E-01
**DPF1**	0.46	2.54E-01	3.09E-01
**PCLO**	-0.37	2.54E-01	3.09E-01
**BX322557.10**	0.31	2.55E-01	3.09E-01
**WFDC10B**	1.21	2.55E-01	3.09E-01
**RP11-165F24.5**	-1.19	2.55E-01	3.10E-01
**RPL32**	0.17	2.55E-01	3.10E-01
**VAMP3**	0.15	2.55E-01	3.10E-01
**RSF1-IT1**	-0.78	2.55E-01	3.10E-01
**LINC00473**	1.40	2.56E-01	3.10E-01
**RP11-10C24.1**	-0.27	2.56E-01	3.10E-01
**LINC00683**	-0.73	2.56E-01	3.11E-01
**GCC1**	-0.13	2.56E-01	3.11E-01
**RP11-22A3.2**	-1.16	2.56E-01	3.11E-01
**XIRP1**	0.96	2.56E-01	3.11E-01
**NPIPA1**	0.34	2.56E-01	3.11E-01
**RP11-438D8.2**	-1.04	2.56E-01	3.11E-01

**DNASE1L2**	-0.40	2.56E-01	3.11E-01
**HCG15**	-0.71	2.56E-01	3.11E-01
**SLC12A2**	-0.52	2.56E-01	3.11E-01
**ESPL1**	-0.22	2.56E-01	3.11E-01
**PARP15**	-0.57	2.57E-01	3.11E-01
**NUTM2A**	-0.60	2.57E-01	3.12E-01
**RP1-56K13.2**	-0.61	2.57E-01	3.12E-01
**TRANK1**	0.29	2.57E-01	3.12E-01
**CCDC81**	0.43	2.57E-01	3.12E-01
**RP11-138B9.1**	-1.16	2.57E-01	3.12E-01
**MRC1**	1.11	2.57E-01	3.12E-01
**RP11-680G10.1**	-1.51	2.57E-01	3.12E-01
**RP11-255E6.6**	0.91	2.57E-01	3.12E-01
**EDC4**	-0.13	2.57E-01	3.12E-01
**PPP1R27**	-0.64	2.57E-01	3.12E-01
**RP11-496D24.2**	-1.32	2.58E-01	3.12E-01
**THOC2**	-0.12	2.58E-01	3.12E-01
**RP3-404F18.5**	0.72	2.58E-01	3.12E-01
**MRPS17**	0.21	2.58E-01	3.13E-01
**XLOC_009869**	-0.92	2.58E-01	3.13E-01
**CTD-2033A16.2**	-1.09	2.58E-01	3.13E-01
**RPS12P5**	1.21	2.58E-01	3.13E-01
**KRTAP5-4**	1.11	2.58E-01	3.13E-01
**RP11-253I19.3**	-0.66	2.58E-01	3.13E-01
**CWH43**	0.24	2.58E-01	3.13E-01
**RP11-44M6.3**	-0.68	2.58E-01	3.13E-01
**VDAC1P4**	-1.05	2.58E-01	3.13E-01
**RP1-92O14.3**	-0.33	2.58E-01	3.13E-01
**RP11-567J20.1**	1.05	2.59E-01	3.14E-01
**LRP12**	-0.17	2.59E-01	3.14E-01
**G16904**	-0.46	2.59E-01	3.14E-01
**CTA-445C9.14**	-0.25	2.59E-01	3.14E-01
**RP11-157F20.3**	-0.91	2.59E-01	3.14E-01
**RABL2A**	-0.24	2.59E-01	3.14E-01
**SPDYE6**	-0.45	2.59E-01	3.14E-01
**GPR143**	-0.35	2.59E-01	3.14E-01
**G38528**	0.92	2.59E-01	3.14E-01
**DNAJC15**	0.22	2.59E-01	3.14E-01
**RP11-95P2.3**	-0.43	2.59E-01	3.14E-01
**FSD1L**	-0.30	2.60E-01	3.14E-01
**SERPINB8**	0.25	2.60E-01	3.15E-01
**C5orf24**	-0.14	2.60E-01	3.15E-01
**G29048**	0.64	2.60E-01	3.15E-01
**TMEM251**	-0.21	2.60E-01	3.15E-01
**DUSP18**	0.18	2.60E-01	3.15E-01

**MCCC2**	-0.12	2.60E-01	3.15E-01
**RP11-989F5.1**	-1.62	2.60E-01	3.15E-01
**CKAP2**	-0.21	2.60E-01	3.16E-01
**RP11-418J17.1**	-0.24	2.61E-01	3.16E-01
**SPAG8**	-0.46	2.61E-01	3.16E-01
**C22orf31**	-0.40	2.61E-01	3.16E-01
**RP11-417L19.5**	-0.53	2.61E-01	3.16E-01
**LINC00470**	-2.02	2.61E-01	3.16E-01
**RP11-758M4.4**	-0.96	2.61E-01	3.16E-01
**XLOC_008666**	-0.50	2.61E-01	3.16E-01
**RP11-356N1.2**	-0.69	2.61E-01	3.16E-01
**CTAGE8**	-0.59	2.61E-01	3.17E-01
**RP11-298C3.2**	-1.04	2.62E-01	3.17E-01
**RP11-130L8.2**	-0.85	2.62E-01	3.17E-01
**AP001604.3**	-1.38	2.62E-01	3.17E-01
**BRWD1-AS1**	-0.86	2.62E-01	3.17E-01
**G3878**	1.15	2.62E-01	3.17E-01
**AL357515.1**	-0.64	2.62E-01	3.18E-01
**RMND5B**	0.15	2.62E-01	3.18E-01
**RP11-661A12.12**	0.76	2.63E-01	3.18E-01
**BBS12**	-0.19	2.63E-01	3.18E-01
**SLBP**	-0.14	2.63E-01	3.18E-01
**ZNF425**	-0.19	2.63E-01	3.18E-01
**RP1-170O19.24**	0.93	2.63E-01	3.18E-01
**TM4SF1-AS1**	1.11	2.63E-01	3.18E-01
**HIST1H2APS4**	-1.33	2.63E-01	3.18E-01
**EPHA6**	-0.69	2.63E-01	3.19E-01
**RP11-398E10.1**	-1.12	2.63E-01	3.19E-01
**RP3-325F22.5**	-0.64	2.63E-01	3.19E-01
**CTB-147C22.9**	-0.64	2.63E-01	3.19E-01
**RAB39B**	-0.54	2.64E-01	3.19E-01
**MMRN1**	0.47	2.64E-01	3.19E-01
**C7orf25**	-0.34	2.64E-01	3.19E-01
**AC006111.1**	-0.91	2.64E-01	3.19E-01
**RP11-116O18.1**	0.86	2.64E-01	3.19E-01
**IGHV4-59**	1.61	2.64E-01	3.20E-01
**SLC7A13**	-1.38	2.64E-01	3.20E-01
**SNORA7**	-0.54	2.64E-01	3.20E-01
**CBR3**	0.21	2.64E-01	3.20E-01
**TMEM262**	-0.45	2.64E-01	3.20E-01
**SLC25A5P1**	-1.10	2.64E-01	3.20E-01
**MBTPS2**	-0.13	2.64E-01	3.20E-01
**GJB1**	0.49	2.64E-01	3.20E-01
**FAM177A1**	0.15	2.64E-01	3.20E-01
**HMGN1P38**	0.68	2.64E-01	3.20E-01

**HLA-J**	-0.85	2.64E-01	3.20E-01
**G11189**	-0.55	2.65E-01	3.20E-01
**SMPDL3B**	0.41	2.65E-01	3.20E-01
**RPL35A**	0.18	2.65E-01	3.20E-01
**JADE3**	-0.12	2.65E-01	3.20E-01
**RNF128**	-0.27	2.65E-01	3.20E-01
**AC013474.4**	-1.25	2.65E-01	3.21E-01
**TPT1P5**	-0.95	2.65E-01	3.21E-01
**NPFFR2**	1.17	2.65E-01	3.21E-01
**RP11-234K24.6**	1.28	2.65E-01	3.21E-01
**RP11-718B12.2**	-1.30	2.66E-01	3.21E-01
**METTL23**	0.13	2.66E-01	3.21E-01
**AC007950.2**	-0.78	2.66E-01	3.21E-01
**RHAG**	1.08	2.66E-01	3.21E-01
**SORT1**	-0.15	2.66E-01	3.21E-01
**RP11-713M15.2**	1.08	2.66E-01	3.22E-01
**COA5**	0.12	2.66E-01	3.22E-01
**PTCH2**	0.63	2.66E-01	3.22E-01
**ASXL3**	0.69	2.66E-01	3.22E-01
**RNF14**	-0.13	2.66E-01	3.22E-01
**AC008982.2**	-0.45	2.66E-01	3.22E-01
**RP11-50I19.2**	-0.36	2.67E-01	3.22E-01
**RP11-403I13.5**	0.47	2.67E-01	3.22E-01
**G38146**	-0.50	2.67E-01	3.22E-01
**RP11-205A8.4**	-0.79	2.67E-01	3.22E-01
**RBM5-AS1**	0.57	2.67E-01	3.22E-01
**CCT4P2**	1.40	2.67E-01	3.22E-01
**FAM103A1**	0.22	2.67E-01	3.22E-01
**GOLGA8K**	0.89	2.67E-01	3.22E-01
**RP11-505E24.3**	-0.88	2.67E-01	3.22E-01
**PTCHD3**	0.89	2.67E-01	3.22E-01
**STK4-AS1**	0.53	2.67E-01	3.23E-01
**RP11-421L21.3**	-0.32	2.67E-01	3.23E-01
**MYHAS**	0.91	2.67E-01	3.23E-01
**LINC01122**	-1.37	2.67E-01	3.23E-01
**G913**	-0.65	2.68E-01	3.23E-01
**NBPF9**	-0.20	2.68E-01	3.23E-01
**GPN1**	0.13	2.68E-01	3.23E-01
**DENND5B-AS1**	-0.98	2.68E-01	3.24E-01
**RP11-540O11.1**	-0.48	2.68E-01	3.24E-01
**PINK1-AS**	-0.21	2.68E-01	3.24E-01
**ZNF627**	-0.16	2.68E-01	3.24E-01
**FAM131A**	0.16	2.68E-01	3.24E-01
**RP11-385F5.5**	-0.42	2.69E-01	3.24E-01
**RP11-365O16.1**	-1.23	2.69E-01	3.24E-01

**ZNF446**	0.22	2.69E-01	3.24E-01
**CHRNA1**	1.40	2.69E-01	3.24E-01
**CTC-428G20.2**	-1.10	2.69E-01	3.25E-01
**CTAGE5**	-0.25	2.69E-01	3.25E-01
**CTD-2035E11.5**	-0.49	2.69E-01	3.25E-01
**EPHA1-AS1**	-0.41	2.69E-01	3.25E-01
**G26124**	-0.96	2.69E-01	3.25E-01
**PPIAP16**	-0.93	2.69E-01	3.25E-01
**RP11-73M18.7**	-0.33	2.69E-01	3.25E-01
**BRCC3**	-0.15	2.69E-01	3.25E-01
**NKPD1**	0.36	2.69E-01	3.25E-01
**RP3-395M20.9**	0.71	2.69E-01	3.25E-01
**RERGL**	0.34	2.70E-01	3.25E-01
**TMEM257**	-0.95	2.70E-01	3.25E-01
**CTD-3032J10.2**	-1.21	2.70E-01	3.25E-01
**G7779**	0.96	2.70E-01	3.25E-01
**NAP1L1P1**	0.97	2.70E-01	3.25E-01
**FDX1**	-0.16	2.70E-01	3.25E-01
**XLOC_005944**	0.84	2.70E-01	3.25E-01
**RP11-158I9.8**	0.33	2.70E-01	3.25E-01
**XLOC_013914**	-0.83	2.70E-01	3.26E-01
**G23691**	0.82	2.70E-01	3.26E-01
**PRRT3-AS1**	-0.48	2.70E-01	3.26E-01
**AF127936.7**	-0.61	2.70E-01	3.26E-01
**ALG13-AS1**	-0.65	2.70E-01	3.26E-01
**RP11-554D14.4**	-1.33	2.70E-01	3.26E-01
**STAM-AS1**	-0.54	2.70E-01	3.26E-01
**PRUNE2**	-0.31	2.70E-01	3.26E-01
**SEPHS1P6**	1.10	2.71E-01	3.26E-01
**CLP1**	0.16	2.71E-01	3.26E-01
**AL033378.1**	-1.23	2.71E-01	3.26E-01
**OSR2**	0.43	2.71E-01	3.27E-01
**UBE2J1**	0.18	2.71E-01	3.27E-01
**RP11-1096D5.2**	1.78	2.71E-01	3.27E-01
**SPG21**	0.15	2.71E-01	3.27E-01
**TRAJ31**	1.04	2.71E-01	3.27E-01
**G32331**	-0.78	2.71E-01	3.27E-01
**BCAT2**	0.39	2.71E-01	3.27E-01
**RP4-669P10.20**	0.52	2.72E-01	3.27E-01
**MYO18A**	0.12	2.72E-01	3.27E-01
**RP5-894D12.4**	-1.28	2.72E-01	3.27E-01
**NANOGP8**	-1.46	2.72E-01	3.28E-01
**MAP1LC3B2**	-0.43	2.72E-01	3.28E-01
**CD69**	0.45	2.72E-01	3.28E-01
**CCDC160**	1.25	2.72E-01	3.28E-01

**HM13-IT1**	0.63	2.72E-01	3.28E-01
**GMPR**	0.33	2.72E-01	3.28E-01
**CCDC90B**	-0.18	2.72E-01	3.28E-01
**TUBGCP6**	0.27	2.72E-01	3.28E-01
**G38688**	0.55	2.72E-01	3.28E-01
**RP11-188P17.2**	-0.96	2.72E-01	3.28E-01
**RP11-141M3.6**	-0.58	2.72E-01	3.28E-01
**ANXA1**	0.21	2.72E-01	3.28E-01
**SIDT1**	0.40	2.73E-01	3.29E-01
**ANKH**	0.12	2.73E-01	3.29E-01
**RP11-172C16.4**	-1.54	2.73E-01	3.29E-01
**CTB-129O4.1**	1.21	2.73E-01	3.29E-01
**MYH11**	0.42	2.73E-01	3.29E-01
**AP000807.2**	-0.93	2.73E-01	3.29E-01
**GAPDHP76**	-1.19	2.73E-01	3.29E-01
**RP11-1212A22.1**	0.35	2.73E-01	3.29E-01
**RP11-332H14.1**	0.99	2.73E-01	3.29E-01
**ST13P5**	-1.23	2.74E-01	3.29E-01
**RP11-49G2.3**	-0.82	2.74E-01	3.30E-01
**RP11-66B24.4**	-0.27	2.74E-01	3.30E-01
**CTD-2196E14.6**	-0.50	2.74E-01	3.30E-01
**RP11-761I4.5**	-0.86	2.74E-01	3.30E-01
**TSPAN2**	0.31	2.74E-01	3.30E-01
**EVC**	-0.15	2.74E-01	3.30E-01
**RP11-617B3.2**	-1.47	2.74E-01	3.30E-01
**PIGG**	0.13	2.74E-01	3.30E-01
**DDB1**	-0.11	2.74E-01	3.30E-01
**RP11-651L5.3**	-0.61	2.74E-01	3.30E-01
**TUBBP1**	-0.74	2.74E-01	3.30E-01
**PPP3R1**	0.14	2.74E-01	3.30E-01
**MIR186**	-0.53	2.74E-01	3.30E-01
**RP11-702H23.2**	-0.91	2.75E-01	3.31E-01
**RP5-1050D4.5**	0.55	2.75E-01	3.31E-01
**TNFAIP8L2**	0.44	2.75E-01	3.31E-01
**FAM27A**	-0.51	2.75E-01	3.31E-01
**A3GALT2**	-1.22	2.75E-01	3.31E-01
**ENPP7P7**	-0.72	2.75E-01	3.31E-01
**RP3-495K2.2**	0.92	2.75E-01	3.31E-01
**AC112198.1**	0.95	2.75E-01	3.31E-01
**ZNF750**	0.19	2.75E-01	3.31E-01
**RP11-654A16.3**	-0.77	2.75E-01	3.31E-01
**SNORD123**	-1.04	2.76E-01	3.32E-01
**MIR135A1**	0.67	2.76E-01	3.32E-01
**RP11-384L8.1**	0.31	2.76E-01	3.32E-01
**PDHA1**	0.24	2.76E-01	3.32E-01

**FCRL6**	0.49	2.76E-01	3.32E-01
**RP11-291B21.2**	-1.00	2.76E-01	3.32E-01
**PI16**	-0.51	2.76E-01	3.32E-01
**EGFEM1P**	1.19	2.76E-01	3.32E-01
**CLUAP1**	0.18	2.76E-01	3.32E-01
**RAB41**	-0.40	2.76E-01	3.32E-01
**CCDC144CP**	-0.81	2.76E-01	3.33E-01
**FAM177B**	-0.84	2.77E-01	3.33E-01
**RP5-915N17.11**	-1.07	2.77E-01	3.33E-01
**RP3-375P9.2**	-1.28	2.77E-01	3.33E-01
**UBE2F**	0.12	2.77E-01	3.33E-01
**EIF4E**	-0.14	2.77E-01	3.33E-01
**G27789**	-1.04	2.77E-01	3.33E-01
**RP11-370F5.4**	-1.10	2.77E-01	3.33E-01
**MRPL35P3**	-1.29	2.77E-01	3.33E-01
**RP11-96H19.1**	0.39	2.77E-01	3.33E-01
**LINC01121**	1.10	2.77E-01	3.34E-01
**FAM3B**	0.57	2.77E-01	3.34E-01
**CTB-109A12.1**	-0.92	2.78E-01	3.34E-01
**CFTR**	0.71	2.78E-01	3.34E-01
**HADHB**	0.15	2.78E-01	3.34E-01
**IGF2BP2**	0.29	2.78E-01	3.34E-01
**RP1-18D14.7**	-0.80	2.78E-01	3.34E-01
**FUCA1**	0.22	2.78E-01	3.34E-01
**FPR2**	0.77	2.78E-01	3.34E-01
**CD68**	-1.44	2.78E-01	3.35E-01
**KCNN1**	0.77	2.78E-01	3.35E-01
**GRK4**	0.25	2.78E-01	3.35E-01
**RP11-529E10.6**	-0.37	2.78E-01	3.35E-01
**ZNF781**	-0.51	2.78E-01	3.35E-01
**RP11-455G16.1**	-0.78	2.79E-01	3.35E-01
**COPS5**	-0.11	2.79E-01	3.35E-01
**RAET1E-AS1**	-0.25	2.79E-01	3.35E-01
**TMEM30C**	-1.06	2.79E-01	3.35E-01
**AC009005.2**	0.78	2.79E-01	3.35E-01
**SIRPB1**	0.99	2.79E-01	3.35E-01
**DHFRP1**	1.02	2.79E-01	3.36E-01
**C11orf73**	0.12	2.80E-01	3.36E-01
**NTN3**	-0.94	2.80E-01	3.36E-01
**TMEM71**	-0.43	2.80E-01	3.36E-01
**RP11-22P6.2**	0.72	2.80E-01	3.36E-01
**AC005304.1**	-1.16	2.80E-01	3.36E-01
**RP11-337N6.3**	-0.84	2.80E-01	3.36E-01
**NCAPGP1**	1.11	2.80E-01	3.36E-01
**RP11-7O11.3**	0.54	2.80E-01	3.36E-01

**STARD4-AS1**	-0.34	2.80E-01	3.36E-01
**RP11-461O7.1**	-0.61	2.80E-01	3.36E-01
**ANKRD20A8P**	1.16	2.80E-01	3.36E-01
**G31133**	-0.49	2.80E-01	3.36E-01
**MIR126**	0.99	2.80E-01	3.36E-01
**SIAH2-AS1**	-0.47	2.80E-01	3.37E-01
**BNIP3L**	0.22	2.80E-01	3.37E-01
**RP4-686C3.7**	1.08	2.80E-01	3.37E-01
**STX6**	0.12	2.81E-01	3.37E-01
**PPIL1**	0.23	2.81E-01	3.37E-01
**LIN52**	0.13	2.81E-01	3.37E-01
**PKD2**	0.14	2.81E-01	3.37E-01
**CYP2A6**	-0.54	2.81E-01	3.37E-01
**PDCD6**	-0.09	2.81E-01	3.37E-01
**LIF**	0.49	2.81E-01	3.37E-01
**DRAM2**	0.12	2.81E-01	3.38E-01
**XLOC_001061**	1.23	2.81E-01	3.38E-01
**RP11-225N10.1**	-1.48	2.81E-01	3.38E-01
**G17695**	-1.52	2.81E-01	3.38E-01
**RP11-848P1.5**	0.81	2.81E-01	3.38E-01
**RP11-164J13.1**	-0.53	2.81E-01	3.38E-01
**NAP1L1**	0.14	2.81E-01	3.38E-01
**hsa-mir-3181**	-0.98	2.82E-01	3.38E-01
**CCBL2**	-0.15	2.82E-01	3.38E-01
**SAPCD1**	-0.79	2.82E-01	3.38E-01
**TBX22**	1.11	2.82E-01	3.38E-01
**SREK1IP1**	-0.19	2.82E-01	3.38E-01
**RP13-225O21.5**	-1.13	2.82E-01	3.39E-01
**RP11-159D12.6**	-0.36	2.82E-01	3.39E-01
**AGAP11**	-1.05	2.82E-01	3.39E-01
**CTC-490E21.11**	0.51	2.82E-01	3.39E-01
**TMEFF2**	-0.89	2.82E-01	3.39E-01
**MRPL30**	-0.14	2.83E-01	3.39E-01
**GUCY2D**	0.50	2.83E-01	3.39E-01
**RP11-831F12.3**	-0.76	2.83E-01	3.39E-01
**G31329**	0.69	2.83E-01	3.39E-01
**CTC-428G20.1**	-0.78	2.83E-01	3.39E-01
**G22374**	-0.51	2.83E-01	3.39E-01
**CCNB1IP1**	0.13	2.83E-01	3.39E-01
**DIS3L2**	0.15	2.83E-01	3.39E-01
**NPAS2**	0.32	2.83E-01	3.39E-01
**RUFY4**	-1.39	2.83E-01	3.40E-01
**PRKAR2A-AS1**	0.39	2.83E-01	3.40E-01
**SNRPCP16**	-1.37	2.84E-01	3.40E-01
**STEAP2-AS1**	-1.13	2.84E-01	3.40E-01

**IFT172**	-0.15	2.84E-01	3.40E-01
**RP11-418H16.1**	-0.90	2.84E-01	3.40E-01
**XKR6**	-0.26	2.84E-01	3.40E-01
**AKAP7**	0.21	2.84E-01	3.40E-01
**RP11-70C1.3**	-1.16	2.84E-01	3.40E-01
**RP11-37L2.1**	-0.89	2.84E-01	3.41E-01
**AC007040.11**	-0.98	2.85E-01	3.41E-01
**RP1-43E13.2**	-0.67	2.85E-01	3.41E-01
**BBOX1**	-0.30	2.85E-01	3.41E-01
**PTPRO**	-0.48	2.85E-01	3.42E-01
**RP11-634H22.1**	0.74	2.85E-01	3.42E-01
**BTF3L4P2**	-0.65	2.85E-01	3.42E-01
**S100A3**	0.68	2.85E-01	3.42E-01
**RP11-702H23.4**	-1.37	2.85E-01	3.42E-01
**OGFOD1**	-0.13	2.85E-01	3.42E-01
**SPACA4**	-0.55	2.86E-01	3.42E-01
**FBXO24**	-0.51	2.86E-01	3.42E-01
**RP11-54O7.1**	-1.14	2.86E-01	3.42E-01
**ACY1**	0.41	2.86E-01	3.42E-01
**AL132868.1**	-1.23	2.86E-01	3.42E-01
**AMELX**	-1.63	2.86E-01	3.42E-01
**AC007879.7**	0.73	2.86E-01	3.42E-01
**G36500**	-0.83	2.86E-01	3.42E-01
**RNU6-130P**	-0.69	2.86E-01	3.43E-01
**RTN4IP1**	0.18	2.86E-01	3.43E-01
**BAALC-AS1**	0.22	2.86E-01	3.43E-01
**RP11-692N5.2**	-0.88	2.86E-01	3.43E-01
**G16208**	5.61	2.86E-01	3.43E-01
**G40027**	-0.81	2.86E-01	3.43E-01
**RP11-47P18.1**	-2.80	2.86E-01	3.43E-01
**CRCP**	-0.12	2.86E-01	3.43E-01
**LINC01468**	0.94	2.86E-01	3.43E-01
**RARSP1**	-1.44	2.86E-01	3.43E-01
**VIPR1**	0.25	2.86E-01	3.43E-01
**TTC38**	0.25	2.86E-01	3.43E-01
**RP11-677M14.3**	0.47	2.86E-01	3.43E-01
**AC092669.3**	-0.53	2.87E-01	3.43E-01
**RP11-532F12.5**	0.27	2.87E-01	3.43E-01
**C2CD2L**	0.18	2.87E-01	3.43E-01
**TMEM14B**	0.17	2.87E-01	3.44E-01
**NAMA**	-1.06	2.87E-01	3.44E-01
**ZNF98**	-0.92	2.87E-01	3.44E-01
**RP11-65B7.2**	-1.33	2.87E-01	3.44E-01
**PLPP3**	-0.37	2.88E-01	3.44E-01
**RP11-646E18.4**	0.51	2.88E-01	3.44E-01

**DDX59**	-0.11	2.88E-01	3.44E-01
**AC002550.6**	0.48	2.88E-01	3.44E-01
**RP11-70C1.1**	-1.12	2.88E-01	3.44E-01
**R3HDM1**	0.15	2.88E-01	3.44E-01
**G19167**	-0.97	2.88E-01	3.44E-01
**CHPT1**	-0.24	2.88E-01	3.44E-01
**AC005301.8**	-1.09	2.88E-01	3.44E-01
**ERC2**	0.51	2.88E-01	3.45E-01
**CCR7**	0.50	2.88E-01	3.45E-01
**CES1**	-0.46	2.88E-01	3.45E-01
**DUX4L50**	-0.34	2.88E-01	3.45E-01
**RP11-697E2.9**	-0.84	2.88E-01	3.45E-01
**ADRA1A**	-0.62	2.88E-01	3.45E-01
**RBMS1**	0.11	2.89E-01	3.45E-01
**JARID2**	-0.17	2.89E-01	3.45E-01
**MIXL1**	-0.86	2.89E-01	3.45E-01
**CTD-2373H9.3**	0.63	2.89E-01	3.45E-01
**PDLIM4**	0.24	2.89E-01	3.46E-01
**EFCAB2**	0.38	2.89E-01	3.46E-01
**AOX3P**	-0.96	2.89E-01	3.46E-01
**CIITA**	-0.23	2.89E-01	3.46E-01
**TRAV13-1**	1.04	2.89E-01	3.46E-01
**PCAT14**	0.89	2.89E-01	3.46E-01
**ADAMTS7P3**	0.60	2.89E-01	3.46E-01
**KCTD7**	0.25	2.89E-01	3.46E-01
**NDUFAF4**	-0.20	2.90E-01	3.46E-01
**RP11-109P11.1**	-0.72	2.90E-01	3.46E-01
**G20113**	-0.66	2.90E-01	3.47E-01
**PNMT**	0.96	2.90E-01	3.47E-01
**SRSF10P1**	-1.28	2.90E-01	3.47E-01
**ABCC12**	-1.73	2.90E-01	3.47E-01
**TRAPPC3L**	1.10	2.90E-01	3.47E-01
**FXYD7**	0.90	2.90E-01	3.47E-01
**KCNJ16**	-0.79	2.91E-01	3.47E-01
**AK4P3**	-0.93	2.91E-01	3.47E-01
**RPS24**	-0.19	2.91E-01	3.48E-01
**MGST2**	0.17	2.91E-01	3.48E-01
**RP11-231N9.1**	-1.44	2.91E-01	3.48E-01
**CCDC144A**	-1.12	2.91E-01	3.48E-01
**FABP5P2**	0.88	2.91E-01	3.48E-01
**PVRL3-AS1**	-0.88	2.91E-01	3.48E-01
**KIAA1257**	0.34	2.92E-01	3.48E-01
**RP11-507J18.2**	-1.23	2.92E-01	3.49E-01
**RP11-770J1.3**	0.71	2.92E-01	3.49E-01
**PIGC**	0.14	2.92E-01	3.49E-01

**EPB41L3**	0.22	2.92E-01	3.49E-01
**RP11-399K21.11**	-1.23	2.92E-01	3.49E-01
**LHX8**	5.53	2.92E-01	3.49E-01
**FAM114A2**	0.13	2.92E-01	3.49E-01
**RNF34**	-0.12	2.92E-01	3.49E-01
**C12orf71**	-0.86	2.92E-01	3.49E-01
**C3orf20**	-0.55	2.92E-01	3.49E-01
**HPX**	0.43	2.93E-01	3.50E-01
**FAM212B**	0.18	2.93E-01	3.50E-01
**MIS12**	-0.17	2.93E-01	3.50E-01
**RCVRN**	-0.61	2.93E-01	3.50E-01
**RP11-187A9.3**	0.57	2.93E-01	3.50E-01
**IFITM4P**	-1.23	2.93E-01	3.50E-01
**AGAP8**	0.66	2.93E-01	3.50E-01
**ABCG1**	0.27	2.93E-01	3.50E-01
**RP11-160O5.1**	-0.45	2.93E-01	3.50E-01
**XLOC_005081**	1.19	2.93E-01	3.50E-01
**G32994**	0.62	2.93E-01	3.50E-01
**UBE2QL1**	0.59	2.93E-01	3.50E-01
**RPL21P44**	-0.50	2.94E-01	3.51E-01
**TTC8**	-0.14	2.94E-01	3.51E-01
**LMF1-AS1**	-0.68	2.94E-01	3.51E-01
**TBX15**	0.40	2.94E-01	3.51E-01
**KEL**	0.67	2.94E-01	3.51E-01
**CD27-AS1**	0.30	2.94E-01	3.51E-01
**CHRM3**	0.82	2.94E-01	3.51E-01
**RP11-572O17.1**	-0.49	2.94E-01	3.51E-01
**P2RX1**	0.48	2.94E-01	3.51E-01
**AC104306.1**	-1.27	2.95E-01	3.52E-01
**RP11-755E23.2**	-0.88	2.95E-01	3.52E-01
**LINC00115**	-0.42	2.95E-01	3.52E-01
**CMTM1**	0.42	2.95E-01	3.52E-01
**P2RY11**	-0.25	2.95E-01	3.52E-01
**NCALD**	0.51	2.95E-01	3.52E-01
**RP11-889D3.2**	2.14	2.95E-01	3.52E-01
**XRN2**	0.12	2.95E-01	3.52E-01
**RP11-42O15.3**	-0.81	2.95E-01	3.52E-01
**SEMA4G**	-0.30	2.95E-01	3.52E-01
**UNC5CL**	0.39	2.95E-01	3.52E-01
**RP11-338E21.3**	-1.34	2.95E-01	3.53E-01
**DDB2**	0.14	2.96E-01	3.53E-01
**FAHD2B**	0.19	2.96E-01	3.53E-01
**TIGD2**	0.15	2.96E-01	3.53E-01
**XXbac-BPG249D20.9**	-0.61	2.96E-01	3.53E-01
**RAB4A**	-0.14	2.96E-01	3.53E-01

**AC139099.5**	-0.88	2.96E-01	3.53E-01
**RP11-96C23.13**	-1.20	2.96E-01	3.53E-01
**G26311**	1.15	2.96E-01	3.53E-01
**CHD1L**	-0.12	2.97E-01	3.54E-01
**CTD-2651B20.3**	-0.73	2.97E-01	3.54E-01
**AC015815.2**	-1.18	2.97E-01	3.54E-01
**ETNK2**	0.30	2.97E-01	3.54E-01
**CTD-2288F12.1**	-0.51	2.97E-01	3.54E-01
**AC079305.10**	0.43	2.97E-01	3.54E-01
**SLA**	0.32	2.97E-01	3.54E-01
**GNMT**	0.62	2.97E-01	3.54E-01
**CTD-3131K8.2**	-0.40	2.97E-01	3.54E-01
**ZNF142**	0.17	2.97E-01	3.54E-01
**AL137127.1**	-0.76	2.97E-01	3.55E-01
**RP11-1024P17.1**	-0.32	2.97E-01	3.55E-01
**CTC-325J23.2**	-1.10	2.98E-01	3.55E-01
**LINC00674**	0.23	2.98E-01	3.55E-01
**RP11-755F10.3**	-0.69	2.98E-01	3.55E-01
**PAPSS1**	0.13	2.98E-01	3.55E-01
**LA16c-385E7.1**	-1.13	2.98E-01	3.55E-01
**AMBP**	-0.59	2.98E-01	3.55E-01
**CTD-2530N21.4**	-0.43	2.98E-01	3.55E-01
**RP5-1057I20.4**	-0.76	2.98E-01	3.55E-01
**RP11-251G23.2**	0.83	2.98E-01	3.55E-01
**XLOC_013863**	-0.90	2.98E-01	3.55E-01
**RP4-647J21.1**	0.62	2.98E-01	3.55E-01
**RP11-760H22.2**	-0.25	2.98E-01	3.55E-01
**RPS17P1**	-1.25	2.98E-01	3.56E-01
**PARP2**	-0.12	2.99E-01	3.56E-01
**CATSPERB**	-0.41	2.99E-01	3.56E-01
**GABARAPL2**	0.19	2.99E-01	3.56E-01
**MRPL35P2**	-0.86	2.99E-01	3.56E-01
**G31837**	0.67	2.99E-01	3.56E-01
**8-Sep**	0.16	2.99E-01	3.56E-01
**C16orf87**	-0.17	2.99E-01	3.56E-01
**SOX21-AS1**	-0.19	2.99E-01	3.56E-01
**TPI1P2**	-0.29	2.99E-01	3.57E-01
**THAP10**	0.24	3.00E-01	3.57E-01
**RP11-10C24.3**	0.26	3.00E-01	3.57E-01
**RP11-46F15.2**	0.69	3.00E-01	3.57E-01
**RP11-320P7.2**	-1.07	3.00E-01	3.57E-01
**bP-2171C21.4**	1.15	3.00E-01	3.58E-01
**ZNF391**	0.23	3.00E-01	3.58E-01
**PIGW**	-0.20	3.00E-01	3.58E-01
**CLDN14**	0.83	3.00E-01	3.58E-01

**DHRS9**	-0.44	3.01E-01	3.58E-01
**CTB-75G16.3**	-1.15	3.01E-01	3.58E-01
**ANP32E**	-0.19	3.01E-01	3.58E-01
**FAM168A**	0.12	3.01E-01	3.58E-01
**GTF3C2**	0.12	3.01E-01	3.58E-01
**PPTC7**	0.16	3.01E-01	3.58E-01
**BRAFP1**	-1.02	3.01E-01	3.59E-01
**AC087793.1**	-0.81	3.01E-01	3.59E-01
**RP11-136K14.3**	-1.01	3.02E-01	3.59E-01
**AC008592.5**	-0.87	3.02E-01	3.59E-01
**ALKBH1**	0.11	3.02E-01	3.59E-01
**MRPL48**	0.18	3.02E-01	3.59E-01
**OR1L8**	0.94	3.02E-01	3.59E-01
**EHHADH**	0.18	3.02E-01	3.59E-01
**ZSCAN30**	-0.17	3.02E-01	3.59E-01
**RPL10AP6**	0.27	3.02E-01	3.60E-01
**TONSL-AS1**	-1.00	3.02E-01	3.60E-01
**SREK1**	0.20	3.02E-01	3.60E-01
**RP11-247A12.7**	1.02	3.02E-01	3.60E-01
**RP11-194N12.2**	0.62	3.02E-01	3.60E-01
**AF001548.5**	-0.46	3.02E-01	3.60E-01
**RP11-386G11.5**	-0.55	3.03E-01	3.60E-01
**G7791**	0.65	3.03E-01	3.60E-01
**AC137932.6**	0.77	3.03E-01	3.60E-01
**NPIPB15**	-0.82	3.03E-01	3.61E-01
**IGKV1-39**	-1.71	3.03E-01	3.61E-01
**C19orf80**	-0.64	3.03E-01	3.61E-01
**AC007249.3**	-1.09	3.03E-01	3.61E-01
**PTPRG-AS1**	-0.39	3.03E-01	3.61E-01
**AC114494.1**	0.98	3.03E-01	3.61E-01
**TECTA**	0.40	3.03E-01	3.61E-01
**BIRC7**	-0.41	3.04E-01	3.61E-01
**KCNS1**	0.39	3.04E-01	3.61E-01
**FER1L6**	0.89	3.04E-01	3.61E-01
**GK**	-0.29	3.04E-01	3.61E-01
**RFTN2**	0.29	3.04E-01	3.61E-01
**FTH1P8**	0.83	3.04E-01	3.61E-01
**RP11-544M22.1**	0.85	3.04E-01	3.61E-01
**AC073850.6**	0.92	3.04E-01	3.61E-01
**RP11-640I15.1**	0.82	3.04E-01	3.62E-01
**CLEC2L**	0.85	3.04E-01	3.62E-01
**SDCCAG8**	0.13	3.04E-01	3.62E-01
**RP11-154H23.4**	-0.82	3.04E-01	3.62E-01
**RP5-1074L1.1**	-0.62	3.04E-01	3.62E-01
**RP11-347I19.8**	-0.54	3.04E-01	3.62E-01

**MAP1A**	-0.38	3.04E-01	3.62E-01
**SYNM**	-0.41	3.04E-01	3.62E-01
**FOXD4**	-0.74	3.04E-01	3.62E-01
**SOCS5**	-0.14	3.04E-01	3.62E-01
**CRB3**	0.20	3.05E-01	3.62E-01
**AC005336.4**	0.90	3.05E-01	3.62E-01
**PRB3**	-0.66	3.05E-01	3.62E-01
**XLOC_009782**	-1.12	3.05E-01	3.62E-01
**RP11-843B15.4**	-0.94	3.05E-01	3.62E-01
**NFKB1**	0.09	3.05E-01	3.62E-01
**SELL**	0.48	3.05E-01	3.63E-01
**RP4-550H1.4**	-0.98	3.05E-01	3.63E-01
**GAPDHP14**	0.86	3.05E-01	3.63E-01
**SRR**	-0.21	3.06E-01	3.63E-01
**RAB14**	-0.12	3.06E-01	3.63E-01
**C2orf48**	-0.56	3.06E-01	3.63E-01
**RNASE7**	-0.36	3.06E-01	3.64E-01
**SUB1**	0.19	3.06E-01	3.64E-01
**LINC01088**	0.47	3.06E-01	3.64E-01
**BIVM**	0.16	3.06E-01	3.64E-01
**RP11-848G14.5**	-0.60	3.06E-01	3.64E-01
**ZSCAN21**	0.14	3.07E-01	3.64E-01
**RPL39P3**	-0.42	3.07E-01	3.64E-01
**ALKBH6**	-0.35	3.07E-01	3.64E-01
**ANKRD26P3**	-1.05	3.07E-01	3.64E-01
**RUSC1-AS1**	-0.45	3.07E-01	3.65E-01
**RP11-539L10.5**	-0.98	3.07E-01	3.65E-01
**RP11-5C23.1**	0.24	3.07E-01	3.65E-01
**TBC1D3**	0.59	3.07E-01	3.65E-01
**RP3-333H23.9**	-0.62	3.07E-01	3.65E-01
**UPK2**	1.01	3.08E-01	3.65E-01
**CLEC2B**	-0.23	3.08E-01	3.65E-01
**RP4-751H13.5**	-0.35	3.08E-01	3.66E-01
**ZNF415P1**	-0.69	3.08E-01	3.66E-01
**LRRIQ3**	0.47	3.08E-01	3.66E-01
**OR1J1**	-1.52	3.09E-01	3.66E-01
**MIR27A**	-0.62	3.09E-01	3.67E-01
**TMEM185AP1**	-0.79	3.09E-01	3.67E-01
**AC097381.1**	-1.05	3.09E-01	3.67E-01
**AC112229.1**	1.17	3.09E-01	3.67E-01
**BCAS2**	0.18	3.09E-01	3.67E-01
**FIGN**	-0.27	3.09E-01	3.67E-01
**CTD-2595P9.4**	1.07	3.09E-01	3.67E-01
**C4orf19**	-0.35	3.09E-01	3.67E-01
**ARHGEF10L**	0.13	3.10E-01	3.67E-01

**SLC29A1**	0.18	3.10E-01	3.67E-01
**RP6-65G23.3**	-0.31	3.10E-01	3.67E-01
**NIPSNAP3A**	-0.21	3.10E-01	3.68E-01
**XLOC_005449**	-0.76	3.10E-01	3.68E-01
**AC084809.2**	-0.62	3.10E-01	3.68E-01
**CTA-286B10.7**	-1.20	3.10E-01	3.68E-01
**PTPRG**	0.31	3.10E-01	3.68E-01
**WI2-1896O14.1**	-0.59	3.10E-01	3.68E-01
**CTC-479C5.12**	-0.44	3.10E-01	3.68E-01
**UPF3BP2**	-0.91	3.10E-01	3.68E-01
**ERVFRD-3**	-1.04	3.11E-01	3.69E-01
**OTOP3**	0.68	3.11E-01	3.69E-01
**PAPPA**	-0.37	3.11E-01	3.69E-01
**RP11-507E23.1**	-0.83	3.11E-01	3.69E-01
**RP11-438E8.2**	-1.24	3.11E-01	3.69E-01
**RFK**	0.21	3.11E-01	3.69E-01
**AF196972.9**	0.68	3.11E-01	3.69E-01
**MTMR11**	-0.19	3.11E-01	3.69E-01
**BTN2A2**	-0.15	3.11E-01	3.69E-01
**IGF2BP2-AS1**	-0.68	3.11E-01	3.69E-01
**PVRL4**	-0.17	3.11E-01	3.69E-01
**LDLRAD3**	-0.14	3.11E-01	3.69E-01
**G28250**	0.73	3.11E-01	3.69E-01
**RP11-641C17.1**	-0.87	3.11E-01	3.69E-01
**RP11-20I23.6**	-0.97	3.11E-01	3.69E-01
**G32695**	0.87	3.11E-01	3.69E-01
**ANKRD20A19P**	-0.78	3.11E-01	3.69E-01
**CHRNA10**	-0.42	3.11E-01	3.69E-01
**BTK**	-0.32	3.12E-01	3.69E-01
**ST8SIA1**	0.24	3.12E-01	3.70E-01
**PSMC2**	0.17	3.12E-01	3.70E-01
**RP3-402G11.25**	0.57	3.12E-01	3.70E-01
**CD86**	0.40	3.12E-01	3.70E-01
**LINC01230**	1.25	3.13E-01	3.71E-01
**CASP3**	0.15	3.13E-01	3.71E-01
**TNR**	-1.15	3.13E-01	3.71E-01
**MIR106B**	-1.36	3.13E-01	3.71E-01
**KRT75**	0.88	3.13E-01	3.71E-01
**TREML2**	-0.87	3.13E-01	3.71E-01
**RP5-1096J16.1**	-1.30	3.13E-01	3.71E-01
**SDAD1P1**	-0.21	3.13E-01	3.71E-01
**RP11-166N17.3**	-0.79	3.13E-01	3.71E-01
**CTC-492K19.4**	0.58	3.14E-01	3.72E-01
**RP11-97O12.7**	0.71	3.14E-01	3.72E-01
**RP11-267M23.4**	-0.40	3.14E-01	3.72E-01

**PRAC1**	5.29	3.14E-01	3.72E-01
**KB-1448A5.1**	-1.06	3.14E-01	3.72E-01
**TCEAL5**	1.11	3.14E-01	3.72E-01
**VGLL1**	1.72	3.14E-01	3.73E-01
**RP11-427J23.1**	-0.82	3.15E-01	3.73E-01
**LDLRAD4**	-0.29	3.15E-01	3.73E-01
**PTGFR**	0.53	3.15E-01	3.73E-01
**RP1-20N2.8**	-0.39	3.15E-01	3.73E-01
**SKA2**	-0.18	3.15E-01	3.73E-01
**AC005387.2**	-0.64	3.15E-01	3.73E-01
**RP11-876N24.3**	-0.37	3.15E-01	3.73E-01
**RP11-365O16.3**	0.54	3.15E-01	3.73E-01
**CRYZ**	-0.27	3.15E-01	3.73E-01
**MBNL1-AS1**	-0.17	3.15E-01	3.73E-01
**G23403**	-0.60	3.15E-01	3.73E-01
**UGT1A1**	-0.52	3.15E-01	3.73E-01
**RP11-356M20.3**	-0.68	3.15E-01	3.74E-01
**SH2D4B**	0.69	3.15E-01	3.74E-01
**CCL26**	0.48	3.16E-01	3.74E-01
**RP11-495P10.1**	-0.72	3.16E-01	3.74E-01
**CTD-2649C14.3**	0.92	3.16E-01	3.74E-01
**G12797**	-0.88	3.16E-01	3.74E-01
**RP11-552F3.10**	-0.52	3.16E-01	3.74E-01
**EGFL8**	0.50	3.16E-01	3.74E-01
**RP11-659E9.2**	-1.00	3.16E-01	3.74E-01
**TOX3**	0.58	3.16E-01	3.74E-01
**NOCT**	0.23	3.16E-01	3.74E-01
**SLAMF6**	0.52	3.16E-01	3.74E-01
**RP11-439A17.9**	0.58	3.16E-01	3.74E-01
**RP11-423O2.5**	-0.53	3.16E-01	3.74E-01
**DGKA**	0.18	3.16E-01	3.74E-01
**ZFP41**	0.16	3.17E-01	3.75E-01
**ZFY**	-1.23	3.17E-01	3.75E-01
**CSTF2T**	0.12	3.17E-01	3.75E-01
**GRAP2**	-0.35	3.17E-01	3.75E-01
**RP11-373D23.3**	-0.45	3.17E-01	3.75E-01
**RP4-621F18.2**	-0.48	3.17E-01	3.75E-01
**GUSBP5**	0.35	3.18E-01	3.76E-01
**TX2P1-UPK3BP1-PMS2P1**	-1.00	3.18E-01	3.76E-01
**EHF**	0.18	3.18E-01	3.76E-01
**RP11-345P4.10**	1.02	3.18E-01	3.76E-01
**KCNMB3**	-0.37	3.18E-01	3.76E-01
**RP5-851M4.1**	-1.28	3.18E-01	3.76E-01
**RP11-121E16.1**	0.79	3.18E-01	3.76E-01
**MPDZ**	0.23	3.18E-01	3.76E-01

**POLD3**	0.10	3.18E-01	3.76E-01
**MYBPC1**	-0.73	3.18E-01	3.76E-01
**CCBL1**	0.21	3.18E-01	3.76E-01
**AP001625.6**	-0.61	3.18E-01	3.76E-01
**SIGLEC10**	-0.42	3.18E-01	3.76E-01
**ZNF57**	0.28	3.18E-01	3.76E-01
**AC009159.1**	-1.65	3.18E-01	3.77E-01
**PNISR**	0.21	3.18E-01	3.77E-01
**RP11-497H16.6**	0.87	3.19E-01	3.77E-01
**TTC32**	-0.23	3.19E-01	3.77E-01
**G4026**	0.98	3.19E-01	3.77E-01
**OSBPL9**	0.10	3.19E-01	3.78E-01
**C8orf22**	-0.87	3.19E-01	3.78E-01
**FIRRE**	-0.89	3.20E-01	3.78E-01
**CTD-3105H18.18**	-1.12	3.20E-01	3.78E-01
**NPIPP1**	-0.32	3.20E-01	3.78E-01
**EXOSC7**	0.12	3.20E-01	3.79E-01
**RP11-1094H24.4**	0.38	3.20E-01	3.79E-01
**AP001877.1**	-0.33	3.21E-01	3.79E-01
**CEACAMP5**	-1.26	3.21E-01	3.79E-01
**SNX21**	-0.14	3.21E-01	3.79E-01
**ZWINT**	0.17	3.21E-01	3.80E-01
**RP11-122K13.12**	-0.38	3.21E-01	3.80E-01
**G25699**	-0.30	3.21E-01	3.80E-01
**MYO6**	-0.16	3.22E-01	3.80E-01
**ELF5**	0.66	3.22E-01	3.80E-01
**DSTN**	0.15	3.22E-01	3.80E-01
**RAD51**	0.17	3.22E-01	3.81E-01
**RP4-734C18.1**	-0.72	3.22E-01	3.81E-01
**XXbac-BPG283O16.9**	-0.22	3.22E-01	3.81E-01
**LINC00384**	-1.26	3.22E-01	3.81E-01
**RP11-214N9.1**	-0.42	3.23E-01	3.81E-01
**GP6**	-0.42	3.23E-01	3.81E-01
**C14orf180**	1.74	3.23E-01	3.81E-01
**RP11-258F22.1**	-0.51	3.23E-01	3.82E-01
**LINC00337**	0.87	3.23E-01	3.82E-01
**SNORD103A**	-1.30	3.23E-01	3.82E-01
**RP11-163G10.4**	-1.15	3.23E-01	3.82E-01
**UTP6**	0.11	3.23E-01	3.82E-01
**CCNE1**	0.24	3.23E-01	3.82E-01
**WWC2**	-0.13	3.23E-01	3.82E-01
**PHF14**	0.15	3.24E-01	3.82E-01
**RBM6**	0.20	3.24E-01	3.82E-01
**KLF7-IT1**	-0.46	3.24E-01	3.82E-01
**RP11-684B2.3**	-1.14	3.24E-01	3.83E-01

**GPNMB**	-0.19	3.24E-01	3.83E-01
**LEF1**	0.21	3.24E-01	3.83E-01
**CTRC**	-1.37	3.24E-01	3.83E-01
**G20695**	0.83	3.24E-01	3.83E-01
**SLC39A5**	-0.68	3.24E-01	3.83E-01
**RP11-284F21.10**	0.74	3.24E-01	3.83E-01
**CTC-453G23.7**	1.06	3.24E-01	3.83E-01
**RP11-68I18.10**	0.48	3.25E-01	3.83E-01
**HPS4**	-0.11	3.25E-01	3.84E-01
**RP11-54G14.1**	-0.75	3.25E-01	3.84E-01
**TRAPPC12**	-0.11	3.25E-01	3.84E-01
**RP3-522P13.2**	1.05	3.26E-01	3.84E-01
**LA16c-395F10.2**	-1.08	3.26E-01	3.84E-01
**SLC34A1**	0.43	3.26E-01	3.85E-01
**ZNF343**	-0.11	3.26E-01	3.85E-01
**RP11-597D13.9**	0.35	3.26E-01	3.85E-01
**RP11-90B9.2**	-0.54	3.26E-01	3.85E-01
**ASB1**	0.11	3.26E-01	3.85E-01
**RP11-649A18.5**	-0.79	3.26E-01	3.85E-01
**RP11-89H19.2**	-0.37	3.26E-01	3.85E-01
**GS1-5L10.1**	-1.32	3.26E-01	3.85E-01
**UPRT**	0.15	3.26E-01	3.85E-01
**NOL8P1**	-1.07	3.26E-01	3.85E-01
**AC011294.3**	0.67	3.26E-01	3.85E-01
**TDP1**	-0.13	3.26E-01	3.85E-01
**DEFB1**	0.27	3.27E-01	3.85E-01
**MMD**	-0.16	3.27E-01	3.85E-01
**AGAP4**	0.50	3.27E-01	3.85E-01
**KDELC2**	-0.17	3.27E-01	3.85E-01
**TPRG1-AS1**	0.57	3.27E-01	3.85E-01
**UQCR11**	-0.15	3.27E-01	3.86E-01
**ATOH8**	0.37	3.27E-01	3.86E-01
**XLOC_014081**	-0.60	3.27E-01	3.86E-01
**AC092580.4**	0.83	3.27E-01	3.86E-01
**RP11-350J20.12**	0.53	3.27E-01	3.86E-01
**G30522**	-0.64	3.27E-01	3.86E-01
**CHKB**	0.50	3.27E-01	3.86E-01
**RAD23B**	-0.15	3.28E-01	3.86E-01
**TIPRL**	-0.16	3.28E-01	3.86E-01
**SLC15A1**	-0.29	3.28E-01	3.87E-01
**EPHA7**	0.70	3.28E-01	3.87E-01
**AGPAT3**	-0.29	3.28E-01	3.87E-01
**LINC00441**	-0.50	3.28E-01	3.87E-01
**DOCK2**	-0.33	3.28E-01	3.87E-01
**AKR7L**	-0.48	3.29E-01	3.87E-01

**MAPKAPK3**	-0.13	3.29E-01	3.87E-01
**RP11-152N13.5**	-0.34	3.29E-01	3.87E-01
**ATP6V0A4**	0.66	3.29E-01	3.87E-01
**RP11-334J6.6**	-1.32	3.29E-01	3.87E-01
**RP11-93B14.9**	0.30	3.29E-01	3.88E-01
**RNF225**	0.23	3.29E-01	3.88E-01
**CKLF**	-0.59	3.29E-01	3.88E-01
**XLOC_007767**	-1.15	3.29E-01	3.88E-01
**TMC2**	0.53	3.29E-01	3.88E-01
**EPM2A**	-0.17	3.30E-01	3.88E-01
**AZI2**	-0.11	3.30E-01	3.89E-01
**CTD-2325M2.1**	0.58	3.30E-01	3.89E-01
**XLOC_004182**	-0.92	3.30E-01	3.89E-01
**BCAS4**	-0.30	3.30E-01	3.89E-01
**RP11-502H18.2**	1.22	3.30E-01	3.89E-01
**CTD-2298J14.2**	-0.62	3.30E-01	3.89E-01
**UBE2D3**	0.11	3.30E-01	3.89E-01
**G31094**	1.04	3.30E-01	3.89E-01
**GS1-166A23.1**	0.68	3.30E-01	3.89E-01
**AC010127.3**	-0.82	3.30E-01	3.89E-01
**MTCH2**	0.13	3.31E-01	3.89E-01
**RP11-134L10.1**	-0.55	3.31E-01	3.90E-01
**GRM3**	-0.84	3.31E-01	3.90E-01
**HHATL**	-0.79	3.31E-01	3.90E-01
**HSBP1L1**	-0.10	3.31E-01	3.90E-01
**DENND6A**	-0.10	3.31E-01	3.90E-01
**NUF2**	-0.23	3.31E-01	3.90E-01
**RP11-556I14.2**	-0.76	3.31E-01	3.90E-01
**SLC25A35**	0.50	3.31E-01	3.90E-01
**RP11-551L14.4**	-0.56	3.31E-01	3.90E-01
**MTND1P36**	-1.12	3.31E-01	3.90E-01
**CTD-3220F14.3**	0.50	3.31E-01	3.90E-01
**RP11-295G24.5**	0.55	3.32E-01	3.91E-01
**AC009403.2**	0.27	3.32E-01	3.91E-01
**RP11-360F5.3**	0.64	3.32E-01	3.91E-01
**COMMD6**	0.16	3.32E-01	3.91E-01
**RP11-259G18.1**	-0.68	3.32E-01	3.91E-01
**IGKV1-5**	-1.04	3.32E-01	3.91E-01
**HSPE1**	-0.18	3.32E-01	3.91E-01
**NPHP3**	0.24	3.32E-01	3.91E-01
**PHKA1**	0.13	3.32E-01	3.91E-01
**PFN3**	0.95	3.32E-01	3.91E-01
**XLOC_006333**	0.78	3.33E-01	3.92E-01
**C11orf63**	-0.19	3.33E-01	3.92E-01
**TNFAIP8**	-0.16	3.33E-01	3.92E-01

**RP11-500G22.4**	-1.04	3.33E-01	3.92E-01
**SMARCD2**	0.12	3.33E-01	3.92E-01
**NDUFB1**	0.17	3.33E-01	3.92E-01
**RP11-231C14.7**	-0.89	3.33E-01	3.92E-01
**HYLS1**	-0.18	3.33E-01	3.92E-01
**XLOC_012338**	-0.53	3.33E-01	3.92E-01
**BARHL1**	-0.98	3.33E-01	3.92E-01
**RP11-38L15.8**	0.51	3.33E-01	3.92E-01
**PCK1**	1.15	3.34E-01	3.93E-01
**XLOC_001913**	-0.91	3.34E-01	3.93E-01
**RP11-1H8.5**	-0.51	3.34E-01	3.93E-01
**RP11-423C15.3**	0.99	3.34E-01	3.93E-01
**CCL11**	4.84	3.34E-01	3.93E-01
**PBX1**	0.14	3.34E-01	3.93E-01
**ANKMY1**	0.16	3.34E-01	3.93E-01
**CLVS1**	-0.33	3.34E-01	3.93E-01
**ATRN**	-0.09	3.34E-01	3.93E-01
**PAQR5**	0.18	3.34E-01	3.93E-01
**SNRPEP4**	0.65	3.34E-01	3.93E-01
**CHST5**	-1.07	3.34E-01	3.93E-01
**FLNC**	-0.28	3.34E-01	3.93E-01
**DHX32**	-0.14	3.34E-01	3.93E-01
**CTC-398G3.1**	-0.52	3.35E-01	3.94E-01
**RP11-111F5.2**	0.77	3.35E-01	3.94E-01
**RPSAP54**	-0.78	3.35E-01	3.94E-01
**AC002511.3**	-0.71	3.35E-01	3.94E-01
**CTD-3126B10.5**	0.79	3.35E-01	3.94E-01
**RP11-462G2.1**	-0.38	3.35E-01	3.94E-01
**ZNF215**	0.50	3.36E-01	3.95E-01
**CTD-2525I3.2**	0.92	3.36E-01	3.95E-01
**AC004791.2**	0.79	3.36E-01	3.95E-01
**CTC-510F12.7**	0.64	3.37E-01	3.96E-01
**NTRK3**	0.37	3.37E-01	3.96E-01
**FAM26E**	-0.59	3.37E-01	3.97E-01
**ANKRD37**	0.24	3.38E-01	3.97E-01
**TEKT1**	-0.70	3.38E-01	3.97E-01
**AP000476.1**	0.80	3.38E-01	3.97E-01
**RAP1GAP2**	0.15	3.38E-01	3.97E-01
**XLOC_013960**	-0.45	3.38E-01	3.97E-01
**HOXC-AS2**	0.45	3.38E-01	3.97E-01
**PTMAP5**	-0.20	3.38E-01	3.97E-01
**RP11-796A5.4**	-1.22	3.38E-01	3.98E-01
**RP3-413H6.2**	-0.79	3.38E-01	3.98E-01
**ZNF232**	-0.18	3.38E-01	3.98E-01
**CTC-575D19.1**	-0.29	3.39E-01	3.98E-01

**AC018804.7**	-0.84	3.39E-01	3.98E-01
**C9orf43**	-0.34	3.39E-01	3.98E-01
**RP11-715J22.3**	0.56	3.39E-01	3.98E-01
**PLCXD3**	0.62	3.39E-01	3.98E-01
**RP4-800G7.2**	-0.37	3.39E-01	3.98E-01
**RP11-59H7.3**	-0.76	3.39E-01	3.99E-01
**RP11-583F2.6**	-1.12	3.39E-01	3.99E-01
**G3899**	-0.72	3.39E-01	3.99E-01
**RBM22P2**	0.80	3.39E-01	3.99E-01
**AC007773.2**	-0.80	3.40E-01	3.99E-01
**RPL14P1**	0.21	3.40E-01	3.99E-01
**FAM65B**	0.29	3.40E-01	3.99E-01
**CTD-2286N8.2**	0.39	3.40E-01	3.99E-01
**RP11-74J13.8**	-0.35	3.40E-01	3.99E-01
**EFNA3**	-0.21	3.40E-01	3.99E-01
**LL09NC01-139C3.1**	-0.73	3.40E-01	4.00E-01
**RP11-16E12.2**	0.69	3.40E-01	4.00E-01
**RP11-651P23.4**	-0.31	3.40E-01	4.00E-01
**AC125232.1**	-0.21	3.40E-01	4.00E-01
**CAGE1**	-0.82	3.40E-01	4.00E-01
**NEMF**	0.10	3.41E-01	4.00E-01
**C17orf97**	0.38	3.41E-01	4.00E-01
**NUCKS1**	-0.13	3.41E-01	4.00E-01
**RNF216P1**	0.15	3.41E-01	4.00E-01
**HMSD**	-0.51	3.41E-01	4.00E-01
**LINC00861**	-0.71	3.41E-01	4.00E-01
**NBPF15**	0.14	3.41E-01	4.00E-01
**UGT1A7**	0.39	3.41E-01	4.00E-01
**RP11-414C23.1**	-0.72	3.41E-01	4.00E-01
**JMJD7-PLA2G4B**	-0.42	3.41E-01	4.00E-01
**RP11-111M22.4**	-0.68	3.41E-01	4.00E-01
**DUX4L26**	5.00	3.41E-01	4.01E-01
**AC046143.3**	-0.45	3.42E-01	4.01E-01
**G32186**	-0.86	3.42E-01	4.01E-01
**ZNRF2P2**	-0.81	3.42E-01	4.01E-01
**AC009501.4**	-0.32	3.42E-01	4.01E-01
**LINC00601**	1.16	3.42E-01	4.02E-01
**SNORA2**	-0.38	3.42E-01	4.02E-01
**RP11-231E6.1**	-0.68	3.42E-01	4.02E-01
**G33271**	0.66	3.42E-01	4.02E-01
**LINC01166**	-1.13	3.42E-01	4.02E-01
**NUDT19**	-0.12	3.43E-01	4.02E-01
**DCUN1D2**	0.13	3.43E-01	4.02E-01
**MAML3**	-0.12	3.43E-01	4.02E-01
**RP11-426A6.5**	0.73	3.43E-01	4.02E-01

**RP11-299L17.3**	1.37	3.43E-01	4.02E-01
**C2orf42**	-0.14	3.43E-01	4.02E-01
**NCF2**	-0.25	3.43E-01	4.02E-01
**RP11-524D16__A.3**	0.54	3.43E-01	4.02E-01
**RP11-478C1.8**	-0.93	3.43E-01	4.02E-01
**CTD-2287O16.1**	-0.30	3.43E-01	4.02E-01
**SLC24A4**	-0.53	3.43E-01	4.02E-01
**UFC1**	0.16	3.43E-01	4.03E-01
**RP11-488C13.5**	-0.54	3.43E-01	4.03E-01
**CFAP99**	-1.02	3.43E-01	4.03E-01
**ELMO2**	-0.11	3.44E-01	4.03E-01
**DLK1**	-1.25	3.44E-01	4.03E-01
**QRICH2**	-0.28	3.44E-01	4.03E-01
**POM121B**	0.54	3.44E-01	4.03E-01
**G34667**	-0.62	3.44E-01	4.03E-01
**PCTP**	0.39	3.44E-01	4.03E-01
**CTC-471J1.10**	-0.83	3.44E-01	4.03E-01
**RP11-351I21.11**	1.07	3.44E-01	4.04E-01
**G16199**	-0.78	3.44E-01	4.04E-01
**RPL13AP5**	0.18	3.44E-01	4.04E-01
**GSN**	-0.21	3.45E-01	4.04E-01
**ANAPC5**	-0.09	3.45E-01	4.04E-01
**RP11-315I20.1**	0.48	3.45E-01	4.04E-01
**RP11-182J1.14**	0.49	3.45E-01	4.04E-01
**RP11-123B3.2**	-0.81	3.45E-01	4.04E-01
**LINC01215**	0.83	3.45E-01	4.04E-01
**SYT17**	-0.28	3.45E-01	4.04E-01
**NSMCE4A**	-0.14	3.45E-01	4.05E-01
**G32329**	-0.95	3.45E-01	4.05E-01
**RP11-82O19.1**	0.48	3.45E-01	4.05E-01
**AC005822.1**	-0.69	3.45E-01	4.05E-01
**THA1P**	0.88	3.45E-01	4.05E-01
**SYTL2**	0.20	3.46E-01	4.05E-01
**RP11-204M4.2**	0.37	3.46E-01	4.05E-01
**EEPD1**	0.17	3.46E-01	4.05E-01
**NR1D2**	-0.37	3.46E-01	4.05E-01
**HNRNPA3P6**	0.42	3.46E-01	4.05E-01
**RP11-85G18.6**	-1.08	3.46E-01	4.05E-01
**AC100830.3**	-0.51	3.46E-01	4.05E-01
**RP11-436M15.3**	-0.81	3.46E-01	4.06E-01
**WI2-87327B8.2**	-0.47	3.46E-01	4.06E-01
**DDX18**	-0.13	3.46E-01	4.06E-01
**G41882**	-0.85	3.46E-01	4.06E-01
**RP11-93O14.2**	-0.62	3.46E-01	4.06E-01
**RP11-4B16.3**	-0.64	3.47E-01	4.06E-01

**LINC00950**	-0.38	3.47E-01	4.06E-01
**REM2**	0.33	3.47E-01	4.06E-01
**MTHFS**	-0.42	3.47E-01	4.06E-01
**CYB5R1**	0.16	3.47E-01	4.06E-01
**G2580**	-0.96	3.47E-01	4.06E-01
**G16821**	0.55	3.47E-01	4.06E-01
**CTD-2008P7.9**	0.29	3.47E-01	4.06E-01
**STARD7-AS1**	0.17	3.48E-01	4.07E-01
**CLCN4**	-0.34	3.48E-01	4.07E-01
**XLOC_005289**	-0.65	3.48E-01	4.07E-01
**SLC35G1**	-0.24	3.48E-01	4.07E-01
**TLK2P1**	-0.50	3.48E-01	4.07E-01
**YWHAZ**	0.15	3.48E-01	4.07E-01
**TMEM130**	0.77	3.48E-01	4.07E-01
**CTB-178M22.2**	-0.48	3.48E-01	4.07E-01
**GPR19**	-0.42	3.48E-01	4.08E-01
**RP11-72M17.1**	-0.41	3.48E-01	4.08E-01
**CCT6P3**	-0.23	3.48E-01	4.08E-01
**TCP1**	0.12	3.49E-01	4.08E-01
**RP11-269F19.2**	-1.18	3.49E-01	4.08E-01
**RASSF8**	-0.27	3.49E-01	4.08E-01
**EEF1DP1**	-0.41	3.49E-01	4.08E-01
**BICD2**	-0.15	3.49E-01	4.08E-01
**G36323**	0.82	3.49E-01	4.08E-01
**CHRDL1**	-0.49	3.49E-01	4.08E-01
**CCNL2**	0.29	3.49E-01	4.08E-01
**RNU6-96P**	-1.28	3.49E-01	4.08E-01
**RP11-463I20.2**	-0.98	3.49E-01	4.08E-01
**RNU2-5P**	-0.86	3.49E-01	4.09E-01
**RP11-335L23.5**	0.73	3.49E-01	4.09E-01
**GBP7**	-0.93	3.49E-01	4.09E-01
**XLOC_005332**	-0.89	3.50E-01	4.09E-01
**ZBTB8OS**	0.12	3.50E-01	4.09E-01
**ATP8B5P**	-0.60	3.50E-01	4.09E-01
**RP11-206L10.2**	0.24	3.50E-01	4.09E-01
**CH507-9B2.8**	0.82	3.50E-01	4.09E-01
**WDR44**	-0.12	3.50E-01	4.09E-01
**AC096669.3**	0.71	3.50E-01	4.10E-01
**HTT-AS**	-1.07	3.50E-01	4.10E-01
**IPPK**	-0.13	3.51E-01	4.10E-01
**RN7SL262P**	-0.72	3.51E-01	4.10E-01
**DUT**	0.14	3.51E-01	4.10E-01
**SACS-AS1**	-0.81	3.51E-01	4.11E-01
**CEP290**	-0.17	3.51E-01	4.11E-01
**RP11-20G13.2**	-1.14	3.51E-01	4.11E-01

**C6orf62**	0.10	3.51E-01	4.11E-01
**EIF4BP3**	-0.22	3.51E-01	4.11E-01
**TTC34**	0.41	3.51E-01	4.11E-01
**RP11-249C24.10**	-1.16	3.51E-01	4.11E-01
**CTD-2124B8.2**	-0.22	3.51E-01	4.11E-01
**FAM13C**	0.17	3.52E-01	4.11E-01
**RBM4B**	0.14	3.52E-01	4.11E-01
**RP11-17M16.2**	0.28	3.52E-01	4.11E-01
**IGHD**	-1.43	3.52E-01	4.11E-01
**ADORA2A**	-0.63	3.52E-01	4.11E-01
**XLOC_014378**	0.74	3.52E-01	4.11E-01
**RP11-522I20.3**	0.28	3.52E-01	4.12E-01
**HSPH1**	-0.19	3.52E-01	4.12E-01
**G10551**	-0.91	3.52E-01	4.12E-01
**USP46-AS1**	-0.21	3.52E-01	4.12E-01
**COL24A1**	-0.34	3.52E-01	4.12E-01
**ELP3**	0.08	3.52E-01	4.12E-01
**IMP3**	-0.13	3.52E-01	4.12E-01
**RP11-449D8.1**	0.69	3.52E-01	4.12E-01
**CENPQ**	-0.21	3.53E-01	4.12E-01
**MT1P1**	-0.93	3.53E-01	4.12E-01
**RP11-70D24.4**	-0.93	3.53E-01	4.12E-01
**NAF1**	-0.15	3.53E-01	4.12E-01
**HLA-V**	0.60	3.53E-01	4.12E-01
**AP001065.15**	1.04	3.53E-01	4.12E-01
**HMGA1P4**	0.59	3.53E-01	4.12E-01
**RP11-187C18.2**	0.67	3.53E-01	4.12E-01
**CTD-3105H18.14**	-0.79	3.53E-01	4.12E-01
**TMEM108**	-0.29	3.53E-01	4.12E-01
**LINC00222**	-0.70	3.53E-01	4.12E-01
**OR56B4**	-1.43	3.53E-01	4.12E-01
**ATG7**	-0.08	3.53E-01	4.12E-01
**MT-TF**	0.62	3.53E-01	4.13E-01
**SPHKAP**	1.01	3.53E-01	4.13E-01
**B4GALNT3**	0.18	3.53E-01	4.13E-01
**RP11-1391J7.1**	-0.49	3.54E-01	4.13E-01
**RP4-597N16.4**	0.53	3.54E-01	4.13E-01
**RP11-351J23.2**	0.99	3.54E-01	4.13E-01
**ENPP7P2**	-1.04	3.54E-01	4.13E-01
**FAM118A**	0.32	3.54E-01	4.13E-01
**RP11-312B8.1**	0.40	3.54E-01	4.13E-01
**IL21R-AS1**	-1.09	3.54E-01	4.13E-01
**CTD-2528A14.5**	1.16	3.54E-01	4.13E-01
**POLB**	-0.17	3.54E-01	4.13E-01
**MROH8**	-0.22	3.54E-01	4.13E-01

**AC018755.1**	-0.67	3.54E-01	4.13E-01
**NPPA-AS1**	-0.80	3.54E-01	4.13E-01
**RP11-339B21.13**	-0.42	3.54E-01	4.14E-01
**TRIM50**	0.89	3.54E-01	4.14E-01
**LINC00862**	-1.17	3.55E-01	4.14E-01
**IDSP1**	-0.78	3.55E-01	4.14E-01
**ARHGEF4**	0.21	3.55E-01	4.14E-01
**AC107079.1**	-0.75	3.55E-01	4.14E-01
**RP3-418A9.3**	-0.88	3.55E-01	4.14E-01
**UNGP3**	-0.41	3.55E-01	4.14E-01
**RP11-706O15.1**	-0.16	3.55E-01	4.14E-01
**DUSP5**	-0.15	3.55E-01	4.14E-01
**SCTR**	-0.42	3.55E-01	4.14E-01
**CTD-3065J16.6**	0.43	3.55E-01	4.14E-01
**AMT**	-0.35	3.55E-01	4.15E-01
**RP11-31F15.2**	-0.41	3.55E-01	4.15E-01
**ORC6**	-0.27	3.56E-01	4.15E-01
**RP11-446H18.1**	-1.04	3.56E-01	4.15E-01
**G3928**	-1.10	3.56E-01	4.16E-01
**RP11-544M22.13**	0.68	3.56E-01	4.16E-01
**ESRRAP2**	-0.76	3.56E-01	4.16E-01
**PCNA**	0.18	3.57E-01	4.16E-01
**CHST6**	-0.34	3.57E-01	4.16E-01
**OLIG1**	1.37	3.57E-01	4.16E-01
**GINS2**	-0.20	3.57E-01	4.16E-01
**RP11-529G21.2**	0.76	3.57E-01	4.16E-01
**WDR4**	0.20	3.57E-01	4.16E-01
**RP3-467N11.1**	-0.28	3.57E-01	4.16E-01
**BTBD1**	-0.13	3.57E-01	4.16E-01
**ZSCAN29**	-0.08	3.57E-01	4.16E-01
**ACTR6**	-0.15	3.57E-01	4.16E-01
**RP11-69E11.4**	0.41	3.57E-01	4.17E-01
**CLNS1A**	-0.11	3.57E-01	4.17E-01
**RP11-264B17.4**	0.70	3.58E-01	4.17E-01
**SLC41A1**	0.25	3.58E-01	4.17E-01
**FPR3**	0.41	3.58E-01	4.17E-01
**RP11-187C18.4**	0.81	3.58E-01	4.17E-01
**CNTFR**	-0.36	3.58E-01	4.17E-01
**PDPK1**	0.08	3.58E-01	4.17E-01
**LINC00239**	-0.43	3.58E-01	4.17E-01
**RP11-425A6.5**	1.08	3.58E-01	4.17E-01
**KCNE2**	1.09	3.58E-01	4.17E-01
**RP11-697K23.3**	-1.26	3.58E-01	4.18E-01
**SEMA3C**	-0.26	3.59E-01	4.18E-01
**TINCR**	-0.16	3.59E-01	4.18E-01

**XLOC_006303**	0.85	3.59E-01	4.18E-01
**RP11-413B19.2**	-1.05	3.59E-01	4.18E-01
**RDH14**	0.12	3.59E-01	4.18E-01
**CTD-2013N17.7**	-1.07	3.59E-01	4.18E-01
**RP11-115L11.1**	-0.94	3.59E-01	4.19E-01
**RP11-982M15.6**	-0.79	3.59E-01	4.19E-01
**RN7SL499P**	1.07	3.59E-01	4.19E-01
**CTA-126B4.7**	-1.00	3.59E-01	4.19E-01
**FKBP1B**	0.23	3.60E-01	4.19E-01
**AP3M2**	-0.15	3.60E-01	4.19E-01
**SYBU**	-0.16	3.60E-01	4.20E-01
**LIPT1**	-0.19	3.60E-01	4.20E-01
**CCDC77**	0.14	3.60E-01	4.20E-01
**RP11-284N8.3**	-0.42	3.60E-01	4.20E-01
**TSNAX**	0.18	3.61E-01	4.20E-01
**DAPK1-IT1**	-0.83	3.61E-01	4.20E-01
**CCDC50**	-0.16	3.61E-01	4.20E-01
**RP11-656D10.6**	0.60	3.61E-01	4.20E-01
**BEX1**	0.57	3.61E-01	4.20E-01
**G43235**	-0.43	3.61E-01	4.20E-01
**RP11-91J19.3**	-0.58	3.61E-01	4.20E-01
**PYY**	0.95	3.61E-01	4.21E-01
**RPL12P10**	0.99	3.61E-01	4.21E-01
**SULT1B1**	-0.50	3.61E-01	4.21E-01
**RP11-76E17.3**	0.97	3.61E-01	4.21E-01
**LA16c-380A1.2**	-0.46	3.62E-01	4.21E-01
**AC003956.1**	-0.70	3.62E-01	4.21E-01
**AC105049.1**	-0.98	3.62E-01	4.21E-01
**RB1**	0.12	3.62E-01	4.21E-01
**G14029**	0.80	3.62E-01	4.21E-01
**FBF1**	-0.21	3.62E-01	4.21E-01
**RNF103-CHMP3**	-0.56	3.62E-01	4.21E-01
**PLEKHA7**	0.18	3.62E-01	4.22E-01
**RP11-506M12.1**	-0.67	3.62E-01	4.22E-01
**ERI1**	-0.12	3.62E-01	4.22E-01
**SYS1**	0.10	3.62E-01	4.22E-01
**RP11-511B23.3**	1.28	3.62E-01	4.22E-01
**CACNB1**	0.25	3.62E-01	4.22E-01
**RP1-47M23.3**	-0.72	3.62E-01	4.22E-01
**PROX1**	-0.36	3.62E-01	4.22E-01
**ST6GALNAC4P1**	-0.56	3.62E-01	4.22E-01
**AC005785.5**	-0.40	3.63E-01	4.22E-01
**UBE2K**	-0.14	3.63E-01	4.22E-01
**ATXN2**	0.15	3.63E-01	4.22E-01
**MTRF1**	-0.14	3.63E-01	4.22E-01

**SPTLC1**	-0.12	3.63E-01	4.23E-01
**SLC25A34**	-0.50	3.63E-01	4.23E-01
**RNU7-12P**	-1.42	3.63E-01	4.23E-01
**STAM**	-0.12	3.63E-01	4.23E-01
**CTC-297N7.9**	0.57	3.64E-01	4.23E-01
**RP11-169F17.1**	4.78	3.64E-01	4.23E-01
**RP11-178L8.7**	-0.64	3.64E-01	4.23E-01
**RP3-337H4.10**	-0.66	3.64E-01	4.23E-01
**RP5-1142A6.9**	-0.40	3.64E-01	4.23E-01
**RP11-621L6.2**	-0.82	3.64E-01	4.23E-01
**UBE2CP2**	-0.82	3.64E-01	4.23E-01
**IL12A**	0.96	3.64E-01	4.23E-01
**ANKRD26**	0.16	3.64E-01	4.23E-01
**RP11-511B23.2**	1.02	3.64E-01	4.23E-01
**RP11-613D13.4**	-0.43	3.64E-01	4.23E-01
**HPSE2**	0.52	3.64E-01	4.23E-01
**HSPA1L**	0.18	3.64E-01	4.23E-01
**CHL1-AS2**	-1.04	3.64E-01	4.23E-01
**ZFP69B**	0.20	3.64E-01	4.23E-01
**STK38L**	-0.10	3.64E-01	4.23E-01
**RP11-80H5.2**	-1.20	3.64E-01	4.24E-01
**BAIAP2L1**	-0.15	3.64E-01	4.24E-01
**3-Sep**	-0.23	3.65E-01	4.24E-01
**ERLEC1**	0.13	3.65E-01	4.24E-01
**RP5-973N23.4**	-0.89	3.65E-01	4.24E-01
**RP11-248J23.5**	1.26	3.65E-01	4.24E-01
**ATP5F1**	0.14	3.65E-01	4.24E-01
**CTC-558O2.1**	-0.58	3.65E-01	4.24E-01
**CTD-2282P23.2**	-0.62	3.65E-01	4.24E-01
**KIAA1324L**	-0.24	3.65E-01	4.24E-01
**NRDE2**	0.10	3.65E-01	4.25E-01
**ALDH1L1**	0.57	3.65E-01	4.25E-01
**C17orf49**	0.52	3.65E-01	4.25E-01
**NPR2**	-0.21	3.65E-01	4.25E-01
**IGLV1-50**	-1.29	3.66E-01	4.25E-01
**RP11-554D14.8**	-0.81	3.66E-01	4.25E-01
**IL17REL**	-0.79	3.66E-01	4.25E-01
**RPSAP15**	-0.28	3.66E-01	4.25E-01
**LAMTOR4**	-0.14	3.66E-01	4.25E-01
**MICU3**	-0.31	3.66E-01	4.26E-01
**RP3-406P24.3**	-0.92	3.66E-01	4.26E-01
**CTC-338M12.1**	0.64	3.66E-01	4.26E-01
**RP11-61K12.2**	-1.02	3.66E-01	4.26E-01
**MELK**	-0.20	3.66E-01	4.26E-01
**EPHA5-AS1**	-1.06	3.67E-01	4.26E-01

**CTD-2008E3.1**	-0.35	3.67E-01	4.26E-01
**PRELID1P4**	0.54	3.67E-01	4.27E-01
**SNX4**	0.14	3.67E-01	4.27E-01
**CHRNE**	-0.34	3.67E-01	4.27E-01
**ACADM**	-0.35	3.68E-01	4.27E-01
**G27544**	-0.65	3.68E-01	4.27E-01
**NFYA**	0.16	3.68E-01	4.27E-01
**KB-1836B5.4**	-0.86	3.68E-01	4.27E-01
**RP11-21L23.3**	0.36	3.68E-01	4.27E-01
**PENK**	0.84	3.68E-01	4.28E-01
**XGY1**	-0.58	3.68E-01	4.28E-01
**RP5-991G20.1**	0.24	3.69E-01	4.28E-01
**RP13-131K19.6**	0.81	3.69E-01	4.28E-01
**TXLNB**	-0.32	3.69E-01	4.28E-01
**RNU6-834P**	-0.77	3.69E-01	4.28E-01
**NAT8L**	0.28	3.69E-01	4.28E-01
**SRY**	-0.34	3.69E-01	4.28E-01
**SOSTDC1**	0.37	3.69E-01	4.29E-01
**PKD1L1**	-0.30	3.69E-01	4.29E-01
**HFM1**	-0.47	3.69E-01	4.29E-01
**CTC-487M23.5**	-0.30	3.70E-01	4.29E-01
**CDO1**	-0.49	3.70E-01	4.29E-01
**RP11-532F6.4**	-0.67	3.70E-01	4.29E-01
**CTC-479C5.10**	0.65	3.70E-01	4.30E-01
**RNU6-945P**	-0.92	3.70E-01	4.30E-01
**RP11-26L20.4**	-0.73	3.70E-01	4.30E-01
**CTD-2062F14.2**	1.05	3.70E-01	4.30E-01
**CTC-537E7.2**	1.02	3.70E-01	4.30E-01
**RP1-102D24.5**	-0.81	3.71E-01	4.30E-01
**SCRN3**	-0.15	3.71E-01	4.30E-01
**NFYB**	-0.14	3.71E-01	4.31E-01
**TRIM31**	0.33	3.71E-01	4.31E-01
**LA16c-306A4.2**	-0.64	3.71E-01	4.31E-01
**PGBD3**	-0.68	3.71E-01	4.31E-01
**RBFOX2**	0.09	3.72E-01	4.31E-01
**AC138783.12**	0.64	3.72E-01	4.31E-01
**RP11-667F14.1**	-0.37	3.72E-01	4.31E-01
**RP11-285E9.6**	-0.46	3.72E-01	4.31E-01
**AC006129.1**	0.75	3.72E-01	4.32E-01
**AC007792.1**	-1.06	3.72E-01	4.32E-01
**CTD-2036P10.6**	-0.49	3.73E-01	4.32E-01
**RNU6-59P**	-1.23	3.73E-01	4.32E-01
**FITM1**	-0.47	3.73E-01	4.32E-01
**RPRD2**	-0.10	3.73E-01	4.32E-01
**AC098828.2**	-0.84	3.73E-01	4.32E-01

**CAPS**	-0.28	3.73E-01	4.32E-01
**ADD3-AS1**	-0.33	3.73E-01	4.33E-01
**PAQR7**	0.18	3.73E-01	4.33E-01
**TMEM30B**	0.10	3.73E-01	4.33E-01
**SLC7A2**	0.24	3.73E-01	4.33E-01
**RP5-1047A19.4**	-1.25	3.73E-01	4.33E-01
**AC009120.3**	0.36	3.73E-01	4.33E-01
**TMEM243**	-0.12	3.73E-01	4.33E-01
**RP11-727F15.13**	-0.70	3.74E-01	4.33E-01
**RP11-572O6.1**	-0.70	3.74E-01	4.33E-01
**RP11-9L18.2**	-0.69	3.74E-01	4.33E-01
**SRPK1**	-0.21	3.74E-01	4.33E-01
**CUX2**	-0.79	3.74E-01	4.33E-01
**RP11-121A14.2**	-0.65	3.74E-01	4.33E-01
**TMEM125**	0.24	3.74E-01	4.33E-01
**XLOC_012740**	0.34	3.74E-01	4.33E-01
**RP11-532F6.3**	0.49	3.74E-01	4.33E-01
**G31873**	0.52	3.74E-01	4.33E-01
**AC005546.2**	-0.65	3.74E-01	4.34E-01
**POLR2J3**	0.34	3.74E-01	4.34E-01
**PTGER2**	0.43	3.74E-01	4.34E-01
**LINC01431**	0.35	3.75E-01	4.35E-01
**TRIM5**	0.16	3.75E-01	4.35E-01
**XLOC_001477**	0.68	3.75E-01	4.35E-01
**HOMER1**	0.16	3.75E-01	4.35E-01
**OXGR1**	0.81	3.75E-01	4.35E-01
**TEX15**	1.08	3.76E-01	4.35E-01
**DRD4**	0.46	3.76E-01	4.35E-01
**XLOC_009765**	-0.51	3.76E-01	4.35E-01
**FAM161A**	0.19	3.76E-01	4.35E-01
**PELI3**	0.16	3.76E-01	4.36E-01
**FAM200A**	-0.12	3.76E-01	4.36E-01
**G26553**	0.79	3.76E-01	4.36E-01
**RP11-21K12.3**	-0.73	3.76E-01	4.36E-01
**MIR659**	-1.01	3.76E-01	4.36E-01
**FAM205BP**	-1.04	3.76E-01	4.36E-01
**RP11-472F14.4**	-1.37	3.77E-01	4.36E-01
**C9orf85**	0.11	3.77E-01	4.36E-01
**RP11-561I11.3**	-1.20	3.77E-01	4.36E-01
**RP5-965G21.3**	-0.55	3.77E-01	4.36E-01
**BAIAP2**	0.13	3.77E-01	4.36E-01
**MRPL57P3**	-0.93	3.77E-01	4.37E-01
**CD81-AS1**	-0.52	3.77E-01	4.37E-01
**CAPN12**	0.37	3.77E-01	4.37E-01
**RP4-781K5.5**	-0.80	3.78E-01	4.37E-01

**PCDHA8**	-1.08	3.78E-01	4.37E-01
**TRPV3**	-0.17	3.78E-01	4.37E-01
**BANK1**	0.24	3.78E-01	4.37E-01
**LINC01485**	-0.90	3.78E-01	4.38E-01
**ACSF2**	-0.15	3.78E-01	4.38E-01
**CRTAM**	0.58	3.78E-01	4.38E-01
**RP11-26P13.2**	-0.97	3.78E-01	4.38E-01
**CGB7**	0.50	3.78E-01	4.38E-01
**RP11-333E13.4**	-1.09	3.79E-01	4.38E-01
**RP11-490K7.4**	-0.56	3.79E-01	4.38E-01
**KDM1A**	0.07	3.79E-01	4.38E-01
**AC002550.5**	0.98	3.79E-01	4.38E-01
**ARHGAP5-AS1**	0.23	3.79E-01	4.38E-01
**RP11-174O3.6**	-4.22	3.79E-01	4.38E-01
**RP11-17M24.1**	-0.89	3.79E-01	4.39E-01
**FA2H**	0.59	3.79E-01	4.39E-01
**CXorf58**	-0.72	3.79E-01	4.39E-01
**FAM57B**	0.76	3.79E-01	4.39E-01
**HECW2**	0.20	3.80E-01	4.39E-01
**NDUFB2**	-0.12	3.80E-01	4.39E-01
**JAKMIP3**	0.35	3.80E-01	4.39E-01
**LINC01011**	-0.32	3.80E-01	4.39E-01
**RP11-378J18.9**	-0.64	3.80E-01	4.40E-01
**RP11-847H18.3**	-0.58	3.80E-01	4.40E-01
**MND1**	-0.23	3.81E-01	4.41E-01
**NDUFAF7**	-0.12	3.81E-01	4.41E-01
**RP11-747H12.3**	-0.96	3.81E-01	4.41E-01
**AC067945.3**	-0.61	3.81E-01	4.41E-01
**FAR2P2**	0.63	3.81E-01	4.41E-01
**STK33**	-0.57	3.82E-01	4.41E-01
**IGHV3-30**	1.69	3.82E-01	4.42E-01
**RP11-661C3.2**	0.72	3.82E-01	4.42E-01
**GPAT4**	0.10	3.82E-01	4.42E-01
**HIP1**	0.17	3.82E-01	4.42E-01
**COPB1**	-0.12	3.82E-01	4.42E-01
**CYP3A7**	-0.72	3.82E-01	4.42E-01
**TRIM66**	-0.24	3.83E-01	4.43E-01
**OAT**	-0.17	3.83E-01	4.43E-01
**SOWAHA**	0.41	3.84E-01	4.44E-01
**CDC25C**	0.26	3.84E-01	4.44E-01
**CTA-246H3.8**	-0.73	3.84E-01	4.44E-01
**CTD-2576N18.1**	-0.89	3.84E-01	4.44E-01
**CLUL1**	-0.48	3.84E-01	4.44E-01
**XLOC_008522**	0.45	3.84E-01	4.44E-01
**UGT2B11**	1.64	3.84E-01	4.44E-01

**CTD-2303H24.2**	-0.91	3.85E-01	4.44E-01
**RNU6-418P**	-0.94	3.85E-01	4.45E-01
**CTD-3099C6.5**	-0.77	3.85E-01	4.45E-01
**TBCEL**	-0.10	3.85E-01	4.45E-01
**SLC5A8**	1.07	3.85E-01	4.45E-01
**RP11-640N20.4**	0.45	3.85E-01	4.45E-01
**COX7B**	-0.16	3.85E-01	4.45E-01
**RP4-563E14.1**	-0.28	3.85E-01	4.45E-01
**RP11-2C24.3**	0.44	3.85E-01	4.45E-01
**GNRH2**	-0.57	3.85E-01	4.45E-01
**RP11-181C3.1**	-0.47	3.85E-01	4.45E-01
**BEST4**	-0.46	3.86E-01	4.45E-01
**L1CAM**	-0.23	3.86E-01	4.45E-01
**PSPN**	-0.21	3.86E-01	4.45E-01
**RP11-499P20.2**	-0.28	3.86E-01	4.46E-01
**CTD-2531D15.4**	-1.23	3.86E-01	4.46E-01
**RP11-307C12.13**	-0.55	3.86E-01	4.46E-01
**AC008592.4**	0.83	3.86E-01	4.46E-01
**CTC1**	-0.16	3.86E-01	4.46E-01
**LINC00184**	0.69	3.86E-01	4.46E-01
**RP11-96K19.2**	-0.26	3.86E-01	4.46E-01
**AC006273.4**	0.75	3.87E-01	4.46E-01
**LYPLAL1-AS1**	-0.72	3.87E-01	4.47E-01
**RP11-496H1.1**	-0.40	3.87E-01	4.47E-01
**C9orf24**	0.47	3.87E-01	4.47E-01
**XLOC_007806**	-0.79	3.87E-01	4.47E-01
**DAP3P2**	0.59	3.87E-01	4.47E-01
**PCDHB17P**	0.88	3.87E-01	4.47E-01
**G27940**	-0.86	3.87E-01	4.47E-01
**XLOC_003985**	-0.87	3.87E-01	4.47E-01
**RP11-474P2.5**	-0.58	3.87E-01	4.47E-01
**DIP2A**	0.11	3.87E-01	4.47E-01
**LINC01554**	0.39	3.87E-01	4.47E-01
**C1QTNF9**	0.92	3.88E-01	4.48E-01
**IGLV1-47**	1.53	3.88E-01	4.48E-01
**RNU6-430P**	-0.98	3.88E-01	4.48E-01
**RPS23**	-0.21	3.88E-01	4.48E-01
**GRPR**	0.65	3.88E-01	4.48E-01
**IGLC2**	1.14	3.88E-01	4.48E-01
**NIPA2**	-0.11	3.88E-01	4.48E-01
**HNRNPA1P3**	-1.22	3.88E-01	4.48E-01
**LA16c-358B7.3**	0.31	3.88E-01	4.48E-01
**BCL2L10**	0.24	3.88E-01	4.48E-01
**RPGR**	-0.14	3.88E-01	4.48E-01
**KIAA0101**	-0.24	3.88E-01	4.48E-01

**LRRC16B**	-0.30	3.89E-01	4.49E-01
**AC007743.1**	0.43	3.89E-01	4.49E-01
**LINC01564**	0.77	3.89E-01	4.49E-01
**AKAP12**	-0.28	3.89E-01	4.49E-01
**RP5-864K19.4**	0.33	3.90E-01	4.49E-01
**PLRG1**	0.10	3.90E-01	4.50E-01
**OLFM4**	0.85	3.90E-01	4.50E-01
**RND2**	-0.25	3.90E-01	4.50E-01
**DIXDC1**	-0.16	3.90E-01	4.50E-01
**LINC01301**	0.39	3.91E-01	4.51E-01
**G5796**	0.54	3.91E-01	4.51E-01
**RP11-325N19.3**	0.93	3.91E-01	4.51E-01
**MOV10L1**	0.55	3.91E-01	4.51E-01
**TAF8**	-0.07	3.91E-01	4.51E-01
**NIFK**	-0.13	3.91E-01	4.51E-01
**TBC1D15**	-0.12	3.91E-01	4.51E-01
**DRD2**	0.62	3.91E-01	4.51E-01
**FTLP14**	-0.33	3.91E-01	4.51E-01
**GPR27**	0.26	3.91E-01	4.51E-01
**DGCR5**	0.27	3.91E-01	4.51E-01
**AC002553.4**	-0.94	3.91E-01	4.51E-01
**RPL22L1**	0.20	3.92E-01	4.52E-01
**GPC1**	0.14	3.92E-01	4.52E-01
**PYY2**	-0.84	3.92E-01	4.52E-01
**DSG2**	0.24	3.92E-01	4.52E-01
**SDHB**	-0.12	3.92E-01	4.52E-01
**PQLC3**	-0.16	3.92E-01	4.52E-01
**RP11-454H19.2**	0.85	3.93E-01	4.53E-01
**STRADB**	-0.15	3.93E-01	4.53E-01
**CLN5**	-0.13	3.93E-01	4.53E-01
**CMA1**	-0.46	3.94E-01	4.54E-01
**RP11-422N16.3**	-0.67	3.94E-01	4.54E-01
**HOXC13-AS**	-0.23	3.94E-01	4.54E-01
**RP13-977J11.2**	0.37	3.94E-01	4.54E-01
**RNU6-123P**	-0.66	3.94E-01	4.54E-01
**RP11-353N14.5**	-0.94	3.94E-01	4.54E-01
**HIATL2**	-0.14	3.94E-01	4.54E-01
**RP11-45A16.4**	-0.95	3.94E-01	4.54E-01
**ANKRD20A11P**	0.79	3.94E-01	4.54E-01
**CACNA2D3**	0.38	3.94E-01	4.54E-01
**SP5**	0.32	3.94E-01	4.54E-01
**ATG5**	0.10	3.94E-01	4.54E-01
**HNRNPDL**	-0.15	3.94E-01	4.54E-01
**DROSHA**	0.10	3.94E-01	4.54E-01
**CCT6B**	0.20	3.94E-01	4.54E-01

**AC138969.4**	-0.59	3.95E-01	4.55E-01
**LINC00643**	-1.47	3.95E-01	4.55E-01
**DNAH8**	-0.43	3.95E-01	4.55E-01
**RP11-347C12.12**	-0.60	3.95E-01	4.55E-01
**TRAM1L1**	-0.23	3.95E-01	4.55E-01
**CTD-2201E18.5**	0.93	3.95E-01	4.55E-01
**TTC41P**	-0.39	3.95E-01	4.55E-01
**IGBP1-AS1**	-0.62	3.95E-01	4.55E-01
**RP11-266L9.4**	0.24	3.96E-01	4.56E-01
**TMBIM4**	-0.19	3.96E-01	4.56E-01
**ADGRB1**	-0.39	3.96E-01	4.56E-01
**RGS7**	0.78	3.96E-01	4.56E-01
**CORT**	0.52	3.96E-01	4.56E-01
**RP11-1060J15.9**	0.47	3.96E-01	4.56E-01
**ZFP69**	0.11	3.96E-01	4.56E-01
**TMCC1**	-0.07	3.97E-01	4.57E-01
**CTC-523E23.4**	-0.33	3.97E-01	4.57E-01
**DAZAP2P1**	-0.50	3.97E-01	4.57E-01
**LLNLR-268E12.1**	-0.81	3.97E-01	4.57E-01
**CASP2**	0.11	3.97E-01	4.57E-01
**LINC00165**	0.80	3.97E-01	4.57E-01
**G4440**	-0.39	3.97E-01	4.57E-01
**PPAN**	-0.21	3.97E-01	4.57E-01
**RP1-92O14.6**	-0.27	3.97E-01	4.57E-01
**RP11-254F19.2**	0.75	3.98E-01	4.58E-01
**LTK**	-0.31	3.98E-01	4.58E-01
**SAMD15**	-0.26	3.98E-01	4.58E-01
**C20orf197**	-0.42	3.98E-01	4.58E-01
**AC019048.1**	0.52	3.98E-01	4.58E-01
**RP11-879F14.3**	-0.98	3.98E-01	4.58E-01
**LGALS4**	-0.44	3.98E-01	4.58E-01
**LINC00638**	0.43	3.99E-01	4.59E-01
**KRT33B**	1.84	3.99E-01	4.59E-01
**RP11-432I13.5**	0.96	3.99E-01	4.59E-01
**HPCAL4**	-0.89	3.99E-01	4.59E-01
**RP11-71L14.4**	-0.98	3.99E-01	4.59E-01
**RP11-191G24.1**	-0.38	3.99E-01	4.59E-01
**AC018766.4**	-0.67	3.99E-01	4.59E-01
**TMEM132C**	-0.44	3.99E-01	4.59E-01
**CLEC9A**	0.52	3.99E-01	4.59E-01
**HSPB2**	0.80	3.99E-01	4.59E-01
**SIGLEC14**	0.82	3.99E-01	4.59E-01
**BEST3**	-0.48	3.99E-01	4.60E-01
**RP3-523C21.2**	0.65	3.99E-01	4.60E-01
**UPP2**	0.65	4.00E-01	4.60E-01

**MBOAT4**	0.72	4.00E-01	4.60E-01
**MAN1A1**	0.26	4.00E-01	4.60E-01
**CCDC53**	-0.10	4.00E-01	4.60E-01
**BBOF1**	0.17	4.00E-01	4.60E-01
**AP001059.6**	-0.77	4.00E-01	4.60E-01
**SLC46A3**	-0.33	4.00E-01	4.60E-01
**RNU6-484P**	-0.99	4.00E-01	4.60E-01
**G12463**	-0.92	4.01E-01	4.61E-01
**NEURL1**	0.31	4.01E-01	4.61E-01
**RP11-278J6.4**	-0.89	4.01E-01	4.61E-01
**SLIT3**	-0.30	4.01E-01	4.61E-01
**GATA5**	1.09	4.01E-01	4.61E-01
**MAGEE2**	0.49	4.01E-01	4.62E-01
**RP3-523E19.2**	-0.53	4.02E-01	4.62E-01
**RP5-884M6.1**	3.58	4.02E-01	4.62E-01
**RNU6-415P**	-0.33	4.02E-01	4.62E-01
**AC000095.11**	-0.92	4.02E-01	4.62E-01
**U47924.6**	-0.33	4.02E-01	4.62E-01
**LHFPL1**	0.59	4.02E-01	4.62E-01
**RP11-55L3.1**	0.77	4.03E-01	4.63E-01
**MRPS10**	0.12	4.03E-01	4.63E-01
**MXI1**	-0.12	4.03E-01	4.63E-01
**TRMT12**	0.12	4.03E-01	4.63E-01
**GAB3**	-0.25	4.03E-01	4.63E-01
**RP3-323P24.3**	-0.46	4.03E-01	4.63E-01
**LSM12P1**	-0.51	4.03E-01	4.64E-01
**RP11-452L6.7**	-0.42	4.03E-01	4.64E-01
**OSCP1**	0.12	4.04E-01	4.64E-01
**GOLGA8A**	-0.39	4.04E-01	4.64E-01
**RP11-261P9.4**	-0.53	4.04E-01	4.64E-01
**RP11-571M6.17**	-0.25	4.04E-01	4.64E-01
**RN7SL349P**	-0.82	4.04E-01	4.64E-01
**RPL24P4**	-0.37	4.04E-01	4.64E-01
**ZNF79**	-0.10	4.04E-01	4.64E-01
**RSPH9**	-0.21	4.04E-01	4.64E-01
**G42509**	0.81	4.05E-01	4.65E-01
**CCL20**	0.77	4.05E-01	4.65E-01
**PPP2CA**	-0.12	4.05E-01	4.65E-01
**RPA4**	-0.50	4.05E-01	4.65E-01
**FSCN2**	0.35	4.05E-01	4.65E-01
**ZNF311**	0.25	4.06E-01	4.66E-01
**RP11-131L23.2**	0.85	4.06E-01	4.66E-01
**CTD-3051D23.4**	-0.69	4.06E-01	4.66E-01
**RP11-8L2.1**	-1.56	4.06E-01	4.66E-01
**AC008592.8**	0.50	4.06E-01	4.66E-01

**LEF1-AS1**	-0.40	4.06E-01	4.66E-01
**CD3G**	0.43	4.06E-01	4.66E-01
**HLA-U**	-1.00	4.06E-01	4.66E-01
**HMGN1P24**	-0.83	4.06E-01	4.67E-01
**RP11-78O7.2**	0.31	4.06E-01	4.67E-01
**ACVR1B**	0.11	4.07E-01	4.67E-01
**RP5-1098D14.1**	0.77	4.07E-01	4.67E-01
**LMLN**	-0.13	4.07E-01	4.67E-01
**NIT2**	-0.09	4.07E-01	4.67E-01
**RP11-422P24.10**	0.35	4.07E-01	4.67E-01
**RP11-778D9.12**	-0.49	4.07E-01	4.68E-01
**MAN2A2**	-0.17	4.07E-01	4.68E-01
**DPH3**	-0.13	4.07E-01	4.68E-01
**RNU4-40P**	0.71	4.08E-01	4.68E-01
**FGF16**	-0.82	4.08E-01	4.68E-01
**CHMP1B**	0.13	4.08E-01	4.68E-01
**RP11-741G21.1**	-0.99	4.08E-01	4.68E-01
**RP11-301O19.1**	0.47	4.08E-01	4.68E-01
**ZNF835**	0.36	4.08E-01	4.69E-01
**PDP2**	-0.14	4.08E-01	4.69E-01
**PARP4**	-0.12	4.09E-01	4.69E-01
**C7orf61**	0.90	4.09E-01	4.69E-01
**EIF5**	-0.14	4.09E-01	4.69E-01
**AC133106.2**	0.64	4.09E-01	4.69E-01
**EEF1E1P1**	-1.07	4.09E-01	4.69E-01
**ZSWIM1**	-0.11	4.09E-01	4.69E-01
**CELF5**	0.62	4.09E-01	4.69E-01
**HIST3H3**	-1.11	4.09E-01	4.69E-01
**RP11-603J24.21**	-0.69	4.09E-01	4.69E-01
**DDX10**	-0.11	4.09E-01	4.70E-01
**RPS3AP47**	-0.37	4.11E-01	4.71E-01
**NPC1L1**	0.58	4.11E-01	4.71E-01
**CTB-147C22.8**	0.82	4.11E-01	4.71E-01
**RPS25**	0.13	4.11E-01	4.71E-01
**XLOC_001110**	-1.14	4.11E-01	4.71E-01
**MFSD9**	-0.09	4.11E-01	4.71E-01
**XLOC_013263**	-0.61	4.11E-01	4.71E-01
**ITGA9**	-0.15	4.11E-01	4.71E-01
**RP11-651P23.2**	-0.77	4.11E-01	4.71E-01
**NINJ1**	-0.18	4.11E-01	4.71E-01
**VDR**	0.12	4.11E-01	4.72E-01
**RP11-325L7.1**	-0.91	4.12E-01	4.72E-01
**PCBP3-OT1**	-0.57	4.12E-01	4.72E-01
**CCZ1B**	-0.15	4.12E-01	4.72E-01
**ERLIN2**	-0.12	4.12E-01	4.72E-01

**LINC01549**	0.59	4.12E-01	4.72E-01
**BAD**	-0.13	4.12E-01	4.72E-01
**RP11-309L24.4**	0.23	4.12E-01	4.72E-01
**SMARCC2**	0.09	4.12E-01	4.73E-01
**MSTO2P**	0.31	4.12E-01	4.73E-01
**RPSAP8**	-0.90	4.12E-01	4.73E-01
**RPS26P15**	0.69	4.12E-01	4.73E-01
**RP11-342K6.4**	0.82	4.13E-01	4.73E-01
**SLC25A15**	-0.14	4.13E-01	4.73E-01
**AP000783.1**	0.88	4.13E-01	4.73E-01
**FAM83G**	-0.15	4.13E-01	4.73E-01
**CTD-2384B11.2**	-0.89	4.13E-01	4.73E-01
**RN7SL344P**	-1.05	4.13E-01	4.73E-01
**RP11-157J24.2**	-0.55	4.13E-01	4.73E-01
**ZSCAN26**	-0.13	4.13E-01	4.73E-01
**SNRNP200**	0.08	4.14E-01	4.74E-01
**RAP2C**	0.13	4.14E-01	4.74E-01
**C5orf67**	-0.45	4.14E-01	4.74E-01
**AP000295.10**	0.77	4.14E-01	4.74E-01
**TPTEP1**	0.30	4.14E-01	4.75E-01
**G12855**	-0.77	4.14E-01	4.75E-01
**SLC52A1**	0.28	4.14E-01	4.75E-01
**PHACTR4**	0.12	4.14E-01	4.75E-01
**IFIH1**	-0.14	4.15E-01	4.75E-01
**ORAI1**	0.13	4.15E-01	4.75E-01
**PEX13**	-0.10	4.15E-01	4.75E-01
**ACE2**	-0.31	4.15E-01	4.76E-01
**AHRR**	0.15	4.15E-01	4.76E-01
**NLGN4Y**	0.62	4.15E-01	4.76E-01
**CTD-2517O10.6**	-0.26	4.15E-01	4.76E-01
**RP11-153M3.1**	-0.97	4.16E-01	4.76E-01
**ICMT**	0.12	4.16E-01	4.76E-01
**RPS6P8**	-0.89	4.16E-01	4.76E-01
**DES**	-0.39	4.16E-01	4.76E-01
**FGFBP1**	-0.24	4.16E-01	4.76E-01
**GABPB1**	0.09	4.16E-01	4.76E-01
**RP11-1072C15.6**	0.49	4.16E-01	4.76E-01
**MYLIP**	-0.12	4.16E-01	4.76E-01
**ZCWPW2**	-0.17	4.16E-01	4.77E-01
**SHQ1**	-0.09	4.16E-01	4.77E-01
**FAM129C**	-0.54	4.16E-01	4.77E-01
**RP11-6J21.2**	-0.70	4.17E-01	4.77E-01
**XLOC_008756**	-0.57	4.17E-01	4.77E-01
**AC105760.2**	-0.27	4.17E-01	4.77E-01
**ALG1L9P**	-0.24	4.17E-01	4.77E-01

**FAM229A**	0.30	4.17E-01	4.78E-01
**RNU6-1024P**	0.67	4.17E-01	4.78E-01
**CTNNA1**	0.08	4.17E-01	4.78E-01
**RPL32P34**	-0.72	4.18E-01	4.78E-01
**TIPARP-AS1**	0.23	4.18E-01	4.78E-01
**ZNF683**	-0.49	4.18E-01	4.78E-01
**HMGN4**	0.11	4.18E-01	4.78E-01
**KLRG2**	0.29	4.18E-01	4.78E-01
**KRT8P43**	-1.09	4.18E-01	4.79E-01
**MIR4737**	-0.54	4.18E-01	4.79E-01
**TTLL11**	0.14	4.19E-01	4.79E-01
**NUP210**	0.17	4.19E-01	4.79E-01
**AC114730.11**	-0.73	4.19E-01	4.79E-01
**GS1-24F4.2**	-0.69	4.19E-01	4.79E-01
**CDK7**	0.14	4.19E-01	4.80E-01
**NPM1P27**	-0.18	4.20E-01	4.80E-01
**F5**	-0.43	4.20E-01	4.80E-01
**RP11-342D14.1**	-0.72	4.20E-01	4.80E-01
**TRIM58**	0.46	4.20E-01	4.81E-01
**LDHC**	-1.01	4.20E-01	4.81E-01
**BCL2L2**	0.10	4.20E-01	4.81E-01
**CTD-3203P2.1**	-0.57	4.20E-01	4.81E-01
**G33515**	0.72	4.20E-01	4.81E-01
**IGHV3-23**	1.43	4.21E-01	4.81E-01
**SLITRK4**	0.48	4.21E-01	4.81E-01
**SETP14**	0.39	4.21E-01	4.81E-01
**XLOC_011075**	0.44	4.21E-01	4.81E-01
**SLC16A5**	-0.12	4.21E-01	4.81E-01
**AL359836.1**	-0.77	4.21E-01	4.81E-01
**CTC-301O7.4**	-0.57	4.21E-01	4.82E-01
**ZNF229**	-0.27	4.22E-01	4.82E-01
**GPM6B**	-0.23	4.22E-01	4.83E-01
**RP11-254I22.1**	-0.47	4.22E-01	4.83E-01
**UPF3BP4**	-0.90	4.22E-01	4.83E-01
**GGCT**	-0.21	4.22E-01	4.83E-01
**COX6A1P2**	-0.30	4.22E-01	4.83E-01
**LRP5L**	0.22	4.23E-01	4.83E-01
**RN7SL689P**	-0.48	4.23E-01	4.83E-01
**MGAT5**	0.08	4.23E-01	4.83E-01
**SLC25A17**	0.14	4.23E-01	4.83E-01
**RP11-292F22.7**	-0.50	4.23E-01	4.83E-01
**DNTTIP2**	0.13	4.23E-01	4.84E-01
**SLC10A4**	0.70	4.23E-01	4.84E-01
**RP11-195B21.3**	-0.81	4.23E-01	4.84E-01
**CTD-2523D13.2**	0.37	4.24E-01	4.84E-01

**RP11-20I20.1**	1.05	4.24E-01	4.84E-01
**RP11-576N17.5**	-1.08	4.24E-01	4.84E-01
**XLOC_005856**	-0.94	4.24E-01	4.84E-01
**IL36B**	0.34	4.24E-01	4.84E-01
**RP11-49I11.4**	0.46	4.24E-01	4.85E-01
**AKR1D1**	-1.26	4.24E-01	4.85E-01
**RP11-571M6.18**	-0.37	4.24E-01	4.85E-01
**RP11-107E5.4**	-0.52	4.24E-01	4.85E-01
**RP11-650P15.1**	0.88	4.25E-01	4.85E-01
**CYP1B1**	-0.32	4.25E-01	4.85E-01
**ZNF436-AS1**	-0.27	4.25E-01	4.85E-01
**RHD**	0.70	4.25E-01	4.85E-01
**KPNB1**	0.07	4.25E-01	4.85E-01
**FAM209A**	0.57	4.25E-01	4.86E-01
**LINC00449**	-0.72	4.25E-01	4.86E-01
**HORMAD1**	-0.87	4.25E-01	4.86E-01
**RP11-30K9.6**	-0.27	4.25E-01	4.86E-01
**RP11-557H15.4**	-0.43	4.25E-01	4.86E-01
**G22135**	-0.81	4.25E-01	4.86E-01
**RP11-474I16.8**	0.66	4.25E-01	4.86E-01
**AP001469.9**	-0.33	4.26E-01	4.86E-01
**CTD-2026G6.3**	-1.03	4.26E-01	4.86E-01
**CHRNA7**	0.41	4.26E-01	4.87E-01
**RP1-136B1.1**	-0.78	4.26E-01	4.87E-01
**RP5-867C24.4**	0.49	4.26E-01	4.87E-01
**COX17**	0.12	4.26E-01	4.87E-01
**LINC01186**	-0.78	4.26E-01	4.87E-01
**RP11-286N22.16**	-1.14	4.26E-01	4.87E-01
**YWHAQP5**	0.83	4.26E-01	4.87E-01
**COIL**	0.10	4.26E-01	4.87E-01
**TRGC2**	-0.59	4.26E-01	4.87E-01
**RP11-281O15.4**	0.62	4.27E-01	4.87E-01
**RP11-557L19.1**	1.14	4.27E-01	4.87E-01
**SLC16A4**	-0.30	4.27E-01	4.87E-01
**HSPD1**	-0.14	4.27E-01	4.87E-01
**RP11-284F21.9**	0.95	4.27E-01	4.88E-01
**RDX**	0.12	4.27E-01	4.88E-01
**CTB-113I20.2**	0.27	4.27E-01	4.88E-01
**AP000304.1**	-0.77	4.28E-01	4.88E-01
**SULT1A2**	-0.55	4.28E-01	4.88E-01
**CTD-3060P21.1**	0.75	4.28E-01	4.88E-01
**RP11-572M11.1**	0.94	4.28E-01	4.88E-01
**CFHR3**	0.94	4.28E-01	4.88E-01
**PARP6**	0.12	4.28E-01	4.88E-01
**RP11-14C10.3**	-0.77	4.28E-01	4.88E-01

**DNALI1**	0.23	4.28E-01	4.88E-01
**SCP2**	-0.14	4.28E-01	4.88E-01
**APOL6**	0.20	4.28E-01	4.89E-01
**G9514**	-0.53	4.28E-01	4.89E-01
**ENOPH1**	-0.12	4.28E-01	4.89E-01
**XLOC_003808**	-0.80	4.29E-01	4.89E-01
**IGSF1**	0.59	4.29E-01	4.89E-01
**LCE1C**	0.27	4.29E-01	4.89E-01
**RABEP1**	-0.07	4.29E-01	4.89E-01
**PLPP6**	-0.10	4.29E-01	4.89E-01
**IGSF5**	0.81	4.29E-01	4.89E-01
**RP4-635A23.4**	0.49	4.29E-01	4.89E-01
**PLSCR2**	-0.59	4.29E-01	4.90E-01
**KIAA1161**	-0.15	4.29E-01	4.90E-01
**XLOC_001013**	-0.75	4.29E-01	4.90E-01
**LUC7L2**	-0.09	4.30E-01	4.90E-01
**RP11-56M3.1**	0.73	4.30E-01	4.90E-01
**BAAT**	-0.99	4.30E-01	4.90E-01
**RP11-481A20.10**	-0.99	4.30E-01	4.90E-01
**RP11-430C7.5**	0.72	4.30E-01	4.90E-01
**ITGA2B**	-0.37	4.30E-01	4.90E-01
**RP11-74E22.4**	-0.64	4.30E-01	4.90E-01
**ATP5L**	-0.13	4.30E-01	4.91E-01
**C3orf67**	0.25	4.30E-01	4.91E-01
**MRPS17P1**	-0.85	4.31E-01	4.91E-01
**SNX20**	-0.27	4.31E-01	4.91E-01
**SNHG8**	-0.22	4.31E-01	4.91E-01
**RP1-172I22.1**	-1.34	4.31E-01	4.91E-01
**SOGA1**	-0.17	4.31E-01	4.91E-01
**STARD6**	0.58	4.31E-01	4.91E-01
**RPL23P2**	0.43	4.31E-01	4.91E-01
**AC090587.5**	0.44	4.31E-01	4.91E-01
**RP4-593H12.1**	-0.74	4.31E-01	4.92E-01
**RP11-484N16.1**	-0.71	4.31E-01	4.92E-01
**MIR4664**	0.29	4.32E-01	4.92E-01
**UBE3D**	-0.16	4.32E-01	4.92E-01
**SUGT1P**	-0.32	4.32E-01	4.92E-01
**YEATS4**	-0.15	4.32E-01	4.92E-01
**RP11-402J7.2**	-0.96	4.32E-01	4.92E-01
**CNTLN**	0.13	4.32E-01	4.92E-01
**CTNNA2**	-0.47	4.32E-01	4.92E-01
**UPF3AP1**	0.63	4.32E-01	4.92E-01
**RP5-1120P11.1**	0.62	4.32E-01	4.92E-01
**SLC7A11**	-0.30	4.32E-01	4.93E-01
**RP11-148B18.4**	-0.66	4.32E-01	4.93E-01

**RP11-77K12.5**	-0.49	4.32E-01	4.93E-01
**CTLA4**	-0.55	4.33E-01	4.93E-01
**TTTY14**	0.33	4.33E-01	4.93E-01
**RNU6-731P**	-0.89	4.33E-01	4.93E-01
**SF3B6**	0.15	4.33E-01	4.93E-01
**RP11-175P13.2**	-0.87	4.33E-01	4.94E-01
**CYP4F24P**	-0.90	4.33E-01	4.94E-01
**TLDC2**	-0.32	4.34E-01	4.94E-01
**CCDC183-AS1**	0.38	4.34E-01	4.94E-01
**CTD-3022L24.1**	-0.45	4.34E-01	4.94E-01
**RP11-284M14.1**	0.35	4.34E-01	4.94E-01
**AK6**	-0.14	4.34E-01	4.94E-01
**TRIM31-AS1**	-0.63	4.34E-01	4.94E-01
**TDRD7**	0.11	4.34E-01	4.94E-01
**LRRK1**	-0.11	4.34E-01	4.94E-01
**SPX**	-1.02	4.34E-01	4.94E-01
**TEX30**	0.12	4.34E-01	4.94E-01
**ALG12**	0.09	4.34E-01	4.94E-01
**LBR**	-0.09	4.34E-01	4.94E-01
**UXT**	-0.11	4.34E-01	4.94E-01
**CTD-2583A14.11**	0.34	4.34E-01	4.94E-01
**RP11-218L14.4**	0.98	4.34E-01	4.95E-01
**MDGA1**	0.54	4.35E-01	4.95E-01
**RP11-191L17.1**	0.82	4.35E-01	4.95E-01
**AC073343.13**	-0.67	4.35E-01	4.95E-01
**RP5-866L20.1**	-0.64	4.35E-01	4.95E-01
**AC091729.8**	-0.71	4.35E-01	4.95E-01
**CTD-2341M24.1**	0.47	4.35E-01	4.95E-01
**RP11-689P11.2**	0.39	4.35E-01	4.95E-01
**SNAI3**	0.33	4.35E-01	4.95E-01
**KREMEN2**	0.25	4.35E-01	4.95E-01
**IL1RL1**	-0.38	4.36E-01	4.96E-01
**NNT-AS1**	0.14	4.36E-01	4.96E-01
**IL23R**	0.66	4.36E-01	4.96E-01
**YARS2**	0.09	4.36E-01	4.96E-01
**GBF1**	0.08	4.36E-01	4.96E-01
**RP11-1149M10.2**	-0.72	4.36E-01	4.96E-01
**CCT2**	0.11	4.36E-01	4.96E-01
**GAL3ST2**	-0.68	4.36E-01	4.96E-01
**HSPB8**	0.13	4.37E-01	4.97E-01
**XLOC_006932**	0.77	4.37E-01	4.97E-01
**RP11-977G19.11**	-0.47	4.37E-01	4.97E-01
**LINC00680**	0.14	4.37E-01	4.97E-01
**SEC16A**	0.10	4.37E-01	4.97E-01
**CALCR**	0.86	4.37E-01	4.97E-01

**DCD**	1.27	4.37E-01	4.97E-01
**AGAP6**	0.23	4.37E-01	4.97E-01
**RIBC1**	-0.22	4.37E-01	4.97E-01
**1-Mar**	0.32	4.37E-01	4.97E-01
**G1115**	0.93	4.38E-01	4.97E-01
**MYL12BP1**	-0.58	4.38E-01	4.98E-01
**LMO1**	0.21	4.38E-01	4.98E-01
**ZNF878**	0.49	4.38E-01	4.98E-01
**MOB3B**	-0.09	4.38E-01	4.98E-01
**GLI1**	-0.37	4.38E-01	4.98E-01
**ZNF610**	-0.30	4.38E-01	4.98E-01
**KRTAP4-11**	-2.00	4.38E-01	4.98E-01
**RP11-230F18.5**	-0.26	4.38E-01	4.98E-01
**POLR1C**	0.10	4.38E-01	4.98E-01
**RP11-316M21.7**	0.38	4.39E-01	4.99E-01
**HIGD2A**	-0.11	4.39E-01	4.99E-01
**RP1-50J22.4**	0.65	4.39E-01	4.99E-01
**LRRTM1**	-0.83	4.39E-01	4.99E-01
**MATN1-AS1**	-0.20	4.39E-01	4.99E-01
**PSMD5**	-0.08	4.39E-01	4.99E-01
**RP11-10K16.1**	0.53	4.39E-01	4.99E-01
**ATG4C**	-0.14	4.39E-01	4.99E-01
**AC004490.1**	-0.36	4.39E-01	4.99E-01
**MOK**	-0.15	4.40E-01	4.99E-01
**LINC00892**	-0.63	4.40E-01	5.00E-01
**AC005253.2**	-0.31	4.40E-01	5.00E-01
**C6orf58**	-0.59	4.40E-01	5.00E-01
**VWA3B**	0.59	4.40E-01	5.00E-01
**PCGF3**	-0.09	4.40E-01	5.00E-01
**TMPRSS5**	-0.28	4.41E-01	5.00E-01
**CTD-2349P21.5**	0.53	4.41E-01	5.01E-01
**SMN2**	-0.53	4.41E-01	5.01E-01
**RP11-549B18.1**	-0.32	4.41E-01	5.01E-01
**G18287**	0.41	4.41E-01	5.01E-01
**AGPAT1**	0.25	4.41E-01	5.01E-01
**OR7E106P**	-1.14	4.41E-01	5.01E-01
**KIAA0753**	-0.10	4.41E-01	5.01E-01
**AGBL5**	0.08	4.41E-01	5.01E-01
**LINC00540**	-0.64	4.41E-01	5.01E-01
**RP1-199J3.5**	0.54	4.41E-01	5.01E-01
**C2orf73**	-0.55	4.41E-01	5.01E-01
**BEX4**	0.16	4.42E-01	5.02E-01
**AC006277.2**	-0.61	4.42E-01	5.02E-01
**RP11-497E19.1**	0.28	4.42E-01	5.02E-01
**DNAH5**	-0.54	4.42E-01	5.02E-01

**TAB2**	0.09	4.42E-01	5.02E-01
**ERMARD**	-0.10	4.42E-01	5.02E-01
**RP11-140I16.3**	-0.45	4.42E-01	5.02E-01
**CTD-2410N18.4**	-0.40	4.42E-01	5.02E-01
**RP11-266K4.14**	0.33	4.42E-01	5.02E-01
**RNU6-790P**	0.71	4.43E-01	5.02E-01
**AC139099.4**	-0.63	4.43E-01	5.02E-01
**SYCE1**	0.92	4.43E-01	5.03E-01
**RP11-534C12.1**	-0.44	4.43E-01	5.03E-01
**LINC00636**	-0.77	4.43E-01	5.03E-01
**KCNN2**	0.26	4.43E-01	5.03E-01
**AC096670.3**	0.62	4.43E-01	5.03E-01
**FAM150B**	-0.23	4.43E-01	5.03E-01
**AC009227.2**	-1.20	4.44E-01	5.03E-01
**SNORD51**	0.40	4.44E-01	5.03E-01
**FLJ43879**	-0.76	4.44E-01	5.03E-01
**G36480**	-1.16	4.44E-01	5.03E-01
**CHMP5**	0.13	4.44E-01	5.04E-01
**RNU6-125P**	-0.61	4.44E-01	5.04E-01
**AC002451.3**	-0.52	4.44E-01	5.04E-01
**RP11-351I24.1**	0.45	4.45E-01	5.05E-01
**RP3-449M8.9**	-0.48	4.45E-01	5.05E-01
**FAM156A**	-0.28	4.45E-01	5.05E-01
**FOXO3**	-0.08	4.45E-01	5.05E-01
**RP11-88E10.5**	0.23	4.45E-01	5.05E-01
**GS1-124K5.4**	0.28	4.45E-01	5.05E-01
**HSPA8P4**	0.55	4.46E-01	5.05E-01
**TWF1**	0.15	4.46E-01	5.06E-01
**CTD-2527I21.15**	0.42	4.46E-01	5.06E-01
**GTF2H1**	-0.07	4.46E-01	5.06E-01
**KRTAP19-3**	-2.97	4.46E-01	5.06E-01
**CTD-2095E4.5**	0.26	4.47E-01	5.07E-01
**RP11-278C7.4**	-0.26	4.47E-01	5.07E-01
**CTD-2270L9.2**	-0.55	4.47E-01	5.07E-01
**CELA2B**	-0.72	4.47E-01	5.07E-01
**C12orf57**	0.15	4.47E-01	5.07E-01
**C2orf27A**	0.28	4.47E-01	5.07E-01
**DLEU7-AS1**	-0.68	4.47E-01	5.07E-01
**PSORS1C3**	-0.55	4.48E-01	5.08E-01
**RP11-39C10.1**	-1.06	4.49E-01	5.09E-01
**PPP1R36**	-0.19	4.49E-01	5.09E-01
**TFAP2C**	0.11	4.49E-01	5.09E-01
**LINC00882**	0.42	4.49E-01	5.09E-01
**RP11-1012E15.2**	-0.98	4.49E-01	5.09E-01
**RP11-252I14.1**	-0.79	4.49E-01	5.09E-01

**WDR11-AS1**	0.78	4.49E-01	5.10E-01
**RP11-666A20.4**	0.47	4.50E-01	5.10E-01
**TYMSOS**	0.51	4.50E-01	5.10E-01
**FAM69A**	0.13	4.50E-01	5.10E-01
**RP11-162J8.3**	0.64	4.50E-01	5.10E-01
**GSTT1**	-0.86	4.50E-01	5.11E-01
**MORN1**	-0.15	4.51E-01	5.11E-01
**BRD7**	-0.08	4.51E-01	5.11E-01
**KRT18P34**	-0.38	4.51E-01	5.11E-01
**HIGD1A**	-0.15	4.51E-01	5.11E-01
**RP11-126O1.5**	0.87	4.51E-01	5.11E-01
**LCE4A**	-0.33	4.51E-01	5.11E-01
**CARD8**	0.15	4.51E-01	5.11E-01
**RP11-44B19.1**	0.69	4.51E-01	5.11E-01
**DPY19L2P4**	-0.80	4.52E-01	5.12E-01
**G42804**	-0.78	4.52E-01	5.12E-01
**RP11-344B5.2**	-0.27	4.52E-01	5.12E-01
**G35261**	-0.27	4.52E-01	5.12E-01
**G38764**	-0.61	4.52E-01	5.12E-01
**LY6G5B**	-0.34	4.52E-01	5.12E-01
**G34374**	-0.67	4.52E-01	5.13E-01
**TATDN3**	0.13	4.53E-01	5.13E-01
**CTD-2020K17.1**	0.39	4.53E-01	5.13E-01
**OVGP1**	0.27	4.53E-01	5.13E-01
**DLD**	-0.10	4.53E-01	5.13E-01
**PPP1R32**	0.26	4.53E-01	5.13E-01
**CTD-3032J10.4**	0.58	4.53E-01	5.14E-01
**TCP11**	0.52	4.54E-01	5.14E-01
**G32318**	0.89	4.54E-01	5.14E-01
**LINC01184**	-0.13	4.54E-01	5.14E-01
**RAD51AP1**	-0.15	4.54E-01	5.14E-01
**RN7SL832P**	0.41	4.54E-01	5.14E-01
**FAIM**	0.12	4.54E-01	5.14E-01
**SRP72**	0.09	4.54E-01	5.14E-01
**AXL**	0.24	4.54E-01	5.14E-01
**KCNE5**	0.66	4.55E-01	5.15E-01
**XLOC_005247**	-0.20	4.55E-01	5.15E-01
**PDCD4**	-0.13	4.55E-01	5.15E-01
**SLC25A36P1**	-0.69	4.55E-01	5.15E-01
**DGCR9**	0.60	4.55E-01	5.15E-01
**RP11-380G5.3**	0.34	4.55E-01	5.15E-01
**RP11-140K17.3**	0.21	4.55E-01	5.15E-01
**RP11-533E19.5**	0.47	4.55E-01	5.15E-01
**GPR34**	0.35	4.56E-01	5.16E-01
**CTD-2024P10.1**	-0.76	4.56E-01	5.16E-01

**MORF4L1P4**	-0.96	4.56E-01	5.16E-01
**ADAMTS15**	0.36	4.56E-01	5.17E-01
**RP11-680G24.5**	-0.38	4.56E-01	5.17E-01
**SHISA6**	0.31	4.56E-01	5.17E-01
**KRT4**	0.64	4.57E-01	5.17E-01
**AC144831.1**	0.21	4.57E-01	5.17E-01
**RAB5A**	-0.13	4.57E-01	5.17E-01
**BLM**	-0.14	4.57E-01	5.17E-01
**SLC5A11**	-0.54	4.57E-01	5.17E-01
**RP11-379F4.6**	0.36	4.57E-01	5.17E-01
**CTA-984G1.5**	-0.53	4.57E-01	5.17E-01
**RP11-182J1.17**	0.68	4.58E-01	5.18E-01
**RP5-1139B12.2**	-0.28	4.58E-01	5.18E-01
**NUP88**	-0.09	4.58E-01	5.18E-01
**RP11-1348G14.4**	0.34	4.58E-01	5.18E-01
**MPZ**	-0.29	4.58E-01	5.18E-01
**DNAJB5-AS1**	-0.86	4.58E-01	5.18E-01
**RP11-21B23.1**	-0.66	4.58E-01	5.18E-01
**CATSPER2P1**	0.21	4.58E-01	5.18E-01
**SLC25A29**	0.17	4.58E-01	5.19E-01
**GS1-590J6.3**	-0.52	4.59E-01	5.19E-01
**ACOT1**	-0.67	4.59E-01	5.19E-01
**COX14**	0.12	4.59E-01	5.19E-01
**MT1XP1**	0.57	4.59E-01	5.19E-01
**CTD-2554C21.3**	0.31	4.59E-01	5.19E-01
**PKNOX2**	-0.19	4.59E-01	5.19E-01
**LDLRAD4-AS1**	0.41	4.59E-01	5.19E-01
**IGFLR1**	-0.38	4.60E-01	5.20E-01
**CAPN13**	-0.61	4.60E-01	5.20E-01
**VDAC1P1**	-0.69	4.60E-01	5.20E-01
**CRMP1**	-0.16	4.60E-01	5.20E-01
**DNAJA1**	0.15	4.60E-01	5.20E-01
**HEPACAM**	1.20	4.60E-01	5.20E-01
**IWS1**	0.07	4.60E-01	5.20E-01
**MYCL**	-0.20	4.60E-01	5.20E-01
**RP11-340C20.3**	-1.32	4.60E-01	5.20E-01
**CTC-340D7.1**	-0.95	4.60E-01	5.20E-01
**RP1-86C11.7**	-0.61	4.60E-01	5.20E-01
**TUBB4B**	-0.14	4.60E-01	5.20E-01
**B9D1**	0.16	4.61E-01	5.21E-01
**ATG3**	0.10	4.61E-01	5.21E-01
**MRPS28**	-0.11	4.61E-01	5.21E-01
**SETD4**	-0.13	4.61E-01	5.21E-01
**DSCR3**	0.06	4.61E-01	5.21E-01
**UMPS**	0.09	4.61E-01	5.21E-01

**XLOC_005254**	-0.69	4.61E-01	5.21E-01
**CTD-2541J13.1**	0.46	4.61E-01	5.21E-01
**PDCD1LG2**	0.32	4.61E-01	5.21E-01
**G8261**	0.78	4.61E-01	5.21E-01
**KRTAP5-9**	0.75	4.61E-01	5.21E-01
**RP11-538D16.3**	-0.69	4.61E-01	5.21E-01
**KB-1507C5.4**	0.40	4.62E-01	5.21E-01
**MIR5094**	0.65	4.62E-01	5.22E-01
**AGAP2-AS1**	0.35	4.62E-01	5.22E-01
**11-Sep**	0.25	4.62E-01	5.22E-01
**RP11-1102P22.1**	-0.44	4.62E-01	5.22E-01
**RP11-699C17.1**	-0.41	4.62E-01	5.22E-01
**POU5F1**	0.25	4.62E-01	5.22E-01
**FAM66D**	-0.56	4.63E-01	5.22E-01
**bP-21264C1.2**	0.39	4.63E-01	5.22E-01
**GZMK**	0.86	4.63E-01	5.22E-01
**NFRKB**	0.08	4.63E-01	5.22E-01
**SIN3A**	-0.08	4.63E-01	5.23E-01
**C1QTNF2**	0.25	4.63E-01	5.23E-01
**PPP1R10**	0.11	4.63E-01	5.23E-01
**SKAP2**	-0.12	4.63E-01	5.23E-01
**RAD51AP2**	0.42	4.64E-01	5.23E-01
**RP11-69I8.3**	0.49	4.64E-01	5.23E-01
**ZNF763**	0.21	4.64E-01	5.23E-01
**MFSD7**	0.18	4.64E-01	5.24E-01
**LINC00707**	0.48	4.64E-01	5.24E-01
**RBM5**	-0.14	4.64E-01	5.24E-01
**ZSCAN25**	-0.08	4.64E-01	5.24E-01
**IGLV1-44**	0.94	4.64E-01	5.24E-01
**G39661**	-0.37	4.64E-01	5.24E-01
**CDNF**	-0.16	4.65E-01	5.24E-01
**MLLT3**	-0.11	4.65E-01	5.25E-01
**JMJD1C-AS1**	-0.35	4.65E-01	5.25E-01
**RP1-29C18.9**	0.81	4.65E-01	5.25E-01
**CTB-79E8.3**	-0.34	4.65E-01	5.25E-01
**RP11-73K9.2**	0.23	4.65E-01	5.25E-01
**ACSL3**	0.15	4.65E-01	5.25E-01
**IL11**	0.37	4.65E-01	5.25E-01
**MLLT11**	-0.18	4.66E-01	5.25E-01
**RP11-210M15.2**	0.43	4.66E-01	5.25E-01
**AGPAT5**	-0.15	4.66E-01	5.25E-01
**TNFSF18**	0.57	4.66E-01	5.25E-01
**PFN1P3**	0.35	4.66E-01	5.26E-01
**TRPM3**	0.33	4.66E-01	5.26E-01
**RP11-435B5.3**	0.96	4.66E-01	5.26E-01

**C12orf77**	-0.54	4.66E-01	5.26E-01
**BLOC1S2**	0.13	4.66E-01	5.26E-01
**GOLGA7**	-0.12	4.66E-01	5.26E-01
**CEP55**	0.20	4.67E-01	5.26E-01
**RP1-29C18.8**	-0.61	4.67E-01	5.26E-01
**SPINK13**	-0.90	4.67E-01	5.26E-01
**MRC1L1**	0.42	4.67E-01	5.27E-01
**ZNF77**	-0.12	4.67E-01	5.27E-01
**PDLIM2**	-0.12	4.67E-01	5.27E-01
**THRAP3**	-0.08	4.67E-01	5.27E-01
**SLC24A2**	-0.47	4.67E-01	5.27E-01
**IGSF22**	0.20	4.67E-01	5.27E-01
**PDSS1**	0.12	4.68E-01	5.27E-01
**RNU6ATAC27P**	-0.54	4.68E-01	5.27E-01
**TENM4**	-0.12	4.68E-01	5.27E-01
**RP11-131M11.3**	-0.29	4.68E-01	5.27E-01
**XXbac-B476C20.9**	-0.28	4.68E-01	5.27E-01
**BMS1**	-0.08	4.68E-01	5.27E-01
**RP5-994D16.11**	0.46	4.68E-01	5.28E-01
**FSIP1**	0.35	4.68E-01	5.28E-01
**TMEM52**	-0.31	4.68E-01	5.28E-01
**RAMP2-AS1**	0.34	4.69E-01	5.28E-01
**FXR1**	0.07	4.69E-01	5.29E-01
**ZNF114**	-0.28	4.69E-01	5.29E-01
**RP1-74B13.2**	-0.72	4.69E-01	5.29E-01
**CTC-490E21.10**	0.36	4.69E-01	5.29E-01
**RNU6-722P**	0.60	4.70E-01	5.30E-01
**PPIP5K1**	-0.10	4.70E-01	5.30E-01
**UGT2B7**	0.53	4.70E-01	5.30E-01
**LINC00173**	-0.33	4.70E-01	5.30E-01
**NARS2**	0.09	4.70E-01	5.30E-01
**CTB-58E17.5**	0.47	4.71E-01	5.30E-01
**RPS4XP5**	0.50	4.71E-01	5.30E-01
**IGHV3-74**	-0.92	4.71E-01	5.30E-01
**LINC01115**	-0.86	4.71E-01	5.30E-01
**CSPP1**	0.11	4.71E-01	5.30E-01
**PROS1**	0.12	4.71E-01	5.31E-01
**PPM1D**	0.08	4.71E-01	5.31E-01
**RP4-761J14.10**	-0.32	4.71E-01	5.31E-01
**G11043**	-0.31	4.71E-01	5.31E-01
**SMPD2**	-0.13	4.72E-01	5.31E-01
**RP11-347C12.11**	0.67	4.72E-01	5.31E-01
**RNU6-312P**	-0.80	4.72E-01	5.31E-01
**CLCNKA**	0.87	4.72E-01	5.32E-01
**RP11-723O4.9**	0.47	4.72E-01	5.32E-01

**DIRAS3**	-0.35	4.73E-01	5.32E-01
**TEX35**	-0.68	4.73E-01	5.32E-01
**TMPRSS9**	-0.30	4.73E-01	5.33E-01
**RP11-756P10.6**	0.32	4.73E-01	5.33E-01
**C2orf66**	0.49	4.73E-01	5.33E-01
**RN7SL749P**	-0.76	4.73E-01	5.33E-01
**RP11-466F5.6**	-0.64	4.73E-01	5.33E-01
**RPL19**	0.10	4.73E-01	5.33E-01
**LA16c-316G12.2**	-0.63	4.73E-01	5.33E-01
**RP11-400F19.6**	-0.69	4.73E-01	5.33E-01
**RPL12P12**	-0.51	4.74E-01	5.33E-01
**RMI2**	0.11	4.74E-01	5.33E-01
**AL023807.1**	0.63	4.74E-01	5.33E-01
**CELF4**	0.28	4.74E-01	5.33E-01
**POLR2F**	0.43	4.74E-01	5.33E-01
**RP11-341G23.4**	-0.79	4.74E-01	5.34E-01
**RRN3P3**	-0.16	4.74E-01	5.34E-01
**GRK7**	0.52	4.74E-01	5.34E-01
**SLIRP**	-0.11	4.74E-01	5.34E-01
**RHOXF1**	0.59	4.74E-01	5.34E-01
**RP11-556H2.1**	0.51	4.75E-01	5.34E-01
**LINC01118**	0.46	4.75E-01	5.34E-01
**CASC2**	0.24	4.75E-01	5.34E-01
**KIF4A**	0.16	4.75E-01	5.34E-01
**TMPO-AS1**	0.23	4.75E-01	5.34E-01
**TIMM23**	0.09	4.75E-01	5.34E-01
**G41968**	-0.74	4.75E-01	5.35E-01
**ACOX2**	-0.45	4.75E-01	5.35E-01
**CTD-2012J19.3**	-0.30	4.76E-01	5.35E-01
**FAAH2**	-0.12	4.76E-01	5.35E-01
**RP1-315G1.3**	0.53	4.76E-01	5.35E-01
**RP11-4O1.2**	0.20	4.76E-01	5.36E-01
**AC104135.2**	-0.89	4.76E-01	5.36E-01
**LINC01232**	-0.18	4.76E-01	5.36E-01
**DCAF4**	-0.09	4.76E-01	5.36E-01
**RP11-521B24.5**	-0.33	4.76E-01	5.36E-01
**RP11-429D19.1**	0.33	4.76E-01	5.36E-01
**CTC-431G16.2**	-0.98	4.76E-01	5.36E-01
**WDR87**	-0.86	4.76E-01	5.36E-01
**XLOC_000852**	0.53	4.76E-01	5.36E-01
**CTD-2600O9.2**	0.54	4.77E-01	5.36E-01
**CDC16**	0.08	4.77E-01	5.36E-01
**SSC4D**	0.25	4.77E-01	5.37E-01
**AL672294.1**	-1.07	4.77E-01	5.37E-01
**AC009299.5**	0.54	4.77E-01	5.37E-01

**G42353**	-0.80	4.78E-01	5.37E-01
**TSN**	-0.09	4.78E-01	5.37E-01
**RHOU**	0.17	4.78E-01	5.37E-01
**RP11-217B7.2**	-0.35	4.78E-01	5.37E-01
**OR8T1P**	0.74	4.78E-01	5.37E-01
**G38642**	-0.88	4.78E-01	5.38E-01
**HEXA**	0.17	4.78E-01	5.38E-01
**DACT2**	0.16	4.78E-01	5.38E-01
**ZNF197-AS1**	-0.66	4.78E-01	5.38E-01
**RP11-569A2.2**	-0.64	4.79E-01	5.38E-01
**RNU7-181P**	-0.61	4.79E-01	5.38E-01
**RP11-45A17.2**	-0.26	4.79E-01	5.38E-01
**RP1-40E16.12**	-0.34	4.79E-01	5.38E-01
**RP11-5O17.1**	-0.30	4.79E-01	5.38E-01
**RBMS3-AS2**	-0.46	4.79E-01	5.38E-01
**RP11-95P13.1**	0.40	4.79E-01	5.38E-01
**EEF1A1P22**	-0.85	4.79E-01	5.38E-01
**ERP44**	0.08	4.79E-01	5.38E-01
**MYLK3**	0.51	4.79E-01	5.39E-01
**AL133245.2**	-0.38	4.80E-01	5.39E-01
**CTD-2179L22.1**	-0.45	4.80E-01	5.39E-01
**SRP54**	-0.09	4.80E-01	5.39E-01
**LL09NC01-251B2.3**	-0.57	4.80E-01	5.39E-01
**GP5**	-0.51	4.80E-01	5.39E-01
**G5246**	-0.53	4.80E-01	5.39E-01
**ZAN**	-0.45	4.80E-01	5.40E-01
**RP3-403A15.5**	-0.82	4.80E-01	5.40E-01
**RP11-324L17.1**	-0.55	4.80E-01	5.40E-01
**RP5-908M14.10**	-0.35	4.81E-01	5.40E-01
**SLC27A5**	0.15	4.81E-01	5.40E-01
**RPL9**	-0.15	4.81E-01	5.40E-01
**LHFPL3-AS1**	0.40	4.81E-01	5.40E-01
**CTC-499B15.5**	-0.31	4.81E-01	5.41E-01
**RP11-133K1.11**	-0.62	4.81E-01	5.41E-01
**RP11-295D4.1**	-0.36	4.82E-01	5.41E-01
**RNF146**	0.07	4.82E-01	5.41E-01
**RPS20P22**	0.48	4.82E-01	5.41E-01
**DAPK2**	0.17	4.82E-01	5.42E-01
**PRRC2C**	0.10	4.82E-01	5.42E-01
**PABPC1P4**	-0.27	4.83E-01	5.42E-01
**RNU2-69P**	-0.66	4.83E-01	5.42E-01
**RP11-165M1.1**	-0.50	4.83E-01	5.42E-01
**IFNA5**	-0.92	4.83E-01	5.42E-01
**AP000640.10**	-0.35	4.83E-01	5.42E-01
**RP4-756H11.3**	-0.21	4.83E-01	5.42E-01

**PEX7**	-0.11	4.83E-01	5.43E-01
**TLR1**	-0.19	4.84E-01	5.43E-01
**4-Mar**	-0.50	4.84E-01	5.43E-01
**MAVS**	-0.07	4.84E-01	5.43E-01
**ITM2B**	0.13	4.84E-01	5.44E-01
**TAS2R42**	-0.87	4.84E-01	5.44E-01
**RP11-167H9.4**	-0.70	4.84E-01	5.44E-01
**RP11-723O4.2**	-0.30	4.85E-01	5.44E-01
**TRPC6**	0.26	4.85E-01	5.44E-01
**SHISA2**	-0.40	4.85E-01	5.44E-01
**ZNF512**	0.14	4.85E-01	5.45E-01
**VMAC**	-0.17	4.85E-01	5.45E-01
**G31717**	-0.29	4.86E-01	5.45E-01
**CDC45**	-0.15	4.86E-01	5.45E-01
**PAEP**	0.55	4.86E-01	5.45E-01
**ZNF699**	0.22	4.86E-01	5.45E-01
**GACAT2**	0.61	4.86E-01	5.45E-01
**RN7SL180P**	-0.59	4.86E-01	5.45E-01
**BLZF2P**	0.52	4.86E-01	5.46E-01
**C5orf15**	-0.12	4.87E-01	5.46E-01
**DUSP27**	-0.77	4.87E-01	5.47E-01
**RP11-228B15.4**	0.27	4.87E-01	5.47E-01
**RP11-259O2.1**	-0.24	4.87E-01	5.47E-01
**MIR429**	-0.67	4.87E-01	5.47E-01
**RP11-451B8.1**	0.65	4.88E-01	5.47E-01
**ERICH5**	-0.29	4.88E-01	5.47E-01
**RP11-6L6.2**	0.65	4.88E-01	5.47E-01
**RP11-35G9.5**	-0.26	4.88E-01	5.47E-01
**TRPM8**	0.67	4.88E-01	5.47E-01
**AP001046.5**	0.15	4.88E-01	5.48E-01
**PTMAP4**	0.31	4.89E-01	5.48E-01
**RP11-996F15.4**	0.35	4.89E-01	5.48E-01
**COLCA1**	0.40	4.89E-01	5.48E-01
**MRGPRE**	0.52	4.89E-01	5.48E-01
**RRM2B**	0.13	4.89E-01	5.48E-01
**TBL1Y**	-0.32	4.89E-01	5.48E-01
**XLOC_011616**	-0.57	4.89E-01	5.48E-01
**SLC5A1**	-0.15	4.89E-01	5.49E-01
**TNS1**	0.16	4.89E-01	5.49E-01
**ZNF333**	-0.10	4.90E-01	5.49E-01
**PDK4**	0.27	4.90E-01	5.49E-01
**AC004985.12**	0.56	4.90E-01	5.49E-01
**PTGR2**	-0.13	4.90E-01	5.49E-01
**CLDN25**	-0.91	4.90E-01	5.50E-01
**RAB23**	0.13	4.90E-01	5.50E-01

**METTL24**	-0.37	4.91E-01	5.50E-01
**RP3-340B19.2**	-0.28	4.91E-01	5.50E-01
**AP000679.2**	-0.40	4.91E-01	5.50E-01
**G34216**	-0.83	4.91E-01	5.50E-01
**FAM221B**	-0.57	4.91E-01	5.50E-01
**FAM85B**	-0.40	4.91E-01	5.50E-01
**COX20P1**	-1.12	4.91E-01	5.51E-01
**CHMP4C**	-0.22	4.91E-01	5.51E-01
**ACOT4**	0.39	4.92E-01	5.51E-01
**RP11-17E13.2**	-0.53	4.92E-01	5.52E-01
**RNU6-758P**	0.64	4.92E-01	5.52E-01
**CTC-575I10.1**	-0.32	4.93E-01	5.52E-01
**XLOC_013994**	0.56	4.93E-01	5.52E-01
**CIDEA**	0.52	4.93E-01	5.52E-01
**FAM205A**	0.42	4.93E-01	5.52E-01
**TSPAN5**	0.11	4.93E-01	5.52E-01
**ANKRD20A7P**	-0.39	4.93E-01	5.53E-01
**LRP8**	-0.14	4.93E-01	5.53E-01
**RBMX**	-0.08	4.94E-01	5.53E-01
**RP11-1134I14.8**	0.52	4.94E-01	5.53E-01
**G18962**	-0.42	4.94E-01	5.53E-01
**ANAPC16**	0.11	4.94E-01	5.53E-01
**ABCA17P**	-0.28	4.94E-01	5.53E-01
**TMEM199**	0.09	4.94E-01	5.53E-01
**AP000807.1**	-0.71	4.94E-01	5.53E-01
**EP400**	-0.13	4.94E-01	5.53E-01
**CTD-2235C13.2**	-0.62	4.94E-01	5.53E-01
**MEF2B**	-0.59	4.94E-01	5.53E-01
**AC017002.2**	-0.65	4.94E-01	5.54E-01
**HOXB-AS2**	0.48	4.94E-01	5.54E-01
**CTD-2525I3.8**	-0.69	4.95E-01	5.54E-01
**IGSF6**	-0.16	4.95E-01	5.54E-01
**ADPRM**	0.11	4.95E-01	5.55E-01
**TXN**	0.14	4.95E-01	5.55E-01
**XLOC_009415**	-0.89	4.96E-01	5.55E-01
**RIOK3**	0.10	4.96E-01	5.55E-01
**RP11-156K23.3**	0.19	4.96E-01	5.55E-01
**BEX5**	0.25	4.96E-01	5.55E-01
**CORO2A**	-0.12	4.96E-01	5.55E-01
**RP1-313I6.12**	0.18	4.96E-01	5.55E-01
**NKAIN3**	-0.51	4.96E-01	5.55E-01
**RP11-60A14.1**	0.57	4.96E-01	5.55E-01
**PCAT18**	-0.99	4.96E-01	5.55E-01
**TMEM126B**	-0.09	4.96E-01	5.55E-01
**LINC00648**	-0.71	4.96E-01	5.55E-01

**RP11-45P15.4**	0.16	4.97E-01	5.56E-01
**KRT33A**	-1.06	4.97E-01	5.56E-01
**CLECL1**	-0.42	4.97E-01	5.56E-01
**RP11-1334A24.5**	0.45	4.97E-01	5.56E-01
**AC142528.1**	-0.58	4.97E-01	5.56E-01
**RP11-568A7.1**	-0.70	4.97E-01	5.57E-01
**TRIM72**	0.28	4.97E-01	5.57E-01
**PRKAA2**	-0.30	4.98E-01	5.57E-01
**CETN4P**	-0.71	4.98E-01	5.57E-01
**RP11-561B11.1**	0.68	4.98E-01	5.57E-01
**NDUFS7**	0.17	4.98E-01	5.57E-01
**DOCK8**	0.13	4.98E-01	5.57E-01
**PRKAR1A**	-0.09	4.98E-01	5.57E-01
**HOGA1**	-0.22	4.98E-01	5.58E-01
**DCAF11**	0.07	4.98E-01	5.58E-01
**CREBBP**	-0.10	4.99E-01	5.58E-01
**AC002059.10**	-0.80	4.99E-01	5.58E-01
**EIF3FP3**	-0.23	4.99E-01	5.58E-01
**CLK1**	-0.16	4.99E-01	5.58E-01
**G12891**	-0.37	4.99E-01	5.58E-01
**RP13-16H11.8**	-0.69	4.99E-01	5.58E-01
**RNF11**	0.12	4.99E-01	5.58E-01
**RP11-486B10.4**	-0.28	4.99E-01	5.58E-01
**RP1-122P22.2**	0.30	4.99E-01	5.58E-01
**ZKSCAN4**	0.09	5.00E-01	5.59E-01
**RP11-453E17.3**	-0.31	5.00E-01	5.59E-01
**C6orf141**	0.25	5.00E-01	5.59E-01
**RP11-667M19.9**	-0.41	5.00E-01	5.59E-01
**GOLGA8Q**	0.43	5.00E-01	5.59E-01
**RP11-16B9.1**	-0.62	5.01E-01	5.60E-01
**ASNSD1**	0.10	5.01E-01	5.60E-01
**XLOC_003411**	-0.23	5.01E-01	5.60E-01
**PTGDR**	0.27	5.01E-01	5.60E-01
**DYX1C1**	-0.38	5.01E-01	5.60E-01
**ATP10A**	-0.16	5.01E-01	5.60E-01
**ZNF385C**	0.32	5.01E-01	5.61E-01
**GNAS-AS1**	0.40	5.02E-01	5.61E-01
**RP11-774O3.3**	-0.21	5.02E-01	5.61E-01
**G10734**	-0.33	5.02E-01	5.62E-01
**SERPINA5**	0.39	5.02E-01	5.62E-01
**RP11-81H14.2**	0.46	5.03E-01	5.62E-01
**RNA5SP278**	-0.93	5.03E-01	5.62E-01
**RDM1**	-0.55	5.03E-01	5.62E-01
**RPL23AP25**	-0.65	5.03E-01	5.62E-01
**LINC01514**	-0.34	5.03E-01	5.62E-01

**MIR4520A**	-0.48	5.03E-01	5.62E-01
**GSTM2**	-0.27	5.03E-01	5.62E-01
**AC068831.3**	0.64	5.04E-01	5.63E-01
**LINC01271**	0.72	5.04E-01	5.63E-01
**AP001623.1**	-0.32	5.04E-01	5.63E-01
**HYDIN**	0.29	5.04E-01	5.63E-01
**RP11-333A23.4**	-1.12	5.04E-01	5.63E-01
**CITED1**	0.56	5.04E-01	5.63E-01
**PIANP**	0.27	5.05E-01	5.64E-01
**RPS26P47**	0.56	5.05E-01	5.64E-01
**UBE2Q2L**	-0.37	5.05E-01	5.64E-01
**ACTL6A**	0.10	5.05E-01	5.64E-01
**CTD-2619J13.9**	-0.40	5.05E-01	5.64E-01
**GHITM**	0.10	5.05E-01	5.64E-01
**LINC01285**	-0.58	5.05E-01	5.64E-01
**CENPL**	-0.13	5.05E-01	5.65E-01
**FAM71E1**	0.26	5.06E-01	5.65E-01
**IRX6**	0.31	5.06E-01	5.65E-01
**G17690**	0.42	5.06E-01	5.65E-01
**AF127577.8**	-0.75	5.06E-01	5.65E-01
**PLAC8**	0.33	5.06E-01	5.65E-01
**AC010894.3**	0.51	5.06E-01	5.65E-01
**ZNF48**	0.11	5.06E-01	5.65E-01
**ANO4**	0.45	5.06E-01	5.65E-01
**RPL23AP32**	0.36	5.06E-01	5.65E-01
**FAM72A**	0.19	5.06E-01	5.65E-01
**G8455**	0.32	5.06E-01	5.65E-01
**RP1-7G5.6**	-0.30	5.06E-01	5.65E-01
**PGBD2**	-0.11	5.06E-01	5.65E-01
**FRMPD4**	-0.62	5.06E-01	5.65E-01
**CBS**	-0.58	5.06E-01	5.65E-01
**SPIN2A**	-0.43	5.06E-01	5.65E-01
**TIMM10B**	-0.08	5.06E-01	5.65E-01
**RPS26P6**	0.67	5.07E-01	5.66E-01
**RAPGEFL1**	0.13	5.07E-01	5.66E-01
**RP11-120E11.2**	0.36	5.07E-01	5.66E-01
**POLR2J4**	-0.18	5.07E-01	5.66E-01
**NSG1**	0.14	5.07E-01	5.66E-01
**BRWD1-IT2**	-0.25	5.07E-01	5.66E-01
**MEMO1**	-0.20	5.07E-01	5.66E-01
**SLC13A3**	0.17	5.07E-01	5.66E-01
**PSMD14**	-0.11	5.08E-01	5.66E-01
**RP11-3K24.1**	0.43	5.08E-01	5.67E-01
**LINC01546**	-0.33	5.08E-01	5.67E-01
**PCCB**	-0.13	5.08E-01	5.67E-01

**RP11-20I23.10**	0.42	5.08E-01	5.67E-01
**CYP2U1**	-0.14	5.08E-01	5.67E-01
**RP11-429J17.2**	0.30	5.08E-01	5.67E-01
**C12orf4**	-0.11	5.08E-01	5.67E-01
**TMEM151B**	-0.76	5.08E-01	5.67E-01
**TCAF1P1**	-0.14	5.08E-01	5.67E-01
**GABBR1**	0.21	5.09E-01	5.67E-01
**STAMBP**	0.06	5.09E-01	5.67E-01
**SNRNP40**	0.08	5.09E-01	5.68E-01
**NBPF24**	-0.26	5.09E-01	5.68E-01
**BRMS1L**	-0.09	5.09E-01	5.68E-01
**CDC42BPG**	0.13	5.09E-01	5.68E-01
**BMS1P8**	0.58	5.09E-01	5.68E-01
**AC116366.6**	-0.29	5.09E-01	5.68E-01
**RP11-1348G14.1**	-0.65	5.10E-01	5.69E-01
**MRPS14**	0.09	5.10E-01	5.69E-01
**IFT122**	0.07	5.10E-01	5.69E-01
**PGAM5**	0.10	5.11E-01	5.69E-01
**RP11-464D20.2**	-0.44	5.11E-01	5.69E-01
**KIAA0513**	0.12	5.11E-01	5.70E-01
**CTD-2135D7.5**	0.36	5.11E-01	5.70E-01
**WWC3**	-0.05	5.11E-01	5.70E-01
**RP11-184A2.3**	-0.33	5.11E-01	5.70E-01
**G19448**	-0.50	5.11E-01	5.70E-01
**AC124944.3**	0.52	5.11E-01	5.70E-01
**RP11-174G17.3**	0.56	5.11E-01	5.70E-01
**RP11-429J17.5**	-0.73	5.12E-01	5.70E-01
**NBAT1**	0.46	5.12E-01	5.70E-01
**GRXCR2**	-0.42	5.12E-01	5.70E-01
**XLOC_001062**	-0.68	5.12E-01	5.70E-01
**MPRIP**	0.07	5.12E-01	5.70E-01
**ZNF702P**	-0.21	5.12E-01	5.70E-01
**BLK**	-0.57	5.12E-01	5.71E-01
**RP11-11N9.4**	0.34	5.12E-01	5.71E-01
**RP11-9N20.3**	-0.64	5.12E-01	5.71E-01
**RP11-481J2.2**	0.39	5.12E-01	5.71E-01
**HSD17B7**	0.12	5.12E-01	5.71E-01
**WBP1LP2**	-0.47	5.13E-01	5.71E-01
**ZNF646**	0.08	5.13E-01	5.71E-01
**RP11-1080G15.1**	0.64	5.13E-01	5.71E-01
**FAM86HP**	-0.21	5.13E-01	5.72E-01
**MEIG1**	-0.56	5.13E-01	5.72E-01
**AC069200.1**	0.51	5.13E-01	5.72E-01
**C18orf21**	-0.10	5.13E-01	5.72E-01
**RP11-253M7.6**	-0.78	5.13E-01	5.72E-01

**STOML3**	-0.62	5.13E-01	5.72E-01
**AF038458.3**	-0.58	5.14E-01	5.72E-01
**ZNF281**	0.10	5.14E-01	5.72E-01
**HS3ST3B1**	0.18	5.14E-01	5.72E-01
**AGBL5-IT1**	-0.27	5.14E-01	5.73E-01
**GLP2R**	-0.79	5.14E-01	5.73E-01
**LINC01474**	0.75	5.14E-01	5.73E-01
**LIPF**	-0.89	5.15E-01	5.73E-01
**CTD-3222D19.7**	-0.23	5.15E-01	5.73E-01
**RP11-80H18.4**	-0.72	5.15E-01	5.73E-01
**CTC-205M6.1**	0.34	5.15E-01	5.73E-01
**XLOC_013913**	-0.63	5.15E-01	5.73E-01
**ELANE**	-0.46	5.15E-01	5.73E-01
**G38630**	0.61	5.15E-01	5.73E-01
**ZNF695**	0.54	5.15E-01	5.73E-01
**MRPS36**	-0.11	5.15E-01	5.74E-01
**AC074289.1**	0.23	5.15E-01	5.74E-01
**G4573**	-0.44	5.15E-01	5.74E-01
**LILRB5**	0.33	5.15E-01	5.74E-01
**CDC42**	-0.10	5.15E-01	5.74E-01
**RP11-454P21.1**	-0.66	5.16E-01	5.74E-01
**RP11-167P11.2**	-0.40	5.16E-01	5.74E-01
**RHBDL2**	0.14	5.16E-01	5.74E-01
**OR7E14P**	0.48	5.16E-01	5.74E-01
**FLJ26850**	0.53	5.16E-01	5.74E-01
**TRAK1**	-0.07	5.16E-01	5.74E-01
**XLOC_002997**	-0.41	5.16E-01	5.74E-01
**RP1-101A2.1**	-0.19	5.16E-01	5.74E-01
**ERICH6-AS1**	-0.32	5.16E-01	5.74E-01
**LINC00885**	-0.22	5.16E-01	5.74E-01
**TVP23A**	-0.23	5.17E-01	5.75E-01
**RP11-415I12.3**	0.40	5.17E-01	5.75E-01
**CEP250**	-0.09	5.17E-01	5.75E-01
**TCTN3**	-0.06	5.17E-01	5.75E-01
**NANOGP9**	0.62	5.17E-01	5.75E-01
**AC018766.6**	-0.49	5.17E-01	5.76E-01
**DKFZP434A062**	-0.55	5.17E-01	5.76E-01
**BCRP3**	0.57	5.17E-01	5.76E-01
**DPYSL3**	0.21	5.17E-01	5.76E-01
**ADCY8**	0.98	5.18E-01	5.76E-01
**RP11-362F19.1**	-0.59	5.18E-01	5.76E-01
**RP11-74E22.8**	-0.22	5.18E-01	5.76E-01
**ARHGAP35**	0.07	5.18E-01	5.76E-01
**GS1-279B7.2**	0.43	5.18E-01	5.76E-01
**L3HYPDH**	0.15	5.18E-01	5.76E-01

**PRAME**	0.93	5.18E-01	5.76E-01
**TRIM69**	0.14	5.19E-01	5.77E-01
**RP11-184I16.3**	-0.85	5.19E-01	5.77E-01
**CLDN34**	-0.60	5.19E-01	5.77E-01
**LINC01353**	0.47	5.19E-01	5.77E-01
**XLOC_004988**	0.61	5.19E-01	5.77E-01
**LLPH-AS1**	0.34	5.20E-01	5.78E-01
**C3orf36**	0.46	5.20E-01	5.78E-01
**CDK3**	0.47	5.20E-01	5.78E-01
**RN7SL269P**	0.60	5.20E-01	5.78E-01
**RP11-106M3.2**	0.47	5.20E-01	5.78E-01
**SCIMP**	0.32	5.20E-01	5.78E-01
**CTD-2013N17.6**	0.33	5.20E-01	5.78E-01
**RP11-405M12.4**	0.68	5.20E-01	5.78E-01
**LINC01600**	-0.52	5.20E-01	5.79E-01
**ACTR10**	-0.09	5.20E-01	5.79E-01
**AKR1E2**	-0.18	5.21E-01	5.79E-01
**AC078899.4**	-0.79	5.21E-01	5.79E-01
**OR7D2**	0.87	5.21E-01	5.79E-01
**SERPINH1P1**	-0.62	5.21E-01	5.79E-01
**RP11-226L15.1**	-0.74	5.21E-01	5.79E-01
**CTC-204F22.1**	-0.29	5.21E-01	5.79E-01
**AC068669.1**	-0.87	5.21E-01	5.79E-01
**RP11-467D6.1**	-0.16	5.21E-01	5.79E-01
**EIF4A1**	-0.23	5.21E-01	5.79E-01
**G3016**	-0.55	5.22E-01	5.80E-01
**AGA**	0.13	5.22E-01	5.80E-01
**HSPE1P18**	-0.67	5.22E-01	5.80E-01
**GDI2**	-0.10	5.22E-01	5.80E-01
**SNAP23P**	-0.65	5.23E-01	5.81E-01
**PDP1**	0.09	5.23E-01	5.81E-01
**GHET1**	-0.25	5.23E-01	5.81E-01
**RNU6-204P**	-0.54	5.23E-01	5.81E-01
**RP11-1275H24.3**	0.16	5.23E-01	5.81E-01
**RNU1-122P**	-0.40	5.23E-01	5.81E-01
**G42780**	-0.69	5.24E-01	5.82E-01
**DUSP11**	-0.07	5.24E-01	5.82E-01
**RP11-17A4.2**	-0.87	5.24E-01	5.82E-01
**PANX1**	0.11	5.24E-01	5.82E-01
**MIR203**	-0.25	5.24E-01	5.82E-01
**IL2RB**	-0.20	5.24E-01	5.82E-01
**RP11-195F19.9**	0.20	5.24E-01	5.82E-01
**PPP1R3B**	-0.11	5.24E-01	5.82E-01
**G35913**	0.40	5.24E-01	5.82E-01
**C1orf100**	-0.72	5.25E-01	5.82E-01

**CTD-2306A12.1**	-0.55	5.25E-01	5.82E-01
**FOXN1**	0.16	5.25E-01	5.83E-01
**JMJD4**	0.10	5.25E-01	5.83E-01
**CTD-2621I17.3**	-0.33	5.25E-01	5.83E-01
**RP11-831H9.3**	-0.86	5.25E-01	5.83E-01
**XLOC_002951**	-0.47	5.25E-01	5.83E-01
**ANO2**	0.22	5.25E-01	5.83E-01
**BSCL2**	-0.18	5.26E-01	5.84E-01
**GS1-57L11.1**	0.57	5.26E-01	5.84E-01
**DPRXP4**	0.30	5.26E-01	5.84E-01
**XLOC_013461**	-0.27	5.26E-01	5.84E-01
**RHBDL3**	-0.23	5.26E-01	5.84E-01
**AC008063.2**	0.59	5.26E-01	5.84E-01
**SEMA3B-AS1**	0.47	5.26E-01	5.84E-01
**RP11-226L15.5**	-0.13	5.27E-01	5.85E-01
**AKR1C7P**	-0.69	5.27E-01	5.85E-01
**SLC9A7P1**	0.40	5.27E-01	5.85E-01
**RPS12**	0.12	5.27E-01	5.85E-01
**RP11-589C21.6**	0.71	5.27E-01	5.85E-01
**ESRRG**	0.34	5.27E-01	5.85E-01
**KCNK1**	-0.10	5.27E-01	5.85E-01
**TULP2**	0.44	5.28E-01	5.85E-01
**RP11-439A17.7**	-0.41	5.28E-01	5.85E-01
**RN7SL444P**	-0.53	5.28E-01	5.85E-01
**TRAPPC2P1**	0.12	5.28E-01	5.86E-01
**PHEX**	-0.21	5.28E-01	5.86E-01
**RP11-57H14.2**	-0.67	5.28E-01	5.86E-01
**RP11-59H7.4**	-0.48	5.29E-01	5.87E-01
**SPIB**	0.52	5.29E-01	5.87E-01
**PRKCE**	0.10	5.29E-01	5.87E-01
**RNF150**	0.21	5.29E-01	5.87E-01
**PDCL**	-0.09	5.29E-01	5.87E-01
**MS4A2**	-0.29	5.29E-01	5.87E-01
**KRT23**	-0.16	5.30E-01	5.88E-01
**COL6A6**	0.37	5.30E-01	5.88E-01
**CHP1**	-0.09	5.30E-01	5.88E-01
**RP11-483L5.1**	-0.24	5.30E-01	5.88E-01
**AC097523.1**	-0.45	5.30E-01	5.88E-01
**KLHL20**	-0.06	5.30E-01	5.88E-01
**RP11-834C11.7**	0.44	5.30E-01	5.88E-01
**C1orf127**	0.50	5.30E-01	5.88E-01
**RP4-545L17.11**	-0.53	5.30E-01	5.88E-01
**CACYBP**	0.10	5.30E-01	5.88E-01
**EARS2**	-0.08	5.31E-01	5.88E-01
**FTCDNL1**	-0.16	5.31E-01	5.88E-01

**JAKMIP1**	0.48	5.31E-01	5.88E-01
**EME2**	0.20	5.31E-01	5.88E-01
**TDRD9**	0.43	5.31E-01	5.88E-01
**RP11-736N17.10**	0.55	5.31E-01	5.88E-01
**COPS2**	-0.09	5.31E-01	5.88E-01
**ESRRB**	0.58	5.31E-01	5.89E-01
**CHRM3-AS2**	-0.77	5.31E-01	5.89E-01
**LINC01117**	-0.30	5.32E-01	5.89E-01
**SSPO**	-0.28	5.32E-01	5.89E-01
**RP11-360L9.4**	-0.55	5.32E-01	5.90E-01
**FBP1**	-0.48	5.32E-01	5.90E-01
**ATP6V1G2**	0.45	5.32E-01	5.90E-01
**RP11-132A1.4**	0.27	5.32E-01	5.90E-01
**RP11-64D24.4**	-0.61	5.33E-01	5.90E-01
**AC091153.4**	-0.61	5.33E-01	5.90E-01
**RP11-51F16.9**	0.26	5.33E-01	5.90E-01
**DNAJB9**	-0.12	5.33E-01	5.90E-01
**DNAJC22**	0.23	5.33E-01	5.90E-01
**XLOC_011798**	-0.50	5.33E-01	5.90E-01
**RPL21P122**	0.44	5.33E-01	5.90E-01
**ARSK**	-0.11	5.33E-01	5.90E-01
**RP11-435B5.5**	-0.39	5.33E-01	5.91E-01
**RP11-566K19.6**	0.57	5.33E-01	5.91E-01
**TTI1**	-0.07	5.34E-01	5.91E-01
**AC011625.1**	-0.85	5.34E-01	5.91E-01
**DLGAP5**	0.17	5.34E-01	5.91E-01
**FGD5P1**	0.23	5.34E-01	5.91E-01
**SLC16A11**	0.20	5.34E-01	5.92E-01
**GTSF1**	0.52	5.34E-01	5.92E-01
**LPIN1**	-0.24	5.34E-01	5.92E-01
**AL023583.1**	-0.84	5.34E-01	5.92E-01
**ACTC1**	-0.41	5.35E-01	5.92E-01
**TMEM234**	-0.13	5.35E-01	5.92E-01
**PSMD11**	0.07	5.35E-01	5.93E-01
**RPRM**	0.40	5.35E-01	5.93E-01
**CTD-2194D22.4**	-0.46	5.35E-01	5.93E-01
**C2CD4D**	-0.19	5.35E-01	5.93E-01
**GATA3-AS1**	-0.37	5.36E-01	5.93E-01
**RP11-323I15.3**	0.53	5.36E-01	5.93E-01
**IRAK3**	-0.20	5.36E-01	5.94E-01
**G35948**	-0.28	5.36E-01	5.94E-01
**G28051**	-0.48	5.36E-01	5.94E-01
**M1AP**	0.40	5.36E-01	5.94E-01
**RP11-93B14.10**	0.28	5.37E-01	5.94E-01
**RP11-720L2.2**	-0.45	5.37E-01	5.94E-01

**LHFPL3**	0.43	5.37E-01	5.94E-01
**PCDH19**	0.23	5.37E-01	5.94E-01
**MAP1LC3C**	-0.44	5.37E-01	5.95E-01
**AK4P1**	-0.43	5.37E-01	5.95E-01
**GPA33**	-0.49	5.37E-01	5.95E-01
**CAND2**	0.17	5.38E-01	5.95E-01
**SNORD88**	-0.61	5.38E-01	5.95E-01
**SETD3**	-0.07	5.38E-01	5.95E-01
**C8orf89**	-0.48	5.38E-01	5.95E-01
**TM4SF19**	0.47	5.38E-01	5.95E-01
**PPIAP19**	0.67	5.38E-01	5.96E-01
**SYTL3**	0.15	5.38E-01	5.96E-01
**RP11-68I3.10**	-0.65	5.38E-01	5.96E-01
**RP11-219E7.1**	-0.40	5.38E-01	5.96E-01
**G4330**	-0.30	5.39E-01	5.96E-01
**RP11-156K13.1**	0.48	5.39E-01	5.96E-01
**TRPS1**	-0.14	5.39E-01	5.96E-01
**AC114271.2**	-0.61	5.39E-01	5.96E-01
**LINC01123**	-0.30	5.39E-01	5.96E-01
**PAQR6**	0.25	5.39E-01	5.97E-01
**CLEC4A**	-0.21	5.39E-01	5.97E-01
**RNF5P1**	0.69	5.39E-01	5.97E-01
**DLL3**	0.86	5.40E-01	5.97E-01
**ZSWIM7**	-0.08	5.40E-01	5.97E-01
**RN7SL637P**	-0.45	5.40E-01	5.98E-01
**AC015849.16**	-0.63	5.40E-01	5.98E-01
**FANK1**	-0.13	5.40E-01	5.98E-01
**SPSB4**	-0.42	5.41E-01	5.98E-01
**AC004980.7**	-0.21	5.41E-01	5.98E-01
**FCHO1**	-0.12	5.41E-01	5.98E-01
**LYVE1**	0.30	5.41E-01	5.99E-01
**CTD-2145A24.4**	-0.59	5.41E-01	5.99E-01
**FAM149A**	-0.18	5.42E-01	5.99E-01
**ZSCAN12P1**	-0.23	5.42E-01	5.99E-01
**POLA2**	-0.07	5.42E-01	6.00E-01
**XYLB**	-0.16	5.43E-01	6.00E-01
**RP11-22B23.2**	-0.51	5.43E-01	6.00E-01
**KCTD9P2**	0.51	5.43E-01	6.00E-01
**RP3-500L14.2**	-0.31	5.43E-01	6.00E-01
**GLIPR1L2**	0.24	5.43E-01	6.00E-01
**TOP3A**	0.07	5.43E-01	6.00E-01
**SLC25A14**	0.09	5.43E-01	6.00E-01
**G24769**	-0.73	5.43E-01	6.01E-01
**SCT**	0.53	5.43E-01	6.01E-01
**ZNF30**	-0.12	5.43E-01	6.01E-01

**ACSM1**	-0.27	5.43E-01	6.01E-01
**G39215**	-0.23	5.44E-01	6.01E-01
**TAF1A-AS1**	-0.44	5.44E-01	6.01E-01
**RAB9A**	-0.10	5.44E-01	6.01E-01
**SULT1C2**	0.50	5.44E-01	6.01E-01
**RP11-848P1.2**	-0.19	5.44E-01	6.02E-01
**COX5BP6**	-0.30	5.44E-01	6.02E-01
**RP11-338K13.1**	0.49	5.45E-01	6.02E-01
**CTC-523E23.1**	0.24	5.45E-01	6.02E-01
**RRAGD**	-0.07	5.45E-01	6.02E-01
**RP11-561O23.9**	0.54	5.46E-01	6.03E-01
**SERBP1P1**	-0.38	5.46E-01	6.03E-01
**GTSE1-AS1**	0.21	5.46E-01	6.04E-01
**KDM4D**	-0.12	5.47E-01	6.04E-01
**TTC16**	-0.39	5.47E-01	6.04E-01
**ZNF826P**	0.20	5.47E-01	6.05E-01
**AC079776.1**	0.49	5.47E-01	6.05E-01
**CASC4**	0.12	5.47E-01	6.05E-01
**AC139530.1**	-0.46	5.48E-01	6.05E-01
**CABYR**	-0.17	5.48E-01	6.05E-01
**GS1-44D20.1**	-0.65	5.48E-01	6.05E-01
**FBXL22**	0.16	5.48E-01	6.05E-01
**IRF9**	0.24	5.48E-01	6.05E-01
**CCDC169**	-0.36	5.48E-01	6.05E-01
**ASIC4**	-0.34	5.48E-01	6.05E-01
**ADORA2B**	0.09	5.48E-01	6.06E-01
**CDK5RAP1**	0.06	5.48E-01	6.06E-01
**RP11-324D17.2**	0.65	5.49E-01	6.06E-01
**RP11-378A13.1**	-0.11	5.49E-01	6.06E-01
**RP11-533O20.2**	-0.54	5.49E-01	6.06E-01
**MAP3K13**	0.06	5.49E-01	6.06E-01
**IVNS1ABP**	0.08	5.49E-01	6.06E-01
**XLOC_007697**	-0.72	5.49E-01	6.06E-01
**GTF2H2**	-0.28	5.50E-01	6.07E-01
**RP5-890O3.9**	-0.19	5.50E-01	6.07E-01
**GPR83**	-0.48	5.50E-01	6.07E-01
**PPOX**	-0.14	5.50E-01	6.08E-01
**RP11-350G8.5**	0.32	5.50E-01	6.08E-01
**RNU6-242P**	-0.38	5.51E-01	6.08E-01
**AD000864.6**	-0.64	5.51E-01	6.08E-01
**CTC-497E21.3**	-0.23	5.51E-01	6.08E-01
**Z83851.4**	0.23	5.51E-01	6.08E-01
**RASL10A**	0.29	5.51E-01	6.09E-01
**HCG9**	-0.76	5.52E-01	6.09E-01
**CTD-2184D3.5**	-0.28	5.52E-01	6.09E-01

**RP11-51B23.3**	-0.71	5.52E-01	6.09E-01
**CCDC177**	0.58	5.52E-01	6.09E-01
**IGKV3-11**	1.02	5.52E-01	6.09E-01
**CMB9-55F22.1**	0.24	5.52E-01	6.09E-01
**FAM219B**	0.10	5.53E-01	6.10E-01
**RP11-563N4.1**	0.41	5.53E-01	6.10E-01
**AC006946.15**	-0.69	5.53E-01	6.10E-01
**RP4-758J18.13**	-0.17	5.53E-01	6.10E-01
**ARHGAP8**	-0.34	5.53E-01	6.10E-01
**XLOC_003400**	-0.56	5.53E-01	6.10E-01
**AC009950.2**	0.24	5.53E-01	6.10E-01
**RP11-360F5.1**	0.46	5.53E-01	6.10E-01
**GPR12**	0.44	5.53E-01	6.10E-01
**PRSS12**	-0.12	5.53E-01	6.10E-01
**XLOC_014103**	-0.18	5.53E-01	6.10E-01
**ITM2A**	0.21	5.53E-01	6.10E-01
**RP11-638I2.9**	-0.56	5.54E-01	6.11E-01
**G12993**	-0.32	5.54E-01	6.11E-01
**MZT2B**	-0.10	5.54E-01	6.11E-01
**LINC00319**	-0.46	5.54E-01	6.11E-01
**CD27**	-0.33	5.54E-01	6.11E-01
**AC019117.2**	0.37	5.54E-01	6.11E-01
**AC091729.7**	-0.30	5.54E-01	6.11E-01
**CTA-293F17.1**	-0.19	5.55E-01	6.11E-01
**ATP5E**	0.09	5.55E-01	6.11E-01
**SGCD**	-0.18	5.55E-01	6.12E-01
**G34396**	-0.61	5.55E-01	6.12E-01
**CTB-129P6.11**	-0.42	5.55E-01	6.12E-01
**G33828**	0.40	5.55E-01	6.12E-01
**RP11-855A2.3**	-0.29	5.55E-01	6.12E-01
**TBRG1**	0.08	5.55E-01	6.12E-01
**TESPA1**	-0.26	5.55E-01	6.12E-01
**SLC2A4**	0.23	5.55E-01	6.12E-01
**PLS3**	-0.09	5.55E-01	6.12E-01
**IDO1**	0.35	5.55E-01	6.12E-01
**FAM86DP**	0.24	5.55E-01	6.12E-01
**ITSN1**	0.11	5.55E-01	6.12E-01
**IGLC3**	0.90	5.56E-01	6.12E-01
**G19572**	0.62	5.56E-01	6.12E-01
**STAU2**	-0.08	5.56E-01	6.12E-01
**LINC01410**	0.29	5.56E-01	6.13E-01
**AC138393.1**	0.53	5.56E-01	6.13E-01
**TNFSF15**	-0.17	5.56E-01	6.13E-01
**STK40**	-0.08	5.56E-01	6.13E-01
**RP11-674P19.2**	0.69	5.56E-01	6.13E-01

**TNFRSF11A**	-0.11	5.57E-01	6.13E-01
**TG**	-0.23	5.57E-01	6.14E-01
**RAC1P2**	0.18	5.57E-01	6.14E-01
**PSPC1P1**	-0.61	5.57E-01	6.14E-01
**CTD-2514C3.1**	0.38	5.57E-01	6.14E-01
**ERICH2**	0.34	5.57E-01	6.14E-01
**ADD2**	-0.20	5.58E-01	6.15E-01
**RBMS2P1**	0.42	5.58E-01	6.15E-01
**RP11-110L15.2**	-0.70	5.58E-01	6.15E-01
**PEBP4**	0.41	5.58E-01	6.15E-01
**RP11-265E18.1**	-0.71	5.58E-01	6.15E-01
**G14979**	-0.39	5.58E-01	6.15E-01
**LINC00324**	0.10	5.58E-01	6.15E-01
**PPP2R2D**	0.04	5.59E-01	6.15E-01
**RP11-154F14.2**	-0.44	5.59E-01	6.15E-01
**GRB14**	0.41	5.59E-01	6.16E-01
**FAM86B2**	-0.45	5.60E-01	6.17E-01
**MFSD6L**	-0.31	5.60E-01	6.17E-01
**LINC01133**	-0.12	5.61E-01	6.17E-01
**AC084219.4**	-0.51	5.61E-01	6.17E-01
**AC098823.3**	-0.39	5.61E-01	6.17E-01
**HMGN1P15**	0.48	5.61E-01	6.17E-01
**RP11-351J23.1**	0.78	5.61E-01	6.17E-01
**RP11-548P2.2**	-0.45	5.61E-01	6.18E-01
**RP4-568B10.1**	-0.73	5.61E-01	6.18E-01
**RP11-66N24.3**	-0.32	5.62E-01	6.18E-01
**PEX2**	-0.07	5.62E-01	6.18E-01
**CTD-3138B18.5**	-0.14	5.62E-01	6.18E-01
**AC013444.1**	-0.74	5.62E-01	6.19E-01
**ZFHX3**	0.11	5.62E-01	6.19E-01
**POC5**	0.07	5.62E-01	6.19E-01
**ENOX1-AS1**	-0.57	5.62E-01	6.19E-01
**3-Mar**	-0.12	5.62E-01	6.19E-01
**AC008073.7**	-0.55	5.62E-01	6.19E-01
**TMSB4Y**	-0.14	5.62E-01	6.19E-01
**PDPR**	0.13	5.63E-01	6.19E-01
**G30963**	0.58	5.63E-01	6.19E-01
**RPL21P135**	-0.37	5.63E-01	6.19E-01
**RP11-265N7.1**	0.49	5.63E-01	6.20E-01
**DNAAF3**	0.24	5.63E-01	6.20E-01
**HNRNPLL**	-0.07	5.63E-01	6.20E-01
**GMEB1**	-0.06	5.63E-01	6.20E-01
**ELN**	0.25	5.63E-01	6.20E-01
**HSPB11**	0.07	5.64E-01	6.20E-01
**RP11-23J18.1**	0.61	5.64E-01	6.20E-01

**EIF4A1P10**	-0.23	5.64E-01	6.20E-01
**RP11-90P13.1**	0.97	5.64E-01	6.20E-01
**HR**	0.14	5.64E-01	6.21E-01
**IGHV3-9**	0.89	5.64E-01	6.21E-01
**ZFP2**	-0.13	5.64E-01	6.21E-01
**DDR2**	-0.21	5.65E-01	6.21E-01
**CTC-260F20.3**	0.54	5.65E-01	6.21E-01
**C3orf70**	-0.11	5.65E-01	6.22E-01
**RP11-94C24.6**	0.35	5.66E-01	6.22E-01
**CTC-471J1.9**	-0.46	5.66E-01	6.22E-01
**RPS28P7**	0.29	5.66E-01	6.22E-01
**RP3-414A15.11**	-0.38	5.66E-01	6.22E-01
**UBE2T**	0.12	5.66E-01	6.22E-01
**HHIPL2**	-0.66	5.66E-01	6.22E-01
**HPGD**	0.18	5.66E-01	6.22E-01
**LIPJ**	-0.24	5.66E-01	6.22E-01
**G7052**	0.51	5.66E-01	6.22E-01
**PIGM**	-0.05	5.66E-01	6.22E-01
**POLG2**	0.13	5.66E-01	6.22E-01
**XIRP2**	-0.60	5.66E-01	6.22E-01
**PCDHGA1**	0.22	5.66E-01	6.22E-01
**BNIP3P1**	0.47	5.66E-01	6.22E-01
**RP11-539L10.3**	-0.28	5.66E-01	6.22E-01
**ABCB5**	-0.37	5.66E-01	6.22E-01
**CNTNAP3P1**	-0.61	5.66E-01	6.23E-01
**AP001925.1**	-0.55	5.67E-01	6.23E-01
**RP11-196G11.2**	-0.36	5.67E-01	6.23E-01
**STX12**	0.08	5.67E-01	6.23E-01
**OR7E28P**	-0.42	5.67E-01	6.23E-01
**TMEM159**	-0.09	5.67E-01	6.23E-01
**CLMN**	-0.16	5.67E-01	6.23E-01
**COL4A2-AS2**	-0.68	5.67E-01	6.23E-01
**CYP2D7**	0.26	5.67E-01	6.24E-01
**NAP1L6**	-0.42	5.67E-01	6.24E-01
**SNX29P2**	1.02	5.68E-01	6.24E-01
**AC032027.1**	-0.47	5.68E-01	6.24E-01
**RN7SL452P**	-0.53	5.68E-01	6.24E-01
**RAET1E**	0.15	5.68E-01	6.24E-01
**ECHDC2**	0.16	5.68E-01	6.24E-01
**CLEC2D**	-0.19	5.68E-01	6.24E-01
**CTC-1337H24.4**	0.39	5.68E-01	6.24E-01
**CTD-2081C10.7**	-0.26	5.68E-01	6.24E-01
**PRIMA1**	-0.17	5.69E-01	6.25E-01
**G20694**	-0.36	5.69E-01	6.25E-01
**IQCH-AS1**	-0.11	5.69E-01	6.25E-01

**RP11-762L8.6**	0.31	5.69E-01	6.25E-01
**ZNF792**	0.11	5.69E-01	6.25E-01
**EPHB3**	0.10	5.70E-01	6.26E-01
**G26738**	-0.55	5.70E-01	6.26E-01
**RP11-307B6.3**	-0.57	5.70E-01	6.26E-01
**ABHD17B**	-0.12	5.70E-01	6.26E-01
**AC133785.1**	0.24	5.70E-01	6.26E-01
**RP11-732A19.6**	-0.76	5.70E-01	6.26E-01
**CTD-2331H12.5**	-0.22	5.70E-01	6.26E-01
**RP5-1171I10.5**	0.31	5.70E-01	6.26E-01
**CALML6**	-0.42	5.71E-01	6.26E-01
**LRRC37A3**	0.17	5.71E-01	6.26E-01
**CHCHD2P9**	-0.36	5.71E-01	6.26E-01
**MAST1**	-0.15	5.71E-01	6.27E-01
**RPL23A**	-0.10	5.71E-01	6.27E-01
**BTBD11**	-0.09	5.71E-01	6.27E-01
**G42800**	0.49	5.71E-01	6.27E-01
**B3GALT1**	-0.51	5.71E-01	6.27E-01
**G5901**	-0.33	5.71E-01	6.27E-01
**RP11-572M11.4**	0.37	5.71E-01	6.27E-01
**RP11-635L1.2**	-0.29	5.71E-01	6.27E-01
**AC005498.3**	-0.37	5.71E-01	6.27E-01
**BNIPL**	-0.11	5.72E-01	6.27E-01
**PMFBP1**	-0.27	5.72E-01	6.27E-01
**UBE3B**	-0.05	5.72E-01	6.27E-01
**F2R**	0.11	5.72E-01	6.27E-01
**CACNA1C-AS2**	-0.54	5.72E-01	6.27E-01
**ANKRA2**	-0.07	5.72E-01	6.28E-01
**KRT8P15**	-0.49	5.72E-01	6.28E-01
**AAK1**	0.09	5.72E-01	6.28E-01
**RP11-111E14.1**	0.25	5.72E-01	6.28E-01
**CTB-25B13.5**	-0.17	5.73E-01	6.28E-01
**BEGAIN**	0.17	5.73E-01	6.29E-01
**RP11-270M14.1**	0.49	5.73E-01	6.29E-01
**AC004471.10**	0.38	5.73E-01	6.29E-01
**UCP3**	-0.21	5.74E-01	6.29E-01
**LINC01320**	-0.53	5.74E-01	6.29E-01
**NEBL-AS1**	0.20	5.74E-01	6.29E-01
**QRICH1**	0.05	5.74E-01	6.29E-01
**XLOC_013968**	-0.33	5.74E-01	6.29E-01
**G5385**	0.40	5.74E-01	6.30E-01
**A2ML1**	0.18	5.74E-01	6.30E-01
**RGAG1**	-0.53	5.74E-01	6.30E-01
**GADL1**	-0.64	5.74E-01	6.30E-01
**SCGB2A2**	0.83	5.74E-01	6.30E-01

**OR2W3**	0.50	5.75E-01	6.30E-01
**KCNQ5**	0.31	5.75E-01	6.30E-01
**UNG**	0.10	5.75E-01	6.30E-01
**RP11-356B19.11**	-0.26	5.75E-01	6.30E-01
**FAM182A**	0.24	5.75E-01	6.31E-01
**RP11-379B8.1**	-0.33	5.75E-01	6.31E-01
**TMEM218**	-0.10	5.75E-01	6.31E-01
**NAT2**	-0.71	5.76E-01	6.31E-01
**INHBC**	0.55	5.76E-01	6.31E-01
**EZH2**	0.09	5.76E-01	6.32E-01
**CASP1**	0.11	5.76E-01	6.32E-01
**TMEM39A**	0.06	5.77E-01	6.32E-01
**ANKRD20A10P**	-0.68	5.77E-01	6.32E-01
**G356**	-0.42	5.77E-01	6.32E-01
**CCDC189**	0.43	5.77E-01	6.32E-01
**ACCS**	-0.19	5.77E-01	6.32E-01
**RP11-927P21.8**	0.62	5.77E-01	6.32E-01
**RP11-1000B6.3**	-0.26	5.77E-01	6.33E-01
**RP11-68I3.4**	-0.43	5.77E-01	6.33E-01
**AC000403.4**	0.21	5.77E-01	6.33E-01
**G26459**	0.24	5.78E-01	6.33E-01
**HERC2P5**	0.72	5.78E-01	6.34E-01
**ROPN1**	0.41	5.78E-01	6.34E-01
**TRERF1**	0.07	5.78E-01	6.34E-01
**RP11-62H7.2**	0.48	5.79E-01	6.34E-01
**IL27**	0.60	5.79E-01	6.34E-01
**B3GALNT1P1**	-0.31	5.79E-01	6.34E-01
**C11orf91**	0.39	5.79E-01	6.34E-01
**PTPRD**	-0.25	5.79E-01	6.34E-01
**RN7SL695P**	0.52	5.79E-01	6.34E-01
**RP4-647C14.2**	0.16	5.79E-01	6.34E-01
**PI4KA**	0.08	5.79E-01	6.35E-01
**HCG16**	0.53	5.79E-01	6.35E-01
**VWC2**	-0.21	5.79E-01	6.35E-01
**MFSD2B**	0.25	5.80E-01	6.35E-01
**AC010492.2**	0.64	5.80E-01	6.35E-01
**ZFP90**	0.07	5.80E-01	6.36E-01
**RP11-488L18.10**	-0.15	5.81E-01	6.36E-01
**MST1P2**	0.27	5.81E-01	6.36E-01
**CHD4**	0.05	5.81E-01	6.36E-01
**RP11-307E17.8**	-0.37	5.81E-01	6.37E-01
**ADAD2**	-0.21	5.81E-01	6.37E-01
**LRRC6**	0.12	5.82E-01	6.37E-01
**TNN**	0.41	5.82E-01	6.37E-01
**HEPACAM2**	0.52	5.82E-01	6.38E-01

**G10706**	-0.55	5.82E-01	6.38E-01
**HOXD3**	0.27	5.83E-01	6.38E-01
**ERO1A**	0.10	5.83E-01	6.38E-01
**LL22NC03-2H8.4**	0.41	5.83E-01	6.38E-01
**LUCAT1**	0.29	5.83E-01	6.38E-01
**GOLGA5**	0.08	5.83E-01	6.39E-01
**METTL21A**	0.11	5.83E-01	6.39E-01
**RP11-495P10.9**	0.51	5.84E-01	6.39E-01
**LINC00936**	0.13	5.84E-01	6.40E-01
**ALKBH5**	0.06	5.85E-01	6.40E-01
**CTD-2562J17.6**	0.11	5.85E-01	6.40E-01
**B2M**	0.12	5.85E-01	6.40E-01
**RP4-635E18.6**	0.45	5.85E-01	6.40E-01
**CBX3P7**	0.39	5.85E-01	6.40E-01
**G26894**	-0.40	5.85E-01	6.41E-01
**G5756**	0.59	5.85E-01	6.41E-01
**MICE**	-0.29	5.85E-01	6.41E-01
**MIR5695**	0.57	5.86E-01	6.41E-01
**NUMA1**	0.06	5.86E-01	6.41E-01
**RP5-1021I20.1**	0.24	5.86E-01	6.41E-01
**RP11-1035H13.2**	-0.57	5.86E-01	6.41E-01
**RN7SL417P**	-0.74	5.86E-01	6.41E-01
**AQP8**	0.52	5.86E-01	6.41E-01
**AMHR2**	0.76	5.86E-01	6.41E-01
**TCEB1P21**	-0.74	5.87E-01	6.42E-01
**RP11-706O15.7**	0.92	5.87E-01	6.42E-01
**EME1**	-0.18	5.87E-01	6.42E-01
**PLA2G2F**	0.15	5.88E-01	6.43E-01
**RBM47**	0.10	5.88E-01	6.43E-01
**UVRAG**	0.05	5.88E-01	6.43E-01
**RP11-750H9.7**	0.36	5.88E-01	6.43E-01
**LINC01124**	-0.22	5.88E-01	6.43E-01
**KRT3**	0.35	5.88E-01	6.43E-01
**RP11-483E17.1**	-0.76	5.88E-01	6.43E-01
**RP11-83N9.6**	-0.29	5.88E-01	6.43E-01
**SLC25A53**	0.11	5.88E-01	6.43E-01
**SLC14A2**	0.29	5.88E-01	6.43E-01
**G24400**	0.27	5.88E-01	6.43E-01
**PRKAR2B**	0.16	5.88E-01	6.43E-01
**RP11-96C23.12**	0.22	5.88E-01	6.43E-01
**CTD-2517M22.14**	0.30	5.89E-01	6.44E-01
**AC002044.1**	0.36	5.89E-01	6.44E-01
**RP11-550I24.2**	-0.26	5.89E-01	6.44E-01
**TCEAL2**	0.35	5.89E-01	6.44E-01
**RP11-398C13.6**	-0.16	5.89E-01	6.44E-01

**RPL21P119**	-0.35	5.89E-01	6.44E-01
**SLC17A5**	-0.08	5.89E-01	6.44E-01
**RP5-1157M23.2**	0.31	5.89E-01	6.44E-01
**SCN1A**	0.66	5.90E-01	6.44E-01
**AC079210.1**	-0.28	5.90E-01	6.44E-01
**CTC-441N14.4**	-0.68	5.90E-01	6.44E-01
**G30718**	-0.82	5.90E-01	6.44E-01
**PRSS53**	-0.26	5.90E-01	6.45E-01
**RP11-1029J19.5**	0.50	5.90E-01	6.45E-01
**TVP23B**	0.09	5.90E-01	6.45E-01
**FTCD**	0.47	5.90E-01	6.45E-01
**RP11-475I24.3**	-0.23	5.90E-01	6.45E-01
**RN7SL200P**	-0.42	5.91E-01	6.45E-01
**PLA2G12AP1**	0.29	5.91E-01	6.46E-01
**CFB**	0.25	5.91E-01	6.46E-01
**G19624**	-0.42	5.91E-01	6.46E-01
**GATA6-AS1**	0.59	5.91E-01	6.46E-01
**MR1**	0.06	5.92E-01	6.46E-01
**TMEM5**	0.07	5.92E-01	6.46E-01
**RP11-677M14.5**	0.32	5.92E-01	6.46E-01
**DCP1A**	-0.05	5.92E-01	6.47E-01
**LINC01001**	-0.33	5.92E-01	6.47E-01
**CD40LG**	0.31	5.92E-01	6.47E-01
**MIR4668**	-0.53	5.92E-01	6.47E-01
**RP11-422P24.11**	-0.16	5.92E-01	6.47E-01
**AC002456.2**	0.51	5.93E-01	6.47E-01
**TAMM41**	-0.11	5.93E-01	6.47E-01
**NDUFA2**	0.09	5.93E-01	6.47E-01
**AC073133.1**	-0.67	5.93E-01	6.47E-01
**GPR89A**	-0.10	5.93E-01	6.47E-01
**SBSPON**	0.16	5.93E-01	6.47E-01
**AKNAD1**	-0.19	5.93E-01	6.48E-01
**RP13-638C3.6**	0.30	5.93E-01	6.48E-01
**RPL18AP7**	-0.41	5.93E-01	6.48E-01
**AMTN**	0.59	5.93E-01	6.48E-01
**TFAP2B**	-0.11	5.93E-01	6.48E-01
**GPR132**	-0.19	5.94E-01	6.48E-01
**CTD-2207P18.1**	-0.58	5.94E-01	6.48E-01
**RP11-155D18.13**	0.31	5.94E-01	6.48E-01
**KRT18P61**	-0.33	5.94E-01	6.49E-01
**RP11-445P17.8**	-0.58	5.94E-01	6.49E-01
**AP001372.2**	0.09	5.95E-01	6.49E-01
**YBX2**	-0.24	5.95E-01	6.49E-01
**RP11-390P24.1**	0.17	5.95E-01	6.49E-01
**RP1-120G22.11**	0.52	5.95E-01	6.50E-01

**RP11-539G18.3**	0.41	5.96E-01	6.50E-01
**RP11-598D12.4**	-0.86	5.96E-01	6.50E-01
**RP11-321G12.1**	-0.35	5.96E-01	6.50E-01
**ACBD3**	-0.07	5.96E-01	6.50E-01
**RP11-1007O24.2**	-0.28	5.96E-01	6.50E-01
**TSHZ2**	0.12	5.96E-01	6.50E-01
**RP11-254F19.4**	-0.53	5.96E-01	6.51E-01
**RP3-483K16.4**	-0.56	5.96E-01	6.51E-01
**MRPL38**	0.18	5.96E-01	6.51E-01
**PDIA3P2**	-0.36	5.97E-01	6.51E-01
**BEST1**	0.16	5.97E-01	6.51E-01
**SMIM4**	0.07	5.97E-01	6.51E-01
**G43078**	0.48	5.97E-01	6.51E-01
**TCHH**	-0.70	5.97E-01	6.51E-01
**CTD-2020K17.3**	0.54	5.97E-01	6.51E-01
**POGZ**	0.08	5.97E-01	6.51E-01
**G40185**	-0.66	5.97E-01	6.52E-01
**CCDC158**	0.29	5.97E-01	6.52E-01
**RP11-395C3.1**	-0.31	5.98E-01	6.52E-01
**VPS29**	0.07	5.98E-01	6.52E-01
**RP11-399B17.1**	0.20	5.98E-01	6.52E-01
**CTD-2062F14.3**	-0.65	5.98E-01	6.52E-01
**RP11-39H3.2**	-0.62	5.98E-01	6.53E-01
**CTD-2017C7.2**	0.20	5.99E-01	6.53E-01
**RP11-524F11.1**	-0.32	5.99E-01	6.53E-01
**AC003986.5**	0.60	5.99E-01	6.53E-01
**RP11-30K9.5**	-0.52	5.99E-01	6.53E-01
**RP11-543C4.1**	-0.21	5.99E-01	6.53E-01
**ARSI**	0.16	5.99E-01	6.53E-01
**RP11-128P10.1**	-0.54	5.99E-01	6.54E-01
**ANXA13**	0.32	6.00E-01	6.54E-01
**RP11-6N17.2**	-0.49	6.00E-01	6.54E-01
**7-Sep**	0.08	6.00E-01	6.54E-01
**SUCLA2-AS1**	-0.35	6.00E-01	6.54E-01
**VPS4A**	0.05	6.00E-01	6.54E-01
**RP11-170K4.2**	-0.26	6.00E-01	6.54E-01
**FAM25C**	-0.35	6.00E-01	6.54E-01
**KIF5A**	0.34	6.00E-01	6.55E-01
**RP11-549J18.1**	-0.16	6.01E-01	6.55E-01
**CCDC26**	-0.67	6.01E-01	6.55E-01
**AF001548.6**	-0.69	6.01E-01	6.55E-01
**PPP2R3C**	0.07	6.01E-01	6.55E-01
**LINC01234**	-0.68	6.01E-01	6.55E-01
**KLHL40**	-0.55	6.02E-01	6.56E-01
**AGTR1**	-0.27	6.02E-01	6.56E-01

**TRMT1L**	0.07	6.02E-01	6.56E-01
**MYO5C**	-0.16	6.02E-01	6.56E-01
**CTD-2083E4.6**	0.32	6.02E-01	6.56E-01
**10-Mar**	-0.48	6.02E-01	6.56E-01
**RP11-1250I15.3**	0.29	6.02E-01	6.56E-01
**SAMD3**	0.24	6.03E-01	6.57E-01
**WWC1**	-0.09	6.03E-01	6.57E-01
**RP5-968P14.2**	0.16	6.03E-01	6.57E-01
**CRISP3**	0.60	6.03E-01	6.57E-01
**NEDD4L**	0.14	6.03E-01	6.57E-01
**FOXO6**	0.17	6.03E-01	6.57E-01
**LINC00665**	0.10	6.03E-01	6.57E-01
**AC128709.3**	-0.43	6.03E-01	6.57E-01
**RP11-707G14.8**	-0.49	6.03E-01	6.57E-01
**NLRC3**	-0.13	6.04E-01	6.57E-01
**LINC00964**	0.21	6.04E-01	6.57E-01
**TMED2**	-0.07	6.04E-01	6.58E-01
**AAMDC**	-0.10	6.04E-01	6.58E-01
**ING5**	0.08	6.04E-01	6.58E-01
**INO80C**	-0.07	6.04E-01	6.58E-01
**CDKN1B**	0.07	6.04E-01	6.58E-01
**XPNPEP2**	0.26	6.04E-01	6.58E-01
**AC025165.8**	0.46	6.04E-01	6.58E-01
**AL353671.4**	0.30	6.05E-01	6.59E-01
**PTOV1-AS2**	-0.24	6.05E-01	6.59E-01
**ALS2CL**	-0.16	6.05E-01	6.59E-01
**DNM1P46**	0.32	6.05E-01	6.59E-01
**RN7SL130P**	-0.28	6.05E-01	6.59E-01
**G5173**	-0.57	6.05E-01	6.59E-01
**MDFIC**	0.10	6.05E-01	6.59E-01
**LINC00243**	0.30	6.05E-01	6.59E-01
**GJC3**	-0.20	6.06E-01	6.60E-01
**RNU6-353P**	-0.37	6.06E-01	6.60E-01
**RP5-1024N4.5**	-0.46	6.06E-01	6.60E-01
**CHCHD7**	0.07	6.06E-01	6.60E-01
**RP3-406A7.7**	-0.17	6.06E-01	6.60E-01
**RP4-616B8.5**	-0.30	6.06E-01	6.60E-01
**KIZ**	0.07	6.06E-01	6.60E-01
**RN7SL409P**	-0.50	6.07E-01	6.60E-01
**FOXD3**	-0.28	6.07E-01	6.60E-01
**KRT81**	-0.84	6.07E-01	6.61E-01
**CYP4F23P**	-0.39	6.07E-01	6.61E-01
**RP11-762B21.5**	-0.68	6.07E-01	6.61E-01
**TRGC1**	0.38	6.08E-01	6.61E-01
**TTLL5**	0.06	6.08E-01	6.62E-01

**ZNF660**	-0.17	6.08E-01	6.62E-01
**RP11-862P13.1**	0.31	6.08E-01	6.62E-01
**AC011475.1**	-0.47	6.08E-01	6.62E-01
**HSPA9**	0.07	6.08E-01	6.62E-01
**RP11-298I3.4**	-0.18	6.09E-01	6.62E-01
**GK-IT1**	0.41	6.09E-01	6.62E-01
**FAM117A**	-0.10	6.09E-01	6.62E-01
**NFS1**	-0.06	6.09E-01	6.62E-01
**RP11-321A17.6**	0.19	6.09E-01	6.62E-01
**FAM217B**	0.06	6.09E-01	6.62E-01
**LINC01412**	0.50	6.09E-01	6.62E-01
**RP11-1072A3.3**	0.40	6.09E-01	6.63E-01
**TNFAIP8L3**	-0.10	6.09E-01	6.63E-01
**G33346**	0.57	6.10E-01	6.64E-01
**C17orf51**	0.13	6.10E-01	6.64E-01
**DLX6-AS1**	-0.17	6.10E-01	6.64E-01
**RP3-355L5.5**	-0.24	6.11E-01	6.64E-01
**LL22NC03-75H12.2**	0.48	6.11E-01	6.64E-01
**RP11-15E18.1**	-0.34	6.11E-01	6.64E-01
**GATSL3**	-0.22	6.11E-01	6.64E-01
**G1863**	-0.21	6.11E-01	6.64E-01
**RP11-262A16.1**	0.28	6.11E-01	6.65E-01
**TPM3P9**	-0.12	6.11E-01	6.65E-01
**MAP2K4P1**	-0.42	6.12E-01	6.65E-01
**SUCLG2**	-0.08	6.12E-01	6.65E-01
**OR7E126P**	0.27	6.12E-01	6.66E-01
**BX255923.1**	-0.40	6.12E-01	6.66E-01
**CTC-350I8.1**	-0.40	6.12E-01	6.66E-01
**RP13-766D20.2**	-0.23	6.13E-01	6.66E-01
**HIF3A**	-0.22	6.13E-01	6.66E-01
**PIK3R2**	0.46	6.13E-01	6.66E-01
**RP11-214F16.8**	0.50	6.13E-01	6.66E-01
**SLED1**	-0.20	6.13E-01	6.66E-01
**ZNF785**	0.09	6.13E-01	6.67E-01
**MIR4458HG**	0.13	6.13E-01	6.67E-01
**GOLGA6A**	-0.60	6.13E-01	6.67E-01
**METTL16**	0.05	6.13E-01	6.67E-01
**RP1-308E4.1**	-0.31	6.14E-01	6.67E-01
**RP11-104L21.2**	-0.23	6.14E-01	6.67E-01
**SLC25A21-AS1**	-0.18	6.14E-01	6.67E-01
**PRPF39**	-0.09	6.14E-01	6.67E-01
**CRIM1**	0.07	6.14E-01	6.67E-01
**RP11-1038A11.3**	0.43	6.14E-01	6.67E-01
**XLOC_012894**	-0.12	6.14E-01	6.67E-01
**CFAP74**	0.42	6.14E-01	6.68E-01

**RDH10**	-0.07	6.14E-01	6.68E-01
**STAG3L3**	0.13	6.15E-01	6.68E-01
**DYNC1I2P1**	-0.21	6.15E-01	6.68E-01
**AC012358.4**	0.18	6.15E-01	6.68E-01
**ARV1**	0.07	6.15E-01	6.68E-01
**CBWD2**	0.05	6.15E-01	6.68E-01
**RP11-64K12.8**	-0.52	6.15E-01	6.69E-01
**NAP1L3**	-0.15	6.16E-01	6.69E-01
**GSR**	0.08	6.16E-01	6.69E-01
**C14orf166**	-0.07	6.16E-01	6.69E-01
**RNF148**	-0.42	6.16E-01	6.69E-01
**RP11-442N24__B.1**	-0.23	6.16E-01	6.69E-01
**AC068620.1**	-0.37	6.16E-01	6.69E-01
**XLOC_001025**	-0.33	6.16E-01	6.69E-01
**RP11-467K18.2**	-0.53	6.16E-01	6.69E-01
**AC016292.3**	0.31	6.17E-01	6.70E-01
**AGAP10**	-0.50	6.17E-01	6.70E-01
**CTD-2540B15.12**	-0.61	6.17E-01	6.70E-01
**IGHG2**	0.56	6.17E-01	6.70E-01
**DPP9-AS1**	-0.26	6.17E-01	6.70E-01
**DEPDC1B**	-0.11	6.17E-01	6.70E-01
**MYL5**	0.13	6.18E-01	6.70E-01
**AC090587.4**	-0.17	6.18E-01	6.71E-01
**HERC2P2**	-0.20	6.18E-01	6.71E-01
**EAF1**	-0.07	6.18E-01	6.71E-01
**OSR1**	-0.20	6.18E-01	6.71E-01
**RPL26P30**	0.43	6.18E-01	6.71E-01
**YPEL1**	0.17	6.19E-01	6.71E-01
**RP11-313P22.1**	-0.37	6.19E-01	6.71E-01
**AUH**	0.06	6.19E-01	6.71E-01
**SART3**	-0.05	6.19E-01	6.72E-01
**AC226118.1**	0.39	6.19E-01	6.72E-01
**RP1-232L22__B.1**	-0.32	6.19E-01	6.72E-01
**APIP**	-0.09	6.19E-01	6.72E-01
**SDHDP6**	0.46	6.19E-01	6.72E-01
**STYK1**	0.11	6.19E-01	6.72E-01
**TP53AIP1**	-0.11	6.20E-01	6.72E-01
**ACBD7**	0.25	6.20E-01	6.72E-01
**CDRT15**	-0.43	6.20E-01	6.72E-01
**CLEC12A**	0.40	6.20E-01	6.73E-01
**CTD-2005H7.1**	0.61	6.20E-01	6.73E-01
**ZXDC**	-0.06	6.20E-01	6.73E-01
**CCNF**	-0.09	6.20E-01	6.73E-01
**FLJ12825**	-0.23	6.21E-01	6.73E-01
**DDX11L2**	0.20	6.21E-01	6.73E-01

**UPF3BP1**	0.30	6.21E-01	6.73E-01
**CPSF1P1**	0.37	6.21E-01	6.73E-01
**CPNE7**	0.26	6.21E-01	6.74E-01
**KCTD15**	0.06	6.21E-01	6.74E-01
**RP11-92C4.3**	0.51	6.22E-01	6.74E-01
**AP002495.1**	-0.47	6.22E-01	6.74E-01
**SNORA70B**	-0.55	6.22E-01	6.74E-01
**RP11-287D1.4**	-0.44	6.22E-01	6.74E-01
**DGUOK-AS1**	-0.11	6.22E-01	6.74E-01
**PPP5D1**	0.16	6.22E-01	6.74E-01
**G9812**	-0.48	6.22E-01	6.75E-01
**LINC00926**	0.21	6.22E-01	6.75E-01
**RP11-15A1.7**	0.29	6.23E-01	6.75E-01
**XLOC_002344**	0.35	6.23E-01	6.75E-01
**ZNF438**	0.06	6.23E-01	6.75E-01
**KLK11**	-0.09	6.23E-01	6.76E-01
**RPA3**	-0.11	6.23E-01	6.76E-01
**MNDA**	0.17	6.23E-01	6.76E-01
**XXbac-BPG181B23.7**	0.25	6.23E-01	6.76E-01
**RP5-999L4.2**	-0.51	6.24E-01	6.76E-01
**FER1L4**	0.26	6.24E-01	6.76E-01
**NDUFB2-AS1**	-0.26	6.24E-01	6.76E-01
**C8orf88**	0.12	6.24E-01	6.76E-01
**PRDX3P2**	0.36	6.24E-01	6.76E-01
**TFB2M**	0.07	6.24E-01	6.77E-01
**LAMTOR5**	-0.08	6.24E-01	6.77E-01
**RNF183**	-0.40	6.24E-01	6.77E-01
**RP11-62F24.2**	-0.21	6.24E-01	6.77E-01
**CTD-2194D22.3**	-0.19	6.25E-01	6.77E-01
**ACER1**	-0.11	6.25E-01	6.77E-01
**DMGDH**	0.13	6.25E-01	6.77E-01
**RP11-661A12.5**	-0.63	6.25E-01	6.77E-01
**COLEC12**	0.15	6.25E-01	6.77E-01
**CLYBL**	0.10	6.25E-01	6.78E-01
**SLC9A3**	0.22	6.25E-01	6.78E-01
**C1orf226**	-0.15	6.26E-01	6.78E-01
**LINC00662**	-0.08	6.26E-01	6.78E-01
**RP1-267D11.6**	0.10	6.26E-01	6.78E-01
**GNG10**	0.39	6.26E-01	6.78E-01
**GRTP1**	-0.09	6.26E-01	6.78E-01
**RP11-596C23.6**	-0.17	6.26E-01	6.78E-01
**FAM86B1**	-0.24	6.27E-01	6.79E-01
**CTD-2639E6.9**	0.17	6.27E-01	6.79E-01
**RANBP9**	-0.07	6.27E-01	6.79E-01
**DERL2**	-0.05	6.27E-01	6.79E-01

**ASIP**	0.25	6.27E-01	6.79E-01
**KCNB2**	-0.55	6.27E-01	6.80E-01
**RP11-861E21.3**	0.43	6.27E-01	6.80E-01
**CLEC10A**	-0.21	6.28E-01	6.80E-01
**ANKEF1**	0.09	6.28E-01	6.80E-01
**AC131097.4**	-0.23	6.28E-01	6.80E-01
**RP11-3K16.2**	-0.54	6.28E-01	6.80E-01
**RP11-779O18.1**	0.65	6.28E-01	6.80E-01
**RP11-378E13.3**	-0.33	6.28E-01	6.80E-01
**RP11-234G16.5**	0.53	6.28E-01	6.80E-01
**RP11-2B6.3**	0.17	6.28E-01	6.80E-01
**CXCL14**	0.16	6.28E-01	6.80E-01
**SPINK5**	-0.10	6.28E-01	6.80E-01
**RP11-1069G10.2**	-0.65	6.28E-01	6.80E-01
**FNDC3B**	0.10	6.28E-01	6.80E-01
**RP11-274H2.5**	0.35	6.29E-01	6.81E-01
**ZNF883**	0.22	6.29E-01	6.81E-01
**RDH12**	0.11	6.29E-01	6.81E-01
**RP11-7F17.8**	-0.18	6.29E-01	6.81E-01
**PTP4A1**	-0.08	6.30E-01	6.82E-01
**PHAX**	0.06	6.30E-01	6.82E-01
**RP11-817J15.3**	0.47	6.30E-01	6.82E-01
**SNX6**	-0.08	6.30E-01	6.82E-01
**MKRN2OS**	-0.11	6.30E-01	6.82E-01
**KIAA1549**	0.16	6.30E-01	6.82E-01
**CTD-2342J14.6**	0.34	6.30E-01	6.82E-01
**RP11-273G15.2**	-0.19	6.30E-01	6.82E-01
**RP11-286H14.4**	-0.41	6.30E-01	6.82E-01
**XLOC_006644**	0.28	6.31E-01	6.82E-01
**RDH11**	-0.13	6.31E-01	6.82E-01
**RP11-15A1.3**	0.27	6.31E-01	6.82E-01
**OPRK1**	-0.48	6.31E-01	6.82E-01
**CPA3**	0.26	6.31E-01	6.82E-01
**BORCS7**	0.10	6.31E-01	6.83E-01
**RP11-346C20.3**	0.25	6.31E-01	6.83E-01
**RP11-262H14.4**	-0.12	6.31E-01	6.83E-01
**LMCD1-AS1**	0.21	6.31E-01	6.83E-01
**G22770**	-0.37	6.31E-01	6.83E-01
**ARMC4P1**	-0.54	6.31E-01	6.83E-01
**PGAM1P7**	-0.59	6.31E-01	6.83E-01
**RP11-759A24.1**	0.46	6.31E-01	6.83E-01
**PIK3R1**	-0.09	6.32E-01	6.83E-01
**AC128709.4**	-0.51	6.32E-01	6.83E-01
**CREG1**	0.10	6.32E-01	6.83E-01
**CTB-33G10.1**	0.19	6.32E-01	6.84E-01

**LINC01134**	-0.34	6.32E-01	6.84E-01
**G34066**	-0.24	6.32E-01	6.84E-01
**MAPKAPK5-AS1**	0.08	6.32E-01	6.84E-01
**SLC25A24**	-0.09	6.33E-01	6.84E-01
**SEC23A**	0.07	6.33E-01	6.84E-01
**SAPCD2P3**	-0.54	6.34E-01	6.85E-01
**GLRA4**	0.40	6.34E-01	6.86E-01
**GUCA1A**	0.46	6.34E-01	6.86E-01
**PRKY**	-0.51	6.35E-01	6.86E-01
**RN7SL118P**	-0.36	6.35E-01	6.86E-01
**ZNF599**	-0.07	6.35E-01	6.86E-01
**LINC00618**	0.37	6.35E-01	6.86E-01
**G9021**	-0.32	6.35E-01	6.86E-01
**ACSL5**	0.19	6.35E-01	6.86E-01
**NME8**	0.34	6.35E-01	6.86E-01
**RP4-730K3.3**	0.40	6.35E-01	6.86E-01
**G26243**	-0.44	6.35E-01	6.86E-01
**ATP6V0E2-AS1**	0.24	6.35E-01	6.86E-01
**CSAD**	0.17	6.35E-01	6.86E-01
**RP11-69L16.5**	-0.37	6.35E-01	6.87E-01
**RPSAP18**	-0.28	6.35E-01	6.87E-01
**RP11-332H14.2**	-0.14	6.35E-01	6.87E-01
**RP11-115H18.1**	0.36	6.35E-01	6.87E-01
**RPSAP47**	0.51	6.36E-01	6.87E-01
**NSUN2**	-0.05	6.36E-01	6.87E-01
**HPSE**	0.12	6.36E-01	6.88E-01
**RP1-111C20.4**	0.15	6.36E-01	6.88E-01
**FXYD6**	0.16	6.37E-01	6.88E-01
**ARHGEF7**	-0.06	6.37E-01	6.88E-01
**AC006128.2**	0.23	6.37E-01	6.88E-01
**RP11-182J1.12**	-0.54	6.37E-01	6.88E-01
**GPATCH8**	0.05	6.37E-01	6.88E-01
**HSPA13**	0.10	6.37E-01	6.88E-01
**LINC00506**	-0.28	6.37E-01	6.88E-01
**RNU5B-2P**	-0.27	6.37E-01	6.89E-01
**CHKB-CPT1B**	0.36	6.37E-01	6.89E-01
**RP11-6O2.4**	-0.42	6.38E-01	6.89E-01
**RP11-1260E13.1**	-0.60	6.38E-01	6.89E-01
**RP11-391M20.1**	-0.12	6.38E-01	6.89E-01
**KB-68A7.1**	0.22	6.38E-01	6.89E-01
**RP11-1017G21.6**	0.22	6.38E-01	6.89E-01
**RP11-546D6.3**	-0.12	6.38E-01	6.89E-01
**ZNF528-AS1**	0.10	6.38E-01	6.89E-01
**AGMAT**	0.19	6.38E-01	6.89E-01
**RP1L1**	-0.15	6.38E-01	6.89E-01

**XLOC_012254**	-0.28	6.39E-01	6.90E-01
**PPP2R5A**	-0.06	6.39E-01	6.90E-01
**CLSPN**	-0.11	6.39E-01	6.90E-01
**LUZP1**	0.06	6.39E-01	6.90E-01
**CARD9**	0.13	6.39E-01	6.90E-01
**AC010524.2**	0.44	6.39E-01	6.90E-01
**AC072052.7**	-0.26	6.39E-01	6.90E-01
**RP5-1159O4.1**	0.17	6.39E-01	6.90E-01
**KCNIP2-AS1**	-0.39	6.39E-01	6.90E-01
**DLG1-AS1**	-0.29	6.39E-01	6.90E-01
**AL359091.2**	-0.30	6.39E-01	6.90E-01
**RN7SL574P**	-0.50	6.39E-01	6.90E-01
**ANKLE1**	0.26	6.40E-01	6.90E-01
**G8343**	-0.30	6.40E-01	6.90E-01
**TSPAN13**	0.09	6.40E-01	6.91E-01
**LINC00996**	-0.48	6.40E-01	6.91E-01
**G22813**	-0.53	6.40E-01	6.91E-01
**GSTA8P**	-0.52	6.40E-01	6.91E-01
**RIBC2**	-0.13	6.40E-01	6.91E-01
**GLIPR1**	0.12	6.40E-01	6.91E-01
**BEAN1**	-0.17	6.41E-01	6.91E-01
**RP6-201G10.2**	-0.21	6.41E-01	6.91E-01
**LRRC43**	0.23	6.41E-01	6.91E-01
**RSPH14**	-0.15	6.41E-01	6.91E-01
**CTD-2323K18.2**	-0.40	6.41E-01	6.92E-01
**RNU7-123P**	-0.53	6.42E-01	6.92E-01
**ASAP3**	0.07	6.42E-01	6.92E-01
**RP11-341N2.1**	-0.33	6.42E-01	6.92E-01
**LINC00536**	0.44	6.42E-01	6.93E-01
**PSTPIP2**	0.06	6.42E-01	6.93E-01
**RP11-350J20.5**	-0.16	6.42E-01	6.93E-01
**SHISA7**	-0.46	6.42E-01	6.93E-01
**ARHGEF6**	0.12	6.42E-01	6.93E-01
**KCNK13**	0.30	6.43E-01	6.93E-01
**BAK1P1**	-0.26	6.43E-01	6.93E-01
**CTC-558O2.2**	-0.44	6.43E-01	6.93E-01
**PLEK2**	0.07	6.43E-01	6.93E-01
**RP11-900F13.3**	-0.46	6.43E-01	6.93E-01
**LDHD**	0.23	6.43E-01	6.94E-01
**CASC8**	-0.21	6.44E-01	6.94E-01
**U47924.31**	-0.47	6.44E-01	6.95E-01
**PLN**	0.18	6.44E-01	6.95E-01
**LINC00431**	0.53	6.45E-01	6.95E-01
**CTD-3138B18.6**	0.21	6.45E-01	6.95E-01
**RP5-1050D4.4**	-0.42	6.45E-01	6.95E-01

**RP11-793H13.10**	-0.30	6.45E-01	6.95E-01
**CCDC85A**	-0.21	6.45E-01	6.95E-01
**GRID2**	-0.51	6.45E-01	6.96E-01
**RP11-311F12.2**	0.40	6.45E-01	6.96E-01
**RP2**	-0.07	6.45E-01	6.96E-01
**FAM19A3**	0.36	6.45E-01	6.96E-01
**VPREB3**	0.41	6.46E-01	6.96E-01
**MIR219-1**	0.43	6.46E-01	6.96E-01
**PGBD4P3**	0.52	6.46E-01	6.96E-01
**AC108676.1**	0.45	6.46E-01	6.96E-01
**CMTM5**	0.35	6.46E-01	6.96E-01
**PRELP**	0.14	6.46E-01	6.97E-01
**CTC-453G23.8**	-0.42	6.47E-01	6.97E-01
**CTD-2545G14.6**	-0.30	6.47E-01	6.98E-01
**BHLHE40-AS1**	-0.15	6.47E-01	6.98E-01
**AC004257.1**	0.48	6.47E-01	6.98E-01
**MCM6**	-0.07	6.47E-01	6.98E-01
**G33960**	-0.35	6.47E-01	6.98E-01
**TOMM20L**	-0.20	6.48E-01	6.98E-01
**EIF5AL1**	-0.22	6.48E-01	6.98E-01
**AC073283.4**	-0.40	6.48E-01	6.98E-01
**BEND3P1**	-0.27	6.48E-01	6.98E-01
**ADAMTS19**	-0.37	6.48E-01	6.99E-01
**RP4-704D23.1**	-0.42	6.48E-01	6.99E-01
**MEGF10**	-0.26	6.48E-01	6.99E-01
**TRGV3**	0.41	6.49E-01	6.99E-01
**RP11-1293J14.1**	-0.27	6.49E-01	6.99E-01
**VPS26A**	0.07	6.49E-01	6.99E-01
**ENPP6**	-0.28	6.49E-01	6.99E-01
**HPR**	-0.25	6.49E-01	6.99E-01
**FAM21B**	-0.47	6.49E-01	6.99E-01
**SCARB2**	-0.07	6.49E-01	6.99E-01
**ACA64**	0.29	6.50E-01	7.00E-01
**PHYH**	0.08	6.50E-01	7.00E-01
**RASGRP4**	0.17	6.50E-01	7.00E-01
**RP1-102K2.8**	0.31	6.50E-01	7.00E-01
**CHADL**	0.11	6.50E-01	7.00E-01
**DNAJC9**	0.06	6.50E-01	7.00E-01
**TMEM62**	0.04	6.51E-01	7.01E-01
**UCK2**	-0.07	6.51E-01	7.01E-01
**PDCD2L**	-0.12	6.51E-01	7.01E-01
**CBX3P9**	0.45	6.51E-01	7.01E-01
**SLC46A1**	0.14	6.52E-01	7.02E-01
**PRG2**	-0.31	6.52E-01	7.02E-01
**TRIM34**	-0.36	6.53E-01	7.03E-01

**G25345**	0.28	6.53E-01	7.03E-01
**ZFP64**	-0.06	6.53E-01	7.03E-01
**PLEKHM1P**	-0.11	6.53E-01	7.03E-01
**KLF4**	-0.09	6.53E-01	7.03E-01
**LINC00299**	0.39	6.53E-01	7.03E-01
**POLR2K**	-0.09	6.54E-01	7.04E-01
**KRCC1**	0.07	6.54E-01	7.04E-01
**KIAA1024**	-0.13	6.54E-01	7.04E-01
**COQ10B**	-0.06	6.54E-01	7.04E-01
**C12orf49**	0.05	6.55E-01	7.05E-01
**CTD-2527I21.14**	-0.48	6.55E-01	7.05E-01
**HMGN2P23**	-0.40	6.55E-01	7.05E-01
**PMPCB**	0.06	6.55E-01	7.05E-01
**RNY5P8**	-0.38	6.55E-01	7.05E-01
**SNTG1**	-0.38	6.55E-01	7.05E-01
**RP4-565E6.1**	0.35	6.56E-01	7.05E-01
**RP11-48B3.4**	-0.27	6.56E-01	7.05E-01
**C17orf100**	0.11	6.56E-01	7.06E-01
**TBCCD1**	-0.06	6.56E-01	7.06E-01
**COMMD8**	-0.10	6.56E-01	7.06E-01
**RP11-779O18.3**	0.40	6.56E-01	7.06E-01
**RP11-59C5.3**	-0.12	6.57E-01	7.06E-01
**RP11-407G23.2**	0.26	6.57E-01	7.07E-01
**PDGFRL**	-0.22	6.57E-01	7.07E-01
**ZGLP1**	0.29	6.57E-01	7.07E-01
**DMRT2**	0.23	6.57E-01	7.07E-01
**UNC5B-AS1**	-0.26	6.58E-01	7.07E-01
**RP11-386B13.4**	0.19	6.58E-01	7.07E-01
**RP11-510P12.1**	0.57	6.58E-01	7.07E-01
**RP11-863K10.7**	-0.25	6.58E-01	7.07E-01
**AL354718.1**	-0.19	6.58E-01	7.08E-01
**MRPS16**	-0.05	6.58E-01	7.08E-01
**C18orf54**	0.10	6.58E-01	7.08E-01
**RP11-460I13.6**	0.27	6.58E-01	7.08E-01
**G36779**	-0.20	6.58E-01	7.08E-01
**LARP1**	-0.05	6.58E-01	7.08E-01
**AC006538.1**	0.23	6.58E-01	7.08E-01
**PPP1R9A**	-0.22	6.59E-01	7.08E-01
**AC104438.1**	0.27	6.59E-01	7.08E-01
**SMUG1**	0.04	6.59E-01	7.08E-01
**RP11-182J1.1**	0.39	6.59E-01	7.08E-01
**SATB1**	0.06	6.59E-01	7.08E-01
**ZNF185**	0.07	6.59E-01	7.08E-01
**GTF2IP12**	-0.14	6.59E-01	7.09E-01
**CALCOCO1**	0.05	6.59E-01	7.09E-01

**ARRB1**	0.05	6.59E-01	7.09E-01
**VCAN**	0.23	6.59E-01	7.09E-01
**AC018892.9**	-0.22	6.59E-01	7.09E-01
**RCN2**	-0.06	6.59E-01	7.09E-01
**RP11-20D14.6**	0.40	6.60E-01	7.09E-01
**BMI1**	-0.07	6.60E-01	7.09E-01
**XLOC_005409**	-0.44	6.60E-01	7.09E-01
**XLOC_011614**	-0.37	6.60E-01	7.09E-01
**ARL6IP5**	0.08	6.60E-01	7.09E-01
**YBX1P1**	0.15	6.60E-01	7.09E-01
**DDO**	0.28	6.60E-01	7.10E-01
**RP11-818F20.5**	0.31	6.60E-01	7.10E-01
**GSTA9P**	-0.44	6.61E-01	7.10E-01
**LINC01449**	0.38	6.61E-01	7.10E-01
**KRTAP5-10**	-0.58	6.61E-01	7.10E-01
**CTR9**	0.05	6.61E-01	7.10E-01
**GUCA2B**	0.37	6.61E-01	7.10E-01
**C2orf74**	0.26	6.61E-01	7.10E-01
**RP11-861L17.3**	0.54	6.61E-01	7.10E-01
**G39721**	-0.30	6.61E-01	7.11E-01
**MRPL57**	0.07	6.61E-01	7.11E-01
**UNC13B**	-0.06	6.62E-01	7.11E-01
**SNRPD1**	0.07	6.62E-01	7.11E-01
**RP11-144O23.8**	-0.42	6.62E-01	7.11E-01
**CTB-175E5.7**	-0.18	6.62E-01	7.11E-01
**TRMT10C**	-0.07	6.62E-01	7.11E-01
**MIR193A**	-0.26	6.62E-01	7.11E-01
**TNFSF14**	0.16	6.63E-01	7.12E-01
**NXNL2**	-0.29	6.63E-01	7.12E-01
**RP11-242P2.1**	-0.55	6.63E-01	7.12E-01
**RP11-38L15.3**	-0.12	6.63E-01	7.12E-01
**CCDC144NL-AS1**	0.25	6.63E-01	7.12E-01
**MAD2L1**	-0.10	6.63E-01	7.12E-01
**ALG14**	-0.07	6.63E-01	7.12E-01
**LIN37**	0.15	6.64E-01	7.12E-01
**RNU6-510P**	0.30	6.64E-01	7.13E-01
**RP11-678G14.3**	0.34	6.64E-01	7.13E-01
**ATP6V1C2**	0.13	6.64E-01	7.13E-01
**KRTCAP2**	0.16	6.64E-01	7.13E-01
**IL22RA2**	-0.19	6.64E-01	7.13E-01
**NOP56P1**	0.69	6.64E-01	7.13E-01
**AC106801.1**	-0.43	6.64E-01	7.13E-01
**HIPK2**	0.07	6.65E-01	7.13E-01
**MIR194-2HG**	-0.42	6.65E-01	7.13E-01
**G41779**	0.28	6.65E-01	7.13E-01

**SETBP1**	-0.07	6.65E-01	7.13E-01
**TERT**	-0.44	6.65E-01	7.13E-01
**NICN1**	0.09	6.65E-01	7.13E-01
**CXADR**	0.08	6.65E-01	7.13E-01
**XLOC_012945**	-0.52	6.65E-01	7.14E-01
**RBFOX1**	-0.27	6.66E-01	7.14E-01
**RP11-645C24.5**	0.29	6.66E-01	7.15E-01
**RP11-80A15.1**	0.24	6.66E-01	7.15E-01
**MOSPD1**	-0.09	6.66E-01	7.15E-01
**IDS**	0.06	6.66E-01	7.15E-01
**CASC21**	-0.32	6.67E-01	7.15E-01
**HERC2P3**	0.29	6.67E-01	7.15E-01
**RP11-53M11.3**	-0.51	6.67E-01	7.15E-01
**RP11-79P5.9**	0.16	6.67E-01	7.15E-01
**PRELID3B**	-0.08	6.67E-01	7.15E-01
**TCAF2P1**	0.24	6.67E-01	7.15E-01
**RNY4P10**	0.31	6.67E-01	7.15E-01
**AF124730.4**	-0.45	6.67E-01	7.15E-01
**ADGRF5P1**	-0.26	6.67E-01	7.16E-01
**PTGIS**	-0.20	6.67E-01	7.16E-01
**TUBB3**	0.26	6.67E-01	7.16E-01
**RP4-680D5.2**	0.34	6.68E-01	7.16E-01
**GNAZ**	0.13	6.68E-01	7.16E-01
**CTD-3131K8.3**	0.23	6.68E-01	7.16E-01
**LINC00163**	0.38	6.68E-01	7.16E-01
**SLC19A3**	0.11	6.68E-01	7.17E-01
**AC004538.3**	-0.27	6.69E-01	7.17E-01
**AC006042.6**	-0.26	6.69E-01	7.17E-01
**ENTPD7**	-0.07	6.69E-01	7.17E-01
**PROX1-AS1**	-0.29	6.69E-01	7.18E-01
**CD33**	0.17	6.69E-01	7.18E-01
**C15ORF31**	-0.40	6.69E-01	7.18E-01
**PPP1R2P3**	0.36	6.70E-01	7.18E-01
**ZNF815P**	0.11	6.70E-01	7.18E-01
**RP5-1116H23.4**	-0.39	6.70E-01	7.18E-01
**RP1-153G14.4**	0.17	6.71E-01	7.19E-01
**MT4**	-0.54	6.71E-01	7.20E-01
**RTN4**	0.05	6.71E-01	7.20E-01
**CCNK**	-0.08	6.72E-01	7.20E-01
**MED30**	0.07	6.72E-01	7.21E-01
**IGHV2-5**	-0.62	6.72E-01	7.21E-01
**RP11-293A21.1**	-0.17	6.73E-01	7.21E-01
**RPS7P1**	-0.10	6.73E-01	7.21E-01
**RGS18**	-0.23	6.73E-01	7.21E-01
**CH17-140K24.5**	-0.44	6.73E-01	7.21E-01

**RNU6-172P**	0.53	6.73E-01	7.21E-01
**RP11-568N6.1**	0.11	6.73E-01	7.21E-01
**LRRC37A16P**	0.09	6.74E-01	7.22E-01
**TRIM74**	0.29	6.74E-01	7.22E-01
**GPD1**	-0.39	6.74E-01	7.22E-01
**HMGCLL1**	0.26	6.74E-01	7.22E-01
**G656**	0.47	6.74E-01	7.22E-01
**CELSR1**	-0.08	6.74E-01	7.23E-01
**ATP6AP2**	0.07	6.75E-01	7.23E-01
**CAMSAP3**	-0.10	6.75E-01	7.23E-01
**SPATC1**	0.52	6.75E-01	7.23E-01
**DHRS12**	-0.06	6.75E-01	7.23E-01
**RN7SL76P**	-0.36	6.75E-01	7.23E-01
**DCLK3**	-0.16	6.75E-01	7.23E-01
**RPL9P16**	-0.35	6.75E-01	7.23E-01
**KCNS2**	0.17	6.75E-01	7.23E-01
**COL25A1**	-0.16	6.76E-01	7.24E-01
**SCN2A**	0.19	6.76E-01	7.24E-01
**UBOX5**	-0.06	6.76E-01	7.24E-01
**RSPH6A**	-0.45	6.76E-01	7.24E-01
**TMEM138**	-0.06	6.76E-01	7.24E-01
**SETP12**	-0.30	6.76E-01	7.24E-01
**HIVEP3**	0.11	6.76E-01	7.24E-01
**PTBP2**	-0.08	6.77E-01	7.24E-01
**NEIL1**	-0.15	6.77E-01	7.25E-01
**MYO10**	-0.04	6.77E-01	7.25E-01
**LANCL2**	-0.05	6.77E-01	7.25E-01
**CTB-147C22.3**	-0.33	6.77E-01	7.25E-01
**DNAJA2**	0.06	6.77E-01	7.25E-01
**RP11-235E17.2**	-0.45	6.78E-01	7.26E-01
**RP3-331H24.7**	-0.27	6.78E-01	7.26E-01
**HCFC1**	0.06	6.78E-01	7.26E-01
**NT5E**	0.16	6.78E-01	7.26E-01
**RP11-306G20.1**	-0.48	6.78E-01	7.26E-01
**RP11-546M4.1**	0.41	6.78E-01	7.26E-01
**KCND2**	-0.27	6.78E-01	7.26E-01
**PCDH8**	0.39	6.78E-01	7.26E-01
**RP11-285C1.2**	-0.42	6.79E-01	7.26E-01
**FKBP14**	0.09	6.79E-01	7.26E-01
**LINC00899**	0.11	6.79E-01	7.27E-01
**CNN2P1**	-0.33	6.79E-01	7.27E-01
**PRICKLE2-AS3**	0.29	6.79E-01	7.27E-01
**SUCLA2**	-0.05	6.79E-01	7.27E-01
**RP11-881M11.2**	-0.35	6.79E-01	7.27E-01
**SLC15A2**	0.18	6.80E-01	7.27E-01

**UHRF1**	0.08	6.80E-01	7.27E-01
**SNX25P1**	0.35	6.80E-01	7.28E-01
**KDM4C**	-0.04	6.81E-01	7.28E-01
**PRCAT47**	0.55	6.81E-01	7.28E-01
**CAPN11**	0.16	6.81E-01	7.29E-01
**DIMT1**	-0.04	6.81E-01	7.29E-01
**NTS**	0.39	6.81E-01	7.29E-01
**GABRQ**	0.15	6.81E-01	7.29E-01
**RP11-297D21.4**	0.13	6.81E-01	7.29E-01
**RP11-279O9.4**	0.45	6.81E-01	7.29E-01
**DUSP13**	-0.23	6.81E-01	7.29E-01
**MID1IP1-AS1**	0.31	6.82E-01	7.29E-01
**RAB1C**	0.34	6.82E-01	7.29E-01
**CEBPA-AS1**	0.09	6.82E-01	7.30E-01
**RQCD1**	0.04	6.82E-01	7.30E-01
**TMEM63A**	-0.10	6.82E-01	7.30E-01
**RP11-403B2.6**	2.16	6.82E-01	7.30E-01
**CCDC78**	-0.21	6.83E-01	7.30E-01
**RPL3P2**	-0.28	6.83E-01	7.30E-01
**NUP37**	-0.05	6.83E-01	7.30E-01
**CYP2B7P**	-0.35	6.83E-01	7.31E-01
**LSMEM1**	-0.14	6.83E-01	7.31E-01
**CAPNS2**	0.09	6.84E-01	7.31E-01
**FBXO7**	0.03	6.84E-01	7.31E-01
**DNAJC3-AS1**	-0.10	6.84E-01	7.31E-01
**RP11-525E9.1**	-0.53	6.84E-01	7.31E-01
**SEMA5A**	-0.09	6.84E-01	7.31E-01
**TRAM1**	0.07	6.84E-01	7.31E-01
**PCP2**	-0.21	6.84E-01	7.31E-01
**LINC00621**	0.48	6.85E-01	7.32E-01
**UMAD1**	-0.06	6.85E-01	7.33E-01
**AP003774.1**	-0.52	6.85E-01	7.33E-01
**RP11-573M3.6**	-0.40	6.85E-01	7.33E-01
**G22769**	-0.28	6.86E-01	7.33E-01
**VDAC1**	0.05	6.86E-01	7.33E-01
**PCSK6-AS1**	-0.50	6.86E-01	7.33E-01
**GFOD1**	0.06	6.87E-01	7.34E-01
**GPR78**	-0.34	6.87E-01	7.34E-01
**CRISP2**	-0.36	6.87E-01	7.34E-01
**RPRML**	-0.29	6.87E-01	7.34E-01
**RP11-27I1.6**	-0.26	6.87E-01	7.34E-01
**MAP1B**	-0.11	6.87E-01	7.34E-01
**RP11-381N20.2**	-0.40	6.87E-01	7.34E-01
**CCDC40**	-0.12	6.88E-01	7.35E-01
**RHOQP2**	-0.31	6.88E-01	7.35E-01

**G17702**	-0.18	6.88E-01	7.35E-01
**RP11-539L10.2**	0.27	6.88E-01	7.35E-01
**TSNAXIP1**	0.16	6.88E-01	7.35E-01
**LIME1**	0.19	6.88E-01	7.35E-01
**ITPR1-AS1**	0.39	6.88E-01	7.35E-01
**LINC01341**	-0.28	6.88E-01	7.35E-01
**CBWD1**	0.09	6.89E-01	7.35E-01
**PUDPP2**	-0.30	6.89E-01	7.36E-01
**CYB5R4**	-0.05	6.89E-01	7.36E-01
**RP11-54D18.2**	0.27	6.89E-01	7.36E-01
**UBE2L5P**	0.28	6.89E-01	7.36E-01
**RP11-554D15.1**	-0.42	6.89E-01	7.36E-01
**MMADHC**	0.07	6.89E-01	7.36E-01
**RP11-30J20.1**	0.63	6.89E-01	7.36E-01
**CALHM1**	-0.54	6.89E-01	7.36E-01
**FUT9**	-0.51	6.90E-01	7.36E-01
**AK8**	-0.10	6.90E-01	7.36E-01
**C1QL2**	-0.36	6.90E-01	7.37E-01
**MTPN**	-0.06	6.90E-01	7.37E-01
**C14orf2**	0.06	6.90E-01	7.37E-01
**RP11-106M3.3**	-0.19	6.90E-01	7.37E-01
**RP11-129M6.1**	-0.59	6.90E-01	7.37E-01
**RP11-390F4.6**	-0.53	6.91E-01	7.37E-01
**ZFPM2-AS1**	-0.25	6.91E-01	7.37E-01
**PRRC2B**	-0.06	6.91E-01	7.37E-01
**CBWD5**	-0.10	6.91E-01	7.37E-01
**C2orf16**	-0.11	6.91E-01	7.37E-01
**AC005481.5**	0.29	6.91E-01	7.37E-01
**MTRF1L**	-0.06	6.91E-01	7.38E-01
**XLOC_014288**	0.14	6.91E-01	7.38E-01
**BRINP3**	-0.51	6.91E-01	7.38E-01
**C4orf46**	-0.08	6.92E-01	7.38E-01
**PDSS2**	-0.04	6.92E-01	7.39E-01
**ZDHHC6**	0.05	6.93E-01	7.39E-01
**PSMD9**	-0.07	6.93E-01	7.40E-01
**RDH13**	-0.08	6.94E-01	7.40E-01
**CTD-3001H11.2**	-0.23	6.94E-01	7.40E-01
**TRIM48**	-0.71	6.94E-01	7.40E-01
**CRTC3-AS1**	-0.17	6.94E-01	7.41E-01
**TNMD**	-0.28	6.94E-01	7.41E-01
**CTC-510F12.4**	-0.16	6.95E-01	7.41E-01
**LRRC63**	0.34	6.95E-01	7.41E-01
**SPDL1**	-0.06	6.95E-01	7.41E-01
**KRTAP5-5**	-0.53	6.95E-01	7.41E-01
**GOLGA8VP**	0.33	6.95E-01	7.42E-01

**CCDC63**	0.24	6.96E-01	7.42E-01
**CTA-313A17.2**	0.42	6.96E-01	7.42E-01
**U47924.32**	-0.35	6.96E-01	7.42E-01
**LINC00891**	0.28	6.96E-01	7.42E-01
**TULP3**	0.07	6.96E-01	7.43E-01
**PCDHA6**	-0.28	6.96E-01	7.43E-01
**KCNB1**	0.16	6.97E-01	7.43E-01
**OBSCN**	-0.12	6.97E-01	7.43E-01
**CILP**	0.19	6.97E-01	7.43E-01
**SNHG7**	0.12	6.97E-01	7.44E-01
**AC145676.2**	-0.25	6.97E-01	7.44E-01
**GVINP1**	-0.12	6.97E-01	7.44E-01
**SUGP2**	-0.09	6.98E-01	7.44E-01
**MED31**	0.06	6.98E-01	7.44E-01
**XXbac-B444P24.8**	-0.35	6.98E-01	7.44E-01
**G10060**	0.18	6.98E-01	7.44E-01
**SYPL1**	-0.06	6.98E-01	7.44E-01
**RPS2P46**	-0.13	6.98E-01	7.44E-01
**CTD-2561B21.11**	0.26	6.98E-01	7.45E-01
**ATXN10**	-0.06	6.98E-01	7.45E-01
**MRPS31**	0.06	6.99E-01	7.45E-01
**IQCE**	0.05	6.99E-01	7.45E-01
**XLOC_005592**	-0.14	6.99E-01	7.45E-01
**PSMD6**	0.05	6.99E-01	7.45E-01
**ADCK2**	0.05	6.99E-01	7.45E-01
**C16orf96**	0.19	6.99E-01	7.45E-01
**FAM120A**	0.02	6.99E-01	7.45E-01
**RP11-60C6.5**	0.29	6.99E-01	7.45E-01
**RP11-29H23.5**	-0.15	6.99E-01	7.45E-01
**RP11-686G8.2**	0.34	6.99E-01	7.45E-01
**ZNF671**	0.10	7.00E-01	7.46E-01
**CTC-308K20.1**	-0.22	7.00E-01	7.46E-01
**LAPTM4B**	0.07	7.00E-01	7.46E-01
**RP11-254I22.2**	0.39	7.00E-01	7.46E-01
**RP11-323I15.2**	-0.33	7.01E-01	7.47E-01
**XLOC_012707**	0.39	7.01E-01	7.47E-01
**RNU6-610P**	0.37	7.01E-01	7.47E-01
**ENPP4**	-0.14	7.01E-01	7.47E-01
**HSD17B7P2**	-0.11	7.01E-01	7.47E-01
**ADCK1**	-0.08	7.01E-01	7.47E-01
**RP11-347C18.3**	-0.14	7.01E-01	7.47E-01
**POU5F1P3**	0.28	7.01E-01	7.47E-01
**ABL2**	-0.05	7.02E-01	7.47E-01
**PKN2-AS1**	-0.25	7.02E-01	7.48E-01
**GRM2**	0.16	7.02E-01	7.48E-01

**RPS4Y1**	0.52	7.02E-01	7.48E-01
**RP11-254F7.4**	0.35	7.02E-01	7.48E-01
**VHL**	-0.04	7.02E-01	7.48E-01
**HCAR2**	0.06	7.02E-01	7.48E-01
**RP11-286B14.2**	0.26	7.03E-01	7.48E-01
**MIR4292**	0.17	7.03E-01	7.48E-01
**TBC1D10C**	0.15	7.03E-01	7.49E-01
**DDX43**	-0.19	7.03E-01	7.49E-01
**RP11-690G19.4**	0.35	7.03E-01	7.49E-01
**RP11-286H15.1**	0.44	7.03E-01	7.49E-01
**HESX1**	0.10	7.04E-01	7.49E-01
**EXOSC8**	0.04	7.04E-01	7.49E-01
**RP4-607I7.1**	0.21	7.04E-01	7.50E-01
**RP11-131L23.1**	0.20	7.04E-01	7.50E-01
**BEX2**	0.09	7.04E-01	7.50E-01
**RP11-307C12.11**	0.10	7.04E-01	7.50E-01
**TUBGCP4**	-0.07	7.04E-01	7.50E-01
**RP11-521D12.5**	0.14	7.04E-01	7.50E-01
**NPPA**	-0.28	7.05E-01	7.50E-01
**ARSG**	0.05	7.05E-01	7.50E-01
**G36296**	-0.29	7.05E-01	7.50E-01
**TMEM158**	0.12	7.05E-01	7.50E-01
**RP11-326I19.2**	-0.25	7.05E-01	7.50E-01
**ADAMTSL5**	-0.15	7.05E-01	7.51E-01
**AL022393.9**	-0.41	7.06E-01	7.51E-01
**RPL23AP66**	-0.44	7.06E-01	7.51E-01
**RP4-633I8.4**	-0.23	7.06E-01	7.51E-01
**LL0XNC01-116E7.2**	0.34	7.06E-01	7.51E-01
**ATG16L2**	-0.12	7.06E-01	7.52E-01
**ZNF584**	-0.05	7.06E-01	7.52E-01
**SLC25A41**	-0.33	7.07E-01	7.52E-01
**RP11-823E8.3**	-0.30	7.07E-01	7.52E-01
**KCNU1**	-0.43	7.07E-01	7.52E-01
**TTLL7**	0.13	7.07E-01	7.52E-01
**SLITRK3**	0.47	7.07E-01	7.52E-01
**PCDHGB7**	0.06	7.07E-01	7.52E-01
**RP11-297N6.4**	0.15	7.07E-01	7.52E-01
**AC006978.6**	0.06	7.07E-01	7.52E-01
**DPH6-AS1**	-0.27	7.07E-01	7.53E-01
**KLF10**	0.07	7.07E-01	7.53E-01
**AL590822.1**	0.18	7.08E-01	7.53E-01
**SNORA77**	0.23	7.08E-01	7.53E-01
**CTC-255N20.1**	0.25	7.08E-01	7.53E-01
**ADGRG5**	-0.12	7.08E-01	7.53E-01
**AF146191.4**	-0.17	7.09E-01	7.54E-01

**SPDYE2B**	0.30	7.09E-01	7.54E-01
**ST13P20**	-0.14	7.09E-01	7.54E-01
**GABARAP**	0.15	7.09E-01	7.54E-01
**RP11-1376P16.2**	0.32	7.09E-01	7.54E-01
**CCT8**	0.07	7.09E-01	7.54E-01
**RP11-280K24.4**	-0.26	7.10E-01	7.55E-01
**AGBL2**	-0.16	7.10E-01	7.55E-01
**INSC**	0.35	7.10E-01	7.55E-01
**NMNAT1**	0.05	7.10E-01	7.55E-01
**MIF**	0.22	7.10E-01	7.55E-01
**RP11-355F16.1**	0.16	7.10E-01	7.55E-01
**RNU1-143P**	-0.46	7.10E-01	7.55E-01
**POT1-AS1**	0.31	7.11E-01	7.55E-01
**SLC51B**	-0.17	7.11E-01	7.55E-01
**MIR181A2HG**	0.14	7.11E-01	7.55E-01
**LINC00989**	0.22	7.11E-01	7.55E-01
**LINC00202-1**	0.18	7.11E-01	7.56E-01
**AC068580.6**	-0.15	7.11E-01	7.56E-01
**RP11-15B17.1**	0.42	7.11E-01	7.56E-01
**RP11-417O11.5**	-0.27	7.11E-01	7.56E-01
**RP11-478C6.1**	-0.23	7.11E-01	7.56E-01
**PRPH2**	-0.18	7.11E-01	7.56E-01
**RP11-1082L8.3**	-0.31	7.11E-01	7.56E-01
**IRF2BP2**	-0.04	7.11E-01	7.56E-01
**MZF1**	-0.07	7.11E-01	7.56E-01
**DISP2**	0.23	7.12E-01	7.56E-01
**RP11-472B18.1**	0.17	7.12E-01	7.56E-01
**RP11-44F14.1**	0.40	7.12E-01	7.56E-01
**IL1RAPL1**	0.23	7.12E-01	7.56E-01
**TAC3**	-0.43	7.12E-01	7.56E-01
**LINC01447**	-0.34	7.12E-01	7.56E-01
**SQLE**	-0.10	7.12E-01	7.56E-01
**ADAT1**	0.05	7.13E-01	7.57E-01
**RP11-681L4.2**	-0.40	7.13E-01	7.58E-01
**MREG**	-0.09	7.13E-01	7.58E-01
**G39423**	-0.22	7.13E-01	7.58E-01
**NUCB1-AS1**	-0.43	7.14E-01	7.58E-01
**ITIH4**	0.26	7.14E-01	7.58E-01
**PAK2**	0.04	7.14E-01	7.58E-01
**RNU1-59P**	-0.32	7.14E-01	7.58E-01
**RP11-440D17.3**	0.11	7.14E-01	7.58E-01
**ANKRD35**	-0.09	7.14E-01	7.58E-01
**CTD-3214K23.1**	-0.27	7.14E-01	7.58E-01
**RP11-1M18.1**	-0.45	7.14E-01	7.58E-01
**U91328.19**	0.15	7.14E-01	7.58E-01

**RP11-262H14.5**	0.18	7.14E-01	7.59E-01
**TMEM64**	-0.07	7.15E-01	7.59E-01
**ACTA2-AS1**	0.15	7.15E-01	7.59E-01
**RP11-812E19.3**	0.67	7.15E-01	7.59E-01
**AC004699.1**	0.23	7.15E-01	7.59E-01
**FLJ31104**	0.22	7.15E-01	7.59E-01
**ZMIZ1-AS1**	0.10	7.15E-01	7.60E-01
**AASS**	0.06	7.16E-01	7.60E-01
**DCAF12L2**	-0.29	7.16E-01	7.60E-01
**RN7SL512P**	-0.47	7.16E-01	7.60E-01
**SAR1A**	-0.04	7.16E-01	7.60E-01
**MIR3605**	-0.35	7.16E-01	7.61E-01
**AC007364.1**	-0.31	7.16E-01	7.61E-01
**NRK**	-0.21	7.17E-01	7.61E-01
**EVC2**	0.07	7.17E-01	7.61E-01
**RPL37**	0.06	7.17E-01	7.61E-01
**PSMG2**	-0.05	7.17E-01	7.61E-01
**PPP4R1L**	0.07	7.17E-01	7.61E-01
**RP11-190C22.8**	-0.38	7.17E-01	7.61E-01
**LINC00909**	-0.05	7.17E-01	7.61E-01
**U2AF1**	0.12	7.17E-01	7.61E-01
**FLJ41941**	-0.39	7.17E-01	7.61E-01
**RP11-8H2.1**	0.19	7.18E-01	7.61E-01
**TMEM217**	0.10	7.18E-01	7.62E-01
**SRD5A2**	-0.33	7.18E-01	7.62E-01
**NPM2**	-0.14	7.18E-01	7.62E-01
**DSEL**	0.11	7.18E-01	7.62E-01
**ZNF663P**	-0.30	7.18E-01	7.62E-01
**PPP1R2**	-0.06	7.18E-01	7.62E-01
**NRG3**	0.35	7.18E-01	7.62E-01
**DPYD**	-0.08	7.18E-01	7.62E-01
**PABPC5**	0.16	7.18E-01	7.62E-01
**ARHGEF11**	-0.03	7.19E-01	7.62E-01
**TEX14**	0.17	7.19E-01	7.62E-01
**AC011933.2**	0.23	7.19E-01	7.63E-01
**RNA5SP82**	0.35	7.19E-01	7.63E-01
**PLEKHD1**	0.17	7.19E-01	7.63E-01
**NEK2**	-0.10	7.19E-01	7.63E-01
**COA7**	0.06	7.19E-01	7.63E-01
**DET1**	0.08	7.19E-01	7.63E-01
**SPG20-AS1**	0.28	7.20E-01	7.63E-01
**RP11-61I13.3**	-0.22	7.20E-01	7.63E-01
**RP11-616K22.1**	-0.26	7.20E-01	7.63E-01
**HAT1**	0.06	7.20E-01	7.64E-01
**IGFALS**	-0.18	7.20E-01	7.64E-01

**CALML4**	0.10	7.20E-01	7.64E-01
**RPL7AP6**	-0.09	7.21E-01	7.64E-01
**RP11-771K4.3**	-0.33	7.21E-01	7.64E-01
**DDX3X**	-0.06	7.21E-01	7.64E-01
**CTD-3236F5.1**	-0.21	7.21E-01	7.64E-01
**CCNL1**	-0.07	7.21E-01	7.65E-01
**G4016**	-0.37	7.21E-01	7.65E-01
**MIR1245A**	0.33	7.21E-01	7.65E-01
**CCR2**	0.17	7.21E-01	7.65E-01
**SLAMF1**	0.17	7.21E-01	7.65E-01
**NIPSNAP3B**	-0.11	7.21E-01	7.65E-01
**WNT7B**	0.11	7.22E-01	7.65E-01
**GPR89B**	-0.08	7.22E-01	7.65E-01
**VIP**	0.36	7.22E-01	7.65E-01
**RP4-633O19__A.1**	-0.43	7.22E-01	7.65E-01
**G19311**	-0.34	7.22E-01	7.66E-01
**G30851**	0.32	7.23E-01	7.66E-01
**XLOC_002900**	0.25	7.23E-01	7.66E-01
**CASC11**	-0.20	7.23E-01	7.66E-01
**CTD-2135J3.3**	0.15	7.23E-01	7.66E-01
**RP11-128M1.1**	-0.25	7.23E-01	7.66E-01
**BTBD10**	-0.04	7.23E-01	7.66E-01
**RARRES1**	-0.17	7.23E-01	7.66E-01
**KRT15**	0.13	7.23E-01	7.66E-01
**PCDHB13**	0.07	7.23E-01	7.67E-01
**EIF4A2P1**	0.25	7.24E-01	7.67E-01
**ZNF200**	0.04	7.24E-01	7.67E-01
**LPAL2**	0.14	7.24E-01	7.67E-01
**CTD-2129N1.1**	0.27	7.24E-01	7.67E-01
**HSPA8**	0.08	7.25E-01	7.68E-01
**HOMER2**	0.08	7.25E-01	7.68E-01
**ZNF37CP**	-0.37	7.25E-01	7.68E-01
**RP11-375N15.2**	0.41	7.25E-01	7.68E-01
**LA16c-390E6.4**	0.22	7.25E-01	7.68E-01
**PROS2P**	-0.36	7.25E-01	7.68E-01
**PTRHD1**	0.05	7.25E-01	7.68E-01
**GPR182**	-0.21	7.25E-01	7.68E-01
**RP11-727F15.9**	0.29	7.26E-01	7.68E-01
**RP11-6N17.3**	0.17	7.26E-01	7.68E-01
**HCG20**	-0.31	7.26E-01	7.69E-01
**PCDHAC1**	0.20	7.26E-01	7.69E-01
**SPTLC2**	-0.05	7.26E-01	7.69E-01
**RP11-323C15.2**	0.29	7.26E-01	7.69E-01
**RP11-95D17.1**	-0.06	7.26E-01	7.69E-01
**RP11-504A18.1**	0.35	7.27E-01	7.69E-01

**RP11-770E5.2**	-0.37	7.27E-01	7.69E-01
**NDEL1**	0.05	7.27E-01	7.69E-01
**ARHGAP24**	0.06	7.27E-01	7.70E-01
**RP11-353K11.1**	-0.14	7.27E-01	7.70E-01
**XLOC_007240**	-0.24	7.27E-01	7.70E-01
**CTD-3088G3.8**	-0.14	7.27E-01	7.70E-01
**VWA7**	-0.12	7.28E-01	7.70E-01
**RASEF**	0.11	7.28E-01	7.70E-01
**RP11-1081L13.4**	0.13	7.28E-01	7.71E-01
**PLD6**	-0.09	7.28E-01	7.71E-01
**DHFRL1**	-0.04	7.29E-01	7.71E-01
**G18525**	0.33	7.29E-01	7.72E-01
**CBFB**	0.03	7.29E-01	7.72E-01
**KPNA3**	-0.05	7.29E-01	7.72E-01
**FAM35BP**	-0.27	7.29E-01	7.72E-01
**COL9A1**	0.39	7.29E-01	7.72E-01
**RP11-631F7.1**	-0.41	7.29E-01	7.72E-01
**RP11-799B12.4**	0.26	7.29E-01	7.72E-01
**XX-FW83563B9.5**	-0.13	7.29E-01	7.72E-01
**RP11-1055B8.9**	-0.18	7.29E-01	7.72E-01
**XLOC_002675**	0.20	7.30E-01	7.72E-01
**C1orf140**	0.32	7.30E-01	7.72E-01
**GATA6**	-0.17	7.30E-01	7.72E-01
**PDCD6IPP2**	-0.24	7.30E-01	7.72E-01
**GLUD1P3**	-0.11	7.30E-01	7.72E-01
**RP11-22C11.2**	-0.10	7.30E-01	7.72E-01
**XLOC_002882**	-0.26	7.30E-01	7.73E-01
**IL6R**	0.06	7.30E-01	7.73E-01
**RET**	0.11	7.30E-01	7.73E-01
**RP11-73M7.1**	-0.42	7.30E-01	7.73E-01
**LDLRAD1**	-0.30	7.30E-01	7.73E-01
**NBPF3**	0.09	7.31E-01	7.73E-01
**CTB-33O18.3**	-0.19	7.31E-01	7.73E-01
**EIF3C**	-0.08	7.31E-01	7.73E-01
**OR7E39P**	-0.29	7.31E-01	7.73E-01
**EZR**	0.06	7.31E-01	7.73E-01
**RP11-758P17.3**	-0.26	7.31E-01	7.73E-01
**RP11-65J3.15**	0.39	7.31E-01	7.73E-01
**TMEM200C**	-0.17	7.31E-01	7.73E-01
**SOX2-OT**	-0.14	7.32E-01	7.74E-01
**ADH4**	-0.21	7.32E-01	7.74E-01
**ALDH2**	0.06	7.32E-01	7.74E-01
**SATB1-AS1**	-0.27	7.33E-01	7.75E-01
**PMM2**	-0.06	7.33E-01	7.75E-01
**RNU6-482P**	-0.43	7.33E-01	7.75E-01

**PASK**	-0.08	7.34E-01	7.76E-01
**BRD7P2**	0.17	7.34E-01	7.76E-01
**RP11-1072A3.4**	0.18	7.34E-01	7.76E-01
**RP11-330L19.1**	-0.29	7.34E-01	7.76E-01
**BORCS8**	-0.07	7.34E-01	7.76E-01
**PRPF3**	-0.05	7.34E-01	7.76E-01
**HSPB1P1**	0.22	7.34E-01	7.76E-01
**AC083873.4**	-0.28	7.34E-01	7.76E-01
**RP11-983P16.4**	-0.10	7.34E-01	7.76E-01
**TLK2**	-0.03	7.35E-01	7.76E-01
**AC005264.2**	-0.14	7.35E-01	7.76E-01
**CTD-2619J13.5**	-0.33	7.35E-01	7.76E-01
**XLOC_000625**	0.26	7.35E-01	7.76E-01
**GPR87**	-0.06	7.35E-01	7.76E-01
**RP11-950C14.7**	0.30	7.35E-01	7.76E-01
**RP11-441F2.5**	-0.48	7.35E-01	7.76E-01
**DECR1**	0.04	7.35E-01	7.77E-01
**ATP6AP1L**	0.10	7.35E-01	7.77E-01
**RP11-834C11.5**	-0.34	7.35E-01	7.77E-01
**SPPL3**	-0.05	7.35E-01	7.77E-01
**VGF**	-0.18	7.35E-01	7.77E-01
**RP11-523O18.5**	-0.33	7.35E-01	7.77E-01
**EDARADD**	0.06	7.36E-01	7.77E-01
**SLC9A2**	-0.17	7.36E-01	7.77E-01
**RP11-791G16.2**	0.26	7.36E-01	7.77E-01
**SNORD125**	0.19	7.36E-01	7.77E-01
**SYPL2**	0.33	7.36E-01	7.77E-01
**TRGV9**	0.37	7.36E-01	7.77E-01
**RBM12**	0.05	7.36E-01	7.77E-01
**SP7**	-0.32	7.36E-01	7.78E-01
**MRPS25**	-0.06	7.37E-01	7.78E-01
**COPB2**	-0.04	7.37E-01	7.78E-01
**PARD3-AS1**	0.17	7.37E-01	7.78E-01
**XLOC_007667**	0.32	7.37E-01	7.78E-01
**GGT2**	-0.42	7.37E-01	7.78E-01
**ZNF143**	-0.07	7.37E-01	7.78E-01
**CASP10**	0.07	7.37E-01	7.78E-01
**USP32P2**	-0.44	7.37E-01	7.78E-01
**HCN4**	0.27	7.37E-01	7.78E-01
**CD84**	0.12	7.37E-01	7.78E-01
**AC022819.3**	-0.23	7.37E-01	7.78E-01
**SKA1**	-0.10	7.38E-01	7.79E-01
**FGF5**	0.45	7.38E-01	7.79E-01
**RP11-463O9.9**	-0.20	7.38E-01	7.79E-01
**RP11-640L9.2**	-0.16	7.38E-01	7.79E-01

**SCN11A**	-0.16	7.38E-01	7.79E-01
**FZD8**	0.08	7.38E-01	7.80E-01
**RP11-243M5.2**	-0.46	7.39E-01	7.80E-01
**PREX2**	0.10	7.39E-01	7.80E-01
**RP11-723O4.6**	0.13	7.39E-01	7.80E-01
**G42332**	-0.17	7.39E-01	7.80E-01
**OR7E121P**	0.56	7.39E-01	7.80E-01
**WDR55**	0.04	7.40E-01	7.80E-01
**HMGCS2**	0.19	7.40E-01	7.81E-01
**RP11-305D15.8**	-0.25	7.40E-01	7.81E-01
**G33207**	0.19	7.40E-01	7.81E-01
**SLC25A13**	-0.04	7.40E-01	7.81E-01
**MED14OS**	-0.25	7.40E-01	7.81E-01
**ST20-AS1**	0.09	7.41E-01	7.82E-01
**AC003075.4**	-0.12	7.41E-01	7.82E-01
**RP11-958N24.2**	0.33	7.41E-01	7.82E-01
**UBE2SP1**	-0.21	7.41E-01	7.82E-01
**RP11-700F16.3**	-0.33	7.41E-01	7.82E-01
**RNU6-736P**	-0.26	7.42E-01	7.83E-01
**P2RX6**	-0.14	7.42E-01	7.83E-01
**TIGD6**	-0.04	7.42E-01	7.83E-01
**TNPO1P3**	-0.45	7.42E-01	7.83E-01
**RPP40**	-0.07	7.42E-01	7.83E-01
**SLC25A15P2**	-0.40	7.43E-01	7.84E-01
**GPC6-AS2**	-0.38	7.43E-01	7.84E-01
**RP1-283E3.4**	-0.11	7.43E-01	7.84E-01
**RP11-347H15.1**	0.32	7.43E-01	7.84E-01
**RP11-320N21.2**	-0.16	7.43E-01	7.84E-01
**PGAP2**	-0.06	7.43E-01	7.84E-01
**G26223**	0.27	7.43E-01	7.84E-01
**HCG4B**	-0.25	7.43E-01	7.84E-01
**RN7SKP38**	-0.34	7.43E-01	7.84E-01
**LINC00910**	-0.07	7.43E-01	7.84E-01
**LINC01358**	-0.18	7.44E-01	7.84E-01
**CKS1BP3**	0.28	7.44E-01	7.84E-01
**RNU2-22P**	-0.39	7.44E-01	7.84E-01
**OTUB2**	0.10	7.44E-01	7.84E-01
**RP11-114H24.6**	0.17	7.44E-01	7.84E-01
**G11195**	-0.23	7.44E-01	7.85E-01
**RP11-212P7.2**	-0.06	7.44E-01	7.85E-01
**RPL30**	0.05	7.45E-01	7.85E-01
**ZC3H10**	-0.05	7.45E-01	7.85E-01
**RP11-230G5.2**	-0.34	7.45E-01	7.85E-01
**CATSPER1**	0.15	7.45E-01	7.85E-01
**RP11-213H15.1**	0.26	7.45E-01	7.85E-01

**ZNF561**	0.04	7.45E-01	7.85E-01
**CENPO**	0.05	7.45E-01	7.85E-01
**KIAA1671**	-0.05	7.45E-01	7.86E-01
**ANKRD20A1**	-0.27	7.45E-01	7.86E-01
**NKX3-2**	0.25	7.46E-01	7.86E-01
**ABO**	-0.11	7.46E-01	7.86E-01
**PEG13**	0.14	7.46E-01	7.86E-01
**CTGLF12P**	-0.21	7.46E-01	7.86E-01
**LINC01315**	0.09	7.46E-01	7.86E-01
**PBX2P1**	0.28	7.46E-01	7.86E-01
**G5552**	-0.19	7.46E-01	7.86E-01
**TRPV1**	-0.11	7.46E-01	7.86E-01
**C10orf111**	-0.31	7.46E-01	7.86E-01
**TCAM1P**	0.18	7.47E-01	7.87E-01
**ANKRD42**	0.04	7.47E-01	7.87E-01
**XLOC_007096**	0.36	7.47E-01	7.87E-01
**LINC00930**	-0.39	7.47E-01	7.87E-01
**APOB**	0.26	7.47E-01	7.87E-01
**AP006621.9**	0.30	7.47E-01	7.87E-01
**UBQLNL**	-0.25	7.47E-01	7.87E-01
**CNN2P9**	0.23	7.48E-01	7.88E-01
**RP11-756A22.7**	0.29	7.48E-01	7.88E-01
**RGS7BP**	-0.33	7.48E-01	7.88E-01
**KDM5D**	-0.34	7.48E-01	7.88E-01
**HOXA-AS2**	0.12	7.48E-01	7.88E-01
**EIF2B3**	-0.04	7.48E-01	7.88E-01
**G31047**	-0.20	7.48E-01	7.88E-01
**HSDL2**	0.07	7.48E-01	7.88E-01
**CGN**	-0.08	7.48E-01	7.88E-01
**ST3GAL6-AS1**	0.15	7.48E-01	7.88E-01
**OCIAD1**	0.04	7.49E-01	7.88E-01
**ZBED4**	-0.04	7.49E-01	7.88E-01
**RP11-967K21.1**	-0.13	7.49E-01	7.88E-01
**CTD-3195I5.3**	-0.17	7.49E-01	7.89E-01
**RNU6-97P**	0.35	7.49E-01	7.89E-01
**ATP7B**	0.07	7.49E-01	7.89E-01
**XLOC_002952**	0.21	7.49E-01	7.89E-01
**PAXIP1-AS2**	-0.08	7.49E-01	7.89E-01
**RP11-613M5.2**	-0.28	7.49E-01	7.89E-01
**RP11-124N2.1**	0.11	7.50E-01	7.89E-01
**SPIN4**	0.07	7.50E-01	7.89E-01
**CTD-2287O16.5**	0.08	7.50E-01	7.89E-01
**FMO9P**	-0.30	7.50E-01	7.89E-01
**RP11-62C7.2**	0.35	7.50E-01	7.90E-01
**G18696**	0.24	7.50E-01	7.90E-01

**RP11-79O8.1**	-0.30	7.50E-01	7.90E-01
**SALL2**	0.08	7.50E-01	7.90E-01
**RWDD4**	0.05	7.51E-01	7.90E-01
**CTD-2017C7.1**	-0.19	7.51E-01	7.90E-01
**FAM183A**	-0.13	7.51E-01	7.90E-01
**HARBI1**	0.05	7.52E-01	7.91E-01
**C4orf36**	0.05	7.52E-01	7.91E-01
**TIMD4**	-0.32	7.52E-01	7.91E-01
**MATN1**	-0.18	7.52E-01	7.91E-01
**RP11-989E6.3**	0.41	7.52E-01	7.91E-01
**LINC00622**	0.13	7.52E-01	7.92E-01
**AC079776.2**	-0.29	7.53E-01	7.92E-01
**SCN2B**	0.11	7.53E-01	7.92E-01
**C12orf60**	-0.11	7.53E-01	7.92E-01
**RP11-174O3.1**	0.27	7.53E-01	7.92E-01
**MDH1B**	0.14	7.54E-01	7.93E-01
**RP11-1152H14.1**	0.18	7.54E-01	7.93E-01
**HOXB1**	-0.40	7.55E-01	7.94E-01
**NSUN5P2**	-0.27	7.55E-01	7.94E-01
**CYCS**	0.06	7.55E-01	7.94E-01
**RP11-117D22.2**	0.31	7.55E-01	7.94E-01
**PFKFB2**	-0.07	7.55E-01	7.94E-01
**MIR663AHG**	0.21	7.56E-01	7.95E-01
**PTMAP1**	0.25	7.56E-01	7.95E-01
**MCM2**	0.06	7.56E-01	7.95E-01
**RPS20P33**	-0.18	7.56E-01	7.95E-01
**LINC00854**	-0.13	7.56E-01	7.95E-01
**CDRT15P1**	0.15	7.56E-01	7.95E-01
**ZBED9**	-0.13	7.57E-01	7.95E-01
**RP11-253M7.4**	-0.27	7.57E-01	7.96E-01
**GABRA3**	-0.13	7.57E-01	7.96E-01
**CTA-228A9.3**	-0.16	7.57E-01	7.96E-01
**TSGA10IP**	-0.22	7.57E-01	7.96E-01
**CHURC1**	-0.06	7.57E-01	7.96E-01
**GEMIN7**	0.05	7.58E-01	7.96E-01
**LETMD1**	-0.05	7.58E-01	7.97E-01
**CFAP45**	-0.10	7.58E-01	7.97E-01
**RP11-120K24.3**	0.19	7.58E-01	7.97E-01
**RP11-435O5.2**	-0.13	7.58E-01	7.97E-01
**LINC01291**	-0.44	7.58E-01	7.97E-01
**PMS2P5**	0.11	7.59E-01	7.98E-01
**XLOC_004203**	-0.25	7.59E-01	7.98E-01
**NPY2R**	0.50	7.59E-01	7.98E-01
**SYCP2L**	-0.15	7.59E-01	7.98E-01
**ASS1**	-0.07	7.59E-01	7.98E-01

**CDCA3**	0.07	7.59E-01	7.98E-01
**EZH1**	-0.05	7.59E-01	7.98E-01
**AC018442.1**	-0.24	7.59E-01	7.98E-01
**RP11-567M16.6**	0.07	7.59E-01	7.98E-01
**RN7SL9P**	-0.45	7.59E-01	7.98E-01
**RP11-95G6.1**	0.26	7.60E-01	7.98E-01
**RBM43**	0.05	7.60E-01	7.98E-01
**EIF4A2**	-0.07	7.60E-01	7.98E-01
**NMNAT2**	0.10	7.60E-01	7.98E-01
**IMPDH1P10**	-0.30	7.60E-01	7.99E-01
**CSPG5**	-0.11	7.60E-01	7.99E-01
**ALG1L12P**	0.26	7.60E-01	7.99E-01
**TBC1D22B**	0.04	7.60E-01	7.99E-01
**RP11-507K2.3**	-0.09	7.60E-01	7.99E-01
**CGA**	-0.41	7.61E-01	7.99E-01
**RIMS2**	0.16	7.61E-01	7.99E-01
**GFRA3**	0.23	7.61E-01	7.99E-01
**MFSD1**	0.04	7.61E-01	7.99E-01
**DCN**	0.12	7.61E-01	7.99E-01
**TTLL4**	-0.05	7.61E-01	7.99E-01
**C22orf29**	-0.05	7.61E-01	7.99E-01
**RNASEH2CP1**	-0.17	7.61E-01	7.99E-01
**RAP2A**	-0.05	7.61E-01	7.99E-01
**CTD-2026D20.2**	-0.19	7.61E-01	7.99E-01
**EDNRB**	0.09	7.62E-01	8.00E-01
**RP11-170N16.3**	0.15	7.62E-01	8.00E-01
**INTS10**	-0.02	7.62E-01	8.00E-01
**RP11-161I6.2**	0.35	7.62E-01	8.00E-01
**RPL17P50**	-0.15	7.62E-01	8.00E-01
**RNF115**	0.03	7.62E-01	8.00E-01
**XLOC_012849**	0.23	7.62E-01	8.00E-01
**WAC**	0.02	7.63E-01	8.01E-01
**RP11-483F11.7**	-0.24	7.63E-01	8.01E-01
**RAB19**	0.30	7.63E-01	8.01E-01
**PHKA1-AS1**	-0.32	7.63E-01	8.01E-01
**DEPDC5**	0.04	7.63E-01	8.01E-01
**RP11-70O5.2**	0.28	7.63E-01	8.01E-01
**RP11-92G12.3**	0.28	7.64E-01	8.01E-01
**RP11-249L21.4**	-0.32	7.64E-01	8.02E-01
**ROCK1P1**	0.19	7.64E-01	8.02E-01
**AC024937.4**	-0.36	7.64E-01	8.02E-01
**SDR16C5**	-0.07	7.65E-01	8.02E-01
**LINC00689**	0.31	7.65E-01	8.02E-01
**TCEA3**	0.04	7.65E-01	8.02E-01
**RP11-964E11.3**	0.36	7.65E-01	8.02E-01

**RP4-785G19.5**	-0.28	7.65E-01	8.03E-01
**G36419**	-0.18	7.65E-01	8.03E-01
**TNFRSF25**	0.12	7.65E-01	8.03E-01
**AC017006.2**	0.33	7.65E-01	8.03E-01
**AC022007.5**	-0.09	7.65E-01	8.03E-01
**PEX19**	-0.04	7.66E-01	8.03E-01
**LINC00528**	0.22	7.66E-01	8.03E-01
**C8orf37-AS1**	0.14	7.66E-01	8.04E-01
**ATXN7L1**	-0.04	7.66E-01	8.04E-01
**CTB-40H15.4**	0.31	7.66E-01	8.04E-01
**RNU7-115P**	0.27	7.66E-01	8.04E-01
**KCNK15-AS1**	-0.26	7.66E-01	8.04E-01
**RP11-1223D19.3**	-0.25	7.67E-01	8.04E-01
**CH17-437K3.1**	0.20	7.67E-01	8.04E-01
**ZFPM2**	-0.09	7.67E-01	8.04E-01
**LINC00877**	-0.19	7.67E-01	8.05E-01
**RNU7-77P**	-0.31	7.67E-01	8.05E-01
**RP11-554A11.9**	0.24	7.67E-01	8.05E-01
**PLXNA3**	0.07	7.67E-01	8.05E-01
**SPATA41**	0.16	7.68E-01	8.05E-01
**MAPK6**	0.05	7.68E-01	8.05E-01
**NAMPTP1**	0.17	7.68E-01	8.05E-01
**CENPH**	0.07	7.68E-01	8.06E-01
**FOXL2NB**	-0.32	7.69E-01	8.06E-01
**RP11-104G3.7**	0.30	7.69E-01	8.06E-01
**RP1-102E24.6**	-0.23	7.69E-01	8.06E-01
**TMC3**	-0.16	7.69E-01	8.06E-01
**SLC14A1**	-0.31	7.70E-01	8.07E-01
**XLOC_008785**	-0.43	7.70E-01	8.07E-01
**GSTA4**	0.06	7.70E-01	8.07E-01
**BAZ1A**	-0.04	7.70E-01	8.07E-01
**XLOC_008020**	0.16	7.70E-01	8.07E-01
**CLCN3P1**	-0.22	7.70E-01	8.07E-01
**TIRAP**	-0.05	7.70E-01	8.07E-01
**FAM180A**	0.12	7.70E-01	8.07E-01
**RP11-486A14.2**	0.25	7.70E-01	8.07E-01
**RP11-131L12.2**	-0.16	7.70E-01	8.07E-01
**RIC8B**	-0.04	7.70E-01	8.08E-01
**TTYH1**	-0.12	7.71E-01	8.08E-01
**MRPL53**	-0.11	7.71E-01	8.08E-01
**ALG1L6P**	0.14	7.71E-01	8.08E-01
**RP11-725P16.2**	-0.15	7.72E-01	8.09E-01
**RP13-401N8.1**	0.11	7.72E-01	8.09E-01
**TMEM72-AS1**	-0.19	7.72E-01	8.09E-01
**RP11-95O2.5**	0.25	7.73E-01	8.10E-01

**RARA-AS1**	0.13	7.73E-01	8.10E-01
**CTCF**	-0.03	7.73E-01	8.10E-01
**CSNK1A1**	-0.04	7.73E-01	8.10E-01
**MDH1**	-0.04	7.73E-01	8.10E-01
**AC005237.4**	-0.20	7.73E-01	8.10E-01
**ACAA2**	0.13	7.74E-01	8.10E-01
**GCNT1**	0.09	7.74E-01	8.11E-01
**SLC7A5P1**	-0.13	7.74E-01	8.11E-01
**HNRNPA1P16**	0.12	7.74E-01	8.11E-01
**MAEL**	-0.35	7.74E-01	8.11E-01
**XLOC_011519**	-0.59	7.74E-01	8.11E-01
**RP11-290F5.2**	0.21	7.74E-01	8.11E-01
**RP11-148K1.12**	0.22	7.74E-01	8.11E-01
**RP11-23P13.6**	-0.23	7.74E-01	8.11E-01
**NDUFA5**	0.05	7.74E-01	8.11E-01
**MAGI3**	-0.05	7.74E-01	8.11E-01
**G40988**	0.12	7.75E-01	8.11E-01
**RP11-291I6.2**	-0.36	7.75E-01	8.11E-01
**NAT10**	0.03	7.75E-01	8.11E-01
**AC005013.5**	0.24	7.75E-01	8.11E-01
**AP006222.2**	0.12	7.75E-01	8.12E-01
**G39663**	0.16	7.75E-01	8.12E-01
**C1orf56**	0.06	7.75E-01	8.12E-01
**FAM83C-AS1**	-0.13	7.75E-01	8.12E-01
**OR2C1**	0.23	7.75E-01	8.12E-01
**RP11-548H18.2**	0.12	7.76E-01	8.12E-01
**RP11-138E2.1**	-0.29	7.76E-01	8.12E-01
**FAM63A**	0.04	7.76E-01	8.12E-01
**AC003986.7**	-0.22	7.76E-01	8.12E-01
**RP11-253I19.4**	-0.28	7.76E-01	8.12E-01
**PIWIL2**	-0.12	7.76E-01	8.12E-01
**GYG2P1**	-0.20	7.76E-01	8.12E-01
**RNU2-65P**	-0.30	7.76E-01	8.13E-01
**MAGIX**	0.07	7.76E-01	8.13E-01
**ASAP1-IT2**	-0.16	7.77E-01	8.13E-01
**RP11-362J17.1**	0.22	7.77E-01	8.14E-01
**RP4-614O4.13**	-0.06	7.77E-01	8.14E-01
**FAM90A25P**	-0.38	7.77E-01	8.14E-01
**FMO6P**	-0.21	7.77E-01	8.14E-01
**HMBOX1-IT1**	0.25	7.78E-01	8.14E-01
**PARK2**	-0.07	7.78E-01	8.14E-01
**MAP7**	0.05	7.78E-01	8.14E-01
**ERP29P1**	0.28	7.78E-01	8.14E-01
**MYL12BP2**	-0.25	7.78E-01	8.14E-01
**G33789**	0.10	7.78E-01	8.14E-01

**TMEM45A**	-0.06	7.79E-01	8.15E-01
**HSP90AB3P**	-0.20	7.79E-01	8.15E-01
**RPL37P6**	-0.25	7.79E-01	8.15E-01
**G41275**	0.09	7.79E-01	8.15E-01
**COPS4**	0.04	7.80E-01	8.15E-01
**RP11-490B18.5**	0.15	7.80E-01	8.16E-01
**AF213884.2**	0.18	7.80E-01	8.16E-01
**NPBWR1**	-0.17	7.80E-01	8.16E-01
**MIR616**	-0.19	7.80E-01	8.16E-01
**AC104134.2**	-0.25	7.80E-01	8.16E-01
**RP11-299G20.2**	-0.11	7.80E-01	8.16E-01
**RP11-1399P15.1**	-0.23	7.80E-01	8.16E-01
**G30725**	0.31	7.80E-01	8.16E-01
**NLRP14**	0.28	7.81E-01	8.16E-01
**CD96**	0.13	7.81E-01	8.16E-01
**BANF1P1**	-0.21	7.81E-01	8.16E-01
**XLOC_000019**	0.18	7.81E-01	8.17E-01
**ACVR2B-AS1**	-0.10	7.81E-01	8.17E-01
**NAIP**	0.10	7.81E-01	8.17E-01
**ATP5C1**	-0.04	7.81E-01	8.17E-01
**AC027601.1**	-0.14	7.81E-01	8.17E-01
**PNPO**	-0.05	7.82E-01	8.17E-01
**RP11-730B22.1**	0.22	7.82E-01	8.17E-01
**ZNF706**	0.04	7.82E-01	8.17E-01
**MZF1-AS1**	-0.07	7.82E-01	8.18E-01
**RP11-21L19.1**	-0.33	7.82E-01	8.18E-01
**LA16c-313D11.9**	-0.28	7.83E-01	8.18E-01
**RADIL**	0.10	7.83E-01	8.18E-01
**RIMBP2**	0.07	7.83E-01	8.18E-01
**RP11-729I10.2**	0.38	7.83E-01	8.18E-01
**MAPK8IP2**	0.12	7.83E-01	8.18E-01
**RP11-46I8.3**	-0.40	7.83E-01	8.18E-01
**RNU1-11P**	-0.31	7.83E-01	8.18E-01
**RP11-791G15.2**	0.14	7.83E-01	8.19E-01
**RP11-867G23.8**	-0.07	7.83E-01	8.19E-01
**TGM6**	-0.38	7.84E-01	8.19E-01
**SGPP2**	0.07	7.84E-01	8.19E-01
**CCDC129**	0.19	7.84E-01	8.19E-01
**MTFR2**	-0.07	7.84E-01	8.19E-01
**BZW1P2**	-0.09	7.84E-01	8.19E-01
**KLF17P1**	-0.26	7.84E-01	8.19E-01
**RP11-525K10.3**	-0.14	7.84E-01	8.19E-01
**RP3-465N24.5**	-0.20	7.84E-01	8.19E-01
**RP11-357N13.3**	0.16	7.84E-01	8.19E-01
**CTA-797E19.1**	0.22	7.85E-01	8.20E-01

**FMR1-IT1**	-0.13	7.85E-01	8.20E-01
**A2MP1**	0.19	7.85E-01	8.20E-01
**CTB-51J22.1**	-0.26	7.86E-01	8.21E-01
**DNM3OS**	-0.12	7.86E-01	8.21E-01
**APPL2**	-0.04	7.86E-01	8.21E-01
**HM13-AS1**	-0.15	7.87E-01	8.22E-01
**RIPK2**	-0.04	7.87E-01	8.22E-01
**OR7E83P**	0.27	7.87E-01	8.22E-01
**CAHM**	0.10	7.87E-01	8.22E-01
**PGM5P4-AS1**	-0.18	7.87E-01	8.22E-01
**TMTC1**	-0.09	7.88E-01	8.22E-01
**MIR25**	0.16	7.88E-01	8.23E-01
**RP11-615I2.2**	0.10	7.88E-01	8.23E-01
**RN7SL500P**	-0.30	7.88E-01	8.23E-01
**JMY**	-0.05	7.88E-01	8.23E-01
**UQCRQ**	-0.04	7.88E-01	8.23E-01
**LUZP2**	-0.15	7.89E-01	8.23E-01
**RP11-318E3.9**	0.27	7.89E-01	8.23E-01
**KCNH4**	-0.25	7.89E-01	8.23E-01
**ASTN2**	-0.11	7.89E-01	8.23E-01
**DKFZp434J0226**	0.11	7.89E-01	8.23E-01
**MARK2P9**	0.18	7.89E-01	8.24E-01
**RP11-789C17.1**	-0.15	7.89E-01	8.24E-01
**TRBV5-4**	-0.37	7.89E-01	8.24E-01
**TOMM20P2**	0.20	7.89E-01	8.24E-01
**RPL21P10**	-0.16	7.89E-01	8.24E-01
**ERN1**	-0.03	7.89E-01	8.24E-01
**YIPF5**	0.04	7.89E-01	8.24E-01
**A1BG-AS1**	0.12	7.90E-01	8.24E-01
**NUP214**	0.04	7.90E-01	8.24E-01
**RP11-10A14.5**	-0.21	7.90E-01	8.24E-01
**RNU6-238P**	-0.19	7.90E-01	8.24E-01
**C1orf68**	-0.10	7.90E-01	8.25E-01
**POLN**	-0.06	7.90E-01	8.25E-01
**AC007193.6**	-0.13	7.90E-01	8.25E-01
**CAV2**	0.06	7.90E-01	8.25E-01
**RN7SL521P**	-0.21	7.91E-01	8.25E-01
**CCDC140**	-0.26	7.91E-01	8.25E-01
**RP11-455O6.9**	-0.21	7.91E-01	8.25E-01
**CDC37L1-AS1**	-0.10	7.91E-01	8.25E-01
**LINC01176**	-0.10	7.91E-01	8.25E-01
**HIPK4**	0.14	7.91E-01	8.25E-01
**TMEM136**	-0.06	7.91E-01	8.25E-01
**KLRC1**	-0.23	7.91E-01	8.25E-01
**XLOC_007617**	-0.13	7.91E-01	8.25E-01

**RP11-696N14.1**	-0.08	7.91E-01	8.26E-01
**LINC01606**	-0.52	7.92E-01	8.26E-01
**RP11-615I2.6**	-0.25	7.92E-01	8.26E-01
**RRM2**	0.07	7.92E-01	8.26E-01
**PSMD7**	0.03	7.93E-01	8.27E-01
**RNU6-407P**	-0.22	7.93E-01	8.27E-01
**TM2D3**	0.03	7.93E-01	8.27E-01
**AC006116.22**	0.26	7.93E-01	8.27E-01
**CTD-2015H6.3**	0.05	7.93E-01	8.27E-01
**PCOLCE-AS1**	-0.18	7.93E-01	8.27E-01
**CTD-2286N8.1**	0.19	7.94E-01	8.28E-01
**HMGB1P31**	-0.10	7.94E-01	8.28E-01
**FGF2**	0.11	7.94E-01	8.28E-01
**RASSF10**	-0.05	7.94E-01	8.28E-01
**SNRNP27**	0.04	7.94E-01	8.28E-01
**XLOC_008619**	-0.23	7.94E-01	8.28E-01
**DDX3Y**	-0.37	7.94E-01	8.28E-01
**C2orf76**	-0.04	7.94E-01	8.28E-01
**G25250**	-0.14	7.95E-01	8.29E-01
**ARHGAP26**	-0.04	7.95E-01	8.29E-01
**ARHGEF7-IT1**	-0.23	7.95E-01	8.29E-01
**MTCO2P12**	0.25	7.95E-01	8.29E-01
**RP11-92K2.2**	-0.11	7.95E-01	8.29E-01
**G40767**	-0.13	7.95E-01	8.29E-01
**AC004637.1**	-0.24	7.95E-01	8.29E-01
**MED12**	-0.03	7.95E-01	8.29E-01
**GRIK4**	-0.14	7.96E-01	8.30E-01
**MAPK13**	-0.04	7.96E-01	8.30E-01
**RP11-227G15.12**	0.12	7.96E-01	8.30E-01
**RP11-20G13.5**	-0.23	7.96E-01	8.30E-01
**AMBRA1**	0.02	7.97E-01	8.30E-01
**CTD-2619J13.13**	-0.05	7.97E-01	8.30E-01
**USP32P3**	-0.19	7.97E-01	8.31E-01
**FOXQ1**	0.08	7.97E-01	8.31E-01
**NSUN5P1**	0.09	7.97E-01	8.31E-01
**KLHL18**	0.05	7.97E-01	8.31E-01
**G42720**	0.20	7.98E-01	8.31E-01
**SDHAP3**	0.07	7.98E-01	8.31E-01
**CNTN3**	0.11	7.98E-01	8.31E-01
**SEPT4-AS1**	0.30	7.98E-01	8.31E-01
**RP11-181G12.4**	0.30	7.98E-01	8.31E-01
**PMEL**	-0.11	7.98E-01	8.31E-01
**RP11-680G24.4**	-0.14	7.98E-01	8.31E-01
**RP11-426L16.3**	0.21	7.98E-01	8.31E-01
**C9orf139**	0.14	7.99E-01	8.32E-01

**RP5-1017F8.2**	0.25	7.99E-01	8.32E-01
**AC012358.8**	0.10	7.99E-01	8.32E-01
**HNRNPA1P48**	-0.07	7.99E-01	8.32E-01
**CYP26B1**	0.05	7.99E-01	8.32E-01
**ZNF667-AS1**	-0.08	8.00E-01	8.33E-01
**RP11-187C18.3**	-0.18	8.00E-01	8.33E-01
**TRGV4**	-0.29	8.00E-01	8.33E-01
**AC132008.1**	-0.22	8.01E-01	8.34E-01
**RP3-412A9.17**	0.17	8.01E-01	8.34E-01
**C3orf33**	0.06	8.01E-01	8.34E-01
**AC025171.1**	-0.10	8.01E-01	8.34E-01
**XLOC_008979**	0.13	8.01E-01	8.34E-01
**PPP1R8**	-0.03	8.01E-01	8.34E-01
**RP11-165J3.6**	-0.11	8.02E-01	8.35E-01
**RP11-445N20.2**	-0.28	8.02E-01	8.35E-01
**FAM86B3P**	0.08	8.02E-01	8.35E-01
**RP13-516M14.4**	0.19	8.02E-01	8.35E-01
**PMS2P4**	0.05	8.02E-01	8.35E-01
**MYO15A**	-0.11	8.02E-01	8.35E-01
**C6orf25**	-0.07	8.02E-01	8.35E-01
**RP11-321F8.4**	0.15	8.03E-01	8.36E-01
**CACNA1H**	-0.18	8.03E-01	8.36E-01
**SCARA3**	-0.06	8.03E-01	8.36E-01
**CACTIN-AS1**	0.17	8.04E-01	8.37E-01
**PROC**	0.14	8.04E-01	8.37E-01
**RAD51D**	0.04	8.04E-01	8.37E-01
**TSPEAR-AS1**	-0.19	8.05E-01	8.37E-01
**CTD-2591A1.1**	0.20	8.05E-01	8.37E-01
**CTD-2378E12.1**	-0.13	8.05E-01	8.37E-01
**PCID2**	-0.03	8.05E-01	8.37E-01
**RP1-34B20.4**	-0.31	8.05E-01	8.38E-01
**CYP4V2**	0.07	8.05E-01	8.38E-01
**FCRLA**	0.13	8.06E-01	8.38E-01
**FAM209B**	-0.19	8.06E-01	8.38E-01
**CCDC82**	-0.05	8.06E-01	8.39E-01
**TMEM145**	-0.15	8.06E-01	8.39E-01
**RP11-256L11.3**	0.32	8.06E-01	8.39E-01
**MITD1**	0.03	8.07E-01	8.39E-01
**RP11-867G23.1**	-0.22	8.07E-01	8.39E-01
**PABPC3**	0.16	8.07E-01	8.39E-01
**ALX4**	-0.13	8.07E-01	8.39E-01
**RP11-327J17.9**	-0.23	8.07E-01	8.39E-01
**TANGO2**	-0.04	8.07E-01	8.39E-01
**SLC35E2B**	-0.06	8.07E-01	8.39E-01
**RP11-430G17.3**	-0.30	8.07E-01	8.39E-01

**RP11-706O15.5**	-0.41	8.07E-01	8.39E-01
**DNAJC19**	-0.03	8.07E-01	8.39E-01
**ABCC8**	-0.31	8.08E-01	8.40E-01
**RP11-354E11.2**	-0.13	8.08E-01	8.40E-01
**LINC00284**	-0.26	8.08E-01	8.40E-01
**ITGA4**	0.10	8.08E-01	8.40E-01
**NUP50-AS1**	-0.04	8.08E-01	8.40E-01
**LCE2C**	0.08	8.08E-01	8.41E-01
**CASP12**	0.12	8.09E-01	8.41E-01
**NT5C3A**	0.06	8.09E-01	8.41E-01
**PAN2**	0.05	8.09E-01	8.41E-01
**AC011738.4**	0.21	8.09E-01	8.41E-01
**CCNT2-AS1**	-0.11	8.09E-01	8.41E-01
**SRRM4**	-0.28	8.09E-01	8.42E-01
**HKR1**	-0.03	8.10E-01	8.42E-01
**LINC01415**	-0.08	8.10E-01	8.42E-01
**RP3-428L16.1**	-0.19	8.10E-01	8.42E-01
**RP11-467J12.4**	0.14	8.10E-01	8.42E-01
**DNAL1**	0.06	8.10E-01	8.42E-01
**MFSD5**	-0.04	8.11E-01	8.43E-01
**CPO**	0.18	8.11E-01	8.43E-01
**TYW1**	-0.02	8.11E-01	8.43E-01
**CTB-133G6.2**	0.16	8.11E-01	8.43E-01
**DERL3**	-0.08	8.11E-01	8.43E-01
**G34931**	-0.11	8.11E-01	8.43E-01
**RP11-271M24.2**	-0.23	8.12E-01	8.43E-01
**PIWIL4**	-0.07	8.12E-01	8.43E-01
**NACA**	-0.04	8.12E-01	8.44E-01
**HMGN1P7**	-0.14	8.12E-01	8.44E-01
**KCNJ14**	-0.09	8.12E-01	8.44E-01
**FASLG**	0.17	8.12E-01	8.44E-01
**RP11-351M8.1**	0.22	8.12E-01	8.44E-01
**TTC9**	0.04	8.12E-01	8.44E-01
**HCAR3**	0.07	8.12E-01	8.44E-01
**PRB1**	-0.23	8.13E-01	8.44E-01
**AC007182.6**	-0.09	8.13E-01	8.45E-01
**SLC22A14**	0.25	8.13E-01	8.45E-01
**RANBP17**	-0.09	8.13E-01	8.45E-01
**RP11-342D11.2**	-0.31	8.13E-01	8.45E-01
**SOX21**	0.04	8.13E-01	8.45E-01
**RP11-459I19.1**	-0.17	8.13E-01	8.45E-01
**HTATSF1**	-0.03	8.14E-01	8.45E-01
**RP11-434D2.11**	-0.21	8.14E-01	8.45E-01
**SNRPD3**	0.04	8.14E-01	8.46E-01
**RP11-416I2.1**	-0.13	8.14E-01	8.46E-01

**G28030**	-0.28	8.15E-01	8.46E-01
**C12orf80**	-0.21	8.15E-01	8.46E-01
**MIR3936**	-0.13	8.15E-01	8.46E-01
**TIGIT**	-0.12	8.15E-01	8.46E-01
**IL7R**	0.09	8.15E-01	8.47E-01
**RP11-264M12.2**	0.16	8.16E-01	8.47E-01
**SELE**	0.10	8.16E-01	8.47E-01
**RP5-1139B12.3**	0.13	8.16E-01	8.47E-01
**TIMP4**	0.18	8.16E-01	8.47E-01
**MTND5P1**	-0.19	8.16E-01	8.47E-01
**RP11-214O1.2**	-0.09	8.16E-01	8.47E-01
**G42645**	-0.11	8.16E-01	8.47E-01
**SMPD3**	-0.07	8.17E-01	8.48E-01
**PDE8A**	0.03	8.17E-01	8.48E-01
**FSD1**	0.30	8.17E-01	8.48E-01
**MADD**	-0.03	8.17E-01	8.48E-01
**FBXO43**	-0.10	8.17E-01	8.48E-01
**RP11-466C23.5**	-0.24	8.17E-01	8.48E-01
**RP11-803B1.8**	-0.11	8.17E-01	8.48E-01
**MIR4768**	0.26	8.18E-01	8.49E-01
**KLF15**	0.10	8.18E-01	8.49E-01
**RP3-391O22.3**	-0.24	8.18E-01	8.49E-01
**CLEC18A**	-0.22	8.18E-01	8.49E-01
**RP11-288G3.4**	0.23	8.18E-01	8.49E-01
**RNU6-969P**	-0.20	8.18E-01	8.49E-01
**FRMD6-AS1**	-0.07	8.18E-01	8.49E-01
**PHOSPHO1**	-0.07	8.18E-01	8.49E-01
**XLOC_006699**	-0.28	8.18E-01	8.49E-01
**NUDT16P1**	-0.06	8.19E-01	8.49E-01
**RP11-585P4.6**	-0.11	8.19E-01	8.50E-01
**FRMPD1**	0.08	8.20E-01	8.50E-01
**WDR72**	-0.11	8.20E-01	8.50E-01
**VKORC1**	0.05	8.20E-01	8.50E-01
**PRRG4**	0.05	8.20E-01	8.50E-01
**AC017060.1**	0.22	8.20E-01	8.50E-01
**ALB**	-0.16	8.20E-01	8.50E-01
**SYN3**	0.16	8.20E-01	8.50E-01
**RP11-481J13.1**	0.20	8.20E-01	8.51E-01
**MYBPC2**	-0.20	8.20E-01	8.51E-01
**SEMA4D**	-0.04	8.21E-01	8.51E-01
**GTF2E1**	0.03	8.21E-01	8.51E-01
**RNF216-IT1**	-0.25	8.21E-01	8.51E-01
**SOX9-AS1**	0.12	8.21E-01	8.51E-01
**AP001437.1**	0.26	8.21E-01	8.52E-01
**HMGB1P21**	0.21	8.21E-01	8.52E-01

**ASCL5**	0.24	8.21E-01	8.52E-01
**SNX18P13**	0.32	8.22E-01	8.52E-01
**RP11-492E3.2**	0.20	8.22E-01	8.52E-01
**SCGB1D2**	0.25	8.22E-01	8.52E-01
**G30964**	-0.15	8.22E-01	8.52E-01
**EGFLAM-AS4**	-0.31	8.22E-01	8.52E-01
**ACAD8**	-0.04	8.22E-01	8.53E-01
**ANXA8**	-0.10	8.23E-01	8.53E-01
**UBASH3A**	-0.12	8.23E-01	8.53E-01
**XLOC_006132**	-0.19	8.23E-01	8.53E-01
**RP11-7K24.3**	0.08	8.23E-01	8.53E-01
**RP11-660L16.2**	-0.14	8.23E-01	8.53E-01
**YAE1D1**	0.03	8.23E-01	8.53E-01
**CTD-2515A14.1**	0.12	8.23E-01	8.53E-01
**AC073316.1**	-0.20	8.23E-01	8.53E-01
**AFG3L1P**	0.05	8.23E-01	8.53E-01
**RP11-350J20.9**	-0.12	8.24E-01	8.54E-01
**ZNF69**	-0.07	8.24E-01	8.54E-01
**CCDC80**	0.09	8.24E-01	8.54E-01
**CHEK1**	-0.04	8.24E-01	8.54E-01
**ZNF354A**	0.03	8.24E-01	8.54E-01
**MLIP**	0.08	8.24E-01	8.54E-01
**KIF12**	0.12	8.24E-01	8.54E-01
**ADAMTS5**	-0.10	8.25E-01	8.54E-01
**RP11-783K16.13**	0.11	8.25E-01	8.54E-01
**FBXO27**	-0.06	8.25E-01	8.55E-01
**AC008753.6**	0.19	8.25E-01	8.55E-01
**ADAL**	0.04	8.26E-01	8.55E-01
**PCNP**	0.03	8.26E-01	8.56E-01
**SLC6A4**	0.07	8.26E-01	8.56E-01
**FCF1P7**	0.11	8.26E-01	8.56E-01
**UBIAD1**	-0.08	8.27E-01	8.56E-01
**TRIM61**	0.20	8.27E-01	8.57E-01
**RP11-135F9.3**	0.03	8.27E-01	8.57E-01
**RPL5P18**	-0.21	8.27E-01	8.57E-01
**CEBPZOS**	0.03	8.27E-01	8.57E-01
**RP11-522M21.3**	0.23	8.27E-01	8.57E-01
**ELOVL2**	0.10	8.28E-01	8.57E-01
**RP1-102E24.8**	-0.09	8.28E-01	8.57E-01
**RP1-59D14.5**	-0.10	8.28E-01	8.57E-01
**ABCG2**	-0.07	8.28E-01	8.57E-01
**GALR1**	-0.18	8.28E-01	8.57E-01
**C11orf21**	-0.09	8.28E-01	8.58E-01
**RN7SL543P**	-0.22	8.28E-01	8.58E-01
**ACYP2**	0.03	8.28E-01	8.58E-01

**AC006026.13**	-0.12	8.29E-01	8.58E-01
**RP11-1280N14.3**	-0.09	8.29E-01	8.58E-01
**UBE2V2**	-0.03	8.29E-01	8.58E-01
**MIR93**	-0.22	8.29E-01	8.59E-01
**TRIM10**	0.13	8.30E-01	8.59E-01
**DHRS4L1**	0.06	8.30E-01	8.59E-01
**ATP8B4**	-0.06	8.30E-01	8.59E-01
**RP3-486I3.4**	-0.21	8.30E-01	8.60E-01
**PSORS1C1**	-0.06	8.30E-01	8.60E-01
**RP11-569A11.1**	-0.19	8.31E-01	8.60E-01
**COX6C**	-0.04	8.31E-01	8.60E-01
**GS1-124K5.9**	0.27	8.31E-01	8.60E-01
**FMN2**	-0.14	8.31E-01	8.60E-01
**INPP4A**	-0.02	8.31E-01	8.60E-01
**DPF3**	-0.05	8.32E-01	8.61E-01
**DONSON**	0.04	8.32E-01	8.61E-01
**ELL2**	0.03	8.32E-01	8.61E-01
**G26897**	0.11	8.32E-01	8.61E-01
**DPY19L1**	-0.04	8.32E-01	8.61E-01
**ACO1**	0.07	8.32E-01	8.61E-01
**OOEP**	0.12	8.32E-01	8.61E-01
**RP11-115H13.1**	0.11	8.32E-01	8.61E-01
**S100A1**	-0.11	8.32E-01	8.61E-01
**RN7SL270P**	-0.24	8.33E-01	8.62E-01
**RP11-388C12.8**	-0.16	8.33E-01	8.62E-01
**HDAC10**	-0.10	8.33E-01	8.62E-01
**LINC00265**	-0.06	8.33E-01	8.62E-01
**TRBV28**	0.20	8.33E-01	8.62E-01
**MGAM2**	0.30	8.33E-01	8.62E-01
**RP11-454K7.1**	-0.18	8.33E-01	8.62E-01
**CTC-523E23.3**	-0.07	8.34E-01	8.63E-01
**KREMEN1**	0.03	8.34E-01	8.63E-01
**GARNL3**	-0.03	8.34E-01	8.63E-01
**RP11-736K20.6**	-0.13	8.34E-01	8.63E-01
**TMEM156**	-0.14	8.35E-01	8.63E-01
**AL163953.3**	0.09	8.35E-01	8.63E-01
**FAM86C1**	0.05	8.35E-01	8.63E-01
**CTD-2524L6.3**	0.13	8.35E-01	8.64E-01
**OR7E108P**	-0.10	8.35E-01	8.64E-01
**XLOC_006901**	0.16	8.35E-01	8.64E-01
**RNU6-1011P**	-0.15	8.36E-01	8.64E-01
**G35278**	-0.13	8.36E-01	8.64E-01
**RP11-638I2.4**	-0.25	8.36E-01	8.64E-01
**RP5-997D16.2**	0.04	8.36E-01	8.64E-01
**LINC01265**	0.13	8.36E-01	8.64E-01

**THOC5**	-0.01	8.36E-01	8.65E-01
**HERC2P4**	0.16	8.36E-01	8.65E-01
**RP11-712B9.2**	0.06	8.36E-01	8.65E-01
**G32325**	-0.21	8.37E-01	8.65E-01
**GOLGA8M**	0.20	8.37E-01	8.65E-01
**GRAMD4**	-0.03	8.37E-01	8.65E-01
**AC096558.1**	0.15	8.37E-01	8.65E-01
**FOXI2**	-0.15	8.37E-01	8.65E-01
**AC114765.2**	-0.23	8.37E-01	8.65E-01
**SNX29**	0.03	8.37E-01	8.65E-01
**TMEM236**	-0.11	8.37E-01	8.65E-01
**RP11-488I20.9**	-0.27	8.37E-01	8.65E-01
**TRHDE**	-0.12	8.37E-01	8.66E-01
**SLC6A12**	0.10	8.38E-01	8.66E-01
**HMGN2P28**	-0.18	8.38E-01	8.66E-01
**UNC5C**	-0.07	8.38E-01	8.66E-01
**RP1-221C16.8**	-0.13	8.38E-01	8.66E-01
**NDUFA9P1**	0.12	8.38E-01	8.66E-01
**RP11-317J9.1**	0.21	8.38E-01	8.66E-01
**OXSR1**	0.02	8.38E-01	8.66E-01
**AF064860.5**	0.30	8.38E-01	8.66E-01
**PBK**	-0.05	8.38E-01	8.66E-01
**RP11-367G6.3**	-0.20	8.39E-01	8.66E-01
**KLK7**	0.05	8.39E-01	8.67E-01
**CTB-32O4.3**	0.19	8.39E-01	8.67E-01
**RMRPP2**	0.07	8.39E-01	8.67E-01
**RP11-414H17.5**	-0.16	8.39E-01	8.67E-01
**ABI1**	-0.02	8.39E-01	8.67E-01
**FAM161B**	0.03	8.39E-01	8.67E-01
**RP11-314A20.1**	-0.23	8.39E-01	8.67E-01
**RP11-435O5.7**	-0.11	8.40E-01	8.67E-01
**NCMAP**	-0.14	8.40E-01	8.67E-01
**RP11-296E3.2**	-0.21	8.40E-01	8.68E-01
**AC005363.11**	-0.22	8.40E-01	8.68E-01
**MEST**	-0.08	8.40E-01	8.68E-01
**ASB12**	0.19	8.40E-01	8.68E-01
**RP11-68I3.5**	-0.14	8.40E-01	8.68E-01
**RP11-15F12.6**	0.18	8.40E-01	8.68E-01
**PDCL3P4**	-0.10	8.41E-01	8.68E-01
**RP11-671E7.1**	-0.16	8.41E-01	8.68E-01
**GGPS1**	0.03	8.41E-01	8.69E-01
**ODF2L**	0.04	8.41E-01	8.69E-01
**RP11-512F24.1**	0.16	8.41E-01	8.69E-01
**RP11-145M9.6**	-0.15	8.41E-01	8.69E-01
**SERP1**	0.03	8.42E-01	8.69E-01

**G33807**	0.14	8.42E-01	8.69E-01
**ANGPT4**	0.09	8.42E-01	8.69E-01
**MIR4263**	0.14	8.42E-01	8.69E-01
**RP11-428L9.2**	0.24	8.42E-01	8.69E-01
**RP3-508I15.21**	-0.11	8.42E-01	8.69E-01
**RP11-404F10.2**	0.19	8.42E-01	8.69E-01
**DNAH17**	0.08	8.42E-01	8.69E-01
**ZCCHC14**	-0.02	8.42E-01	8.69E-01
**DOCK4**	-0.05	8.42E-01	8.69E-01
**FAM86FP**	0.06	8.42E-01	8.69E-01
**U73166.2**	-0.10	8.43E-01	8.70E-01
**PFKFB1**	0.08	8.43E-01	8.70E-01
**RP11-876N24.4**	0.06	8.43E-01	8.70E-01
**ZBED5-AS1**	-0.04	8.43E-01	8.70E-01
**RP11-298D21.3**	-0.14	8.43E-01	8.70E-01
**DUSP5P1**	-0.15	8.44E-01	8.70E-01
**NUP62CL**	-0.05	8.44E-01	8.70E-01
**PCP4**	0.14	8.44E-01	8.71E-01
**AC068831.6**	-0.09	8.44E-01	8.71E-01
**TCAP**	0.07	8.44E-01	8.71E-01
**STAG3L2**	0.05	8.45E-01	8.71E-01
**PRKAG3**	0.17	8.45E-01	8.72E-01
**CPVL**	0.07	8.45E-01	8.72E-01
**CTD-2024I7.18**	0.11	8.45E-01	8.72E-01
**TUBD1**	0.03	8.46E-01	8.72E-01
**NGRN**	0.03	8.46E-01	8.72E-01
**LINC01108**	0.21	8.46E-01	8.72E-01
**CTD-2292P10.4**	0.08	8.46E-01	8.73E-01
**LA16c-429E7.1**	-0.10	8.46E-01	8.73E-01
**C12orf54**	0.11	8.46E-01	8.73E-01
**WARS2-IT1**	0.09	8.46E-01	8.73E-01
**GTF2H2C**	0.05	8.46E-01	8.73E-01
**DSN1**	0.03	8.46E-01	8.73E-01
**PART1**	-0.04	8.46E-01	8.73E-01
**KRTAP5-3**	0.38	8.46E-01	8.73E-01
**HSD17B3**	-0.17	8.47E-01	8.73E-01
**LINC01561**	0.19	8.47E-01	8.73E-01
**DCTN6**	-0.03	8.47E-01	8.73E-01
**RP11-586K2.1**	-0.23	8.47E-01	8.73E-01
**RP4-671O14.7**	-0.23	8.47E-01	8.73E-01
**RP11-554D15.3**	0.17	8.47E-01	8.73E-01
**TNFRSF10A**	-0.04	8.47E-01	8.73E-01
**OR2A20P**	0.14	8.47E-01	8.73E-01
**DPH5**	0.03	8.47E-01	8.74E-01
**RPL3P7**	-0.18	8.47E-01	8.74E-01

**UBXN10-AS1**	0.17	8.47E-01	8.74E-01
**SPINK2**	-0.14	8.47E-01	8.74E-01
**AC116035.1**	0.17	8.48E-01	8.74E-01
**RPS4X**	0.03	8.48E-01	8.74E-01
**C12orf73**	-0.03	8.48E-01	8.74E-01
**RP11-314C16.1**	0.09	8.48E-01	8.74E-01
**ACSM4**	-0.13	8.48E-01	8.74E-01
**CTC-332L22.1**	0.13	8.48E-01	8.74E-01
**RP11-436G20.1**	-0.31	8.48E-01	8.74E-01
**RP11-314A20.2**	0.21	8.48E-01	8.74E-01
**PDXDC1**	0.02	8.48E-01	8.74E-01
**FAM71D**	-0.12	8.49E-01	8.74E-01
**FAM19A5**	0.07	8.49E-01	8.75E-01
**PREP**	-0.03	8.49E-01	8.75E-01
**RAB40A**	0.08	8.49E-01	8.75E-01
**GOLGA8O**	-0.12	8.49E-01	8.75E-01
**AC016995.3**	0.07	8.50E-01	8.76E-01
**AC008171.1**	-0.20	8.50E-01	8.76E-01
**KCNH2**	0.12	8.50E-01	8.76E-01
**RP11-120K24.5**	-0.14	8.50E-01	8.76E-01
**PIWIL1**	0.17	8.51E-01	8.76E-01
**RP11-407N17.5**	-0.09	8.51E-01	8.76E-01
**NME1**	-0.03	8.51E-01	8.76E-01
**MYO1E**	-0.02	8.51E-01	8.77E-01
**CTB-113P19.4**	-0.07	8.51E-01	8.77E-01
**SNF8**	-0.02	8.51E-01	8.77E-01
**PPEF1**	0.08	8.51E-01	8.77E-01
**CCDC183**	0.07	8.51E-01	8.77E-01
**CBX1P1**	0.23	8.51E-01	8.77E-01
**PITPNA-AS1**	-0.11	8.51E-01	8.77E-01
**RP11-295D4.3**	-0.11	8.52E-01	8.77E-01
**NUFIP1**	0.03	8.52E-01	8.77E-01
**CCDC13-AS1**	-0.17	8.52E-01	8.77E-01
**CTC-523E23.5**	0.09	8.52E-01	8.77E-01
**TPMT**	-0.03	8.52E-01	8.77E-01
**CYP4F2**	-0.14	8.52E-01	8.78E-01
**IL1RAPL2**	0.22	8.52E-01	8.78E-01
**SLCO3A1**	0.03	8.52E-01	8.78E-01
**RP11-1060J15.5**	-0.20	8.52E-01	8.78E-01
**WASH5P**	0.09	8.53E-01	8.78E-01
**LINC01361**	-0.19	8.53E-01	8.79E-01
**AC092431.3**	0.14	8.54E-01	8.79E-01
**BRD1**	-0.02	8.54E-01	8.79E-01
**ACTG1P17**	-0.12	8.54E-01	8.79E-01
**G36224**	-0.12	8.54E-01	8.79E-01

**HOXA5**	0.07	8.54E-01	8.79E-01
**CEND1**	0.08	8.54E-01	8.79E-01
**CNGA4**	-0.19	8.54E-01	8.79E-01
**SIGLEC17P**	0.12	8.54E-01	8.79E-01
**ZNF592**	-0.02	8.54E-01	8.79E-01
**RBMS3**	-0.06	8.54E-01	8.79E-01
**RPS10L**	0.10	8.54E-01	8.79E-01
**RP11-977G19.5**	-0.07	8.55E-01	8.80E-01
**CADM1**	0.04	8.55E-01	8.80E-01
**C10orf82**	0.10	8.55E-01	8.80E-01
**AC005224.2**	-0.08	8.55E-01	8.80E-01
**IFI30**	-0.10	8.55E-01	8.80E-01
**TNNI1**	-0.05	8.55E-01	8.80E-01
**RP11-47I22.2**	0.09	8.56E-01	8.80E-01
**CHRNA5**	0.09	8.56E-01	8.80E-01
**RP11-153K11.3**	-0.23	8.56E-01	8.80E-01
**FAM157A**	0.08	8.56E-01	8.80E-01
**LYRM2**	0.02	8.56E-01	8.81E-01
**PRSS50**	-0.19	8.56E-01	8.81E-01
**RP11-303E16.5**	-0.19	8.56E-01	8.81E-01
**APCDD1L**	-0.07	8.57E-01	8.81E-01
**TACC1**	-0.04	8.57E-01	8.81E-01
**NDUFS4**	0.03	8.57E-01	8.81E-01
**INTS4**	-0.02	8.57E-01	8.81E-01
**XLOC_005046**	-0.17	8.57E-01	8.82E-01
**KIN**	-0.02	8.57E-01	8.82E-01
**PGPEP1L**	-0.15	8.57E-01	8.82E-01
**XLOC_007047**	0.17	8.58E-01	8.82E-01
**KANSL3**	0.02	8.58E-01	8.82E-01
**RP11-176H8.1**	-0.08	8.58E-01	8.82E-01
**CKMT2**	-0.07	8.58E-01	8.83E-01
**LBX2**	0.09	8.58E-01	8.83E-01
**LINC00330**	-0.21	8.59E-01	8.83E-01
**RPS4XP1**	-0.17	8.59E-01	8.83E-01
**RPAIN**	-0.04	8.59E-01	8.83E-01
**FAM210A**	0.02	8.59E-01	8.83E-01
**DDX11-AS1**	0.09	8.59E-01	8.84E-01
**ETFA**	-0.02	8.59E-01	8.84E-01
**IQGAP3**	-0.04	8.59E-01	8.84E-01
**RP11-598F7.4**	0.18	8.60E-01	8.84E-01
**AC068831.10**	-0.11	8.60E-01	8.84E-01
**RUBCN**	0.02	8.60E-01	8.84E-01
**FARSB**	0.02	8.60E-01	8.84E-01
**CTD-2224J9.8**	0.18	8.60E-01	8.84E-01
**TARS**	-0.03	8.60E-01	8.84E-01

**CERS3**	-0.03	8.60E-01	8.84E-01
**AP000344.4**	0.10	8.60E-01	8.84E-01
**STIM2**	0.02	8.61E-01	8.85E-01
**DACH1**	-0.07	8.61E-01	8.85E-01
**PIK3IP1-AS1**	0.09	8.61E-01	8.85E-01
**COA1**	-0.02	8.61E-01	8.85E-01
**PCDHGB2**	-0.05	8.61E-01	8.85E-01
**ZNF7**	0.02	8.61E-01	8.85E-01
**ART4**	-0.09	8.62E-01	8.86E-01
**CTC-439O9.1**	-0.15	8.62E-01	8.86E-01
**PEAK1**	0.03	8.63E-01	8.86E-01
**RP5-1115A15.2**	0.17	8.63E-01	8.86E-01
**DDX5**	0.02	8.63E-01	8.87E-01
**G2196**	0.16	8.63E-01	8.87E-01
**DNM1**	0.07	8.63E-01	8.87E-01
**RP1-159A19.3**	0.15	8.63E-01	8.87E-01
**ZNF846**	0.03	8.63E-01	8.87E-01
**C1QTNF1-AS1**	0.14	8.63E-01	8.87E-01
**TACR3**	-0.17	8.64E-01	8.87E-01
**KIAA0556**	-0.02	8.64E-01	8.87E-01
**TEN1-CDK3**	0.08	8.64E-01	8.87E-01
**PSMA3**	0.02	8.64E-01	8.87E-01
**AC093159.1**	0.16	8.64E-01	8.88E-01
**G8415**	0.15	8.64E-01	8.88E-01
**AC141928.1**	0.09	8.64E-01	8.88E-01
**RP1**	0.15	8.64E-01	8.88E-01
**RPL7AP28**	-0.12	8.64E-01	8.88E-01
**ZNF222**	0.03	8.64E-01	8.88E-01
**SBDS**	-0.03	8.64E-01	8.88E-01
**PPP1CC**	-0.02	8.65E-01	8.88E-01
**CYP26C1**	0.17	8.65E-01	8.88E-01
**MRPL3**	-0.03	8.65E-01	8.88E-01
**RP13-104F24.3**	0.05	8.65E-01	8.88E-01
**RP11-770E5.1**	-0.08	8.65E-01	8.89E-01
**G25996**	0.12	8.65E-01	8.89E-01
**FAM72B**	0.05	8.66E-01	8.89E-01
**RNF20**	0.02	8.66E-01	8.89E-01
**NPM1P39**	0.16	8.66E-01	8.89E-01
**LMO7-AS1**	0.10	8.66E-01	8.89E-01
**NUAK2**	0.04	8.66E-01	8.90E-01
**AP001610.5**	-0.16	8.67E-01	8.90E-01
**CRYM**	-0.05	8.67E-01	8.90E-01
**NSL1**	-0.03	8.67E-01	8.90E-01
**DFNB59**	-0.06	8.67E-01	8.90E-01
**CTD-2537I9.18**	-0.09	8.67E-01	8.90E-01

**RP11-277A4.4**	0.08	8.67E-01	8.90E-01
**HUS1**	-0.02	8.67E-01	8.90E-01
**CTB-31O20.4**	-0.07	8.67E-01	8.90E-01
**SOX1**	-0.08	8.67E-01	8.90E-01
**C14orf80**	-0.05	8.67E-01	8.90E-01
**RNU6ATAC39P**	-0.14	8.67E-01	8.90E-01
**ANKRD2**	0.07	8.68E-01	8.90E-01
**UBE2R2-AS1**	0.07	8.68E-01	8.90E-01
**BLOC1S6**	0.02	8.68E-01	8.91E-01
**AC006369.2**	0.16	8.68E-01	8.91E-01
**TSR1**	-0.02	8.68E-01	8.91E-01
**ATE1-AS1**	0.09	8.68E-01	8.91E-01
**OR7E22P**	-0.15	8.68E-01	8.91E-01
**NAT1**	0.03	8.68E-01	8.91E-01
**RP11-867G23.12**	-0.11	8.69E-01	8.91E-01
**RP11-76P2.4**	0.13	8.69E-01	8.91E-01
**RP11-99E15.2**	0.19	8.69E-01	8.92E-01
**MIR155HG**	-0.07	8.70E-01	8.92E-01
**KIRREL3**	0.08	8.70E-01	8.92E-01
**AC002454.1**	-0.15	8.70E-01	8.92E-01
**XLOC_008855**	0.09	8.70E-01	8.92E-01
**XLOC_010097**	-0.06	8.70E-01	8.92E-01
**RPL3L**	-0.08	8.71E-01	8.93E-01
**SNHG21**	0.07	8.71E-01	8.93E-01
**MRGPRX1**	0.23	8.71E-01	8.93E-01
**LINC00535**	0.18	8.71E-01	8.94E-01
**CTD-3252C9.2**	-0.15	8.71E-01	8.94E-01
**EPO**	0.09	8.72E-01	8.94E-01
**RP11-227G15.8**	0.09	8.72E-01	8.94E-01
**BDKRB1**	0.04	8.72E-01	8.95E-01
**GCSHP5**	0.13	8.73E-01	8.95E-01
**RAB2A**	0.03	8.73E-01	8.95E-01
**TMEM221**	0.07	8.73E-01	8.95E-01
**MTA2**	-0.02	8.73E-01	8.96E-01
**KANK4**	0.07	8.74E-01	8.96E-01
**NOP14**	-0.02	8.74E-01	8.96E-01
**XLOC_002973**	-0.15	8.74E-01	8.96E-01
**RP5-1116H23.5**	0.15	8.74E-01	8.96E-01
**LINC00266-1**	0.15	8.74E-01	8.96E-01
**PLEKHG5**	0.04	8.74E-01	8.96E-01
**MAP7D2**	0.05	8.75E-01	8.96E-01
**G16268**	-0.06	8.75E-01	8.97E-01
**HJURP**	0.04	8.75E-01	8.97E-01
**RPL7AP26**	-0.17	8.75E-01	8.97E-01
**GLDC**	-0.15	8.75E-01	8.97E-01

**RN7SL721P**	-0.12	8.75E-01	8.97E-01
**RP3-510D11.4**	0.13	8.75E-01	8.97E-01
**RP11-650L12.2**	0.08	8.75E-01	8.97E-01
**RP11-626G11.6**	-0.05	8.75E-01	8.97E-01
**RSRC2**	-0.02	8.75E-01	8.97E-01
**RNU7-45P**	-0.08	8.75E-01	8.97E-01
**HNRNPA1P10**	0.05	8.76E-01	8.97E-01
**AANAT**	-0.10	8.76E-01	8.98E-01
**ZNF554**	0.03	8.76E-01	8.98E-01
**TMOD1**	0.04	8.76E-01	8.98E-01
**CTD-2396E7.11**	-0.03	8.76E-01	8.98E-01
**EIF1AY**	0.17	8.76E-01	8.98E-01
**RP11-372K14.2**	-0.05	8.77E-01	8.98E-01
**XLOC_005166**	0.15	8.77E-01	8.98E-01
**IPCEF1**	-0.04	8.77E-01	8.98E-01
**RP11-359E3.4**	-0.05	8.77E-01	8.98E-01
**EID1**	0.04	8.77E-01	8.98E-01
**RTN4RL1**	-0.03	8.77E-01	8.99E-01
**RP11-108M9.5**	0.11	8.77E-01	8.99E-01
**HERC2P9**	-0.03	8.78E-01	8.99E-01
**SLC37A1**	0.03	8.78E-01	8.99E-01
**KRT222**	-0.11	8.78E-01	8.99E-01
**ZC3H15**	-0.02	8.78E-01	8.99E-01
**TCEAL8**	0.03	8.78E-01	8.99E-01
**GALNT15**	0.10	8.78E-01	8.99E-01
**G8687**	-0.14	8.78E-01	8.99E-01
**AC092573.2**	-0.07	8.79E-01	9.00E-01
**NOMO3**	-0.06	8.79E-01	9.00E-01
**XLOC_001907**	-0.15	8.79E-01	9.00E-01
**EXOSC9**	-0.02	8.79E-01	9.00E-01
**ACTN2**	0.09	8.79E-01	9.00E-01
**STK10**	0.03	8.79E-01	9.00E-01
**XRCC3**	0.04	8.79E-01	9.00E-01
**ZNF341-AS1**	-0.11	8.79E-01	9.00E-01
**B3GNT5**	0.03	8.79E-01	9.00E-01
**AC004069.2**	-0.10	8.80E-01	9.01E-01
**G5553**	0.14	8.80E-01	9.01E-01
**AC002310.12**	-0.05	8.80E-01	9.01E-01
**LINC01220**	0.15	8.80E-01	9.01E-01
**BAZ1B**	0.01	8.81E-01	9.01E-01
**USP40**	0.02	8.81E-01	9.01E-01
**EIF4B**	-0.02	8.81E-01	9.01E-01
**TM4SF19-AS1**	0.09	8.81E-01	9.02E-01
**SPATA32**	-0.13	8.81E-01	9.02E-01
**CELSR2**	0.04	8.81E-01	9.02E-01

**AC118754.4**	0.11	8.82E-01	9.02E-01
**RP1-197B17.4**	0.11	8.82E-01	9.03E-01
**THCAT158**	0.09	8.82E-01	9.03E-01
**RP11-599B13.3**	0.14	8.82E-01	9.03E-01
**GET4**	-0.04	8.82E-01	9.03E-01
**CES1P1**	0.14	8.82E-01	9.03E-01
**PPL**	-0.03	8.83E-01	9.03E-01
**ZYG11A**	-0.06	8.83E-01	9.04E-01
**RP11-710C12.1**	0.11	8.84E-01	9.04E-01
**HSD17B2**	-0.07	8.84E-01	9.04E-01
**LINC01214**	0.05	8.84E-01	9.04E-01
**SUMO2P1**	0.10	8.84E-01	9.05E-01
**RNU4-80P**	-0.14	8.84E-01	9.05E-01
**LIPG**	-0.06	8.85E-01	9.05E-01
**DNAH10**	0.05	8.85E-01	9.05E-01
**SKP1P1**	-0.06	8.85E-01	9.05E-01
**SLC16A8**	0.06	8.85E-01	9.05E-01
**GDNF-AS1**	0.09	8.85E-01	9.05E-01
**RP11-635N19.1**	0.05	8.85E-01	9.06E-01
**RP11-85B7.4**	-0.15	8.85E-01	9.06E-01
**C2orf50**	-0.15	8.86E-01	9.06E-01
**ZFP57**	0.24	8.86E-01	9.06E-01
**BRICD5**	-0.07	8.86E-01	9.06E-01
**MGAT3-AS1**	-0.13	8.86E-01	9.06E-01
**ITGB3**	0.05	8.86E-01	9.06E-01
**XLOC_002066**	-0.07	8.86E-01	9.06E-01
**RP1-102K2.9**	-0.11	8.86E-01	9.06E-01
**KLHL23**	-0.03	8.87E-01	9.07E-01
**SNORD114-3**	0.15	8.87E-01	9.07E-01
**NDP**	-0.09	8.87E-01	9.07E-01
**RP13-270P17.2**	-0.07	8.87E-01	9.07E-01
**INTS4P1**	-0.09	8.87E-01	9.07E-01
**RP11-333I13.1**	0.06	8.87E-01	9.07E-01
**CCT4**	-0.02	8.88E-01	9.08E-01
**G2077**	-0.16	8.88E-01	9.08E-01
**SEPT14P4**	-0.14	8.88E-01	9.08E-01
**RP11-502M1.2**	-0.14	8.88E-01	9.08E-01
**TMEM91**	0.06	8.88E-01	9.08E-01
**IGHV3-15**	-0.19	8.88E-01	9.08E-01
**PPIAP31**	-0.09	8.89E-01	9.08E-01
**CLDN24**	0.12	8.89E-01	9.08E-01
**ATF1**	-0.02	8.89E-01	9.08E-01
**DIAPH1**	-0.03	8.89E-01	9.08E-01
**G25704**	0.10	8.89E-01	9.08E-01
**XLOC_008811**	-0.15	8.89E-01	9.09E-01

**XLOC_010266**	0.16	8.89E-01	9.09E-01
**BIRC2**	-0.01	8.90E-01	9.09E-01
**RASSF2**	0.04	8.90E-01	9.10E-01
**ANKRD7**	-0.12	8.90E-01	9.10E-01
**TFEC**	0.07	8.90E-01	9.10E-01
**SEPT7P2**	-0.02	8.91E-01	9.10E-01
**RP11-626G11.4**	0.08	8.91E-01	9.10E-01
**NDUFA4**	-0.02	8.91E-01	9.10E-01
**KB-208E9.1**	0.11	8.91E-01	9.11E-01
**SEC24C**	0.01	8.91E-01	9.11E-01
**SLC7A6OS**	-0.02	8.91E-01	9.11E-01
**RP11-304L19.13**	0.06	8.91E-01	9.11E-01
**OIP5**	0.04	8.92E-01	9.11E-01
**RP11-111F5.3**	-0.15	8.92E-01	9.12E-01
**RP5-1172N10.2**	-0.16	8.92E-01	9.12E-01
**RP11-177J6.1**	-0.08	8.93E-01	9.12E-01
**ASNSP6**	-0.10	8.93E-01	9.12E-01
**MSANTD2**	-0.02	8.93E-01	9.12E-01
**AL161645.2**	-0.11	8.93E-01	9.12E-01
**CENPV**	0.04	8.94E-01	9.13E-01
**LY9**	0.06	8.94E-01	9.13E-01
**RP11-77I22.2**	-0.07	8.94E-01	9.13E-01
**TRIM44**	-0.01	8.94E-01	9.13E-01
**PKD1**	-0.02	8.94E-01	9.13E-01
**RALY-AS1**	0.04	8.94E-01	9.13E-01
**RP11-483I13.6**	0.09	8.94E-01	9.13E-01
**XLOC_007992**	-0.09	8.95E-01	9.14E-01
**RP11-366M4.11**	-0.08	8.95E-01	9.14E-01
**IFIT5**	-0.03	8.95E-01	9.14E-01
**SC5D**	0.04	8.95E-01	9.14E-01
**RP1-101D8.1**	0.14	8.95E-01	9.14E-01
**RP5-998N21.4**	0.14	8.95E-01	9.14E-01
**RP11-359P5.1**	-0.12	8.96E-01	9.14E-01
**LYSMD4**	-0.02	8.96E-01	9.14E-01
**LINC01152**	-0.10	8.96E-01	9.14E-01
**OTOP2**	-0.09	8.96E-01	9.14E-01
**PWP1**	0.02	8.96E-01	9.15E-01
**FAM106A**	-0.14	8.96E-01	9.15E-01
**GAS2L2**	-0.06	8.96E-01	9.15E-01
**FAM120AOS**	0.01	8.96E-01	9.15E-01
**RP11-114M1.2**	0.10	8.97E-01	9.15E-01
**XKR9**	0.08	8.97E-01	9.15E-01
**OGFR-AS1**	0.09	8.97E-01	9.15E-01
**LBHD1**	-0.05	8.97E-01	9.15E-01
**PPM1K**	-0.05	8.97E-01	9.15E-01

**RP11-129M16.4**	0.04	8.97E-01	9.16E-01
**SPAG6**	-0.12	8.97E-01	9.16E-01
**TMLHE-AS1**	-0.11	8.98E-01	9.16E-01
**RP3-414A15.12**	0.07	8.98E-01	9.16E-01
**TRMT61B**	0.02	8.98E-01	9.17E-01
**RP11-501J20.5**	-0.09	8.98E-01	9.17E-01
**CTB-63M22.1**	0.03	8.99E-01	9.17E-01
**SLC38A4**	-0.04	8.99E-01	9.17E-01
**RP11-1275H24.1**	0.03	8.99E-01	9.17E-01
**CTB-58E17.9**	-0.18	8.99E-01	9.17E-01
**ARHGEF38**	-0.07	8.99E-01	9.17E-01
**PTGS2**	0.05	8.99E-01	9.17E-01
**AP003068.18**	-0.06	8.99E-01	9.17E-01
**MYBPH**	0.12	9.00E-01	9.18E-01
**AC009404.2**	0.04	9.00E-01	9.18E-01
**C1orf186**	-0.05	9.00E-01	9.18E-01
**HSPE1P5**	-0.09	9.00E-01	9.18E-01
**TMEM126A**	0.02	9.00E-01	9.18E-01
**FBN1**	-0.07	9.00E-01	9.18E-01
**AOX1**	-0.07	9.00E-01	9.18E-01
**RP11-731C17.2**	0.03	9.00E-01	9.18E-01
**ZNF641**	-0.02	9.01E-01	9.19E-01
**PMS2P2**	-0.08	9.01E-01	9.19E-01
**STAU2-AS1**	-0.08	9.01E-01	9.19E-01
**CFAP54**	0.08	9.01E-01	9.19E-01
**PTMAP2**	-0.03	9.02E-01	9.19E-01
**CICP14**	-0.03	9.02E-01	9.19E-01
**PFN4**	0.05	9.02E-01	9.19E-01
**AGPAT4**	0.03	9.02E-01	9.20E-01
**G16276**	0.10	9.02E-01	9.20E-01
**TYW1B**	-0.04	9.02E-01	9.20E-01
**RNU6-1111P**	0.09	9.02E-01	9.20E-01
**RP6-91H8.5**	-0.14	9.02E-01	9.20E-01
**MYOCD**	0.05	9.02E-01	9.20E-01
**Z69720.3**	-0.13	9.03E-01	9.20E-01
**STMND1**	0.15	9.03E-01	9.21E-01
**RP11-30B1.1**	0.09	9.03E-01	9.21E-01
**AGAP9**	-0.07	9.03E-01	9.21E-01
**KIAA0922**	0.02	9.03E-01	9.21E-01
**TRIM15**	0.08	9.04E-01	9.21E-01
**RN7SKP150**	0.12	9.04E-01	9.21E-01
**HSPA8P14**	0.10	9.04E-01	9.21E-01
**PBLD**	-0.02	9.04E-01	9.21E-01
**PCDHA3**	-0.08	9.04E-01	9.22E-01
**RP11-686D22.7**	0.05	9.04E-01	9.22E-01

**RP11-89B16.1**	0.09	9.05E-01	9.22E-01
**DUOXA2**	0.04	9.05E-01	9.22E-01
**AP1S3**	-0.03	9.05E-01	9.22E-01
**PHBP19**	0.08	9.05E-01	9.22E-01
**HNRNPUL2-BSCL2**	-0.09	9.05E-01	9.23E-01
**AC024580.1**	0.09	9.06E-01	9.23E-01
**CCT6A**	-0.02	9.06E-01	9.23E-01
**G35086**	-0.05	9.06E-01	9.23E-01
**TAF7**	-0.02	9.06E-01	9.23E-01
**TMLHE**	0.02	9.07E-01	9.24E-01
**MTFR1**	-0.01	9.07E-01	9.24E-01
**PLIN2**	0.04	9.07E-01	9.24E-01
**COX7C**	-0.02	9.07E-01	9.24E-01
**RP11-308D13.3**	0.06	9.08E-01	9.25E-01
**RNF224**	-0.05	9.08E-01	9.25E-01
**AC004951.5**	0.05	9.08E-01	9.25E-01
**IP6K3**	0.09	9.08E-01	9.25E-01
**CA6**	-0.08	9.08E-01	9.25E-01
**RP11-490H24.5**	0.08	9.08E-01	9.25E-01
**AC005753.1**	-0.11	9.08E-01	9.25E-01
**LRRC66**	0.06	9.08E-01	9.25E-01
**NME6**	0.01	9.08E-01	9.25E-01
**RP11-676M6.1**	0.04	9.09E-01	9.25E-01
**CTB-32O4.2**	0.07	9.09E-01	9.25E-01
**TLR4**	-0.05	9.09E-01	9.26E-01
**ZDHHC8P1**	0.05	9.09E-01	9.26E-01
**FASTKD5**	-0.02	9.09E-01	9.26E-01
**RP11-152F13.7**	-0.06	9.09E-01	9.26E-01
**CTD-2035E11.4**	-0.07	9.09E-01	9.26E-01
**PCDHGA12**	0.04	9.09E-01	9.26E-01
**MRPL47**	-0.02	9.09E-01	9.26E-01
**IFT57**	-0.02	9.09E-01	9.26E-01
**KB-173C10.2**	0.06	9.10E-01	9.26E-01
**RP11-756G20.1**	0.09	9.10E-01	9.26E-01
**RP11-180P8.5**	0.13	9.10E-01	9.26E-01
**SVIL**	0.01	9.10E-01	9.27E-01
**SET**	0.02	9.11E-01	9.27E-01
**PLA2G4F**	0.03	9.11E-01	9.27E-01
**PIP**	-0.21	9.11E-01	9.27E-01
**MYH3**	-0.03	9.11E-01	9.27E-01
**TNRC6C**	0.02	9.11E-01	9.27E-01
**MAP3K8**	-0.02	9.11E-01	9.27E-01
**HOTAIRM1**	0.02	9.11E-01	9.27E-01
**RP11-730A19.9**	-0.07	9.11E-01	9.27E-01
**DARS-AS1**	0.03	9.12E-01	9.28E-01

**CEBPA**	-0.03	9.12E-01	9.28E-01
**PROSER1**	0.02	9.12E-01	9.28E-01
**MRPL35**	-0.01	9.12E-01	9.28E-01
**RRN3P2**	0.06	9.12E-01	9.28E-01
**RP11-121A8.1**	-0.06	9.12E-01	9.28E-01
**NPAS3**	0.04	9.12E-01	9.28E-01
**RP11-246A10.1**	0.06	9.13E-01	9.28E-01
**RP11-44N11.3**	-0.11	9.13E-01	9.29E-01
**ASPDH**	-0.05	9.13E-01	9.29E-01
**CTA-305I2.1**	0.07	9.13E-01	9.29E-01
**RP11-436K8.1**	0.08	9.13E-01	9.29E-01
**RSF1**	-0.01	9.13E-01	9.29E-01
**GRIK2**	0.08	9.13E-01	9.29E-01
**RP11-211G23.2**	-0.09	9.13E-01	9.29E-01
**ZNF821**	0.02	9.14E-01	9.29E-01
**ARGLU1**	-0.02	9.14E-01	9.29E-01
**TXNDC15**	-0.02	9.14E-01	9.29E-01
**ERMN**	0.07	9.14E-01	9.29E-01
**NCKAP5**	-0.02	9.14E-01	9.29E-01
**TMEM123**	0.02	9.14E-01	9.29E-01
**KCNC4**	0.03	9.14E-01	9.30E-01
**HADH**	0.02	9.15E-01	9.30E-01
**CTD-3035K23.7**	0.04	9.15E-01	9.30E-01
**RPS3AP43**	-0.12	9.15E-01	9.30E-01
**BMP5**	0.08	9.15E-01	9.30E-01
**GRIN2C**	-0.05	9.15E-01	9.30E-01
**MINPP1**	-0.02	9.16E-01	9.31E-01
**RP11-307L14.1**	-0.04	9.16E-01	9.32E-01
**SRCIN1**	0.03	9.17E-01	9.32E-01
**CHAC2**	0.03	9.17E-01	9.32E-01
**TRMT6**	0.02	9.17E-01	9.32E-01
**XLOC_002825**	0.13	9.17E-01	9.32E-01
**SOWAHC**	0.02	9.17E-01	9.32E-01
**CTC-457L16.1**	0.11	9.17E-01	9.32E-01
**hsa-mir-3149**	0.10	9.18E-01	9.33E-01
**PRKXP1**	0.04	9.18E-01	9.33E-01
**G34277**	0.05	9.18E-01	9.33E-01
**RP11-339B21.8**	0.09	9.18E-01	9.33E-01
**P3H2-AS1**	-0.10	9.18E-01	9.33E-01
**RP11-259O18.5**	0.12	9.18E-01	9.33E-01
**RP11-490M8.1**	-0.02	9.18E-01	9.33E-01
**RALGDS**	-0.02	9.18E-01	9.33E-01
**RP13-753N3.1**	0.05	9.18E-01	9.33E-01
**RP11-496I9.1**	0.08	9.18E-01	9.33E-01
**CEP104**	-0.01	9.18E-01	9.33E-01

**RP11-497H16.2**	-0.11	9.19E-01	9.34E-01
**ANKRD55**	0.05	9.19E-01	9.34E-01
**RP11-498P14.3**	0.05	9.19E-01	9.34E-01
**REEP1**	-0.02	9.19E-01	9.34E-01
**CTD-2554C21.1**	0.10	9.19E-01	9.34E-01
**XLOC_014403**	-0.09	9.20E-01	9.34E-01
**RPL7P23**	-0.05	9.20E-01	9.34E-01
**XLOC_007243**	-0.14	9.20E-01	9.35E-01
**RP11-358B23.1**	-0.08	9.20E-01	9.35E-01
**CTD-2554C21.2**	-0.04	9.20E-01	9.35E-01
**HPN**	-0.08	9.20E-01	9.35E-01
**RPL34**	-0.02	9.20E-01	9.35E-01
**CACNA1E**	-0.06	9.21E-01	9.35E-01
**XLOC_004680**	0.08	9.21E-01	9.35E-01
**MRAP2**	0.03	9.21E-01	9.36E-01
**CMTM2**	0.06	9.21E-01	9.36E-01
**COG1**	0.01	9.21E-01	9.36E-01
**CTC-786C10.1**	0.05	9.22E-01	9.36E-01
**RP11-535M15.2**	0.08	9.22E-01	9.36E-01
**RP4-756G23.5**	-0.04	9.22E-01	9.36E-01
**FLJ30679**	-0.09	9.22E-01	9.36E-01
**RP11-245P10.4**	0.05	9.22E-01	9.36E-01
**RP11-875O11.2**	0.08	9.22E-01	9.36E-01
**AC144652.1**	0.05	9.22E-01	9.36E-01
**RPL7L1**	0.01	9.22E-01	9.37E-01
**AC022182.1**	0.07	9.23E-01	9.37E-01
**H2AFZ**	-0.02	9.23E-01	9.37E-01
**PBX4**	0.02	9.23E-01	9.37E-01
**MIR4728**	0.12	9.23E-01	9.37E-01
**PLIN5**	-0.08	9.23E-01	9.37E-01
**LINC00668**	-0.08	9.24E-01	9.38E-01
**MEIS1-AS3**	0.09	9.24E-01	9.38E-01
**AC004158.3**	-0.09	9.24E-01	9.38E-01
**TRIP13**	-0.02	9.24E-01	9.38E-01
**KLK5**	0.02	9.24E-01	9.38E-01
**RP11-298I3.6**	0.05	9.24E-01	9.38E-01
**RP1-309F20.4**	-0.09	9.24E-01	9.38E-01
**BTBD9-AS1**	-0.05	9.25E-01	9.38E-01
**TRAV8-2**	-0.12	9.25E-01	9.38E-01
**RP11-407G23.4**	-0.04	9.25E-01	9.38E-01
**SLC23A1**	-0.04	9.26E-01	9.39E-01
**RP11-1252I4.2**	0.09	9.26E-01	9.40E-01
**RP11-62I21.1**	-0.08	9.26E-01	9.40E-01
**CTC-246B18.10**	0.07	9.26E-01	9.40E-01
**MGST1**	0.07	9.26E-01	9.40E-01

**RP11-1008C21.2**	-0.03	9.26E-01	9.40E-01
**XLOC_002005**	-0.13	9.27E-01	9.40E-01
**MT-RNR2**	-0.06	9.27E-01	9.40E-01
**G22953**	0.04	9.27E-01	9.40E-01
**HEMK1**	-0.02	9.27E-01	9.40E-01
**RP11-318C24.2**	0.07	9.27E-01	9.40E-01
**KB-1517D11.4**	0.09	9.28E-01	9.41E-01
**TMEM81**	-0.02	9.28E-01	9.41E-01
**RNA5SP370**	-0.14	9.28E-01	9.41E-01
**FOXD3-AS1**	0.05	9.28E-01	9.41E-01
**8-Mar**	0.01	9.28E-01	9.41E-01
**MIR4451**	0.06	9.28E-01	9.41E-01
**THBS3**	-0.02	9.28E-01	9.42E-01
**ZNF630**	-0.02	9.29E-01	9.42E-01
**PLEKHG6**	-0.02	9.29E-01	9.42E-01
**ASGR2**	-0.03	9.29E-01	9.42E-01
**PEX12**	0.01	9.29E-01	9.42E-01
**RP11-411K7.1**	-0.02	9.29E-01	9.42E-01
**RP11-408H1.3**	0.06	9.29E-01	9.42E-01
**IL1B**	0.04	9.29E-01	9.43E-01
**IGHA1**	-0.08	9.30E-01	9.43E-01
**FOLR3**	-0.07	9.30E-01	9.43E-01
**AC079354.3**	0.09	9.30E-01	9.43E-01
**RP11-567O18.1**	-0.06	9.30E-01	9.43E-01
**AP000255.6**	-0.05	9.31E-01	9.43E-01
**CCDC175**	0.11	9.31E-01	9.44E-01
**G26215**	0.09	9.31E-01	9.44E-01
**LINC00844**	0.09	9.31E-01	9.44E-01
**RP11-15B24.5**	0.11	9.31E-01	9.44E-01
**RP1-137D17.1**	0.06	9.31E-01	9.44E-01
**ZNF793-AS1**	0.02	9.31E-01	9.44E-01
**G7635**	0.05	9.31E-01	9.44E-01
**G31132**	0.05	9.31E-01	9.44E-01
**C5orf28**	0.01	9.31E-01	9.44E-01
**KIF18BP1**	-0.07	9.31E-01	9.44E-01
**AC002472.1**	-0.08	9.31E-01	9.44E-01
**SNRPCP3**	0.06	9.32E-01	9.44E-01
**SRD5A3-AS1**	-0.03	9.32E-01	9.44E-01
**RP11-524H19.2**	0.05	9.32E-01	9.44E-01
**RP11-488C13.6**	-0.06	9.32E-01	9.45E-01
**EML6**	-0.03	9.32E-01	9.45E-01
**RHOQP1**	0.06	9.32E-01	9.45E-01
**CTB-35F21.4**	0.09	9.32E-01	9.45E-01
**CPA5**	-0.05	9.33E-01	9.45E-01
**ERICH1-AS1**	0.08	9.33E-01	9.45E-01

**PDZPH1P**	-0.09	9.33E-01	9.45E-01
**CAPN8**	0.06	9.33E-01	9.45E-01
**RP11-528I4.2**	-0.10	9.33E-01	9.45E-01
**EFNB2**	0.01	9.33E-01	9.45E-01
**RPL9P29**	-0.04	9.33E-01	9.45E-01
**C5orf45**	0.03	9.34E-01	9.46E-01
**SRP14-AS1**	0.02	9.34E-01	9.46E-01
**XLOC_007866**	0.08	9.34E-01	9.46E-01
**ROBO1**	-0.01	9.34E-01	9.46E-01
**RP11-349H17.2**	0.09	9.34E-01	9.46E-01
**XLOC_013474**	0.05	9.34E-01	9.46E-01
**GSTM5**	0.04	9.34E-01	9.46E-01
**TCERG1L**	-0.04	9.35E-01	9.47E-01
**TTLL11-IT1**	0.09	9.35E-01	9.47E-01
**MIR1249**	0.06	9.35E-01	9.47E-01
**RP11-517O13.1**	-0.06	9.35E-01	9.47E-01
**NRBP2**	-0.03	9.35E-01	9.47E-01
**NPHP4**	0.02	9.35E-01	9.47E-01
**ZPR1**	-0.01	9.35E-01	9.47E-01
**HEBP2**	-0.01	9.36E-01	9.47E-01
**MIR621**	-0.02	9.36E-01	9.47E-01
**RDH5**	0.03	9.36E-01	9.48E-01
**NUAK1**	-0.02	9.36E-01	9.48E-01
**DNAH17-AS1**	0.07	9.36E-01	9.48E-01
**RPL9P7**	-0.05	9.36E-01	9.48E-01
**GLIS2-AS1**	0.06	9.37E-01	9.49E-01
**AP001627.1**	0.08	9.37E-01	9.49E-01
**CXCR1**	0.04	9.38E-01	9.49E-01
**DTX3L**	0.01	9.38E-01	9.49E-01
**MERTK**	-0.04	9.38E-01	9.49E-01
**RP11-963H4.3**	0.06	9.38E-01	9.49E-01
**RP11-296O14.3**	-0.03	9.39E-01	9.50E-01
**RP3-402G11.27**	-0.04	9.39E-01	9.50E-01
**APBA2**	0.03	9.39E-01	9.50E-01
**GAB2**	-0.01	9.39E-01	9.50E-01
**SLC27A6**	0.04	9.39E-01	9.50E-01
**ADGRD1**	-0.03	9.39E-01	9.51E-01
**RNASEH1**	0.01	9.40E-01	9.51E-01
**MXRA5**	-0.02	9.40E-01	9.51E-01
**LA16c-313F4.1**	0.03	9.40E-01	9.51E-01
**RPP21**	0.03	9.40E-01	9.51E-01
**HNRNPCP1**	0.03	9.41E-01	9.52E-01
**CYP4F29P**	-0.08	9.41E-01	9.52E-01
**RPS27A**	-0.01	9.41E-01	9.52E-01
**RP11-264B17.3**	-0.07	9.41E-01	9.52E-01

**RP11-384M15.3**	-0.07	9.41E-01	9.52E-01
**RNU4-68P**	0.07	9.41E-01	9.52E-01
**RPS27**	0.01	9.41E-01	9.52E-01
**MIPEPP3**	0.02	9.41E-01	9.52E-01
**G1580**	-0.06	9.41E-01	9.52E-01
**GRIA2**	0.11	9.42E-01	9.53E-01
**BZRAP1**	-0.02	9.42E-01	9.53E-01
**RN7SL378P**	-0.06	9.42E-01	9.53E-01
**RTEL1-TNFRSF6B**	-0.04	9.42E-01	9.53E-01
**RP11-465N4.4**	-0.05	9.42E-01	9.53E-01
**RP11-288L9.4**	-0.07	9.42E-01	9.53E-01
**RBM8B**	0.06	9.42E-01	9.53E-01
**CDC25A**	-0.02	9.42E-01	9.53E-01
**PABPC1L**	-0.03	9.42E-01	9.53E-01
**RP11-141O11.2**	0.09	9.42E-01	9.53E-01
**DPP4**	0.03	9.43E-01	9.53E-01
**LYPD5**	0.02	9.43E-01	9.53E-01
**FBXO41**	0.01	9.43E-01	9.53E-01
**AC068282.3**	-0.03	9.43E-01	9.54E-01
**COX19**	0.01	9.43E-01	9.54E-01
**RP13-317D12.3**	-0.06	9.44E-01	9.54E-01
**RPSAP69**	-0.07	9.44E-01	9.54E-01
**IQGAP2**	0.03	9.44E-01	9.54E-01
**CTD-2588E21.1**	0.05	9.44E-01	9.54E-01
**HAND2**	0.03	9.44E-01	9.54E-01
**KRT8P48**	-0.08	9.44E-01	9.55E-01
**EFCAB12**	-0.05	9.44E-01	9.55E-01
**DPY19L2**	0.03	9.44E-01	9.55E-01
**UBE2U**	-0.07	9.45E-01	9.55E-01
**G14802**	-0.04	9.45E-01	9.55E-01
**LINC01135**	0.03	9.45E-01	9.55E-01
**RP11-134O21.1**	0.03	9.45E-01	9.55E-01
**CATSPERD**	-0.08	9.45E-01	9.55E-01
**LLNLR-246C6.1**	0.03	9.45E-01	9.56E-01
**XLOC_011518**	-0.04	9.45E-01	9.56E-01
**SP6**	0.01	9.46E-01	9.56E-01
**FAM213A**	0.02	9.46E-01	9.56E-01
**G34509**	-0.07	9.46E-01	9.56E-01
**HCG23**	-0.04	9.46E-01	9.56E-01
**AC020571.3**	-0.04	9.46E-01	9.56E-01
**TMEM133**	-0.01	9.46E-01	9.56E-01
**PLCL2**	0.02	9.46E-01	9.56E-01
**RPS3AP26**	0.02	9.46E-01	9.56E-01
**RP11-597D13.7**	-0.04	9.46E-01	9.56E-01
**RP11-322E11.2**	0.07	9.47E-01	9.57E-01

**ABAT**	0.02	9.47E-01	9.57E-01
**TRAPPC9**	-0.01	9.47E-01	9.57E-01
**SUSD3**	-0.02	9.48E-01	9.57E-01
**RP11-640L9.1**	-0.05	9.48E-01	9.58E-01
**STARD5**	0.01	9.48E-01	9.58E-01
**RP11-353N14.2**	-0.06	9.48E-01	9.58E-01
**LYRM9**	-0.01	9.49E-01	9.58E-01
**LINC00923**	0.07	9.49E-01	9.58E-01
**GVQW2**	-0.03	9.49E-01	9.58E-01
**NGLY1**	-0.01	9.49E-01	9.59E-01
**OR7A5**	-0.04	9.49E-01	9.59E-01
**OCM**	-0.05	9.50E-01	9.59E-01
**CERK**	-0.01	9.50E-01	9.59E-01
**CTD-2372A4.1**	-0.07	9.50E-01	9.59E-01
**AARSD1**	0.03	9.50E-01	9.59E-01
**RNU6-925P**	0.05	9.50E-01	9.59E-01
**CTD-2110K23.1**	-0.07	9.51E-01	9.60E-01
**IGHG4**	-0.16	9.51E-01	9.60E-01
**RP11-561C5.4**	0.05	9.51E-01	9.60E-01
**PLPPR3**	-0.06	9.51E-01	9.60E-01
**RP11-350N15.4**	-0.06	9.51E-01	9.60E-01
**CTD-2278I10.4**	-0.04	9.51E-01	9.60E-01
**C15orf65**	0.02	9.51E-01	9.60E-01
**SPIRE1**	-0.01	9.51E-01	9.60E-01
**CAMSAP1**	0.01	9.52E-01	9.61E-01
**ELAVL2**	0.03	9.52E-01	9.61E-01
**JPH3**	-0.04	9.52E-01	9.61E-01
**ATP5A1**	0.01	9.52E-01	9.61E-01
**RP11-706C16.8**	0.06	9.52E-01	9.61E-01
**RP11-276H19.2**	-0.02	9.52E-01	9.61E-01
**NPHP3-AS1**	0.05	9.52E-01	9.61E-01
**G511**	-0.05	9.52E-01	9.61E-01
**ZNF182**	-0.01	9.52E-01	9.61E-01
**AC022431.3**	0.05	9.52E-01	9.61E-01
**GAL3ST1**	0.03	9.52E-01	9.61E-01
**LTB4R2**	0.01	9.53E-01	9.61E-01
**AC022154.7**	0.04	9.53E-01	9.61E-01
**RP11-357D18.1**	0.06	9.53E-01	9.62E-01
**FGGY**	-0.01	9.53E-01	9.62E-01
**RP11-339B9.1**	0.07	9.53E-01	9.62E-01
**RPL21P75**	-0.03	9.53E-01	9.62E-01
**SPCS2P4**	-0.02	9.53E-01	9.62E-01
**CNOT6LP1**	-0.03	9.54E-01	9.62E-01
**NHLRC3**	0.01	9.54E-01	9.62E-01
**PAM16**	0.02	9.54E-01	9.63E-01

**RAD21-AS1**	0.02	9.54E-01	9.63E-01
**AL591025.1**	-0.04	9.55E-01	9.63E-01
**SAMHD1**	-0.02	9.55E-01	9.63E-01
**RP11-552F3.12**	-0.05	9.55E-01	9.63E-01
**WDR61**	-0.01	9.55E-01	9.63E-01
**CTNNB1**	-0.01	9.55E-01	9.63E-01
**CISD2**	0.01	9.55E-01	9.63E-01
**XLOC_005087**	-0.05	9.56E-01	9.64E-01
**RP11-783K16.5**	0.05	9.56E-01	9.64E-01
**MAP3K5**	0.01	9.56E-01	9.64E-01
**RPL41P5**	0.02	9.56E-01	9.64E-01
**XLOC_008975**	0.04	9.57E-01	9.65E-01
**LIPC**	-0.03	9.57E-01	9.65E-01
**SLC25A25-AS1**	0.02	9.57E-01	9.65E-01
**ACSS3**	-0.02	9.57E-01	9.65E-01
**RN7SL329P**	0.04	9.58E-01	9.66E-01
**RP5-1007F24.1**	-0.04	9.58E-01	9.66E-01
**SRPK2**	0.01	9.58E-01	9.66E-01
**G34668**	0.04	9.58E-01	9.66E-01
**NOG**	0.04	9.58E-01	9.66E-01
**AC006116.24**	0.06	9.58E-01	9.66E-01
**RP11-363J20.2**	-0.03	9.58E-01	9.66E-01
**FBXO39**	-0.02	9.59E-01	9.66E-01
**ZNF684**	-0.01	9.59E-01	9.66E-01
**RP4-669K10.8**	0.02	9.59E-01	9.66E-01
**OR5BA1P**	0.03	9.59E-01	9.67E-01
**XLOC_008955**	0.03	9.60E-01	9.67E-01
**MYCBPAP**	0.02	9.60E-01	9.67E-01
**RP11-666A8.7**	-0.03	9.60E-01	9.67E-01
**RP11-44N11.2**	-0.03	9.60E-01	9.68E-01
**DPM1**	-0.01	9.60E-01	9.68E-01
**POU5F1B**	0.02	9.60E-01	9.68E-01
**AC009961.2**	-0.03	9.60E-01	9.68E-01
**RP11-46A10.5**	-0.02	9.60E-01	9.68E-01
**ANXA9**	0.01	9.61E-01	9.68E-01
**AC006262.5**	-0.03	9.61E-01	9.68E-01
**TSEN15**	-0.01	9.61E-01	9.68E-01
**TBX19**	0.01	9.61E-01	9.68E-01
**LA16c-359F1.1**	-0.04	9.61E-01	9.68E-01
**RP11-49I11.1**	0.05	9.62E-01	9.69E-01
**AC005776.1**	-0.03	9.62E-01	9.69E-01
**RP11-64B16.2**	0.01	9.62E-01	9.69E-01
**RP11-429J17.8**	0.03	9.62E-01	9.69E-01
**HOXA6**	0.02	9.63E-01	9.70E-01
**GSTM1**	-0.09	9.63E-01	9.70E-01

**PLD5**	0.03	9.63E-01	9.70E-01
**G12586**	-0.03	9.63E-01	9.70E-01
**RP11-748H22.1**	0.03	9.63E-01	9.70E-01
**APMAP**	0.02	9.64E-01	9.71E-01
**SLC5A5**	-0.05	9.64E-01	9.71E-01
**PLCH2**	0.01	9.64E-01	9.71E-01
**FNTB**	-0.01	9.64E-01	9.71E-01
**TTLL3**	0.02	9.64E-01	9.71E-01
**PTPRJ-AS1**	0.04	9.65E-01	9.71E-01
**SOGA3**	0.04	9.65E-01	9.72E-01
**C9orf173-AS1**	-0.03	9.66E-01	9.72E-01
**IGSF9**	0.01	9.66E-01	9.72E-01
**PIGP**	0.01	9.66E-01	9.72E-01
**UBE2Q2P6**	0.05	9.66E-01	9.73E-01
**STAP1**	0.02	9.66E-01	9.73E-01
**CH17-140K24.6**	0.02	9.66E-01	9.73E-01
**RP11-313D6.3**	-0.04	9.66E-01	9.73E-01
**RP11-612B6.2**	-0.01	9.67E-01	9.73E-01
**LRRC29**	0.01	9.67E-01	9.73E-01
**NBPF12**	-0.01	9.67E-01	9.73E-01
**OLA1**	-0.01	9.67E-01	9.73E-01
**AL357115.1**	0.04	9.67E-01	9.74E-01
**SSC5D**	-0.02	9.67E-01	9.74E-01
**ZNF321P**	-0.01	9.67E-01	9.74E-01
**SLC6A10P**	0.04	9.67E-01	9.74E-01
**RP11-588G21.2**	0.03	9.68E-01	9.74E-01
**UG0898H09**	-0.02	9.68E-01	9.74E-01
**TH**	-0.03	9.68E-01	9.74E-01
**AC142472.6**	0.01	9.68E-01	9.75E-01
**RP11-430K21.2**	-0.03	9.68E-01	9.75E-01
**PCDHA5**	-0.04	9.68E-01	9.75E-01
**CTD-2583P5.3**	0.03	9.69E-01	9.75E-01
**KCTD17**	-0.01	9.69E-01	9.75E-01
**AC010136.2**	0.04	9.69E-01	9.75E-01
**HELT**	-0.03	9.69E-01	9.75E-01
**ENDOD1**	-0.01	9.69E-01	9.75E-01
**GATA1**	-0.03	9.69E-01	9.75E-01
**PPP1R12B**	0.01	9.70E-01	9.76E-01
**STAT1**	-0.01	9.70E-01	9.76E-01
**AC015815.6**	-0.02	9.70E-01	9.76E-01
**LINC01021**	-0.05	9.70E-01	9.76E-01
**HNRNPA1P5**	0.02	9.70E-01	9.76E-01
**CYCSP34**	0.02	9.70E-01	9.76E-01
**NRAP**	0.04	9.70E-01	9.76E-01
**NUDT6**	0.01	9.70E-01	9.76E-01

**RP11-497G19.7**	-0.03	9.70E-01	9.76E-01
**C12orf76**	-0.01	9.70E-01	9.76E-01
**G31554**	-0.02	9.70E-01	9.76E-01
**RP3-466P17.1**	0.04	9.70E-01	9.76E-01
**ST13P4**	-0.02	9.70E-01	9.76E-01
**AC004895.4**	-0.04	9.70E-01	9.76E-01
**RNA5SP490**	-0.04	9.71E-01	9.76E-01
**LCE2D**	0.01	9.71E-01	9.76E-01
**RAPSN**	-0.02	9.71E-01	9.77E-01
**RP11-553L6.5**	0.01	9.71E-01	9.77E-01
**KLHL12**	0.00	9.71E-01	9.77E-01
**PDIA2**	0.03	9.72E-01	9.77E-01
**AL132709.8**	-0.02	9.72E-01	9.77E-01
**IMPACT**	-0.01	9.72E-01	9.77E-01
**RP11-296E7.1**	-0.03	9.72E-01	9.77E-01
**SBDSP1**	0.00	9.72E-01	9.77E-01
**RP11-84C10.1**	0.02	9.72E-01	9.77E-01
**RP11-43P8.2**	-0.02	9.72E-01	9.77E-01
**RP11-110G21.2**	-0.02	9.72E-01	9.78E-01
**PDZD3**	0.02	9.73E-01	9.78E-01
**CTD-2280E9.1**	-0.05	9.73E-01	9.78E-01
**SLU7**	0.00	9.73E-01	9.78E-01
**CTD-2267D19.4**	-0.03	9.73E-01	9.78E-01
**ANKRD19P**	0.01	9.73E-01	9.78E-01
**LHB**	-0.02	9.73E-01	9.79E-01
**ABTB2**	0.01	9.73E-01	9.79E-01
**RP11-617F23.1**	0.01	9.74E-01	9.79E-01
**PTPN5**	-0.02	9.74E-01	9.79E-01
**RP4-529N6.1**	0.02	9.74E-01	9.79E-01
**LOXL4**	0.01	9.74E-01	9.79E-01
**RRN3P1**	0.01	9.74E-01	9.79E-01
**AC007255.8**	-0.03	9.75E-01	9.80E-01
**MTG1**	0.01	9.75E-01	9.80E-01
**CTB-60B18.18**	0.02	9.75E-01	9.80E-01
**RP11-327P2.5**	0.01	9.75E-01	9.80E-01
**PHYKPL**	0.01	9.75E-01	9.80E-01
**CDR2**	0.01	9.75E-01	9.80E-01
**RP11-77P16.4**	-0.02	9.75E-01	9.80E-01
**AMH**	0.02	9.75E-01	9.80E-01
**RP11-173M1.4**	-0.03	9.76E-01	9.80E-01
**PURG**	-0.02	9.76E-01	9.80E-01
**RP1-193H18.3**	0.02	9.76E-01	9.80E-01
**WNT9B**	-0.01	9.76E-01	9.80E-01
**RP1-45I4.3**	-0.02	9.76E-01	9.80E-01
**RP4-583P15.16**	0.03	9.76E-01	9.80E-01

**RP4-604G5.1**	-0.03	9.76E-01	9.81E-01
**IFRD1**	0.01	9.76E-01	9.81E-01
**RP3-473L9.4**	0.02	9.76E-01	9.81E-01
**LINC00237**	-0.03	9.77E-01	9.81E-01
**PCDHA11**	-0.02	9.77E-01	9.81E-01
**CTB-50L17.8**	0.01	9.77E-01	9.81E-01
**MT-RNR1**	0.02	9.77E-01	9.81E-01
**RP11-74J13.9**	-0.01	9.77E-01	9.81E-01
**RP3-416H24.1**	-0.03	9.77E-01	9.81E-01
**CTD-2026K11.4**	0.01	9.78E-01	9.82E-01
**AL162424.1**	-0.02	9.78E-01	9.82E-01
**XLOC_002085**	-0.03	9.78E-01	9.82E-01
**CYP24A1**	-0.01	9.78E-01	9.82E-01
**AC079781.5**	0.03	9.78E-01	9.82E-01
**AC087491.2**	0.02	9.78E-01	9.82E-01
**NCAPH**	0.01	9.79E-01	9.83E-01
**PPP1R14BP3**	-0.01	9.79E-01	9.83E-01
**RP11-686D22.8**	-0.02	9.79E-01	9.83E-01
**CD47**	0.00	9.79E-01	9.83E-01
**RP11-452F19.3**	-0.01	9.79E-01	9.83E-01
**TMX2**	0.00	9.79E-01	9.83E-01
**GPR156**	0.01	9.79E-01	9.83E-01
**RPL12P21**	0.02	9.79E-01	9.83E-01
**NRADDP**	-0.01	9.80E-01	9.83E-01
**ZNF738**	-0.01	9.80E-01	9.83E-01
**CTD-2008L17.2**	0.02	9.80E-01	9.84E-01
**RP11-35G9.3**	0.01	9.80E-01	9.84E-01
**LINC00315**	-0.03	9.80E-01	9.84E-01
**FOXO3B**	-0.01	9.80E-01	9.84E-01
**NRTN**	-0.01	9.81E-01	9.84E-01
**TRIM73**	-0.02	9.81E-01	9.84E-01
**ACLY**	0.01	9.81E-01	9.84E-01
**RP11-84G21.1**	-0.01	9.81E-01	9.84E-01
**TNKS2-AS1**	-0.02	9.81E-01	9.85E-01
**RP11-1000B6.7**	-0.02	9.81E-01	9.85E-01
**RP11-66B24.9**	0.02	9.82E-01	9.85E-01
**C1orf234**	-0.02	9.82E-01	9.85E-01
**CD19**	0.02	9.82E-01	9.85E-01
**RP11-1191J2.5**	-0.02	9.82E-01	9.85E-01
**LRRC70**	0.01	9.82E-01	9.85E-01
**RP11-277B15.3**	0.02	9.82E-01	9.85E-01
**TCAF1**	0.00	9.82E-01	9.85E-01
**KHK**	-0.01	9.82E-01	9.85E-01
**RP11-467L13.4**	0.02	9.82E-01	9.86E-01
**BTN3A1**	0.00	9.83E-01	9.86E-01

**XLOC_005410**	0.03	9.83E-01	9.86E-01
**TMEM229B**	0.01	9.83E-01	9.86E-01
**EIF4ENIF1**	0.00	9.83E-01	9.86E-01
**TMEM179B**	0.00	9.83E-01	9.86E-01
**SLC7A9**	-0.01	9.83E-01	9.86E-01
**RP11-131N11.4**	-0.01	9.83E-01	9.86E-01
**MROH1**	0.00	9.84E-01	9.87E-01
**ZFP82**	0.01	9.84E-01	9.87E-01
**XLOC_010575**	0.01	9.84E-01	9.87E-01
**DNASE1L3**	0.00	9.84E-01	9.87E-01
**RPS6KA2-IT1**	-0.01	9.84E-01	9.87E-01
**SEC11C**	0.00	9.85E-01	9.87E-01
**TUBA1B**	0.00	9.85E-01	9.88E-01
**RP11-483P21.2**	0.01	9.85E-01	9.88E-01
**RNASEL**	0.00	9.85E-01	9.88E-01
**AC005618.6**	0.01	9.85E-01	9.88E-01
**FKBP15**	0.00	9.85E-01	9.88E-01
**NBPF13P**	0.01	9.85E-01	9.88E-01
**IL13RA2**	0.02	9.86E-01	9.88E-01
**LINC00654**	-0.01	9.86E-01	9.88E-01
**G32068**	-0.02	9.86E-01	9.89E-01
**RP11-927P21.2**	-0.02	9.86E-01	9.89E-01
**MPPE1**	0.00	9.87E-01	9.89E-01
**RP11-468E2.4**	0.01	9.87E-01	9.89E-01
**PSMG4**	0.00	9.87E-01	9.89E-01
**RN7SL37P**	0.01	9.87E-01	9.89E-01
**G19302**	-0.02	9.87E-01	9.90E-01
**AP000347.4**	-0.01	9.88E-01	9.90E-01
**ACACB**	0.00	9.88E-01	9.90E-01
**RP3-495K2.4**	0.01	9.88E-01	9.90E-01
**LINC01446**	-0.03	9.88E-01	9.90E-01
**C9orf173**	-0.02	9.88E-01	9.90E-01
**RP11-734I18.1**	0.02	9.88E-01	9.90E-01
**GALC**	0.00	9.89E-01	9.91E-01
**FDPS**	0.00	9.89E-01	9.91E-01
**G23907**	0.01	9.89E-01	9.91E-01
**AP001055.1**	0.01	9.89E-01	9.91E-01
**C1RL-AS1**	0.00	9.89E-01	9.91E-01
**RP11-609D21.3**	-0.01	9.90E-01	9.92E-01
**CYP51A1P2**	0.01	9.90E-01	9.92E-01
**RP11-2K6.1**	-0.01	9.90E-01	9.92E-01
**RP11-403B2.7**	-0.01	9.90E-01	9.92E-01
**AHCYL1**	0.00	9.91E-01	9.93E-01
**OR7E2P**	0.01	9.91E-01	9.93E-01
**PDE6B**	0.00	9.91E-01	9.93E-01

**RP11-44F14.9**	-0.01	9.91E-01	9.93E-01
**MPP2**	0.00	9.91E-01	9.93E-01
**TRIM7**	0.00	9.91E-01	9.93E-01
**PKD1L2**	0.01	9.92E-01	9.93E-01
**AC093162.5**	0.00	9.92E-01	9.93E-01
**RP11-1198D22.1**	-0.01	9.92E-01	9.94E-01
**RSL24D1**	0.00	9.92E-01	9.94E-01
**HLA-DOB**	0.00	9.92E-01	9.94E-01
**RP13-554M15.7**	0.00	9.93E-01	9.94E-01
**C17orf75**	0.00	9.93E-01	9.94E-01
**RNU6-80P**	0.01	9.93E-01	9.94E-01
**PLA2G4B**	0.00	9.93E-01	9.94E-01
**PSIP1**	0.00	9.93E-01	9.95E-01
**RP11-166D19.1**	0.00	9.93E-01	9.95E-01
**MKLN1-AS**	0.00	9.94E-01	9.95E-01
**TTC9C**	0.00	9.94E-01	9.95E-01
**RP11-326C3.12**	0.01	9.94E-01	9.95E-01
**NYAP1**	0.00	9.94E-01	9.95E-01
**RFC4**	0.00	9.94E-01	9.95E-01
**ANAPC1P1**	-0.01	9.94E-01	9.95E-01
**ALG1L8P**	0.00	9.94E-01	9.95E-01
**SRSF2**	0.00	9.94E-01	9.95E-01
**RBM8A**	0.00	9.95E-01	9.96E-01
**PRDX1P1**	0.01	9.95E-01	9.96E-01
**XLOC_014386**	0.00	9.95E-01	9.96E-01
**G15642**	0.00	9.95E-01	9.96E-01
**EFEMP1**	0.00	9.95E-01	9.96E-01
**GTF2H4**	-0.01	9.95E-01	9.96E-01
**CTSH**	0.00	9.95E-01	9.96E-01
**GRM6**	0.00	9.95E-01	9.96E-01
**AC073109.2**	-0.01	9.95E-01	9.96E-01
**SLC1A4**	0.00	9.96E-01	9.96E-01
**RP11-428J1.5**	0.00	9.96E-01	9.97E-01
**ZPLD1**	0.00	9.96E-01	9.97E-01
**MLH1**	0.00	9.96E-01	9.97E-01
**RP11-1149O23.3**	0.00	9.96E-01	9.97E-01
**MAP3K15**	0.00	9.96E-01	9.97E-01
**IGFL4**	0.00	9.96E-01	9.97E-01
**LIG1**	0.00	9.96E-01	9.97E-01
**CCDC127**	0.00	9.97E-01	9.98E-01
**BGLAP**	0.00	9.97E-01	9.98E-01
**ADH1B**	0.00	9.97E-01	9.98E-01
**RP11-510J16.5**	0.00	9.98E-01	9.98E-01
**TIMM23B**	0.00	9.98E-01	9.98E-01
**AADAC**	0.00	9.98E-01	9.99E-01

**ACADSB**	0.00	9.98E-01	9.99E-01
**G26269**	0.00	9.99E-01	9.99E-01
**PCDH7**	0.00	9.99E-01	9.99E-01
**RP11-15M15.2**	0.00	9.99E-01	9.99E-01
**EIF1AD**	0.00	9.99E-01	1.00E+00
**SGMS1-AS1**	0.00	1.00E+00	1.00E+00
**STARD4**	0.00	1.00E+00	1.00E+00
**ADGRF3**	0.00	1.00E+00	1.00E+00
**YBX1P10**	0.00	1.00E+00	1.00E+00
**RP11-50D9.1**	0.00	1.00E+00	1.00E+00
